# Feasibility of implementing molecular-guided therapy for the treatment of patients with relapsed or refractory neuroblastoma

**DOI:** 10.1002/cam4.436

**Published:** 2015-02-26

**Authors:** Giselle L Saulnier Sholler, Jeffrey P Bond, Genevieve Bergendahl, Akshita Dutta, Julie Dragon, Kathleen Neville, William Ferguson, William Roberts, Don Eslin, Jacqueline Kraveka, Joel Kaplan, Deanna Mitchell, Nehal Parikh, Melinda Merchant, Takamaru Ashikaga, Gina Hanna, Pamela Jean Lescault, Ashley Siniard, Jason Corneveaux, Matthew Huentelman, Jeffrey Trent

**Affiliations:** 1 Helen DeVos Children's HospitalGrand Rapids, Michigan; 2 Michigan State University College of MedicineGrand Rapids, Michigan; 3 Department of Microbiology and Molecular Genetics, University of Vermont College of MedicineBurlington, Vermont; 4 Children's Mercy HospitalKansas City, Missouri; 5 Cardinal Glennon Children's Hospital, St. Louis UniversitySt. Louis, Missouri; 6 UC San Diego School of Medicine and Rady Children's HospitalSan Diego, California; 7 Arnold Palmer Hospital for ChildrenOrlando, Florida; 8 Medical University of South CarolinaCharleston, South Carolina; 9 Levine Children's HospitalCharlotte, North Carolina; 10 Connecticut Children's Medical CenterHartford, Connecticut; 11 NCI Center for Cancer ResearchBethesda, Maryland; 12 Medical Biostatistics, University of Vermont College of MedicineBurlington, Vermont; 13 Josephine Bay Paul Center for Comparative Molecular Biology and Evolution, The Marine Biological LaboratoryWoods Hole, Massachusetts; 14 Translational Genomics Research InstitutePhoenix, Arizona

**Keywords:** Genomic profiling, molecular-guided therapy, molecular tumor board, neuroblastoma, pediatric oncology

## Abstract

The primary objective of the study was to evaluate the feasibility and safety of a process which would utilize genome-wide expression data from tumor biopsies to support individualized treatment decisions. Current treatment options for recurrent neuroblastoma are limited and ineffective, with a survival rate of <10%. Molecular profiling may provide data which will enable the practitioner to select the most appropriate therapeutic option for individual patients, thus improving outcomes. Sixteen patients with neuroblastoma were enrolled of which fourteen were eligible for this study. Feasibility was defined as completion of tumor biopsy, pathological evaluation, RNA quality control, gene expression profiling, bioinformatics analysis, generation of a drug prediction report, molecular tumor board yielding a treatment plan, independent medical monitor review, and treatment initiation within a 21 day period. All eligible biopsies passed histopathology and RNA quality control. Expression profiling by microarray and RNA sequencing were mutually validated. The average time from biopsy to report generation was 5.9 days and from biopsy to initiation of treatment was 12.4 days. No serious adverse events were observed and all adverse events were expected. Clinical benefit was seen in 64% of patients as stabilization of disease for at least one cycle of therapy or partial response. The overall response rate was 7% and the progression free survival was 59 days. This study demonstrates the feasibility and safety of performing real-time genomic profiling to guide treatment decision making for pediatric neuroblastoma patients.

## Introduction

Pioneering a new chapter in medicine, this study is the first completed pediatric trial utilizing personalized medicine in the United States. We evaluated the feasibility and safety of using predictive modeling based on genome-wide mRNA expression profiles of neuroblastoma tumor biopsies to create therapeutic regimens individualized to each patient. Neuroblastoma is the most common extra cranial solid tumor in children. With 700 new diagnoses per year, it accounts for 7–10% of childhood cancers 1,2. Currently, children diagnosed after 12–15 months of age have a poor long-term survival rate despite aggressive multimodal therapies 3,4. Even for children who are able to complete high-dose chemotherapy (HDC) followed by hematopoietic stem cell transplantation (HSCT) and maintenance therapy consisting of immune therapy with antiGD2 antibody and retinoic acid, the 5-year event-free survival remains at only 50% 5,6. Long-term survival of patients following relapse is <5%, and neuroblastoma accounts for 15% of all pediatric cancer deaths in the United States 7. Given the small number of patients available, the diversity of genomic profiles 8,9, and the limited number of drugs available for testing, a deeper understanding of the genomics of neuroblastoma and its treatment is critical 10.

The management of relapsed neuroblastoma patients is particularly challenging: there are currently few treatment options from which tumor boards can select with any degree of confidence. There are no established standard-of-care treatments for relapsed neuroblastoma: options include a variety of Phase I or Phase II therapies with relatively modest response rates (10–35%) 4,11. Even in patients who initially respond to current therapies, tumors often progress on to further rapid relapses. Novel strategies are urgently needed. Recent evidence establishing the genetic heterogeneity of the disease reveals the existence of several major molecular subsets that collectively may provide prognostic value for future disease management 8,9. The identification of agents that target-specific molecular pathways associated with the development and/or progression of neoplastic diseases holds promise. Molecularly-guided approaches that identify existing agents which target-specific alterations in tumors may improve patient survival while avoiding the toxicity associated with agents that are unlikely to be beneficial 12.

It is now firmly established that cancer results from perturbations in the molecular pathways that disturb the normal cellular homeostatic state 13–16. Fluctuations in these networks may result from genetic or epigenetic events that cause gene expression changes in tumor cells. This study utilizes an approach by which the expanding knowledge of molecular pathways and the mechanisms of action of targeted drug therapies 17,18 can be utilized to create individualized therapeutic regimens using a Tumor Profiling Analysis Platform (TPAP) in real-time for patients with neuroblastoma. In our study, patients undergoing tumor biopsy have a sample sent for pathological evaluation and gene expression profiling from which bioinformatics analysis and generation of a drug prediction report is created. This is reviewed by a molecular tumor board which yields an individualized treatment plan for each patient, who is then followed for safety and response.

## Materials and Methods

### Study population

This was an open label, multicenter prospective feasibility study in patients with refractory or recurrent neuroblastoma. Patients were scheduled to undergo a standard-of-care surgical resection and/or diagnostic biopsy procedure and gave consent for additional samples to be collected during this procedure. A voluntary consent for optional biology studies was obtained. The Institutional Review Board (IRB) at WIRB, Helen DeVos Children's Hospital (MI), Arnold Palmer Children's Hospital (FL), National Cancer Institute (NCI), Children's Mercy Hospitals and Clinics (MO), Connecticut Children's Hospital (CT), Dell Children's Hospital (TX), Cardinal Glennon Children's Hospital (MO), and Levine Children's Hospital (NC) approved this trial. An IRB approved consent was obtained from each subject or subject guardian. (Clinical Trials identifier: NCT01355679; Study ID: NMTRC001). This study was conducted under FDA approval for IDE G100111.

### Eligibility

Patients with refractory or recurrent neuroblastoma disease initially diagnosed during or under the age of 21 years were eligible for this study. Current disease state was required to be one without any known curative therapy. Inclusion criteria also defined a Lansky Play score >50. Adequate bone marrow and liver function was required; no other significant organ toxicity as above Grade 2 by National Cancer Institute Common Toxicity Criteria for Adverse Events, version 4 NCI-CTCAE.

Exclusion criteria included patients who were administered chemotherapy within 7 days prior to enrollment and 14 days prior to study treatment start; patients receiving antitumor therapy for their disease or any other investigational drug; patients who had received any radiotherapy within the last 30 days without another site of disease to follow; serious infections or a life-threatening illness that is >Grade 2 (NCI-CTCAE V4.0). There was no limit put on the number of previous treatments.

### Study design

#### Primary objective

The primary objective of this study was to determine the feasibility of using predictive modeling based on genome-wide mRNA expression profiles of bone-marrow-derived neuroblastoma cells or tumor biopsies to make real-time treatment decisions. The measure was defined as “Enrollment onto study, quality mRNA obtained, gene chip completed, tumor board held, medical monitor review and approval, start of treatment by 21 days post biopsy/surgical resection date, and completion of 1 cycle of therapy.” For statistical reporting a binomial distribution was used for the testing process with a combination of Type I error levels (10%) and Power (70%) with an overall basic design as a MiniMax approach. The study accepted the null hypothesis if the observed feasibility rate was less than or equal to 9/14. Otherwise, stop and reject the null hypothesis.

#### Secondary objectives

The secondary objectives of this study were to determine the safety of allowing a molecular tumor board to determine individualized treatment plans and to determine the activity of treatments chosen based on overall response rate (ORR) and progression free survival (PFS).

### Definition of overall response for each patient

This definition is utilized to describe response in all lesions defined as measurable in this study, including CT/MRI lesions which meet RECIST criteria, MIBG-positive lesions, and bone marrow disease. These criteria are used in the statistical analysis to define the overall response of the patient. Complete response (CR) was defined as the disappearance of all target lesions. No evidence of tumor at any site (chest, abdomen, liver, bone, bone marrow, nodes, etc.), and homovanillic acid/vanillyl mandelic acid (HVA/VMA) normal. Partial response (PR) was defined as at least a 30% decrease in the disease measurement for CT/MRI target lesions, taking as reference the disease measurement done to confirm measurable disease in target lesions at study entry. Bone marrow with CR. MIBG with either PR/CR in bone lesions; MIBG may be SD or CR in soft tissue lesions corresponding to lesions on CT/MRI. HVA/VMA may still be elevated. Progressive disease (PD) was defined as any one of the following: at least a 20% increase in the disease measurement for CT/MRI target lesions, taking as reference the smallest disease measurement recorded since the start of treatment, appearance of one or more new lesions or new sites of tumor, or new disease in either the bone marrow or new MIBG lesions. Stable disease (SD) was defined as no new lesions; no new sites of disease, and they do not fit the criteria for PD/PR/CR as above.

Time to progression was defined as the period from the first day of study drug administration until the criteria for progression was met. Duration of response was defined as the period of time from when measurement criteria are met for CR or PR, whichever is first recorded, until the first date that recurrent or PD is objectively documented. The assessment of response included the initial measurable targets, was performed again after the first and second cycle, then performed again after every other cycle.

### Sample procurement and gene expression profiling

**Figure 1:**
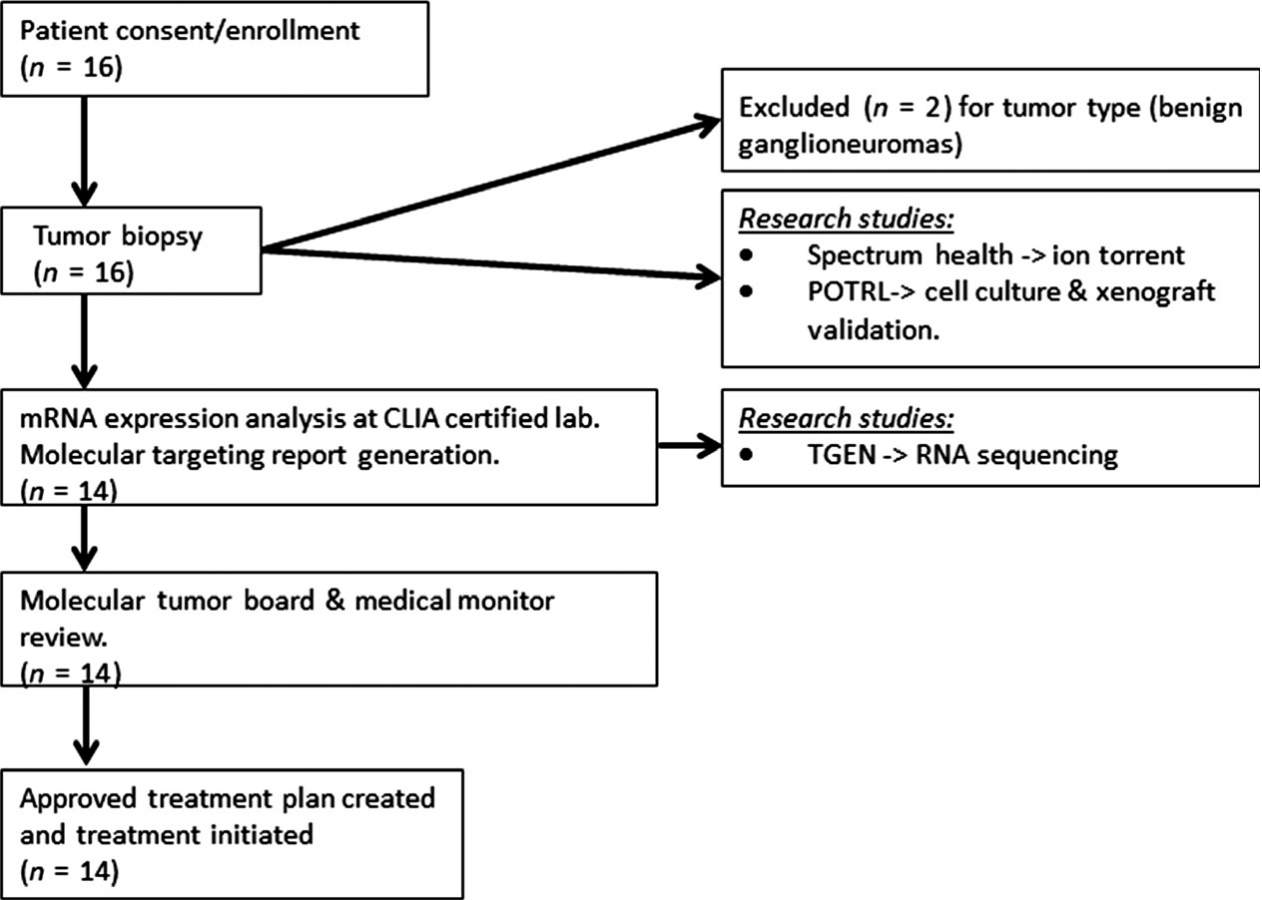
Study flow diagram. Patient biopsy was sent directly to CLIA-certified laboratory CRL and POTRL. CRL, Clinical Reference Laboratory; TGEN, Translational Genomics Research Institute; POTRL, Pediatric Oncology Translational Research Laboratory at Helen DeVos Children's Hospital.

Patients enrolled on this study were scheduled to undergo a biopsy or resection per treating oncologist as part of their treatment plan. At the time of biopsy, a fresh tumor sample was committed for this research study and prepared immediately. This subject sample was de-identified and sent to various sites for assessments: A single tumor biopsy in RNAlater was shipped to the CLIA-certified laboratory Clinical Reference Laboratory (CRL) for mRNA expression analysis using U133 Plus 2.0 GeneChip and from which the remaining mRNA was sent to Translational Genomics Research Institute (TGen) for high-performance RNA-seq analysis. A biopsy sample was sent to the Pediatric Oncology Translational Research Laboratory (POTRL) for in vitro/in vivo biology studies (Fig. 1).

### Sample quality control

To pass quality control, tumor samples were read by clinical pathology for a ≥75% viable tumor by nuclei, and <20% necrosis. Sample was then processed by CRL. The RNA extraction, amplification, Affymetrix U133 Plus 2.0 GeneChip® hybridization (Santa Clara, CA), and scanning procedures utilized CLIA-certified CRL standard protocols. Passing criteria include: (1) RNA integrity number (RIN) >6.5 using the Agilent (Waldbronn, Germany) 2100 BioAnalyzer; (2) RNA 260/280 and 260/230 absorbance ratios >1.8 by NanoDrop; (3) total cDNA yield ≥5 *μ*g/30 *μ*L; (4) cDNA 260/280 and 260/230 absorbance ratios ≥1.8 by NanoDrop. Data files were processed using the Affymetrix Expression Console™ and the MAS5.0 statistical algorithm.

### Drug prediction report

The reported drugs were predicted using microarray expression data from patient tumors which were compared to a series of normal biological controls. In this preprocessing step, each probe set was represented by a *Z*-score, which is a measure of relative expression of genes in tumor versus normal reference as described previously 19. The normal reference set is a whole body bank of 45 normal tissue gene expression levels which are used as the reference set for the normalization calculations. A whole body reference was chosen to provide a wider variance of tissue-specific gene expression for comparison in order to best identify expression differences from tumor tissue. The reference also helps to decrease toxicity risk by not identify targets that are highly expressed in normal tissues. Data were submitted to a database of algorithms designed to predict relevant medications which are then presented in a report to the molecular tumor board 18. These algorithms included; biomarker rules, drug target expression, network-based methods, drug response, and drug sensitivity signatures.

The biomarker rules method employed predefined and published rules maintained in a drug-biomarker knowledge base in which the efficacy of a specific drug has been associated with the expression of a specific molecular marker 20. Unlike the other methods described, this method has rules that predict both drug sensitivity and drug resistance based on the expression of biomarkers. The drug target expression method identifies genes overexpressed in the tumor (*Z*-scores ≥ +3) that represented a therapeutic target which was submitted and therapeutic compounds that met the rule requirement based upon their confirmed mechanism of action (MOA). The MOA of drugs and the alignment to therapeutic targets was performed using a variety of public and commercial knowledge bases including DrugBank 17, PharmGKB 21, GeneGo-Thomson Reuters (www.genego.com), UptoDate (www.uptodate.com), MedTrack (www.medtrack.com) and DrugDex (http://thomsonreuters.com/products_services/healthcare/healthcare_products/a-z/drugdex_system/) as well as extensive literature searches to confirm the drug target evidence.

The network-based methods, developed in partnership with Gene-Go-Thomson Reuters 22–24, predicted activity of drug targets is based on topological analysis. Various derivatives of this tool (referred to as the “hidden nodes” algorithms) are described in detail and freely available at http://www.genego.com/hidden_nodes.php. In brief, these systems biology based methodologies were developed to identify key regulators of the observed transcriptional profile after constructing molecular networks on the basis of prior protein–protein interaction knowledge. The key nodes (putative targets) within the identified and topologically enriched networks may be “hidden” as they do not necessarily represent genes differentially expressed in the patient's tumor. Derivatives of this methodology included the analysis of target genes that represent key points of information convergence and divergence, which can be considered putative effectors and drivers respectively. After these respective analyses, the overlay of the drug target knowledge base with topologically significant nodes provided a method to predict drug efficacy.

The drug response signatures reproduced the Connectivity Map concept initially developed by the Broad Institute 25 in which the genomic consequence of drug exposure is used to connect drug effect to disease signatures. The hypothesis underlying this method is that drugs that reverse the disease genotype (gene expression profile) toward normalcy have the potential to reverse the disease phenotype. Up to 500 of the most over and underexpressed genes in the patient's tumor (*Z*-scores ≥ +1.5 or ≤ −1.5, respectively) were submitted to this method. Rank-based statistics were used to identify drugs with a significant inverse connectivity to the disease genotype.

The drug sensitivity signatures implemented the Parametric Gene Set Enrichment Analysis method to align NCI-60 cell line sensitivity signatures that are predictive across at least two independent cell contexts with the patient's differentially expressed genes. All genes that passed the preprocessing thresholds were evaluated. The NCI-60 drug signature mapped over and under expressed genes (determined by predrug treatment) to the observed in vitro drug sensitivity as measured by the half maximal inhibitory concentration (IC50) of the various cell lines studied 26,27.

Upon execution of these analyses, a compiled report was generated. The report allowed the molecular tumor board to quickly navigate to the underlying knowledge and evidence at multiple levels, including the molecular predictions and inferring methodologies, and any evidence from published literature and clinical trials that may support the use of the predicted agent in the patient's disease context. The total FDA approved drugs with pediatric dosing available at the time of this study was 108 drugs.

### Treatment protocol decision

Treatment protocols were devised by a tumor board which consisted of pediatric oncologists, pharmacists, bioinformaticians, and pathologists utilizing the drug prediction report which was generated through analysis of the gene expression profile of the patient's tumor. The drug prediction report provided a list of potentially effective agents based on the analyses described above. Decision rules for the tumor board included: (1) All drugs with predicted efficacy were reported to the tumor board with an associated predicted efficacy score and rank. (2) Drugs chosen must be FDA approved with established standard and safe dosing schedules (see Table S3 for the clinical trial drug list). Those without known pediatric dosing were excluded. (3) Potential drug choices were analyzed with regards to safety, mechanism, availability, and cost. Focus was on low-toxicity, targeted therapies. (4) Drug combinations were allowed, up to a maximum of four agents. Literature searches were conducted to assemble data on previously established and tested regimens which were given priority. (5) The pharmacist performed analysis of possible drug interactions between the potentially effective agents and the subject's routine medications and supplements. For drug interactions and known toxicities the following databases were used: MicroMedex (Greenwood Village, CO), LexiComp (Hudson, OH), E-facts and Natural Medicines Database. (6) Patients' history and previously received treatments were reviewed. Drugs which a patient had failed were given low priority and used only if there was a rationale for synergy in combination therapy.

### Prioritization rules

The following prioritization rules were used to choose drugs for each patient's individualized treatment plan. For a given proposed combination of drugs, the first priority to establish doses was to identify the same combination of drugs in a peer-reviewed journal article or presented as a reviewed abstract, or part of an ongoing peer-reviewed clinical trial registered with clinical trials.gov. When a proposed combination of drugs had not previously been reported, dosing was established by studying how each component of the proposed combination had been combined with other cytotoxic agents similar to those being considered for combination therapy. Again, the source of information was a peer-reviewed journal article or presented as a reviewed abstract, or part of an ongoing peer-reviewed clinical trial registered with clinical trials.gov. When a proposed combination of drugs had no available combination data, dosing guidelines started with the maximum tolerated dose (MTD) determined by a phase I/II pediatric study. Per pharmacy review, doses were reduced to compensate for potential additive toxicities of combination agents.

The treatment regimens were discussed with families and included review of known side effects, serious adverse effects of possible new drug combinations and any additional clinical monitoring that might be recommended by the FDA and/or the tumor board. The families were given the option to proceed with therapy and were asked to sign a treatment-specific memo.

### Safety measures

All adverse events, whether serious or not, were described in the source documents and Grade 2 or higher (per CTCAE 4.0) adverse events were captured on the adverse event case report forms. All Grade 2 or higher new events were captured, including those that worsened in intensity or frequency relative to baseline, and those which occurred after administration of study drug through the period of protocol-specified follow up. Regardless of suspected cause, adverse events were collected for 30 days following the last treatment and any suspected study drug-related toxicities at the 30 day follow-up visit were followed until resolution to baseline or ≤Grade 2 or stabilization of the event.

### Research methods

#### RNA-sequencing research studies

RNA sequencing was performed using 1.0 µg of total RNA quantified via Nanodrop (Thermo Scientific, Pittsburgh, PA). A sequencing library was prepared with Illumina's Truseq RNA Sample Preparation Kit v2 (Illumina Inc, San Diego, CA) following the manufacturer's protocol. In brief, poly-A containing mRNA molecules were purified using poly-T oligo attached magnetic beads. The mRNA was then thermally fragmented and converted to double-stranded cDNA. The cDNA fragments were end-repaired, a single “A” nucleotide was incorporated, sequencing adapters were ligated, and fragments were enriched with 15 cycles of PCR. Final PCR-enriched fragments were validated on a 2100 Bioanalyzer (Agilent Technologies, Waldbronn, Germany) and quantified by qPCR using Kapa's Library Quantification Kit (Kapa Biosystems, Woburn, MA) on the 7900HT (Applied Biosystems, Foster City, CA). The final library was sequenced by 50 bp paired-end sequencing on a HiSeq2000 (Illumina, San Diego, CA).

Raw reads passing Illumina quality filters were converted to FASTQ format in Phred33 scale with CASAVA 1.8.3. RNA-Seq reads were aligned with TopHat (v2.0.8) 28 which first utilizes Bowtie (v2.1.0.0) 29 to map reads with “splice-aware” alignments to the Homo Sapiens build GRCh37 from Ensembl 30. To estimate the library fragment size for TopHat, we initially mapped a subset of 1 million reads with bwa (v0.6.1) to the human genome, followed by picard version 1.80 31 module CollectInsertSizeMetrics and provided these values to TopHat options “–mate-inner-dist 87 –mate-std-dev 86.” Additional TopHat flags utilized were –transcriptome-index (to Ensembl GRCh37.70), –no-coverage-search, –b2-sensitive and –keep-fasta-order. Next, we calculated gene expression values expressed as fragments per kilobase pair of exon per million fragments mapped using cufflinks version 2.1.1 28. We used the –GTF option in cufflinks to annotate to human gene models GRCh37.70. Additionally we used the –multi-read-correct and –frag-bias-correct options in cufflinks and masked tRNAs, rRNAs, and mtRNAs as suggested in the cufflinks documentation.

#### Ion torrent

Ion torrent deep amplicon sequencing of tumor samples: deoxyribonucleic acid was extracted from tumor tissues and quantitated using the Qubit2 fluorometer (Invitrogen, Grand Island, NY). Ten nanograms of DNA was used for multiplex PCR of a panel covering 739 mutations in 46 cancer-related genes (Ion AmpliSeq Cancer Panel, Life Technologies, Grand Island, NY). Subsequent processing of samples was performed according to the manufacturer's protocol. Library constructions of the amplicons and subsequent enrichment of the sequencing beads was performed using the OneTouch (Grand Island, NY) system. Sequencing was done on the 314 chip with 10 megabases capacity using the Ion Torrent Personal Genome Machine (Life Technologies) as per the manufacturer's protocol. Data analysis, including alignment to the hg19 human reference genome and base calling, was done using built-in software.

## Results

### Feasibility and safety

The primary objective of this study was to evaluate the feasibility and safety of a process using predictive modeling based on genome-wide mRNA expression profiles of neuroblastoma tumor biopsies to make real-time treatment decisions. Feasibility was defined as “completion of enrollment onto study, quality mRNA obtained, gene chip completed, tumor board held, medical monitor review and approval, start of treatment by 21 days post biopsy/surgical resection date, and completion of 1 cycle of therapy.”

**Figure 2:**
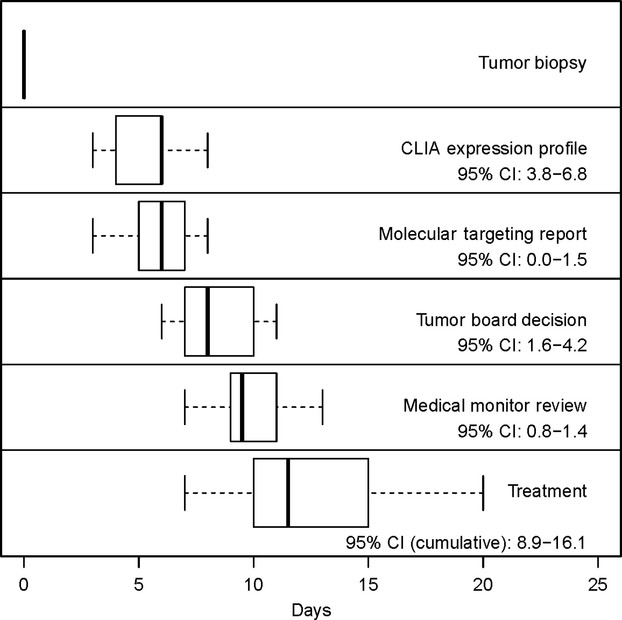
Box-and-whisker representation of the completion times (Days) for each step in the study process relative to the date of biopsy. The median, interquartile range, and range are represented by the central band, box, and whiskers, respectively.

There were 16 subjects enrolled with multiply relapsed or refractory neuroblastoma of which 14 were eligible: eight males and six females with a median age of 10.1 years (see Table 1A). Subjects were between 1–11 years post diagnosis. The patients presented with actively progressing neuroblastoma and had exhausted relapse therapies (see Table S1). All subjects had soft tissue disease in which biopsy was possible. All biopsies were adequate by pathology evaluation (>75% viable tumor) and RNA quality (>6.5 RIN). Two subjects were deemed ineligible due to benign tumor type after biopsy, therefore 14 subjects were eligible to remain on study. Gene chips were completed in 3–8 days (95% CI: 3.8–6.8), report generation took 0–3 days (95% CI: 0.0–1.5), tumor board took 1–6 days (95% CI: 1.6–4.2), medical monitor sign off took 1–2 days (95% CI: 0.8–1.4). The total time from date of biopsy to tumor board was 6–11 days (95% CI: 7.5–10.2) for all subjects and 7–20 days to treatment (95% CI: 8.9–16.1) (Fig. 2). The tumor board successfully created individualized therapy regimens for all subjects. Patients received between 2–4 drugs chosen from the predicted list. All patients completed at least one cycle of therapy, resulting in 100% feasibility.

**Table 1:** Clinical trial patient data

	*N*
(A) Patient characteristics	
Patients enrolled	14
Male	8
Female	6
Median age at enrollment (range)	10.1 (5–22)
Median age at diagnosis	4
Race	
Caucasian	11
Black or African American	1
Other	2

Disease status on entry	Response		Bone marrow response	>15% LDH decrease	>15% VMA decrease	Median PFS (95% CI)	Years post diagnosis (range)	No. of previous treatments (range)	No. of cycles completed (range)
(B) Response assessment									
PD 100% (14/14)	PD	36% (5/14)	7% (1/4)	42% (6/14)	50% (7/14)	59 (43)	4.75 (1–11)	5.71 (1–18)	3.07 (1–7)
	PR	7% (1/14)							
	SD	57% (8/14)							

PFS, progression free survival; PD, progressive disease; PR, partial response; SD, stable disease.

There were no serious adverse events reported on this study. The most common adverse events were the effects on bone marrow (neutropenia, anemia, thrombocytopenia) see Table 2A. These adverse events are expected with chemotherapy, generally occurring in greater than 50% of patients receiving standard chemotherapy for neuroblastoma 32. In this study, the incidence of grade 3 and 4 events was found to be neutropenia (43%), anemia (14%), and thrombocytopenia (36%).

**Table 2:** Adverse events

	No. subjects (*n* = 14)		
	Grade		
Adverse event	2	3	4
(A) Related adverse events by event name			
Abdominal pain	1 (7%)		
ALT elevated		2 (14%)	
AST elevated	1 (7%)	1 (7%)	
Anemia	4 (29%)	2 (14%)	
Bilirubin increase		2 (14%)	
Constipation	1 (7%)		
Dehydration	1 (7%)		
Fatigue	1 (7%)		
Fever	1 (7%)		
Hypoalbuminemia	1 (7%)	1 (7%)	
Hypocalcemia	1 (7%)		
Hypophosphatemia	1 (7%)		
Infection		2 (14%)	
Leukopenia	3 (21%)	3 (21%)	1 (7%)
Lymphocytopenia	1 (7%)	2 (14%)	
Mucositis	2 (14%)		
Myalgia	1 (7%)		
Nausea	1 (7%)		
Neutropenia		1 (7%)	6 (43%)
Pain	2 (14%)		
Rash		1 (7%)	
Tachycardia	1 (7%)		
Thrombocytopenia	1 (7%)	1 (7%)	6 (43%)
Vomiting	1 (7%)		
Weight loss	1 (7%)		

Eligible pt no.	Drug and dose chosen	Targets	Method used to choose drug	Cycles completed	Related adverse events experienced (grade)
(B) Targeted therapeutic recommendations with adverse events by individual patient/drug combination					
MGT-002-13	Topotecan 0.75 mg/m^2^ per dose	TOP1	Drug target expression	1	Thrombocytopenia (4)
	Bupropion 1.5 mg/kg per day	SLC6A2	Drug target expression		
	Radiation	–	–		
MGT-003-08	Bortezomib 1 mg/m^2^ per dose	NFKB1, NFKB2	Network target activity	3	ALT increase (3)
	Vorinostat 230 mg/m^2^ per day	HDAC1, HDAC2, HDAC4	Drug response signatures		AST increase (2)
			Network target activity		Leukopenia (3)
			Drug target expression		
	Doxorubicin 30 mg/m^2^ per dose	TOP2A	Drug target expression		Neutropenia (4)
					Thrombocytopenia (4)
MGT-004-13	Bevacizumab 625 mg/day	VEGFA	Network target activity	1	Fever (2)
	Vorinostat 300 mg/day	HDAC1	Drug response signatures		
			Network target activity		
MGT-006-13	Topotecan 1 mg/m^2^ per dose	TOP1	Drug target expression	1	Leukopenia (4)
	Bupropion 1.5 mg/kg per day	SLC6A2	Drug target expression		Lymphopenia (3)
	Vinblastine 3 mg/m^2^ per dose	TUBB	Drug target expression		Neutropenia (4)
	Zometa 4 mg/m^2^ per dose	FDPS	Drug target expression		Elevated Bilirubin (3)
	Max: 4 mg				Pain (Esophagus) (2)
					Thrombocytopenia (4)
MGT-007-04	Bortezomib 1.3 mg/m^2^ per dose	AKT1, NFKB1	Network target activity	2	Pain (abdominal) (2)
			Drug target expression		Neutropenia (3)
	Vorinostat 230 mg/m^2^ per day	HDAC1, HDAC3, HDAC7	Network target activity		Anemia (2)
			Drug target expression		Myalgia (2)
	Doxorubicin25 mg/m^2^ per dose	TPO2B	Drug sensitivity signatures		Leukopenia (2)
			Network target activity		Thrombocytopenia (2)
	Simvastin 20 mg/day	IGF1, RHOA	Network target activity		
			Drug target expression		
MGT-008-08	Donepezil 5 mg QOD	ACHE	Drug target expression	6	Anemia (2)
	Vorinostat 230 mg/m^2^ per day	HDAC2, HDAC6	Drug target expression		Leukopenia (3)
	Vinblastine 4 mg/m^2^ per dose	TUBB	Drug target expression		Neutropenia (4)
	Zometa 4 mg/m^2^ per dose	FDPS	Drug target expression		Thrombocytopenia (4)
	Max: 4 mg				
MGT-009-08	Bortezomib 1.3 mg/m^2^ per dose	AKT1, NFKB1, NFKB2	Network target activity	1	Constipation (2)
			Drug target expression		Dehydration (2)
	Sorafenib 150 mg/m^2^ per dose BID	RET	Drug target expression		Hypoalbunemia (3)
	Doxorubicin 30 mg/m^2^ per dose	TOP2A, TOP2B	Drug sensitivity signatures		Hypophosphatemia (2)
			Network target activity		Nausea (2)
			Drug target expression		Thrombocytopenia (4)
MGT-010-08	Cytarabine 50 mg/m^2^ per dose	SLC29A1	Biomarker-based rules	2	Neutropenia (4)
	Sorafenib 200 mg/m^2^ per BID	PDGFRB, FLT3, FLT4, RET	Network target activity		Anemia (4)
			Drug target expression		Thrombocytopenia (4)
					Leukopenia (4)
					Pain (2)
MGT-011-13	Vorinostat 230 mg/m^2^ per day	HDAC3, HADC6	Network target activity	5	Neutropenia (2)
			Drug target expression		Anemia (3)
	Sorafenib 200 mg/m^2^ per day	PDGFRB, RET	Network target activity		Hypoalbunemia (2)
			Drug target expression		Thrombocytopenia (3)
	Vinblastine 4 mg/m^2^ per dose	TUBB	Drug target expression		Vomiting (2)
					Leukopenia (3)
					Lymphocytopenia (3)
					Tachycardia (2)
MGT-012-08	Lapatinib 700 mg/m^2^ per dose BID	EGFR, ERBB2	Network target activity	5	ALT increase (3)
	Doxycycline 4 mg/kg per day	MMP3, MMP9, IL1A, TNF	Network target activity		AST increase (2)
			Drug target expression		Hypokalemia (3)
	Mitoxantrone 12.5 mg/m^2^ per dose start Cycle 2	TOP2A, DHFR	Drug target expression		Pain-Stomach (2)
	Radiation- Cycle 1 only	—	—		Rash (2)
MGT-013-08	Bortezomib 1.3 mg/m^2^ per dose	AKT1, NFKB1	Network target activity	1	ALT increase (3)
	Sorafenib 200 mg/m^2^ per dose BID	RAF1, KDR, FLT1, KIT	Network target activity		AST increase (3)
	Doxycycline 2 mg/kg per dose BID	MMP9, MMP13, IL1A, TNF	Network target activity		Anemia (3)
			Drug target expression		Elevated Bilirubin (2)
					Hypocalcemia (2)
					Leukopenia (2)
					Mucositis (2)
MGT-014-11	Vinblastine 3.7 mg/m^2^ per dose	TUBB	Drug target expression	2	Anemia (2)
	Sorafenib 400 mg BID	RET, RAF1	Network target activity		Fatigue (2)
			Drug target expression		Pain-Skin (2)
	Bupropion 100 mg BID	SLC6A2	Drug target expression		Leukopenia (2)
					Lymphocytopenia (2)
					Neutropenia (4)
					Rash (3)
					Weight loss (2)
MGT-015-08	Topotecan 0.75 mg/m^2^ per dose	TOP1	Drug target expression	6	Neutropenia (4)
	Sorafenib 160 mg/m^2^ per dose BID	RET	Drug target expression		Thrombocytopenia (4)
	Vorinostat 230 mg/m^2^ per day	HDAC4	Drug target expression		Anemia (2)
	Doxycycline 4 mg/kg per day	MMP9, MMP13	Drug target expression		Anorexia (2)
					Weight loss (2)
					Sinus Infection (2)
MGT-016-08	Doxorubicin 30 mg/m^2^ per dose	TOP2A	Drug sensitivity signatures	7	Rash (2)
			Drug target expression		Neutropenia (4)
	Sorafenib 200 mg/m^2^ per dose BID	RET, FLT1, PDGFRB	Network target activity		Anemia (2)
			Drug target expression		Fungal Pneumonia (3)
	Vorinostat 230 mg/m^2^ per dose	HDAC2, HDAC3, HDAC6	Network target activity		Bacterial Blood Infection (3)
			Drug target expression		Mucositis (2)
		Doxycycline 4 mg/kg per day	MMP1, MMP9, MMP13	Network target activity	
				Drug target expression	

ALT, alanine transaminase; AST, aspartate aminotransferase.

### Response and PFS

Of the 14 patients enrolled on study, 100% of patients had PD as indicated by radiologic imaging prior to study entry. All patients were able to complete one cycle of molecular-guided therapy and were evaluable for response. There was one patient who met PR criteria with a greater than 50% decrease in brain lesions by MRI (7%), 8/14 had stable disease (57%) and 5/14 had PD (36%) (Table 1B).

The median PFS from entry onto study was 59 days with a lower 95% confidence interval of 43 days (Table 1B).

### RNA expression and sequencing

#### Reproducibility of profiling

**Figure 3:**
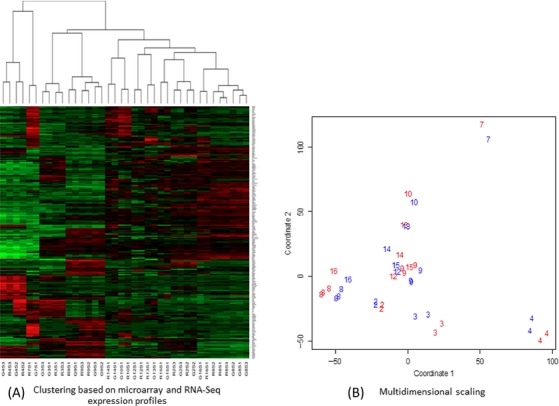
Exploratory multivariate analysis of combined microarray and RNA-Seq gene expression profiles. (A) Heat map and sample dendrogram. Red indicates relatively high expression while green indicates relatively low expression. The first character of the sample label indicates a GeneChip (G) or an RNA-Seq (R) profile, the following integer indicates the biopsy, and the final two characters (e.g., S3) indicate the biopsy section. (B) Multidimensional scaling. Samples are represented by their biopsy number, colored by the technology (GeneChip, red; RNA-Seq, blue).

A reproducibility study was performed within the study to evaluate the variation among multiple biopsy sections from the same tumor. Expression profiling and drug predictions based on triplicate sections were analyzed. Distance-based nonparametric multivariate analysis of variance 33,34 allowed us to reject the null hypothesis that variation between biopsies can be accounted for by the variation within biopsies (*P* = 0.001). That the variation among expression profiles associated with the same biopsy is small compared with the variation between expression profiles associated with different biopsies is also apparent from Multidimensional Scaling (Fig. 3; 19). Similarly, the variation among drug sets associated with the same biopsy was small compared with the variation among drug lists associated with different biopsies (*P* = 0.001). The reproducibility averaged over patients, replicates, and drugs is 0.68. As the threshold score increased to score >10, the reproducibility increased to 1 35. Table S2 provides the RNA expression profiles for study patients.

#### Comparison between RNA expression profiling and RNA sequencing

**Figure 4:**
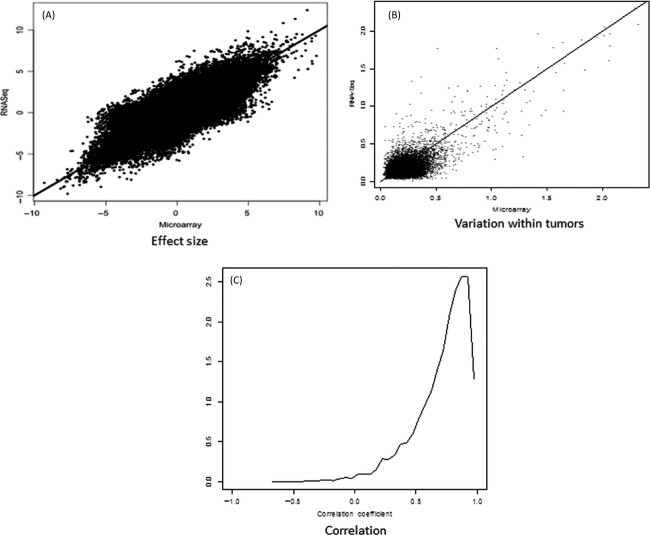
Comparison of microarray and RNA-Seq gene expression statistics. (A) Effect size, expressed as log_2_(fold change), estimated using microarray or RNA-Seq. Each point corresponds to a gene and a pair of samples. The line corresponds to agreement of the two technologies. (B) Variation within tumors, expressed as the standard deviation, estimated using microarray or RNA-Seq. Each point corresponds to a gene. The line corresponds to agreement. (C) Correlation of each microarray expression profile of a gene across samples with the RNA-Seq profile.

Differences between samples from the same patient (arising either from differences between biopsy sections or from differences between oligonucleotide microarrays and sequencing) is shown to be much smaller than differences between patients (Fig. 3). We found agreement between RNA sequencing and gene chip differential expression levels (Fig. 4). Analysis of the variation within biopsy suggests that it is dominated by biology and not the technology (Fig. 4B). The correlation between gene expression profiles is high (Fig. 4C). Oligonucleotide microarrays and RNA-Seq mutually validate.

### Ion torrent analysis

While not included in the decision-making process in this clinical trial, the Ion Torrent Cancer Panel gene chip was performed to assess use in future studies. One actionable mutation was found, (7% of patients), which was in the ALK gene and was validated by Sanger sequencing. In this study, actionable mutations are defined as: “mutations which can be targeted by an existing drug as reported in the current body of evidence.”

## Discussion

The benefits of a molecular-guided treatment plan are easy to conceptualize: a more targeted approach, a reduction in unnecessary interventions, and the potential for improved outcomes. To date, there have been significant barriers to this approach: the amount of time necessary for genomic profiling, the ability to identify actionable targets, the availability of therapies to act on those targets, and the need for rebiopsy. This study is the first completed pediatric clinical trial in the US which evaluates the feasibility and safety of using a TPAP based on genome-wide mRNA expression profiles of neuroblastoma tumor biopsies to create individualized therapeutic regimens.

As we enter an era where individualized medicine is increasingly possible, a high degree of cooperation among many disciplines will be critical. Oncologists, bioinformaticians, geneticists, pharmacists, pathologists, information services, and computational experts will provide key input to the discussion of the target gene and its role in molecular-guided therapy. This will enable the creation of individualized treatment plans which more effectively target the disease.

Predictive biomarkers may be based on any of a variety of molecular features; possibilities include genomic sequence, epigenetic modification, transcription, protein expression, posttranslational modification, and metabolite profiles. FDA-approved companion diagnostics used in the treatment of adult cancers have been based on DNA sequence (for example, BRAF V600E/K) or on protein expression (for example, HER2/neu). An important hypothesis underlying our work is that expression technologies will supplement DNA sequence and protein expression information by quantifying the summary effects of genetic and epigenetic drivers genome-wide.

The primary endpoint of this study was to determine the feasibility of using this process (TPAP) for the treatment of children with neuroblastoma. We have shown that this was feasible in all 14 patients. Initially, there was a concern with regard to the amount of time required for profiling and the generation of a tumor board treatment plan. However, the mean of 12 days was sufficient: no patients experienced significant disease progression prior to initiation of therapy.

The second primary endpoint of safety for this study showed that there were no serious or unexpected adverse events. The events seen were those typically seen in children with neuroblastoma receiving the medications prescribed. Our observation is that the approach used in this study appeared to result in less severe side effects than we have observed in children who receive nontargeted therapy for relapsed disease and warrants further evaluation in a larger study.

As all patients had shown radiological progression of disease prior to study enrollment, the expectation would be continued progression if the molecular-guided therapy were not effective. In this heavily pretreated patient population, stabilization of disease in 57% and response in 7% may suggest benefit and should be further studied. The combined clinical benefit in 64% of patients suggests an improvement over the 17–48% combined benefit of recent Phase I neuroblastoma studies 36–41.

The clustering analysis demonstrates that genetic differences occur even within the same class of tumor, emphasizing the need for personalized and highly targeted therapies. In addition, patients may group into “treatment clusters,” which may lead to novel clinical trial designs that classify patients to a particular treatment plan based on genomic expression differences. The regimens chosen in this study suggest that treatment clusters may occur. Certain medications emerged repeatedly from the drug prediction report: vorinostat (HDAC overexpression), and sorafenib (RET overexpression) were each used in eight of 16 patients (see Table 2B). A larger patient sample would be required to test this.

Another important aspect of this study was the importance of biopsy. Biopsy of one patient revealed a neuroendocrine carcinoma which had been incorrectly diagnosed as neuroblastoma. This subject was allowed to remain on study. Biopsy of two other patients revealed ganglioneuroma (benign tumor) making them ineligible for this study. One patient was enrolled a second time with biopsy revealing that genomic differences had occurred between relapses, suggesting that prior therapy may have had an impact which would have been undetected without biopsy: this subject counted as two separate encounters in the enrollment numbers. These examples clearly emphasize the need for rebiopsy at relapse for all patients since 3/16 (19%) would have been inappropriately treated without biopsy. Rebiopsy has not been favored due to ethical considerations of an unnecessary procedure. Yet, in this study, rebiopsy revealed critical information about 3/16 patients who would have been misdiagnosed or inappropriately treated. In addition, this study demonstrates that rebiopsy can safely be performed with minimal risk as there were no adverse events associated with any patient biopsies.

A reproducibility analysis of triplicate biopsy sampling was undertaken during this study. This showed significant correlation in overall expression profiling as well as drug predictions confirmed in RNA Sequencing. High-throughput Sequencing (HTS) to determine changes in gene expression is rapidly becoming a viable choice and is referred to as RNA-seq. The methods studied appear to mutually validate each other and therefore either could be used in the same context (such as drug prediction). RNA sequencing may add further understanding through identification of gene fusions or possibly greater sensitivity. As such, RNA sequencing may provide greater transcriptome coverage, and further allow complete annotation and quantification of all genes and their isoforms in a given sample. An important development during this study was that previously RNA sequencing required up to 2 months but has now been optimized to completion in 2 weeks in a CLIA-certified laboratory. As we move toward deeper RNA-Seq, we chose to evaluate this in comparison to RNA expression profiling and found that these methods do correlate in patient samples.

We also evaluated the ability of the Ion Torrent DNA mutation panel to find actionable mutations in our patients for incorporation into future studies. We found that 7% of patients in this small sample size had identified actionable mutations. This was in the low range of the literature reports of ∽10–22% actionable mutation rate in adults 42. The actionable mutation identified was ALK, which has been identified in 7% of neuroblastoma patients 43 and ALK inhibitors, such as Crizotinib are currently being tested in pediatrics. This method was validated with Sanger sequencing, although this should continue to be evaluated in a larger sample set to show statistical power prior to recommending this test alone. This method was integrated into the decision-making process for the tumor board in the follow-up clinical trial.

Understanding of known genetic mutations and their effects on therapeutic choices such as undertaken in this trial will help us gain the knowledge to improve predictions. With the establishment of patient cell lines and mice models in over 50% of cases it is possible to study drug effectiveness in vitro and in vivo. Future directions include an ongoing validation study using patient-derived cell lines and mice models to improve drug prediction algorithms.

The future of oncology lies in a process using data-driven genetic and mechanistic understanding of patients' tumors for choosing therapies. A better understanding of tumor-specific information will pave the way for individualized, targeted treatment plans. The continued development of a TPAP will allow improved and more accurate predictions in the future. We believe that this study is an initial step pointing the way toward future advances in molecular-guided therapy which will improve the selection of treatment options and open new avenues of investigation.

## Supporting Information

**Table S1.** Enrollment characteristics and previous relapse therapies prior to enrollment.

**Table S2.** Oligonucleotide microarray gene expression data. Each row is a probe set on the Affymetrix GeneChip® Human Genome U133 Plus 2.0 Array. Columns are labelled <Enrollment ID>. <Feature> where <Enrollment ID> is one of 15 enrollment identifiers (‘MGT-001-13’ … ‘MGT-016-08’) and <Feature> reflects whether it was included in the analysis (‘MAS5’), whether it was called present or absent (‘PMed’), the MAS5 expression statistic (‘Raw’) and the Z-score (‘ZScore’).

**Table S3.** Clinical trial drug list.
