# Supplementary material for: Feasibility of implementing molecular-guided therapy for the treatment of patients with relapsed or refractory neuroblastoma
Source: Cancer Med. 2015 Feb 26;4(6):871–86. doi: 10.1002/cam4.436 (PMC4472210; doi:10.1002/cam4.436)
Supplement: Supplementary file 1 [file cam40004-0871-sd1.pdf]

Patient #	Age at study entry	MYCN	Relapsed/ Refractory	Disease Status at entry	Number of previous relapse therapies	Previous Relapse Treatments
MGT-002-13	12	Neg	Refractory	Progressing	16	1. MIBG therapy<br>2. Cyclophosphamide/Topotecan/ Vincristine/3F8<br>3. Temodar/Irinotecan/3F8<br>4. Cyclophosphamide/topotecan<br>5. 3F8<br>6. Etoposide/Carboplatin/ Irinotecan/Temodar/Cyclophosphamide<br>7. ABT-751<br>8. Cyclophosphamide/Topotecan<br>9. TPI-287/Etoposide<br>10. Velcade/Cyclophosphamide<br>11. NK cell transplant<br>12. Velcade/Etoposide<br>13. DFMO/Etoposide<br>14. Vinblastine/Rapamycin<br>15. Nifurtimox/Cyclophosphamide /Topotecan<br>16. Cisplatin
MGT-003-08	8	Pos	Relapsed	Progressing	7	1. Cyclophosphamide/Topotecan/ Vincristine<br>2. Ifosphamide/Etoposide<br>3. Irinotecan/Temozolomide/Radiation<br>4. MIBG therapy with stem cell rescue<br>5. Irradiation followed by Irinotecan/Temozolomide<br>6. Ifosphamide/Carboplatin/Etoposide with stem cell rescue<br>7. Nifurtimox/Cyclophosphamide /Topotecan

MGT-004-13	18	Neg	Relapsed	Progressing	8	1. Cixutumumab<br>2. Irinotecan/temozolomide<br>3. Ifosphamide/Carboplatin/Etoposide<br>4. Irinotecan/Temozolomide/Bevacizumab<br>5. Stem cell transplantation (conditioning Thiotepia/ Cyclophosphamide)<br>6. Stem cell transplant (conditioning Carboplatin/Etoposide/Melphalan) and cis-retinoic acid<br>7. Cyclophosphamide /Topotecan<br>8. Bevacizumab/Irinotecan/Temozolomide
MGT-006-13	10	Neg	Refractory	Progressing	5	1. ANBL0532<br>2. temodar and irinotecan<br>3. I-131 MIBG<br>4. oral etoposide<br>5. radiation
MGT-007-04	12	Pos	Refractory	Progressing	3	1. Irinotecan/Temozolomide<br>2. Cyclophosphamide/Topotecan<br>3. Accutane
MGT-008-08	5	Pos	Refractory	Progressing	5	1. Irinotecan/temozolomide<br>2. Avastin/Irinotecan/Temozolomide<br>3. Humanized 3F8 antibody<br>4. Ifosphamide/Carboplatin/Etoposide<br>5. MIBG Therapy
MGT-009-08	6	Neg	Refractory	Progressing	1	1. Cyclophosphamide/topotecan
MGT-010-08	8	Pos	Refractory	Progressing	7	1. Topotecan/Vincristine/Doxorubicin<br>2. Irinotecan/Temozolomide<br>3. MIBG therapy<br>4. Busulfan/Melphalan

						conditioning followed by stem cell rescue<br>5. Immunotherapy with CH 14.18/CHO monoclonal antibody and Interleukin 2<br>6. Irinotecan/Temozolomide<br>7. Cyclophosphamide/Topotecan
MGT-011-13	9	Neg	Refractory	Progressing	5	1. MIBG Therapy<br>2. Irinotecan/Temozolomide<br>3. Ifosphamide/Carboplatin/Etoposide<br>4. Cyclophosphamide/ Topotecan<br>5. Nifurtimox/Cyclophosphamide/Topotecan
MGT-012-08	12	Neg	Relapsed	Progressing	8	1. Radiation followed by Irinotecan/Temozolomide<br>2. Cyclophosphamide/Topotecan<br>3. MIBG Therapy<br>4. ABT-751<br>5. MLN-8237<br>6. Crizotinib<br>7. Radiation followed by Irinotecan/Temozolomide<br>8. DFMO/Etoposide
MGT-013-08	7	UNK	Relapsed	Progressing	14	1. Vincristine/Topotecan/Cyclophosphamide followed by radiation<br>2. Irinotecan/Temozolomide<br>3. Vincristine/Topotecan/Cyclophosphamide<br>4. Maternal NK Cells<br>5. 3F8 antibody<br>6. MIBG Therapy/RA-223<br>7. Ifosphamide/Carboplatin/Etoposide<br>8. ch14:18 Antibody<br>9. Etoposide/radiation<br>10. Irinotecan/temozolomide<br>11. Irinotecan/radiation

						12. Cyclophosphamide/topotecan<br>13. Fenretinide<br>14. Ifosphamide/Carboplatin/Etoposide with Stem Cell rescue
MGT-014-11	22	Neg	Refractory	Stable	7	1. Cyclophosphamide/Topotecan<br>2. MIBG therapy with stem cell transplant<br>3. Accutane<br>4. Etoposide<br>5. POG#9754- methotrexate/ dexrazoxane/ doxorubicin/cisplatin (secondary osteosarcoma)<br>6. Irinotecan/temodar/TPI-287<br>7. Nifurtimox/Cyclophosphamide/Topotecan
MGT-015-08	7	Pos	Relapsed	Progressing	2	1. Vinblastine/Rapamycin<br>2. DFMO
MGT-016-08	5	Pos	Refractory	Progressing	6	1. Irinotecan/temozolomide<br>2. Avastin/Irinotecan/Temozolomide<br>3. Humanized 3F8 antibody<br>4. Ifosphamide/Carboplatin/Etoposide<br>5. MIBG Therapy<br>6. Vinblastine/Vorinostat/Zometa/Donepezil

Supplementary Table #1 Enrollment Characteristics and Previous Relapse Therapies prior to Enrollment
