# Supplementary material for: Feasibility of implementing molecular-guided therapy for the treatment of patients with relapsed or refractory neuroblastoma
Source: Cancer Med. 2015 Feb 26;4(6):871–86. doi: 10.1002/cam4.436 (PMC4472210; doi:10.1002/cam4.436)
Supplement: Supplementary file 2 [file cam40004-0871-sd2.pdf]

Probe_Set_I	MGT-001-13_	MGT-001-13_	MGT-001-13_	MGT-001-13_	MGT-002-13_	MGT-002-13_	MGT-002-13_	MGT-002-13_	MGT-003-08_	MGT-003-08_	MGT-003-08_	MGT-003-08_	MGT-004-13_	MGT-004-13_	MGT-004-13_	MGT-004-13_	MGT-006-13_	MGT-006-13_	MGT-006-13_	MGT-006-13_	MGT-007-04_	MGT-007-04_	MGT-007-04_	MGT-007-04_	MGT-008-08_
1007_s_at	included	present	377.49	-0.94	included	present	470.07	-0.83	included	present	183.71	-1.18	included	present	2143.8	1.24	included	absent	78.54	-1.31	included	present	497.53	-0.79	included
1053_at	excluded	absent	205.08	1.23	included	present	507.26	5.28	included	present	441.68	4.4	included	present	775	8.87	included	present	473.32	4.83	included	present	213.53	1.35	included
117_at	included	present	908.52	2.24	included	absent	62.11	-0.99	included	present	178.32	-0.55	included	absent	110.73	-0.81	included	absent	184.9	-0.52	included	present	609.31	1.1	included
121_at	excluded	absent	79.58	-0.71	excluded	absent	205.43	0.03	excluded	absent	113.13	-0.51	excluded	absent	129.99	-0.41	excluded	absent	151.87	-0.28	excluded	absent	148.08	-0.31	included
1255_g_at	included	present	671.91	2.67	included	present	1428.55	6.29	excluded	absent	84.68	-0.14	excluded	absent	18.56	-0.45	included	present	726.82	2.93	excluded	absent	50	-0.3	included
1294_at	included	present	371.48	-0.23	included	absent	24.91	-1.18	included	absent	43.94	-1.13	included	absent	107.22	-0.96	included	absent	40.62	-1.14	included	present	1459.81	2.75	included
1316_at	included	present	713.88	1.02	included	present	368.63	-0.48	included	present	328.98	-0.65	included	present	400.4	-0.34	included	present	379.44	-0.43	included	present	711.61	1.01	included
1320_at	included	present	665.87	1.21	included	present	516.89	0.63	included	present	786.62	1.69	included	present	152.32	-0.81	included	present	514.99	0.62	included	present	293.19	-0.26	included
1405_i_at	included	present	310.4	0.02	included	present	228.97	-0.13	excluded	absent	8.01	-0.52	excluded	absent	56.34	-0.44	included	present	283.64	-0.03	included	present	284.94	-0.03	included
1431_at	included	present	90.74	-0.15	included	present	274.11	-0.12	included	present	475.33	-0.09	excluded	absent	58.26	-0.16	excluded	absent	67	-0.16	included	present	243.73	-0.13	excluded
1438_at	excluded	absent	55.55	0.96	excluded	absent	7.95	-0.72	excluded	absent	9.35	-0.67	excluded	absent	16.04	-0.44	excluded	absent	24.85	-0.12	excluded	absent	130.59	3.61	excluded
1487_at	included	absent	171.95	-0.35	included	absent	58.29	-0.87	included	absent	103.73	-0.66	included	absent	86.56	-0.74	included	absent	223.58	-0.12	included	present	285.52	0.17	included
1494_f_at	excluded	absent	8.61	-0.17	excluded	absent	4.97	-0.17	excluded	absent	89.18	-0.15	excluded	absent	63.82	-0.15	excluded	absent	11.26	-0.16	excluded	absent	9.58	-0.17	excluded
1552256_a_a	excluded	absent	97.44	-0.14	excluded	absent	28.67	-0.26	excluded	absent	110.01	-0.11	excluded	absent	162.82	-0.02	excluded	absent	32.09	-0.26	excluded	absent	132.62	-0.07	included
1552257_a_a	included	present	164.09	-1.42	included	present	345.7	-0.34	included	present	491.65	0.52	included	present	290.41	-0.67	included	present	353.83	-0.29	included	present	257.29	-0.87	included
1552258_at	excluded	absent	169.81	1.09	excluded	absent	85.05	-0.19	excluded	absent	43.01	-0.83	included	present	73.27	-0.37	excluded	absent	124.51	0.4	included	present	187.22	1.35	excluded
1552261_at	excluded	absent	6.08	-0.63	excluded	absent	22.84	-0.37	excluded	absent	53.34	0.1	excluded	absent	49.87	0.05	excluded	absent	5.36	-0.64	excluded	absent	8.41	-0.59	excluded
1552263_at	included	present	99.39	-0.27	excluded	absent	3.14	-1	included	present	92.19	-0.33	excluded	absent	64.21	-0.54	excluded	absent	110.78	-0.19	included	present	271.73	1.03	excluded
1552264_a_a	included	present	505.03	-0.34	included	present	366.38	-0.86	included	present	295.69	-1.12	included	present	437.27	-0.6	included	present	396.37	-0.75	included	present	790.51	0.71	excluded
1552266_at	excluded	absent	112.72	-0.25	excluded	absent	45.22	-0.31	excluded	absent	171.72	-0.21	excluded	absent	65.21	-0.29	excluded	absent	238.75	-0.16	excluded	absent	79.35	-0.28	excluded
1552269_at	excluded	absent	41.85	-0.22	excluded	absent	78.74	-0.19	excluded	absent	4.86	-0.26	excluded	absent	109.22	-0.16	excluded	absent	14.24	-0.25	excluded	absent	45.75	-0.22	excluded
1552271_at	excluded	absent	15.37	-0.43	excluded	absent	32.12	0.21	excluded	absent	3.89	-0.87	excluded	absent	8.77	-0.69	excluded	absent	4.87	-0.84	excluded	absent	12.71	-0.54	excluded
1552272_a_a	excluded	absent	78.88	0.54	excluded	absent	9.47	-0.85	excluded	absent	70.01	0.37	excluded	absent	21.53	-0.6	excluded	absent	50.86	-0.02	excluded	absent	76.77	0.5	excluded
1552274_at	included	present	536.06	-0.37	included	absent	107.37	-0.8	included	present	197.13	-0.71	included	present	179.11	-0.73	included	present	491.25	-0.41	included	present	434.45	-0.47	included
1552275_s_a	included	present	512	-0.48	included	present	238.44	-0.76	included	present	406.44	-0.59	included	present	182.2	-0.81	included	present	710.34	-0.28	included	present	748.24	-0.24	included
1552276_a_a	excluded	absent	91.46	0.09	excluded	absent	162.56	1.23	excluded	absent	6.81	-1.27	excluded	absent	38.6	-0.76	excluded	absent	146.1	0.97	excluded	absent	109.49	0.38	excluded
1552277_a_a	included	present	987.53	1.74	included	present	1235.73	2.76	included	present	1876.69	5.4	included	present	865.22	1.24	included	present	1339.18	3.19	included	present	753.72	0.78	included
1552278_a_a	excluded	absent	85.35	-0.01	excluded	absent	34.89	-0.49	excluded	absent	113.13	0.26	excluded	absent	6.23	-0.77	included	present	204.91	1.14	excluded	absent	7.86	-0.75	included
1552279_a_a	excluded	absent	155.23	0.47	excluded	absent	126.35	0.21	excluded	absent	118.23	0.14	excluded	absent	66.66	-0.3	excluded	absent	154.1	0.46	excluded	absent	133	0.27	included
1552280_at	excluded	absent	22.66	-0.2	excluded	absent	63.27	-0.08	excluded	absent	4.93	-0.26	excluded	absent	29.91	-0.18	included	present	237.74	0.45	excluded	absent	61.54	-0.08	excluded
1552281_at	excluded	absent	55.24	-0.2	excluded	absent	13.08	-0.49	excluded	absent	65.54	-0.13	excluded	absent	75.03	-0.07	excluded	absent	16.81	-0.46	excluded	absent	136.48	0.35	excluded
1552283_s_a	included	present	133.72	-0.92	included	present	715.62	-0.01	included	present	203.44	-0.81	included	present	445.93	-0.44	included	present	154.49	-0.89	included	absent	225.05	-0.78	included
1552286_at	included	absent	119.64	-0.35	included	absent	182.12	-0.26	included	absent	164.46	-0.29	included	absent	48.86	-0.46	included	present	265.42	-0.14	included	present	222.61	-0.2	included
1552287_s_a	included	present	356.4	0.42	included	present	566.85	2.35	included	present	182.64	-1.18	included	present	431.25	1.1	included	present	547.83	2.17	included	present	299.14	-0.11	included
1552288_at	excluded	absent	63.4	0.46	excluded	absent	110.36	1.54	excluded	absent	69.26	0.6	excluded	absent	29.16	-0.33	excluded	absent	7.63	-0.82	included	present	327.87	6.55	excluded
1552289_a_a	excluded	absent	2.22	-0.73	excluded	absent	85.38	1.49	excluded	absent	64.75	0.94	excluded	absent	11.55	-0.48	excluded	absent	4.69	-0.66	included	present	1026.99	26.59	excluded
1552291_at	included	present	323.28	-0.94	included	present	463.1	-0.14	included	present	682.24	1.1	included	present	560.55	0.41	included	present	322.63	-0.94	included	present	367.64	-0.69	included
1552293_at	included	present	245.36	1.46	included	present	221.93	1.21	included	present	222.8	1.22	excluded	absent	20.48	-0.96	included	present	507.29	4.29	excluded	absent	50.84	-0.63	included
1552295_a_a	excluded	absent	109.59	-0.07	excluded	absent	5.45	-1.56	excluded	absent	101.25	-0.19	excluded	absent	55.12	-0.85	excluded	absent	163.12	0.7	included	present	219.99	1.52	included
1552296_at	excluded	absent	5.1	-0.91	included	present	643.86	3.56	excluded	absent	36.01	-0.69	included	present	127.03	-0.05	excluded	present	19.21	-0.81	included	absent	3.72	-0.91	excluded
1552299_at	included	present	653.77	-1.14	included	present	3777.75	3.72	included	present	839.65	-0.85	included	present	1598.81	0.33	included	present	1257.73	-0.2	included	present	278	-1.72	included
1552301_a_a	excluded	absent	86.76	-0.32	excluded	absent	97.22	-0.3	excluded	absent	28.99	-0.43	excluded	absent	1.97	-0.48	included	present	320.63	0.12	included	present	491.76	0.45	included
1552302_at	included	present	308.53	0.34	included	present	288.94	0.26	included	present	311.84	0.35	included	absent	32.02	-0.82	included	present	219.53	-0.04	included	present	446.63	0.92	included
1552303_a_a	excluded	absent	126.62	0.65	excluded	absent	69.03	-0.09	excluded	absent	45.66	-0.39	excluded	absent	10.77	-0.84	excluded	absent	81.16	0.07	excluded	absent	116.67	0.52	excluded
1552304_at	included	present	176.05	1.59	excluded	absent	51.09	-0.77	included	present	229.13	2.6	included	present	196.71	1.98	excluded	absent	146.61	1.04	included	present	129.59	0.72	included
1552306_at	included	present	276.9	0.47	included	present	204.82	-0.47	included	present	462.02	2.88	included	present	754.54	6.69	included	present	220.91	-0.26	included	present	192.83	-0.63	included
1552307_a_a	included	present	586.17	-0.21	included	present	631.43	-0.16	included	present	1198.28	0.38	included	present	1003.11	0.19	included	present	1400.98	0.58	included	present	288.68	-0.49	included
1552309_a_a	included	present	412.07	-0.42	included	present	174.16	-0.5	included	present	258.69	-0.47	included	present	210.82	-0.49	included	present	197.49	-0.5	included	present	3101.24	0.48	included
1552310_at	included	present	180.58	-0.49	included	present	254.08	0.56	included	present	185.76	-0.42	included	present	153.1	-0.89	included	present	234.02	0.27	included	present	208.39	-0.1	included
1552311_a_a	excluded	absent	25.5	-0.57	excluded	absent	117.88	1.33	excluded	absent	98.66	0.93	excluded	absent	29.22	-0.49	excluded	absent	83.68	0.63	excluded	absent	16.53	-0.75	excluded
1552312_a_a	included	present	137.44	-0.44	included	present	200.74	0.37	included	present	643.66	6.05	included	present	492.99	4.12	included	present	456.78	3.65	included	present	397.11	2.89	included
1552314_a_a	included	present	158.58	-1.66	included	present	254.2	-0.41	included	absent	202.46	-1.09	included	present	143.93	-1.85	included	present	409.65	1.61	included	present	393.02	1.39	included
1552315_at	excluded	absent	4.1	-0.87	excluded	absent	44.01	0.11	excluded	absent	41.7	0.05	excluded	absent	3.38	-0.89	excluded	absent	18.88	-0.51	excluded	absent	87.19	1.17	excluded
1552316_a_a	included	present	1251.86	0.23	included	present	274.24	-0.8	included	present	270.82	-0.8	included	absent	78.67	-1.01	included	present	577.35	-0.48	included	present	1915.57	0.94	included
1552318_at	included	present	346.76	0.02	included	present	75.89	-0.67	included	absent	124.94	-0.54	included	absent	2.16	-0.85	included	present	241.7	-0.24	included	present	701.58	0.93	included
1552319_a_a	excluded	absent	68.59	0.53	excluded	absent	6.29	-0.84	excluded	absent	46.86	0.05	excluded	absent	4.13	-0.89	excluded	absent	51.94	0.17	excluded	absent	6.28	-0.84	excluded
1552320_a_a	included	absent	87.9	-0.36	included	present	214.07	-0.22	included	absent	75.31	-0.38	included	absent	72.68	-0.38	included	present	69.06	-0.39	included	absent	52.2	-0.4	included
1552321_a_a	excluded	absent	154.83	-0.28	excluded	absent	121.19	-0.33	included	present	151.08	-0.29	excluded	absent	133.96	-0.32	excluded	absent	224.03	-0.18	excluded	absent	131.81	-0.32	excluded
1552322_at	included	present	117.41	0.51	excluded	absent	79.63	-0.15	excluded	absent	35.56	-0.92	included	present	99.58	0.2	excluded	absent	83.22	-0.09	included	present	116.53	0.5	excluded
1552323_s_a	excluded	absent	93.56	-0.59	excluded	absent	11.28	-1.4	excluded	absent	56.72	-0.95	included	present	137.55	-0.15	excluded	absent	3.65	-1.47	excluded	absent	97.69	-0.55	excluded
1552325_at	included	present	142.2	-0.31	included	absent	51.23	-0.34	included	absent	22.42	-0.34	included	present	133.87	-0.31	included	absent	57.75	-0.33	included	absent	43.65	-0.34	included
1552326_a_a	included	absent	90.89	-0.28	included	present	205.03	-0.25	included	absent	38.41	-0.3	included	present	110.33	-0.28	included	absent	4.23	-0.31	included	absent	7.47	-0.31	included
1552327_at	included	present	177.99	0.16	included	present	143.53	-0.26	included	present	194.6	0.37	included	present	122.32	-0.52	included	present	18.51	-1.8	included	present	147.83	-0.21	included
1552329_at	included	present	251.88	-1.44	included	present	411.1	-0.65	included	present	329.33	-1.05	included	present	502.01	-0.2	included	present	368.9	-0.86	included	present	487.26	-0.28	included
1552330_at	included	present	316.34	4.61	included	present	1244.65	26.08	included	present	127.6	0.25	included	present	421.1	7.04	included	present	368.61	5.82	included	present	143.67	0.62	included

1552760_at	included	present	345.84	0.12	included	present	1395.54	4.68	included	present	4004.47	16.04	included	present	348.12	0.13	included	present	1157.66	3.65	included	present	198.03	-0.53	included	present	179.95	-0.61	included	present	343.92	0.11	included	present	239.37	-0.35	included	present	821.96	2.19	included	present	592.05	1.19	included	present	252.75	-0.29	included	present	336.86	0.08	included	present	1010.24	3.01	included	present	278.9	-0.17
1552761_at	excluded	absent	111.08	1.04	excluded	absent	22.84	-0.64	excluded	absent	48.66	-0.15	excluded	absent	95.94	0.76	excluded	absent	17.59	-0.74	excluded	absent	101.47	0.86	excluded	absent	33.31	-0.44	excluded	absent	87.34	0.59	excluded	absent	88.49	0.61	excluded	absent	5.55	-0.97	excluded	absent	36.49	-0.38	excluded	absent	21.03	-0.67	excluded	absent	20.6	-0.68	excluded	absent	9.41	-0.89	excluded	absent	31.27	-0.48
1552763_at	excluded	absent	115.71	0.76	excluded	absent	54.75	-0.38	excluded	absent	37.89	-0.7	included	present	101.97	0.5	excluded	absent	96.37	0.4	excluded	absent	66.42	-0.16	excluded	absent	7.01	-1.27	excluded	absent	26.17	-0.92	included	present	102.94	0.52	excluded	absent	40.98	-0.64	excluded	absent	21.4	-1	excluded	absent	48.14	-0.5	included	present	96.05	0.39	excluded	absent	26.61	-0.91	excluded	absent	1.63	-1.37
1552765_x_a	excluded	absent	70.14	-0.43	included	present	113.01	0.39	included	present	112.83	0.38	excluded	absent	87.48	-0.1	excluded	absent	95.54	0.05	excluded	absent	56.12	-0.7	included	present	102.1	0.18	excluded	absent	74.47	-0.35	included	present	137.08	0.85	included	present	53.59	-0.75	included	present	83.98	-0.17	excluded	absent	2.98	-1.72	included	present	124.3	0.6	included	present	68.04	-0.47	excluded	absent	13.07	-1.53
1552766_at	included	present	136.92	0.08	included	present	249.19	0.82	included	present	213.81	0.59	excluded	absent	4.7	-0.79	included	present	917.09	5.22	excluded	absent	90.53	-0.22	included	present	800.74	4.45	included	present	503.18	2.49	included	present	58.25	-0.44	included	present	241.58	0.77	included	present	806.65	4.49	excluded	absent	62.43	-0.41	included	present	410.45	1.88	included	present	496.14	2.45	included	present	217.68	0.61
1552767_a_a	included	present	372.67	-0.39	included	present	1724.22	1.33	included	present	715.07	0.05	included	absent	42.18	-0.8	included	present	3736.75	3.88	included	present	305.59	-0.47	included	present	3386.76	3.43	included	present	1353.57	0.86	included	present	223.44	-0.58	included	present	1179.03	0.64	included	present	1867.72	1.51	included	present	538.54	-0.18	included	present	4273.01	4.55	included	present	5780.64	6.46	included	present	688.61	0.01
1552768_at	excluded	absent	17.95	-0.31	excluded	absent	17.54	-0.32	excluded	absent	5.55	-0.71	excluded	absent	42.25	0.48	excluded	absent	42.64	0.5	excluded	absent	11.85	-0.51	excluded	absent	28	0.02	excluded	absent	11.61	-0.51	excluded	absent	8.65	-0.61	excluded	absent	6.49	-0.68	excluded	absent	16.14	-0.37	excluded	absent	5	-0.73	excluded	absent	5.39	-0.72	excluded	absent	3.81	-0.77	excluded	absent	15.66	-0.38
1552769_at	included	present	214.6	2.02	excluded	absent	74.97	-0.29	included	present	405.09	5.17	included	present	573.29	7.96	excluded	absent	179.78	1.44	included	present	201.72	1.81	included	present	168.8	1.26	included	present	378.57	4.73	included	present	181.51	1.47	included	present	328.91	3.91	included	present	314.97	3.68	excluded	absent	58.06	-0.57	included	present	126.28	0.56	included	present	148.71	0.93	included	present	294.68	3.35
1552770_s_a	excluded	absent	10.88	-1.31	excluded	absent	122.86	1.54	excluded	absent	59.38	-0.08	excluded	absent	34.71	-0.7	excluded	absent	18.57	-1.11	included	present	55.29	-0.18	excluded	absent	56.27	-0.16	excluded	absent	22.56	-1.01	excluded	absent	63.6	0.03	excluded	absent	10.85	-1.31	excluded	absent	6.98	-1.41	excluded	absent	3.65	-1.49	excluded	absent	20.56	-1.06	excluded	absent	4.91	-1.46	excluded	absent	18.99	-1.1
1552771_a_a	excluded	absent	44.94	-0.09	excluded	absent	79.18	0.52	excluded	absent	25.5	-0.44	excluded	absent	41.76	-0.15	excluded	absent	50.53	0.01	excluded	absent	60.76	0.19	excluded	absent	64.8	0.26	excluded	absent	7.37	-0.77	excluded	absent	49.91	-0.01	excluded	absent	67.55	0.31	excluded	absent	56.76	0.12	excluded	absent	72.08	0.39	excluded	absent	32.01	-0.33	excluded	absent	28.54	-0.39	excluded	absent	25.21	-0.45
1552772_at	excluded	absent	3.57	-0.79	excluded	absent	2.5	-0.83	excluded	absent	5.13	-0.74	excluded	absent	2.99	-0.81	excluded	absent	85.53	2.17	excluded	absent	4.87	-0.75	excluded	absent	1.64	-0.86	excluded	absent	20.78	-0.17	excluded	absent	56.96	1.13	excluded	absent	33.51	0.29	excluded	absent	51.73	0.95	included	present	175.24	5.4	excluded	absent	21.36	-0.15	excluded	absent	3.61	-0.79	excluded	absent	36.82	0.41
1552773_at	excluded	absent	1.3	-1.08	excluded	absent	83.74	0.96	excluded	absent	4.52	-1	excluded	absent	34.88	-0.25	included	present	160.62	2.86	excluded	absent	62.09	0.42	excluded	absent	36.11	-0.22	excluded	absent	70.68	0.64	excluded	absent	59.48	0.36	excluded	absent	46.13	0.03	included	present	50.46	0.14	included	present	93.24	1.19	excluded	absent	41.32	-0.09	excluded	absent	41.3	-0.09	excluded	absent	16.51	-0.7
1552774_a_a	included	present	294.16	-1.06	included	present	163.11	-1.32	included	present	278.82	-1.09	included	present	102.42	-1.44	included	present	578.52	-0.49	included	present	248.76	-1.15	included	present	600.27	-0.44	included	present	467.25	-0.71	included	present	266	-1.11	included	present	262.55	-1.12	included	present	348.63	-0.95	included	present	544.25	-0.56	included	present	865.52	0.09	included	present	520.69	-0.6	included	present	144.94	-1.35
1552775_at	excluded	absent	47.16	0.26	excluded	absent	3.39	-0.8	excluded	absent	79.87	1.05	excluded	absent	11.36	-0.61	excluded	absent	91.45	1.33	excluded	absent	41.25	0.11	excluded	absent	3.75	-0.79	excluded	absent	23.03	-0.33	excluded	absent	14.56	-0.53	excluded	absent	9.84	-0.64	excluded	absent	38.78	0.05	excluded	absent	8.14	-0.69	excluded	absent	87.38	1.23	excluded	absent	11.51	-0.6	excluded	absent	0.88	-0.86
1552777_a_a	excluded	absent	2.07	-0.8	excluded	absent	96.81	0.57	excluded	absent	2.2	-0.79	excluded	absent	4.76	-0.76	excluded	absent	6.73	-0.73	excluded	absent	89.09	0.46	excluded	absent	3.12	-0.78	excluded	absent	12.24	-0.65	excluded	absent	25.42	-0.46	excluded	absent	70.24	0.19	excluded	absent	4.19	-0.77	excluded	absent	2.67	-0.79	excluded	absent	36.1	-0.31	excluded	absent	28.86	-0.41	excluded	absent	0.99	-0.81
1552779_a_a	excluded	absent	9.17	-0.8	included	present	219.47	1.88	excluded	absent	52.42	-0.25	included	present	182.18	1.41	included	present	561.26	6.24	excluded	absent	129.25	0.73	included	present	932.15	10.97	included	present	186.08	1.46	excluded	absent	4.69	-0.85	included	present	143.08	0.91	excluded	absent	30.37	-0.53	excluded	absent	61.79	-0.13	included	present	257.73	2.37	excluded	absent	25.59	-0.59	included	present	128.66	0.73
1552782_at	included	present	212.73	0.01	included	present	1923.4	7.84	included	present	378.13	0.77	included	present	573.56	1.66	included	present	1851.84	7.52	included	present	177.58	-0.15	included	present	2410.98	10.08	included	present	160.82	-0.23	included	present	118.94	-0.42	included	present	606.82	1.81	included	present	106.28	-0.48	included	present	205.58	-0.02	included	present	473.12	1.2	excluded	absent	99.33	-0.51	included	present	236.23	0.12
1552783_at	included	present	140.77	0.23	excluded	absent	50.99	-1.11	included	present	144.67	0.29	included	present	291.29	2.48	excluded	absent	137.08	0.18	excluded	absent	98.29	-0.4	excluded	absent	48.2	-1.15	included	present	95.95	-0.44	included	present	67.89	-0.85	included	present	61.46	-0.95	included	present	51.99	-1.09	included	present	146.29	0.32	excluded	absent	57.48	-1.01	excluded	absent	24.8	-1.5	included	present	49.58	-1.13
1552785_at	included	present	278.57	0.15	included	present	546.58	2.02	included	present	565.41	2.15	included	present	367.51	0.77	included	present	286.34	0.2	included	present	184.76	-0.5	included	present	432.25	1.22	included	present	501.73	1.7	included	present	535.29	1.94	included	present	472.69	1.5	included	present	608.52	2.45	included	present	518.38	1.82	included	present	588.86	2.31	included	present	683.7	2.97	included	present	406.92	1.04
1552787_at	included	present	301.81	0.67	included	present	97.71	-0.35	included	present	361.26	0.97	included	present	263.73	0.48	included	absent	95.69	-0.36	included	present	139.73	-0.14	included	absent	39.19	-0.65	included	present	222.28	0.27	included	present	257.65	0.45	included	present	228.91	0.3	included	present	158.91	-0.05	included	present	113.66	-0.27	included	present	293.49	0.63	included	present	327.73	0.8	included	absent	36.65	-0.66
1552788_a_a	included	present	251.98	-0.12	excluded	absent	317.72	0.12	included	present	410.62	0.45	included	present	390.32	0.38	excluded	absent	234.55	-0.18	excluded	absent	280.09	-0.02	excluded	absent	159.09	-0.45	excluded	absent	303.57	0.07	included	present	453.15	0.6	included	present	323.94	0.14	included	present	313.97	0.11	excluded	absent	195.36	-0.32	included	present	315.72	0.11	included	present	320.35	0.13	included	present	236.46	-0.17
1552789_at	included	present	691.51	-0.29	included	present	705.21	-0.26	included	present	381.98	-0.9	included	present	500.25	-0.66	included	present	147.79	-1.36	included	present	297.79	-1.07	included	present	358.98	-0.95	included	present	453.64	-0.76	included	present	427.8	-0.81	included	present	144.41	-1.37	included	present	237.2	-1.19	included	present	212.5	-1.24	included	present	608.72	-0.45	included	present	1024.9	0.38	included	present	451.84	-0.76
1552790_a_a	included	present	635.52	0.17	included	present	568.04	-0.1	included	present	416.58	-0.71	included	present	396.6	-0.79	included	present	223.13	-1.49	included	present	355.72	-0.95	included	present	423.35	-0.68	included	present	477.8	-0.46	included	present	517.19	-0.3	included	present	451.55	-0.57	included	present	290.93	-1.21	included	present	374.45	-0.88	included	present	800.07	0.84	included	present	1151.94	2.26	included	present	538	-0.22
1552791_a_a	excluded	absent	40.74	-0.14	excluded	absent	8.66	-0.17	excluded	absent	2.71	-0.18	excluded	absent	40.7	-0.14	excluded	absent	39.4	-0.15	included	present	768.7	0.53	excluded	absent	1.76	-0.18	included	present	62.12	-0.12	excluded	absent	1.93	-0.18	included	present	764.91	0.53	excluded	absent	2.55	-0.18	excluded	absent	4.59	-0.18	excluded	absent	3.33	-0.18	excluded	absent	0.59	-0.18	excluded	absent	3.63	-0.18
1552792_at	included	present	996.13	2.15	included	present	714.26	0.66	included	present	1597.51	5.32	included	present	1433.3	4.46	included	present	1339.41	3.96	included	present	537.99	-0.27	included	present	769.49	0.95	included	present	935.75	1.83	included	present	816.31	1.2	included	present	625.44	0.19	included	present	1052.11	2.44	included	present	525.99	-0.33	included	present	421.4	-0.89	included	present	547.18	-0.22	included	present	1002.87	2.18
1552793_at	included	present	87.37	1.11	excluded	absent	5.61	-0.72	excluded	absent	41.49	0.08	excluded	absent	62.61	0.55	excluded	absent	2.78	-0.79	excluded	absent	131.86	2.11	excluded	absent	46.91	0.2	excluded	absent	39.4	0.03	excluded	absent	34.92	-0.07	excluded	absent	3.86	-0.76	excluded	absent	53.97	0.36	excluded	absent	15.27	-0.51	excluded	absent	57.06	0.43	excluded	absent	1.61	-0.81	included	present	64.3	0.59
1552794_a_a	excluded	absent	100.87	0.4	included	present	72.96	-0.17	excluded	absent	73.32	-0.16	included	present	208.38	2.57	excluded	absent	153.99	1.47	excluded	absent	51.05	-0.61	included	present	85.44	0.09	included	present	168.32	1.76	excluded	absent	40.03	-0.83	included	present	85.78	0.09	included	present	93.02	0.24	included	present	194.98	2.3	included	present	98.82	0.36	included	present	200.14	2.41	included	present	143.55	1.26
1552796_a_a	excluded	absent	4.28	-0.44	excluded	absent	7.17	-0.32	excluded	absent	6.96	-0.33	included	present	335.38	12.95	excluded	absent	9.41	-0.23	excluded	absent	5.48	-0.39	excluded	absent	14.19	-0.04	excluded	absent	2.93	-0.49	excluded	absent	3.91	-0.45	excluded	absent	5.95	-0.37	excluded	absent	15.63	0.02	excluded	absent	15.57	0.02	excluded	absent	9.67	-0.22	excluded	absent	7.75	-0.3	excluded	absent	4.31	-0.44
1552797_s_a	included	present	517.58	-0.39	included	present	288.36	-0.48	included	present	497.35	-0.4	included	present	348.58	-0.46	included	present	238.18	-0.5	included	present	260.47	-0.49	included	present	232.5	-0.5	included	absent	42.38	-0.58	included	present	311.24	-0.47	included	present	258.75	-0.49	included	present	125.17	-0.54	included	present	608.15	-0.35	included	present	840.96	-0.26	included	present	548.93	-0.38	included	present	110.92	-0.55
1552798_a_a	included	absent	194.06	-0.42	included	present	116.11	-0.95	included	absent	85.52	-1.15	included	present	129.95	-0.85	included	present	317.78	0.4	included	present	548.27	1.94	included	absent	82.98	-1.17	included	absent	81.07	-1.18	included	present	489.13	1.55	included	present	203.72	-0.36	included	absent	141.88	-0.77	excluded	absent	280.33	0.15	included	absent	55.56	-1.35	included	absent	64.35	-1.29	included	present	207.24	-0.34
1552799_at	excluded	absent	4.06	-0.8	excluded	absent	6.93	-0.72	excluded	absent	6.7	-0.73	excluded	absent	6.91	-0.72	excluded	absent	7.69	-0.7	excluded	absent	7.63	-0.7	excluded	absent	10.06	-0.64	excluded	absent	29.47	-0.13	excluded	absent	7.12	-0.72	excluded	absent	6.77	-0.72	excluded	absent	117.56	2.18	excluded	absent	8.53	-0.68	excluded	absent	17	-0.46	included	present	65.42	0.81	excluded	absent	4.59	-0.78
1552800_at	excluded	absent	11.62	-0.58	excluded	absent	43.41	0.31	excluded	absent	7.21	-0.7	excluded	absent	40.3	0.22	excluded	absent	82.02	1.39	excluded	absent	93.49	1.71	excluded	absent	8.26	-0.67	excluded	absent	5.04	-0.76	excluded	absent	34.43	0.06	excluded	absent	55.57	0.65	excluded	absent	79.12	1.31	excluded	absent	2.75	-0.82	excluded	absent	18.72	-0.38	excluded	absent	4.15	-0.78	excluded	absent	43.1	0.3
1552801_at	excluded	absent	5.48	-0.25	excluded	absent	3.4	-0.35	excluded	absent	1.87	-0.42	excluded	absent	2.27	-0.4	excluded	absent	5.56	-0.25	excluded	absent	1.51	-0.43	excluded	absent	4.4	-0.3	excluded	absent	1.46	-0.43	excluded	absent	3.36	-0.35	excluded	absent	4.2	-0.31	excluded	absent	1.98	-0.41	excluded	absent	1.62	-0.43	excluded	absent	7.7	-0.15	excluded	absent	2.4	-0.39	excluded	absent	1.51	-0.43
1552803_a_a	included	present	105.27	0.16	included	present	164.58	0.68	excluded	absent	4.46	-0.73	included	present	65.9	-0.19	included	present	281.43	1.7	excluded	absent	45.1	-0.37	included	present	431.3	3.01	excluded	absent	1.56	-0.75	included	present	164.61	0.68	included	present	209.46	1.07	included	present	64.96	-0.2	excluded	absent	83.6	-0.03	included	present	104.67	0.15	included	present	126.1	0.34	included	present	152.88	0.57
[illegible]																																																													

1553257_at	excluded	absent	4.96	-0.75	excluded	absent	64.96	1.5	excluded	absent	42.78	[illegible]
1553258_at	excluded	absent	36.43	-0.09	excluded	absent	34.08	-0.15	excluded	absent	29.75	[illegible]
1553260_s_a	excluded	absent	125.29	-0.21	included	present	237.46	-0.1	excluded	absent	6.36	[illegible]
1553261_x_a	excluded	absent	76.39	-0.18	included	present	206.56	0.17	excluded	absent	16.62	[illegible]
1553262_a_a	excluded	absent	240.18	1.04	excluded	absent	43.31	-1.12	excluded	absent	141.5	[illegible]
1553263_at	excluded	absent	65.22	0.81	excluded	absent	19.56	-0.48	excluded	absent	1.86	[illegible]
1553264_a_a	included	present	296.3	0.92	included	present	187.36	0.18	excluded	absent	114.64	[illegible]
1553265_at	excluded	absent	59.49	-0.27	excluded	absent	2.5	-0.86	included	present	104.27	[illegible]
1553266_at	included	present	162.9	-0.44	included	absent	111.41	-0.66	included	present	144.72	[illegible]
1553267_a_a	included	present	963.85	-0.25	included	present	299.19	-1.47	included	present	1380.24	[illegible]
1553268_at	excluded	absent	13.74	-1.13	excluded	absent	70.15	0.13	excluded	absent	68.23	[illegible]
1553269_at	included	present	612.86	2.87	included	present	201.74	-0.3	included	present	625.88	[illegible]
1553271_at	excluded	absent	114.84	1.51	excluded	absent	77.85	0.47	excluded	absent	2.09	[illegible]
1553272_at	included	present	272.92	1	included	present	125.02	-0.23	excluded	absent	17.57	[illegible]
1553274_a_a	included	present	801.19	-0.62	included	present	739.13	-0.75	included	present	1083.5	[illegible]
1553275_s_a	excluded	absent	94.12	-0.42	excluded	absent	92.79	-0.43	excluded	absent	2.35	[illegible]
1553276_at	excluded	absent	1.65	-0.43	excluded	absent	40.32	1.45	excluded	absent	1.85	[illegible]
1553277_at	excluded	absent	71.88	-0.75	excluded	absent	46.7	-1.09	included	present	140.42	[illegible]
1553279_at	excluded	absent	43.08	-0.49	excluded	absent	29.02	-0.63	excluded	absent	7.68	[illegible]
1553280_at	included	present	257.62	-0.44	included	present	296.85	-0.16	included	present	274.98	[illegible]
1553281_at	excluded	absent	3.37	-0.66	excluded	absent	36.8	-0.22	excluded	absent	5.27	[illegible]
1553282_at	excluded	absent	11.07	-0.42	excluded	absent	46.38	0.73	excluded	absent	9.92	[illegible]
1553284_s_a	included	present	183.09	-0.09	excluded	absent	67.07	-0.85	excluded	absent	53.21	[illegible]
1553285_s_a	excluded	absent	18.18	-0.44	excluded	absent	72.69	-0.12	excluded	absent	5.16	[illegible]
1553286_at	included	present	189.63	0.31	included	present	233.12	0.89	included	present	218.71	[illegible]
1553288_a_a	included	present	129.26	-0.6	included	present	651.34	0.7	included	present	113.06	[illegible]
1553292_s_a	included	present	263.87	-0.69	included	present	186.12	-0.87	included	present	185.11	[illegible]
1553293_at	excluded	absent	38.59	0.64	excluded	absent	21.46	-0.04	excluded	absent	3.72	[illegible]
1553295_at	included	present	114.12	2.87	excluded	absent	65.22	1.3	excluded	absent	49.19	[illegible]
1553296_at	excluded	absent	2.14	-0.3	excluded	absent	19.25	-0.26	excluded	absent	2.64	[illegible]
1553297_a_a	excluded	absent	79.37	-0.07	excluded	absent	12.46	-0.74	excluded	absent	62.8	[illegible]
1553298_at	excluded	absent	73.72	-0.11	excluded	absent	25.73	-1.12	excluded	absent	11.76	[illegible]
1553299_at	excluded	absent	76.32	1.13	included	present	127.31	2.62	excluded	absent	54.19	[illegible]
1553300_a_a	included	present	299.12	-0.26	included	present	167.25	-0.59	included	present	222.92	[illegible]

[illegible]

1553728_at	excluded	absent	5.67	-0.62	excluded	absent	47.83	0.61	excluded	absent	10.9	-0.47	excluded	absent	4.46	-0.66	excluded	absent	52.93	0.76	excluded	absent	88.73	1.81	excluded	absent	69.61	1.25	excluded	absent	22.95	-0.11	excluded	absent	13.98	-0.38	excluded	absent	10.73	-0.47	excluded	absent	55.84	0.85	excluded	absent	6.41	-0.6	excluded	absent	5.32	-0.63	excluded	absent	6.8	-0.59	excluded	absent	48.18	0.62
1553729_s_a	excluded	absent	49.62	0	excluded	absent	74.01	0.4	excluded	absent	28.13	-0.35	excluded	absent	1.53	-0.79	excluded	absent	128.81	1.3	excluded	absent	8.1	-0.68	excluded	absent	10.07	-0.65	excluded	absent	33.81	-0.26	excluded	absent	68.15	0.31	excluded	absent	68.57	0.31	excluded	absent	93.18	0.72	excluded	absent	37.77	-0.19	excluded	absent	95.6	0.76	included	present	68.38	0.31	included	present	90.07	0.67
1553730_x_a	excluded	absent	66.92	0.79	excluded	absent	30.79	-0.08	excluded	absent	39.92	0.14	excluded	absent	27.53	-0.16	excluded	absent	99.56	1.57	excluded	absent	5.03	-0.7	excluded	absent	49.22	0.36	excluded	absent	22.27	-0.29	excluded	absent	38.89	0.11	excluded	absent	3.23	-0.74	excluded	absent	89.94	1.34	excluded	absent	39.21	0.12	excluded	absent	8.71	-0.61	excluded	absent	48.12	0.33	excluded	absent	25.33	-0.21
1553732_s_a	excluded	absent	83.18	0.08	included	present	218.75	2.33	excluded	absent	4.69	-1.22	excluded	absent	108.51	0.5	excluded	absent	175.01	1.61	excluded	absent	211.78	2.22	excluded	absent	130.8	0.87	excluded	absent	66.74	-0.19	excluded	absent	81.47	0.05	excluded	absent	66.84	-0.19	excluded	absent	154.88	1.27	excluded	absent	69.41	-0.15	excluded	absent	99.59	0.35	excluded	absent	56.85	-0.36	excluded	absent	87.71	0.16
1553734_at	included	present	155.46	0.09	excluded	absent	77.85	-0.43	excluded	absent	37.62	-0.69	included	present	282.52	0.93	excluded	absent	97.02	-0.3	excluded	absent	14.44	-0.85	excluded	absent	14.83	-0.84	included	present	168.2	0.17	included	present	62.2	-0.53	included	present	71.47	-0.47	included	present	211.76	0.46	excluded	absent	73.32	-0.46	excluded	absent	73.04	-0.46	included	present	129.6	-0.09	excluded	absent	36.07	-0.7
1553736_at	included	present	316.74	-0.08	included	present	279.83	-0.38	included	present	362.83	0.3	included	present	394.63	0.56	included	present	336.65	0.08	included	present	279.56	-0.38	included	absent	81.69	-2	included	present	123.66	-1.65	included	present	415.2	0.72	included	absent	68.78	-2.1	included	present	365.26	0.32	included	present	264.77	-0.5	included	present	347.81	0.17	included	present	393.53	0.55	included	absent	61.41	-2.16
1553739_at	excluded	absent	2.34	-0.9	excluded	absent	5.53	-0.77	excluded	absent	7.59	-0.69	excluded	absent	10.12	-0.59	excluded	absent	12.1	-0.5	excluded	absent	31.12	0.27	excluded	absent	36.1	0.48	excluded	absent	8.36	-0.66	excluded	absent	22.91	-0.06	excluded	absent	3.77	-0.84	excluded	absent	3.3	-0.86	excluded	absent	3.26	-0.87	excluded	absent	30.42	0.24	excluded	absent	2.7	-0.89	excluded	absent	4.1	-0.83
1553740_a_a	excluded	absent	1.03	-1.09	excluded	absent	17.82	-0.71	excluded	absent	60.59	0.26	excluded	absent	6.66	-0.96	excluded	absent	58.45	0.21	excluded	absent	4.57	-1.01	excluded	absent	6.75	-0.96	excluded	absent	64.51	0.35	excluded	absent	33.78	-0.35	excluded	absent	56.2	0.16	excluded	absent	9.83	-0.89	excluded	absent	9.94	-0.89	excluded	absent	52.22	0.07	excluded	absent	3.58	-1.03	excluded	absent	9.12	-0.91
1553741_at	excluded	absent	43.23	-0.3	included	present	153.96	2.89	excluded	absent	2.66	-1.47	excluded	absent	38.69	-0.43	excluded	absent	73.96	0.58	excluded	absent	56.59	0.08	excluded	absent	58.59	0.14	excluded	absent	50.17	-0.1	excluded	absent	72.66	0.55	excluded	absent	43.63	-0.29	excluded	absent	4.44	-1.42	excluded	absent	35.03	-0.54	excluded	absent	61.49	0.22	excluded	absent	13.44	-1.16	included	present	64.79	0.32
1553743_at	included	present	226.85	-0.77	included	present	223.25	-0.81	included	present	194.35	-1.13	included	present	441.21	1.63	included	present	259.49	-0.4	included	present	291.74	-0.04	included	present	261.56	-0.38	included	present	200.54	-1.06	included	present	329.3	0.38	included	present	235.81	-0.67	included	present	327.76	0.36	included	present	225.96	-0.78	included	present	200.79	-1.06	included	present	218.43	-0.86	included	present	220.92	-0.84
1553746_a_a	excluded	absent	49.6	-0.27	excluded	absent	7.37	-0.71	excluded	absent	4.69	-0.73	excluded	absent	5.86	-0.72	excluded	absent	110.64	0.37	excluded	absent	77.19	0.02	excluded	absent	4.32	-0.74	excluded	absent	18.7	-0.59	excluded	absent	2.33	-0.76	excluded	absent	37.9	-0.39	excluded	absent	60.9	-0.15	excluded	absent	70.2	-0.05	excluded	absent	85.13	0.1	included	present	80.38	0.05	excluded	absent	12.83	-0.65
1553747_at	excluded	absent	85.98	0.69	excluded	absent	56.39	0.01	excluded	absent	62.96	0.16	excluded	absent	47.94	-0.18	excluded	absent	5.27	-1.17	excluded	absent	42.72	-0.31	excluded	absent	58.77	0.07	excluded	absent	3.39	-1.21	excluded	absent	83.4	0.63	excluded	absent	38.7	-0.4	excluded	absent	72.31	0.38	excluded	absent	89.13	0.77	excluded	absent	76.68	0.48	excluded	absent	46.87	-0.21	excluded	absent	40.5	-0.36
1553749_at	included	present	3075.34	1.05	included	present	2820.39	0.61	included	present	5271.21	4.85	included	present	3638.65	2.02	included	present	2463.77	-0.01	included	present	2067.22	-0.69	included	present	3493.34	1.77	included	present	2824.77	0.62	included	present	4017.17	2.68	included	present	1520.79	-1.64	included	present	3440.61	1.68	included	present	1186.23	-2.21	included	present	1263.39	-2.08	included	present	1234.15	-2.13	included	present	1799.41	-1.15
1553750_a_a	included	present	360.55	-0.15	included	present	271.16	-0.41	included	present	487.3	0.23	included	present	221.46	-0.55	included	present	294.97	-0.34	included	present	260.31	-0.44	included	present	137.53	-0.8	included	present	314.93	-0.28	included	present	294.22	-0.34	included	present	122.39	-0.85	included	present	285.89	-0.36	included	present	179.97	-0.68	included	present	194.31	-0.63	included	present	182.78	-0.67	included	present	218.44	-0.56
1553752_at	excluded	absent	40.34	-1.03	excluded	absent	61.85	-0.61	excluded	absent	72.06	-0.41	excluded	absent	58.12	-0.68	excluded	absent	103.95	0.22	excluded	absent	82.99	-0.19	excluded	absent	77.37	-0.3	excluded	absent	56.18	-0.72	excluded	absent	90.95	-0.04	excluded	absent	130.62	0.74	excluded	absent	117	0.48	excluded	absent	147.73	1.08	excluded	absent	111.35	0.36	excluded	absent	43.75	-0.97	excluded	absent	62.41	-0.6
1553755_at	excluded	absent	46.91	-0.59	excluded	absent	108.36	0.59	excluded	absent	18.1	-1.15	excluded	absent	32.04	-0.88	excluded	absent	107.93	0.59	excluded	absent	54.5	-0.45	excluded	absent	3.66	-1.43	excluded	absent	71.44	-0.12	excluded	absent	16.58	-1.18	excluded	absent	71.53	-0.12	excluded	absent	87.16	0.18	excluded	absent	14.92	-1.21	excluded	absent	11.64	-1.28	excluded	absent	10.18	-1.3	excluded	absent	56.99	-0.4
1553756_at	excluded	absent	46.33	-0.12	excluded	absent	6.04	-1.05	excluded	absent	2.89	-1.12	excluded	absent	31.85	-0.45	excluded	absent	137.84	1.99	excluded	absent	129.11	1.79	excluded	absent	41.25	-0.23	excluded	absent	56.76	0.12	excluded	absent	44.3	-0.16	excluded	absent	24.5	-0.62	excluded	absent	4.15	-1.09	excluded	absent	56.94	0.13	excluded	absent	57.24	0.13	excluded	absent	3.33	-1.11	excluded	absent	10.48	-0.94
1553757_at	included	present	1613.09	-0.07	included	present	4521.38	3.45	included	present	1691.48	0.02	included	present	1033.29	-0.78	included	present	1927.13	0.31	included	present	482.48	-1.44	included	present	1044.45	-0.76	included	present	443.87	-1.49	included	present	1610.95	-0.07	included	present	1154.53	-0.63	included	present	1540.96	-0.16	included	present	248.28	-1.73	included	present	999.02	-0.82	included	present	545.43	-1.37	included	present	893.86	-0.94
1553759_at	excluded	absent	71	-0.23	excluded	absent	32.06	-1.01	included	present	103.86	0.43	included	present	150.35	1.36	excluded	absent	91.19	0.18	excluded	absent	2.2	-1.6	excluded	absent	94.66	0.25	included	present	68.66	-0.27	included	present	167.48	1.7	excluded	absent	82.42	0	included	present	76.07	-0.13	excluded	absent	31.47	-1.02	excluded	absent	60.16	-0.44	excluded	absent	46.9	-0.71	included	present	35.36	-0.94
1553761_at	excluded	absent	6.23	-0.58	excluded	absent	2.89	-0.7	excluded	absent	1.79	-0.73	excluded	absent	2.1	-0.72	excluded	absent	24.43	0.03	excluded	absent	47.71	0.81	excluded	absent	2.93	-0.69	excluded	absent	9.6	-0.47	excluded	absent	20.08	-0.12	excluded	absent	67.85	1.49	excluded	absent	15.9	-0.26	excluded	absent	35.23	0.39	excluded	absent	3.84	-0.66	excluded	absent	12.21	-0.38	excluded	absent	13.71	-0.33
1553764_a_a	included	present	342.64	0.03	excluded	absent	49.6	-0.56	included	present	183.85	-0.29	included	present	154.18	-0.35	included	present	176.51	-0.3	included	present	538.12	0.43	excluded	absent	15.8	-0.63	excluded	absent	61.48	-0.54	included	present	173.74	-0.31	included	present	57.5	-0.54	included	present	130.54	-0.4	included	present	131.53	-0.39	included	present	105.74	-0.45	included	present	121.52	-0.41	excluded	absent	25.16	-0.61
1553765_a_a	included	present	77.83	-0.55	excluded	absent	111.76	-0.49	excluded	absent	16.23	-0.65	included	present	129.58	-0.46	excluded	absent	7.74	-0.66	excluded	absent	2.58	-0.67	included	present	128.88	-0.46	included	present	138.05	-0.45	excluded	absent	71.87	-0.56	included	present	82.6	-0.54	included	present	64.39	-0.57	included	present	77.14	-0.55	included	present	218.02	-0.32	included	present	232.86	-0.3	included	present	68.83	-0.56
1553768_a_a	included	present	151.93	0.39	excluded	absent	93.03	-0.27	excluded	absent	48.79	-0.76	included	present	184.99	0.77	excluded	absent	109.79	-0.08	excluded	absent	67.62	-0.55	excluded	absent	7	-1.23	included	present	216.27	1.12	excluded	absent	87.69	-0.33	included	present	118.85	0.02	included	present	156.16	0.44	excluded	absent	54.38	-0.7	excluded	absent	9.57	-1.2	included	present	94.43	-0.25	excluded	absent	19.51	-1.09
1553769_at	excluded	absent	33.76	-0.02	excluded	absent	80.96	1.35	excluded	absent	20.77	-0.39	excluded	absent	2.69	-0.92	excluded	absent	34.06	-0.01	excluded	absent	14.59	-0.57	excluded	absent	1.63	-0.95	excluded	absent	13.12	-0.62	excluded	absent	1.86	-0.94	excluded	absent	6.25	-0.82	excluded	absent	3.59	-0.89	excluded	absent	45.91	0.34	excluded	absent	1.09	-0.96	excluded	absent	1.22	-0.96	excluded	absent	16.95	-0.5
1553770_a_a	excluded	absent	2.55	-1	excluded	absent	3.73	-0.96	excluded	absent	2.72	-0.99	excluded	absent	1.93	-1.02	excluded	absent	2.95	-0.99	excluded	absent	3.71	-0.96	excluded	absent	12.39	-0.67	excluded	absent	6.15	-0.88	excluded	absent	52.91	0.68	excluded	absent	17.24	-0.51	excluded	absent	20.11	-0.42	included	present	162.98	4.34	excluded	absent	15.97	-0.55	excluded	absent	19.12	-0.45	excluded	absent	17.91	-0.49
1553772_at	included	present	558.72	0.83	included	present	122.3	-1.55	included	present	595.61	1.04	included	present	254.9	-0.83	included	present	433.85	0.15	included	absent	223.31	-1	included	present	192.02	-1.17	included	present	207.43	-1.09	included	present	360.72	-0.25	included	present	110.74	-1.62	included	present	244.58	-0.89	included	present	144.41	-1.43	included	present	267.58	-0.76	included	present	185.34	-1.21	included	present	101.28	-1.67
1553775_at	excluded	absent	12.53	-0.59	excluded	absent	71.36	1.5	excluded	absent	2.05	-0.97	excluded	absent	50.31	0.75	excluded	absent	23.54	-0.2	excluded	absent	82.23	1.89	excluded	absent	3.46	-0.92	excluded	absent	38.5	0.33	excluded	absent	25.49	-0.13	excluded	absent	37.63	0.3	excluded	absent	52.98	0.85	excluded	absent	36.96	0.28	excluded	absent	7.23	-0.78	excluded	absent	10.23	-0.68	excluded	absent	23.49	-0.2
1553776_at	excluded	absent	5.31	-0.25	excluded	absent	1.55	-0.26	excluded	absent	18.37	-0.2	excluded	absent	22.66	-0.19	excluded	absent	78.45	0.01	excluded	absent	2.85	-0.26	excluded	absent	1.79	-0.26	excluded	absent	0.64	-0.27	excluded	absent	1.25	-0.26	excluded	absent	23.68	-0.18	excluded	absent	5.8	-0.25	excluded	absent	3.35	-0.26	excluded	absent	12.68	-0.22	excluded	absent	3.51	-0.26	excluded	absent	1.7	-0.26
1553778_at	excluded	absent	76.49	2.45	excluded	absent	7.11	-0.49	excluded	absent	68.53	2.11	included	present	153.63	5.71	excluded	absent	122.55	4.39	excluded	absent	12.55	-0.26	excluded	absent	5.06	-0.57	included	present	68.92	2.13	excluded	absent	10.47	-0.35	excluded	absent	12.04	-0.28	excluded	absent	106.6	3.72	excluded	absent	118.54	4.22	excluded	absent	45.04	1.12	excluded	absent	13.42	-0.22	excluded	absent	54.28	1.51
1553780_at	included	present	128.44	1.66	excluded	absent	80.16	0.6	excluded	absent	44.11	-0.2	included	present	101.86	1.08	excluded	absent	143.01	1.98	excluded	absent	6.63	-1.02	excluded	absent	8.53	-0.98	excluded	absent	6.19	-1.03	excluded	absent	9.41	-0.96	excluded	absent	98.61	1	excluded	absent	46.48	-0.14	excluded	absent	5.69	-1.04	excluded	absent	20.69	-0.71	excluded	absent	14.59	-0.85	excluded	absent	44.82	-0.18
1553781_at	included	present	801.06	3.2	included	present	652.33	2.35	included	present	1323.78	6.19	included	present	2522.42	13.05	included	present	621.06	2.17	included	present	658.18	2.39	included	present	380.8	0.8	included	present	1312.23	6.13	included	present	866.29	3.58	included	present	234.47	-0.04	included	present	851.18	3.49	included	present	261.49	0.12	included	present	138.76	-0.58	included	present	144.02	-0.55	included	present	309.16	0.39
1553785_at	included	present	118.98	-0.56	included	present	112.52	-0.59	included	absent	2.54	-1.16	included	present	167.27	-0.31	included	absent	76.78	-0.77	included	present	217.37	-0.05	included	present	280.53	0.28	included	present	121.9	-0.54	included	present	664.87	2.26	included	present	99.73	-0.65	included	present	192.13	-0.18	included	present	375.42	0.77	included	absent	84.29	-0.73	included	present	63.15	-0.84	included	present	182.21	-0.23
1553787_at	included	present	335.19	0.45	included	present	188.11	-0.14	included	absent	67.66	-0.62	included	absent	120.73	-0.41	included	absent	93.56	-0.52	included	present	121.2	-0.41	included	present	89.76	-0.53	included	absent	67.91	-0.62	included	present	90.68	-0.53	included	absent	50	-0.69	included	absent	49.42	-0.7	included	absent	14.8	-0.83	included	present	79.73	-0.57	included	present	176.48	-0.19	included	present	61.07	-0.65
1553789_a_a	excluded	absent	126.73	0.15	included	present	287.18	2.3	included	present	488.57	4.99	included	present	709.21	7.95	included	present	435.14	4.28	excluded	absent	72.75	-0.58	excluded	absent	155.71	0.54	excluded	absent	100.13	-0.21	included	present	163.05	0.63	excluded	absent	53.6	-0.83	excluded	absent	188.67	0.98	included	present	318.61	2.72	included	present	270.23	2.07	included	present	559.52	5.94	included	present	268.68	2.05
[illegible]																																																												

1554256_a_a	included	present	903.51	0.17	included	present	890.52	0.15	included	present	1083.22	0.45	included	present	341.14	-0.68	included	present	938.48	0.23	included	present	212.55	-0.88	included	present	594.43	-0.3	included	present	803.07	0.02	included	present	669.46	-0.18	included	present	2575.54	2.72	included	present	1179.62	0.59	included	present	672.53	-0.18	included	present	755.02	-0.05	included	present	412.97	-0.58	included	present	964.81	0.27
1554258_a_a	excluded	absent	38.78	-0.2	excluded	absent	50.78	-0.18	excluded	absent	4.56	-0.26	excluded	absent	72.53	-0.14	excluded	absent	144.19	0	excluded	absent	4.42	-0.26	excluded	absent	7.65	-0.26	excluded	absent	3.95	-0.26	excluded	absent	74.05	-0.13	excluded	absent	3.02	-0.26	excluded	absent	129.35	-0.03	excluded	absent	67.2	-0.15	excluded	absent	63.17	-0.15	excluded	absent	58.5	-0.16	included	present	244.48	0.19
1554259_at	excluded	absent	46.18	-0.13	excluded	absent	26.48	-0.44	excluded	absent	4.25	-0.8	excluded	absent	94.11	0.64	excluded	absent	94.88	0.66	excluded	absent	88.84	0.56	excluded	absent	96.37	0.68	excluded	absent	76.82	0.37	excluded	absent	75.56	0.35	excluded	absent	55.94	0.03	excluded	absent	56.13	0.03	excluded	absent	116.41	1	excluded	absent	9.93	-0.71	excluded	absent	10.08	-0.7	excluded	absent	62.35	0.13
1554260_a_a	included	present	934.17	-0.2	included	present	855.24	-0.31	included	present	4221.68	4.34	included	present	1689.54	0.84	included	present	1600.88	0.72	included	present	977.23	-0.15	included	present	500.56	-0.8	included	present	1404.82	0.45	included	present	982.11	-0.14	included	present	1047.77	-0.05	included	present	1228.11	0.2	included	present	686.17	-0.55	included	present	869.78	-0.29	included	present	962.58	-0.17	included	present	576.71	-0.7
1554261_at	included	present	271.12	4.08	excluded	absent	88.3	0.25	excluded	absent	39.64	-0.76	excluded	absent	2.4	-1.54	excluded	absent	130.82	1.14	excluded	absent	9	-1.4	excluded	absent	28.59	-0.99	excluded	absent	32.17	-0.92	excluded	absent	95.98	0.42	included	present	100.42	0.51	included	present	165.5	1.87	excluded	absent	4.33	-1.5	excluded	absent	34.3	-0.87	excluded	absent	24	-1.09	excluded	absent	85.17	0.19
1554262_s_a	included	present	153.35	2.22	excluded	absent	57.5	0.02	excluded	absent	2.04	-1.26	excluded	absent	2.53	-1.24	excluded	absent	5.16	-1.18	excluded	absent	156.15	2.29	excluded	absent	98.49	0.96	excluded	absent	72.26	0.36	excluded	absent	88.53	0.73	included	present	88.67	0.74	excluded	absent	73.55	0.39	excluded	absent	58.7	0.05	excluded	absent	57.94	0.03	excluded	absent	26.46	-0.69	included	present	49.77	-0.16
1554264_at	excluded	absent	65.64	0.78	excluded	absent	5.76	-0.83	included	present	98.64	1.67	excluded	absent	52.86	0.44	excluded	absent	143.91	2.9	excluded	absent	1.77	-0.94	included	present	66.57	0.81	included	present	176.55	3.78	included	present	52.06	0.42	excluded	absent	60.78	0.65	included	present	129.03	2.5	excluded	absent	83.57	1.27	included	present	135.08	2.66	excluded	absent	44.7	0.22	included	present	56.92	0.55
1554266_at	included	present	79.68	0.68	excluded	absent	79.06	0.66	excluded	absent	67.87	0.38	included	present	95.33	1.07	excluded	absent	72.05	0.48	included	present	162.28	2.77	excluded	absent	41.37	-0.3	excluded	absent	59.57	0.17	excluded	absent	46.68	-0.16	included	present	85.91	0.83	included	present	90.5	0.95	excluded	absent	70.25	0.44	excluded	absent	53.9	0.02	excluded	absent	48.39	-0.12	excluded	absent	24.44	-0.73
1554268_at	excluded	absent	71.12	0.66	excluded	absent	12.68	-0.85	excluded	absent	45.29	-0.01	excluded	absent	47.74	0.06	excluded	absent	5.86	-1.02	excluded	absent	4.07	-1.07	excluded	absent	12.64	-0.85	excluded	absent	25.86	-0.51	excluded	absent	71.68	0.67	excluded	absent	40.41	-0.13	excluded	absent	9.28	-0.93	excluded	absent	125.07	2.04	excluded	absent	61.75	0.42	excluded	absent	3.19	-1.09	excluded	absent	49.64	0.1
1554271_a_a	excluded	absent	59.04	-0.08	excluded	absent	84.11	0.38	included	present	240.24	3.21	included	present	107.8	0.81	excluded	absent	111.21	0.87	excluded	absent	68.8	0.1	excluded	absent	87.39	0.44	included	present	166.91	1.88	included	present	114.63	0.93	included	present	94.57	0.57	included	present	144.51	1.47	excluded	absent	109.71	0.84	excluded	absent	82.46	0.35	included	present	122.85	1.08	included	present	169.05	1.92
1554272_at	excluded	absent	25.4	0.03	excluded	absent	2.21	-0.66	excluded	absent	0.73	-0.7	excluded	absent	53.91	0.87	excluded	absent	3.9	-0.61	excluded	absent	2.26	-0.66	excluded	absent	29.95	0.16	excluded	absent	0.48	-0.71	excluded	absent	1.59	-0.68	excluded	absent	1.8	-0.67	excluded	absent	5.32	-0.57	excluded	absent	3.1	-0.63	excluded	absent	1.52	-0.68	excluded	absent	1.21	-0.69	excluded	absent	0.49	-0.71
1554273_a_a	included	present	487.23	-0.44	included	present	448.4	-0.47	included	present	135.02	-0.71	included	present	2291.05	0.96	included	present	945.56	-0.08	included	present	1411.91	0.28	included	present	349.65	-0.55	included	present	375.02	-0.53	included	present	313.41	-0.58	included	present	301.35	-0.59	included	present	120.29	-0.73	included	absent	64.76	-0.77	included	present	152.13	-0.7	included	present	105.47	-0.74	included	present	685.72	-0.29
1554274_a_a	included	present	415.52	1.69	included	absent	125.8	-1.25	included	present	218.85	-0.31	included	present	283.93	0.35	included	present	203.51	-0.46	included	present	374.91	1.28	included	present	135.86	-1.15	included	present	330.33	0.82	included	present	408.85	1.62	included	present	134.92	-1.16	included	present	363.38	1.16	included	present	294.3	0.46	included	present	203.49	-0.46	included	present	308.44	0.6	included	present	79.01	-1.73
1554276_at	excluded	absent	67.64	-0.14	excluded	absent	88.75	0.29	excluded	absent	84.66	0.21	included	present	88.27	0.28	excluded	absent	101.21	0.54	excluded	absent	60.43	-0.28	excluded	absent	60.38	-0.28	included	present	110.04	0.72	included	present	61.71	-0.26	excluded	absent	55.69	-0.38	included	present	315.33	4.84	excluded	absent	164.66	1.81	excluded	absent	51	-0.47	included	present	189.68	2.32	included	present	39.99	-0.69
1554277_s_a	included	present	540.28	0.11	included	present	1073.81	1.87	included	present	1445.58	3.09	included	present	984.98	1.57	included	present	714.55	0.68	included	present	148.51	-1.18	included	present	103.3	-1.33	included	present	181.4	-1.08	included	present	259.5	-0.82	included	present	232.05	-0.91	included	present	480.57	-0.09	included	present	210.19	-0.98	included	present	434.05	-0.24	included	present	642.38	0.44	included	present	282.08	-0.74
1554279_a_a	excluded	absent	65.6	0.85	excluded	absent	4.27	-0.96	excluded	absent	4.14	-0.96	excluded	absent	70.21	0.98	excluded	absent	68.48	0.93	included	present	101.67	1.91	excluded	absent	1.9	-1.03	excluded	absent	50.11	0.39	excluded	absent	61.76	0.73	excluded	absent	7.59	-0.86	excluded	absent	35.75	-0.03	excluded	absent	18.28	-0.55	excluded	absent	20.93	-0.47	excluded	absent	25.11	-0.35	excluded	absent	17.76	-0.56
1554280_a_a	excluded	absent	42.84	-0.22	excluded	absent	20.66	-0.28	excluded	absent	2.34	-0.33	excluded	absent	6.75	-0.31	excluded	absent	117.18	-0.02	excluded	absent	5.04	-0.32	excluded	absent	45.93	-0.21	excluded	absent	16.67	-0.29	excluded	absent	9.64	-0.31	excluded	absent	5.6	-0.32	included	present	86.11	-0.1	excluded	absent	20	-0.28	excluded	absent	35.6	-0.24	excluded	absent	31.54	-0.25	excluded	absent	35.09	-0.24
1554281_at	included	absent	2.81	-1.12	included	absent	1.55	-1.12	included	present	77.45	-0.75	included	absent	68.63	-0.79	included	present	176.11	-0.27	included	absent	16.24	-1.05	included	absent	1.2	-1.13	included	absent	36.74	-0.95	included	absent	7.14	-1.1	included	absent	6.82	-1.1	included	absent	61.36	-0.83	included	absent	4.24	-1.11	included	present	79.11	-0.74	included	present	174.14	-0.28	included	absent	9.69	-1.08
1554283_at	included	present	84.93	-0.6	included	present	245.16	0.39	included	present	386.5	1.27	included	present	239.45	0.36	included	present	298.75	0.72	included	present	350.19	1.04	included	present	184.14	0.01	included	present	237.18	0.34	included	present	258.47	0.47	included	present	179.96	-0.01	included	present	436.92	1.58	included	present	147.52	-0.21	included	present	92.2	-0.56	included	present	161.54	-0.13	included	present	271.47	0.55
1554285_at	included	present	270.09	0.53	included	present	108.91	-0.5	included	present	111.66	-0.49	included	absent	75.11	-0.72	included	present	285.15	0.62	included	present	222.7	0.22	included	absent	58.44	-0.83	included	absent	48.57	-0.89	included	present	190.77	0.02	included	present	89.38	-0.63	included	present	211.39	0.15	included	present	778.42	3.77	included	present	151.8	-0.23	included	absent	69.28	-0.76	included	present	265.04	0.49
1554286_at	excluded	absent	12.79	-0.25	excluded	absent	2.49	-0.29	excluded	absent	2.24	-0.29	excluded	absent	26.78	-0.2	excluded	absent	157.91	0.32	excluded	absent	121.86	0.18	excluded	absent	30.64	-0.18	excluded	absent	23.47	-0.21	excluded	absent	19.92	-0.22	excluded	absent	11.3	-0.26	excluded	absent	32.45	-0.17	excluded	absent	12.2	-0.25	excluded	absent	8.67	-0.27	excluded	absent	15.92	-0.24	excluded	absent	1.98	-0.29
1554287_at	included	present	1119.14	-0.13	included	present	4401.89	5.74	included	present	2321.68	2.02	included	present	4511.22	5.94	included	present	6715.49	9.88	included	present	329.32	-1.54	included	present	1194.27	0	included	present	1130.2	-0.11	included	present	888.74	-0.54	included	present	947.29	-0.44	included	present	2080.68	1.59	included	present	631.87	-1	included	present	1930.9	1.32	included	present	2820.6	2.91	included	present	373.89	-1.46
1554288_at	excluded	absent	2.72	-0.65	excluded	absent	3.42	-0.64	excluded	absent	2.18	-0.65	excluded	absent	1.44	-0.67	excluded	absent	82.65	0.57	excluded	absent	8.7	-0.56	excluded	absent	4.98	-0.61	excluded	absent	42.2	-0.05	excluded	absent	5.8	-0.6	excluded	absent	5.82	-0.6	excluded	absent	54.25	0.14	excluded	absent	29.95	-0.23	excluded	absent	9.18	-0.55	excluded	absent	16.3	-0.44	excluded	absent	39.62	-0.09
1554290_at	included	present	111.22	-0.85	included	absent	95.11	-1.02	included	present	92.78	-1.05	included	absent	60.75	-1.38	included	absent	112.07	-0.85	included	absent	2.27	-1.99	included	present	117.29	-0.79	included	absent	55.32	-1.44	included	present	136.71	-0.59	included	present	74.1	-1.24	included	present	108.62	-0.88	included	absent	8.54	-1.93	included	present	161.72	-0.33	included	present	62.98	-1.36	included	present	65.92	-1.33
1554291_at	included	absent	76.68	-1.02	included	present	109.25	-0.44	included	present	125.75	-0.14	included	present	113.93	-0.35	included	present	294.34	2.85	included	present	119.02	-0.26	included	present	121.34	-0.22	included	present	104.98	-0.51	included	present	263.44	2.3	included	present	152.46	0.33	included	present	496.84	6.45	included	present	163.93	0.53	included	absent	85.56	-0.86	included	present	196.26	1.11	included	present	191.69	1.03
1554292_a_a	included	present	994.48	-0.56	included	present	859.36	-0.87	included	present	915.64	-0.74	included	present	545.1	-1.57	included	present	861.61	-0.86	included	present	973.91	-0.61	included	present	1358.77	0.26	included	present	2034.18	1.78	included	present	1586.45	0.77	included	present	1183.49	-0.14	included	present	1451.28	0.46	included	present	1691.52	1	included	present	1660.24	0.93	included	present	2763.09	3.42	included	present	1248.01	0.01
1554293_at	included	present	253.93	-0.14	included	present	297.85	0.1	included	present	225.82	-0.3	included	present	216.96	-0.35	included	present	359.96	0.45	included	present	125.9	-0.85	included	present	370	0.5	included	present	385.52	0.59	included	present	355.82	0.43	included	present	481.37	1.12	included	present	330.64	0.29	included	present	84.84	-1.08	included	present	144.29	-0.75	included	present	145.04	-0.75	included	present	351.18	0.4
1554294_s_a	included	present	347.78	0.87	included	present	177.02	-0.35	included	present	338.88	0.81	included	present	144.98	-0.58	included	present	216.74	-0.07	included	absent	120.77	-0.76	included	present	257.98	0.23	included	present	178.16	-0.35	included	present	292.03	0.47	included	present	317.95	0.66	included	present	271.19	0.32	included	absent	102.88	-0.89	included	present	225.26	-0.01	included	absent	104.03	-0.88	included	present	240.09	0.1
1554295_x_a	included	present	302.17	0.3	included	present	257.89	0.04	included	present	270.22	0.11	included	present	179.35	-0.41	included	present	328.35	0.45	included	present	190.73	-0.35	included	present	401.84	0.87	included	present	265.82	0.09	included	present	311.87	0.35	included	present	353.67	0.6	included	present	229.28	-0.12	included	present	83.08	-0.97	included	present	312.35	0.36	included	present	146.98	-0.6	included	present	240.54	-0.06
1554296_at	excluded	absent	40.09	1.02	excluded	absent	42.03	1.1	excluded	absent	4.03	-0.54	excluded	absent	31.18	0.63	excluded	absent	3.35	-0.57	excluded	absent	47.73	1.35	excluded	absent	18.8	0.1	excluded	absent	43.63	1.17	excluded	absent	41.17	1.06	included	present	66.66	2.16	excluded	absent	2.1	-0.63	excluded	absent	30.09	0.58	excluded	absent	1.14	-0.67	excluded	absent	1.67	-0.64	excluded	absent	0.52	-0.69
1554298_a_a	excluded	absent	2.42	-1.17	excluded	absent	2.36	-1.17	excluded	absent	2.88	-1.16	excluded	absent	27.4	-0.61	excluded	absent	61	0.14	excluded	absent	45.35	-0.21	excluded	absent	2.7	-1.16	excluded	absent	2.72	-1.16	included	present	39.75	-0.34	excluded	absent	1.01	-1.2	excluded	absent	1.66	-1.19	excluded	absent	29.34	-0.57	excluded	absent	11.79	-0.96	excluded	absent	1.18	-1.2	excluded	absent	0.35	-1.22
1554299_at	excluded	absent	35.84	-0.32	excluded	absent	38.12	-0.26	excluded	absent	56.21	0.18	excluded	absent	12.89	-0.88	excluded	absent	11.13	-0.92	excluded	absent	62.32	0.33	included	present	101.92	1.29	excluded	absent	40.2	-0.21	included	present	53.45	0.11	excluded	absent	34.49	-0.35	excluded	absent	48.34	-0.01	excluded	absent	88.54	0.97	excluded	absent	17.34	-0.77	excluded	absent	28.79	-0.49	excluded	absent	40.63	-0.2
1554300_a_a	excluded	absent	34.91	-0.09	excluded	absent	6.65	-0.84	excluded	absent	29.34	-0.24	excluded	absent	8.07	-0.8	excluded	absent	7.01	-0.83	excluded	absent	4.93	-0.89	excluded	absent	46.9	0.23	excluded	absent	42.51	0.11	excluded	absent	32.85	-0.15	excluded	absent	25.93	-0.33	excluded	absent	30.26	-0.21	excluded	absent	51.29	0.34	excluded	absent	58.46	0.53	excluded	absent	28.59	-0.26	included	present	78.73	1.07
1554301_at	excluded	absent	45.59	0	excluded	absent	384.56	5.47	excluded	absent	1.45	-0.71	excluded	absent	1.06	-0.71	excluded	absent	9.8	-0.57	excluded	absent	3.74	-0.67	excluded	absent	0.9	-0.72	included	present	96.28	0.82	excluded	absent	0.68	-0.72	excluded	absent	0.89	-0.72	excluded	absent	2.02	-0.7	excluded	absent	6.55	-0.62	excluded	absent	13.76	-0.51	excluded	absent	14.4	-0.5	excluded	absent	2.44	-0.69

1554742_at	excluded	absent	41.22	-0.41	excluded	absent	2.13	-1.47	excluded	absent	85.52	0.8	excluded	absent	48.44	-0.21	excluded	absent	40.42	-0.43	excluded	absent	66.64	0.28
1554743_x_a	excluded	absent	35.09	-0.9	excluded	absent	8.57	-1.57	excluded	absent	155.45	2.14	excluded	absent	128.52	1.46	excluded	absent	81.65	0.28	included	present	92.37	0.55
1554744_at	included	present	70.84	0.46	excluded	absent	1.99	-0.82	excluded	absent	5.94	-0.75	excluded	absent	0.66	-0.84	excluded	absent	67.24	0.39	excluded	absent	15.94	-0.56
1554745_at	excluded	absent	18.8	-0.91	excluded	absent	67.84	0.53	excluded	absent	3.96	-1.35	excluded	absent	102.04	1.54	excluded	absent	132.44	2.44	excluded	absent	50.23	0.01
1554747_a_a	included	present	3995.07	1.99	included	present	2179.6	-0.35	included	present	2303.51	-0.19	included	present	2944.43	0.63	included	present	2056.92	-0.51	included	present	3446.11	1.28
1554748_at	excluded	absent	6.73	-0.24	included	present	205.39	-0.19	excluded	absent	17.61	-0.24	excluded	absent	79.12	-0.22	excluded	absent	144.45	-0.2	excluded	absent	16.71	-0.24
1554749_s_a	excluded	absent	2.64	-0.86	excluded	absent	5.33	-0.77	excluded	absent	31.88	0.14	excluded	absent	35.03	0.25	excluded	absent	68.41	1.4	excluded	absent	7.22	-0.7
1554751_at	excluded	absent	11.74	-1.14	excluded	absent	52.9	0.08	excluded	absent	44.17	-0.18	included	present	64.62	0.43	excluded	absent	100.53	1.5	excluded	absent	54.35	0.13
1554752_a_a	excluded	absent	11.11	-0.86	excluded	absent	81.12	1.24	excluded	absent	39.94	0	excluded	absent	46.83	0.21	excluded	absent	6.35	-1	excluded	absent	12.06	-0.83
1554755_a_a	excluded	absent	61.99	-0.2	excluded	absent	55.15	-0.27	excluded	absent	3.74	-0.78	included	present	198.23	1.16	excluded	absent	72.02	-0.1	excluded	absent	14.46	-0.67
1554757_a_a	included	present	232	-0.62	included	absent	82.25	-1.02	included	present	92.23	-1	included	present	118.41	-0.93	included	absent	126.16	-0.91	included	present	347.34	-0.31
1554758_a_a	included	present	184.53	0.51	excluded	absent	32.69	-1.18	excluded	absent	3.33	-1.51	excluded	absent	109.36	-0.33	excluded	absent	90.33	-0.54	included	present	279.13	1.57
1554759_at	excluded	absent	4.69	-0.87	excluded	absent	51.82	0.04	excluded	absent	5.23	-0.86	excluded	absent	3.94	-0.89	excluded	absent	63	0.26	excluded	absent	1.51	-0.94
1554761_a_a	excluded	absent	205.53	0.66	included	present	264.62	1.56	included	present	289.52	1.94	included	present	208.42	0.7	excluded	absent	246.31	1.28	included	present	280.27	1.8
1554762_a_a	included	present	371.3	0.63	excluded	absent	69.57	-1.27	excluded	absent	11.23	-1.63	included	present	197.05	-0.46	excluded	absent	188.11	-0.52	included	present	350.7	0.5
1554763_at	excluded	absent	42.76	0.27	excluded	absent	3.47	-0.74	excluded	absent	39.21	0.18	excluded	absent	5.4	-0.69	excluded	absent	62.66	0.78	excluded	absent	7.95	-0.63
1554764_a_a	excluded	absent	1.19	-0.25	excluded	absent	1.27	-0.25	excluded	absent	2.78	-0.25	excluded	absent	13.97	-0.22	excluded	absent	81.88	-0.03	excluded	absent	41.32	-0.14
1554765_a_a	excluded	absent	1.83	-0.26	excluded	absent	3.55	-0.25	excluded	absent	41.16	-0.19	excluded	absent	4.17	-0.25	excluded	absent	94.45	-0.09	excluded	absent	45.34	-0.18
1554766_s_a	excluded	absent	39.04	-0.34	excluded	absent	68.82	0.39	excluded	absent	8.3	-1.1	excluded	absent	5.89	-1.15	excluded	absent	108.13	1.35	excluded	absent	72.96	0.49
1554767_s_a	included	present	1363.92	0.28	included	present	412.5	-1.94	included	present	1561.27	0.74	included	present	1634.69	0.91	included	present	1373.54	0.3	included	present	588.68	-1.53
1554768_a_a	included	present	115.65	-0.18	included	present	359.81	3.32	included	present	1010.81	12.65	included	present	448.57	4.59	included	present	657.48	7.58	excluded	absent	90.08	-0.55
1554769_at	included	present	89.64	0.45	excluded	absent	67.29	-0.09	included	present	142.86	1.73	excluded	absent	43.15	-0.66	excluded	absent	103.04	0.77	included	present	71.4	0.01
1554770_x_a	excluded	absent	92.46	0.43	excluded	absent	98.34	0.54	included	present	123.88	1.04	excluded	absent	54.04	-0.33	excluded	absent	6.89	-1.25	included	present	42.51	-0.55
1554771_at	included	present	413.51	0.59	included	present	271.15	-0.13	included	present	334.84	0.19	included	present	411.77	0.58	included	present	247.72	-0.25	included	absent	128.22	-0.86
1554772_at	included	present	338.13	1.03	included	absent	197.09	-0.42	included	present	474.91	2.44	included	present	329.9	0.95	included	absent	129.48	-1.12	included	present	167.16	-0.73
1554773_at	excluded	absent	58.51	1.47	excluded	absent	4.64	-0.71	excluded	absent	52.23	1.22	excluded	absent	5.28	-0.68	excluded	absent	2.57	-0.79	excluded	absent	9.51	-0.51
1554774_at	included	present	305.19	0.26	included	present	249.57	-0.29	included	present	240.48	-0.39	included	absent	63.78	-2.15	included	present	264.89	-0.14	included	present	212.77	-0.66
1554776_at	excluded	absent	47.64	0.4	excluded	absent	19.82	-0.29	excluded	absent	48.1	0.41	excluded	absent	102.29	1.76	excluded	absent	6.95	-0.61	excluded	absent	63.77	0.8
1554777_at	excluded	absent	10.38	-1.06	excluded	absent	49.71	-0.05	excluded	absent	49.02	-0.07	included	present	99	1.21	excluded	absent	116.68	1.66	excluded	absent	36.96	-0.38
1554778_at	excluded	absent	33.48	-0.52	excluded	absent	3.12	-1.37	excluded	absent	38.83	-0.37	excluded	absent	22.58	-0.82	excluded	absent	66.62	0.42	excluded	absent	10.15	-1.18
1554779_s_a	excluded	absent	57.17	-0.34	excluded	absent	5.22	-0.98	excluded	absent	3.06	-1	included	present	93.67	0.1	excluded	absent	2.12	-1.01	excluded	absent	71.41	-0.17
1554780_a_a	included	present	193.63	0.22	included	present	148.87	-0.21	included	present	381.55	2	included	present	506.5	3.19	included	present	370.3	1.9	included	present	194.84	0.23
1554781_at	excluded	absent	5.88	-1.23	excluded	absent	43.02	-0.64	excluded	absent	7.32	-1.21	included	present	117.86	0.56	excluded	absent	3.73	-1.26	excluded	absent	8.94	-1.18
1554782_at	excluded	absent	13.47	-0.34	excluded	absent	4.76	-0.63	excluded	absent	4.9	-0.62	excluded	absent	4.46	-0.63	excluded	absent	17.12	-0.22	excluded	absent	7.12	-0.55
1554783_s_a	excluded	absent	70.4	0.1	excluded	absent	9.49	-0.9	excluded	absent	143.76	1.31	excluded	absent	125.51	1.01	excluded	absent	74.85	0.18	excluded	absent	86.14	0.36
1554784_at	included	present	190.47	-0.36	included	present	233.46	-0.25	included	absent	113.89	-0.55	excluded	absent	33.49	-0.74	included	present	374.05	0.09	excluded	absent	16.58	-0.79
1554785_at	excluded	absent	56.44	-0.49	excluded	absent	80.64	0.09	excluded	absent	93.91	0.4	included	present	60.78	-0.38	excluded	absent	86.13	0.22	included	present	134.1	1.36
1554786_at	included	present	91.38	0.69	included	present	81.99	0.5	excluded	absent	77.23	0.4	excluded	absent	2.55	-1.11	excluded	absent	159.74	2.08	excluded	absent	48.46	-0.18
1554787_at	excluded	absent	2.05	-0.76	excluded	absent	51.56	0.88	excluded	absent	42.59	0.58	excluded	absent	31.45	0.21	excluded	absent	9.67	-0.51	excluded	absent	17.65	-0.25
1554789_a_a	included	present	519.61	-0.76	included	present	101.61	-1.14	included	present	423.75	-0.85	included	present	296.75	-0.96	included	present	424.43	-0.85	included	present	263.56	-0.99
1554790_at	excluded	absent	71.12	1.54	excluded	absent	2.7	-0.82	excluded	absent	38.82	0.42	excluded	absent	88.11	2.12	excluded	absent	94.39	2.34	excluded	absent	12	-0.5
1554791_a_a	included	present	597.75	0.29	included	present	354.54	-0.42	included	present	501.3	0.01	included	present	1289.3	2.32	included	present	465.56	-0.09	included	present	298.59	-0.58
1554793_at	excluded	absent	0.67	-1.66	excluded	absent	1.71	-1.62	excluded	absent	7.22	-1.41	excluded	absent	58.94	0.62	excluded	absent	15.01	-1.1	excluded	absent	96.71	2.1
1554794_a_a	included	present	350.56	-0.35	included	present	333.31	-0.45	included	present	616.25	1.18	included	present	638.04	1.3	included	present	418.75	0.04	included	present	344.15	-0.39
1554795_a_a	included	present	112.25	0.97	excluded	absent	4.78	-0.99	excluded	absent	22.04	-0.67	excluded	absent	4.91	-0.98	excluded	absent	64.3	0.1	included	present	506.49	8.13
1554796_at	excluded	absent	44.45	-0.26	included	present	259.97	3.54	excluded	absent	5.94	-0.94	excluded	absent	22.77	-0.64	excluded	absent	26.98	-0.57	excluded	absent	115.89	1
1554797_at	excluded	absent	3.14	-0.96	excluded	absent	37.12	-0.6	excluded	absent	66.95	-0.3	excluded	absent	2.93	-0.96	excluded	absent	7	-0.92	excluded	absent	2.04	-0.97
1554798_at	included	present	848.66	2.04	excluded	absent	123.68	-0.66	excluded	absent	5.02	-1.1	excluded	absent	276.24	-0.09	excluded	absent	179.57	-0.45	included	present	807.36	1.89
1554800_at	included	present	409.2	3.73	included	present	578.73	5.82	included	present	2687.4	31.87	included	present	1239.55	13.99	included	present	458.39	4.34	included	present	209.18	1.26
1554801_at	excluded	absent	72.04	-0.4	included	present	160.72	-0.2	excluded	absent	82.11	-0.38	included	present	107.27	-0.32	included	present	232.63	-0.03	excluded	absent	38.59	-0.48
1554803_s_a	excluded	absent	45.07	-0.25	excluded	absent	82.18	-0.2	excluded	absent	79.57	-0.2	excluded	absent	83.2	-0.2	excluded	absent	93.3	-0.19	included	present	772.04	0.71
1554804_a_a	included	present	533.23	11.54	excluded	absent	3.6	-0.77	excluded	absent	2.5	-0.8	excluded	absent	2.77	-0.79	excluded	absent	7	-0.69	excluded	absent	47.19	0.24
1554805_at	excluded	absent	90.23	1.1	excluded	absent	52.66	0.21	excluded	absent	3	-0.98	excluded	absent	35.23	-0.21	excluded	absent	7.03	-0.88	excluded	absent	3.88	-0.95
1554806_a_a	included	present	311.13	-0.64	included	present	227.55	-1.27	included	present	2188.35	13.62	included	present	450.44	0.42	included	present	328.81	-0.5	included	present	263.58	-1
1554807_a_a	included	present	3034.13	0.93	included	present	2722.62	0.64	included	present	2890.43	0.8	included	present	2997.55	0.9	included	present	2457.21	0.39	included	present	932.8	-1.04
1554808_at	excluded	absent	130.08	0.13	excluded	absent	23.26	-1.56	included	present	284.24	2.58	included	present	125.25	0.06	excluded	absent	79.52	-0.67	included	present	41.43	-1.27
1554809_at	excluded	absent	45.53	-0.12	excluded	absent	38.34	-0.29	excluded	absent	51.12	0	excluded	absent	52.19	0.03	excluded	absent	131.3	1.8	excluded	absent	74.6	0.53
1554810_at	excluded	absent	68.8	-0.26	excluded	absent	92.82	0.12	excluded	absent	87.39	0.04	excluded	absent	70.04	-0.24	excluded	absent	107.3	0.35	excluded	absent	105.57	0.33
1554812_at	included	absent	195.62	-0.77	included	absent	87.18	-1.59	included	present	317.91	0.15	included	present	410.21	0.85	included	present	263.24	-0.26	included	absent	209.07	-0.67
1554813_at	excluded	absent	2.89	-1.13	excluded	absent	92.75	1.41	included	present	149.9	3.02	excluded	absent	32.13	-0.3	excluded	absent	40.4	-0.07	excluded	absent	65.86	0.65
1554814_at	excluded	absent	58.12	0.09	excluded	absent	63.01	0.18	excluded	absent	113.09	1.14	excluded	absent	48.34	-0.1	excluded	absent	127.21	1.41	excluded	absent	115.51	1.18
1554816_at	excluded	absent	86.19	0.3	excluded	absent	26.85	-0.74	excluded	absent	73.69	0.08	excluded	absent	2.38	-1.16	excluded	absent	190.62	2.11	excluded	absent	10.8	-1.02
1554818_s_a	included	present	229.59	0.55	included	present	579.34	3.67	included	present	208.69	0.36	included	present	232.38	0.57	included	present	438.79	2.42	included	absent	117.8	-0.45
1554819_a_a	excluded	absent	74.45	-0.48	excluded	absent	59.56	-0.57	excluded	absent	6.18	-0.89	excluded	absent	84.98	-0.42	included	present	121.48	-0.2	included	present	414.38	1.56
1554820_at	excluded	absent	115.74	-0.03	excluded	absent	56.93	-0.6	excluded	absent	5.58	-1.09	excluded	absent	35.69	-0.8	excluded	absent	174.82	0.55	excluded	absent	58.12	-0.58
1554821_a_a	included	present	237.54	-0.24	included	present	176.53	-0.69	included	absent	100.05	-1.25	included	present	320.63	0.38	included	present	222.17	-0.35	included	present	494.18	1.66
1554822_at	included	present	209.58	-0.78	included	present	193.5	-0.83	included	present	436.97	0.03	included	present	475.84	0.17	included	present	539.64	0.39	included	present	218.08	-0.75
1554824_at	included	present	401.34	-0.75	included	present	318.17	-1.18	included	present	572.98	0.13	included	present	751.38	1.05	included	absent	314.63	-1.19	included	present	291.89	-1.31
1554825_at	included	present	333.54	0.76	included	present	210.5	-0.03	included	present	239.13	0.16	included	absent	142.8	-0.46	included	present	211.59	-0.02	included	present	173.74	-0.26
1554826_at	excluded	absent	122.09	1.62	excluded	absent	5.18	-1.7	excluded	absent	70.09	0.14	excluded	absent	76.16	0.32	excluded	absent	26.21	-1.1	excluded	absent	141.76	2.18
1554827_a_a	excluded	absent	12.41	-0.37	excluded	absent	20.18	-0.14	excluded	absent	17.69	-0.22	excluded	absent	14	-0.33	excluded	absent	4.06	-0.62	excluded	absent	25.16	0.01
1554828_at	excluded	absent	46.94	-0.61	included	present	117.9	0.03	excluded	absent	2.24	-1.01	excluded	absent	14.76	-0.9	excluded	absent	114.59	0	excluded	absent	3.95	-1
1554830_a_a	included	present	863.49	0.56	included	present	295.96	-0.12	included	present	267.65	-0.15	included	present	156.56	-0.28	included	present	248.63	-0.17	included	present	263.67	-0.16
1554831_x_a	included	present	83.44	-0.23	included	present	193.16	0	excluded	absent	10.98	-0.38	included	present	155.62	-0.08	included	absent	132.04	-0.13	included	absent	57.74	-0.29
1554833_at	excluded	absent	73.53	-0.07	excluded	absent	3.32	-0.84	excluded	absent	69.27	-0.12	included	present	153.1	0.8	excluded	absent	28.44	-0.57	excluded	absent	8.44	-0.79
1554834_a_a	included	present	204.88	-0.06	excluded	absent	4.97	-0.77	excluded	absent	18.36	-0.72	included	present	203.2	-0.07	included	present	141.62	-0.29	included	present	200.72	-0.08
1554835_a_a	included	present	268.25	-0.38	included	present	316.16	-0.25	included	present	444.39	0.11	included	present	1422.09	2.85	included	present	513.88	0.3	included	present	106.5	-0.84
1554836_at	excluded	absent	26.98	-1.19	excluded	absent	51.8	-0.82	excluded	absent	105.13	-0.02	excluded	absent	100.85	-0.08	excluded	absent	96.1	-0.15	excluded	absent	2.2	-1.56

1555219_at	excluded	absent	110.12	1.49	excluded	absent	3.92	-0.66	excluded	absent	2.47	[illegible]
1555220_a_a	included	absent	114.33	-0.52	included	present	195.7	-0.32	included	present	585.3	[illegible]
1555221_at	excluded	absent	3.08	-0.91	excluded	absent	58.55	0.75	excluded	absent	38.6	[illegible]
1555222_a_a	excluded	absent	74.26	0.2	excluded	absent	42.94	-0.4	excluded	absent	38.92	[illegible]
1555224_at	excluded	absent	10.25	-0.36	excluded	absent	5.44	-0.54	excluded	absent	11.65	[illegible]
1555225_at	included	present	700.2	-0.67	included	present	1280.43	0.18	included	present	2625.4	[illegible]
1555226_s_a	included	present	5539.77	1.31	included	present	3428.21	-0.17	included	present	12279.23	[illegible]
1555227_a_a	included	present	183.38	0.14	included	present	169.48	0.02	included	present	180.43	[illegible]
1555229_a_a	included	present	1827.69	0.55	included	present	181.29	-0.5	included	present	209.55	[illegible]
1555230_a_a	excluded	absent	6.93	-0.61	excluded	absent	51.26	-0.43	excluded	absent	2.62	[illegible]
1555231_a_a	excluded	absent	77.77	0.01	excluded	absent	97.56	0.27	excluded	absent	38.8	[illegible]
1555233_at	included	present	7421.09	3.59	included	present	488.28	-0.93	included	present	211.7	[illegible]
1555234_a_a	included	present	1934.65	3.86	included	present	107.74	-1.2	included	absent	78.87	[illegible]
1555235_s_a	excluded	absent	23.04	-0.22	excluded	absent	11.92	-0.23	excluded	absent	2.98	[illegible]
1555236_a_a	excluded	absent	55.15	1.24	excluded	absent	30.3	0.25	excluded	absent	2.85	[illegible]
1555237_at	excluded	absent	85.46	1.2	excluded	absent	8.6	-0.73	excluded	absent	3.14	[illegible]
1555238_at	excluded	absent	83.92	0.94	excluded	absent	10.84	-0.77	excluded	absent	71.46	[illegible]
1555240_s_a	included	present	3353.37	1.81	included	present	332.41	-1.05	included	present	289.41	[illegible]
1555241_at	included	present	707.59	0.93	included	present	828.72	1.41	included	present	858.11	[illegible]
1555243_x_a	included	present	738.96	0.67	included	present	1118.5	1.99	included	present	929.46	[illegible]
1555244_at	excluded	absent	66.11	0.68	excluded	absent	51.72	0.3	excluded	absent	37.1	[illegible]
1555245_s_a	excluded	absent	61.13	0.74	excluded	absent	4.26	-0.96	excluded	absent	70.08	[illegible]
1555246_a_a	excluded	absent	3.5	-0.73	excluded	absent	61.68	-0.53	excluded	absent	39.97	[illegible]
1555247_a_a	included	present	1374.08	0.39	included	present	1028.75	-0.09	included	present	4502.84	[illegible]
1555248_a_a	included	present	108.28	-0.62	included	present	328.46	0.48	included	present	175.56	[illegible]
1555250_a_a	included	present	522.72	-0.28	included	present	268.84	-0.92	included	present	478.69	[illegible]
1555251_a_a	excluded	absent	50.54	2.34	excluded	absent	5.55	-0.36	excluded	absent	5.02	[illegible]
1555252_a_a	excluded	absent	197.63	0.19	excluded	absent	52.22	-0.59	excluded	absent	78.86	[illegible]
1555253_at	included	present	110.66	-0.47	included	absent	3.46	-0.77	included	absent	63.09	[illegible]
1555255_a_a	included	present	152.29	-0.35	included	absent	53.17	-1.44	included	present	249.34	[illegible]
1555256_at	included	present	83.18	0.44	excluded	absent	4.69	-0.99	excluded	absent	37.64	[illegible]
1555257_a_a	included	present	104.61	0.45	excluded	absent	37.08	-0.74	included	present	81.71	[illegible]
1555259_at	excluded	absent	4.46	-1.29	excluded	absent	32.62	-0.97	excluded	absent	77.27	[illegible]
1555261_at	included	present	88.36	-0.74	included	present	101.78	-0.53	excluded	absent	136.21	[illegible]

1555711_x_a	excluded	absent	27.77	-0.25	excluded	absent	2.79	-1.02	excluded	absent	4.6	-0.97	excluded	absent	49.58	0.43	excluded	absent	10.83	-0.77	excluded	absent	6.56	-0.91	excluded	absent	3.62	-1	excluded	absent	8.47	-0.85
1555713_at	excluded	absent	160.28	2.55	excluded	absent	70.42	0.42	excluded	absent	87.03	0.81	excluded	absent	6.8	-1.09	excluded	absent	223.02	4.04	excluded	absent	63.53	0.25	excluded	absent	54.31	0.04	excluded	absent	10.02	-1.02
1555715_a_a	excluded	absent	3.47	-1.78	excluded	absent	16.27	-1.51	excluded	absent	65.29	-0.44	excluded	absent	7.94	-1.69	included	present	175.21	1.95	excluded	absent	7.45	-1.7	excluded	absent	119.54	0.74	excluded	absent	72.97	-0.27
1555716_a_a	excluded	absent	4.03	-1.32	included	present	196.75	0.35	included	present	292.91	1.19	included	present	469.5	2.72	excluded	absent	263.55	0.93	excluded	absent	35.39	-1.05	included	present	96.14	-0.52	excluded	absent	73.02	-0.72
1555717_at	included	absent	51.16	-0.96	included	absent	80.62	-0.67	included	present	161.15	0.11	included	absent	121.53	-0.27	included	present	149.21	-0.01	included	present	81.55	-0.66	included	present	71.73	-0.76	included	present	70.13	-0.78
1555718_x_a	included	absent	117.84	-0.59	included	present	110.16	-0.65	included	present	173.26	-0.14	included	present	145.08	-0.37	included	present	128.63	-0.5	included	present	163.2	-0.22	included	present	128.91	-0.5	included	absent	58.89	-1.07
1555719_a_a	included	present	169.26	-0.63	included	present	244.28	-0.48	included	absent	61.89	-0.85	included	present	554.49	0.14	included	present	153.74	-0.66	included	absent	93.17	-0.79	included	absent	48.86	-0.87	included	present	164.13	-0.64
1555722_at	excluded	absent	38.77	0.32	excluded	absent	4.23	-0.77	excluded	absent	1.78	-0.85	excluded	absent	24.18	-0.14	excluded	absent	54.4	0.82	excluded	absent	2.15	-0.84	excluded	absent	1.55	-0.86	excluded	absent	10.25	-0.58
1555723_at	included	present	190.76	0.24	included	absent	46.84	-1.18	included	absent	102.1	-0.64	included	present	82.32	-0.83	included	present	116.41	-0.49	included	present	183.61	0.16	included	present	190.54	0.23	included	present	176.87	0.1
1555724_s_a	excluded	absent	172.08	-0.46	excluded	absent	156.9	-0.48	excluded	absent	137.63	-0.5	included	present	220.34	-0.41	excluded	absent	183.02	-0.45	included	present	5481.23	5.06	excluded	absent	23.61	-0.62	included	present	131.41	-0.5
1555725_a_a	included	present	705.1	0.06	included	present	2344.91	2.41	included	present	259.76	-0.58	included	present	759.11	0.14	included	present	7189.88	9.35	included	present	1911.48	1.79	included	present	8653.56	11.45	included	present	23434.13	32.63
1555726_at	included	present	653.17	0.18	included	present	384.95	-0.5	included	present	350.41	-0.58	included	present	563.23	-0.05	included	present	345.85	-0.59	included	present	415.63	-0.42	included	present	90.66	-1.23	included	present	411.07	-0.43
1555727_at	excluded	absent	68.67	1.46	excluded	absent	3.95	-0.88	excluded	absent	5.54	-0.82	excluded	absent	31.32	0.11	excluded	absent	65.7	1.35	excluded	absent	43.8	0.56	excluded	absent	4.86	-0.84	excluded	absent	22.44	-0.21
1555728_a_a	included	present	566.7	0.81	included	present	561.9	0.79	included	present	766.18	1.52	included	present	87.02	-0.91	included	present	696.95	1.27	included	present	1089.59	2.67	included	present	919.59	2.07	included	present	162.41	-0.64
1555729_a_a	included	present	96.65	-0.18	excluded	absent	87.79	-0.19	excluded	absent	5.25	-0.28	excluded	absent	68.19	-0.21	excluded	absent	141.95	-0.13	included	present	493.32	0.25	excluded	absent	77.3	-0.2	included	present	112.51	-0.16
1555730_a_a	included	present	2347.33	0.28	included	present	2495.67	0.46	included	present	1748.76	-0.44	included	present	1107.43	-1.21	included	present	2809.04	0.83	included	present	4531.93	2.9	included	present	2526.04	0.49	included	present	4374.78	2.71
1555731_a_a	excluded	absent	111.98	0.07	excluded	absent	69.63	-0.4	excluded	absent	57.78	-0.52	excluded	absent	32.45	-0.8	excluded	absent	108.19	0.03	excluded	absent	63.87	-0.46	excluded	absent	74.07	-0.35	excluded	absent	61.26	-0.49
1555733_s_a	excluded	absent	41.05	-0.19	excluded	absent	5.88	-0.83	excluded	absent	3.93	-0.87	excluded	absent	70.94	0.36	excluded	absent	78.95	0.5	excluded	absent	79.06	0.5	excluded	absent	37.43	-0.26	excluded	absent	49.72	-0.03
1555734_x_a	excluded	absent	59.72	-0.41	excluded	absent	1.82	-1.03	excluded	absent	4.86	-1	excluded	absent	91.54	-0.07	excluded	absent	134.46	0.39	excluded	absent	47.02	-0.55	excluded	absent	62.21	-0.38	excluded	absent	61.06	-0.4
1555735_a_a	excluded	absent	39.33	-0.81	excluded	absent	93.93	0.51	included	present	104.84	0.77	excluded	absent	39.09	-0.82	excluded	absent	4.33	-1.66	included	present	190.51	2.84	included	present	174.47	2.45	included	present	121.92	1.18
1555736_a_a	included	present	158.4	0.34	excluded	absent	111.25	-0.37	included	present	89.88	-0.69	excluded	absent	103.96	-0.48	excluded	absent	122.05	-0.2	included	present	276.26	2.12	excluded	absent	34.9	-1.52	included	present	170.89	0.53
1555737_a_a	excluded	absent	109.27	0.27	excluded	absent	74.18	0.08	excluded	absent	109.67	0.27	excluded	absent	5.25	-0.3	excluded	absent	5.59	-0.3	excluded	absent	4.79	-0.3	excluded	absent	61.76	0.01	included	present	265.24	1.13
1555738_at	excluded	absent	88.49	1.05	excluded	absent	175.38	3.06	excluded	absent	110.03	1.55	excluded	absent	16.35	-0.63	excluded	absent	78.72	0.82	excluded	absent	120.23	1.78	excluded	absent	151.1	2.5	excluded	absent	132.63	2.07
1555740_a_a	excluded	absent	1.27	-0.66	excluded	absent	3.79	-0.6	excluded	absent	4.92	-0.58	excluded	absent	3.4	-0.61	excluded	absent	77.21	1.1	excluded	absent	6.41	-0.54	excluded	absent	1.63	-0.65	excluded	absent	1.54	-0.65
1555741_at	included	present	266.28	1.07	included	present	318.74	1.69	included	present	272.13	1.14	included	present	322.65	1.73	included	present	264.98	1.05	included	present	150.08	-0.3	included	present	201.46	0.31	included	present	234.05	0.69
1555742_at	excluded	absent	1.8	-0.9	excluded	absent	4.79	-0.81	excluded	absent	55.33	0.72	excluded	absent	69.31	1.14	excluded	absent	75.06	1.32	excluded	absent	6.04	-0.77	excluded	absent	60.56	0.88	excluded	absent	6.32	-0.76
1555743_s_a	excluded	absent	122.09	0.05	excluded	absent	53.65	-0.82	excluded	absent	3.27	-1.47	excluded	absent	133.6	0.2	excluded	absent	141.3	0.3	excluded	absent	116.38	-0.02	excluded	absent	183.31	0.83	excluded	absent	41.38	-0.98
1555745_a_a	included	present	917.17	-0.28	included	present	2443.01	-0.22	included	present	1794.01	-0.24	included	present	842.98	-0.28	included	present	7723.71	-0.01	included	present	21254.93	0.52	included	present	1575.35	-0.25	included	present	627.92	-0.29
1555746_at	excluded	absent	118.34	0.2	excluded	absent	92.82	-0.33	excluded	absent	56.69	-1.08	excluded	absent	10.49	-2.04	excluded	absent	65.73	-0.89	excluded	absent	14	-1.96	excluded	absent	96.01	-0.27	excluded	absent	87.1	-0.45
1555748_x_a	excluded	absent	44.2	-0.8	excluded	absent	102.07	0.15	excluded	absent	104.08	0.18	included	present	104.68	0.19	included	present	167.84	1.23	excluded	absent	91.96	-0.02	excluded	absent	85.26	-0.13	excluded	absent	18.83	-1.22
1555749_at	included	present	616.02	-0.13	included	present	222.36	-1.51	included	present	380.99	-0.95	included	present	521.64	-0.46	included	present	627.39	-0.09	included	present	433.66	-0.77	included	present	376.61	-0.97	included	present	490.15	-0.57
1555751_a_a	excluded	absent	211.93	1.64	excluded	absent	205.21	1.52	excluded	absent	117.18	0.03	excluded	absent	248.9	2.26	excluded	absent	54.23	-1.04	included	present	323.54	3.53	included	present	340.29	3.82	included	present	431.61	5.37
1555752_at	included	present	135.58	-0.53	included	present	382.85	0.39	included	present	849.1	2.12	excluded	absent	183.29	-0.35	included	present	553.15	1.02	included	present	65.15	-0.79	included	present	574.63	1.1	included	present	171.25	-0.4
1555753_x_a	excluded	absent	5.3	-1.09	excluded	absent	18.29	-0.78	excluded	absent	39.51	-0.26	excluded	absent	96.61	1.11	excluded	absent	80.3	0.72	excluded	absent	58.92	0.2	excluded	absent	82.79	0.78	excluded	absent	8.51	-1.01
1555754_s_a	included	present	758.15	0.59	included	present	1546.15	3.43	included	present	700.67	0.38	included	present	663.3	0.25	included	present	775.34	0.65	included	present	1173.05	2.09	included	present	1612.1	3.67	included	present	987.66	1.42
1555755_at	excluded	absent	57.1	1.34	excluded	absent	8.11	-0.59	excluded	absent	2.9	-0.8	excluded	absent	3.92	-0.76	excluded	absent	5.89	-0.68	excluded	absent	5.76	-0.68	excluded	absent	49	1.02	excluded	absent	3.23	-0.78
1555756_a_a	included	present	276.15	2.23	excluded	absent	50.69	-0.72	excluded	absent	3.53	-1.34	excluded	absent	1.46	-1.36	excluded	absent	9.11	-1.26	included	present	332.23	2.96	included	present	266.96	2.11	included	present	103.54	-0.03
1555757_at	excluded	absent	43.31	-0.41	excluded	absent	13.36	-0.77	excluded	absent	61.03	-0.2	excluded	absent	22.02	-0.66	excluded	absent	73.91	-0.05	excluded	absent	61.78	-0.19	excluded	absent	10.61	-0.8	excluded	absent	24.69	-0.63
1555758_a_a	included	present	284.04	-0.16	included	present	1216.93	0.31	included	present	2611.19	1.01	included	present	1307.92	0.35	included	present	1729.67	0.56	included	present	448.61	-0.08	included	present	121.14	-0.24	included	present	1789.13	0.59
1555759_a_a	included	present	486.6	0.18	included	present	295.74	-0.13	included	absent	90.87	-0.46	included	absent	121.54	-0.41	included	present	299.04	-0.12	included	present	2035.04	2.67	included	present	768.19	0.63	included	present	280.92	-0.15
1555760_a_a	included	present	1002.21	0.79	included	present	901.78	0.44	included	present	2123.62	4.67	included	present	2190.81	4.91	included	present	2111.1	4.63	included	present	252.45	-1.8	included	present	329.78	-1.54	included	present	660.19	-0.39
1555761_x_a	included	present	414.83	0.56	included	present	285.21	-0.46	included	present	297.35	-0.36	included	present	391.13	0.37	included	present	558.44	1.68	included	present	293.32	-0.39	included	present	296.94	-0.37	included	present	318.88	-0.19
1555762_s_a	included	present	1280.09	0.25	included	present	1161.07	-0.04	included	present	2169.76	2.43	included	present	1870.72	1.7	included	present	1560.81	0.94	included	present	1097.52	-0.2	included	present	598.89	-1.42	included	present	1000	-0.44
1555763_x_a	excluded	absent	258.74	0.8	excluded	absent	173.12	-0.27	excluded	absent	146.97	-0.6	excluded	absent	167.41	-0.34	excluded	absent	237.31	0.53	excluded	absent	170.93	-0.3	excluded	absent	255.71	0.76	excluded	absent	141.48	-0.67
1555764_s_a	included	present	3169.27	0.42	included	present	5648.86	2.67	included	present	7027.52	3.93	included	present	2567.56	-0.13	included	present	3473.26	0.69	included	present	1979.25	-0.67	included	present	1864.78	-0.77	included	present	3197.48	0.44
1555765_a_a	included	present	439.73	1.08	included	present	1154.11	4.22	included	present	2117.39	8.45	included	present	1389.16	5.25	included	present	1654.12	6.41	included	present	206.8	0.06	included	present	1396.78	5.28	included	present	3674.86	15.28
1555766_a_a	included	present	4661.4	4.61	included	present	1987.89	1.48	included	present	854.5	0.16	included	present	997.55	0.32	included	present	2854.71	2.5	included	present	804.45	0.1	included	present	6050.01	6.23	included	present	1509.24	0.92
1555772_a_a	excluded	absent	4.61	-0.75	included	present	193.55	2.31	included	present	278.7	3.69	included	present	216.19	2.68	excluded	absent	91.31	0.66	excluded	absent	67.05	0.27	excluded	absent	44.96	-0.09	included	present	159.62	1.77
1555773_at	excluded	absent	69.08	-0.26	excluded	absent	52.42	-0.29	excluded	absent	6.47	-0.36	excluded	absent	2.25	-0.36	excluded	absent	100.43	-0.21	excluded	absent	3.07	-0.36	excluded	absent	32.68	-0.32	excluded	absent	25.29	-0.33
1555774_at	excluded	absent	9.19	-1.39	excluded	absent	39.49	-0.8	included	present	1176.38	21.38	excluded	absent	54.03	-0.52	included	present	319.66	4.67	excluded	absent	51.72	-0.56	included	present	92	0.22	included	present	550.48	9.17
1555775_a_a	excluded	absent	6.5	-0.63	excluded	absent	41.31	-0.02	included	present	205.39	2.86	excluded	absent	43.29	0.02	excluded	absent	63.49	0.37	excluded	absent	45.84	0.06	excluded	absent	32.04	-0.18	included	present	743.86	12.33
1555777_at	excluded	absent	5.63	-0.85	excluded	absent	55.71	0	excluded	absent	35.91	-0.34	excluded	absent	60.19	0.07	excluded	absent	199.13	2.41	excluded	absent	99.34	0.73	excluded	absent	43.11	-0.22	excluded	absent	36.29	-0.33
1555778_a_a	included	present	106.32	-0.4	included	present	855.2	-0.18	included	present	2187.12	0.21	included	present	805.54	-0.2	included	present	8377	2.02	included	present	8781.14	2.13	included	present	302.38	-0.34	included	present	788.29	-0.2
1555779_a_a	excluded	absent	113	-0.02	excluded	absent	50.16	-0.32	excluded	absent	4.23	-0.55	excluded	absent	63.29	-0.26	excluded	absent	132.56	0.07	excluded	absent	177.61	0.29	excluded	absent	72.17	-0.22	excluded	absent	46.62	-0.34
1555780_a_a	included	present	1279.18	0.84	included	present	1267.27	0.8	included	present	1008.68	0.04	included	present	945.37	-0.15	included	present	2227.11	3.64	included	present	1168.94	0.51	included	present	1744.78	2.21	included	present	2099.74	3.26
1555781_at	included	present	148.07	1.91	excluded	absent	148.42	1.92	excluded	absent	64.86	0.02	excluded	absent	10.41	-1.21	excluded	absent	89.93	0.59	excluded	absent	115.5	1.17	included	present	194.66	2.96	included	present	299.8	5.34
1555783_x_a	excluded	absent	152.21	0.84	excluded	absent	98.46	-0.01	excluded	absent	86.82	-0.2	excluded	absent	8.48	-1.45	excluded	absent	138.07	0.62	included	present	270.36	2.73	included	present	200.41	1.61	included	present	304.76	3.28
1555784_s_a	excluded	absent	59.84	0	excluded	absent	28.27	-0.58	excluded	absent	55	-0.09	excluded	absent	10.48	-0.9	excluded	absent	75.98	0.3	included	present	229.7	3.11	excluded	absent	153.92	1.72	included	present	406.11	6.34
1555785_a_a	included	present	370.27	-0.26	included	present	151.78	-1.35	included	present	304.75	-0.59	included	present	457.15	0.17	included	present	402.37	-0.1	included	present	841.47	2.09	included	present	578.4	0.78	included	present	833.49	2.05
1555786_s_a	excluded	absent	63.71	-0.29	included	present	80.13	-0.2	excluded	absent	58.26	-0.32	excluded	absent	5.28	-0.6	excluded	absent	280.26	0.86	included	present	154.09	0.19	excluded	absent	18	-0.53	excluded	absent	4.73	-0.6
1555787_at	excluded	absent	60.45	0.68	excluded	absent	54.72	0.52	excluded	absent	2.09	-0.87	excluded	absent	69.13	0.91	excluded	absent	49.44	0.38	excluded	absent	30.99	-0.11	excluded	absent	31.88	-0.08	excluded	absent	39.76	0.13
1555788_a_a	excluded	absent	12.46	-0.62	excluded	absent	38.9	0.08	excluded	absent	50.37	0.38	excluded	absent	2.18	-0.89	excluded	absent	208.99	4.57	excluded	absent	108.46	1.92	excluded	absent	63.58	0.73	excluded	absent	67.58	0.84

1556232_at	excluded	absent	49.63	-0.24	excluded	absent	13.33	-1.19	excluded	absent	57.69	[illegible]
1556233_s_a	excluded	absent	2.2	-1.04	included	present	120.62	0.64	excluded	absent	33.08	[illegible]
1556235_at	included	present	305.96	-0.65	included	present	1377.95	1.91	included	absent	72.06	[illegible]
1556236_at	excluded	absent	111.78	0.45	included	present	208.76	2.18	included	present	203.4	[illegible]
1556238_at	excluded	absent	10.78	-0.48	excluded	absent	1.96	-0.81	excluded	absent	40.44	[illegible]
1556239_a_a	excluded	absent	68.54	-0.28	excluded	absent	31.42	-1.26	excluded	absent	57.57	[illegible]
1556240_at	included	present	139.44	0.34	excluded	absent	11.53	-1.68	excluded	absent	60.87	[illegible]
1556242_a_a	included	present	301.42	0.03	excluded	absent	437.19	0.95	included	absent	79.58	[illegible]
1556244_s_a	included	present	70.32	-0.61	included	present	264.89	2.74	included	present	76.49	[illegible]
1556247_a_a	excluded	absent	3.81	-0.24	excluded	absent	101.62	0.12	excluded	absent	3.36	[illegible]
1556248_at	excluded	absent	13.24	-0.86	excluded	absent	14.02	-0.84	excluded	absent	71.5	[illegible]
1556249_a_a	excluded	absent	63.68	-0.62	included	present	81.6	-0.32	excluded	absent	11.07	[illegible]
1556250_at	excluded	absent	80.03	0.38	excluded	absent	79.32	0.36	excluded	absent	29.62	[illegible]
1556253_s_a	included	present	52.24	-0.67	included	present	256.18	2.37	excluded	absent	3.54	[illegible]
1556255_a_a	excluded	absent	40.13	-0.11	excluded	absent	7.41	-0.9	excluded	absent	3.63	[illegible]
1556256_a_a	excluded	absent	15.56	-0.39	excluded	absent	3.48	-0.56	excluded	absent	46.71	[illegible]
1556257_at	included	absent	406.76	-0.67	included	present	338.35	-0.82	included	present	951.37	[illegible]
1556259_at	excluded	absent	9.19	-1.33	excluded	absent	78.04	0.54	excluded	absent	37.38	[illegible]
1556261_a_a	included	present	275.07	0.75	included	present	843.63	3.92	included	present	135.27	[illegible]
1556262_at	excluded	absent	32.69	-0.56	excluded	absent	44.86	-0.54	excluded	absent	5.81	[illegible]
1556263_s_a	excluded	absent	21.52	-0.44	excluded	absent	33.36	-0.41	excluded	absent	3.42	[illegible]
1556265_at	excluded	absent	40.54	0.33	excluded	absent	46.27	0.52	excluded	absent	3.84	[illegible]
1556266_a_a	excluded	absent	51.73	-0.28	excluded	absent	16.43	-1.04	excluded	absent	3.39	[illegible]
1556267_at	excluded	absent	52.1	-0.41	excluded	absent	50.71	-0.41	excluded	absent	86.95	[illegible]
1556269_at	included	present	94.32	0.22	included	present	601.86	7.26	included	present	538.99	[illegible]
1556271_at	excluded	absent	1.2	-1	excluded	absent	6.4	-0.85	excluded	absent	9.78	[illegible]
1556272_a_a	included	present	87.61	-0.11	included	present	152.61	0.89	included	present	141.4	[illegible]
1556274_at	excluded	absent	2.83	-0.7	excluded	absent	12.54	-0.42	excluded	absent	45.27	[illegible]
1556277_a_a	included	present	1244.42	-0.29	included	present	304.85	-1.48	included	present	2011.93	[illegible]
1556279_at	included	present	103.06	-0.32	included	present	113.01	-0.11	included	present	187.33	[illegible]
1556281_at	excluded	absent	8.6	-0.97	excluded	absent	5.37	-1.05	excluded	absent	6.93	[illegible]
1556282_at	included	present	712.45	-0.08	included	absent	90.25	-2.34	included	present	232.42	[illegible]
1556283_s_a	included	present	1058.68	-0.19	included	present	638.52	-1.19	included	present	466.91	[illegible]
1556284_at	excluded	absent	75.56	0.53	excluded	absent	1.21	-1.33	excluded	absent	74.73	[illegible]
1556285_s_a	included	present	293.81	-1.53	included	present	189.07	-1.91	included	present	281.97	[illegible]
1556287_a_a	included	present	137.95	-0.03	excluded	absent	34.49	-1.04	included	present	171.57	[illegible]
1556288_at	excluded	absent	2.69	-0.74	excluded	absent	5.96	-0.68	excluded	absent	2.83	[illegible]
1556289_at	excluded	absent	5.17	-0.98	excluded	absent	69.28	0.5	excluded	absent	39.28	[illegible]
1556291_at	excluded	absent	12.64	-0.59	excluded	absent	15.5	-0.52	excluded	absent	23.75	[illegible]
1556292_s_a	excluded	absent	3.86	-0.66	excluded	absent	2.25	-0.7	excluded	absent	2.72	[illegible]
1556294_at	excluded	absent	48.46	-0.71	excluded	absent	60.5	-0.66	excluded	absent	42.7	[illegible]
1556296_at	excluded	absent	41.34	-0.27	excluded	absent	46.82	-0.17	excluded	absent	2.58	[illegible]
[illegible]												

1556784_at	excluded	absent	72.47	1.1	excluded	absent	34.27	-0.05	excluded	absent	8.89	-0.81	included	present	94.99	1.78
1556786_at	excluded	absent	3.05	-0.69	excluded	absent	52.49	0.75	excluded	absent	1.51	-0.73	excluded	absent	1.45	-0.73
1556787_s_a	excluded	absent	49.38	0.13	excluded	absent	26.35	-0.62	excluded	absent	5.76	-1.3	excluded	absent	41.81	-0.12
1556789_a_a	excluded	absent	115.22	0.23	included	present	172.55	0.99	included	present	142.97	0.6	included	present	277.87	2.4
1556793_a_a	excluded	absent	81.84	-0.28	excluded	absent	107.57	-0.28	excluded	absent	52.36	-0.29	included	present	65.95	-0.29
1556794_at	excluded	absent	54.24	0.13	excluded	absent	41.26	-0.14	excluded	absent	58.98	0.23	excluded	absent	38.42	-0.2
1556796_at	excluded	absent	61.99	0.34	excluded	absent	2.99	-0.66	excluded	absent	79.26	0.63	excluded	absent	62.84	0.35
1556797_at	included	present	278.5	-0.08	included	present	453.64	0.36	excluded	absent	124.17	-0.47	included	present	621.05	0.78
1556798_a_a	excluded	absent	72.61	-0.19	included	present	175.3	0.41	included	present	91.58	-0.08	included	present	173.83	0.41
1556800_a_a	excluded	absent	45.56	-0.47	included	present	404.05	3.91	excluded	absent	26.28	-0.71	included	present	377.32	3.58
1556801_at	excluded	absent	24.66	-0.36	excluded	absent	16.38	-0.56	excluded	absent	8.23	-0.75	excluded	absent	9.29	-0.73
1556803_at	excluded	absent	74.65	-0.64	included	present	128.23	0.64	included	present	71.43	-0.71	included	present	142.24	0.97
1556804_s_a	included	absent	60.83	-0.79	included	present	340.52	0.96	included	present	231.93	0.28	included	present	398.54	1.32
1556805_at	excluded	absent	64.54	0.77	excluded	absent	23	-0.19	excluded	absent	53.04	0.51	excluded	absent	44.83	0.32
1556806_at	included	present	278.96	0.24	excluded	absent	217.25	-0.1	included	present	115.41	-0.64	excluded	absent	118.76	-0.63
1556808_at	excluded	absent	79.9	0.64	excluded	absent	28.22	-0.93	excluded	absent	104.06	1.37	excluded	absent	33.59	-0.76
1556809_a_a	excluded	absent	121.7	0.22	excluded	absent	92.48	-0.16	excluded	absent	99.43	-0.07	included	present	139.36	0.45
1556810_a_a	excluded	absent	102.24	-0.18	excluded	absent	6.59	-1.78	included	present	145.64	0.55	included	present	148.69	0.6
1556812_a_a	included	present	171.26	-0.24	included	absent	87.04	-0.45	included	present	187.92	-0.2	included	present	208.13	-0.16
1556813_at	included	present	233.26	-0.16	included	absent	69.43	-1.6	included	present	178.96	-0.64	included	absent	80.28	-1.51
1556814_a_a	excluded	absent	42.54	0.42	excluded	absent	2.38	-0.96	excluded	absent	2.21	-0.97	excluded	absent	40.07	0.33
1556815_at	excluded	absent	80.19	0.45	excluded	absent	72.03	0.26	included	present	138.16	1.78	excluded	absent	63.3	0.06
1556817_a_a	included	present	146.39	0.76	excluded	absent	1.23	-1.38	excluded	absent	83.02	-0.17	excluded	absent	34.83	-0.88
1556818_at	included	present	134.24	-0.26	included	absent	40.51	-1.6	included	absent	86.15	-0.95	included	absent	3.91	-2.12
1556820_a_a	included	present	322.96	1.45	included	present	136.29	-0.59	included	present	534.16	3.76	included	present	222.23	0.35
1556821_x_a	included	present	168.67	2.23	excluded	absent	111.09	0.93	included	present	257.39	4.25	excluded	absent	46.82	-0.53
1556822_s_a	excluded	absent	2.06	-0.76	excluded	absent	44.33	0.48	excluded	absent	10.18	-0.52	excluded	absent	85.84	1.7
1556823_s_a	excluded	absent	73.96	2.01	excluded	absent	32.3	0.38	excluded	absent	54.72	1.26	excluded	absent	1.7	-0.81
1556824_at	excluded	absent	3.37	-0.7	excluded	absent	41.79	0.83	excluded	absent	43.11	0.88	excluded	absent	36.82	0.63
1556826_s_a	included	present	121.65	1.65	excluded	absent	114.78	1.49	excluded	absent	38.58	-0.33	excluded	absent	58.75	0.15
1556827_at	excluded	absent	129.16	0.54	excluded	absent	61.25	-0.73	excluded	absent	81.06	-0.36	excluded	absent	50.48	-0.93
1556828_at	excluded	absent	6.61	-0.92	excluded	absent	9.73	-0.83	excluded	absent	2	-1.05	excluded	absent	71.6	0.96
1556829_at	excluded	absent	7.51	-0.55	excluded	absent	2.34	-0.61	excluded	absent	7.95	-0.55	excluded	absent	6.4	-0.57
1556831_at	excluded	absent	59.47	-0.21	excluded	absent	73.46	0.12	excluded	absent	27.43	-0.96	excluded	absent	10.87	-1.35
1556832_at	excluded	absent	54.7	-0.58	excluded	absent	1.73	-1.02	excluded	absent	1.95	-1.02	excluded	absent	36.59	-0.73
1556834_at	included	absent	0.56	-0.51	included	absent	46.45	-0.48	included	present	532.01	-0.13	included	present	3158.97	1.72
1556835_s_a	excluded	absent	10.76	-1	excluded	absent	83.86	-0.28	excluded	absent	114.91	0.02	included	present	354.75	2.39
1556836_at	excluded	absent	8.61	-0.74	excluded	absent	42.8	0.07	excluded	absent	28.79	-0.26	excluded	absent	59.48	0.47
1556837_a_a	excluded	absent	4.82	-0.45	excluded	absent	5.92	-0.4	excluded	absent	11.86	-0.1	excluded	absent	3.14	-0.54
1556839_s_a	included	present	155	-0.34	included	absent	20.05	-0.51	included	present	122.18	-0.38	included	present	1358.91	1.17
1556841_a_a	included	present	261.26	1.14	included	absent	254.35	1.06	included	present	506.71	3.91	included	present	175.49	0.17
1556842_at	excluded	absent	76.09	0.4	excluded	absent	30.59	-0.19	excluded	absent	50.4	0.07	included	present	488.32	5.74

[illegible]

1557350_at	excluded	absent	13.22	-0.93	excluded	absent	41.06	-0.45	excluded	absent	136.68	1.2	excluded	absent	140.25	1.26	excluded	absent	75.23	0.14	excluded	absent	4.06	-1.09	excluded	absent	54.43	-0.22	excluded	absent	59.22	-0.14	excluded	absent	95.47	0.49	included	present	102.61	0.61	excluded	absent	91.79	0.43	excluded	absent	32.65	-0.59	excluded	absent	6.78	-1.04	excluded	absent	13.72	-0.92	excluded	absent	45	-0.38
1557352_at	included	present	179.92	0.91	included	present	139.6	0.27	included	present	192.53	1.11	excluded	absent	66.27	-0.9	included	present	231.07	1.72	included	present	160.28	0.59	included	present	142.51	0.31	included	present	51.13	-1.14	included	present	192.89	1.11	included	present	90.51	-0.51	included	present	209.89	1.38	excluded	absent	6.53	-1.84	included	present	130.34	0.12	included	present	173.7	0.81	included	present	198.88	1.21
1557353_at	included	present	231.75	0.41	included	absent	52.76	-1.73	included	absent	124.75	-0.87	included	present	307.05	1.31	included	absent	118.67	-0.94	included	present	195.75	-0.02	included	present	108.78	-1.06	included	present	70.67	-1.52	included	present	234.68	0.44	included	present	173.77	-0.28	included	absent	133.77	-0.76	included	present	148.28	-0.59	included	absent	58.03	-1.67	included	absent	114.75	-0.99	included	present	90.49	-1.28
1557354_at	included	present	85.27	0.11	excluded	absent	46.37	-0.71	excluded	absent	53.04	-0.57	included	present	71.33	-0.18	excluded	absent	132.2	1.1	excluded	absent	49.48	-0.64	included	present	111.34	0.66	excluded	absent	31.31	-1.03	included	present	84.79	0.1	included	present	122.42	0.89	included	present	137.31	1.2	excluded	absent	114.29	0.72	included	present	116.38	0.76	excluded	absent	52.87	-0.57	excluded	absent	31.63	-1.02
1557357_at	included	present	234.59	0.31	included	absent	138.39	-0.76	included	present	301.32	1.05	included	present	212.78	0.07	included	absent	11.22	-2.17	included	present	243.07	0.4	included	present	159.39	-0.53	included	present	217.51	0.12	included	present	170.79	-0.4	included	present	137.53	-0.77	included	present	207.47	0.01	included	present	150.38	-0.63	included	present	166.11	-0.45	included	present	269.94	0.7	included	present	109.06	-1.09
1557359_at	included	present	250.18	1.67	excluded	absent	5.83	-1.38	excluded	absent	3.73	-1.4	excluded	absent	37.54	-0.98	excluded	absent	88.33	-0.35	excluded	absent	78.96	-0.47	excluded	absent	10.03	-1.33	excluded	absent	34.71	-1.02	included	present	206.1	1.12	excluded	absent	47.72	-0.86	excluded	absent	99.58	-0.21	included	present	81.23	-0.44	included	present	212.04	1.2	excluded	absent	39.76	-0.95	excluded	absent	55.09	-0.76
1557360_at	included	absent	89.75	-1.17	included	present	140.61	-0.71	included	present	451.28	2.09	included	present	206.89	-0.12	included	present	169.44	-0.45	included	absent	111.42	-0.98	included	present	280.18	0.55	included	present	86.58	-1.2	included	present	125.48	-0.85	included	present	171.42	-0.44	included	present	369.23	1.35	included	absent	36.42	-1.65	included	present	101.16	-1.07	included	present	198.58	-0.19	included	present	95.16	-1.12
1557362_at	excluded	absent	56.95	-0.15	excluded	absent	26.82	-0.62	excluded	absent	50.25	-0.26	excluded	absent	109.73	0.66	excluded	absent	76.2	0.14	excluded	absent	2.7	-0.99	excluded	absent	7.19	-0.93	excluded	absent	5.34	-0.95	included	present	67.9	0.02	included	present	140.69	1.14	excluded	absent	102.77	0.56	excluded	absent	133.72	1.04	excluded	absent	90.13	0.36	excluded	absent	88.93	0.34	excluded	absent	66.68	0
1557363_a_a	excluded	absent	52.21	-0.11	excluded	absent	57.15	-0.03	excluded	absent	0.92	-1.01	excluded	absent	81.97	0.41	excluded	absent	76.62	0.31	excluded	absent	2.13	-0.99	included	present	117.9	1.04	excluded	absent	28.03	-0.54	excluded	absent	34.74	-0.42	included	present	76.17	0.31	included	present	83.26	0.43	excluded	absent	66.58	0.14	included	present	86.08	0.48	included	present	71.64	0.23	included	present	55.15	-0.06
1557366_at	included	present	282.33	-0.85	included	present	1337.11	0.22	included	present	1224.09	0.1	included	absent	87.58	-1.04	included	present	348	-0.78	included	present	653.86	-0.47	included	present	1556.88	0.44	included	present	2689.91	1.58	included	present	1003.31	-0.12	included	present	937.33	-0.19	included	present	2535.63	1.43	included	present	820.99	-0.3	included	present	1733.68	0.62	included	present	1739.08	0.62	included	present	549.35	-0.58
1557369_a_a	excluded	absent	118.16	1.65	excluded	absent	59.72	0.35	excluded	absent	40.3	-0.08	excluded	absent	42.69	-0.03	excluded	absent	133.14	1.99	excluded	absent	25.72	-0.4	excluded	absent	2.9	-0.91	excluded	absent	31.39	-0.28	excluded	absent	3.01	-0.91	excluded	absent	32.65	-0.25	excluded	absent	9.4	-0.77	excluded	absent	3.12	-0.91	excluded	absent	26.28	-0.39	excluded	absent	10.13	-0.75	excluded	absent	34.06	-0.22
1557370_s_a	included	present	2335.4	-0.02	included	present	2975.62	0.52	included	present	2687.86	0.28	included	present	1643.82	-0.61	included	present	3860.67	1.28	included	present	2544.62	0.15	included	present	3651.63	1.1	included	present	2639.8	0.24	included	present	3556.5	1.02	included	present	3461.56	0.94	included	present	1939.56	-0.36	included	present	2485.63	0.1	included	present	8985.52	5.64	included	present	2845.92	0.41	included	present	3596.49	1.05
1557371_a_a	excluded	absent	115.57	0.21	excluded	absent	20.25	-0.74	excluded	absent	75.69	-0.19	excluded	absent	2.22	-0.92	excluded	absent	27.99	-0.66	excluded	absent	270.85	1.77	excluded	absent	67.02	-0.27	excluded	absent	57.83	-0.36	excluded	absent	121.37	0.27	excluded	absent	60.61	-0.34	excluded	absent	176.45	0.82	excluded	absent	198.85	1.05	excluded	absent	118.25	0.24	included	present	92.81	-0.01	excluded	absent	3.64	-0.91
1557372_at	excluded	absent	38.62	-1.01	included	present	141.19	1.27	excluded	absent	106.14	0.49	excluded	absent	47.06	-0.83	excluded	absent	98.36	0.31	excluded	absent	60.26	-0.53	included	present	771.9	15.28	included	present	81.62	-0.06	included	present	161.46	1.72	excluded	absent	56.01	-0.63	included	present	317.32	5.18	included	present	146.24	1.38	included	present	166.21	1.82	included	present	74.52	-0.22	included	present	187.99	2.31
1557373_at	excluded	absent	1.12	-0.96	excluded	absent	3.09	-0.89	excluded	absent	13.01	-0.55	excluded	absent	11.21	-0.61	excluded	absent	69.52	1.42	excluded	absent	9.42	-0.67	excluded	absent	36.69	0.28	excluded	absent	2.6	-0.91	excluded	absent	3.62	-0.88	excluded	absent	3.74	-0.87	excluded	absent	31.45	0.09	excluded	absent	14.11	-0.51	excluded	absent	20.32	-0.29	excluded	absent	7.16	-0.75	excluded	absent	30.44	0.06
1557374_at	excluded	absent	7.96	-0.76	excluded	absent	43.38	-0.6	excluded	absent	40.04	-0.62	included	present	128.29	-0.24	excluded	absent	113.5	-0.3	included	present	179.7	-0.02	excluded	absent	40.41	-0.62	included	present	100.52	-0.36	included	present	65.84	-0.51	included	present	91.18	-0.4	excluded	absent	53.37	-0.56	excluded	absent	16.5	-0.72	excluded	absent	3.01	-0.78	included	present	106.14	-0.33	excluded	absent	1.83	-0.78
1557375_at	included	present	172.09	1.93	excluded	absent	41.6	-0.41	included	present	125.56	1.1	excluded	absent	36.32	-0.5	included	present	129.07	1.16	excluded	absent	75.74	0.21	excluded	absent	12.96	-0.92	included	present	186.67	2.19	included	present	263.74	3.57	excluded	absent	76.91	0.23	included	present	374.43	5.56	excluded	absent	108.23	0.79	included	present	64.38	0	included	present	71.48	0.13	included	present	49.02	-0.27
1557380_at	included	present	282.85	0.44	included	present	100.78	-0.71	included	absent	64.06	-0.94	included	present	107.48	-0.66	included	absent	58.96	-0.97	included	present	419.99	1.3	included	present	133.43	-0.5	included	present	108.08	-0.66	included	present	291.11	0.49	included	present	89.91	-0.77	included	present	156.26	-0.36	included	absent	74.07	-0.87	included	present	204.3	-0.06	included	present	149.38	-0.4	included	present	91.19	-0.77
1557382_x_a	included	present	298.75	-0.23	included	absent	101.92	-1.39	included	present	255.73	-0.48	included	present	266.36	-0.42	included	present	351.21	0.08	included	present	548.63	1.23	included	present	292.99	-0.27	included	present	268.51	-0.41	included	present	491.42	0.9	included	present	252.2	-0.5	included	present	324.72	-0.08	included	present	88.22	-1.47	included	present	107.58	-1.35	included	present	161.78	-1.04	included	present	157.29	-1.06
1557383_a_a	included	present	664.06	0.28	included	present	159.05	-0.55	included	absent	211.07	-0.46	included	absent	60.36	-0.71	included	present	435.29	-0.1	included	present	326.46	-0.27	included	present	793.86	0.49	included	present	310.74	-0.3	included	present	502.91	0.01	included	present	683.21	0.31	included	present	427.46	-0.11	included	present	361.5	-0.22	included	present	617.47	0.2	included	present	437.3	-0.09	included	present	999.6	0.82
1557384_at	included	present	214.71	0.06	included	present	174.71	-0.28	included	present	344.97	1.17	included	present	276.18	0.58	included	present	349.81	1.21	included	present	220.09	0.11	included	present	307.05	0.85	included	present	162.89	-0.38	included	present	252.83	0.38	included	present	357.78	1.28	included	present	325.62	1	included	absent	87.16	-1.03	included	present	163.35	-0.38	included	present	123.6	-0.72	included	present	229.47	0.19
1557385_at	included	present	171.63	-0.37	included	present	841.07	2.84	included	present	499.83	1.21	included	present	1051.97	3.85	included	present	349.21	0.49	included	absent	96.54	-0.73	included	present	680.8	2.07	included	present	458.38	1.01	included	present	326.52	0.38	included	present	553.7	1.47	included	present	671.75	2.03	included	present	416.17	0.81	included	present	699.36	2.16	included	present	1163.36	4.39	included	present	300.17	0.25
1557386_at	excluded	absent	42.64	-0.44	excluded	absent	19.84	-0.81	excluded	absent	88.4	0.3	excluded	absent	69.44	-0.01	excluded	absent	75.1	0.09	included	present	150.82	1.31	excluded	absent	7.46	-1.01	excluded	absent	46.16	-0.38	excluded	absent	10.08	-0.96	excluded	absent	55.1	-0.24	excluded	absent	22.44	-0.76	excluded	absent	87.91	0.29	excluded	absent	103.72	0.55	excluded	absent	64.68	-0.08	excluded	absent	30.81	-0.63
1557388_at	included	present	303.76	-0.16	included	absent	145.81	-1.18	included	present	204.8	-0.8	included	present	545.14	1.4	included	present	617.1	1.87	included	absent	102.34	-1.46	included	present	243.47	-0.55	included	present	236.85	-0.59	included	present	362.47	0.22	included	present	165.88	-1.05	included	present	761.11	2.8	included	present	188.21	-0.91	included	present	283	-0.29	included	present	335.13	0.04	included	present	192.2	-0.88
1557389_at	included	present	237.51	2.17	excluded	absent	2.32	-1.04	excluded	absent	88.29	0.14	excluded	absent	62.32	-0.22	excluded	absent	69.51	-0.12	excluded	absent	99.03	0.28	excluded	absent	58.59	-0.27	excluded	absent	6.93	-0.97	excluded	absent	88.04	0.13	excluded	absent	48.28	-0.41	excluded	absent	108.28	0.41	excluded	absent	60.3	-0.25	excluded	absent	6.63	-0.98	included	present	71.03	-0.1	included	present	90.71	0.17
1557394_at	excluded	absent	153.18	1.89	excluded	absent	33.12	-0.44	excluded	absent	11.91	-0.85	included	present	94.97	0.76	excluded	absent	6.58	-0.96	excluded	absent	124.87	1.34	excluded	absent	108.6	1.03	excluded	absent	36.61	-0.37	included	present	168.54	2.19	included	present	205.89	2.92	included	present	265.62	4.08	included	present	253.85	3.85	excluded	absent	80.94	0.49	excluded	absent	5.59	-0.98	excluded	absent	50.71	-0.1
1557395_at	excluded	absent	39.78	-0.31	excluded	absent	61.58	-0.15	excluded	absent	45.65	-0.26	excluded	absent	1.82	-0.58	excluded	absent	69.88	-0.09	excluded	absent	48.43	-0.24	included	present	149.07	0.49	excluded	absent	2.13	-0.58	excluded	absent	27.23	-0.4	excluded	absent	52.16	-0.22	excluded	absent	5.43	-0.55	excluded	absent	22.79	-0.43	excluded	absent	4.98	-0.56	excluded	absent	33.27	-0.35	excluded	absent	25.57	-0.41
1557398_at	included	present	191.94	1.8	excluded	absent	71.68	-0.51	included	present	155.95	1.11	included	present	152.37	1.04	included	present	148.68	0.97	excluded	absent	47.22	-0.98	included	present	74.74	-0.45	included	present	83.54	-0.28	included	present	116.39	0.35	included	present	135.55	0.72	included	present	77.69	-0.39	excluded	absent	45.73	-1.01	included	present	55.55	-0.82	excluded	absent	78.25	-0.38	included	present	46.59	-0.99
1557399_at	included	present	134.77	0.13	excluded	absent	90.97	-0.54	excluded	absent	87.76	-0.59	included	present	128.39	0.03	included	present	241.58	1.77	included	present	143.16	0.26	excluded	absent	61.56	-0.99	excluded	absent	26.78	-1.53	included	present	59.39	-1.03	included	present	90.73	-0.55	included	present	92.22	-0.52	excluded	absent	71.06	-0.85	excluded	absent	75.91	-0.77	excluded	absent	60.29	-1.01	excluded	absent	33.3	-1.43
1557400_at	included	present	321.13	1.41	included	absent	92.22	-0.79	included	absent	92.6	-0.79	included	present	146.42	-0.27	included	absent	110.46	-0.62	excluded	absent	214.38	0.38	included	present	343.4	1.62	included	present	219.52	0.43	included	present	188.23	0.13	included	present	104.94	-0.67	included	present	248.17	0.71	included	absent	106.26	-0.66	included	absent	104.56	-0.68	included	present	101.02	-0.71	included	present	225.92	0.49
1557402_at	excluded	absent	43.75	0.32	included	present	117.86	2.51	excluded	absent	47.89	0.45	excluded	absent	88.15	1.63	excluded	absent	105.45	2.14	excluded	absent	11.85	-0.62	included	present	193.77	4.75	excluded	absent	26.2	-0.19	excluded	absent	55.1	0.66	excluded	absent	22.87	-0.29	excluded	absent	14.72	-0.53	excluded	absent	36.46	0.11	included	present	89.62	1.68	excluded	absent	68.3	1.05	included	present	74.23	1.22
1557403_s_a	included	present	136.22	-0.54	included	absent	42.04	-1.31	included	absent	77.15	-1.02	included	present	96.37	-0.86	included	absent	75.15	-1.04	included	present	179.79	-0.19	included	absent	17.34	-1.51	included	absent	32.51	-1.38	included	present	81.4	-0.99	included	present	104.13	-0.8	included	present	119.31	-0.68	included	absent	32.2	-1.39	included	absent	32.11	-1.39	included	absent	20.78	-1.48	included	absent	9.08	-1.57
1557404_at	excluded	absent	27.29	-0.13	excluded	absent	6.01	-0.81	excluded	absent	2.69	-0.91	excluded	absent	64.8	1.05	excluded	absent	2.52	-0.92	excluded	absent	29.55	-0.06	excluded	absent	53.98	0.71	excluded	absent	39.21	0.24	excluded	absent	9.98	-0.68	excluded	absent	2.18	-0.93	excluded	absent	33.97	0.08	excluded	absent	48.91	0.55	excluded	absent	3.51	-0.89	excluded	absent	5.04	-0.84	excluded	absent	2.23	-0.93
1557405_at	excluded	absent	2.54	-0.56	excluded	absent	12.21	-0.11	excluded	absent	4.6	-0.47	excluded	absent	15.69	0.05	excluded	absent	4.82	-0.46	excluded	absent	1.89	-0.59	excluded	absent	4.31	-0.48	excluded	absent	3.19	-0.53	excluded	absent	1.83	-0.59	excluded	absent	29.1	0.67	excluded	absent	7.35	-0.34	excluded	absent	3.49	-0.52	excluded	absent	5.61	-0.42	excluded	absent	1.41	-0.61	excluded	absent	0.93	-0.64

1557867_s_a	included	absent	184.88	-0.32	included	present	626.27	1.96	included	present	107.81	-0.71	included	absent	109.07	-0.71	excluded	absent	265.65
1557869_at	excluded	absent	24.21	-0.38	excluded	absent	16.8	-0.56	excluded	absent	8.31	-0.77	excluded	absent	69.23	0.75	excluded	absent	71.02
1557871_at	excluded	absent	3.15	-0.57	excluded	absent	1.37	-0.63	excluded	absent	2.25	-0.6	excluded	absent	10.03	-0.36	excluded	absent	7.13
1557873_at	excluded	absent	112.5	0.1	excluded	absent	88.23	-0.27	excluded	absent	6.77	-1.51	excluded	absent	37.56	-1.04	excluded	absent	126.62
1557874_at	excluded	absent	41.99	-0.21	excluded	absent	39.18	-0.27	excluded	absent	57.5	0.13	excluded	absent	4.62	-1.03	excluded	absent	3.02
1557875_at	excluded	absent	66.22	-0.11	excluded	absent	6.51	-1.1	excluded	absent	74.19	0.03	included	present	180.81	1.79	excluded	absent	4.04
1557876_at	excluded	absent	4.03	-0.63	excluded	absent	4.41	-0.62	excluded	absent	8.25	-0.5	excluded	absent	0.93	-0.73	excluded	absent	3.91
1557877_s_a	excluded	absent	2.1	-0.66	excluded	absent	2.96	-0.63	excluded	absent	4.11	-0.59	excluded	absent	1.39	-0.69	excluded	absent	73.81
1557878_at	excluded	absent	70.67	1.1	excluded	absent	48.12	0.41	excluded	absent	8.37	-0.83	excluded	absent	63.32	0.88	excluded	absent	141.74
1557879_at	excluded	absent	56.69	-0.49	excluded	absent	100.73	0.19	excluded	absent	2.34	-1.31	excluded	absent	70.05	-0.28	excluded	absent	78.25
1557880_at	excluded	absent	2.02	-0.61	excluded	absent	3.75	-0.53	excluded	absent	67.3	2.61	excluded	absent	3.02	-0.56	excluded	absent	4.55
1557881_at	excluded	absent	3.5	-0.66	excluded	absent	4.4	-0.63	excluded	absent	36.16	0.5	excluded	absent	14.14	-0.29	excluded	absent	2.94
1557882_at	excluded	absent	48.94	1.6	excluded	absent	75.26	2.84	excluded	absent	35.49	0.96	excluded	absent	23.49	0.4	excluded	absent	37.68
1557883_a_a	excluded	absent	1.73	-0.69	excluded	absent	39.03	0.72	excluded	absent	29.15	0.34	excluded	absent	2.33	-0.67	excluded	absent	62.28
1557885_at	excluded	absent	4.92	-1.26	excluded	absent	37.87	-0.7	included	present	105.71	0.46	included	present	131.27	0.9	excluded	absent	59.83
1557886_at	excluded	absent	54.96	-0.54	excluded	absent	21.92	-1.22	excluded	absent	3.56	-1.6	included	present	391.76	6.37	excluded	absent	132.99
1557888_at	excluded	absent	54.64	0.34	excluded	absent	86.55	1.26	excluded	absent	4	-1.13	excluded	absent	47.93	0.14	excluded	absent	18.09
1557889_at	included	present	593.33	1.65	included	present	200.12	-0.33	included	present	341.94	0.39	included	absent	95.67	-0.86	included	present	376.74
1557890_at	excluded	absent	56.81	-0.44	excluded	absent	3.03	-1.22	excluded	absent	35.88	-0.74	excluded	absent	54.59	-0.48	excluded	absent	96.49
1557891_s_a	excluded	absent	61.74	-0.15	excluded	absent	32.46	-0.35	excluded	absent	15.76	-0.46	excluded	absent	2.11	-0.55	excluded	absent	8.23
1557892_at	excluded	absent	13.85	0.07	excluded	absent	7	-0.33	excluded	absent	2.75	-0.58	excluded	absent	38.68	1.53	excluded	absent	5.26
1557893_a_a	excluded	absent	36.08	0.98	excluded	absent	6.97	-0.47	excluded	absent	4.3	-0.6	excluded	absent	3.49	-0.64	excluded	absent	103.84
1557895_at	included	present	158.15	-0.57	included	absent	93.5	-0.97	included	present	499.17	1.55	included	present	226.54	-0.14	included	present	347.86
1557896_at	excluded	absent	8.9	-1.02	excluded	absent	25.95	-0.64	excluded	absent	46.29	-0.18	excluded	absent	1.56	-1.19	excluded	absent	70.41
1557897_at	excluded	absent	39.85	0.03	excluded	absent	101.95	1.8	excluded	absent	3.06	-1.02	excluded	absent	3.85	-1	excluded	absent	32.73
1557898_at	excluded	absent	90.52	1.89	excluded	absent	29.35	-0.02	excluded	absent	2.35	-0.87	excluded	absent	1.98	-0.88	excluded	absent	7.2
1557900_at	excluded	absent	2.13	-0.84	excluded	absent	3.39	-0.79	excluded	absent	2.86	-0.81	excluded	absent	4.84	-0.74	excluded	absent	23.43
1557905_s_a	included	present	14521.14	1.53	included	present	3625.06	-0.38	included	present	7995.54	0.38	included	present	496.78	-0.93	included	present	3629.67
1557906_at	excluded	absent	118.67	0.05	excluded	absent	6.51	-0.24	excluded	absent	5.04	-0.24	excluded	absent	35.16	-0.16	excluded	absent	6.67
1557907_x_a	excluded	absent	69.95	-0.14	excluded	absent	9.6	-0.22	excluded	absent	7.6	-0.22	excluded	absent	71.1	-0.13	excluded	absent	76.07
1557908_at	excluded	absent	66.09	0.44	excluded	absent	49.96	0.08	excluded	absent	80.31	0.76	excluded	absent	65.52	0.43	excluded	absent	7.31
1557910_at	included	present	25728.21	3.51	included	present	16547.81	1.14	included	present	33756.5	5.58	included	present	19208.22	1.83	included	present	21558.28
1557914_s_a	excluded	absent	1.76	-1.12	excluded	absent	5.87	-1	excluded	absent	40.26	0.04	excluded	absent	4.74	-1.03	excluded	absent	66.9
1557915_s_a	included	present	1440.23	-0.21	included	present	972.37	-0.71	included	present	1897.08	0.28	included	present	772.13	-0.92	included	present	1464.82
1557918_s_a	included	present	526.97	-0.52	included	absent	174.22	-1.16	included	present	1299.89	0.89	included	absent	166.7	-1.18	included	absent	137.73
1557919_a_a	excluded	absent	78.37	0.03	excluded	absent	241.34	3.37	excluded	absent	97.99	0.43	included	present	158.57	1.67	excluded	absent	76.52
1557921_s_a	excluded	absent	2.37	-0.71	excluded	absent	3.76	-0.69	excluded	absent	65.75	0.41	excluded	absent	1.64	-0.73	excluded	absent	4.8
1557924_s_a	excluded	absent	2.45	-0.33	excluded	absent	50.02	0.03	excluded	absent	5.92	-0.3	excluded	absent	4.4	-0.31	excluded	absent	3.73
1557938_s_a	included	present	2248.28	0.68	included	present	163.38	-0.7	included	present	185.64	-0.68	included	present	161.6	-0.7	included	present	341.69
1557943_at	included	present	1124.14	-0.25	included	present	370.56	-0.53	included	present	353.91	-0.53	included	present	274.33	-0.56	included	present	792.31
1557944_s_a	included	present	1123.77	0.56	included	present	371.05	-0.81	included	present	736.1	-0.14	included	present	1040.91	0.41	included	present	608.34
1557945_at	included	present	224.98	-1.62	included	present	226.11	-1.61	included	present	483.12	-0.13	included	present	918.8	2.37	included	present	274.26
1557948_at	excluded	absent	50.16	-0.77	excluded	absent	168.18	1.68	excluded	absent	56.93	-0.63	excluded	absent	117.83	0.63	excluded	absent	36.08
1557950_at	included	present	1653.63	3.41	included	present	1040.27	0.76	included	present	1405.68	2.34	included	present	1376.78	2.21	included	present	1461.17
1557953_at	included	present	772.99	0.93	included	present	851.51	1.24	included	present	1517.08	3.83	included	present	1636.17	4.29	included	present	2023.05
1557954_at	included	present	283.52	0.08	included	present	181.08	-0.9	included	present	352.25	0.73	included	present	414.81	1.32	included	present	257.84
1557961_s_a	included	present	585.56	-0.66	included	present	113.51	-0.81	included	present	137.63	-0.8	included	absent	53.26	-0.83	included	present	213.41
1557963_at	included	present	118.42	-1.16	included	present	107.38	-1.25	included	present	111.55	-1.22	included	present	175.19	-0.65	included	present	230.38
1557964_at	excluded	absent	113.23	0.44	excluded	absent	92.89	0.11	excluded	absent	80.01	-0.1	excluded	absent	49.14	-0.6	excluded	absent	99.9
1557965_at	included	present	1952.89	0.21	included	present	4115.96	3.35	included	present	1825.56	0.03	included	present	3099.3	1.87	included	present	1384.7
1557966_x_a	included	present	1386.68	-0.29	included	present	3257.09	3.3	included	present	1328.12	-0.4	included	present	2203.01	1.28	included	present	1219.86
1557970_s_a	included	present	174.63	-0.53	included	absent	7.4	-1.46	included	present	258.58	-0.07	included	absent	54.09	-1.2	included	absent	141.41
1557981_at	excluded	absent	47.72	-0.27	excluded	absent	83.88	0.45	excluded	absent	16.24	-0.9	excluded	absent	65.21	0.08	excluded	absent	153.76
1557984_s_a	included	present	1176.77	0.65	included	present	1211.24	0.76	included	present	1429.99	1.48	included	present	1362.65	1.26	included	present	2860.34
1557985_s_a	included	present	165.22	-0.48	included	present	226.23	-0.11	included	present	1236.78	6.05	included	present	452.75	1.27	included	present	612.2
1557986_s_a	included	present	599.32	1.54	included	present	371.44	0.1	included	present	739.82	2.43	included	present	503.28	0.93	included	present	941.51
1557987_at	included	present	716.46	1.81	included	present	214.87	-0.64	included	present	593.07	1.21	included	present	180.27	-0.81	included	present	489.95
1557991_at	included	present	111.26	0.95	included	present	99.8	0.67	included	present	200.09	3.12	excluded	absent	36.46	-0.87	excluded	absent	108.77
1557993_at	excluded	absent	5.7	-0.7	excluded	absent	5.2	-0.71	excluded	absent	3.83	-0.74	excluded	absent	43.75	0.31	excluded	absent	2.35
1557994_at	included	present	457.01	-0.16	included	absent	44.56	-0.18	included	absent	16.34	-0.18	included	present	274.46	-0.17	included	present	715.5
1557996_at	included	present	1878.75	0.72	included	present	5635.44	6.99	included	present	2557.92	1.85	included	present	1443.73	-0.01	included	present	2339.05
1557998_at	included	present	265.61	1.31	excluded	absent	7.26	-0.91	excluded	absent	72.44	-0.35	excluded	absent	61.66	-0.44	excluded	absent	17.9
1558000_at	included	present	600.04	-0.65	included	absent	92.59	-0.95	included	absent	90.87	-0.95	included	present	210.28	-0.88	included	present	725.37
1558001_s_a	included	present	563.76	-0.56	included	absent	39	-1.08	included	absent	132.56	-0.98	included	present	303.37	-0.81	included	present	380.94
1558002_at	included	present	421.18	-0.09	included	present	705.22	1.27	included	present	562.04	0.59	included	present	758.39	1.53	included	present	850.81
1558007_s_a	included	present	184.1	0.89	excluded	absent	165.66	0.52	included	present	222.75	1.67	excluded	absent	45.3	-1.89	excluded	absent	146.89

[illegible]

1558613_at	included	present	474.36	5.3	excluded	absent	38.08	-0.61	excluded	absent	2.99	-1.08	excluded	absent	4.29	-1.06	excluded	absent	13.25	-0.94	included	present	212.61	1.76	excluded	absent	35.24	-0.64	excluded	absent	11.01	-0.97	included	present	226.57	1.95	excluded	absent	41.14	-0.56	excluded	absent	51.4	-0.42	excluded	absent	1.9	-1.09	excluded	absent	160.71	1.05	excluded	absent	42.87	-0.54	excluded	absent	3.88	-1.07
1558619_at	excluded	absent	4.79	-0.91	excluded	absent	8.26	-0.81	excluded	absent	4.33	-0.92	excluded	absent	20.81	-0.46	excluded	absent	14.68	-0.63	excluded	absent	15.75	-0.6	excluded	absent	66.16	0.82	excluded	absent	9.25	-0.78	excluded	absent	15.26	-0.61	excluded	absent	8.85	-0.79	excluded	absent	20.25	-0.47	excluded	absent	29.88	-0.2	excluded	absent	22.41	-0.41	excluded	absent	56.46	0.55	excluded	absent	14.87	-0.62
1558620_at	included	present	949.44	0.16	included	present	374.27	-1.68	included	present	873.69	-0.08	included	present	1076.64	0.56	included	present	300.71	-1.91	included	present	712.39	-0.6	included	present	218.03	-2.18	included	present	739.68	-0.51	included	present	518.51	-1.22	included	present	298.48	-1.92	included	present	449.08	-1.44	included	present	498.58	-1.28	included	present	602.6	-0.95	included	present	700.96	-0.64	included	present	364.54	-1.71
1558621_at	excluded	absent	27.54	-1.1	excluded	absent	82.73	-0.39	excluded	absent	116.01	0.05	excluded	absent	37.04	-0.98	excluded	absent	88.3	-0.31	excluded	absent	78.38	-0.44	excluded	absent	19.38	-1.2	excluded	absent	50.04	-0.81	excluded	absent	66.16	-0.6	excluded	absent	56.74	-0.72	excluded	absent	86.07	-0.34	excluded	absent	99.16	-0.17	excluded	absent	116.88	0.06	excluded	absent	59.08	-0.69	excluded	absent	1.68	-1.43
1558622_a_a	included	present	635.01	-1.43	included	present	548.1	-1.69	included	present	1744.88	1.77	included	present	3289.26	6.23	included	present	764.32	-1.06	included	present	566.46	-1.63	included	present	573.35	-1.61	included	present	649.65	-1.39	included	present	502.35	-1.82	included	present	1101.16	-0.09	included	present	642.27	-1.41	included	present	369.99	-2.2	included	present	197.96	-2.7	included	present	260.74	-2.52	included	present	515.69	-1.78
1558623_at	excluded	absent	7.74	-0.32	excluded	absent	3.98	-0.35	excluded	absent	40.92	-0.02	excluded	absent	20.45	-0.2	excluded	absent	12.51	-0.27	excluded	absent	2.18	-0.37	excluded	absent	1.38	-0.38	excluded	absent	1.19	-0.38	excluded	absent	6.7	-0.33	excluded	absent	2.45	-0.37	excluded	absent	49.82	0.07	excluded	absent	5.16	-0.34	excluded	absent	6.56	-0.33	excluded	absent	43.77	0.01	excluded	absent	2.09	-0.37
1558624_at	included	present	71.25	-0.79	excluded	absent	28.14	-1.43	excluded	absent	99.86	-0.37	included	present	49.96	-1.11	included	present	154.39	0.44	included	present	143.68	0.28	excluded	absent	76.79	-0.71	excluded	absent	60.25	-0.96	excluded	absent	70.74	-0.8	excluded	absent	53.81	-1.05	included	present	178.53	0.79	excluded	absent	80.48	-0.66	excluded	absent	58.32	-0.98	excluded	absent	58.47	-0.98	included	present	39.98	-1.25
1558626_at	included	present	313.53	-1.25	included	present	760.93	0.02	included	present	252.98	-1.42	included	present	338.65	-1.18	included	present	408.09	-0.98	included	present	292.73	-1.31	included	present	501.75	-0.71	included	present	368.12	-1.09	included	present	594.78	-0.45	included	present	220.21	-1.51	included	present	348.65	-1.15	included	present	352.21	-1.14	included	present	869.75	0.33	included	present	443.43	-0.88	included	present	247.08	-1.44
1558628_at	included	present	47.88	-0.85	excluded	absent	26.14	-1.23	excluded	absent	74.23	-0.4	excluded	absent	77.27	-0.35	excluded	absent	68.51	-0.5	included	present	86.25	-0.19	excluded	absent	42.97	-0.94	excluded	absent	2.59	-1.63	included	present	76.8	-0.35	excluded	absent	42.67	-0.94	excluded	absent	6.69	-1.56	excluded	absent	41.07	-0.97	excluded	absent	7.98	-1.54	included	present	40.76	-0.98	included	present	40.43	-0.98
1558630_at	included	present	300.88	1.36	excluded	absent	122.02	-0.39	included	present	184.88	0.23	included	present	196.93	0.34	included	present	278.42	1.14	included	present	219.83	0.57	included	present	238.17	0.75	excluded	absent	29.72	-1.29	included	present	257.37	0.93	included	present	279.6	1.15	included	present	198.37	0.36	excluded	absent	76.09	-0.84	included	present	91.49	-0.69	excluded	absent	115.38	-0.45	included	present	67.63	-0.92
1558631_at	excluded	absent	61.57	-0.34	excluded	absent	7.2	-0.92	excluded	absent	84.15	-0.1	excluded	absent	93.32	0	excluded	absent	98.73	0.05	included	present	181.65	0.94	excluded	absent	60.25	-0.36	included	present	97.96	0.04	excluded	absent	67.25	-0.28	included	present	94.64	0.01	included	present	105.72	0.13	excluded	absent	5	-0.94	excluded	absent	6.22	-0.93	excluded	absent	0.89	-0.99	excluded	absent	29.4	-0.68
1558636_s_a	included	present	152.18	-0.21	included	present	108.1	-0.31	excluded	absent	56.65	-0.44	excluded	absent	62.8	-0.42	included	present	149.02	-0.22	included	present	294.62	0.14	excluded	absent	3.21	-0.57	excluded	absent	5.06	-0.56	included	present	305.12	0.16	excluded	absent	57.76	-0.43	included	present	167.57	-0.17	included	present	318.81	0.19	excluded	absent	85.12	-0.37	included	present	215.68	-0.05	included	present	41.55	-0.47
1558640_a_a	included	present	817.48	-0.34	included	present	249.6	-1.51	included	present	1348.13	0.75	included	present	3062.54	4.27	included	present	1356.13	0.77	included	present	759.97	-0.46	included	present	1593.48	1.25	included	present	338.4	-1.33	included	present	1885.24	1.85	included	present	1075.19	0.19	included	present	1874.92	1.83	included	present	1507.31	1.08	included	present	1911.54	1.91	included	present	976.53	-0.01	included	present	740.5	-0.5
1558641_at	excluded	absent	2.95	-1.19	excluded	absent	8.29	-1.11	excluded	absent	90.24	0.07	excluded	absent	46.27	-0.56	excluded	absent	19.07	-0.95	excluded	absent	188.49	1.49	excluded	absent	107.48	0.32	excluded	absent	8.3	-1.11	excluded	absent	48.55	-0.53	excluded	absent	79.07	-0.09	excluded	absent	16.48	-0.99	excluded	absent	3.93	-1.17	excluded	absent	2.56	-1.19	excluded	absent	55.03	-0.44	excluded	absent	53.47	-0.46
1558643_s_a	included	present	174.17	-0.63	included	absent	111.88	-0.72	included	present	60.88	-0.79	included	present	93.57	-0.74	included	present	547.72	-0.12	included	present	1560.15	1.27	included	present	505.92	-0.18	included	present	306.33	-0.45	included	present	239.19	-0.54	included	present	364.03	-0.37	included	present	284.89	-0.48	included	present	245.29	-0.53	included	present	208.4	-0.59	included	present	168.45	-0.64	included	present	58.7	-0.79
1558645_at	included	present	146.47	-0.14	included	present	138.25	-0.19	included	present	152.64	-0.11	included	present	257.58	0.46	included	present	230.92	0.32	included	present	446.68	1.49	included	present	97.41	-0.41	included	present	56.89	-0.63	included	present	172.15	0	included	present	1236.77	5.78	included	present	193.93	0.12	included	present	161.89	-0.06	included	present	233.69	0.33	included	present	175.28	0.01	included	present	73.18	-0.54
1558647_at	included	present	15263.51	0.31	included	present	2709.35	-1.19	included	present	3240.95	-1.12	included	present	3086.63	-1.14	included	present	2016.88	-1.27	included	present	7279.36	-0.64	included	present	1296.99	-1.35	included	present	2839.84	-1.17	included	present	4843.23	-0.93	included	present	1981.7	-1.27	included	present	519.29	-1.45	included	present	673.38	-1.43	included	present	2238.89	-1.24	included	present	1787.15	-1.3	included	present	317.62	-1.47
1558648_at	included	present	159.36	0.48	excluded	absent	139.61	0.17	excluded	absent	36.5	-1.47	excluded	absent	99.26	-0.47	excluded	absent	112.32	-0.27	excluded	absent	76.31	-0.84	included	present	111.81	-0.28	excluded	absent	102.39	-0.43	included	present	168.7	0.63	excluded	absent	141.84	0.2	excluded	absent	93.09	-0.57	excluded	absent	97.55	-0.5	excluded	absent	141.52	0.2	excluded	absent	43.11	-1.37	excluded	absent	77.61	-0.82
1558649_at	excluded	absent	114.46	1.3	excluded	absent	91.85	0.85	included	present	215.78	3.3	excluded	absent	2.57	-0.91	excluded	absent	150.39	2.01	excluded	absent	84.35	0.71	included	present	103.54	1.09	excluded	absent	50.33	0.03	excluded	absent	6.92	-0.82	excluded	absent	4.06	-0.88	included	present	233.28	3.65	excluded	absent	96.29	0.94	excluded	absent	5.33	-0.85	excluded	absent	114.55	1.3	included	present	80.21	0.62
1558651_at	excluded	absent	11.81	-0.94	excluded	absent	37.24	-0.21	excluded	absent	50.1	0.16	excluded	absent	1.39	-1.24	excluded	absent	3	-1.19	excluded	absent	59.74	0.44	excluded	absent	19.64	-0.71	excluded	absent	42.21	-0.07	included	present	34.94	-0.27	excluded	absent	38.7	-0.17	included	present	83.18	1.11	excluded	absent	0.81	-1.25	excluded	absent	36.75	-0.22	included	present	50.01	0.16	excluded	absent	47.07	0.07
1558652_at	excluded	absent	81.47	-0.66	included	present	56.37	-1.08	included	present	118.69	-0.05	excluded	absent	52.71	-1.14	excluded	absent	52.64	-1.14	excluded	absent	85.32	-0.6	excluded	absent	49.18	-1.2	included	present	61.14	-1	excluded	absent	52.12	-1.15	excluded	absent	64.91	-0.94	included	present	105.16	-0.27	included	present	94.27	-0.45	excluded	absent	57.5	-1.06	excluded	absent	44.08	-1.28	excluded	absent	38.62	-1.37
1558653_at	excluded	absent	46.66	-0.7	excluded	absent	26.87	-0.84	excluded	absent	75.83	-0.51	included	present	91.49	-0.4	excluded	absent	90.94	-0.41	excluded	absent	136.49	-0.1	excluded	absent	44.23	-0.72	included	present	119.7	-0.21	excluded	absent	90.56	-0.41	excluded	absent	70.51	-0.54	excluded	absent	26.78	-0.84	excluded	absent	2.07	-1	excluded	absent	36.39	-0.77	excluded	absent	39.22	-0.75	excluded	absent	28.27	-0.83
1558654_at	included	absent	80.71	-0.72	included	absent	75.9	-0.73	included	absent	62.99	-0.76	included	absent	121.16	-0.62	included	absent	138.78	-0.57	included	absent	86.14	-0.7	included	absent	3.08	-0.91	included	absent	65.64	-0.75	included	absent	7.49	-0.9	included	absent	5.1	-0.9	included	absent	24.38	-0.85	included	absent	4.24	-0.9	included	absent	115.4	-0.63	included	present	294.67	-0.19	included	absent	6.72	-0.9
1558658_at	excluded	absent	4.06	-0.5	excluded	absent	6.83	-0.35	excluded	absent	114.84	5.27	excluded	absent	37.1	1.22	excluded	absent	20.59	0.36	excluded	absent	2.77	-0.56	excluded	absent	6.41	-0.37	excluded	absent	3.69	-0.51	excluded	absent	5.14	-0.44	excluded	absent	1.54	-0.63	excluded	absent	3.2	-0.54	excluded	absent	7.87	-0.3	excluded	absent	1.98	-0.6	excluded	absent	1.25	-0.64	excluded	absent	0.99	-0.66
1558660_at	excluded	absent	55.3	-0.05	excluded	absent	13.1	-1.27	excluded	absent	40.22	-0.49	excluded	absent	62.28	0.15	excluded	absent	2.84	-1.56	excluded	absent	70.22	0.38	excluded	absent	8.97	-1.39	excluded	absent	0.62	-1.63	excluded	absent	49.48	-0.22	excluded	absent	23.74	-0.96	excluded	absent	63.05	0.17	excluded	absent	29.2	-0.8	included	present	61.02	0.11	excluded	absent	45.91	-0.32	excluded	absent	19.2	-1.09
1558661_at	excluded	absent	1.86	-0.95	excluded	absent	2.3	-0.93	excluded	absent	4.69	-0.85	excluded	absent	1.47	-0.96	excluded	absent	100.75	2.45	excluded	absent	2.27	-0.93	excluded	absent	1.66	-0.95	excluded	absent	13.45	-0.55	excluded	absent	22.3	-0.24	excluded	absent	1.21	-0.97	excluded	absent	31.39	0.07	excluded	absent	2.65	-0.92	excluded	absent	27.38	-0.07	excluded	absent	1.53	-0.96	excluded	absent	0.79	-0.98
1558662_s_a	excluded	absent	47.29	-0.28	excluded	absent	6.68	-0.34	excluded	absent	2.15	-0.35	excluded	absent	2.3	-0.35	excluded	absent	13.25	-0.33	excluded	absent	8.49	-0.34	excluded	absent	14.79	-0.33	excluded	absent	22.9	-0.31	included	present	264.5	0.06	excluded	absent	22.99	-0.31	excluded	absent	30.24	-0.3	excluded	absent	67.32	-0.25	excluded	absent	45.77	-0.28	excluded	absent	3.49	-0.34	excluded	absent	15.81	-0.33
1558666_at	excluded	absent	42.94	-0.92	excluded	absent	105.36	0.52	excluded	absent	60.05	-0.52	excluded	absent	10.92	-1.65	excluded	absent	108.16	0.59	excluded	absent	51.44	-0.72	excluded	absent	65.21	-0.4	excluded	absent	44.51	-0.88	excluded	absent	61.88	-0.48	excluded	absent	55.1	-0.64	excluded	absent	79.25	-0.08	excluded	absent	122.48	0.91	excluded	absent	58.9	-0.55	included	present	87.97	0.12	excluded	absent	35.1	-1.1
1558667_at	excluded	absent	84.95	0.86	excluded	absent	43.58	0.17	excluded	absent	1.87	-0.52	excluded	absent	1.19	-0.54	excluded	absent	3.1	-0.5	excluded	absent	7.46	-0.43	included	present	43.21	0.16	excluded	absent	14.99	-0.31	excluded	absent	8.03	-0.42	excluded	absent	2.05	-0.52	excluded	absent	1.8	-0.53	excluded	absent	2.01	-0.52	excluded	absent	20.96	-0.21	excluded	absent	1.41	-0.53	excluded	absent	15.16	-0.3
1558668_s_a	excluded	absent	82.13	-0.19	excluded	absent	77.25	-0.19	excluded	absent	9.71	-0.22	excluded	absent	33.75	-0.21	excluded	absent	2.12	-0.23	excluded	absent	6.39	-0.22	included	present	63.3	-0.2	excluded	absent	2.07	-0.23	included	present	63.24	-0.2	excluded	absent	11.51	-0.22	excluded	absent	14.21	-0.22	excluded	absent	101.96	-0.18	included	present	74.6	-0.19	excluded	absent	24.63	-0.22	excluded	absent	40.37	-0.21
1558670_at	excluded	absent	80.28	0.21	excluded	absent	30.05	-0.74	excluded	absent	71.05	0.03	excluded	absent	43.1	-0.5	excluded	absent	185.05	2.19	excluded	absent	131.49	1.18	excluded	absent	14.36	-1.04	excluded	absent	106.1	0.7	excluded	absent	60.56	-0.17	excluded	absent	74.2	0.09	excluded	absent	79.82	0.2	excluded	absent	77.58	0.16	excluded	absent	64.5	-0.09	excluded	absent	20.28	-0.93	excluded	absent	112.75	0.82
1558672_at	included	present	195.9	-0.36	excluded	absent	6.02	-0.79	included	present	130.2	-0.51	excluded	absent	113.87	-0.54	included	present	318.32	-0.08	excluded	absent	108.63	-0.56	included	present	537.19	0.41	excluded	absent	67.58	-0.65	included	present	197.84	-0.36	included	present	138.8	-0.49	included	present	398.93	0.1	included	present	592.25	0.53	included	present	396.31	0.09	included	present	628.36	0.61	included	present	416.13	0.13
1558673_s_a	excluded	absent	31.36	-0.47	excluded	absent	40.32	-0.19	excluded	absent	28.45	-0.56	excluded	absent	66.11	0.62	excluded	absent	118.92	2.27	excluded	absent	36.64	-0.3	excluded	absent	63.48	0.54	included	present	119.1	2.27	excluded	absent	10.52	-1.12	excluded	absent	23.78	-0.7	excluded	absent	49.93	0.11	included	present	81.14	1.09	excluded	absent	7.74	-1.21	excluded	absent	5.07	-1.29	excluded	absent	29.21	-0.54
1558675_s_a	included	present	1013.71	-0.21	included	present	972.08	-0.36	included	present	863.35	-0.76	included	present	874.06	-0.72	included	present	1009	-0.23	included	present	753.2	-1.16	included	present	728.09	-1.25	included	present	966.31	-0.38	included	present	868.22	-0.74	included	present	829.14	-0.88	included	present	943.32	-0.46	included	present	568.76	-1.83	included	present	930	-0.51	included	present	1620.66	2	included	present	783.77	-1.05
[illegible]																																																												

1559228_at	excluded	absent	97.31	-0.48	excluded	absent	9.42	-1.02	excluded	absent	88.5	-0.53	[illegible]
1559229_at	excluded	absent	32.28	0.07	excluded	absent	40.49	0.36	excluded	absent	20.44	-0.35	[illegible]
1559232_a_a	included	present	117.52	0.71	excluded	absent	61.23	-0.41	included	present	111.07	0.58	[illegible]
1559235_a_a	excluded	absent	50.62	0.05	excluded	absent	165.04	2.52	excluded	absent	2.75	-0.98	[illegible]
1559237_a_a	excluded	absent	25.12	-0.24	excluded	absent	226.41	4.61	excluded	absent	8.37	-0.65	[illegible]
1559239_s_a	included	present	193.74	-0.25	included	present	148.52	-0.76	included	present	344.82	1.44	[illegible]
1559240_at	excluded	absent	13.56	-1	included	present	156.95	1.12	excluded	absent	37.03	-0.66	[illegible]
1559244_at	included	present	306.79	0.38	excluded	absent	104.72	-0.46	included	present	192.04	-0.1	[illegible]
1559245_at	included	absent	153.99	-0.05	included	absent	2.17	-1.35	included	absent	73.02	-0.75	[illegible]
1559249_at	excluded	absent	69.94	-1.49	excluded	absent	41.98	-1.95	excluded	absent	1.88	-2.61	[illegible]
1559252_a_a	excluded	absent	94.87	-0.09	excluded	absent	99.25	-0.03	excluded	absent	74.71	-0.37	[illegible]
1559254_at	excluded	absent	46.99	0.55	excluded	absent	3.01	-0.76	excluded	absent	4.56	-0.71	[illegible]
1559255_a_a	included	present	95.09	0.52	excluded	absent	4.94	-1.02	excluded	absent	50.3	-0.25	[illegible]
1559256_at	excluded	absent	93.75	-0.23	included	present	282.75	2.05	included	present	76.76	-0.43	[illegible]
1559257_a_a	included	present	134.18	-0.52	included	present	393.44	1.21	included	present	181.47	-0.2	[illegible]
1559258_a_a	excluded	absent	1.83	-0.27	excluded	absent	47.39	-0.16	excluded	absent	3.44	-0.26	[illegible]
1559259_at	excluded	absent	79.13	0.27	excluded	absent	61.71	-0.11	included	present	88.45	0.48	[illegible]
1559261_a_a	excluded	absent	10.23	-1.11	excluded	absent	7.55	-1.16	excluded	absent	2.87	-1.25	[illegible]
1559263_s_a	excluded	absent	85.88	-0.21	excluded	absent	8.42	-0.5	excluded	absent	94.08	-0.18	[illegible]
1559264_at	excluded	absent	2.62	-0.86	excluded	absent	56.45	0.59	excluded	absent	26.63	-0.21	[illegible]
1559265_at	included	present	216.77	1.32	included	present	193.95	1.08	included	present	934.59	8.92	[illegible]
1559266_s_a	excluded	absent	59.96	-0.33	included	present	244.72	2.46	included	present	479.61	6	[illegible]
1559268_at	included	present	508.42	8.04	excluded	absent	40.86	-0.33	excluded	absent	54.86	-0.08	[illegible]
1559270_at	included	present	168.09	0.56	excluded	absent	115.27	0	excluded	absent	9.48	-1.12	[illegible]
1559272_at	excluded	absent	78.34	0.52	excluded	absent	121.41	1.42	excluded	absent	74.69	0.45	[illegible]
1559274_at	excluded	absent	21.14	-0.54	included	present	130.81	2.25	excluded	absent	40.11	-0.05	[illegible]
1559275_x_a	excluded	absent	50.83	-0.79	included	present	207.38	1.66	included	present	177.08	1.18	[illegible]
1559276_at	excluded	absent	101.7	0.7	excluded	absent	0.97	-0.93	excluded	absent	46.46	-0.19	[illegible]
1559277_at	excluded	absent	17.22	-0.71	excluded	absent	84.56	-0.03	excluded	absent	41.39	-0.47	[illegible]
1559278_at	excluded	absent	6.22	-0.15	excluded	absent	5.87	-0.17	excluded	absent	48.69	2.72	[illegible]
1559280_a_a	excluded	absent	1.08	-0.55	excluded	absent	2.53	-0.53	excluded	absent	17.44	-0.31	[illegible]
[illegible]	[illegible]	[illegible]	[illegible]	[illegible]	[illegible]	[illegible]	[illegible]	[illegible]	[illegible]	[illegible]	[illegible]	[illegible]	[illegible]

1559848_at	excluded	absent	75.97	-0.42	included	present	89.5	-0.19	excluded	absent	40.04	-1.03	included	present	90.01	-0.18
1559849_at	excluded	absent	8.69	-1.57	included	present	73.46	0	included	present	90.65	0.41	included	present	107.05	0.81
1559856_s_a	excluded	absent	2.71	-1.48	excluded	absent	66.85	-0.5	included	present	97.86	-0.02	included	present	79.28	-0.31
1559861_at	excluded	absent	90.37	0.59	included	present	318.66	4.03	included	present	1502.12	21.86	excluded	absent	7.83	-0.65
1559862_at	excluded	absent	106.15	1.25	excluded	absent	81.41	0.66	excluded	absent	63.78	0.24	excluded	absent	23.83	-0.7
1559863_a_a	excluded	absent	3.53	-0.63	excluded	absent	5.18	-0.39	excluded	absent	4.38	-0.51	excluded	absent	2.69	-0.75
1559864_at	excluded	absent	85.33	-0.27	excluded	absent	77.03	-0.41	excluded	absent	51.55	-0.82	excluded	absent	4.36	-1.59
1559865_at	excluded	absent	4.77	-0.53	excluded	absent	3.59	-0.57	excluded	absent	1.02	-0.68	excluded	absent	37.93	0.81
1559867_at	excluded	absent	127.29	-0.37	included	present	166.76	0.05	excluded	absent	45.41	-1.25	included	present	122.61	-0.42
1559870_at	included	present	421.02	-0.1	included	present	209.74	-0.66	included	present	158.75	-0.79	included	absent	186.21	-0.72
1559871_s_a	excluded	absent	140.06	0.52	excluded	absent	4.1	-1.19	excluded	absent	44.62	-0.68	excluded	absent	63.21	-0.44
1559876_at	excluded	absent	6.22	-0.97	excluded	absent	5.56	-0.98	excluded	absent	41.69	-0.07	excluded	absent	30.16	-0.36
1559877_at	excluded	absent	87.78	0.99	excluded	absent	42.54	-0.1	excluded	absent	2.73	-1.06	excluded	absent	69.75	0.56
1559879_a_a	excluded	absent	41.95	-0.13	excluded	absent	7.92	-1.01	excluded	absent	17.73	-0.75	excluded	absent	2.19	-1.16
1559880_at	excluded	absent	95.41	2.76	excluded	absent	3.78	-0.71	excluded	absent	6.9	-0.6	excluded	absent	28.13	0.21
1559881_s_a	included	present	938.44	0.16	included	present	557.25	-1.03	included	present	1033.48	0.46	included	present	1443.66	1.74
1559882_at	included	present	214.15	1.9	excluded	absent	7.52	-0.94	excluded	absent	114.05	0.52	excluded	absent	1.89	-1.02
1559883_s_a	included	present	2138.88	2.66	included	present	364.91	-0.54	included	present	621.85	-0.07	included	present	225.84	-0.79
1559884_at	excluded	absent	8.39	-0.5	excluded	absent	36.14	0.4	excluded	absent	3.08	-0.67	included	present	162.16	4.45
1559889_at	included	present	371.99	-1	included	present	125.99	-1.43	included	present	2917.46	3.52	included	present	336.54	-1.06
1559890_a_a	included	present	318.03	-1.03	included	present	173.12	-1.42	included	present	264.9	-1.17	included	present	338.35	-0.97
1559891_at	excluded	absent	16.25	-0.54	excluded	absent	12.37	-0.67	excluded	absent	29.04	-0.12	excluded	absent	10.47	-0.73
1559893_at	included	present	853.46	0.81	included	present	1008.2	1.35	included	present	1527.77	3.19	included	present	765.79	0.5
1559895_x_a	included	present	115	0.21	excluded	absent	97.64	-0.06	excluded	absent	142.62	0.63	excluded	absent	102.46	0.01
1559901_s_a	included	present	1240.87	-0.68	included	absent	105.98	-1.14	included	present	4283.83	0.55	included	present	504.49	-0.98
1559902_at	included	present	494.55	0.24	included	present	516.75	0.36	included	present	268.8	-0.97	included	present	244.86	-1.1
1559909_a_a	excluded	absent	3.59	-0.64	excluded	absent	64.11	1.49	excluded	absent	11.65	-0.36	excluded	absent	5.46	-0.58
1559910_at	included	present	238.87	0.25	included	absent	51.81	-0.81	included	absent	10.18	-1.04	included	absent	68.3	-0.72
1559911_at	included	present	329.97	-0.53	included	present	194.56	-0.59	included	present	300.68	-0.54	included	present	317.74	-0.53
1559914_at	excluded	absent	76.71	0.54	excluded	absent	4.6	-1.05	excluded	absent	1.53	-1.12	excluded	absent	3.53	-1.08
1559915_at	excluded	absent	43.47	-0.12	excluded	absent	55.98	0.14	excluded	absent	32.52	-0.35	excluded	absent	39.91	-0.2
1559916_a_a	excluded	absent	151.77	0.08	included	present	229.96	1.09	included	present	364.36	2.84	excluded	absent	109.65	-0.46
1559917_a_a	excluded	absent	79.46	0.12	excluded	absent	38.39	-0.16	excluded	absent	72.66	0.07	excluded	absent	180.07	0.83
1559920_a_a	excluded	absent	31.45	-0.15	excluded	absent	77.18	1.25	included	present	73.8	1.14	excluded	absent	1.82	-1.06
1559921_at	excluded	absent	11.29	-0.83	excluded	absent	8.07	-0.84	excluded	absent	45.49	-0.67	excluded	absent	4.14	-0.86
1559922_at	excluded	absent	94.35	-0.24	excluded	absent	88.74	-0.35	excluded	absent	51.94	-1.06	included	present	83.25	-0.45
1559923_at	included	present	276.43	2.01	included	absent	62.06	-1.09	included	absent	64.38	-1.06	included	present	75.36	-0.9
1559924_at	excluded	absent	3.35	-0.88	excluded	absent	6.34	-0.85	excluded	absent	9.58	-0.81	excluded	absent	67.45	-0.12
1559925_s_a	excluded	absent	9.03	-0.59	excluded	absent	10.66	-0.54	excluded	absent	45.26	0.51	excluded	absent	30.68	0.07
1559926_at	excluded	absent	15.11	-0.58	excluded	absent	5.64	-0.79	excluded	absent	6.42	-0.78	excluded	absent	38.13	-0.06
1559927_a_a	excluded	absent	5.25	-0.55	excluded	absent	101.31	0.43	excluded	absent	40.81	-0.19	excluded	absent	71.24	0.12
1559928_at	included	present	116.59	1.22	excluded	absent	58.16	-0.24	excluded	absent	29.74	-0.94	excluded	absent	1.8	-1.64

1560495_at	excluded	absent	68.57	0.86	excluded	absent	69.93	0.9	excluded	absent	33.62	-0.26	excluded	absent	1.38	-1.29	excluded	absent	9.48	-1.03	excluded	absent	4.97	-1.17	excluded	absent	103.87	1.98	excluded	absent	41.52	-0.01	included	present	61.64	0.63	excluded	absent	2.41	-1.26	excluded	absent	39.49	-0.07	excluded	absent	45.93	0.13	excluded	absent	39.2	-0.08	excluded	absent	1.97	-1.27	excluded	absent	16.46	-0.81
1560496_at	excluded	absent	2.88	-0.67	excluded	absent	5.72	-0.59	excluded	absent	2.66	-0.68	excluded	absent	10.12	-0.46	excluded	absent	5.99	-0.58	excluded	absent	4.68	-0.62	excluded	absent	4.48	-0.63	excluded	absent	3.42	-0.66	excluded	absent	6.22	-0.58	excluded	absent	2.34	-0.69	excluded	absent	24.12	-0.04	excluded	absent	20.06	-0.17	excluded	absent	3.44	-0.66	excluded	absent	4.59	-0.62	excluded	absent	1.23	-0.72
1560498_at	excluded	absent	13.83	-0.45	excluded	absent	5.08	-0.64	excluded	absent	3.75	-0.67	excluded	absent	61.22	0.58	excluded	absent	14.81	-0.43	excluded	absent	78.3	0.95	excluded	absent	3.93	-0.66	excluded	absent	41.8	0.16	excluded	absent	20.02	-0.31	excluded	absent	41.63	0.16	excluded	absent	66.79	0.7	excluded	absent	14.05	-0.44	excluded	absent	23.21	-0.24	excluded	absent	5.87	-0.62	excluded	absent	5.09	-0.64
1560499_at	excluded	absent	3.93	-1.13	excluded	absent	39.49	0.37	excluded	absent	5.45	-1.07	excluded	absent	1.03	-1.26	excluded	absent	36.82	0.26	excluded	absent	45.34	0.62	excluded	absent	35.27	0.19	excluded	absent	42.88	0.51	excluded	absent	12.86	-0.76	excluded	absent	4.28	-1.12	excluded	absent	45.44	0.62	excluded	absent	6.36	-1.03	excluded	absent	7.41	-0.99	excluded	absent	2.04	-1.21	excluded	absent	1.56	-1.23
1560500_at	included	present	200.54	0.02	included	present	85.12	-0.73	included	present	124.53	-0.48	included	present	192.94	-0.03	included	present	470.42	1.77	included	absent	95.72	-0.66	included	present	307.46	0.71	included	present	127.47	-0.46	included	present	381.74	1.2	included	present	226.19	0.19	included	present	190.7	-0.05	included	present	94.24	-0.67	included	present	438	1.56	included	absent	74.74	-0.8	included	present	90.11	-0.7
1560503_a_a	included	present	67.22	0.39	excluded	absent	7.04	-0.53	excluded	absent	1.82	-0.61	included	present	368.38	5	excluded	absent	64.44	0.35	excluded	absent	2.51	-0.6	excluded	absent	17.91	-0.37	excluded	absent	38.33	-0.05	excluded	absent	45.69	0.06	excluded	absent	22.64	-0.29	excluded	absent	2.23	-0.61	excluded	absent	53.5	0.18	excluded	absent	2.63	-0.6	excluded	absent	2.39	-0.61	excluded	absent	9.72	-0.49
1560506_at	excluded	absent	6.88	-0.86	excluded	absent	41.88	-0.21	excluded	absent	36.07	-0.32	excluded	absent	12.2	-0.76	included	present	137.05	1.56	excluded	absent	7.61	-0.85	excluded	absent	14.55	-0.72	excluded	absent	3.06	-0.93	included	present	55.86	0.05	excluded	absent	123.14	1.31	excluded	absent	3.62	-0.92	excluded	absent	1.69	-0.96	excluded	absent	2.45	-0.94	excluded	absent	9.22	-0.82	excluded	absent	12.02	-0.76
1560507_at	excluded	absent	7.48	-0.49	excluded	absent	44.8	1.49	excluded	absent	6.78	-0.52	excluded	absent	2.41	-0.75	excluded	absent	5.39	-0.6	excluded	absent	3.88	-0.68	excluded	absent	46.23	1.57	excluded	absent	10.56	-0.32	excluded	absent	7.89	-0.46	excluded	absent	14.72	-0.1	excluded	absent	54.86	2.02	excluded	absent	29.73	0.69	excluded	absent	2.64	-0.74	excluded	absent	9.6	-0.37	excluded	absent	3.62	-0.69
1560509_at	excluded	absent	18.61	-0.89	excluded	absent	114.17	1.1	included	present	102.95	0.86	included	present	142.92	1.69	excluded	absent	91.47	0.62	excluded	absent	85.01	0.49	excluded	absent	2.52	-1.23	excluded	absent	12.99	-1.01	excluded	absent	32.14	-0.61	included	present	64.34	0.06	included	present	207.37	3.04	excluded	absent	68.99	0.16	excluded	absent	61.87	0.01	included	present	61.58	0	excluded	absent	38.33	-0.48
1560510_at	excluded	absent	1.58	-0.67	excluded	absent	4.73	-0.57	excluded	absent	35.88	0.39	excluded	absent	1.18	-0.68	excluded	absent	56.48	1.03	excluded	absent	2.65	-0.63	excluded	absent	50.76	0.85	excluded	absent	37.45	0.44	excluded	absent	41.69	0.57	excluded	absent	27.66	0.14	excluded	absent	5.06	-0.56	excluded	absent	1.84	-0.66	excluded	absent	6.68	-0.51	excluded	absent	7.77	-0.47	excluded	absent	20.36	-0.09
1560511_at	excluded	absent	11.95	-0.75	excluded	absent	60.03	0.46	excluded	absent	4.16	-0.95	excluded	absent	22.43	-0.49	excluded	absent	117.79	1.92	excluded	absent	4.37	-0.94	excluded	absent	77.9	0.91	excluded	absent	43.98	0.05	excluded	absent	56.91	0.38	excluded	absent	80.98	0.99	excluded	absent	49.11	0.18	excluded	absent	14.16	-0.7	excluded	absent	55.16	0.34	excluded	absent	11.12	-0.77	excluded	absent	53.49	0.29
1560512_at	excluded	absent	9.22	-1.36	excluded	absent	125.11	0.04	excluded	absent	40.58	-0.98	included	present	175.45	0.65	excluded	absent	4.05	-1.42	excluded	absent	3.34	-1.43	excluded	absent	22.82	-1.2	excluded	absent	8.01	-1.38	excluded	absent	26.55	-1.15	included	present	114.31	-0.09	excluded	absent	11.18	-1.34	excluded	absent	60.48	-0.74	included	present	168.85	0.57	excluded	absent	85.05	-0.44	excluded	absent	42.83	-0.95
1560513_at	excluded	absent	86.31	0.39	excluded	absent	4.65	-0.32	excluded	absent	3.28	-0.33	excluded	absent	3.2	-0.33	excluded	absent	6.66	-0.3	excluded	absent	73.89	0.29	excluded	absent	16.94	-0.21	excluded	absent	8.01	-0.29	excluded	absent	40.82	0	excluded	absent	39.02	-0.02	excluded	absent	15.64	-0.22	excluded	absent	34.24	-0.06	excluded	absent	60.62	0.17	excluded	absent	4.71	-0.32	excluded	absent	6.2	-0.31
1560514_at	excluded	absent	72.74	1.39	excluded	absent	76.94	1.52	excluded	absent	71.41	1.35	excluded	absent	11.04	-0.55	excluded	absent	34.76	0.2	excluded	absent	50.29	0.68	excluded	absent	5.12	-0.74	excluded	absent	36.52	0.25	included	present	104.24	2.38	excluded	absent	18.66	-0.31	excluded	absent	50.65	0.7	excluded	absent	6.98	-0.68	excluded	absent	111.34	2.6	excluded	absent	2.17	-0.83	excluded	absent	72.53	1.38
1560516_at	excluded	absent	6.87	-0.53	excluded	absent	2.41	-0.61	excluded	absent	1.09	-0.63	excluded	absent	23.48	-0.25	excluded	absent	120.7	1.43	excluded	absent	2.04	-0.62	excluded	absent	5.68	-0.55	excluded	absent	1.39	-0.63	excluded	absent	24.09	-0.24	excluded	absent	9.76	-0.48	excluded	absent	3.23	-0.6	excluded	absent	55.69	0.31	included	present	106.75	1.19	excluded	absent	22.97	-0.26	excluded	absent	1.28	-0.63
1560517_s_a	excluded	absent	73.89	0.03	excluded	absent	84.19	0.1	excluded	absent	2.2	-0.44	excluded	absent	4.16	-0.43	excluded	absent	79.59	0.07	excluded	absent	1.49	-0.44	excluded	absent	1.95	-0.44	excluded	absent	4	-0.43	excluded	absent	22.06	-0.31	excluded	absent	1.72	-0.44	excluded	absent	12.51	-0.37	excluded	absent	2.19	-0.44	included	present	68.74	0	excluded	absent	19.84	-0.32	excluded	absent	2.26	-0.44
1560520_at	excluded	absent	29.12	-0.86	excluded	absent	94.13	0.15	excluded	absent	53.76	-0.48	excluded	absent	19.54	-1.01	excluded	absent	121.48	0.58	included	present	141.01	0.89	excluded	absent	2.93	-1.27	excluded	absent	69.42	-0.23	included	present	91.88	0.12	excluded	absent	79.97	-0.07	excluded	absent	47.81	-0.57	excluded	absent	84.01	0	included	present	69.5	-0.23	excluded	absent	48.97	-0.55	excluded	absent	26.3	-0.91
1560522_at	excluded	absent	2.28	-1.33	excluded	absent	1.53	-1.36	excluded	absent	38.62	-0.15	excluded	absent	16.85	-0.86	excluded	absent	69.99	0.87	excluded	absent	2.35	-1.33	excluded	absent	3.07	-1.31	excluded	absent	4.25	-1.27	included	present	29.35	-0.45	excluded	absent	74.15	1.01	excluded	absent	37.34	-0.19	excluded	absent	2.8	-1.32	excluded	absent	24.3	-0.62	excluded	absent	30.39	-0.42	excluded	absent	21.04	-0.72
1560523_at	excluded	absent	48.3	0.52	excluded	absent	59.95	0.87	excluded	absent	3.41	-0.84	excluded	absent	14.01	-0.52	excluded	absent	5.18	-0.79	excluded	absent	64.64	1.02	excluded	absent	34.68	0.11	included	present	175.03	4.37	excluded	absent	29.93	-0.04	excluded	absent	43.9	0.39	excluded	absent	11.88	-0.58	excluded	absent	4.29	-0.81	excluded	absent	54.48	0.71	excluded	absent	2.51	-0.87	excluded	absent	3.17	-0.85
1560524_at	excluded	absent	107.87	0.9	excluded	absent	72.09	0.36	excluded	absent	9.31	-0.59	excluded	absent	40.08	-0.13	excluded	absent	71.74	0.35	excluded	absent	126.43	1.18	excluded	absent	89.76	0.63	excluded	absent	44.85	-0.05	included	present	167.6	1.81	excluded	absent	47.04	-0.02	included	present	264.63	3.28	excluded	absent	27.22	-0.32	excluded	absent	69.66	0.32	excluded	absent	60.51	0.18	excluded	absent	74.56	0.4
1560525_at	excluded	absent	10.45	-0.79	excluded	absent	44.52	-0.58	excluded	absent	66.77	-0.44	excluded	absent	64.54	-0.45	excluded	absent	127.1	-0.06	excluded	absent	68.83	-0.43	included	present	152.5	0.1	excluded	absent	0.94	-0.85	excluded	absent	25	-0.7	included	present	135.61	-0.01	excluded	absent	54.27	-0.52	excluded	absent	29.97	-0.67	excluded	absent	42.4	-0.59	excluded	absent	67.38	-0.44	excluded	absent	28.27	-0.68
1560526_at	included	present	110.23	0.41	excluded	absent	76.13	-0.14	excluded	absent	132.16	0.76	excluded	absent	43.61	-0.66	included	present	488.61	6.47	excluded	absent	102.86	0.29	included	present	174.02	1.43	excluded	absent	17.36	-1.08	included	present	87.45	0.04	included	present	256.96	2.76	excluded	absent	13.63	-1.14	included	present	70.73	-0.22	included	present	99.57	0.24	included	present	169.44	1.36	excluded	absent	69.83	-0.24
1560527_at	excluded	absent	2.51	-0.43	excluded	absent	10.78	-0.36	excluded	absent	39.72	-0.14	excluded	absent	66.47	0.06	excluded	absent	132.36	0.57	excluded	absent	55.35	-0.02	excluded	absent	60.59	0.02	excluded	absent	1.87	-0.43	excluded	absent	34.69	-0.18	excluded	absent	6.94	-0.39	excluded	absent	36.55	-0.17	included	present	154.89	0.74	excluded	absent	68.52	0.08	excluded	absent	19.73	-0.3	excluded	absent	4.78	-0.41
1560528_at	excluded	absent	24.61	-0.3	excluded	absent	75.41	1.21	excluded	absent	42.21	0.23	excluded	absent	48.32	0.41	excluded	absent	101.03	1.96	excluded	absent	27.45	-0.21	excluded	absent	79.97	1.34	excluded	absent	18.62	-0.47	excluded	absent	2.91	-0.94	excluded	absent	2.05	-0.96	excluded	absent	23.62	-0.32	excluded	absent	1.58	-0.98	excluded	absent	28.82	-0.17	excluded	absent	24.23	-0.31	excluded	absent	21.41	-0.39
1560531_at	excluded	absent	7.49	-0.27	excluded	absent	63.23	-0.26	excluded	absent	21.84	-0.27	excluded	absent	13.38	-0.27	excluded	absent	4.71	-0.27	excluded	absent	11.39	-0.27	excluded	absent	79.75	-0.26	excluded	absent	7.93	-0.27	excluded	absent	17.52	-0.27	excluded	absent	8.21	-0.27	excluded	absent	21.14	-0.27	excluded	absent	9.58	-0.27	excluded	absent	12.92	-0.27	excluded	absent	15.81	-0.27	excluded	absent	7.6	-0.27
1560533_at	excluded	absent	40.55	-1.05	excluded	absent	120.1	0.45	excluded	absent	27.42	-1.3	included	present	102.74	0.13	excluded	absent	112.63	0.31	excluded	absent	95.2	-0.02	excluded	absent	58.39	-0.71	excluded	absent	53.11	-0.81	excluded	absent	34.2	-1.17	excluded	absent	94.98	-0.02	included	present	74.87	-0.4	excluded	absent	45.9	-0.95	included	present	152.86	1.07	excluded	absent	40.16	-1.06	excluded	absent	0.95	-1.8
1560536_at	included	absent	127.25	-0.77	included	absent	109.5	-1.06	included	present	175.18	0.01	excluded	absent	249.52	1.22	excluded	absent	259.12	1.38	included	absent	112.44	-1.01	included	present	206.49	0.52	included	present	223.96	0.8	included	present	196.8	0.36	included	present	226.03	0.84	included	present	338.82	2.67	included	present	268.78	1.53	included	absent	113.16	-1	included	present	257.92	1.36	included	present	148.23	-0.43
1560537_at	excluded	absent	63.79	0.7	excluded	absent	48.08	0.28	excluded	absent	52.32	0.39	excluded	absent	1.36	-0.98	excluded	absent	8.21	-0.8	excluded	absent	48.89	0.3	excluded	absent	3.13	-0.93	excluded	absent	2.21	-0.96	excluded	absent	57.57	0.53	excluded	absent	30.94	-0.18	excluded	absent	41.27	0.09	excluded	absent	38.8	0.03	excluded	absent	0.74	-1	excluded	absent	40.92	0.08	excluded	absent	23.71	-0.38
1560538_at	included	present	76.6	0.47	excluded	absent	79.51	0.54	excluded	absent	76.9	0.48	excluded	absent	10.57	-1.08	excluded	absent	108.73	1.23	excluded	absent	49.96	-0.15	included	present	112.97	1.33	excluded	absent	23.98	-0.76	included	present	75.27	0.44	excluded	absent	1.62	-1.29	included	present	77.61	0.5	excluded	absent	59.79	0.08	included	present	122.45	1.55	included	present	81.41	0.58	included	present	42.12	-0.34
1560540_x_a	included	present	88.53	0.6	excluded	absent	44.26	-0.22	included	present	173.33	2.17	excluded	absent	50.53	-0.11	excluded	absent	100.55	0.82	excluded	absent	59.63	0.06	excluded	absent	64.71	0.16	excluded	absent	18.95	-0.7	included	present	97.71	0.77	excluded	absent	45.08	-0.21	included	present	100.41	0.82	excluded	absent	50.29	-0.11	excluded	absent	46.85	-0.18	excluded	absent	11.22	-0.84	excluded	absent	30.7	-0.48
1560542_at	included	present	89.5	0.57	excluded	absent	44.84	-0.44	included	present	76.51	0.28	excluded	absent	45.8	-0.41	excluded	absent	142.39	1.77	excluded	absent	38.28	-0.58	excluded	absent	141.86	1.76	excluded	absent	33.94	-0.68	included	present	83.98	0.45	included	present	94.82	0.69	excluded	absent	60.78	-0.08	excluded	absent	97.5	0.75	excluded	absent	53.72	-0.24	included	present	84.83	0.47	included	present	56.07	-0.18
1560543_at	excluded	absent	108.97	0.55	included	present	137.57	1.13	included	present	183.94	2.07	excluded	absent	105.52	0.48	excluded	absent	16.68	-1.33	excluded	absent	26.04	-1.14	included	present	205.92	2.51	included	present	121.11	0.79	included	present	128.68	0.95	excluded	absent	59.75	-0.45	included	present	198.59	2.37	included	present	214.4	2.69	excluded	absent	149.25	1.36	excluded	absent	247.63	3.36	included	present	176.82	1.92
1560547_at	excluded	absent	46.42	0.23	excluded	absent	57.01	0.51	included	present	136.75	2.59	excluded	absent	120.77	2.18	excluded	absent	64.99	0.72	excluded	absent	4.69	-0.86	excluded	absent	66.34	0.75	excluded	absent	3.16	-0.9	excluded	absent	62.28	0.65	excluded	absent	45.92	0.22	excluded	absent	32.68	-0.13	excluded	absent	34.32	-0.08	excluded	absent	25.3	-0.32	excluded	absent	2.52	-0.91	excluded	absent	29.97	-0.2
1560548_at	excluded	absent	1.72	-0.71	excluded	absent	49.51	0.98	excluded	absent	1.52	-0.71	excluded	absent	6.7	-0.53	excluded	absent	70.88	1.73	excluded	absent	6.97	-0.52	excluded	absent	4.87	-0.6	excluded	absent	32.77	0.39	excluded	absent	3.01	-0.66	excluded	absent	4.17	-0.62	excluded	absent	25.21	0.12	excluded	absent	2.54	-0.68	excluded	absent	1.79	-0.7	excluded	absent	4.02	-0.63	excluded	absent	1.53	-0.71

[illegible]

1561079_at	included	present	663.91	-0.82	included	present	575.17	-1.16	included	present	614.55	-1.01	included	present	349.21	-2.03	included	present	507.4	-1.42
1561080_at	excluded	absent	46.61	0.8	excluded	absent	1.35	-0.87	excluded	absent	2.53	-0.83	excluded	absent	31.83	0.26	excluded	absent	3.32	-0.8
1561082_at	excluded	absent	123.87	0.9	excluded	absent	86.71	0.27	excluded	absent	33.59	-0.64	excluded	absent	5.96	-1.11	excluded	absent	150.65	1.35
1561083_at	excluded	absent	2.49	-0.87	excluded	absent	47.05	0.35	excluded	absent	12.68	-0.59	excluded	absent	3.26	-0.85	excluded	absent	71.81	1.03
1561084_at	excluded	absent	2.14	-0.8	excluded	absent	71.71	1.73	excluded	absent	6.25	-0.65	excluded	absent	3.27	-0.76	excluded	absent	3.23	-0.76
1561085_at	excluded	absent	1.98	-0.79	excluded	absent	3.77	-0.73	excluded	absent	2.66	-0.77	excluded	absent	3.29	-0.75	excluded	absent	5.44	-0.68
1561086_at	excluded	absent	7.56	-0.96	excluded	absent	12.34	-0.88	excluded	absent	51.26	-0.2	excluded	absent	18.86	-0.76	excluded	absent	4.12	-1.02
1561087_at	excluded	absent	10.21	-0.25	excluded	absent	14.05	-0.21	excluded	absent	11.7	-0.23	excluded	absent	35.52	-0.01	excluded	absent	3	-0.31
1561088_at	excluded	absent	11.11	-0.17	excluded	absent	2.46	-0.59	excluded	absent	86.13	3.43	excluded	absent	1.37	-0.64	excluded	absent	118.98	5.01
1561089_at	excluded	absent	63	-0.13	excluded	absent	77.01	0.19	included	present	122.75	1.24	excluded	absent	72.25	0.08	excluded	absent	164.2	2.19
1561090_at	excluded	absent	6.74	-0.44	excluded	absent	4.09	-0.57	excluded	absent	1.75	-0.68	excluded	absent	1.36	-0.7	excluded	absent	55.88	1.94
1561091_at	excluded	absent	38.32	0.67	excluded	absent	5.83	-0.4	excluded	absent	3.3	-0.49	excluded	absent	7.5	-0.35	excluded	absent	8.04	-0.33
1561092_at	excluded	absent	156.67	0.87	included	present	268.74	2.38	included	present	100.51	0.11	included	present	75.49	-0.23	included	present	289.89	2.67
1561093_at	excluded	absent	152.15	-0.07	excluded	absent	87.59	-0.39	excluded	absent	43.7	-0.61	excluded	absent	19.09	-0.74	excluded	absent	29.39	-0.68
1561094_a_a	excluded	absent	22.43	-0.13	excluded	absent	2.09	-0.2	excluded	absent	2.01	-0.2	excluded	absent	9.24	-0.17	excluded	absent	68.55	0.03
1561096_at	excluded	absent	75.01	0.57	excluded	absent	70.9	0.48	excluded	absent	49.18	0.02	excluded	absent	43.73	-0.1	excluded	absent	3.91	-0.95
1561097_at	excluded	absent	13.09	-0.29	excluded	absent	7.04	-0.51	excluded	absent	27.44	0.26	excluded	absent	32.96	0.46	excluded	absent	8.21	-0.47
1561098_at	excluded	absent	2.54	-0.69	excluded	absent	8.74	-0.5	excluded	absent	2.61	-0.69	excluded	absent	9.54	-0.48	excluded	absent	68.23	1.32
1561099_at	excluded	absent	20.78	-0.77	excluded	absent	11.38	-0.98	excluded	absent	92.37	0.78	excluded	absent	51.79	-0.1	excluded	absent	97.39	0.89
1561100_at	excluded	absent	1.16	-0.71	excluded	absent	3.53	-0.58	excluded	absent	38.59	1.29	excluded	absent	69.21	2.92	excluded	absent	3.82	-0.57
1561101_at	excluded	absent	89.51	1.39	excluded	absent	77.5	1.05	included	present	121.06	2.3	excluded	absent	35.1	-0.16	included	present	245.41	5.85
1561102_at	excluded	absent	7.65	-0.67	excluded	absent	34.32	-0.21	excluded	absent	12.36	-0.59	excluded	absent	73	0.45	excluded	absent	15.64	-0.53
1561103_at	excluded	absent	59.52	1.15	excluded	absent	15.21	-0.35	excluded	absent	19.37	-0.21	excluded	absent	1.99	-0.8	excluded	absent	117.19	3.11
1561104_at	excluded	absent	2.64	-0.72	excluded	absent	4.14	-0.66	excluded	absent	10.68	-0.41	excluded	absent	2.18	-0.74	excluded	absent	4.14	-0.66
1561105_at	included	present	94.82	0.9	excluded	absent	3.35	-1.07	excluded	absent	8.03	-0.97	excluded	absent	1.95	-1.1	excluded	absent	36.94	-0.34
1561106_at	excluded	absent	33.82	-0.61	excluded	absent	2.09	-1.31	excluded	absent	30.59	-0.68	excluded	absent	8.57	-1.17	excluded	absent	25.73	-0.79
1561107_at	excluded	absent	63.67	-0.05	excluded	absent	15.32	-0.95	excluded	absent	17.64	-0.91	excluded	absent	23.48	-0.8	excluded	absent	80.15	0.25
1561110_at	excluded	absent	115.19	-0.18	included	present	78.39	-0.57	excluded	absent	58.49	-0.78	included	present	84.75	-0.5	included	present	137.88	0.06
1561112_at	excluded	absent	3.38	-0.85	excluded	absent	5.18	-0.8	excluded	absent	3.3	-0.85	excluded	absent	1.23	-0.91	excluded	absent	5.81	-0.78
1561113_at	excluded	absent	42.22	-0.51	excluded	absent	50.52	-0.37	included	present	137.64	1.1	excluded	absent	98.23	0.44	excluded	absent	95.82	0.39
1561114_a_a	excluded	absent	8.46	-1.11	excluded	absent	114.23	0.99	excluded	absent	70.89	0.13	included	present	154.16	1.78	excluded	absent	161.44	1.92
1561116_at	excluded	absent	14.1	-0.6	excluded	absent	7.81	-0.76	excluded	absent	2.72	-0.9	excluded	absent	1.81	-0.92	excluded	absent	137.48	2.62
1561121_at	included	present	186.69	-0.04	included	absent	82.9	-1.01	included	absent	102.02	-0.83	included	present	156.16	-0.32	included	absent	102.51	-0.83
1561122_a_a	excluded	absent	51.74	0.29	excluded	absent	1.94	-0.7	excluded	absent	3.92	-0.66	excluded	absent	6.99	-0.6	excluded	absent	5.75	-0.62
1561123_at	excluded	absent	79.67	-0.29	excluded	absent	87.81	-0.23	excluded	absent	1.69	-0.89	excluded	absent	18.77	-0.76	excluded	absent	74.3	-0.33
1561125_at	excluded	absent	37.52	-0.66	excluded	absent	77.71	0.1	excluded	absent	44.32	-0.53	excluded	absent	35.74	-0.69	excluded	absent	6.31	-1.25
1561126_at	excluded	absent	75.64	0.19	excluded	absent	6.31	-1.61	included	present	115.6	1.23	included	present	121.71	1.39	excluded	absent	140.49	1.88
1561127_at	excluded	absent	72.6	0.15	excluded	absent	32.42	-0.59	excluded	absent	101.81	0.69	excluded	absent	68.48	0.07	excluded	absent	12.84	-0.95
1561128_at	excluded	absent	34.3	-0.11	excluded	absent	7.31	-0.84	excluded	absent	57.44	0.53	excluded	absent	54.93	0.46	excluded	absent	27.49	-0.29
1561129_at	included	present	199.77	0.65	excluded	absent	94.74	-0.58	excluded	absent	46.49	-1.14	included	present	260.79	1.36	included	present	405.3	3.05
1561130_at	included	present	147.34	-1.25	included	present	190.93	-0.67	excluded	absent	243.91	0.03	included	present	390.46	1.96	included	present	284.24	0.56
1561132_at	included	present	45.24	-1.24	excluded	absent	113.15	-0.08	excluded	absent	122.81	0.09	excluded	absent	29.73	-1.51	included	present	211.94	1.61
1561133_at	excluded	absent	4.42	-0.87	excluded	absent	2.68	-0.94	excluded	absent	23.17	-0.17	excluded	absent	72.45	1.67	excluded	absent	108.03	3.01
1561134_at	excluded	absent	9.32	-0.82	included	present	133.05	2	excluded	absent	7.34	-0.87	excluded	absent	2.6	-0.98	excluded	absent	3.01	-0.97
1561135_at	included	present	135.48	-0.32	included	present	124.97	-0.37	included	absent	50.26	-0.73	included	absent	1.47	-0.97	included	present	144.53	-0.27
1561136_at	excluded	absent	39.42	0.37	excluded	absent	30.68	0.09	excluded	absent	4.81	-0.72	excluded	absent	0.72	-0.85	excluded	absent	10.75	-0.53
1561137_s_a	excluded	absent	61.06	0.94	excluded	absent	49.63	0.58	excluded	absent	39.48	0.26	excluded	absent	2.39	-0.93	excluded	absent	2.26	-0.93
1561139_at	excluded	absent	74.35	0.04	excluded	absent	124.62	1.05	excluded	absent	105.5	0.67	excluded	absent	29.3	-0.87	included	present	232.69	3.23
1561140_at	excluded	absent	3.37	-0.55	excluded	absent	10.95	-0.23	excluded	absent	40.17	1	excluded	absent	1.15	-0.64	excluded	absent	58.16	1.76
1561141_at	excluded	absent	74.78	0.5	excluded	absent	4.81	-0.87	excluded	absent	66.01	0.33	excluded	absent	80.52	0.62	excluded	absent	160.13	2.18
1561143_at	excluded	absent	37.18	0.03	excluded	absent	6.57	-0.75	excluded	absent	6.25	-0.75	excluded	absent	5.2	-0.78	excluded	absent	3.87	-0.82
1561144_at	excluded	absent	33.05	-0.06	excluded	absent	101.22	1.77	excluded	absent	2.53	-0.87	excluded	absent	15.13	-0.54	excluded	absent	71.17	0.97
1561146_at	excluded	absent	74.48	-0.43	excluded	absent	25.04	-1.58	included	present	166.29	1.71	included	present	91.18	-0.04	included	present	145.99	1.24
1561148_at	excluded	absent	67.56	0.33	excluded	absent	6.03	-0.87	excluded	absent	91.98	0.81	included	present	129.9	1.55	excluded	absent	87.66	0.72
1561149_at	excluded	absent	46.63	-0.16	excluded	absent	69.39	0.07	excluded	absent	66.92	0.05	excluded	absent	7.7	-0.56	excluded	absent	2.76	-0.61
1561151_a_a	excluded	absent	47.92	-0.25	excluded	absent	1.99	-1.47	excluded	absent	1.1	-1.5	excluded	absent	127.15	1.85	excluded	absent	3.73	-1.43
1561152_a_a	excluded	absent	2.25	-1.04	excluded	absent	40.65	0.16	excluded	absent	3.81	-0.99	excluded	absent	49.68	0.45	excluded	absent	117.17	2.56
1561153_at	excluded	absent	36.06	-0.31	excluded	absent	44.69	-0.09	excluded	absent	91.04	1.06	excluded	absent	2.72	-1.14	excluded	absent	104.47	1.4
1561154_at	included	present	139.14	0.34	excluded	absent	6.4	-1.48	excluded	absent	104.35	-0.14	excluded	absent	100.79	-0.19	excluded	absent	120.83	0.09
1561155_at	included	present	207.42	-0.92	included	present	214.51	-0.89	included	present	351.31	-0.28	included	present	265.63	-0.66	included	absent	169.66	-1.09
1561156_at	excluded	absent	1.96	-0.59	excluded	absent	3.43	-0.53	excluded	absent	6.77	-0.38	excluded	absent	1.35	-0.62	excluded	absent	106.85	4.07
1561158_at	included	present	268.87	3.94	excluded	absent	60.34	-0.14	excluded	absent	80.45	0.26	included	present	104.86	0.73	excluded	absent	85.15	0.35
1561161_at	included	present	100.62	-1.4	included	absent	111.69	-1.29	included	absent	41.46	-1.95	included	present	174.97	-0.71	included	present	409.62	1.47
1561164_at	included	present	164.73	1.87	excluded	absent	23.39	-0.96	excluded	absent	65.89	-0.11	excluded	absent	33.04	-0.77	included	present	105.45	0.68
1561165_a_a	excluded	absent	19.71	-1.48	excluded	absent	36.41	-1.15	included	present	124.56	0.61	included	present	95.57	0.03	excluded	absent	116.28	0.44
1561166_a_a	excluded	absent	16.19	-0.37	excluded	absent	2.04	-0.81	excluded	absent	2.19	-0.8	excluded	absent	1.25	-0.83	excluded	absent	57.69	0.92

[illegible]

1561562_at	excluded	absent	8.28	-0.93	excluded	absent	41.67	-0.05	included	present	87.06	1.14	excluded	absent
1561564_at	included	present	233.6	0.41	included	present	121.66	-0.85	included	present	224.41	0.31	included	present
1561565_at	excluded	absent	2.79	-0.72	excluded	absent	2.34	-0.75	excluded	absent	2.99	-0.71	excluded	absent
1561566_at	excluded	absent	32.59	0.36	excluded	absent	28.35	0.22	excluded	absent	1.81	-0.67	excluded	absent
1561567_at	excluded	absent	2.61	-1.48	excluded	absent	48.5	-0.39	excluded	absent	14.6	-1.19	excluded	absent
1561571_at	excluded	absent	6.64	-1.04	excluded	absent	16.68	-0.82	excluded	absent	11.54	-0.94	excluded	absent
1561572_at	excluded	absent	2.75	-0.97	excluded	absent	37.15	-0.02	excluded	absent	2.73	-0.97	excluded	absent
1561573_at	excluded	absent	11.92	-0.83	excluded	absent	1.93	-1.08	excluded	absent	25.33	-0.49	excluded	absent
1561574_at	included	present	312.8	0.64	included	present	205.75	0.01	included	present	541.08	2	included	absent
1561575_at	excluded	absent	12.66	-0.39	excluded	absent	2.11	-0.73	excluded	absent	21.87	-0.1	excluded	absent
1561576_a_a	excluded	absent	9.42	0.03	excluded	absent	1.94	-0.44	excluded	absent	1.36	-0.48	excluded	absent
1561577_at	excluded	absent	5.2	-1.08	excluded	absent	52.71	0.5	excluded	absent	29.9	-0.26	excluded	absent
1561578_s_a	excluded	absent	78.29	-0.3	excluded	absent	80.43	-0.27	included	present	149.04	0.76	included	present
1561579_at	excluded	absent	41.86	-0.02	excluded	absent	22.11	-0.54	excluded	absent	3.27	-1.04	excluded	absent
1561581_at	excluded	absent	54.93	0.3	excluded	absent	39.82	-0.14	excluded	absent	2.03	-1.23	excluded	absent
1561584_at	excluded	absent	37.45	0.42	excluded	absent	46.62	0.72	excluded	absent	85.33	1.97	excluded	absent
1561585_at	excluded	absent	7.27	-0.22	excluded	absent	3.11	-0.52	excluded	absent	3.14	-0.52	excluded	absent
1561587_at	excluded	absent	9.96	-0.85	excluded	absent	3.46	-0.99	excluded	absent	72.29	0.43	excluded	absent
1561589_a_a	included	present	220.09	0.94	included	absent	49.87	-1.42	included	absent	118.25	-0.47	included	present
1561590_a_a	excluded	absent	38.85	-0.41	excluded	absent	15.38	-0.73	excluded	absent	4.2	-0.88	excluded	absent
1561591_at	included	present	76.8	-0.32	excluded	absent	21.76	-1.28	excluded	absent	49.39	-0.8	excluded	absent
1561592_at	excluded	absent	82.95	0.71	excluded	absent	15.31	-0.9	excluded	absent	3.02	-1.2	excluded	absent
1561593_at	excluded	absent	6.65	-0.41	excluded	absent	39.95	1.21	excluded	absent	2.47	-0.62	excluded	absent
1561595_x_a	included	present	151.01	-0.24	included	present	307.18	1.58	included	absent	41.38	-1.52	included	present
1561596_at	included	present	240.82	-0.28	included	present	183.8	-0.64	included	present	568.8	1.8	included	present
1561597_x_a	included	present	272.65	-0.37	included	present	287.52	-0.3	included	present	334.83	-0.06	included	present
1561598_at	excluded	absent	121.71	3.28	excluded	absent	57.55	1.07	excluded	absent	14.8	-0.4	excluded	absent
1561599_at	excluded	absent	55.28	-0.3	excluded	absent	40.55	-0.69	included	present	72.89	0.16	included	present
1561600_at	excluded	absent	104.56	0.21	excluded	absent	93.09	0.01	excluded	absent	70.46	-0.39	excluded	absent
1561602_at	excluded	absent	70.15	0.17	excluded	absent	21.03	-0.88	excluded	absent	3.09	-1.26	excluded	absent
1561604_at	excluded	absent	92.68	0.04	included	present	237.83	2.77	included	present	116.97	0.5	included	present
1561605_at	excluded	absent	2.72	-0.41	excluded	absent	32.65	1.25	excluded	absent	3.27	-0.38	excluded	absent
1561606_at	excluded	absent	23.41	-0.18	excluded	absent	2.7	-0.76	excluded	absent	74.83	1.26	excluded	absent
1561607_at	excluded	absent	28.98	0.2	excluded	absent	11.55	-0.4	excluded	absent	2.24	-0.72	excluded	absent
1561608_at	excluded	absent	4.29	-1.14	excluded	absent	82.72	1.2	excluded	absent	40.84	-0.05	excluded	absent
1561609_at	excluded	absent	57.24	0.16	excluded	absent	49.35	-0.06	excluded	absent	29.67	-0.62	excluded	absent
1561610_at	excluded	absent	50.7	0.41	excluded	absent	58.29	0.61	excluded	absent	8.69	-0.69	excluded	absent
1561611_at	excluded	absent	66.69	0.75	excluded	absent	41.47	0.04	excluded	absent	5.09	-0.99	excluded	absent
1561612_at	excluded	absent	4.28	-0.88	excluded	absent	31.3	0	excluded	absent	2.77	-0.93	excluded	absent
1561613_at	excluded	absent	68.82	0.83	excluded	absent	79.54	1.12	excluded	absent	3.82	-0.88	excluded	absent
1561614_at	excluded	absent	3.12	-1.11	included	present	115.21	0.51	included	present	158.64	1.14	included	present
1561615_s_a	included	present	1127.97	-0.75	included	present	1570.51	-0.64	included	present	3709.86	-0.08	included	present

1562229_at	excluded	absent	4.25	-1.1	excluded	absent	51.61	-0.06	excluded	absent	5.16	[illegible]
1562230_at	excluded	absent	114.08	1.04	excluded	absent	54.73	-0.28	included	present	77.58	[illegible]
1562231_at	excluded	absent	17.45	-0.68	excluded	absent	99.01	1.23	excluded	absent	31.06	[illegible]
1562232_at	excluded	absent	52.48	-0.23	excluded	absent	60.48	-0.1	excluded	absent	3.58	[illegible]
1562234_a_a	included	present	750.29	0.47	included	present	346.43	-0.33	included	absent	35.75	[illegible]
1562235_s_a	included	present	105.73	-0.69	included	present	112.58	-0.68	included	present	153.75	[illegible]
1562236_at	excluded	absent	35.26	-0.69	excluded	absent	26.87	-0.86	excluded	absent	63.69	[illegible]
1562238_at	excluded	absent	38.05	-0.25	excluded	absent	51.82	0.05	excluded	absent	115.91	[illegible]
1562239_at	included	present	114.34	1.42	excluded	absent	63.2	0.34	excluded	absent	38.93	[illegible]
1562240_at	excluded	absent	115.52	-0.42	included	present	179.87	0	excluded	absent	138.37	[illegible]
1562244_at	excluded	absent	5.68	-0.87	excluded	absent	98.63	2.1	excluded	absent	40.61	[illegible]
1562245_a_a	excluded	absent	41.37	-0.03	excluded	absent	46.97	0.11	excluded	absent	81.74	[illegible]
1562247_at	excluded	absent	51.2	0.89	excluded	absent	3.15	-0.48	excluded	absent	1.56	[illegible]
1562249_at	excluded	absent	43.13	-0.88	excluded	absent	3.76	-1.27	excluded	absent	2.25	[illegible]
1562250_at	excluded	absent	73.4	0.1	excluded	absent	2.59	-0.91	excluded	absent	49.31	[illegible]
1562251_a_a	excluded	absent	2.02	-0.64	excluded	absent	4.42	-0.51	excluded	absent	9.36	[illegible]
1562253_at	excluded	absent	1.8	-0.97	excluded	absent	14.92	-0.81	excluded	absent	4.13	[illegible]
1562254_at	included	present	301.51	-0.04	included	absent	103.77	-1.79	included	present	266.35	[illegible]
1562255_at	excluded	absent	5.35	-0.85	excluded	absent	9.7	-0.8	excluded	absent	43.07	[illegible]
1562256_at	included	present	69.56	0.35	excluded	absent	3.77	-1.27	included	present	67.48	[illegible]
1562257_x_a	excluded	absent	65.46	0.17	excluded	absent	26.67	-0.83	excluded	absent	8.07	[illegible]
1562259_at	excluded	absent	75.72	0.04	excluded	absent	124.1	0.99	excluded	absent	35.57	[illegible]
1562260_at	excluded	absent	8.19	-1.45	excluded	absent	74.52	0.03	excluded	absent	57.2	[illegible]
1562261_at	excluded	absent	82.97	0.54	excluded	absent	16.81	-0.75	excluded	absent	23.28	[illegible]
1562263_at	excluded	absent	80	0.41	excluded	absent	5.96	-1.29	excluded	absent	38.46	[illegible]
1562264_at	excluded	absent	16.2	-0.63	excluded	absent	72.02	1.28	excluded	absent	3.96	[illegible]
1562265_at	excluded	absent	2.79	-0.66	excluded	absent	102.83	1.64	excluded	absent	7.1	[illegible]
1562267_s_a	excluded	absent	75.38	-0.49	excluded	absent	67.59	-0.65	excluded	absent	69.04	[illegible]
1562269_at	excluded	absent	3.48	-0.55	excluded	absent	2.03	-0.56	excluded	absent	32.6	[illegible]
1562270_at	excluded	absent	172.23	0.32	excluded	absent	152.06	0.14	excluded	absent	75.31	[illegible]
1562271_x_a	included	present	272.05	0.02	included	present	258.08	-0.08	excluded	absent	230.29	[illegible]
1562272_at	excluded	absent	2.51	-0.66	excluded	absent	8.84	-0.36	excluded	absent	2.59	[illegible]
1562273_at	excluded	absent	40.88	-0.31	excluded	absent	41.46	-0.3	excluded	absent	3.91	[illegible]
1562274_at	excluded	absent	3.15	-0.72	excluded	absent	106.69	3.13	excluded	absent	56.9	[illegible]
[illegible]	[illegible]	[illegible]	[illegible]	[illegible]	[illegible]	[illegible]	[illegible]	[illegible]	[illegible]	[illegible]	[illegible]	[illegible]

1562792_at	included	present	334.07	0.24	included	present	241.67	-0.72	included	present	173.37	-1.43	included	present	214.39	-1.01	included	present	281.71	-0.31	included	absent	191.4	-1.24
1562793_at	excluded	absent	32.27	0.4	excluded	absent	4.92	-0.53	excluded	absent	16.9	-0.12	excluded	absent	1.47	-0.65	excluded	absent	1.74	-0.64	excluded	absent	2.82	-0.6
1562794_at	excluded	absent	5.89	-1.07	excluded	absent	33.39	-0.61	excluded	absent	14.08	-0.93	excluded	absent	107.43	0.62	excluded	absent	67.88	-0.04	excluded	absent	67.49	-0.04
1562797_at	excluded	absent	46.17	0.57	excluded	absent	32.61	0.13	excluded	absent	59.23	1	excluded	absent	1.33	-0.89	excluded	absent	8.79	-0.65	excluded	absent	9.21	-0.64
1562799_at	excluded	absent	38.91	-0.56	excluded	absent	41.61	-0.5	excluded	absent	44.35	-0.44	excluded	absent	52.9	-0.26	excluded	absent	36.18	-0.61	excluded	absent	88.83	0.49
1562800_at	excluded	absent	4.06	-0.61	excluded	absent	84.7	2.67	excluded	absent	16.14	-0.12	excluded	absent	7.21	-0.48	excluded	absent	48.66	1.21	excluded	absent	88.71	2.83
1562801_at	excluded	absent	31.73	0.42	excluded	absent	5.33	-0.62	excluded	absent	49.09	1.11	excluded	absent	23.42	0.1	excluded	absent	102.37	3.21	excluded	absent	46.83	1.02
1562802_at	excluded	absent	3.65	-0.65	excluded	absent	6.27	-0.5	excluded	absent	76.63	3.54	excluded	absent	39.85	1.43	excluded	absent	25.13	0.58	excluded	absent	9.96	-0.29
1562803_at	excluded	absent	7.69	-0.56	excluded	absent	41.86	0.67	excluded	absent	78.55	1.98	excluded	absent	29.48	0.23	excluded	absent	42.04	0.68	excluded	absent	70.65	1.7
1562805_at	excluded	absent	5.46	-0.77	excluded	absent	37.82	0.29	excluded	absent	1.72	-0.89	excluded	absent	2.73	-0.86	excluded	absent	7.57	-0.7	excluded	absent	45.1	0.53
1562810_at	excluded	absent	16.53	-0.72	included	present	371.46	7.38	excluded	absent	49.1	0.02	excluded	absent	2.68	-1.04	included	present	721.72	15.39	excluded	absent	143.69	2.18
1562811_at	excluded	absent	40.01	-0.04	excluded	absent	35.44	-0.18	excluded	absent	60.68	0.59	excluded	absent	46.73	0.17	excluded	absent	3.53	-1.16	excluded	absent	5.8	-1.09
1562812_at	excluded	absent	12.64	-1.2	excluded	absent	54.2	-0.16	excluded	absent	63.93	0.08	included	present	68.07	0.19	excluded	absent	31.72	-0.72	excluded	absent	20.93	-1
1562815_at	included	present	255.12	-0.3	included	present	217.32	-0.35	included	absent	146.61	-0.42	included	present	712.08	0.21	included	present	299.75	-0.25	included	present	93.83	-0.48
1562817_at	excluded	absent	85.57	0.29	excluded	absent	49.06	-0.33	excluded	absent	86.38	0.31	excluded	absent	79.33	0.19	excluded	absent	192.26	2.11	excluded	absent	85.32	0.29
1562818_at	excluded	absent	138.67	2.35	excluded	absent	40.92	-0.04	excluded	absent	13.64	-0.7	excluded	absent	60.23	0.43	excluded	absent	39.67	-0.07	excluded	absent	21.46	-0.51
1562820_at	excluded	absent	36.55	-0.07	excluded	absent	54.95	0.16	included	present	1190.47	13.94	excluded	absent	1.17	-0.5	excluded	absent	3.66	-0.47	excluded	absent	63.94	0.27
1562821_a_a	excluded	absent	14.58	-0.87	excluded	absent	11.46	-0.96	excluded	absent	7.41	-1.09	excluded	absent	1.49	-1.27	excluded	absent	4.25	-1.18	excluded	absent	24.27	-0.58
1562822_at	excluded	absent	70.33	-0.18	excluded	absent	4.79	-1.09	excluded	absent	60.13	-0.32	excluded	absent	2.41	-1.12	excluded	absent	117.02	0.46	included	present	96.14	0.18
1562823_at	included	present	119.78	1.44	excluded	absent	42.51	-0.14	excluded	absent	2.92	-0.95	excluded	absent	17.75	-0.65	excluded	absent	88.68	0.8	excluded	absent	10.37	-0.8
1562825_at	included	present	70.16	-0.89	excluded	absent	141.72	0.61	excluded	absent	111.35	-0.03	excluded	absent	71.75	-0.86	excluded	absent	177.01	1.34	excluded	absent	61.44	-1.07
1562826_at	excluded	absent	55.47	-0.01	excluded	absent	5.34	-1.04	excluded	absent	22.85	-0.68	excluded	absent	28.73	-0.56	excluded	absent	161.08	2.15	excluded	absent	7.94	-0.99
1562827_at	excluded	absent	38.93	-0.51	included	present	107.71	1.12	excluded	absent	135.63	1.79	excluded	absent	2.16	-1.39	excluded	absent	110.9	1.2	excluded	absent	90.01	0.7
1562828_at	excluded	absent	53.33	-0.52	excluded	absent	101.91	0.04	excluded	absent	66.13	-0.37	included	present	97.96	-0.01	included	present	386.31	3.29	excluded	absent	46.71	-0.59
1562829_at	excluded	absent	68.73	1.83	excluded	absent	64.56	1.66	excluded	absent	2.08	-0.86	excluded	absent	1.29	-0.89	excluded	absent	3.2	-0.81	excluded	absent	55.92	1.31
1562830_at	included	present	279.85	0.25	included	absent	86.72	-1.46	included	present	216.84	-0.31	included	present	238.84	-0.11	included	present	284.96	0.3	included	present	252.16	0.01
1562831_a_a	excluded	absent	73.54	-0.38	excluded	absent	78.33	-0.33	excluded	absent	87.67	-0.23	excluded	absent	67.22	-0.45	excluded	absent	33.58	-0.81	excluded	absent	131.45	0.25
1562832_at	excluded	absent	14.33	-0.6	excluded	absent	8.01	-0.72	excluded	absent	10.26	-0.68	excluded	absent	11.66	-0.65	excluded	absent	6.5	-0.75	excluded	absent	2.74	-0.82
1562834_at	included	present	82.66	1.11	included	present	90.27	1.29	excluded	absent	61.69	0.59	excluded	absent	3.77	-0.84	excluded	absent	111.13	1.81	excluded	absent	4.16	-0.83
1562835_at	excluded	absent	70	-0.3	excluded	absent	38.88	-1.07	excluded	absent	76.27	-0.15	excluded	absent	33.28	-1.21	excluded	absent	32.13	-1.23	excluded	absent	2.27	-1.97
1562836_at	included	present	1360.08	1.38	included	present	626.96	-1.25	included	present	3098.58	7.59	included	present	2759.21	6.38	included	present	637.98	-1.21	included	present	1149.19	0.62
1562839_at	excluded	absent	112.51	0.64	excluded	absent	0.82	-2.07	excluded	absent	37.21	-1.18	excluded	absent	38.37	-1.16	excluded	absent	28.25	-1.4	included	present	114.27	0.69
1562841_at	excluded	absent	36.31	-0.27	excluded	absent	6.83	-1.04	excluded	absent	92.05	1.2	excluded	absent	17.56	-0.76	excluded	absent	32.84	-0.36	excluded	absent	62.33	0.42
1562844_at	excluded	absent	3.53	-0.57	excluded	absent	2.47	-0.6	excluded	absent	36.92	0.28	excluded	absent	3.94	-0.56	excluded	absent	5.21	-0.53	excluded	absent	49.73	0.61
1562845_at	excluded	absent	44.83	-0.19	excluded	absent	78.7	0.42	excluded	absent	16.29	-0.69	excluded	absent	160.27	1.86	excluded	absent	62.81	0.13	excluded	absent	154.88	1.77
1562847_at	excluded	absent	2.41	-0.66	excluded	absent	2.85	-0.64	excluded	absent	2.76	-0.65	excluded	absent	4.14	-0.61	excluded	absent	3.08	-0.64	excluded	absent	62.87	1.03
1562848_at	included	present	256.72	-0.55	included	present	179.75	-1.37	included	present	481.4	1.83	included	present	211.05	-1.04	included	absent	195.94	-1.2	included	present	214.07	-1.01
1562849_at	included	present	118.08	0.4	included	present	200.4	1.69	excluded	absent	74.4	-0.29	excluded	absent	41.19	-0.81	excluded	absent	248.1	2.44	excluded	absent	225.98	2.09
1562850_at	excluded	absent	14.18	-0.27	excluded	absent	2	-0.67	excluded	absent	25.8	0.1	excluded	absent	8.76	-0.45	excluded	absent	8.77	-0.45	excluded	absent	30.41	0.25
1562852_at	included	present	218.36	0.99	excluded	absent	116.79	-0.2	excluded	absent	234.36	1.18	excluded	absent	122.68	-0.13	excluded	absent	118.99	-0.17	excluded	absent	11.27	-1.43
1562853_x_a	included	present	263.91	2.1	excluded	absent	120.23	0	excluded	absent	134.47	0.21	excluded	absent	116.05	-0.06	included	present	251.4	1.92	excluded	absent	44.73	-1.11
1562854_at	excluded	absent	33.34	0.06	excluded	absent	15.28	-0.55	excluded	absent	16.15	-0.52	excluded	absent	49.45	0.6	excluded	absent	79.69	1.62	excluded	absent	1.15	-1.02
1562856_at	excluded	absent	3.2	-0.65	excluded	absent	3.29	-0.64	excluded	absent	5.99	-0.46	excluded	absent	2.5	-0.69	excluded	absent	6.75	-0.41	excluded	absent	6.67	-0.42
1562857_at	excluded	absent	1.97	-0.81	excluded	absent	7.26	-0.66	excluded	absent	64.18	0.95	excluded	absent	3.64	-0.76	excluded	absent	2.64	-0.79	excluded	absent	57.39	0.76
1562860_at	excluded	absent	1.44	-0.64	excluded	absent	1.4	-0.65	excluded	absent	6	-0.44	excluded	absent	37.54	0.96	excluded	absent	29.38	0.59	excluded	absent	1.49	-0.64
1562861_at	excluded	absent	2.89	-1.14	excluded	absent	32.56	-0.55	excluded	absent	28.1	-0.64	excluded	absent	39.9	-0.4	excluded	absent	119.89	1.2	excluded	absent	4.05	-1.12
1562862_at	excluded	absent	51.84	0.09	excluded	absent	81.98	0.75	excluded	absent	2.42	-1	excluded	absent	94.24	1.02	excluded	absent	65.73	0.39	excluded	absent	19.17	-0.63
1562863_at	excluded	absent	67.9	0.15	excluded	absent	1.87	-1.23	included	present	84.14	0.5	excluded	absent	44.27	-0.34	excluded	absent	44.69	-0.33	excluded	absent	40.82	-0.41
1562864_at	excluded	absent	4.12	-0.42	excluded	absent	14.2	-0.37	excluded	absent	2.57	-0.43	excluded	absent	39.83	-0.24	excluded	absent	161	0.38	excluded	absent	8.42	-0.4
1562865_at	excluded	absent	101.82	0.83	excluded	absent	104.35	0.88	included	present	169.58	2.19	excluded	absent	69.56	0.18	excluded	absent	165.08	2.1	excluded	absent	70.81	0.21
1562866_at	excluded	absent	4.28	-1.01	excluded	absent	12.56	-0.74	excluded	absent	2.03	-1.09	excluded	absent	30.11	-0.16	excluded	absent	68.3	1.11	excluded	absent	1.22	-1.11
1562869_at	excluded	absent	53.33	-0.03	excluded	absent	56.27	0.06	excluded	absent	80.53	0.81	excluded	absent	35.04	-0.59	excluded	absent	54.82	0.02	excluded	absent	44.78	-0.29
1562870_at	excluded	absent	38.47	-0.24	excluded	absent	10.79	-0.98	excluded	absent	3.82	-1.17	excluded	absent	28.12	-0.52	excluded	absent	67.28	0.53	excluded	absent	7.22	-1.08
1562871_at	excluded	absent	1.91	-0.78	excluded	absent	9.85	-0.69	excluded	absent	65.5	-0.06	excluded	absent	31.45	-0.44	excluded	absent	5.6	-0.74	excluded	absent	42.76	-0.32
1562873_at	excluded	absent	4.38	-0.94	excluded	absent	43.6	-0.62	excluded	absent	24	-0.78	excluded	absent	67.94	-0.41	excluded	absent	39.93	-0.65	excluded	absent	2.17	-0.96
1562876_s_a	included	present	87.02	1.4	excluded	absent	64.25	0.69	excluded	absent	28.52	-0.41	excluded	absent	56.83	0.46	excluded	absent	220.01	5.52	excluded	absent	132.97	2.82
1562878_at	included	present	89.94	-0.33	excluded	absent	71.55	-0.63	excluded	absent	112.7	0.03	excluded	absent	63.46	-0.75	excluded	absent	114.15	0.05	included	present	141.74	0.49
1562879_at	excluded	absent	5.24	-0.79	excluded	absent	78.95	0.12	excluded	absent	2.96	-0.82	excluded	absent	53.75	-0.19	excluded	absent	40.86	-0.35	excluded	absent	4.01	-0.81
1562880_at	excluded	absent	4.45	-0.85	excluded	absent	54.38	-0.15	excluded	absent	24.05	-0.58	excluded	absent	3.17	-0.87	excluded	absent	3.76	-0.86	excluded	absent	15.62	-0.69
1562885_at	excluded	absent	75.79	0.55	excluded	absent	149.07	2.23	excluded	absent	18.85	-0.75	excluded	absent	5.24	-1.06	excluded	absent	82.68	0.71	excluded	absent	72.95	0.49
1562886_at	excluded	absent	25.01	-0.2	excluded	absent	62.6	0.95	excluded	absent	2.67	-0.89	excluded	absent	6.23	-0.78	excluded	absent	2.62	-0.89	excluded	absent	3.07	-0.87
1562888_at	excluded	absent	25.16	-0.6	excluded	absent	12.61	-0.97	included	present	79.06	1.01	excluded	absent	2.69	-1.27	excluded	absent	8.64	-1.09	excluded	absent	83.6	1.15
1562890_at	excluded	absent	47.97	0	excluded	absent	4.42	-1.12	excluded	absent	20.39	-0.71	excluded	absent	4.68	-1.11	excluded	absent	13.04	-0.9	excluded	absent	8	-1.02
1562891_at	excluded	absent	1.85	-0.85	excluded	absent	2.44	-0.83	excluded	absent	7.59	-0.69	excluded	absent	1.85	-0.85	excluded	absent	3.46	-0.8	excluded	absent	5.12	-0.76
1562892_at	included	present	117.24	1.81	excluded	absent	40.39	-0.29	included	present	126.59	2.06	excluded	absent	87.18	0.99	excluded	absent	112.26	1.67	excluded	absent	144	2.54
1562893_at	excluded	absent	16.29	-1.08	excluded	absent	41.54	-0.45	excluded	absent	45.09	-0.36	excluded	absent	26.27	-0.83	excluded	absent	85.59	0.66	excluded	absent	84.87	0.64
1562894_at	included	present	209.77	0.15	excluded	absent	7.02	-1.3	excluded	absent	48.28	-1.01	excluded	absent	4.79	-1.32	excluded	absent	128.45	-0.43	included	present	253.35	0.46
1562895_at	excluded	absent	54.38	1.15	excluded	absent	39.54	0.56	excluded	absent	5.5	-0.79	excluded	absent	41.54	0.64	excluded	absent	2.93	-0.89	excluded	absent	6.44	-0.75
1562896_at	excluded	absent	104.75	0.11	excluded	absent	90.56	-0.11	excluded	absent	63.58	-0.51	excluded	absent	142.92	0.68	excluded	absent	115.35	0.27	excluded	absent	165.06	1.02
1562898_at	excluded	absent	142.17	-0.22	excluded	absent	6.88	-1.69	excluded	absent	7.08	-1.69	included	present	254.06	1	excluded	absent	19.26	-1.56	excluded	absent	182.81	0.22
1562899_at	included	present	114.99	-1.03	included	absent	79	-1.45	included	present	124.46	-0.92	included	absent	89.31	-1.33	included	absent	123.83	-0.93	included	absent	120.62	-0.96
1562901_at	included	present	378.55	5.21	excluded	absent	55.92	-0.34	excluded	absent	3.04	-1.25	excluded	absent	52.78	-0.39	excluded	absent	61.12	-0.25	included	present	279.64	3.51
1562902_at	excluded	absent	33.46	0.63	excluded	absent	1.37	-0.63	excluded	absent	4.73	-0.5	excluded	absent	1.41	-0.62	excluded	absent	13.15	-0.17	excluded	absent	9.64	-0.3
1562903_at	excluded	absent	38.3	0.04	excluded	absent	7.43	-0.79	excluded	absent	54.25	0.46	excluded	absent	41.92	0.13	excluded	absent	8.94	-0.75	excluded	absent	39.19	0.06
1562904_s_a	excluded	absent	84.21	-0.15	excluded	absent	57.8	-0.62	excluded	absent	85.24	-0.13	excluded	absent	72.01	-0.37	excluded	absent	88.78	-0.07	excluded	absent	57.54	-0.63
1562905_at	excluded	absent	5.05	-1.2	excluded	absent	167.76	-0.13	included	present	231.24	0.29	excluded	absent	16.85	-1.12	excluded	absent	134.25	-0.35	excluded	absent	60.98	-0.83
1562906_at	excluded	absent	5.29	-0.26	excluded	absent	4.8	-0.26	excluded	absent	76.9	0.18	excluded	absent	2.53	-0.28	excluded	absent	7.54	-0.25	excluded	absent	9.08	-0.24
1562907_at	excluded	absent	48.32	0.69	excluded	absent	58.17	1.02	excluded	absent	3.48	-0.85	excluded	absent	27.95	-0.01	excluded	absent	21.62	-0.23	excluded	absent	6.57	-0.74
1562908_at	excluded	absent	64.57	0.52	excluded	absent	40.14	-0.05	excluded	absent	4.24	-0.88	excluded	absent	32.99	-0.21	excluded	absent	29.45	-0.3	excluded	absent	13.7	-0.66
1562909_at	excluded	absent	59.08	-0.04	excluded	absent	77.26	0.28	included	present	133.69	1.27	excluded	absent	29.34	-0.57	excluded	absent	126.29	1.14	excluded	absent	42.46	-0.34
1562910_at	excluded	absent	46.87	-0.87	excluded	absent	175.75	1.37	excluded	absent	115.36	0.32	excluded	absent	3.53	-1.62	excluded	absent	134.95	0.66	included	present	199.49	1.78
1562914_a_a	excluded	absent	43.32	0.06	excluded	absent	5.04	-0.5	excluded	absent	3.15	-0.53	excluded	absent	5.76	-0.49	excluded	absent	6.95	-0.47	excluded	absent	46.34	0.11
1562915_at	excluded	absent	4.91	-0.65	excluded	absent	41.57	0.34	excluded	absent	23.02	-0.16	excluded	absent	1.79	-0.73	excluded	absent	5.39	-0.64	excluded	absent	8.39	-0.55
1562916_at	excluded	absent	121.09	2.03	excluded	absent	5.4	-1.2	excluded	absent	38.43	-0.28	excluded	absent	51.27	0.08	excluded	absent	61.55	0.37	excluded	absent	70.07	0.61
1562918_at	excluded	absent	5.03	-0.95	excluded	absent	39.51	0.2	excluded	absent	1.85	-1.06	excluded	absent	52.72	0.64	excluded	absent	32.27	-0.04	excluded	absent	4.91	-0.96
1562919_at	excluded	absent	28.59	0.64	excluded	absent	41.6	1.33	excluded	absent	5.85	-0.56	excluded	absent	66.94	2.67	excluded	absent	15.28	-0.06	excluded	absent	17.64	0.06
1562920_at	excluded	absent	68.83	1.02	excluded	absent	4.65	-0.93	excluded	absent	32.95	-0.07	excluded	absent	3.63	-0.96	excluded	absent	0.63	-1.05	excluded	absent	4.91	-0.92
1562921_at	included	present	71.19	-0.57	included	present	471.57	0.2	included	present	231.37	-0.26	included	present	677.72	0.6	included	absent	140.85	-0.44	included	present	191.11	-0.34
1562922_at	excluded	absent	3.19	-0.65	excluded	absent	4.64	-0.64	excluded	absent	11.25	-0.56	excluded	absent	6.85	-0.61	excluded	absent	79.25	0.23	excluded	absent	66.82	0.08
1562923_at	excluded	absent	0.86	-0.88	excluded	absent	2.72	-0.82	excluded	absent	2.15	-0.84	excluded	absent	36.07	0.27	excluded	absent	74.46	1.52	excluded	absent	2.22	-0.83
1562924_at	excluded	absent	69.3	0.69	excluded	absent	5.29	-0.9	excluded	absent	3.45	-0.95	excluded	absent	28.21	-0.33	excluded	absent	4.22	-0.93	excluded	absent	17.2	-0.61
1562925_at	included	present	302.98	0.03	included	present	874.79	3.41	included	present	526.94	1.35	included	present	115.52	-1.07	included	present	1613.1	7.76	included	present	134.17	-0.96
1562926_at	excluded	absent	76.05	-0.16	excluded	absent	77.02	-0.15	excluded	absent	81.78	-0.1	excluded	absent	54.92	-0.4	excluded	absent	85.19	-0.06	excluded	absent	89.6	-0.01
1562927_at	excluded	absent	44.95	-0.45	excluded	absent	48.69	-0.39	excluded	absent	70.02	0	excluded	absent	6.42	-1.15	excluded	absent	118.37	0.88	excluded	absent	49.68	-0.37
1562928_at	excluded	absent	5.72	-0.84	excluded	absent	3.64	-0.92	excluded	absent	2.55	-0.95	excluded	absent	4.15	-0.9	excluded	absent	5.41	-0.85	excluded	absent	7.8	-0.77
1562930_at	included	present	38.61	-0.08	excluded	absent	68.92	0.55	excluded	absent	46.79	0.09	excluded	absent	25.22	-0.36	excluded	absent	82.15	0.82	excluded	absent	4.82	-0.78
1562932_at	excluded	absent	3.96	-0.55	excluded	absent	2.42	-0.62	excluded	absent	1.48	-0.66	excluded	absent	2.32	-0.62	excluded	absent	2.91	-0.6	excluded	absent	6.29	-0.45
1562933_at	excluded	absent	36.91	0.36	excluded	absent	2.99	-0.74	excluded	absent	27.64	0.06	excluded	absent	21.2	-0.15	excluded	absent	11.54	-0.46	excluded	absent	60.4	1.12
1562934_at	excluded	absent	3	-0.73	excluded	absent	8.1	-0.51	excluded	absent	4.73	-0.66	excluded	absent	1.64	-0.79	excluded	absent	4.81	-0.65	excluded	absent	46.67	1.19
1562935_at	included	present	69.42	-0.76	excluded	absent	66.45	-0.81	included	present	75.91	-0.64	included	present	84.07	-0.48	excluded	absent	11.74	-1.83	excluded	absent	54.82	-1.03
1562937_at	included	present	109.07	1.14	excluded	absent	52.46	-0.22	excluded	absent	7.93	-1.29	excluded	absent	43.09	-0.45	excluded	absent	133.33	1.72	included	present	90.19	0.68
1562938_at	included	absent	86.43	-0.59	included	present	86.71	-0.59	included	present	198.93	0.01	included	absent	81.89	-0.61	included	absent	6.29	-1.02	included	absent	51.65	-0.77

1563376_at	excluded	absent	56.48	1.33	excluded	absent	3.68	-0.69	excluded	absent	42.48	0.8	excluded	absent	22.11	0.01	excluded	absent	26.64	0.19	excluded	absent	78.81	2.19	excluded	absent	3.31	-0.71	excluded	absent	1.86	-0.76	excluded	absent	22.69	0.04	excluded	absent	6.56	-0.58	excluded	absent	4.41	-0.66	excluded	absent	20.31	-0.05	excluded	absent	28.34	0.25	excluded	absent	38.27	0.63	excluded	absent	0.65	-0.81
1563379_at	excluded	absent	70.67	-0.49	excluded	absent	66.61	-0.57	included	present	144.15	0.9	excluded	absent	73.12	-0.44	included	present	148.74	0.99	excluded	absent	7.36	-1.68	excluded	absent	80.59	-0.3	excluded	absent	41.15	-1.05	included	present	149.59	1	included	present	121.76	0.48	included	present	319.18	4.2	included	present	86.24	-0.19	included	present	106.54	0.19	excluded	absent	2.22	-1.78	excluded	absent	37.77	-1.11
1563385_at	excluded	absent	3.51	-0.77	excluded	absent	53.08	1.36	excluded	absent	55.2	1.45	excluded	absent	3.11	-0.79	excluded	absent	11.72	-0.42	excluded	absent	2.13	-0.83	excluded	absent	42.01	0.89	excluded	absent	2.18	-0.83	excluded	absent	4.16	-0.74	excluded	absent	13.12	-0.36	excluded	absent	29.39	0.34	excluded	absent	30.1	0.37	excluded	absent	2	-0.83	excluded	absent	4.59	-0.72	excluded	absent	12.79	-0.37
1563386_at	excluded	absent	85.1	-0.26	excluded	absent	3.08	-0.48	excluded	absent	15.7	-0.44	excluded	absent	33.54	-0.4	excluded	absent	32.46	-0.4	excluded	absent	4.78	-0.47	excluded	absent	2.9	-0.48	excluded	absent	1.01	-0.48	excluded	absent	89.04	-0.25	excluded	absent	3.32	-0.48	excluded	absent	3.51	-0.48	excluded	absent	15.68	-0.44	excluded	absent	50.6	-0.35	excluded	absent	1.96	-0.48	excluded	absent	0.91	-0.48
1563389_at	included	present	594.58	0.87	included	present	488.66	0.32	included	present	802.28	1.93	included	present	444.8	0.1	included	present	511.87	0.44	included	present	417.84	-0.04	included	present	333.49	-0.47	included	present	366.26	-0.3	included	present	362.71	-0.32	included	present	481.04	0.28	included	present	500.8	0.39	included	present	171.78	-1.3	included	absent	99.03	-1.67	included	present	261.76	-0.84	included	present	443.95	0.09
1563391_at	excluded	absent	10.1	-0.4	excluded	absent	9.68	-0.42	excluded	absent	8.18	-0.48	excluded	absent	5.72	-0.59	excluded	absent	12.72	-0.29	excluded	absent	8.09	-0.49	excluded	absent	3.7	-0.68	excluded	absent	2.03	-0.75	excluded	absent	5.34	-0.61	excluded	absent	1.91	-0.76	excluded	absent	6.06	-0.58	excluded	absent	2.43	-0.73	excluded	absent	2	-0.75	excluded	absent	5.43	-0.6	excluded	absent	1.98	-0.75
1563392_at	excluded	absent	68.53	-0.19	excluded	absent	5.27	-1.13	excluded	absent	6.7	-1.11	excluded	absent	5.11	-1.13	excluded	absent	66.7	-0.22	included	present	128.87	0.7	excluded	absent	2.88	-1.16	excluded	absent	3.41	-1.16	included	present	131.18	0.73	excluded	absent	47.61	-0.5	excluded	absent	64.39	-0.26	included	present	119.55	0.56	excluded	absent	27.28	-0.8	excluded	absent	29.34	-0.77	excluded	absent	6.71	-1.11
1563396_x_a	excluded	absent	63.39	1.77	excluded	absent	34.19	0.52	excluded	absent	0.78	-0.91	excluded	absent	37.39	0.66	excluded	absent	2.73	-0.82	excluded	absent	1.63	-0.87	excluded	absent	4.26	-0.76	excluded	absent	2.13	-0.85	excluded	absent	21.43	-0.02	excluded	absent	5.3	-0.71	included	present	57.31	1.51	excluded	absent	39.23	0.74	excluded	absent	23.51	0.06	excluded	absent	13.24	-0.37	excluded	absent	0.56	-0.92
1563397_at	excluded	absent	3.37	-0.59	excluded	absent	5.73	-0.48	excluded	absent	24.14	0.41	excluded	absent	3	-0.61	excluded	absent	22.96	0.35	excluded	absent	2.67	-0.62	excluded	absent	7.4	-0.4	excluded	absent	3.89	-0.56	excluded	absent	7.53	-0.39	excluded	absent	36.94	1.02	excluded	absent	8.96	-0.32	excluded	absent	4.21	-0.55	excluded	absent	46.28	1.47	excluded	absent	2.78	-0.62	excluded	absent	33.14	0.84
1563398_at	excluded	absent	98.09	0.68	excluded	absent	98.92	0.7	excluded	absent	94.41	0.6	included	present	73.68	0.13	excluded	absent	2.98	-1.48	excluded	absent	158.69	2.06	excluded	absent	78.51	0.24	excluded	absent	48.82	-0.44	included	present	52.78	-0.35	included	present	80.03	0.27	excluded	absent	15.68	-1.19	excluded	absent	33.99	-0.78	excluded	absent	6.43	-1.4	included	present	39.68	-0.65	excluded	absent	17.91	-1.14
1563404_at	excluded	absent	7.84	-1.24	excluded	absent	124.27	0.97	excluded	absent	4.88	-1.3	excluded	absent	83.3	0.19	excluded	absent	77.02	0.07	excluded	absent	16.88	-1.07	excluded	absent	115.85	0.81	excluded	absent	31.6	-0.79	excluded	absent	167.76	1.8	excluded	absent	21.35	-0.99	excluded	absent	83.73	0.2	excluded	absent	203.16	2.47	excluded	absent	187.1	2.16	excluded	absent	9.81	-1.21	excluded	absent	44.36	-0.55
1563405_at	excluded	absent	4.92	-1.14	excluded	absent	5.33	-1.13	excluded	absent	44.71	-0.25	excluded	absent	11.68	-0.99	excluded	absent	90.04	0.76	excluded	absent	14.76	-0.92	excluded	absent	69.07	0.29	excluded	absent	7.32	-1.09	excluded	absent	102.42	1.04	excluded	absent	11.03	-1	excluded	absent	9.41	-1.04	excluded	absent	13.03	-0.96	excluded	absent	27.35	-0.64	excluded	absent	2.37	-1.2	excluded	absent	42.95	-0.29
1563407_x_a	excluded	absent	3.4	-1.06	excluded	absent	6.31	-0.98	excluded	absent	14.79	-0.75	excluded	absent	3.35	-1.06	excluded	absent	64.82	0.57	excluded	absent	11.62	-0.84	excluded	absent	5.59	-1	excluded	absent	9.92	-0.88	excluded	absent	17.38	-0.69	excluded	absent	8.46	-0.92	excluded	absent	11.9	-0.83	excluded	absent	14.3	-0.77	excluded	absent	6.84	-0.97	excluded	absent	17.32	-0.69	excluded	absent	10.79	-0.86
1563414_at	included	present	232.11	0.51	included	present	643.27	2.43	included	present	455.6	1.55	included	present	719.55	2.79	included	present	983.35	4.02	excluded	absent	40.3	-0.39	included	present	778.06	3.06	included	present	111.34	-0.06	included	present	223.52	0.47	included	present	819.39	3.26	included	present	628.79	2.36	included	present	1118.97	4.66	included	present	2275.71	10.07	included	present	767.89	3.01	included	present	198.89	0.35
1563420_at	excluded	absent	70.17	-0.02	excluded	absent	4.15	-0.51	excluded	absent	2.31	-0.52	excluded	absent	16.36	-0.42	excluded	absent	84.79	0.09	included	present	150.94	0.58	excluded	absent	2.91	-0.52	excluded	absent	39.3	-0.25	excluded	absent	10.05	-0.46	excluded	absent	58.96	-0.1	excluded	absent	19.52	-0.39	excluded	absent	4.44	-0.5	excluded	absent	28.83	-0.32	excluded	absent	12.01	-0.45	excluded	absent	3.82	-0.51
1563425_at	excluded	absent	4.01	-0.82	excluded	absent	10.73	-0.56	excluded	absent	35.08	0.39	excluded	absent	4.55	-0.8	excluded	absent	21.42	-0.14	excluded	absent	8.48	-0.64	excluded	absent	2.24	-0.89	excluded	absent	11.38	-0.53	excluded	absent	30.53	0.21	excluded	absent	44.91	0.77	excluded	absent	16.25	-0.34	excluded	absent	33.59	0.33	excluded	absent	4.87	-0.78	excluded	absent	1.41	-0.92	excluded	absent	30.91	0.23
1563426_a_a	excluded	absent	74.41	-0.52	included	present	366.11	3.61	excluded	absent	68.76	-0.6	included	present	133.49	0.32	excluded	absent	149.54	0.55	excluded	absent	93.77	-0.24	excluded	absent	84.36	-0.38	excluded	absent	73.32	-0.53	excluded	absent	97.01	-0.2	excluded	absent	84.24	-0.38	included	present	182.83	1.02	included	present	248.68	1.95	excluded	absent	11.65	-1.41	included	present	101.61	-0.13	included	present	152.56	0.59
1563427_at	excluded	absent	5.34	-0.83	excluded	absent	2.43	-0.93	excluded	absent	18.13	-0.39	excluded	absent	17.21	-0.42	excluded	absent	41.48	0.42	excluded	absent	80.23	1.76	excluded	absent	28.75	-0.02	excluded	absent	21.29	-0.28	excluded	absent	5.97	-0.81	excluded	absent	1.51	-0.96	excluded	absent	16.33	-0.45	excluded	absent	5.15	-0.84	excluded	absent	33.34	0.14	excluded	absent	1.6	-0.96	excluded	absent	1.27	-0.97
1563431_x_a	included	present	17579.29	-0.58	included	present	15306.31	-0.75	included	present	10444.95	-1.11	included	present	11685.76	-1.02	included	present	13270.41	-0.9	included	present	12258.41	-0.98	included	present	10251.54	-1.12	included	present	11957.84	-1	included	present	10284.35	-1.12	included	present	12859.68	-0.93	included	present	12461.3	-0.96	included	present	30682.15	0.39	included	present	25016.4	-0.03	included	present	23641.22	-0.13	included	present	12667.27	-0.94
1563445_x_a	included	present	202.01	-0.16	included	present	186.6	-0.33	included	present	168.24	-0.54	included	present	158.56	-0.65	excluded	absent	217.85	0.02	included	absent	176.42	-0.45	included	present	130.2	-0.97	included	present	156.91	-0.67	included	present	256.13	0.45	included	present	117.46	-1.11	included	present	256.88	0.46	included	present	302.75	0.97	included	present	122.07	-1.06	included	absent	25.87	-2.14	included	present	161.23	-0.62
1563450_at	excluded	absent	44.18	1.02	excluded	absent	22.37	0.05	excluded	absent	2.98	-0.81	excluded	absent	1.35	-0.88	excluded	absent	57.9	1.63	excluded	absent	1.85	-0.86	excluded	absent	4.29	-0.75	excluded	absent	44.54	1.04	excluded	absent	34.44	0.59	excluded	absent	59.74	1.71	excluded	absent	39.82	0.83	excluded	absent	53.94	1.45	excluded	absent	2.27	-0.84	excluded	absent	1.62	-0.87	excluded	absent	3.01	-0.81
1563452_at	included	present	89.28	0.74	excluded	absent	52.8	0.05	excluded	absent	87.83	0.71	excluded	absent	30.16	-0.38	excluded	absent	77.45	0.52	excluded	absent	44.72	-0.1	excluded	absent	83.32	0.63	excluded	absent	4.18	-0.87	excluded	absent	32.65	-0.33	excluded	absent	27.91	-0.42	included	present	150.74	1.91	excluded	absent	60.2	0.19	excluded	absent	7.06	-0.82	excluded	absent	40.36	-0.19	excluded	absent	8.59	-0.79
1563453_at	excluded	absent	9.1	-0.88	excluded	absent	6.77	-0.91	excluded	absent	10.46	-0.87	excluded	absent	3.36	-0.94	excluded	absent	99.46	0.1	excluded	absent	57.37	-0.36	excluded	absent	16.37	-0.8	excluded	absent	6.71	-0.91	excluded	absent	14.28	-0.82	excluded	absent	47.25	-0.47	excluded	absent	36.02	-0.59	excluded	absent	11.77	-0.85	excluded	absent	84.95	-0.06	excluded	absent	4.88	-0.93	excluded	absent	21.83	-0.74
1563454_at	included	present	185.64	1.94	excluded	absent	69.35	-0.08	excluded	absent	3.47	-1.23	excluded	absent	2.51	-1.25	excluded	absent	22.87	-0.89	excluded	absent	41.14	-0.57	excluded	absent	30.08	-0.77	excluded	absent	34.7	-0.69	included	present	58.04	-0.28	excluded	absent	50.17	-0.42	included	present	260.75	3.25	excluded	absent	48.62	-0.44	excluded	absent	45.07	-0.51	excluded	absent	8.94	-1.14	excluded	absent	1.42	-1.27
1563455_at	included	present	171.49	-0.48	included	absent	2.7	-1.39	included	absent	147.38	-0.61	included	absent	78.07	-0.99	included	absent	126.43	-0.73	included	absent	95.45	-0.89	included	present	97.33	-0.88	included	present	79.06	-0.98	included	absent	85.6	-0.95	included	present	96.4	-0.89	included	present	118.16	-0.77	included	absent	9.62	-1.35	included	absent	4.99	-1.38	included	absent	65.24	-1.05	included	present	54.21	-1.11
1563456_at	excluded	absent	1.59	-1.14	excluded	absent	42.43	0.11	excluded	absent	42.62	0.12	excluded	absent	28.29	-0.32	excluded	absent	27.75	-0.34	excluded	absent	56.1	0.53	excluded	absent	4.12	-1.07	excluded	absent	2.23	-1.12	excluded	absent	17.38	-0.66	excluded	absent	13.62	-0.77	excluded	absent	1.65	-1.14	excluded	absent	8.91	-0.92	excluded	absent	4.55	-1.05	excluded	absent	22.05	-0.51	excluded	absent	0.73	-1.17
1563458_at	included	present	201.7	0.77	excluded	absent	107.02	-0.03	included	present	168.51	0.49	excluded	absent	3.13	-0.9	included	present	107.08	-0.03	included	present	255.29	1.22	excluded	absent	65.18	-0.38	excluded	absent	9.94	-0.84	included	present	190.1	0.67	included	present	146.25	0.3	excluded	absent	65.22	-0.38	excluded	absent	73.65	-0.31	excluded	absent	4.21	-0.89	included	present	57.15	-0.45	excluded	absent	58.46	-0.44
1563460_at	excluded	absent	33.74	-1.01	excluded	absent	1.75	-1.51	excluded	absent	63.9	-0.54	included	present	136.65	0.58	included	present	277.64	2.77	excluded	absent	74.08	-0.39	excluded	absent	53.06	-0.71	excluded	absent	25.45	-1.14	excluded	absent	55.27	-0.68	excluded	absent	64.44	-0.54	included	present	115.09	0.25	excluded	absent	2.52	-1.49	excluded	absent	2.57	-1.49	excluded	absent	22	-1.19	excluded	absent	2.85	-1.49
1563461_at	excluded	absent	60.04	-0.19	excluded	absent	4.12	-1.33	excluded	absent	47.99	-0.44	excluded	absent	3.71	-1.34	excluded	absent	27.18	-0.86	excluded	absent	85.68	0.33	excluded	absent	35.43	-0.69	excluded	absent	1.3	-1.38	excluded	absent	33.52	-0.73	excluded	absent	3.71	-1.34	included	present	239.22	3.45	excluded	absent	33.3	-0.73	excluded	absent	11.56	-1.18	excluded	absent	70.55	0.02	excluded	absent	1.16	-1.39
1563462_at	excluded	absent	70.96	0.43	included	present	216.29	2.72	excluded	absent	1.7	-0.65	excluded	absent	3.8	-0.62	excluded	absent	6.19	-0.58	excluded	absent	50.06	0.11	excluded	absent	4.42	-0.61	excluded	absent	1.16	-0.66	excluded	absent	49.31	0.09	excluded	absent	41.88	-0.02	excluded	absent	2.79	-0.64	excluded	absent	19.48	-0.37	excluded	absent	16.72	-0.42	excluded	absent	30.61	-0.2	excluded	absent	2.05	-0.65
1563465_at	excluded	absent	78.1	0.98	excluded	absent	3.89	-0.88	excluded	absent	43.78	0.12	excluded	absent	7.54	-0.79	excluded	absent	5.38	-0.84	excluded	absent	49.53	0.27	excluded	absent	4.08	-0.87	excluded	absent	20.99	-0.45	excluded	absent	20.17	-0.47	excluded	absent	41.31	0.06	excluded	absent	57.79	0.47	excluded	absent	56.16	0.43	excluded	absent	82.82	1.1	excluded	absent	17.41	-0.54	excluded	absent	22.65	-0.41
1563466_at	included	present	223.06	-0.62	included	present	112.16	-0.86	included	present	186.68	-0.7	included	present	209.32	-0.65	included	absent	123.82	-0.84	included	present	225.13	-0.62	included	absent	62.19	-0.97	included	present	120.7	-0.84	included	present	186.81	-0.7	included	absent	117.3	-0.85	included	present	147.05	-0.79	included	present	216.17	-0.64	included	present	115.13	-0.85	included	present	128.57	-0.83	included	absent	74.76	-0.94
1563467_at	included	absent	82.48	-0.69	included	present	76.91	-0.73	included	absent	41.2	-0.94	included	present	193.82	-0.04	included	absent	70.6	-0.76	included	present	120.11	-0.47	included	absent	45.02	-0.91	included	absent	1.64	-1.17	included	present	111.12	-0.53	included	absent	55.63	-0.85	included	absent	28.27	-1.01	included	present	115.84	-0.5	included	present	126.69	-0.43	included	present	77.98	-0.72	included	absent	8.02	-1.13
1563468_at	excluded	absent	111.32	-0.46	excluded	absent	5.39	-0.87	included	present	162.17	-0.26	included	present	189.52	-0.15	excluded	absent	3.27	-0.88	excluded	absent	109.61	-0.46	excluded	absent	20.54	-0.81	excluded	absent	34.38	-0.76	included	present	160.26	-0.26	excluded	absent	85.13	-0.56	excluded	absent	53.36	-0.68	excluded	absent	1.84	-0.88	excluded	absent	62.69	-0.65	excluded	absent	38.83	-0.74	excluded	absent	17.11	-0.82

[illegible]

1564248_at	included	present	193.6	0.38	excluded	absent	20.67	-0.7	excluded	absent	14.7	-0.74	excluded	absent	75.36	-0.36	excluded	absent	109.21	-0.15	excluded
1564250_at	excluded	absent	75.29	1.1	excluded	absent	12.51	-0.89	included	present	146.47	3.36	excluded	absent	25.88	-0.47	excluded	absent	153.04	3.56	excluded
1564251_at	excluded	absent	33.96	0.1	excluded	absent	10.79	-0.56	excluded	absent	3.91	-0.76	excluded	absent	2.06	-0.81	excluded	absent	4.25	-0.75	excluded
1564253_at	excluded	absent	1.46	-1.19	excluded	absent	40.57	-0.18	excluded	absent	28.6	-0.49	excluded	absent	32.9	-0.38	excluded	absent	24.31	-0.6	excluded
1564254_at	excluded	absent	61.48	0.25	excluded	absent	39.23	-0.16	excluded	absent	22.57	-0.47	excluded	absent	56.6	0.16	excluded	absent	170.89	2.28	excluded
1564257_at	included	present	374.07	-0.57	included	absent	182.28	-0.62	included	present	356.32	-0.58	included	present	487.07	-0.55	included	present	402.43	-0.57	included
1564259_at	excluded	absent	2.93	-0.89	excluded	absent	80.3	1.6	excluded	absent	2.28	-0.91	excluded	absent	63.38	1.05	excluded	absent	3.11	-0.88	excluded
1564262_at	excluded	absent	29.46	-0.58	excluded	absent	0.74	-1.03	excluded	absent	15.24	-0.8	included	present	210.44	2.26	excluded	absent	157.99	1.44	excluded
1564263_at	excluded	absent	35.9	-0.36	excluded	absent	53.79	0.12	excluded	absent	6.58	-1.14	excluded	absent	34.15	-0.4	excluded	absent	61.78	0.34	excluded
1564264_at	excluded	absent	70.61	0.24	excluded	absent	5.47	-1.14	excluded	absent	47.96	-0.24	excluded	absent	74.55	0.33	excluded	absent	49.56	-0.21	excluded
1564272_a_a	excluded	absent	37.28	-0.42	excluded	absent	3.76	-1.44	excluded	absent	2.66	-1.47	excluded	absent	34.64	-0.5	excluded	absent	17.49	-1.02	excluded
1564273_at	excluded	absent	51.06	1.19	excluded	absent	39.71	0.78	excluded	absent	48.4	1.1	excluded	absent	2.85	-0.56	excluded	absent	16.64	-0.06	excluded
1564274_at	included	present	133.59	0.21	excluded	absent	96.24	-0.24	excluded	absent	43.5	-0.89	excluded	absent	43.23	-0.89	included	present	236.55	1.47	excluded
1564276_at	included	present	520.05	-0.12	included	absent	108.21	-1.18	included	present	203.28	-0.94	included	present	858.83	0.75	included	present	198.19	-0.95	included
1564277_a_a	included	present	961.61	9.8	excluded	absent	9.72	-1.02	excluded	absent	78	-0.24	excluded	absent	23.07	-0.86	excluded	absent	123.18	0.27	excluded
1564280_x_a	included	present	200.46	3.85	excluded	absent	15.15	-0.76	excluded	absent	16.5	-0.73	excluded	absent	29.8	-0.4	excluded	absent	15.67	-0.75	excluded
1564281_at	excluded	absent	5.62	-0.43	excluded	absent	6.33	-0.4	excluded	absent	3.08	-0.53	included	present	519.39	21.21	excluded	absent	6.72	-0.38	excluded
1564282_a_a	excluded	absent	35.48	0.29	excluded	absent	2.53	-0.71	excluded	absent	12.96	-0.4	included	present	382.65	10.86	excluded	absent	7.35	-0.57	excluded
1564285_at	excluded	absent	1.38	-1.14	excluded	absent	45.22	-0.06	excluded	absent	76.11	0.69	excluded	absent	44.33	-0.08	included	present	177.78	3.18	excluded
1564287_at	excluded	absent	36.97	-0.05	excluded	absent	20.68	-0.57	excluded	absent	77.32	1.25	excluded	absent	2.04	-1.17	excluded	absent	70.03	1.02	excluded
1564291_at	excluded	absent	8.71	-0.46	excluded	absent	7.57	-0.51	excluded	absent	33.47	0.54	excluded	absent	2.77	-0.7	excluded	absent	4.27	-0.64	excluded
1564292_at	excluded	absent	3.52	-0.54	excluded	absent	8.09	-0.33	included	present	65.87	2.32	excluded	absent	77.3	2.84	excluded	absent	3.48	-0.54	excluded
1564294_at	included	present	111.11	1.49	excluded	absent	38.63	-0.35	included	present	126.72	1.89	excluded	absent	64.92	0.31	included	present	199.48	3.74	excluded
1564295_at	excluded	absent	1.71	-0.98	excluded	absent	51.11	-0.23	excluded	absent	4.77	-0.94	excluded	absent	7.56	-0.9	excluded	absent	64.27	-0.03	excluded
1564299_at	included	present	97.64	0.71	excluded	absent	7.26	-0.33	excluded	absent	42.98	0.08	excluded	absent	1.9	-0.39	excluded	absent	90.16	0.62	excluded
1564300_at	excluded	absent	1.9	-0.8	excluded	absent	9.37	-0.58	excluded	absent	8.87	-0.59	excluded	absent	3.69	-0.74	excluded	absent	11.64	-0.51	excluded
1564301_a_a	included	present	558.94	-0.17	included	present	1457.78	3.06	included	present	1655.16	3.77	included	present	1003.37	1.43	included	present	1320.09	2.56	included
1564306_at	included	present	122.96	-0.16	excluded	absent	89.18	-0.65	excluded	absent	124.18	-0.14	included	present	97.37	-0.53	excluded	absent	89.94	-0.64	excluded
1564307_a_a	excluded	absent	28.5	-0.36	excluded	absent	17.41	-0.38	excluded	absent	50.97	-0.31	excluded	absent	19.74	-0.38	excluded	absent	53.79	-0.31	excluded
1564308_a_a	included	absent	118.95	-0.75	included	absent	61.15	-0.91	included	absent	45.36	-0.96	included	absent	31.08	-1	included	absent	95.55	-0.82	included
1564309_at	excluded	absent	41.71	-0.04	excluded	absent	3.81	-1	excluded	absent	2.26	-1.04	excluded	absent	1.85	-1.05	excluded	absent	53.92	0.27	excluded
1564310_a_a	excluded	absent	4.03	-0.41	excluded	absent	11.26	-0.4	excluded	absent	4.9	-0.41	excluded	absent	1.61	-0.42	excluded	absent	126.47	-0.16	excluded
1564314_at	excluded	absent	41.38	0.17	excluded	absent	5.45	-0.67	excluded	absent	8.79	-0.59	excluded	absent	27.35	-0.16	excluded	absent	4.97	-0.68	excluded
1564315_at	excluded	absent	12.96	-0.71	excluded	absent	29.22	-0.38	excluded	absent	6.25	-0.85	excluded	absent	20.15	-0.56	excluded	absent	43.61	-0.09	excluded
1564317_at	excluded	absent	74.3	-0.27	excluded	absent	111.44	0.5	included	present	81.42	-0.12	excluded	absent	95.26	0.17	excluded	absent	77.68	-0.2	excluded
1564318_at	included	present	132.33	0	excluded	absent	46.26	-1.42	excluded	absent	81.5	-0.84	included	present	118.76	-0.23	excluded	absent	141.34	0.15	excluded
1564319_at	excluded	absent	9.22	-0.58	excluded	absent	3.96	-0.79	excluded	absent	7.68	-0.64	excluded	absent	32.03	0.32	excluded	absent	14.74	-0.36	excluded
1564323_at	excluded	absent	7	-0.59	excluded	absent	1.37	-0.75	excluded	absent	2.76	-0.71	excluded	absent	15.93	-0.35	excluded	absent	9.62	-0.52	excluded
1564327_at	excluded	absent	67.24	-0.03	excluded	absent	63.75	-0.09	excluded	absent	22.62	-0.79	excluded	absent	72.44	0.06	excluded	absent	5.9	-1.07	excluded
1564331_at	included	present	164.68	-0.12	included	absent	75.33	-1.19	included	absent	42.65	-1.58	included	present	138.88	-0.43	included	absent	51.3	-1.47	included
1564333_a_a	excluded	absent	3.79	-0.31	excluded	absent	6.51	-0.3	excluded	absent	7.29	-0.3	excluded	absent	6	-0.3	excluded	absent	33.9	-0.22	excluded
1564334_at	excluded	absent	84.58	1.2	excluded	absent	1.87	-1.19	excluded	absent	3.54	-1.14	excluded	absent	13	-0.87	excluded	absent	10.46	-0.94	excluded
[illegible]																					

1565608_at	included	present	85.09	-0.04	included	present	186.92	2	included
1565610_at	excluded	absent	107.99	0.33	excluded	absent	112.97	0.42	included
1565611_at	excluded	absent	191	0.39	excluded	absent	141.34	-0.24	included
1565612_at	excluded	absent	112.27	0.46	excluded	absent	101.3	0.24	excluded
1565613_at	excluded	absent	129.56	-0.07	excluded	absent	7.76	-0.47	excluded
1565614_at	included	present	671.42	1.21	included	present	330.92	-0.64	included
1565616_at	included	present	209.57	-0.18	included	present	209.69	-0.18	included
1565617_at	excluded	absent	135.62	0.8	excluded	absent	17.65	-1.26	excluded
1565618_at	included	present	348.97	2.59	excluded	absent	39.24	-1.38	excluded
1565620_at	included	present	215.75	-0.55	included	present	124.89	-1.57	included
1565621_at	included	present	1057.72	1.05	included	present	932.88	0.6	included
1565627_a_a	included	present	1209.75	1.96	included	present	471.3	-0.27	included
1565628_at	included	present	416.71	2.49	included	present	154.31	-0.09	excluded
1565633_at	included	present	2315.67	0.06	included	present	595.51	-0.77	included
1565635_at	included	present	1446.43	-0.2	included	present	724.67	-0.65	included
1565637_at	included	present	323.05	1.28	excluded	absent	9.95	-1.37	included
1565638_at	included	present	598.12	2.55	included	present	100.74	-0.84	included
1565639_a_a	included	present	993.28	2.42	included	present	191.55	-0.62	included
1565641_at	included	present	451.55	0.91	included	present	364.9	0.33	included
1565644_at	included	present	269.3	-0.67	included	present	214.15	-0.99	included
1565650_at	included	present	170.44	-0.15	included	present	133.93	-0.5	included
1565651_at	included	present	250.39	1.58	excluded	absent	156.58	0.16	included
1565653_at	excluded	absent	58.53	0.31	excluded	absent	4.71	-1.31	excluded
1565656_x_a	included	present	168.08	1.15	excluded	absent	32.31	-1.13	excluded
1565657_at	excluded	absent	68.77	1.1	excluded	absent	21.33	-0.24	excluded
1565659_at	excluded	absent	3.91	-0.24	excluded	absent	6.77	-0.23	excluded
1565660_at	excluded	absent	5.27	-0.79	excluded	absent	12.02	-0.65	excluded
1565661_x_a	excluded	absent	36.72	-0.34	excluded	absent	41.52	-0.27	excluded
1565662_at	included	present	5306.68	0.98	included	present	2189.76	-0.33	included
1565666_s_a	included	present	3444.67	1.29	included	present	1567.65	-0.11	included
1565668_at	excluded	absent	64.66	-0.01	excluded	absent	101.83	0.73	included
1565669_at	included	present	193.8	0.28	included	present	387.87	2.56	included
1565671_a_a	excluded	absent	63.83	-0.65	excluded	absent	46.43	-0.94	excluded
1565672_at	excluded	absent	10.53	-1	excluded	absent	15.26	-0.92	excluded

1566424_at	excluded	absent	106.02	0.43	excluded	absent	5.81	-1.4	included	present	105.95	0.43	excluded	absent	1.92	-1.47	excluded	absent	103.39	0.38	excluded	absent	186.71	1.91	included	present	109.88	0.5	excluded	absent	29.16	-0.97	excluded	absent	49.34	-0.6	excluded	absent	36.26	-0.84	included	present	58.76	-0.43	excluded	absent	16.88	-1.2	included	present	104.04	0.4	excluded	absent	43.42	-0.71	excluded	absent	32.27	-0.92
1566425_at	excluded	absent	6.1	-1.29	excluded	absent	96.05	0.68	excluded	absent	4.42	-1.32	excluded	absent	5.82	-1.29	excluded	absent	128.34	1.38	excluded	absent	136.29	1.55	excluded	absent	95.44	0.66	included	present	47.05	-0.39	included	present	178.82	2.48	included	present	173.43	2.36	included	present	117.81	1.15	excluded	absent	8.61	-1.23	excluded	absent	99.91	0.76	excluded	absent	76.39	0.25	excluded	absent	41.36	-0.52
1566426_at	included	present	126.27	-0.94	included	present	135.8	-0.79	included	present	216.61	0.48	included	present	72.14	-1.8	included	absent	130.95	-0.87	included	present	148.73	-0.59	included	present	103.93	-1.3	included	absent	45.36	-2.22	included	present	121.31	-1.02	included	present	162.3	-0.38	included	present	175.31	-0.17	included	present	242.85	0.9	included	present	193.86	0.12	included	present	133.03	-0.84	included	present	34.78	-2.39
1566427_at	excluded	absent	3.84	-0.52	excluded	absent	2.24	-0.55	excluded	absent	89.12	1.34	excluded	absent	84.61	1.25	excluded	absent	6.11	-0.47	excluded	absent	1.83	-0.56	excluded	absent	44.44	0.37	excluded	absent	56.73	0.64	excluded	absent	45.25	0.39	excluded	absent	1.99	-0.56	excluded	absent	2.41	-0.55	excluded	absent	56.34	0.63	excluded	absent	5.35	-0.48	excluded	absent	1.49	-0.57	excluded	absent	7.75	-0.43
1566428_at	excluded	absent	4.94	-0.28	excluded	absent	48.61	-0.2	included	present	171.19	0.04	included	present	154.47	0.01	excluded	absent	89.94	-0.12	excluded	absent	3.15	-0.28	excluded	absent	3.19	-0.28	excluded	absent	39.62	-0.21	excluded	absent	103.7	-0.09	excluded	absent	0.92	-0.29	excluded	absent	5.73	-0.28	excluded	absent	72.86	-0.15	excluded	absent	70.97	-0.15	excluded	absent	2.83	-0.28	excluded	absent	1.77	-0.29
1566432_at	excluded	absent	12.8	-0.53	excluded	absent	22.88	-0.17	excluded	absent	12.75	-0.53	excluded	absent	39.24	0.4	excluded	absent	6.39	-0.75	excluded	absent	18.29	-0.33	excluded	absent	4.65	-0.81	excluded	absent	9	-0.66	excluded	absent	65.91	1.34	excluded	absent	46.45	0.66	excluded	absent	7.68	-0.71	excluded	absent	19.08	-0.31	excluded	absent	6.23	-0.76	excluded	absent	9.24	-0.65	excluded	absent	8.55	-0.67
1566433_at	excluded	absent	77.21	1.45	excluded	absent	2.21	-1	excluded	absent	2.22	-1	excluded	absent	40.5	0.25	excluded	absent	69.08	1.19	excluded	absent	72.63	1.3	excluded	absent	56.87	0.79	excluded	absent	20.49	-0.4	excluded	absent	4.16	-0.93	excluded	absent	7.33	-0.83	excluded	absent	61.98	0.95	excluded	absent	10.81	-0.72	excluded	absent	68.72	1.18	excluded	absent	4.48	-0.92	excluded	absent	26.52	-0.2
1566437_at	excluded	absent	1.39	-1.18	excluded	absent	77.48	-0.03	excluded	absent	3.14	-1.15	excluded	absent	1.83	-1.17	excluded	absent	3.59	-1.15	excluded	absent	36.93	-0.64	excluded	absent	34.35	-0.68	included	present	92.55	0.2	included	present	62.02	-0.27	included	present	272.12	2.91	excluded	absent	1.71	-1.18	included	present	64.4	-0.23	excluded	absent	14.73	-0.98	included	present	91.33	0.18	excluded	absent	25.23	-0.82
1566438_at	included	absent	4.7	-1.22	included	present	133.82	-0.31	included	absent	52.01	-0.89	included	absent	12.32	-1.17	included	absent	95.1	-0.58	included	absent	52.29	-0.89	included	present	114.47	-0.45	included	present	150.24	-0.19	included	absent	85.03	-0.66	included	present	588.99	2.91	included	absent	41.1	-0.97	included	absent	85.95	-0.65	included	absent	4.51	-1.23	included	absent	63.17	-0.81	included	absent	35.94	-1
1566439_at	excluded	absent	1.52	-0.93	excluded	absent	36.01	-0.5	excluded	absent	27.53	-0.61	excluded	absent	1.36	-0.94	excluded	absent	202.86	1.59	excluded	absent	1.86	-0.93	included	present	82.63	0.08	excluded	absent	2.83	-0.92	included	present	32.03	-0.55	included	present	23.84	-0.65	included	present	93.49	0.22	excluded	absent	1.53	-0.93	excluded	absent	38.52	-0.47	excluded	absent	70.44	-0.07	excluded	absent	30.76	-0.57
1566440_at	excluded	absent	4.18	-1.05	excluded	absent	40.42	-0.5	excluded	absent	2.64	-1.07	excluded	absent	2.61	-1.07	excluded	absent	119.68	0.7	excluded	absent	16.45	-0.86	included	present	101.13	0.42	excluded	absent	5.82	-1.02	excluded	absent	70.3	-0.05	included	present	60.36	-0.2	excluded	absent	93.17	0.3	excluded	absent	5.25	-1.03	excluded	absent	115.95	0.65	excluded	absent	33.1	-0.61	excluded	absent	3.21	-1.06
1566441_at	excluded	absent	81.53	0.01	excluded	absent	58.43	-0.26	excluded	absent	80.36	0	excluded	absent	53.93	-0.31	excluded	absent	8.1	-0.85	excluded	absent	77.4	-0.04	excluded	absent	88.29	0.09	excluded	absent	34.16	-0.55	excluded	absent	64.09	-0.19	included	present	287.33	2.42	excluded	absent	84.18	0.04	excluded	absent	7.88	-0.85	excluded	absent	144.54	0.75	excluded	absent	61.62	-0.22	excluded	absent	45.2	-0.42
1566442_at	included	present	91.48	-0.29	excluded	absent	33.78	-0.72	included	present	107.46	-0.17	included	present	99.68	-0.23	excluded	absent	92.94	-0.28	excluded	absent	53.71	-0.57	excluded	absent	57.11	-0.55	excluded	absent	23.22	-0.8	included	present	65.42	-0.49	included	present	194.46	0.48	excluded	absent	1.35	-0.97	excluded	absent	71.95	-0.44	included	present	86.91	-0.33	included	present	68.55	-0.46	included	present	40.2	-0.68
1566445_at	included	present	1156.87	0.34	included	present	1069.29	0.25	included	present	887.06	0.06	included	present	371.89	-0.46	included	present	1438.14	0.62	included	present	232.71	-0.6	included	present	450.29	-0.38	included	absent	88.98	-0.75	included	present	309.29	-0.52	included	present	398.26	-0.43	included	present	313.11	-0.52	included	present	217.69	-0.62	included	present	1020.36	0.2	included	present	516.14	-0.31	included	present	143.65	-0.69
1566446_at	included	present	2204.2	0.46	included	present	2570.37	0.68	included	present	1337.68	-0.07	included	present	599.35	-0.52	included	present	3073.49	0.99	included	present	690.08	-0.47	included	present	1637.72	0.11	included	present	220.33	-0.75	included	present	1207.63	-0.15	included	present	1037.18	-0.25	included	present	875.01	-0.35	included	present	344.66	-0.68	included	present	3493.61	1.24	included	present	1872.62	0.26	included	present	567.69	-0.54
1566447_at	excluded	absent	5.23	-0.35	excluded	absent	2.29	-0.37	excluded	absent	44.63	-0.1	excluded	absent	2.03	-0.37	excluded	absent	15.12	-0.29	excluded	absent	5.42	-0.35	excluded	absent	2.44	-0.37	excluded	absent	7.16	-0.34	excluded	absent	132.03	0.45	excluded	absent	1.12	-0.38	excluded	absent	97.79	0.24	excluded	absent	2.67	-0.37	excluded	absent	21.85	-0.25	excluded	absent	51.66	-0.06	excluded	absent	1.19	-0.38
1566448_at	excluded	absent	3.52	-0.76	excluded	absent	5.7	-0.74	excluded	absent	26.51	-0.5	excluded	absent	5.19	-0.74	excluded	absent	43.21	-0.3	excluded	absent	77.61	0.1	excluded	absent	53.82	-0.18	excluded	absent	12.48	-0.66	included	present	83.18	0.17	excluded	absent	31.21	-0.44	excluded	absent	8.98	-0.7	excluded	absent	1.79	-0.78	excluded	absent	3.15	-0.77	excluded	absent	44.43	-0.29	excluded	absent	24.11	-0.52
1566449_at	excluded	absent	34.3	-0.26	excluded	absent	50.05	0.12	excluded	absent	12.57	-0.78	excluded	absent	48.42	0.08	excluded	absent	76.37	0.76	excluded	absent	3.42	-1	excluded	absent	8.08	-0.89	excluded	absent	4.09	-0.99	excluded	absent	13.16	-0.77	excluded	absent	3.41	-1	excluded	absent	69.47	0.59	excluded	absent	74.36	0.71	excluded	absent	33.37	-0.28	excluded	absent	1.88	-1.04	excluded	absent	1.15	-1.06
1566452_at	included	absent	93.58	-1.15	included	absent	35.14	-1.38	included	present	138.69	-0.97	included	present	186.14	-0.78	included	present	68.54	-1.25	included	present	200.26	-0.72	included	present	161.74	-0.88	included	absent	134.16	-0.99	included	present	381.44	0	included	present	132.63	-0.99	included	present	220.04	-0.64	included	present	176.44	-0.82	included	present	225.18	-0.62	included	absent	98.95	-1.13	included	present	125.36	-1.02
1566454_at	excluded	absent	69.46	-0.33	excluded	absent	51.37	-0.65	excluded	absent	3.39	-1.48	excluded	absent	2.57	-1.49	excluded	absent	22.81	-1.14	excluded	absent	80.07	-0.15	excluded	absent	88.87	0	included	present	75.43	-0.23	excluded	absent	117.79	0.5	excluded	absent	54.73	-0.59	excluded	absent	53.77	-0.61	excluded	absent	9.89	-1.36	excluded	absent	68.39	-0.35	excluded	absent	48.41	-0.7	excluded	absent	56.85	-0.55
1566455_at	excluded	absent	36.88	-0.25	excluded	absent	60.37	0.29	excluded	absent	34.09	-0.32	excluded	absent	4.33	-1.01	excluded	absent	68.53	0.48	excluded	absent	15.3	-0.76	excluded	absent	54.66	0.16	excluded	absent	10.13	-0.88	excluded	absent	51.54	0.09	excluded	absent	49.2	0.03	excluded	absent	89.74	0.98	excluded	absent	5.93	-0.97	excluded	absent	121.26	1.71	excluded	absent	6.26	-0.97	excluded	absent	52.77	0.12
1566456_at	excluded	absent	83.69	0.02	excluded	absent	2.51	-1.43	included	present	95.26	0.23	included	present	66.42	-0.29	excluded	absent	23.11	-1.06	excluded	absent	59.68	-0.41	excluded	absent	57.98	-0.44	excluded	absent	77.69	-0.08	excluded	absent	16.18	-1.19	excluded	absent	26.11	-1.01	included	present	96.61	0.26	excluded	absent	9.89	-1.3	excluded	absent	35.24	-0.85	excluded	absent	30.66	-0.93	excluded	absent	42.29	-0.72
1566457_at	excluded	absent	75.95	1.56	excluded	absent	4.77	-0.7	excluded	absent	1.35	-0.81	excluded	absent	1.97	-0.79	excluded	absent	15.59	-0.36	excluded	absent	12.48	-0.46	excluded	absent	3.25	-0.75	excluded	absent	6.29	-0.65	excluded	absent	5.31	-0.68	excluded	absent	1.6	-0.8	excluded	absent	22.41	-0.14	excluded	absent	4.45	-0.71	included	present	93.48	2.12	excluded	absent	1.3	-0.81	excluded	absent	1.06	-0.82
1566459_at	excluded	absent	25.28	-0.38	excluded	absent	1.79	-1.22	excluded	absent	0.95	-1.25	excluded	absent	42.9	0.25	excluded	absent	49.37	0.48	excluded	absent	1.53	-1.23	excluded	absent	37.1	0.04	excluded	absent	26.11	-0.35	excluded	absent	0.46	-1.27	excluded	absent	3.82	-1.15	excluded	absent	28.09	-0.28	excluded	absent	43.18	0.26	excluded	absent	1.33	-1.24	excluded	absent	1.91	-1.21	excluded	absent	11.64	-0.87
1566461_at	excluded	absent	80.04	1.99	excluded	absent	78.17	1.92	excluded	absent	1.63	-0.93	excluded	absent	2.61	-0.89	excluded	absent	68.25	1.55	excluded	absent	62.68	1.35	excluded	absent	1.48	-0.93	excluded	absent	21.69	-0.18	excluded	absent	4.91	-0.81	excluded	absent	1.82	-0.92	excluded	absent	48.28	0.81	excluded	absent	22.36	-0.16	excluded	absent	2.89	-0.88	excluded	absent	6.34	-0.75	excluded	absent	1.88	-0.92
1566462_at	excluded	absent	6.21	-0.68	excluded	absent	6.26	-0.68	excluded	absent	2.05	-0.8	excluded	absent	33.15	0.09	excluded	absent	3.5	-0.76	excluded	absent	28.14	-0.05	excluded	absent	37.12	0.21	excluded	absent	0.18	-0.85	excluded	absent	17.36	-0.36	excluded	absent	2.43	-0.79	excluded	absent	7.23	-0.65	excluded	absent	33.23	0.09	excluded	absent	5.06	-0.71	excluded	absent	0.7	-0.84	excluded	absent	0.59	-0.84
1566463_at	excluded	absent	5.09	-0.6	excluded	absent	3.98	-0.63	excluded	absent	2.11	-0.67	excluded	absent	1.29	-0.69	excluded	absent	5.51	-0.59	excluded	absent	9.99	-0.48	excluded	absent	4.38	-0.62	excluded	absent	5.72	-0.58	excluded	absent	13.46	-0.39	excluded	absent	2.45	-0.67	excluded	absent	34.9	0.15	excluded	absent	69.27	1.01	excluded	absent	56.86	0.7	excluded	absent	31.07	0.05	excluded	absent	1.4	-0.69
1566464_at	excluded	absent	37.68	-0.28	excluded	absent	37.47	-0.28	excluded	absent	5.25	-1.02	excluded	absent	40.03	-0.22	excluded	absent	74.25	0.57	excluded	absent	2.87	-1.08	excluded	absent	32.71	-0.39	excluded	absent	0.96	-1.12	excluded	absent	23.72	-0.6	excluded	absent	3	-1.08	excluded	absent	4.86	-1.03	excluded	absent	12.83	-0.85	excluded	absent	10.62	-0.9	excluded	absent	1.32	-1.11	excluded	absent	14.2	-0.82
1566465_at	excluded	absent	2.17	-1.22	excluded	absent	3.45	-1.21	excluded	absent	38.53	-0.89	excluded	absent	33.1	-0.94	excluded	absent	3.23	-1.21	excluded	absent	42.83	-0.85	excluded	absent	2.63	-1.22	excluded	absent	2.84	-1.22	excluded	absent	1.49	-1.23	excluded	absent	1.22	-1.23	excluded	absent	55.77	-0.73	excluded	absent	0.92	-1.24	excluded	absent	1.77	-1.23	excluded	absent	1.54	-1.23	excluded	absent	1.59	-1.23
1566468_at	excluded	absent	1.3	-0.85	excluded	absent	43.76	0.58	excluded	absent	4.7	-0.74	excluded	absent	22.56	-0.14	excluded	absent	127.99	3.43	excluded	absent	56.14	1	excluded	absent	2.62	-0.81	excluded	absent	3.75	-0.77	excluded	absent	44.43	0.6	excluded	absent	4.3	-0.75	excluded	absent	1.92	-0.83	excluded	absent	24.97	-0.05	excluded	absent	10.18	-0.55	excluded	absent	19.71	-0.23	excluded	absent	26.56	0
1566469_at	included	present	72.29	0.1	excluded	absent	58.05	-0.16	excluded	absent	27.66	-0.69	excluded	absent	3.91	-1.11	excluded	absent	89.56	0.4	excluded	absent	4.31	-1.11	included	present	87.12	0.36	excluded	absent	45.71	-0.37	included	present	75.12	0.15	excluded	absent	49.82	-0.3	excluded	absent	46.37	-0.36	excluded	absent	60.98	-0.1	included	present	95.34	0.5	included	present	110.21	0.77	included	present	78.84	0.21
1566471_at	excluded	absent	5.76	-0.55	excluded	absent	5.77	-0.55	excluded	absent	4.46	-0.6	excluded	absent	6.82	-0.51	excluded	absent	135.25	4.18	excluded	absent	102.7	2.99	excluded	absent	29.13	0.3	excluded	absent	7.31	-0.49	excluded	absent	13.35	-0.27	excluded	absent	74.95	1.98	excluded	absent	26.12	0.19	excluded	absent	21.46	0.02	excluded	absent	5.96	-0.54	excluded	absent	4.56	-0.59	excluded	absent	9.8	-0.4
1566472_s_a	included	present	332.19	0.18	included	absent	40.63	-0.74	included	present	153.89	-0.38	included	absent	76.18	-0.63	included	absent	87.6	-0.59	included	present	223.49	-0.16	included	present	197.74	-0.25	included	present	283.43	0.03	included	present	599.39	1.02	included	present	327.89	0.17	included	present	140.07	-0.43	included	present	127.78	-0.47	included	absent	51.34	-0.71	included	absent	27.7	-0.78	included	present	140.1	-0.43
1566473_a_a	excluded	absent	17.84	-1.36	excluded	absent	79.78	-0.62	excluded	absent	60.6	-0.85	excluded	absent	104.25	-0.33	excluded	absent	119.82	-0.15	excluded	absent	235.43	1.22	excluded	absent	68.58	-0.75	excluded	absent	16	-1.38	excluded	absent	166.95	0.41	excluded	absent	21.55	-1.31	excluded	absent	14.88	-1.39	excluded	absent	62.82	-0.82	excluded	absent	69.12	-0.75	excluded	absent	50.49	-0.97	excluded	absent	62.77	-0.82
[illegible]	[illegible]	[illegible]	[illegible]	[illegible]	[illegible]	[illegible]	[illegible]	[illegible]	[illegible]	[illegible]	[illegible]	[illegible]	[illegible]	[illegible]	[illegible]	[illegible]	[illegible]	[illegible]	[illegible]	[illegible]	[illegible]	[illegible]	[illegible]	[illegible]	[illegible]	[illegible]	[illegible]	[illegible]	[illegible]	[illegible]	[illegible]	[illegible]	[illegible]	[illegible]	[illegible]	[illegible]	[illegible]	[illegible]	[illegible]	[illegible]	[illegible]	[illegible]	[illegible]	[illegible]	[illegible]	[illegible]	[illegible]	[illegible]	[illegible]	[illegible]	[illegible]	[illegible]	[illegible]	[illegible]	[illegible]	[illegible]	[illegible]	[illegible]	[illegible]	[illegible]

1567245_at	excluded	absent	43.89	0.27	excluded	absent	29.73	-0.19	excluded	absent	32.59	[illegible]
1567246_at	excluded	absent	1.81	-0.76	excluded	absent	39.82	0.56	excluded	absent	0.67	[illegible]
1567247_at	excluded	absent	2.72	-0.67	excluded	absent	2.1	-0.7	excluded	absent	1.51	[illegible]
1567248_at	excluded	absent	94.37	0.13	excluded	absent	16.86	-1.2	included	present	132.94	[illegible]
1567249_at	excluded	absent	100.54	-0.08	excluded	absent	74.26	-0.44	excluded	absent	144.85	[illegible]
1567250_at	excluded	absent	80.65	1.51	excluded	absent	10.5	-0.57	excluded	absent	4.75	[illegible]
1567251_at	excluded	absent	35.69	0.54	excluded	absent	2.29	-0.56	excluded	absent	1.76	[illegible]
1567252_at	excluded	absent	44.71	0.25	excluded	absent	48.57	0.33	excluded	absent	2.7	[illegible]
1567253_at	excluded	absent	7.5	-0.32	included	present	108.14	1.91	excluded	absent	1.94	[illegible]
1567254_at	excluded	absent	17.88	-0.51	excluded	absent	7.94	-0.79	excluded	absent	4.06	[illegible]
1567255_at	excluded	absent	72.14	1.61	excluded	absent	33.14	0.3	excluded	absent	46.8	[illegible]
1567256_at	excluded	absent	2.59	-1.01	excluded	absent	44.79	-0.16	excluded	absent	78.99	[illegible]
1567257_at	excluded	absent	2.3	-0.75	excluded	absent	3.25	-0.72	excluded	absent	43.65	[illegible]
1567270_at	excluded	absent	2.71	-0.56	excluded	absent	2.85	-0.56	excluded	absent	3.22	[illegible]
1567271_at	excluded	absent	2.54	-0.63	excluded	absent	3.44	-0.59	excluded	absent	21.86	[illegible]
1567272_at	excluded	absent	15.97	-0.75	excluded	absent	87.05	0.84	excluded	absent	5.45	[illegible]
1567273_at	excluded	absent	2.72	-0.74	excluded	absent	4.87	-0.65	excluded	absent	3.21	[illegible]
1567274_at	excluded	absent	2.57	-0.53	excluded	absent	2.36	-0.54	excluded	absent	2.56	[illegible]
1567277_at	excluded	absent	2.96	-0.53	excluded	absent	4.81	-0.43	excluded	absent	1.63	[illegible]
1567280_at	excluded	absent	3.52	-0.5	excluded	absent	3.06	-0.52	excluded	absent	4.69	[illegible]
1567281_at	excluded	absent	51.85	0.52	excluded	absent	31.46	-0.16	excluded	absent	30.74	[illegible]
1567282_at	excluded	absent	4.88	-0.67	excluded	absent	21.33	-0.07	excluded	absent	6.97	[illegible]
1567284_at	excluded	absent	1.68	-1.05	excluded	absent	38.8	0.23	excluded	absent	16.78	[illegible]
1567285_at	included	present	84.93	-0.02	excluded	absent	8.28	-1.39	excluded	absent	56.44	[illegible]
1567286_at	excluded	absent	80.26	1.36	excluded	absent	20.01	-0.54	excluded	absent	5.99	[illegible]
1567287_at	excluded	absent	33.41	0.37	excluded	absent	2.8	-0.79	excluded	absent	1.59	[illegible]
1567288_at	excluded	absent	1.96	-1.08	excluded	absent	2.56	-1.06	excluded	absent	1.47	[illegible]
1567297_at	excluded	absent	13.96	-0.49	excluded	absent	34.61	0.07	excluded	absent	9.3	[illegible]
1567298_at	excluded	absent	35.44	0.11	excluded	absent	15.72	-0.4	excluded	absent	11.44	[illegible]
1567303_at	included	present	110.68	-0.47	included	absent	79.34	-0.55	included	present	117.54	[illegible]
1567304_at	included	present	288.36	-0.48	included	present	199.75	-0.59	included	present	185.82	[illegible]
1567319_at	excluded	absent	39.57	-0.54	excluded	absent	160.46	1.84	excluded	absent	89.89	[illegible]
1567320_at	excluded	absent	10.86	-0.73	excluded	absent	59.7	0.28	excluded	absent	9.46	[illegible]
1567333_at	excluded	absent	22.95	0.3	excluded	absent	14.14	-0.08	excluded	absent	1.48	[illegible]
[illegible]	[illegible]	[illegible]	[illegible]	[illegible]	[illegible]	[illegible]	[illegible]	[illegible]	[illegible]	[illegible]	[illegible]	[illegible]

1568987_at	included	present	256.43	0.71	included	present	689.1	5.24	included	present	1662.91	15.42	included	present	404.12	2.26	included	present	565.51
1568997_at	included	present	410.2	1.51	included	present	161.93	-0.64	included	present	1341.62	9.59	included	present	809.07	4.97	included	present	554.46
1568999_at	excluded	absent	70.94	0.04	excluded	absent	66.83	-0.03	excluded	absent	4.31	-1.11	excluded	absent	54.67	-0.24	excluded	absent	9.8
1569001_at	excluded	absent	4.26	-1.08	excluded	absent	41.95	-0.06	excluded	absent	27.76	-0.44	excluded	absent	4.65	-1.07	excluded	absent	61.86
1569002_x_a	excluded	absent	47.38	-0.17	excluded	absent	13.97	-1.07	excluded	absent	18.02	-0.96	excluded	absent	10.67	-1.16	excluded	absent	141.54
1569003_at	included	present	2545.69	-0.56	included	present	1920.29	-0.66	included	present	3484.8	-0.41	included	present	5672.29	-0.05	included	present	4356.89
1569004_at	excluded	absent	29.23	-0.2	excluded	absent	3.04	-0.79	excluded	absent	48.16	0.22	excluded	absent	2.43	-0.8	excluded	absent	60.73
1569005_at	included	present	246.53	-0.32	included	absent	29.57	-0.92	included	present	174.88	-0.52	included	present	175.13	-0.52	included	absent	308.31
1569006_at	excluded	absent	10.53	-0.59	excluded	absent	11.53	-0.56	excluded	absent	2.99	-0.79	excluded	absent	63.52	0.81	excluded	absent	35.61
1569008_at	excluded	absent	27.13	-0.7	excluded	absent	23.56	-0.75	excluded	absent	11.3	-0.93	excluded	absent	12.55	-0.91	excluded	absent	57.89
1569009_s_a	excluded	absent	192.98	-0.31	excluded	absent	280.45	0.23	excluded	absent	337.73	0.59	excluded	absent	265.28	0.14	excluded	absent	388
1569013_s_a	included	present	3915.13	0.79	included	present	2575.72	-0.58	included	present	6117.29	3.03	included	present	4858.91	1.75	included	present	2893.71
1569020_at	included	absent	96.83	-0.85	included	absent	60.8	-0.96	included	present	126.68	-0.76	included	present	99.9	-0.84	included	absent	165.99
1569021_at	included	absent	91.07	-0.83	excluded	absent	142.52	0.21	included	absent	60.38	-1.45	included	present	95.15	-0.75	included	absent	137.16
1569022_a_a	included	present	2495.46	0.23	included	present	2145.86	-0.33	included	present	1907.35	-0.71	included	present	2385.51	0.06	included	present	2692.06
1569023_a_a	excluded	absent	1.54	-0.87	excluded	absent	1.61	-0.86	excluded	absent	15.65	-0.3	excluded	absent	52.58	1.18	excluded	absent	10.04
1569024_at	excluded	absent	32.9	-0.89	excluded	absent	44.76	-0.65	included	present	135.5	1.24	excluded	absent	37.76	-0.79	excluded	absent	11.98
1569025_s_a	included	present	593.77	-0.2	included	present	214.12	-1.17	included	present	759.06	0.22	included	present	221.43	-1.15	included	present	188.82
1569029_at	excluded	absent	73.7	0.01	excluded	absent	75.45	0.06	excluded	absent	58.42	-0.39	excluded	absent	30.27	-1.14	excluded	absent	125.83
1569030_s_a	included	present	540.75	-0.44	included	present	1992.73	4.19	included	present	483.25	-0.63	included	present	577.88	-0.32	included	present	1322.29
1569032_at	excluded	absent	38.57	-0.17	excluded	absent	180.09	2.74	excluded	absent	5.1	-0.86	included	present	94.81	0.98	excluded	absent	151.23
1569033_at	excluded	absent	44.71	0.68	included	present	160.74	4.54	included	present	305.58	9.35	excluded	absent	2.87	-0.71	included	present	187.51
1569034_a_a	excluded	absent	28.39	0.05	excluded	absent	9.97	-0.59	excluded	absent	1.27	-0.9	excluded	absent	1.56	-0.89	excluded	absent	5.77
1569039_s_a	included	present	153.3	-0.4	included	present	254.57	-0.28	included	present	423.15	-0.07	included	present	514.92	0.05	included	present	177.27
1569040_s_a	included	absent	33.32	-0.51	included	absent	81.24	-0.48	included	present	69.84	-0.49	included	absent	41.58	-0.5	included	present	271.13
1569041_at	excluded	absent	42.91	0.82	excluded	absent	19.21	-0.04	excluded	absent	47.24	0.98	excluded	absent	1.48	-0.69	excluded	absent	2.72
1569042_at	included	present	84.3	0.98	included	present	97.25	1.3	included	present	95.91	1.26	excluded	absent	61.01	0.41	excluded	absent	13.86
1569044_at	excluded	absent	52.31	-0.39	excluded	absent	158.73	0.05	excluded	absent	11.04	-0.56	included	present	559.74	1.69	excluded	absent	81.17
1569048_s_a	excluded	absent	10.14	-1.3	excluded	absent	73.53	-0.03	excluded	absent	93.85	0.38	excluded	absent	15.49	-1.2	excluded	absent	196.99
1569052_at	included	present	129.68	-0.42	included	absent	109.44	-0.62	included	absent	108.26	-0.63	included	present	117.62	-0.54	included	absent	155.64
1569053_at	included	present	82.73	-1.09	included	present	121.49	-0.61	included	present	150.98	-0.26	included	present	228.54	0.69	included	present	307.59
1569054_at	excluded	absent	36.11	-0.72	excluded	absent	41.35	-0.67	excluded	absent	2.99	-1.02	excluded	absent	1.56	-1.04	excluded	absent	103.3
1569057_s_a	included	present	820.75	0.96	included	present	454.69	-0.97	included	present	691.44	0.28	included	present	796.67	0.84	included	present	653.04
1569058_at	excluded	absent	64.92	-0.54	excluded	absent	12.26	-1.66	included	present	182.56	1.97	excluded	absent	75.96	-0.3	excluded	absent	131.77

[illegible]

1569620_s_a	excluded	absent	54.35	-0.48	excluded	absent	58.16	-0.47	excluded	absent	5.67	-0.65
1569624_at	included	present	174.93	0.26	included	present	183.95	0.37	excluded	absent	71.22	-1
1569629_x_a	included	absent	100.71	-1.07	included	present	121.4	-0.8	included	present	175.33	-0.08
1569630_a_a	excluded	absent	7.37	-0.95	excluded	absent	52.79	0.29	excluded	absent	41.02	-0.03
1569631_at	included	present	293.56	0.1	included	absent	81.34	-2.1	included	present	164.1	-1.24
1569634_at	excluded	absent	21.69	-0.22	excluded	absent	5.74	-0.68	excluded	absent	59.55	0.87
1569637_at	included	present	296.09	-0.5	included	present	347.46	-0.13	included	present	578.72	1.55
1569638_at	included	present	925.36	6.9	excluded	absent	5.01	-1.2	excluded	absent	31.35	-0.96
1569640_s_a	excluded	absent	59.69	0.32	excluded	absent	7.45	-0.91	excluded	absent	9.26	-0.87
1569641_at	included	absent	102.52	-1.04	included	present	162.33	-0.49	included	absent	92.59	-1.13
1569642_at	excluded	absent	4.65	-0.7	excluded	absent	32.76	0.06	excluded	absent	11.76	-0.51
1569644_at	excluded	absent	15.56	-0.26	excluded	absent	37.02	0.59	excluded	absent	4.28	-0.7
1569646_a_a	excluded	absent	132.84	-0.34	excluded	absent	213.78	-0.1	excluded	absent	36.32	-0.64
1569647_at	excluded	absent	19.76	-0.98	excluded	absent	71.66	0.35	excluded	absent	65.03	0.18
1569648_at	excluded	absent	88.64	-0.05	excluded	absent	95.58	0.05	excluded	absent	49.9	-0.61
1569652_at	included	present	150.29	-1.24	included	present	133.85	-1.32	included	present	120.34	-1.38
1569656_at	excluded	absent	104.91	-0.06	excluded	absent	5.52	-1.1	excluded	absent	93.27	-0.18
1569659_at	excluded	absent	42.56	-0.26	excluded	absent	31.09	-0.51	excluded	absent	2.51	-1.13
1569660_at	included	present	78.3	-0.46	excluded	absent	45.5	-0.91	excluded	absent	28.27	-1.14
1569661_at	excluded	absent	78.61	-0.75	excluded	absent	79.5	-0.74	excluded	absent	50.04	-0.96
1569663_at	excluded	absent	2.49	-0.76	excluded	absent	1.08	-0.81	excluded	absent	38.17	0.55
1569664_at	included	present	1519.3	2.49	included	present	6371.3	16.91	included	present	1959.18	3.8
1569665_at	included	present	159.66	0.09	included	absent	76.4	-0.92	included	present	162.98	0.13
1569666_s_a	excluded	absent	84.72	-0.72	included	present	245.88	1.19	included	present	159.08	0.16
1569668_at	excluded	absent	37.39	-1.16	excluded	absent	95.73	0.32	excluded	absent	44.17	-0.99
1569669_at	excluded	absent	3.73	-0.53	included	present	846.46	8.69	excluded	absent	2.54	-0.54
1569670_at	excluded	absent	9.31	-1.14	excluded	absent	3.19	-1.29	excluded	absent	11.11	-1.1
1569672_at	included	present	643.19	2.25	included	present	200.14	-0.43	included	present	809.07	3.25
1569673_at	excluded	absent	6.88	-1.4	included	present	106.86	1.49	excluded	absent	45.03	-0.3
1569674_at	included	present	126.62	-0.73	included	present	99.12	-1.08	included	present	98.23	-1.09
1569675_at	excluded	absent	20.14	-0.5	excluded	absent	38.2	-0.05	excluded	absent	18.49	-0.54
1569676_at	included	present	538.83	-0.37	included	present	520.97	-0.42	included	present	1926.7	3.65
1569677_a_a	excluded	absent	61.78	-0.16	excluded	absent	80.72	-0.05	excluded	absent	2.03	-0.51
1569679_at	excluded	absent	11.3	-1.08	excluded	absent	118.46	0.73	excluded	absent	6.12	-1.17
[illegible]												

1570192_at	excluded	absent	70.41	1.5	excluded	absent	41.34	0.56	excluded	absent	2.3	-0.7	excluded	absent	1.03	-0.74	excluded	absent	66.96	1.39	excluded	absent
1570194_x_a	excluded	absent	4.09	-0.36	excluded	absent	1.59	-0.48	excluded	absent	1.81	-0.47	excluded	absent	2.2	-0.45	excluded	absent	55.54	2.22	excluded	absent
1570196_at	included	present	166.19	2.66	excluded	absent	43.25	-0.24	included	present	197.59	3.4	excluded	absent	20.6	-0.77	excluded	absent	5.2	-1.14	excluded	absent
1570197_at	excluded	absent	62.27	0.36	excluded	absent	3.18	-0.93	excluded	absent	1.88	-0.96	excluded	absent	1.12	-0.98	excluded	absent	3.32	-0.93	excluded	absent
1570198_x_a	excluded	absent	27.02	-0.6	excluded	absent	54.88	0.16	excluded	absent	6.27	-1.16	excluded	absent	2.24	-1.27	excluded	absent	63.07	0.38	included	present
1570200_at	excluded	absent	48.46	-0.31	excluded	absent	16.58	-1	excluded	absent	94.99	0.71	included	present	76.33	0.3	excluded	absent	96.7	0.75	included	present
1570202_a_a	included	present	1575.04	-0.41	included	present	1230.27	-0.61	included	present	779.47	-0.86	included	present	4757.58	1.39	included	present	1393.8	-0.52	included	present
1570204_at	included	present	80.61	-0.19	excluded	absent	41.76	-0.9	included	present	183.51	1.68	included	present	124.68	0.61	excluded	absent	6.84	-1.53	excluded	absent
1570205_at	excluded	absent	37.99	-0.34	excluded	absent	44.12	-0.22	excluded	absent	35.64	-0.39	excluded	absent	6.07	-0.98	excluded	absent	50.32	-0.09	excluded	absent
1570206_at	excluded	absent	21.07	-0.41	excluded	absent	184.16	4.67	included	present	71.36	1.15	included	present	125.09	2.83	excluded	absent	95.56	1.91	excluded	absent
1570207_at	excluded	absent	1.6	-0.7	excluded	absent	4.53	-0.66	excluded	absent	19.23	-0.45	excluded	absent	32.23	-0.28	excluded	absent	80.59	0.39	excluded	absent
1570208_at	excluded	absent	41.38	0.4	excluded	absent	32.54	0.07	excluded	absent	25.51	-0.19	excluded	absent	1.51	-1.07	excluded	absent	28.03	-0.09	excluded	absent
1570210_x_a	excluded	absent	82.86	0.02	excluded	absent	151.59	1.18	excluded	absent	63.09	-0.32	excluded	absent	126.65	0.76	excluded	absent	116.35	0.58	excluded	absent
1570212_a_a	excluded	absent	35.93	-0.07	excluded	absent	50.15	0.37	excluded	absent	40.92	0.09	excluded	absent	39.34	0.04	excluded	absent	68.31	0.94	excluded	absent
1570213_at	included	present	124.84	0.82	excluded	absent	146.91	1.06	included	present	269.49	2.38	excluded	absent	40.94	-0.08	excluded	absent	277.8	2.47	excluded	absent
1570215_at	excluded	absent	2.39	-0.74	excluded	absent	7.88	-0.39	excluded	absent	59.84	2.96	excluded	absent	3.55	-0.67	excluded	absent	14.54	0.04	excluded	absent
1570219_at	excluded	absent	54.81	-0.78	excluded	absent	107.72	0.1	included	present	173.94	1.19	excluded	absent	108.73	0.11	excluded	absent	6.37	-1.59	excluded	absent
1570221_at	excluded	absent	57.78	0.08	excluded	absent	17.96	-0.87	excluded	absent	43.04	-0.27	excluded	absent	91.19	0.87	excluded	absent	116.76	1.48	excluded	absent
1570222_at	excluded	absent	1.5	-0.65	excluded	absent	5.65	-0.52	excluded	absent	2.96	-0.6	included	present	46.53	0.8	excluded	absent	2.83	-0.61	excluded	absent
1570224_at	included	present	81.56	0.97	excluded	absent	10.32	-0.84	excluded	absent	32.68	-0.27	excluded	absent	13.51	-0.76	excluded	absent	8.16	-0.89	excluded	absent
1570225_at	excluded	absent	27.48	0.05	excluded	absent	6.57	-0.6	excluded	absent	40.65	0.46	excluded	absent	8.15	-0.55	excluded	absent	6.69	-0.59	excluded	absent
1570226_at	excluded	absent	75.79	0.07	excluded	absent	77.57	0.11	excluded	absent	72.56	0.01	excluded	absent	40.14	-0.65	excluded	absent	52.04	-0.41	excluded	absent
1570227_at	included	present	474.14	-1.08	included	present	1308.91	1.74	included	present	914.17	0.41	included	present	645.84	-0.5	included	present	924.84	0.44	included	present
1570228_at	excluded	absent	7.13	-0.78	excluded	absent	22.15	-0.33	excluded	absent	44.81	0.36	excluded	absent	29.7	-0.1	excluded	absent	37.14	0.13	excluded	absent
1570229_at	excluded	absent	2.38	-0.82	excluded	absent	2.22	-0.82	excluded	absent	45.57	0.56	excluded	absent	97.98	2.22	excluded	absent	3.12	-0.79	excluded	absent
1570230_at	excluded	absent	43.96	0.31	excluded	absent	5.4	-0.61	excluded	absent	3.36	-0.66	excluded	absent	69.03	0.91	excluded	absent	67.5	0.88	excluded	absent
1570231_at	excluded	absent	3.06	-1.42	excluded	absent	46.3	-0.5	included	present	89.19	0.42	included	present	87.96	0.39	excluded	absent	43.31	-0.56	excluded	absent
1570233_at	excluded	absent	1.16	-0.61	excluded	absent	1.13	-0.61	excluded	absent	3.01	-0.49	excluded	absent	4.05	-0.42	excluded	absent	2.52	-0.52	excluded	absent
1570234_at	excluded	absent	28.05	-0.24	excluded	absent	39.61	0.04	excluded	absent	1.44	-0.9	excluded	absent	49.43	0.29	excluded	absent	3.58	-0.85	excluded	absent
1570235_at	excluded	absent	1.58	-0.61	excluded	absent	3.32	-0.56	excluded	absent	13.87	-0.25	excluded	absent	3.15	-0.57	excluded	absent	22.81	0.01	excluded	absent
1570237_at	excluded	absent	31.45	-0.57	excluded	absent	80.97	0.71	included	present	331.69	7.21	excluded	absent	3.6	-1.29	excluded	absent	3.1	-1.3	excluded	absent
1570238_at	included	absent	132.79	-1.25	included	absent	105.74	-1.57	included	present	241.22	0.02	included	present	900.35	7.74	included	absent	193.43	-0.54	included	present
1570239_a_a	excluded	absent	31.93	-0.57	excluded	absent	5.05	-0.83	excluded	absent	6.77	-0.81	included	present	526.37	4.3	excluded	absent	71.39	-0.18	excluded	absent
1570241_at	excluded	absent	6.2	-0.67	excluded	absent	8.04	-0.58	excluded	absent	6.67	-0.65	excluded	absent	9.36	-0.51	excluded	absent	14.19	-0.27	excluded	absent
1570242_x_a	excluded	absent	13.79	-0.75	excluded	absent	43.85	0.19	excluded	absent	17.89	-0.62	excluded	absent	10.45	-0.86	excluded	absent	9.05	-0.9	excluded	absent
1570243_at	included	absent	87.7	-0.64	included	absent	78.12	-0.73	included	absent	33.63	-1.19	included	present	171.51	0.22	included	present	146.59	-0.04	included	absent
1570244_at	excluded	absent	45.23	0.05	excluded	absent	53.86	0.28	excluded	absent	83.06	1.05	excluded	absent	52.48	0.24	excluded	absent	20.07	-0.61	excluded	absent
1570246_at	excluded	absent	5	-0.34	excluded	absent	7	-0.25	excluded	absent	2.19	-0.46	excluded	absent	1.12	-0.5	excluded	absent	19.21	0.28	excluded	absent
1570248_at	excluded	absent	1.77	-1.58	excluded	absent	54.74	-0.25	excluded	absent	89.1	0.61	excluded	absent	36.43	-0.71	excluded	absent	42.56	-0.56	excluded	absent
1570249_x_a	excluded	absent	71.89	-0.53	included	present	143.54	0.98	excluded	absent	45.54	-1.09	excluded	absent	61.86	-0.75	excluded	absent	79.56	-0.37	included	present
1570250_at	excluded	absent	2.48	-0.8	excluded	absent	63.37	0.29	excluded	absent	3.95	-0.77	excluded	absent	46.22	-0.02	excluded	absent	3.75	-0.77	excluded	absent
1570251_at	excluded	absent	90.43	0.63	excluded	absent	42.86	-0.46	excluded	absent	100.35	0.86	excluded	absent	5.03	-1.33	excluded	absent	15.08	-1.1	included	present
1570252_at	excluded	absent	15.22	-0.33	excluded	absent	37.46	0.2	excluded	absent	13.75	-0.36	excluded	absent	2.78	-0.62	excluded	absent	5.54	-0.56	excluded	absent
1570253_a_a	excluded	absent	146.37	2.47	included	present	156.82	2.73	excluded	absent	87.31	1	excluded	absent	17.65	-0.73	excluded	absent	3.32	-1.08	excluded	absent
1570255_s_a	excluded	absent	170.89	-0.14	included	present	300.56	0.42	included	present	109.24	-0.41	included	present	100.28	-0.44	included	present	315.35	0.49	excluded	absent
1570257_x_a	excluded	absent	4.14	-1.13	excluded	absent	56.98	0.47	excluded	absent	27.96	-0.41	excluded	absent	2.28	-1.19	excluded	absent	12.71	-0.87	excluded	absent
1570259_at	included	present	101.89	-0.38	excluded	absent	86.76	-0.52	excluded	absent	45.89	-0.9	excluded	absent	52.32	-0.84	excluded	absent	65.48	-0.72	excluded	absent
1570261_at	excluded	absent	5.22	-0.65	excluded	absent	44.85	0.65	excluded	absent	1.45	-0.78	excluded	absent	21.48	-0.12	excluded	absent	67.74	1.4	excluded	absent
1570262_at	excluded	absent	2.89	-0.63	excluded	absent	4.59	-0.52	excluded	absent	6.05	-0.43	excluded	absent	7.09	-0.36	excluded	absent	3.01	-0.62	excluded	absent
1570263_at	excluded	absent	2.1	-0.58	excluded	absent	5.47	-0.44	excluded	absent	4.72	-0.48	excluded	absent	2.91	-0.55	excluded	absent	69.01	2.1	excluded	absent
1570264_at	included	present	256.81	-0.99	included	present	363.91	-0.67	included	present	176.28	-1.23	included	absent	130.86	-1.37	included	present	334.33	-0.76	included	absent
1570266_x_a	included	present	878.39	-1.45	included	present	1235.6	-0.59	included	present	1153.12	-0.79	included	present	1098.38	-0.92	included	present	2071.21	1.44	included	present
1570267_at	excluded	absent	5.55	-0.97	excluded	absent	125.89	1.94	excluded	absent	11.13	-0.83	excluded	absent	2.51	-1.04	excluded	absent	116.1	1.7	excluded	absent
1570268_at	excluded	absent	2.56	-0.92	excluded	absent	40.58	0.37	excluded	absent	39.8	0.35	excluded	absent	2.48	-0.92	excluded	absent	65.35	1.21	excluded	absent
1570269_at	excluded	absent	2.53	-0.52	excluded	absent	1.73	-0.55	excluded	absent	2.41	-0.52	excluded	absent	58.03	1.84	excluded	absent	3.68	-0.47	excluded	absent
1570270_at	excluded	absent	48.5	0.56	excluded	absent	48.7	0.57	excluded	absent	1.22	-0.9	excluded	absent	1.67	-0.88	excluded	absent	10.67	-0.61	excluded	absent
1570271_at	excluded	absent	10.04	-0.62	excluded	absent	39.99	-0.09	excluded	absent	4.59	-0.72	excluded	absent	3.66	-0.73	excluded	absent	129.81	1.52	excluded	absent
1570272_at	excluded	absent	25.07	-0.8	excluded	absent	89.33	0.09	excluded	absent	18.66	-0.89	excluded	absent	55.02	-0.38	excluded	absent	80.48	-0.03	excluded	absent
1570273_at	excluded	absent	41.06	-0.4	excluded	absent	46.98	-0.34	excluded	absent	6.74	-0.74	excluded	absent	13.31	-0.68	included	present	206.54	1.25	excluded	absent
1570274_at	excluded	absent	67.03	0.23	excluded	absent	78.56	0.47	excluded	absent	11.92	-0.88	included	present	97.09	0.84	excluded	absent	28.27	-0.55	excluded	absent
1570276_a_a	excluded	absent	2.04	-0.8	excluded	absent	5.14	-0.71	excluded	absent	3.93	-0.74	excluded	absent	65.48	0.95	excluded	absent	3.96	-0.74	excluded	absent
1570279_at	excluded	absent	73.89	0.24	excluded	absent	7.82	-0.78	excluded	absent	8.12	-0.77	excluded	absent	2.58	-0.86	excluded	absent	11.69	-0.72	excluded	absent
[illegible]																						

200603_at	included	present	7954.1	0.52	included	present	4824.6	-0.86	included	present	5761.4	-0.45	included	present	7156.45	0.17	included	present	5065.17	-0.75	included	present	6683.83	-0.04	included	present	10635.51	1.69	included	present	6696.75	-0.04	included	present	7564.05	0.34	included	present	4893.32	-0.83	included	present	5216.83	-0.69	included	present	6300.72	-0.21	included	present	7919.53	0.5	included	present	6800.98	0.01	included	present	6147.31	-0.28
200604_s_at	included	present	9892.01	2.04	included	present	7572.29	1.02	included	present	5875.37	0.27	included	present	5923.23	0.29	included	present	8710.24	1.52	included	present	4591.54	-0.3	included	present	6600.87	0.59	included	present	5640.94	0.16	included	present	6787.95	0.67	included	present	6082.35	0.36	included	present	4903.36	-0.16	included	present	3257.44	-0.89	included	present	2096.66	-1.4	included	present	3617.98	-0.73	included	present	7562.43	1.01
200605_s_at	included	present	8012.97	1.06	included	present	4862.63	-0.49	included	present	5025.36	-0.41	included	present	7015.46	0.57	included	present	5897.13	0.02	included	present	9956.82	2.03	included	present	10682.05	2.38	included	present	6540.94	0.34	included	present	8947.17	1.53	included	present	4323	-0.76	included	present	5388.17	-0.23	included	present	1623.14	-2.1	included	present	3426.2	-1.2	included	present	3402.13	-1.22	included	present	6150.52	0.14
200606_at	excluded	absent	81.07	-0.38	excluded	absent	2.39	-0.4	included	present	283.63	-0.33	included	present	4516.97	0.63	excluded	absent	49.39	-0.39	included	present	337.89	-0.32	excluded	absent	5.88	-0.4	excluded	absent	5.04	-0.4	included	present	202.78	-0.35	excluded	absent	2.45	-0.4	included	present	128.33	-0.37	excluded	absent	56.07	-0.39	included	present	119.36	-0.37	excluded	absent	53.87	-0.39	excluded	absent	42.12	-0.39
200607_s_at	included	present	4757.93	0.95	included	present	3374.05	-0.42	included	present	15221.73	11.27	included	present	5967.76	2.14	included	present	8023.2	4.17	included	present	1663.81	-2.1	included	present	4139.34	0.34	included	present	3424.35	-0.37	included	present	2680.11	-1.1	included	present	2799.86	-0.98	included	present	6251.4	2.42	included	present	1342.76	-2.42	included	present	2802.04	-0.98	included	present	3415.63	-0.38	included	present	6054.79	2.23
200608_s_at	included	present	1691.52	-0.53	included	present	1526.08	-0.88	included	present	4226.31	4.73	included	present	2484.12	1.11	included	present	3203.77	2.61	included	present	1624.68	-0.67	included	present	4375.58	5.04	included	present	3351.67	2.91	included	present	2812.39	1.79	included	present	1885.85	-0.13	included	present	5540.23	7.45	included	present	1786.85	-0.33	included	present	2536.72	1.22	included	present	2120.67	0.36	included	present	4833.1	5.99
200609_s_at	included	present	1699.35	-0.48	included	present	1627.59	-0.54	included	present	928.46	-1.19	included	present	935.81	-1.18	included	present	1808.94	-0.37	included	present	4322.42	1.95	included	present	959.49	-1.16	included	present	1744.79	-0.43	included	present	2123.16	-0.08	included	present	2099.83	-0.11	included	present	1251.95	-0.89	included	present	1318.22	-0.83	included	present	950.6	-1.17	included	present	1127.9	-1	included	present	1503.3	-0.66
200610_s_at	included	present	7641.96	-0.75	included	present	12019.6	1.12	included	present	11012.86	0.69	included	present	9282.23	-0.05	included	present	5827.44	-1.53	included	present	8105.58	-0.55	included	present	6121.55	-1.4	included	present	9544.12	0.06	included	present	6948.04	-1.05	included	present	6421.97	-1.27	included	present	7598.52	-0.77	included	present	8769.96	-0.27	included	present	8933.16	-0.2	included	present	13142.55	1.6	included	present	7979.27	-0.61
200611_s_at	included	present	768.6	-0.49	included	present	1298.53	0.48	included	present	357.73	-1.24	included	present	363.69	-1.23	included	present	640.3	-0.72	included	present	2715.04	3.07	included	present	524.98	-0.93	included	present	1192.67	0.29	included	present	1261.57	0.41	included	present	1725.41	1.26	included	present	1245.02	0.38	included	present	925.87	-0.2	included	present	175.24	-1.57	included	present	192.07	-1.54	included	present	990.61	-0.08
200612_s_at	included	present	571.86	-0.38	included	present	1323.28	1.71	included	present	478.11	-0.64	included	present	306.22	-1.12	included	present	878.64	0.47	included	present	272.66	-1.21	included	present	1000.09	0.81	included	present	1335.66	1.74	included	present	392.56	-0.88	included	present	629.04	-0.22	included	present	440.23	-0.75	included	present	241.35	-1.3	included	present	154.11	-1.54	included	absent	63.32	-1.8	included	present	967.59	0.72
200613_at	included	present	1301.56	0.96	included	present	884.3	-0.1	included	present	505.83	-1.05	included	present	433.12	-1.23	included	present	938.69	0.04	included	present	2313.04	3.51	included	present	2250.2	3.35	included	present	2712.15	4.52	included	present	1555.19	1.6	included	present	2579.84	4.18	included	present	2143.15	3.08	included	present	1825.13	2.28	included	present	540.67	-0.96	included	present	397.63	-1.32	included	present	4062.47	7.93
200614_at	included	present	10214.45	4.4	included	present	7081.01	2.01	included	present	2935.63	-1.17	included	present	6860.4	1.84	included	present	6238.4	1.36	included	present	5839.83	1.06	included	present	9357.63	3.75	included	present	8362.88	2.99	included	present	6851.45	1.83	included	present	8409.13	3.02	included	present	5083.93	0.48	included	present	8579.56	3.15	included	present	11724.83	5.56	included	present	19821.77	11.75	included	present	9964.54	4.21
200615_s_at	included	present	1872.15	0.48	included	present	2743.86	1.51	included	present	1854.48	0.46	included	present	1321.96	-0.17	included	present	3343.33	2.22	included	present	1581.3	0.14	included	present	5712.91	5.03	included	present	6674.97	6.17	included	present	3109.74	1.95	included	present	4257	3.31	included	present	2452.43	1.17	included	present	1821.58	0.42	included	present	2746.1	1.52	included	present	1723.61	0.3	included	present	4485.68	3.58
200616_s_at	included	present	5562.98	0.58	included	present	5328.23	0.46	included	present	5207.82	0.41	included	present	8031.48	1.76	included	present	5413.9	0.5	included	present	2755.01	-0.77	included	present	2375.64	-0.95	included	present	2355.54	-0.96	included	present	2077.49	-1.1	included	present	1675.74	-1.29	included	present	3435.87	-0.45	included	present	2125.39	-1.07	included	present	1604.73	-1.32	included	present	2569.35	-0.86	included	present	2371.69	-0.96
200617_at	included	present	1084.4	-0.1	included	present	1360.1	0.32	included	present	900.72	-0.38	included	present	1402.95	0.39	included	present	892.72	-0.39	included	present	1281.82	0.2	included	present	1790.4	0.98	included	present	930.99	-0.33	included	present	1405.82	0.39	included	present	1633.02	0.74	included	present	1723.06	0.88	included	present	906.8	-0.37	included	present	412.62	-1.12	included	present	453.69	-1.06	included	present	1709.03	0.86
200618_at	included	present	3832.93	1.96	included	present	2886.04	1.08	included	present	1091.51	-0.58	included	present	2671.04	0.88	included	present	2618.26	0.84	included	present	5959.89	3.92	included	present	4030.36	2.14	included	present	2498.81	0.72	included	present	3498.19	1.65	included	present	3081.99	1.26	included	present	2347.19	0.58	included	present	4352	2.44	included	present	6529.95	4.45	included	present	7778.12	5.6	included	present	3132.89	1.31
200619_at	included	present	7749.4	0.76	included	present	14362.97	6.14	included	present	8147.59	1.08	included	present	6108.39	-0.57	included	present	6408.08	-0.33	included	present	5466.85	-1.1	included	present	8987.42	1.77	included	present	7093.61	0.23	included	present	5959.38	-0.69	included	present	5938.12	-0.71	included	present	7243.26	0.35	included	present	2793.11	-3.27	included	present	4479.89	-1.9	included	present	7907.22	0.89	included	present	8290.16	1.2
200620_at	included	present	4395.6	-0.15	included	present	3459.86	-0.6	included	present	2011.52	-1.31	included	present	5056.6	0.17	included	present	6963.83	1.1	included	present	3397.26	-0.63	included	present	5512.4	0.4	included	present	6072.31	0.67	included	present	5436.88	0.36	included	present	4806.44	0.05	included	present	3554.09	-0.56	included	present	8103.36	1.65	included	present	4748.51	0.02	included	present	6378.89	0.82	included	present	5535.24	0.41
200621_at	included	present	6518.08	-0.4	included	present	1437.15	-0.95	included	present	515.8	-1.05	included	present	1398.77	-0.96	included	present	1790.08	-0.91	included	present	11632.75	0.15	included	present	1728.85	-0.92	included	present	1756.15	-0.92	included	present	4396.47	-0.63	included	present	1276.9	-0.97	included	present	1321.02	-0.96	included	present	2320.71	-0.86	included	present	1682.55	-0.92	included	present	1887.71	-0.9	included	present	1409.63	-0.95
200622_x_at	included	present	3514.75	-0.33	included	present	2056.68	-0.72	included	present	1342.78	-0.91	included	present	2186.55	-0.68	included	present	2104.98	-0.7	included	present	3115.02	-0.43	included	present	1844.31	-0.77	included	present	1508.18	-0.86	included	present	2822.4	-0.51	included	present	3605.73	-0.3	included	present	3945.05	-0.21	included	present	1969.87	-0.74	included	present	395.44	-1.16	included	present	357.01	-1.17	included	present	3852.62	-0.24
200623_s_at	included	present	6261.04	0.08	included	present	3681.06	-0.4	included	present	2115.46	-0.69	included	present	2933.33	-0.54	included	present	2826.4	-0.56	included	present	3255.77	-0.48	included	present	3202.36	-0.49	included	present	4063.54	-0.33	included	present	3513.33	-0.43	included	present	3690.57	-0.4	included	present	3763.41	-0.38	included	present	2604.52	-0.6	included	present	1421.87	-0.82	included	present	1237.95	-0.85	included	present	4676.54	-0.21
200624_s_at	included	present	5703.68	-0.64	included	present	7171.65	0.39	included	present	13706.73	4.96	included	present	9211.06	1.81	included	present	7957.12	0.94	included	present	3090.35	-2.47	included	present	6387.49	-0.16	included	present	4750.72	-1.31	included	present	5566.73	-0.74	included	present	5677.54	-0.66	included	present	5988	-0.44	included	present	4047.56	-1.8	included	present	8276.2	1.16	included	present	8052.93	1	included	present	5469.02	-0.8
200625_s_at	included	present	5239.14	2.29	included	present	5397	2.43	included	present	1122.05	-1.54	included	present	2046.27	-0.68	included	present	6410.54	3.38	included	present	5775.35	2.78	included	present	3015.43	0.22	included	present	4738.49	1.82	included	present	3462.6	0.63	included	present	3390.6	0.57	included	present	2702.34	-0.07	included	present	7315.33	4.22	included	present	8564.03	5.38	included	present	11004.58	7.65	included	present	4396.92	1.5
200626_s_at	included	present	7014.86	-0.57	included	present	7659.85	-0.35	included	present	13584.52	1.73	included	present	9742.51	0.38	included	present	8550.02	-0.03	included	present	6089.01	-0.9	included	present	9218.27	0.2	included	present	6581.82	-0.72	included	present	7546.99	-0.39	included	present	7200.08	-0.51	included	present	8006.03	-0.22	included	present	7667.93	-0.34	included	present	10609.53	0.69	included	present	7562.27	-0.38	included	present	7116.69	-0.54
200627_at	included	present	5996.74	-0.75	included	present	5444.2	-1.05	included	present	12171.62	2.59	included	present	8657.72	0.69	included	present	8253.96	0.47	included	present	5193.37	-1.19	included	present	6677.42	-0.39	included	present	7922.72	0.29	included	present	7269.39	-0.06	included	present	6604.86	-0.42	included	present	10248.06	1.55	included	present	6817.32	-0.31	included	present	6131.02	-0.68	included	present	5924.65	-0.79	included	present	8548.65	0.63
200628_s_at	included	present	1604.58	1.56	included	present	856.09	0.47	included	present	324.29	-0.3	included	present	149.59	-0.56	included	present	399.51	-0.19	included	present	2081.14	2.25	included	present	403.3	-0.19	included	present	781.06	0.36	included	present	943.49	0.6	included	present	408.13	-0.18	included	present	604.96	0.11	included	present	627.68	0.14	included	present	210.3	-0.47	included	present	88.29	-0.65	included	present	2913.29	3.46
200629_at	included	present	3037.63	1.26	included	present	1301.8	0.03	included	present	625.3	-0.45	included	present	606.93	-0.46	included	present	540.1	-0.51	included	present	3206.08	1.38	included	present	770.51	-0.35	included	present	1122.59	-0.1	included	present	1323.63	0.05	included	present	525.12	-0.52	included	present	883.84	-0.27	included	present	1098.88	-0.11	included	present	1018.25	-0.17	included	present	577.1	-0.48	included	present	2830.01	1.11
200630_x_at	included	present	9942.21	1.48	included	present	7982.42	0.57	included	present	29573.04	10.69	included	present	11934.14	2.42	included	present	15030.63	3.87	included	present	9004.12	1.04	included	present	11504.4	2.22	included	present	10218.09	1.61	included	present	9306.06	1.19	included	present	6003.93	-0.36	included	present	18797.99	5.64	included	present	11420.39	2.18	included	present	4587.96	-1.03	included	present	5827.34	-0.44	included	present	10998.78	1.98
200631_s_at	included	present	4882.31	0.16	included	present	3985.91	-0.64	included	present	15185.7	9.32	included	present	6534.16	1.63	included	present	7493.05	2.48	included	present	3749.06	-0.85	included	present	7204.61	2.23	included	present	7438.97	2.43	included	present	5019.04	0.28	included	present	5552.13	0.76	included	present	9685.05	4.43	included	present	4018.67	-0.61	included	present	5144.51	0.39	included	present	6140.23	1.28	included	present	8880.07	3.72
200632_s_at	included	present	12904.16	0.5	included	present	5041.13	-0.73	included	present	1873.41	-1.23	included	present	7141.37	-0.4	included	present	7431.92	-0.36	included	present	5517.89	-0.66	included	present	7461.78	-0.35	included	present	3206.88	-1.02	included	present	5196.28	-0.71	included	present	3096.84	-1.04	included	present	3089.36	-1.04	included	present	4358.17	-0.84	included	present	3809.51	-0.92	included	present	3998.32	-0.89	included	present	6937.35	-0.43
200633_at	included	present	22163.37	-0.59	included	present	24411.54	-0.23	included	present	28210.79	0.36	included	present	17596.67	-1.3	included	present	37638.57	1.84	included	present	17163.3	-1.37	included	present	16880.37	-1.42	included	present	17372.98	-1.34	included	present	15267.85	-1.67	included	present	25118.89	-0.12	included	present	18773.01	-1.12	included	present	38486.07	1.98	included	present	24856.36	-0.16	included	present	25248.43	-0.1	included	present	22059.42	-0.6
200634_at	included	present	4059.63	-0.41	included	present	9925.15	1.23	included	present	4977.68	-0.15	included	present	4873.12	-0.18	included	present	6550.58	0.29	included	present	16714.94	3.12	included	present	5859.97	0.09	included	present	18063.83	3.5	included	present	8867.15	0.93	included	present	6998.22	0.41	included	present	11293.07	1.61	included	present	20282.47	4.12	included	present	1618.75	-1.09	included	present	1775.92	-1.05	included	present	14380.32	2.47

200962_at	included	present	322.57	-0.07	included	present	317.65	-0.1	included	present	351.45	0.09	included	present	214.35	-0.68	included	present	225.16
200963_x_at	included	present	21513.13	-0.9	included	present	40648.39	0.65	included	present	31000.18	-0.13	included	present	17137.87	-1.25	included	present	22015.14
200964_at	included	present	4852.61	2.7	included	present	6084.08	4.55	included	present	2507.71	-0.82	included	present	2056.82	-1.49	included	present	3325.63
200965_s_at	included	present	4109.04	-0.85	included	present	5305.97	-0.54	included	present	4571.06	-0.73	included	present	1120.51	-1.61	included	present	1297.44
200966_x_at	included	present	4989.56	0.28	included	present	2337.89	-0.36	included	present	1216.45	-0.62	included	present	1929.28	-0.45	included	present	1913.69
200967_at	included	present	5079.86	0.38	included	present	6625.56	0.96	included	present	2983.92	-0.41	included	present	4823.26	0.28	included	present	2596.49
200968_s_at	included	present	2828.52	1.38	included	present	2205.94	0.72	included	present	1483.83	-0.05	included	present	2083.95	0.59	included	present	1747.56
200969_at	included	present	1229.37	-0.57	included	present	1175.43	-0.62	included	present	907.62	-0.88	included	present	2284.62	0.47	included	present	760.27
200970_s_at	included	present	587.34	-0.6	included	present	601.34	-0.59	included	present	797.19	-0.34	included	present	1198.15	0.18	included	present	386.52
200971_s_at	included	present	2695.62	-0.45	included	present	2212.58	-0.63	included	present	2258.73	-0.61	included	present	3705.84	-0.07	included	present	2035.76
200972_at	included	present	3874.22	0.64	included	present	5057.23	1.46	included	present	2633	-0.22	included	present	2017.14	-0.65	included	present	1994.38
200973_s_at	included	present	2587.76	-0.04	included	present	1676.81	-0.72	included	present	1850.45	-0.59	included	present	1528.67	-0.83	included	present	1007.55
200974_at	included	present	1359.18	-0.65	included	present	1791.45	-0.62	included	present	2589.04	-0.57	included	present	7064.87	-0.28	included	present	3006.02
200975_at	included	present	3234.22	-0.14	included	present	3971.13	0.44	included	present	1549.11	-1.46	included	present	4234.99	0.64	included	present	4247.88
200976_s_at	included	present	3481.31	0.77	included	present	3463.39	0.74	included	present	1653.99	-2.14	included	present	3092.1	0.15	included	present	10060.83
200977_s_at	included	present	4422.8	-0.76	included	present	6269.56	0.23	included	present	2474.91	-1.81	included	present	3073.75	-1.48	included	present	9470.61
200978_at	included	present	2895.5	-0.5	included	present	3549.92	-0.3	included	present	1747.34	-0.85	included	present	1806.33	-0.83	included	present	2298.81
200979_at	included	present	265.51	-0.77	included	present	178.91	-1.12	included	present	208.87	-1	included	present	210.15	-0.99	included	present	390.08
200980_s_at	included	present	2376.64	-0.65	included	present	3533.78	-0.28	included	present	3773.1	-0.2	included	present	2408.14	-0.64	included	present	4182.15
200981_x_at	included	present	38461.72	0.48	included	present	51400.59	1.37	included	present	42044.48	0.73	included	present	48231.14	1.15	included	present	75221.92
200982_s_at	included	present	1108.72	-0.06	included	present	348.69	-1.3	included	present	393.36	-1.22	included	absent	51.8	-1.78	included	present	323.93
200983_x_at	included	present	5243.99	0.92	included	present	1763.37	-0.56	included	present	1624.46	-0.62	included	present	1097.49	-0.84	included	present	2616.39
200984_s_at	included	present	8218.1	1.91	included	present	1246.94	-1.19	included	present	2137.33	-0.79	included	present	1327.92	-1.15	included	present	2009.28
200985_s_at	included	present	27816.68	2.12	included	present	6911.02	-1.22	included	present	12428	-0.34	included	present	5756.78	-1.4	included	present	9932.73
200986_at	included	present	2042.7	0.4	included	present	264.01	-0.4	included	present	529.73	-0.28	included	present	106.03	-0.47	included	present	391.45
200987_x_at	included	present	1897.57	0.14	included	present	3705.48	2.45	included	present	4794.84	3.85	included	present	1936.9	0.19	included	present	2479.34
200988_s_at	included	present	190.36	0.71	included	present	170.62	0.32	included	present	281.6	2.49	included	present	199.48	0.88	included	present	221.85
200989_at	included	present	1465.24	-0.73	included	present	2845.25	0.77	included	present	1934.04	-0.22	included	present	2366.2	0.25	included	present	1763.78
200990_at	excluded	absent	116.5	-0.82	included	present	275.22	0.85	excluded	absent	193.27	-0.01	included	present	265.54	0.75	excluded	absent	95.61
200991_s_at	included	present	881.9	1.14	included	present	888.17	1.18	included	present	771.62	0.61	included	present	389.98	-1.24	included	present	685.24
200992_at	included	present	2920.06	-0.13	included	present	4765.43	1.38	included	present	3628.92	0.45	included	present	3335.88	0.21	included	present	1622
200993_at	included	present	2173.93	-0.1	included	present	6628.58	4.82	included	present	6132.97	4.27	included	present	3479.63	1.34	included	present	2897.71
200994_at	included	present	376.85	-1.03	included	present	877.68	1.1	included	present	575.22	-0.18	included	present	768.84	0.64	included	present	299.89
200995_at	included	present	1061.94	-0.73	included	present	2496.18	0.74	included	present	3403.36	1.67	included	present	2175.86	0.42	included	present	1205.77

[illegible]

201321_s_at	included	present	284.09	-0.39	included	present	387.44	0.64	included	present	330.8
201322_at	included	present	7524.35	0.21	included	present	9474.99	0.72	included	present	10663
201323_at	included	present	2279.33	-0.53	included	present	7471.88	5.18	included	present	1617.42
201324_at	included	present	4322.06	0.03	included	present	472.89	-0.89	included	present	260.81
201325_s_at	included	present	969.99	-0.09	included	absent	139.72	-0.78	included	absent	150.88
201326_at	included	present	3936.94	-0.62	included	present	10307.75	3.2	included	present	8712.78
201327_s_at	included	present	3106.91	0.22	included	present	7623.05	5.21	included	present	7538.95
201328_at	included	present	1200.36	-0.38	included	present	433.1	-0.85	included	present	604.83
201329_s_at	included	present	580.6	-0.52	included	present	453.3	-0.74	included	present	324.63
201330_at	included	present	410.21	0.09	excluded	absent	624.92	1.88	included	present	589.53
201331_s_at	included	present	1636.45	-0.44	included	present	1461.74	-0.55	included	present	447.62
201332_s_at	included	present	120.82	-0.19	excluded	absent	73.16	-0.69	excluded	absent	5.29
201333_s_at	included	present	2130.56	3.51	included	present	744.55	-0.57	included	present	1353.45
201334_s_at	included	present	2058.2	0.41	included	present	977.23	-1.38	included	present	1030.29
201335_s_at	included	present	333.14	-0.39	included	present	125.75	-1.57	included	present	153
201336_at	included	present	2564.79	0.28	included	present	1795.47	-0.82	included	present	649.11
201337_s_at	included	present	3174.4	-0.21	included	present	1198.72	-2.11	included	present	1242.3
201338_x_at	included	present	7707.95	0.57	included	present	12189.71	1.91	included	present	10757.46
201339_s_at	included	present	2520.16	-0.3	included	present	1852.9	-0.5	included	present	1662.93
201340_s_at	excluded	absent	67.1	-0.51	excluded	absent	75.02	-0.49	excluded	absent	33.66
201341_at	included	present	478.64	-0.5	included	present	337.27	-0.53	included	present	314.88
201342_at	included	present	567.6	-0.37	included	present	1103.31	1.58	included	present	608.83
201343_at	included	present	4549.93	-1.03	included	present	4328.38	-1.16	included	present	7443.47
201344_at	included	present	772.34	0.03	included	present	690.96	-0.25	included	present	1814.74
201345_s_at	included	present	3218.33	0.04	included	present	3260.66	0.09	included	present	5805.54
201346_at	included	present	477.19	-0.85	included	present	448.65	-0.89	included	present	429.6
201347_x_at	included	present	2445.28	0.26	included	present	1686.1	-0.16	included	present	2195.58
201348_at	included	present	3447.64	-0.3	included	present	590.53	-0.48	included	present	740.93
201349_at	included	present	369.92	-0.51	included	absent	111.25	-0.8	included	present	289.07
201350_at	excluded	absent	198.58	0.16	included	present	144.54	-0.38	excluded	absent	33.75
201351_s_at	included	present	2208.96	-0.57	included	present	3021.85	0.46	included	present	2689.77
201352_at	included	present	2340.25	0.23	included	present	2164.96	-0.05	included	present	1850.89
201353_s_at	included	present	136.74	-0.55	included	absent	98.05	-0.91	included	absent	92.42
201354_s_at	included	present	2085.97	-0.55	included	present	2162.26	-0.48	included	present	2039.95

[illegible]

201680_x_at	included	present	971.46	-0.63	included	present	4340.41	5.27	included	present	1981	1.14	included	present
201681_s_at	included	present	7828.65	-0.27	included	present	8566.29	-0.13	included	present	14338.82	0.91	included	present
201682_at	included	present	1677.01	-0.76	included	present	3273.19	1.72	included	present	2699.31	0.83	included	present
201683_x_at	included	present	2727.21	3.2	included	present	2526.74	2.67	included	present	1500.21	-0.05	included	present
201684_s_at	included	present	1022.82	-1.64	included	present	1666.6	0.65	included	present	1062.8	-1.5	included	present
201685_s_at	included	present	1710.02	-0.05	included	present	2062.23	0.92	included	present	1336.45	-1.07	included	present
201686_x_at	included	present	1511.94	1.81	included	present	901.25	-1.21	included	present	1743.61	2.95	included	present
201687_s_at	included	present	2472.83	-0.61	included	present	2149.57	-1.15	included	present	2185.78	-1.09	included	present
201688_s_at	included	present	247.86	-0.64	included	absent	70.99	-1.04	included	absent	121.66	-0.92	included	present
201689_s_at	included	present	1421.09	-0.34	included	present	441.39	-1.04	included	present	623.48	-0.91	included	present
201690_s_at	included	present	1363.26	-0.83	included	present	1130.5	-0.91	included	present	609.11	-1.07	included	present
201691_s_at	included	present	398.91	-0.59	included	present	178.88	-0.99	included	present	212.99	-0.93	included	present
201692_at	included	present	1128.66	1.48	included	present	1640.47	2.76	included	present	960.55	1.06	included	present
201693_s_at	included	present	914.94	0.66	included	absent	58.39	-0.7	included	present	62.75	-0.69	included	present
201694_s_at	included	present	31475.91	1.23	included	present	2185.68	-0.8	included	present	2043.88	-0.81	included	present
201695_s_at	included	present	265.81	-0.34	included	present	801.58	0.75	included	present	688.28	0.52	included	present
201696_at	included	present	1522.41	-1.39	included	present	2792.75	0.45	included	present	1081.76	-2.03	included	present
201697_s_at	included	present	210.75	-0.85	included	present	1037.73	3.99	included	present	394.07	0.22	included	present
201698_s_at	included	present	3937.01	-0.54	included	present	7007.5	1.78	included	present	8202.5	2.69	included	present
201699_at	included	present	3355.11	-0.16	included	present	4115.5	0.66	included	present	3246.99	-0.27	included	present
201700_at	included	present	4656.67	0.83	included	present	326.84	-1.39	included	present	1303.26	-0.89	included	present
201701_s_at	included	present	1313.58	0	included	present	1050.28	-0.31	included	present	1447.88	0.16	included	present
201702_s_at	included	present	3649.09	1.99	included	present	6790.34	6.5	included	present	3325.14	1.52	included	present
201703_s_at	included	present	590.04	1.23	included	present	1043.26	4.72	included	present	588.07	1.22	included	present
201704_at	included	present	1711.64	0.13	included	present	1661.42	0.08	included	present	655.19	-1.06	included	present
201705_at	included	present	1308.88	-0.48	included	present	2326.32	1.43	included	present	2278.1	1.34	included	present
201706_s_at	included	present	427.96	-0.37	included	present	727.26	0.74	included	present	277.07	-0.93	included	present
201707_at	included	present	766.66	0.38	included	present	543.58	-0.7	included	present	727.87	0.19	included	present
201708_s_at	included	present	459.16	0.17	included	present	1206.35	2.04	included	present	656.11	0.66	included	present
201709_s_at	included	present	870.44	-0.2	included	present	4296.65	2.69	included	present	2570.8	1.23	included	present
201710_at	excluded	absent	16.05	-0.14	excluded	absent	26.8	0.29	excluded	absent	95.64	3.03	excluded	absent
201711_x_at	included	present	3519.7	-0.94	included	present	3270.32	-1.03	included	present	4401.17	-0.63	included	present
201712_s_at	included	present	477.42	-0.29	included	present	274.38	-1.46	included	present	598.84	0.41	included	present
201713_s_at	included	present	1988.24	-0.2	included	present	1499.9	-0.94	included	present	2156.38	0.06	included	present
[illegible]														

202039_at	included	present	841.64	-0.05	included	present	755.38	-0.21	included	present	116.8	-1.38	included	present	293.57	-1.06	[illegible]
202040_s_at	included	present	679.57	-0.42	included	present	1087.87	0.42	included	present	438.13	-0.91	included	present	1249.55	0.75	[illegible]
202041_s_at	included	present	872.35	1.48	included	present	2004.51	6.84	included	present	772.14	1	included	present	369.99	-0.91	[illegible]
202042_at	included	present	1392.3	0.63	included	present	2229.02	2.74	included	present	1334.19	0.48	included	present	865.49	-0.7	[illegible]
202043_s_at	included	present	4713.58	0.85	included	present	5986.99	1.62	included	present	8422.02	3.09	included	present	4310.24	0.61	[illegible]
202044_at	included	present	138.67	-0.15	excluded	absent	73.7	-0.85	excluded	absent	124.03	-0.31	included	present	142.92	-0.11	[illegible]
202045_s_at	included	present	294.24	0.32	included	present	184.86	-0.58	included	present	227.29	-0.23	included	present	224.04	-0.26	[illegible]
202046_s_at	included	present	2328.98	1.68	included	present	1943.03	1.05	included	present	2048.96	1.23	included	present	2035.51	1.21	[illegible]
202047_s_at	included	present	683.91	0.18	included	present	365.15	-0.69	included	present	315.93	-0.83	included	present	442.9	-0.48	[illegible]
202048_s_at	included	present	3808.94	0.12	included	present	1653.84	-0.83	included	present	1253.22	-1.01	included	present	3613.36	0.03	[illegible]
202049_s_at	included	present	687.97	0.42	included	present	660.43	0.33	included	present	367.48	-0.64	included	present	762.98	0.66	[illegible]
202050_s_at	included	present	634.79	1.69	included	present	1004.08	5.18	included	present	601.17	1.38	included	present	791.37	3.17	[illegible]
202051_s_at	included	present	1088.51	-0.07	included	present	1408.29	0.83	included	present	858.63	-0.73	included	present	1788.68	1.92	[illegible]
202052_s_at	included	present	1441.39	0.87	included	present	371.11	-0.38	included	present	259.07	-0.51	included	present	445.56	-0.29	[illegible]
202053_s_at	included	present	4614.03	-0.19	included	present	4129.04	-0.31	included	present	3534.87	-0.47	included	present	4272.56	-0.27	[illegible]
202054_s_at	included	present	2707.18	-0.32	included	present	2118.85	-0.51	included	present	1858.73	-0.59	included	present	2592.35	-0.36	[illegible]
202055_at	included	present	1109.88	0.27	included	present	1164.77	0.4	included	present	717.39	-0.68	included	present	1012.99	0.04	[illegible]
202056_at	included	present	941.01	0.21	included	present	646.56	-0.5	included	present	809.85	-0.1	included	present	858.39	0.01	[illegible]
202057_at	included	present	567.02	0.67	included	present	671.99	1.4	included	present	576.29	0.74	included	present	513.67	0.3	[illegible]
202058_s_at	included	present	1950.06	0.66	included	present	1379.01	-0.56	included	present	1785.09	0.31	included	present	1355.35	-0.61	[illegible]
202059_s_at	included	present	902.97	-0.05	included	present	899.38	-0.06	included	present	944.67	0.06	included	present	748.53	-0.46	[illegible]
202060_at	included	present	636.33	-0.17	included	present	1492.17	2.57	included	present	528.82	-0.52	included	present	728.57	0.12	[illegible]
202061_s_at	included	present	1548.42	0.18	included	present	980.14	-0.92	included	present	988.71	-0.9	included	present	1566.79	0.22	[illegible]
202062_s_at	included	present	2123.23	2.87	included	present	751.65	-1.37	included	present	931.7	-0.81	included	present	1326.08	0.4	[illegible]
202063_s_at	excluded	absent	45.25	-0.4	excluded	absent	44	-0.43	excluded	absent	76.48	0.39	included	present	100.19	0.98	[illegible]
202064_s_at	included	present	2306.95	-0.52	included	present	1650.06	-1.26	included	present	1570.5	-1.35	included	present	2431.18	-0.39	[illegible]
202065_s_at	included	present	527	1.6	excluded	absent	319.32	0.02	included	present	332.72	0.12	excluded	absent	284.63	-0.25	[illegible]
202066_at	included	present	776.75	-0.8	included	present	486.86	-1.94	included	present	734.23	-0.97	included	present	890.04	-0.35	[illegible]
202067_s_at	excluded	absent	141.69	-0.37	excluded	absent	135.79	-0.41	excluded	absent	93	-0.73	included	present	189.43	-0.02	[illegible]
202068_s_at	included	present	263.87	-0.1	included	present	168.67	-0.38	included	absent	165.34	-0.39	included	present	317.36	0.05	[illegible]
202069_s_at	included	present	1606.67	-0.45	included	present	1144.32	-0.72	included	present	1186.65	-0.69	included	present	997.2	-0.8	[illegible]
202070_s_at	included	present	541.58	-0.87	included	present	313.32	-1.14	included	present	386.65	-1.05	included	present	352.67	-1.09	[illegible]
202071_at	included	present	1203.67	-0.3	included	absent	120.2	-1.57	included	present	217.29	-1.45	included	present	330.1	-1.32	[illegible]
202072_at	included	present	3147.56	-0.07	included	present	2854.72	-0.41	included	present	6335.41	3.63	included	present	6154.95	3.42	[illegible]
202073_at	included	present	1573.11	0.27	included	present	476.89	-0.65	included	present	483.11	-0.64	included	present	682.92	-0.48	[illegible]
202074_s_at	included	present	2394.62	-0.24	included	present	3488.7	-0.11	included	present	1247.3	-0.38	included	present	1609.44	-0.34	[illegible]
202075_s_at	included	present	675.26	-0.21	included	present	465.28	-0.53	included	absent	150.42	-1	included	absent	25.76	-1.19	[illegible]
202076_at	included	present	2785.27	0.15	included	present	1838.71	-0.93	included	present	1868.7	-0.9	included	present	1584.71	-1.22	[illegible]
202077_at	included	present	3599.9	-0.22	included	present	4724.9	0.33	included	present	3826.81	-0.11	included	present	2676.53	-0.68	[illegible]
202078_at	included	present	1505.59	-0.45	included	present	2423.68	0.36	included	present	1945.91	-0.06	included	present	2565	0.49	[illegible]
202079_s_at	included	present	1184.06	1.36	included	present	1136.99	1.21	included	present	1088.68	1.06	included	present	698.84	-0.16	[illegible]
202080_s_at	included	present	1488.14	0.58	included	present	1511.26	0.63	included	present	961.42	-0.55	included	present	886.04	-0.71	[illegible]
202081_at	included	present	2504.84	0.28	included	present	1196.78	-0.43	included	present	1229.19	-0.41	included	present	1581.74	-0.22	[illegible]
202082_s_at	included	present	1445.04	-0.75	included	present	1928.45	-0.3	included	present	1448.43	-0.75	included	present	6008.49	3.54	[illegible]
202083_s_at	included	present	174.4	0.01	excluded	absent	68.26	-0.81	included	present	97.15	-0.59	included	present	466.28	2.27	[illegible]
202084_s_at	included	present	6028.41	-0.63	included	present	8669.17	0.28	included	present	5559.22	-0.79	included	present	21787.73	4.79	[illegible]
202085_at	included	present	263.51	-1.33	included	present	253.42	-1.34	included	present	340.25	-1.24	included	present	1497.16	0.16	[illegible]
202086_at	included	present	529.4	0.61	included	present	222.1	-0.52	included	absent	80.38	-1.04	included	absent	153.95	-0.77	[illegible]
202087_s_at	included	present	4166.32	-0.28	included	present	4224.77	-0.26	included	present	3482.34	-0.55	included	present	1968.99	-1.13	[illegible]
202088_at	included	present	3559.42	0.23	included	present	7161.57	1.79	included	present	4171.83	0.49	included	present	4847.04	0.79	[illegible]
202089_s_at	included	present	3141.14	1.47	included	present	4720.73	2.85	included	present	3938.77	2.17	included	present	5111.35	3.2	[illegible]
202090_s_at	included	present	2343.99	-0.43	included	present	1989.77	-0.61	included	present	1518.47	-0.85	included	present	2155.91	-0.53	[illegible]
202091_at	included	present	1565.16	2.12	included	present	671.48	-0.92	included	present	1452.32	1.73	included	present	507.25	-1.47	[illegible]
202092_s_at	included	present	406.66	1	included	present	337.03	0.36	included	present	446.98	1.37	included	present	226.38	-0.66	[illegible]
202093_s_at	included	present	3923.23	-0.14	included	present	7752.22	3.51	included	present	3437.98	-0.6	included	present	4943.49	0.83	[illegible]
202094_at	excluded	absent	44.7	-0.02	included	present	287.84	3.52	included	present	639.48	8.63	included	present	290.56	3.56	[illegible]
202095_s_at	excluded	absent	62.28	-0.35	included	present	4232.57	3.95	included	present	6545.07	6.33	included	present	2460.9	2.12	[illegible]
202096_s_at	excluded	absent	208.11	0.2	excluded	absent	38.51	-0.85	excluded	absent	77.45	-0.61	excluded	absent	56.57	-0.74	[illegible]
202097_at	included	present	2458.36	-1.13	included	present	7349.05	3.48	included	present	4731.37	1.02	included	present	6186.55	2.39	[illegible]
202098_s_at	included	present	243.6	1.19	included	present	181.16	0.48	excluded	absent	55.47	-0.95	included	present	125.85	-0.15	[illegible]
202099_s_at	excluded	absent	74.98	-0.47	excluded	absent	102.54	-0.03	excluded	absent	41.8	-1.01	excluded	absent	146.05	0.68	[illegible]
202100_at	included	present	751.29	-0.18	included	present	702.73	-0.37	included	present	335.94	-1.78	included	present	328.1	-1.81	[illegible]
202101_s_at	included	present	383.28	-0.44	included	present	190.62	-1.51	included	absent	89.05	-2.07	included	present	137.72	-1.8	[illegible]
202102_s_at	included	present	5429.04	4.83	included	present	8481.17	9.44	included	present	4174.29	2.93	included	present	6670.8	6.7	[illegible]
202103_at	excluded	absent	99.67	0.41	included	present	123.7	0.82	excluded	absent	65.91	-0.18	excluded	absent	67.74	-0.15	[illegible]
202104_s_at	included	present	599.51	0.94	included	present	617.23	1.06	included	present	279.78	-1.38	included	present	411.63	-0.42	[illegible]
202105_at	included	present	1288.24	-0.98	included	present	2736.56	0.53	included	present	1968.1	-0.27	included	present	1886.47	-0.36	[illegible]
202106_at	included	present	5150.99	1.41	included	present	5274.24	1.52	included	present	3073.66	-0.47	included	present	3989.59	0.36	[illegible]
202107_s_at	included	present	322.54	1.02	included	present	1755.45	14.84	included	present	1588.36	13.23	included	present	1578.99	13.14	[illegible]
202108_at	included	present	517.62	-0.12	included	present	640.88	-0.01	included	present	165.5	-0.45	included	present	248.75	-0.38	[illegible]
202109_at	included	present	224.29	0.76	included	present	671.44	6.25	included	absent	92.62	-0.85	included	present	157.57	-0.05	[illegible]
202110_at	included	present	3494.38	-0.85	included	present	2761.15	-1.01	included	present	4381.49	-0.66	included	present	3695.44	-0.81	[illegible]
202111_at	included	present	211.3	-0.25	included	present	523.43	2.19	included	present	178.33	-0.5	included	absent	60.94	-1.42	[illegible]
202112_at	included	present	1581.88	-0.32	included	present	2161.34	-0.19	included	present	379.94	-0.58	included	present	175.47	-0.62	[illegible]
202113_s_at	included	present	3633.18	-0.02	included	present	3745.49	0.08	included	present	6423.72	2.35	included	present	4159.19	0.43	[illegible]
202114_at	included	present	877.79	-0.36	included	present	893.21	-0.33	included	present	1712.31	1.23	included	present	1565.36	0.95	[illegible]
202115_s_at	excluded	absent	104.59	-0.58	included	present	350.98	3.76	excluded	absent	14.73	-2.16	excluded	absent	41.39	-1.69	[illegible]
202116_at	included	present	501.35	-0.2	included	present	581.58	0.15	included	present	412.97	-0.59	included	present	305.06	-1.06	[illegible]
202117_at	included	present	1656.24	0.65	included	present	1210.72	-0.26	included	present	441.39	-1.83	included	present	999.61	-0.69	[illegible]
202118_s_at	included	present	2617.77	1.33	included	present	1194.94	-0.83	included	present	2268.11	0.8	included	present	1994.85	0.39	[illegible]
202119_s_at	included	present	1507.87	1.09	included	present	1002.84	-0.21	included	present	870.28	-0.55	included	present	1000.42	-0.22	[illegible]
202120_x_at	included	present	472.2	1.32	included	present	344.06	0.06	included	present	241.1	-0.95	included	present	422.63	0.83	[illegible]
202121_s_at	included	present	1836.49	-0.44	included	present	3378.94	1.59	included	present	2138.83	-0.04	included	present	1770.29	-0.52	[illegible]
202122_s_at	included	present	200.23	-0.26	excluded	absent	36.13	-1.42	excluded	absent	131.79	-0.74	excluded	absent	38.98	-1.4	[illegible]
202123_s_at	included	present	538.26	0.09	included	present	277.46	-0.86	included	present	564.36	0.18	included	present	503.66	-0.04	[illegible]
202124_s_at	included	present	3110.47	0.46	included	present	3261.37	0.58	included	present	1923.71	-0.42	included	present	1372.29	-0.84	[illegible]
202125_s_at	included	present	6338.05	0.57	included	present	5374.77	0.26	included	present	2971.37	-0.54	included	present	1842.9	-0.91	[illegible]
202126_at	included	present	871.08	-0.78	included	present	742.63	-1.3	included	present	1118.68	0.21	included	present	1489.78	1.69	[illegible]
202127_at	included	present	196.49	-1.43	included	present	347.28	-0.67	included	present	418.12	-0.31	included	present	757.06	1.4	[illegible]
202128_at	included	present	760.24	1.17	included	present	1654.22	6.61	included	present	651.61	0.51	included	present	706.03	0.84	[illegible]
202129_s_at	included	present	588.36	-0.22	included	present	953.92	0.58	included	present	609.72	-0.18	included	present	584.67	-0.23	[illegible]
202130_at	included	present	875.99	-0.28	included	present	1654.57	0.95	included	present	722.07	-0.52	included	present	977.9	-0.12	[illegible]
202131_s_at	included	present	250.38	0.5	included	present	324.19	1.15	included	present	172.42	-0.19	included	present	282.66	0.78	[illegible]
202132_at	included	present	3407.85	0.86	included	present	339.52	-0.95	included	present	399.78	-0.92	included	present	257.16	-1	[illegible]
202133_at	included	present	4168.87	0.35	included	present	432.2	-0.83	included	present	488.07	-0.81	included	present	256.96	-0.88	[illegible]
202134_s_at	included	present	2586.32	0.99	included	present	217.76	-0.9	included	present	290.2	-0.84	included	present	154.96	-0.95	[illegible]
202135_s_at	included	present	347.99	0.1	included	present	358.4	0.2	included	present	222.7	-1.18	included	present	248.09	-0.93	[illegible]
202136_at	included	present	2691.2	-0.8	included	present	1909.16	-1.4	included	present	1856.49	-1.44	included	present	2620.21	-0.86	[illegible]
202137_s_at	included	present	1515.07	1.18	included	present	538.64	-1.52	included	present	892.03	-0.54	included	present	915.67	-0.48	[illegible]
202138_x_at	included	present	1319.17	0.93	included	present	2828.89	4.42	included	present	2642.77	3.99	included	present	1284.69	0.85	[illegible]
202139_at	included	present	263.16	0.11	included	present	226.67	-0.16	included	absent	94.46	-1.14	included	present	67.39	-1.35	[illegible]
202140_s_at	included	present	340.9	-0.11	included	present	661.76	2.04	included	absent	164.3	-1.29	included	present	291.81	-0.44	[illegible]
202141_s_at	included	present	1792.19	0.22	included	present	2419.36	1.17	included	present	1114.86	-0.81	included	present	1300.86	-0.52	[illegible]
202142_at	included	present	1635.96	0.68	included	present	2462.62	2.13	included	present	1050.05	-0.35	included	present	973.81	-0.48	[illegible]
202143_s_at	included	present	823.15	0.32	included	present	780.38	0.18	included	present	323.55	-1.3	included	present	443.8	-0.91	[illegible]
202144_s_at	included	present	1115.03	-0.37	included	present	4349.57	4.61	included	present	2624.89	1.96	included	present	1362.67	0.01	[illegible]
202145_at	included	present	227.04	0.35	excluded	absent	8.03	-1.38	included	present	121.3	-0.48	excluded	absent	45.7	-1.08	[illegible]
202146_at	included	present	1057.46	-0.79	included	present	1227.08	-0.41	included	present	764.37	-1.45	included	present	1242.47	-0.38	[illegible]
202147_s_at	included	present	720.35	-1.15	included	present	1493.11	0.77	included	present	940.09	-0.6	included	present	856.74	-0.81	[illegible]
202148_s_at	excluded	absent	89.88	-0.24	included	present	138.32	0.45	included	present	114.01	0.1	included	present	101.11	-0.08	[illegible]
202149_at	included	present	335.19	-0.58	included	absent	130.67	-1.05	included	present	393.8	-0.45	included	present	294.59	-0.67	[illegible]
202150_s_at	included	present	131.92	-0.8	included	present	134.71	-0.79	included	present	202.04	-0.57	included	present	196.6	-0.59	[illegible]
202151_s_at	included	present	276.58	-0.53	included	present	347.62	-0.38	included	present	242.15	-0.61	included	present	137.71	-0.83	[illegible]
202152_x_at	included	present	362.49	1	included	present	214.79	-0.47	included	present	197.34	-0.64	included	present	252.01	-0.1	[illegible]
202153_s_at	included	present	544.26	0.77	included	present	890.8	3.03	included	present	832.42	2.65	included	present	2198.6	11.55	[illegible]
202154_x_at	included	present	9558.6	1.7	included	present	21901.74	6.03	included	present	4267.66	-0.16	included	present	2673.2	-0.72	[illegible]
202155_s_at	included	present	1908.8	-0.14	included	present	2632.04	0.43	included	present	2224.75	0.11	included	present	1699.41	-0.3	[illegible]
202156_s_at	included	present	1311.67	2.74	included	present	149.41	-1.24	included	present	277.56	-0.8	included	absent	2.33	-1.74	[illegible]
202157_s_at	included	present	6374.24	2.4	included	present	420.86	-1.14	included	present	689.56	-0.98	included	absent	76.14	-1.34	[illegible]
202158_s_at	included	present	3124.43	1.51	included	present	319.65	-1.5	included	present	604.85	-1.19	included	present	154.27	-1.68	[illegible]
202159_at	included	absent	164.87	-1.44	included	present	750.12	2.52	included	present	317.79	-0.4	included	present	652.02	1.86	[illegible]
202160_at	included	present	3742.43	1.06	included	present	3695.03	1.01	included	present	3823.51	1.15	included	present	3256.19	0.52	[illegible]
202161_at	included	present	237.79	-0.44	included	present	555.67	1.67	included	present	349.42	0.3	included	present	195	-0.72	[illegible]
202162_s_at	included	present	1486.34	-0.59	included	present	2538.64	1.25	included	present	3539.65	3.01	included	present	3194.11	2.4	[illegible]
202163_s_at	included	present	1209.85	-1.7	included	present	3189.23	0.32	included	present	3660.58	0.8	included	present	3731.33	0.87	[illegible]
202164_s_at	included	present	358.51	-0.67	included	present	516.64	0.36	included	present	1107.27	4.19	included	present	868.05	2.64	[illegible]
202165_at	included	present	1406.13	-0.41	included	present	1454.04	-0.33	included	present	1091.9	-0.92	included	present	1054.72	-0.98	[illegible]
202166_s_at	included	present	412.4	-0.72	included	present	461.18	-0.61	included	present	459.01	-0.62	included	present	358.48	-0.84	[illegible]
202167_s_at	included	present	552.49	0.25	included	present	542.43	0.18	included	present	426.3	-0.53	included	present	399.1	-0.7	[illegible]
202168_at	included	present	1874.67	0.1	included	present	2365	0.43	included	present	3263.89	1.02	included	present	1778.77	0.04	[illegible]
202169_s_at	included	present	1448.14	-0.63	included	present	4646.36	1.78	included	present	3966.16	1.27	included	present	2529.92	0.19	[illegible]
202170_s_at	included	present	819.33	-0.88	included	present	2976.56	1.67	included	present	2296.4	0.87	included	present	1247.93	-0.37	[illegible]
202171_at	included	present	1672.02	1.16	included	present	1996.92	1.86	included	present	2636.99	3.24	included	present	4169.05	6.55	[illegible]
202172_at	included	present	1642.15	0.02	included	present	2097.7	0.58	included	present	2014.85	0.48	included	present	3110.77	1.85	[illegible]
202173_s_at	included	present	678.38	-0.29	included	present	657.47	-0.36	included	present	1014.02	0.8	included	present	1743.83	3.16	[illegible]
202174_s_at	included	present	3762.71	-0.03	included	present	4022.1	0.07	included	present	5960.56	0.79	included	present	6961.83	1.16	[illegible]
202175_at	excluded	absent	234.11	1.54	excluded	absent	113.21	0.05	excluded	absent	114.41	0.06	excluded	absent	15.93	-1.15	[illegible]
202176_at	included	present	503.33	-0.61	included	present	984.7	3.2	included	present	469.2	-0.88	included	present	218.13	-2.86	[illegible]
202177_at	included	present	178.49	1.15	excluded	absent	35.47	-0.87	excluded	absent	39.31	-0.82	excluded	absent	89.35	-0.11	[illegible]
202178_at	included	present	439.27	-0.52	included	present	651.62	-0.1	included	absent	270.22	-0.86	included	absent	125.64	-1.15	[illegible]
202179_at	included	present	675.05	-0.04	included	present	1766.05	1.12	included	present	707.19	0	included	present	664.41	-0.05	[illegible]
202180_s_at	excluded	absent	179.29	0.24	excluded	absent	73.8	-0.67	excluded	absent	18.63	-1.15	excluded	absent	91.23	-0.52	[illegible]
202181_at	included	present	1529.17	-0.08	included	present	864.75	-0.96	included	present	547.77	-1.37	included	present	4215.7	3.45	[illegible]
202182_at	included	absent	137	-1.57	included	present	490.53	2.17	included	present	256.74	-0.3	included	absent	150.23	-1.43	[illegible]
202183_s_at	included	present	150.83	-0.01	included	present	602.3	4.85	included	present	610.21	4.94	included	present	207.56	0.6	[illegible]
202184_s_at	included	present	700.45	-0.63	included	present	1367.53	1.94	included	present	1539.37	2.6	included	present	1531.16	2.57	[illegible]
202185_at	included	present	1130.82	4.21	included	present	550.52	1.08	included	present	294.08	-0.3	included	present	532.15	0.98	[illegible]
202186_x_at	excluded	absent	13.78	-0.49	excluded	absent	13.25	-0.5	excluded	absent	3.39	-0.77	excluded	absent	12.09	-0.53	[illegible]
202187_s_at	included	present	640.69	-1.51	included	present	287.11	-2.04	included	present	602.17	-1.57	included	present	2307.78	0.95	[illegible]
202188_at	included	present	1013.75	-0.37	included	present	2256.37	1.81	included	present	3158.94	3.39	included	present	872.21	-0.61	[illegible]
202189_x_at	included	present	1693.77	0.89	included	present	758.74	-0.64	included	present	3233.09	3.41	included	present	2814.84	2.72	[illegible]
202190_at	included	present	295.67	1.08	excluded	absent	227.08	0.43	included	present	379.05	1.87	excluded	absent	273.68	0.87	[illegible]
202191_s_at	included	present	20183.43	1.95	included	present	708.56	-1.04	included	present	1249.41	-0.95	included	present	83.22	-1.13	[illegible]
202192_s_at	included	present	11644.49	1.43	included	present	938.33	-0.78	included	present	389.06	-0.9	included	absent	71.76	-0.96	[illegible]
202193_at	included	present	2692.48	-0.09	included	present	4392.97	0.96	included	present	2255.16	-0.36	included	present	5471.58	1.62	[illegible]
202194_at	included	present	637.53	-0.62	included	present	1370.15	0.79	included	present	254.1	-1.36	included	present	1165.97	0.39	[illegible]
202195_s_at	included	present	1104.68	-0.1	included	present	1090.27	-0.12	included	present	672.03	-0.6	included	present	1746.58	0.64	[illegible]
202196_s_at	included	present	6182	0.5	included	present	1447.4	-0.54	included	present	414.7	-0.76	included	present	476.36	-0.75	[illegible]
202197_at	included	present	1398.13	-0.77	included	present	1744.27	-0.08	included	present	1229.33	-1.1	included	present	1701.11	-0.16	[illegible]
202198_s_at	included	present	162.5	-0.95	included	absent	63.9	-1.8	included	present	204.63	-0.59	included	present	275.88	0.03	[illegible]
202199_s_at	included	present	519.46	-0.67	included	present	1535.69	2.48	included	present	1508.25	2.4	included	present	1752.99	3.16	[illegible]
202200_s_at	included	present	599.75	-0.66	included	present	2277.22	2.1	included	present	3841.47	4.67	included	present	2686.34	2.77	[illegible]
202201_at	included	present	460.58	-0.08	included	present	199.29	-0.61	included	present	188.25	-0.63	included	absent	105.34	-0.8	[illegible]
202202_s_at	included	present	8557.93	3.48	included	present	466.47	-0.66	included	present	275.55	-0.76	included	present	784.11	-0.5	[illegible]
202203_s_at	included	present	999.44	-0.24	included	present	168.82	-1.4	included	present	741.82	-0.6	included	present	267.11	-1.26	[illegible]
202204_s_at	included	present	400.01	1.17	included	absent	177.29	-0.45	included	present	286.61	0.35	included	absent	60.82	-1.3	[illegible]
202205_at	excluded	absent	6.6	-0.96	excluded	absent	18	-0.69	excluded	absent	26.08	-0.5	excluded	absent	8.95	-0.9	[illegible]
202206_at	included	present	710.62	1.46	included	present	313.46	0.09	included	present	386.29	0.34	included	present	464.87	0.61	[illegible]
202207_at	included	present	1610.52	1.89	included	present	838.36	0.45	included	present	803.22	0.39	included	present	965.8	0.69	[illegible]
202208_s_at	included	present	461.92	1.5	included	present	399.4	1.09	included	present	217.97	-0.1	excluded	absent	163.11	-0.46	[illegible]
202209_at	included	present	2532.57	-0.17	included	present	4081.05	1.66	included	present	5422.85	3.25	included	present	3055.61	0.45	[illegible]
202210_x_at	excluded	absent	26.06	-1.36	excluded	absent	280.93	2.73	excluded	absent	67.28	-0.7	included	present	100.45	-0.16	[illegible]
202211_at	included	present	748.95	-0.65	included	present	912.9	-0.45	included	present	721.79	-0.68	included	present	2330.79	1.21	[illegible]
202212_at	included	absent	231.6	-0.39	included	present	716.1	5.39	included	present	265.56	0.02	included	absent	93.06	-2.04	[illegible]
202213_s_at	included	present	1132.71	1.68	included	present	656.61	-0.33	included	present	980.52	1.04	included	present	1741.78	4.24	[illegible]
202214_s_at	included	present	1486.08	-0.31	included	present	651.15	-1.93	included	present	1018.66	-1.22	included	present	2156.24	0.98	[illegible]
202215_s_at	included	present	2926.77	-1.57	included	present	4274.44	-0.16	included	present	2864.71	-1.63	included	present	5213.62	0.83	[illegible]
202216_x_at	included	present	371	-0.04	included	present	355.84	-0.15	included	present	247.29	-0.93	included	present	407.75	0.22	[illegible]
202217_at	included	present	2832.09	0.02	included	present	3911.51	0.62	included	present	2047.73	-0.42	included	present	3766.85	0.54	[illegible]
202218_s_at	excluded	absent	255.42	-0.04	included	present	684.14	1.35	excluded	absent	34.19	-0.76	included	present	147.29	-0.39	[illegible]
202219_at	included	present	743.57	-0.54	included	present	906.2	-0.44	included	present	609.75	-0.63	included	present	161.81	-0.93	[illegible]
202220_at	included	present	2717.48	-2.08	included	present	3478.45	-1.46	included	present	5833.98	0.46	included	present	9075.9	3.11	[illegible]
202221_s_at	included	present	1789.71	1.69	included	present	1088.46	-0.23	included	present	710.97	-1.26	included	present	1889.87	1.96	[illegible]
202222_s_at	excluded	absent	67.55	-0.4	excluded	absent	77.25	-0.39	excluded	absent	18.24	-0.43	excluded	absent	71.23	-0.39	[illegible]
202223_at	included	present	1526.21	-0.35	included	present	1168.67	-0.67	included	present	2351.34	0.39	included	present	2938.95	0.92	[illegible]
202224_at	included	present	907.01	4.14	included	present	658.11	2.23	included	present	987.58	4.75	included	present	549.5	1.4	[illegible]
202225_at	included	present	1283.34	0.21	included	present	1759.8	1.48	included	present	1291.94	0.23	included	present	1033.92	-0.46	[illegible]
202226_s_at	included	present	1633.15	2.54	included	present	811	-0.19	included	present	1812.16	3.13	included	present	874.33	0.02	[illegible]
202227_s_at	included	present	1934.54	-1.09	included	present	2692.91	-0.04	included	present	3346.76	0.85	included	present	3883.71	1.59	[illegible]
202228_s_at	included	present	3865.18	-0.78	included	present	2393.46	-1.32	included	present	1815.19	-1.53	included	present	4211.25	-0.65	[illegible]
202229_s_at	excluded	absent	12.08	-0.9	excluded	absent	72.84	1.14	excluded	absent	68.5	0.99	excluded	absent	4.56	-1.15	[illegible]
202230_s_at	included	present	1796.76	0.46	included	present	3133.56	4	included	present	2670.4	2.77	included	present	5224.93	9.53	[illegible]
202231_at	included	present	4248.54	-0.52	included	present	9114.03	1.5	included	present	9199.31	1.54	included	present	5443.05	-0.03	[illegible]
202232_s_at	included	present	2971.79	-0.28	included	present	4851.2	0.9	included	present	4893.64	0.93	included	present	3820.98	0.26	[illegible]
202233_s_at	included	present	5741.28	-0.88	included	present	18082.85	2.19	included	present	7282.58	-0.5	included	present	13273.15	0.99	[illegible]
202234_s_at	included	present	513.52	-0.71	included	present	500.17	-0.73	included	present	1260.48	0.35	included	present	308.05	-1.01	[illegible]
202235_at	included	present	186.65	-0.76	included	present	297.32	-0.21	included	present	640.11	1.5	included	absent	109.78	-1.14	[illegible]
202236_s_at	included	present	517.19	-0.78	included	present	699.39	-0.55	included	present	2544.35	1.75	included	present	443.32	-0.87	[illegible]
202237_at	included	present	2031.02	-0.34	included	present	1338.49	-0.38	included	present	1557.33	-0.37	included	present	569.86	-0.43	[illegible]
202238_s_at	included	present	252.82	-0.26	excluded	absent	303.54	-0.24	included	present	214.86	-0.27	excluded	absent	123.07	-0.3	[illegible]
202239_at	included	present	944.31	0.24	included	absent	280.97	-0.9	included	present	1044.76	0.42	included	present	329.89	-0.82	[illegible]
202240_at	excluded	absent	62.81	0.62	excluded	absent	9.43	-0.62	excluded	absent	83.5	1.1	excluded	absent	3.98	-0.74	[illegible]
202241_at	included	present	362.49	-0.62	included	present	416.4	-0.58	included	present	674.39	-0.36	included	present	559.61	-0.46	[illegible]
202242_at	included	present	1500.51	-0.49	included	present	1763.42	-0.41	included	present	2028.57	-0.33	included	absent	56.95	-0.93	[illegible]
202243_s_at	included	present	7796.77	1.31	included	present	8118.42	1.56	included	present	11177.68	3.96	included	present	8195.46	1.62	[illegible]
202244_at	included	present	6610.48	-0.17	included	present	9833.53	1.96	included	present	10065.28	2.12	included	present	8093.49	0.81	[illegible]
202245_at	included	present	609.7	0.07	included	present	516.27	-0.17	included	present	167.66	-1.04	included	present	241.9	-0.86	[illegible]
202246_s_at	included	present	1997.26	0.5	included	present	3749.04	2.46	included	present	3830.8	2.55	included	present	4076.05	2.83	[illegible]
202247_s_at	included	present	105.81	1.19	excluded	absent	40.56	-0.46	excluded	absent	45.52	-0.33	excluded	absent	36.65	-0.56	[illegible]
202248_at	excluded	absent	11.97	-0.84	excluded	absent	131.01	1.71	excluded	absent	71.35	0.43	excluded	absent	10.47	-0.87	[illegible]
202249_s_at	included	present	1145.27	-1.04	included	present	963.89	-1.42	included	present	827.29	-1.7	included	present	1154.59	-1.02	[illegible]
202250_s_at	included	present	633.3	-0.27	included	present	353.19	-1.32	included	present	802.76	0.37	included	present	608.91	-0.36	[illegible]
202251_at	included	present	1016.79	-1.13	included	present	1441.17	-0.39	included	present	2878.38	2.11	included	present	1531.19	-0.24	[illegible]
202252_at	included	present	12981.55	-0.29	included	present	13317.47	-0.24	included	present	11230.44	-0.54	included	present	11871.06	-0.45	[illegible]
202253_s_at	excluded	absent	4.13	-0.97	excluded	absent	3.46	-0.98	excluded	absent	46.54	-0.44	excluded	absent	26.94	-0.69	[illegible]
202254_at	included	present	500.52	-0.29	included	absent	104.44	-0.75	included	absent	4.26	-0.87	included	present	155.11	-0.69	[illegible]
202255_s_at	included	present	721.9	0.11	included	absent	140.38	-1.14	included	present	151.22	-1.11	included	present	216.36	-0.97	[illegible]
202256_at	included	present	1525.59	1.72	included	present	3180.74	7.19	included	present	1437.04	1.43	included	present	889.77	-0.38	[illegible]
202257_s_at	included	present	1703.91	-0.07	included	present	3568.52	2.27	included	present	1188.3	-0.72	included	present	862.41	-1.12	[illegible]
202258_s_at	included	present	3704.31	-0.77	included	present	4329.45	-0.46	included	present	5125.3	-0.06	included	present	2828.39	-1.2	[illegible]
202259_s_at	included	present	756.68	-1.16	included	present	535.9	-1.56	included	present	1104.98	-0.54	included	present	553.47	-1.53	[illegible]
202260_s_at	included	present	3494.31	0.16	included	present	3106.28	0.04	included	present	3989.96	0.3	included	present	1914.86	-0.31	[illegible]
202261_at	included	present	181.72	0.09	included	present	317.89	2.7	included	present	333.18	2.99	included	present	427.26	4.8	[illegible]
202262_x_at	included	present	310.64	1.06	included	present	886.94	5.34	excluded	absent	126.13	-0.31	excluded	absent	20.94	-1.09	[illegible]
202263_at	included	present	604.08	2.19	included	present	202.34	-0.65	excluded	absent	190.43	-0.73	included	present	443.33	1.06	[illegible]
202264_s_at	included	present	624.58	-0.58	included	present	1238.66	1.35	included	present	954.44	0.46	included	present	601.49	-0.65	[illegible]
202265_at	included	present	2244.48	-0.46	included	present	3196.64	0.52	included	present	1305.21	-1.42	included	present	826.17	-1.91	[illegible]
202266_at	included	present	955.24	-0.4	included	present	1967.36	0.18	included	present	1041.63	-0.35	included	present	2497.91	0.48	[illegible]
202267_at	excluded	absent	8.33	-1.39	excluded	absent	127.87	0.28	included	present	204.84	1.35	included	present	73.19	-0.49	[illegible]
202268_s_at	included	present	1230.86	-0.57	included	present	1601.72	0.43	included	present	4008.29	6.9	included	present	1955.45	1.38	[illegible]
202269_x_at	included	present	1885.81	-0.02	included	present	362.68	-0.49	included	present	341.12	-0.5	included	present	218.27	-0.54	[illegible]
202270_at	included	present	174.6	-0.29	included	present	105.98	-0.35	included	absent	78.83	-0.38	included	absent	65.76	-0.39	[illegible]
202271_at	included	present	699.37	-0.04	included	present	481.86	-0.96	included	present	744.03	0.15	included	present	1012.76	1.29	[illegible]
202272_s_at	included	present	641.59	0.5	included	present	334.89	-1.5	included	present	886.63	2.09	included	present	918.06	2.29	[illegible]
202273_at	included	present	312.14	0.62	excluded	absent	51.98	-1.21	excluded	absent	156.75	-0.47	excluded	absent	92.13	-0.93	[illegible]
202274_at	excluded	absent	3.83	-0.44	excluded	absent	80.91	-0.43	included	present	124.65	-0.43	included	present	169.97	-0.43	[illegible]
202275_at	excluded	absent	93.9	1.21	excluded	absent	99.76	1.34	excluded	absent	5.33	-0.71	excluded	absent	3.34	-0.75	[illegible]
202276_at	included	present	12636.24	-0.97	included	present	13758.11	-0.72	included	present	23174.11	1.4	included	present	20270.72	0.75	[illegible]
202277_at	included	present	945.9	-0.37	included	present	670.33	-1.16	included	present	1308.18	0.66	included	present	1076.05	0	[illegible]
202278_s_at	included	present	756.14	1.3	included	absent	221.85	-2.17	included	present	1313.44	4.91	included	present	581.13	0.16	[illegible]
202279_at	included	present	2197.33	-0.71	included	present	3639.73	-0.26	included	present	1876.5	-0.81	included	present	2045.39	-0.76	[illegible]
202280_at	included	present	840.6	0.2	included	present	343.03	-1.65	included	present	437.25	-1.3	included	present	323.65	-1.72	[illegible]
202281_at	included	present	242.32	0.18	excluded	absent	421.93	2.31	excluded	absent	49.97	-2.1	excluded	absent	124.27	-1.22	[illegible]
202282_at	included	present	720.13	-0.16	included	present	1212.22	0.35	included	present	768.07	-0.11	included	present	740.2	-0.13	[illegible]
202283_at	included	present	14680.17	2.92	included	present	1753.42	-0.52	included	present	10946.73	1.93	included	present	602.37	-0.82	[illegible]
202284_s_at	included	present	4821.6	0.35	included	present	5594.87	0.55	included	present	2614.4	-0.2	included	present	5743.01	0.59	[illegible]
202285_s_at	excluded	absent	13.51	-0.8	excluded	absent	9.3	-0.96	excluded	absent	2.71	-1.2	excluded	absent	2.02	-1.22	[illegible]
202286_s_at	included	present	221.91	-0.42	included	present	172.47	-0.42	included	present	1419.57	-0.29	included	present	98.76	-0.43	[illegible]
202287_s_at	excluded	absent	2.55	-0.54	excluded	absent	4.61	-0.51	excluded	absent	7.64	-0.46	excluded	absent	13.73	-0.35	[illegible]
202288_at	included	present	235.94	0.69	excluded	absent	213.08	0.36	included	absent	97.63	-1.29	included	absent	81.04	-1.52	[illegible]
202289_s_at	included	present	683.3	-0.7	included	present	2140.18	-0.02	included	present	1225.04	-0.45	included	present	186.94	-0.93	[illegible]
202290_at	included	present	376.85	-0.63	included	present	1018.4	0.74	included	present	451.01	-0.47	included	present	217.91	-0.97	[illegible]
202291_s_at	included	present	1493.4	-0.73	included	present	4924.4	-0.44	included	present	1265.06	-0.75	included	present	3275.11	-0.58	[illegible]
202292_x_at	excluded	absent	4.02	-2.6	excluded	absent	131.35	0.09	excluded	absent	74.34	-1.11	excluded	absent	107.83	-0.4	[illegible]
202293_at	included	present	1151.45	-0.47	included	present	1343.07	-0.12	included	present	1596.72	0.33	included	present	1392.72	-0.03	[illegible]
202294_at	included	present	679.92	0.21	included	present	674.08	0.18	included	present	582.63	-0.17	included	present	638.82	0.05	[illegible]
202295_s_at	included	present	1881.17	-0.4	included	present	7030.52	1.19	included	present	1082.53	-0.64	included	present	191.01	-0.92	[illegible]
202296_s_at	included	present	971.03	-0.49	included	present	967.74	-0.49	included	present	696.62	-0.98	included	present	406.75	-1.49	[illegible]
202297_s_at	included	present	2237.22	0.19	included	present	2540.9	0.55	included	present	1231.66	-1.01	included	present	783.25	-1.54	[illegible]
202298_at	included	present	11867.37	-0.63	included	present	6262.72	-1.31	included	present	10198.13	-0.83	included	present	9272.51	-0.95	[illegible]
202299_s_at	included	present	5279.04	0.38	included	present	5534.89	0.5	included	present	5049.73	0.26	included	present	4117.09	-0.19	[illegible]
202300_at	included	present	3897.63	0.25	included	present	4696.25	0.74	included	present	3896.63	0.25	included	present	2979.15	-0.32	[illegible]
202301_s_at	included	present	2530.14	-0.67	included	present	3493.09	1.22	included	present	4282.15	2.78	included	present	4319.14	2.85	[illegible]
202302_s_at	included	present	4503.69	-1.67	included	present	7388.76	0.77	included	present	8179.22	1.44	included	present	7839.26	1.15	[illegible]
202303_x_at	included	present	2674.19	0.29	included	present	4280.22	2.17	included	present	4039.54	1.89	included	present	3153.55	0.85	[illegible]
202304_at	included	present	2017.68	0.2	included	present	2120.64	0.33	included	present	712.77	-1.35	included	present	1665.23	-0.21	[illegible]
202305_s_at	included	present	2186.55	0.46	included	present	1072.07	-0.27	included	present	1190.76	-0.19	included	present	589.91	-0.59	[illegible]
202306_at	included	present	2266.67	-0.21	included	present	5343.72	4.8	included	present	3144.7	1.22	included	present	1859.49	-0.87	[illegible]
202307_s_at	included	absent	561.63	-0.44	included	absent	321.68	-0.79	included	present	449.74	-0.6	included	present	769.91	-0.14	[illegible]
202308_at	excluded	absent	105.49	-0.42	excluded	absent	44.05	-0.88	excluded	absent	3.59	-1.18	excluded	absent	114.37	-0.36	[illegible]
202309_at	included	present	441.55	-0.23	included	present	908.53	0.92	included	present	707.45	0.42	included	present	1143.72	1.5	[illegible]
202310_s_at	included	present	23760.3	2.28	included	present	9417.07	0.6	included	present	5700.04	0.16	included	present	4678.42	0.05	[illegible]
202311_s_at	included	present	2070.58	0.23	included	present	736.15	-0.2	included	present	837.24	-0.17	included	present	820.3	-0.18	[illegible]
202312_s_at	excluded	absent	62.11	0.13	excluded	absent	69.68	0.29	excluded	absent	14.33	-0.85	excluded	absent	36.28	-0.4	[illegible]
202313_at	included	present	1393.78	0.23	included	present	894.78	-1.28	included	present	1607.07	0.87	included	present	1081.91	-0.71	[illegible]
202314_at	included	present	5655.18	1.91	included	present	3341.24	0.55	included	present	2755.85	0.21	included	present	8125.38	3.36	[illegible]
202315_s_at	included	present	87.4	-0.89	excluded	absent	190.78	0.24	included	absent	73.57	-1.04	included	present	160.35	-0.09	[illegible]
202316_x_at	included	present	490.91	-0.05	included	present	257.99	-1.32	included	present	344.23	-0.85	included	absent	269.31	-1.26	[illegible]
202317_s_at	included	present	4684.24	0.63	included	present	3145.06	-0.82	included	present	2574.5	-1.35	included	present	1833.52	-2.05	[illegible]
202318_s_at	included	present	3031.67	-0.72	included	present	4673.49	0.98	included	present	3408.5	-0.33	included	present	2792.43	-0.96	[illegible]
202319_at	included	present	180.63	-0.35	included	absent	72.04	-1.45	included	present	238.95	0.24	included	present	166.15	-0.5	[illegible]
202320_at	excluded	absent	10.77	-0.94	excluded	absent	15.02	-0.85	excluded	absent	10.15	-0.95	excluded	absent	4.08	-1.07	[illegible]
202321_at	included	present	619.28	-0.06	included	present	1210.93	2.87	included	present	955.26	1.61	included	present	891.02	1.29	[illegible]
202322_s_at	included	present	1229.63	-0.3	included	present	2672.65	3.19	included	present	1641.63	0.69	included	present	1385.16	0.08	[illegible]
202323_s_at	included	present	273.64	0.32	included	present	193.9	-0.44	included	present	190.8	-0.47	included	present	272.93	0.31	[illegible]
202324_s_at	included	present	994.99	-0.52	included	present	1451.6	0.68	included	present	1529.98	0.88	included	present	1703.02	1.34	[illegible]
202325_s_at	included	present	12057.04	-0.97	included	present	21587.11	0.11	included	present	20469.21	-0.01	included	present	18893.69	-0.19	[illegible]
202326_at	included	present	449.91	0.62	included	present	2327.45	11.53	included	present	649.96	1.78	included	present	645.38	1.76	[illegible]
202327_s_at	excluded	absent	87.54	0.41	included	present	97.23	0.6	excluded	absent	41.61	-0.47	excluded	absent	49.04	-0.33	[illegible]
202328_s_at	excluded	absent	162.51	-0.43	included	present	288.43	1.49	excluded	absent	115.65	-1.14	excluded	absent	110.35	-1.22	[illegible]
202329_at	included	present	316.8	0.12	included	present	1107.05	4.06	included	present	330.91	0.19	included	present	282.29	-0.05	[illegible]
202330_s_at	included	present	440.93	-0.91	included	present	1935.3	1.43	included	present	1615.44	0.93	included	present	4334.63	5.19	[illegible]
202331_at	included	present	118.25	-0.41	excluded	absent	63.02	-1.09	excluded	absent	11.24	-1.73	included	present	118.97	-0.4	[illegible]
202332_at	excluded	absent	292.05	0.95	included	present	547.27	3.13	excluded	absent	83.04	-0.84	excluded	absent	134	-0.4	[illegible]
202333_s_at	included	present	2766.5	-0.38	included	present	901.65	-1.94	included	present	4209.03	0.82	included	present	4084.82	0.71	[illegible]
202334_s_at	included	present	1320.06	-1.1	included	present	341.58	-2.23	included	present	1621.91	-0.75	included	present	2064.76	-0.24	[illegible]
202335_s_at	excluded	absent	70.85	0.32	excluded	absent	2.06	-1.89	excluded	absent	63.67	0.09	excluded	absent	48.73	-0.39	[illegible]
202336_s_at	included	present	3193.92	-0.25	included	present	555.79	-1.05	included	present	1003.86	-0.92	included	present	6397.45	0.72	[illegible]
202337_at	included	present	264.5	-0.24	included	present	279.19	-0.16	included	present	198.71	-0.59	included	present	303.31	-0.03	[illegible]
202338_at	excluded	absent	5.37	-0.78	excluded	absent	180.12	3.5	excluded	absent	136.7	2.44	included	present	162.48	3.07	[illegible]
202339_at	excluded	absent	129.14	-1.15	excluded	absent	421.66	2.04	excluded	absent	128.33	-1.15	included	present	154.41	-0.87	[illegible]
202340_x_at	included	present	251.64	0.25	excluded	absent	130.61	-0.49	excluded	absent	84.92	-0.77	excluded	absent	68.7	-0.86	[illegible]
202341_s_at	included	present	3316.01	-0.37	included	present	7653.1	0.57	included	present	2951.71	-0.45	included	present	878.64	-0.89	[illegible]
202342_s_at	included	present	2785.24	-0.53	included	present	3083.13	-0.46	included	present	3772.72	-0.31	included	present	817.55	-0.96	[illegible]
202343_x_at	included	present	1078.3	-1.21	included	present	2471.81	-0.33	included	present	1617.06	-0.87	included	present	768.85	-1.41	[illegible]
202344_at	excluded	absent	10.15	-0.92	excluded	absent	102.88	0.71	excluded	absent	115.95	0.94	excluded	absent	61.1	-0.03	[illegible]
202345_s_at	included	present	2771.83	-0.66	included	present	6116.36	-0.01	included	present	6364.45	0.04	included	present	5116.04	-0.21	[illegible]
202346_at	included	present	1149.43	-0.38	included	present	1248.21	-0.15	included	present	1852.21	1.24	included	present	1539.61	0.52	[illegible]
202347_s_at	included	present	2724.28	0.61	included	present	3442.58	1.8	included	present	3714.89	2.25	included	present	3193.99	1.39	[illegible]
202348_s_at	included	present	665.47	-1.15	included	present	898.09	-0.15	included	present	1267.24	1.44	included	present	888.98	-0.19	[illegible]
202349_at	included	present	2840.76	2.09	included	present	2240.82	0.69	included	present	4171.1	5.22	included	present	4529.26	6.06	[illegible]
202350_s_at	included	present	11311.85	6.53	included	present	280.61	-0.74	included	present	184.6	-0.81	included	present	311.95	-0.72	[illegible]
202351_at	included	present	6216.4	0.41	included	present	2331.65	-1.13	included	present	2740.99	-0.96	included	present	1845.65	-1.32	[illegible]
202352_s_at	included	present	3579.76	3.13	included	present	2007.6	0.57	included	present	3304.59	2.68	included	present	2802.08	1.86	[illegible]
202353_s_at	included	present	2898.36	4.77	included	present	972.36	-0.14	included	present	1490.58	1.19	included	present	2053.74	2.62	[illegible]
202354_s_at	included	present	310.69	-0.45	included	present	296.98	-0.53	included	present	596.3	1.26	included	present	397.45	0.07	[illegible]
202355_s_at	included	present	388.21	-1.34	included	present	1015.84	1.92	included	present	418.12	-1.19	included	present	475.85	-0.89	[illegible]
202356_s_at	excluded	absent	42.41	-0.94	included	present	109.12	0.84	excluded	absent	74.47	-0.08	excluded	absent	42.32	-0.94	[illegible]
202357_s_at	included	present	745.53	-0.13	included	present	195.84	-0.26	included	present	791.79	-0.12	included	present	150.67	-0.27	[illegible]
202358_s_at	included	present	358.6	-0.77	included	present	411.92	-0.35	included	present	337.96	-0.94	included	present	292.37	-1.3	[illegible]
202359_s_at	included	present	445.82	-1.5	included	present	1386.16	2.13	included	present	792.24	-0.16	included	present	486.52	-1.34	[illegible]
202360_at	included	present	371.95	-0.49	included	present	307.97	-0.86	included	present	352.09	-0.6	included	present	636.02	1.04	[illegible]
202361_at	included	present	808.64	0.69	included	present	870.72	1.01	included	present	806.09	0.68	included	present	693.02	0.12	[illegible]
202362_at	included	present	1544.09	-0.1	included	present	1290.17	-0.29	included	present	1104.2	-0.43	included	present	413.47	-0.94	[illegible]
202363_at	included	present	1816.14	0.09	included	present	683.73	-0.63	included	present	4617.58	1.88	included	present	1502.45	-0.11	[illegible]
202364_at	included	present	1594.69	-1.32	included	present	2452.3	-0.91	included	present	4425.98	0.03	included	present	2663.38	-0.81	[illegible]
202365_at	included	present	704.13	0.09	included	present	679	0.01	included	present	902.87	0.7	included	present	1924.96	3.82	[illegible]
202366_at	excluded	absent	274.49	-0.37	excluded	absent	243.34	-0.49	excluded	absent	139.87	-0.9	excluded	absent	153.05	-0.85	[illegible]
202367_at	excluded	absent	141.95	0.11	included	present	291.67	1.98	excluded	absent	50.54	-1.03	excluded	absent	96.25	-0.46	[illegible]
202368_s_at	excluded	absent	600.36	1.68	included	absent	94.98	-1.06	included	present	93.55	-1.07	included	present	298.9	0.05	[illegible]
202369_s_at	included	present	332.97	0.6	included	absent	6.66	-1.65	included	absent	156.42	-0.62	included	present	219.53	-0.18	[illegible]
202370_s_at	included	present	1575.5	0.4	included	present	1917.11	1.03	included	present	1787.35	0.79	included	present	1803.05	0.82	[illegible]
202371_at	included	present	16964.33	-0.07	included	present	16106.95	-0.17	included	present	16644.17	-0.11	included	present	12332.15	-0.64	[illegible]
202372_at	included	present	1033.04	0.04	included	present	735.16	-0.94	included	present	904.39	-0.39	included	present	1131.91	0.36	[illegible]
202373_s_at	included	present	2538.27	4.66	included	present	1887.22	2.44	included	present	2020.96	2.9	included	present	2655.11	5.06	[illegible]
202374_s_at	included	present	613.19	1.79	included	present	221.17	-1.35	included	present	660.29	2.17	included	present	719.21	2.64	[illegible]
202375_at	included	present	511.4	-0.2	included	present	327.06	-0.53	included	present	227.98	-0.7	included	present	533.69	-0.16	[illegible]
202376_at	included	present	601.13	-0.3	included	absent	22.14	-0.34	included	present	3402.5	-0.1	included	absent	100.69	-0.34	[illegible]
202377_at	included	present	1848.82	0.51	included	present	878.19	-0.8	included	present	498.66	-1.31	included	present	1603.31	0.17	[illegible]
202378_s_at	included	present	2442.15	0.8	included	present	1197.32	-0.69	included	present	818.86	-1.15	included	present	1731.45	-0.05	[illegible]
202379_s_at	included	present	3625.2	-0.72	included	present	3862.37	-0.61	included	present	4168.88	-0.45	included	present	4412.21	-0.33	[illegible]
202380_s_at	included	present	698.68	-1.24	included	present	656.45	-1.3	included	present	777.11	-1.12	included	present	1058.72	-0.7	[illegible]
202381_at	included	present	1113.71	-0.19	included	present	280.19	-1.33	included	present	826.46	-0.58	included	present	741.68	-0.7	[illegible]
202382_s_at	included	present	419.18	-0.54	included	present	461.08	-0.4	included	present	596.97	0.06	included	present	342.15	-0.8	[illegible]
202383_at	excluded	absent	206.14	0.16	included	present	311.7	1.59	excluded	absent	67.06	-1.73	excluded	absent	83.97	-1.5	[illegible]
202384_s_at	excluded	absent	145.57	-0.77	included	present	263.04	0.82	excluded	absent	104.73	-1.33	included	present	250.9	0.66	[illegible]
202385_s_at	included	present	410.64	2.32	included	present	661.18	5.55	included	present	598.12	4.73	included	present	310.49	1.03	[illegible]
202386_s_at	included	present	1665.48	0.02	included	present	1409.49	-0.53	included	present	1203.37	-0.97	included	present	2057.6	0.86	[illegible]
202387_at	included	present	1665.69	0.16	included	present	1717.49	0.23	included	present	1287.75	-0.34	included	present	924.7	-0.82	[illegible]
202388_at	included	present	4468.55	-0.53	included	present	10122	-0.08	included	present	3402.84	-0.61	included	present	2626.19	-0.67	[illegible]
202389_s_at	excluded	absent	136.77	0.99	excluded	absent	61.06	-0.42	excluded	absent	8.35	-1.41	excluded	absent	98.79	0.28	[illegible]
202390_s_at	included	present	330.06	0.03	included	present	353.83	0.15	included	absent	165.82	-0.8	included	present	195.79	-0.65	[illegible]
202391_at	included	present	7057.31	-0.16	included	present	15038.4	0.86	included	present	10128.73	0.23	included	present	10559.94	0.29	[illegible]
202392_s_at	included	present	297.54	-0.24	included	present	375.14	0.39	included	present	217.62	-0.88	included	present	359.04	0.26	[illegible]
202393_s_at	included	present	1147.25	-0.38	included	present	794.24	-0.55	included	present	2472.7	0.24	included	present	4166.39	1.04	[illegible]
202394_s_at	excluded	absent	240.56	0.49	included	present	357.8	1.66	excluded	absent	16.08	-1.74	excluded	absent	153.53	-0.37	[illegible]
202395_at	included	present	4994.7	1.29	included	present	1380.74	-0.37	included	present	756.71	-0.66	included	present	1115.46	-0.49	[illegible]
202396_at	included	present	947.15	-0.91	included	present	1482.19	0.47	included	present	2012.48	1.85	included	present	1912.88	1.59	[illegible]
202397_at	included	present	2724.78	0.72	included	present	3139	1.34	included	present	5537.03	4.93	included	present	1929.96	-0.47	[illegible]

202398_at	included	present	230.9	1.54	included	present	180.9	0.5	excluded	absent	97.32	-1.23	included	present	143.3	-0.28	[illegible]
202399_s_at	included	present	2644.7	2.24	included	present	3164.76	3.26	included	present	1537.03	0.05	included	present	1381.07	-0.26	[illegible]
202400_s_at	excluded	absent	32.73	-0.29	excluded	absent	90.21	1.22	excluded	absent	34.44	-0.25	excluded	absent	8.29	-0.94	[illegible]
202401_s_at	included	present	647.24	-0.65	included	present	1223.79	0.27	included	present	674.49	-0.61	included	present	821.95	-0.37	[illegible]
202402_s_at	included	present	1427.06	2.52	included	present	2915.19	8.38	included	present	719.39	-0.26	included	present	603.39	-0.72	[illegible]
202403_s_at	included	present	21810.37	1.9	included	present	3168.06	-0.36	included	present	3341.02	-0.34	included	present	5063.71	-0.13	[illegible]
202404_s_at	included	present	8562	0.61	included	present	3970.67	-0.1	included	present	4182.94	-0.06	included	present	6178.5	0.25	[illegible]
202405_at	included	present	339.04	0.14	included	present	360.85	0.36	included	present	380.7	0.57	included	present	588.03	2.73	[illegible]
202406_s_at	included	present	2426.66	-0.08	included	present	2183.16	-0.46	included	present	4531.05	3.19	included	present	3234.2	1.17	[illegible]
202407_s_at	excluded	absent	3.5	-0.91	excluded	absent	17.5	-0.55	excluded	absent	13.8	-0.64	excluded	absent	58.6	0.52	[illegible]
202408_s_at	included	present	241.81	-1.13	included	present	436.61	0.96	included	present	186.46	-1.72	included	present	312.12	-0.37	[illegible]
202409_at	included	present	218.06	-0.32	included	absent	245.6	-0.27	included	absent	91.9	-0.55	included	present	580.91	0.36	[illegible]
202410_x_at	included	present	310.6	1.61	excluded	absent	179.05	0.57	excluded	absent	50.2	-0.44	included	present	741.54	5.01	[illegible]
202411_at	included	present	2000.3	0.73	included	absent	304.64	-0.45	included	absent	194.53	-0.53	included	absent	184.92	-0.54	[illegible]
202412_s_at	included	present	908.39	0.94	included	present	1237.84	1.88	included	present	970.76	1.12	included	present	3431.61	8.13	[illegible]
202413_s_at	included	present	848.35	-0.14	included	present	1768.2	1.39	included	present	1270.7	0.56	included	present	4222.16	5.46	[illegible]
202414_at	included	present	1743.97	-1.17	included	present	3655.18	0.75	included	present	1444.45	-1.47	included	present	1822.24	-1.09	[illegible]
202415_s_at	excluded	absent	48.66	-0.25	excluded	absent	86.94	0.47	excluded	absent	56.48	-0.1	excluded	absent	104.08	0.79	[illegible]
202416_at	included	present	1622.42	0.53	included	present	2155.85	1.88	included	present	3747.66	5.91	included	present	883.6	-1.34	[illegible]
202417_at	included	present	730.24	-0.68	included	present	1219.87	0.04	included	present	911.67	-0.41	included	present	1231.6	0.06	[illegible]
202418_at	excluded	absent	135.38	0.44	excluded	absent	44.19	-0.35	excluded	absent	37.23	-0.41	excluded	absent	69	-0.14	[illegible]
202419_at	included	present	2003.83	0.58	included	present	1942.33	0.47	included	present	695.3	-1.85	included	present	1995.98	0.57	[illegible]
202420_s_at	included	present	2490.93	-1.98	included	present	9163.67	4.71	included	present	5291.03	0.83	included	present	4324.44	-0.14	[illegible]
202421_at	included	present	226.03	-0.3	included	present	1797.55	5.43	included	present	259.69	-0.18	included	present	246.67	-0.22	[illegible]
202422_s_at	included	present	513.12	-0.22	included	present	345.82	-0.74	included	present	281.2	-0.94	included	present	422.93	-0.5	[illegible]
202423_at	included	present	2583.45	1.16	included	present	1531.39	-0.37	included	present	2524.91	1.07	included	present	1928.12	0.21	[illegible]
202424_at	excluded	absent	168.86	1.23	excluded	absent	126.82	0.6	excluded	absent	21.93	-0.96	excluded	absent	101.16	0.22	[illegible]
202425_x_at	included	present	2406.08	-0.54	included	present	3919.6	-0.24	included	present	2454.08	-0.53	included	present	3515.66	-0.32	[illegible]
202426_s_at	excluded	absent	37.45	-0.85	excluded	absent	53.85	-0.78	excluded	absent	98.24	-0.59	excluded	absent	37.31	-0.85	[illegible]
202427_s_at	included	present	1729.08	-0.88	included	present	2739.74	-0.58	included	present	2243.95	-0.72	included	present	3376.79	-0.39	[illegible]
202428_x_at	included	present	5435.16	-0.86	included	present	4999.88	-0.93	included	present	3924.31	-1.08	included	present	1676.19	-1.41	[illegible]
202429_s_at	included	present	815.77	-0.59	included	present	1033.09	-0.52	included	present	683.74	-0.64	included	present	1408.43	-0.38	[illegible]
202430_s_at	included	present	463.71	-0.14	included	present	118.71	-0.79	included	present	374.12	-0.31	included	present	536.14	0	[illegible]
202431_s_at	included	present	772.18	-0.35	included	present	523.42	-0.46	included	present	315.27	-0.55	included	present	327.88	-0.55	[illegible]
[illegible]	[illegible]	[illegible]	[illegible]	[illegible]	[illegible]	[illegible]	[illegible]	[illegible]	[illegible]	[illegible]	[illegible]	[illegible]	[illegible]	[illegible]	[illegible]	[illegible]	[illegible]

202757_at	included	present	669.62	-0.07	included	present	2110.57	5.3	included	present	867.19	0.67	included	present	385.86	-1.12	included	present	598.79	-0.33	included	present	1097.4	1.53	included	present	851.74	0.61	included	present	1203.15	1.92	included	present	662.19	-0.09	included	present	1025.58	1.26	included	present	828.25	0.53	included	present	2009.78	4.93	included	present	243.78	-1.65	included	absent	49.24	-2.38	included	present	1018	1.23
202758_s_at	excluded	absent	272.84	1.31	excluded	absent	222.26	0.81	excluded	absent	7.32	-1.33	excluded	absent	212.88	0.71	excluded	absent	26.37	-1.14	included	present	458.96	3.16	excluded	absent	96.15	-0.45	included	present	289.43	1.47	excluded	absent	116.41	-0.24	excluded	absent	309.44	1.67	excluded	absent	96.9	-0.44	excluded	absent	150.75	0.1	excluded	absent	20.97	-1.19	excluded	absent	43.78	-0.97	included	present	256.9	1.15
202759_s_at	included	present	1860.11	0.54	included	present	318.54	-0.85	included	present	234.94	-0.93	included	present	553.22	-0.64	included	present	973.06	-0.26	included	present	3598.01	2.12	included	present	476.52	-0.71	included	present	899.56	-0.33	included	present	1937.81	0.61	included	present	1012.32	-0.22	included	present	949.91	-0.28	included	present	1442.04	0.17	included	present	1015.79	-0.22	included	present	1195.62	-0.06	included	present	474.51	-0.71
202760_s_at	included	present	14406.98	1.45	included	present	3030.54	-0.81	included	present	3309.28	-0.75	included	present	3118.89	-0.79	included	present	6713.63	-0.07	included	present	8837.82	0.35	included	present	1417.68	-1.13	included	present	2078.95	-0.99	included	present	5654.92	-0.28	included	present	2333.32	-0.94	included	present	3197.37	-0.77	included	present	914.41	-1.23	included	present	3341.87	-0.74	included	present	3535.75	-0.71	included	present	1354.57	-1.14
202761_s_at	included	present	1029.54	-0.22	included	present	1167.89	-0.14	included	present	871.91	-0.31	included	present	2882.07	0.84	included	present	879.67	-0.31	included	present	1059.49	-0.2	included	present	814.35	-0.34	included	present	1024.02	-0.22	included	present	1236.9	-0.1	included	present	1124.12	-0.17	included	present	1877.27	0.26	included	present	402.49	-0.58	included	present	378	-0.59	included	present	535.53	-0.5	included	present	1075.42	-0.19
202762_at	included	present	576.5	-0.64	included	present	227.78	-1.44	included	present	693.56	-0.38	included	present	470.19	-0.89	included	present	383.34	-1.08	included	present	733.98	-0.28	included	present	803.45	-0.12	included	present	489.76	-0.84	included	present	694.06	-0.37	included	present	757.7	-0.23	included	present	958.93	0.23	included	present	713.79	-0.33	included	present	768.69	-0.2	included	present	704.75	-0.35	included	present	516.01	-0.78
202763_at	included	present	468.29	-0.39	included	present	709.75	0.13	included	present	1237.12	1.26	included	present	650.25	0	included	present	1813.73	2.49	included	present	915.54	0.57	included	present	2515.39	4	included	present	707.32	0.12	included	present	659.91	0.02	included	present	1916.93	2.71	included	present	1416.26	1.64	included	present	897.42	0.53	included	present	691.04	0.09	included	present	311.57	-0.73	included	present	1503.66	1.83
202764_at	included	present	470.76	-0.04	included	present	265.82	-0.86	included	present	145.93	-1.34	included	present	354.53	-0.5	included	absent	278.82	-0.81	included	present	723.26	0.98	included	present	465.73	-0.06	included	present	319.94	-0.64	included	present	570.68	0.37	included	present	921.67	1.78	included	present	244.67	-0.94	included	absent	265.84	-0.86	included	present	268.62	-0.85	included	absent	140.69	-1.36	included	present	347.21	-0.53
202765_s_at	included	present	4674.39	1.86	included	present	478.99	-0.46	included	present	400.84	-0.5	included	present	533.29	-0.43	included	present	1652.22	0.19	included	present	9890.39	4.75	included	present	354.77	-0.53	included	present	863.6	-0.24	included	present	2241.36	0.52	included	present	978.33	-0.18	included	present	451.89	-0.47	included	present	783.65	-0.29	included	present	1539.92	0.13	included	present	1701.09	0.22	included	present	210.88	-0.6
202766_s_at	included	present	2298.73	0.58	included	present	245.72	-0.64	included	present	359.93	-0.57	included	present	296.03	-0.61	included	present	1084.24	-0.14	included	present	11237.21	5.89	included	present	323.66	-0.59	included	present	1165.79	-0.09	included	present	3371.13	1.22	included	present	888.08	-0.25	included	present	401.22	-0.54	included	present	1780.69	0.28	included	present	1091.92	-0.13	included	present	1205.27	-0.07	included	present	359.39	-0.57
202767_at	included	present	234.13	0.83	included	present	175.99	0.24	excluded	absent	89.9	-0.64	excluded	absent	100.28	-0.53	included	present	312.07	1.62	included	present	197.05	0.45	included	present	222.63	0.71	excluded	absent	118.44	-0.35	included	present	230.99	0.8	included	present	208.16	0.57	included	present	259.07	1.08	included	present	483.31	3.37	excluded	absent	108.12	-0.46	excluded	absent	60.69	-0.94	included	present	410.17	2.63
202768_at	included	present	1824.02	-0.04	included	absent	57.12	-0.49	included	absent	6.91	-0.51	included	absent	73.82	-0.49	included	absent	3.76	-0.51	included	present	8245.79	1.62	included	present	514.03	-0.37	included	absent	66.16	-0.49	included	present	252.46	-0.44	included	present	1241.63	-0.19	included	absent	51.24	-0.49	included	absent	30.31	-0.5	included	present	583.88	-0.36	included	absent	84.73	-0.49	included	absent	114.53	-0.48
202769_at	included	present	4402.3	0.03	included	present	6345.26	0.85	included	present	5375.19	0.44	included	present	4154.69	-0.07	included	present	4511.11	0.08	included	present	3039.82	-0.54	included	present	4710.35	0.16	included	present	4025.49	-0.13	included	present	3890.78	-0.18	included	present	2288.81	-0.86	included	present	4213.52	-0.05	included	present	5814.3	0.63	included	present	9669.83	2.25	included	present	7068.12	1.16	included	present	3563.59	-0.32
202770_s_at	included	present	430.34	0	included	present	603.78	0.66	included	present	429.95	-0.01	included	present	328.92	-0.4	included	present	403.9	-0.11	included	present	385.53	-0.18	included	present	558.41	0.49	included	present	370.35	-0.24	included	present	619.8	0.73	included	present	242.15	-0.73	included	present	612.78	0.7	included	present	203.83	-0.88	included	present	313.78	-0.45	included	present	173.47	-0.99	included	present	351.3	-0.31
202771_at	excluded	absent	114.19	0.34	excluded	absent	10.7	-1.09	excluded	absent	39.91	-0.69	excluded	absent	4.36	-1.18	excluded	absent	13.53	-1.05	included	present	324.83	3.25	excluded	absent	60.96	-0.4	excluded	absent	19.56	-0.97	excluded	absent	200.92	1.54	excluded	absent	110.44	0.29	excluded	absent	45.86	-0.6	excluded	absent	125.09	0.49	excluded	absent	35.45	-0.75	excluded	absent	2.22	-1.21	excluded	absent	92.48	0.04
202772_at	included	absent	569.17	-0.22	included	absent	198.12	-0.55	included	absent	139.96	-0.6	included	absent	187.01	-0.56	included	absent	38.93	-0.69	included	absent	617.31	-0.17	included	absent	538.07	-0.24	included	absent	596.23	-0.19	included	absent	475.82	-0.3	included	absent	551.72	-0.23	included	absent	397.9	-0.37	included	absent	191.23	-0.55	included	absent	81.42	-0.65	included	absent	30.45	-0.7	included	present	633.91	-0.16
202773_s_at	included	present	2597.94	0.52	included	present	1917.02	-0.44	included	present	3288.84	1.5	included	present	2651.81	0.6	included	present	4324.58	2.96	included	present	1570.17	-0.93	included	present	2554.35	0.46	included	present	2323.15	0.14	included	present	2875.51	0.92	included	present	2198.66	-0.04	included	present	5135.78	4.11	included	present	3818.93	2.25	included	present	1754.29	-0.67	included	present	3926.25	2.4	included	present	2131.87	-0.13
202774_s_at	included	present	2365.25	0.07	included	present	5771.98	3.94	included	present	3003.44	0.79	included	present	2242.2	-0.07	included	present	4679.73	2.7	included	present	1242.61	-1.21	included	present	1600.34	-0.8	included	present	1484.45	-0.93	included	present	1596.52	-0.81	included	present	1281.43	-1.16	included	present	2816.87	0.58	included	present	1115.61	-1.35	included	present	2175.1	-0.15	included	present	2107.49	-0.22	included	present	1413.61	-1.01
202775_s_at	included	present	191.48	0.52	included	present	210.7	0.82	included	present	145.99	-0.21	included	present	130.88	-0.45	included	absent	131.59	-0.44	included	present	303.23	2.3	included	present	587.34	6.83	included	present	408.46	3.98	included	present	487.59	5.24	included	present	487.97	5.25	included	present	549.16	6.22	included	present	301.9	2.28	included	present	153.87	-0.08	included	present	109.69	-0.79	included	present	591.12	6.89
202776_at	included	present	2363.44	0.28	included	present	3335.58	1.97	included	present	1963.93	-0.42	included	present	3331.08	1.96	included	present	1727.75	-0.83	included	present	1153.18	-1.83	included	present	1276.08	-1.62	included	present	2367.78	0.29	included	present	1271.12	-1.63	included	present	894.49	-2.28	included	present	1350.34	-1.49	included	present	1954.22	-0.44	included	present	2550.85	0.6	included	present	4635.24	4.24	included	present	1448.95	-1.32
202777_at	included	present	2763.35	0.03	included	present	2438.59	-0.46	included	present	2575.83	-0.26	included	present	2994.24	0.38	included	present	2511.57	-0.35	included	present	2185.28	-0.85	included	present	2028.52	-1.08	included	present	3355.06	0.92	included	present	2202.35	-0.82	included	present	1616.67	-1.71	included	present	1239.15	-2.28	included	present	3547.35	1.21	included	present	3127.87	0.58	included	present	2929.29	0.28	included	present	1685.15	-1.6
202778_s_at	included	present	2358.54	-1.15	included	present	4298.43	0.57	included	present	4644.69	0.88	included	present	3103.2	-0.49	included	present	3402.16	-0.23	included	present	2405.51	-1.11	included	present	3572.27	-0.08	included	present	4050.62	0.35	included	present	2488.07	-1.04	included	present	1923.85	-1.54	included	present	2657.34	-0.89	included	present	5429.38	1.58	included	present	2689.01	-0.86	included	present	3063.04	-0.53	included	present	2814.93	-0.75
202779_s_at	excluded	absent	36.22	-0.61	excluded	absent	162.26	1.6	included	present	250.15	3.14	included	present	136.38	1.15	excluded	absent	95.82	0.43	excluded	absent	129.32	1.02	included	present	122.27	0.9	included	present	784.1	12.51	included	present	137.07	1.16	included	present	333.33	4.6	included	present	926.07	15	included	present	306.68	4.13	excluded	absent	63.33	-0.14	excluded	absent	16.34	-0.96	included	present	826.99	13.26
202780_at	included	present	632.65	-0.95	included	present	1491.93	-0.01	included	present	683	-0.89	included	present	1275.06	-0.24	included	present	1040.64	-0.5	included	present	781.03	-0.78	included	absent	61.38	-1.57	included	present	900.33	-0.65	included	present	467.18	-1.13	included	present	818.64	-0.74	included	present	107.36	-1.52	included	present	161.33	-1.46	included	present	968.45	-0.58	included	absent	170.63	-1.45	included	present	59.39	-1.58
202781_s_at	included	present	127.83	-0.03	excluded	absent	142.31	0.17	excluded	absent	81.08	-0.69	excluded	absent	32.44	-1.38	excluded	absent	117.06	-0.18	included	present	249.46	1.68	excluded	absent	112.15	-0.25	included	present	196.03	0.93	included	present	209.8	1.12	included	present	268.66	1.95	included	present	179.8	0.7	excluded	absent	57.46	-1.02	excluded	absent	127.66	-0.03	excluded	absent	43.92	-1.21	included	present	158.12	0.39
202782_s_at	excluded	absent	48.68	-0.63	excluded	absent	97.54	0.56	excluded	absent	15.06	-1.45	excluded	absent	56.37	-0.44	excluded	absent	60.45	-0.34	excluded	absent	165.83	2.23	included	present	128.46	1.31	excluded	absent	98.01	0.57	excluded	absent	106.68	0.78	included	present	145.7	1.74	excluded	absent	84.1	0.23	excluded	absent	138.48	1.56	excluded	absent	82.75	0.2	excluded	absent	82.83	0.2	included	present	143.11	1.67
202783_at	included	present	162.64	-0.73	included	present	322.92	-0.3	included	present	505.9	0.18	included	present	990.4	1.46	included	present	450.86	0.03	included	present	409.72	-0.07	included	present	493.83	0.15	included	present	921.89	1.28	included	present	572.52	0.36	included	present	436.91	0	included	present	420.29	-0.05	included	present	356.26	-0.22	included	present	375.37	-0.17	included	present	425.58	-0.03	included	present	369.91	-0.18
202784_s_at	included	present	591.28	-0.71	included	present	1428.99	-0.02	included	present	2260.48	0.67	included	present	2769.69	1.08	included	present	2055.79	0.5	included	present	711.75	-0.61	included	present	704.32	-0.61	included	present	2246.63	0.65	included	present	775.36	-0.55	included	present	1358.62	-0.07	included	present	1031.73	-0.34	included	present	668.99	-0.64	included	present	864.56	-0.48	included	present	1589.55	0.11	included	present	1146.09	-0.25
202785_at	excluded	absent	255.35	0.44	excluded	absent	86.42	-1.11	excluded	absent	82.34	-1.15	included	present	199.45	-0.07	excluded	absent	143.8	-0.58	included	present	769.99	5.18	included	present	901.07	6.38	included	present	1790.3	14.56	included	present	630.6	3.89	included	present	1451.15	11.44	included	present	786.83	5.33	included	present	715.49	4.67	excluded	absent	142.23	-0.6	excluded	absent	90.98	-1.07	included	present	1300.98	10.06
202786_at	included	present	767.99	-0.68	included	absent	604.54	-0.82	included	present	2251.98	0.6	included	present	1938.61	0.33	included	present	1342.9	-0.19	included	present	490.5	-0.92	included	present	2007.08	0.38	included	present	1678.93	0.1	included	present	1443.96	-0.1	included	present	1311.01	-0.21	included	present	740.79	-0.71	included	present	2928.07	1.18	included	present	2041.36	0.41	included	present	2080.51	0.45	included	present	1893.13	0.29
202787_s_at	included	present	126.67	-0.26	excluded	absent	75.81	-0.62	excluded	absent	19.45	-1.02	excluded	absent	66.98	-0.69	excluded	absent	82.43	-0.58	included	present	218.43	0.39	included	present	129.54	-0.24	included	present	346	1.3	included	present	113.92	-0.35	included	present	88.38	-0.53	included	present	163.56	0	included	present	422.83	1.84	included	present	124.49	-0.28	excluded	absent	72.99	-0.64	included	present	216.83	0.38
202788_at	included	present	488.51	-0.39	included	present	1523.11	0.16	included	present	329.99	-0.47	included	present	172.77	-0.55	included	present	439.72	-0.41	included	present	1537.16	0.17	included	present	378.74	-0.44	included	present	1388.64	0.09	included	present	398.45	-0.43	included	present	297.22	-0.49	included	present	231.45	-0.52	included	present	2954.9	0.92	included	present	371.13	-0.45	included	present	215.26	-0.53	included	present	1047.64	-0.09
202789_at	included	present	1818.23	0.01	included	present	3729.3	2.95	included	present	1476.89	-0.51	included	present	621.11	-1.83	included	present	2427.94	0.95	included	present	1592.8	-0.33	included	present	917.33	-1.37	included	present	1078.38	-1.13	included	present	953.53	-1.32	included	present	1434.85	-0.58	included	present	1266	-0.84	included	present	567.08	-1.91	included	present	938.39	-1.34	included	present	448.42	-2.09	included	present	1353.12	-0.7
202790_at	excluded	absent	9.47	-0.37	excluded	absent	4.76	-0.39	excluded	absent	27.43	-0.32	included	present	177.23	0.17	excluded	absent	8.26	-0.38	excluded	absent	108.65	-0.05	excluded	absent	17.17	-0.35	excluded	absent	19.88	-0.34	excluded	absent	75.78	-0.16	excluded	absent	6.05	-0.38	excluded	absent	43.25	-0.26	excluded	absent	34.22	-0.29	excluded	absent	6.62	-0.38	excluded	absent	13.96	-0.36	excluded	absent	26.78	-0.32
[illegible]																																																													

203115_at	included	absent	123.93	-1.29	included	present	400.73	0.07	included	present	500.83	0.57	included	present	319.15	-0.33	included	absent	262.59	[illegible]	[illegible]
203116_s_at	included	present	413.51	-0.57	included	present	1242.31	2.49	included	present	795.99	0.84	included	present	463.65	-0.38	included	present	1216.45	[illegible]	[illegible]
203117_s_at	included	present	371.51	-0.86	included	present	516.66	-0.25	included	present	264.06	-1.32	included	present	436.14	-0.59	included	present	400.49	[illegible]	[illegible]
203118_at	included	present	206.25	-0.51	included	present	254.11	-0.35	included	absent	89.55	-0.9	included	present	167.02	-0.64	included	absent	11.64	[illegible]	[illegible]
203119_at	included	present	372.13	0.15	included	present	712.43	1.81	included	present	210.04	-0.65	included	present	215.85	-0.62	included	present	388.27	[illegible]	[illegible]
203120_at	included	present	304.73	-1.23	included	present	790.64	-0.04	included	present	455.54	-0.86	included	present	593.31	-0.52	included	absent	397.2	[illegible]	[illegible]
203122_at	included	present	1286.51	0.03	included	present	1913.01	0.87	included	present	2660.72	1.87	included	present	1168.73	-0.13	included	present	1089.44	[illegible]	[illegible]
203123_s_at	included	present	1565.35	-0.5	included	present	1385.7	-0.67	included	present	803.17	-1.22	included	present	1985.92	-0.1	included	present	1314.07	[illegible]	[illegible]
203124_s_at	included	present	656.71	-0.65	included	present	795.16	-0.49	included	present	394.45	-0.96	included	present	781.78	-0.5	included	present	430.22	[illegible]	[illegible]
203125_x_at	included	present	577.07	0.28	included	present	317.65	-0.99	included	present	432.51	-0.43	included	present	596.99	0.38	included	present	635.1	[illegible]	[illegible]
203126_at	included	absent	86.87	-0.42	included	absent	139.75	-0.38	included	present	222.05	-0.33	included	present	585.55	-0.09	included	absent	237.02	[illegible]	[illegible]
203127_s_at	included	present	1703.13	0.84	included	present	861.29	-0.8	included	present	1144.18	-0.25	included	present	2109	1.64	included	present	749.02	[illegible]	[illegible]
203128_at	included	present	3818.96	1.57	included	present	1338.39	-0.38	included	present	3141.42	1.04	included	present	5889.33	3.2	included	present	1176.83	[illegible]	[illegible]
203129_s_at	included	present	9534.89	0.52	included	present	12363.65	0.91	included	present	12173.51	0.88	included	present	5065.03	-0.11	included	present	12065.75	[illegible]	[illegible]
203130_s_at	included	present	9083.94	-0.14	included	present	21987.9	0.86	included	present	12606.39	0.13	included	present	6895.02	-0.31	included	present	13471.44	[illegible]	[illegible]
203131_at	included	present	1268.73	-0.62	included	present	421.22	-1.04	included	absent	60.8	-1.22	included	absent	7.65	-1.24	included	present	485.21	[illegible]	[illegible]
203132_at	included	present	1135.4	1.09	included	present	890.99	0.32	included	present	986.51	0.62	included	present	600.48	-0.6	included	present	949.97	[illegible]	[illegible]
203133_at	included	present	985.64	-0.17	included	present	608.88	-0.65	included	present	901.78	-0.28	included	present	1577.91	0.57	included	present	1414.4	[illegible]	[illegible]
203134_at	excluded	absent	366.83	0.7	included	present	333.46	0.4	included	present	402.66	1.03	excluded	absent	168.37	-1.1	excluded	absent	299.13	[illegible]	[illegible]
203135_at	included	present	1013.79	-0.34	included	present	865.86	-0.5	included	present	1686.72	0.4	included	present	1359.69	0.04	included	present	1135.25	[illegible]	[illegible]
203136_at	included	present	680.73	2.67	included	present	492.69	1.25	included	present	256.21	-0.53	included	present	273.32	-0.4	included	present	157.19	[illegible]	[illegible]
203137_at	included	present	1043.11	-0.44	included	present	719.07	-0.92	included	present	894.3	-0.66	included	present	1274.33	-0.1	included	present	1853.45	[illegible]	[illegible]
203138_at	included	present	3508.16	-0.04	included	present	3358.63	-0.13	included	present	4892.2	0.87	included	present	6555.96	1.97	included	present	4135.6	[illegible]	[illegible]
203139_at	included	present	1344.16	-0.51	included	present	3376.09	1.06	included	present	2656.63	0.5	included	present	3163.99	0.89	included	present	3700.44	[illegible]	[illegible]
203140_at	included	present	662.78	-0.45	included	absent	147.94	-0.64	included	present	154.82	-0.64	included	present	292.45	-0.59	included	present	788.09	[illegible]	[illegible]
203141_s_at	included	present	1310.97	0.17	included	present	569.11	-1.69	included	present	1409.04	0.42	included	present	851.96	-0.98	included	present	1605.71	[illegible]	[illegible]
203142_s_at	included	present	337.92	0.54	included	absent	93.07	-1.79	included	present	244.5	-0.35	included	present	300.82	0.19	included	present	264.45	[illegible]	[illegible]
203143_s_at	included	present	156.99	-0.23	excluded	absent	165.24	-0.18	excluded	absent	41.82	-0.97	excluded	absent	115.58	-0.49	included	present	311.32	[illegible]	[illegible]
203144_s_at	excluded	absent	51.56	-0.97	included	present	198.57	0.43	excluded	absent	3.08	-1.44	excluded	absent	40.12	-1.08	included	present	273.74	[illegible]	[illegible]
203145_at	included	present	149.02	-0.32	included	present	1640.59	1.68	included	present	1570.53	1.58	included	present	713.8	0.44	included	present	1820.27	[illegible]	[illegible]
203146_s_at	included	present	1749.22	-0.34	included	present	3428.3	0.31	included	present	400.74	-0.87	included	present	455.1	-0.85	included	present	738.41	[illegible]	[illegible]
203147_s_at	included	present	1195.47	1.21	included	present	1240.09	1.31	included	present	1552.57	1.97	included	present	291.71	-0.71	included	present	827.37	[illegible]	[illegible]
203148_s_at	included	present	349.8	0.37	included	present	410.78	0.62	included	present	572.64	1.28	included	absent	112.24	-0.6	included	present	323.2	[illegible]	[illegible]
203149_at	excluded	absent	200.41	-0.28	excluded	absent	151.74	-0.5	excluded	absent	118.5	-0.65	included	present	806.52	2.48	excluded	absent	24.21	[illegible]	[illegible]
[illegible]	[illegible]	[illegible]	[illegible]	[illegible]	[illegible]	[illegible]	[illegible]	[illegible]	[illegible]	[illegible]	[illegible]	[illegible]	[illegible]	[illegible]	[illegible]	[illegible]	[illegible]	[illegible]	[illegible]	[illegible]	[illegible]

203476_at	included	present	756.49	-0.25	included	absent	168.49	-0.58	included	present	140.36	-0.6	included	present	1374.01	0.1	included	present	165.16	-0.59	included	present	1943.36	0.42	included	present	1115.56	-0.05	included	present	124.61	-0.61	included	present	766.07	-0.25	included	present	192.93	-0.57	included	present	121.15	-0.61	included	present	586.16	-0.35	included	present	414.43	-0.44	included	present	474.56	-0.41	included	present	108.59	-0.62
203477_at	included	present	3861.24	2.78	included	present	716.47	-0.14	included	present	344.41	-0.49	included	present	219.95	-0.6	included	present	506.76	-0.33	included	present	4730.95	3.59	included	present	516.13	-0.33	included	present	382.49	-0.45	included	present	2576.36	1.59	included	present	1351.83	0.45	included	present	289.82	-0.54	included	present	1830.68	0.89	included	present	3175.99	2.14	included	present	1638.63	0.72	included	present	255.51	-0.57
203478_at	included	present	901.01	-0.75	included	present	1222.84	-0.33	included	present	772.56	-0.92	included	present	1161.88	-0.41	included	present	1019.69	-0.6	included	present	3376.17	2.45	included	present	3080.15	2.07	included	present	1710.55	0.3	included	present	1581.39	0.13	included	present	3931.69	3.17	included	present	1148.61	-0.43	included	present	2079.61	0.78	included	present	1776.8	0.38	included	present	1972.89	0.64	included	present	3743.65	2.93
203479_s_at	included	present	686.2	0.69	included	present	817.24	1.1	included	present	709.55	0.76	included	present	844.68	1.19	included	present	1062.08	1.87	included	present	470.58	0.01	included	present	399.62	-0.22	included	present	944.17	1.5	included	present	612.16	0.45	included	present	579.21	0.35	included	present	572.14	0.33	included	present	482.45	0.04	included	present	350.84	-0.37	included	present	558.36	0.28	included	present	662.83	0.61
203480_s_at	included	present	1092.74	-0.54	included	present	1076.05	-0.58	included	present	1140.75	-0.43	included	present	1550.03	0.55	included	present	851.94	-1.12	included	present	1786.87	1.12	included	present	1084.65	-0.56	included	present	1337.85	0.05	included	present	1059	-0.62	included	present	866.97	-1.08	included	present	1055.11	-0.63	included	present	1210.48	-0.26	included	present	2101.12	1.88	included	present	2219.96	2.16	included	present	955.61	-0.87
203481_at	included	present	2041	0.59	included	present	775.92	-1.08	included	present	1499.98	-0.12	included	present	1328.01	-0.35	included	present	771.73	-1.08	included	present	958.63	-0.84	included	present	677.56	-1.21	included	present	866.56	-0.96	included	present	1092.51	-0.66	included	present	1492.34	-0.13	included	present	1243.26	-0.46	included	present	596.74	-1.31	included	present	1294.99	-0.39	included	present	948.9	-0.85	included	present	1030.12	-0.74
203482_at	included	present	2056.16	0.22	included	present	1090.83	-1.07	included	present	1132.62	-1.01	included	present	1608.09	-0.38	included	present	623.78	-1.69	included	present	1222.67	-0.89	included	present	818.23	-1.43	included	present	976.69	-1.22	included	present	910.42	-1.31	included	present	1900.7	0.01	included	present	1223.71	-0.89	included	present	2206.22	0.42	included	present	1888.07	0	included	present	1658.17	-0.31	included	present	1295.35	-0.79
203483_at	excluded	absent	4.39	-0.87	excluded	absent	3.52	-0.9	excluded	absent	5.24	-0.84	excluded	absent	23.22	-0.17	excluded	absent	11.29	-0.61	excluded	absent	6.27	-0.8	excluded	absent	12.25	-0.58	excluded	absent	16.59	-0.42	excluded	absent	50.46	0.84	excluded	absent	21.96	-0.22	excluded	absent	26.73	-0.04	excluded	absent	11.3	-0.61	excluded	absent	57.41	1.1	excluded	absent	34.48	0.25	excluded	absent	21.15	-0.25
203484_at	included	present	5723.28	-0.62	included	present	6598.19	-0.44	included	present	11653.12	0.6	included	present	11487.69	0.56	included	present	7883.8	-0.18	included	present	6715.98	-0.42	included	present	8593.2	-0.03	included	present	6034.19	-0.56	included	present	3948.89	-0.99	included	present	5982.04	-0.57	included	present	9180.72	0.09	included	present	10722.71	0.4	included	present	5557.91	-0.66	included	present	8160.74	-0.12	included	present	9175.7	0.09
203485_at	included	present	15705.03	0.31	included	present	21947.09	0.75	included	present	16370.26	0.35	included	present	3916.05	-0.52	included	present	15996.06	0.33	included	present	996.19	-0.73	included	present	21952.02	0.75	included	present	5875.66	-0.39	included	present	14765.39	0.24	included	present	20375.02	0.64	included	present	13521.78	0.15	included	present	27387.64	1.13	included	present	21315.62	0.7	included	present	13426.81	0.15	included	present	16622.28	0.37
203486_s_at	included	present	635.22	-0.16	included	present	539.36	-0.57	included	present	1023.09	1.46	included	present	745.39	0.3	included	present	925.45	1.05	included	present	766.71	0.39	included	present	689.3	0.06	included	present	658.33	-0.07	included	present	676.74	0.01	included	present	843.95	0.71	included	present	549.32	-0.52	included	present	729.15	0.23	included	present	545.67	-0.54	included	present	448.04	-0.95	included	present	509.64	-0.69
203487_s_at	included	present	451.26	-0.67	included	present	305.38	-1.13	included	present	561	-0.32	included	present	576.15	-0.28	included	present	434.76	-0.72	included	present	524.06	-0.44	included	present	670.12	0.02	included	present	781.08	0.37	included	present	593.06	-0.22	included	present	851.56	0.59	included	present	691.46	0.09	included	present	1059.35	1.24	included	present	1196.46	1.68	included	present	1081.31	1.31	included	present	731.64	0.21
203488_at	excluded	absent	91.9	-0.37	excluded	absent	205.2	0.59	excluded	absent	73.27	-0.53	included	present	248.05	0.96	excluded	absent	153.81	0.15	included	present	400.16	2.25	included	present	651.79	4.39	included	present	596.91	3.93	included	present	447.1	2.65	included	present	643.77	4.33	included	present	239.76	0.89	included	present	262.98	1.08	excluded	absent	7.91	-1.09	excluded	absent	48.59	-0.74	included	present	1125.24	8.43
203489_at	excluded	absent	170.24	0.91	excluded	absent	22.67	-1.5	excluded	absent	149.62	0.58	excluded	absent	203.82	1.46	excluded	absent	12.15	-1.67	included	present	323.87	3.43	excluded	absent	79.42	-0.57	included	present	280.88	2.72	excluded	absent	169.55	0.9	included	present	191.67	1.26	included	present	419.02	4.99	excluded	absent	118.37	0.06	excluded	absent	75.6	-0.64	excluded	absent	124.39	0.16	included	present	262.3	2.42
203490_at	included	absent	116.98	-0.59	included	absent	164.82	-0.33	included	absent	64.91	-0.87	included	present	502.16	1.49	included	present	177.48	-0.26	included	present	811.91	3.16	included	present	93.14	-0.72	included	present	540.38	1.7	included	present	239.33	0.07	included	absent	100.91	-0.68	included	present	195.5	-0.17	included	present	719.84	2.66	included	present	117.16	-0.59	included	present	160.33	-0.36	included	present	223.72	-0.01
203491_s_at	included	present	803.38	-0.47	included	present	1995.32	2.16	included	present	1712.01	1.54	included	present	1697.42	1.5	included	present	781.61	-0.52	included	present	752.82	-0.58	included	present	613.38	-0.89	included	present	910.93	-0.23	included	present	669.33	-0.76	included	present	871.18	-0.32	included	present	743.22	-0.6	included	present	340.71	-1.49	included	present	1390.12	0.83	included	present	1414.75	0.88	included	present	1161.65	0.32
203492_x_at	included	present	725.68	-0.69	included	present	1439.79	0.75	included	present	1362.83	0.6	included	present	1500.34	0.87	included	present	544.99	-1.05	included	present	972.41	-0.19	included	present	476.96	-1.19	included	present	1386.32	0.64	included	present	893.14	-0.35	included	present	872.33	-0.39	included	present	768.93	-0.6	included	present	975.61	-0.19	included	present	837.93	-0.46	included	present	915.97	-0.31	included	present	735.65	-0.67
203493_s_at	included	present	490.15	-0.17	included	present	912.82	1.46	included	present	1301.55	2.95	included	present	1291.26	2.91	included	present	459.48	-0.29	included	present	472.99	-0.24	included	present	351.31	-0.7	included	present	684.1	0.58	included	present	491.34	-0.17	included	present	201.19	-1.28	included	present	345.46	-0.73	included	present	704.99	0.66	included	present	1040.71	1.95	included	present	509.23	-0.1	included	present	344.62	-0.73
203494_s_at	included	present	765.41	-0.69	included	present	2335.85	1.34	included	present	1998.46	0.9	included	present	2017.69	0.93	included	present	528.23	-1	included	present	1217.44	-0.11	included	present	1267.54	-0.04	included	present	1670.89	0.48	included	present	1615.32	0.41	included	present	795.25	-0.65	included	present	926.21	-0.48	included	present	3205.11	2.46	included	present	2710.8	1.82	included	present	1612.61	0.4	included	present	1098.36	-0.26
203495_at	excluded	absent	25.28	-2.97	excluded	absent	133.72	-0.9	excluded	absent	123.43	-1.1	excluded	absent	128.57	-1	excluded	absent	164.39	-0.32	included	present	499.01	6.08	included	present	393.3	4.06	included	present	363.07	3.48	included	present	268.38	1.67	excluded	absent	168.69	-0.23	included	present	237.1	1.07	included	present	471.98	5.56	excluded	absent	222.28	0.79	excluded	absent	131.02	-0.95	excluded	absent	269.14	1.69
203496_s_at	included	present	673.42	0.34	included	present	1579.57	4.64	included	present	816.2	1.02	included	present	707.77	0.5	included	present	1429.73	3.93	included	present	435.89	-0.78	included	present	1017.92	1.98	included	present	1071.72	2.23	included	present	647.27	0.22	included	present	861.16	1.23	included	present	1199.38	2.84	included	present	662.71	0.29	included	present	1129.79	2.51	included	present	2014.49	6.7	included	present	1355.02	3.57
203497_at	included	present	1808.03	1.37	included	present	3694.04	6.56	included	present	4375.49	8.44	included	present	3231.79	5.29	included	present	4467.81	8.69	included	present	2115.45	2.22	included	present	3813.23	6.89	included	present	3193.7	5.18	included	present	2146.59	2.3	included	present	1646.54	0.93	included	present	3763.33	6.75	included	present	1655.18	0.95	included	present	3156.01	5.08	included	present	3547.65	6.16	included	present	3756.77	6.73
203498_at	included	present	2505.19	-0.58	included	present	501.06	-1.05	included	present	119.79	-1.14	included	present	309.01	-1.1	included	present	569.42	-1.04	included	present	1937.51	-0.71	included	present	962.23	-0.94	included	present	371.51	-1.08	included	present	935.16	-0.95	included	present	1226.84	-0.88	included	present	299.26	-1.1	included	present	1328.9	-0.85	included	present	4493.89	-0.1	included	present	1557.32	-0.8	included	present	123.86	-1.14
203499_at	included	present	344.29	0.07	included	present	155.34	-0.87	included	absent	143.06	-0.93	included	absent	36.4	-1.45	included	absent	78.49	-1.25	included	present	629.1	1.47	included	absent	81.43	-1.23	included	absent	38.46	-1.44	included	present	361.87	0.15	included	present	211.61	-0.59	included	present	229.03	-0.5	included	present	222.91	-0.53	included	absent	151.66	-0.88	included	absent	97.58	-1.15	included	present	105.82	-1.11
203500_at	included	absent	164.03	-0.54	included	present	221.97	-0.29	included	absent	137.53	-0.65	included	present	163.47	-0.54	included	absent	143.93	-0.62	included	absent	212.38	-0.33	included	absent	172.6	-0.5	included	present	344.83	0.23	included	absent	158.06	-0.56	included	present	276.2	-0.06	included	absent	129.13	-0.68	included	present	253	-0.16	included	absent	101.38	-0.8	included	absent	83.17	-0.88	included	present	126.88	-0.69
203501_at	included	present	557.28	0.4	included	present	194.6	-1.04	included	present	158.78	-1.18	included	absent	108.35	-1.38	included	absent	126.4	-1.31	included	present	1282.28	3.28	included	absent	98.7	-1.42	included	present	231.16	-0.89	included	present	672.01	0.85	included	present	340.1	-0.46	included	absent	279.79	-0.7	included	present	788.71	1.32	included	present	439.85	-0.07	included	present	520.42	0.25	included	present	96.89	-1.43
203502_at	included	present	1196.09	1.28	included	present	1086.3	0.95	included	present	531.01	-0.76	included	present	647.81	-0.4	included	present	1026.57	0.76	included	present	556.03	-0.68	included	present	1337.8	1.72	included	present	747.02	-0.1	included	present	1285.66	1.56	included	present	291.28	-1.5	included	present	748.1	-0.09	included	present	879.08	0.31	included	present	926.76	0.46	included	present	557.9	-0.68	included	present	808.92	0.09
203503_s_at	included	present	127.13	0.85	included	present	137.55	1.09	excluded	absent	51.48	-0.84	excluded	absent	60.02	-0.65	excluded	absent	85.71	-0.07	excluded	absent	125.71	0.82	included	present	243.79	3.46	included	present	283.53	4.35	excluded	absent	147.93	1.32	included	present	221.29	2.96	excluded	absent	109.92	0.47	included	present	154.76	1.47	excluded	absent	60.19	-0.64	excluded	absent	67	-0.49	included	present	337.08	5.54
203504_s_at	included	present	1872.33	0.93	included	present	888.75	-0.18	included	present	445.4	-0.67	included	present	412.16	-0.71	included	present	710.56	-0.38	included	present	1719.65	0.75	included	present	1287.17	0.27	included	present	858.13	-0.21	included	present	1638.83	0.66	included	present	671.6	-0.42	included	present	1033.66	-0.01	included	present	5298.74	4.76	included	present	1727.34	0.76	included	present	2023.93	1.1	included	present	581.3	-0.52
203505_at	included	present	308.32	0.09	included	present	214.36	-0.27	included	present	144.19	-0.54	included	present	137.48	-0.56	included	absent	64.05	-0.85	included	present	399.44	0.44	included	present	281.52	-0.01	included	present	188.18	-0.37	included	present	427.19	0.54	included	present	196.14	-0.34	included	present	259.6	-0.1	included	present	1155.66	3.33	included	present	109.64	-0.67	included	present	233.38	-0.2	included	present	128.56	-0.6
203506_s_at	included	present	223.57	0.09	included	present	265.46	0.52	included	present	227.36	0.13	included	absent	139.56	-0.77	included	present	275.24	0.62	included	present	426.31	2.17	included	present	474.41	2.67	included	present	592.2	3.87	included	present	387.74	1.78	included	present	486.56	2.79	included	present	594.51	3.9	included	present	375.24	1.65	included	absent	178.5	-0.37	included	present	335.88	1.25	included	present	540.58	3.34
203507_at	excluded	absent	155.07	0.51	excluded	absent	117.92	0.22	excluded	absent	35.25	-0.43	excluded	absent	11.4	-0.62	excluded	absent	41.6	-0.38	included	present	250.56	1.26	excluded	absent	35.78	-0.43	excluded	absent	52.76	-0.29	included	present	247.02	1.23	excluded	absent	158.75	0.54	included	present	221.98	1.04	included	present	918.64	6.51	excluded	absent	136.36	0.36	excluded	absent	152.65	0.49	included	present	231.71	1.11
203508_at	included	present	553.39	0.26	included	present	382.89	-0.31	included	absent	245.8	-0.77	included	absent	17.5	-1.53	included	present	506.23	0.11	included	present	1771.33	4.35	included	present	299.47	-0.59	included	present	525.41	0.17	included	present	680.22	0.69	included	present	435.94	-0.13	included	present	523.95	0.17	included	present	958.86	1.62	included	present	477.25	0.01	included	absent	214.69	-0.87	included	present	804.97	1.11
203509_at	included	present	1275.03	-0.08	included	present	1117.46	-0.24	included	present	798.07	-0.55	included	present	397.12	-0.96	included	absent	566.87	-0.79	included	present	421.42	-0.93	included	present	821.86	-0.53	included	present	573.47	-0.78	included	present	874.24	-0.48	included	present	754.2	-0.6	included	present	1599.76	0.25	included	present	694.58	-0.66	included	present	394.62	-0.96	included	present	152.63	-1.2	included	present	722.08	-0.63
[illegible]																																																													

203835_at	included	present	156.47	-0.1	excluded	absent	22.33	-0.81	excluded	absent	39.8	-0.72
203836_s_at	included	present	482.38	-0.77	included	absent	139.44	-1.41	included	present	146.26	-1.4
203837_at	included	present	637	-0.54	included	present	237.58	-1.44	included	absent	121.83	-1.7
203838_s_at	excluded	absent	46.63	-0.4	excluded	absent	44.59	-0.43	excluded	absent	79.24	0
203839_s_at	included	absent	307.72	-0.5	included	absent	296.59	-0.54	included	present	159.77	-1.03
203840_at	included	present	148.35	-0.17	included	present	133.16	-0.39	included	present	177.97	0.26
203841_x_at	included	present	681.54	0.29	included	present	261.25	-0.56	included	present	252.98	-0.57
203842_s_at	included	present	290.27	-0.48	included	present	530.01	0.11	included	present	335	-0.37
203843_at	included	present	552.56	-0.15	included	absent	122.1	-1.6	included	present	320.13	-0.93
203844_at	included	present	178.11	0.35	excluded	absent	104.06	-0.69	excluded	absent	52.62	-1.42
203845_at	included	present	797.36	-0.39	included	present	154.13	-1.52	included	present	220.79	-1.41
203846_at	included	present	523.95	0.89	included	present	358.85	-0.24	included	present	839.95	3.07
203847_s_at	included	absent	484.52	-0.27	included	present	1090.48	4.57	included	absent	493.59	-0.2
203848_at	included	present	358.93	-1.58	included	present	689.38	-0.32	included	present	409.84	-1.38
203849_s_at	included	present	4236.74	1.81	included	present	4154.11	1.76	included	present	993.89	-0.15
203850_s_at	included	present	297.83	1.05	included	present	540.89	3.85	included	present	592.61	4.44
203851_at	included	present	2869.98	1.07	included	present	177.38	-0.45	included	present	90.27	-0.49
203852_s_at	included	present	1305.5	-0.26	included	present	922.54	-1.03	included	present	1921.38	0.97
203853_s_at	included	present	626.75	-0.73	included	present	1163.35	0.72	included	present	969.92	0.2
203854_at	included	present	1074.18	0.07	included	absent	119.98	-0.31	included	present	368.81	-0.21
203855_at	included	present	2080.56	0.53	included	present	1640.01	0.16	included	present	1371.07	-0.06
203856_at	included	present	675.12	-0.73	included	present	2070.05	1.08	included	present	2116.78	1.14
203857_s_at	included	present	730.77	0.27	included	present	189.28	-0.61	included	present	275.64	-0.47
203858_s_at	included	present	132.73	-0.58	included	present	245.71	0.13	included	present	172.71	-0.33
203859_s_at	excluded	absent	260	0.54	included	present	308.74	0.95	excluded	absent	166.62	-0.24
203860_at	included	present	668.89	-0.57	included	present	1454.34	0.1	included	present	798.46	-0.46
203861_s_at	excluded	absent	91.43	-0.24	included	present	901.92	-0.03	excluded	absent	17.91	-0.26
203862_s_at	excluded	absent	74.23	-0.39	included	present	825.06	-0.15	excluded	absent	38.53	-0.4
203863_at	excluded	absent	54.47	-0.3	included	present	139.81	-0.22	excluded	absent	4.15	-0.34
203864_s_at	included	present	74.95	-0.28	included	present	281.13	-0.14	excluded	absent	41.8	-0.3
203865_s_at	included	present	1050.22	-0.04	included	present	186.27	-1.03	included	present	149.68	-1.07
203866_at	excluded	absent	80.17	-0.28	included	present	392.88	5.72	excluded	absent	120.32	0.49
203867_s_at	excluded	absent	136.45	0.89	included	present	251.32	3.2	excluded	absent	52.22	-0.81
203868_s_at	included	present	560.1	-0.4	included	present	7582.99	0.62	included	present	457.29	-0.42

204194_at	included	present	897.29	1.19	included	present	817.87	0.93	included	present	482.8	-0.18	included	present	[illegible]
204195_s_at	included	present	339.61	0.69	included	absent	209.53	-0.28	included	present	314.99	0.51	included	present	[illegible]
204196_x_at	included	present	953.86	3.67	included	present	254.68	-1.39	included	present	1181.36	5.32	included	present	[illegible]
204197_s_at	excluded	absent	19.54	-0.44	included	present	142.42	-0.11	excluded	absent	77.22	-0.29	excluded	absent	[illegible]
204198_s_at	excluded	absent	5.92	-0.56	included	present	201.47	0.07	excluded	absent	92.2	-0.28	included	present	[illegible]
204199_at	included	present	101.04	-0.55	included	present	169.16	-0.01	included	present	103.45	-0.53	included	absent	[illegible]
204200_s_at	excluded	absent	76.52	0.34	excluded	absent	58.41	-0.06	excluded	absent	40.79	-0.45	excluded	absent	[illegible]
204201_s_at	included	present	684.89	0.64	included	present	152.25	-1.13	included	absent	98.46	-1.31	included	absent	[illegible]
204202_at	excluded	absent	114.03	-0.39	excluded	absent	129.76	-0.18	excluded	absent	26.89	-1.56	excluded	absent	[illegible]
204203_at	included	present	320.99	-0.65	included	present	473.99	-0.13	included	present	968.04	1.54	included	present	[illegible]
204204_at	included	present	254.08	-0.39	included	present	374.81	-0.31	included	present	168.77	-0.44	included	present	[illegible]
204205_at	included	present	692.96	0.23	included	present	383.2	-0.24	included	present	243.66	-0.45	included	present	[illegible]
204206_at	included	present	688.15	-0.77	included	present	3520.2	6.11	included	present	820.73	-0.44	included	present	[illegible]
204207_s_at	included	present	263.7	0.37	included	present	997.07	7.03	included	present	307.45	0.77	included	present	[illegible]
204208_at	included	present	244.05	-0.98	included	present	1019.83	3.8	included	present	365.55	-0.23	included	present	[illegible]
204209_at	included	present	400.21	0.37	included	present	230.23	-0.87	included	present	191.66	-1.16	included	present	[illegible]
204210_s_at	included	present	626.35	0.53	included	present	228.09	-1.21	included	present	587.82	0.36	included	present	[illegible]
204211_x_at	included	present	3522.9	4.52	included	present	977.48	-0.4	included	present	4553.99	6.51	included	present	[illegible]
204212_at	excluded	absent	123.65	0.14	excluded	absent	23.49	-1.57	excluded	absent	55.16	-1.03	excluded	absent	[illegible]
204213_at	excluded	absent	6.57	-0.3	excluded	absent	5.68	-0.3	excluded	absent	28.23	-0.29	excluded	absent	[illegible]
204214_s_at	included	present	472.82	0.45	included	present	413.9	0.23	included	present	418.78	0.25	excluded	absent	[illegible]
204215_at	included	present	483.61	-0.23	included	present	489.74	-0.21	included	present	413.62	-0.45	included	present	[illegible]
204216_s_at	included	present	1841.32	-0.47	included	present	3477.13	0.41	included	present	3254.72	0.29	included	present	[illegible]
204217_s_at	excluded	absent	157.44	0.48	excluded	absent	243.96	1.07	excluded	absent	23.48	-0.45	excluded	absent	[illegible]
204218_at	included	present	539.32	-1.98	included	present	1315.53	0.57	included	present	885.56	-0.84	included	present	[illegible]
204219_s_at	included	present	6144.64	-0.19	included	present	7368.05	0.35	included	present	6911.81	0.15	included	present	[illegible]
[illegible]	[illegible]	[illegible]	[illegible]	[illegible]	[illegible]	[illegible]	[illegible]	[illegible]	[illegible]	[illegible]	[illegible]	[illegible]	[illegible]	[illegible]	[illegible]

204553_x_at	included	present	774.96	0.82	included	present	625.99	0.28	included	present	613.03	0.24	included	absent	127.2	-1.52	included	present	760.87	0.77	included	present	449.12	-0.36	included	present	557.85	0.04	included	present	777.35	0.83	included	present	655.03	0.39	included	present	757.35	0.76	included	present	531.71
204554_at	included	present	220.7	0.02	included	present	181.1	-0.34	included	absent	101.64	-1.06	included	present	104.44	-1.04	included	absent	137.51	-0.73	included	present	209.57	-0.08	included	present	217.83	0	included	present	161.26	-0.52	included	present	244.05	0.23	included	present	176.03	-0.38	included	present	91.85
204555_s_at	excluded	absent	39.26	-1.41	excluded	absent	105.87	-0.38	excluded	absent	100.91	-0.45	included	present	132.72	0.04	excluded	absent	142.34	0.19	excluded	absent	212.52	1.28	excluded	absent	123.61	-0.1	included	present	226.13	1.49	excluded	absent	147.11	0.26	included	present	158.03	0.43	excluded	absent	76.8
204556_s_at	included	present	846.51	-0.32	included	present	1017.76	-0.23	included	present	1441.17	-0.01	included	present	50.75	-0.73	included	present	1053.34	-0.21	included	present	447.45	-0.53	included	present	547.2	-0.47	included	present	1263.83	-0.1	included	present	566.61	-0.46	included	present	682.96	-0.4	included	absent	73.03
204557_s_at	included	present	726.31	-0.01	included	present	566.86	-0.32	included	present	869.43	0.27	included	absent	46.19	-1.33	included	present	459.91	-0.53	included	present	434.2	-0.58	included	present	1099.6	0.71	included	present	1157.67	0.83	included	present	664.69	-0.13	included	present	405.45	-0.64	included	present	57.45
204558_at	excluded	absent	134.27	0.52	included	present	322.89	3.27	excluded	absent	143.42	0.66	included	present	295.5	2.87	included	present	181.98	1.22	excluded	absent	42.94	-0.8	excluded	absent	146.69	0.71	excluded	absent	222.56	1.81	excluded	absent	120.91	0.33	excluded	absent	26.1	-1.05	excluded	absent	235.28
204559_s_at	included	present	892.14	0.22	included	present	1645.61	2.49	included	present	2248.48	4.31	included	present	1741.87	2.78	included	present	1033.53	0.65	included	present	1414.62	1.8	included	present	2009.62	3.59	included	present	2449.48	4.92	included	present	1437.62	1.87	included	present	2148.33	4.01	included	present	1496.06
204560_at	included	present	1375.62	-0.5	included	present	959.54	-0.59	included	present	3185.38	-0.11	included	absent	133.56	-0.77	included	present	1878.09	-0.4	included	absent	353.41	-0.72	included	present	500.74	-0.69	included	present	1503.47	-0.48	included	present	864.9	-0.61	included	present	1824.72	-0.41	included	present	1761.99
204561_x_at	included	present	333.36	-0.15	excluded	absent	218.47	-0.17	included	present	450.35	-0.13	excluded	absent	13.31	-0.2	included	present	320.82	-0.15	excluded	absent	108.98	-0.18	included	present	531.36	-0.11	excluded	absent	29.88	-0.2	included	present	294.48	-0.15	included	present	854.07	-0.06	included	present	825.54
204562_at	excluded	absent	47.55	-0.37	excluded	absent	12.54	-0.5	excluded	absent	41.24	-0.39	excluded	absent	2.19	-0.54	excluded	absent	202.99	0.22	excluded	absent	9.48	-0.51	excluded	absent	35.48	-0.41	excluded	absent	23.3	-0.46	included	present	164.41	0.08	excluded	absent	2.84	-0.53	excluded	absent	79.03
204563_at	included	present	204.83	-0.22	included	present	178.69	-0.25	included	absent	70.34	-0.35	included	present	66.98	-0.36	included	present	382.26	-0.05	included	present	671.6	0.24	included	present	94.07	-0.33	included	absent	1.56	-0.42	included	present	949.22	0.51	included	absent	3.98	-0.42	included	present	111.12
204564_at	included	present	347.9	-0.3	excluded	absent	468.23	0.54	included	present	522.61	0.92	included	present	541.32	1.05	included	absent	249.99	-0.98	included	present	653.49	1.83	included	present	350.61	-0.28	included	present	603.88	1.49	included	present	1003.33	4.28	included	present	622.7	1.62	included	present	510.13
204565_at	included	present	1449.85	-0.34	included	present	1974.69	0.11	included	present	403.83	-1.23	included	present	2334.92	0.42	included	present	1147.72	-0.59	included	present	1611.23	-0.2	included	present	1369.63	-0.4	included	present	951.31	-0.76	included	present	722.72	-0.96	included	present	1152.36	-0.59	included	present	387.83
204566_at	included	present	585.14	0.09	included	present	996.52	1.57	included	present	1831.46	4.58	included	present	2249.64	6.09	included	present	1129.09	2.05	included	present	585.84	0.09	included	present	2398.11	6.62	included	present	1551.89	3.57	included	present	1020.24	1.66	included	present	1113.3	1.99	included	present	1473.45
204567_s_at	included	present	1000.12	-0.34	included	present	1969.53	0.79	included	present	976.11	-0.36	included	present	4692.05	3.96	included	present	1053.72	-0.27	included	present	1203.39	-0.1	included	present	1690.93	0.47	included	present	758.92	-0.62	included	present	1012.72	-0.32	included	present	930.69	-0.42	included	present	1200.16
204568_at	included	present	1209.31	-0.72	included	present	1121.76	-0.89	included	present	1088.96	-0.95	included	present	1509.79	-0.13	included	present	1131.77	-0.87	included	present	515.67	-2.07	included	present	1093.37	-0.94	included	present	1388.68	-0.37	included	present	1386.29	-0.37	included	present	1362.29	-0.42	included	present	1108.28
204569_at	included	present	529.25	-0.3	included	present	326.81	-0.71	included	present	789.33	0.22	included	present	607.53	-0.14	included	present	261.02	-0.84	included	present	477.6	-0.4	included	present	1115.71	0.87	included	present	313.02	-0.73	included	present	551.68	-0.26	included	present	402.24	-0.55	included	present	766.45
204570_at	included	present	1602.52	-0.32	included	absent	263.62	-0.46	included	present	225.34	-0.47	included	present	564.41	-0.43	included	present	2869.68	-0.18	included	present	5672.67	0.13	included	present	274.99	-0.46	included	present	2439.64	-0.22	included	present	2032.92	-0.27	included	present	11455.4	0.77	included	present	2122.75
204571_x_at	included	present	4264	-0.46	included	present	9690.24	2.02	included	present	8659.31	1.55	included	present	5074.83	-0.09	included	present	5525.04	0.12	included	present	3879.76	-0.63	included	present	3872.17	-0.64	included	present	3551.55	-0.78	included	present	2458.2	-1.29	included	present	3472.83	-0.82	included	present	4065.29
204572_s_at	included	present	568.63	0.05	included	present	261.85	-1.4	included	present	900.35	1.61	included	present	444.51	-0.54	included	present	939.92	1.79	included	present	280.79	-1.31	included	present	2406.31	8.7	included	present	451.4	-0.51	included	present	499.05	-0.28	included	present	508.09	-0.24	included	present	645.49
204573_at	included	present	1521.63	0.88	included	present	1293.71	0.57	included	present	643.2	-0.31	included	present	203.08	-0.91	included	present	527.87	-0.47	included	present	701.7	-0.23	included	present	1060.25	0.25	included	present	608.15	-0.36	included	present	1052.36	0.24	included	present	744.92	-0.18	included	present	503.39
204574_s_at	included	present	1069.14	4.66	included	present	409.06	0.56	included	present	434.01	0.72	included	present	137.24	-1.13	included	present	267.03	-0.32	included	present	325.5	0.04	included	present	203.13	-0.72	included	present	225.37	-0.58	included	present	225.94	-0.58	included	present	319.58	0	included	present	434
204575_s_at	excluded	absent	222.09	0.71	excluded	absent	8.63	-0.78	excluded	absent	9.52	-0.78	excluded	absent	1.43	-0.83	excluded	absent	121.37	0.01	included	present	716.88	4.17	included	present	177.61	0.4	excluded	absent	28.65	-0.64	included	present	195.69	0.53	excluded	absent	82.3	-0.27	included	present	146.15
204576_s_at	included	present	1526.33	-0.26	included	present	2647.38	0.81	included	present	1692.04	-0.1	included	present	2175.15	0.36	included	present	1209.88	-0.56	included	present	1056.51	-0.71	included	present	1070.41	-0.7	included	present	1867.45	0.06	included	present	1269.24	-0.51	included	present	940.62	-0.82	included	present	958.81
204577_s_at	included	present	362.17	2.96	included	present	282.55	1.88	included	present	310.09	2.25	excluded	absent	152.63	0.1	excluded	absent	28.77	-1.59	excluded	absent	19.71	-1.72	excluded	absent	209.5	0.88	excluded	absent	128.67	-0.23	included	present	224.78	1.09	included	present	251.73	1.45	excluded	absent	149.91
204578_at	included	present	293.37	-0.98	included	present	170.65	-1.62	included	present	486.57	0.02	included	present	387.2	-0.5	included	present	488.23	0.03	included	present	228.22	-1.32	included	present	436.09	-0.24	included	present	973.04	2.55	included	present	418.05	-0.34	included	present	683.59	1.04	included	present	434.34
204579_at	excluded	absent	38.47	-0.86	included	present	157.63	0.11	excluded	absent	48.25	-0.78	excluded	absent	117.89	-0.22	included	present	153.73	0.08	included	present	221	0.62	excluded	absent	75.8	-0.56	excluded	absent	144.7	0	excluded	absent	99.09	-0.37	excluded	absent	89.6	-0.45	excluded	absent	99.96
204580_at	excluded	absent	1.08	-0.29	included	present	552.8	1.37	excluded	absent	46.78	-0.15	included	present	301.63	0.62	included	present	312.69	0.65	excluded	absent	56.01	-0.13	included	present	2231.2	6.44	excluded	absent	28.11	-0.21	included	present	446.72	1.05	excluded	absent	7.44	-0.27	included	present	364.1
204581_at	excluded	absent	11.78	-0.65	excluded	absent	8.37	-0.66	excluded	absent	11.64	-0.65	excluded	absent	4.87	-0.66	excluded	absent	5.54	-0.66	excluded	absent	64.51	-0.53	excluded	absent	3.43	-0.67	excluded	absent	3.08	-0.67	included	present	236.69	-0.16	excluded	absent	2.96	-0.67	excluded	absent	11.52
204582_s_at	excluded	absent	41.06	-0.16	excluded	absent	76.27	-0.15	excluded	absent	75.12	-0.15	excluded	absent	2.92	-0.16	excluded	absent	64.99	-0.15	excluded	absent	61.02	-0.15	excluded	absent	85.31	-0.15	included	present	487.87	-0.12	excluded	absent	63.85	-0.15	excluded	absent	41.33	-0.16	excluded	absent	11.19
204583_x_at	excluded	absent	4.04	-0.15	excluded	absent	9.11	-0.15	excluded	absent	54.66	-0.14	excluded	absent	6.95	-0.15	excluded	absent	15.73	-0.15	excluded	absent	19.55	-0.15	excluded	absent	8.06	-0.15	excluded	absent	35.25	-0.15	excluded	absent	12.97	-0.15	excluded	absent	10.45	-0.15	excluded	absent	19.91
204584_at	included	present	4225.3	13.34	included	present	2784.98	8.4	included	present	1171.87	2.87	included	present	158.01	-0.61	included	present	1606.14	4.36	included	absent	259.81	-0.26	included	present	5187.23	16.63	included	present	2670.07	8	included	present	2178.71	6.32	included	present	2680.13	8.04	included	present	1780.68
204585_s_at	excluded	absent	181.19	4.22	excluded	absent	30.02	0.15	excluded	absent	40.36	0.43	excluded	absent	4.86	-0.53	excluded	absent	90.29	1.77	excluded	absent	54.25	0.8	included	present	295.15	7.28	excluded	absent	165.31	3.79	excluded	absent	234.1	5.64	included	present	464.63	11.84	excluded	absent	362.82
204586_at	included	present	123.5	-0.5	included	present	535.68	0.4	included	present	87.19	-0.58	included	present	291.81	-0.13	excluded	absent	427.89	0.16	excluded	absent	179.48	-0.38	included	present	821.22	1.02	included	present	707.77	0.78	included	present	286.03	-0.15	included	present	331.56	-0.05	included	present	493.46
204587_at	included	present	1143.7	-0.38	included	present	2480.71	1.25	included	present	1182.26	-0.33	included	present	831.21	-0.76	included	present	1330.82	-0.15	included	present	678.55	-0.94	included	present	1338.6	-0.14	included	present	1056.23	-0.48	included	present	773.71	-0.83	included	present	1181.06	-0.33	included	present	862.8
204588_s_at	included	present	271.27	-0.3	included	present	517.69	-0.16	included	present	237.5	-0.32	included	present	185.24	-0.35	included	present	540.93	-0.15	included	present	491.7	-0.18	included	present	263.79	-0.31	included	present	243.53	-0.32	included	present	347.21	-0.26	included	present	128.29	-0.38	included	present	581.82
204589_at	included	present	1975.75	-0.49	included	present	1414.87	-0.67	included	present	302.57	-1.03	included	present	5666.65	0.72	included	present	871.35	-0.85	included	present	7356.33	1.27	included	present	556.09	-0.95	included	present	1026.69	-0.8	included	present	1708.29	-0.57	included	present	1902.17	-0.51	included	present	1770.38
204590_x_at	included	present	573.53	-0.25	included	present	1392.01	4.02	included	present	391.73	-1.2	included	present	388.5	-1.21	included	present	926.34	1.59	included	present	869.04	1.29	included	present	1435.59	4.24	included	present	1017.47	2.06	included	present	971.63	1.83	included	present	1190.65	2.97	included	present	793
204591_at	included	present	4948.56	1.38	included	present	1252.11	-0.31	included	absent	128.52	-0.82	included	present	4.84	-0.88	included	present	397.93	-0.7	included	present	561.37	-0.62	included	present	1443.03	-0.22	included	present	217.83	-0.78	included	present	498.58	-0.65	included	present	1293.48	-0.29	included	present	344.98
204592_at	excluded	absent	61.87	-0.66	included	present	90.58	-0.6	included	absent	41.48	-0.69	excluded	absent	50.92	-0.68	excluded	absent	5.69	-0.76	excluded	absent	182.64	-0.44	included	present	112.34	-0.56	included	present	133.14	-0.53	included	present	162.39	-0.47	excluded	absent	73.68	-0.63	excluded	absent	108.74
204593_s_at	included	absent	179.2	-0.91	included	present	247.25	-0.13	included	present	489.71	2.64	included	present	402.93	1.65	included	absent	203.22	-0.64	included	absent	212.89	-0.52	included	present	431.74	1.97	included	present	833.94	6.57	included	present	439.45	2.06	included	present	304.31	0.52	included	present	414.32
204594_s_at	included	present	232.57	-0.36	included	present	517.35	3.13	included	present	411.44	1.83	included	absent	210.17	-0.63	included	present	212.07	-0.61	included	present	327.3	0.8	included	present	539.15	3.39	included	present	1158.42	10.97	included	present	465	2.49	included	present	644.72	4.69	included	present	521.6
204595_s_at	included	present	227.59	0.25	excluded	absent	9.85	-0.59	excluded	absent	76.6	-0.33	excluded	absent	22.66	-0.54	excluded	absent	106.81	-0.21	excluded	absent	120.7	-0.16	included	present	104.57	-0.22	included	present	144.2	-0.07	excluded	absent	75.01	-0.34	excluded	absent	68.85	-0.36	included	present	368.29
204596_s_at	included	present	341.03	0.2	excluded	absent	10.98	-0.57	excluded	absent	96.4	-0.37	excluded	absent	43.52	-0.49	excluded	absent	197.5	-0.14	included	present	450.24	0.45	included	present	318.25	0.14	included	present	397.74	0.33	included	present	163.4	-0.21	included	present	211.37	-0.1	included	present	542.95
204597_x_at	included	present	805.12	-0.13	included	present	353.5	-0.43	included	present	449.79	-0.37	included	present	99.7	-0.6	included	present	559.17	-0.3	included	present	2412.49	0.93	included	present	1606.63	0.4	included	present	1755.41	0.5	included	present	586.93	-0.28	included	present	347.58	-0.44	included	present	1489.56
204598_at	excluded	absent	124.7	0.18	excluded	absent	71.6	-0.85	excluded	absent	73.45	-0.81	excluded	absent	82.08	-0.65	excluded	absent	98.25	-0.34	excluded	absent	91.51	-0.47	excluded	absent	152.99	0.72	excluded	absent	140.9	0.49	excluded	absent	122.21	0.13	included	present	279.43	3.17	included	present	206.55
204599_s_at	excluded	absent	249.3	-0.69	included	present	521.87	1.83	included	present	284.81	-0.36	excluded	absent	123.09	-1.86	excluded	absent	292.71	-0.29	included	present	492.6	1.56	included	present	567.56	2.25	included	present	2131.09	16.71	included	present	513.54	1.75	included	present	637.44	2.9	excluded	absent	553.19
204600_at	excluded	absent	48.31	-0.83	excluded	absent	138.17	0.75	excluded	absent	13.26	-1.44	excluded	absent	85.12	-0.18	excluded	absent	64.48	-0.54	excluded	absent	239.42	2.52	included	present	240.02	2.53	included	present	285.65	3.33	excluded	absent	193.36	1.71	excluded	absent	503.22	7.14	excluded	absent	139.56
204601_at	included	present	472.45	1.57	included	present	534.91	2.12	included	present	723.45	3.8	included	present	599.85	2.7	included	present	330.33	0.3	included	present	355.87	0.53	included	present	477.65	1.61	included	present	620.02	2.88	included	present	509.02	1.89	included	present	495.92	1.77	included	present	392.32
204602_at	excluded	absent	9.18	-0.37	excluded	absent	39.69	-0.2	excluded	absent	2.34	-0.41	excluded	absent	1.65	-0.41	included	present	127.43	0.3	included	present	137.67	0.36	excluded	absent	35.07	-0.22	excluded	absent	3.86	-0.4	excluded	absent	45.54	-0.16	excluded	absent	4.36	-0.4	excluded	absent	0.42
204603_at	excluded	absent	38.64	-0.17	included	present	863.67	14.04	included	present	1331.37	22.09	included	present	702.6	11.26	excluded	absent	193.55	2.5	excluded	absent	125.27	1.32	excluded	absent	32.64	-0.28	included	present	466.34	7.19	excluded	absent	63.03	0.25	excluded	absent	108.72	1.04	included	present	877.98
204604_at	included	present	1647.21	-0.19	included	present	1182.16	-0.45	included	present	227.12	-0.98	included	present	2639.39	0.36	included	present	1453.38	-0.3	included	present	1850.95	-0.08	included	present	910.39	-0.6	included	present	938.36	-0.58	included	present	1187.77	-0.45	included	present	1435.18	-0.31	included	present	1877.04
204605_at	included	present	684.06	-0.16	included	present	632.91	-0.31	included	present	552.49	-0.54	included	present	1042.13	0.88	included	present	551.49	-0.54	included	present	475.07	-0.76	included	present	636.57	-0.29	included	present	1256.17	1.49	included	present	805.64	0.19	included	present	649.49	-0.26	included	present	752.18
204606_at	included	present	573.66	-0.16	included	present	3480.29	0.39	included	absent	38.95	-0.26	included	absent	36.26	-0.26	included	absent	57.04	-0.26	included	present	9107.16	1.47	included	present	935.47	-0.09	included	present	492.58	-0.17	included	present	6297.48	0.93	included	absent	83.9	-0.25	included	present	377.26
204607_at	excluded	absent	3.3	-0.27	excluded	absent	3.15	-0.27	included	present	240.99	-0.18	included	present	79.65	-0.24	excluded	absent	4.19	-0.27	excluded	absent	110.17	-0.23	excluded	absent	9.16	-0.26	excluded	absent	39.8	-0.25	excluded	absent	59.89	-0.25	included	present	64.07	-0.24	excluded	absent	2.42
204608_at	included	present	347.14	-0.14	included	present	777.17	0.22	included	present	247.96	-0.23	included	absent	238.86	-0.24	included	present	127.86	-0.33	included	present	619.5	0.08	included	present	680.09	0.14	included	present	780.98	0.22	included	present	472.96	-0.04	included	present	636.51	0.1	included	present	542.02
204609_at	excluded	absent	38.35	-0.08	excluded	absent	9.71	-0.89	excluded	absent	2	-1.11	excluded	absent	44.04	0.09	excluded	absent	25.07	-0.45	excluded	absent	72.05	0.88	excluded	absent	24.48	-0.47	excluded	absent	31.48	-0.27	excluded	absent	1.83	-1.12	excluded	absent	2.65	-1.09	excluded	absent	3.87
204610_s_at	excluded	absent	50.85	-1.19	excluded	absent	173.99	0.2	excluded	absent	78.02	-0.88	excluded	absent	35.47	-1.37	excluded	absent	73.86	-0.93	included	present	514.41	4.06	excluded	absent	116.97	-0.44	included	present	403.7	2.8	included	present	155.8	0	included	present	210.88	0.62	excluded	absent	59.98
204611_s_at	excluded	absent	133.96	-0.43	excluded	absent	151.45	-0.19	excluded	absent	3.89	-2.29	excluded	absent	110.01	-0.78	excluded	absent	155.18	-0.13	excluded	absent	223.06	0.83	excluded	absent	395.65	3.29	excluded	absent	199.99	0.51	excluded	absent	369.47	2.92	excluded	absent	235.38	1.01	excluded	absent	72.26
204612_at	included	present	766.99	-0.46	included	present	3537.83	1.2	included	present	682.33	-0.51	included	present	346.46	-0.71	included	present	2267.32	0.44	included	present	1128.29	-0.24	included	present	5825.13	2.56	included	present	1883.73	0.21	included	present	2310.36	0.46	included	present	4878.38	2	included	present	2130.84
204613_at	included	present	889.97	-0.39	included	present	590.72	-0.64	included	present	261.64	-0.92	included	present	196.41	-0.97	included	present	1213.6	-0.11	included	present	1107.22	-0.2	included	present	179.54	-0.99	included	present	380.67	-0.82	included	present	824.57	-0.44	included	present	448.49	-0.76	included	present	393.82
204614_at	excluded	absent	79.63	-0.18	excluded	absent	5.74	-0.32	excluded	absent	4.47	-0.32	excluded	absent	4.29	-0.32	excluded	absent	101.52	-0.13	excluded	absent	44.75	-0.24	excluded	absent	30.91	-0.27	excluded	absent	0.61	-0.33	excluded	absent	1.21	-0.33	included	present	1092.86	1.75	excluded	absent	31.33
204615_x_at	included	present	7699.24	0.82	included	present	7375.38	0.71	included	present	3322.2	-0.68	included	present	4207.32	-0.38	included	present	8628.84	1.14	included	present	1872.86	-1.18	included	present	1689.25	-1.24	included	present	1765.72	-1.22	included	present	1793.75	-1.21	included	present	3388.04	-0.66	included	present	2461.64
204616_at	included	present	705.2	-0.5	included	present	1067.06	0.35	included	present	876.9	-0.1	included	present	406.21	-1.2	included	present	1204.19	0.67	included	present	948.29	0.07	included	present	861.26	-0.13	included	present	1198.3	0.66	included	present	690.59	-0.53	included	present	1043.76	0.3	included	present	757.58
204617_s_at	included	absent	143.79	-0.57	included	present	645.36	5.61	included	present	259.36	0.85	included	present	202.96	0.16	included	absent	74.3	-1.43	included	present	462.64	3.36	included	present	723.1	6.57	included	present	816.85	7.72	included	present	339.68	1.84	included	present	1578.72	17.11	included	present	667.04
204618_s_at	included	present	567.26	-0.51	included	present	815.97	0.22	included	present	1545.28	2.39	included	present	1312.34	1.7	included	present	909.13	0.5	included	present	645.91	-0.28	included	present	939.22	0.59	included	present	1675.89	2.78	included	present	946.53	0.61	included	present	935.4	0.58	included	present	1550
204619_s_at	included	present	477.06	0.7	included	present	298.35	-0.03	included	present	535.97	0.94	included	present	642.15	1.38	included	present	257.8	-0.2	included	present	3253.72	12.08	included	present	360.58	0.22	included	absent	52.61	-1.04	included	present	1055.85	3.07	included	present	164.75	-0.58	included	present	149.39
204620_s_at	included	present	2748.37	2.08	included	present	1539.75	0.64	included	present	3245.57	2.68	included	present	4545.2	4.23	included	present	2324.44	1.58	included	present	10451.32	11.27	included	present	958.41	-0.05	included	present	96.08	-1.08	included	present	2351.09	1.61	included	present	1007.64	0.01	included	present	588.01
204621_s_at	included	present	888.4	0.84	included	absent	63.45	-0.67	included	absent	29.86	-0.73	included	present	374.45	-0.1	included	absent	9.07	-0.77	included	present	666.18	0.44	included	absent	64.08	-0.67	included	absent	68.87	-0.66	included	present	307.65	-0.22	included	present	688.4	0.48	included	present	75.37
204622_x_at	included	present	744.4	0.24	included	absent	92.7	-0.59	included	absent	32.51	-0.67	included	present	367.05	-0.24	included	present	179.09	-0.48	included	present	1173.63	0.79	included	present	96.16	-0.58	included	absent	23.05	-0.68	included	present	246.24	-0.39	included	present	678.68	0.16	included	absent	45.75
204623_at	excluded	absent	86.1	-0.2	excluded	absent	81.45	-0.22	excluded	absent	20.1	-0.49	included	present	573.24	1.98	excluded	absent	62.38	-0.3	included	present	253.61	0.55	included	present	75.52	-0.24	included	absent	68.97	-0.27	included	present	346.17	0.96	excluded	absent	49.28	-0.36	excluded	absent	20.43
204624_at	excluded	absent	69.28	-0.56	included	present	240.06	1.52	included	present	159.52	0.54	excluded	absent	57.91	-0.69	excluded	absent	94.03	-0.25	excluded	absent	62.95	-0.63	included	present	247.95	1.62	included	present	73.74	-0.5	excluded	absent	52.81	-0.76	excluded	absent	64.16	-0.62	excluded	absent	10.37
204625_s_at	excluded	absent	94.88	0.43	excluded	absent	71.59	0.02	excluded	absent	11.04	-1.04	excluded	absent	6.72	-1.12	included	present	359.72	5.06	included	present	186.8	2.03	excluded	absent	88.82	0.32	excluded	absent	9.87	-1.06	excluded	absent	34.87	-0.62	excluded	absent	20.24	-0.88	excluded	absent	61.4
204626_s_at	included	present	1027.27	0.98	included	present	309.69	-0.3	included	present	303.53	-0.31	included	present	310.54	-0.29	included	present	1985.43	2.68	included	present	1533.84	1.88	included	present	485.86	0.02	included	present	234.29	-0.43	included	present	665.94	0.34	included	present	337.98	-0.25	included	present	728.42
204627_s_at	included	present	1683.47	-0.03	included	present	269.58	-0.64	included	present	582	-0.51	included	present	413.48	-0.58	included	present	3283.15	0.67	included	present	2085.07	0.15	included	present	299.73	-0.63	included	present	404.21	-0.59	included	present	785.34	-0.42	included	present	212.97	-0.67	included	present	748.16
204628_s_at	included	present	411.73	0.61	included	absent	88.14	-0.65	included	present	191.63	-0.25	included	absent	120.74	-0.52	included	present	765.95	1.99	included	present	626.53	1.45	included	present	123.13	-0.51	included	absent	60.09	-0.76	included	present	255.95	0	included	absent	79.24	-0.68	included	present	299.64
204629_at	included	present	149.35	-0.1	excluded	absent	212.72	0.37	excluded	absent	88.98	-0.54	excluded	absent	98.98	-0.46	included	present	151.33	-0.08	included	present	338.97	1.29	excluded	absent	142.43	-0.15	included	present	380.19	1.59	included	present	267.31	0.76	included	present	218.49	0.41	included	present	322.95
204630_s_at	included	present	445.89	-1.32	included	present	401.45	-1.54	included	present	472.09	-1.19	included	present	617.01	-0.48	included	present	451.73	-1.29	included	present	1240.06	2.56	included	present	1304.69	2.88	included	present	1359.92	3.15	included	present	721.93	0.03	included	present	648.48	-0.33	included	present	617.03
204631_at	excluded	absent	15.48	-0.17	excluded	absent	33.49	-0.17	excluded	absent	8.91	-0.18	excluded	absent	1.27	-0.18	excluded	absent	14.47	-0.17	included	present	20731.59	6.03	excluded	absent	1.97	-0.18	included	present	125.8	-0.14	excluded	absent	28.16	-0.17	included	present	16212.89	4.67	excluded	absent	6.07
204632_at	excluded	absent	100.43	0.71	excluded	absent	9.18	-1.02	excluded	absent	13.61	-0.94	excluded	absent	28.39	-0.66	excluded	absent	16.81	-0.88	excluded	absent	130.95	1.29	excluded	absent	113.37	0.95	excluded	absent	115.58	0.99	excluded	absent	78.5	0.29	excluded	absent	74.61	0.22	excluded	absent	77.88
204633_s_at	included	present	304.99	-0.79	included	present	275.44	-0.88	included	present	337.07	-0.69	included	present	566.99	0	included	present	259.56	-0.92	included	present	242.61	-0.97	included	present	318.43	-0.75	included	present	369.37	-0.59	included	present	431.02	-0.41	included	present	460.04	-0.32	included	present	488.29
204634_at	included	present	153.32	-0.63	included	present	191.74	-0.5	included	present	268.48	-0.25	included	present	279.88	-0.21	included	present	277.09	-0.22	included	present	303.42	-0.13	included	present	460.2	0.4	included	present	335.59	-0.02	included	present	285.62	-0.19	included	present	176.59	-0.55	included	present	495.02

[illegible]

204912_at	included	present	284.96	-0.12	included	present	382.56	0.13	included	absent	72.42	-0.67	included	absent	52.35	-0.72	included	absent	212.77
204913_s_at	included	present	543.65	1.83	included	present	7219.47	34.17	included	present	15871.19	76.09	included	present	525.38	1.75	included	present	4358.28
204914_s_at	included	present	227.8	1.73	included	present	4679.58	53.54	included	present	7182.28	82.67	included	present	169.22	1.04	included	present	2396.17
204915_s_at	included	present	637.33	1.36	included	present	16496.94	54.37	included	present	12717.23	41.73	included	present	620.05	1.3	included	present	6120.07
204916_at	included	absent	119.18	-0.86	included	present	190.06	-0.56	included	present	233.47	-0.38	included	absent	65.24	-1.08	included	absent	185.16
204917_s_at	included	present	362.07	-0.68	included	present	471.41	-0.26	included	present	713.36	0.67	included	present	733.47	0.75	included	present	397.11
204918_s_at	included	absent	133.73	-0.9	included	absent	144.03	-0.81	included	present	104.15	-1.15	included	present	246.04	0.05	included	present	193.08
204919_at	included	present	741.69	-0.2	included	present	1035.59	-0.19	included	present	921.17	-0.19	included	present	1014.1	-0.19	included	present	910.98
204920_at	included	present	134.68	-0.23	included	present	117.4	-0.23	included	present	1312.35	-0.12	included	present	186.37	-0.23	included	absent	181.57
204921_at	excluded	absent	5.82	-2.21	included	present	269.43	1.94	excluded	absent	5.86	-2.21	excluded	absent	57.87	-1.39	excluded	absent	66.91
204922_at	excluded	absent	132.37	0.5	excluded	absent	149.3	0.73	excluded	absent	113.43	0.25	excluded	absent	163.88	0.92	excluded	absent	25.85
204923_at	included	present	292.55	-0.09	included	present	320.53	-0.04	included	absent	106.96	-0.45	included	absent	51.82	-0.55	included	present	304.79
204924_at	included	present	2988.5	1.31	included	present	864.8	-0.51	included	present	349.79	-0.95	included	absent	110.63	-1.16	included	present	942.21
204925_at	included	present	269.43	1.57	included	present	350.62	2.4	excluded	absent	79.25	-0.38	excluded	absent	67.49	-0.5	excluded	absent	222.19
204926_at	included	present	177.18	-0.05	excluded	absent	41.94	-0.43	included	present	181.03	-0.04	included	present	216.09	0.06	included	present	605.28
204927_at	excluded	absent	81.63	-1.21	excluded	absent	97.52	-0.99	excluded	absent	94.73	-1.03	excluded	absent	156.43	-0.19	excluded	absent	5.63
204928_s_at	excluded	absent	176.49	0.56	excluded	absent	139.87	0.16	excluded	absent	63.29	-0.68	excluded	absent	136.89	0.12	excluded	absent	15.49
204929_s_at	included	present	956.62	1.31	included	absent	180.27	-1.2	included	absent	239.6	-1.01	included	absent	104.61	-1.45	excluded	absent	569.51
204930_s_at	included	present	114.29	-0.1	included	present	151.22	0.48	included	present	121.11	0.01	included	present	138.47	0.28	excluded	absent	183.32
204931_at	excluded	absent	36.88	-0.53	included	present	79.58	-0.37	excluded	absent	5.63	-0.65	excluded	absent	96.35	-0.31	excluded	absent	91.87
204932_at	excluded	absent	63.14	0.4	excluded	absent	3.8	-0.93	excluded	absent	4.5	-0.91	excluded	absent	1.42	-0.98	excluded	absent	5.62
204933_s_at	excluded	absent	78.57	-0.65	included	present	183.82	-0.35	excluded	absent	235.37	-0.21	excluded	absent	75.58	-0.66	excluded	absent	156.97
204934_s_at	excluded	absent	41.34	-0.3	excluded	absent	124.17	-0.2	included	present	108.03	-0.22	excluded	absent	11.7	-0.34	excluded	absent	68.1
204935_at	excluded	absent	69.58	0.18	excluded	absent	3.35	-1.35	excluded	absent	114.85	1.23	excluded	absent	83.81	0.51	excluded	absent	86.26
204936_at	excluded	absent	42.64	-0.41	excluded	absent	103.11	0.53	excluded	absent	2.57	-1.03	excluded	absent	19.03	-0.78	excluded	absent	57.2
204937_s_at	included	present	343.09	-0.55	included	present	681.85	1.24	included	present	154.19	-1.54	included	present	1574.63	5.95	included	present	483.86
204938_s_at	included	present	601.05	-0.49	included	present	128.84	-0.67	included	present	200.98	-0.64	included	present	179.09	-0.65	included	present	285.29
204939_s_at	included	absent	60.08	-0.5	included	absent	2.79	-0.51	included	absent	42.12	-0.5	included	absent	63.06	-0.5	included	present	198.18
204940_at	excluded	absent	42.48	-0.52	excluded	absent	37.6	-0.53	excluded	absent	18.02	-0.57	excluded	absent	1.35	-0.6	excluded	absent	97.33
204941_s_at	excluded	absent	36.17	0.06	excluded	absent	5.35	-0.39	excluded	absent	47.51	0.22	excluded	absent	17.87	-0.21	excluded	absent	8.07
204942_s_at	excluded	absent	69.67	-0.22	excluded	absent	4.74	-0.33	excluded	absent	74.11	-0.21	excluded	absent	6.2	-0.32	excluded	absent	3.75
204943_at	excluded	absent	16.79	-0.35	excluded	absent	12.23	-0.46	excluded	absent	4.37	-0.65	excluded	absent	8.33	-0.56	excluded	absent	105.11
204944_at	included	present	599.36	-0.17	included	present	415.52	-0.78	included	present	366.24	-0.94	included	present	678.87	0.1	included	present	495.9
204945_at	included	present	200.38	0.21	included	present	366.24	1.21	included	present	215.24	0.3	excluded	absent	144.26	-0.13	excluded	absent	149.92

[illegible]

205271_s_at	included	present	341.02	-0.07	included	present	409.82	0.24	included	present	238.21	-0.54	included	present	249.31	-0.49
205272_s_at	excluded	absent	15.41	-0.17	excluded	absent	37.07	-0.17	excluded	absent	114.66	-0.17	excluded	absent	120.77	-0.17
205273_s_at	included	present	610.27	-0.78	included	present	272.83	-1.77	included	present	677.18	-0.58	included	present	541.11	-0.98
205274_at	excluded	absent	72.97	0.18	excluded	absent	9.6	-1.26	excluded	absent	40.06	-0.56	excluded	absent	67.94	0.07
205275_at	excluded	absent	48.86	-0.83	excluded	absent	132.49	0.61	excluded	absent	63.2	-0.58	excluded	absent	8.57	-1.52
205276_s_at	excluded	absent	99.6	0.14	excluded	absent	48.07	-0.74	excluded	absent	112.01	0.35	excluded	absent	8.91	-1.4
205277_at	included	present	911.8	-0.46	included	present	1115.96	-0.23	included	present	241.14	-1.24	included	present	233.98	-1.24
205278_at	included	absent	83.54	-0.62	included	absent	35.44	-0.64	included	absent	2.71	-0.66	included	present	1385.9	-0.01
205279_s_at	included	present	103.17	-0.54	excluded	absent	48.07	-0.83	included	present	113.15	-0.49	included	present	385.4	0.97
205280_at	included	present	188.79	-0.14	included	present	111.6	-0.45	excluded	absent	34.82	-0.75	included	present	439.46	0.84
205281_s_at	included	present	70.91	-1.21	included	absent	126.19	-0.85	included	present	306.15	0.33	included	present	187.85	-0.44
205282_at	included	present	112.52	-0.26	included	present	324.19	0.49	included	present	125.59	-0.21	included	present	216.45	0.11
205283_at	included	present	294.05	0.8	included	present	159.94	-0.83	included	present	325	1.18	included	present	222.95	-0.06
205284_at	excluded	absent	181.19	0.08	included	present	453.91	3.12	included	present	979.77	8.97	included	present	514.38	3.79
205285_s_at	included	present	642.96	0.24	included	present	263.13	-0.21	included	present	140.12	-0.35	included	absent	54.93	-0.45
205286_at	included	present	184.25	-0.34	included	absent	3.18	-0.41	included	absent	68.84	-0.39	included	absent	2.51	-0.41
205287_s_at	excluded	absent	7.66	-0.38	excluded	absent	4.02	-0.4	excluded	absent	4.31	-0.39	excluded	absent	19.42	-0.35
205288_at	included	present	92.57	-0.96	included	present	203.32	-0.22	included	present	100.75	-0.91	included	present	103.03	-0.89
205289_at	excluded	absent	34.92	-0.67	excluded	absent	68.26	-0.46	excluded	absent	49.21	-0.58	included	present	151.65	0.07
205290_s_at	included	absent	29.14	-0.91	included	absent	43.35	-0.83	included	absent	55.92	-0.75	included	present	329.19	0.83
205291_at	included	present	76.18	-0.37	included	present	407.32	0.33	excluded	absent	37.05	-0.45	included	present	122.26	-0.27
205292_s_at	included	present	19329.52	-1.57	included	present	33355.76	0.71	included	present	39584.3	1.72	included	present	27149.75	-0.3
205293_x_at	excluded	absent	67.32	-0.22	excluded	absent	48.35	-0.45	excluded	absent	71	-0.17	excluded	absent	35.81	-0.6
205294_at	included	present	334.13	-0.26	included	absent	146.15	-0.66	included	absent	27.3	-0.92	included	present	99.04	-0.76
205295_at	included	present	773.97	-0.25	included	present	233.04	-0.3	included	present	331.59	-0.29	included	present	685.38	-0.26
205296_at	included	present	213.9	0.45	included	present	325.93	1.63	included	present	539.63	3.87	included	present	384.17	2.24
205297_s_at	excluded	absent	79.93	-0.37	excluded	absent	14.03	-0.79	excluded	absent	24.61	-0.73	excluded	absent	64.42	-0.47
205298_s_at	included	present	643.65	-0.03	included	present	450.75	-0.33	included	present	208.92	-0.71	included	present	348.09	-0.49
205299_s_at	included	present	105.4	-0.17	excluded	absent	57.71	-0.74	included	present	106.8	-0.16	excluded	absent	59.4	-0.72
205300_s_at	included	present	943.94	-0.41	included	present	2780.42	3.86	included	present	1513.45	0.92	included	present	2047.07	2.15
205301_s_at	included	present	124.32	-0.44	included	present	171.76	0.07	included	absent	110.58	-0.59	included	absent	62.86	-1.1
205302_at	excluded	absent	6.96	-0.19	excluded	absent	3.59	-0.19	excluded	absent	63.85	-0.16	excluded	absent	56.57	-0.16
205303_at	included	absent	16.2	-0.85	included	present	392.8	-0.19	included	present	157.05	-0.6	included	present	585.66	0.14
205304_s_at	included	present	161.57	-0.7	included	present	660.57	-0.22	included	present	421.69	-0.45	included	present	1108.1	0.21

205630_at	included	present	297.2	0.58	excluded	absent	6.59	-0.37	excluded	absent	86.15	-0.11	excluded	absent	3.03	-0.38	excluded	absent	172.23	0.17	excluded	absent	41.94	-0.25	included	present	1357.94	4.04	included	present	1450.86	4.35	included	present	2033.67	6.25	excluded	absent	116.9	-0.01	excluded	absent	33.6	-0.28	excluded	absent	4.15	-0.38	included	present	129.47	0.03	included	present	11627.75	37.56	included	present	5232.16	16.69
205631_at	included	present	237.22	-0.13	included	present	301.52	0.33	included	present	172.99	-0.59	included	present	185.93	-0.5	included	absent	193.63	-0.44	included	present	207.53	-0.34	included	absent	124.72	-0.93	included	present	191.98	-0.45	included	present	137.75	-0.84	included	present	281.07	0.18	included	present	186.3	-0.49	included	present	140.94	-0.82	included	present	387.57	0.95	included	present	316.76	0.44	included	present	174.45	-0.58
205632_s_at	included	present	133	-0.52	included	absent	39.94	-0.7	included	present	17.88	-0.74	included	present	582.85	0.33	included	absent	44.63	-0.69	included	absent	161.41	-0.47	included	present	77.77	-0.63	included	present	207.88	-0.38	included	present	132.73	-0.52	included	present	65.23	-0.65	included	present	78.51	-0.63	included	present	201.01	-0.39	included	present	50.77	-0.68	included	absent	21.18	-0.74	included	absent	37.48	-0.71
205633_s_at	included	present	505.2	-0.22	included	present	1052.09	0.01	included	present	358.24	-0.29	included	present	221.76	-0.35	included	present	390.21	-0.27	included	present	763.87	-0.11	included	present	861.72	-0.07	included	present	1601.55	0.25	included	present	828.95	-0.08	included	present	841.84	-0.08	included	present	565.8	-0.2	included	present	509.81	-0.22	included	present	439.8	-0.25	included	present	344.02	-0.29	included	present	1912.07	0.39
205634_x_at	excluded	absent	31.86	-0.76	excluded	absent	14.58	-1.15	excluded	absent	50.23	-0.35	excluded	absent	91.27	0.57	excluded	absent	117.83	1.17	included	present	233.24	3.76	included	present	211.82	3.28	included	present	205.98	3.15	included	present	220.26	3.47	included	present	217.29	3.4	excluded	absent	117.17	1.15	included	present	147.75	1.84	excluded	absent	27.34	-0.87	excluded	absent	126.17	1.36	included	present	220.62	3.48
205635_at	excluded	absent	78.39	-0.57	excluded	absent	189.69	-0.47	excluded	absent	5.02	-0.64	excluded	absent	2.14	-0.64	excluded	absent	30.05	-0.62	excluded	absent	55.32	-0.59	excluded	absent	83.91	-0.57	included	present	102.23	-0.55	excluded	absent	110.51	-0.54	included	present	577.84	-0.11	excluded	absent	166.04	-0.49	excluded	absent	95.17	-0.56	included	present	230.65	-0.43	included	present	96.27	-0.56	excluded	absent	79.04	-0.57
205636_at	included	present	127.59	-0.38	included	present	121.73	-0.41	excluded	absent	75.86	-0.65	excluded	absent	29.9	-0.89	excluded	absent	11.47	-0.98	excluded	absent	128.06	-0.38	excluded	absent	81.85	-0.62	excluded	absent	28.99	-0.89	included	present	124.29	-0.4	excluded	absent	40.53	-0.83	included	present	247.4	0.24	included	present	129.94	-0.37	included	present	164.15	-0.19	included	present	67.24	-0.69	included	present	48.53	-0.79
205637_s_at	included	present	114.53	-0.67	excluded	absent	106.22	-0.67	included	present	298.86	-0.57	excluded	absent	2.33	-0.73	excluded	absent	7.52	-0.73	excluded	absent	7.93	-0.73	included	present	483.43	-0.47	included	present	197.58	-0.62	included	present	310.81	-0.56	included	present	148.69	-0.65	included	present	1290.62	-0.02	included	present	291.78	-0.57	included	present	891.67	-0.24	included	present	895.11	-0.24	included	present	1377.67	0.02
205638_at	included	present	1148.41	0.57	included	present	1622.4	1.17	included	present	530.93	-0.22	included	absent	1.76	-0.89	included	present	471.99	-0.29	included	absent	121.79	-0.74	included	present	1429.66	0.92	included	present	212.03	-0.62	included	present	479.71	-0.28	included	present	1700.08	1.27	included	present	272.29	-0.55	included	present	318.37	-0.49	included	present	1602.53	1.14	included	present	1092.41	0.49	included	present	909.44	0.26
205639_at	included	present	209.88	-0.06	included	present	306.96	0.52	included	present	153.3	-0.4	excluded	absent	72.51	-0.88	included	present	405.82	1.11	included	present	361.48	0.84	included	present	162.29	-0.34	excluded	absent	118.34	-0.61	included	present	369.64	0.89	excluded	absent	116.67	-0.62	included	present	250.59	0.18	included	present	443.35	1.33	included	present	249.77	0.18	included	present	114.64	-0.63	included	present	367.9	0.88
205640_at	included	present	384.07	0.84	excluded	absent	52.62	-0.65	included	present	225.98	0.13	excluded	absent	100.41	-0.43	included	present	186.62	-0.05	included	present	345.03	0.66	excluded	absent	19.2	-0.8	excluded	absent	76.97	-0.54	included	present	121.58	-0.34	excluded	absent	38.51	-0.71	excluded	absent	164.59	-0.15	included	present	335.4	0.62	excluded	absent	15.32	-0.82	excluded	absent	14.36	-0.82	included	present	132.37	-0.29
205641_s_at	excluded	absent	79.97	-1.44	excluded	absent	185.21	-0.53	excluded	absent	104.26	-1.23	excluded	absent	169.48	-0.66	excluded	absent	7.58	-2.08	included	present	598.29	3.07	excluded	absent	155.53	-0.79	excluded	absent	247.46	0.02	excluded	absent	296.37	0.44	excluded	absent	200.08	-0.4	excluded	absent	199.97	-0.4	excluded	absent	357.35	0.97	excluded	absent	394.49	1.3	included	present	293.6	0.42	excluded	absent	223.27	-0.2
205642_at	included	present	201	-0.34	included	absent	44.36	-0.75	included	present	352.42	0.07	included	present	280.03	-0.13	included	absent	193.9	-0.35	included	absent	186.17	-0.37	included	absent	136.17	-0.51	included	present	223.16	-0.28	included	present	405.49	0.21	included	present	261.81	-0.17	included	present	288	-0.1	included	present	256.73	-0.19	included	absent	232.69	-0.25	included	present	320.01	-0.02	included	present	237.46	-0.24
205643_s_at	included	present	357.44	1.27	excluded	absent	37.81	-0.69	excluded	absent	30.87	-0.73	included	present	83.19	-0.41	included	present	217.62	0.42	excluded	absent	64.37	-0.52	included	present	390.31	1.47	excluded	absent	56.75	-0.57	included	present	184.64	0.21	excluded	absent	47	-0.63	excluded	absent	37.43	-0.69	excluded	absent	84.94	-0.4	included	present	138.73	-0.07	excluded	absent	5.09	-0.89	included	present	448.4	1.83
205644_s_at	included	present	6212.03	-0.97	included	present	13024.42	1.28	included	present	13633.08	1.48	included	present	8478.5	-0.22	included	present	8648.19	-0.17	included	present	7760.23	-0.46	included	present	4797.65	-1.44	included	present	8455.94	-0.23	included	present	4271.24	-1.62	included	present	4894.58	-1.41	included	present	6918.7	-0.74	included	present	11177.19	0.67	included	present	7736.82	-0.47	included	present	5716.43	-1.14	included	present	7111.46	-0.68
205645_at	included	present	302.4	-0.71	included	present	160.2	-1.06	included	absent	103.52	-1.2	included	present	155.11	-1.07	included	absent	275.12	-0.78	included	absent	148.38	-1.09	included	present	208.09	-0.94	included	present	252.22	-0.83	included	present	397.82	-0.47	included	present	166.05	-1.04	included	absent	127.42	-1.14	included	absent	154.55	-1.07	included	present	158.54	-1.06	included	absent	56.24	-1.31	included	present	159.39	-1.06
205646_s_at	excluded	absent	80.88	-0.62	excluded	absent	9.97	-0.89	excluded	absent	3.92	-0.91	included	present	347.07	0.36	excluded	absent	13.77	-0.87	excluded	absent	6.43	-0.9	excluded	absent	51.44	-0.73	excluded	absent	1.97	-0.92	excluded	absent	6.97	-0.9	excluded	absent	35.86	-0.79	included	present	90.25	-0.59	excluded	absent	86.76	-0.6	excluded	absent	14.09	-0.87	excluded	absent	30.75	-0.81	excluded	absent	8.15	-0.89
205647_at	included	present	958.97	0.23	included	present	881.99	-0.01	included	present	754.36	-0.41	included	present	998.97	0.35	included	present	961.65	0.23	included	present	638.48	-0.76	included	present	825.13	-0.19	included	present	318.45	-1.75	included	present	649.71	-0.73	included	present	888.55	0.01	included	present	1314.69	1.32	included	present	577.87	-0.95	included	present	1541.25	2.02	included	present	1507.08	1.92	included	present	387.54	-1.54
205648_at	excluded	absent	5.58	-0.41	excluded	absent	77.71	0.1	excluded	absent	5.33	-0.41	excluded	absent	1.65	-0.43	excluded	absent	5.16	-0.41	included	present	86.5	0.16	excluded	absent	35.28	-0.2	excluded	absent	16.47	-0.33	excluded	absent	29.83	-0.24	excluded	absent	49.99	-0.09	excluded	absent	22.91	-0.28	excluded	absent	7.67	-0.39	excluded	absent	60.37	-0.02	excluded	absent	3.7	-0.42	excluded	absent	6.29	-0.4
205649_s_at	excluded	absent	4.01	-0.15	excluded	absent	3.15	-0.15	included	present	134.6	-0.1	excluded	absent	43.38	-0.14	excluded	absent	2.29	-0.15	included	present	119.94	-0.11	excluded	absent	5.88	-0.15	excluded	absent	29.37	-0.14	excluded	absent	1.66	-0.15	excluded	absent	6.69	-0.15	excluded	absent	7.96	-0.15	excluded	absent	45.61	-0.14	excluded	absent	39.8	-0.14	excluded	absent	1.15	-0.15	excluded	absent	0.99	-0.15
205650_s_at	excluded	absent	3.48	-0.15	excluded	absent	2.44	-0.15	included	present	3337.38	0.1	included	present	370.31	-0.12	excluded	absent	22.24	-0.15	included	present	655.89	-0.1	excluded	absent	2.46	-0.15	excluded	absent	9.45	-0.15	excluded	absent	2.05	-0.15	excluded	absent	1.66	-0.15	excluded	absent	3.53	-0.15	included	present	321.76	-0.13	excluded	absent	0.84	-0.15	excluded	absent	1.33	-0.15	excluded	absent	4.26	-0.15
205651_x_at	included	present	256.22	-0.75	included	present	714.52	-0.47	included	present	775.33	-0.43	included	present	620.61	-0.53	included	present	805.5	-0.42	included	present	247.39	-0.76	included	present	1403.67	-0.05	included	present	1132.57	-0.22	included	present	752.51	-0.45	included	present	605.68	-0.54	included	present	1129.55	-0.22	included	present	1542.77	0.03	included	present	446.96	-0.63	included	present	948.26	-0.33	included	present	569.88	-0.56
205652_s_at	included	present	376.49	2.25	included	present	323.22	1.63	included	present	181.12	-0.02	included	present	112.14	-0.82	included	present	367.58	2.15	excluded	absent	193.29	0.12	included	present	452.14	3.13	included	present	594.11	4.78	included	present	276.2	1.09	included	present	586.41	4.69	included	present	318.61	1.58	included	present	179.45	-0.04	included	present	220.83	0.44	included	present	286.59	1.21	included	present	413.22	2.68
205653_at	excluded	absent	39.34	-0.43	excluded	absent	37.77	-0.43	excluded	absent	50.75	-0.42	excluded	absent	5.82	-0.48	included	present	1610.66	1.71	included	present	358.94	0	excluded	absent	2.97	-0.48	excluded	absent	37.65	-0.43	excluded	absent	257.94	-0.13	excluded	absent	24.42	-0.45	excluded	absent	6	-0.48	included	present	4634.82	5.82	excluded	absent	58.04	-0.41	excluded	absent	28.53	-0.45	excluded	absent	5.11	-0.48
205654_at	excluded	absent	13.46	-0.17	excluded	absent	7.64	-0.17	included	present	128.03	-0.15	excluded	absent	40.18	-0.17	excluded	absent	66.33	-0.16	excluded	absent	9.79	-0.17	excluded	absent	9.58	-0.17	excluded	absent	8.93	-0.17	excluded	absent	13.34	-0.17	excluded	absent	41.11	-0.17	excluded	absent	19.57	-0.17	excluded	absent	24.72	-0.17	excluded	absent	10.12	-0.17	excluded	absent	3.05	-0.17	excluded	absent	13.83	-0.17
205655_at	included	present	2337.02	0.35	included	present	1167.35	-0.82	included	present	4183.51	2.21	included	present	3316.19	1.34	included	present	5528.92	3.57	included	present	1944.86	-0.04	included	present	2249.71	0.27	included	present	2418.37	0.44	included	present	2328.75	0.35	included	present	2864.57	0.89	included	present	2038.42	0.05	included	present	1120.42	-0.87	included	present	1895.07	-0.09	included	present	1528.05	-0.46	included	present	1165.63	-0.83
205656_at	included	present	342.6	-0.53	included	present	1535.17	1.41	included	present	111.8	-0.9	included	present	182.77	-0.79	included	present	1106.88	0.71	included	present	294.71	-0.6	included	present	573.41	-0.15	included	present	491.36	-0.29	included	present	745.35	0.13	included	present	1499.07	1.35	included	present	725.94	0.09	included	present	3513.68	4.62	included	present	2088.46	2.31	included	present	2029.2	2.21	included	present	1709.25	1.69
205657_at	included	present	133.46	0.42	excluded	absent	89.9	0	excluded	absent	31.81	-0.57	excluded	absent	45.23	-0.44	excluded	absent	60.09	-0.29	excluded	absent	257.56	1.63	excluded	absent	59.36	-0.3	excluded	absent	60.12	-0.29	excluded	absent	77.1	-0.13	excluded	absent	95.31	0.05	excluded	absent	73.15	-0.17	excluded	absent	23.36	-0.65	excluded	absent	54.36	-0.35	excluded	absent	8.86	-0.79	excluded	absent	95.95	0.06
205658_s_at	excluded	absent	6.61	-1.35	excluded	absent	286.21	3.72	excluded	absent	9.58	-1.3	excluded	absent	28.56	-0.96	excluded	absent	69.09	-0.22	excluded	absent	180.74	1.81	excluded	absent	125.58	0.8	included	present	402.96	5.84	excluded	absent	122.89	0.76	excluded	absent	100.49	0.35	excluded	absent	206.21	2.27	excluded	absent	91.33	0.18	excluded	absent	65.36	-0.29	included	present	121.06	0.72	excluded	absent	80.52	-0.01
205659_at	included	present	459.46	-0.34	included	present	223.38	-0.9	included	present	4785.47	9.81	included	present	656.88	0.12	included	present	368.06	-0.56	included	present	591.48	-0.03	included	present	567.42	-0.09	included	present	658.75	0.13	included	present	1095.34	1.15	included	present	814.62	0.49	included	present	507	-0.23	included	present	505.59	-0.23	included	present	665.09	0.14	included	present	1059.87	1.07	included	present	360.18	-0.57
205660_at	excluded	absent	129.53	0.38	excluded	absent	32.21	-0.36	excluded	absent	63.53	-0.13	excluded	absent	15.05	-0.49	excluded	absent	42.32	-0.29	included	present	504.56	3.22	included	absent	65.25	-0.11	excluded	absent	28.93	-0.39	included	present	53.11	-0.21	excluded	absent	50.01	-0.23	excluded	absent	6.57	-0.56	excluded	absent	91.41	0.09	excluded	absent	4.59	-0.57	excluded	absent	26.19	-0.41	included	present	136.31	0.43
205661_s_at	excluded	absent	94.15	-0.64	included	present	287.32	2.82	included	present	95.74	-0.61	included	present	94.79	-0.63	included	present	209.2	1.42	excluded	absent	188.82	1.06	included	present	347.35	3.89	included	present	382.04	4.52	included	present	287.67	2.83	included	present	301.07	3.07	included	present	327.93	3.55	included	present	129.41	-0.01	excluded	absent	67.85	-1.11	excluded	absent	75.09	-0.98	included	present	436.41	5.49
205662_at	excluded	absent	127.37	0.44	excluded	absent	109.92	0.22	excluded	absent	53.21	-0.48	excluded	absent	74.41	-0.22	excluded	absent	62.45	-0.37	excluded	absent	69.67	-0.28	included	present	147.48	0.69	excluded	absent	80.85	-0.14	excluded	absent	90.11	-0.02	included	present	155.3	0.79	excluded	absent	106.72	0.18	excluded	absent	16.31	-0.94	excluded	absent	4.96	-1.08	excluded	absent	27.9	-0.79	included	present	95.35	0.04
205663_at	included	present	163.87	0.85	excluded	absent	78.26	-0.57	excluded	absent	105.66	-0.12	included	present	160.41	0.79	included	present	165.72	0.88	included	present	326.99	3.55	included	present	484.35	6.17	included	present	355.59	4.03	included	present	382.76	4.48	included	present	795.63	11.33	included	present	466.01	5.86	included	present	442.55	5.47	included	present	118.07	0.09	excluded	absent	57.51	-0.92	included	present	529.94	6.92

205989_s_at	excluded	absent	33.52	-0.5	excluded	absent	2.46	-0.51	excluded	absent	27.94	-0.5	excluded	absent	1.85	-0.51	excluded	absent	9.41
205990_s_at	included	present	188.95	-0.48	included	present	207.93	-0.46	included	absent	58.38	-0.64	included	present	997.88	0.51	included	absent	4.26
205991_s_at	included	present	1455.4	-0.22	included	present	526.76	-0.75	included	present	129.19	-0.98	included	present	274.32	-0.89	included	present	962.98
205992_s_at	included	present	228.97	0.17	included	present	117.15	-0.38	included	absent	59.38	-0.66	included	present	171.44	-0.11	included	present	161.64
205993_s_at	excluded	absent	60.72	-0.54	excluded	absent	35.54	-1.09	excluded	absent	36.97	-1.06	excluded	absent	27.04	-1.27	excluded	absent	10.02
205994_at	included	present	1582.49	0.75	included	present	470.94	-1.01	included	present	843.19	-0.42	included	present	1541.89	0.69	included	present	1310.14
205995_x_at	included	present	199.07	-1.08	included	present	330.93	0.07	included	absent	139.33	-1.6	included	present	303.97	-0.17	included	absent	187.61
205996_s_at	included	present	178.46	-0.1	included	absent	80.47	-1.24	included	present	135.34	-0.6	included	present	176.88	-0.11	included	present	208.3
205997_at	included	present	805.97	0.26	included	present	254.34	-0.45	included	present	121.39	-0.62	included	present	109.28	-0.64	included	absent	135.01
205998_x_at	included	present	145.74	-0.22	included	absent	159.83	-0.22	included	present	548.94	-0.02	included	present	126.14	-0.23	included	present	113.02
205999_x_at	excluded	absent	86.96	-0.23	excluded	absent	38.23	-0.24	included	present	1023.04	0.06	excluded	absent	65.95	-0.23	included	present	158.71
206000_at	excluded	absent	10.91	-0.16	excluded	absent	38.71	-0.14	excluded	absent	1.86	-0.17	excluded	absent	2.28	-0.17	excluded	absent	4.48
206001_at	included	present	1823.03	0.61	included	present	26613.65	14.77	included	present	3781.47	1.73	included	absent	29.02	-0.41	excluded	absent	7.68
206002_at	excluded	absent	57.27	-0.45	included	present	116.56	0.42	excluded	absent	19.73	-1	excluded	absent	35.27	-0.77	excluded	absent	128.01
206003_at	included	present	422.64	-0.64	included	present	1377.32	1.25	included	present	1204.72	0.91	included	present	814.97	0.14	included	present	1701.21
206004_at	excluded	absent	73.31	-0.06	excluded	absent	76.45	-0.05	excluded	absent	38.83	-0.24	excluded	absent	36.07	-0.26	excluded	absent	131.85
206005_s_at	included	present	390.98	-0.26	included	present	1334.08	2.45	included	present	439.28	-0.12	included	present	390.74	-0.26	included	present	728.14
206006_s_at	included	present	206.49	0.58	included	present	303.84	1.9	included	present	139.47	-0.34	included	absent	150.25	-0.19	excluded	absent	173.91
206007_at	excluded	absent	49.59	-0.4	excluded	absent	6.44	-0.84	excluded	absent	131.23	0.45	excluded	absent	58.53	-0.3	excluded	absent	198.62
206008_at	excluded	absent	19.67	-0.27	excluded	absent	78.74	0.77	excluded	absent	4.78	-0.53	excluded	absent	2.8	-0.57	excluded	absent	4.17
206009_at	included	present	375.99	-0.38	included	present	228.08	-0.71	included	present	245.36	-0.67	included	present	516.34	-0.06	included	present	614.47
206010_at	excluded	absent	1.86	-0.2	excluded	absent	4.13	-0.19	included	present	287.51	0.02	included	present	274.14	0.01	excluded	absent	174.06
206011_at	included	present	751.14	-0.09	included	present	299.8	-0.38	included	present	154.38	-0.47	included	absent	94.87	-0.51	included	present	513.53
206012_at	excluded	absent	7.37	-0.24	excluded	absent	50.9	-0.23	excluded	absent	9.14	-0.24	excluded	absent	38.88	-0.23	excluded	absent	110.25
206013_s_at	excluded	absent	78.31	0.42	included	present	219.77	2.83	excluded	absent	154.36	1.72	excluded	absent	67.07	0.23	excluded	absent	331.22
206014_at	excluded	absent	324.3	0.05	included	present	2457.34	5.13	included	present	798.55	1.18	included	present	401.62	0.24	included	present	1062.68
206015_s_at	included	present	990.22	-1.13	included	present	1900.61	2.01	included	present	1029.69	-0.99	included	present	3434.74	7.3	included	present	1056.95
206016_at	included	present	202.01	0.15	excluded	absent	176.97	-0.16	included	present	158.72	-0.38	excluded	absent	108.94	-1	included	present	323.68
206017_at	included	present	281.2	-0.33	included	present	404.52	-0.13	excluded	absent	30.82	-0.74	included	present	319.35	-0.27	included	present	291.38
206018_at	excluded	absent	1.93	-0.71	excluded	absent	57.96	-0.68	excluded	absent	31.91	-0.69	included	present	1876.4	0.2	excluded	absent	30.83
206019_at	excluded	absent	39.22	-1.55	excluded	absent	204.29	0	excluded	absent	173.43	-0.29	excluded	absent	153.12	-0.48	excluded	absent	349.09
206020_at	included	present	338.38	0.83	included	present	151.63	-1.01	included	present	226.37	-0.27	included	present	313.83	0.59	included	present	150.23
206021_at	included	present	154.77	-0.61	included	present	196.36	-0.13	included	present	334.37	1.48	included	present	251.25	0.51	included	absent	137.89
206022_at	included	present	810.35	0.62	included	absent	99.11	-0.88	included	absent	27.18	-1.03	included	absent	2.17	-1.08	included	absent	90.61
206023_at	excluded	absent	40.23	-0.33	included	present	324.96	0.6	included	present	832.14	2.25	excluded	absent	93.57	-0.16	excluded	absent	124.28
206024_at	excluded	absent	2.33	-0.22	excluded	absent	3.44	-0.22	excluded	absent	39.29	-0.19	excluded	absent	4.23	-0.22	excluded	absent	69.28
206025_s_at	included	present	659.83	1.78	excluded	absent	94.71	-0.36	excluded	absent	114.47	-0.28	excluded	absent	126.85	-0.24	excluded	absent	215.78
206026_s_at	included	present	697.18	2.89	included	present	126.38	-0.27	excluded	absent	77.4	-0.55	included	present	241.95	0.37	excluded	absent	186.48
206027_at	excluded	absent	37.99	-0.43	excluded	absent	74.69	-0.1	excluded	absent	72.3	-0.12	excluded	absent	34.59	-0.46	excluded	absent	59.71
206028_s_at	included	absent	261.14	-0.8	included	absent	206.33	-0.92	included	absent	99.26	-1.14	included	absent	106.28	-1.13	included	absent	275.47
206029_at	excluded	absent	16.79	-0.2	excluded	absent	70.72	-0.19	excluded	absent	91.67	-0.19	excluded	absent	13.17	-0.2	excluded	absent	61.19
206030_at	included	present	4861.67	1.65	included	present	132.02	-0.77	included	absent	8.02	-0.83	included	present	100.72	-0.79	included	absent	137.53
206031_s_at	excluded	absent	33.43	0.32	excluded	absent	131.75	4.1	excluded	absent	4.9	-0.78	excluded	absent	44.76	0.75	excluded	absent	11.57
206032_at	excluded	absent	26.6	-0.29	excluded	absent	51.99	-0.28	excluded	absent	62.78	-0.28	excluded	absent	31.88	-0.29	excluded	absent	69.29
206033_s_at	excluded	absent	3.09	-0.29	excluded	absent	2.49	-0.29	excluded	absent	2.05	-0.29	excluded	absent	71.15	-0.25	excluded	absent	10.74
206034_at	included	present	316.07	0.01	included	present	471.92	0.41	excluded	absent	80.74	-0.58	included	present	327.88	0.04	included	present	252.63
206035_at	included	present	417.78	-0.11	included	present	163.23	-0.76	included	present	362.01	-0.25	included	present	746.54	0.74	included	present	327.21
206036_s_at	included	present	164.12	-0.11	included	absent	135.13	-0.31	included	present	258.59	0.53	included	present	417.65	1.6	included	present	237.33
206037_at	excluded	absent	24.42	-0.56	excluded	absent	56.6	0.04	excluded	absent	100.38	0.87	excluded	absent	40.5	-0.26	excluded	absent	115.72
206038_s_at	included	present	1264.02	0.97	included	present	624.34	-1.63	included	present	1148.33	0.5	included	present	1054.59	0.12	included	present	1474.99
206039_at	included	present	434.08	-0.12	included	present	578.09	0.11	included	present	869.82	0.57	included	present	114.25	-0.62	included	present	597.42
206040_s_at	excluded	absent	86.55	0.72	excluded	absent	23.05	-0.65	excluded	absent	13.51	-0.85	excluded	absent	33.53	-0.42	excluded	absent	28.4
206042_x_at	included	present	7198.58	-0.05	included	present	7196.91	-0.05	included	present	5319.01	-0.37	included	present	3657.98	-0.64	included	present	5784.06
206043_s_at	excluded	absent	31.28	-0.61	excluded	absent	110.19	0.1	excluded	absent	3.68	-0.85	excluded	absent	56.95	-0.37	excluded	absent	13.66
206044_s_at	included	present	872.36	-0.56	included	present	700.06	-1.04	included	present	637.47	-1.21	included	present	916.92	-0.44	included	present	835.07
206045_s_at	excluded	absent	75.54	-0.58	included	present	1239.99	1.75	included	present	614.35	0.5	included	present	7115	13.53	excluded	absent	436.96
206046_at	included	present	4903.46	6.33	included	present	657.38	0.14	included	present	210.93	-0.51	included	absent	134.82	-0.62	included	present	258.76
206047_at	excluded	absent	11.9	-1.02	excluded	absent	104.7	0.95	excluded	absent	35.83	-0.51	excluded	absent	21.53	-0.82	excluded	absent	66.03
206048_at	excluded	absent	32.89	-0.56	excluded	absent	14.66	-0.83	excluded	absent	10.65	-0.89	excluded	absent	139.69	1.06	excluded	absent	21.38
206049_at	included	present	274.44	0.13	included	present	367.31	0.48	excluded	absent	4.37	-0.89	excluded	absent	84.66	-0.59	excluded	absent	70.16
206050_s_at	included	present	552.46	0.18	included	present	534.58	0.09	included	present	300.41	-1.02	included	present	268.82	-1.17	included	present	261.48
206051_at	included	present	2957.96	2.66	included	present	16084.8	17.52	included	present	4143.24	4.01	included	present	2596.6	2.26	included	present	12655.88
206052_s_at	included	present	374.68	-0.64	included	present	696.07	0.69	included	present	766.58	0.99	included	present	1056.57	2.19	included	present	626.47
206053_at	included	present	146.45	0.12	included	present	184.43	0.79	included	present	345.71	3.63	included	present	131.23	-0.15	included	present	181.77
206054_at	included	present	80.51	-0.18	excluded	absent	11.56	-0.19	included	present	586.61	-0.09	excluded	absent	67.71	-0.18	excluded	absent	42.86
206055_s_at	included	present	1919.19	-1.31	included	present	2922.88	-0.57	included	present	4737.64	0.78	included	present	3513.78	-0.13	included	present	2032.6

206349_at	included	present	840.64	0.34	excluded	absent	3.05	-0.76	excluded	absent	1.86	-0.76	excluded	absent	16.2	-0.74	excluded	absent	6.6	-0.75	excluded	absent	55.41	-0.69	included	present	341	-0.31	excluded	absent	28.95	-0.72	included	present	301.04	-0.37	excluded	absent	40.1	-0.71	excluded	absent	48.95	-0.7	excluded	absent	3.36	-0.76	included	present	740.78	0.21	included	present	70.23	-0.67	included	present	116.94	-0.61
206350_at	excluded	absent	10.06	-0.15	excluded	absent	72.34	-0.15	included	present	2328.16	0.02	included	present	248.53	-0.14	excluded	absent	53.36	-0.15	excluded	absent	35.68	-0.15	excluded	absent	31.57	-0.15	excluded	absent	47.59	-0.15	excluded	absent	58.32	-0.15	excluded	absent	12.61	-0.15	excluded	absent	3.48	-0.15	included	present	160.99	-0.14	excluded	absent	84.6	-0.15	excluded	absent	24.21	-0.15	excluded	absent	16.57	-0.15
206351_s_at	included	present	116.55	-0.33	included	present	100.24	-0.66	excluded	absent	72.11	-1.23	excluded	absent	73.8	-1.2	included	present	279.64	3	included	present	307.81	3.57	included	present	202.21	1.42	included	present	322.7	3.87	included	present	262.49	2.65	included	present	189.01	1.15	included	present	239.66	2.18	included	present	172.29	0.81	excluded	absent	78.82	-1.1	included	present	93.4	-0.8	included	present	149.15	0.34
206352_s_at	included	present	1378.36	2.72	included	present	1610.92	3.75	included	present	452.28	-1.39	included	present	427.91	-1.5	included	present	2027.8	5.6	included	present	1389	2.76	included	present	1687.66	4.09	included	present	2040.47	5.66	included	present	731.67	-0.15	included	present	2378.59	7.16	included	present	1938.91	5.21	included	present	1102.92	1.5	included	present	622.13	-0.64	included	present	563.08	-0.9	included	present	2052.9	5.71
206353_at	excluded	absent	19.75	-0.47	excluded	absent	76.33	-0.23	excluded	absent	53.27	-0.33	excluded	absent	17.97	-0.48	excluded	absent	141.01	0.06	included	present	1078.15	4.16	excluded	absent	68.71	-0.26	excluded	absent	95.07	-0.14	excluded	absent	88.24	-0.17	included	present	1532.93	6.16	excluded	absent	59.55	-0.3	excluded	absent	98.6	-0.13	excluded	absent	12.93	-0.5	excluded	absent	3.32	-0.55	excluded	absent	32.43	-0.42
206354_at	excluded	absent	15.76	-0.19	excluded	absent	54.98	-0.14	excluded	absent	37.65	-0.16	excluded	absent	2.65	-0.2	excluded	absent	6.17	-0.2	excluded	absent	91.69	-0.09	excluded	absent	9.24	-0.2	excluded	absent	24.04	-0.18	excluded	absent	12.72	-0.19	excluded	absent	10.24	-0.19	excluded	absent	31.41	-0.17	excluded	absent	56.21	-0.14	excluded	absent	31.58	-0.17	excluded	absent	86.45	-0.1	excluded	absent	2.64	-0.2
206355_at	included	present	183.77	-0.54	included	absent	49.36	-0.73	included	present	168.02	-0.56	included	present	262.45	-0.42	included	absent	162.73	-0.57	included	present	250.5	-0.44	included	present	758.23	0.29	included	absent	40.98	-0.75	included	present	379.43	-0.25	included	present	123.85	-0.63	included	present	78.84	-0.69	included	absent	124.25	-0.62	included	present	239.63	-0.46	included	present	276.68	-0.4	included	present	537.32	-0.03
206356_s_at	included	present	268.97	-0.38	included	absent	3.04	-0.69	included	present	179.89	-0.48	included	absent	50.3	-0.63	included	present	149.09	-0.52	included	absent	189.41	-0.47	included	present	785.76	0.22	included	absent	6.12	-0.69	included	present	274.43	-0.37	included	absent	120.46	-0.55	included	absent	83.7	-0.6	included	absent	160.27	-0.51	included	present	317.76	-0.32	included	present	109.12	-0.57	included	present	491.52	-0.12
206357_at	excluded	absent	150.82	0.32	included	present	165.74	0.48	included	present	151.36	0.33	included	present	154.27	0.36	excluded	absent	138.77	0.2	included	present	333.7	2.27	included	present	104	-0.17	included	present	308.99	2.01	included	present	155.36	0.37	included	present	144.84	0.26	included	present	175.81	0.59	included	present	241.33	1.29	excluded	absent	6.77	-1.21	excluded	absent	17.76	-1.09	included	present	79.16	-0.44
206358_at	excluded	absent	20.45	-0.22	excluded	absent	78.66	-0.21	excluded	absent	4.49	-0.22	excluded	absent	47.92	-0.21	excluded	absent	138.35	-0.2	excluded	absent	16.74	-0.22	excluded	absent	16.65	-0.22	excluded	absent	79.57	-0.21	excluded	absent	103.25	-0.21	excluded	absent	71.62	-0.21	excluded	absent	38.6	-0.21	excluded	absent	78.86	-0.21	excluded	absent	74	-0.21	excluded	absent	49.85	-0.21	excluded	absent	43.5	-0.21
206359_at	excluded	absent	150.83	0.03	excluded	absent	7.72	-1.03	excluded	absent	6.41	-1.03	included	present	190.48	0.32	excluded	absent	51.12	-0.71	included	present	317.34	1.26	excluded	absent	26.72	-0.89	excluded	absent	82.52	-0.47	excluded	absent	138.2	-0.06	excluded	absent	159.77	0.1	excluded	absent	95.26	-0.38	excluded	absent	117.44	-0.22	excluded	absent	62.63	-0.62	excluded	absent	2.17	-1.07	included	present	334.25	1.38
206360_s_at	included	present	205.78	0.18	excluded	absent	80.03	-0.47	included	present	125.34	-0.23	included	present	302.09	0.68	excluded	absent	82.37	-0.45	included	present	450.81	1.45	included	present	93.49	-0.4	included	present	102.34	-0.35	included	present	194.44	0.12	included	present	238.74	0.35	included	present	130.01	-0.21	included	present	184.4	0.07	included	present	135.49	-0.18	excluded	absent	51.5	-0.61	included	present	269.92	0.51
206361_at	excluded	absent	114.53	-0.2	excluded	absent	105.71	-0.31	excluded	absent	123.98	-0.08	excluded	absent	70.19	-0.75	excluded	absent	75.35	-0.68	included	present	202.29	0.89	excluded	absent	34.24	-1.19	excluded	absent	72.21	-0.72	included	present	138.25	0.1	excluded	absent	167.54	0.46	excluded	absent	138.87	0.11	included	present	153.59	0.29	excluded	absent	100.5	-0.37	excluded	absent	78.23	-0.65	excluded	absent	59.15	-0.88
206362_x_at	excluded	absent	30.38	-0.37	excluded	absent	83.78	0.8	excluded	absent	33.16	-0.31	excluded	absent	16.62	-0.67	excluded	absent	16.09	-0.68	excluded	absent	25.57	-0.47	excluded	absent	58.5	0.25	excluded	absent	96.6	1.08	excluded	absent	37.1	-0.22	excluded	absent	43.89	-0.07	excluded	absent	22.91	-0.53	excluded	absent	22.16	-0.55	excluded	absent	20.31	-0.59	excluded	absent	34.48	-0.28	excluded	absent	11.61	-0.78
206363_at	included	present	1388.09	0.05	included	present	310.06	-0.63	included	present	221.26	-0.68	included	present	452.06	-0.54	included	present	966.89	-0.22	included	present	1084.18	-0.14	included	present	330.19	-0.61	included	present	153.32	-0.72	included	present	644.05	-0.42	included	present	509.18	-0.5	included	present	337.37	-0.61	included	present	1705.53	0.24	included	present	778.78	-0.33	included	present	1812.26	0.31	included	present	241.65	-0.67
206364_at	excluded	absent	0.82	-0.95	included	present	103.85	1.69	included	present	497	11.77	included	present	374.56	8.63	included	present	353.72	8.1	excluded	absent	80.47	1.09	included	present	116.7	2.02	included	present	366.89	8.44	excluded	absent	56.34	0.47	excluded	absent	3.24	-0.89	included	present	698.82	16.95	included	present	104.69	1.71	included	present	197.55	4.09	included	present	121.08	2.13	included	present	302.04	6.77
206365_at	excluded	absent	2.89	-1.18	included	present	159.33	3.38	excluded	absent	39.97	-0.1	included	present	129.15	2.5	excluded	absent	2.94	-1.18	excluded	absent	53.21	0.29	excluded	absent	1.36	-1.22	included	present	77.26	0.99	excluded	absent	6.48	-1.07	excluded	absent	1.42	-1.22	excluded	absent	1.33	-1.22	included	present	237.56	5.66	included	present	190.07	4.27	excluded	absent	21.42	-0.64	excluded	absent	14.67	-0.84
206366_x_at	excluded	absent	43.33	-0.33	included	present	705.87	3.63	excluded	absent	68.3	-0.18	included	present	495.6	2.38	excluded	absent	129.46	0.18	excluded	absent	116.64	0.11	excluded	absent	65.97	-0.2	included	present	273.85	1.05	excluded	absent	125.94	0.16	excluded	absent	2.63	-0.58	excluded	absent	67.89	-0.18	included	present	391.79	1.75	included	present	539.73	2.64	excluded	absent	2.72	-0.58	included	present	742.26	3.85
206367_at	excluded	absent	2.14	-0.5	excluded	absent	19.26	-0.32	excluded	absent	32.27	-0.17	excluded	absent	5.03	-0.47	excluded	absent	50.98	0.03	excluded	absent	130.08	0.89	excluded	absent	4.36	-0.48	excluded	absent	8.45	-0.43	excluded	absent	3.8	-0.48	excluded	absent	37.78	-0.11	excluded	absent	53.83	0.06	excluded	absent	4.28	-0.48	excluded	absent	5.66	-0.46	excluded	absent	2.6	-0.5	excluded	absent	25.91	-0.24
206368_at	excluded	absent	78.16	-0.5	excluded	absent	74.26	-0.51	excluded	absent	113.07	-0.45	included	present	225.23	-0.28	excluded	absent	217.3	-0.29	excluded	absent	87.07	-0.49	included	present	152.61	-0.39	included	present	359.71	-0.08	included	present	203.37	-0.31	included	present	320.88	-0.14	excluded	absent	202.79	-0.31	included	present	279.76	-0.2	excluded	absent	76.21	-0.5	excluded	absent	3.47	-0.61	included	present	312.66	-0.15
206369_s_at	included	present	160.49	0.05	excluded	absent	44.45	-0.56	excluded	absent	75.72	-0.4	excluded	absent	35.42	-0.6	included	present	231.47	0.41	included	present	181.8	0.16	excluded	absent	35.31	-0.61	excluded	absent	5.04	-0.76	included	present	157.06	0.03	excluded	absent	52.14	-0.52	included	present	138.07	-0.07	excluded	absent	95.32	-0.29	excluded	absent	106.92	-0.23	excluded	absent	57.36	-0.49	included	present	101.85	-0.26
206370_at	included	present	155.63	1.26	excluded	absent	76.95	-0.07	excluded	absent	66.77	-0.24	excluded	absent	33.26	-0.81	included	present	176.05	1.61	excluded	absent	125.03	0.75	excluded	absent	57.85	-0.39	excluded	absent	20.87	-1.02	included	present	116	0.59	excluded	absent	43.65	-0.63	excluded	absent	92.5	0.19	included	present	107.9	0.46	excluded	absent	47.01	-0.58	excluded	absent	27.76	-0.9	included	present	72.74	-0.14
206371_at	excluded	absent	3.03	-0.78	excluded	absent	64.38	0.66	excluded	absent	22.89	-0.32	excluded	absent	26.74	-0.23	excluded	absent	5.04	-0.74	excluded	absent	6.71	-0.7	excluded	absent	51.39	0.36	excluded	absent	2.32	-0.8	excluded	absent	37.53	0.03	excluded	absent	3.43	-0.77	excluded	absent	8.61	-0.65	included	present	120.84	1.99	excluded	absent	9.23	-0.64	excluded	absent	3.29	-0.78	excluded	absent	19.6	-0.39
206372_at	excluded	absent	21.67	-0.15	excluded	absent	51.54	-0.13	excluded	absent	29.86	-0.14	excluded	absent	2.46	-0.17	excluded	absent	124.97	-0.07	included	present	533.08	0.24	excluded	absent	2.81	-0.17	excluded	absent	26.37	-0.15	excluded	absent	5.62	-0.16	included	present	1326.66	0.85	excluded	absent	53.61	-0.13	excluded	absent	76.21	-0.11	excluded	absent	28.78	-0.15	excluded	absent	12.78	-0.16	excluded	absent	5.87	-0.16
206373_at	excluded	absent	38.33	-0.47	excluded	absent	126.86	-0.4	excluded	absent	2.3	-0.5	included	present	75.11	-0.44	excluded	absent	90.49	-0.43	excluded	absent	158.64	-0.38	excluded	absent	28.33	-0.48	excluded	absent	47.4	-0.46	excluded	absent	39.85	-0.47	excluded	absent	84.07	-0.43	included	present	386.01	-0.2	excluded	absent	119.32	-0.41	excluded	absent	2.09	-0.5	excluded	absent	2.79	-0.49	excluded	absent	8.01	-0.49
206374_at	included	absent	152.52	-0.61	included	present	214.18	-0.18	included	present	186.52	-0.37	included	present	150.26	-0.63	included	present	200.32	-0.28	included	absent	167.49	-0.51	included	present	680.99	3.08	included	absent	131.67	-0.76	included	present	256.42	0.11	included	present	437.51	1.38	included	present	434.77	1.36	included	present	459.83	1.53	included	present	204.53	-0.25	included	present	172.6	-0.47	included	present	767.19	3.68
206375_s_at	excluded	absent	2.96	-0.33	excluded	absent	33.42	-0.31	excluded	absent	44.97	-0.3	excluded	absent	2.97	-0.33	excluded	absent	3.76	-0.33	included	present	492.88	0.06	excluded	absent	7.52	-0.33	excluded	absent	53.12	-0.29	excluded	absent	16.56	-0.32	included	present	344.25	-0.06	excluded	absent	17.62	-0.32	excluded	absent	13.38	-0.33	excluded	absent	7.57	-0.33	excluded	absent	1.13	-0.34	excluded	absent	6.07	-0.33
206376_at	included	present	1791.8	2.74	included	present	1424.17	2.01	included	present	2767.94	4.68	excluded	absent	33.63	-0.75	included	present	1142.22	1.45	excluded	absent	4.22	-0.81	included	present	1412.68	1.99	included	present	297.08	-0.23	included	present	911.18	0.99	included	present	904.54	0.98	excluded	absent	3.63	-0.81	excluded	absent	27.06	-0.76	included	present	984.24	1.14	included	present	1463.76	2.09	included	present	961.85	1.09
206377_at	included	present	157.08	-0.2	excluded	absent	15.96	-0.42	excluded	absent	20.34	-0.41	included	present	72.9	-0.33	excluded	absent	70.37	-0.33	excluded	absent	104.07	-0.28	excluded	absent	1.36	-0.44	excluded	absent	1.24	-0.44	included	present	77.13	-0.32	excluded	absent	30.83	-0.39	included	present	145.51	-0.22	excluded	absent	53.63	-0.36	included	present	105.75	-0.28	excluded	absent	41.55	-0.38	excluded	absent	0.96	-0.44
206378_at	excluded	absent	3.59	-0.22	excluded	absent	29.36	-0.21	excluded	absent	4.56	-0.22	excluded	absent	8.01	-0.22	excluded	absent	73.58	-0.2	excluded	absent	6.2	-0.22	excluded	absent	25.62	-0.21	excluded	absent	2.86	-0.22	excluded	absent	10.61	-0.22	excluded	absent	3.92	-0.22	excluded	absent	7.88	-0.22	excluded	absent	2.22	-0.22	excluded	absent	31.39	-0.21	excluded	absent	3.39	-0.22	excluded	absent	0.78	-0.22
206379_at	included	present	381.07	-0.67	included	present	416.1	-0.46	included	present	172.91	-1.92	included	present	526.71	0.21	included	present	787.81	1.78	included	present	676.66	1.11	included	present	589.68	0.59	included	present	1176.08	4.11	included	present	822.41	1.98	included	present	1094.76	3.62	included	present	687.99	1.18	included	present	273.21	-1.32	included	present	231.59	-1.57	included	present	200.56	-1.75	included	present	771.06	1.68
206380_s_at	excluded	absent	4.37	-0.54	excluded	absent	72.81	0.54	excluded	absent	6.84	-0.5	excluded	absent	2.17	-0.57	excluded	absent	60.66	0.34	excluded	absent	133.38	1.49	excluded	absent	9.02	-0.47	excluded	absent	5.83	-0.52	excluded	absent	96.95	0.91	excluded	absent	59.48	0.33	excluded	absent	51.81	0.21	excluded	absent	128.68	1.41	excluded	absent	28.92	-0.15	excluded	absent	7.71	-0.49	excluded	absent	61.23	0.35
206381_at	included	present	239.95	-0.55	included	present	1090.88	0.03	excluded	absent	94.55	-0.65	included	present	3488.93	1.66	included	present	433.03	-0.42	excluded	absent	38.34	-0.69	included	present	999	-0.04	included	present	127.27	-0.63	included	present	825.6	-0.15	included	present	905.94	-0.1	included	present	189.62	-0.59	included	present	386.27	-0.45	included	present	884.63	-0.11	included	present	535.88	-0.35	included	present	400.1	-0.44
206382_s_at	excluded	absent	112.51	-0.21	excluded	absent	104.71	-0.24	included	present	188.46	0.14	excluded	absent	133.5	-0.11	excluded	absent	135.45	-0.1	excluded	absent	25.26	-0.61	excluded	absent	14.43	-0.66	included	present	430.32	1.25	excluded	absent	53.3	-0.48	included	present	583.26	1.96	excluded	absent	80.55	-0.36	included	present	88.35	-0.32	included	present	184.46	0.12	included	present	125.48	-0.15	excluded	absent	58.3	-0.46
[illegible]																																																												

206708_at	included	present	1121.83	1.93	included	present	466.93	-0.14	included	present	772.13	0.82
206709_x_at	excluded	absent	17.39	-2.22	included	present	192.59	-0.42	excluded	absent	151.29	-0.85
206710_s_at	included	present	1243.34	2.44	included	absent	4.59	-1.32	included	present	326.49	-0.34
206711_at	excluded	absent	96.83	-0.34	excluded	absent	101.14	-0.32	excluded	absent	6.56	-0.87
206712_at	excluded	absent	12.05	-0.74	excluded	absent	18.63	-0.64	excluded	absent	5.9	-0.83
206713_at	included	present	173.93	0.59	included	present	274.1	1.43	included	present	2242.62	17.94
206714_at	excluded	absent	1.82	-0.28	included	present	116.23	-0.06	excluded	absent	38.46	-0.21
206715_at	included	present	270.61	0.35	included	present	115.98	-0.18	excluded	absent	31.54	-0.47
206716_at	excluded	absent	6.28	-0.16	excluded	absent	2.7	-0.16	excluded	absent	11.83	-0.15
206717_at	excluded	absent	3.95	-0.64	excluded	absent	2.34	-0.65	excluded	absent	8.94	-0.58
206718_at	excluded	absent	75.36	0.39	included	present	189.89	2.88	excluded	absent	124.14	1.45
206719_at	excluded	absent	6.03	-0.63	excluded	absent	4.41	-0.65	excluded	absent	10.42	-0.58
206720_at	included	present	160.59	0.49	included	present	244.29	1.68	included	present	165.81	0.56
206721_at	included	present	313.88	-0.37	included	present	882.63	0.46	included	present	221.4	-0.51
206722_s_at	excluded	absent	14.9	-0.32	excluded	absent	16.45	-0.27	excluded	absent	39.01	0.45
206723_s_at	excluded	absent	136.33	-0.77	included	present	674.13	6	included	present	215.29	0.22
206724_at	excluded	absent	4.99	-0.88	excluded	absent	62.98	0.61	excluded	absent	89.92	1.3
206725_x_at	excluded	absent	104.86	1.33	excluded	absent	39.35	-0.12	excluded	absent	53.1	0.18
206726_at	included	present	376.02	0.02	included	present	149.63	-0.77	included	absent	95.99	-0.96
206727_at	excluded	absent	2.16	-0.17	excluded	absent	3.93	-0.16	excluded	absent	69.16	-0.14
206728_at	excluded	absent	27.5	-0.07	excluded	absent	53.58	0.63	excluded	absent	63.66	0.91
206729_at	excluded	absent	90.82	0.4	excluded	absent	44.01	-0.49	excluded	absent	38.15	-0.61
206730_at	included	present	152.71	-0.66	included	present	113.69	-0.68	included	absent	127.73	-0.67
206731_at	excluded	absent	82.46	-0.51	excluded	absent	2.96	-0.75	excluded	absent	53.28	-0.6
206732_at	included	absent	2.02	-0.89	included	present	634.05	0.56	included	absent	2.42	-0.89
206733_at	excluded	absent	2.71	-0.24	excluded	absent	20.01	-0.19	excluded	absent	4.55	-0.24
206734_at	included	present	442.29	0.04	included	present	688.06	1.7	included	present	377.58	-0.4
206735_at	excluded	absent	45.03	0.11	excluded	absent	6.14	-0.87	excluded	absent	8.07	-0.82
206736_x_at	excluded	absent	11.66	-0.12	excluded	absent	2.57	-0.63	excluded	absent	6.58	-0.41
206737_at	excluded	absent	66.47	-0.53	excluded	absent	121.22	-0.35	excluded	absent	79.57	-0.48
206738_at	excluded	absent	69.95	-0.16	included	present	164.17	-0.11	included	present	106.77	-0.14
206739_at	excluded	absent	105.05	1.5	excluded	absent	47.36	0.03	excluded	absent	86.84	1.03
206740_x_at	excluded	absent	4.49	-0.25	excluded	absent	2.69	-0.26	excluded	absent	1.84	-0.26
206741_at	included	present	113.9	0.1	excluded	absent	5.01	-0.45	excluded	absent	70.33	-0.12

207067_s_at	excluded	absent	55.24	-0.26	excluded	absent	67	-0.21	excluded	absent	1.89	-0.5	excluded	absent	5.11	-0.49	excluded	absent	5.32
207068_at	excluded	absent	85.68	-0.32	excluded	absent	77.74	-0.45	included	present	154.19	0.78	included	present	128.34	0.37	excluded	absent	102.26
207069_s_at	included	present	277.32	-0.27	included	absent	82.96	-1.05	included	absent	70.14	-1.1	included	present	155.17	-0.76	included	absent	126.48
207070_at	excluded	absent	55.16	-0.01	excluded	absent	27.5	-0.47	excluded	absent	9.5	-0.77	excluded	absent	6.85	-0.82	excluded	absent	6.66
207071_s_at	included	present	495.75	-0.37	included	present	220.8	-0.71	included	present	439.61	-0.44	included	present	244.59	-0.68	included	absent	188.12
207072_at	excluded	absent	63.57	-0.17	excluded	absent	3.41	-0.62	excluded	absent	4.43	-0.61	excluded	absent	1.59	-0.63	included	present	150.39
207073_at	included	present	173.14	-0.61	included	present	151.17	-0.65	included	present	465.47	-0.02	included	absent	34.1	-0.89	included	present	395.41
207074_s_at	excluded	absent	5.97	-0.58	included	present	300.48	2.69	excluded	absent	2.88	-0.61	included	present	249.98	2.13	included	present	695.62
207075_at	included	present	173.99	0.25	excluded	absent	55.39	-1.32	excluded	absent	4.02	-2	excluded	absent	37.74	-1.56	excluded	absent	6.05
207076_s_at	included	present	361.5	-0.42	included	present	779.56	-0.36	included	present	341.6	-0.42	included	present	285.58	-0.43	included	absent	168.36
207077_at	excluded	absent	18.96	-0.34	excluded	absent	60.56	0.92	excluded	absent	4.19	-0.78	excluded	absent	4.11	-0.79	excluded	absent	19.6
207078_at	included	present	116.32	-1.06	included	present	203.55	0.12	included	absent	118.58	-1.03	included	present	226.89	0.44	included	absent	160.25
207079_s_at	included	present	1235.62	3.24	included	present	747.35	0.53	included	present	1391.09	4.11	included	present	1399.75	4.16	included	present	1230.12
207080_s_at	excluded	absent	8.5	-0.38	excluded	absent	3.83	-0.55	excluded	absent	35.87	0.57	excluded	absent	9.25	-0.36	excluded	absent	72.95
207081_s_at	included	present	840.86	0.05	included	present	1300.34	0.85	included	present	519.1	-0.51	included	present	631.92	-0.31	included	present	764.41
207082_at	included	present	224.86	1.41	excluded	absent	120.87	0.09	excluded	absent	107.15	-0.08	excluded	absent	72.76	-0.51	included	present	159.18
207083_s_at	included	present	425.08	3.53	excluded	absent	213.33	0.61	excluded	absent	89.88	-1.08	excluded	absent	142.53	-0.36	excluded	absent	138.8
207084_at	included	present	676.66	0.37	included	present	1659.25	2.04	excluded	absent	14.43	-0.76	included	present	919.95	0.78	included	present	671.86
207085_x_at	included	present	231.58	3.08	excluded	absent	19.29	-0.91	excluded	absent	73.23	0.1	excluded	absent	4.92	-1.18	excluded	absent	80.48
207086_x_at	excluded	absent	4.01	-0.22	included	present	332.51	0.09	excluded	absent	4.18	-0.22	excluded	absent	2.31	-0.23	excluded	absent	9.95
207087_x_at	included	present	118.94	0.24	excluded	absent	15.14	-0.71	excluded	absent	93.35	0.01	included	present	367.9	2.54	included	present	398.98
207088_s_at	included	present	266.43	0.03	included	present	282.24	0.16	included	present	157.39	-0.88	included	absent	105.38	-1.31	included	present	307.25
207089_at	excluded	absent	2.47	-0.2	excluded	absent	3.23	-0.2	excluded	absent	3.01	-0.2	excluded	absent	24.04	-0.19	excluded	absent	30.53
207090_x_at	excluded	absent	460.56	0.54	included	present	647.26	1.86	included	present	1058.96	4.78	included	present	1118.34	5.2	excluded	absent	444.35
207091_at	excluded	absent	167.39	0.87	excluded	absent	20.15	-0.67	excluded	absent	14.3	-0.73	excluded	absent	16.49	-0.71	excluded	absent	13.96
207092_at	included	present	138.63	-0.13	included	present	210.11	-0.04	included	present	253.67	0.01	excluded	absent	34.73	-0.25	excluded	absent	264.63
207093_s_at	excluded	absent	55.8	-0.7	excluded	absent	3.74	-0.75	excluded	absent	72.89	-0.69	excluded	absent	1.22	-0.75	excluded	absent	8.97
207094_at	excluded	absent	2.57	-0.39	excluded	absent	2.9	-0.38	excluded	absent	72.5	1.01	excluded	absent	2.79	-0.38	excluded	absent	23.78
207095_at	excluded	absent	39.68	-0.19	excluded	absent	21.09	-0.23	excluded	absent	4.69	-0.26	excluded	absent	5.9	-0.26	excluded	absent	153.54
207096_at	excluded	absent	7.46	-0.16	excluded	absent	3.66	-0.16	excluded	absent	93.31	-0.14	excluded	absent	4.61	-0.16	excluded	absent	61.49
207097_s_at	excluded	absent	3.69	-0.16	excluded	absent	4.08	-0.16	excluded	absent	2.47	-0.17	excluded	absent	3.12	-0.17	excluded	absent	46.59
207098_s_at	included	present	198.78	-0.98	included	present	502.34	1.81	included	present	244.66	-0.56	included	present	240.59	-0.59	included	present	289.72
207099_s_at	included	present	430.37	1.06	included	absent	121.27	-1.58	included	present	317.98	0.1	included	present	422.58	0.99	included	present	303.45
207100_s_at	included	present	305.84	0.67	excluded	absent	19.1	-1.15	excluded	absent	3.41	-1.25	excluded	absent	123	-0.49	excluded	absent	133.29
207101_at	included	present	424.8	0.27	excluded	absent	11.94	-0.97	excluded	absent	114.55	-0.66	excluded	absent	172.66	-0.48	excluded	absent	230.99
207102_at	excluded	absent	4.12	-0.16	excluded	absent	1.73	-0.16	excluded	absent	7.95	-0.16	excluded	absent	39.03	-0.13	excluded	absent	131.54
207103_at	included	present	173.83	0	included	present	420.52	1.6	included	absent	1.14	-1.13	included	present	177.84	0.02	included	absent	88.48
207104_x_at	excluded	absent	146.35	0.32	excluded	absent	128.01	0.15	excluded	absent	80.23	-0.27	excluded	absent	34.63	-0.67	included	present	283.89
207105_s_at	excluded	absent	109	1.04	excluded	absent	70.41	0.35	excluded	absent	150.49	1.79	excluded	absent	186.82	2.45	excluded	absent	175.93
207106_s_at	excluded	absent	2.25	-0.9	excluded	absent	44.7	0.38	excluded	absent	47	0.44	excluded	absent	93.96	1.85	excluded	absent	5.69
207107_at	excluded	absent	27.85	0.11	excluded	absent	1.42	-0.7	excluded	absent	16.62	-0.23	excluded	absent	1.01	-0.71	excluded	absent	3.32
207108_s_at	included	present	1158.01	1.86	included	present	1111.93	1.68	included	present	1155.62	1.85	included	present	1499.4	3.19	included	present	1407.98

[illegible]

207430_s_at	excluded	absent	2.97	-0.22	excluded	absent	3.37	-0.22	excluded	absent	1.97
207431_s_at	included	present	1026.55	0.18	included	present	409.73	-0.34	included	present	1150.74
207432_at	excluded	absent	2.41	-0.8	excluded	absent	4.04	-0.71	excluded	absent	64.76
207433_at	excluded	absent	2.91	-0.7	excluded	absent	1.46	-0.74	excluded	absent	36.9
207434_s_at	excluded	absent	5.8	-0.2	excluded	absent	6.31	-0.2	included	present	195.73
207435_s_at	included	present	2538.85	-1.25	included	present	3153.11	-0.72	included	present	3519.91
207436_x_at	included	present	687.48	0.32	included	absent	547.17	-0.05	included	absent	343.51
207437_at	included	present	129.2	-0.29	included	present	127.19	-0.31	included	absent	39.29
207438_s_at	included	present	748.97	-0.39	included	present	655.15	-0.65	included	present	279.42
207439_s_at	excluded	absent	4.46	-1.17	excluded	absent	6.73	-1.11	excluded	absent	38.54
207440_at	included	present	172.85	0.78	excluded	absent	44.73	-1.07	excluded	absent	60.87
207441_at	excluded	absent	52.24	-0.15	excluded	absent	37.29	-0.15	excluded	absent	2.79
207442_at	excluded	absent	14.99	-0.55	excluded	absent	48.23	-0.24	excluded	absent	8.03
207443_at	excluded	absent	3.71	-0.73	excluded	absent	61.83	-0.68	excluded	absent	112.44
207444_at	excluded	absent	32.87	-0.09	excluded	absent	7.18	-0.39	excluded	absent	7.25
207445_s_at	excluded	absent	2.08	-0.76	excluded	absent	41.46	0.35	excluded	absent	8.82
207446_at	included	present	289.57	1.03	included	present	106.16	-0.29	excluded	absent	80.7
207447_s_at	included	present	62.99	-0.09	excluded	absent	40.92	-0.46	included	present	567.09
207448_at	excluded	absent	48.26	-0.33	excluded	absent	47.54	-0.34	excluded	absent	10.71
207449_s_at	excluded	absent	30.2	-0.86	excluded	absent	18.99	-1.07	included	present	144.73
207450_s_at	excluded	absent	8.86	-1	excluded	absent	23.06	-0.87	excluded	absent	85.02
207451_at	excluded	absent	49.4	-0.75	excluded	absent	120.87	0.57	excluded	absent	41.45
207452_s_at	excluded	absent	81.85	0.76	excluded	absent	38.7	-0.1	excluded	absent	16.52
207453_s_at	included	present	471.1	0.06	included	present	1020.28	1.16	included	present	558.52
207454_at	excluded	absent	131.21	3.67	excluded	absent	83.7	1.99	excluded	absent	2.96
207455_at	included	present	2316.71	1.22	included	absent	30.57	-1.22	included	present	223.34
207456_at	excluded	absent	3.4	-0.22	excluded	absent	29.9	-0.19	excluded	absent	42.88
207457_s_at	excluded	absent	12.08	-0.57	excluded	absent	10.5	-0.6	excluded	absent	98.75
207458_at	excluded	absent	54.46	-0.67	excluded	absent	66.7	-0.4	excluded	absent	49.03
207459_x_at	excluded	absent	38.59	-0.32	excluded	absent	78.16	0.53	excluded	absent	17.52
207460_at	excluded	absent	81.19	-0.25	excluded	absent	185.22	1.59	excluded	absent	8.9
207461_at	excluded	absent	39.47	0.97	excluded	absent	16.33	-0.04	excluded	absent	10.12
207462_at	excluded	absent	79.62	0.56	excluded	absent	6.04	-0.82	excluded	absent	49.45
207463_x_at	included	present	383.58	0.41	included	present	368.04	0.38	included	present	177.98
207464_at	included	present	168.3	-0.64	included	present	151.59	-0.72	included	present	178.61
207465_at	included	absent	107.04	-0.38	included	absent	48.52	-1.13	included	present	166.34
207466_at	excluded	absent	15.6	-0.54	included	present	133.14	0.8	included	present	300.71
207467_x_at	included	present	1717.51	-0.24	included	present	470.98	-1.14	included	present	709.8
207468_s_at	excluded	absent	49.43	0.39	excluded	absent	7.56	-0.73	excluded	absent	13.05
207469_s_at	included	present	262.81	-0.68	included	absent	155.89	-1.23	included	present	223.34
207470_at	excluded	absent	140	0.62	excluded	absent	40.3	-0.49	excluded	absent	11.52
207471_at	included	present	54.01	-0.19	excluded	absent	80.11	0.33	excluded	absent	92.39
207472_at	excluded	absent	42.57	0.14	excluded	absent	35.67	-0.04	excluded	absent	9.93
207473_at	excluded	absent	8.57	-0.17	excluded	absent	10.73	-0.17	excluded	absent	43.45
207474_at	included	present	189.54	0.27	included	present	141.97	-0.18	included	present	91.17
207475_at	excluded	absent	51.59	-0.21	excluded	absent	15.99	-0.33	excluded	absent	6.26
207476_at	excluded	absent	9.62	-1.4	excluded	absent	8.41	-1.42	included	present	89.33
207477_at	included	present	149.21	-0.93	included	absent	159.66	-0.8	included	present	151.22
207478_at	included	present	199.55	-0.58	included	absent	152.92	-0.95	included	present	205.84
207479_at	excluded	absent	14.05	-1.32	excluded	absent	47.1	-0.62	excluded	absent	37.72
207480_s_at	included	present	376.56	-0.8	included	present	2807.39	1.27	included	present	2082.96
207481_at	excluded	absent	47.19	0.46	excluded	absent	61.69	0.88	excluded	absent	97.3
207482_at	excluded	absent	72.5	-0.33	excluded	absent	28.48	-0.46	excluded	absent	92.64
207483_s_at	included	present	1094.86	-0.44	included	present	1939.34	2.1	included	present	1854.16

207790_at	included	present	176.34	-0.73	included	present	359.71	-0.19	included	present	154.35	[illegible]
207791_s_at	included	present	4696.94	-0.09	included	present	4980.72	0.13	included	present	5509.44	[illegible]
207792_at	excluded	absent	15.59	-0.11	excluded	absent	10.23	-0.36	excluded	absent	45.64	[illegible]
207793_s_at	excluded	absent	78.09	-0.02	excluded	absent	75.16	-0.07	included	present	149.12	[illegible]
207794_at	excluded	absent	57.23	0.12	excluded	absent	38.37	-0.26	excluded	absent	24.02	[illegible]
207795_s_at	excluded	absent	153.58	0.48	excluded	absent	73.04	-0.17	excluded	absent	24.45	[illegible]
207796_x_at	included	present	210.7	1.11	included	present	128.46	0	excluded	absent	103.19	[illegible]
207797_s_at	included	present	187.52	-1.02	included	present	553.06	-0.03	included	present	193.4	[illegible]
207798_s_at	included	present	224.49	-0.7	included	absent	219.74	-0.74	included	present	289.48	[illegible]
207799_x_at	included	present	197.81	-0.06	included	absent	83.77	-1.35	included	present	183.72	[illegible]
207800_at	included	present	106.17	-0.45	excluded	absent	120.71	-0.42	included	present	176.5	[illegible]
207801_s_at	included	present	699.96	-1.09	included	present	939.13	-0.48	included	present	490.64	[illegible]
207802_at	excluded	absent	2.33	-0.17	excluded	absent	3.47	-0.17	excluded	absent	7.88	[illegible]
207803_s_at	excluded	absent	9.05	-0.17	excluded	absent	12.09	-0.16	excluded	absent	14.92	[illegible]
207804_s_at	excluded	absent	34.93	-0.2	excluded	absent	8.69	-0.28	excluded	absent	2.73	[illegible]
207805_s_at	included	present	1291.32	-0.25	included	present	1964.23	0.91	included	present	1217.46	[illegible]
207808_s_at	included	present	9160.92	2.56	included	present	647.4	-0.49	included	present	842.14	[illegible]
207809_s_at	included	present	634.5	0.65	included	present	650.1	0.73	included	present	188.45	[illegible]
207810_at	excluded	absent	1.68	-0.28	excluded	absent	1.24	-0.29	excluded	absent	74.02	[illegible]
207811_at	excluded	absent	8.8	-0.38	excluded	absent	2.69	-0.63	excluded	absent	8.24	[illegible]
207812_s_at	included	present	1509.41	-0.55	included	present	1108.79	-1.47	included	present	1198.57	[illegible]
207813_s_at	included	present	478.81	0.04	included	present	1333.94	1.18	included	present	260.85	[illegible]
207814_at	excluded	absent	3.77	-0.17	excluded	absent	9.19	-0.17	excluded	absent	28.91	[illegible]
207815_at	excluded	absent	1.74	-0.53	excluded	absent	2.6	-0.5	excluded	absent	36.49	[illegible]
207816_at	excluded	absent	2.61	-0.65	excluded	absent	1.41	-0.68	excluded	absent	1.14	[illegible]
207817_at	excluded	absent	1.37	-0.91	excluded	absent	8.68	-0.71	excluded	absent	44.29	[illegible]
207818_s_at	excluded	absent	77.51	1.18	excluded	absent	7.05	-0.96	excluded	absent	3.28	[illegible]
207819_s_at	excluded	absent	154.03	-0.23	included	present	263.92	-0.03	excluded	absent	129.31	[illegible]
207820_at	excluded	absent	54.89	-0.14	excluded	absent	4.57	-0.19	excluded	absent	97.38	[illegible]
207821_s_at	included	present	783.01	-0.29	included	present	382.66	-1.01	included	present	1766.3	[illegible]
207822_at	excluded	absent	268.1	0.39	excluded	absent	66.72	-0.89	excluded	absent	139.25	[illegible]
207823_s_at	excluded	absent	16.02	-0.04	excluded	absent	2.5	-0.6	excluded	absent	41.57	[illegible]
207824_s_at	included	absent	92.06	-0.95	included	present	242.35	1.18	included	present	267.77	[illegible]
207825_s_at	excluded	absent	40.06	-0.12	excluded	absent	80.14	-0.03	excluded	absent	2.05	[illegible]
[illegible]	[illegible]	[illegible]	[illegible]	[illegible]	[illegible]	[illegible]	[illegible]	[illegible]	[illegible]	[illegible]	[illegible]	[illegible]

208163_s_at	excluded	absent	18.43	-0.07	excluded	absent	7.76	-0.55	excluded	absent	5.17	-0.67	excluded	absent	3.55	-0.74
208164_s_at	excluded	absent	42.97	1.62	excluded	absent	5.2	-0.64	excluded	absent	4.01	-0.71	excluded	absent	38.41	1.35
208165_s_at	excluded	absent	2.58	-0.48	excluded	absent	4.82	-0.43	excluded	absent	1.83	-0.49	included	present	1047.95	21.63
208166_at	included	present	88.27	1.9	excluded	absent	44.76	0.46	excluded	absent	40.15	0.3	excluded	absent	2.35	-0.95
208167_s_at	included	present	376.23	1.48	included	present	145.31	-0.22	included	present	402.97	1.68	included	present	118.51	-0.42
208168_s_at	excluded	absent	55.51	1.09	excluded	absent	40.55	0.54	excluded	absent	43.3	0.64	excluded	absent	8.14	-0.67
208169_s_at	excluded	absent	44.64	-0.26	excluded	absent	4.08	-0.45	excluded	absent	51.69	-0.22	excluded	absent	3.05	-0.46
208170_s_at	excluded	absent	20.4	-0.24	excluded	absent	1.67	-0.28	excluded	absent	4.33	-0.27	excluded	absent	46.33	-0.19
208172_s_at	excluded	absent	125.48	0.7	included	present	217.59	1.91	included	present	564.12	6.47	included	present	649.53	7.6
208173_at	excluded	absent	33.62	0.76	excluded	absent	4.69	-0.46	excluded	absent	4.87	-0.45	excluded	absent	7.7	-0.33
208174_x_at	included	present	2071.12	1.09	included	present	1829.65	0.6	included	present	1483.38	-0.09	included	present	1146.93	-0.77
208175_s_at	excluded	absent	18.26	-0.39	excluded	absent	9.57	-0.67	excluded	absent	1.95	-0.92	excluded	absent	30.48	0.01
208176_at	excluded	absent	12.88	-0.78	excluded	absent	9.52	-0.88	excluded	absent	8.78	-0.91	excluded	absent	5.03	-1.03
208177_at	excluded	absent	14.38	-0.15	excluded	absent	112.18	-0.13	excluded	absent	2.98	-0.16	excluded	absent	8.12	-0.15
208178_x_at	included	present	746.5	3.35	included	present	539.25	1.79	included	present	1250.32	7.12	included	present	246.97	-0.4
208179_x_at	excluded	absent	9.6	-0.82	excluded	absent	52.11	0.08	excluded	absent	67.66	0.41	excluded	absent	3.02	-0.97
208180_s_at	included	present	635.86	-0.18	included	present	542.65	-0.47	included	present	2414.34	5.28	included	present	1358.42	2.04
208181_at	included	present	84.64	0.84	excluded	absent	79.5	0.71	excluded	absent	81.94	0.77	included	present	112.46	1.51
208182_x_at	excluded	absent	2.37	-0.54	excluded	absent	2.84	-0.51	excluded	absent	2.26	-0.54	excluded	absent	2.54	-0.53
208183_at	excluded	absent	55.64	0.22	excluded	absent	61.32	0.36	excluded	absent	31.34	-0.4	excluded	absent	52.71	0.14
208184_s_at	included	present	878.37	0.33	included	present	1137.09	1.36	included	present	632.74	-0.65	included	present	1764.29	3.86
208185_x_at	included	present	1081.91	0.35	included	absent	477.52	-1.55	included	present	1255.82	0.9	included	present	839.77	-0.41
208186_s_at	excluded	absent	4.32	-0.8	excluded	absent	41.39	-0.29	excluded	absent	56.7	-0.08	excluded	absent	3.4	-0.82
208187_s_at	excluded	absent	50.87	-0.45	excluded	absent	1.74	-1.24	included	present	171.17	1.48	excluded	absent	35.28	-0.7
208188_at	excluded	absent	125.49	0.62	excluded	absent	168.32	1.14	excluded	absent	84.74	0.12	excluded	absent	55.57	-0.23
208189_s_at	excluded	absent	89.97	-0.14	excluded	absent	158.72	0.36	excluded	absent	56.35	-0.39	excluded	absent	80.28	-0.21
208190_s_at	excluded	absent	99.7	-0.61	excluded	absent	54.85	-0.81	excluded	absent	116.74	-0.53	excluded	absent	481.04	1.15
208191_x_at	included	present	92.16	-0.5	included	present	133.33	-0.12	excluded	absent	20.88	-1.16	excluded	absent	11.36	-1.24
208192_at	excluded	absent	42.22	0.38	excluded	absent	68.17	1.1	excluded	absent	38.03	0.27	excluded	absent	33.12	0.13
208193_at	excluded	absent	46.84	0.15	excluded	absent	4.12	-0.53	excluded	absent	1.39	-0.57	excluded	absent	3.73	-0.54
208194_s_at	included	present	2132.52	-1.73	included	present	2078.58	-1.81	included	present	1934.7	-2	included	present	2326	-1.47
208195_at	excluded	absent	82.95	-0.2	excluded	absent	81.99	-0.2	included	present	144.09	-0.2	included	present	149.78	-0.2
208196_x_at	excluded	absent	49.97	-0.24	excluded	absent	86.26	0.36	excluded	absent	89.16	0.41	excluded	absent	104.51	0.66
208198_x_at	excluded	absent	38.78	0.12	excluded	absent	80.48	1.21	excluded	absent	52.71	0.48	excluded	absent	27.57	-0.17
208199_s_at	included	present	540.91	1.07	included	present	214.22	-1.19	included	present	318.68	-0.47	included	present	497.48	0.77
208200_at	included	present	151.33	0.46	excluded	absent	40.91	-0.96	included	present	120	0.06	included	present	190.51	0.96
208201_at	excluded	absent	16.33	-0.4	excluded	absent	6.52	-0.7	excluded	absent	36.54	0.24	excluded	absent	11.79	-0.54
208202_s_at	excluded	absent	32.86	-0.68	excluded	absent	16.15	-1	excluded	absent	5.47	-1.2	excluded	absent	21.63	-0.89
208203_x_at	excluded	absent	99.83	0.82	excluded	absent	156.33	1.74	excluded	absent	85.84	0.59	excluded	absent	44.9	-0.08
208204_s_at	excluded	absent	83.79	0.07	excluded	absent	4.35	-0.4	excluded	absent	70.94	-0.01	excluded	absent	63.04	-0.05
208205_at	excluded	absent	41.03	-0.52	excluded	absent	78.54	0.48	included	present	229.01	4.5	included	present	115.62	1.47
208206_s_at	included	present	299.79	0.16	included	present	206	-0.17	included	absent	46.71	-0.74	included	absent	8.07	-0.88
208207_at	excluded	absent	86.25	1.54	excluded	absent	12	-0.92	excluded	absent	55.89	0.53	excluded	absent	2.86	-1.22
208208_at	excluded	absent	70.87	0.65	excluded	absent	13.72	-0.74	excluded	absent	34.42	-0.24	excluded	absent	1.79	-1.03
208209_s_at	excluded	absent	2.82	-0.18	excluded	absent	4.32	-0.17	excluded	absent	191.05	-0.07	excluded	absent	14.24	-0.17
208210_at	excluded	absent	2.66	-0.81	excluded	absent	26.29	-0.26	excluded	absent	15.52	-0.51	excluded	absent	2.62	-0.81
208211_s_at	excluded	absent	90.23	0.57	included	present	690.23	13.71	included	present	753.83	15.11	excluded	absent	10.08	-1.18
208212_s_at	included	present	518.3	1.68	included	present	6979.7	42.65	included	present	4383.07	26.19	included	absent	3.92	-1.58

208531_at	excluded	absent	8	-0.6	excluded	absent	4.01	-0.73	excluded	absent	4.82	-0.7	excluded	absent	2.28	-0.78	excluded	absent	8.1	[illegible]		
208532_x_at	excluded	absent	7.38	-0.49	excluded	absent	11.2	-0.3	excluded	absent	14.65	-0.14	excluded	absent	10.77	-0.32	excluded	absent	7.55	[illegible]		
208533_at	excluded	absent	25.12	1.54	excluded	absent	9.05	0	excluded	absent	3.39	-0.54	excluded	absent	3.22	-0.55	excluded	absent	3.25	[illegible]		
208534_s_at	excluded	absent	69.9	-0.24	excluded	absent	137.04	0.43	excluded	absent	39.23	-0.54	excluded	absent	115.37	0.21	excluded	absent	19.01	[illegible]		
208535_x_at	excluded	absent	14.72	-1.33	excluded	absent	77.13	-0.13	excluded	absent	65.51	-0.35	excluded	absent	2.82	-1.56	excluded	absent	15.33	[illegible]		
208536_s_at	included	absent	113.04	-0.83	included	absent	45.75	-1.25	included	present	327.09	0.53	included	present	319.28	0.48	included	present	189.33	[illegible]		
208537_at	excluded	absent	25.19	-0.66	excluded	absent	16.78	-0.82	excluded	absent	138.52	1.48	excluded	absent	50.89	-0.17	excluded	absent	24.79	[illegible]		
208538_at	excluded	absent	109.3	-0.01	excluded	absent	97.96	-0.13	excluded	absent	11.55	-1.07	included	present	47.22	-0.68	excluded	absent	158.28	[illegible]		
208539_x_at	included	present	106.77	-0.22	excluded	absent	174.5	-0.1	excluded	absent	95.98	-0.24	excluded	absent	97.24	-0.23	excluded	absent	131.18	[illegible]		
208540_x_at	excluded	absent	296.91	0.23	included	present	163.76	-0.56	excluded	absent	181.5	-0.46	included	present	99.67	-0.94	included	present	352.6	[illegible]		
208541_x_at	included	present	1283.95	-0.52	included	present	1957.85	0.37	included	present	2418.86	0.99	included	present	1819.02	0.19	included	present	1849.88	[illegible]		
208542_x_at	included	present	114.27	-0.87	included	present	798.45	1.14	included	absent	45.66	-1.08	included	present	78.57	-0.98	included	present	317.77	[illegible]		
208543_at	excluded	absent	2.87	-0.5	excluded	absent	40.49	1.32	excluded	absent	1.97	-0.54	excluded	absent	4.8	-0.4	excluded	absent	43.79	[illegible]		
208544_at	excluded	absent	30.31	-0.49	excluded	absent	182.22	2.81	excluded	absent	16.6	-0.78	excluded	absent	28.78	-0.52	excluded	absent	59.38	[illegible]		
208545_x_at	included	present	212.89	-0.31	included	present	262.19	0.15	included	present	471.03	2.06	included	present	460.53	1.97	included	present	369.71	[illegible]		
208546_x_at	included	present	587.9	0.14	included	present	1377.83	1.82	included	present	5362.27	10.25	included	present	1511.65	2.1	included	present	2819.85	[illegible]		
208547_at	excluded	absent	3.16	-0.9	excluded	absent	14.44	-0.62	included	present	164.43	3.05	excluded	absent	90.34	1.24	excluded	absent	87.59	[illegible]		
208548_at	excluded	absent	2.36	-0.95	excluded	absent	5.33	-0.84	excluded	absent	18.58	-0.36	excluded	absent	18.17	-0.37	excluded	absent	7.57	[illegible]		
208549_x_at	included	present	7999.53	-0.69	included	present	12942.93	0.98	included	present	11135.41	0.37	included	present	13325.28	1.11	included	present	9809.16	[illegible]		
208550_x_at	excluded	absent	38.91	-0.04	excluded	absent	19.83	-0.45	excluded	absent	52.75	0.25	excluded	absent	6.35	-0.74	excluded	absent	118.47	[illegible]		
208551_at	excluded	absent	2.76	-1.1	excluded	absent	49.24	0.39	excluded	absent	104.68	2.17	excluded	absent	63.87	0.86	excluded	absent	75.37	[illegible]		
208552_at	excluded	absent	5.79	-0.78	excluded	absent	23.08	-0.36	excluded	absent	48.67	0.28	excluded	absent	3.97	-0.83	excluded	absent	16.03	[illegible]		
208553_at	included	present	1192.64	0.54	included	present	707.87	-0.16	included	present	1904.58	1.56	included	present	1808.92	1.43	included	present	1827.73	[illegible]		
208554_at	excluded	absent	3.91	-0.61	excluded	absent	5.51	-0.49	excluded	absent	15.13	0.25	excluded	absent	3.05	-0.68	excluded	absent	6.64	[illegible]		
208555_x_at	excluded	absent	92.14	-0.15	excluded	absent	77.64	-0.15	excluded	absent	15.27	-0.15	excluded	absent	3.87	-0.15	excluded	absent	117.11	[illegible]		
208556_at	excluded	absent	4.6	-0.3	excluded	absent	34.73	3.67	excluded	absent	2.88	-0.53	excluded	absent	2.23	-0.62	excluded	absent	19.51	[illegible]		
208557_at	excluded	absent	51.51	-0.53	excluded	absent	166.07	1.55	excluded	absent	73.85	-0.13	excluded	absent	58.39	-0.41	excluded	absent	107.79	[illegible]		
208558_at	excluded	absent	56.66	0.62	excluded	absent	89.29	1.48	excluded	absent	6.04	-0.71	excluded	absent	11.58	-0.57	excluded	absent	8.38	[illegible]		
208559_at	excluded	absent	5	-0.72	excluded	absent	3.74	-0.86	excluded	absent	4.08	-0.82	excluded	absent	3.15	-0.93	excluded	absent	17.11	[illegible]		
208560_at	excluded	absent	44.86	1.06	excluded	absent	63.31	1.84	excluded	absent	3.52	-0.67	excluded	absent	52.51	1.38	excluded	absent	69.6	[illegible]		
208561_at	included	present	107.15	-0.84	included	present	211.13	-0.73	included	absent	2.52	-0.95	included	present	838.04	-0.06	included	present	652.82	[illegible]		
208562_s_at	included	absent	50.26	-0.93	included	absent	74.25	-0.86	included	absent	48.52	-0.94	included	present	349.25	-0.02	included	absent	207.88	[illegible]		
208563_x_at	excluded	absent	4.63	-0.6	excluded	absent	5.29	-0.58	excluded	absent	100.67	1.81	excluded	absent	2.43	-0.66	excluded	absent	5.35	[illegible]		
208564_at	included	present	389.14	0.63	excluded	absent	75.84	-0.51	excluded	absent	2.2	-0.78	excluded	absent	1.48	-0.78	excluded	absent	32.25	[illegible]		
[illegible]																						

208893_s_at	included	present	1407.54	4.29	included	present	351.37	0.08	included	present	252.9	-0.31	included	present	317.44	-0.06	included	present	436.51	0.42	included	present	759.14	1.7	included	present	213.63	-0.47	included	present	415.91	0.34	included	present	600.06	1.07	included	present	315.73	-0.06	included	present	585.27	1.01	included	present	161.36	-0.68	included	present	786.06	1.81	included	present	627.49	1.18	included	present	584.33	1.01
208894_at	included	present	16176.63	1.3	included	present	3886.05	-0.23	included	present	3703.48	-0.26	included	present	2123.77	-0.45	included	present	5760.04	0	included	present	12025.19	0.78	included	present	5117.54	-0.08	included	present	1478.75	-0.53	included	present	12134.81	0.8	included	present	2794.96	-0.37	included	present	7143.06	0.17	included	present	3987.76	-0.22	included	present	1830.18	-0.49	included	present	605.25	-0.64	included	present	13001.72	0.9
208895_s_at	included	present	2604.12	0.72	included	present	3132.27	1.6	included	present	3247.25	1.79	included	present	1748.71	-0.72	included	present	2095.92	-0.13	included	present	2781.57	1.01	included	present	2030.94	-0.24	included	present	5493.61	5.55	included	present	1850.33	-0.55	included	present	2357.04	0.3	included	present	2551.52	0.63	included	present	1597.22	-0.97	included	present	2800.24	1.04	included	present	4770.22	4.34	included	present	2182.93	0.01
208896_at	included	present	1161.19	-0.26	included	present	1790.02	0.97	included	present	2298.42	1.96	included	present	907.63	-0.75	included	present	774.6	-1.01	included	present	857.92	-0.85	included	present	2006.69	1.39	included	present	3382.65	4.07	included	present	1512.97	0.43	included	present	970.32	-0.63	included	present	1411.2	0.23	included	present	725.02	-1.11	included	present	1043.21	-0.49	included	present	1650.12	0.69	included	present	1099.23	-0.38
208897_s_at	included	present	2036.53	-2.14	included	present	4873.25	1.39	included	present	3903.29	0.18	included	present	2047.89	-2.13	included	present	2092.35	-2.07	included	present	2017.29	-2.17	included	present	2208.89	-1.93	included	present	5991.02	2.78	included	present	2604.67	-1.44	included	present	2482.79	-1.59	included	present	3390.32	-0.46	included	present	1949.31	-2.25	included	present	7068.26	4.12	included	present	11674.81	9.84	included	present	2237.54	-1.89
208898_at	included	present	6231.6	0.87	included	present	6450.51	0.97	included	present	4736.9	0.21	included	present	4856.18	0.27	included	present	3945.04	-0.13	included	present	1423.14	-1.24	included	present	2805.33	-0.64	included	present	3177.63	-0.47	included	present	2335.74	-0.84	included	present	3903.18	-0.15	included	present	1770.82	-1.09	included	present	4625.66	0.17	included	present	3125.08	-0.49	included	present	4766.25	0.23	included	present	2746.14	-0.66
208899_x_at	included	present	2083.12	-0.31	included	present	2856.86	0.31	included	present	1805.64	-0.53	included	present	1797.61	-0.54	included	present	1775.79	-0.55	included	present	950.96	-1.21	included	present	1737.78	-0.58	included	present	2664.15	0.16	included	present	1629.26	-0.67	included	present	2267.83	-0.16	included	present	1355.93	-0.89	included	present	1363.05	-0.88	included	present	618.24	-1.48	included	present	1212.21	-1	included	present	1510.86	-0.77
208900_s_at	included	present	1862.13	-0.42	included	present	4806.16	4.71	included	present	2321.81	0.38	included	present	2275.04	0.3	included	present	4245.61	3.73	included	present	2063.45	-0.07	included	present	1878.74	-0.39	included	present	3441.29	2.33	included	present	1696.69	-0.71	included	present	2491.23	0.68	included	present	2881.73	1.36	included	present	2900.6	1.39	included	present	4385.07	3.98	included	present	4521.87	4.22	included	present	2384.8	0.49
208901_s_at	included	present	858.74	-1.2	included	present	1757.87	0.23	included	present	1562.4	-0.08	included	present	1680.95	0.11	included	present	1964.67	0.56	included	present	1437.27	-0.28	included	present	1022.54	-0.94	included	present	2532.29	1.46	included	present	913.16	-1.11	included	present	1829.24	0.34	included	present	1359.79	-0.4	included	present	797.1	-1.29	included	present	865.8	-1.18	included	present	619.41	-1.57	included	present	1482.88	-0.21
208902_s_at	excluded	absent	165.75	-0.05	excluded	absent	93	-0.9	included	present	183.77	0.16	excluded	absent	61.82	-1.27	excluded	absent	179.32	0.11	included	present	397.39	2.67	included	present	408.21	2.8	included	present	164.82	-0.06	included	present	469.27	3.52	included	present	265.29	1.12	included	present	452.24	3.32	excluded	absent	72.67	-1.14	excluded	absent	133.72	-0.43	excluded	absent	89.84	-0.94	included	present	458.42	3.39
208903_at	included	present	199.74	-0.99	included	present	425.5	0.2	included	present	338.35	-0.26	included	present	326.34	-0.32	included	present	720.14	1.75	included	absent	193.67	-1.02	included	present	1463.61	5.67	included	present	952.92	2.98	included	present	920.51	2.81	included	present	486.08	0.52	included	present	1043.82	3.46	included	present	741.02	1.86	included	present	573.88	0.98	included	present	641.01	1.34	included	present	1200.04	4.28
208904_s_at	included	present	11131.25	-0.8	included	present	16670.2	-0.16	included	present	14796.69	-0.38	included	present	16709.16	-0.15	included	present	13938.12	-0.47	included	present	22533.78	0.52	included	present	9425.42	-1	included	present	27052.95	1.05	included	present	10669.47	-0.85	included	present	14436.72	-0.42	included	present	10981.67	-0.82	included	present	34093.83	1.86	included	present	14871.73	-0.37	included	present	16151.1	-0.22	included	present	13780.47	-0.49
208905_at	included	present	2395.93	-0.95	included	present	3664.42	-0.51	included	present	6580.7	0.52	included	present	3343.8	-0.62	included	present	9414.72	1.52	included	present	2594.95	-0.88	included	present	3823.34	-0.45	included	present	3437.3	-0.59	included	present	2799.05	-0.81	included	present	4079.24	-0.36	included	present	5119.82	0	included	present	6797.6	0.6	included	present	5752.81	0.23	included	present	6027.5	0.32	included	present	5410.24	0.11
208906_at	included	present	5118.17	5.14	included	present	1696.29	0.84	included	present	809.42	-0.27	included	present	315.02	-0.9	included	present	824.54	-0.26	included	present	966.88	-0.08	included	present	5347.6	5.43	included	present	1666.52	0.8	included	present	4677.71	4.59	included	present	8337.02	9.19	included	present	2999.25	2.48	included	present	960.91	-0.08	included	present	585.22	-0.56	included	present	215.12	-1.02	included	present	6183.98	6.48
208907_s_at	included	present	727.97	1.36	included	present	1383.57	4.13	included	present	899.56	2.09	included	present	945.26	2.28	included	present	1052.41	2.73	included	present	512.21	0.46	included	present	935.01	2.24	included	present	803.26	1.68	included	present	825.14	1.77	included	present	815.25	1.73	included	present	962.18	2.35	included	absent	116.62	-1.21	included	present	132.94	-1.14	included	absent	21.43	-1.61	included	present	1223.14	3.45
208908_s_at	included	present	2363.25	-0.35	included	present	797.8	-1.01	included	present	1142.73	-0.87	included	present	1985.35	-0.51	included	present	1678.53	-0.64	included	present	2422.6	-0.32	included	present	524.63	-1.13	included	present	1818.14	-0.58	included	present	2633.63	-0.23	included	present	1481.22	-0.72	included	present	772.15	-1.02	included	present	1431.07	-0.74	included	present	1716.28	-0.62	included	present	1737.42	-0.61	included	present	905.81	-0.97
208909_at	included	present	1672.39	-0.81	included	present	2966.1	-0.26	included	present	2651.68	-0.39	included	present	3755.5	0.08	included	present	1703.71	-0.8	included	present	3050.02	-0.22	included	present	1985.55	-0.68	included	present	4184.98	0.27	included	present	2000.63	-0.67	included	present	3328.74	-0.1	included	present	2474.8	-0.47	included	present	4549.5	0.42	included	present	1792.75	-0.76	included	present	2071.6	-0.64	included	present	3408.29	-0.07
208910_s_at	included	present	2669.63	-1.07	included	present	7191.54	2.24	included	present	6030.87	1.39	included	present	2587.78	-1.13	included	present	5322.77	0.87	included	present	3110.37	-0.75	included	present	2737.98	-1.02	included	present	6299.07	1.59	included	present	1993.41	-1.57	included	present	2592.06	-1.13	included	present	3149.36	-0.72	included	present	3440.46	-0.51	included	present	5553.98	1.04	included	present	8476.22	3.18	included	present	3615.53	-0.38
208911_s_at	included	present	2565.13	0.63	included	present	2156.61	0.01	included	present	3562.63	2.14	included	present	986.03	-1.76	included	present	2887.2	1.12	included	present	1643.72	-0.76	included	present	2196.05	0.07	included	present	2886.98	1.12	included	present	1801.8	-0.52	included	present	2136.96	-0.02	included	present	1204.66	-1.43	included	present	733.22	-2.14	included	present	342.33	-2.73	included	present	658.66	-2.25	included	present	2146.23	0
208912_s_at	included	present	7790.4	0.1	included	present	3677.2	-0.33	included	present	2880.1	-0.41	included	present	1238.14	-0.59	included	present	1818.07	-0.53	included	present	3553.24	-0.34	included	present	5235.56	-0.17	included	present	2435.86	-0.46	included	present	3110.26	-0.39	included	present	3631.6	-0.33	included	present	3801.15	-0.32	included	present	1577.61	-0.55	included	present	1366.69	-0.57	included	present	965.36	-0.62	included	present	7678.8	0.09
208913_at	included	present	3283.6	-0.8	included	present	9300.67	1.01	included	present	4679.55	-0.38	included	present	6198.77	0.08	included	present	6908.79	0.29	included	present	3373.32	-0.77	included	present	2865.64	-0.92	included	present	4241.47	-0.51	included	present	2846.27	-0.93	included	present	2248.84	-1.11	included	present	3546.44	-0.72	included	present	6700.51	0.23	included	present	5507.58	-0.13	included	present	7082.83	0.34	included	present	2590.47	-1.01
208914_at	included	present	481.38	-0.04	included	present	364.48	-0.37	included	present	627.72	0.36	included	present	574.77	0.21	included	present	517.97	0.06	included	present	480.41	-0.05	included	present	269.59	-0.63	included	present	685.28	0.52	included	present	611.39	0.32	included	present	344.27	-0.43	included	present	998	1.39	included	present	386.89	-0.31	included	present	198.66	-0.83	included	present	135.37	-1.01	included	present	469.21	-0.08
208915_s_at	included	present	319.03	-0.31	included	present	241.53	-0.65	included	present	384.22	-0.02	included	present	351.47	-0.17	included	present	260.45	-0.56	excluded	absent	425.68	0.16	excluded	absent	288.85	-0.44	included	present	529.37	0.61	included	present	537.52	0.65	included	present	264.48	-0.55	included	present	625.39	1.03	included	present	214.98	-0.76	excluded	absent	122.02	-1.17	excluded	absent	70.1	-1.4	included	present	395.27	0.02
208916_at	excluded	absent	94.83	-0.49	excluded	absent	134.51	-0.1	excluded	absent	168.4	0.24	included	present	221.93	0.77	excluded	absent	67.74	-0.76	included	present	245.11	1	excluded	absent	149.93	0.06	included	present	193.23	0.48	excluded	absent	143	-0.01	excluded	absent	120.15	-0.24	excluded	absent	165.42	0.21	included	present	261.03	1.15	excluded	absent	90.17	-0.53	excluded	absent	99.5	-0.44	included	present	135.66	-0.08
208917_x_at	excluded	absent	36.58	-0.79	excluded	absent	122.03	-0.06	excluded	absent	15.71	-0.96	excluded	absent	8.58	-1.02	excluded	absent	253.56	1.05	excluded	absent	240.49	0.94	excluded	absent	81.64	-0.4	excluded	absent	127.61	-0.02	included	present	233.15	0.88	excluded	absent	140.27	0.09	excluded	absent	113.63	-0.13	included	present	216.41	0.74	excluded	absent	34.01	-0.81	excluded	absent	68.05	-0.52	included	present	152.03	0.19
208918_s_at	included	present	789.94	0.31	included	present	1759.83	2.78	included	present	258.79	-1.04	included	present	448.3	-0.56	included	present	1737.15	2.72	included	present	1022.81	0.9	included	present	615.99	-0.13	included	present	921.64	0.65	included	present	958.59	0.74	included	present	570.96	-0.25	included	present	779.4	0.28	included	present	2080.19	3.59	included	present	205.81	-1.17	included	present	1002.67	0.85	included	present	964.95	0.76
208919_s_at	included	present	418.39	-0.52	included	present	1063.63	2.3	included	present	177.9	-1.57	included	present	207.21	-1.44	included	present	659.61	0.54	included	present	622.76	0.37	included	present	436.25	-0.44	included	present	493.41	-0.19	included	present	422.82	-0.5	included	present	521.57	-0.07	included	present	342.8	-0.85	included	present	961.09	1.85	included	present	174.07	-1.58	included	absent	389.31	-0.64	included	present	566.07	0.13
208920_at	included	present	643.51	-0.23	included	present	244.76	-0.87	included	present	225.25	-0.9	included	present	180.1	-0.98	included	present	454.96	-0.54	included	present	392.77	-0.64	included	present	1178.51	0.62	included	present	541.08	-0.4	included	present	696.28	-0.15	included	present	1121.96	0.53	included	present	872.2	0.13	included	present	518.39	-0.43	included	present	1069.31	0.45	included	present	687.5	-0.16	included	present	985.73	0.31
208921_s_at	included	present	5623.46	-0.1	included	present	10126.91	1.21	included	present	6573.93	0.18	included	present	3038.29	-0.85	included	present	7594.78	0.47	included	present	3464.81	-0.73	included	present	4728.51	-0.36	included	present	4118.81	-0.54	included	present	4696.88	-0.37	included	present	5549.22	-0.12	included	present	5120.97	-0.25	included	present	7372.05	0.41	included	present	3769.51	-0.64	included	present	5899.1	-0.02	included	present	5334.4	-0.18
208922_s_at	included	present	1090.16	-0.18	included	present	1058.24	-0.25	included	present	874.71	-0.63	included	present	856.7	-0.67	included	present	890.23	-0.6	included	present	1414.07	0.51	included	present	1194.01	0.04	included	present	1026.23	-0.31	included	present	1144.75	-0.06	included	present	1681.52	1.07	included	present	1224.34	0.1	included	present	465.86	-1.5	included	present	837.67	-0.71	included	present	495.85	-1.43	included	present	1424.57	0.53
208923_at	included	present	3298.81	0.2	included	present	2095.45	-0.95	included	present	3684.91	0.56	included	present	2358.35	-0.7	included	present	1269.4	-1.74	included	present	4445.65	1.29	included	present	1257.7	-1.75	included	present	4565.02	1.4	included	present	2517.21	-0.55	included	present	1146.13	-1.86	included	present	2030.51	-1.01	included	present	2879.61	-0.2	included	present	2335.76	-0.72	included	present	2503.98	-0.56	included	present	1315.64	-1.69
208924_at	included	present	1786.15	-0.5	included	present	1296.61	-1.01	included	present	761.49	-1.56	included	present	2301.12	0.04	included	present	1411.83	-0.89	included	present	1638.89	-0.65	included	present	2889.31	0.65	included	present	1805.97	-0.48	included	present	2678.7	0.43	included	present	1220.95	-1.08	included	present	1441.39	-0.86	included	present	2031.88	-0.24	included	present	2658.56	0.41	included	present	1612.01	-0.68	included	present	1357.13	-0.94
208925_at	included	present	1176.92	-0.43	included	present	471.6	-0.64	included	present	995.24	-0.48	included	present	582.81	-0.61	included	present	477.76	-0.64	included	present	691.22	-0.57	included	present	1046.61	-0.47	included	present	1414.83	-0.35	included	present	1153.07	-0.43	included	present	807.82	-0.54	included	present	894.02	-0.51	included	present	306.68	-0.69	included	present	625.38	-0.59	included	present	241.02	-0.71	included	present	1125.59	-0.44
208926_at	included	present	263.46	0.84	included	present	183.18	0.09	excluded	absent	113.69	-0.56	included	present	267.15	0.87	included	present	207.21	0.31	included	present	392.4	2.04	included	present	496.48	3.01	included	present	633.26	4.29	included	present	324.64	1.41	included	present	244.16	0.65	excluded	absent	189.58	0.14	included	present	115.14	-0.55	excluded	absent	106.87	-0.63	included	present	76.78	-0.91	included	present	437.84	2.46

[illegible]

209253_at	excluded	absent	142.32	0.06	included	present	195.13	0.76	excluded	absent	159.47	0.29	excluded	absent	49.67	-1.15	[illegible]
209254_at	included	present	1389.47	1.14	included	present	1147.72	0.47	included	present	1116.22	0.38	included	present	1639.31	1.84	[illegible]
209255_at	included	present	1224.21	1.64	included	present	1535.45	2.71	included	present	1309.25	1.94	included	present	2182.09	4.94	[illegible]
209256_s_at	included	present	2206.63	2.47	included	present	1708.49	1.1	included	present	1917.97	1.67	included	present	2949.11	4.52	[illegible]
209257_s_at	included	present	1719.33	1.54	included	present	2595.88	3.65	included	present	4757.4	8.85	included	present	3206.29	5.12	[illegible]
209258_s_at	included	present	6417.1	-0.26	included	present	16232.04	3.85	included	present	21378.19	6.01	included	present	13828.64	2.85	[illegible]
209259_s_at	included	present	2169.02	-0.28	included	present	3201.11	0.71	included	present	6418.97	3.81	included	present	3855.61	1.34	[illegible]
209260_at	excluded	absent	47.99	-0.27	excluded	absent	136.11	-0.22	excluded	absent	41.01	-0.27	excluded	absent	105.93	-0.24	[illegible]
209261_s_at	excluded	absent	17.21	-0.42	excluded	absent	15.72	-0.45	excluded	absent	11.15	-0.54	excluded	absent	4.98	-0.67	[illegible]
209262_s_at	excluded	absent	88.13	-0.69	excluded	absent	279.37	0.23	excluded	absent	181.84	-0.24	included	present	320.96	0.43	[illegible]
209263_x_at	excluded	absent	189.96	0.54	excluded	absent	293.04	1.6	excluded	absent	141.26	0.04	excluded	absent	11.6	-1.29	[illegible]
209264_s_at	excluded	absent	3.31	-1.27	excluded	absent	15.91	-0.96	excluded	absent	73.91	0.47	excluded	absent	42.05	-0.32	[illegible]
209265_s_at	included	present	2000.53	-0.79	included	present	4120.35	0.97	included	present	2714.67	-0.2	included	present	3334.02	0.32	[illegible]
209266_s_at	excluded	absent	42.29	0.52	excluded	absent	23.92	-0.07	excluded	absent	7.32	-0.6	excluded	absent	18.76	-0.24	[illegible]
209267_s_at	included	present	152.95	-0.42	included	present	218.8	-0.37	included	absent	79.58	-0.48	included	present	396.51	-0.23	[illegible]
209268_at	included	present	1662.24	0.79	included	present	2319.13	2.72	included	present	1789.56	1.16	included	present	3074.44	4.93	[illegible]
209269_s_at	included	present	233.71	0.49	excluded	absent	193.43	0.22	included	present	137.67	-0.15	included	present	272.92	0.75	[illegible]
209270_at	excluded	absent	95.66	-0.38	excluded	absent	169.71	-0.28	excluded	absent	41.84	-0.45	excluded	absent	2.78	-0.5	[illegible]
209271_at	included	present	899.49	0.53	included	present	1129.08	1.47	included	present	1349.48	2.37	included	present	1943.04	4.79	[illegible]
209272_at	included	present	2205.33	1.02	included	present	1237.91	-0.23	included	present	1451.22	0.05	included	present	4078.04	3.45	[illegible]
209273_s_at	included	present	1410.41	-0.03	included	present	761.54	-0.84	included	present	2997.25	1.97	included	present	1527.37	0.12	[illegible]
209274_s_at	included	present	1082.45	-1.05	included	present	1444.59	-0.52	included	present	1939.94	0.2	included	present	1626.37	-0.26	[illegible]
209275_s_at	excluded	absent	96.7	-0.03	excluded	absent	176.29	0.97	excluded	absent	45.04	-0.68	excluded	absent	16.08	-1.05	[illegible]
209276_s_at	included	present	461.32	-0.33	included	present	181.99	-0.54	included	present	295.12	-0.46	included	present	117.25	-0.59	[illegible]
209277_at	excluded	absent	49.54	-0.11	included	present	123.24	0.45	excluded	absent	8.43	-0.42	excluded	absent	5.53	-0.44	[illegible]
209278_s_at	excluded	absent	155.01	0.28	excluded	absent	5.52	-0.6	excluded	absent	90.88	-0.1	excluded	absent	5.98	-0.6	[illegible]
209279_s_at	included	present	289.51	0.8	included	present	176.04	0.01	included	present	152.72	-0.15	excluded	absent	120.91	-0.38	[illegible]
209280_at	excluded	absent	25.61	-1.37	excluded	absent	62.58	-0.79	excluded	absent	17.17	-1.5	excluded	absent	14.49	-1.54	[illegible]
209281_s_at	included	present	1266.11	-0.51	included	present	1562.83	-0.41	included	present	511.91	-0.74	included	present	766.74	-0.66	[illegible]
209282_at	excluded	absent	189.64	0.08	included	present	197.31	0.13	excluded	absent	111.47	-0.41	included	present	200.18	0.15	[illegible]
209283_at	included	present	14613.94	0.13	included	present	675.55	-0.8	included	present	653.69	-0.8	included	present	325.64	-0.83	[illegible]
209284_s_at	included	present	675.95	-0.4	included	present	373.85	-1.36	included	present	1068.68	0.86	included	present	401.33	-1.27	[illegible]
209285_s_at	included	present	1412.11	-0.69	included	present	1195.72	-1	included	present	1368.09	-0.75	included	present	509.47	-1.98	[illegible]
209286_at	included	present	885.27	-0.49	included	present	2594.3	0.29	included	present	4558.3	1.2	included	present	1146.95	-0.37	[illegible]
209287_s_at	included	present	201.68	-0.41	included	present	494.47	0.65	included	present	648.64	1.2	included	absent	135.34	-0.65	[illegible]
209288_s_at	included	present	523.27	-0.54	included	present	1201.65	-0.11	included	present	2291.64	0.58	included	present	734.54	-0.4	[illegible]
209289_at	included	present	1526.7	0.83	included	present	989.45	-0.16	included	present	5429.74	8.01	included	present	675.68	-0.73	[illegible]
209290_s_at	included	present	3827.53	0.56	included	present	2177.22	-0.55	included	present	11528.7	5.75	included	present	1684.79	-0.88	[illegible]
209291_at	included	present	1469.42	-0.86	included	present	1666.74	-0.77	included	present	343.2	-1.37	included	present	16330.2	5.95	[illegible]
209292_at	included	present	371.27	-0.87	included	present	100.62	-1.27	included	absent	5.2	-1.41	included	present	8263.97	10.6	[illegible]
209293_x_at	excluded	absent	192.71	0.44	excluded	absent	139.86	-0.29	excluded	absent	79.88	-1.13	included	present	234.72	1.03	[illegible]
209294_x_at	included	present	517.97	1.64	excluded	absent	164.51	-0.31	included	present	164.96	-0.31	excluded	absent	8.9	-1.17	[illegible]
[illegible]																	

209616_s_at	excluded	absent	7.39	-0.29	excluded	absent	95.63	-0.26	included	present	227.97	-0.22	excluded	absent	58.63	-0.27	excluded	absent	40.37	-0.28	excluded	absent	210.15	-0.22	excluded	absent	16.36	-0.28	excluded	absent	21.7	-0.28	included	present	196.89	-0.23	excluded	absent	47.79	-0.27	excluded	absent	96.49	-0.26	excluded	absent	96.83	-0.26	excluded	absent	22.78	-0.28	excluded	absent	2.46	-0.29	included	present	52.68	-0.27
209617_s_at	included	present	630.59	-0.54	included	present	1356.92	-0.24	included	present	998.35	-0.39	included	absent	24.78	-0.78	included	present	1670.36	-0.12	included	absent	92.71	-0.75	included	present	1207.19	-0.3	included	present	522.32	-0.58	included	present	1422.75	-0.22	included	present	1372.7	-0.24	included	present	1069.51	-0.36	included	present	1432.61	-0.21	included	present	846.63	-0.45	included	present	550.47	-0.57	included	present	1615.59	-0.14
209618_at	included	present	250.93	-0.62	included	present	272.15	-0.6	included	present	227.97	-0.64	excluded	absent	14.99	-0.81	included	present	406.47	-0.49	excluded	absent	9.51	-0.81	included	present	855.17	-0.13	included	present	242.69	-0.62	included	present	619.45	-0.32	included	present	543.58	-0.38	included	present	493.62	-0.42	included	present	851.38	-0.14	included	present	1000.78	-0.02	included	present	800.19	-0.18	included	present	719.41	-0.24
209619_at	included	present	31058.66	1.61	included	present	12707.23	0.11	included	present	5768.6	-0.46	included	present	3172.56	-0.67	included	present	11501.46	0.01	included	present	28182.46	1.37	included	present	8232.69	-0.26	included	present	3971.63	-0.61	included	present	17589.05	0.51	included	present	7358.1	-0.33	included	present	11222.52	-0.02	included	present	13802.26	0.2	included	present	10004.56	-0.12	included	present	5042.29	-0.52	included	present	17844.48	0.53
209620_s_at	included	present	667.69	-0.94	included	present	833.79	-0.44	included	present	1172.31	0.56	included	present	1252.69	0.8	included	present	827.94	-0.46	included	present	677.58	-0.91	included	present	815.37	-0.5	included	present	1172.46	0.56	included	present	794.81	-0.56	included	present	748.2	-0.7	included	present	643.59	-1.01	included	present	1767.68	2.33	included	present	679.81	-0.9	included	present	1411.92	1.27	included	present	824.98	-0.47
209621_s_at	included	absent	210.36	-0.45	included	present	413.72	-0.41	included	present	357.8	-0.42	included	absent	281.51	-0.43	included	present	830.93	-0.33	included	present	6188.13	0.66	included	absent	352.44	-0.42	included	present	1535.43	-0.2	included	present	939.74	-0.31	included	present	6488.33	0.71	included	absent	139.56	-0.46	included	absent	267.46	-0.44	included	absent	240.77	-0.44	included	absent	267.74	-0.44	included	present	483.19	-0.4
209622_at	included	present	282.26	0.84	included	present	347.93	1.5	included	present	179.41	-0.19	included	present	164.92	-0.33	excluded	absent	307.18	1.09	included	present	151.78	-0.47	included	present	144.25	-0.54	included	present	712.55	5.17	included	present	228.94	0.31	included	present	233.8	0.36	included	present	278.42	0.81	included	absent	160.69	-0.38	included	absent	7.82	-1.91	included	absent	74.29	-1.25	included	present	322.96	1.25
209623_at	included	present	711.13	0.05	included	present	562.83	-0.27	included	present	1158.98	1	included	present	1209.82	1.1	included	present	562.42	-0.27	included	present	490.25	-0.42	included	present	555.25	-0.28	included	present	1554.28	1.83	included	present	462.34	-0.48	included	present	354.74	-0.71	included	present	429.98	-0.55	included	present	803.84	0.24	included	present	427.82	-0.55	included	present	507.32	-0.38	included	present	539.06	-0.32
209624_s_at	included	present	827.23	-0.66	included	present	765.98	-0.7	included	present	1719.64	0	included	present	1147.42	-0.42	included	present	1191.6	-0.39	included	present	451.19	-0.94	included	present	343.48	-1.02	included	present	1589.01	-0.09	included	present	723.97	-0.73	included	present	423.21	-0.96	included	present	608.47	-0.82	included	present	328.02	-1.03	included	present	684.7	-0.76	included	present	523.35	-0.88	included	present	499.52	-0.9
209625_at	included	present	254.69	-0.66	included	absent	253.66	-0.67	included	present	276.03	-0.48	included	present	410.48	0.69	included	present	344.72	0.12	included	present	325.62	-0.05	included	present	374.73	0.38	included	present	438.66	0.94	included	present	345.04	0.12	included	present	423.09	0.8	included	present	272.32	-0.51	included	present	300.95	-0.26	included	present	196.75	-1.16	included	absent	79.88	-2.18	included	present	473.72	1.24
209626_s_at	included	present	939.2	0.82	included	present	490.43	-0.07	included	present	3906.07	6.71	included	present	66.49	-0.91	included	present	775.36	0.49	included	present	418.76	-0.21	included	present	512.8	-0.03	included	present	1716.35	2.36	included	present	1948	2.82	included	present	2504.65	3.93	included	present	1911.51	2.75	included	present	863.19	0.67	included	present	528.31	0	included	present	429.17	-0.19	included	present	555.56	0.06
209627_s_at	included	present	286.8	0.73	included	absent	69.13	-0.86	included	present	1283.29	7.99	included	absent	38.08	-1.09	excluded	absent	239.69	0.38	included	present	238.98	0.38	included	present	538.96	2.57	included	present	1186.64	7.29	included	present	1572.78	10.11	included	present	1962.29	12.95	included	present	1255.74	7.79	included	present	725.02	3.92	included	present	700.01	3.74	included	present	245.29	0.42	included	present	501.37	2.29
209628_at	included	present	207.97	-0.58	included	present	586.43	0.35	included	present	148.83	-0.72	included	present	260.25	-0.45	included	present	356.06	-0.21	included	present	545.74	0.25	included	present	264.65	-0.44	included	present	2014.3	3.85	included	present	522.33	0.19	included	present	426.82	-0.04	included	present	155.42	-0.71	included	present	1448.07	2.46	included	present	579.25	0.33	included	present	2411.47	4.83	included	present	516.45	0.18
209629_s_at	included	present	162.2	-0.54	included	present	229.84	-0.3	included	present	171.24	-0.51	included	present	223.83	-0.32	included	present	329.5	0.06	included	absent	92.21	-0.79	included	present	95.44	-0.78	included	present	991.67	2.45	included	present	338.54	0.1	included	present	170.1	-0.51	included	present	126.61	-0.67	included	present	83.94	-0.82	included	present	214.41	-0.35	included	present	180.34	-0.48	included	present	159.96	-0.55
209630_s_at	included	present	1424.84	2.11	included	present	640.77	-0.34	included	present	2137.98	4.34	included	present	2070.05	4.13	included	present	1225.97	1.49	included	present	1287.29	1.68	included	present	1388.3	1.99	included	present	1214.76	1.45	included	present	1827.74	3.37	included	present	1386.88	1.99	included	present	1984.78	3.86	included	present	1474.22	2.26	included	present	652.46	-0.31	included	present	799.41	0.15	included	present	1824.45	3.36
209631_s_at	included	absent	78.26	-0.54	included	absent	116.16	-0.52	included	absent	38.64	-0.57	included	absent	1.56	-0.59	included	absent	12.71	-0.58	included	absent	7.98	-0.59	included	absent	36.79	-0.57	included	absent	71.48	-0.55	included	present	55.85	-0.56	included	absent	15.2	-0.58	included	absent	4.56	-0.59	included	present	69.62	-0.55	included	absent	45.52	-0.56	included	present	127.02	-0.51	included	present	45.94	-0.56
209632_at	included	present	489.65	1.65	included	absent	120.79	-0.94	included	present	266.64	0.08	included	present	190.34	-0.46	included	present	230.78	-0.17	included	present	400.25	1.02	included	present	310.47	0.39	included	present	341.04	0.6	included	present	324.31	0.48	included	present	559.98	2.14	included	present	243.99	-0.08	included	present	352.92	0.69	included	present	399.65	1.01	included	present	251.09	-0.03	included	present	181.53	-0.52
209633_at	included	present	567.01	0.62	included	present	244.97	-0.51	included	present	231.24	-0.55	included	present	178.66	-0.74	included	absent	154.45	-0.82	included	present	571.41	0.64	included	present	748.49	1.26	included	present	437.98	0.17	included	present	398.41	0.03	included	present	1003.31	2.16	included	present	333.03	-0.2	included	present	288.67	-0.35	included	present	438.76	0.17	included	present	276.26	-0.4	included	present	349.72	-0.14
209635_at	included	present	349.54	-0.39	included	present	539.15	0.2	included	present	186.93	-0.89	included	present	264.62	-0.65	included	absent	120.31	-1.1	included	present	338.58	-0.42	included	present	690.98	0.67	included	present	494.26	0.06	included	present	304.81	-0.53	included	present	229.61	-0.76	included	present	674.01	0.61	included	present	554.35	0.24	included	present	299.77	-0.54	included	present	226.51	-0.77	included	present	608.8	0.41
209636_at	excluded	absent	29.03	-0.4	excluded	absent	59.1	0.03	excluded	absent	56.66	0	excluded	absent	9.18	-0.68	excluded	absent	20.89	-0.52	excluded	absent	114.98	0.84	excluded	absent	29.77	-0.39	excluded	absent	72.27	0.22	excluded	absent	116.68	0.86	excluded	absent	8.77	-0.69	excluded	absent	65.66	0.13	excluded	absent	64.86	0.12	excluded	absent	57.84	0.02	excluded	absent	12.86	-0.63	excluded	absent	118.67	0.89
209637_s_at	excluded	absent	41.01	-0.16	excluded	absent	48.12	-0.03	excluded	absent	77.22	0.52	excluded	absent	54.79	0.1	excluded	absent	69.76	0.38	excluded	absent	58.39	0.16	excluded	absent	90.29	0.77	excluded	absent	92.93	0.82	included	present	62.04	0.23	included	present	123.75	1.4	included	present	198.53	2.82	included	present	90.58	0.78	excluded	absent	30.65	-0.36	excluded	absent	33.8	-0.3	included	present	167.38	2.23
209638_x_at	included	present	194.98	-0.47	included	present	348.86	0.55	included	present	625.03	2.36	included	present	1036.64	5.07	included	present	470.71	1.35	included	present	174.3	-0.6	included	present	301.88	0.24	included	present	99.66	-1.09	included	present	249.65	-0.11	included	present	296.35	0.2	included	present	839.66	3.78	included	present	87.95	-1.17	included	present	430.05	1.08	included	present	570.17	2	included	present	131.49	-0.88
209639_s_at	excluded	absent	22.84	-0.77	excluded	absent	94.21	0.83	excluded	absent	38.93	-0.41	excluded	absent	44.56	-0.28	excluded	absent	8.74	-1.09	excluded	absent	12.63	-1	excluded	absent	54.46	-0.06	excluded	absent	65.31	0.18	excluded	absent	28.03	-0.65	excluded	absent	129.93	1.63	excluded	absent	19.28	-0.85	excluded	absent	100.26	0.96	excluded	absent	29.85	-0.61	excluded	absent	6.48	-1.14	excluded	absent	57.81	0.01
209640_at	included	present	152.74	0.5	excluded	absent	143.67	0.36	included	present	111.5	-0.13	excluded	absent	5.39	-1.75	excluded	absent	99.47	-0.31	included	present	575.84	6.94	included	present	235.44	1.76	included	present	552.24	6.58	included	present	271.67	2.31	included	present	192.4	1.1	included	present	167.63	0.72	included	present	199.24	1.21	excluded	absent	50.75	-1.06	included	present	48.72	-1.09	included	present	190.11	1.07
209641_s_at	excluded	absent	7.7	-0.53	excluded	absent	111.1	0.06	excluded	absent	165.28	0.36	excluded	absent	9.32	-0.52	excluded	absent	11.5	-0.51	excluded	absent	58.98	-0.24	excluded	absent	29.25	-0.41	excluded	absent	7.76	-0.53	excluded	absent	8.43	-0.53	excluded	absent	38.71	-0.35	excluded	absent	95.7	-0.03	excluded	absent	98.82	-0.01	excluded	absent	61.06	-0.23	excluded	absent	12.2	-0.5	excluded	absent	11.77	-0.51
209642_at	excluded	absent	75.68	-0.16	included	present	330.95	0.57	included	present	610.92	1.37	included	present	268.99	0.4	included	present	356.76	0.65	excluded	absent	100.78	-0.08	excluded	absent	32.44	-0.28	included	present	553.35	1.21	excluded	absent	72.09	-0.17	excluded	absent	68.22	-0.18	included	present	399.72	0.77	included	present	217.19	0.25	included	present	237.06	0.3	included	present	273.95	0.41	included	present	303.62	0.49
209643_s_at	excluded	absent	116.38	-0.1	excluded	absent	236.93	1.7	included	present	75.36	-0.71	excluded	absent	85.51	-0.56	excluded	absent	142.69	0.29	included	present	145.49	0.33	excluded	absent	130.45	0.11	included	present	158.99	0.53	included	present	166.31	0.64	excluded	absent	113.7	-0.14	included	present	194.25	1.06	excluded	absent	113.36	-0.15	excluded	absent	84.47	-0.58	excluded	absent	97.77	-0.38	excluded	absent	88.8	-0.51
209644_x_at	included	present	277.78	1.71	excluded	absent	50.25	-0.53	included	present	137.66	0.33	included	present	461.28	3.51	excluded	absent	174.72	0.69	included	present	264.47	1.57	excluded	absent	11.02	-0.91	included	present	369.62	2.61	included	present	277.93	1.71	included	present	176.16	0.71	included	present	468.27	3.57	included	present	554.97	4.42	included	present	142.65	0.38	included	present	205.58	1	included	present	157.59	0.53

[illegible]

209979_at	excluded	absent	29.54	-0.42	excluded	absent	24.84	-0.54	excluded	absent	42.2	[illegible]	[illegible]
209980_s_at	excluded	absent	58.01	-0.1	excluded	absent	52.32	-0.13	excluded	absent	10.34	[illegible]	[illegible]
209981_at	included	present	119.14	-0.15	excluded	absent	19.14	-0.5	excluded	absent	6.8	[illegible]	[illegible]
209982_s_at	excluded	absent	31.34	-0.67	included	present	211.41	1.31	excluded	absent	4.02	[illegible]	[illegible]
209983_s_at	included	present	378.1	-0.44	included	present	1181.15	0.5	included	present	327.82	[illegible]	[illegible]
209984_at	included	present	179.68	-2.32	included	absent	144.17	-2.66	included	present	226.06	[illegible]	[illegible]
209985_s_at	excluded	absent	5.83	-0.62	excluded	absent	182.26	1.81	included	present	71.72	[illegible]	[illegible]
209986_at	excluded	absent	13.05	-0.19	excluded	absent	32.53	0.21	included	present	254.05	[illegible]	[illegible]
209987_s_at	excluded	absent	47.99	0.16	excluded	absent	83.2	0.98	excluded	absent	85.27	[illegible]	[illegible]
209988_s_at	excluded	absent	114.17	-0.22	included	present	739.62	2.89	included	present	1014.43	[illegible]	[illegible]
209989_at	included	present	201.82	-0.5	included	present	432.51	2	included	present	355.95	[illegible]	[illegible]
209990_s_at	included	present	222.69	-0.48	excluded	absent	84.96	-0.55	excluded	absent	4.46	[illegible]	[illegible]
209991_x_at	excluded	absent	166.26	-0.42	excluded	absent	39.24	-0.56	excluded	absent	27.15	[illegible]	[illegible]
209992_at	included	present	89.97	-0.49	included	present	153.26	-0.34	included	absent	51.44	[illegible]	[illegible]
209993_at	included	present	561.59	-0.11	included	present	986.98	0.23	included	present	268.83	[illegible]	[illegible]
209994_s_at	included	present	736.36	-0.05	included	present	962.21	0.12	included	present	255.52	[illegible]	[illegible]
209995_s_at	excluded	absent	43.72	-0.12	excluded	absent	3.45	-0.38	excluded	absent	43.1	[illegible]	[illegible]
209996_x_at	included	present	4369.68	0.91	included	present	2493.57	-0.65	included	present	4583.39	[illegible]	[illegible]
209997_x_at	included	present	2881.22	0.78	included	present	1385.97	-1.16	included	present	3451.61	[illegible]	[illegible]
209998_at	included	present	962.36	1.01	included	present	883.61	0.76	included	present	1523.62	[illegible]	[illegible]
209999_x_at	excluded	absent	37.79	0.33	excluded	absent	11.71	-0.56	excluded	absent	12.03	[illegible]	[illegible]
210000_s_at	excluded	absent	2.61	-0.43	excluded	absent	36.45	1.29	excluded	absent	4.54	[illegible]	[illegible]
210001_s_at	excluded	absent	39.53	-0.76	excluded	absent	20.61	-1.02	excluded	absent	69.17	[illegible]	[illegible]
210002_at	excluded	absent	58.74	-0.55	included	present	112.25	-0.48	included	present	108.15	[illegible]	[illegible]
210004_at	included	present	160.27	-0.03	excluded	absent	20.61	-0.68	included	present	160.23	[illegible]	[illegible]
210005_at	included	present	272.34	0.31	included	present	1222.83	11.48	included	present	300.73	[illegible]	[illegible]
210006_at	included	present	286.25	2.38	excluded	absent	175.14	0.81	included	present	155.73	[illegible]	[illegible]
210007_s_at	included	present	1401.63	0.19	included	present	1093.47	-0.26	included	present	685.36	[illegible]	[illegible]
210008_s_at	excluded	absent	39.67	-0.05	excluded	absent	55.64	0.41	excluded	absent	76.38	[illegible]	[illegible]
210009_s_at	included	present	473.61	0.53	included	present	476.9	0.55	included	present	549.82	[illegible]	[illegible]
210010_s_at	included	present	140.67	0.11	included	present	292.72	2	excluded	absent	86.62	[illegible]	[illegible]
210011_s_at	included	absent	264.11	-0.79	included	present	347.53	-0.02	included	present	315.45	[illegible]	[illegible]
210012_s_at	included	present	193.37	-0.96	included	present	141.95	-1.31	included	present	232	[illegible]	[illegible]
210013_at	excluded	absent	4	-0.16	excluded	absent	3.86	-0.16	excluded	absent	58.84	[illegible]	[illegible]

[illegible]

210345_s_at	excluded	absent	7.35	-0.57	excluded	absent	[illegible]
210346_s_at	included	present	1514.74	-0.79	included	present	[illegible]
210347_s_at	included	absent	19.64	-0.99	included	present	[illegible]
210348_at	excluded	absent	70.42	-0.39	excluded	absent	[illegible]
210349_at	excluded	absent	115.97	-0.64	excluded	absent	[illegible]
210350_x_at	excluded	absent	83.03	0.38	excluded	absent	[illegible]
210352_at	excluded	absent	37.63	-0.21	excluded	absent	[illegible]
210353_s_at	excluded	absent	1.77	-1.28	included	present	[illegible]
210354_at	excluded	absent	32.54	-0.35	excluded	absent	[illegible]
210355_at	included	present	217.54	0.99	excluded	absent	[illegible]
210356_x_at	excluded	absent	43.84	-0.23	excluded	absent	[illegible]
210357_s_at	included	present	163.52	-0.43	included	absent	[illegible]
210358_x_at	excluded	absent	91.67	-0.18	included	present	[illegible]
210359_at	included	present	510.83	0.64	included	present	[illegible]
210360_s_at	included	present	1304.19	1.19	included	present	[illegible]
210361_s_at	included	present	467.49	-0.8	included	present	[illegible]
210362_x_at	excluded	absent	86.57	2.52	excluded	absent	[illegible]
210363_s_at	excluded	absent	7.82	-0.89	excluded	absent	[illegible]
210364_at	excluded	absent	9.63	-0.81	excluded	absent	[illegible]
210365_at	excluded	absent	109.89	0.19	excluded	absent	[illegible]
210366_at	excluded	absent	5.65	-0.19	excluded	absent	[illegible]
210367_s_at	included	present	1066.34	3.43	excluded	absent	[illegible]
210368_at	included	present	479.71	-0.51	included	absent	[illegible]
210369_at	included	present	156.16	1.6	excluded	absent	[illegible]
210370_s_at	excluded	absent	7.64	-0.37	excluded	absent	[illegible]
210371_s_at	included	present	3004.08	-0.51	included	present	[illegible]
210372_s_at	included	present	485.14	-0.31	included	absent	[illegible]
210373_at	excluded	absent	20.55	-1.24	excluded	absent	[illegible]
210374_x_at	excluded	absent	82.28	-0.33	included	present	[illegible]
210375_at	excluded	absent	91.11	-0.42	excluded	absent	[illegible]
210376_x_at	excluded	absent	103.19	-0.57	excluded	absent	[illegible]
210377_at	excluded	absent	4.76	-0.62	excluded	absent	[illegible]
210378_s_at	included	absent	206.82	-1.17	included	present	[illegible]
210379_s_at	included	present	1667.07	-0.75	included	present	[illegible]
210380_s_at	excluded	absent	2.96	-0.79	excluded	absent	[illegible]
210381_s_at	included	present	203.15	-0.07	included	present	[illegible]
210382_at	excluded	absent	11.9	-0.49	excluded	absent	[illegible]
210383_at	excluded	absent	35.2	-0.68	excluded	absent	[illegible]
210384_at	included	present	539.25	1.33	included	present	[illegible]
210385_s_at	included	present	324.2	-0.16	included	absent	[illegible]
210386_s_at	included	present	352.14	-0.18	included	present	[illegible]
210387_at	included	present	90	-0.33	included	present	[illegible]
210388_at	excluded	absent	60.98	0.42	excluded	absent	[illegible]
210389_x_at	included	present	223.08	0.32	included	present	[illegible]
210390_s_at	excluded	absent	3.01	-0.34	excluded	absent	[illegible]
210391_at	excluded	absent	2.02	-0.48	excluded	absent	[illegible]
210392_x_at	excluded	absent	44.64	-0.2	excluded	absent	[illegible]
210393_at	excluded	absent	73.46	-0.07	excluded	absent	[illegible]
210394_x_at	excluded	absent	77.23	-0.15	included	present	[illegible]
210395_x_at	excluded	absent	40.07	-0.18	excluded	absent	[illegible]
210396_s_at	included	present	1794.18	0.23	included	present	[illegible]
210397_at	excluded	absent	61.57	-0.3	excluded	absent	[illegible]
210398_x_at	excluded	absent	10.04	-0.28	excluded	absent	[illegible]
210399_x_at	excluded	absent	41.28	-0.48	excluded	absent	[illegible]
210400_at	excluded	absent	8.85	-0.86	excluded	absent	[illegible]
210401_at	excluded	absent	63.54	-0.65	excluded	absent	[illegible]
210402_at	excluded	absent	41.14	-0.16	excluded	absent	[illegible]
210403_s_at	excluded	absent	63.74	-0.18	excluded	absent	[illegible]
210404_x_at	included	present	416.04	-0.17	included	present	[illegible]
210405_x_at	included	present	546.29	1.81	excluded	absent	[illegible]
210406_s_at	included	present	10609.23	-0.15	included	present	[illegible]
210407_at	included	present	209.38	0.19	included	absent	[illegible]
210408_s_at	excluded	absent	88.92	0.22	excluded	absent	[illegible]
210409_at	excluded	absent	72.32	-0.1	included	present	[illegible]
210410_s_at	included	present	189.27	-0.47	included	present	[illegible]
210411_s_at	excluded	absent	58.13	-0.48	excluded	absent	[illegible]
210412_at	excluded	absent	12.78	-0.7	excluded	absent	[illegible]
210413_x_at	excluded	absent	26.12	-0.13	excluded	absent	[illegible]
210414_at	included	present	951.83	1.6	included	present	[illegible]
210415_s_at	included	present	463.91	-0.23	included	present	[illegible]
210416_s_at	included	present	143.25	0.6	included	present	[illegible]
210417_s_at	included	present	414.16	1.24	included	present	[illegible]
210418_s_at	included	present	941.32	-0.61	included	present	[illegible]
210419_at	excluded	absent	176.77	1.23	excluded	absent	[illegible]
210420_at	included	present	161.24	0.13	included	absent	[illegible]
210421_s_at	excluded	absent	26.49	-0.6	excluded	absent	[illegible]
210422_x_at	included	present	126.36	-0.66	included	present	[illegible]
210423_s_at	excluded	absent	6.26	-0.65	excluded	absent	[illegible]
210424_s_at	included	present	551.2	-1.08	included	present	[illegible]
210425_x_at	included	present	1209.84	-0.64	included	present	[illegible]
210426_x_at	included	present	642.39	-0.64	included	present	[illegible]
210427_x_at	included	present	17861.36	1.82	included	present	[illegible]
210428_s_at	included	present	320.66	0.89	included	present	[illegible]
210429_at	excluded	absent	185.73	0.41	excluded	absent	[illegible]
210430_x_at	excluded	absent	37.01	-0.68	excluded	absent	[illegible]
210431_at	excluded	absent	5.57	-0.59	excluded	absent	[illegible]
210432_s_at	included	present	1176.63	0.52	included	present	[illegible]
210433_at	excluded	absent	74.28	-0.14	included	present	[illegible]
210434_x_at	included	present	1201.03	-0.94	included	present	[illegible]
210435_at	included	present	256.34	-0.73	included	present	[illegible]
210436_at	included	present	246.38	0.46	included	absent	[illegible]
210437_at	included	present	426.16	1.3	included	present	[illegible]
210438_x_at	included	present	1496.07	0.37	included	present	[illegible]
210439_at	excluded	absent	32.72	-0.22	excluded	absent	[illegible]
210440_s_at	included	present	726.7	-0.31	included	present	[illegible]
210441_at	excluded	absent	37.29	-0.21	excluded	absent	[illegible]
210442_at	excluded	absent	88.71	-0.22	excluded	absent	[illegible]
210443_x_at	excluded	absent	99.61	0.79	excluded	absent	[illegible]
210444_at	included	absent	84.78	-0.28	included	absent	[illegible]
210445_at	included	present	903.37	0.23	included	present	[illegible]
210446_at	excluded	absent	58.91	0.27	excluded	absent	[illegible]
210447_at	excluded	absent	1.61	-0.44	excluded	absent	[illegible]
210448_s_at	included	present	236.45	-0.23	included	absent	[illegible]
210449_x_at	included	present	386.28	-0.91	included	present	[illegible]
210450_at	excluded	absent	4.23	-0.67	excluded	absent	[illegible]
210451_at	excluded	absent	5.18	-0.3	excluded	absent	[illegible]
210452_x_at	excluded	absent	44.28	-0.25	excluded	absent	[illegible]
210453_x_at	included	present	5104.73	-0.94	included	present	[illegible]
210454_s_at	excluded	absent	28.09	-0.51	excluded	absent	[illegible]
210455_at	included	present	261.34	-0.39	included	present	[illegible]
210456_at	included	present	500.6	0.54	included	present	[illegible]
210457_x_at	included	present	201.39	0.9	included	present	[illegible]
210458_s_at	included	present	1068.24	-0.64	included	present	[illegible]
210459_at	excluded	absent	26.36	-1.41	excluded	absent	[illegible]
210460_s_at	included	present	3013.06	0.92	included	present	[illegible]
210461_s_at	included	present	3293.73	-0.17	included	present	[illegible]
210462_at	included	present	91.78	0.26	excluded	absent	[illegible]
210463_x_at	excluded	absent	25.05	-0.71	excluded	absent	[illegible]
210464_at	excluded	absent	36.5	0.1	excluded	absent	[illegible]
210465_s_at	included	present	1296.52	-0.42	included	present	[illegible]
210466_s_at	included	present	3185.35	-0.57	included	present	[illegible]
210467_x_at	included	present	170.79	2.99	included	present	[illegible]
210469_at	excluded	absent	104.54	-0.29	excluded	absent	[illegible]
210470_x_at	included	present	2695.3	1.06	included	present	[illegible]
210471_s_at	included	present	283.89	-0.42	included	absent	[illegible]
210472_at	excluded	absent	6.11	-0.54	excluded	absent	[illegible]
210473_s_at	included	absent	111.84	-1.01	included	present	[illegible]
210474_s_at	excluded	absent	98.13	0.81	excluded	absent	[illegible]
210475_at	excluded	absent	111.5	0.6	excluded	absent	[illegible]
210476_s_at	excluded	absent	183.68	-0.35	excluded	absent	[illegible]
210477_x_at	included	present	429.85	-0.09	included	present	[illegible]
210479_s_at	included	present	716.53	-0.63	included	present	[illegible]
210480_s_at	included	present	737.9	-0.27	included	present	[illegible]
210481_s_at	excluded	absent	45.16	-0.35	excluded	absent	[illegible]
210482_x_at	included	present	416.05	2.24	included	present	[illegible]
210483_at	excluded	absent	54.1	-0.19	excluded	absent	[illegible]
210484_s_at	excluded	absent	79.64	0.36	excluded	absent	[illegible]
210486_at	excluded	absent	98.67	-0.59	excluded	absent	[illegible]
210487_at	excluded	absent	8.25	-0.28	excluded	absent	[illegible]
210491_at	included	present	1022.5	-0.06	included	present	[illegible]
210492_at	included	present	239.44	-0.46	included	present	[illegible]
210493_s_at	included	present	159.52	-0.05	included	absent	[illegible]
210495_x_at	included	present	20535.72	1.82	included	present	[illegible]
210496_at	excluded	absent	145.19	0.7	excluded	absent	[illegible]
210497_x_at	excluded	absent	86.59	-0.21	included	present	[illegible]
210498_at	included	present	277.15	0.61	included	present	[illegible]
210499_s_at	excluded	absent	154.38	-0.41	included	present	[illegible]
210500_at	excluded	absent	2.75	-1.13	excluded	absent	[illegible]
210501_x_at	included	present	2266.13	-0.33	included	present	[illegible]
210502_s_at	included	present	282.07	-0.11	included	present	[illegible]
210503_at	excluded	absent	59.39	0.91	included	present	[illegible]
210504_at	excluded	absent	2.6	-0.55	excluded	absent	[illegible]
210505_at	excluded	absent	10.68	-0.31	excluded	absent	[illegible]
210506_at	excluded	absent	118.01	2.3	excluded	absent	[illegible]
210507_s_at	included	present	197.07	-0.38	excluded	absent	[illegible]
210508_s_at	excluded	absent	4.72	-0.92	included	present	[illegible]
210510_s_at	included	present	1451.82	0.56	included	present	[illegible]
210511_s_at	included	present	455.56	0.03	included	present	[illegible]
210512_s_at	included	present	471.9	-0.74	included	absent	[illegible]
210513_s_at	included	present	478.66	-0.56	included	absent	[illegible]
210514_x_at	included	present	569.16	0.41	excluded	absent	[illegible]
210515_at	excluded	absent	12.78	-0.68	excluded	absent	[illegible]
210516_at	excluded	absent	63.3	-0.79	excluded	absent	[illegible]
210517_s_at	included	present	6630.58	2.67	included	present	[illegible]
210518_at	excluded	absent	53.55	-0.47	included	present	[illegible]
210519_s_at	included	present	692.86	-0.37	included	present	[illegible]
210520_at	excluded	absent	35.87	-0.34	included	present	[illegible]
210521_s_at	excluded	absent	35.88	-0.23	excluded	absent	[illegible]
210523_at	included	present	132.82	-0.25	included	present	[illegible]
210524_x_at	included	present	811.99	-0.57	included	present	[illegible]
210525_x_at	included	present	141.47	-0.39	included	absent	[illegible]
210527_x_at	included	present	686.33	-0.42	included	present	[illegible]
210528_at	included	present	102.64	0.4	excluded	absent	[illegible]
210529_s_at	included	present	1723.28	0.43	included	present	[illegible]
210530_s_at	included	absent	109.95	-0.78	included	present	[illegible]
210531_at	excluded	absent	12.04	-1.16	excluded	absent	[illegible]
210532_s_at	included	present	6521.71	-0.75	included	present	[illegible]
210533_at	excluded	absent	5.82	-0.44	excluded	absent	[illegible]
210534_s_at	included	present	129.19	-0.13	included	absent	[illegible]
210535_at	included	present	248.61	0.79	excluded	absent	[illegible]
210536_s_at	excluded	absent	5.76	-0.35	excluded	absent	[illegible]
210537_s_at	included	present	479.52	0.58	included	present	[illegible]
210538_s_at	included	present	360.92	-0.26	included	present	[illegible]
210539_at	excluded	absent	63.69	0.59	excluded	absent	[illegible]
210540_s_at	included	present	257	-0.68	included	present	[illegible]
210541_s_at	included	present	260.75	0.29	included	present	[illegible]
210542_s_at	excluded	absent	87.25	-0.49	included	present	[illegible]
210543_s_at	included	present	999.46	1.79	included	present	[illegible]
210544_s_at	included	present	1187.86	-0.15	included	present	[illegible]
210545_at	excluded	absent	72.44	-0.48	excluded	absent	[illegible]
210546_x_at	excluded	absent	14.45	-0.43	included	present	[illegible]
210547_x_at	included	absent	142.08	-0.92	included	present	[illegible]
210548_at	excluded	absent	2.34	-0.65	excluded	absent	[illegible]
210549_s_at	excluded	absent	22.43	-0.45	excluded	absent	[illegible]
210550_s_at	included	present	396.19	-0.06	excluded	absent	[illegible]
210551_s_at	excluded	absent	3.52	-0.85	excluded	absent	[illegible]
210552_s_at	excluded	absent	128.17	-0.96	excluded	absent	[illegible]
210553_x_at	excluded	absent	28.81	-0.5	excluded	absent	[illegible]
210554_s_at	included	present	2707.88	0.4	included	present	[illegible]
210555_s_at	included	present	345.58	-0.13	included	present	[illegible]
210556_at	excluded	absent	42.21	0	excluded	absent	[illegible]
210557_x_at	excluded	absent	101.74	-0.18	excluded	absent	[illegible]
210558_at	excluded	absent	14.45	-0.16	excluded	absent	[illegible]
210559_s_at	excluded	absent	59.44	-0.29	included	present	[illegible]
210560_at	excluded	absent	72.66	1.24	excluded	absent	[illegible]
210561_s_at	included	present	4743.35	-0.23	included	present	[illegible]
210562_at	excluded	absent	3.51	-0.62	excluded	absent	[illegible]
210563_x_at	included	present	1890.78	-0.43	included	present	[illegible]
210564_x_at	included	present	1985.2	-0.24	included	present	[illegible]
210565_at	excluded	absent	67.28	0.01	excluded	absent	[illegible]
210567_s_at	included	present	231.16	-0.35	included	present	[illegible]
210568_s_at	included	present	623.45	0.4	included	present	[illegible]
210569_s_at	excluded	absent	40.46	0.57	excluded	absent	[illegible]
210570_x_at	included	present	1530.7	0.07	included	present	[illegible]
210571_s_at	included	present	273.1	-0.37	included	present	[illegible]
210572_at	excluded	absent	20.64	-1	included	present	[illegible]
210573_s_at	included	present	264.98	-0.43	included	present	[illegible]
210574_s_at	included	present	3617.69	-0.53	included	present	[illegible]
210575_at	excluded	absent	30.44	0.19	excluded	absent	[illegible]
210576_at	excluded	absent	97.7	0.85	excluded	absent	[illegible]
210577_at	excluded	absent	4.5	-0.49	excluded	absent	[illegible]
210578_at	excluded	absent	48.13	1.37	excluded	absent	[illegible]
210579_s_at	excluded	absent	2.37	-0.88	excluded	absent	[illegible]
210580_x_at	included	present	1400.01	-0.41	included	present	[illegible]
210581_x_at	included	present	231.52	1.41	included	present	[illegible]
210582_s_at	included	present	190.43	-0.85	included	present	[illegible]
210583_at	excluded	absent	45.45	0.22	excluded	absent	[illegible]
210584_s_at	excluded	absent	1.45	-1.1	excluded	absent	[illegible]
210585_s_at	excluded	absent	231.61	1.16	excluded	absent	[illegible]
210586_x_at	excluded	absent	109.64	0.74	excluded	absent	[illegible]
210587_at	included	present	98.69	-0.11	included	present	[illegible]
210588_x_at	included	present	7602.28	-0.91	included	present	[illegible]
210589_s_at	included	present	697.14	1.14	included	present	[illegible]
210592_s_at	included	present	6863.6	-0.89	included	present	[illegible]
210593_at	included	present	293.53	-0.27	included	present	[illegible]
210594_x_at	included	present	552.35	0.24	included	present	[illegible]
210595_at	included	present	377.76	-0.13	included	absent	[illegible]
210596_at	included	present	4026.88	0.94	included	present	[illegible]
210597_x_at	excluded	absent	52.82	-0.29	excluded	absent	[illegible]
210598_at	included	present	97.58	0.63	excluded	absent	[illegible]
210599_at	excluded	absent	108.66	0.23	excluded	absent	[illegible]
210600_s_at	excluded	absent	85	-0.21	excluded	absent	[illegible]
210601_at	excluded	absent	43.11	0.09	excluded	absent	[illegible]
210602_s_at	included	present	817.57	10.39	excluded	absent	[illegible]
210603_at	excluded	absent	5.41	-0.52	excluded	absent	[illegible]
210604_at	included	present	183.01	0.88	excluded	absent	[illegible]
210605_s_at	included	present	1133.07	0.22	included	present	[illegible]
210606_x_at	excluded	absent	56.18	-0.33	excluded	absent	[illegible]
210607_at	excluded	absent	125.33	0.1	excluded	absent	[illegible]
210608_s_at	excluded	absent	71.06	-0.49	excluded	absent	[illegible]
210609_s_at	included	present	925.67	1	included	present	[illegible]
210610_at	excluded	absent	105.27	-0.19	excluded	absent	[illegible]
210611_s_at	excluded	absent	49.32	-0.52	excluded	absent	[illegible]
210612_s_at	included	present	198.74	-0.66	included	absent	[illegible]
210613_s_at	excluded	absent	285.84	0.41	excluded	absent	[illegible]
210614_at	excluded	absent	43.6	-0.35	excluded	absent	[illegible]
210615_at	excluded	absent	87.66	-0.63	excluded	absent	[illegible]
210616_s_at	included	present	4094.18	1.8	included	present	[illegible]
210617_at	excluded	absent	60.02	-0.54	excluded	absent	[illegible]
210618_at	excluded	absent	18.72	-0.92	excluded	absent	[illegible]
210619_s_at	included	present	215.49	-0.28	included	absent	[illegible]
210620_s_at	excluded	absent	128.87	0.63	excluded	absent	[illegible]
210621_s_at	included	present	727.71	-0.32	included	present	[illegible]
210622_x_at	excluded	absent	73.61	-0.36	excluded	absent	[illegible]
210623_at	excluded	absent	64.89	-1.2	excluded	absent	[illegible]
210624_s_at	included	present	112.75	-0.71	excluded	absent	[illegible]
210625_s_at	included	present	332.34	-0.3	included	present	[illegible]
210626_at	included	absent	132.64	-0.81	included	present	[illegible]
210627_s_at	excluded	absent	8.64	-1.68	excluded	absent	[illegible]
210628_x_at	included	present	311.42	2.13	included	present	[illegible]
210629_x_at	included	present	211.46	0.09	included	present	[illegible]
210630_s_at	included	present	123.49	0.19	excluded	absent	[illegible]
210631_at	included	absent	55.43	-1.26	included	absent	[illegible]
210632_s_at	excluded	absent	4.74	-0.48	excluded	absent	[illegible]
210633_x_at	included	present	479.06	-0.29	included	present	[illegible]
210634_at	excluded	absent	5.41	-1.13	excluded	absent	[illegible]
210635_s_at	included	present	311.44	-0.15	included	present	[illegible]
210636_at	excluded	absent	69.84	-0.04	excluded	absent	[illegible]
210637_at	excluded	absent	55.56	-0.21	excluded	absent	[illegible]
210638_s_at	included	present	2362.1	-0.77	included	present	[illegible]
210639_s_at	included	present	680.75	0.77	included	present	[illegible]
210640_s_at	excluded	absent	162.8	0.31	excluded	absent	[illegible]
210641_at	excluded	absent	2.4	-1.15	excluded	absent	[illegible]
210642_at	excluded	absent	13.54	-0.24	excluded	absent	[illegible]
210643_at	excluded	absent	1.75	-0.39	excluded	absent	[illegible]
210644_s_at	included	present	464.45	1.13	included	absent	[illegible]
210645_s_at	included	present	6667.34	0.45	included	present	[illegible]
210646_x_at	included	present	19232.45	-0.6	included	present	[illegible]
210647_x_at	included	absent	110.95	-0.89	included	absent	[illegible]
210648_x_at	included	present	3631.36	-1.16	included	present	[illegible]
210649_s_at	included	present	623.88	1.38	included	present	[illegible]
210650_s_at	excluded	absent	46.79	-0.61	included	present	[illegible]
210651_s_at	excluded	absent	11.16	-0.87	included	present	[illegible]
210652_s_at	included	present	476.96	1.76	included	present	[illegible]
210653_s_at	included	present	548.71	0.17	included	present	[illegible]
210654_at	excluded	absent	85.79	0.53	excluded	absent	[illegible]
210655_s_at	included	present	508.34	0.02	included	present	[illegible]
210656_at	included	present	161.87	-0.52	included	present	[illegible]
210657_s_at	included	present	876.4	-0.42	included	present	[illegible]
210658_s_at	included	present	565.19	-0.72	included	present	[illegible]
210659_at	included	present	137.59	-0.21	included	present	[illegible]
210660_at	included	present	239.72	1.24	excluded	absent	[illegible]
210661_at	included	present	214.14	0.78	excluded	absent	[illegible]
210662_at	excluded	absent	55.56	0.23	excluded	absent	[illegible]
210663_s_at	included	present	181.7	-0.28	included	present	[illegible]
210664_s_at	included	present	3325.96	0.48	included	present	[illegible]
210665_at	excluded	absent	24.62	-0.72	excluded	absent	[illegible]
210666_at	excluded	absent	35.12	-1.54	excluded	absent	[illegible]
210667_s_at	excluded	absent	40.83	-0.54	excluded	absent	[illegible]
210669_at	excluded	absent	95.84	-0.09	excluded	absent	[illegible]
210670_at	excluded	absent	3.46	-1.03	excluded	absent	[illegible]
210671_x_at	included	present	651.34	0.31	included	present	[illegible]
210672_s_at	excluded	absent	133.57	0.28	excluded	absent	[illegible]
210673_x_at	excluded	absent	20.12	-1.35	excluded	absent	[illegible]
210674_s_at	included	present	347.75	-0.06	included	present	[illegible]
210675_s_at	included	present	161.06	-0.47	included	present	[illegible]
210676_x_at	included	present	612.17	-0.51	included	present	[illegible]
210677_at	excluded	absent	49.57	0.28	excluded	absent	[illegible]
210678_s_at	excluded	absent	128.74	-0.08	excluded	absent	[illegible]
210679_x_at	included	present	4501.41	-0.04	included	present	[illegible]
210680_s_at	excluded	absent	16.92	-0.31	excluded	absent	[illegible]
210681_s_at	included	present	468.15	-0.72	included	present	[illegible]
210682_at	excluded	absent	41.42	-0.16	excluded	absent	[illegible]
210683_at	excluded	absent	37.02	0.18	excluded	absent	[illegible]
210684_s_at	included	present	1002.51	-0.4	included	present	[illegible]
210685_s_at	included	present	1532.01	0.39	included	present	[illegible]
210686_x_at	included	present	6634.59	-0.79	included	present	[illegible]
210687_at	excluded	absent	76.77	-0.6	excluded	absent	[illegible]
210688_s_at	included	present	473.49	1.25	included	present	[illegible]
210689_at	excluded	absent	67.75	0.07	excluded	absent	[illegible]
210690_at	excluded	absent	2.01	-1.33	excluded	absent	[illegible]
210691_s_at	included	present	2133.16	-0.5	included	present	[illegible]
210692_s_at	included	present	500.78	3.09	included	present	[illegible]
210693_at	excluded	absent	19.13	-0.46	excluded	absent	[illegible]
210694_s_at	included	present	1398.01	-0.41	included	present	[illegible]
210695_s_at	excluded	absent	56.16	-1.21	excluded	absent	[illegible]
210697_at	excluded	absent	41.39	-0.5	included	present	[illegible]
210698_at	included	present	204.8	1.16	included	present	[illegible]
210699_at	excluded	absent	70.59	-1.09	excluded	absent	[illegible]
210701_at	included	present	361.34	1.23	included	present	[illegible]
210702_s_at	excluded	absent	5.21	-0.7	excluded	absent	[illegible]
210703_at	excluded	absent	16.74	-0.72	excluded	absent	[illegible]
210704_at	included	present	123.04	1.77	excluded	absent	[illegible]
210705_s_at	included	present	642.97	0.95	included	present	[illegible]
210706_s_at	included	present	725.55	2.08	included	present	[illegible]
210707_x_at	included	present	2081.58	-0.32	included	present	[illegible]
210708_x_at	excluded	absent	156.78	-0.03	excluded	absent	[illegible]
210709_at	included	present	244.11	-0.68	included	absent	[illegible]
210710_at	excluded	absent	51.17	0.55	excluded	absent	[illegible]
210711_at	excluded	absent	23.11	-0.85	excluded	absent	[illegible]
210712_at	excluded	absent	51.37	-0.17	excluded	absent	[illegible]
210713_at	excluded	absent	13.04	-0.76	excluded	absent	[illegible]
210714_at	excluded	absent	135.12	-0.1	excluded	absent	[illegible]
210715_s_at	included	present	634.57	-0.53	included	present	[illegible]
210716_s_at	included	present	416.56	-0.06	included	present	[illegible]
210717_at	included	absent	131.83	-0.61	included	absent	[illegible]
210718_s_at	included	present	1061.57	-0.14	included	present	[illegible]
210719_s_at	included	present	178.92	0.45	included	absent	[illegible]
210720_s_at	excluded	absent	195.95	0.71	included	present	[illegible]

210721_s_at	excluded	absent	51.88	-0.41	included	present	234.01	0.88	excluded	absent	53.23	-0.4	excluded	absent	53.88	-0.4	included	present	219.45
210722_at	excluded	absent	4.72	-0.67	excluded	absent	2.56	-0.75	excluded	absent	33.3	0.32	excluded	absent	2.23	-0.76	excluded	absent	14.39
210723_x_at	included	present	182.21	0.99	excluded	absent	119.66	-0.16	excluded	absent	89.86	-0.7	excluded	absent	111.3	-0.31	excluded	absent	104.07
210724_at	excluded	absent	10.38	-0.81	excluded	absent	91.34	0.77	excluded	absent	3.87	-0.93	excluded	absent	68.55	0.33	excluded	absent	104.97
210726_at	excluded	absent	74.77	-0.29	excluded	absent	7.57	-1.45	excluded	absent	132.89	0.71	excluded	absent	10.78	-1.39	excluded	absent	56.86
210727_at	included	present	206.17	4.47	excluded	absent	11.23	-0.68	excluded	absent	7.35	-0.78	excluded	absent	5.74	-0.83	excluded	absent	2.99
210728_s_at	included	present	151.41	4.04	excluded	absent	4.53	-0.78	excluded	absent	7.64	-0.68	excluded	absent	36.59	0.27	excluded	absent	55.92
210729_at	excluded	absent	25.07	-0.22	excluded	absent	51.72	0.4	excluded	absent	1.45	-0.76	excluded	absent	9.07	-0.59	excluded	absent	109.82
210730_s_at	excluded	absent	61.54	0.65	excluded	absent	43.96	0.19	excluded	absent	44.82	0.21	excluded	absent	2.04	-0.89	excluded	absent	2.32
210731_s_at	included	present	71.3	-0.94	excluded	absent	70.16	-0.97	excluded	absent	87.94	-0.59	included	present	130.07	0.29	excluded	absent	48.86
210732_s_at	included	present	832.49	-0.07	included	present	557.11	-0.47	included	present	1040.31	0.23	included	present	1621.57	1.07	included	present	952.25
210733_at	included	present	2314.92	-0.3	included	present	1197.69	-0.88	included	present	2027.57	-0.45	included	present	2886.96	0	included	present	1468.92
210734_x_at	included	present	4255.69	1	included	present	4326.78	1.06	included	present	3736.56	0.51	included	present	3809.87	0.58	included	present	2231.5
210735_s_at	excluded	absent	18.81	-0.51	excluded	absent	3.19	-0.55	excluded	absent	27.33	-0.49	excluded	absent	48.45	-0.45	excluded	absent	51.38
210736_x_at	included	present	398.01	-0.73	included	present	910.21	-0.57	included	present	825.39	-0.6	included	present	2166.63	-0.18	included	present	1103.93
210737_at	included	present	302.85	0.95	excluded	absent	245.56	0.44	included	present	673.4	4.23	excluded	absent	7.83	-1.67	excluded	absent	213.34
210738_s_at	excluded	absent	5.26	-0.55	excluded	absent	3.18	-0.55	excluded	absent	83.17	-0.39	excluded	absent	5.42	-0.55	excluded	absent	72.82
210739_x_at	excluded	absent	123.23	-0.6	excluded	absent	90.56	-0.61	excluded	absent	580.27	-0.41	excluded	absent	72.74	-0.62	excluded	absent	4.56
210740_s_at	included	present	6169.81	-0.53	included	present	5097.09	-0.72	included	present	1980.84	-1.26	included	present	1764.67	-1.3	included	present	2828.98
210741_at	included	present	268.55	1.02	included	absent	78.39	-0.72	included	present	186.91	0.28	included	present	86.13	-0.64	included	present	186.07
210742_at	excluded	absent	38.03	-0.53	excluded	absent	45	-0.43	excluded	absent	30.32	-0.63	excluded	absent	5.59	-0.97	excluded	absent	4.39
210743_s_at	included	present	331.52	-0.36	included	present	311.44	-0.4	included	present	295.38	-0.44	included	present	167.36	-0.74	included	present	253.86
210744_s_at	excluded	absent	51.76	-0.95	excluded	absent	17.85	-1.68	excluded	absent	38.43	-1.23	excluded	absent	91.31	-0.09	included	present	208.93
210745_at	excluded	absent	33.1	-0.31	included	present	380.06	0.87	included	present	286.88	0.55	excluded	absent	91.03	-0.11	excluded	absent	21.76
210746_s_at	excluded	absent	69.91	0.35	excluded	absent	5.4	-0.88	excluded	absent	3.11	-0.92	excluded	absent	3.41	-0.91	excluded	absent	160.36
210747_at	excluded	absent	4.15	-0.53	excluded	absent	3.15	-0.6	excluded	absent	4.11	-0.53	excluded	absent	2.31	-0.66	excluded	absent	7.34
210748_at	included	present	492.42	-0.34	included	present	402.98	-0.98	included	present	403.82	-0.97	included	present	171.84	-2.62	included	present	740.36
210749_x_at	included	present	259.27	-0.96	included	present	374.59	-0.67	included	present	150.29	-1.23	included	present	1189.2	1.38	included	absent	99.7
210750_s_at	excluded	absent	38.68	-0.65	excluded	absent	4.07	-0.96	excluded	absent	7.83	-0.93	excluded	absent	11.96	-0.89	excluded	absent	121.87
210751_s_at	included	present	276.9	-0.46	included	present	155.11	-0.49	included	present	221.23	-0.47	included	present	176.09	-0.48	included	absent	68.57
210752_s_at	included	present	837.48	1.44	included	present	2628.97	7.25	included	present	3581.28	10.34	included	present	1377.49	3.19	included	present	1505.69
210753_s_at	excluded	absent	4.93	-1.16	excluded	absent	90.59	-0.36	excluded	absent	38.9	-0.84	excluded	absent	39.55	-0.84	excluded	absent	32.06
210754_s_at	included	present	231.24	-0.27	included	present	161.72	-0.44	included	present	172.73	-0.41	included	present	178.45	-0.4	included	present	368.72
210755_at	excluded	absent	84.89	0.72	excluded	absent	38.58	-0.38	excluded	absent	31.47	-0.54	excluded	absent	5.1	-1.17	excluded	absent	5.58

211098_x_at	included	present	286.87	-1.13	included	present	300.67	-1.03	included	present	514.75	0.47	included	present	496.13	0.34	included	present	395.64	-0.36	included	present	760	2.2	included	present	887.98	3.09	included	present	997.73	3.87	included	present	961.77	3.61	included	present	662.48	1.51	included	present	483.99	0.26	included	present	417.89	-0.21	included	present	224.39	-1.57	included	present	194.89	-1.77	included	present	657.93	1.48
211099_s_at	excluded	absent	69.97	0.54	excluded	absent	10.01	-0.7	excluded	absent	82.59	0.8	excluded	absent	6.24	-0.77	excluded	absent	9.81	-0.7	excluded	absent	12.36	-0.65	excluded	absent	117.59	1.51	excluded	absent	12.3	-0.65	included	present	259.44	4.43	included	present	412.46	7.57	excluded	absent	231.07	3.85	excluded	absent	217.77	3.57	excluded	absent	25.7	-0.37	excluded	absent	7.65	-0.74	excluded	absent	225.43	3.73
211100_x_at	excluded	absent	66.28	-0.13	excluded	absent	4.39	-0.84	excluded	absent	2.05	-0.86	excluded	absent	31.49	-0.53	excluded	absent	198.05	1.38	excluded	absent	209.45	1.52	excluded	absent	57.22	-0.23	excluded	absent	21.88	-0.64	included	present	187.6	1.27	excluded	absent	32.4	-0.52	excluded	absent	156.3	0.91	included	present	329.57	2.89	excluded	absent	8.92	-0.79	excluded	absent	29.6	-0.55	excluded	absent	124.02	0.54
211101_x_at	excluded	absent	4.23	-0.44	excluded	absent	4.99	-0.43	excluded	absent	57.24	0.23	excluded	absent	47.91	0.12	excluded	absent	7.97	-0.39	excluded	absent	41.47	0.04	excluded	absent	36.37	-0.03	excluded	absent	9.66	-0.37	excluded	absent	60.02	0.27	excluded	absent	9.33	-0.37	excluded	absent	6.1	-0.41	included	present	142.67	1.32	excluded	absent	36.09	-0.03	excluded	absent	8.25	-0.38	excluded	absent	70.7	0.4
211102_s_at	excluded	absent	15.49	-0.35	excluded	absent	40.77	0.26	excluded	absent	3.45	-0.64	excluded	absent	5.69	-0.59	excluded	absent	30.06	0	excluded	absent	7.32	-0.55	excluded	absent	3.16	-0.65	excluded	absent	3.38	-0.64	excluded	absent	9.84	-0.49	excluded	absent	7.31	-0.55	excluded	absent	4.18	-0.62	excluded	absent	87.25	1.38	excluded	absent	3.32	-0.64	excluded	absent	27.83	-0.05	excluded	absent	3.98	-0.63
211103_at	excluded	absent	12.54	-0.56	excluded	absent	89.31	0.02	excluded	absent	55.65	-0.24	excluded	absent	58.49	-0.21	excluded	absent	89.47	0.02	excluded	absent	136.62	0.38	included	present	108.44	0.16	excluded	absent	29.63	-0.43	included	present	88.78	0.02	included	present	111.8	0.19	excluded	absent	126.53	0.3	included	present	238.06	1.15	excluded	absent	118.03	0.24	included	present	204.04	0.89	included	present	103.27	0.12
211104_s_at	included	present	129.32	0.12	included	present	142.04	0.29	excluded	absent	76.89	-0.56	excluded	absent	49.16	-0.92	excluded	absent	123.14	0.04	excluded	absent	132.06	0.16	included	present	188.4	0.89	excluded	absent	84.88	-0.46	included	present	133.24	0.17	included	present	186.53	0.86	included	present	120.21	0	included	present	315.67	2.54	included	present	118.62	-0.02	excluded	absent	32.27	-1.14	included	present	46.31	-0.96
211105_s_at	included	present	461.7	1.04	included	absent	68.93	-1.15	included	present	107.32	-0.93	included	present	308.12	0.19	included	absent	71.8	-1.13	included	present	506.32	1.29	included	present	152.96	-0.68	included	present	169.14	-0.59	included	present	381.15	0.6	included	present	273.26	-0.01	included	present	140.68	-0.74	included	present	274.92	0	included	present	121.41	-0.85	included	present	116.1	-0.88	included	present	109.71	-0.92
211106_at	included	present	228.7	0.06	included	present	260.85	0.31	included	present	209.19	-0.09	included	present	315.34	0.73	included	present	324.26	0.8	included	absent	99.11	-0.94	included	present	223.29	0.02	included	absent	30.78	-1.46	included	present	115.33	-0.81	included	present	100.02	-0.93	included	absent	61.14	-1.23	included	present	286.79	0.51	included	present	692.2	3.64	included	present	486.89	2.06	included	present	62.82	-1.22
211107_s_at	excluded	absent	33.04	-0.33	included	present	898.58	1.32	excluded	absent	13.01	-0.36	included	present	157.26	-0.09	excluded	absent	189.64	-0.03	excluded	absent	169.35	-0.07	included	present	194.22	-0.02	included	present	297.49	0.18	included	present	189.93	-0.03	excluded	absent	38.19	-0.32	excluded	absent	67.38	-0.26	included	present	270.76	0.12	excluded	absent	8.38	-0.37	excluded	absent	61.86	-0.27	included	present	458.9	0.48
211108_s_at	excluded	absent	3.82	-0.43	excluded	absent	7.54	-0.32	excluded	absent	23.99	0.21	excluded	absent	43.45	0.82	excluded	absent	3.49	-0.44	excluded	absent	127.37	3.48	excluded	absent	36.37	0.6	excluded	absent	12.75	-0.15	excluded	absent	114.71	3.08	excluded	absent	23.66	0.2	excluded	absent	96.53	2.51	excluded	absent	25.89	0.27	excluded	absent	50.47	1.05	excluded	absent	3	-0.46	included	present	108.91	2.9
211109_at	excluded	absent	2.27	-0.64	excluded	absent	3.58	-0.55	excluded	absent	6.38	-0.37	excluded	absent	6.81	-0.35	excluded	absent	12.55	0.02	excluded	absent	46.35	2.18	excluded	absent	5.43	-0.44	excluded	absent	6.94	-0.34	excluded	absent	7.09	-0.33	excluded	absent	2.58	-0.62	excluded	absent	23.44	0.72	excluded	absent	75.98	4.08	excluded	absent	9.28	-0.19	excluded	absent	8.86	-0.22	excluded	absent	1.64	-0.68
211110_s_at	included	absent	48.22	-0.68	included	absent	9.4	-0.74	included	absent	73.12	-0.64	included	absent	2.98	-0.75	included	absent	42.26	-0.69	included	absent	100.4	-0.6	included	absent	2.15	-0.75	included	absent	1.33	-0.75	included	present	153.9	-0.52	included	present	149.11	-0.53	included	absent	18.07	-0.72	included	absent	2.43	-0.75	included	present	100.57	-0.6	included	absent	35.05	-0.7	included	absent	2.81	-0.75
211111_at	excluded	absent	118.86	1.13	excluded	absent	9.68	-0.94	excluded	absent	11.76	-0.9	excluded	absent	64.38	0.1	excluded	absent	145.25	1.63	excluded	absent	20.16	-0.74	included	present	722.34	12.58	excluded	absent	2.88	-1.07	excluded	absent	74.19	0.28	included	present	207.57	2.81	excluded	absent	12.43	-0.89	included	present	888.56	15.74	excluded	absent	297.22	4.51	included	present	582.68	9.93	included	present	548.7	9.29
211112_at	excluded	absent	6.59	-0.93	excluded	absent	21.61	-0.56	excluded	absent	108.99	1.56	excluded	absent	8.55	-0.88	excluded	absent	3.55	-1	excluded	absent	102.23	1.4	excluded	absent	51.85	0.17	excluded	absent	10.91	-0.82	excluded	absent	29.02	-0.38	excluded	absent	94.87	1.22	excluded	absent	13.44	-0.76	excluded	absent	40.82	-0.1	excluded	absent	117.34	1.77	excluded	absent	8.72	-0.88	excluded	absent	35.97	-0.21
211113_s_at	included	present	115.82	-0.62	included	present	128.97	-0.5	included	present	82.97	-0.91	included	present	317.33	1.17	included	present	207.53	0.2	included	present	295.25	0.97	included	present	334.54	1.32	excluded	absent	168.88	-0.15	included	present	206	0.18	included	present	253.85	0.61	included	present	278.32	0.82	included	present	146.92	-0.34	included	present	60.46	-1.11	included	present	83.5	-0.9	included	present	190.62	0.05
211114_x_at	included	present	296.41	0.44	included	present	653.71	4.51	included	present	323.41	0.75	included	present	551.67	3.35	included	present	463.13	2.34	included	present	198.53	-0.68	included	present	288.57	0.35	included	present	369.12	1.27	included	present	308.12	0.57	included	present	206.43	-0.59	included	present	297.09	0.45	included	present	120.34	-1.57	included	present	327.63	0.8	included	present	156.19	-1.16	included	present	338.13	0.92
211115_x_at	included	present	561.4	-0.66	included	present	2330.37	6.37	included	present	1124.81	1.58	included	present	1386.34	2.62	included	present	1062.04	1.33	included	present	667.84	-0.24	included	present	498.47	-0.91	included	present	898.97	0.68	included	present	529.69	-0.79	included	present	568.03	-0.63	included	present	666.4	-0.24	included	present	493.31	-0.93	included	present	672.48	-0.22	included	present	567.93	-0.63	included	present	665.96	-0.24
211116_at	excluded	absent	4.38	-0.79	excluded	absent	16.69	-0.71	excluded	absent	72.42	-0.33	excluded	absent	5.13	-0.79	excluded	absent	16.22	-0.71	excluded	absent	11.68	-0.74	excluded	absent	29.48	-0.62	included	present	207.8	0.58	excluded	absent	60.68	-0.41	excluded	absent	51.95	-0.47	excluded	absent	3.38	-0.8	excluded	absent	3.28	-0.8	excluded	absent	6.16	-0.78	excluded	absent	27.59	-0.64	excluded	absent	42.93	-0.53
211117_x_at	excluded	absent	81.83	-0.13	excluded	absent	8.1	-0.87	excluded	absent	62.48	-0.32	included	present	140.68	0.47	excluded	absent	109.25	0.15	excluded	absent	11.71	-0.84	excluded	absent	47.4	-0.48	excluded	absent	17.14	-0.78	excluded	absent	90.3	-0.04	excluded	absent	127.79	0.34	excluded	absent	105.02	0.11	excluded	absent	19.82	-0.76	excluded	absent	38.81	-0.56	excluded	absent	37.71	-0.57	excluded	absent	68.81	-0.26
211118_x_at	included	present	161.72	0.09	excluded	absent	128.79	-0.11	excluded	absent	10	-0.86	excluded	absent	97.64	-0.31	excluded	absent	14.74	-0.83	excluded	absent	87.07	-0.37	excluded	absent	146.53	0	excluded	absent	102.79	-0.28	excluded	absent	174.32	0.17	excluded	absent	126.52	-0.13	excluded	absent	201.78	0.35	excluded	absent	165.46	0.12	excluded	absent	70.65	-0.48	excluded	absent	123.03	-0.15	excluded	absent	96.87	-0.31
211119_at	included	present	166.81	0.63	excluded	absent	43.7	-0.65	excluded	absent	84.96	-0.22	included	present	146.48	0.42	excluded	absent	120.19	0.14	excluded	absent	65.33	-0.43	excluded	absent	5.33	-1.05	excluded	absent	38.99	-0.7	excluded	absent	102.29	-0.04	excluded	absent	56.89	-0.52	excluded	absent	143.16	0.38	excluded	absent	33.15	-0.76	excluded	absent	47.99	-0.61	excluded	absent	46.78	-0.62	excluded	absent	37.53	-0.72
211120_x_at	excluded	absent	75.28	-0.13	excluded	absent	43.81	-0.54	excluded	absent	32.01	-0.7	excluded	absent	8.92	-1.01	excluded	absent	7.41	-1.03	excluded	absent	106.05	0.28	excluded	absent	49.92	-0.46	excluded	absent	56.99	-0.37	excluded	absent	85.57	0.01	excluded	absent	47.9	-0.49	excluded	absent	47.61	-0.49	excluded	absent	17.25	-0.9	excluded	absent	4.69	-1.06	excluded	absent	7.5	-1.03	excluded	absent	6.55	-1.04
211121_s_at	excluded	absent	13.71	-1.19	excluded	absent	8.06	-1.3	excluded	absent	35.45	-0.73	excluded	absent	109.08	0.81	excluded	absent	8.96	-1.29	excluded	absent	93.47	0.48	excluded	absent	34.81	-0.75	excluded	absent	24.26	-0.97	excluded	absent	120.86	1.05	excluded	absent	114.22	0.92	excluded	absent	116.83	0.97	excluded	absent	90.66	0.42	excluded	absent	6.49	-1.34	excluded	absent	3.29	-1.4	excluded	absent	104.99	0.72
211122_s_at	included	present	87.74	-0.09	excluded	absent	4.8	-0.4	excluded	absent	42	-0.26	excluded	absent	12.08	-0.37	excluded	absent	3.44	-0.41	included	present	137.47	0.09	excluded	absent	0.29	-0.42	included	present	96.95	-0.06	excluded	absent	38.94	-0.27	excluded	absent	1.04	-0.41	excluded	absent	17.1	-0.35	excluded	absent	36.49	-0.28	excluded	absent	22.96	-0.33	included	present	110.89	-0.01	included	present	283.45	0.63
211123_at	excluded	absent	5.8	-1.31	excluded	absent	4.38	-1.34	excluded	absent	2.05	-1.39	excluded	absent	6.72	-1.29	excluded	absent	7.39	-1.27	excluded	absent	52.39	-0.25	excluded	absent	46.43	-0.38	included	present	82.61	0.44	excluded	absent	57.83	-0.12	excluded	absent	10.2	-1.21	excluded	absent	39.32	-0.55	excluded	absent	15.47	-1.09	excluded	absent	50.83	-0.28	excluded	absent	44.18	-0.43	excluded	absent	22.66	-0.92
211124_s_at	included	present	239.88	-0.69	included	absent	81.32	-1.23	included	present	419.59	-0.07	included	present	502.31	0.22	included	present	273.35	-0.57	included	present	273.02	-0.57	included	absent	52.5	-1.33	included	absent	69.22	-1.28	included	present	470.15	0.11	included	present	115	-1.12	included	present	74.65	-1.26	included	present	106.75	-1.15	included	present	195.4	-0.84	included	present	272.9	-0.57	included	absent	66.05	-1.29
211125_x_at	excluded	absent	85.83	-0.47	excluded	absent	104.33	-0.31	included	present	94.31	-0.4	excluded	absent	7.74	-1.14	excluded	absent	64.01	-0.66	excluded	absent	89.98	-0.43	excluded	absent	119.49	-0.18	excluded	absent	75.1	-0.56	excluded	absent	113.23	-0.23	excluded	absent	81.4	-0.51	excluded	absent	72.46	-0.58	excluded	absent	65.92	-0.64	excluded	absent	70.96	-0.6	excluded	absent	10.66	-1.11	excluded	absent	110.56	-0.26
211126_s_at	included	present	540.06	-0.69	included	present	825.27	-0.03	included	present	684.85	-0.35	included	present	592.19	-0.57	included	present	850.64	0.03	included	present	1476.56	1.48	included	present	244.84	-1.38	included	present	621.33	-0.5	included	present	1003.78	0.39	included	present	505.09	-0.77	included	present	1540.01	1.63	included	present	833.68	-0.01	included	present	375.6	-1.07	included	present	972.8	0.31	included	present	813.95	-0.05
211127_x_at	excluded	absent	11.46	-1.27	excluded	absent	56.11	-0.31	excluded	absent	13.06	-1.24	excluded	absent	74.8	0.09	included	present	155.75	1.84	excluded	absent	60.55	-0.21	excluded	absent	40.6	-0.64	excluded	absent	14.48	-1.21	included	present	85.85	0.33	excluded	absent	24.41	-0.99	excluded	absent	9.38	-1.32	included	present	162.25	1.98	included	present	71.38	0.02	excluded	absent	36.02	-0.74	included	present	32.74	-0.81
211128_at	excluded	absent	3.2	-0.56	excluded	absent	14.45	-0.21	excluded	absent	17.16	-0.12	excluded	absent	2.46	-0.59	excluded	absent	9.08	-0.38	excluded	absent	5.8	-0.48	excluded	absent	5.53	-0.49	excluded	absent	65.06	1.38	excluded	absent	30.27	0.29	excluded	absent	23.97	0.09	excluded	absent	9.31	-0.37	excluded	absent	42.05	0.66	excluded	absent	4.89	-0.51	excluded	absent	6.5	-0.46	excluded	absent	4.6	-0.52
211129_x_at	included	present	121.96	-0.42	included	present	188.94	0.3	excluded	absent	1.54	-1.72	included	present	106.26	-0.59	excluded	absent	154.36	-0.07	excluded	absent	164.43	0.04	excluded	absent	100.33	-0.65	excluded	absent	126.56	-0.37	included	present	115.67	-0.49	excluded	absent	99.08	-0.67	included	present	109.62	-0.55	excluded	absent	187.85	0.29	excluded	absent	50.14	-1.19	excluded	absent	29.25	-1.42	included	present	69.47	-0.99
211130_x_at	excluded	absent	175.49	-0.02	excluded	absent	95.57	-0.76	excluded	absent	96.65	-0.75	included	present	136.39	-0.38	included	present	205.53	0.26	excluded	absent	208.43	0.29	included	present	162.52	-0.14	included	present	122.7	-0.51	included	present	158.52	-0.18	included	present	206.23	0.27	included	present	167.87	-0.09	included	present	418.37	2.25	included	present	206.47	0.27	excluded	absent	137.93	-0.37	included	present	134.7	-0.4
211131_s_at	excluded	absent	2.3	-0.99	excluded	absent	0.71	-1.04	excluded	absent	38.83	0.13	excluded	absent	14.11	-0.63	excluded	absent	11.84	-0.7	excluded	absent	75.64	1.27	excluded	absent	42.26	0.24	excluded	absent	16.74	-0.55	excluded	absent	27.79	-0.21	excluded	absent	14.84	-0.61	excluded	absent	5.96	-0.88	excluded	absent	4.53	-0.93	excluded	absent	48.45	0.43	excluded	absent	1.84	-1.01	excluded	absent	0.19	-1.06
211132_at	included	present	199.36	-0.34	included	absent	150.38	-0.85	included	present	277.59	0.48	included	present	200.88	-0.32	included	present	358.45	1.32	included	present	666.22	4.53	included	present	541.75	3.23	included	present	731.45	5.21	included	present	663.17	4.5	included	present	719.49	5.08	included	present	374.33	1.49	included	absent	137.71	-0.98	included	present	239.89	0.09	included	present	158.17	-0.77	included	present	451.62	2.29
211133_x_at	excluded	absent	83.43	-0.79	included	present	338.97	0.62	excluded	absent	55.11	-0.95	excluded	absent	39.31	-1.04	excluded	absent	125.97	-0.56	included	present	564.96	1.88	excluded	absent	141.38	-0.47	included	present	102.03	-0.69	included	present	374.31	0.82	included	present	156.83	-0.39	included	present	385.12	0.88	included	present	619.3	2.18	included	present	194.5	-0.18	excluded	absent	135.12	-0.51	included	present	372.58	0.81
211135_x_at	included	present	169.58	0.06	included	present	306.62	1.12	excluded	absent	73.95	-0.67	excluded	absent	128.18	-0.26	included	present	250.61	0.69	included	present	545.65	2.96	excluded	absent	220.58	0.46	excluded	absent	100.86	-0.47	included	present	291.17	1	excluded	absent	170.24	0.07	included	present	213.82	0.4	included	present	443.94	2.18	included	present	220.41	0.46	included	present	148.87	-0.1	included	present	337.54	1.36
211136_s_at	excluded	absent	204.57	0.19	included	present	283.16	1.03	excluded	absent	83.46	-1.11	included	present	215.46	0.3	included	present	212.33	0.27	included	present	352.51	1.77	included	present	477.01	3.1	included	present	874.54	7.35	included	present	469.33	3.02	included	present	614.44	4.57	included	present	351.47	1.76	included	present	276.7	0.96	included	absent	63.92	-1.32	included	present	152.82	-0.37	included	present	712.54	5.62
211137_s_at	included	present	1365.5	0.21	included	present	1293.09	0.08	included	present	1586.73	0.6	included	present	864.14	-0.67	included	present	1077.72	-0.29	included	present	520.22	-1.27	included	present	2815.55	2.74	included	present	779.61	-0.81	included	present	1925.59	1.19	included	present	1572.07	0.57	included	present	2065.52	1.43	included	present	2182.01	1.63	included	present	3250.51	3.5	included	present	2573.61	2.32	included	present	1788.14	0.95
211138_s_at	excluded	absent	96.67	-0.23	included	present	179	-0.06	excluded	absent	65.42	-0.29	excluded	absent	85.16	-0.25	excluded	absent	199.08	-0.02	excluded	absent	55.58	-0.31	excluded	absent	29.81	-0.36	excluded	absent	78.93	-0.26	included	present	177.26	-0.06	excluded	absent	2.52	-0.42	included	present	78.25	-0.26	included	present	405.85	0.4	included	present	121.62	-0.18	included	present	185.08	-0.05	included	present	173.67	-0.07
211139_s_at	included	present	2338.37	0.91	included	present	1017.74	-0.72	included	present	1366.72	-0.29	included	present	3210.99	1.99	included	present	1728.95	0.16	included	present	1062.96	-0.67	included	present	469.68	-1.4	included	present	1433.42	-0.21	included	present	1607.7	0.01	included	present	978.28	-0.77	included	present	1060.95	-0.67	included	present	953.88	-0.8	included	present	2303.22	0.87	included	present	2304.3	0.87	included	present	1140.51	-0.57
211140_s_at	included	present	324	0.72	excluded	absent	303.12	0.59	included	present	477.04	1.66	included	present	657.39	2.78	included	present	488.45	1.74	excluded	absent	137.33	-0.43	included	present	192.26	-0.09	included	present	485.17	1.71	included	present	389.2	1.12	included	present	278.48	0.44	included	present	1375.12	7.2	excluded	absent	313.46	0.66	excluded	absent	25.38	-1.12	excluded	absent	94.35	-0.69	included	present	562.22	2.19

[illegible]

211494_s_at	included	present	251.36	-0.65	included	absent	2.33	-0.72	included	present	1178.86	-0.41	included	present	275.05	-0.64	included	absent	81.6	-0.7	included	present	182.98	-0.67	included	absent	51.4	-0.7	included	present	785.73
211495_x_at	excluded	absent	161.55	0.07	included	absent	71.39	-0.69	included	absent	60.57	-0.78	included	absent	13.14	-1.18	included	absent	140.92	-0.11	included	present	172.59	0.16	included	absent	57.62	-0.81	included	absent	69.7
211496_s_at	included	present	114.51	-0.4	included	absent	6.42	-1.45	included	present	127.37	-0.28	included	present	144.65	-0.11	included	absent	126.41	-0.28	included	absent	80.32	-0.73	included	present	195.07	0.38	included	absent	21.14
211497_x_at	excluded	absent	2.28	-0.55	excluded	absent	3.9	-0.54	excluded	absent	10.03	-0.48	excluded	absent	56.02	-0.08	excluded	absent	11.18	-0.47	excluded	absent	51.54	-0.12	excluded	absent	1.05	-0.56	excluded	absent	51.64
211498_s_at	excluded	absent	2.91	-0.32	excluded	absent	23.33	-0.12	excluded	absent	19.93	-0.15	excluded	absent	2.9	-0.32	excluded	absent	36.88	0.01	excluded	absent	16.68	-0.18	excluded	absent	5.03	-0.3	excluded	absent	6.51
211499_s_at	excluded	absent	131.69	-0.39	included	present	547.31	2.6	excluded	absent	40.83	-1.05	excluded	absent	1.41	-1.33	excluded	absent	87.72	-0.71	included	present	585.36	2.87	included	present	253.29	0.48	included	present	338.87
211500_at	excluded	absent	95.68	-0.05	excluded	absent	14.4	-1.24	excluded	absent	56.14	-0.63	excluded	absent	77.16	-0.32	excluded	absent	60.93	-0.56	excluded	absent	103.05	0.06	excluded	absent	69.65	-0.43	excluded	absent	24.11
211501_s_at	included	present	963.5	-0.24	included	present	2105.5	2.25	included	present	1533.61	1	included	present	992.18	-0.18	included	present	1261.84	0.41	included	present	1376.46	0.66	included	present	1164.19	0.19	included	present	1726.07
211502_s_at	included	present	798.46	0.9	included	present	408.59	-0.21	included	present	106.29	-1.07	included	present	657.71	0.5	included	present	487.18	0.01	included	present	422.39	-0.17	included	present	212.7	-0.77	included	present	246.91
211503_s_at	included	present	3467.93	1.61	included	present	1928.4	-1.07	included	present	5976.75	5.98	included	present	3276.51	1.28	included	present	4628.37	3.63	included	present	1841.02	-1.22	included	present	2151.25	-0.68	included	present	2812.25
211504_x_at	included	present	392.5	-0.32	included	absent	315.49	-0.75	included	present	365.64	-0.47	included	present	199.24	-1.4	included	absent	245.58	-1.14	included	absent	371.02	-0.44	included	present	379.89	-0.39	included	present	192.63
211505_s_at	included	present	802.5	-0.71	included	present	1091.03	-0.04	included	present	1067.78	-0.1	included	present	950.67	-0.37	included	present	1168.3	0.14	included	present	1088	-0.05	included	present	1211.22	0.24	included	present	1571.39
211506_s_at	excluded	absent	27.69	-0.43	excluded	absent	66.63	-0.32	included	present	434.67	0.74	included	present	76.93	-0.29	excluded	absent	245.58	0.2	excluded	absent	82.75	-0.27	excluded	absent	11.03	-0.48	excluded	absent	71.86
211507_s_at	included	present	123.5	-1.02	included	absent	64.18	-1.68	included	present	159.49	-0.62	included	present	186.05	-0.33	included	absent	167.16	-0.54	included	present	251.49	0.4	included	present	327.99	1.25	included	present	301.85
211508_s_at	excluded	absent	0.76	-0.81	excluded	absent	3.98	-0.68	excluded	absent	67.75	1.85	excluded	absent	1.93	-0.77	excluded	absent	2.1	-0.76	excluded	absent	76.94	2.22	excluded	absent	8.17	-0.52	excluded	absent	1.08
211509_s_at	included	present	20072.32	-0.49	included	present	22131.41	-0.22	included	present	15665.9	-1.09	included	present	10258.95	-1.83	included	present	15126.16	-1.17	included	present	11183.51	-1.7	included	present	18281.18	-0.74	included	present	8763.61
211510_s_at	excluded	absent	78.17	1.11	excluded	absent	16.37	-0.55	excluded	absent	46.8	0.27	excluded	absent	93.3	1.52	excluded	absent	50.24	0.36	excluded	absent	120.44	2.25	excluded	absent	28.45	-0.22	excluded	absent	17.79
211512_s_at	excluded	absent	19.02	-0.48	excluded	absent	12.66	-0.64	excluded	absent	12.57	-0.64	excluded	absent	24.29	-0.34	excluded	absent	9.28	-0.73	excluded	absent	134.44	2.5	excluded	absent	118.46	2.09	excluded	absent	64.65
211513_s_at	excluded	absent	58.92	-0.4	excluded	absent	60.97	-0.37	excluded	absent	49.02	-0.53	excluded	absent	6.33	-1.1	excluded	absent	101.77	0.18	excluded	absent	160.65	0.97	excluded	absent	117.04	0.38	excluded	absent	131.96
211514_at	included	present	150.4	0.73	excluded	absent	62.43	-0.85	excluded	absent	87.72	-0.4	excluded	absent	71.18	-0.7	excluded	absent	108.87	-0.02	excluded	absent	22.23	-1.58	included	present	89.38	-0.37	excluded	absent	52.28
211515_s_at	excluded	absent	106.75	0.37	excluded	absent	40.22	-0.9	excluded	absent	90.63	0.06	excluded	absent	61.88	-0.48	excluded	absent	143.86	1.07	excluded	absent	19.73	-1.28	included	present	103.62	0.31	included	present	100.06
211516_at	excluded	absent	33.77	0.18	excluded	absent	8.86	-0.9	excluded	absent	25.12	-0.2	excluded	absent	36.52	0.3	excluded	absent	5.01	-1.07	excluded	absent	37.64	0.35	excluded	absent	14.72	-0.65	excluded	absent	2.38
211517_s_at	excluded	absent	26.66	-0.26	excluded	absent	2.75	-0.88	excluded	absent	5.26	-0.81	excluded	absent	73.37	0.94	excluded	absent	21.18	-0.4	excluded	absent	3.39	-0.86	excluded	absent	20.25	-0.43	excluded	absent	54.95
211518_s_at	excluded	absent	104.45	-0.17	excluded	absent	189.51	0.44	excluded	absent	63.42	-0.46	excluded	absent	3.21	-0.89	excluded	absent	117.1	-0.08	excluded	absent	220.08	0.65	excluded	absent	8.07	-0.86	excluded	absent	101.99
211519_s_at	excluded	absent	72.19	-0.13	included	present	171.46	0.08	excluded	absent	140.42	0.01	excluded	absent	161.81	0.06	excluded	absent	129.43	-0.01	excluded	absent	5.72	-0.27	excluded	absent	70.75	-0.13	excluded	absent	237.98
211520_s_at	included	present	229.2	-0.24	excluded	absent	172.2	-0.32	excluded	absent	6.47	-0.56	included	present	199.56	-0.28	excluded	absent	14.47	-0.55	excluded	absent	5.24	-0.56	included	present	746.66	0.52	excluded	absent	23.51
211521_s_at	excluded	absent	83.94	1.2	excluded	absent	105.41	1.75	excluded	absent	55.24	0.45	excluded	absent	7.23	-0.79	excluded	absent	35.64	-0.06	excluded	absent	8.42	-0.76	excluded	absent	9.42	-0.74	excluded	absent	22.95
211522_s_at	excluded	absent	81.98	0.02	excluded	absent	72.55	-0.12	included	present	120.09	0.59	excluded	absent	7.05	-1.1	excluded	absent	126.2	0.68	excluded	absent	87.24	0.1	excluded	absent	64.31	-0.24	excluded	absent	16.94
211523_at	excluded	absent	43.31	-0.16	excluded	absent	35.35	-0.33	excluded	absent	72.87	0.48	excluded	absent	35.13	-0.34	excluded	absent	101.02	1.08	excluded	absent	52.04	0.03	excluded	absent	59.79	0.19	excluded	absent	18.93
211524_at	excluded	absent	15.47	-0.9	excluded	absent	91.95	1.33	excluded	absent	49.91	0.1	excluded	absent	17.55	-0.84	excluded	absent	8.65	-1.1	excluded	absent	62.35	0.46	excluded	absent	48.83	0.07	excluded	absent	9.01
211525_s_at	excluded	absent	1.63	-0.72	excluded	absent	1.91	-0.7	excluded	absent	1.37	-0.73	excluded	absent	1.27	-0.73	excluded	absent	64.21	2.23	excluded	absent	2.05	-0.7	excluded	absent	39.53	1.07	excluded	absent	1.54
211526_s_at	excluded	absent	6.25	-1.25	excluded	absent	60.21	0.02	excluded	absent	43.32	-0.38	excluded	absent	3.14	-1.32	excluded	absent	35.27	-0.57	excluded	absent	39.32	-0.47	excluded	absent	61.18	0.04	excluded	absent	54.06
211527_x_at	included	present	714.49	-0.7	included	absent	226.03	-0.86	included	present	502.08	-0.77	included	present	2144.65	-0.24	included	present	2718.44	-0.05	included	present	500.58	-0.77	included	present	1745.79	-0.37	included	present	756.45
211528_x_at	included	present	1529.79	0.11	included	present	957.21	-0.24	included	present	402.21	-0.58	included	present	878.45	-0.29	included	present	715.98	-0.39	included	present	8233.26	4.17	included	present	1089.23	-0.16	included	present	1018.4
211529_x_at	included	present	728.37	-0.08	included	present	525.96	-0.32	included	present	54.8	-0.87	included	present	624.05	-0.2	included	present	462.94	-0.39	included	present	4928.88	4.85	included	present	553.65	-0.28	included	present	394.41
211530_x_at	excluded	absent	239.62	0.98	excluded	absent	32.87	-0.77	excluded	absent	95.69	-0.24	excluded	absent	90.91	-0.28	excluded	absent	34.75	-0.76	included	present	1072.83	8.04	excluded	absent	153.49	0.25	included	present	183.26
211531_x_at	excluded	absent	66.37	-0.24	excluded	absent	72.74	-0.23	excluded	absent	75.24	-0.23	included	present	65.44	-0.24	excluded	absent	106.67	-0.19	included	present	32.36	-0.28	excluded	absent	68.34	-0.24	excluded	absent	9.62
211532_x_at	excluded	absent	18.98	-0.54	excluded	absent	238.11	3.56	excluded	absent	40.6	-0.13	excluded	absent	5.79	-0.78	excluded	absent	12.24	-0.66	excluded	absent	7.61	-0.75	excluded	absent	58	0.19	excluded	absent	64.37
211533_at	excluded	absent	3.27	-0.63	excluded	absent	43.65	1.22	excluded	absent	35.61	0.85	excluded	absent	5.24	-0.54	excluded	absent	54.28	1.71	excluded	absent	5.35	-0.53	excluded	absent	3.78	-0.6	excluded	absent	21.58
211534_x_at	included	present	866.47	0.03	included	present	1845.64	1.16	included	present	260.07	-0.68	included	present	1648.65	0.93	included	present	197.05	-0.75	included	present	240.02	-0.7	included	present	2817.41	2.29	included	present	1022.52
211535_s_at	included	present	257.97	0.25	included	absent	134.04	-0.75	included	absent	85.75	-1.14	included	present	172.81	-0.44	included	absent	184.61	-0.35	included	present	817.71	4.76	included	present	215.29	-0.1	included	present	274.78
211536_x_at	included	present	1264.43	-0.56	included	present	2702.34	2.69	included	present	1888.22	0.85	included	present	1853.96	0.77	included	present	2029.95	1.17	included	present	620.09	-2.02	included	present	751.7	-1.72	included	present	1613.75
211537_x_at	included	present	1686.58	0.41	included	present	3413.29	4.25	included	present	2049.11	1.22	included	present	1532.72	0.07	included	present	2342.68	1.87	included	present	724.8	-1.72	included	present	786.91	-1.59	included	present	1604.46
211538_s_at	included	present	292.92	-0.91	included	present	4408.34	0	included	present	264.93	-0.92	included	present	1746.41	-0.59	included	present	335.83	-0.9	included	present	2101.25	-0.51	included	present	267.81	-0.92	included	present	1245.51
211540_s_at	included	present	1591.99	2.93	included	present	647.72	-0.65	included	present	1577.33	2.87	included	present	722.03	-0.37	included	present	1613.45	3.01	included	present	1054.99	0.89	included	present	1031.41	0.8	included	present	1937.66
211541_s_at	included	present	787.85	-0.4	included	present	953.37	0.07	included	present	1082.97	0.43	included	present	1410.93	1.35	included	present	1149.91	0.62	included	present	734.9	-0.54	included	present	984.05	0.16	included	present	653.31
211542_x_at	included	present	7264.44	-0.23	included	present	10384.34	0.41	included	present	7293.65	-0.22	included	present	11258.68	0.59	included	present	5716.37	-0.54	included	present	15685.71	1.49	included	present	16425.38	1.64	included	present	18931.93
211543_s_at	excluded	absent	125.62	-0.12	excluded	absent	127.46	-0.09	excluded	absent	67.51	-0.93	excluded	absent	21.69	-1.57	excluded	absent	185.09	0.72	excluded	absent	157.7	0.33	excluded	absent	116.27	-0.25	excluded	absent	134.58
211544_s_at	excluded	absent	61.82	-0.09	excluded	absent	105.01	-0.01	excluded	absent	27.87	-0.14	excluded	absent	2.06	-0.19	excluded	absent	4.66	-0.18	excluded	absent	6.49	-0.18	excluded	absent	25.29	-0.15	excluded	absent	8.77
211545_at	excluded	absent	5.85	-0.45	excluded	absent	32.93	-0.1	excluded	absent	3.88	-0.47	excluded	absent	2.26	-0.49	excluded	absent	56.31	0.2	excluded	absent	20.39	-0.26	excluded	absent	80.04	0.51	excluded	absent	11.63
211546_x_at	included	present	6492.84	0.96	included	present	3728.95	0.16	included	absent	169.66	-0.86	included	absent	58.65	-0.9	included	present	1989.98	-0.34	included	present	663.85	-0.72	included	present	1381.97	-0.51	included	present	738.02
211547_s_at	included	present	1113.46	1.46	included	present	345.07	-1.22	included	present	952.3	0.89	included	present	627.57	-0.24	included	present	1367.02	2.34	included	present	621.3	-0.26	included	present	1496.67	2.79	included	present	793.48
211548_s_at	included	present	267.3	-0.22	excluded	absent	66.97	-0.52	excluded	absent	32.36	-0.57	excluded	absent	3.61	-0.61	excluded	absent	63.71	-0.52	excluded	absent	71.54	-0.51	excluded	absent	55.91	-0.53	included	present	258.32
211549_s_at	excluded	absent	174.45	-0.1	excluded	absent	70.55	-0.58	excluded	absent	12.13	-0.86	excluded	absent	3.33	-0.9	excluded	absent	124.78	-0.33	excluded	absent	129.56	-0.31	excluded	absent	60.27	-0.63	included	present	185.01
211550_at	excluded	absent	13.24	-0.97	excluded	absent	3.34	-1.15	excluded	absent	8.19	-1.06	excluded	absent	33.67	-0.61	excluded	absent	68.67	0	excluded	absent	101.06	0.57	excluded	absent	6.94	-1.08	excluded	absent	46.88
211551_at	included	present	154.34	1.68	excluded	absent	14.06	-1.43	excluded	absent	85.29	0.15	excluded	absent	39.26	-0.87	excluded	absent	68.87	-0.21	excluded	absent	54.63	-0.53	included	present	129.43	1.13	excluded	absent	71.33
211552_s_at	included	present	303.14	-0.2	included	present	202.35	-0.33	included	present	143.55	-0.4	included	absent	52.29	-0.52	included	present	166.12	-0.37	included	present	464.37	0	included	present	170.88	-0.37	included	present	376.26
211553_x_at	excluded	absent	81.86	0.05	excluded	absent	28.92	-1.03	excluded	absent	118.97	0.8	included	present	125.58	0.94	included	present	221.43	2.89	excluded	absent	98.07	0.38	excluded	absent	67.19	-0.25	included	present	90.49
[illegible]																															

211871_x_at	included	present	244.52	0.05	included	present	269.43	0.22	included	present	160.76	-0.53	included	present	394.41	1.08	included	present	358.29	0.83	included	absent	169.13	-0.47	included	present	489.18	1.73	included	present	388.62	1.04
211872_s_at	excluded	absent	37.66	0.17	excluded	absent	2.9	-0.7	excluded	absent	3.6	-0.68	excluded	absent	7.03	-0.6	excluded	absent	16.18	-0.37	excluded	absent	62.95	0.81	excluded	absent	153.19	3.07	excluded	absent	11.64	-0.48
211873_s_at	included	present	239.84	2.14	included	present	111.09	0.21	excluded	absent	3.94	-1.4	excluded	absent	68.11	-0.44	excluded	absent	149.1	0.78	included	present	157.72	0.91	excluded	absent	74.33	-0.34	included	present	81.47	-0.23
211874_s_at	included	present	432.81	-0.31	included	present	411.34	-0.46	included	present	310.37	-1.12	included	present	614.75	0.88	included	present	597.14	0.77	included	present	409.56	-0.47	included	present	591	0.73	included	present	860.17	2.5
211875_x_at	included	present	137.24	0.55	excluded	absent	8.63	-1.29	excluded	absent	49.85	-0.7	included	present	185.89	1.25	excluded	absent	59.76	-0.56	included	present	171.09	1.04	excluded	absent	64.08	-0.49	included	present	188.11	1.28
211876_x_at	included	present	720.53	2.21	included	absent	80.72	-1.26	included	present	114.04	-1.08	included	absent	246.06	-0.36	included	present	371.01	0.31	included	present	467.77	0.84	included	present	692.59	2.06	included	present	360.96	0.26
211877_s_at	excluded	absent	74.86	0.29	excluded	absent	19	-0.67	excluded	absent	61.65	0.06	included	present	190.3	2.27	excluded	absent	4.07	-0.92	excluded	absent	129.64	1.23	excluded	absent	17.02	-0.7	included	present	64.36	0.11
211878_at	excluded	absent	6.23	-0.66	excluded	absent	53.13	1.28	excluded	absent	42.78	0.85	excluded	absent	24.13	0.08	excluded	absent	35.99	0.57	excluded	absent	2.83	-0.8	excluded	absent	20.69	-0.06	excluded	absent	32.84	0.44
211879_x_at	excluded	absent	56.14	-0.63	excluded	absent	74.16	-0.41	excluded	absent	36.03	-0.88	included	present	420.96	3.84	excluded	absent	77.38	-0.37	excluded	absent	110	0.03	excluded	absent	276.94	2.07	excluded	absent	234.22	1.55
211880_x_at	excluded	absent	75.24	0.14	excluded	absent	83.91	0.29	excluded	absent	96.72	0.51	included	present	300.85	4.02	excluded	absent	11.12	-0.97	included	present	141.66	1.28	included	present	265.5	3.41	included	present	139.35	1.24
211881_x_at	excluded	absent	8.02	-0.49	excluded	absent	81.99	-0.29	excluded	absent	51.19	-0.37	excluded	absent	77.59	-0.3	included	present	1044.14	2.35	excluded	absent	65.69	-0.33	included	present	196.84	0.03	excluded	absent	92.65	-0.26
211882_x_at	excluded	absent	38.01	0.32	excluded	absent	11.05	-0.38	excluded	absent	54.88	0.76	excluded	absent	3.86	-0.56	excluded	absent	8.37	-0.45	excluded	absent	43.9	0.48	excluded	absent	39.44	0.36	excluded	absent	5.83	-0.51
211883_x_at	included	absent	13.01	-0.29	included	absent	74.25	-0.26	included	present	135.47	-0.23	included	present	135.87	-0.23	included	absent	130.53	-0.23	included	present	256.97	-0.17	included	absent	88.81	-0.25	included	present	88.54	-0.25
211884_s_at	excluded	absent	10.34	-0.35	excluded	absent	5.23	-0.44	excluded	absent	5.44	-0.44	excluded	absent	2.76	-0.49	excluded	absent	18.24	-0.2	excluded	absent	5.04	-0.45	excluded	absent	3.44	-0.48	excluded	absent	2.78	-0.49
211885_x_at	excluded	absent	8.99	-0.75	excluded	absent	47.25	0.09	excluded	absent	21.61	-0.48	excluded	absent	12.01	-0.69	excluded	absent	10.84	-0.71	excluded	absent	38.27	-0.11	excluded	absent	9.1	-0.75	excluded	absent	47.06	0.08
211886_s_at	excluded	absent	11.58	-0.41	excluded	absent	126.47	0.26	excluded	absent	44.29	-0.22	excluded	absent	2.58	-0.47	excluded	absent	91.91	0.06	excluded	absent	40.71	-0.24	excluded	absent	34.81	-0.28	excluded	absent	2.65	-0.47
211887_x_at	included	present	3088.58	6.12	included	present	1084.47	1.44	included	present	1265.81	1.87	included	present	428.13	-0.09	included	present	1599.87	2.65	included	present	1516.76	2.45	included	present	473.96	0.02	included	present	309.21	-0.37
211888_x_at	excluded	absent	88.96	-0.08	excluded	absent	10.75	-0.76	excluded	absent	17.59	-0.71	excluded	absent	2.66	-0.84	excluded	absent	92.25	-0.05	excluded	absent	166.91	0.6	excluded	absent	5.6	-0.81	excluded	absent	2.14	-0.84
211889_x_at	excluded	absent	71.83	-0.3	excluded	absent	150.62	-0.22	excluded	absent	124.79	-0.25	included	present	187.75	-0.18	excluded	absent	127.87	-0.24	excluded	absent	199.47	-0.17	excluded	absent	8.39	-0.36	included	present	126.25	-0.24
211890_x_at	included	present	1292.91	-0.28	included	absent	146.07	-0.74	included	absent	148.07	-0.74	included	absent	139.67	-0.75	included	present	269.62	-0.69	included	present	744.49	-0.5	included	absent	381.23	-0.65	included	absent	72	-0.77
211891_s_at	excluded	absent	22.95	-0.98	excluded	absent	114.53	-0.44	excluded	absent	49.2	-0.82	excluded	absent	52.92	-0.8	excluded	absent	105.46	-0.49	excluded	absent	5.82	-1.08	excluded	absent	109.7	-0.47	excluded	absent	4.05	-1.09
211892_s_at	excluded	absent	12.74	-0.69	excluded	absent	11.27	-0.7	excluded	absent	10.34	-0.7	excluded	absent	10.18	-0.7	excluded	absent	27.21	-0.65	included	present	1258.28	3.36	excluded	absent	57.97	-0.55	excluded	absent	35.98	-0.62
211893_x_at	excluded	absent	42.42	-0.6	excluded	absent	21.53	-0.87	excluded	absent	138.28	0.62	excluded	absent	48.71	-0.52	excluded	absent	110.18	0.26	excluded	absent	101.49	0.15	excluded	absent	42.06	-0.61	excluded	absent	56.39	-0.42
211894_x_at	included	present	334.87	0.34	included	present	329.73	0.33	excluded	absent	118.79	-0.28	included	present	1096.05	2.53	included	present	483.47	0.77	excluded	absent	10.48	-0.59	included	present	342.85	0.37	included	present	2307.11	6
211896_s_at	included	present	19132.83	0.6	included	present	311.4	-0.81	included	present	435.06	-0.8	included	present	511.32	-0.8	included	present	2209.69	-0.67	included	present	18766.64	0.58	included	present	232.23	-0.82	included	present	246.01	-0.82
211897_s_at	excluded	absent	9.7	-0.38	excluded	absent	3.1	-0.62	excluded	absent	30.87	0.37	excluded	absent	2.9	-0.63	excluded	absent	13.11	-0.26	excluded	absent	5.27	-0.54	excluded	absent	7.59	-0.46	excluded	absent	89.76	2.48
211898_s_at	excluded	absent	5.44	-0.74	excluded	absent	26.98	0.16	excluded	absent	38.2	0.62	excluded	absent	62.33	1.62	excluded	absent	8.12	-0.62	excluded	absent	28.52	0.22	excluded	absent	3.59	-0.81	excluded	absent	4.68	-0.77
211899_s_at	excluded	absent	56.86	0.37	excluded	absent	39.2	-0.06	excluded	absent	51.97	0.25	excluded	absent	48.55	0.17	excluded	absent	8.4	-0.82	excluded	absent	12.81	-0.71	excluded	absent	22.27	-0.48	included	present	226.74	4.54
211900_x_at	excluded	absent	1.75	-0.94	excluded	absent	49.35	-0.29	excluded	absent	2.35	-0.93	excluded	absent	2.96	-0.92	excluded	absent	72.37	0.02	excluded	absent	118.93	0.65	excluded	absent	44.91	-0.35	excluded	absent	51.27	-0.27
211901_s_at	excluded	absent	75.04	0.77	excluded	absent	29.75	-0.34	excluded	absent	5.55	-0.93	excluded	absent	37.76	-0.14	excluded	absent	23.15	-0.5	excluded	absent	12.72	-0.75	excluded	absent	65.71	0.54	excluded	absent	43.13	-0.01
211902_x_at	included	present	276.95	-0.28	included	absent	99.08	-0.41	included	absent	64.28	-0.43	included	absent	49.97	-0.44	included	present	154.33	-0.36	included	present	584.27	-0.05	included	present	161.65	-0.36	included	present	156.35	-0.36
211903_s_at	excluded	absent	3.09	-0.8	excluded	absent	108.43	2.37	excluded	absent	6.27	-0.7	excluded	absent	5.79	-0.72	excluded	absent	38.05	0.25	excluded	absent	9.75	-0.6	excluded	absent	24.18	-0.16	excluded	absent	16.89	-0.38
211904_x_at	included	present	400.06	0.7	included	present	279.35	-0.47	included	present	319.06	-0.08	included	present	291.52	-0.35	included	present	428.41	0.97	included	present	242.62	-0.82	included	present	363.2	0.34	included	present	176.52	-1.46
211905_s_at	included	present	468.32	1.56	included	absent	108.53	-0.99	included	absent	24.6	-1.58	included	present	192.24	-0.39	included	absent	6.89	-1.7	included	present	295.59	0.34	included	absent	139.79	-0.76	included	absent	127.14	-0.85
211906_s_at	excluded	absent	34.73	0.82	excluded	absent	11.85	-0.19	excluded	absent	2.1	-0.62	excluded	absent	1.75	-0.64	excluded	absent	4.28	-0.52	excluded	absent	31	0.66	excluded	absent	0.93	-0.67	excluded	absent	3.51	-0.56
211907_s_at	included	present	164.51	-0.19	included	absent	127.9	-0.32	included	present	250.13	0.1	included	present	352.47	0.44	included	present	273.21	0.17	included	absent	4.8	-0.74	included	present	202.46	-0.07	included	present	226.85	0.02
211908_x_at	excluded	absent	61.08	-0.31	excluded	absent	120.63	-0.23	excluded	absent	59.84	-0.32	excluded	absent	107.03	-0.25	excluded	absent	646.92	0.53	excluded	absent	87.74	-0.28	excluded	absent	12.45	-0.38	excluded	absent	94.6	-0.27
211909_x_at	excluded	absent	39.03	-0.36	excluded	absent	85.54	-0.21	excluded	absent	6.71	-0.47	excluded	absent	2.62	-0.48	excluded	absent	5.98	-0.47	excluded	absent	109.51	-0.13	excluded	absent	74.27	-0.25	excluded	absent	29.76	-0.39
211910_at	excluded	absent	4.26	-0.5	excluded	absent	5.84	-0.44	excluded	absent	19.7	0.11	excluded	absent	2.74	-0.56	excluded	absent	11.96	-0.2	excluded	absent	8.09	-0.35	excluded	absent	14.15	-0.11	excluded	absent	6.58	-0.41
211911_x_at	included	present	3684.65	0.35	included	present	1272.41	-0.47	included	present	796.47	-0.63	included	present	1739.94	-0.31	included	present	2266.1	-0.13	included	present	10193.61	2.55	included	present	927.59	-0.58	included	present	1883.84	-0.26
211912_at	excluded	absent	37.76	1.27	excluded	absent	2.92	-0.64	excluded	absent	4.79	-0.54	excluded	absent	37.73	1.27	excluded	absent	13.74	-0.05	excluded	absent	8.78	-0.32	excluded	absent	1.25	-0.73	excluded	absent	2.04	-0.69
211913_s_at	included	present	377.99	-0.29	included	absent	194.11	-0.86	included	present	281.02	-0.59	included	absent	115.03	-1.11	included	present	335.21	-0.43	included	present	218.64	-0.79	included	present	200.34	-0.84	included	present	281.52	-0.59
211914_x_at	included	present	1133.23	2.34	included	present	787.59	0.39	included	present	1203.38	2.74	included	present	594.92	-0.7	included	present	1515.64	4.5	included	present	596.86	-0.69	included	present	1474.04	4.27	included	present	1354.37	3.59
211915_s_at	excluded	absent	45.17	-0.12	excluded	absent	7.26	-0.97	excluded	absent	3.05	-1.06	excluded	absent	6.24	-0.99	excluded	absent	63.69	0.29	excluded	absent	97.96	1.05	excluded	absent	8.05	-0.95	excluded	absent	28.37	-0.5
211916_s_at	excluded	absent	45.7	-0.15	excluded	absent	9.34	-0.19	excluded	absent	2.24	-0.2	excluded	absent	3.64	-0.2	excluded	absent	5.25	-0.2	excluded	absent	55.45	-0.13	excluded	absent	9.72	-0.19	excluded	absent	29.83	-0.17
211917_s_at	excluded	absent	84.91	-0.43	excluded	absent	2.07	-0.6	excluded	absent	70.29	-0.46	excluded	absent	26.45	-0.55	excluded	absent	15.66	-0.57	excluded	absent	17.94	-0.57	excluded	absent	22.78	-0.56	excluded	absent	2.47	-0.6
211918_x_at	excluded	absent	103.76	-0.56	excluded	present	193.58	0.29	excluded	absent	93.04	-0.66	excluded	absent	100.08	-0.6	excluded	absent	196.65	0.32	excluded	absent	376.13	2.01	excluded	absent	61.41	-0.96	excluded	present	483.82	3.03
211919_s_at	included	present	286.22	-0.31	included	present	1498.12	0.1	included	present	255.78	-0.32	included	present	996.1	-0.07	included	present	980.39	-0.07	included	present	1168.72	-0.01	included	present	1079.53	-0.04	included	present	865.7	-0.11
211920_at	excluded	absent	109.93	0.4	excluded	absent	158.68	1.15	excluded	absent	41.84	-0.64	excluded	absent	26.41	-0.88	excluded	absent	93.69	0.15	excluded	absent	111.24	0.42	excluded	absent	37.07	-0.72	excluded	absent	24.3	-0.91
211921_x_at	included	present	39159.19	-1.11	included	present	78635.59	0.63	included	present	59066.25	-0.23	included	present	77849.9	0.6	included	present	61135.54	-0.14	included	present	34800.55	-1.3	included	present	17774.88	-2.05	included	present	27412.2	-1.63
211922_s_at	included	present	1317.98	0.4	included	present	333.81	-0.86	included	present	850.45	-0.2	included	present	340.66	-0.85	included	present	1205.03	0.25	included	present	910.19	-0.13	included	present	599.77	-0.52	included	present	295.29	-0.91
211923_s_at	included	present	418.65	-0.42	included	present	389.7	-0.52	included	present	504.16	-0.12	included	present	799.52	0.91	included	present	305.99	-0.81	included	absent	195.51	-1.19	included	present	345.63	-0.67	included	present	195.46	-1.19
211924_s_at	included	present	49.93	-0.4	excluded	absent	12.48	-0.63	excluded	absent	81.7	-0.2	excluded	absent	47.18	-0.41	excluded	absent	107.64	-0.03	included	present	620.47	3.17	included	present	244.22	0.82	included	present	115.54	0.02
211925_s_at	included	present	652.99	-0.5	included	present	283.22	-0.73	included	present	428.11	-0.64	included	present	315.35	-0.71	included	present	474.43	-0.61	included	present	537.24	-0.57	included	present	523.51	-0.58	included	present	125.5	-0.83
211926_s_at	included	present	745.08	0.63	included	present	198.75	-0.81	included	present	342.71	-0.43	included	present	288.39	-0.57	included	absent	291.92	-0.56	included	present	5075.48	12.03	included	present	309.05	-0.52	included	present	560.6	0.14
211927_x_at	included	present	21874.81	-0.44	included	present	34211.06	0.59	included	present	43357.21	1.36	included	present	24649.06	-0.21	included	present	22457.86	-0.39	included	present	20781.56	-0.53	included	present	18127.92	-0.76	included	present	20688.16	-0.54
211928_at	included	present	4235.22	2.58	included	present	3993.04	2.28	included	present	1466.47	-0.82	included	present	1713.93	-0.52	included	present	1856.71	-0.34	included	present	3902.03	2.17	included	present	3813.71	2.06	included	present	4066.44	2.37
211929_at	included	present	4704.97	-1.1	included	present	3805.83	-1.6	included	present	10126.61	1.95	included	present	7307.16	0.37	included	present	4682.68	-1.11	included	present	3540.81	-1.75	included	present	7435.39	0.44	included	present	5060.5	-0.9
211930_at	included	present	1119.84	-0.29	included	present	1091.78	-0.37	included	present	1896.65	1.93	included	present	1830.1	1.74	included	present	1308.94	0.25	included	present	667.93	-1.57	included	present	1243.12	0.06	included	present	1046.68	-0.49
211931_s_at	included	present	8328.21	-0.54	included	present	10429.92	-0.04	included	present	22870.26	2.89	included	present	13128.33	0.59	included	present	9976.61	-0.15	included	present	6692.07	-0.92	included	present	6477.92	-0.98	included	present	7307.64	-0.78
211932_at	included	present	3617.09	-0.31	included	present	2148.54	-1.42	included	present	5766.81	1.31	included	present	6272	1.69	included	present	3247.83	-0.59	included	present	2848.28	-0.89	included	present	4094.16	0.05	included	present	4235.9	0.16
211933_s_at	included	present	10099.1	-1.03	included	present	8304.49	-1.63	included	present	20413.56	2.47	included	present	16304.82	1.08	included	present	10874.62	-0.76	included	present	6815.98	-2.14	included	present	8738.81	-1.49	included	present	8546.63	-1.55
211934_x_at	included	present	1207.07	1.55	included	present	826.98	0.42	included	present	878.4	0.58	included	present	1823.83	3.39	included	present	648.97	-0.11	included	present	2356.48	4.97	included	present	1294.78	1.81	included	present	1571.93	2.64
211935_at	included	present	10427.11	-0.33	included	present	17041.85	0.87	included	present	13465.21	0.22	included	present	10878.54	-0.25	included	present	12373.92	0.02	included	present	6102.6	-1.12	included	present	12819.89	0.1	included	present	10488.42	-0.32
211936_at	included	present	10363.66	0.14	included	present	8081.93	-0.34	included	present	11856.98	0.46	included	present	11675.24	0.42	included	present	8371.93	-0.28	included	present	7539.48	-0.45	included	present	5218.8	-0.95	included	present	6504.33	-0.67
211937_at	included	present	5863.06	-0.8	included	present	11813.29	0.85	included	present	11360.48	0.72	included	present	10313.15	0.43	included	present	7182.21	-0.44	included	present	3830.31	-1.36	included	present	4693.37	-1.12	included	present	7517.12	-0.34
211938_at	included	present	5365.12	-0.48	included	present	6704.22	-0.03	included	present	11584.83	1.59	included	present	9398.79	0.86	included	present	6697.68	-0.04	included	present	4298.45	-0.84	included	present	6747.52	-0.02	included	present	7741.12	0.31
211939_x_at	included	present	4478.87	-0.98	included	present	6552.96	-0.33	included	present	12059.38	1.4	included	present	9170.76	0.49	included	present	7753.14	0.05	included	present	7493.29	-0.03	included	present	6910.33	-0.22	included	present	12273.45	1.47
211940_x_at	included	present	7711.38	-0.3	included	present	22719.94	3.46	included	present	25051.13	4.05	included	present	16616.55	1.93	included	present	16704.64	1.95	included	present	9212.49	0.07	included	present	12340.01	0.86	included	present	9834.34	0.23
211941_s_at	included	present	3362.63	0.22	included	present	1397.77	-0.71	included	present	1772.01	-0.53	included	present	1952.85	-0.45	included	present	2215.17	-0.32	included	present	3667.37	0.36	included	present	6447.92	1.67	included	present	2525.28	-0.18
211942_x_at	included	present	5443.6	-0.07	included	present	2299.96	-0.84	included	present	6506.36	0.19	included	present	5559.33	-0.04	included	present	3157.86	-0.63	included	present	5902	0.04	included	present	4991.96	-0.18	included	present	8045.12	0.57
211943_x_at	included	present	44770.16	-0.69	included	present	49004.77	-0.53	included	present	53589.82	-0.36	included	present	34232.05	-1.09	included	present	47674.04	-0.58	included	present	37140.48	-0.98	included	present	24497.37	-1.46	included	present	21753.57	-1.57
211944_at	included	present	1235.58	0.64	included	present	1224.02	0.61	included	present	1449.73	1.3	included	present	1696.25	2.06	included	present	1664.02	1.96	included	present	1638.69	1.89	included	present	1945.72	2.83	included	present	1186.83	0.49
211945_s_at	included	present	17455.5	0.3	included	present	9159.77	-0.54	included	present	18869.02	0.45	included	present	12599.31	-0.19	included	present	12353	-0.21	included	present	18874.34	0.45	included	present	8944.9	-0.56	included	present	9739.92	-0.48
211946_s_at	included	present	3285.11	-1.18	included	present	6237.38	0.99	included	present	6133.3	0.91	included	present	5444.44	0.41	included	present	4254.3	-0.47	included	present	5346.07	0.33	included	present	5729.92	0.62	included	present	3605.44	-0.94
211947_s_at	included	present	2147.17	1.43	included	present	2587.67	2.67	included	present	2886.81	3.51	included	present	2298.7	1.86	included	present	2737.54	3.09	included	present	2627.41	2.78	included	present	4032.95	6.75	included	present	2571.59	2.62
211948_x_at	included	present	2554.49	0.2	included	present	3518.71	1.57	included	present	3184.88	1.1	included	present	2370.56	-0.07	included	present	3503.87	1.55	included	present	1890.69	-0.75	included	present	3001.58	0.83	included	present	2039.55	-0.54
211949_s_at	included	present	253.26	-0.78	included	present	488.64	0.65	included	present	979.47	3.65	included	present	344.52	-0.23	included	present	407.56	0.16	included	present	480.16	0.6	included	present	478.23	0.59	included	present	408.26	0.16
211950_at	included	present	1533.73	0.15	included	present	1511.78	0.12	included	present	456.19	-1.28	included	present	331.7	-1.44	included	present	726.38	-0.92	included	present	1927.24	0.68	included	present	1476.46	0.08	included	present	1670.09	0.33
211951_at	included	present	2992.46	-0.94	included	present	6700.61	1.36	included	present	10084.65	3.45	included	present	4349.61	-0.1	included	present	6955.84	1.51	included	present	2397.99	-1.31	included	present	6616.69	1.3	included	present	4959.9	0.28
211952_at	included	present	518.29	-0.64	included	present	1991.35	3.6	included	present	803.82	0.18	included	present	877.21	0.39	included	present	706.44	-0.1	included	present	186.51	-1.6	included	present	684.53	-0.16	included	present	780.57	0.11
211953_s_at	included	present	644.43	-0.25	included	present	1262.27	1.65	included	present	812.56	0.27	included	present	788.89	0.19	included	present	712.04	-0.04	included	present	866.91	0.43	included	present	1079.33	1.09	included	present	1676.18	2.93
211954_s_at	included	present	1900.75	-0.23	included	present	5267.21	3.11	included	present	3262.51	1.12	included	present	2882.52	0.75	included	present	2655.5	0.52	included	present	1958.08	-0.17	included	present	2937.83	0.8	included	present	3999.18	1.86
211955_at	included	present	1243.51	-0.13	included	present	2554.54	0.64	included	present	2896.9	0.84	included	present	1936.39	0.28	included	present	1840.99	0.22	included	present	1549.99	0.05	included	present	1906.63	0.26	included	present	2764.68	0.76
211956_s_at	included	present	22807.62	0.13	included	present	16165.39	-0.82	included	present	41132.76	2.75	included	present	16679.6	-0.75	included	present	19158.16	-0.4	included	present	21067.55	-0.12	included	present	17565.3	-0.62	included	present	18783.6	-0.45

212233_at	included	present	20292.94	2.95	included	present	13106.08	1.55	included	present	7217.6 [illegible]
212234_at	included	present	1025.19	-0.54	included	present	2334.81	2.32	included	present	2030.55 [illegible]
212235_at	included	present	960.91	1.98	included	present	553.54	0.36	excluded	absent	245.92 [illegible]
212236_x_at	excluded	absent	154.49	-0.26	excluded	absent	74.03	-0.34	excluded	absent	79.2 [illegible]
212237_at	included	present	481.69	-0.07	included	present	722.67	1.12	included	present	443.5 [illegible]
212238_at	included	present	152.33	-0.69	included	present	546.35	2.1	included	present	155.08 [illegible]
212239_at	included	present	3894.88	-0.02	included	present	2800.05	-0.61	included	present	3046.79 [illegible]
212240_s_at	included	present	1133.58	-0.23	included	present	369.72	-1.18	included	present	820.41 [illegible]
212241_at	included	present	938.87	0.33	included	present	685.38	-0.32	included	present	521.97 [illegible]
212242_at	included	present	9403.04	0.3	included	present	539.78	-1.04	included	present	747.74 [illegible]
212243_at	included	present	1054.73	0.74	included	present	517.67	-1.01	included	present	742.07 [illegible]
212244_at	included	present	1007.68	-0.41	included	present	1088.34	-0.34	included	present	1108.94 [illegible]
212245_at	included	present	3478.52	-0.26	included	present	2959.77	-0.47	included	present	1912.3 [illegible]
212246_at	included	present	1664.77	0.48	included	present	752.14	-0.42	included	present	978.45 [illegible]
212247_at	included	present	437.99	-0.11	included	present	1074.77	3.51	included	present	860.65 [illegible]
212248_at	included	present	1326	2.97	included	present	580.28	-0.37	included	present	3543.22 [illegible]
212249_at	included	present	594.84	-0.67	included	present	533.47	-0.82	included	present	568.65 [illegible]
212250_at	included	present	3550.29	-0.33	included	present	2355.87	-1.4	included	present	7171.34 [illegible]
212251_at	included	present	2883.07	-0.05	included	present	2763.46	-0.17	included	present	7105.12 [illegible]
212252_at	included	present	254.09	-0.55	included	present	140.3	-0.83	included	present	448.47 [illegible]
212253_x_at	included	present	4890.26	1.84	included	present	2745.38	0.29	included	present	1761.78 [illegible]
212254_s_at	included	present	10133.41	0.31	included	present	8337.54	-0.04	included	present	5401.84 [illegible]
212255_s_at	included	present	842.96	-0.44	included	present	815.81	-0.51	included	present	962.57 [illegible]
212256_at	included	present	1891.99	1.09	included	present	396.79	-1.4	included	present	562.1 [illegible]
212257_s_at	included	present	861.41	-1.18	included	absent	121.61	-2.97	included	present	1429.09 [illegible]
212258_s_at	excluded	absent	131.1	-1.18	excluded	absent	9.32	-2.4	excluded	absent	82.3 [illegible]
212259_s_at	included	present	147.29	-1.37	included	absent	83.48	-1.6	included	absent	60.61 [illegible]
212260_at	included	present	2353.03	0.79	included	present	1390.68	-1.3	included	present	1258.08 [illegible]
[illegible]											

212593_s_at	included	present	5946.74	-0.36	included	present	3953.46	-0.71	included	present	4887.6	-0.55
212594_at	included	present	2958.65	0.29	included	present	2412.73	-0.06	included	present	2740.41	0.15
212595_s_at	included	present	2725.47	0.02	included	present	966.32	-1.87	included	present	1517.38	-1.28
212596_s_at	included	present	345.19	0.4	included	present	379.6	0.74	included	present	511.42	2.04
212597_s_at	included	present	286.89	-0.98	included	present	520.25	1.17	included	present	519.68	1.16
212598_at	included	present	1998.25	0.41	included	present	921.87	-0.76	included	present	2149.53	0.57
212599_at	included	present	2006.98	1.61	included	present	4747.12	6.69	included	present	3607.88	4.58
212600_s_at	included	present	3985.36	-1.02	included	present	4739.07	-0.7	included	present	7346.74	0.4
212601_at	excluded	absent	204.14	0.17	excluded	absent	128.44	-0.67	excluded	absent	87.58	-1.13
212602_at	included	present	1467.08	-0.43	included	present	2074.82	0.28	included	present	1292.12	-0.64
212603_at	included	present	1118.54	-1.04	included	present	2308.94	1.32	included	present	2089.29	0.89
212604_at	included	present	1185.92	0.6	included	present	1353.47	1.25	included	present	1942.43	3.51
212605_s_at	included	present	4736.69	1.66	included	present	2423.04	0.06	included	present	3081.71	0.51
212606_at	included	present	502.57	0.19	included	present	315.97	-0.54	included	present	159.39	-1.15
212607_at	included	present	1127.47	0.72	included	present	1558.27	1.8	included	present	2783.55	4.89
212608_s_at	included	present	4444.24	1.16	included	present	4648.46	1.28	included	present	2315.53	-0.11
212609_s_at	included	present	3803.18	0.27	included	present	6592.16	2.5	included	present	7439.21	3.18
212610_at	included	present	1617.32	-0.9	included	present	1962.52	-0.63	included	present	1254.11	-1.19
212611_at	included	present	796.11	1.64	included	present	1083.11	2.77	included	absent	110.12	-1.05
212612_at	included	present	442.68	-0.89	included	present	590.47	-0.35	included	present	567.59	-0.44
212613_at	included	present	476.91	0.2	included	absent	159.03	-0.56	included	absent	174.55	-0.53
212614_at	included	present	752.04	-0.74	included	present	263	-0.93	included	present	175.91	-0.96
212615_at	included	present	458.14	1.45	included	present	260.43	-0.33	included	present	538.55	2.17
212616_at	included	present	950.61	0.8	included	present	324.99	-1.91	included	present	1471.17	3.06
212617_at	included	present	230.6	-0.3	included	present	342.34	1.07	included	present	164.63	-1.11
212618_at	included	present	178.45	-0.09	included	present	526.05	3.43	included	present	386.2	2.01
212619_at	included	present	102.92	-0.6	included	present	188.43	0.73	included	present	373.36	3.61
212620_at	included	present	1334.85	0.99	included	present	1616.73	1.89	included	present	1439.09	1.32
212621_at	included	present	385.83	-0.09	included	present	1351.36	3.81	included	present	1096.03	2.78
212622_at	included	present	2232.7	-0.24	included	present	4603.48	2.47	included	present	2408.68	-0.04
212623_at	included	present	1097.45	0.41	included	present	1625.39	1.61	included	present	834.16	-0.2
212624_s_at	included	present	2782.71	-0.55	included	present	1899.86	-0.62	included	present	3438.14	-0.5
212625_at	included	present	183.06	1.03	included	present	203.83	1.45	excluded	absent	123.27	-0.16
212626_x_at	included	present	3314.24	-1.98	included	present	6101.72	-0.35	included	present	7161.79	0.27
212627_s_at	included	present	1205.09	-0.86	included	present	3493.55	1.54	included	present	2284.42	0.27
212628_at	included	present	961.8	-0.07	included	present	605.74	-1.06	included	present	539.74	-1.25
212629_s_at	included	present	2129.05	-0.66	included	present	1697.51	-1.15	included	present	1525.59	-1.34
212630_at	included	present	232.02	-0.86	included	present	484.22	1.17	included	present	176.83	-1.3
212631_at	included	present	5012.66	0.65	included	present	2002.4	-1.44	included	present	3693.14	-0.27
212632_at	included	present	1822.03	0.19	included	present	760.89	-1.37	included	present	976.96	-1.05
212633_at	included	present	176.7	-1.72	included	present	231.13	-1.46	included	present	143.77	-1.88
212634_at	included	present	922.15	0.67	included	present	396.95	-1.71	included	present	217.12	-2.52

[illegible]

212954_at	included	present	516.15	-0.34	included	present	593.89	-0.14	included	present	433.76	-0.56	included	present	367.97	-0.73	[illegible]	[illegible]
212955_s_at	included	present	924.86	0.02	included	present	1111.08	0.43	included	present	892.08	-0.05	included	present	1628.65	1.59	[illegible]	[illegible]
212956_at	included	present	1016.63	0.27	included	present	970.54	0.18	included	present	727.07	-0.28	included	present	630.02	-0.47	[illegible]	[illegible]
212957_s_at	included	present	1024	1.08	included	present	705.08	0.05	included	present	705.51	0.05	included	present	1387.74	2.24	[illegible]	[illegible]
212958_x_at	included	present	2874.74	0.13	included	present	736.27	-1.3	included	present	1403.29	-0.85	included	present	3675.32	0.67	[illegible]	[illegible]
212959_s_at	included	present	889.05	-0.24	included	present	1444.63	0.94	included	present	518.96	-1.03	included	present	1275.24	0.58	[illegible]	[illegible]
212960_at	excluded	absent	28.99	-0.82	excluded	absent	114.92	0.98	excluded	absent	7.88	-1.26	excluded	absent	7.09	-1.27	[illegible]	[illegible]
212961_x_at	included	present	958.69	-0.82	included	present	597.48	-1.88	included	present	809.16	-1.26	included	present	495.36	-2.18	[illegible]	[illegible]
212962_at	excluded	absent	55.63	-0.32	excluded	absent	4.58	-0.93	excluded	absent	6.77	-0.9	included	present	108.89	0.31	[illegible]	[illegible]
212963_at	included	present	857.07	0.29	included	present	450.48	-1.28	included	present	564.77	-0.84	included	present	1263.84	1.87	[illegible]	[illegible]
212964_at	included	present	326.13	0.14	included	present	950.16	3.61	included	absent	63.76	-1.31	included	present	408.37	0.6	[illegible]	[illegible]
212965_at	excluded	absent	89.6	0.35	excluded	absent	116.08	0.85	excluded	absent	12.37	-1.13	excluded	absent	63.63	-0.15	[illegible]	[illegible]
212966_at	included	present	277.64	1.21	included	present	750.51	7.67	included	present	141.89	-0.64	included	present	600.9	5.63	[illegible]	[illegible]
212967_x_at	included	present	8788.25	-0.35	included	present	13625.58	0.93	included	present	16318.43	1.65	included	present	15484.01	1.43	[illegible]	[illegible]
212968_at	excluded	absent	13.41	-0.46	excluded	absent	110.66	1.9	excluded	absent	4.8	-0.67	excluded	absent	11.57	-0.5	[illegible]	[illegible]
212969_x_at	included	present	399.52	0.53	excluded	absent	410.4	0.6	included	present	194.49	-0.7	included	present	209.7	-0.6	[illegible]	[illegible]
212970_at	excluded	absent	370.27	0.55	included	present	398.03	0.73	included	absent	127.78	-1.03	included	present	621.66	2.19	[illegible]	[illegible]
212971_at	included	present	4417.95	0.49	included	present	8893.09	4.99	included	present	2925.71	-1.01	included	present	2658.19	-1.28	[illegible]	[illegible]
212972_x_at	included	present	3221.86	1.16	included	present	1610.04	-1.02	included	present	2392.93	0.04	included	present	3469.28	1.49	[illegible]	[illegible]
212973_at	included	present	521.39	-0.73	included	present	1548.42	2.26	included	present	1612.41	2.45	included	present	837.84	0.19	[illegible]	[illegible]
212974_at	excluded	absent	89.08	-0.26	excluded	absent	4.41	-1.31	excluded	absent	81.75	-0.35	excluded	absent	65.54	-0.55	[illegible]	[illegible]
212975_at	included	present	199.14	-1.03	included	present	175.22	-1.23	included	absent	135.55	-1.57	included	present	111.59	-1.77	[illegible]	[illegible]
212976_at	included	present	301	-0.52	included	present	731.43	-0.03	included	absent	90.9	-0.75	included	present	662.43	-0.11	[illegible]	[illegible]
212977_at	included	present	1838.02	-0.11	included	present	321.97	-0.99	included	present	487.43	-0.9	included	present	719.42	-0.76	[illegible]	[illegible]
212978_at	included	present	492.45	-0.39	included	present	586.53	-0.24	included	present	129.52	-0.94	included	present	1233.7	0.74	[illegible]	[illegible]
212979_s_at	included	present	2881.21	0.48	included	present	6207.68	2.67	included	present	3543.71	0.91	included	present	2170.72	0.01	[illegible]	[illegible]
212980_at	included	present	1860.66	-0.56	included	present	2753.4	0.15	included	present	2141	-0.34	included	present	2124.62	-0.35	[illegible]	[illegible]
212981_s_at	included	present	3349.58	-0.64	included	present	8048.17	0.75	included	present	4432.75	-0.32	included	present	2800.24	-0.81	[illegible]	[illegible]
212982_at	included	present	1621.85	-0.6	included	present	2396.29	0.42	included	present	1478.12	-0.78	included	present	1682.15	-0.52	[illegible]	[illegible]
212983_at	included	present	898.9	2.5	included	present	1133.25	3.7	included	present	355.27	-0.29	included	present	370.2	-0.21	[illegible]	[illegible]
212984_at	included	present	2485.55	-1.05	included	present	2870.48	-0.75	included	present	3509.31	-0.25	included	present	5488.33	1.3	[illegible]	[illegible]
212985_at	included	present	566.82	0.03	included	present	583.33	0.08	included	present	210.98	-1.08	included	present	885.6	1.02	[illegible]	[illegible]
212986_s_at	included	present	480.97	1.32	included	present	354.65	0.52	included	present	660.94	2.46	included	present	491.55	1.39	[illegible]	[illegible]
212987_at	included	present	5201.36	-0.83	included	present	9235.34	0.92	included	present	5681.74	-0.62	included	present	9147.17	0.88	[illegible]	[illegible]
[illegible]	[illegible]	[illegible]	[illegible]	[illegible]	[illegible]	[illegible]	[illegible]	[illegible]	[illegible]	[illegible]	[illegible]	[illegible]	[illegible]	[illegible]	[illegible]	[illegible]	[illegible]	[illegible]

213315_x_at	included	present	856.34	-1.52	included	present	613.73	-2.17	included	present	827.76	-1.59	included	present	505.75	-2.46	included	present	989.33	-1.16	included	present	1034.67	-1.03	included	present	1556.91	0.38	included	present	1362.98	-0.15	included	present
213316_at	included	present	621.33	-0.45	included	present	415.06	-0.75	included	absent	148.06	-1.13	included	present	101.12	-1.2	included	present	523.59	-0.59	included	present	1643.84	1.03	included	present	266.8	-0.96	included	present	791.13	-0.2	included	present
213317_at	included	present	104.67	-0.36	excluded	absent	3.6	-0.46	excluded	absent	0.36	-0.46	excluded	absent	2.43	-0.46	excluded	absent	78.97	-0.38	included	present	321.7	-0.13	excluded	absent	50.59	-0.41	excluded	absent	2.08	-0.46	included	present
213318_s_at	included	present	1158	0.78	included	present	3409.39	6.24	included	present	1311.12	1.15	included	present	1561.4	1.76	included	present	1663.39	2	included	present	1143.51	0.74	included	present	2417.64	3.83	included	present	1465.99	1.53	included	present
213319_s_at	included	present	537.56	0.06	included	absent	15.17	-0.48	included	absent	76.58	-0.41	included	absent	38.32	-0.45	included	absent	87.85	-0.4	included	present	1076.56	0.61	included	absent	66.74	-0.42	included	present	85.19	-0.41	included	present
213320_at	included	present	172.33	-0.81	included	present	179.45	-0.7	included	present	208.51	-0.23	included	present	232.06	0.15	included	present	285.37	1.02	included	present	173.99	-0.79	included	present	296.43	1.2	included	present	176.08	-0.75	included	present
213321_at	included	present	148.17	-0.48	included	absent	74.9	-1.09	included	absent	99.62	-0.89	included	present	136.05	-0.58	included	absent	114.63	-0.76	included	absent	136.54	-0.58	included	present	219.76	0.11	included	present	279.16	0.61	included	present
213322_at	included	present	2674.15	-0.28	included	present	6783.92	3.02	included	present	3126.11	0.08	included	present	4141.4	0.9	included	present	3705.08	0.55	included	present	1144.38	-1.51	included	present	2679.08	-0.28	included	present	3139.79	0.09	included	present
213323_s_at	excluded	absent	136.34	0.37	excluded	absent	72.47	-0.38	excluded	absent	128.54	0.28	excluded	absent	76.68	-0.33	excluded	absent	29.43	-0.89	excluded	absent	140.27	0.42	excluded	absent	170.89	0.78	excluded	absent	96.46	-0.1	excluded	absent
213324_at	included	present	202.42	-0.24	included	present	232.35	-0.05	included	present	179.08	-0.39	included	present	68	-1.09	included	present	386.98	0.93	included	present	182.35	-0.37	included	present	286.77	0.29	included	present	1333.18	6.93	included	present
213325_at	included	present	328.75	-0.17	included	present	193.16	-0.48	included	present	195.86	-0.48	included	absent	76.32	-0.76	included	present	332.5	-0.16	included	present	353.52	-0.11	included	present	245.13	-0.36	included	present	2864.89	5.78	included	present
213326_at	included	present	1389.03	0.52	included	present	312.89	-1	included	present	188.34	-1.18	included	present	575.22	-0.63	included	present	782.28	-0.34	included	present	712.58	-0.44	included	present	773.09	-0.35	included	present	761.52	-0.37	included	present
213327_s_at	included	present	2067.23	0.83	included	present	971.14	-0.92	included	present	1188.14	-0.57	included	present	1093.69	-0.72	included	present	1295.82	-0.4	included	present	844.55	-1.12	included	present	2442.24	1.42	included	present	1109.23	-0.7	included	present
213328_at	included	present	2425.6	1.72	included	present	1698.1	0.22	included	present	1672.51	0.17	included	present	938.94	-1.33	included	present	1650.54	0.13	included	present	772.97	-1.67	included	present	1297.04	-0.6	included	present	1629.11	0.08	included	present
213329_at	included	present	414.63	0.47	included	present	412.05	0.45	included	present	177.97	-0.79	included	present	291.95	-0.19	included	present	624.22	1.58	included	present	659.26	1.77	included	present	400.05	0.39	included	present	268.85	-0.31	included	present
213330_s_at	included	present	1228.97	0.83	included	present	2424.17	3.84	included	present	2011.96	2.8	included	present	527.12	-0.93	included	present	1411.8	1.29	included	present	909.17	0.03	included	present	1450	1.39	included	present	1712.71	2.05	included	present
213331_s_at	included	present	5232.9	1.72	included	present	3976.84	0.59	included	present	3206.32	-0.11	included	present	1948.05	-1.25	included	present	2770.71	-0.51	included	present	2194.15	-1.03	included	present	3593.43	0.24	included	present	2319.9	-0.92	included	present
213332_at	excluded	absent	71.33	-0.28	excluded	absent	54.12	-0.51	excluded	absent	7.83	-1.11	excluded	absent	3.34	-1.17	excluded	absent	145.11	0.68	included	present	210.91	1.54	excluded	absent	65.61	-0.36	included	present	426.3	4.35	included	present
213333_at	included	present	537.45	0.73	included	present	313.91	-0.33	included	present	259.91	-0.59	included	present	159.58	-1.06	included	present	461.3	0.37	included	present	235.07	-0.7	included	present	1265.68	4.18	included	present	922.91	2.56	included	present
213334_x_at	included	present	320.29	-0.27	included	present	523.75	1.39	included	present	240.8	-0.92	included	present	235.11	-0.97	included	present	286.91	-0.55	included	present	676.43	2.63	included	present	257.01	-0.79	included	present	461.29	0.88	included	present
213335_s_at	included	present	311.78	2	excluded	absent	127.49	-0.31	included	present	624.52	5.92	excluded	absent	94.24	-0.73	included	present	268.68	1.46	excluded	absent	135.85	-0.21	excluded	absent	193.38	0.51	included	present	171.85	0.24	excluded	absent
213336_at	included	present	1347.33	2.68	included	present	1105.92	1.53	included	present	1239.02	2.16	included	present	1796.64	4.82	included	present	702.4	-0.4	included	present	1528.34	3.54	included	present	1560.12	3.7	included	present	1301.41	2.46	included	present
213337_s_at	excluded	absent	93.48	-0.3	excluded	absent	26.08	-0.97	excluded	absent	8.17	-1.15	included	present	219.84	0.98	excluded	absent	98.4	-0.25	included	present	201.45	0.79	excluded	absent	40.09	-0.83	excluded	absent	10.14	-1.13	excluded	absent
213338_at	included	absent	122.67	-0.7	included	absent	14.87	-1.01	included	absent	72.1	-0.84	included	absent	67.31	-0.86	included	absent	38.08	-0.94	included	present	207.84	-0.45	included	absent	122.13	-0.7	included	absent	102.18	-0.76	included	absent
213339_at	excluded	absent	97.95	-0.58	excluded	absent	166.49	0.28	excluded	absent	85.03	-0.74	excluded	absent	139.22	-0.06	excluded	absent	84.05	-0.75	included	present	160.1	0.2	included	present	200.98	0.72	included	present	360.95	2.73	included	present
213340_s_at	included	present	1256.09	1.1	included	present	2509.56	5.11	included	present	846.68	-0.21	included	present	877.82	-0.11	included	present	1317.78	1.3	included	present	866.4	-0.15	included	present	909.7	-0.01	included	present	1524.99	1.96	included	present
213341_at	included	present	1922.55	0.08	included	present	516.67	-1.71	included	present	2998.21	1.45	included	present	3720.1	2.37	included	present	2169.13	0.4	included	present	1081.66	-0.99	included	present	1412.1	-0.57	included	present	718.04	-1.45	included	present
213342_at	included	present	424	-0.16	included	absent	81.31	-1.05	included	present	120.43	-0.95	included	absent	91.07	-1.03	included	absent	183.68	-0.79	included	present	662.4	0.46	included	absent	49.34	-1.14	included	absent	76.81	-1.07	included	present
213343_s_at	included	present	319.41	0.54	included	present	209.84	0.04	included	absent	80.9	-0.54	included	absent	24.59	-0.79	included	present	187.83	-0.06	included	present	573.11	1.68	included	present	230.42	0.14	included	present	644.49	2	included	present
213344_s_at	included	present	388.88	-0.42	included	present	708.44	0.58	included	present	1437.87	2.86	included	present	616.72	0.29	included	present	274.59	-0.78	included	present	1129.93	1.9	included	present	353.25	-0.53	included	present	4773.48	13.29	included	present
213345_at	included	absent	288.03	-0.41	included	present	130.21	-1.14	included	absent	245.27	-0.61	included	present	215.41	-0.75	included	absent	162.36	-0.99	included	present	508.44	0.61	included	absent	269.95	-0.5	included	present	521.75	0.67	included	present
213346_at	included	absent	43.51	-0.47	included	absent	309.72	-0.04	included	present	206.61	-0.21	included	present	235.46	-0.16	included	absent	273.47	-0.1	included	absent	119.78	-0.34	included	present	199.6	-0.22	included	present	185.29	-0.24	included	present
213347_x_at	included	present	40737.53	-0.49	included	present	41642.67	-0.44	included	present	56586.5	0.4	included	present	48014.86	-0.08	included	present	47706	-0.1	included	present	24734.06	-1.39	included	present	24685.85	-1.4	included	present	24922.77	-1.38	included	present
213348_at	included	present	1539.28	0.36	included	absent	130.1	-1.08	included	present	212.24	-0.99	included	present	420.06	-0.78	included	present	1999.34	0.82	included	present	2288.55	1.12	included	present	5075.9	3.95	included	present	415.15	-0.79	included	present
213349_at	included	present	386.97	-0.67	included	present	226.39	-1.68	included	present	289.97	-1.28	included	present	499.34	0.04	included	present	405.62	-0.55	included	present	634.53	0.89	included	present	550.43	0.36	included	present	419.3	-0.46	included	present
213350_at	included	present	34951.13	-0.23	included	present	30612.35	-0.49	included	present	48111.55	0.53	included	present	44165.16	0.3	included	present	35226.73	-0.22	included	present	39314.1	0.02	included	present	25347.14	-0.79	included	present	31162.77	-0.45	included	present
213351_s_at	included	present	365.01	-0.38	included	present	196.42	-1.45	included	present	199	-1.43	included	present	454.93	0.18	included	present	248.15	-1.12	included	present	469.98	0.28	included	present	437.26	0.07	included	present	319.34	-0.67	included	present
213352_at	included	present	353.98	0.88	included	present	124.82	-1.06	included	present	196.15	-0.46	included	present	352.49	0.86	included	present	270.01	0.17	included	present	265.34	0.13	included	present	638.96	3.28	included	present	342.04	0.77	included	present
213353_at	included	present	747.13	-0.76	included	present	302.75	-1.22	included	present	283.49	-1.24	included	present	2384.7	0.96	included	present	619.99	-0.89	included	present	435.73	-1.08	included	present	752.95	-0.75	included	present	937.69	-0.56	included	present
213354_s_at	excluded	absent	66.83	1.3	excluded	absent	25.64	-0.03	excluded	absent	2.24	-0.79	excluded	absent	9.58	-0.55	excluded	absent	6.99	-0.64	excluded	absent	4.53	-0.72	excluded	absent	55.58	0.94	excluded	absent	22.48	-0.14	excluded	absent
213355_at	included	present	2372.97	0.52	included	present	1390.46	-0.12	included	present	4488.14	1.9	included	present	348.11	-0.8	included	present	3667.39	1.36	included	present	682.5	-0.58	included	present	1569.34	0	included	present	2808.19	0.8	included	present
213356_x_at	included	present	19026.55	-0.77	included	present	34739.65	1.7	included	present	45803.16	3.44	included	present	33258.79	1.47	included	present	22363.31	-0.25	included	present	14043.1	-1.55	included	present	17475.79	-1.01	included	present	17621.42	-0.99	included	present
213357_at	included	present	1075.15	0.36	included	present	230.63	-1.97	included	present	731.61	-0.59	included	present	542.43	-1.11	included	present	733.18	-0.58	included	present	1442.42	1.37	included	present	1360.94	1.15	included	present	1160.83	0.6	included	present
213358_at	included	present	114.03	-0.5	included	present	527.69	3.53	included	present	314.96	1.46	included	present	116.81	-0.47	included	present	538.3	3.63	included	absent	72.62	-0.9	included	present	545.17	3.7	included	present	787.92	6.06	included	present
213359_at	included	present	840.53	-0.77	included	present	1262.01	-0.33	included	present	2548.64	1.03	included	present	1935.77	0.38	included	present	1096.52	-0.5	included	present	635.4	-0.98	included	present	1069.2	-0.53	included	present	450.87	-1.18	included	present
213360_s_at	included	present	3934.47	-0.96	included	present	8504.68	3.91	included	present	5411.42	0.62	included	present	8312.45	3.71	included	present	8636.9	4.05	included	present	3622.1	-1.29	included	present	8904.47	4.34	included	present	4079.96	-0.8	included	present
213361_at	included	present	1157.2	-0.31	included	present	646.09	-0.77	included	present	753.65	-0.67	included	present	579.62	-0.83	included	present	1540.68	0.04	included	present	864.35	-0.57	included	present	736.02	-0.69	included	present	1369.57	-0.11	included	present
213362_at	included	present	120.54	-0.56	included	absent	147.57	-0.47	included	present	240.16	-0.19	included	present	120.14	-0.56	included	present	368.19	0.2	included	present	121.75	-0.55	included	present	197.2	-0.32	included	absent	0.4	-0.92	included	present
213363_at	excluded	absent	79.57	1.14	excluded	absent	64	0.78	excluded	absent	42.47	0.27	excluded	absent	3.62	-0.64	excluded	absent	13.9	-0.4	excluded	absent	71.95	0.96	excluded	absent	34.84	0.09	excluded	absent	65.21	0.8	excluded	absent
213364_s_at	included	present	1712.45	0.09	included	present	621.41	-1.21	included	present	470.44	-1.39	included	present	1330.44	-0.37	included	present	596.65	-1.24	included	present	1024.68	-0.73	included	present	962.63	-0.8	included	present	1270.2	-0.44	included	present
213365_at	included	present	315.72	-0.67	included	present	620.18	0.17	included	present	576.03	0.04	included	present	406.93	-0.42	included	present	216.01	-0.94	included	present	377.12	-0.5	included	present	468.49	-0.25	included	present	779.42	0.6	included	present
213366_x_at	included	present	5464.93	-0.52	included	present	8572.98	0.24	included	present	7471.79	-0.03	included	present	4976.13	-0.64	included	present	7638.53	0.01	included	present	4701.92	-0.71	included	present	4296.51	-0.81	included	present	6237.23	-0.34	included	present
213367_at	included	present	195.63	-0.71	included	present	329.76	0.53	included	absent	9.26	-2.42	included	absent	163.99	-1	included	present	186.31	-0.79	included	present	272.16	0	included	present	313.12	0.37	included	absent	80.29	-1.77	included	present
213368_x_at	excluded	absent	136.9	0.13	excluded	absent	158.91	0.41	excluded	absent	84.03	-0.53	excluded	absent	96.95	-0.37	excluded	absent	10.08	-1.45	excluded	absent	8.88	-1.46	excluded	absent	162.41	0.45	excluded	absent	104.31	-0.27	excluded	absent
213369_at	excluded	absent	101.49	-0.21	excluded	absent	33.45	-0.34	excluded	absent	62.5	-0.28	excluded	absent	147.08	-0.12	excluded	absent	98.1	-0.21	excluded	absent	6.22	-0.39	excluded	absent	67.42	-0.27	included	present	184.57	-0.04	excluded	absent
213370_s_at	included	absent	215.59	-0.18	included	absent	169.36	-0.27	included	present	131.66	-0.35	included	present	140.13	-0.33	included	present	312.05	0.02	included	absent	90.6	-0.43	included	present	247.47	-0.11	included	present	190.85	-0.23	included	present
213371_at	included	absent	90.94	-0.44	included	absent	9.13	-0.51	included	present	137.14	-0.4	included	absent	91.52	-0.44	included	absent	150.73	-0.39	included	present	3404.24	2.4	included	absent	17.66	-0.5	included	present	119.53	-0.41	included	present
213372_at	included	present	269.89	-0.97	included	present	781.06	0.61	included	present	400.17	-0.57	included	absent	38.87	-1.68	included	present	331.5	-0.78	included	present	364.06	-0.68	included	present	1012.66	1.32	included	present	202.09	-1.18	included	present
213373_s_at	included	present	280.77	-0.25	included	present	184.81	-0.45	included	present	130.59	-0.56	included	present	419.77	0.03	included	present	133.26	-0.55	included	present	1205.9	1.65	included	present	200.11	-0.42	included	present	165.06	-0.49	included	present
213374_x_at	included	present	993.21	-0.55	included	present	1149.25	-0.46	included	present	1067.19	-0.5	included	present	877.8	-0.62	included	present	871.55	-0.62	included	present	1381.56	-0.32	included	present	1099.26	-0.49	included	present	1463.35	-0.27	included	present
213375_s_at	included	present	596.29	-1.28	included	present	203.45	-1.84	included	present	845.35	-0.93	included	present	726.42	-1.1	included	present	593.8	-1.29	included	present	578.75	-1.31	included	present	925.31	-0.82	included	present	560.89	-1.33	included	present
213376_at	included	present	1270.24	0.96	included	present	585.73	-1.05	included	present	1000.61	0.17	included	present	2460.78	4.46	included	present	1040.23	0.28	included	present	958.72	0.04	included	present	716.35	-0.67	included	present	910.08	-0.1	included	present

213675_at	included	present	1053.23	0.59	included	present	827.42	0.27	included	present	627.47	-0.01	included	present	256.57	-0.54
213676_at	excluded	absent	106.68	-0.38	included	present	1063.67	2.29	excluded	absent	133.31	-0.31	excluded	absent	100.29	-0.4
213677_s_at	included	present	528.38	-0.46	included	present	498.79	-0.49	included	present	1049.04	0.11	included	present	1592.84	0.69
213678_at	included	present	328.26	-0.04	included	present	954.71	1.01	included	present	708.24	0.6	excluded	absent	61.22	-0.49
213679_at	included	present	93.2	-0.27	included	present	147.19	0.56	included	present	104.72	-0.09	included	present	375.93	4.08
213680_at	excluded	absent	2.16	-0.26	excluded	absent	1.24	-0.27	excluded	absent	38.8	-0.2	excluded	absent	1.16	-0.27
213681_at	excluded	absent	74.26	-0.47	included	present	182.79	1.37	excluded	absent	46.51	-0.94	excluded	absent	16.23	-1.45
213682_at	included	present	1299.24	-0.89	included	present	2121.17	0.43	included	present	2711.45	1.38	included	present	2723.79	1.4
213683_at	excluded	absent	84.02	-0.28	excluded	absent	50.17	-0.55	excluded	absent	35.81	-0.67	excluded	absent	6.43	-0.9
213684_s_at	included	present	131.92	-0.38	included	present	169.68	-0.37	included	present	96.53	-0.4	included	absent	16.32	-0.44
213685_at	included	present	1432.78	-0.32	included	present	1412.21	-0.38	included	present	1414.7	-0.38	included	present	1746.02	0.64
213686_at	included	present	211.69	-0.86	included	absent	171.52	-1	included	present	244.17	-0.74	included	present	276.11	-0.62
213687_s_at	included	present	18393.82	-0.67	included	present	25853.6	-0.02	included	present	31758.61	0.5	included	present	19719.73	-0.56
213688_at	included	present	9626.27	0.6	included	present	1965.08	-0.75	included	present	9106.84	0.51	included	present	2734.29	-0.61
213689_x_at	included	present	139.77	-1.34	included	present	585.78	0.2	included	present	76.69	-1.56	included	present	412.74	-0.4
213690_s_at	included	present	156.24	0.69	excluded	absent	95.75	-0.2	excluded	absent	95.3	-0.2	excluded	absent	59.54	-0.73
213691_at	excluded	absent	116.61	-0.42	excluded	absent	20.86	-1.55	excluded	absent	93.92	-0.69	excluded	absent	73.22	-0.93
213692_s_at	excluded	absent	3.51	-0.55	excluded	absent	44.35	-0.37	excluded	absent	1.5	-0.56	excluded	absent	10.24	-0.52
213693_s_at	excluded	absent	64.95	-0.47	included	present	208.3	-0.26	excluded	absent	127.87	-0.38	included	present	1635.38	1.85
213694_at	included	present	250.26	-0.47	included	present	368.97	0.56	included	present	410.18	0.92	included	present	452.13	1.29
213695_at	excluded	absent	86.83	-0.22	excluded	absent	5.43	-0.27	excluded	absent	111.1	-0.2	included	present	483.37	0.05
213696_s_at	included	present	528.43	0.81	included	present	728.61	2.22	included	present	149.73	-1.86	included	present	442.91	0.2
213697_at	included	present	1320.55	0.57	included	present	239.38	-1.64	included	present	499.94	-1.11	included	present	402.05	-1.31
213698_at	included	present	2379.41	0.93	included	present	1010.12	-0.62	included	present	788.71	-0.87	included	present	2582.14	1.16
213699_s_at	included	present	14109.61	-1.25	included	present	28630.41	0.46	included	present	24328.71	-0.05	included	present	16094.2	-1.02
213700_s_at	included	present	140.92	-1.05	included	present	240.05	0.01	included	present	196.87	-0.45	included	present	302.91	0.68
213701_at	included	present	531.9	-0.81	included	present	480.9	-0.98	included	present	723.3	-0.17	included	present	959.8	0.61
213702_x_at	included	present	5314.23	-0.47	included	present	4336.37	-0.79	included	present	4809.07	-0.63	included	present	3762.12	-0.98
213703_at	included	present	3951.52	-0.57	included	present	6481.51	0.22	included	present	10767.78	1.56	included	present	5435.88	-0.11
213704_at	included	present	1245.98	-0.48	included	present	983.88	-0.97	included	present	993.54	-0.95	included	present	1874.58	0.69
213705_at	included	present	5624.95	-1.22	included	present	10985.92	1.15	included	present	10135.38	0.77	included	present	6284.96	-0.93
213706_at	excluded	absent	3.08	-0.55	excluded	absent	102.41	-0.32	excluded	absent	7.34	-0.54	excluded	absent	7.73	-0.53
213707_s_at	excluded	absent	58.16	-0.51	included	present	117.36	0.06	excluded	absent	89.4	-0.21	included	present	335.3	2.16
213708_s_at	included	present	2122.54	0.01	included	present	11293.48	10.72	included	present	9229.05	8.31	included	present	3562.63	1.69
[illegible]	[illegible]	[illegible]	[illegible]	[illegible]	[illegible]	[illegible]	[illegible]	[illegible]	[illegible]	[illegible]	[illegible]	[illegible]	[illegible]	[illegible]	[illegible]	[illegible]

214035_x_at	included	present	9051.03	-1.09	included	present	13174.37	0.25	included	present	8895.89	-1.14	included	present	6973.96	-1.76	included	present	10356.94	-0.66	included	present	8372.96	-1.31	included	present	7560.09	-1.57	included	present	8266.38	-1.34	included	present	11905.06	-0.16	included	present	12863.43	0.15	included	present	13437.21	0.34	included	present	10146.08	-0.73	included	present	11918.95	-0.16	included	present	14517.69	0.69	included	present	5690.79	-2.18
214036_at	included	present	234.01	-0.28	included	present	113.94	-0.81	included	present	1384.13	4.73	included	present	930.66	2.75	included	present	895.26	2.6	included	present	224.22	-0.33	included	present	1973.67	7.3	included	present	1182.48	3.85	included	present	441.02	0.62	included	present	628.84	1.44	included	present	1338.97	4.53	included	present	1233.27	4.07	included	present	601.68	1.32	included	present	1092.14	3.46	included	present	1007.84	3.09
214037_s_at	included	present	144.75	-1.41	included	absent	247.82	-0.1	included	absent	81.51	-2.21	included	present	113.33	-1.81	included	absent	204.22	-0.65	included	present	252.75	-0.03	included	present	326.11	0.9	included	present	377.24	1.55	included	present	340.86	1.09	included	present	368.68	1.44	included	present	275.13	0.25	included	present	646.19	4.97	included	absent	79.12	-2.24	included	present	223.64	-0.4	included	present	602.95	4.42
214038_at	included	present	217.83	0.27	excluded	absent	65.67	-0.33	excluded	absent	26.54	-0.48	excluded	absent	1.5	-0.58	included	present	167.04	0.07	included	present	2249.66	8.26	excluded	absent	29.03	-0.47	included	present	64.18	-0.34	included	present	109.5	-0.16	excluded	absent	55.19	-0.37	excluded	absent	22.85	-0.5	included	present	3598.72	13.57	included	present	285.92	0.54	included	present	171.78	0.09	included	present	990.38	3.31
214039_s_at	included	present	5462.25	-0.02	included	present	6855.5	0.46	included	present	14246.15	2.98	included	present	17524.31	4.09	included	present	5490.56	-0.01	included	present	4622.51	-0.3	included	present	9782.33	1.46	included	present	5360.3	-0.05	included	present	6755.17	0.43	included	present	4432.83	-0.37	included	present	5692.67	0.06	included	present	7801.52	0.78	included	present	9141.19	1.24	included	present	9759.72	1.45	included	present	7002.6	0.51
214040_s_at	included	present	308.68	1.02	excluded	absent	8	-0.81	excluded	absent	3.09	-0.84	excluded	absent	12.75	-0.78	excluded	absent	24.53	-0.71	excluded	absent	442.31	1.84	excluded	absent	85.17	-0.34	excluded	absent	126.43	-0.09	included	present	572.36	2.63	included	present	213.22	0.44	excluded	absent	15.06	-0.77	excluded	absent	57.13	-0.51	excluded	absent	10.32	-0.79	excluded	absent	32.27	-0.66	excluded	absent	55.59	-0.52
214041_x_at	included	present	34571.34	-0.81	included	present	34219.18	-0.84	included	present	49825.29	0.62	included	present	38067.66	-0.48	included	present	42848.18	-0.03	included	present	46819.1	0.34	included	present	31662.69	-1.08	included	present	26371.55	-1.58	included	present	23278.43	-1.87	included	present	44019.45	0.08	included	present	42362.94	-0.08	included	present	51310.75	0.76	included	present	54234.64	1.04	included	present	34669	-0.8	included	present	39812.5	-0.32
214042_s_at	included	present	8851.98	-1	included	present	12432.33	-0.28	included	present	9465.22	-0.88	included	present	6324.31	-1.51	included	present	12312.65	-0.3	included	present	10319.61	-0.7	included	present	7158.17	-1.34	included	present	15021.98	0.24	included	present	6619.48	-1.45	included	present	8377.15	-1.1	included	present	9189.88	-0.93	included	present	12947.34	-0.18	included	present	17697.8	0.78	included	present	19359.18	1.12	included	present	9123.8	-0.95
214043_at	included	present	864.52	-0.73	included	present	1821.71	-0.53	included	present	1984.86	-0.49	included	present	1992.5	-0.49	included	present	2439.91	-0.4	included	present	1207.66	-0.66	included	present	1292.22	-0.64	included	absent	2.24	-0.91	included	present	1077.43	-0.68	included	present	3831.05	-0.11	included	present	97.84	-0.89	included	present	2921.61	-0.3	included	present	7049.93	0.57	included	present	4941.62	0.13	included	present	1234.76	-0.65
214044_at	included	present	99.7	-0.44	included	present	221.53	-0.33	included	present	85.83	-0.45	excluded	absent	76.21	-0.46	excluded	absent	71.22	-0.46	excluded	absent	12.06	-0.51	included	present	122.54	-0.42	excluded	absent	6.97	-0.52	included	present	61.69	-0.47	included	present	1950.66	1.21	included	present	159.09	-0.38	included	present	1336.1	0.66	excluded	absent	90.33	-0.45	included	present	178.3	-0.37	included	present	229.05	-0.32
214045_at	included	present	640.05	-0.53	included	present	531.22	-0.76	included	present	1008.1	0.26	included	present	862.21	-0.05	included	present	850.41	-0.08	included	present	299.67	-1.26	included	present	972.61	0.18	included	present	397.84	-1.05	included	present	644.07	-0.52	included	present	684.19	-0.44	included	present	781.12	-0.23	included	present	346.97	-1.16	included	present	1795.34	1.95	included	present	1329.76	0.95	included	present	673.17	-0.46
214046_at	excluded	absent	50.04	-0.7	included	present	415.87	-0.25	excluded	absent	2.25	-0.76	excluded	absent	1.98	-0.76	excluded	absent	110.51	-0.63	excluded	absent	33.29	-0.72	included	present	284.55	-0.41	included	present	48.93	-0.7	included	present	217.84	-0.5	included	present	349.96	-0.33	included	present	76.81	-0.67	included	present	301.57	-0.39	included	present	750.72	0.16	included	present	606.08	-0.02	included	present	819.41	0.25
214047_s_at	included	present	936.38	-0.96	included	present	730.58	-1.4	included	present	1006.31	-0.81	included	present	1365.16	-0.04	included	present	466.74	-1.96	included	present	1248.44	-0.29	included	present	877.57	-1.08	included	present	1157.82	-0.49	included	present	1157.7	-0.49	included	present	1151.4	-0.5	included	present	797.7	-1.25	included	present	2094.25	1.51	included	present	1448.2	0.13	included	present	2084.72	1.49	included	present	1080.16	-0.65
214048_at	included	present	104.88	-0.62	included	present	168.1	-0.26	included	present	174.13	-0.22	included	present	382.47	0.96	included	absent	41.03	-0.98	included	absent	114.38	-0.56	included	present	127.84	-0.48	included	absent	62.69	-0.85	included	present	210.48	-0.02	included	present	136.6	-0.44	included	present	281.94	0.39	included	present	99.28	-0.65	included	present	259.34	0.26	included	present	434.98	1.26	included	present	135.94	-0.44
214049_x_at	excluded	absent	81.12	0.84	excluded	absent	23.08	-0.54	excluded	absent	8.2	-0.89	excluded	absent	5.26	-0.96	excluded	absent	8.55	-0.88	excluded	absent	89.42	1.03	excluded	absent	59.24	0.32	excluded	absent	5.82	-0.95	excluded	absent	71.82	0.62	excluded	absent	9.42	-0.86	excluded	absent	79.84	0.81	excluded	absent	52.64	0.16	excluded	absent	11.35	-0.82	included	present	97.66	1.23	excluded	absent	116.56	1.68
214050_at	included	present	978.93	0.57	included	present	481.02	-0.79	included	present	1869.99	3.01	included	present	588.82	-0.5	included	present	1391.9	1.7	included	present	655.27	-0.32	included	present	454.29	-0.87	included	present	706.12	-0.18	included	present	806.33	0.1	included	present	822.03	0.14	included	present	1158.32	1.06	included	present	724.82	-0.13	included	present	656.85	-0.31	included	present	233.26	-1.47	included	present	889.91	0.33
214051_at	included	present	280.55	-0.31	included	present	1547.23	3.32	included	present	2622.03	6.39	included	present	1106.19	2.06	included	present	1539.61	3.29	included	present	422.58	0.1	included	present	2329.53	5.55	included	present	1288.43	2.58	included	present	595.57	0.6	included	present	864.75	1.36	included	present	1757.21	3.92	included	present	3703.97	9.48	included	present	1765.65	3.94	included	present	1883.8	4.28	included	present	2085.2	4.86
214052_x_at	included	present	3809.5	-0.17	included	present	6481.81	1.98	included	present	4011.94	-0.01	included	present	4423.67	0.32	included	present	6058.34	1.64	included	present	5183.56	0.93	included	present	3393.83	-0.51	included	present	2816.75	-0.98	included	present	3238.28	-0.64	included	present	3389.16	-0.51	included	present	2238.75	-1.44	included	present	2905.42	-0.9	included	present	5001.51	0.79	included	present	3953.07	-0.06	included	present	1999.83	-1.64
214053_at	included	present	112.38	-0.85	included	present	152.3	-0.82	included	absent	2.29	-0.94	included	present	5357.15	3.34	included	present	1235.25	0.05	included	absent	5.43	-0.94	included	present	692.87	-0.39	included	absent	15.4	-0.93	included	absent	15.24	-0.93	included	present	3270.83	1.68	included	absent	37.39	-0.91	included	absent	1.47	-0.94	included	present	409.12	-0.61	included	present	1148.12	-0.02	included	absent	0.97	-0.94
214054_at	excluded	absent	104.44	0.15	excluded	absent	71.96	-0.23	excluded	absent	74.14	-0.21	excluded	absent	96.3	0.06	excluded	absent	58.93	-0.39	included	present	408.58	3.79	excluded	absent	124.3	0.39	excluded	absent	84.48	-0.08	excluded	absent	225.77	1.6	excluded	absent	74.19	-0.21	excluded	absent	125.61	0.41	excluded	absent	219.73	1.53	excluded	absent	110.58	0.23	excluded	absent	14.02	-0.93	included	present	359.4	3.2
214055_x_at	included	present	6418.86	-0.04	included	present	9105.01	1.36	included	present	8513.52	1.05	included	present	6376.61	-0.07	included	present	7599.25	0.57	included	present	4361.9	-1.12	included	present	5167.73	-0.7	included	present	3619.09	-1.51	included	present	3588.83	-1.52	included	present	6087.07	-0.22	included	present	4475.29	-1.06	included	present	3395.98	-1.62	included	present	9664.04	1.65	included	present	5124.25	-0.72	included	present	4005.42	-1.3
214056_at	included	present	3344.96	-0.19	included	present	2216.46	-0.61	included	present	3937.79	0.04	included	present	3806.72	-0.01	included	present	2831.88	-0.38	included	present	4213.53	0.14	included	present	1661.66	-0.82	included	present	1973.06	-0.7	included	present	2999.56	-0.32	included	present	2501.21	-0.5	included	present	2807.55	-0.39	included	present	2311.15	-0.57	included	present	1665.57	-0.82	included	present	1182.31	-1	included	present	2034.78	-0.68
214057_at	included	present	8976.09	-0.62	included	present	17162.85	0.59	included	present	9134.79	-0.59	included	present	9227.84	-0.58	included	present	6429.55	-0.99	included	present	10908.37	-0.33	included	present	4155.21	-1.33	included	present	3813.26	-1.38	included	present	5057.46	-1.2	included	present	5660.81	-1.11	included	present	3813.61	-1.38	included	present	7737.09	-0.8	included	present	9488.57	-0.54	included	present	5635.53	-1.11	included	present	4226.12	-1.32
214058_at	included	present	180.75	-0.23	included	present	366.34	0.11	included	absent	23.04	-0.51	included	present	4250.22	7.19	included	present	487.42	0.33	included	present	239.23	-0.12	included	present	186.54	-0.22	included	present	59.82	-0.45	included	present	188.73	-0.21	included	present	184.27	-0.22	included	present	122.27	-0.33	included	present	157.27	-0.27	included	present	183.55	-0.22	included	present	123.14	-0.33	included	present	286.78	-0.03
214059_at	included	present	416.74	1.32	included	absent	41.04	-1.05	included	present	124.81	-0.52	included	absent	2.02	-1.3	included	present	212.22	0.03	included	present	1079.51	5.51	included	absent	46.85	-1.01	included	present	55.84	-0.96	included	present	304	0.61	included	present	99.6	-0.68	included	present	128.69	-0.5	included	present	110.65	-0.61	included	present	138.37	-0.44	included	present	184.17	-0.15	included	absent	26.39	-1.14
214060_at	included	absent	105.75	-1.24	included	present	81.17	-1.48	included	present	109.48	-1.2	included	present	129.31	-1.01	included	present	273.51	0.41	included	present	97.9	-1.31	included	present	245.72	0.14	included	present	170.37	-0.6	included	present	273.76	0.41	included	present	167.14	-0.63	included	present	489.37	2.53	included	present	188.33	-0.43	included	present	311.91	0.79	included	present	661.39	4.22	included	present	156.28	-0.74
214061_at	included	present	214.26	-0.48	included	present	394.62	0.8	included	present	536.21	1.8	included	present	122.19	-1.13	included	absent	265.29	-0.12	included	absent	191.39	-0.64	included	present	349.41	0.48	included	present	426.43	1.02	included	present	240.81	-0.29	included	present	275.71	-0.05	included	present	624.52	2.42	included	present	306.87	0.17	included	present	476.91	1.38	included	present	604.8	2.28	included	present	659.46	2.67
214062_x_at	excluded	absent	164.77	-0.22	excluded	absent	90.63	-0.95	excluded	absent	9.91	-1.75	excluded	absent	41.37	-1.44	excluded	absent	154.26	-0.32	excluded	absent	33.21	-1.52	excluded	absent	114.09	-0.72	excluded	absent	55.43	-1.3	excluded	absent	46.07	-1.39	excluded	absent	51.96	-1.33	excluded	absent	65.17	-1.2	excluded	absent	217.7	0.3	excluded	absent	44.41	-1.41	excluded	absent	36.2	-1.49	excluded	absent	307.45	1.18
214063_s_at	included	present	489.52	-0.6	included	absent	72.55	-0.62	included	present	2001.24	-0.53	included	present	398.26	-0.61	included	present	430.99	-0.61	included	present	1309.14	-0.56	included	present	219.29	-0.62	included	absent	20.33	-0.63	included	present	519.56	-0.6	included	absent	192.25	-0.62	included	present	115.27	-0.62	included	present	714.77	-0.59	included	present	234.83	-0.62	included	present	303.07	-0.61	included	present	263.53	-0.61
214064_at	excluded	absent	6.17	-0.55	excluded	absent	23.37	-0.53	excluded	absent	1.96	-0.55	excluded	absent	29.54	-0.53	excluded	absent	3.66	-0.55	excluded	absent	44.27	-0.52	excluded	absent	103.2	-0.48	excluded	absent	33.82	-0.53	excluded	absent	14.17	-0.54	excluded	absent	46.36	-0.52	excluded	absent	42.97	-0.52	excluded	absent	32.28	-0.53	excluded	absent	42.52	-0.52	excluded	absent	3.31	-0.55	excluded	absent	1.94	-0.55
214065_s_at	excluded	absent	10.98	-0.74	excluded	absent	145.62	2.29	excluded	absent	47.44	0.08	excluded	absent	20.56	-0.52	excluded	absent	31.97	-0.27	excluded	absent	98.4	1.23	included	present	242.37	4.47	included	present	431.39	8.72	included	present	176.24	2.98	included	present	275.18	5.21	included	present	409.81	8.23	included	present	243.8	4.5	excluded	absent	8.53	-0.79	excluded	absent	4.07	-0.89	included	present	628.9	13.16
214066_x_at	included	present	451.95	-0.16	included	present	425.98	-0.24	included	present	316.13	-0.56	included	present	164.02	-1.01	included	present	395.48	-0.33	included	present	449.79	-0.17	included	present	472.17	-0.1	included	present	873.81	1.08	included	present	513.83	0.02	included	present	537.22	0.09	included	present	290.42	-0.64	included	present	543.68	0.11	included	present	290.73	-0.64	included	present	872.64	1.08	included	present	718.63	0.62
214067_at	excluded	absent	3.64	-0.67	excluded	absent	30.67	0.16	excluded	absent	8.92	-0.51	excluded	absent	5.28	-0.62	excluded	absent	12.96	-0.39	excluded	absent	85.32	1.85	excluded	absent	104.05	2.43	excluded	absent	23.62	-0.06	excluded	absent	49.85	0.75	excluded	absent	43.01	0.54	excluded	absent	8.94	-0.51	excluded	absent	62.33	1.14	excluded	absent	5.02	-0.63	excluded	absent	7.28	-0.56	excluded	absent	16.56	-0.28
214068_at	included	present	273.41	4.94	excluded	absent	3.53	-1.21	excluded	absent	21.9	-0.79	excluded	absent	69.48	0.3	excluded	absent	112.56	1.28	excluded	absent	40.01	-0.38	included	present	103.07	1.06	excluded	absent	1.46	-1.25	excluded	absent	16.65	-0.91	excluded	absent	16.36	-0.91	excluded	absent	2.54	-1.23	excluded	absent	4.27	-1.19	excluded	absent	54.92	-0.04	excluded	absent	12.78	-1	excluded	absent	3.58	-1.21

[illegible]

214394_x_at	included	present	30354.24	-0.86	included	present	29898.78	-0.88	included	present	43803.97	-0.16	included	present	22971.02	-1.25	included	present	31704.04	-0.79	included	present	34436.83	-0.65	included	present	12766.61	-1.78	included	present	19424.79	-1.43	included	present	14563.63	-1.69	included	present	17267.46	-1.55	included	present	15074.49	-1.66	included	present	36663.3	-0.53	included	present	35825.39	-0.57	included	present	33506.6	-0.69	included	present	14505.5	-1.69
214395_x_at	included	present	29943.32	-0.76	included	present	26263.46	-0.97	included	present	43842.93	0.02	included	present	23400.41	-1.13	included	present	28859.27	-0.83	included	present	30683.58	-0.72	included	present	12560.92	-1.74	included	present	18626.19	-1.4	included	present	11814.12	-1.78	included	present	15723.17	-1.56	included	present	16143.96	-1.54	included	present	35844.47	-0.43	included	present	31454.65	-0.68	included	present	28271.15	-0.86	included	present	14094.75	-1.65
214396_s_at	excluded	absent	67.1	-0.34	excluded	absent	34.44	-0.86	excluded	absent	39.53	-0.78	excluded	absent	40.77	-0.76	excluded	absent	5.21	-1.33	included	present	113.91	0.41	included	present	117.61	0.47	excluded	absent	110.56	0.36	excluded	absent	118.85	0.49	excluded	absent	60.68	-0.44	included	present	169.94	1.31	included	present	123.73	0.57	excluded	absent	82.05	-0.1	excluded	absent	46.05	-0.68	included	present	51.67	-0.59
214397_at	excluded	absent	1.38	-0.39	included	present	315.2	0.26	excluded	absent	54.43	-0.28	excluded	absent	57.55	-0.27	included	present	208.83	0.04	excluded	absent	65.08	-0.26	included	present	118.01	-0.15	included	present	34	-0.32	included	present	71.38	-0.24	included	present	36.84	-0.32	included	present	324.31	0.28	excluded	absent	66.32	-0.25	included	present	265.58	0.16	included	present	257.22	0.14	included	present	173.99	-0.03
214398_s_at	excluded	absent	125.3	0.7	excluded	absent	11.04	-0.99	excluded	absent	104.86	0.4	excluded	absent	55.59	-0.33	excluded	absent	85.62	0.11	included	present	196.68	1.77	excluded	absent	53.24	-0.37	excluded	absent	47.23	-0.46	excluded	absent	60.4	-0.26	excluded	absent	8.57	-1.03	excluded	absent	28.89	-0.73	excluded	absent	65.53	-0.18	excluded	absent	23.59	-0.81	excluded	absent	4.44	-1.09	excluded	absent	59.41	-0.28
214399_s_at	excluded	absent	3.47	-0.15	excluded	absent	37.81	-0.15	excluded	absent	5.96	-0.15	excluded	absent	3.07	-0.15	excluded	absent	5.77	-0.15	excluded	absent	10.13	-0.15	excluded	absent	2.64	-0.16	excluded	absent	4.9	-0.15	excluded	absent	7.45	-0.15	excluded	absent	10.94	-0.15	excluded	absent	12.63	-0.15	excluded	absent	9.26	-0.15	excluded	absent	3.5	-0.15	excluded	absent	21.09	-0.15	excluded	absent	3.11	-0.15
214400_at	excluded	absent	89.09	0.1	excluded	absent	19.69	-0.3	excluded	absent	7.21	-0.37	excluded	absent	73.57	0.01	excluded	absent	13.65	-0.33	excluded	absent	41.58	-0.17	excluded	absent	8.96	-0.36	excluded	absent	16.07	-0.32	excluded	absent	44.98	-0.15	excluded	absent	13.72	-0.33	excluded	absent	16.77	-0.32	excluded	absent	13.77	-0.33	excluded	absent	5.31	-0.38	excluded	absent	7.97	-0.37	excluded	absent	27.68	-0.25
214401_at	excluded	absent	5.95	-0.29	excluded	absent	41.26	-0.13	excluded	absent	2.13	-0.31	excluded	absent	5.73	-0.29	excluded	absent	71.79	0.01	excluded	absent	227.79	0.72	excluded	absent	11.55	-0.26	excluded	absent	3.09	-0.3	excluded	absent	5.74	-0.29	excluded	absent	74.15	0.02	excluded	absent	4.87	-0.29	excluded	absent	32.46	-0.17	excluded	absent	9.01	-0.28	excluded	absent	51.16	-0.08	excluded	absent	7.41	-0.28
214402_s_at	excluded	absent	12.62	-0.64	excluded	absent	40.53	-0.17	excluded	absent	5.27	-0.77	excluded	absent	122.8	1.21	excluded	absent	73.84	0.38	excluded	absent	23.84	-0.45	excluded	absent	91.48	0.68	excluded	absent	83.21	0.54	excluded	absent	32.09	-0.32	excluded	absent	177.27	2.12	excluded	absent	88.92	0.64	excluded	absent	48.8	-0.04	excluded	absent	133.01	1.38	included	present	316.13	4.45	excluded	absent	78.29	0.46
214403_x_at	excluded	absent	11.88	-0.77	excluded	absent	96.03	-0.06	excluded	absent	39.27	-0.54	excluded	absent	9.67	-0.79	excluded	absent	28.76	-0.63	excluded	absent	51.82	-0.43	excluded	absent	30.09	-0.62	excluded	absent	126.79	0.19	excluded	absent	141.82	0.32	excluded	absent	121.14	0.15	excluded	absent	119.69	0.13	excluded	absent	186.16	0.69	excluded	absent	95.71	-0.07	excluded	absent	52.42	-0.43	excluded	absent	9.38	-0.79
214404_x_at	excluded	absent	137.63	0.53	excluded	absent	8.33	-0.49	excluded	absent	78.62	0.06	excluded	absent	17.86	-0.42	excluded	absent	154.15	0.66	excluded	absent	150.96	0.64	excluded	absent	19.67	-0.4	excluded	absent	8.06	-0.5	excluded	absent	16.47	-0.43	excluded	absent	26.42	-0.35	excluded	absent	20.3	-0.4	excluded	absent	72.46	0.01	excluded	absent	55.53	-0.12	excluded	absent	77.39	0.05	excluded	absent	33.2	-0.3
214405_at	included	present	163.76	-0.21	excluded	absent	9.93	-1	excluded	absent	12.9	-0.98	excluded	absent	37.4	-0.86	excluded	absent	144.84	-0.31	excluded	absent	67.01	-0.71	included	present	98.05	-0.55	excluded	absent	43.88	-0.83	included	present	160.56	-0.23	excluded	absent	66.96	-0.71	included	present	127.17	-0.4	included	present	180.45	-0.13	included	present	107.77	-0.5	included	present	264.13	0.3	excluded	absent	28.74	-0.9
214406_s_at	excluded	absent	8.22	-1.06	excluded	absent	56.24	0.22	excluded	absent	10.27	-1.01	excluded	absent	2.23	-1.22	excluded	absent	6.58	-1.1	excluded	absent	36.8	-0.3	excluded	absent	2.64	-1.21	excluded	absent	95.18	1.26	excluded	absent	46.63	-0.04	included	present	59.23	0.3	excluded	absent	4.48	-1.16	excluded	absent	80.42	0.87	excluded	absent	7.48	-1.08	excluded	absent	16.21	-0.85	excluded	absent	36.83	-0.3
214407_x_at	excluded	absent	11.52	-0.79	excluded	absent	105.32	0.67	excluded	absent	5.55	-0.88	excluded	absent	32.45	-0.47	included	present	347.16	4.43	excluded	absent	2.95	-0.92	excluded	absent	11.45	-0.79	excluded	absent	19.52	-0.67	included	present	216.71	2.4	excluded	absent	11.21	-0.8	excluded	absent	5.32	-0.89	included	present	1763.94	26.44	excluded	absent	73.03	0.17	excluded	absent	68.55	0.1	excluded	absent	33.54	-0.45
214408_s_at	included	present	207.08	-0.23	excluded	absent	174.47	-0.37	excluded	absent	63.35	-0.85	excluded	absent	72.21	-0.81	excluded	absent	143.4	-0.51	excluded	absent	100.51	-0.69	included	present	341.38	0.34	excluded	absent	183.78	-0.33	included	present	278.67	0.07	excluded	absent	443.55	0.78	included	present	241.49	-0.09	excluded	absent	219.07	-0.18	excluded	absent	235	-0.11	included	present	273.95	0.05	included	present	341.84	0.34
214409_at	excluded	absent	29.51	-0.24	excluded	absent	46.47	-0.19	excluded	absent	2.12	-0.32	included	present	87	-0.08	excluded	absent	68.68	-0.13	excluded	absent	30.09	-0.24	excluded	absent	90.23	-0.07	excluded	absent	38.83	-0.21	excluded	absent	27.81	-0.24	included	present	63.12	-0.14	included	present	130.37	0.04	included	present	116.18	0	included	present	133.52	0.05	included	present	215.26	0.28	included	present	51.63	-0.18
214410_at	excluded	absent	9.33	-0.5	excluded	absent	7.32	-0.54	excluded	absent	38.38	-0.05	excluded	absent	28.37	-0.2	excluded	absent	7.64	-0.53	excluded	absent	8.66	-0.51	excluded	absent	44	0.04	excluded	absent	26.41	-0.23	excluded	absent	0.84	-0.64	excluded	absent	2	-0.62	excluded	absent	1.7	-0.62	excluded	absent	1.46	-0.63	excluded	absent	1.45	-0.63	excluded	absent	16.58	-0.39	excluded	absent	18.48	-0.36
214411_x_at	excluded	absent	2.2	-0.63	excluded	absent	3.84	-0.53	excluded	absent	6.67	-0.37	excluded	absent	3.43	-0.56	excluded	absent	6.17	-0.4	excluded	absent	26.63	0.81	excluded	absent	3.02	-0.58	excluded	absent	1.93	-0.65	excluded	absent	6.42	-0.38	excluded	absent	29.78	0.99	excluded	absent	9.06	-0.23	excluded	absent	3.99	-0.52	excluded	absent	6.8	-0.36	excluded	absent	1.98	-0.64	excluded	absent	1.12	-0.69
214412_at	excluded	absent	7.75	-1.1	excluded	absent	177.79	2.22	excluded	absent	30.11	-0.66	excluded	absent	26.89	-0.72	excluded	absent	19.01	-0.88	excluded	absent	52.24	-0.23	excluded	absent	79.47	0.3	excluded	absent	2.52	-1.2	excluded	absent	85.02	0.41	excluded	absent	74.3	0.2	excluded	absent	59.99	-0.08	excluded	absent	15.99	-0.94	excluded	absent	59.67	-0.08	excluded	absent	1.92	-1.21	excluded	absent	55.53	-0.17
214413_at	excluded	absent	50.01	-0.15	excluded	absent	4.17	-0.15	included	present	166.87	-0.14	excluded	absent	31.53	-0.15	excluded	absent	10.37	-0.15	excluded	absent	31.26	-0.15	excluded	absent	7.89	-0.15	excluded	absent	33.49	-0.15	excluded	absent	18.75	-0.15	excluded	absent	3.33	-0.15	excluded	absent	54.03	-0.15	excluded	absent	2.33	-0.15	excluded	absent	27.15	-0.15	excluded	absent	12.64	-0.15	excluded	absent	37.93	-0.15
214414_x_at	included	present	9525.79	0.64	included	present	151.73	-0.7	included	present	5329.35	0.04	included	present	1522.61	-0.5	included	present	49237.2	6.3	included	present	6613.48	0.22	included	present	1252.63	-0.54	included	present	2814.94	-0.32	included	present	28949.42	3.4	included	present	7527	0.35	included	present	2910.21	-0.31	included	present	48059.08	6.13	included	present	3209.13	-0.26	included	present	285.12	-0.68	included	present	3279.86	-0.25
214415_at	included	present	599.54	-0.04	included	present	422.41	-0.17	included	present	673.66	0.01	included	present	1147.35	0.33	included	present	468.08	-0.13	included	present	531.89	-0.09	included	present	657.62	0	included	present	333.42	-0.23	included	present	477.62	-0.13	included	present	696.04	0.02	included	present	789.64	0.09	included	present	700.53	0.03	included	present	2358.49	1.16	included	present	663.6	0	included	present	371.59	-0.2
214416_at	excluded	absent	1.88	-0.79	excluded	absent	2.7	-0.76	excluded	absent	3.72	-0.72	excluded	absent	37.34	0.63	excluded	absent	3.02	-0.75	excluded	absent	1.95	-0.79	excluded	absent	2.12	-0.78	excluded	absent	1.98	-0.79	excluded	absent	4.14	-0.7	excluded	absent	6.44	-0.61	excluded	absent	4.65	-0.68	excluded	absent	3.44	-0.73	excluded	absent	6.62	-0.6	excluded	absent	4.44	-0.69	excluded	absent	0.76	-0.84
214417_s_at	excluded	absent	10.24	-0.45	excluded	absent	18.11	-0.4	excluded	absent	35.5	-0.3	excluded	absent	6.52	-0.48	excluded	absent	6.13	-0.48	excluded	absent	88.45	0.03	excluded	absent	5.13	-0.48	excluded	absent	29.3	-0.33	included	present	87.94	0.03	excluded	absent	43.89	-0.24	excluded	absent	8.1	-0.47	included	present	100.38	0.11	excluded	absent	44.41	-0.24	excluded	absent	46.65	-0.23	excluded	absent	26.65	-0.35
214418_at	excluded	absent	2.03	-0.29	excluded	absent	12.31	-0.24	excluded	absent	74.86	0.04	excluded	absent	77.76	0.05	excluded	absent	25.47	-0.19	excluded	absent	5.78	-0.27	excluded	absent	46.91	-0.09	excluded	absent	3.05	-0.29	excluded	absent	9.75	-0.26	excluded	absent	3.74	-0.28	excluded	absent	4	-0.28	excluded	absent	2.9	-0.29	excluded	absent	7.21	-0.27	excluded	absent	47.31	-0.09	excluded	absent	7.98	-0.26
214419_s_at	excluded	absent	79.81	-0.18	excluded	absent	52.44	-0.19	included	present	449.02	-0.1	excluded	absent	32.25	-0.19	excluded	absent	90.11	-0.18	included	present	105.6	-0.17	excluded	absent	66.62	-0.18	excluded	absent	4.27	-0.2	excluded	absent	59.9	-0.18	excluded	absent	12.03	-0.19	excluded	absent	61.07	-0.18	included	present	168.72	-0.16	excluded	absent	2.47	-0.2	excluded	absent	8.63	-0.19	excluded	absent	21.61	-0.19
214420_s_at	excluded	absent	4.54	-0.18	excluded	absent	88.25	-0.17	included	present	365.85	-0.12	excluded	absent	58.05	-0.17	excluded	absent	3.5	-0.18	excluded	absent	22.86	-0.18	excluded	absent	3.94	-0.18	excluded	absent	2.01	-0.18	excluded	absent	8.04	-0.18	excluded	absent	3.64	-0.18	excluded	absent	46.85	-0.18	excluded	absent	12.93	-0.18	excluded	absent	27.41	-0.18	excluded	absent	3.21	-0.18	excluded	absent	4.77	-0.18
214421_x_at	excluded	absent	32.29	-0.18	excluded	absent	2.22	-0.19	included	present	325.11	-0.11	excluded	absent	68.58	-0.18	excluded	absent	49.87	-0.18	included	present	129.26	-0.16	excluded	absent	62.15	-0.18	excluded	absent	21.31	-0.19	excluded	absent	5.71	-0.19	excluded	absent	5.04	-0.19	excluded	absent	3.45	-0.19	excluded	absent	66.04	-0.18	excluded	absent	2.36	-0.19	excluded	absent	2.1	-0.19	excluded	absent	3.57	-0.19
214422_at	included	present	190.29	-0.12	included	absent	46.69	-1.96	included	present	263.42	0.82	included	present	201.95	0.03	included	present	169.85	-0.38	included	absent	90.87	-1.4	included	present	226.01	0.34	included	absent	63.68	-1.75	included	present	249.58	0.65	included	present	101.8	-1.25	included	present	354.77	2	included	absent	55.39	-1.85	included	absent	108.72	-1.17	included	present	65.37	-1.72	included	present	84	-1.48
214423_x_at	excluded	absent	91.56	-0.26	excluded	absent	3.48	-0.27	included	present	1287.58	-0.18	excluded	absent	15.47	-0.27	excluded	absent	6.17	-0.27	excluded	absent	9.47	-0.27	excluded	absent	39.75	-0.27	excluded	absent	3.76	-0.27	excluded	absent	45.98	-0.27	excluded	absent	84.61	-0.26	excluded	absent	55.67	-0.27	excluded	absent	88.04	-0.26	excluded	absent	7.34	-0.27	excluded	absent	7.21	-0.27	excluded	absent	7.3	-0.27
214424_s_at	excluded	absent	6.04	-0.27	excluded	absent	2.21	-0.27	included	present	168.07	-0.24	excluded	absent	64.42	-0.26	excluded	absent	2.3	-0.27	excluded	absent	5.7	-0.27	excluded	absent	8.78	-0.26	excluded	absent	45.19	-0.26	excluded	absent	2.69	-0.27	excluded	absent	45.41	-0.26	excluded	absent	41.24	-0.26	excluded	absent	28.14	-0.26	excluded	absent	1.93	-0.27	excluded	absent	2.95	-0.27	excluded	absent	15.13	-0.26
214425_at	excluded	absent	6.41	-0.16	excluded	absent	41.62	-0.15	included	present	302.37	-0.05	excluded	absent	87.66	-0.13	excluded	absent	31.64	-0.15	excluded	absent	124.32	-0.12	excluded	absent	57.48	-0.14	excluded	absent	13.91	-0.16	excluded	absent	58.42	-0.14	excluded	absent	6.42	-0.16	excluded	absent	72.01	-0.14	excluded	absent	26.9	-0.15	excluded	absent	19.66	-0.16	excluded	absent	4.82	-0.16	excluded	absent	14.12	-0.16
214426_x_at	included	present	206.19	-0.66	included	present	891.48	4.11	included	present	985.04	4.76	included	present	993.14	4.82	excluded	absent	513.9	1.48	included	present	176.52	-0.87	included	present	163.81	-0.96	included	present	792.96	3.43	included	absent	139.53	-1.13	included	present	253.13	-0.34	included	present	668.19	2.56	included	present	790.02	3.41	included	present	414.54	0.79	included	present	416.07	0.8	included	present	599.83	2.08
214427_at	excluded	absent	120.54	-0.58	included	present	287.42	1.02	excluded	absent	178.04	-0.03	excluded	absent	48.81	-1.26	excluded	absent	132.68	-0.46	included	present	434.63	2.43	excluded	absent	182.1	0.01	included	present	879.54	6.68	included	present	302.01	1.16	included	present	595.58	3.97	included	present	754.43	5.48	included	present	325.19	1.38	included	present	394.82	2.05	included	present	301.74	1.16	included	present	490.12	2.96
[illegible]																																																													

214756_x_at	included	present	1512.86	0.98	included	present	1960.74	2.13	included	present	1633.35	1.28	included	present	1144.12
214757_at	included	present	153.19	0.46	excluded	absent	152.3	0.45	included	present	205.83	1.08	excluded	absent	70.74
214758_at	excluded	absent	104.43	1.85	excluded	absent	77.84	1.1	excluded	absent	100.85	1.75	excluded	absent	13.31
214759_at	included	present	219.05	-0.69	included	present	163.55	-1.14	included	present	277.49	-0.21	included	present	342.21
214760_at	included	present	1251.4	-0.89	included	present	1963.88	0.05	included	present	2512.44	0.76	included	present	2327.43
214761_at	included	present	267.32	-1.2	included	present	856.57	0.29	included	present	3322	6.56	included	present	159.33
214762_at	included	present	1844.31	0.76	included	present	978.54	0.06	included	present	356.27	-0.44	included	present	209.71
214763_at	excluded	absent	45.44	-0.48	included	present	355.17	0.21	excluded	absent	20.77	-0.54	included	present	280.83
214764_at	included	present	291.99	-0.18	included	present	961.42	4.28	included	present	1134.82	5.44	included	present	1033.01
214765_s_at	included	present	455.04	0.75	included	absent	183.93	-0.55	included	present	152.56	-0.7	included	present	237.54
214766_s_at	included	present	771.53	-0.12	included	present	596.33	-0.31	included	present	1203.16	0.33	included	present	1401.13
214767_s_at	included	present	927.45	-0.3	included	present	414.79	-0.4	included	present	143.27	-0.46	included	present	709.17
214768_x_at	included	present	135.51	-0.31	excluded	absent	14.6	-0.37	excluded	absent	53.56	-0.35	excluded	absent	82.41
214769_at	included	present	421.41	-0.6	included	absent	83.13	-0.87	included	present	419.37	-0.6	included	present	209.32
214770_at	excluded	absent	99.57	0.73	included	present	105.79	0.86	included	present	77.18	0.28	excluded	absent	56.98
214771_x_at	included	present	3090.35	0.71	included	present	2072.3	-0.26	included	present	2914.94	0.54	included	present	1097.63
214772_at	included	present	246.77	-0.04	included	present	334.31	0.22	included	present	157.57	-0.3	included	present	175.66
214773_x_at	included	present	736.58	-1.28	included	present	3089.25	1.53	included	present	3718.47	2.29	included	present	2998.54
214774_x_at	excluded	absent	69.12	-0.36	included	present	1384.75	7.4	included	present	625.66	2.92	included	present	856.45
214775_at	excluded	absent	158.97	1.33	excluded	absent	98.06	0.3	included	present	179.68	1.68	excluded	absent	128.5
214776_x_at	included	present	282.03	0.1	included	present	161.09	-0.28	included	present	587.4	1.07	included	present	323.16
214777_at	excluded	absent	15.18	-0.39	excluded	absent	6.08	-0.4	excluded	absent	11.99	-0.4	excluded	absent	132.03
214778_at	included	present	902.93	0.67	included	present	571.02	-0.04	included	present	427.18	-0.35	included	present	624.9
214779_s_at	included	present	223.87	-0.96	included	present	451.3	1.2	included	present	226.67	-0.94	included	present	170.82
214780_s_at	included	present	249.81	1.19	included	present	217.61	0.8	excluded	absent	116.71	-0.44	included	present	174.16
214781_at	included	present	225.67	-0.37	included	present	378.31	-0.19	included	present	272.76	-0.31	included	present	413.91
214782_at	included	present	270.61	-0.09	included	absent	138.84	-1.01	included	present	256.69	-0.19	included	present	231.96
214783_s_at	included	present	262.35	-1.01	included	absent	161.04	-1.4	included	absent	115.14	-1.58	included	present	304.27
214784_x_at	included	present	393.6	0.13	included	present	532.83	1.15	included	present	228.31	-1.09	included	present	126.31
214785_at	included	present	204.64	-0.7	included	present	227.89	-0.64	included	present	384.42	-0.24	included	present	475.94
214786_at	included	present	822.87	1.29	included	absent	142.09	-0.57	included	present	537.15	0.51	included	present	416.73
214787_at	included	present	129.72	-0.54	excluded	absent	90.07	-1.21	excluded	absent	100.74	-1.03	included	present	164.89
214788_x_at	excluded	absent	5.62	-0.66	excluded	absent	50.95	-0.61	excluded	absent	58.63	-0.6	excluded	absent	14.84
214789_x_at	included	present	1906.21	0.2	included	present	2203.54	0.87	included	present	1606.01	-0.47	included	present	1524.2

215117_at	excluded	absent	1.98	-0.88	excluded	absent	2.14	-0.87	excluded	absent	31.82	0.11	excluded	absent	13.81
215118_s_at	excluded	absent	3.22	-0.45	excluded	absent	8.71	-0.43	excluded	absent	5.56	-0.44	excluded	absent	15.47
215119_at	excluded	absent	40.83	-0.62	excluded	absent	184.59	0.1	excluded	absent	3.35	-0.81	included	present	802.86
215120_s_at	included	present	414.48	1.05	excluded	absent	43.94	-1.33	excluded	absent	232.04	-0.12	excluded	absent	92.08
215121_x_at	excluded	absent	273.67	-0.36	excluded	absent	213.05	-0.36	excluded	absent	24.33	-0.37	excluded	absent	32.54
215122_at	included	present	79.48	-0.53	excluded	absent	90.98	-0.31	excluded	absent	116.13	0.18	included	present	99.8
215123_at	included	present	1627.99	0.17	included	present	792.58	-0.85	included	present	2310.63	1	included	present	727.45
215124_at	included	absent	120.44	-0.59	included	absent	61.41	-1.48	excluded	absent	203.14	0.67	included	present	375.43
215125_s_at	excluded	absent	7.49	-0.27	excluded	absent	33.53	-0.26	included	present	661.69	-0.14	excluded	absent	70.16
215126_at	included	present	320.32	-0.04	excluded	absent	8.15	-0.41	excluded	absent	2.93	-0.42	excluded	absent	1.94
215127_s_at	included	present	4238.44	0.59	included	present	1449.92	-0.63	included	present	3627.89	0.32	included	present	444.17
215128_at	included	present	611	2.12	included	present	1108.21	5.79	included	present	690.63	2.7	included	present	673.87
215129_at	excluded	absent	34	-0.31	excluded	absent	2.58	-0.48	excluded	absent	5.87	-0.47	excluded	absent	2.11
215130_s_at	excluded	absent	87.52	-0.51	excluded	absent	118.9	-0.13	excluded	absent	59.25	-0.85	excluded	absent	89.34
215131_at	excluded	absent	47.87	-1.3	excluded	absent	56.29	-1.2	excluded	absent	74.86	-0.99	included	present	97.74
215132_at	excluded	absent	99.95	1.05	excluded	absent	32.83	-0.68	excluded	absent	35.17	-0.62	excluded	absent	56.6
215133_s_at	included	absent	104.15	-0.47	included	absent	46.94	-0.52	included	absent	3.81	-0.56	included	present	313.5
215134_at	included	present	384.54	1.92	included	present	349.65	1.51	included	present	327.92	1.26	included	present	98.37
215135_at	excluded	absent	36.74	-0.98	excluded	absent	38.21	-0.95	excluded	absent	62.92	-0.47	excluded	absent	74.75
215136_s_at	included	present	1440.17	-0.39	included	present	2553.32	1.11	included	present	2662.55	1.25	included	present	1567.89
215137_at	included	present	254.14	1.83	excluded	absent	58.18	-0.81	excluded	absent	67.77	-0.68	excluded	absent	16.1
215138_s_at	excluded	absent	5.05	-1.46	excluded	absent	106.92	0	excluded	absent	83.63	-0.33	excluded	absent	54.16
215139_at	included	present	481.18	2.21	included	absent	76.04	-0.92	included	present	174.94	-0.16	included	present	482.21
215140_at	excluded	absent	104.17	-0.5	excluded	absent	124.92	-0.26	excluded	absent	42.96	-1.21	excluded	absent	91.28
215141_at	included	present	157.37	0.76	excluded	absent	33.52	-0.98	excluded	absent	126.84	0.33	excluded	absent	76.11
215142_at	excluded	absent	6.31	-0.27	excluded	absent	11.02	-0.25	excluded	absent	7.41	-0.27	excluded	absent	5.13
215143_at	included	present	248.99	-0.59	included	present	1225.52	1.48	included	present	1330.19	1.7	included	present	1727.03
215144_at	excluded	absent	208.99	0.31	excluded	absent	12.16	-1.24	excluded	absent	78.69	-0.71	excluded	absent	40.85
215145_s_at	excluded	absent	91.01	-0.11	included	present	229.07	1.24	excluded	absent	43.55	-0.57	included	present	1036.21
215146_s_at	included	present	538.73	0.21	included	present	340.76	-0.85	included	present	669.99	0.91	included	present	673.94
215147_at	included	present	107.27	-0.29	excluded	absent	75.22	-0.64	excluded	absent	42.21	-1	excluded	absent	5.89
215148_s_at	excluded	absent	65.79	-0.28	excluded	absent	28.61	-0.94	excluded	absent	4.31	-1.38	excluded	absent	41.72
215149_at	included	present	179.74	-0.5	included	present	527.64	0.09	excluded	absent	88.37	-0.66	excluded	absent	43.48
215150_at	included	present	275	0.6	included	absent	135.87	-0.51	included	present	117.72	-0.65	included	present	293.65
215151_at	excluded	absent	33.71	-0.5	excluded	absent	41.93	-0.4	excluded	absent	2.67	-0.86	excluded	absent	2.71
215152_at	excluded	absent	20.37	-0.68	excluded	absent	5.39	-0.89	excluded	absent	94.03	0.36	included	present	69.71
215153_at	included	present	261.72	-0.1	included	present	1024.49	2.64	included	present	767.68	1.72	included	present	432.45
215154_at	included	present	182.55	-0.61	included	present	470.05	2.1	included	present	350.05	0.97	included	present	271.87
215155_at	included	present	364.97	-0.03	included	present	358.98	-0.07	included	present	300.25	-0.44	included	present	211.15
215156_at	included	present	260.47	0.03	included	present	281.37	0.22	included	present	329.27	0.66	included	absent	131.62
215157_x_at	included	present	7164.96	0.13	included	present	6115.56	-0.09	included	present	15325.85	1.85	included	present	8821.67
215158_s_at	included	present	679.21	-0.25	included	present	1069.26	1.66	included	present	955.19	1.1	included	present	790.82
215159_s_at	excluded	absent	12.87	-1.05	excluded	absent	75.37	-0.49	excluded	absent	73.46	-0.51	excluded	absent	11
215160_x_at	included	present	3521.19	-0.05	included	present	3743.19	0.15	included	present	4539.54	0.84	included	present	2053.01
215161_at	excluded	absent	6.46	-0.71	excluded	absent	32.54	-0.51	excluded	absent	2.48	-0.74	excluded	absent	2.5
215162_at	included	present	254.66	-0.35	included	present	183.08	-0.99	included	present	255.92	-0.34	included	present	285.67
215163_at	included	present	110.13	-0.69	included	absent	132.35	-0.5	included	present	87.89	-0.88	included	present	112.54
215164_at	excluded	absent	48.64	-0.72	excluded	absent	4.05	-1.68	excluded	absent	32.4	-1.07	excluded	absent	8.69
215165_x_at	included	present	557.55	-0.22	included	present	1133.17	1.57	included	present	2189.1	4.85	included	present	1571.45
215166_at	excluded	absent	10.57	-0.71	excluded	absent	17.67	-0.56	excluded	absent	83.57	0.77	excluded	absent	55.41
215167_at	included	present	205.57	-0.12	included	present	249.85	0.26	included	absent	124.68	-0.82	included	present	128.22
215168_at	excluded	absent	71.36	0.08	excluded	absent	39.27	-0.7	excluded	absent	92.44	0.59	included	present	95.51
215169_at	excluded	absent	41.2	-1.15	excluded	absent	113.61	-0.09	included	present	119.83	0.01	included	present	80.72
215170_s_at	excluded	absent	62.4	-0.45	included	present	149.43	0.73	included	present	478.13	5.2	included	present	153.36
215171_s_at	included	present	2660.32	-0.59	included	present	8247.86	3.41	included	present	4141.12	0.47	included	present	4383.26
215172_at	included	present	139.21	0.24	included	present	180.77	0.61	included	present	456.26	3.09	excluded	absent	6.42
215173_at	included	present	147.24	0.94	included	present	174.33	1.49	excluded	absent	68.5	-0.65	excluded	absent	9.43
215174_at	included	present	158.53	0.91	excluded	absent	4.95	-0.34	excluded	absent	5.4	-0.34	excluded	absent	1.35
215175_at	included	present	211.5	-0.16	included	absent	64.18	-1.17	included	present	253.41	0.12	included	present	243.07
215176_x_at	excluded	absent	44.92	-0.35	excluded	absent	192.62	-0.32	excluded	absent	48.22	-0.35	excluded	absent	80.33
215177_s_at	included	present	16108.95	5.65	included	present	542.28	-1.29	included	present	1035.29	-1.07	included	present	446.96
215178_x_at	included	present	337.17	-0.73	included	present	525.62	0.5	included	present	330.67	-0.77	included	present	356.69
215179_x_at	included	present	1732.77	-0.55	included	present	1439.4	-1.02	included	present	1979.76	-0.16	included	present	1389.92
215180_at	excluded	absent	25.04	-0.81	included	present	163.08	0.66	included	present	388.71	3.05	excluded	absent	11.98
215181_at	excluded	absent	10.69	-0.79	included	present	389.79	0.89	excluded	absent	4.63	-0.81	included	present	268.93
215182_x_at	included	present	2933.89	-0.01	included	present	2859.12	-0.08	included	present	3666.18	0.72	included	present	2266.21
215183_at	included	present	411.68	-0.4	included	present	199.38	-1.49	included	present	697.25	1.07	included	present	236.2
215184_at	included	present	310.51	-0.63	included	present	532.1	-0.33	included	present	152.86	-0.84	included	present	406.97
215185_at	excluded	absent	125.73	0.79	excluded	absent	13.41	-1.15	excluded	absent	10.67	-1.19	excluded	absent	37.68
215186_at	excluded	absent	49.42	-0.86	included	present	141.06	0.59	excluded	absent	134.49	0.49	included	present	192.64
215187_at	included	present	215.55	0.41	excluded	absent	214.85	0.4	excluded	absent	78	-1.68	excluded	absent	67.58
215188_at	included	present	144.93	-0.39	included	present	184.46	-0.23	included	present	210.78	-0.13	included	present	289.97
215189_at	excluded	absent	3.04	-0.77	excluded	absent	8.85	-0.63	excluded	absent	43.69	0.24	excluded	absent	2.51
215190_at	included	present	87.11	-1.15	included	absent	84.24	-1.18	included	present	125.32	-0.84	included	present	171.65
215191_at	excluded	absent	36.38	-1.36	excluded	absent	32.29	-1.45	included	present	119.49	0.35	excluded	absent	61.82
215192_at	included	present	189.85	-0.63	included	absent	81.08	-1.25	included	absent	154.33	-0.83	included	present	214.82
215193_x_at	included	present	7763.85	1.5	included	present	3085.18	0.1	included	present	979.91	-0.53	included	present	461.18
215194_at	included	present	2439.15	1.38	included	present	2274.84	1.19	included	present	1068.83	-0.16	included	present	1022.32
215195_at	included	present	1153.81	0.49	included	present	788.15	-0.13	included	present	515.3	-0.6	included	present	583.35
215196_at	excluded	absent	123.34	1.13	excluded	absent	15.11	-1.31	excluded	absent	17.31	-1.26	excluded	absent	8.91
215197_at	excluded	absent	79.01	0.23	excluded	absent	73.44	0.1	excluded	absent	68.86	0	excluded	absent	14.21
215198_s_at	excluded	absent	124.29	-0.3	excluded	absent	119.07	-0.34	excluded	absent	26.57	-0.91	excluded	absent	3.51
215199_at	excluded	absent	53.88	-0.41	excluded	absent	49.38	-0.46	excluded	absent	36.67	-0.6	excluded	absent	4.58
215200_x_at	excluded	absent	164.92	-0.5	included	present	224.14	-0.25	included	present	261.38	-0.09	included	present	197.16
215201_at	included	present	119.84	-0.97	included	absent	52.48	-1.6	included	present	165.75	-0.54	included	present	117.51
215202_at	excluded	absent	14.87	-0.21	excluded	absent	7.33	-0.49	excluded	absent	39.27	0.69	excluded	absent	3.92
215203_at	included	present	379.03	-0.25	included	absent	213.46	-0.79	included	present	320.16	-0.44	included	present	370.55
215204_at	excluded	absent	66.36	-0.04	excluded	absent	46.24	-0.39	excluded	absent	53.82	-0.26	excluded	absent	62.71
215205_x_at	excluded	absent	229.38	0.74	excluded	absent	306	1.54	excluded	absent	77.5	-0.85	excluded	absent	114.7
215206_at	excluded	absent	124.89	-0.18	excluded	absent	89.24	-0.56	included	present	190.25	0.53	included	present	124.76
215207_x_at	included	present	730.18	-0.36	included	present	543.35	-0.77	included	present	968.71	0.15	included	present	866.43
215208_x_at	included	present	1048.01	-0.46	included	present	907.09	-0.65	included	present	1551.37	0.23	included	present	903.07
215209_at	included	present	104.26	1	excluded	absent	6.64	-1.17	excluded	absent	56.98	-0.05	included	present	66.84
215210_s_at	included	present	318.13	-0.94	included	present	531.79	0.08	included	present	226.86	-1.38	included	present	762.45

215477_at	included	absent	85.76	-0.65	included	present	85.56	-0.65	included	absent	8.46	-1.44	included	present	161.32	0.11	included	absent	95.62
215478_at	included	present	151.17	-0.63	included	present	220.51	-0.25	included	present	504.67	1.3	included	present	363.11	0.53	included	present	535.82
215479_at	excluded	absent	47.06	-0.34	excluded	absent	73.57	0.31	excluded	absent	23.56	-0.92	excluded	absent	63.18	0.05	excluded	absent	78.21
215480_at	excluded	absent	55.41	0.25	excluded	absent	28.18	-0.43	excluded	absent	38.89	-0.16	excluded	absent	82.65	0.93	excluded	absent	76.58
215481_s_at	included	present	63.99	-0.4	excluded	absent	42.23	-0.65	excluded	absent	11.03	-1.01	excluded	absent	88.39	-0.12	excluded	absent	102.8
215482_s_at	included	present	406.11	-0.21	included	present	903.24	3.11	included	present	494.22	0.38	included	present	366.31	-0.47	included	present	468.9
215483_at	included	present	228.63	-1.29	included	present	425.96	-0.35	included	present	496.3	-0.01	included	present	425.51	-0.35	included	present	510.03
215484_at	included	present	130.75	0.64	included	present	549.56	7.93	included	present	137.54	0.76	included	present	201.63	1.88	excluded	absent	247.77
215485_s_at	excluded	absent	160.05	0.14	included	present	275.71	1	excluded	absent	80.37	-0.45	excluded	absent	92.94	-0.36	excluded	absent	47.2
215486_at	excluded	absent	34.52	-0.2	excluded	absent	2.33	-0.23	excluded	absent	56.96	-0.17	excluded	absent	18.3	-0.21	excluded	absent	32.2
215487_x_at	excluded	absent	11.49	-0.87	included	present	290.48	1.18	excluded	absent	12.91	-0.86	excluded	absent	59.87	-0.51	excluded	absent	141.89
215488_at	excluded	absent	118.11	0.1	excluded	absent	227.9	1.94	excluded	absent	41.62	-1.19	excluded	absent	38.05	-1.25	excluded	absent	53.57
215489_x_at	included	present	178.66	0.23	included	present	222.15	0.74	excluded	absent	56.25	-1.22	excluded	absent	38.56	-1.43	excluded	absent	148.24
215490_at	included	present	336.24	-0.28	included	present	165.66	-1.54	included	present	573.94	1.47	included	present	272.11	-0.75	included	present	313.28
215491_at	excluded	absent	4.91	-0.27	excluded	absent	12.62	-0.11	excluded	absent	2.12	-0.32	included	present	532.4	10.26	excluded	absent	56.71
215492_x_at	excluded	absent	27.03	-1.63	excluded	absent	70.7	-0.87	excluded	absent	127.35	0.12	excluded	absent	70.31	-0.88	included	present	140.15
215493_x_at	included	present	863.66	0.04	included	present	2184.85	3.36	included	present	621.87	-0.57	included	present	1188.55	0.85	included	present	622.93
215494_at	excluded	absent	4.79	-0.6	excluded	absent	3.19	-0.67	excluded	absent	6.05	-0.54	excluded	absent	1.67	-0.74	excluded	absent	16.66
215495_s_at	excluded	absent	98.3	-0.14	excluded	absent	20.35	-0.33	excluded	absent	3.41	-0.37	excluded	absent	2.22	-0.37	excluded	absent	51.3
215496_at	included	present	71.18	-0.25	excluded	absent	79.74	-0.15	excluded	absent	64.01	-0.34	excluded	absent	58.26	-0.41	excluded	absent	68.42
215497_s_at	included	present	238.53	0.57	excluded	absent	91.93	-1.03	excluded	absent	47.73	-1.52	excluded	absent	66.43	-1.31	included	present	347.45
215498_s_at	included	present	2238.95	0.73	included	present	249.42	-0.49	excluded	absent	341.01	-0.43	excluded	absent	630.51	-0.26	excluded	absent	1492.46
215499_at	included	present	2046.1	0.32	included	present	693.87	-0.73	included	present	1155.73	-0.37	included	present	937.53	-0.54	included	present	1442.89
215500_at	excluded	absent	60.52	-0.33	excluded	absent	53.12	-0.46	excluded	absent	4.64	-1.3	excluded	absent	8.24	-1.24	excluded	absent	75.1
215501_s_at	included	absent	152.75	-0.78	included	absent	139.96	-0.83	included	present	380.36	0.14	included	present	364.12	0.08	included	present	480.06
215502_at	excluded	absent	6.94	-0.45	excluded	absent	11.58	-0.29	excluded	absent	4.1	-0.56	excluded	absent	3.76	-0.57	excluded	absent	12.52
215503_at	excluded	absent	1.91	-0.77	excluded	absent	2.76	-0.74	excluded	absent	1.85	-0.77	excluded	absent	1.84	-0.77	excluded	absent	23.17
215504_x_at	included	present	5731.63	1.94	included	present	3745.15	0.33	included	present	7837.43	3.66	included	present	7621.84	3.48	included	present	6279.21
215505_s_at	included	present	276.69	-0.74	included	absent	158.2	-1.35	included	present	378.73	-0.21	included	present	469.18	0.25	included	present	322.55
215506_s_at	included	present	547.28	0.32	included	present	696.59	0.54	excluded	absent	8.8	-0.48	included	present	359.16	0.04	included	present	212.06
215507_x_at	included	present	3209.54	2.25	included	present	1570.96	-0.06	included	present	2018.68	0.57	included	present	2064.23	0.64	included	present	2900.02
215508_at	excluded	absent	18.14	-0.3	excluded	absent	6.53	-0.69	excluded	absent	66.67	1.33	excluded	absent	4.7	-0.75	excluded	absent	59.89
215509_s_at	excluded	absent	50.97	-0.28	included	present	548.21	1.04	included	present	1252.2	2.91	included	present	384.51	0.6	included	present	834.35
215510_at	excluded	absent	8.93	-1.51	excluded	absent	146.41	1.34	excluded	absent	51.8	-0.62	excluded	absent	70.35	-0.24	excluded	absent	77.52

215836_s_at	included	present	851.36	0.31	included	absent	322.11	-0.96	included	present	195.69	-1.26	included	present	392.01	-0.79	included	present	469.78	-0.6	included	present	1697.95	2.34	included	present	1796.43	2.58	included	present	711.96	-0.02	included	present
215837_x_at	included	present	236.61	0.51	included	present	173.96	-0.04	included	present	381.55	1.78	included	present	150.66	-0.25	included	present	165.64	-0.11	included	present	190.56	0.1	included	present	206.63	0.24	included	present	110.98	-0.59	included	present
215838_at	excluded	absent	65.76	0.4	excluded	absent	56.22	0.2	excluded	absent	4.14	-0.87	excluded	absent	35.01	-0.23	excluded	absent	20.94	-0.52	excluded	present	224.94	3.67	excluded	absent	69.83	0.48	excluded	absent	22.17	-0.5	excluded	absent
215839_at	excluded	absent	70.59	1.29	excluded	absent	5.46	-0.51	excluded	absent	3	-0.58	excluded	absent	2.66	-0.59	excluded	absent	44.19	0.56	excluded	present	151.96	3.54	excluded	absent	3.11	-0.58	excluded	absent	14.6	-0.26	excluded	absent
215840_at	excluded	absent	3.29	-0.82	excluded	absent	2.8	-0.83	excluded	absent	102.83	1.56	excluded	absent	3.61	-0.81	excluded	absent	5.31	-0.77	excluded	absent	7	-0.73	excluded	absent	63.45	0.62	excluded	absent	43.12	0.13	excluded	absent
215841_at	excluded	absent	140.96	1.87	excluded	absent	44.8	-0.11	excluded	absent	41.61	-0.17	excluded	absent	117.62	1.39	excluded	absent	10.27	-0.82	excluded	absent	23.82	-0.54	included	present	159.04	2.25	excluded	absent	11.82	-0.79	excluded	absent
215842_s_at	included	present	204.64	-0.18	included	absent	55.89	-1.9	included	absent	105.76	-1.32	included	present	259.4	0.45	included	absent	144.87	-0.87	included	present	261.79	0.48	included	absent	165.49	-0.63	included	present	377.86	1.82	included	present
215843_s_at	excluded	absent	69.6	0.45	excluded	absent	9.2	-0.81	excluded	absent	37.07	-0.23	excluded	absent	63.23	0.32	excluded	absent	5.53	-0.89	excluded	absent	2.35	-0.95	excluded	absent	33	-0.31	excluded	absent	26.28	-0.45	excluded	absent
215844_at	excluded	absent	89.04	0.44	excluded	absent	85.88	0.38	excluded	absent	107.59	0.79	excluded	absent	34.64	-0.58	excluded	absent	10.81	-1.03	excluded	absent	8.32	-1.08	excluded	absent	10.88	-1.03	excluded	absent	29.68	-0.68	excluded	absent
215845_x_at	included	present	550.68	-0.31	included	present	553.31	-0.3	included	present	719.02	0.62	included	present	854.21	1.36	included	present	767.83	0.89	included	present	352.65	-1.4	included	present	682.77	0.42	included	present	222.34	-2.12	included	present
215846_at	excluded	absent	44.55	-0.12	excluded	absent	2.74	-0.8	excluded	absent	18.61	-0.54	excluded	absent	40.56	-0.19	excluded	absent	104.41	0.85	included	present	57.71	0.09	excluded	absent	37.35	-0.24	excluded	absent	20.73	-0.51	included	present
215847_at	included	present	781.69	1.16	included	present	644.8	0.82	included	absent	1101.67	1.96	included	absent	2.13	-0.79	included	present	515.96	0.5	included	absent	136.51	-0.45	included	present	1357.86	2.6	included	present	1683.78	3.41	included	absent
215848_at	excluded	absent	122.75	-0.02	excluded	absent	103.87	-0.28	included	present	71.32	-0.72	included	present	347.1	3.04	excluded	absent	90.35	-0.46	excluded	absent	112.66	-0.16	excluded	absent	87.98	-0.5	excluded	absent	36.11	-1.21	excluded	absent
215849_x_at	excluded	absent	65.76	-0.7	excluded	absent	68.55	-0.66	excluded	absent	39.87	-1.08	excluded	absent	33.11	-1.18	excluded	absent	67.93	-0.67	excluded	absent	80.4	-0.49	included	present	109.1	-0.07	excluded	absent	46.62	-0.99	included	present
215850_s_at	excluded	absent	5.56	-1.18	excluded	absent	39.64	-0.2	excluded	absent	77.45	0.89	excluded	absent	85.58	1.12	excluded	absent	6.4	-1.16	excluded	absent	24.28	-0.64	included	present	61.51	0.43	excluded	absent	1.24	-1.31	included	present
215851_at	excluded	absent	34.91	-0.66	excluded	absent	11.55	-1.23	excluded	absent	3.65	-1.43	excluded	absent	35.5	-0.64	excluded	absent	70.05	0.22	included	present	114.85	1.33	excluded	absent	1.65	-1.48	excluded	absent	20.86	-1	excluded	absent
215852_x_at	excluded	absent	89.34	-0.35	excluded	absent	122.06	0.13	excluded	absent	47.54	-0.96	excluded	absent	153.92	0.6	excluded	absent	156.2	0.64	excluded	absent	122.5	0.14	excluded	absent	21.28	-1.35	excluded	absent	46.52	-0.98	excluded	absent
215853_at	included	present	393.76	-0.16	included	present	88.89	-1.74	included	present	480.84	0.29	included	present	356.09	-0.36	included	present	461.58	0.19	included	present	216.82	-1.08	included	present	184.75	-1.24	included	present	74.45	-1.81	included	present
215854_at	included	present	224.97	1.35	excluded	absent	193.56	0.85	included	present	155.43	0.24	excluded	absent	27.44	-1.81	excluded	absent	10.25	-2.09	included	present	174.2	0.54	excluded	absent	120.84	-0.32	excluded	absent	86.07	-0.87	included	present
215855_s_at	included	absent	170.88	-0.1	included	present	119.5	-0.57	included	absent	130.86	-0.46	included	present	118.5	-0.58	included	present	140.88	-0.37	included	present	210.51	0.25	included	absent	113.25	-0.62	included	absent	52.1	-1.17	included	present
215856_at	excluded	absent	114.84	-0.09	excluded	absent	59.02	-0.54	included	present	121.89	-0.03	included	present	187.86	0.5	included	present	215.73	0.73	included	absent	111.85	-0.11	excluded	absent	72.41	-0.43	excluded	absent	19.85	-0.85	excluded	absent
215857_at	excluded	absent	13.99	-0.69	excluded	absent	5.77	-0.77	excluded	absent	77.65	-0.05	excluded	absent	12.62	-0.7	excluded	absent	4.23	-0.78	excluded	absent	96.83	0.14	excluded	absent	44.25	-0.39	excluded	absent	72.24	-0.11	excluded	absent
215858_at	excluded	absent	1.82	-0.62	excluded	absent	34.96	0.57	excluded	absent	2.87	-0.58	excluded	absent	34.97	0.57	excluded	absent	21.64	0.09	excluded	absent	11.25	-0.28	excluded	absent	3.96	-0.54	excluded	absent	48.91	1.06	excluded	absent
215859_at	excluded	absent	63.1	0.87	excluded	absent	4.78	-0.53	excluded	absent	5.25	-0.52	excluded	absent	3.12	-0.57	excluded	absent	30.49	0.08	excluded	absent	37.53	0.25	excluded	absent	8	-0.46	excluded	absent	3.89	-0.56	excluded	absent
215860_at	excluded	absent	9.58	-1.06	excluded	absent	15.73	-0.93	excluded	absent	14.03	-0.97	excluded	absent	8.1	-1.09	excluded	absent	41.32	-0.4	excluded	absent	60.1	-0.02	excluded	absent	8.17	-1.09	excluded	absent	11.54	-1.02	excluded	absent
215861_at	included	present	369.31	0.22	included	absent	58.7	-0.97	included	present	287.25	-0.1	included	absent	71.45	-0.92	included	present	269.02	-0.17	included	present	300.68	-0.05	included	absent	67.12	-0.94	included	absent	92.19	-0.84	included	present
215862_at	excluded	absent	108.48	0.66	excluded	absent	77.77	0.08	excluded	absent	39.21	-0.64	excluded	absent	135.27	1.16	excluded	absent	125.16	0.97	excluded	absent	86.22	0.24	excluded	absent	75.62	0.04	excluded	absent	9.38	-1.19	excluded	absent
215863_at	excluded	absent	155.35	-0.09	excluded	absent	15.89	-1.17	excluded	absent	74.58	-0.71	excluded	absent	13.43	-1.19	excluded	absent	30.59	-1.05	excluded	absent	140.2	-0.21	excluded	absent	136.52	-0.24	excluded	absent	104.94	-0.48	excluded	absent
215864_at	excluded	absent	85.29	0.04	excluded	absent	6.67	-1.02	excluded	absent	7.75	-1	excluded	absent	62.97	-0.26	excluded	absent	8.09	-1	excluded	absent	36.62	-0.61	excluded	absent	2.15	-1.08	excluded	absent	5.62	-1.03	excluded	absent
215865_at	excluded	absent	5.09	-0.91	excluded	absent	5.32	-0.9	excluded	absent	79.86	0.83	excluded	absent	38.81	-0.13	excluded	absent	20.28	-0.56	excluded	absent	21.06	-0.54	excluded	absent	18.13	-0.61	excluded	absent	6.93	-0.87	excluded	absent
215866_at	excluded	absent	67.11	-0.59	excluded	absent	6.85	-1.67	excluded	absent	34.86	-1.17	excluded	absent	70.15	-0.53	excluded	absent	151.04	0.91	excluded	absent	122.91	0.41	excluded	absent	69.41	-0.55	excluded	absent	27.42	-1.3	excluded	absent
215867_x_at	excluded	absent	44.18	-0.54	excluded	absent	37.89	-0.55	excluded	absent	43.1	-0.54	excluded	absent	16.8	-0.57	excluded	absent	12.91	-0.57	excluded	present	358.17	-0.25	excluded	absent	1.85	-0.58	excluded	absent	47.04	-0.54	excluded	present
215868_x_at	included	present	113.1	-0.2	included	present	81.28	-0.5	excluded	absent	49.15	-0.8	excluded	absent	79.35	-0.52	excluded	present	172.91	0.37	excluded	absent	81.87	-0.49	excluded	absent	120.2	-0.13	excluded	absent	89.52	-0.42	excluded	absent
215869_at	excluded	absent	110.45	1.21	excluded	absent	51.81	-0.05	excluded	absent	15.49	-0.83	excluded	absent	69.93	0.34	excluded	absent	26.37	-0.6	excluded	absent	31.11	-0.5	excluded	absent	63.93	0.21	excluded	absent	8.8	-0.97	excluded	absent
215870_s_at	excluded	absent	27.2	-0.4	excluded	absent	27.71	-0.4	excluded	absent	3.37	-0.5	excluded	absent	94.45	-0.12	excluded	absent	10.85	-0.46	included	present	273.39	0.61	excluded	absent	28.87	-0.39	excluded	absent	2.47	-0.5	excluded	absent
215871_at	excluded	absent	16.24	-0.27	excluded	absent	74.1	0.1	excluded	absent	46.38	-0.08	excluded	absent	61.53	0.02	excluded	absent	3.99	-0.35	excluded	absent	117.26	0.37	excluded	absent	13.84	-0.29	excluded	absent	37.88	-0.13	excluded	absent
215872_at	excluded	absent	47.4	-0.15	excluded	absent	8.03	-0.76	excluded	absent	2.7	-0.84	excluded	absent	4.4	-0.82	excluded	absent	26.43	-0.48	excluded	absent	45.74	-0.18	excluded	absent	63.72	0.1	excluded	absent	18.68	-0.6	included	present
215873_x_at	included	present	320.62	0.51	included	present	569.04	3.47	included	present	237.66	-0.48	included	present	262.68	-0.19	included	present	332.77	0.65	included	present	442.81	1.97	included	present	504.26	2.7	included	present	831.73	6.61	included	present
215874_at	excluded	absent	86.82	1.04	excluded	absent	61.39	0.4	excluded	absent	44.09	-0.03	excluded	absent	74.47	0.73	excluded	absent	8.14	-0.93	excluded	absent	13.41	-0.8	excluded	absent	37.07	-0.21	excluded	absent	9.51	-0.9	excluded	absent
215875_at	excluded	absent	37.19	-0.34	excluded	absent	98.13	0.88	excluded	absent	16.65	-0.75	excluded	absent	32.3	-0.44	excluded	absent	129.45	1.51	excluded	absent	37.62	-0.33	excluded	absent	32.56	-0.43	excluded	absent	56.88	0.06	excluded	absent
215876_at	excluded	absent	76.45	0.67	excluded	absent	93.95	1.06	excluded	absent	19.45	-0.58	excluded	absent	10.34	-0.78	excluded	absent	168.78	2.71	excluded	absent	15.6	-0.66	excluded	absent	73.44	0.61	excluded	absent	79.42	0.74	excluded	absent
215877_at	excluded	absent	64.71	-0.15	excluded	absent	102.39	0.15	excluded	absent	76.83	-0.05	excluded	absent	6.43	-0.61	excluded	absent	73.95	-0.07	excluded	absent	45.36	-0.3	excluded	absent	5.14	-0.62	excluded	absent	7.47	-0.6	excluded	absent
215878_at	included	present	305.85	-0.6	included	absent	19.47	-1.55	included	present	484.16	-0.01	included	present	309.13	-0.59	included	present	184.01	-1.01	included	present	396.4	-0.3	included	present	237.18	-0.83	included	present	79.05	-1.35	included	present
215879_at	excluded	absent	79.8	-0.82	excluded	absent	133.93	-0.2	excluded	absent	104.73	-0.53	excluded	absent	62.55	-1.01	excluded	absent	223.98	0.84	excluded	absent	179.3	0.32	excluded	absent	64.81	-0.99	excluded	absent	84.56	-0.76	excluded	absent
215880_at	excluded	absent	6.21	-0.75	excluded	absent	5.89	-0.76	excluded	absent	16.73	-0.29	excluded	absent	10.66	-0.56	excluded	absent	77.2	2.35	excluded	absent	16.75	-0.29	excluded	absent	111.97	3.87	excluded	absent	92.96	3.04	excluded	absent
215881_x_at	excluded	absent	2.81	-0.96	included	present	300.62	2.11	included	present	77.44	-0.19	excluded	absent	16.85	-0.82	included	present	191.37	0.99	excluded	absent	93.58	-0.02	excluded	absent	35.22	-0.63	included	present	1924.95	18.88	excluded	absent
215882_at	included	present	103.21	0.36	included	present	248.95	2.63	included	present	148.76	1.07	included	present	134.5	0.84	included	present	131.04	0.79	included	present	87.28	0.11	excluded	absent	80.2	0	excluded	absent	77.7	-0.04	excluded	absent
215883_at	included	present	147.24	2.83	excluded	absent	9.93	-0.93	excluded	absent	73.18	0.8	excluded	absent	31.18	-0.35	excluded	absent	27.13	-0.46	excluded	absent	130.55	2.37	excluded	absent	38.64	-0.14	excluded	absent	56.38	0.34	excluded	absent
215884_s_at	included	present	3228.51	-0.32	included	present	4329.71	0.33	included	present	4177.32	0.24	included	present	3213.6	-0.33	included	present	2804.3	-0.57	included	present	3424.77	-0.2	included	present	7864.5	2.42	included	present	3917.76	0.09	included	present
215885_at	excluded	absent	7.95	-0.62	excluded	absent	92.97	1.56	excluded	absent	1.48	-0.79	excluded	absent	47.48	0.39	excluded	absent	85.05	1.35	excluded	absent	90.27	1.49	excluded	absent	51.81	0.5	included	present	565.94	13.7	excluded	absent
215886_x_at	included	present	1418.43	1.1	included	present	451.74	-1.52	included	present	741.38	-0.73	included	present	596.45	-1.13	included	present	724.82	-0.78	included	present	825.86	-0.5	included	present	1444.81	1.17	included	present	885.19	-0.34	included	present
215887_at	included	present	464.23	-0.69	included	present	886.28	0.13	included	present	373.11	-0.87	included	present	701.74	-0.23	included	present	637.7	-0.36	included	present	496.08	-0.63	included	present	485.39	-0.65	included	present	250.93	-1.11	included	present
215888_at	excluded	absent	62.17	-0.27	excluded	absent	16.1	-1.26	excluded	absent	50.17	-0.53	excluded	absent	9.28	-1.41	excluded	absent	83.7	0.2	excluded	absent	42.64	-0.69	included	present	151.38	1.67	excluded	absent	66.56	-0.17	excluded	absent
215889_at	included	present	761.71	0.75	included	present	447.13	-0.05	included	present	527.28	0.15	included	present	610.55	0.36	included	present	859.94	1	included	present	779.01	0.79	included	present	465.06	-0.01	included	present	173.34	-0.75	included	present
215890_at	excluded	absent	91.89	0.74	excluded	absent	4.9	-1.27	excluded	absent	55.23	-0.11	excluded	absent	4.14	-1.28	excluded	absent	30.84	-0.67	excluded	absent	57.24	-0.06	excluded	absent	77.34	0.4	excluded	absent	14.08	-1.05	excluded	absent
215891_s_at	included	present	616.12	0.29	included	present	274.26	-0.55	included	present	277.68	-0.54	included	present	139.53	-0.87	included	present	413.04	-0.21	included	present	937.04	1.07	included	present	774.29	0.67	included	present	1856.98	3.31	included	present
215892_at	included	present	237.47	1.96	excluded	absent	86.81	-0.52	included	present	197.75	1.3	included	present	147.22	0.47	excluded	absent	114.08	-0.07	excluded	absent	69.16	-0.81	excluded	absent	12.43	-1.74	excluded	absent	7.46	-1.83	excluded	absent
215893_x_at	excluded	absent	80.04	0.25	excluded	absent	8.45	-1.29	excluded	absent	69.97	0.03	excluded	absent	3.55	-1.4	excluded	absent	134.14	1.42	excluded	absent	10.78	-1.24	excluded	absent	41.48	-0.58	excluded	absent	52.07	-0.35	excluded	absent
215894_at	excluded	absent	77.21	-0.26	excluded	absent	76.24	-0.27	excluded	absent	25.33	-0.7	excluded	absent	42.35	-0.56	excluded	absent	13.72	-0.8	excluded	present	99.09	-0.07	excluded	absent	3.99	-0.89	excluded	absent	2.15	-0.9	excluded	absent
215895_x_at	excluded	absent	86.89	0.43	excluded	absent	100.2	0.67	excluded	absent	90.84	0.5	excluded	absent	30.27	-0.59	excluded	absent	55.02	-0.14	excluded	absent	6.61	-1.02	excluded	absent	49.77	-0.24	excluded	absent	31.94	-0.56	excluded	absent
215896_at	excluded	absent	37.11	-0.53	excluded	absent	71.68	0.16	excluded	absent	3.04	-1.21	excluded	absent	4.9	-1.17	excluded	absent	123.42	1.19	excluded	absent	3.98	-1.19	excluded	absent	8.23	-1.11	excluded	absent	14.66	-0.98	excluded	absent
215897_at	excluded	absent	173.54	-0.36	excluded	absent	146.5	-0.55	excluded	absent	197.81	-0.19	included	present	1000.72	5.48	excluded	absent	286.22	0.43	excluded	absent	155.75	-0.49	excluded	absent	147.5	-0.55	included	present	462.51	1.68	excluded	absent
215898_at	excluded	absent	30.72	-1.61	excluded	absent	47.73	-1.19	excluded	absent	10.92	-2.09	excluded	absent	132.42	0.89	excluded	absent	131.33	0.86	excluded	absent	179.53	2.04	excluded	absent	11.09	-2.09	excluded	absent	1.63	-2.32	excluded	absent
215899_at	included	present	197.8	0	included	absent	43.67	-2.47	included	present	183.03	-0.24	included	present	174.82	-0.37	included	present	85.47	-1.8	included	present	120.69	-1.24	included	absent	7.59	-3.05	included	absent	58.12	-2.24	included	present
215900_at	excluded	absent	37.43	-0.89	excluded	absent	7.33	-1.31	excluded	absent	138.87	0.55	excluded	absent	3.9	-1.36	excluded	absent	6.15	-1.33	excluded	absent	83.46	-0.24	excluded	absent	21.24	-1.12	excluded	absent	2.85	-1.38	excluded	absent
215901_at	included	present	182.78	0.61	included	absent	122.19	-0.33	included	present	242.9	1.54	included	present	150.89	0.12	included	present	227.12	1.29	included	present	225.21	1.26	included	present	240.96	1.51	included	present	303.65	2.48	included	present
215902_at	included	present	147.41	-0.75	included	absent	187.61	-0.24	included	present	207.45	0.01	included	present	231.27	0.3	included	present	312.31	1.32	included	present	147.89	-0.74	included	present	147.46	-0.75	included	present	123.91	-1.04	included	present
215903_s_at	included	present	687.51	-0.09	included	present	611.63	-0.21	included	present	235.45	-0.81	included	present	738.61	-0.01	included	present	438.21	-0.49	included	present	963.68	0.35	included	present	1071.39	0.52	included	present	835.29	0.14	included	present
215904_at	included	present	226.5	-0.46	included	present	111.11	-1.02	included	present	72.62	-1.21	included	present	194.24	-0.62	included	present	229.95	-0.45	included	present	209.99	-0.54	included	present	138.91	-0.89	included	present	141.31	-0.88	included	present
215905_s_at	included	present	501.58	-0.47	included	present	579.43	-0.11	included	present	553.27	-0.23	included	present	873.95	1.24	included	present	714.36	0.51	included	present	722.95	0.55	included	present	821.8	1	included	present	1363.35	3.49	included	present
215906_at	excluded	absent	3.89	-0.81	excluded	absent	39.49	0.67	excluded	absent	59.48	1.51	excluded	absent	5.68	-0.74	excluded	absent	10.79	-0.52	excluded	absent	5.69	-0.74	excluded	absent	7.08	-0.68	excluded	absent	16.51	-0.29	excluded	absent
215907_at	included	absent	378.14	-0.62	included	present	347.7	-0.73	included	present	595.8	0.19	included	present	408.85	-0.51	included	present	593.65	0.18	included	present	176.29	-1.37	included	present	436.38	-0.4	included	absent	137.75	-1.51	included	present
215908_at	included	present	290.52	-0.25	included	present	105.63	-1.52	included	present	541.77	1.48	included	present	343.77	0.12	included	present	478.88	1.05	included	present	92.54	-1.61	included	present	144.57	-1.25	included	absent	52.78	-1.88	included	present
215909_x_at	included	absent	181.8	-0.88	included	present	268.69	-0.46	included	present	117.08	-1.18	included	absent	96.11	-1.28	included	present	271.51	-0.45	included	present	428.27	0.3	included	present	151.91	-1.02	included	present	363.97	-0.01	included	present
215910_s_at	included	absent	233.95	-0.02	included	absent	121.45	-1.04	included	absent	131.25	-0.95	included	present	197.97	-0.35	included	absent	133.13	-0.93	included	absent	302.46	0.6	included	present	460.76	2.04	included	present	720.97	4.41	included	present
215911_x_at	excluded	absent	99.62	0.07	excluded	absent	9.38	-0.9	excluded	absent	12.63	-0.86	excluded	absent	7.25	-0.92	excluded	absent	69	-0.26	excluded	absent	3.94	-0.95	excluded	absent	140.04	0.51	included	present	300.36	2.22	excluded	absent
215912_at	excluded	absent	39.98	-0.74	excluded	absent	12.99	-1.06	excluded	absent	100.28	-0.03	excluded	absent	123.93	0.25	excluded	absent	116.87	0.16	excluded	absent	164.84	0.73	excluded	absent	87.63	-0.18	excluded	absent	206.48	1.22	included	present
215913_s_at	included	present	1575.45	2.73	included	absent	78.36	-0.99	included	present	668.18	0.47	included	present	2698.96	5.52	included	present	334.62	-0.36	included	present	387.57	-0.22	included	present	4.71	-1.17	included	present	588.1	0.27	included	present

216196_at	excluded	absent	22.99	-1.16	excluded	absent	81.12	0.02	excluded	absent	70.58	-0.19	excluded	absent	13.2	-1.36	excluded	absent	174.72	1.93	excluded	absent	102.14	0.45	included	present	238.24	3.22	included	present	158.57	1.6	included	present	126.47	0.95	excluded	absent	74.55	-0.11	included	present	215.57	2.76	excluded	absent	55.48	-0.5	excluded	absent	111.56	0.64	excluded	absent	57.97	-0.45	included	present	102.4	0.46
216197_at	excluded	absent	65.85	-0.05	excluded	absent	53.24	-0.28	excluded	absent	4.78	-1.16	excluded	absent	80.76	0.22	excluded	absent	154.77	1.57	excluded	absent	51.07	-0.32	excluded	absent	60.84	-0.14	excluded	absent	14.92	-0.98	excluded	absent	114.06	0.83	excluded	absent	62.09	-0.12	excluded	absent	69.31	0.01	excluded	absent	2.85	-1.2	excluded	absent	51.44	-0.31	excluded	absent	24.21	-0.81	excluded	absent	29.7	-0.71
216198_at	included	present	400.91	-0.03	included	present	177.52	-0.84	included	present	332.78	-0.28	included	present	304.34	-0.38	included	present	439.19	0.11	included	present	291.1	-0.43	included	present	350.28	-0.21	included	present	78.14	-1.21	included	present	285.01	-0.45	included	present	162.19	-0.9	included	present	176.91	-0.85	included	absent	41.07	-1.34	included	present	332.82	-0.28	included	absent	93.6	-1.15	included	present	134.96	-1
216199_s_at	included	present	571.97	-0.8	included	present	391.75	-1.32	included	present	544.59	-0.88	included	present	799.72	-0.15	included	present	688.81	-0.47	included	present	400.51	-1.29	included	present	614.58	-0.68	included	present	845.48	-0.02	included	present	611.54	-0.69	included	present	968.06	0.33	included	present	408.04	-1.27	included	present	647.39	-0.59	included	present	458.13	-1.13	included	present	1374.23	1.49	included	present	505.28	-0.99
216200_at	excluded	absent	7.47	-0.96	excluded	absent	2.71	-1.12	excluded	absent	4.44	-1.06	excluded	absent	2.32	-1.13	excluded	absent	4.2	-1.07	excluded	absent	4.7	-1.05	excluded	absent	64.47	0.88	excluded	absent	52.73	0.5	included	present	74.86	1.21	excluded	absent	1.99	-1.14	excluded	absent	31.76	-0.18	excluded	absent	64.39	0.88	excluded	absent	27.48	-0.32	excluded	absent	39.94	0.09	excluded	absent	9.82	-0.89
216201_at	excluded	absent	2.63	-0.5	excluded	absent	5.02	-0.45	excluded	absent	1.23	-0.53	excluded	absent	2.1	-0.51	excluded	absent	6.08	-0.42	excluded	absent	41.56	0.37	excluded	absent	53.08	0.63	excluded	absent	0.38	-0.55	excluded	absent	14.03	-0.25	excluded	absent	4.21	-0.47	excluded	absent	3.38	-0.48	excluded	absent	2.44	-0.51	included	present	82.65	1.29	excluded	absent	18.13	-0.15	excluded	absent	10.02	-0.34
216202_s_at	included	present	720.98	0.5	included	present	396.74	-0.72	included	present	760.88	0.66	included	present	1294.86	2.68	included	present	298.57	-1.09	included	present	651.82	0.24	included	present	563.98	-0.09	included	present	1086.26	1.89	included	present	926.04	1.28	included	present	669.28	0.31	included	present	478.39	-0.41	included	present	220.51	-1.39	included	present	221.04	-1.39	included	present	411.61	-0.67	included	present	544.81	-0.16
216203_at	excluded	absent	118.73	1.13	excluded	absent	0.62	-1.48	excluded	absent	51.59	-0.36	excluded	absent	60.56	-0.16	excluded	absent	30.32	-0.83	excluded	absent	126.72	1.31	excluded	absent	94.15	0.59	excluded	absent	47.96	-0.44	included	present	69.66	0.04	excluded	absent	29.44	-0.85	excluded	absent	30.3	-0.83	excluded	absent	64.75	-0.07	excluded	absent	1.83	-1.46	excluded	absent	58.73	-0.2	excluded	absent	0.58	-1.49
216204_at	excluded	absent	1.54	-0.71	excluded	absent	8.45	-0.48	excluded	absent	5.1	-0.59	excluded	absent	31.06	0.26	excluded	absent	4.78	-0.6	excluded	absent	5.18	-0.59	excluded	absent	5.58	-0.58	excluded	absent	2.55	-0.68	excluded	absent	8.83	-0.47	excluded	absent	8.81	-0.47	excluded	absent	4.64	-0.61	excluded	absent	10.73	-0.41	excluded	absent	2.12	-0.69	excluded	absent	2.79	-0.67	excluded	absent	5.48	-0.58
216205_s_at	included	present	82.19	-0.98	included	present	201.08	-0.31	included	absent	111.47	-0.82	included	absent	61.49	-1.1	excluded	absent	259.43	0.02	included	present	312.45	0.32	included	present	296.99	0.23	included	present	333.21	0.43	included	present	460.73	1.16	included	present	482.54	1.28	included	present	348.86	0.52	included	absent	115.1	-0.8	included	absent	43.54	-1.2	included	present	73.12	-1.04	included	present	474.82	1.24
216206_x_at	included	present	154.55	-0.05	included	present	161.29	0.04	excluded	absent	208.07	0.71	included	present	178.6	0.29	excluded	absent	91.27	-0.96	excluded	absent	212.47	0.77	excluded	absent	84.24	-1.06	excluded	absent	81.89	-1.09	included	present	95.55	-0.89	excluded	absent	92.4	-0.94	excluded	absent	74.46	-1.19	excluded	absent	84.25	-1.06	excluded	absent	79.27	-1.13	excluded	absent	24.74	-1.9	excluded	absent	46.38	-1.59
216207_x_at	excluded	absent	67.94	-0.37	excluded	absent	84.19	-0.36	excluded	absent	75.72	-0.37	excluded	absent	27.83	-0.39	included	present	1455.83	0.41	included	present	508.88	-0.12	excluded	absent	155.62	-0.32	included	present	229.45	-0.28	included	present	882.32	0.09	excluded	absent	13.7	-0.4	included	present	666.18	-0.03	included	present	1022.04	0.17	included	present	192	-0.3	excluded	absent	6.08	-0.41	included	present	301.48	-0.24
216208_s_at	excluded	absent	45.14	-0.53	excluded	absent	26.76	-0.89	excluded	absent	18.07	-1.07	excluded	absent	87.32	0.31	excluded	absent	139.06	1.35	included	present	213.8	2.84	included	present	279.45	4.14	included	present	271.88	3.99	included	present	210.41	2.77	included	present	222	3	included	present	244.24	3.44	included	present	127.86	1.12	excluded	absent	21.78	-0.99	excluded	absent	56.66	-0.3	included	present	357.73	5.7
216209_at	excluded	absent	9.19	-0.55	excluded	absent	91.69	0.88	excluded	absent	5.67	-0.61	excluded	absent	1.84	-0.68	excluded	absent	111.79	1.23	excluded	absent	3.82	-0.65	excluded	absent	53.96	0.23	excluded	absent	53.05	0.21	excluded	absent	7.21	-0.59	excluded	absent	34.51	-0.11	excluded	absent	129.29	1.54	excluded	absent	15.87	-0.44	excluded	absent	77.82	0.64	included	present	152.03	1.93	excluded	absent	62.54	0.38
216210_x_at	included	present	1004.29	0.44	included	present	962.73	0.33	included	present	660.93	-0.44	included	present	599.41	-0.59	included	present	763.41	-0.18	included	present	2008.32	3.01	included	present	556.63	-0.7	included	present	2683.33	4.73	included	present	1041.09	0.53	included	present	1767.19	2.39	included	present	1299.45	1.19	included	present	3312.85	6.34	included	present	1548.23	1.83	included	present	1792.86	2.45	included	present	1381.7	1.4
216211_at	included	present	310.41	-0.12	included	present	199.21	-0.8	included	present	190.86	-0.85	included	present	185.28	-0.88	included	present	306.38	-0.15	included	present	424.09	0.57	included	present	161.25	-1.03	included	present	168.09	-0.99	included	present	263.62	-0.41	included	present	246.87	-0.51	included	present	250.45	-0.49	included	present	217.4	-0.69	included	present	130.51	-1.21	included	present	206.08	-0.76	included	present	70.7	-1.58
216212_s_at	included	present	1365.74	-1.03	included	present	1925.78	0.13	included	present	4982.67	6.47	included	present	3071.42	2.5	included	present	2640.84	1.61	included	present	832.8	-2.14	included	present	985.21	-1.82	included	present	2784.99	1.91	included	present	1277.18	-1.22	included	present	1365.22	-1.03	included	present	2311.01	0.93	included	present	1496.7	-0.76	included	present	1245.04	-1.28	included	present	1538.05	-0.68	included	present	1613.58	-0.52
216213_at	included	present	1542.68	2.72	included	present	678.63	-0.05	included	present	698.31	0.02	included	present	440.67	-0.81	included	present	695.16	0.01	included	present	598.8	-0.3	included	present	750.4	0.18	included	present	498.81	-0.62	included	present	974.89	0.9	included	present	754.63	0.2	included	present	488.15	-0.66	included	present	493.18	-0.64	included	present	854.55	0.52	included	present	866.72	0.56	included	present	431.76	-0.84
216214_at	excluded	absent	2.97	-0.56	excluded	absent	66.99	0.63	excluded	absent	2.86	-0.56	excluded	absent	67.27	0.63	excluded	absent	3.45	-0.55	excluded	absent	3.25	-0.55	excluded	absent	13.64	-0.36	excluded	absent	5.62	-0.51	excluded	absent	120.15	1.61	excluded	absent	1.84	-0.58	excluded	absent	5.39	-0.51	excluded	absent	2.31	-0.57	excluded	absent	14.7	-0.34	excluded	absent	19.18	-0.26	excluded	absent	20.49	-0.23
216215_s_at	included	present	2777.77	0.74	included	present	5579.53	3.41	included	present	4716.26	2.59	included	present	1433.38	-0.54	included	present	3152.17	1.1	included	present	2189.14	0.18	included	present	5060.89	2.92	included	present	3023.41	0.97	included	present	3904.05	1.82	included	present	4745.2	2.62	included	present	3946.65	1.86	included	present	2730.59	0.69	included	present	3160.07	1.11	included	present	3676.69	1.6	included	present	5267	3.12
216216_at	included	present	147.8	2.54	excluded	absent	111.29	1.57	excluded	absent	130.14	2.07	included	present	64.38	0.33	excluded	absent	17.51	-0.91	excluded	absent	101.56	1.31	included	present	127.62	2	included	present	208.56	4.15	included	present	82.89	0.82	excluded	absent	69.79	0.47	included	present	145.58	2.48	excluded	absent	32.14	-0.53	included	present	125.37	1.94	included	present	158.65	2.83	included	present	157.63	2.8
216217_at	excluded	absent	3.18	-1.44	excluded	absent	109.07	1.18	excluded	absent	11.69	-1.22	excluded	absent	45.88	-0.38	excluded	absent	153.67	2.28	excluded	absent	32.4	-0.71	excluded	absent	39.29	-0.54	excluded	absent	56.17	-0.13	excluded	absent	43.38	-0.44	excluded	absent	31.11	-0.75	excluded	absent	43.26	-0.45	included	present	99.7	0.95	excluded	absent	5.5	-1.38	excluded	absent	1.44	-1.48	included	present	34.66	-0.66
216218_s_at	included	present	237.59	-0.02	included	present	213.96	-0.17	included	absent	97.94	-0.9	included	present	229.3	-0.08	included	present	168.49	-0.46	included	absent	185.21	-0.35	included	present	171.99	-0.44	included	present	170.74	-0.44	included	present	242.68	0.01	included	present	181.19	-0.38	included	present	238.56	-0.02	included	present	160.94	-0.51	included	present	297.82	0.35	included	present	150.25	-0.57	included	present	205.35	-0.23
216219_at	included	present	120.16	-0.02	excluded	absent	39.72	-0.75	excluded	absent	50.53	-0.65	excluded	absent	54.63	-0.62	excluded	absent	67.09	-0.5	excluded	absent	3.9	-1.07	excluded	absent	64.32	-0.53	excluded	absent	36.92	-0.78	excluded	absent	112.33	-0.09	excluded	absent	89.44	-0.3	excluded	absent	41.15	-0.74	excluded	absent	99.87	-0.21	included	present	85.33	-0.34	excluded	absent	34.96	-0.79	included	present	54.65	-0.62
216220_s_at	excluded	absent	77.41	-0.58	excluded	absent	141.76	0.31	excluded	absent	56.41	-0.88	excluded	absent	15.09	-1.45	excluded	absent	94.55	-0.34	included	present	169.72	0.7	excluded	absent	5.87	-1.58	excluded	absent	13.12	-1.48	excluded	absent	137.1	0.25	excluded	absent	122.1	0.04	excluded	absent	125.2	0.08	excluded	absent	19.18	-1.39	excluded	absent	113.53	-0.08	excluded	absent	55.54	-0.89	excluded	absent	93.85	-0.35
216221_s_at	included	present	1168.07	-0.83	included	present	1224.55	-0.7	included	present	3127.33	3.82	included	present	2519.56	2.38	included	present	1380.21	-0.33	included	present	1489.68	-0.07	included	present	4213.04	6.39	included	present	1767.97	0.59	included	present	2181.3	1.57	included	present	1511.55	-0.01	included	present	4064.52	6.04	included	present	2555.18	2.46	included	present	4263.57	6.51	included	present	2917.75	3.32	included	present	2641.48	2.67
216222_s_at	included	present	807.86	-0.31	included	absent	110.76	-1.14	included	present	355.69	-0.85	included	present	595.01	-0.56	included	present	436.79	-0.75	included	present	423.1	-0.77	included	absent	47.85	-1.22	included	present	88.63	-1.17	included	present	177.02	-1.06	included	absent	23.2	-1.25	included	present	295.27	-0.92	included	present	327.07	-0.89	included	present	229.92	-1	included	present	301.52	-0.92	included	absent	105.03	-1.15
216223_at	excluded	absent	59.14	-0.14	excluded	absent	4.24	-0.18	included	present	37.91	-0.16	excluded	absent	35.02	-0.16	excluded	absent	134.03	-0.1	excluded	absent	42.77	-0.15	excluded	absent	1.4	-0.18	excluded	absent	1.9	-0.18	excluded	absent	22.21	-0.17	excluded	absent	1.85	-0.18	excluded	absent	3.73	-0.18	excluded	absent	93.59	-0.13	excluded	absent	46.49	-0.15	excluded	absent	72.7	-0.14	excluded	absent	14.57	-0.17
216224_s_at	excluded	absent	119.69	0.18	excluded	absent	106.25	-0.03	excluded	absent	95.17	-0.2	excluded	absent	85.26	-0.35	excluded	absent	100.05	-0.13	excluded	absent	73.31	-0.54	included	present	231.68	1.9	excluded	absent	14.84	-1.44	included	present	119.89	0.18	included	present	167.35	0.91	excluded	absent	6.94	-1.56	excluded	absent	110.1	0.03	excluded	absent	81.86	-0.41	excluded	absent	3.45	-1.61	included	present	106	-0.03
216225_at	excluded	absent	1.54	-0.93	excluded	absent	4.9	-0.8	excluded	absent	4.98	-0.79	excluded	absent	5.95	-0.75	excluded	absent	34.3	0.35	excluded	absent	64.22	1.52	excluded	absent	21.42	-0.15	excluded	absent	12.47	-0.5	excluded	absent	44.82	0.76	excluded	absent	26.95	0.06	excluded	absent	55.92	1.19	excluded	absent	33.72	0.33	excluded	absent	31.66	0.25	excluded	absent	29.49	0.16	excluded	absent	13.68	-0.45
216226_at	included	present	247.76	-0.62	included	present	447.92	0.29	included	present	692.26	1.4	included	absent	35.4	-1.58	included	present	543.72	0.73	included	present	201.97	-0.82	included	present	314.97	-0.31	included	present	802.3	1.9	included	present	156.2	-1.03	included	present	166.15	-0.99	included	present	522.74	0.63	included	present	103.99	-1.27	included	present	486.24	0.46	included	present	288.84	-0.43	included	present	316.63	-0.3
216227_at	excluded	absent	83.57	0.46	excluded	absent	34.75	-0.82	excluded	absent	79.28	0.35	included	present	78.9	0.34	excluded	absent	92.21	0.69	excluded	absent	59.23	-0.18	excluded	absent	88.1	0.58	excluded	absent	63.29	-0.07	excluded	absent	54.97	-0.29	excluded	absent	33.71	-0.85	excluded	absent	100.61	0.91	excluded	absent	53.72	-0.32	excluded	absent	34.18	-0.83	excluded	absent	28.75	-0.98	excluded	absent	27.12	-1.02
216228_s_at	included	absent	79.49	-0.52	included	present	549.67	5.07	included	present	557.77	5.17	included	present	1034.96	10.85	included	present	334.39	2.51	excluded	absent	138.25	0.18	included	present	135.07	0.14	included	present	434.4	3.7	included	present	127.65	0.05	included	present	145.75	0.27	included	present	299.7	2.1	included	present	432.15	3.68	included	present	179.1	0.66	included	present	563.85	5.24	included	present	386.15	3.13
216229_x_at	included	present	778.78	-1.01	included	present	614.19	-1.6	included	present	697.64	-1.3	included	present	709.28	-1.26	included	present	486.9	-2.05	included	present	569.91	-1.75	included	present	639.87	-1.5	included	present	428.36	-2.26	included	present	1051.4	-0.03	included	present	333.87	-2.6	included	present	768.36	-1.04	included	present	363.62	-2.49	included	present	683.79	-1.35	included	present	458.14	-2.16	included	present	214.16	-3.03

216557_x_at	excluded	absent	138.45	-0.27	excluded	absent	101.1	-0.28	excluded	absent	116.31	-0.28	included	present	196.84	-0.26	included	present	4664.95	0.79
216558_x_at	excluded	absent	3.77	-0.86	excluded	absent	108.91	0.47	excluded	absent	5.25	-0.84	excluded	absent	41.19	-0.39	excluded	absent	151.61	1.02
216559_x_at	included	present	8334.8	-0.66	included	present	12729.49	0.68	included	present	15137.43	1.42	included	present	13679.81	0.97	included	present	10933.33	0.13
216560_x_at	excluded	absent	6.8	-0.29	excluded	absent	3.95	-0.3	excluded	absent	16.46	-0.25	excluded	absent	4.94	-0.29	excluded	absent	28.94	-0.21
216561_x_at	included	present	113.84	0.69	excluded	absent	4.92	-1.54	excluded	absent	17.74	-1.28	excluded	absent	54.87	-0.51	excluded	absent	83.05	0.06
216562_at	excluded	absent	12.44	-0.52	excluded	absent	15.39	-0.43	excluded	absent	4.09	-0.77	excluded	absent	8.25	-0.64	excluded	absent	5.45	-0.73
216563_at	included	present	6772.17	-1.3	included	present	14709.2	2.21	included	present	6453.47	-1.44	included	present	11570.18	0.82	included	present	9440.8	-0.12
216564_at	excluded	absent	167.56	0.36	excluded	absent	26.24	-1.53	included	present	224.96	1.12	excluded	absent	77.14	-0.85	excluded	absent	71.38	-0.92
216565_x_at	included	present	990.97	1.47	included	absent	93.76	-1.07	included	absent	105.91	-1.04	included	absent	28.87	-1.26	included	absent	293.89	-0.51
216566_at	excluded	absent	30.87	-0.27	excluded	absent	6.77	-0.37	excluded	absent	12.88	-0.35	excluded	absent	3.25	-0.38	excluded	absent	88.64	-0.04
216567_at	excluded	absent	33.89	-0.38	excluded	absent	105.32	1.09	excluded	absent	8.9	-0.9	excluded	absent	150.41	2.02	excluded	absent	9.88	-0.88
216568_x_at	excluded	absent	16.32	-0.39	excluded	absent	6.5	-0.48	excluded	absent	2.85	-0.51	excluded	absent	75.09	0.14	excluded	absent	46.78	-0.12
216569_at	included	present	109.31	1.35	excluded	absent	101.41	1.18	excluded	absent	22.85	-0.52	excluded	absent	3.11	-0.95	excluded	absent	72.19	0.55
216570_x_at	included	present	1040.68	-0.29	included	present	436.25	-0.89	included	present	1557.39	0.23	included	present	262.15	-1.07	included	present	679.88	-0.65
216571_at	excluded	absent	44.77	-0.13	excluded	absent	42.62	-0.18	excluded	absent	82.44	0.64	included	present	66.8	0.32	excluded	absent	12.13	-0.8
216572_at	excluded	absent	5.64	-0.58	excluded	absent	6.75	-0.52	excluded	absent	11.07	-0.27	excluded	absent	41.48	1.5	excluded	absent	4.63	-0.64
216573_at	excluded	absent	8.67	-0.38	excluded	absent	56.94	0.42	excluded	absent	2.46	-0.48	excluded	absent	1.66	-0.5	excluded	absent	115.12	1.37
216574_s_at	included	present	2530.55	-0.61	included	present	4413.15	1.77	included	present	4017.88	1.27	included	present	3855.81	1.07	included	present	2970.47	-0.05
216575_at	excluded	absent	35.94	0.1	excluded	absent	53.15	0.63	excluded	absent	25.75	-0.22	excluded	absent	3.81	-0.9	excluded	absent	12.67	-0.63
216576_x_at	excluded	absent	51.49	-0.35	excluded	absent	34.95	-0.36	excluded	absent	44.95	-0.35	excluded	absent	84.06	-0.34	included	present	1668.14	0.21
216577_at	excluded	absent	60.33	0.23	excluded	absent	44.42	-0.13	excluded	absent	2.38	-1.09	excluded	absent	12.97	-0.85	excluded	absent	5.79	-1.01
216578_at	excluded	absent	6.9	-0.99	excluded	absent	114.22	1.88	excluded	absent	40.63	-0.08	excluded	absent	28.26	-0.42	excluded	absent	87.75	1.17
216579_at	excluded	absent	3.27	-0.49	excluded	absent	33.41	-0.01	excluded	absent	4.39	-0.48	excluded	absent	7.92	-0.42	excluded	absent	2.71	-0.5
216580_at	excluded	absent	63.63	0.98	excluded	absent	5.02	-0.8	excluded	absent	6.03	-0.77	excluded	absent	3.27	-0.85	excluded	absent	107.57	2.31
216581_at	excluded	absent	14.68	0	excluded	absent	3.81	-0.47	excluded	absent	2.66	-0.52	excluded	absent	3.34	-0.49	excluded	absent	2.94	-0.51
216582_at	excluded	absent	2.32	-1.04	excluded	absent	5.63	-0.97	excluded	absent	40.54	-0.22	excluded	absent	75.62	0.53	excluded	absent	51.74	0.02
216583_x_at	included	present	338.16	1.02	excluded	absent	365.62	1.42	included	present	296.92	0.4	included	present	307.33	0.56	included	absent	34.92	-3.49
216584_at	included	present	230.41	-0.23	excluded	absent	111.81	-1.18	included	present	239.41	-0.16	included	present	162.39	-0.77	included	present	366.05	0.86
216585_at	excluded	absent	75.55	0.39	excluded	absent	195.78	2.71	excluded	absent	309.51	4.9	excluded	absent	196.96	2.73	excluded	absent	164.5	2.1
216586_at	excluded	absent	100.34	1.9	excluded	absent	124.12	2.55	excluded	absent	62.42	0.86	excluded	absent	40.25	0.25	excluded	absent	142.96	3.07
216587_s_at	excluded	absent	7.16	-0.46	excluded	absent	5.14	-0.53	excluded	absent	10.18	-0.34	excluded	absent	2.77	-0.62	excluded	absent	73.26	2.09
216588_at	excluded	absent	66.2	-0.89	excluded	absent	81.04	-0.59	included	present	75.79	-0.7	included	present	112.87	0.06	excluded	absent	66.14	-0.89
216589_at	excluded	absent	26.09	-0.95	included	present	126.7	1.23	included	present	78.82	0.19	excluded	absent	47.44	-0.49	excluded	absent	97.83	0.61
216590_at	excluded	absent	5.44	-0.46	excluded	absent	1	-0.58	excluded	absent	25.42	0.12	excluded	absent	23.42	0.06	excluded	absent	6	-0.44
216591_s_at	included	present	1287.64	-0.9	included	present	490.7	-1.79	included	present	1354.29	-0.83	included	present	1268.13	-0.93	included	present	1498.65	-0.67
216592_at	excluded	absent	43.71	0.19	excluded	absent	13.21	-0.67	excluded	absent	3.92	-0.93	excluded	absent	1.85	-0.99	excluded	absent	109.12	2.05
216593_s_at	included	present	2881.02	3.85	included	present	2000.63	1.8	included	present	2993.55	4.11	included	present	1550.15	0.75	included	present	1307.73	0.19
216594_x_at	included	present	4274.85	0.78	included	present	1172.32	-0.14	included	present	901.79	-0.22	included	present	221.19	-0.42	included	present	653.83	-0.29
216595_at	excluded	absent	0.85	-0.38	excluded	absent	2.79	-0.38	included	present	301.4	0.39	included	present	142.26	-0.02	excluded	absent	2.59	-0.38
216596_at	excluded	absent	20.81	-0.7	excluded	absent	177.55	1.53	included	present	514.25	6.33	excluded	absent	9.44	-0.87	included	present	220.12	2.14
216597_at	excluded	absent	8.13	-0.98	excluded	absent	11.81	-0.91	excluded	absent	60	-0.12	excluded	absent	3.92	-1.04	excluded	absent	17.95	-0.81
216598_s_at	included	present	2180.2	0.65	included	present	2953.36	1.16	included	present	600.29	-0.39	included	present	184.13	-0.67	included	present	343.48	-0.56
216599_x_at	excluded	absent	10.02	-0.22	excluded	absent	24.76	-0.13	excluded	absent	7.32	-0.24	excluded	absent	8.27	-0.23	excluded	absent	21.63	-0.15
216600_x_at	excluded	absent	3.7	-0.27	excluded	absent	19.94	-0.27	included	present	1236.79	-0.16	excluded	absent	129.33	-0.26	excluded	absent	7.47	-0.27
216601_at	included	present	104.63	0.72	excluded	absent	23.67	-0.81	excluded	absent	18.53	-0.91	excluded	absent	26.98	-0.75	excluded	absent	155.65	1.68
216602_s_at	included	present	358.54	1.97	included	present	434.86	2.95	included	present	295.92	1.16	included	present	545.33	4.38	excluded	absent	265.85	0.77
216603_at	included	present	224.14	0.05	excluded	absent	8.38	-0.47	excluded	absent	8.24	-0.47	excluded	absent	59.47	-0.35	excluded	absent	35.83	-0.4
216604_s_at	included	present	399.17	-0.36	included	absent	105.55	-0.62	included	present	168.32	-0.57	included	absent	20.77	-0.7	included	present	239.93	-0.5
216605_s_at	excluded	absent	66.86	0.93	excluded	absent	5.63	-0.72	excluded	absent	35.77	0.09	excluded	absent	36.14	0.1	excluded	absent	15.75	-0.44
216606_x_at	excluded	absent	105.97	0.03	excluded	absent	180.48	1.27	excluded	absent	12.78	-1.52	excluded	absent	117.97	0.23	excluded	absent	150.35	0.77
216607_s_at	included	present	2240.51	0.3	included	present	1537.07	-0.07	included	present	1191.52	-0.25	included	present	4455.5	1.45	included	present	2735.41	0.56
216608_at	excluded	absent	4.95	-0.67	excluded	absent	15.94	-0.33	excluded	absent	7.85	-0.58	excluded	absent	36.79	0.3	excluded	absent	17.86	-0.28
216609_at	included	present	4016.01	-0.37	included	present	3837.39	-0.45	included	present	7932.03	1.32	included	present	2671.16	-0.95	included	present	5520.02	0.28
216610_at	excluded	absent	38.12	-0.01	excluded	absent	20.27	-0.58	excluded	absent	9.36	-0.93	excluded	absent	44.64	0.19	excluded	absent	54.56	0.51
216611_s_at	excluded	absent	103.92	0.65	included	present	1097.33	15.55	included	present	789.91	10.94	excluded	absent	3.14	-0.87	included	present	2428.02	35.51
216612_x_at	excluded	absent	9.37	-1.16	excluded	absent	66.37	-0.09	excluded	absent	86.16	0.28	excluded	absent	68.31	-0.05	excluded	absent	13.05	-1.09
216613_at	excluded	absent	11.34	-0.66	excluded	absent	1.63	-0.76	excluded	absent	2.55	-0.75	excluded	absent	7.69	-0.7	excluded	absent	133.93	0.58
216614_at	excluded	absent	90.25	-0.24	excluded	absent	5.11	-0.58	excluded	absent	16.83	-0.53	excluded	absent	65.23	-0.34	excluded	absent	65.92	-0.34
216615_s_at	excluded	absent	8.05	-0.84	included	present	413.1	8.83	excluded	absent	8.33	-0.84	included	present	103.62	1.44	excluded	absent	42.44	-0.02
216616_at	included	present	211.57	2.48	included	present	149.48	1.18	included	present	336.47	5.08	included	present	192.31	2.08	included	present	433.61	7.1
216617_s_at	excluded	absent	9.69	-0.54	excluded	absent	47.92	-0.43	excluded	absent	39.22	-0.45	excluded	absent	4.2	-0.55	excluded	absent	10.59	-0.53
216618_at	excluded	absent	9.7	-0.57	excluded	absent	1.49	-0.85	excluded	absent	33.68	0.24	excluded	absent	1.66	-0.84	excluded	absent	5.13	-0.73
216619_at	excluded	absent	2.31	-0.82	excluded	absent	8.71	-0.62	excluded	absent	3.22	-0.79	excluded	absent	24.13	-0.13	excluded	absent	84.86	1.78
216620_s_at	included	present	3654.38	3.73	included	present	238.42	-1.67	included	present	532.69	-1.21	included	present	2214.3	1.45	included	present	982.5	-0.5
216621_at	included	present	293.07	0.63	included	present	264.98	0.29	included	present	141.91	-1.2	included	present	261.55	0.25	included	present	289.8	0.59
216622_at	excluded	absent	2.07	-0.29	excluded	absent	4.19	-0.28	excluded	absent	64.72	-0.14	excluded	absent	4.81	-0.28	excluded	absent	12.65	-0.26
216623_x_at	excluded	absent	34.97	-0.59	included	present	1813.21	9.64	included	present	544.34	2.34	included	present	860.32	4.16	included	present	610.79	2.72
216624_s_at	included	present	889.32	0.53	included	present	563.85	-0.74	included	present	1238.31	1.89	included	present	1205.56	1.76	included	present	489.48	-1.03
216625_at	included	present	234.3	0.21	excluded	absent	130.88	-0.76	included	present	169.12	-0.4	excluded	absent	108.12	-0.97	included	present	257.95	0.43
216626_at	included	absent	77.47	-1.07	included	absent	83.89	-1.01	included	present	161.33	-0.19	included	present	128.69	-0.53	included	present	111.8	-0.71
216627_s_at	included	present	530.66	-0.36	included	present	148.06	-0.95	included	present	285.84	-0.74	included	present	333.37	-0.67	included	present	551.82	-0.33
216628_at	included	present	274.55	0.93	included	present	102.82	-0.77	included	present	252.41	0.71	included	present	232.8	0.51	included	present	220.88	0.4
216629_at	included	present	199.66	-0.67	included	present	121.34	-1.03	included	present	219.34	-0.58	included	absent	107.18	-1.09	included	absent	150.51	-0.89
216630_at	excluded	absent	83.06	0.64	excluded	absent	15.05	-0.77	excluded	absent	124.49	1.5	excluded	absent	125.7	1.53	excluded	absent	34.86	-0.36
216631_s_at	excluded	absent	4.42	-0.78	excluded	absent	14.34	-0.53	excluded	absent	6.86	-0.72	excluded	absent	37.57	0.06	excluded	absent	8.85	-0.67
216632_at	excluded	absent	166.37	1.39	excluded	absent	23.26	-0.7	excluded	absent	38.76	-0.47	excluded	absent	63.97	-0.1	excluded	absent	4.49	-0.97
216633_s_at	excluded	absent	85.37	-0.27	excluded	absent	95.37	-0.15	excluded	absent	72.52	-0.42	excluded	absent	4.3	-1.24	excluded	absent	126.44	0.22
216634_at	excluded	absent	4.37	-0.71	excluded	absent	2.35	-0.78	excluded	absent	41.22	0.47	excluded	absent	40.69	0.45	excluded	absent	6.94	-0.63
216635_at	excluded	absent	57.63	0.54	excluded	absent	49.47	0.31	excluded	absent	17.65	-0.56	excluded	absent	3.29	-0.96	excluded	absent	67.4	0.81
216636_at	excluded	absent	66.5	0.2	excluded	absent	6.85	-0.86	excluded	absent	42.21	-0.23	excluded	absent	33.33	-0.39	excluded	absent	5.86	-0.88
216637_at	excluded	absent	38.5	0.04	excluded	absent	94.57	1.61	excluded	absent	9.72	-0.76	excluded	absent	2.92	-0.96	excluded	absent	6.3	-0.86
216638_s_at	excluded	absent	66.95	-0.42	excluded	absent	65.43	-0.43	excluded	absent	39.59	-0.52	excluded	absent	1.5	-0.65	excluded	absent	3.71	-0.64
216639_at	included	present	153.27	-0.11	excluded	absent	49.73	-0.65	included	present	381.41	1.09	excluded	absent	42.7	-0.69	excluded	absent	124.65	-0.26
216640_s_at	included	present	1952.94	0.17	included	present	1609.04	-0.09	included	present	2324.53	0.45	included	present	3212.83	1.12	included	present	1305.6	-0.32
216641_s_at	excluded	absent	52.62	-0.54	excluded	absent	136.54	0.11	excluded	absent	21.57	-0.78	excluded	absent	36.18	-0.66	excluded	absent	25.48	-0.75
216642_at	excluded	absent	6.14	-0.81	excluded	absent	62.15	0.89	excluded	absent	16.48	-0.5	excluded	absent	84.15	1.56	excluded	absent	8.86	-0.73
216643_at	excluded	absent	50.62	-0.69	included	present	210.57	0.07	excluded	absent	67.59	-0.61	excluded	absent	34.72	-0.77	excluded	absent	2.89	-0.92
216644_at	included	present	172.53	0.46	excluded	absent	5.52	-1.72	excluded	absent	78.83	-0.77	included	present	146.25	0.12	excluded	absent	117.31	-0.26
216645_at	excluded	absent	30.61	-0.77	excluded	absent	82.94	0.03	excluded	absent	50.72	-0.46	excluded	absent	105.43	0.38	excluded	absent	102.45	0.33
216646_at	included	present	1220.2	3.22	included	absent	69.36	-1.12	included	present	358.47	-0.03	included	present	217.02	-0.56	included	present	238	-0.48

[illegible]

216916_s_at	excluded	absent	54.08	-0.48	excluded	absent	6.4	-0.81	excluded	absent	101.64	-0.15	excluded	absent
216917_s_at	excluded	absent	2.41	-0.27	excluded	absent	4.97	-0.26	excluded	absent	7.91	-0.25	excluded	absent
216918_s_at	included	present	1382.55	-0.34	included	present	2208.93	-0.1	included	present	1548.66	-0.29	included	absent
216919_at	excluded	absent	81.66	1.08	excluded	absent	45.82	0.25	excluded	absent	6.16	-0.65	excluded	absent
216920_s_at	excluded	absent	137.81	-0.29	excluded	absent	65.7	-0.44	excluded	absent	40.17	-0.49	excluded	absent
216921_s_at	excluded	absent	6.98	-0.44	excluded	absent	11.95	-0.39	excluded	absent	7.4	-0.44	excluded	absent
216922_x_at	excluded	absent	35.39	-0.15	excluded	absent	50.64	-0.02	excluded	absent	37.13	-0.14	excluded	absent
216923_at	excluded	absent	4.59	-0.75	excluded	absent	75.28	1.35	excluded	absent	2.69	-0.81	excluded	absent
216924_s_at	included	present	148.16	-0.05	excluded	absent	191.68	0.19	excluded	absent	92.55	-0.36	excluded	absent
216925_s_at	included	present	152.15	-0.31	excluded	absent	135.14	-0.51	excluded	absent	28.65	-1.72	excluded	absent
216926_s_at	included	present	130.46	0.28	excluded	absent	32.13	-1.09	included	present	111.41	0.02	included	present
216927_at	excluded	absent	10.83	-0.38	excluded	absent	65.37	1.33	excluded	absent	5.78	-0.53	excluded	absent
216928_at	excluded	absent	28.93	-0.97	excluded	absent	74.76	0.2	excluded	absent	45.95	-0.54	excluded	absent
216929_x_at	excluded	absent	416.07	-0.31	excluded	absent	338.81	-0.4	excluded	absent	550.07	-0.17	excluded	absent
216930_at	excluded	absent	11.35	-0.88	excluded	absent	27.37	-0.51	excluded	absent	110.75	1.45	excluded	absent
216931_at	excluded	absent	47.46	0.64	excluded	absent	85.45	2.03	excluded	absent	2.99	-0.98	excluded	absent
216932_at	excluded	absent	33.96	0.1	excluded	absent	48.74	0.5	excluded	absent	4	-0.72	excluded	absent
216933_x_at	included	present	2728.73	-0.36	included	present	1578.21	-0.66	included	present	3238.56	-0.22	included	present
216934_at	included	present	149.37	-0.04	included	absent	112.74	-0.4	included	present	180.05	0.26	included	present
216935_at	excluded	absent	37.49	-0.2	excluded	absent	10.9	-0.3	excluded	absent	41.69	-0.18	excluded	absent
216936_at	excluded	absent	88.54	0.53	excluded	absent	5.49	-1	excluded	absent	53.18	-0.12	excluded	absent
216937_s_at	excluded	absent	4.77	-0.8	excluded	absent	2.32	-0.86	excluded	absent	70.81	0.65	excluded	absent
216938_x_at	excluded	absent	10.89	-0.49	included	present	338.81	0.87	excluded	absent	5.43	-0.51	excluded	absent
216939_s_at	excluded	absent	4.09	-0.64	excluded	absent	9.45	-0.45	excluded	absent	3.77	-0.65	excluded	absent
216940_x_at	excluded	absent	17.7	-0.62	excluded	absent	19.45	-0.59	excluded	absent	56.04	0.09	excluded	absent
216941_s_at	included	present	665.51	1.84	included	present	484.79	0.6	included	present	1250.3	5.88	included	present
216942_s_at	included	present	531.02	1.14	included	absent	95.57	-1.07	included	present	89.98	-1.1	included	present
216943_at	included	present	277.09	-0.44	excluded	absent	63.24	-0.56	excluded	absent	6.73	-0.59	included	present
216944_s_at	included	present	462.84	-0.03	included	absent	88.86	-1.24	included	present	91.5	-1.23	included	absent
216945_x_at	excluded	absent	123.87	-0.55	included	present	812.27	0.35	included	present	169.16	-0.49	included	present
216946_at	included	present	103.03	1.28	excluded	absent	4.49	-1.15	included	present	77.15	0.64	excluded	absent
216947_at	excluded	absent	5.89	-1.64	excluded	absent	2.34	-1.72	excluded	absent	75.53	-0.12	excluded	absent
216948_at	excluded	absent	10.29	-0.48	excluded	absent	2.59	-0.81	excluded	absent	38.25	0.71	excluded	absent
216949_s_at	included	present	153.86	0.11	excluded	absent	78.93	-0.69	excluded	absent	78.28	-0.7	excluded	absent

[illegible]

217280_x_at	excluded	absent	4.98	-0.67	excluded	absent	8.06	-0.66	excluded	absent	14.45	-0.63	excluded	absent	28.58	-0.57	excluded	absent	76.44	-0.35	excluded	absent	4.97	-0.67
217281_x_at	excluded	absent	29.97	-0.3	excluded	absent	105.71	-0.28	excluded	absent	56.92	-0.29	excluded	absent	149.07	-0.26	included	present	1599.81	0.3	excluded	absent	156.52	-0.26
217282_at	included	absent	82.45	-1.35	included	present	210.21	0.56	included	present	221.32	0.73	included	present	103.48	-1.03	included	present	375.06	3.03	included	absent	65.4	-1.6
217283_at	excluded	absent	7.88	-0.76	excluded	absent	38.07	-0.17	excluded	absent	8.74	-0.74	excluded	absent	7.58	-0.76	excluded	absent	6.36	-0.78	excluded	absent	142.07	1.86
217284_x_at	excluded	absent	89.69	-0.29	included	present	159.61	0.45	included	present	82.42	-0.37	excluded	absent	16.37	-1.07	excluded	absent	2.97	-1.21	included	present	125.68	0.09
217285_at	excluded	absent	123.02	0.95	excluded	absent	112.16	0.73	excluded	absent	17.09	-1.17	excluded	absent	88.67	0.26	excluded	absent	139.45	1.27	excluded	absent	56.16	-0.39
217286_s_at	included	present	2467.01	0.04	included	present	2477.49	0.05	included	present	1372.62	-0.68	included	present	975.1	-0.94	included	present	2799.81	0.26	included	present	1408.07	-0.65
217287_s_at	included	present	88.27	-0.38	excluded	absent	12.41	-0.7	excluded	absent	5.11	-0.73	included	present	127.56	-0.21	included	present	117.42	-0.25	included	present	300.45	0.52
217288_at	excluded	absent	3.16	-0.54	excluded	absent	5.35	-0.51	excluded	absent	40.24	-0.1	excluded	absent	33.19	-0.18	excluded	absent	7.41	-0.49	excluded	absent	8.17	-0.48
217289_s_at	excluded	absent	3.56	-0.62	excluded	absent	8.11	-0.57	excluded	absent	21.52	-0.4	excluded	absent	98.74	0.53	excluded	absent	5.52	-0.6	excluded	absent	22.03	-0.4
217290_at	excluded	absent	37.3	-0.03	excluded	absent	11.14	-0.76	excluded	absent	58.97	0.58	excluded	absent	18.28	-0.56	excluded	absent	114.75	2.15	excluded	absent	6.83	-0.88
217291_at	excluded	absent	3.14	-0.33	excluded	absent	5.66	-0.32	excluded	absent	19.17	-0.23	excluded	absent	138.57	0.55	excluded	absent	72.84	0.12	excluded	absent	6.17	-0.31
217292_at	excluded	absent	110.38	-0.11	excluded	absent	214.26	1.46	excluded	absent	143.43	0.39	excluded	absent	174.65	0.86	excluded	absent	204.55	1.31	excluded	absent	134.46	0.25
217293_at	included	present	75.9	-0.42	excluded	absent	108.75	0.17	excluded	absent	49.17	-0.9	excluded	absent	88.27	-0.2	excluded	absent	117.83	0.33	excluded	absent	52.15	-0.85
217294_s_at	included	present	846.48	3.68	included	present	165.02	-1.34	included	present	602.53	1.88	included	present	283.91	-0.47	included	present	924.2	4.25	included	present	700.83	2.6
217295_at	excluded	absent	2.56	-0.94	excluded	absent	37.7	0.34	excluded	absent	34.18	0.21	excluded	absent	27.36	-0.03	excluded	absent	103.99	2.77	excluded	absent	10.58	-0.65
217296_at	excluded	absent	60.47	0.19	excluded	absent	2.95	-1.2	excluded	absent	35.7	-0.41	excluded	absent	7.52	-1.09	excluded	absent	141.25	2.15	excluded	absent	19.14	-0.81
217297_s_at	excluded	absent	107.06	0.12	excluded	absent	151.8	0.81	excluded	absent	88.75	-0.16	included	present	43.98	-0.85	excluded	absent	127.13	0.43	included	present	251.02	2.34
217298_at	excluded	absent	26.87	0.03	excluded	absent	8.17	-0.77	excluded	absent	46.55	0.86	excluded	absent	26.95	0.03	excluded	absent	63.12	1.57	excluded	absent	61.56	1.5
217299_s_at	included	present	604.59	-0.27	included	present	252.38	-1.57	included	present	711.12	0.12	included	present	490.19	-0.69	included	present	759.75	0.3	included	present	725.63	0.18
217300_at	included	present	2296.76	1.1	included	present	645.47	-2.97	included	present	2578.2	1.8	included	present	1955.8	0.26	included	present	1359.97	-1.21	included	present	1396.23	-1.12
217301_x_at	included	present	1576.02	0.68	included	present	1927.88	1.44	included	present	2409.34	2.49	included	present	3817.2	5.56	included	present	6629.69	11.7	included	present	1108.04	-0.34
217302_at	excluded	absent	3.02	-0.62	excluded	absent	1.71	-0.67	excluded	absent	2.64	-0.64	excluded	absent	2.06	-0.66	excluded	absent	4.09	-0.58	excluded	absent	7.4	-0.46
217303_s_at	excluded	absent	4.08	-0.77	excluded	absent	2.55	-0.82	excluded	absent	20.58	-0.27	excluded	absent	2.95	-0.8	excluded	absent	4.72	-0.75	excluded	absent	7.41	-0.67
217304_at	excluded	absent	5.32	-1.15	included	present	76.54	0.48	excluded	absent	92.65	0.84	excluded	absent	54.14	-0.04	excluded	absent	7.65	-1.1	excluded	absent	16.17	-0.9
217305_s_at	excluded	absent	43.35	-0.1	excluded	absent	122.48	0.33	excluded	absent	15.14	-0.25	excluded	absent	3.24	-0.31	excluded	absent	6.03	-0.3	excluded	absent	15.74	-0.24
217306_at	excluded	absent	40.2	0.34	excluded	absent	3.38	-0.88	included	present	119.57	2.96	excluded	absent	35.98	0.2	excluded	absent	60.75	1.01	excluded	absent	44.58	0.48
217307_at	excluded	absent	76.92	2.09	excluded	absent	10.94	-0.35	excluded	absent	10.41	-0.37	excluded	absent	15.55	-0.18	excluded	absent	35.07	0.54	excluded	absent	40.26	0.73
217308_at	excluded	absent	83.39	1	excluded	absent	65.73	0.52	excluded	absent	20.85	-0.7	excluded	absent	3.57	-1.17	excluded	absent	2.61	-1.2	excluded	absent	2.32	-1.2
217309_s_at	included	present	456.42	0.7	excluded	absent	335.83	-0.26	included	present	299.74	-0.54	included	present	362.2	-0.05	included	present	344.06	-0.19	included	present	497.56	1.03
217310_s_at	included	present	602.5	2.97	included	present	357.66	0.13	included	present	308.3	-0.44	included	present	1205.79	9.96	included	present	343.77	-0.03	included	present	474.97	1.49
217311_at	excluded	absent	4.68	-0.6	excluded	absent	14.06	-0.35	excluded	absent	2.58	-0.66	excluded	absent	14.7	-0.33	excluded	absent	4.63	-0.6	excluded	absent	2.84	-0.65
217312_s_at	excluded	absent	78.43	0.66	excluded	absent	9.36	-0.39	excluded	absent	4.01	-0.47	excluded	absent	12.17	-0.34	excluded	absent	87.8	0.81	excluded	absent	119.95	1.29
217313_at	excluded	absent	60.55	-1.13	included	present	214.96	0.54	excluded	absent	91.75	-0.79	excluded	absent	95.65	-0.75	included	present	167.8	0.03	included	present	243.7	0.85
217314_at	excluded	absent	13.82	-0.81	excluded	absent	87.86	1.03	excluded	absent	15.43	-0.77	excluded	absent	42.41	-0.1	excluded	absent	21.62	-0.62	excluded	absent	25.06	-0.53
217315_s_at	excluded	absent	9.41	-1.02	excluded	absent	41.8	-0.23	excluded	absent	5.06	-1.12	excluded	absent	61.35	0.24	excluded	absent	77.3	0.63	excluded	absent	51.35	0
217316_at	excluded	absent	63.15	0.02	excluded	absent	58.83	-0.08	excluded	absent	24.59	-0.84	excluded	absent	38.58	-0.52	excluded	absent	56.32	-0.13	excluded	absent	60.54	-0.04
217317_s_at	included	present	2019.23	-0.67	included	present	2896.3	-0.17	included	present	1932.91	-0.72	included	present	2472.91	-0.41	included	present	769.11	-1.38	included	present	2069.73	-0.64
217318_x_at	excluded	absent	2.92	-0.58	excluded	absent	3.11	-0.57	excluded	absent	3.24	-0.56	excluded	absent	14.78	-0.06	excluded	absent	67.89	2.26	excluded	absent	64.84	2.13
217319_x_at	included	present	207.55	-0.22	included	present	182.86	-0.23	included	present	495.64	-0.11	included	present	202.32	-0.22	included	present	264.17	-0.2	included	absent	75.18	-0.27
217320_at	excluded	absent	17.53	-0.34	excluded	absent	7.62	-0.39	excluded	absent	14.69	-0.35	excluded	absent	11.82	-0.37	included	present	231.38	0.77	excluded	absent	9.79	-0.38
217321_x_at	excluded	absent	96.2	0.54	excluded	absent	69.32	-0.09	excluded	absent	100.45	0.64	included	present	117.16	1.03	excluded	absent	108.07	0.82	excluded	absent	7.27	-1.53
217322_x_at	included	present	1223.72	0.33	included	present	1646.77	1.61	included	present	1234.37	0.36	included	present	1158.78	0.13	included	present	1190.94	0.23	included	present	1199.73	0.26
217323_at	excluded	absent	58.94	0.06	excluded	absent	55.6	-0.03	excluded	absent	5.22	-1.34	excluded	absent	27.26	-0.77	excluded	absent	91.35	0.91	excluded	absent	77.75	0.55
217324_at	excluded	absent	10.09	-1.01	excluded	absent	17.46	-0.81	excluded	absent	20.82	-0.71	excluded	absent	63.79	0.46	excluded	absent	12.51	-0.94	excluded	absent	4.1	-1.17
217325_at	excluded	absent	92.33	-0.28	excluded	absent	35.75	-1.05	excluded	absent	121.16	0.11	excluded	absent	80.27	-0.45	excluded	absent	16.12	-1.32	excluded	absent	167.07	0.74
217326_x_at	included	present	168.05	0.95	excluded	absent	12.77	-1.27	excluded	absent	66.31	-0.5	excluded	absent	5.31	-1.38	included	present	197.14	1.37	included	present	187.71	1.23
217327_at	included	present	38.6	-0.77	excluded	absent	34.62	-0.82	excluded	absent	52.55	-0.59	excluded	absent	41.64	-0.73	excluded	absent	12.81	-1.1	excluded	absent	20.32	-1
217328_at	excluded	absent	8.89	-0.68	excluded	absent	8.68	-0.68	excluded	absent	64.84	-0.19	excluded	absent	71.28	-0.14	excluded	absent	71.28	-0.14	included	present	314.03	1.99
217329_x_at	included	present	520.55	-0.39	included	present	424.31	-0.68	included	present	633.22	-0.05	included	present	365.94	-0.86	included	present	426.55	-0.67	included	present	870.66	0.67
217330_at	excluded	absent	16.57	-0.55	excluded	absent	3.82	-0.92	excluded	absent	11.8	-0.69	excluded	absent	50.2	0.44	excluded	absent	32.08	-0.1	excluded	absent	49.77	0.42
217331_at	excluded	absent	103.03	0.72	excluded	absent	29.98	-1.04	excluded	absent	47.72	-0.61	excluded	absent	63.04	-0.24	excluded	absent	15.79	-1.38	excluded	absent	49.05	-0.58
217332_at	excluded	absent	51.57	0.19	excluded	absent	22.83	-0.59	excluded	absent	6.78	-1.02	excluded	absent	6.44	-1.03	excluded	absent	6.31	-1.03	excluded	absent	5.35	-1.06
217333_at	excluded	absent	6.17	-0.38	excluded	absent	9.68	-0.21	excluded	absent	3.99	-0.48	excluded	absent	13.02	-0.05	excluded	absent	92.96	3.7	excluded	absent	2.94	-0.53
217334_at	excluded	absent	18.99	0.02	excluded	absent	8.95	-0.29	excluded	absent	2.03	-0.51	excluded	absent	12.42	-0.18	excluded	absent	12.07	-0.2	excluded	absent	8.91	-0.29
217335_at	excluded	absent	63.52	1.81	excluded	absent	53.03	1.38	excluded	absent	43.1	0.97	excluded	absent	30.42	0.45	excluded	absent	3.34	-0.67	excluded	absent	9.19	-0.43
217336_at	excluded	absent	74.59	-0.94	excluded	absent	154.56	0.49	excluded	absent	136.35	0.17	excluded	absent	88.03	-0.7	included	present	22.26	-1.87	included	present	234.85	1.93
217337_at	excluded	absent	101.06	2.18	excluded	absent	123.51	2.86	excluded	absent	2.6	-0.78	excluded	absent	3.65	-0.75	excluded	absent	46.1	0.53	excluded	absent	7.11	-0.65
217338_at	excluded	absent	39.17	-0.01	excluded	absent	4.71	-0.88	excluded	absent	2.6	-0.93	excluded	absent	1.61	-0.96	excluded	absent	9.89	-0.75	excluded	absent	18.48	-0.53
217339_x_at	excluded	absent	93.57	1.53	included	present	926.24	24.68	excluded	absent	54.64	0.45	excluded	absent	1.14	-1.04	included	present	380.12	9.5	included	present	60.73	0.62
217340_at	excluded	absent	116.1	0.42	excluded	absent	106.49	0.26	excluded	absent	67.89	-0.38	included	present	93.17	0.04	excluded	absent	132.22	0.68	excluded	absent	73.21	-0.29
217341_at	included	present	731.97	0.43	included	present	398.62	-0.19	included	present	376.67	-0.24	excluded	absent	48.24	-0.85	included	present	884.42	0.71	included	present	735.56	0.44
217342_x_at	excluded	absent	23.25	-1	excluded	absent	4.11	-1.27	excluded	absent	2.13	-1.3	excluded	absent	36.15	-0.81	included	present	115.53	0.34	excluded	absent	111.03	0.28
217343_at	excluded	absent	2.23	-1.05	excluded	absent	3.62	-1.01	excluded	absent	41.63	-0.07	excluded	absent	14.97	-0.73	excluded	absent	1.9	-1.06	excluded	absent	3.16	-1.02
217344_at	excluded	absent	113.43	0.34	excluded	absent	80.9	-0.15	excluded	absent	97.98	0.11	excluded	absent	88.46	-0.04	excluded	absent	147.52	0.86	included	present	224.82	2.02
217345_at	excluded	absent	3.02	-1.05	excluded	absent	7.53	-0.92	excluded	absent	51.14	0.32	excluded	absent	32.52	-0.21	excluded	absent	3.73	-1.03	excluded	absent	57.03	0.49

217640_x_at	excluded	absent	0.52	-0.65	included	present	114.68	1.85	excluded	absent	47.58	0.38	excluded	absent	38.87
217641_at	excluded	absent	116.92	-0.34	included	present	275.55	0.58	excluded	absent	61.95	-0.66	excluded	absent	55.48
217642_at	included	present	137.45	0.95	excluded	absent	176.46	1.8	excluded	absent	53.89	-0.86	excluded	absent	68.03
217643_x_at	included	present	1193.57	1.62	included	present	738.35	0.33	included	present	1083.49	1.31	included	present	787.87
217644_s_at	included	present	1005.47	0.86	included	present	845.17	0.25	included	present	942.34	0.62	included	present	1444.29
217645_at	included	present	348.08	1.19	included	present	142.07	-0.64	included	present	398.94	1.65	included	present	383.67
217646_at	excluded	absent	51.91	-0.36	excluded	absent	20.46	-0.93	excluded	absent	78.63	0.13	excluded	absent	81.09
217647_at	excluded	absent	137.28	0.36	excluded	absent	205.1	0.9	included	present	172.04	0.63	excluded	absent	136.86
217648_at	included	present	115.42	0.61	included	present	193.23	1.83	excluded	absent	58.84	-0.28	included	present	111.56
217649_at	included	present	118.22	-0.59	included	absent	14.42	-1.08	included	present	154.22	-0.43	included	present	103.6
217650_x_at	included	present	909.2	0.26	included	present	1586.68	2.25	included	present	2042.4	3.59	included	present	280.2
217651_at	excluded	absent	342.04	0.65	excluded	absent	91.78	-0.89	included	present	586.87	2.17	included	present	324.66
217652_at	excluded	absent	6.75	-0.32	excluded	absent	7.39	-0.3	excluded	absent	29.16	0.54	excluded	absent	31.47
217653_x_at	included	present	619.09	-1.14	included	present	713.54	-0.91	included	present	826.08	-0.64	included	present	439.96
217654_at	excluded	absent	84.73	0.33	excluded	absent	4.79	-1.31	excluded	absent	57.49	-0.23	excluded	absent	33.72
217655_at	excluded	absent	38.9	-0.46	excluded	absent	3.14	-1.05	excluded	absent	4.19	-1.04	excluded	absent	94.48
217656_at	excluded	absent	42.61	-0.74	excluded	absent	76.31	0.04	included	present	122.86	1.11	excluded	absent	109.49
217657_at	included	present	129.21	0.7	excluded	absent	3.54	-1.63	excluded	absent	11.06	-1.49	included	present	160.45
217658_at	excluded	absent	11.06	-0.65	excluded	absent	26.89	-0.19	excluded	absent	13.4	-0.58	excluded	absent	4.8
217659_at	included	present	199.6	-1.08	included	absent	53.63	-2.08	included	present	305.12	-0.35	included	present	211.61
217660_at	excluded	absent	145.96	0.16	excluded	absent	113.17	-0.13	excluded	absent	7.99	-1.05	excluded	absent	78.57
217661_x_at	excluded	absent	21.36	-0.68	excluded	absent	106.7	0.86	excluded	absent	5.24	-0.97	excluded	absent	19.33
217662_x_at	included	present	828.32	-0.25	included	present	320.93	-1.4	included	present	759.77	-0.41	included	present	914.52
217663_at	included	present	242.84	2.75	excluded	absent	125.88	0.14	included	present	144.95	0.57	included	present	300.71
217664_at	excluded	absent	71.89	-0.63	excluded	absent	139.71	0.49	excluded	absent	30.52	-1.31	excluded	absent	60.23
217665_at	included	present	144.42	-0.02	included	present	244.51	1.41	included	present	187.66	0.59	included	present	213.72
217666_at	excluded	absent	71.9	0.3	excluded	absent	44.81	-0.23	included	present	131.44	1.45	excluded	absent	60.32
217667_at	excluded	absent	43.27	-0.04	excluded	absent	93.28	1.29	excluded	absent	77.99	0.88	excluded	absent	76.54
217668_at	included	present	263.22	0.76	included	present	162.58	-0.29	included	present	160.77	-0.31	included	present	141.62
217669_s_at	included	present	1160.57	0.79	included	present	720.86	0.1	included	present	383.58	-0.44	included	present	335.22
217670_at	included	absent	42.75	-2.01	included	present	192.67	0.03	included	present	126.73	-0.86	included	present	156.28
217671_at	included	absent	49.62	-1.26	included	absent	85.58	-0.75	included	present	119.48	-0.27	included	present	182.72
217672_x_at	included	present	479.74	0.03	included	present	782.75	1.43	included	present	368.14	-0.48	included	absent	343.59
217673_x_at	included	present	7688.43	0.74	included	present	10473.51	1.42	included	present	10578.94	1.44	included	present	10857.57
217674_at	excluded	absent	98.82	0.51	excluded	absent	52.2	-0.01	excluded	absent	104.37	0.57	excluded	absent	119.05
217675_at	excluded	absent	104.43	4.01	excluded	absent	15.03	0.05	excluded	absent	4.99	-0.4	excluded	absent	36.97
217676_at	excluded	absent	49.4	0.45	excluded	absent	69.49	1.04	excluded	absent	44.7	0.31	excluded	absent	2.49
217677_at	included	present	449.45	2.11	included	absent	77.44	-1.67	included	absent	97.83	-1.46	included	present	308.3
217678_at	excluded	absent	10.66	-0.72	excluded	absent	4.9	-0.73	excluded	absent	33.91	-0.65	excluded	absent	722.57
217679_x_at	included	present	13207.56	-0.38	included	present	12736.98	-0.48	included	present	17213.78	0.45	included	present	10068.88
217680_x_at	excluded	absent	147.14	1.26	excluded	absent	30.04	-0.91	excluded	absent	45.96	-0.62	excluded	absent	10
217681_at	excluded	absent	13.23	-0.4	excluded	absent	10.22	-0.47	excluded	absent	11.73	-0.44	excluded	absent	5.23
217682_at	included	present	1986.88	3.06	included	present	673.7	-0.74	included	present	1851.53	2.67	included	present	696.29
217683_at	excluded	absent	47.64	0.41	excluded	absent	46.77	0.38	excluded	absent	65.99	1.02	excluded	absent	40.93
217684_at	included	present	99.01	-0.88	included	absent	48.81	-1.2	included	absent	116.06	-0.77	included	present	410.32
217685_at	excluded	absent	30.39	0.31	excluded	absent	38.63	0.64	excluded	absent	119.57	3.87	excluded	absent	7.17
217686_at	excluded	absent	36.78	-0.14	excluded	absent	97.55	1.43	excluded	absent	15.16	-0.7	excluded	absent	72.4
217687_at	included	absent	68.28	-1.04	included	absent	81.87	-0.98	included	absent	74.12	-1.01	included	present	246.32
217688_at	included	absent	68.08	-1.01	included	absent	103.49	-0.9	included	absent	90.59	-0.94	included	present	222.22
217689_at	excluded	absent	100.56	-0.03	excluded	absent	163.66	0.97	included	present	147.97	0.72	included	present	100.58
217690_at	included	present	123.51	-0.63	excluded	absent	81.85	-0.78	included	present	154.98	-0.52	included	present	555.98
217691_x_at	included	present	267.83	-0.1	included	present	385.9	0.79	included	present	159.18	-0.92	included	present	147.74
217692_at	included	present	316.97	0.07	included	present	201.13	-0.74	included	present	577.41	1.88	included	present	451.62
217693_x_at	included	present	165.8	-0.76	included	absent	89.96	-1.55	included	present	473.15	2.41	included	present	200.85
217694_at	excluded	absent	3.32	-0.78	excluded	absent	6.76	-0.62	excluded	absent	8.74	-0.54	excluded	absent	25.88
217695_x_at	included	present	323.37	-0.67	included	present	188.63	-1.31	included	present	373.22	-0.44	included	present	196.73
217696_at	excluded	absent	52.28	-0.11	excluded	absent	43.42	-0.26	excluded	absent	7.81	-0.86	excluded	absent	4.05
217697_at	excluded	absent	79.36	0.46	excluded	absent	44.13	-0.27	excluded	absent	86.1	0.6	excluded	absent	32.91
217698_at	excluded	absent	4.09	-0.84	excluded	absent	12.29	-0.54	excluded	absent	38.83	0.42	excluded	absent	48.43
217699_at	excluded	absent	14.74	0.01	excluded	absent	26.01	0.6	excluded	absent	2.98	-0.6	excluded	absent	1.38
217700_at	excluded	absent	189.41	0.76	excluded	absent	298.96	1.86	excluded	absent	189.65	0.76	included	present	298.11

217998_at	included	present	4651.99	0.78	included	present	1221.17	-0.13	included	present	1329.06	-0.1	included	present	4118.24	0.63
217999_s_at	included	present	1150.86	0.98	included	present	222.91	-0.15	included	present	224.48	-0.15	included	present	760.52	0.51
218000_s_at	included	present	1304.98	1.08	included	present	455.74	0.09	included	present	93.05	-0.34	included	present	513.08	0.15
218001_at	included	absent	108.84	-1.26	excluded	absent	264.59	0.17	included	present	244.67	-0.02	included	present	177.01	-0.63
218002_s_at	included	present	795.1	-0.2	included	absent	190.19	-0.38	included	absent	3.53	-0.43	included	absent	7.78	-0.43
218003_s_at	included	present	6201.05	-0.37	included	present	13751.88	2.35	included	present	9452.54	0.8	included	present	9404.27	0.78
218004_at	included	present	1019.43	-0.55	included	present	1050.96	-0.45	included	present	433.23	-2.46	included	present	2014.7	2.69
218005_at	included	present	2572.91	0.29	included	present	2634.81	0.36	included	present	5492.53	3.54	included	present	4864.02	2.84
218006_s_at	included	present	396.6	1.52	included	present	210.75	-0.45	included	present	656.33	4.27	included	present	441.28	1.99
218007_s_at	included	present	3773.58	-0.16	included	present	5066.09	0.4	included	present	2320.73	-0.8	included	present	2388.59	-0.77
218008_at	included	present	698.44	0.39	included	present	300.2	-1.3	included	present	460.3	-0.62	included	present	863.98	1.09
218009_s_at	included	present	195.71	-0.2	included	present	1452.89	3.2	included	present	2089.24	4.92	included	present	1422.28	3.12
218010_x_at	included	present	441.58	0.55	included	present	524.23	0.96	included	absent	275.54	-0.27	included	absent	211.37	-0.58
218011_at	included	present	2822.02	-0.54	included	present	2867.08	-0.5	included	present	3827.95	0.26	included	present	3131.87	-0.29
218012_at	included	present	368.27	-0.5	included	present	184.83	-1.08	included	absent	103.43	-1.34	included	present	388.81	-0.43
218013_x_at	included	present	218.5	-0.92	included	present	243.77	-0.61	included	present	494.01	2.45	included	present	375.17	1
218014_at	included	present	1002.58	0.69	included	present	1950.34	4.13	included	present	1650.5	3.04	included	present	2737.58	6.98
218015_s_at	excluded	absent	67.06	-0.27	excluded	absent	35.34	-0.9	excluded	absent	68.86	-0.24	excluded	absent	56.11	-0.49
218016_s_at	included	present	1060.92	-0.73	included	present	2418.33	0.89	included	present	1331.05	-0.41	included	present	966.98	-0.85
218017_s_at	included	present	2600.17	2.38	included	present	970.98	-0.34	included	present	1827.75	1.09	included	present	1646.18	0.79
218018_at	included	present	1431.93	-0.55	included	present	1032.1	-0.79	included	present	671.1	-1.01	included	present	839.73	-0.91
218019_s_at	included	present	682.46	-0.59	included	present	716.32	-0.55	included	present	209.67	-1.09	included	present	264.58	-1.03
218020_s_at	excluded	absent	164.17	0.55	included	present	141.92	0.13	excluded	absent	98.13	-0.7	included	present	153.4	0.35
218021_at	included	present	295.25	-0.25	included	present	229.06	-0.37	included	present	205.22	-0.42	included	present	395.41	-0.07
218022_at	included	present	202.87	-0.28	included	absent	15.9	-0.83	included	absent	124.13	-0.51	included	present	394.37	0.29
218023_s_at	included	present	791.38	0.68	included	present	498.32	-0.74	included	present	520.59	-0.63	included	present	716.09	0.32
218024_at	included	present	1102.73	-1.05	included	present	492.76	-1.43	included	present	879.22	-1.19	included	present	1209.75	-0.99
218025_s_at	included	present	562.33	-0.63	included	present	405.96	-0.74	included	present	745.49	-0.49	included	present	430.33	-0.72
218026_at	included	present	2529.58	0.24	included	present	3403.18	1.02	included	present	5945.88	3.27	included	present	1532.23	-0.64
218027_at	included	present	716.83	-0.73	included	present	1025.11	-0.26	included	present	1953.33	1.17	included	present	1158.63	-0.05
218028_at	excluded	absent	233.13	0.31	excluded	absent	100.29	-0.79	excluded	absent	10.43	-1.54	excluded	absent	14.65	-1.5
218029_at	excluded	absent	267.54	0.56	included	present	168.73	-0.25	excluded	absent	32.83	-1.36	excluded	absent	134.33	-0.53
218030_at	included	present	2326.88	-0.19	included	present	2263.04	-0.21	included	present	951.92	-0.71	included	present	430.17	-0.91
218031_s_at	included	present	829.97	-0.29	included	present	1048.94	0.05	included	present	2916.89	2.99	included	present	1367.15	0.55
218032_at	included	present	1369.59	-0.41	included	present	2647.13	0.19	included	present	661.42	-0.74	included	present	350.63	-0.89
218033_s_at	excluded	absent	8.15	-0.88	excluded	absent	40.54	-0.48	excluded	absent	3.37	-0.94	excluded	absent	3.56	-0.94
218034_at	included	present	1051.01	-0.39	included	present	2638.5	2.85	included	present	622.71	-1.26	included	present	457.89	-1.6
218035_s_at	included	present	609.51	-0.33	included	present	320.9	-0.42	included	present	467.37	-0.37	included	present	1151.52	-0.16
218036_x_at	included	present	1253.33	-0.39	included	present	1618.51	0.35	included	present	1843.1	0.8	included	present	1478.42	0.06
218037_at	included	present	1195.68	0.15	included	present	2612.94	2.3	included	present	740.98	-0.53	included	present	741.94	-0.53
218038_at	included	present	339.43	-0.36	included	present	562.23	0.92	included	present	497.62	0.55	included	present	374	-0.16
218039_at	included	present	282.87	-0.08	included	present	2050.92	4.7	included	present	7000.98	18.09	included	present	3348.07	8.21
218040_at	included	present	575.21	-1.06	included	present	451.11	-1.44	included	present	1056.91	0.43	included	present	1081.71	0.51
218041_x_at	included	present	3691.37	-0.65	included	present	4136.61	-0.52	included	present	5678.94	-0.06	included	present	5480.44	-0.12
218042_at	included	present	1426.72	-0.32	included	present	1606.09	-0.03	included	present	1712.15	0.14	included	present	1281.63	-0.55
218043_s_at	included	present	322.78	0.67	included	present	208.44	-0.57	included	present	207.2	-0.58	included	present	237.05	-0.26
218044_x_at	excluded	absent	25.17	-0.61	excluded	absent	95.03	1.19	excluded	absent	70.28	0.56	excluded	absent	61.07	0.32
218045_x_at	included	present	26763.2	2.01	included	present	46574.66	4.77	included	present	30107.94	2.48	included	present	29343	2.37
218046_s_at	included	present	575.89	-0.02	included	present	572.45	-0.03	included	present	602.27	0.08	included	present	517.56	-0.24
218047_at	included	present	2478.05	-0.03	included	present	3633.37	1.08	included	present	1382.16	-1.08	included	present	3191.88	0.65
218048_at	included	present	691.51	-1.47	included	present	1672.9	1.54	included	present	817.77	-1.08	included	present	635.72	-1.64
218049_s_at	included	present	705.37	0.03	included	present	740.49	0.17	included	present	1360.68	2.75	included	present	784.81	0.36
218050_at	included	present	5737.36	0.01	included	present	8424.16	1.87	included	present	4911.17	-0.56	included	present	4012.9	-1.18
218051_s_at	included	present	372.8	2.64	included	present	337.87	2.24	included	present	649.78	5.85	included	present	141.57	-0.03
218052_s_at	excluded	absent	187.92	0.83	included	present	311.52	2.52	excluded	absent	182.71	0.75	included	present	206.69	1.08
218053_at	included	present	1662.75	-0.64	included	present	1271.24	-1.19	included	present	2893.4	1.08	included	present	3408.98	1.8
218054_s_at	included	present	249.66	-0.67	included	present	161.25	-1.23	included	present	281.15	-0.46	included	present	370.7	0.11
218055_s_at	included	present	2067.3	-0.12	included	present	3002.12	1.43	included	present	3128.18	1.63	included	present	2564.56	0.7
218056_at	included	present	1325.35	1.79	included	present	2687.01	6.37	included	present	2160.56	4.6	included	present	2544.52	5.89
218057_x_at	included	present	519.35	0.16	included	present	769.98	1.78	included	present	693.35	1.29	included	present	646.92	0.99
218058_at	excluded	absent	205.66	0.49	included	present	526.81	4.29	excluded	absent	92.15	-0.85	excluded	absent	148.71	-0.18
218059_at	included	present	1462.08	0.25	included	present	835.33	-1.27	included	present	1417.4	0.14	included	present	1064.68	-0.72
218060_s_at	included	present	341.76	1.25	excluded	absent	275.99	0.43	included	present	232.67	-0.1	included	present	220.85	-0.25
218061_at	included	present	2242.16	-0.84	included	present	10400.42	3.01	included	present	2903.93	-0.53	included	present	4024.56	0
218062_x_at	included	present	1871.89	0.71	included	present	3216.63	3.41	included	present	534.1	-1.98	included	present	1824.59	0.62
218063_s_at	excluded	absent	71.14	1.33	excluded	absent	10.61	-0.52	excluded	absent	17.18	-0.32	excluded	absent	7.09	-0.63
218064_s_at	excluded	absent	35.02	-1.56	excluded	absent	115.85	-0.47	excluded	absent	120.9	-0.4	included	present	251.34	1.35
218065_s_at	included	present	3289.94	-0.25	included	present	3143.37	-0.38	included	present	1828.6	-1.58	included	present	2649.34	-0.83
218066_at	included	present	805.31	0.31	included	present	1453.46	2.19	included	present	285.91	-1.21	included	present	666.67	-0.1
218067_s_at	included	present	5257.97	-1.27	included	present	8224.9	0.08	included	present	6465.77	-0.72	included	present	9512.17	0.66
218068_s_at	included	present	424.37	-0.9	included	present	996.03	0.69	included	present	631.44	-0.32	included	present	1094.66	0.96
218069_at	included	present	708.41	0.56	included	present	2404.84	6.01	included	present	2010.86	4.74	included	present	1252.24	2.3
218070_s_at	included	present	165.5	-0.72	included	present	434.92	1.73	included	absent	87.9	-1.42	included	present	149.18	-0.87
218071_s_at	included	present	967.47	0.73	included	present	687.69	-0.51	included	present	1077.28	1.22	included	present	970.48	0.74
218072_at	included	present	819.14	0.72	included	present	1755.85	4.74	included	present	589.36	-0.26	included	present	498.8	-0.65
218073_s_at	included	present	242.57	0.86	included	present	360.6	2.17	included	present	214.83	0.55	included	present	277.48	1.24
218074_at	included	present	508.04	-0.03	included	present	767.93	0.69	included	present	480.41	-0.11	included	present	297.5	-0.61
218075_at	included	present	458.93	0.15	included	present	906.11	2.65	included	present	612.73	1.01	included	present	436.55	0.02
218076_s_at	included	present	773.11	-0.3	included	present	1296.33	0.67	included	present	633.42	-0.56	included	present	469	-0.87
218077_s_at	included	present	612.4	-0.4	included	present	808.55	-0.02	included	present	730.79	-0.17	included	present	468.16	-0.68
218078_s_at	included	present	626.27	-0.36	included	present	1480.44	2.01	included	present	1017.46	0.73	included	present	630.71	-0.34
218079_s_at	included	present	1265.83	-1.52	included	present	2291.77	0.72	included	present	1346.51	-1.34	included	present	2216.85	0.56

218357_s_at	included	present	2776.83	-0.78	included	present	7616.38	1.21	included	present	4988.44	0.13	included	present	2344.17	-0.96	[illegible]
218358_at	included	present	1070.19	-0.33	included	present	871.74	-0.54	included	present	438.81	-0.98	included	present	2299.73	0.92	[illegible]
218359_at	excluded	absent	162.39	-0.14	included	present	272.28	0.51	included	present	158.99	-0.16	excluded	absent	175.8	-0.06	[illegible]
218360_at	included	present	3448.07	1.25	included	present	2968.32	0.45	included	present	2549.51	-0.25	included	present	2789.48	0.15	[illegible]
218361_at	included	present	1160.68	-0.82	included	present	623.77	-1.36	included	present	2184.51	0.22	included	present	4912.79	2.97	[illegible]
218362_s_at	included	present	143.13	-0.29	included	present	214.5	1.2	included	present	219.5	1.31	included	absent	106.65	-1.06	[illegible]
218363_at	included	present	274.87	-0.63	included	present	702.85	1.27	included	present	554.05	0.61	included	present	947.27	2.35	[illegible]
218364_at	included	present	989.51	-1.17	included	present	1550.08	-0.41	included	present	911.54	-1.28	included	present	1229.5	-0.85	[illegible]
218365_s_at	included	present	237.05	0.7	included	present	675.2	6.29	included	present	1111.06	11.85	included	present	427.03	3.13	[illegible]
218366_x_at	included	present	286.36	-0.93	included	present	350.97	-0.46	included	present	369.65	-0.32	included	present	629.21	1.57	[illegible]
218367_x_at	included	present	228.45	-0.72	included	present	507.45	1.62	included	present	499.09	1.55	included	present	400.01	0.72	[illegible]
218368_s_at	excluded	absent	242.72	2.24	excluded	absent	7.26	-0.9	excluded	absent	60.98	-0.18	excluded	absent	37.58	-0.49	[illegible]
218369_s_at	excluded	absent	5.49	-1.5	excluded	absent	6.47	-1.47	included	present	92.7	1.03	excluded	absent	100.34	1.25	[illegible]
218370_s_at	included	present	1364.69	-0.52	included	present	2366.54	0.63	included	present	1093.9	-0.83	included	present	2791.74	1.12	[illegible]
218371_s_at	included	present	501.69	-0.56	included	present	889.44	0.88	included	present	1135.48	1.8	included	present	390.17	-0.98	[illegible]
218372_at	included	present	453.39	-0.37	included	present	517.17	-0.11	included	present	282.35	-1.08	included	present	571.64	0.11	[illegible]
218373_at	included	present	1922.44	-0.27	included	present	1097.48	-1.16	included	present	827.81	-1.45	included	present	936.62	-1.33	[illegible]
218374_s_at	included	present	2105.2	0.4	included	present	2155.48	0.49	included	present	2519.72	1.14	included	present	2640.12	1.35	[illegible]
218375_at	included	present	1750.07	-1	included	present	3496.95	0.95	included	present	1928.63	-0.8	included	present	2049.27	-0.66	[illegible]
218376_s_at	included	present	443.74	-0.3	included	present	2754.36	5.68	included	present	696.41	0.35	included	absent	194.05	-0.95	[illegible]
218377_s_at	included	present	1511.72	0.61	included	present	1385.54	0.36	included	present	2231.45	2.04	included	present	834.63	-0.73	[illegible]
218378_s_at	included	present	589.13	-1.33	included	present	2679.78	9.39	included	present	721.64	-0.65	included	present	587.44	-1.34	[illegible]
218379_at	included	present	845.85	-0.67	included	present	1208.32	-0.12	included	present	1281.14	-0.01	included	present	1928.77	0.98	[illegible]
218380_at	included	present	2430.24	-0.05	included	present	6220.77	1.11	included	present	628.89	-0.61	included	present	268.3	-0.72	[illegible]
218381_s_at	included	present	312.63	-0.4	included	present	575.91	1.23	included	present	403.84	0.16	included	present	488.56	0.69	[illegible]
218382_s_at	excluded	absent	134.37	-0.96	excluded	absent	330.98	0.7	excluded	absent	174.76	-0.62	excluded	absent	122.96	-1.06	[illegible]
218383_at	included	present	715.21	-0.54	included	present	1032.51	0.26	included	present	1058.32	0.33	included	present	1398.8	1.18	[illegible]
218384_at	included	present	306.62	-0.13	included	present	423.91	0.19	included	present	292.65	-0.17	included	present	353.19	0	[illegible]
218385_at	included	present	476.8	-0.1	included	present	694.6	1.33	[illegible]	[illegible]	[illegible]	[illegible]	[illegible]	[illegible]	[illegible]	[illegible]	[illegible]
218386_x_at	included	present	1439.68	-0.05	included	present	1181.58	-0.72	[illegible]	[illegible]	[illegible]	[illegible]	[illegible]	[illegible]	[illegible]	[illegible]	[illegible]
[illegible]	[illegible]	[illegible]	[illegible]	[illegible]	[illegible]	[illegible]	[illegible]	[illegible]	[illegible]	[illegible]	[illegible]	[illegible]	[illegible]	[illegible]	[illegible]	[illegible]	[illegible]

218717_s_at	included	present	104.8	-0.45	excluded	absent	40.57	-0.94	excluded	absent	2.95	-1.23
218718_at	included	present	627.07	-0.12	included	present	191.76	-0.75	included	present	455.21	-0.37
218719_s_at	included	present	246.47	2.82	included	present	215.79	2.27	included	present	265.39	3.16
218720_x_at	included	present	397.84	0.65	included	present	803.55	2.37	included	present	229.46	-0.07
218721_s_at	included	present	943.5	-0.11	included	present	665.11	-1.03	included	present	1294.17	1.04
218722_s_at	included	present	197.39	-0.08	included	present	645.34	5.11	included	present	233.7	0.34
218723_s_at	included	present	326.79	-0.73	included	present	187.97	-0.88	included	absent	13.99	-1.07
218724_s_at	included	present	228.18	1.41	excluded	absent	5.62	-1.15	included	present	134.29	0.33
218725_at	included	present	187.7	-0.06	included	present	459.58	1.81	excluded	absent	105.31	-0.63
218726_at	excluded	absent	52.49	-0.44	included	present	271.08	1.79	included	present	251.95	1.59
218727_at	excluded	absent	151.47	1.2	included	present	256.47	2.83	excluded	absent	71.11	-0.06
218728_s_at	included	present	2191.3	0.14	included	present	1898.86	-0.11	included	present	6717.44	4.07
218729_at	included	present	334.54	-0.46	included	present	338.68	-0.45	included	present	191.99	-0.7
218730_s_at	included	present	3357.73	2.27	included	absent	55.8	-0.63	included	present	199.29	-0.5
218731_s_at	included	present	1095.08	4.73	excluded	absent	162.24	-0.73	excluded	absent	40.04	-1.44
218732_at	included	present	2516.44	1.97	included	present	5368.77	8.34	included	present	3593.98	4.38
218733_at	included	present	507.04	-0.43	included	present	403.8	-0.93	included	present	838.94	1.18
218734_at	excluded	absent	45.18	-0.64	excluded	absent	28.39	-0.96	excluded	absent	66.24	-0.24
218735_s_at	included	present	1192.4	0.15	included	present	2864.55	3.98	included	present	1549.62	0.97
218736_s_at	included	present	263.3	-0.51	included	absent	84.65	-0.71	included	absent	38.84	-0.76
218737_at	included	present	647.39	-0.5	included	present	1091.26	-0.01	included	present	1402.81	0.34
218738_s_at	included	present	781.68	-0.54	included	present	2447.27	1.7	included	present	2473.09	1.74
218739_at	included	present	362.03	-0.75	included	present	453.17	-0.51	included	present	248.96	-1.04
218740_s_at	included	present	3797.09	0.03	included	present	7185.42	2.68	included	present	12002.98	6.45
218741_at	excluded	absent	62.12	0.09	included	present	190.19	1.8	included	present	184.75	1.73
218742_at	excluded	absent	73.72	-0.33	excluded	absent	99.08	0.09	excluded	absent	22.38	-1.19
218743_at	excluded	absent	185.24	1.93	excluded	absent	135.86	1.09	excluded	absent	81.78	0.17
218744_s_at	included	absent	219.53	-0.54	included	absent	8.64	-0.74	included	absent	207.94	-0.55
218745_x_at	excluded	absent	56.54	0.06	excluded	absent	15.59	-0.85	excluded	absent	107.06	1.18
218746_at	excluded	absent	156.84	-0.45	excluded	absent	233.78	0.06	excluded	absent	169.84	-0.36
218747_s_at	included	present	318.6	-0.09	included	absent	137.09	-0.84	included	present	112.83	-0.94
218748_s_at	included	present	1365.1	1.37	included	present	1041.81	0.31	included	present	1499.41	1.81
218749_s_at	included	present	284.41	0.51	included	absent	85.16	-1.15	included	present	112.72	-0.92

219076_s_at	included	absent	142.9	-0.44	included	present	413.81	0	included	present	518.57	0.18	included	present	740.27	0.54	[illegible]
219077_s_at	included	present	193.22	-0.46	included	present	159.5	-0.73	included	absent	100.99	-1.18	included	present	229.44	-0.18	[illegible]
219078_at	included	present	219.06	0.08	included	present	240.74	0.29	included	present	232.82	0.21	included	present	224.58	0.13	[illegible]
219079_at	included	present	382.3	-0.37	included	present	1029.16	0.93	included	present	895.35	0.66	included	present	478.69	-0.17	[illegible]
219080_s_at	included	present	306.88	-0.59	included	present	648.58	0.37	included	present	923.94	1.13	included	present	614.58	0.27	[illegible]
219081_at	included	present	434.42	-1.07	included	present	670.06	-0.44	included	present	1314.15	1.26	included	present	1228.47	1.03	[illegible]
219082_at	excluded	absent	15.45	-0.66	excluded	absent	40.91	-0.13	excluded	absent	13.23	-0.71	excluded	absent	65.32	0.38	[illegible]
219083_at	included	present	737.23	-0.74	included	present	1631.03	2.01	included	present	1195.63	0.67	included	present	547.44	-1.32	[illegible]
219084_at	included	present	864.12	3.47	included	present	685.62	1.95	included	present	901.22	3.79	included	present	745.33	2.46	[illegible]
219085_s_at	excluded	absent	74.88	0.41	excluded	absent	11.1	-1.19	included	present	76.18	0.45	excluded	absent	50.58	-0.2	[illegible]
219086_at	included	present	88.61	-1.08	included	present	177.42	0.21	included	present	198.6	0.51	included	present	253.53	1.31	[illegible]
219087_at	included	present	589.82	-0.4	included	present	389.94	-0.47	included	present	895.63	-0.31	included	present	818.53	-0.33	[illegible]
219088_s_at	excluded	absent	7.59	-1.54	excluded	absent	129	0.85	excluded	absent	118.25	0.64	excluded	absent	136.86	1	[illegible]
219089_s_at	included	present	203.46	0.13	included	present	342.58	1.79	excluded	absent	205.42	0.15	included	present	396.95	2.44	[illegible]
219090_at	included	present	132.31	-0.74	included	absent	85.28	-0.81	included	absent	13.33	-0.9	included	present	74.96	-0.82	[illegible]
219091_s_at	included	present	185.07	-0.12	included	absent	79.18	-0.67	included	absent	67.69	-0.73	included	absent	69.7	-0.72	[illegible]
219092_s_at	included	present	623.58	-0.45	included	present	610.92	-0.47	included	present	884.94	-0.04	included	present	645.72	-0.42	[illegible]
219093_at	included	present	1436.79	0.41	included	absent	80.83	-1.26	included	present	129.96	-1.2	included	present	463.99	-0.79	[illegible]
219094_at	included	present	497.15	-0.88	included	present	214.67	-2.08	included	present	413.84	-1.23	included	present	529.04	-0.75	[illegible]
219095_at	excluded	absent	154.62	-0.6	included	present	246.62	0.39	excluded	absent	151.26	-0.64	excluded	absent	128.98	-0.88	[illegible]
219096_at	included	present	213.27	0.85	included	present	340.59	2.67	excluded	absent	21.36	-1.9	excluded	absent	111.68	-0.61	[illegible]
219097_x_at	included	present	2827.74	-0.38	included	present	2617.29	-0.59	included	present	3160.51	-0.05	included	present	3931.55	0.72	[illegible]
219098_at	included	present	323.61	-0.66	included	present	560.6	1.5	included	present	169.93	-2.07	included	present	187.56	-1.91	[illegible]
219099_at	included	present	301.82	0.65	included	present	478.82	2.35	included	present	194.66	-0.38	included	present	172	-0.6	[illegible]
219100_at	included	present	380.02	-0.29	included	present	511.29	0.4	included	present	430.68	-0.02	included	present	202.89	-1.23	[illegible]
219101_x_at	excluded	absent	30.76	-0.52	excluded	absent	7.57	-1.07	excluded	absent	16.65	-0.86	excluded	absent	47.58	-0.13	[illegible]
219102_at	included	present	240.44	1.17	excluded	absent	89.95	-0.21	excluded	absent	77.11	-0.32	excluded	absent	56.42	-0.51	[illegible]
219103_at	included	present	503.02	-0.24	included	absent	77.87	-1.35	included	present	133.4	-1.21	included	absent	16.45	-1.51	[illegible]
219104_at	included	present	1131.68	-0.41	included	present	1007.61	-0.5	included	present	386.44	-0.99	included	present	360.08	-1.01	[illegible]
219105_x_at	included	present	284.88	-0.8	included	present	2375.7	4.54	included	present	3521.71	7.47	included	present	1104.56	1.29	[illegible]
219106_s_at	included	present	133.83	-0.16	included	absent	74.99	-0.17	included	present	88.1	-0.17	included	present	212.25	-0.16	[illegible]
219107_at	excluded	absent	7.64	-1.1	excluded	absent	16.62	-1	excluded	absent	56.46	-0.57	excluded	absent	41.26	-0.73	[illegible]
219108_x_at	included	present	925.46	-0.81	included	present	3817.16	5.76	included	present	1108.09	-0.39	included	present	936.7	-0.78	[illegible]
219109_at	included	present	805.77	-1.27	included	present	1205.8	-0.56	included	present	1001.23	-0.92	included	present	1407.59	-0.21	[illegible]
[illegible]																	

219435_at	included	absent	161.38	-0.39	included	present	241.23	0.36	included	present	112.44	-0.85	included	present	177.59	-0.24	included	absent	193.62
219436_s_at	included	present	756.9	0.21	included	present	170.12	-0.62	included	absent	83.28	-0.74	included	absent	77.04	-0.75	included	present	375.43
219437_s_at	included	present	1147.33	0.76	included	present	1820.67	2.84	included	absent	96.5	-2.5	included	present	541.11	-1.12	included	present	1387.64
219438_at	excluded	absent	67.8	0	included	present	565.29	7.55	excluded	absent	98.68	0.46	excluded	absent	40.96	-0.41	excluded	absent	149.8
219439_at	included	present	700.72	-0.94	included	present	943.66	-0.54	included	present	632.06	-1.06	included	present	2717.22	2.43	included	present	810.08
219440_at	included	absent	129.09	-1.04	included	present	364.44	-0.73	included	present	706.47	-0.27	included	present	582.07	-0.44	included	present	354.14
219441_s_at	included	present	188.87	0	excluded	absent	68.8	-1.33	excluded	absent	140.63	-0.53	excluded	absent	37.94	-1.67	excluded	absent	18.84
219442_at	excluded	absent	115.29	-0.46	excluded	absent	96.83	-0.68	excluded	absent	82.14	-0.85	excluded	absent	114.86	-0.47	excluded	absent	6.37
219443_at	included	present	289.64	-0.71	included	present	236.61	-1.02	included	present	186.8	-1.31	included	present	594.97	1.09	included	present	278.21
219444_at	included	present	105.43	-0.15	included	present	143.97	0.81	included	present	71.73	-0.98	excluded	absent	100.58	-0.27	included	present	221.22
219445_at	included	present	281.65	-1.11	included	present	492.96	0.76	included	present	227.71	-1.59	included	present	312.9	-0.83	included	present	214.22
219446_at	included	present	240.41	0.16	included	present	288.58	0.59	included	present	296.93	0.66	included	present	247.68	0.22	included	present	356.67
219447_s_at	included	present	108.74	-0.75	included	present	234.35	0.58	included	absent	84.3	-1.01	included	present	147.51	-0.34	included	absent	150.79
219448_at	included	present	599.38	1.73	included	present	230.83	-0.92	included	present	465.36	0.77	included	present	720.94	2.61	included	present	311.83
219449_s_at	included	present	1421.3	-0.25	included	present	767.49	-1.35	included	present	1579.29	0.02	included	present	2722.83	1.94	included	present	1366.6
219450_at	included	present	169.28	-0.48	included	absent	0.47	-0.68	included	present	220.17	-0.42	included	absent	13.83	-0.67	included	absent	4.39
219451_at	included	present	246.16	-0.14	included	present	367.4	0.78	included	absent	132.86	-1.01	included	present	218.84	-0.35	included	present	464.66
219452_at	included	present	238.88	-0.15	included	present	252.4	-0.13	included	absent	74.7	-0.4	included	absent	67.13	-0.42	included	present	256.23
219453_at	excluded	absent	15.49	-1.23	excluded	absent	9.23	-1.36	excluded	absent	6.54	-1.42	included	present	125.65	1.05	excluded	absent	89.6
219454_at	excluded	absent	3.67	-0.51	excluded	absent	71.08	-0.14	excluded	absent	28.5	-0.37	excluded	absent	59.62	-0.2	excluded	absent	80.87
219455_at	included	present	222.93	-0.71	included	present	390.46	-0.31	included	absent	56.14	-1.12	included	present	279.7	-0.58	included	present	200.07
219456_s_at	included	present	108.53	0.8	excluded	absent	4	-1.25	excluded	absent	2.98	-1.27	excluded	absent	65.76	-0.04	excluded	absent	22.83
219457_s_at	excluded	absent	15.52	-0.71	excluded	absent	11.63	-0.81	excluded	absent	8.7	-0.88	excluded	absent	13.12	-0.77	excluded	absent	13.64
219458_s_at	included	present	611.48	1.1	included	present	306.63	-0.36	included	present	431.66	0.23	included	present	401.75	0.09	included	present	846.12
219459_at	included	present	253.22	-0.75	included	present	472.4	0.52	included	present	361.53	-0.12	included	present	446.44	0.37	included	present	394.36
219460_s_at	included	present	1280.52	0.15	included	present	1764.24	0.88	included	present	1081.53	-0.16	included	present	825.77	-0.55	included	present	1313.05
219461_at	included	absent	38.57	-0.79	included	present	323.81	-0.34	included	absent	3.77	-0.84	included	present	464.43	-0.12	included	absent	71.27
219462_at	included	present	206	0.87	excluded	absent	26.62	-1.16	excluded	absent	7.98	-1.37	included	present	125.47	-0.04	excluded	absent	22.89
219463_at	included	present	824.13	-0.36	included	present	310.41	-0.48	included	present	248.38	-0.49	included	present	223.07	-0.5	included	present	355.87
219464_at	excluded	absent	3.94	-0.91	excluded	absent	39.17	-0.57	excluded	absent	72.93	-0.23	included	present	67.61	-0.29	excluded	absent	2.98
219465_at	excluded	absent	201.64	-0.13	excluded	absent	2.08	-0.15	included	present	746.1	-0.06	excluded	absent	115.92	-0.14	excluded	absent	100.02
219466_s_at	excluded	absent	105.38	-0.15	excluded	absent	10.08	-0.15	included	present	2836.7	0.03	included	present	658.22	-0.11	excluded	absent	38.23
219467_at	included	present	364.43	-1.04	included	present	238.77	-1.66	included	present	562.45	-0.08	included	present	600.38	0.11	included	present	616.38
219468_s_at	included	present	226.76	0.79	excluded	absent	179.3	0.21	excluded	absent	41.04	-1.5	included	present	298.57	1.68	excluded	absent	77.72
219469_at	included	present	211.5	-0.65	included	present	154.52	-0.88	included	present	169.56	-0.82	included	absent	79.22	-1.19	included	absent	140.8
219470_x_at	included	present	214.83	-0.65	included	present	130.17	-1.54	included	present	451.43	1.82	included	present	396.47	1.24	included	present	330.31
219471_at	included	present	222.46	-0.29	included	absent	124.17	-0.6	included	present	97.52	-0.68	included	absent	162.86	-0.48	included	present	288.74
219472_at	included	present	507.24	0.92	included	present	553.71	1.24	included	present	413.11	0.28	included	present	240.48	-0.9	included	absent	128.02
219473_at	included	present	398.89	1.4	included	present	175.07	-1.78	included	present	366.06	0.94	included	present	318.7	0.27	included	present	390.07
219474_at	excluded	absent	77.31	-0.35	excluded	absent	44.76	-0.45	excluded	absent	32.34	-0.49	included	present	198.86	0.01	excluded	absent	6.12
219475_at	excluded	absent	88.09	0.18	excluded	absent	19.73	-0.68	excluded	absent	80.42	0.08	excluded	absent	4.55	-0.87	excluded	absent	13.5
219476_at	excluded	absent	69.25	-0.38	excluded	absent	19.77	-0.42	included	present	173.63	-0.3	excluded	absent	121.03	-0.34	excluded	absent	50.62
219477_s_at	included	present	889.06	0.67	included	present	2591.34	6.38	included	present	1037.99	1.17	included	present	402.21	-0.96	included	present	913.84
219478_at	included	present	2518.8	3.25	included	absent	42.03	-0.73	included	present	211.32	-0.45	included	present	115.99	-0.61	included	absent	156.31
219479_at	included	present	374.93	1.36	included	present	712.97	3.91	included	present	759.52	4.26	included	present	284.15	0.67	included	present	415.55
219480_at	included	present	109.94	1.61	excluded	absent	43.24	-0.17	excluded	absent	87.49	1.01	excluded	absent	2.8	-1.25	excluded	absent	78.32
219481_at	included	present	439.88	0.72	included	present	479.51	1.01	included	present	709.88	2.65	included	present	906.7	4.06	included	absent	148.4
219482_at	included	present	612.27	-0.73	included	present	983.4	0.7	included	present	480.82	-1.23	included	present	736.46	-0.25	included	present	718.29
219483_s_at	excluded	absent	191.92	-0.08	included	present	371.84	0.91	excluded	absent	104.07	-0.56	excluded	absent	5.44	-1.1	included	present	307.54
219484_at	included	present	610.12	-0.18	included	present	234.37	-0.58	included	present	483.27	-0.32	included	present	775.65	-0.01	included	present	798.86
219485_s_at	included	present	2028.83	0.16	included	present	1361.95	-0.9	included	present	1451.52	-0.76	included	present	1676.71	-0.4	included	present	1662.3
219486_at	excluded	absent	123.79	-0.13	excluded	absent	148.87	0.23	excluded	absent	45.95	-1.24	excluded	absent	131.06	-0.02	excluded	absent	95.3
219487_at	included	present	263.61	0.33	included	present	236.08	0.08	included	present	167.39	-0.55	included	present	706.48	4.39	included	present	211.89
219488_at	excluded	absent	78.56	-0.36	excluded	absent	207.04	1.16	excluded	absent	8.06	-1.2	excluded	absent	12.21	-1.15	excluded	absent	112.13
[illegible]	[illegible]	[illegible]	[illegible]	[illegible]	[illegible]	[illegible]	[illegible]	[illegible]	[illegible]	[illegible]	[illegible]	[illegible]	[illegible]	[illegible]	[illegible]	[illegible]	[illegible]	[illegible]	[illegible]

219794_at	included	present	148.41	1.03	excluded	absent	4.93	-1.49	excluded	absent	89.88
219795_at	excluded	absent	2.29	-0.34	excluded	absent	6.52	-0.33	excluded	absent	2.13
219796_s_at	excluded	absent	13.35	-0.26	excluded	absent	5.23	-0.26	excluded	absent	51.51
219797_at	included	present	882.65	-0.13	included	present	685.68	-0.44	included	present	383.19
219798_s_at	included	present	304.36	0.02	included	present	282.92	-0.13	included	present	235.38
219799_s_at	excluded	absent	145.38	-0.11	excluded	absent	87.42	-0.2	excluded	absent	7.22
219800_s_at	excluded	absent	40.42	-0.61	excluded	absent	37	-0.69	excluded	absent	76.87
219801_at	included	present	206.2	0.37	included	absent	15.65	-2.36	included	present	307.62
219802_at	included	present	805.69	0.26	included	present	602.56	-0.52	included	present	572.81
219803_at	excluded	absent	53.06	-0.19	excluded	absent	72.03	-0.17	included	present	205.45
219804_at	excluded	absent	4.5	-0.2	excluded	absent	4.26	-0.2	excluded	absent	5.68
219805_at	included	absent	200.08	-0.47	included	present	242	0.12	included	absent	179.2
219806_s_at	included	present	370.16	-0.54	included	present	388.23	-0.52	included	present	241.24
219807_x_at	included	present	318.06	0.41	excluded	absent	251.15	-0.12	excluded	absent	191.33
219808_at	excluded	absent	8.18	-0.8	excluded	absent	13.62	-0.63	excluded	absent	44.89
219809_at	included	present	199.61	1.2	included	present	194.76	1.1	included	absent	114.08
219810_at	included	present	1309.26	0.93	included	present	1079.22	0.25	included	present	1221.21
219811_at	included	present	182.21	0.42	included	present	184.3	0.45	included	present	156.16
219812_at	excluded	absent	59.92	-0.89	included	present	162.56	0.01	excluded	absent	82.97
219813_at	included	present	345.85	-0.02	included	absent	47.7	-1.76	included	present	257.27
219814_at	included	present	89.05	-0.32	excluded	absent	74.57	-0.38	included	present	114.53
219815_at	included	present	1107.25	1.76	included	present	910.7	1.22	included	present	564.54
219816_s_at	included	present	609.28	-1.19	included	present	1353.39	1.42	included	present	777.05
219817_at	included	present	1169.67	-0.3	included	present	2721.26	3.14	included	present	2266.68
219818_s_at	included	present	403.36	-0.09	included	present	437.59	0.23	included	present	381.93
219819_s_at	included	present	1541.08	-0.11	included	present	952.93	-1.06	included	present	1170.61
219820_at	excluded	absent	2.82	-0.31	excluded	absent	63.9	-0.23	excluded	absent	43.65
219821_s_at	included	present	345.07	-0.78	included	present	1096.88	-0.14	included	present	240.02
219822_at	included	present	455.77	-0.95	included	present	1042.95	0.16	included	present	650.32
219823_at	excluded	absent	57.39	-0.67	excluded	absent	104.97	0.19	excluded	absent	11.86
219824_at	included	present	133.25	-0.11	excluded	absent	17.12	-0.5	included	present	119.62
219825_at	included	present	852.16	0.15	included	absent	92.99	-0.6	included	absent	3.51
219826_at	included	present	227.92	-1.8	included	present	225.07	-1.83	included	present	441.71
219827_at	excluded	absent	13.09	-0.21	excluded	absent	10.44	-0.21	excluded	absent	8.04

[illegible]

220153_at	included	absent	75.78	-0.8	included	present	54.16	-1.04	included	present	122.9	-0.27	included	present	79.35	-0.76	included	present	197.47
220154_at	included	present	2599.04	-0.47	included	present	8187.27	1.42	included	present	3812.74	-0.06	included	absent	198.96	-1.28	included	present	4884.75
220155_s_at	included	present	286.1	-0.64	included	present	1002.08	4.88	included	present	437.52	0.53	included	present	383.91	0.11	included	present	291.98
220156_at	excluded	absent	12.94	-0.73	excluded	absent	47.7	-0.38	excluded	absent	60.71	-0.25	excluded	absent	19.68	-0.66	excluded	absent	116.18
220157_x_at	excluded	absent	103.47	-0.63	included	present	525.66	3.71	included	present	382.21	2.23	included	present	355.69	1.96	included	present	807.48
220158_at	excluded	absent	73.98	2.11	excluded	absent	37.69	0.73	excluded	absent	59.04	1.55	excluded	absent	3.43	-0.57	excluded	absent	2.89
220159_at	included	present	321.02	1.25	included	absent	86.48	-1.34	included	present	112.64	-1.06	included	present	161.91	-0.51	included	absent	166.89
220160_s_at	excluded	absent	146.28	0.05	included	present	357.73	2.73	included	present	192.93	0.64	excluded	absent	130.23	-0.15	excluded	absent	3.95
220161_s_at	included	present	1327.88	0.09	included	present	306.84	-0.34	included	present	876.12	-0.1	included	present	1883.09	0.31	included	present	470.39
220162_s_at	included	present	141.59	0.75	excluded	absent	88.89	-0.2	excluded	absent	12.17	-1.58	excluded	absent	2.52	-1.75	excluded	absent	27.27
220163_s_at	excluded	absent	49.1	-0.38	excluded	absent	28.16	-0.69	excluded	absent	20.26	-0.81	excluded	absent	56.96	-0.27	excluded	absent	18.55
220164_s_at	excluded	absent	31.69	0	excluded	absent	48.85	0.18	excluded	absent	10.43	-0.24	excluded	absent	8.71	-0.25	excluded	absent	4.92
220165_at	included	present	1413.38	2.35	included	present	452.19	-0.84	included	present	881.04	0.58	included	present	2080.3	4.57	included	present	714.84
220166_at	excluded	absent	100.4	-0.51	excluded	absent	30.3	-0.85	excluded	absent	112.45	-0.45	excluded	absent	14.22	-0.93	excluded	absent	131.35
220167_s_at	included	present	511.71	-0.02	included	present	6418.88	3.15	included	present	1494.37	0.51	excluded	absent	31.28	-0.28	included	present	1048.7
220168_at	excluded	absent	82.67	-0.3	included	present	92.77	-0.27	excluded	absent	68.19	-0.34	excluded	absent	98.27	-0.26	excluded	absent	54.34
220169_at	included	present	87.31	0.14	excluded	absent	79.63	0.05	excluded	absent	60.97	-0.16	excluded	absent	12.44	-0.72	excluded	absent	89.68
220170_at	included	present	108.57	-0.57	included	absent	6.49	-0.89	included	absent	38.04	-0.79	included	absent	16.99	-0.86	included	absent	39.05
220171_x_at	included	present	268.89	-1.03	included	present	421.77	-0.05	included	present	614.59	1.19	included	present	119.09	-2	included	present	444.97
220172_at	included	present	262.33	0	included	present	118.53	-1.27	included	present	662.24	3.55	included	present	338.74	0.68	included	present	247.95
220173_at	included	absent	43.69	-0.52	included	absent	62.71	-0.46	included	absent	51.17	-0.5	included	present	181.59	-0.11	included	absent	88.29
220174_at	excluded	absent	50.51	-0.14	excluded	absent	46.51	-0.21	excluded	absent	87.24	0.51	excluded	absent	26.32	-0.57	excluded	absent	126.3
220175_s_at	included	present	2581.53	-1.34	included	present	5278.6	0.68	included	present	6890.96	1.89	included	present	3120.01	-0.94	included	present	5074.44
220176_at	included	present	205.56	-0.24	included	absent	23.77	-1.99	included	absent	211.38	-0.19	included	present	151.71	-0.76	included	absent	208.2
220177_s_at	excluded	absent	96.94	-0.17	excluded	absent	34.88	-0.41	excluded	absent	117.38	-0.09	excluded	present	205.65	0.24	excluded	absent	42.27
220178_at	included	present	534.12	1.55	excluded	absent	260.79	-0.2	included	present	396.37	0.67	included	present	395.04	0.66	excluded	absent	148.1
220179_at	excluded	absent	64.87	-0.22	included	present	11992.89	12.03	excluded	absent	42.46	-0.24	excluded	absent	3.3	-0.28	excluded	absent	119.56
220180_at	excluded	absent	40.36	-0.49	excluded	absent	57.17	-0.35	excluded	absent	2.11	-0.83	excluded	absent	6.33	-0.79	excluded	absent	7.09
220181_x_at	included	present	1553.6	-0.79	included	present	2342.05	0.53	included	present	2437.49	0.69	included	present	1643.3	-0.64	included	present	1993.97
220182_at	included	present	141.18	-0.45	included	present	89.46	-0.91	included	absent	75.04	-1.04	included	present	78.24	-1.01	included	absent	185.88
220183_s_at	included	present	2700.63	1.13	included	present	1436.28	-0.36	included	present	967.38	-0.91	included	present	690.31	-1.24	included	present	1122.71
220184_at	included	present	197.85	-0.55	included	absent	126.15	-0.59	included	present	284.57	-0.5	included	present	339.46	-0.48	included	present	296.4
220185_at	excluded	absent	6.84	-0.67	excluded	absent	25.32	-0.35	excluded	absent	10.07	-0.61	excluded	absent	120.21	1.27	excluded	absent	143.09
220186_s_at	excluded	absent	72.9	-0.22	excluded	absent	108.99	-0.19	excluded	absent	44.03	-0.25	excluded	absent	30.49	-0.26	excluded	absent	181.1

[illegible]

220512_at	included	present	287.76	1.44	excluded	absent	44.89	-0.75	included	present	263.8
220513_at	excluded	absent	3.22	-0.62	included	present	868.04	16.45	excluded	absent	2.31
220514_at	included	present	152.53	-0.53	included	present	158.99	-0.5	included	present	454.24
220515_at	excluded	absent	103.54	0.9	excluded	absent	66.54	0.26	excluded	absent	10.55
220516_at	excluded	absent	139.25	1.11	excluded	absent	455.51	5.85	excluded	absent	31.85
220517_at	included	present	77.99	-1.4	included	absent	61.07	-1.56	included	present	215.92
220518_at	included	present	138.34	0.02	excluded	absent	64.63	-0.65	excluded	absent	23.99
220519_s_at	excluded	absent	4.58	-0.58	excluded	absent	4.24	-0.59	excluded	absent	73.71
220520_s_at	excluded	absent	29.44	-0.65	excluded	absent	73.67	-0.31	included	present	131.94
220521_s_at	included	present	153.23	0.34	excluded	absent	112.86	-0.34	excluded	absent	12.78
220522_at	excluded	absent	4.95	-1.01	excluded	absent	48.5	-0.55	excluded	absent	1.85
220523_at	excluded	absent	3.74	-1.17	excluded	absent	0.81	-1.24	excluded	absent	1.12
220524_at	excluded	absent	36.37	-0.63	excluded	absent	3.84	-1.16	excluded	absent	58.36
220525_s_at	included	present	1776.39	-0.57	included	present	2694.68	0.34	included	present	1467.09
220526_s_at	included	present	2427.69	-1.03	included	present	3840.9	0.18	included	present	1783.88
220527_at	excluded	absent	38.93	-0.85	excluded	absent	82.57	-0.04	excluded	absent	82.79
220528_at	excluded	absent	25.66	-0.1	excluded	absent	13.9	-0.27	excluded	absent	2.22
220529_at	excluded	absent	75.4	-1.13	excluded	absent	48.78	-1.43	included	present	239.76
220530_at	included	present	78.69	-0.28	excluded	absent	25.26	-0.47	excluded	absent	66.29
220531_at	excluded	absent	74.87	1.86	excluded	absent	5.88	-0.55	excluded	absent	38.89
220532_s_at	included	present	1282.18	0.75	included	present	666.07	0.11	included	present	1055.95
220533_at	included	present	77.52	0.67	included	present	151.82	2.75	included	present	154.57
220534_at	excluded	absent	2.26	-0.8	included	present	122.83	1.36	excluded	absent	54.15
220535_at	excluded	absent	72.21	-0.33	excluded	absent	84.65	-0.19	excluded	absent	4.31
220536_at	excluded	absent	51.73	-0.68	excluded	absent	24.04	-1.07	excluded	absent	137.88
220537_at	excluded	absent	58.44	2.33	excluded	absent	2.25	-0.66	excluded	absent	0.93
220538_at	excluded	absent	120.42	0.39	excluded	absent	91.81	-0.01	excluded	absent	22.06
220539_at	excluded	absent	10.06	-0.72	excluded	absent	4.05	-0.82	excluded	absent	3.27
220540_at	excluded	absent	1.88	-0.77	excluded	absent	2.27	-0.76	excluded	absent	41.57
220541_at	excluded	absent	1.77	-0.28	excluded	absent	28.12	-0.16	excluded	absent	32.16
220542_s_at	excluded	absent	1.99	-0.16	excluded	absent	7.56	-0.16	excluded	absent	7.59
220543_at	excluded	absent	39.06	-0.55	excluded	absent	4.97	-0.79	excluded	absent	33.89
220544_at	excluded	absent	81.07	0.36	excluded	absent	149.69	1.5	excluded	absent	83.8
220545_s_at	excluded	absent	4.04	-0.39	excluded	absent	5.64	-0.38	excluded	absent	9.12
[illegible]											

220871_at	excluded	absent	3.28	-1.33	excluded	absent	3.51	-1.33	excluded	absent	3.43	-1.33	included	present	143.11	1.03	excluded	absent	92.42	0.17	excluded	absent	63.03	-0.32	excluded	absent	64.69	-0.29	excluded	absent	62.45	-0.33	included	present	158.17	1.28	included	present	96.14	0.24	included	present	213.3	2.21	excluded	absent	71.77	-0.17	included	present	125.52	0.73	excluded	absent	9.22	-1.23	included	present	90.35	0.14
220872_at	included	present	89.67	-0.26	included	present	314.83	1.61	included	present	108.72	-0.1	excluded	absent	57.02	-0.53	included	present	410.13	2.4	excluded	absent	87.92	-0.27	included	present	802.74	5.65	included	present	159.43	0.32	included	present	1291.32	9.7	included	present	1226.77	9.16	included	present	615.53	4.1	included	present	169.59	0.4	included	present	651.8	4.4	included	present	236.01	0.95	included	present	773.43	5.41
220873_at	excluded	absent	62.5	-0.61	excluded	absent	107.25	-0.01	excluded	absent	55.34	-0.71	excluded	absent	5.76	-1.38	excluded	absent	3.24	-1.41	excluded	absent	4.69	-1.39	excluded	absent	3.31	-1.41	included	present	52.63	-0.75	excluded	absent	7.93	-1.35	excluded	absent	30	-1.05	excluded	absent	13.21	-1.28	excluded	absent	8.7	-1.34	excluded	absent	5.69	-1.38	excluded	absent	2.9	-1.42	excluded	absent	3.04	-1.42
220874_at	included	present	489.75	0.7	included	present	187.01	-1.23	included	present	470.63	0.58	included	present	379.49	-0.01	included	present	404.82	0.16	included	present	388.62	0.05	included	present	212.4	-1.07	included	present	162.42	-1.39	included	present	456.98	0.49	included	present	191.08	-1.21	included	present	308.38	-0.46	included	present	277.45	-0.66	included	present	345.49	-0.22	included	present	259.85	-0.77	included	present	165.67	-1.37
220875_at	excluded	absent	71.89	-0.39	excluded	absent	55.2	-0.72	excluded	absent	41.93	-0.98	excluded	absent	36.45	-1.08	excluded	absent	119.52	0.53	excluded	absent	75.42	-0.32	excluded	absent	48.02	-0.86	included	present	65.58	-0.52	included	present	65.76	-0.51	excluded	absent	49.88	-0.82	included	present	120.4	0.55	excluded	absent	74.11	-0.35	excluded	absent	38.53	-1.04	excluded	absent	24.01	-1.32	excluded	absent	22.02	-1.36
220876_at	included	present	1241.44	3.87	excluded	absent	4.51	-0.35	excluded	absent	21.57	-0.29	excluded	absent	38.25	-0.23	excluded	absent	73.54	-0.11	excluded	absent	3.04	-0.35	excluded	absent	33.91	-0.25	included	present	208.82	0.35	excluded	absent	80.42	-0.09	excluded	absent	95.72	-0.04	excluded	absent	6.3	-0.34	excluded	absent	3.98	-0.35	excluded	absent	137.87	0.11	included	present	232.59	0.43	excluded	absent	1.35	-0.36
220877_at	included	present	294.02	0.44	included	absent	9.86	-2.49	included	absent	103.57	-1.53	included	present	305.92	0.56	included	present	380.98	1.34	included	present	216.83	-0.36	included	absent	81.36	-1.76	included	absent	49.22	-2.09	included	present	202.32	-0.51	included	present	263.67	0.13	included	present	349.82	1.02	included	absent	66.33	-1.91	included	present	173.34	-0.81	included	absent	51.1	-2.07	included	absent	53.17	-2.05
220878_at	excluded	absent	37.85	0.04	excluded	absent	39.64	0.07	excluded	absent	38.34	0.05	excluded	absent	32.76	-0.05	excluded	absent	130.81	1.63	excluded	absent	31.49	-0.07	excluded	absent	55.26	0.34	excluded	absent	27	-0.15	excluded	absent	1.67	-0.58	excluded	absent	25.25	-0.18	excluded	absent	27.17	-0.15	excluded	absent	2.73	-0.56	excluded	absent	1.29	-0.59	excluded	absent	6.37	-0.5	excluded	absent	2.71	-0.56
220879_at	excluded	absent	60.15	-0.33	excluded	absent	7.49	-0.61	excluded	absent	86.64	-0.19	excluded	absent	13.36	-0.58	excluded	absent	179.28	0.3	excluded	absent	143.62	0.11	excluded	absent	36.54	-0.46	excluded	absent	56.33	-0.35	included	present	124.67	0.01	excluded	absent	6.6	-0.62	excluded	absent	88.65	-0.18	excluded	absent	74.54	-0.25	excluded	absent	54.32	-0.36	excluded	absent	63.41	-0.31	excluded	absent	26.53	-0.51
220880_at	excluded	absent	3.1	-0.85	excluded	absent	5.24	-0.78	excluded	absent	80.55	1.55	excluded	absent	1.94	-0.89	excluded	absent	2.61	-0.87	excluded	absent	52.32	0.67	excluded	absent	32.06	0.04	excluded	absent	1.52	-0.9	excluded	absent	6.22	-0.75	excluded	absent	4.57	-0.81	excluded	absent	3.73	-0.83	excluded	absent	4.16	-0.82	excluded	absent	29.05	-0.05	excluded	absent	1.33	-0.91	excluded	absent	2.18	-0.88
220881_at	excluded	absent	35.96	-0.31	excluded	absent	2.2	-0.98	excluded	absent	10.76	-0.81	excluded	absent	110.87	1.16	excluded	absent	38.6	-0.26	excluded	absent	54.82	0.06	excluded	absent	5.08	-0.92	excluded	absent	4.65	-0.93	excluded	absent	3.5	-0.95	excluded	absent	14.38	-0.74	excluded	absent	53.57	0.03	excluded	absent	27.8	-0.48	included	present	66.79	0.29	excluded	absent	0.85	-1.01	excluded	absent	0.6	-1.01
220882_at	excluded	absent	3.11	-0.66	excluded	absent	85.32	0.68	excluded	absent	61.28	0.29	excluded	absent	21.8	-0.36	excluded	absent	75.22	0.52	excluded	absent	4.35	-0.64	excluded	absent	5.6	-0.62	excluded	absent	2.58	-0.67	excluded	absent	8.06	-0.58	excluded	absent	7.72	-0.59	excluded	absent	10	-0.55	excluded	absent	13.45	-0.49	excluded	absent	12.96	-0.5	excluded	absent	1.87	-0.68	excluded	absent	2.37	-0.67
220883_at	included	present	221.61	-0.97	included	present	96.27	-1.5	included	present	337.65	-0.48	included	present	337.1	-0.49	included	present	182.35	-1.13	included	present	234.47	-0.92	included	present	342.76	-0.46	included	absent	55.29	-1.67	included	present	351.63	-0.43	included	present	163.85	-1.21	included	present	223.47	-0.96	included	present	170.67	-1.18	included	present	113.13	-1.42	included	absent	52.8	-1.68	included	present	121.76	-1.39
220884_at	included	present	562.35	-0.35	included	present	198.69	-1.48	included	present	424.95	-0.78	included	present	526.39	-0.46	included	present	413.43	-0.81	included	present	257.06	-1.3	included	present	628.05	-0.15	included	present	140.11	-1.66	included	present	513.97	-0.5	included	present	363.8	-0.97	included	present	670.38	-0.02	included	present	513.25	-0.5	included	present	237.16	-1.36	included	present	227.29	-1.39	included	present	192.22	-1.5
220885_s_at	included	present	826.04	-0.12	included	present	3412.91	3.25	included	present	5759.89	6.3	included	present	609.43	-0.4	included	present	5204.92	5.58	included	present	237.93	-0.89	included	present	964.78	0.06	included	present	944.18	0.03	included	present	626.57	-0.38	included	present	486.77	-0.56	included	present	1720.79	1.04	included	present	2276.16	1.77	included	present	3679.83	3.59	included	present	2695.33	2.31	included	present	1605.99	0.89
220886_at	excluded	absent	108.81	2.72	excluded	absent	31.67	0.18	excluded	absent	2.04	-0.79	excluded	absent	3.86	-0.73	excluded	absent	5.09	-0.69	excluded	absent	5.18	-0.69	included	present	81.83	1.83	excluded	absent	16.81	-0.31	included	present	104.77	2.59	excluded	absent	2.47	-0.78	excluded	absent	8.69	-0.57	excluded	absent	47.32	0.7	excluded	absent	12.27	-0.46	excluded	absent	53.6	0.9	excluded	absent	47.15	0.69
220887_at	excluded	absent	1.84	-0.69	included	present	170.62	2.07	excluded	absent	2.45	-0.68	excluded	absent	5.14	-0.64	excluded	absent	139.3	1.56	excluded	absent	1.47	-0.7	excluded	absent	143.6	1.63	excluded	absent	10.36	-0.55	included	present	55.71	0.19	excluded	absent	19.54	-0.4	included	present	131	1.42	excluded	absent	3.25	-0.67	excluded	absent	121.35	1.27	excluded	absent	122.43	1.28	included	present	188.78	2.37
220888_s_at	excluded	absent	60.74	0.87	excluded	absent	2.04	-1.05	excluded	absent	40.12	0.19	excluded	absent	6.87	-0.89	excluded	absent	21.21	-0.42	excluded	absent	4.14	-0.98	excluded	absent	55.13	0.69	excluded	absent	3.21	-1.01	excluded	absent	46.49	0.4	excluded	absent	24.75	-0.31	excluded	absent	56.85	0.74	excluded	absent	67.67	1.1	excluded	absent	5.56	-0.94	excluded	absent	1.69	-1.06	excluded	absent	27.98	-0.2
220889_s_at	included	present	112.59	-0.08	excluded	absent	41.62	-0.51	excluded	absent	49.14	-0.46	excluded	absent	2.69	-0.75	excluded	absent	6.22	-0.73	excluded	absent	56.89	-0.42	included	present	182.57	0.35	excluded	absent	23.57	-0.62	included	present	72.1	-0.32	included	present	1882.02	10.76	excluded	absent	107.37	-0.11	excluded	absent	158.96	0.21	excluded	absent	95.65	-0.18	included	present	479.11	2.17	excluded	absent	67.92	-0.35
220890_s_at	included	present	1789.02	-1.43	included	present	4511.07	0.98	included	present	2397.32	-0.89	included	present	2108.69	-1.15	included	present	3630.81	0.2	included	present	2003.98	-1.24	included	present	2309.85	-0.97	included	present	5285.68	1.66	included	present	2637.33	-0.68	included	present	2058.95	-1.19	included	present	4207.1	0.71	included	present	3876.25	0.42	included	present	4428.93	0.91	included	present	6922.96	3.11	included	present	2203.32	-1.06
220891_at	included	present	129.35	0.07	excluded	absent	91.21	-0.77	included	present	149.38	0.51	included	present	123.31	-0.07	included	present	122.19	-0.09	included	present	117.44	-0.2	included	present	218.16	2.03	included	present	226.75	2.21	included	present	175.14	1.08	included	present	167.25	0.9	included	present	402.11	6.08	included	present	180.15	1.19	included	present	126.88	0.01	included	present	202.04	1.67	included	present	68.62	-1.27
220892_s_at	included	present	724.83	-0.26	included	present	237.52	-0.72	included	present	1455.78	0.44	included	present	462.67	-0.51	included	present	809.27	-0.18	included	present	210.05	-0.75	included	present	124.37	-0.83	included	present	1673.85	0.65	included	present	158.87	-0.8	included	absent	24.14	-0.93	included	present	356.53	-0.61	included	absent	122.66	-0.83	included	absent	76.31	-0.88	included	absent	31.33	-0.92	included	present	279.09	-0.68
220893_at	excluded	absent	14.91	-0.24	excluded	absent	48.61	1.02	excluded	absent	24.37	0.12	excluded	absent	34.88	0.51	excluded	absent	14.17	-0.26	excluded	absent	21.58	0.01	excluded	absent	59.75	1.44	excluded	absent	1.7	-0.73	excluded	absent	3.63	-0.66	excluded	absent	39.66	0.69	excluded	absent	2.29	-0.71	excluded	absent	25.94	0.18	excluded	absent	1.47	-0.74	excluded	absent	2.34	-0.71	excluded	absent	3.9	-0.65
220894_x_at	excluded	absent	70.14	-0.29	excluded	absent	65.24	-0.38	excluded	absent	13.14	-1.35	excluded	absent	8.05	-1.45	excluded	absent	15.11	-1.32	excluded	absent	105.54	0.37	excluded	absent	161.95	1.42	excluded	absent	76.56	-0.17	excluded	absent	144.1	1.09	excluded	absent	20.71	-1.21	excluded	absent	43.16	-0.79	excluded	absent	29.63	-1.05	excluded	absent	48.92	-0.69	excluded	absent	9.29	-1.42	excluded	absent	118.3	0.61
220895_at	excluded	absent	39.28	0.02	excluded	absent	1.89	-0.51	excluded	absent	38.95	0.01	excluded	absent	36.57	-0.02	excluded	absent	2.74	-0.5	excluded	absent	58.36	0.28	excluded	absent	4.39	-0.48	excluded	absent	3.07	-0.49	excluded	absent	6.41	-0.45	excluded	absent	27.21	-0.15	excluded	absent	49.22	0.16	excluded	absent	26.81	-0.16	excluded	absent	15.12	-0.32	excluded	absent	2.92	-0.5	excluded	absent	13.94	-0.34
220896_at	excluded	absent	23.92	0.09	excluded	absent	96.29	2.49	excluded	absent	3.48	-0.59	excluded	absent	3.13	-0.6	excluded	absent	97.95	2.54	excluded	absent	6.88	-0.48	excluded	absent	74.42	1.76	excluded	absent	65.28	1.46	excluded	absent	6.22	-0.5	excluded	absent	19.5	-0.06	excluded	absent	12.15	-0.3	excluded	absent	3.53	-0.59	excluded	absent	3.19	-0.6	excluded	absent	4.79	-0.55	excluded	absent	15.09	-0.21
220897_at	excluded	absent	42.34	1.01	excluded	absent	4.59	-0.57	excluded	absent	1.85	-0.68	excluded	absent	1.99	-0.68	excluded	absent	12.43	-0.24	excluded	absent	8.18	-0.42	excluded	absent	4.17	-0.58	excluded	absent	2.85	-0.64	excluded	absent	1.96	-0.68	excluded	absent	1.49	-0.7	excluded	absent	4.27	-0.58	excluded	absent	2.19	-0.67	excluded	absent	9.34	-0.37	excluded	absent	9.77	-0.35	excluded	absent	1.99	-0.68
220898_at	included	present	142.98	-0.12	included	absent	18.92	-1.16	included	absent	78.79	-0.66	included	present	105.24	-0.44	included	absent	73.05	-0.71	included	absent	166.74	0.08	included	absent	75.31	-0.69	included	absent	25.41	-1.11	included	present	131.18	-0.22	included	absent	16.33	-1.19	included	absent	86.67	-0.59	included	absent	67.03	-0.76	included	absent	70.05	-0.73	included	absent	5.96	-1.27	included	absent	44.87	-0.95
220899_at	included	present	315.5	0.59	included	present	327.95	0.71	included	present	309.35	0.54	included	present	311.95	0.56	included	present	360.73	1.02	included	present	169.23	-0.8	included	present	271.63	0.18	included	present	387.25	1.28	included	present	328.18	0.71	included	present	263.47	0.1	included	present	473.43	2.1	included	present	265.59	0.12	included	present	348.6	0.91	included	present	266.03	0.12	included	present	356	0.98
220900_at	included	present	61.49	0	excluded	absent	2.16	-1.34	excluded	absent	62.98	0.03	excluded	absent	79.84	0.41	excluded	absent	87.34	0.58	excluded	absent	14.65	-1.06	excluded	absent	6.4	-1.24	excluded	absent	23.42	-0.86	excluded	absent	43.34	-0.41	included	present	58.15	-0.08	included	present	79.1	0.4	excluded	absent	2.85	-1.32	excluded	absent	40.09	-0.48	excluded	absent	1.5	-1.35	excluded	absent	18.55	-0.97
220901_at	excluded	absent	64.89	0.35	excluded	absent	6.92	-0.93	excluded	absent	2.7	-1.02	excluded	absent	20.67	-0.62	included	present	167.41	2.6	excluded	absent	49.6	0.01	excluded	absent	68.78	0.43	excluded	absent	60.68	0.25	excluded	absent	51.35	0.05	excluded	absent	60.38	0.25	excluded	absent	53.7	0.1	excluded	absent	49.26	0	excluded	absent	71.19	0.49	excluded	absent	60.12	0.24	excluded	absent	46.59	-0.05
220902_at	excluded	absent	6.54	-0.88	excluded	absent	6.57	-0.88	excluded	absent	10.76	-0.77	excluded	absent	3.39	-0.96	excluded	absent	8.8	-0.82	excluded	absent	95.17	1.4	excluded	absent	15.9	-0.64	excluded	absent	120.2	2.04	excluded	absent	68.86	0.72	excluded	absent	7.48	-0.86	excluded	absent	14.26	-0.68	excluded	absent	149.93	2.8	excluded	absent	20.9	-0.51	excluded	absent	9.5	-0.8	excluded	absent	7.39	-0.86
220903_at	included	present	60.74	0.11	included	present	119.17	1.59	excluded	absent	62.96	0.17	excluded	absent	6.16	-1.27	excluded	absent	24.27	-0.81	excluded	absent	98.42	1.07	excluded	absent	68.4	0.31	excluded	absent	32.87	-0.59	included	present	90.62	0.87	excluded	absent	49.17	-0.18	excluded	absent	49.71	-0.17	excluded	absent	66.79	0.27	excluded	absent	34.36	-0.56	excluded	absent	41.47	-0.38	excluded	absent	53.36	-0.08
220904_at	excluded	absent	51.3	0.44	excluded	absent	70.55	0.94	excluded	absent	6.94	-0.7	excluded	absent	41.06	0.18	excluded	absent	5.94	-0.73	excluded	absent	6.73	-0.71	excluded	absent	62.32	0.72	excluded	absent	5.56	-0.74	excluded	absent	49.95	0.41	excluded	absent	15.23	-0.49	excluded	absent	87.03	1.36	excluded	absent	59.81	0.66	excluded	absent	34.1	0	excluded	absent	43.22	0.23	excluded	absent	59.79	0.66
[illegible]																																																												

221231_s_at	excluded	absent	59.34	0.1	excluded	absent	12.76	-0.89	excluded	absent	37.33	-0.37	included	present
221232_s_at	excluded	absent	7.17	-0.27	excluded	absent	135.28	-0.13	included	present	85.55	-0.18	excluded	absent
221233_s_at	included	absent	115.68	-1.15	included	present	165.01	-0.46	included	present	198.15	0.01	included	present
221234_s_at	included	present	274.1	1.05	included	present	626.12	4.03	included	present	181.71	0.27	included	present
221235_s_at	included	present	302.46	0.69	included	present	256.56	0.11	included	present	201.83	-0.58	included	present
221236_s_at	included	present	4569.65	-0.14	included	present	27536.73	2.59	included	present	9645.54	0.46	excluded	absent
221237_s_at	excluded	absent	77.72	-1	excluded	absent	73.09	-1.07	excluded	absent	59.79	-1.25	excluded	absent
221238_at	excluded	absent	17.63	-1.14	excluded	absent	33.77	-0.91	excluded	absent	1.07	-1.38	excluded	absent
221239_s_at	excluded	absent	5.36	-0.36	excluded	absent	37.33	-0.21	excluded	absent	23.39	-0.28	excluded	absent
221240_s_at	excluded	absent	98.26	0.68	included	present	131.98	1.31	excluded	absent	34.22	-0.53	excluded	absent
221241_s_at	excluded	absent	7.49	-0.38	excluded	absent	58.83	-0.27	excluded	absent	69.89	-0.25	excluded	absent
221242_at	excluded	absent	8.79	-0.92	excluded	absent	95.69	1.4	excluded	absent	34.77	-0.22	excluded	absent
221243_s_at	included	present	84.73	-0.01	included	present	51.35	-0.71	excluded	absent	58.95	-0.55	excluded	absent
221244_s_at	included	present	907.16	-0.54	included	present	861.59	-0.62	included	present	1699.19	0.76	included	present
221245_s_at	included	present	140.86	-0.53	included	present	266.29	-0.43	included	present	321.37	-0.39	included	present
221246_x_at	included	present	803.84	-0.47	included	present	278.94	-0.76	included	absent	177.84	-0.82	included	present
221247_s_at	excluded	absent	147.35	-0.17	excluded	absent	278.32	1.29	excluded	absent	88.4	-0.83	excluded	absent
221248_s_at	included	present	443.82	0.18	included	present	423.92	0.03	included	present	722.45	2.25	included	present
221249_s_at	included	present	466.18	-0.34	included	present	273.38	-0.84	included	present	168.12	-1.11	included	present
221250_s_at	excluded	absent	14.53	-1.48	excluded	absent	185.32	1.82	excluded	absent	180.57	1.73	excluded	absent
221251_x_at	excluded	absent	40.9	-0.79	excluded	absent	89.16	0.2	excluded	absent	3.16	-1.56	excluded	absent
221252_s_at	excluded	absent	2.47	-0.23	excluded	absent	10.71	-0.22	excluded	absent	3.16	-0.23	excluded	absent
221253_s_at	included	present	2585.93	-0.16	included	present	3064.36	-0.01	included	present	2366.51	-0.23	included	present
221254_s_at	excluded	absent	6.45	-0.73	excluded	absent	2.73	-0.85	excluded	absent	13.34	-0.51	excluded	absent
221255_s_at	included	present	109.51	-0.55	included	present	103.43	-0.6	included	present	92.84	-0.7	included	absent
221256_s_at	included	present	191.55	0.03	excluded	absent	34.95	-0.65	excluded	absent	51.01	-0.58	included	present
221257_x_at	included	present	631.27	-0.53	included	present	786.17	0.15	included	present	1237.83	2.12	included	present
221258_s_at	excluded	absent	84.77	-0.04	included	present	249.68	0.77	included	present	329.09	1.16	included	present
221259_s_at	excluded	absent	1.21	-0.73	excluded	absent	19.46	-0.48	excluded	absent	38.43	-0.21	excluded	absent
221260_s_at	included	present	402.36	-0.44	included	present	1464.03	4.76	included	present	341.33	-0.74	included	present
221261_x_at	included	present	1131.77	1.07	included	present	2704.13	4.06	included	present	1216.38	1.23	included	present
221262_s_at	excluded	absent	88.36	0.1	excluded	absent	41.15	-0.66	excluded	absent	39.1	-0.7	excluded	absent
221263_s_at	included	present	628.89	0.47	included	present	303.02	-1.19	included	present	477.95	-0.3	included	present
221264_s_at	included	present	2860.99	0.01	included	present	2597.08	-0.33	included	present	3967.96	1.43	included	present

[illegible]

221591_s_at	excluded	absent	63.42	0.58	excluded	absent	333.6	6.36	included	present	418.19	8.17	excluded	absent	119.76
221592_at	included	present	596.59	0.19	included	present	172.71	-1.33	included	present	510.56	-0.12	included	present	681.84
221593_s_at	included	present	459.85	-1.06	included	present	617.63	-0.63	included	present	1177.15	0.88	included	present	411.88
221594_at	included	present	1058.38	0.97	included	present	1018.2	0.85	included	present	1522.58	2.31	included	present	1690.64
221595_at	excluded	absent	44.67	-0.29	included	present	86.05	0.51	excluded	absent	95.61	0.69	excluded	absent	84.88
221596_s_at	included	present	135.37	-0.77	included	present	139.78	-0.72	included	present	153.48	-0.58	included	present	336.82
221597_s_at	included	present	574.79	0.31	included	present	807.39	1.28	included	present	451.59	-0.2	included	present	255.52
221598_s_at	excluded	absent	130.75	-0.11	included	present	172.16	0.5	included	present	104.23	-0.5	excluded	absent	92.19
221599_at	included	present	3460.25	-0.79	included	present	8858.13	0.03	included	present	4976.07	-0.56	included	present	5896.97
221600_s_at	included	present	353.35	-0.45	included	present	601.39	0.03	included	present	420.25	-0.32	included	present	715.92
221601_s_at	included	present	178.23	-0.14	excluded	absent	7.55	-0.3	excluded	absent	2.62	-0.31	excluded	absent	6.94
221602_s_at	excluded	absent	93.38	-0.18	excluded	absent	28.92	-0.31	excluded	absent	73.72	-0.22	excluded	absent	46.68
221603_at	excluded	absent	6.44	-0.52	excluded	absent	67.41	3.52	excluded	absent	7.36	-0.46	excluded	absent	43
221604_s_at	excluded	absent	128.23	-0.08	excluded	absent	111.98	-0.33	excluded	absent	155.19	0.34	excluded	absent	129.67
221605_s_at	included	present	124.75	-0.25	included	present	214.97	-0.22	included	present	193.14	-0.23	included	present	118.54
221606_s_at	included	present	123.34	-0.62	included	absent	69.04	-0.7	included	absent	39.13	-0.75	included	present	338.87
221607_x_at	included	present	32284.78	0.38	included	present	40437.55	1.41	included	present	37483.73	1.03	included	present	24626.81
221608_at	excluded	absent	87.46	0.83	excluded	absent	3.77	-0.98	excluded	absent	37.17	-0.26	excluded	absent	64.5
221609_s_at	excluded	absent	52.75	0	excluded	absent	65.94	0.27	excluded	absent	4.11	-1.01	excluded	absent	7.97
221610_s_at	excluded	absent	59.75	-0.59	excluded	absent	141.51	-0.37	excluded	absent	88.34	-0.51	included	present	207.21
221611_s_at	excluded	absent	91.81	-0.22	excluded	absent	41.77	-0.22	excluded	absent	9.33	-0.23	excluded	absent	40.81
221612_at	included	absent	86.25	-0.94	included	present	242.82	0.05	included	absent	101.85	-0.84	included	absent	116.37
221613_s_at	included	present	6161.94	-0.28	included	present	5756.83	-0.41	included	present	6042.84	-0.32	included	present	5158.58
221614_s_at	included	present	241.54	0.77	excluded	absent	129.04	-0.12	excluded	absent	89.47	-0.44	included	present	269.75
221615_at	included	present	353.95	-0.32	included	present	169.47	-1.23	included	present	195.31	-1.1	included	present	4637.82
221616_s_at	included	present	2106.81	1.13	included	present	1044.07	-0.94	included	present	2744.07	2.37	included	present	1665.04
221617_at	included	present	2373.09	-0.35	included	present	2398.51	-0.32	included	present	4457.35	2.2	included	present	3492.63
221618_s_at	included	present	1722.79	0.6	included	present	679.46	-2.11	included	present	2410.26	2.39	included	present	2021.54
221619_s_at	included	present	10477.19	-0.19	included	present	9489.83	-0.44	included	present	4103.98	-1.78	included	present	11291.64
221620_s_at	included	present	639.77	0.56	included	present	602.26	0.4	included	present	284.41	-0.91	included	present	422.79
221621_at	included	present	493.45	2.34	included	present	807.91	5.99	included	present	745.05	5.26	included	present	1401.74
221622_s_at	included	present	1484.54	-1.26	included	present	2476.07	0.69	included	present	1690.56	-0.86	included	present	1627.31
221623_at	excluded	absent	1.48	-0.69	excluded	absent	7.91	-0.62	excluded	absent	2.3	-0.68	excluded	absent	39.13
221624_at	excluded	absent	18.17	-0.52	excluded	absent	57.25	0.25	excluded	absent	2.86	-0.83	excluded	absent	44.62

221932_s_at	included	present	1677.77	-0.75	included	present	4177.56	1.15	included	present	1913.98
221933_at	included	present	2412.06	6.94	included	present	437.21	0.42	included	present	512.63
221934_s_at	included	present	156.67	-0.12	included	present	462.87	2.26	included	absent	127.75
221935_s_at	included	present	82.24	-0.91	included	absent	7.45	-1.37	included	absent	83.8
221936_x_at	included	present	227.5	-0.64	included	present	253.71	-0.51	included	present	210.33
221937_at	included	present	433.37	-0.47	included	present	948.27	1.25	included	present	360.68
221938_x_at	excluded	absent	125.24	0.09	excluded	absent	154.55	0.54	excluded	absent	104.41
221939_at	included	present	388.85	-0.25	included	present	600.14	0.87	excluded	absent	320.28
221940_at	included	present	140.27	-0.4	included	present	289.74	1.93	excluded	absent	208.21
221941_at	excluded	absent	154.23	-0.41	excluded	absent	142.33	-0.61	excluded	absent	15.22
221942_s_at	included	present	798.95	-0.74	included	present	885.26	-0.66	included	present	1199.68
221943_x_at	included	present	26302.38	-0.21	included	present	30357.83	0.27	included	present	34676.23
221944_at	included	absent	130.86	-0.36	included	present	232.22	0.91	included	present	204.97
221945_at	excluded	absent	100.15	-0.29	excluded	absent	169.87	0.54	included	present	225.2
221946_at	excluded	absent	13.18	-0.55	excluded	absent	49.8	-0.14	excluded	absent	10.38
221947_at	excluded	absent	296.53	1.05	excluded	absent	125.67	-1.17	excluded	absent	181.27
221948_s_at	excluded	absent	114.83	0.52	excluded	absent	19.19	-0.71	excluded	absent	49.96
221949_at	included	present	267.17	0.77	included	present	399.26	2.77	included	present	209.71
221950_at	included	absent	63.1	-0.7	included	absent	4.58	-0.73	included	absent	36.34
221951_at	included	present	368.78	-0.51	included	present	321.79	-0.76	included	present	248.92

221952_x_at	included	present	7696.31	-0.1	included	present	7826.13	-0.05	included	present	11571.65	1.28	included	present	13440.15	1.95	[illegible]
221953_s_at	included	absent	361.66	-1.03	included	present	1058.33	0.87	included	present	304.49	-1.19	included	present	317.27	-1.15	[illegible]
221954_at	included	present	389.17	-1.36	included	present	330.32	-1.44	included	present	1091.9	-0.48	included	present	672.2	-1.01	[illegible]
221955_at	excluded	absent	117.78	-0.08	excluded	absent	76.33	-0.64	excluded	absent	108.73	-0.2	included	present	213.78	1.23	[illegible]
221956_at	excluded	absent	60.98	0.33	excluded	absent	12.22	-0.79	excluded	absent	6.96	-0.91	excluded	absent	76.09	0.68	[illegible]
221957_at	included	present	810.2	0.32	included	present	182.62	-1.49	included	present	310.71	-1.12	included	present	669.78	-0.08	[illegible]
221958_s_at	included	present	1076.7	-0.61	included	present	121.68	-1.56	included	absent	78.55	-1.6	included	present	4974.41	3.27	[illegible]
221959_at	included	present	1377.59	2.12	included	present	296.84	-1.08	included	present	330.95	-0.98	included	present	734.58	0.21	[illegible]
221960_s_at	included	present	3459.72	1.19	included	present	2261.2	-0.46	included	present	1976.56	-0.85	included	present	1502.81	-1.5	[illegible]
221961_at	excluded	absent	176.94	-0.17	included	present	209.93	0.22	excluded	absent	175.3	-0.19	excluded	absent	103.85	-1.01	[illegible]
221962_s_at	included	present	633.08	-0.15	included	present	382.81	-1.16	included	present	406.62	-1.06	included	present	621.33	-0.2	[illegible]
221963_x_at	included	present	449.82	-0.55	included	present	818.69	0.36	included	present	1655.63	2.44	included	present	1698.89	2.55	[illegible]
221964_at	excluded	absent	281.23	0.38	excluded	absent	113.23	-0.92	excluded	absent	234.92	0.03	included	present	380.6	1.15	[illegible]
221965_at	included	present	137.09	-0.85	included	present	304.01	1.12	included	present	454.38	2.9	included	present	453.22	2.88	[illegible]
221966_at	excluded	absent	82.98	1.11	excluded	absent	19.65	-0.34	excluded	absent	7.76	-0.61	excluded	absent	19.03	-0.35	[illegible]
221967_at	excluded	absent	66.43	0.68	excluded	absent	22.12	-0.77	excluded	absent	23.28	-0.74	excluded	absent	100.71	1.8	[illegible]
221968_s_at	excluded	absent	80.94	0.27	excluded	absent	48.76	-0.22	excluded	absent	32.08	-0.48	excluded	absent	16.76	-0.71	[illegible]
221969_at	excluded	absent	17.08	-0.33	excluded	absent	9.57	-0.36	excluded	absent	354.78	0.96	excluded	absent	327.86	0.86	[illegible]
221970_s_at	included	present	521.08	0.06	included	present	1157.17	3.38	included	present	1142.81	3.31	included	present	1113.84	3.15	[illegible]
221971_x_at	included	present	532.9	-0.88	included	present	940.81	0.45	included	present	502.53	-0.98	included	present	321.86	-1.57	[illegible]
221972_s_at	excluded	absent	3.05	-0.66	excluded	absent	7.51	-0.56	excluded	absent	2.47	-0.67	excluded	absent	1.6	-0.69	[illegible]
221973_at	included	present	1301.84	-0.6	included	present	1580.25	-0.47	included	present	6604.52	1.87	included	present	4584.02	0.93	[illegible]
221974_at	included	present	725.67	-0.39	included	present	1482.94	0.95	included	present	622.55	-0.57	included	present	878.98	-0.12	[illegible]
221975_s_at	excluded	absent	13.84	-0.74	excluded	absent	66.81	0.43	excluded	absent	8.09	-0.87	excluded	absent	41.42	-0.13	[illegible]
221976_s_at	included	present	728.17	-0.52	included	present	2935.87	2.66	included	present	1553.14	0.67	included	present	1493.5	0.58	[illegible]
221977_at	included	present	377.07	1.9	included	present	556.25	3.4	included	present	352.39	1.7	included	present	141.05	-0.07	[illegible]
221978_at	included	absent	170.74	-0.36	included	absent	166.94	-0.36	included	present	116.33	-0.43	included	absent	41.73	-0.53	[illegible]
221979_at	excluded	absent	14.5	-1.09	excluded	absent	69.14	-0.4	excluded	absent	145.26	0.57	excluded	absent	92.76	-0.1	[illegible]
221980_at	excluded	absent	104.74	-0.01	excluded	absent	75.57	-0.48	excluded	absent	229.84	2	excluded	absent	67.83	-0.6	[illegible]
221981_s_at	included	present	580.77	-0.41	included	present	1002.25	2.05	included	present	962.68	1.82	included	present	381.18	-1.58	[illegible]
221982_x_at	included	present	337.04	-0.82	included	present	1664.63	2.17	included	present	433.23	-0.61	included	present	529.56	-0.39	[illegible]
221983_at	included	present	4242.94	0.23	included	present	2980.56	-0.82	included	present	1613.48	-1.96	included	present	1951.24	-1.68	[illegible]
221984_s_at	included	present	3819.93	-0.18	included	present	10003.33	2.09	included	present	3023.27	-0.47	included	present	2742.57	-0.58	[illegible]
221985_at	included	present	2645.04	-0.48	included	present	3505.78	0.33	included	present	4769.95	1.53	included	present	4769.48	1.53	[illegible]
[illegible]	[illegible]	[illegible]	[illegible]	[illegible]	[illegible]	[illegible]	[illegible]	[illegible]	[illegible]	[illegible]	[illegible]	[illegible]	[illegible]	[illegible]	[illegible]	[illegible]	[illegible]

222312_s_at	excluded	absent	9.58	-1.08	excluded	absent	6.22	-1.12	excluded	absent	4.74	[illegible]
222313_at	included	absent	106.27	-0.98	included	present	91.29	-1.12	included	present	109.47	[illegible]
222314_x_at	included	absent	5.29	-1.29	included	absent	13.06	-1.24	included	absent	43.4	[illegible]
222315_at	excluded	absent	30.42	-0.44	excluded	absent	50.85	-0.11	excluded	absent	2.67	[illegible]
222316_at	included	present	494.79	1.92	included	absent	94.58	-1.5	included	present	320.41	[illegible]
222317_at	excluded	absent	49.25	-0.43	excluded	absent	69.34	-0.16	excluded	absent	68.99	[illegible]
222318_at	excluded	absent	124.71	0.66	excluded	absent	109.55	0.43	excluded	absent	66.95	[illegible]
222319_at	included	absent	110.02	-0.75	included	present	176.88	-0.11	included	present	218.88	[illegible]
222320_at	excluded	absent	72.53	-0.74	excluded	absent	3.54	-1.95	included	present	89.69	[illegible]
222321_at	excluded	absent	74.9	0.14	excluded	absent	15.7	-0.51	excluded	absent	2.54	[illegible]
222322_at	excluded	absent	2.71	-0.59	excluded	absent	2.82	-0.58	excluded	absent	40.97	[illegible]
222323_at	excluded	absent	14.16	-1.08	excluded	absent	39.68	-0.44	excluded	absent	6.32	[illegible]
222324_at	excluded	absent	2.57	-0.95	excluded	absent	72.82	-0.2	excluded	absent	72.44	[illegible]
222325_at	excluded	absent	1.21	-0.72	excluded	absent	22.02	-0.09	excluded	absent	2.2	[illegible]
222326_at	excluded	absent	77.12	-0.46	excluded	absent	40.22	-0.68	excluded	absent	5.41	[illegible]
222327_x_at	included	present	854.83	0.59	included	present	241.26	-0.18	included	present	265.89	[illegible]
222328_x_at	included	present	9173.37	0.6	included	present	448	-0.71	included	present	3249.38	[illegible]
222329_x_at	included	present	1997.29	-0.23	included	present	1974.93	-0.25	included	present	2764.99	[illegible]
222330_at	excluded	absent	8.82	-1.06	excluded	absent	40.86	-0.24	excluded	absent	69.42	[illegible]
222331_at	excluded	absent	8.88	-0.8	excluded	absent	20.76	-0.46	excluded	absent	40.1	[illegible]
222332_at	excluded	absent	40.87	-0.64	excluded	absent	81.71	-0.14	excluded	absent	39.34	[illegible]
222333_at	excluded	absent	127.93	-0.51	excluded	absent	188.23	-0.19	excluded	absent	52.07	[illegible]
222334_at	excluded	absent	3.34	-0.69	excluded	absent	38.88	-0.07	excluded	absent	88.5	[illegible]
222335_at	included	present	139.95	1.7	excluded	absent	66.12	-0.03	excluded	absent	114.71	[illegible]
222336_at	included	present	490.18	0.87	included	present	180.42	-0.75	included	present	315.85	[illegible]
222337_at	excluded	absent	132.59	0.12	excluded	absent	80.3	-0.66	excluded	absent	75.42	[illegible]
222338_x_at	included	present	276.17	1.07	included	present	159.1	-0.52	included	present	259.27	[illegible]
222339_x_at	included	present	2066.16	-0.75	included	present	285.85	-1.17	included	present	6845.96	[illegible]
222340_at	excluded	absent	45.82	0.05	excluded	absent	5.8	-0.61	excluded	absent	1.93	[illegible]
222341_x_at	included	present	115.3	-0.03	excluded	absent	106.18	-0.15	excluded	absent	149.45	[illegible]
222342_at	excluded	absent	94.45	1.01	excluded	absent	51.21	0.14	excluded	absent	37.8	[illegible]
222343_at	included	present	429.25	-0.42	included	present	223.65	-0.78	included	present	1317.01	[illegible]
222344_at	included	present	282.98	1.12	excluded	absent	112.82	-0.1	included	present	1359.43	[illegible]
222345_at	excluded	absent	15.21	-0.9	excluded	absent	14.49	-0.91	excluded	absent	45.46	[illegible]
[illegible]	[illegible]	[illegible]	[illegible]	[illegible]	[illegible]	[illegible]	[illegible]	[illegible]	[illegible]	[illegible]	[illegible]	[illegible]

222671_s_at	excluded	absent	53.95	0.01	excluded	absent	12.97	-0.9	excluded	absent	5.68	[illegible]
222672_at	included	present	126.78	0.14	included	present	311.28	3.19	excluded	absent	112.47	[illegible]
222673_x_at	included	present	717	-0.09	included	present	644.64	-0.29	included	present	449.34	[illegible]
222674_at	included	present	210.62	0.62	included	present	217.75	0.72	included	present	119.34	[illegible]
222675_s_at	excluded	absent	113.65	-0.15	excluded	absent	80.91	-0.45	excluded	absent	38.85	[illegible]
222676_at	excluded	absent	19.99	-0.69	excluded	absent	3.08	-0.87	excluded	absent	41.73	[illegible]
222677_x_at	excluded	absent	5.52	-0.57	excluded	absent	6.04	-0.56	excluded	absent	4.38	[illegible]
222678_s_at	included	present	1996.94	-0.34	included	present	1953.33	-0.36	included	present	2730.17	[illegible]
222679_s_at	included	present	1051.63	2.41	included	present	310.47	-0.98	included	present	1186.92	[illegible]
222680_s_at	excluded	absent	40.59	-0.25	included	present	547.02	1.92	included	present	1150.01	[illegible]
222681_at	included	present	341.21	-0.64	included	present	565.44	0.56	included	present	719.85	[illegible]
222682_s_at	included	present	543.16	-0.2	included	present	744.15	0.25	included	present	366.93	[illegible]
222683_at	included	present	1222.78	0.93	included	present	980.68	0.1	included	present	1533.83	[illegible]
222684_s_at	included	present	851.04	-0.5	included	present	1869.27	1.83	included	present	1449.2	[illegible]
222685_at	included	present	86.25	-1	included	present	236.26	0.84	included	present	184.43	[illegible]
222686_s_at	included	present	358.76	0.97	included	present	178.73	-0.52	included	present	526.52	[illegible]
222687_s_at	included	present	598.25	0.37	included	present	403.38	-0.32	included	present	166.49	[illegible]
222688_at	included	present	393.47	-0.38	included	present	250.85	-0.94	included	present	202.41	[illegible]
222689_at	included	present	278.92	0	included	present	206.22	-0.52	included	present	101.6	[illegible]
222690_s_at	included	present	1259.26	0.56	included	present	178.99	-1.46	included	present	483.83	[illegible]
222691_at	included	present	615.49	-0.14	included	present	1201.56	2.23	included	present	410.32	[illegible]
222692_s_at	included	present	1859.69	2.01	included	present	268.32	-0.78	included	present	340.99	[illegible]
222693_at	included	present	647.32	0.95	included	absent	91.95	-0.98	included	present	241.08	[illegible]
222694_at	excluded	absent	25.26	-1.18	excluded	absent	140.55	1.48	excluded	absent	85.59	[illegible]
222695_s_at	excluded	absent	114.36	0.07	excluded	absent	77.86	-0.26	excluded	absent	66.35	[illegible]
222696_at	included	present	323.44	-0.48	included	present	180.42	-0.81	included	present	244.36	[illegible]
222697_s_at	included	present	1523.42	-0.12	included	present	1754.66	0.23	included	present	1236.65	[illegible]
222698_s_at	included	present	273.15	0.23	included	present	242.71	-0.01	included	present	206.23	[illegible]
222699_s_at	included	present	604.95	-0.24	included	present	100.45	-1.06	included	present	246.42	[illegible]
222700_at	included	present	339.71	-0.98	included	present	366.84	-0.94	included	present	584.03	[illegible]
222701_s_at	included	present	1619.06	-0.71	included	present	428.8	-1.71	included	present	1193.45	[illegible]
222702_x_at	included	present	707.97	-1.7	included	present	1686.85	1.15	included	present	1444.78	[illegible]
222703_s_at	included	present	167.93	0.16	included	present	802.17	9.41	included	present	204.08	[illegible]
222704_at	excluded	absent	193.46	0.86	excluded	absent	209.89	1.09	excluded	absent	112.84	[illegible]

[illegible]

223030_at	excluded	absent	101.84	1.87	excluded	absent	10.74	-0.59	excluded	absent	4.99	-0.75	excluded	absent	4.83	-0.75	excluded	absent	132.34	[illegible]
223031_s_at	included	present	362.38	-0.87	included	present	1901.59	9.12	included	present	552.88	0.36	included	present	301.65	-1.27	included	present	398.49	[illegible]
223032_x_at	included	present	1617.86	-0.55	included	present	3251.85	1.9	included	present	3119.45	1.7	included	present	2283.3	0.45	included	present	2523.73	[illegible]
223033_s_at	included	present	474.69	1.98	included	present	365.85	0.8	excluded	absent	196.31	-1.04	included	present	154.88	-1.49	excluded	absent	210.18	[illegible]
223034_s_at	included	present	4520.43	-0.01	included	present	5584.62	0.67	included	present	9749.65	3.34	included	present	8608.13	2.61	included	present	6678.22	[illegible]
223035_s_at	included	present	1462.68	0.54	included	present	3092.55	5.01	included	present	720.23	-1.5	included	present	1378.33	0.31	included	present	1013.42	[illegible]
223036_at	excluded	absent	43.95	0.8	excluded	absent	58.65	1.39	excluded	absent	45.96	0.88	excluded	absent	72.2	1.93	excluded	absent	87.5	[illegible]
223037_at	included	present	2291.37	1.56	included	present	1873.53	0.73	included	present	1676.56	0.34	included	present	1347.71	-0.32	included	present	1919.94	[illegible]
223038_s_at	included	present	312.52	-0.81	included	present	1483.66	0.3	included	present	5215.91	3.83	included	present	3573.61	2.28	included	present	813.01	[illegible]
223039_at	included	present	1208.11	-0.56	included	present	1814.27	-0.17	included	present	1462.57	-0.4	included	present	2690.23	0.39	included	present	1375.73	[illegible]
223040_at	included	present	1907.38	-0.59	included	present	2270.29	-0.22	included	present	2389.28	-0.1	included	present	2518.37	0.03	included	present	2879.46	[illegible]
223041_at	included	present	886.99	1.05	included	present	246.18	-0.81	included	absent	120.32	-1.18	included	present	578	0.15	included	absent	240.36	[illegible]
223042_s_at	included	present	3010.7	-0.24	included	present	2584.48	-0.42	included	present	2817.58	-0.32	included	present	1424.2	-0.93	included	present	1767.03	[illegible]
223043_at	included	present	1869.91	0.21	included	present	2644.63	1.98	included	present	2311.27	1.22	included	present	1731.9	-0.11	included	present	1417.87	[illegible]
223044_at	included	present	1850.95	-0.39	included	present	2745.35	-0.24	included	present	2801.87	-0.23	included	present	1254.51	-0.49	included	present	5429.58	[illegible]
223045_at	excluded	absent	3.73	-0.79	excluded	absent	9.49	-0.62	excluded	absent	4.53	-0.76	excluded	absent	4.88	-0.75	excluded	absent	20.29	[illegible]
223046_at	included	present	2553.11	-0.42	included	present	1153.77	-0.78	included	present	1838.11	-0.6	included	present	3501.16	-0.18	included	present	1920.7	[illegible]
223047_at	included	present	619.53	-0.5	included	present	346.26	-0.82	included	present	453.08	-0.69	included	present	982.77	-0.09	included	present	474.72	[illegible]
223048_at	included	present	674.16	3.4	included	present	431.77	1.02	included	present	488.47	1.58	included	present	319.8	-0.08	included	present	343.55	[illegible]
223049_at	included	present	2128.16	3.34	included	present	2558.56	4.76	included	present	1059.1	-0.17	included	present	923.41	-0.62	included	present	1668.49	[illegible]
223050_s_at	excluded	absent	34.8	-0.83	excluded	absent	28.93	-0.91	excluded	absent	10.96	-1.14	excluded	absent	70.85	-0.37	excluded	absent	108.28	[illegible]
223051_at	included	present	2255.44	-0.25	included	present	2109.17	-0.43	included	present	757.75	-2.09	included	present	1107.08	-1.66	included	present	3021.47	[illegible]
223052_x_at	included	present	681.78	1.02	included	present	322.7	-1.13	included	present	164.05	-2.08	included	present	279.04	-1.39	included	present	1285.22	[illegible]
223053_x_at	included	present	469.45	-0.28	included	present	579.17	0.13	included	present	220.91	-1.21	included	present	206.01	-1.27	included	present	941.8	[illegible]
223054_at	included	present	5108.99	0.57	included	present	11426.94	3.9	included	present	7973.24	2.08	included	present	7655.02	1.91	included	present	5103.9	[illegible]
223055_s_at	included	present	512.25	0.14	included	present	1628.54	4.88	included	present	1271.39	3.36	included	present	761.29	1.2	included	present	777.07	[illegible]
223056_s_at	excluded	absent	266.23	0.13	included	present	1570.55	11.67	included	present	531.93	2.48	included	present	538.89	2.54	included	present	540.7	[illegible]
223057_s_at	included	present	146.18	1.26	excluded	absent	223.07	2.68	included	present	235.05	2.91	included	present	131.4	0.98	excluded	absent	197.24	[illegible]
223058_at	included	present	1303.72	-0.47	included	present	1581.28	-0.3	included	present	2075.66	0.01	included	present	1596.79	-0.29	included	present	1177.1	[illegible]
223059_s_at	included	present	1191.22	-0.35	included	present	957.44	-0.56	included	present	2135.4	0.52	included	present	1391.45	-0.16	included	present	1399.65	[illegible]
223060_at	included	present	2492.72	0.15	included	present	2827.28	0.53	included	present	1857.61	-0.56	included	present	2284.54	-0.08	included	present	2999.85	[illegible]
223061_at	included	present	1097.01	2.02	included	present	1112	2.08	included	present	453.72	-0.38	included	present	373.84	-0.67	included	present	372.35	[illegible]
223062_s_at	included	present	1016.19	-0.43	included	present	338.79	-0.77	included	present	1149.01	-0.36	included	present	532.34	-0.67	included	present	706.75	[illegible]
223063_at	included	present	6035.99	3.42	included	present	833.65	-0.55	included	present	1331.44	-0.17	included	present	1509.64	-0.03	included	present	744.94	[illegible]
223064_at	included	present	1789.91	-0.22	included	present	1633.99	-0.48	included	present	1121.04	-1.31	included	present	1047.81	-1.43	included	present	1293.79	[illegible]
223065_s_at	included	present	1503.92	0.51	included	present	2050.85	1.71	included	present	1028.63	-0.54	included	present	863.18	-0.91	included	present	1769.36	[illegible]
223066_at	included	present	777.41	-0.66	included	present	1047.81	0.29	included	present	1330.74	1.29	included	present	1636.02	2.36	included	present	2432.98	[illegible]
223067_at	included	present	2981.09	-0.52	included	present	5714.2	1.61	included	present	5020.17	1.07	included	present	3499.1	-0.12	included	present	2778.38	[illegible]
223068_at	included	present	1174.83	-0.41	included	present	1844.83	0.13	included	present	2698.21	0.82	included	present	2434.07	0.6	included	present	1998.81	[illegible]
223069_s_at	included	present	674.42	0.31	included	present	900.05	0.93	included	present	1047.73	1.34	included	present	761.84	0.55	included	present	992.31	[illegible]
223070_at	included	present	3285.18	-0.39	included	present	2604.25	-0.87	included	present	2841.7	-0.7	included	present	2567.89	-0.9	included	present	3161.86	[illegible]
223071_at	included	present	1860.35	0.12	included	present	3346.88	1.96	included	present	1306.73	-0.57	included	present	3722.45	2.43	included	present	2303.85	[illegible]
223072_s_at	included	present	310	-0.28	included	present	648	2.44	included	present	306.49	-0.3	included	present	290.21	-0.44	included	present	402.73	[illegible]
223073_at	included	present	1375.91	1.08	included	present	915.38	-0.2	included	present	1020.57	0.1	included	present	1291.55	0.85	included	present	1381.49	[illegible]
223074_s_at	included	present	225.86	-0.19	included	absent	46.99	-0.71	included	absent	42.29	-0.72	included	absent	41.27	-0.73	included	absent	14.2	[illegible]
223075_s_at	included	present	1914.12	-0.46	included	present	212.69	-0.88	included	present	299.5	-0.86	included	present	589.04	-0.79	included	present	364.77	[illegible]
223076_s_at	included	present	1051.57	-1.34	included	present	1852.43	0.77	included	present	1558.18	0	included	present	1493.42	-0.17	included	present	1402.63	[illegible]
223077_at	included	present	649.49	0.07	included	present	669.03	0.13	included	present	609.04	-0.06	included	present	1178.65	1.74	included	present	497.27	[illegible]

223391_at	included	present	522.28	-0.99	included	present	308.13	-1.51	included	present	599.6	-0.8	included	present	695.37	-0.57
223392_s_at	included	present	1580.79	1.23	included	present	966.57	0.37	included	present	775.43	0.1	included	absent	95.42	-0.85
223393_s_at	included	present	1817.51	2.04	included	present	941.12	0.6	included	present	917.45	0.57	included	absent	15.81	-0.91
223394_at	included	present	544.37	0.31	included	absent	122.71	-1.04	included	absent	129.34	-1.02	included	present	215.81	-0.74
223395_at	included	present	753.33	0.47	included	present	107.72	-0.82	included	absent	38.27	-0.96	included	absent	21.81	-1
223396_at	included	present	645.84	1.27	included	present	1064.54	4.61	included	present	392.89	-0.74	included	present	1136.5	5.18
223397_s_at	included	present	379.48	-0.34	included	present	764.46	2.04	included	present	1026.61	3.67	included	present	323.09	-0.69
223398_at	included	present	440.44	2.67	excluded	absent	182.14	-0.17	excluded	absent	22.02	-1.93	excluded	absent	85.89	-1.23
223399_x_at	included	present	464.44	1	included	present	389.35	0.44	included	present	609.09	2.08	included	present	231.58	-0.73
223400_s_at	included	present	360.71	1.63	included	present	208.96	-0.13	included	present	334.29	1.32	included	present	133.81	-1
223401_at	included	present	163.35	-0.78	included	present	348.97	-0.16	included	present	363.6	-0.11	included	present	316.38	-0.27
223402_at	included	present	724.21	0.24	included	present	1512.59	2.87	included	present	232.45	-1.4	included	present	990.64	1.13
223403_s_at	included	present	289.5	-0.32	included	present	1119.23	4.58	included	present	661.28	1.87	included	present	169.61	-1.03
223404_s_at	included	present	427.84	-0.95	included	present	619.71	-0.41	included	present	370.99	-1.11	included	present	489.65	-0.78
223405_at	included	present	189.02	-0.11	included	present	274.65	0.3	included	absent	77.83	-0.64	included	absent	40.63	-0.82
223406_x_at	included	present	872.82	-0.26	included	present	1828.01	3.14	included	present	1892.41	3.37	included	present	564.09	-1.36
223407_at	included	present	253.86	-0.63	included	present	708.97	2.12	included	present	242.99	-0.7	included	present	357.86	-0.01
223408_s_at	included	present	1348.54	1.87	included	present	1740.84	3.07	included	present	3368.44	8.03	included	present	3744.93	9.17
223409_at	included	present	809.82	2.18	included	present	956.52	2.89	included	present	1651	6.25	included	present	2292.22	9.36
223410_s_at	included	present	524.67	1.49	included	present	612.96	2.09	included	present	1809.33	10.32	included	present	869.63	3.86
223411_at	included	present	977.26	-0.36	included	present	2314.69	2.49	included	present	1229.66	0.18	included	present	1308.84	0.35
223412_at	included	present	383.72	-0.26	included	present	404.93	-0.12	included	present	724.66	2.03	included	present	525.04	0.69

223751_x_at	excluded	absent	70.48	-0.14	excluded	absent	48.32	-0.36	excluded	absent	99.89	0.15	excluded	absent	34.04	-0.51	excluded	absent	74.6	-0.1	excluded	absent	9.68	-0.75	included	present	95.79	0.11	excluded	absent	55.45	-0.29	included	present	109.71	0.25	excluded	absent	47.31	-0.37	excluded	absent	72.98	-0.12	excluded	absent	59.36	-0.25	included	present	95.83	0.11	excluded	absent	45.98	-0.39	excluded	absent	72.89	-0.12
223752_at	excluded	absent	4.64	-0.58	excluded	absent	52.94	0.05	excluded	absent	38.38	-0.14	excluded	absent	60.81	0.15	excluded	absent	2.86	-0.6	excluded	absent	12.63	-0.47	excluded	absent	83.82	0.44	excluded	absent	3.99	-0.58	excluded	absent	34.81	-0.19	excluded	absent	57.15	0.1	included	present	81.28	0.41	excluded	absent	67.63	0.24	excluded	absent	34.18	-0.2	excluded	absent	2.87	-0.6	excluded	absent	13.54	-0.46
223753_s_at	excluded	absent	114.91	-0.04	excluded	absent	24.77	-0.65	excluded	absent	3.61	-0.79	included	present	159.82	0.27	excluded	absent	80.22	-0.27	excluded	absent	104.93	-0.1	included	present	153.77	0.23	included	present	68.79	-0.35	included	present	49.18	-0.48	excluded	absent	1.57	-0.81	included	present	185.5	0.45	included	present	226.31	0.72	excluded	absent	8.21	-0.76	excluded	absent	63.2	-0.39	included	present	91.05	-0.2
223754_at	included	present	1478.16	0.45	included	absent	85.29	-0.45	included	absent	196.78	-0.37	included	present	174.25	-0.39	included	absent	122.07	-0.42	included	absent	184.33	-0.38	included	absent	40.03	-0.48	included	absent	131.41	-0.42	included	present	399.46	-0.24	included	present	276.96	-0.32	included	absent	186.31	-0.38	included	absent	129.74	-0.42	included	absent	249.75	-0.34	included	absent	93.04	-0.44	included	absent	91.5	-0.44
223755_at	excluded	absent	90.13	0.89	excluded	absent	76.72	0.59	excluded	absent	119.98	1.57	included	present	631.7	13.18	excluded	absent	14.78	-0.82	excluded	absent	73.94	0.52	excluded	absent	76.57	0.58	excluded	absent	291.47	5.46	excluded	absent	46.38	-0.1	excluded	absent	38.52	-0.28	excluded	absent	59.11	0.19	excluded	absent	75.61	0.56	included	present	274.71	5.08	excluded	absent	14.37	-0.83	excluded	absent	49.71	-0.03
223756_at	included	present	103.55	-0.28	excluded	absent	70.33	-0.75	excluded	absent	34.55	-1.25	included	present	97.15	-0.37	excluded	absent	100.64	-0.32	excluded	absent	49.24	-1.04	excluded	absent	121.88	-0.02	excluded	absent	29.46	-1.32	included	present	121.09	-0.03	excluded	absent	1.36	-1.72	included	present	131.93	0.13	excluded	absent	64.88	-0.82	excluded	absent	6.86	-1.64	excluded	absent	52.75	-0.99	included	present	70.67	-0.74
223757_at	excluded	absent	139.16	0.07	excluded	absent	45.07	-0.72	excluded	absent	1.92	-1.09	excluded	absent	41.64	-0.75	excluded	absent	51.24	-0.67	excluded	absent	436.62	2.58	excluded	absent	62.84	-0.57	excluded	absent	51.08	-0.67	excluded	absent	145.31	0.12	excluded	absent	65.09	-0.55	excluded	absent	103.64	-0.23	excluded	absent	92.08	-0.33	excluded	absent	54.2	-0.65	excluded	absent	10.48	-1.01	excluded	absent	30.52	-0.85
223758_s_at	included	present	3463.41	1.52	included	present	1888.55	-0.24	included	present	5205.39	3.47	included	present	6616.47	5.04	included	present	3968.1	2.09	included	present	1651.72	-0.5	included	present	1483.63	-0.69	included	present	1880.78	-0.24	included	present	1435.63	-0.74	included	present	969.49	-1.26	included	present	1283.37	-0.91	included	present	606.09	-1.67	included	present	1377.26	-0.81	included	present	1077.68	-1.14	included	present	1027.28	-1.2
223759_s_at	excluded	absent	83.31	0.01	excluded	absent	144.13	0.83	included	present	145.42	0.85	included	present	147.78	0.88	included	present	255.08	2.33	excluded	absent	64.33	-0.24	excluded	absent	30.69	-0.7	included	present	110.86	0.38	excluded	absent	94.67	0.17	excluded	absent	66.46	-0.21	included	present	165.18	1.12	excluded	absent	76	-0.09	included	present	118.13	0.48	included	present	82.62	0	included	present	124.99	0.58
223760_s_at	included	present	3322.14	3.24	included	present	3090.73	2.71	included	present	2029.93	0.28	included	present	1138.45	-1.77	included	present	1717.26	-0.44	included	present	3235.15	3.05	included	present	3349.95	3.31	included	present	4614.2	6.21	included	present	3301.73	3.2	included	present	3749.95	4.23	included	present	2847.65	2.16	included	present	1857.32	-0.12	included	present	1569.18	-0.78	included	present	1434.5	-1.09	included	present	4415.08	5.75
223761_at	excluded	absent	54.43	1.71	excluded	absent	35.67	0.84	excluded	absent	42.86	1.18	excluded	absent	3.25	-0.65	excluded	absent	4.11	-0.62	excluded	absent	6.21	-0.52	excluded	absent	64.15	2.16	excluded	absent	65.48	2.22	excluded	absent	11.37	-0.28	excluded	absent	6.27	-0.52	excluded	absent	18.88	0.07	excluded	absent	68.02	2.34	excluded	absent	19.73	0.11	excluded	absent	10.97	-0.3	excluded	absent	3.76	-0.63
223762_at	excluded	absent	43.18	-0.15	excluded	absent	76.45	-0.14	excluded	absent	55.26	-0.14	excluded	absent	15.15	-0.16	excluded	absent	11.95	-0.16	included	present	683.31	0.07	excluded	absent	5.54	-0.16	excluded	absent	52.35	-0.14	excluded	absent	31.46	-0.15	included	present	1933.13	0.5	excluded	absent	57.9	-0.14	excluded	absent	8.01	-0.16	excluded	absent	28.05	-0.15	excluded	absent	15.69	-0.16	excluded	absent	32.26	-0.15
223763_at	excluded	absent	164.21	0.31	excluded	absent	54.1	-0.79	excluded	absent	101.36	-0.32	included	present	199.4	0.66	excluded	absent	159.87	0.27	excluded	absent	87.24	-0.46	excluded	absent	72.09	-0.61	excluded	absent	66.56	-0.66	included	present	132.01	-0.01	excluded	absent	81.17	-0.52	included	present	170.49	0.38	excluded	absent	96.22	-0.37	excluded	absent	112.06	-0.21	excluded	absent	59.59	-0.73	excluded	absent	103.69	-0.29
223764_x_at	excluded	absent	3.95	-1.35	included	present	93.8	-0.36	excluded	absent	78.08	-0.53	excluded	absent	62.29	-0.7	excluded	absent	143.37	0.19	included	present	151.05	0.28	included	present	195.66	0.77	included	present	108.69	-0.19	included	present	199.83	0.81	included	present	103.56	-0.25	included	present	119.28	-0.08	included	present	67.65	-0.65	included	present	101.43	-0.27	included	present	53.04	-0.81	included	present	55.02	-0.78
223765_s_at	excluded	absent	50.37	-0.64	excluded	absent	90.36	0.12	excluded	absent	110.5	0.49	excluded	absent	58.66	-0.48	excluded	absent	86.27	0.04	included	present	137.8	1.01	included	present	122.95	0.73	included	present	105.64	0.4	included	present	146.93	1.18	included	present	160.71	1.44	included	present	203.82	2.25	included	present	153.63	1.31	excluded	absent	131.05	0.88	excluded	absent	58.87	-0.48	included	present	183.14	1.86
223766_at	included	present	584.82	0.6	included	present	411.82	-0.65	included	present	1264.79	5.51	included	present	437.08	-0.47	included	present	823.12	2.32	included	present	349.12	-1.1	included	present	350.14	-1.1	included	present	446.44	-0.4	included	present	505.93	0.03	included	present	362.01	-1.01	included	present	502.76	0.01	included	present	372.46	-0.94	included	present	302.29	-1.44	included	present	315.27	-1.35	included	present	297.11	-1.48
223767_at	excluded	absent	44.98	-0.11	excluded	absent	55.02	0.13	excluded	absent	8.71	-0.98	excluded	absent	6.3	-1.04	excluded	absent	40.98	-0.21	excluded	absent	108.82	1.43	excluded	absent	1.68	-1.15	excluded	absent	16.89	-0.79	excluded	absent	39.35	-0.25	excluded	absent	42.68	-0.17	included	present	106.17	1.36	included	present	140.83	2.2	included	present	55.77	0.15	included	present	108.69	1.42	included	present	120.56	1.71
223768_at	excluded	absent	8.95	-1.32	excluded	absent	55.91	-0.3	excluded	absent	4.16	-1.43	excluded	absent	71.32	0.04	included	present	211.61	3.12	excluded	absent	124.57	1.21	excluded	absent	55.27	-0.31	excluded	absent	47.27	-0.48	excluded	absent	69.31	0	excluded	absent	63.45	-0.13	excluded	absent	77.94	0.19	excluded	absent	46.76	-0.5	excluded	absent	80.44	0.24	excluded	absent	41.83	-0.6	excluded	absent	63.9	-0.12
223769_x_at	included	present	828.35	-0.74	included	present	1785.66	1.23	included	present	443.17	-1.53	included	present	1723.55	1.1	included	present	1709.46	1.07	included	present	979.93	-0.43	included	present	646.05	-1.12	included	present	970.95	-0.45	included	present	1023.39	-0.34	included	present	1126.24	-0.13	included	present	1603.01	0.85	included	present	637.15	-1.13	included	present	417.53	-1.58	included	present	300.87	-1.82	included	present	1071.77	-0.24
223770_x_at	included	present	187.62	-0.45	excluded	absent	211.04	-0.18	excluded	absent	206.61	-0.23	included	present	254.79	0.33	excluded	absent	169.64	-0.66	included	present	377.56	1.76	excluded	absent	303.24	0.89	included	present	452.75	2.64	included	present	392.27	1.93	included	present	541.99	3.68	included	present	438.95	2.48	excluded	absent	233.54	0.08	included	present	165.38	-0.71	excluded	absent	121.29	-1.23	included	present	303.51	0.9
223771_at	excluded	absent	39.33	-1.18	excluded	absent	95.71	-0.28	excluded	absent	55.28	-0.92	excluded	absent	86.63	-0.42	excluded	absent	84.12	-0.46	excluded	absent	58.83	-0.87	included	present	131.62	0.29	excluded	absent	19.83	-1.49	included	present	174.27	0.97	excluded	absent	46.74	-1.06	included	present	91.65	-0.34	excluded	absent	32.11	-1.29	excluded	absent	86.49	-0.43	excluded	absent	77.02	-0.58	excluded	absent	16.89	-1.53
223772_s_at	excluded	absent	53.27	0.1	excluded	absent	46.35	-0.1	excluded	absent	52.58	0.08	included	present	60.46	0.31	excluded	absent	76.29	0.78	excluded	absent	8.39	-1.22	excluded	absent	46.06	-0.11	included	present	82.97	0.98	included	present	68.95	0.56	excluded	absent	10.62	-1.15	included	present	106.67	1.67	excluded	absent	55.86	0.18	excluded	absent	27.22	-0.67	excluded	absent	5.59	-1.3	excluded	absent	15.74	-1
223773_s_at	included	present	1423.68	-1.08	included	present	3643.41	0.03	included	present	5009.23	0.71	included	present	1902.94	-0.84	included	present	3461.41	-0.06	included	present	2631.02	-0.47	included	present	2372.44	-0.6	included	present	3346.54	-0.12	included	present	1882.77	-0.85	included	present	2014.5	-0.78	included	present	1945.21	-0.82	included	present	6919.83	1.67	included	present	5173.41	0.8	included	present	6371.71	1.39	included	present	2020.59	-0.78
223774_at	included	present	249.4	-0.49	included	present	488.2	0.46	included	present	798.9	1.69	included	present	354.92	-0.07	included	present	520.63	0.59	included	present	386.65	0.05	included	present	357.51	-0.06	included	present	541.79	0.67	included	present	378.1	0.02	included	present	409.52	0.15	included	present	373.4	0	included	present	256.52	-0.46	included	present	259.82	-0.45	included	present	142.27	-0.92	included	present	468.85	0.38
223775_at	included	present	113.72	-0.43	excluded	absent	46.19	-0.58	excluded	absent	45.28	-0.58	excluded	absent	4.94	-0.68	included	present	247.18	-0.13	excluded	absent	40.54	-0.6	included	present	572.91	0.59	excluded	absent	3.45	-0.68	included	present	131.39	-0.39	excluded	absent	69.35	-0.53	excluded	absent	37.05	-0.6	excluded	absent	61.2	-0.55	included	present	91.24	-0.48	included	present	129.82	-0.4	excluded	absent	15.22	-0.65
223776_x_at	included	present	646.48	1.3	included	present	863.23	2.61	included	present	346.25	-0.51	included	present	573.09	0.86	included	present	549.33	0.71	included	present	936.09	3.06	included	present	606.97	1.06	included	present	853.96	2.56	included	present	691.84	1.58	included	present	738.81	1.86	included	present	527.11	0.58	included	present	620.86	1.15	included	present	486.58	0.33	included	present	222.64	-1.26	included	present	345.79	-0.52
223777_at	excluded	absent	62.23	-0.72	excluded	absent	6.66	-1.73	excluded	absent	9.46	-1.68	excluded	absent	77.75	-0.44	excluded	absent	3.02	-1.79	included	present	191.72	1.62	included	present	210.32	1.95	excluded	absent	88.19	-0.25	excluded	absent	129.2	0.49	excluded	absent	80.47	-0.39	included	present	206.42	1.88	included	present	135.33	0.6	included	present	195.65	1.69	excluded	absent	6.59	-1.73	included	present	147.62	0.82
223778_at	excluded	absent	39.1	-0.55	excluded	absent	112.31	-0.14	excluded	absent	26.25	-0.62	excluded	absent	92.67	-0.25	excluded	absent	5.69	-0.74	excluded	absent	75.58	-0.35	excluded	absent	122.96	-0.08	excluded	absent	57.53	-0.45	excluded	absent	92.17	-0.25	excluded	absent	82.57	-0.31	excluded	absent	111.24	-0.14	excluded	absent	14	-0.69	excluded	absent	97.79	-0.22	excluded	absent	85.07	-0.29	excluded	absent	34.18	-0.58
223779_at	excluded	absent	81.37	-0.54	excluded	absent	131.23	0.34	excluded	absent	45.49	-1.18	excluded	absent	69.93	-0.75	excluded	absent	86.52	-0.45	excluded	absent	15.98	-1.7	included	present	94.66	-0.31	excluded	absent	55.36	-1.01	included	present	105.16	-0.12	excluded	absent	87.84	-0.43	excluded	absent	63.51	-0.86	included	present	103.73	-0.15	excluded	absent	98.82	-0.24	excluded	absent	61.97	-0.89	included	present	83.02	-0.52
223780_s_at	included	present	1721.78	0.81	included	present	1011.94	-1.07	included	present	2861.64	3.82	included	present	3445.4	5.37	included	present	2473.88	2.8	included	present	1246.2	-0.45	included	present	3725.1	6.11	included	present	2550.48	3	included	present	2821.33	3.72	included	present	2349.85	2.47	included	present	3460.65	5.41	included	present	1137.27	-0.74	included	present	3558.79	5.67	included	present	4487.49	8.12	included	present	4398.53	7.89
223781_x_at	included	present	364.19	-0.27	included	present	401.76	-0.25	included	present	547.88	-0.21	included	absent	359.21	-0.27	included	absent	252.52	-0.3	included	present	635.85	-0.18	included	present	319.87	-0.28	included	present	257.03	-0.3	included	present	425.15	-0.25	included	absent	340.76	-0.27	included	absent	310.3	-0.28	included	present	474.45	-0.23	included	present	498.65	-0.22	included	absent	248.93	-0.3	included	absent	162.43	-0.33
223782_s_at	excluded	absent	11.67	-0.2	excluded	absent	53.67	-0.12	excluded	absent	35.66	-0.15	excluded	absent	19.1	-0.18	excluded	absent	60.15	-0.11	excluded	absent	46.65	-0.13	excluded	absent	58.61	-0.11	excluded	absent	24.82	-0.17	excluded	absent	5.49	-0.21	excluded	absent	3.04	-0.21	excluded	absent	95.06	-0.05	excluded	absent	2.93	-0.21	excluded	absent	21.08	-0.18	excluded	absent	7.41	-0.2	excluded	absent	1.93	-0.21
223783_s_at	excluded	absent	60.29	-0.36	excluded	absent	322.9	0.14	excluded	absent	85.84	-0.31	excluded	absent	75.6	-0.33	included	present	309.15	0.11	excluded	absent	14.23	-0.45	excluded	absent	205.63	-0.08	excluded	absent	98.28	-0.29	excluded	absent	124.29	-0.24	excluded	absent	54.7	-0.37	excluded	absent	206.29	-0.08	excluded	absent	126.66	-0.23	excluded	absent	273.93	0.05	included	present	275.31	0.05	included	present	120.95	-0.24
223784_at	excluded	absent	68.98	-0.17	included	present	79.79	-0.16	included	present	199.3	-0.11	excluded	absent	56.59	-0.17	excluded	absent	20.66	-0.19	excluded	absent	41.31	-0.18	included	present	99.39	-0.15	excluded	absent	24.44	-0.19	excluded	absent	14.16	-0.19	excluded	absent	30.41	-0.19	included	present	71.21	-0.17	excluded	absent	116.87	-0.15	included	present	58.29	-0.17	included	present	117.09	-0.15	included	present	31.2	-0.18

[illegible]

224112_at	included	present	150.53	-0.2	included	absent	39.69	-1.29	included	present	187.6	0.16	included	present	93.45	-0.76	included	absent	157.24	[illegible]	[illegible]
224113_at	excluded	absent	119.78	1	excluded	absent	64.29	0.01	excluded	absent	76.33	0.22	excluded	absent	5.81	-1.04	excluded	absent	5.96	[illegible]	[illegible]
224114_at	excluded	absent	2.2	-0.93	excluded	absent	52.15	0.58	excluded	absent	17.04	-0.48	excluded	absent	16.61	-0.49	excluded	absent	4.38	[illegible]	[illegible]
224115_at	excluded	absent	44.26	0.21	excluded	absent	43.78	0.19	excluded	absent	96.79	1.38	excluded	absent	2.33	-0.73	excluded	absent	115.11	[illegible]	[illegible]
224116_at	excluded	absent	6.22	-1.57	excluded	absent	84.13	0.73	excluded	absent	77.52	0.54	excluded	absent	1.98	-1.69	excluded	absent	73.53	[illegible]	[illegible]
224117_at	excluded	absent	75.48	0	excluded	absent	11.65	-1.08	excluded	absent	79.35	0.07	excluded	absent	25.57	-0.84	included	present	130.29	[illegible]	[illegible]
224118_at	excluded	absent	181.32	-0.16	excluded	absent	15.94	-1.54	excluded	absent	86.89	-0.94	included	present	171.05	-0.24	excluded	absent	160.08	[illegible]	[illegible]
224119_at	excluded	absent	16.52	-0.46	excluded	absent	18.97	-0.38	excluded	absent	87.58	1.8	excluded	absent	47.9	0.54	excluded	absent	133.22	[illegible]	[illegible]
224120_at	excluded	absent	21.14	-0.31	excluded	absent	2.22	-0.7	excluded	absent	6.78	-0.61	excluded	absent	6.48	-0.61	excluded	absent	16.7	[illegible]	[illegible]
224121_x_at	excluded	absent	7.89	-0.97	excluded	absent	41.55	-0.5	excluded	absent	251.3	2.45	excluded	absent	6.55	-0.99	excluded	absent	153.16	[illegible]	[illegible]
224122_at	excluded	absent	4.25	-0.61	excluded	absent	82.64	2.05	excluded	absent	2.65	-0.66	excluded	absent	8.33	-0.47	excluded	absent	8.66	[illegible]	[illegible]
224123_at	excluded	absent	2.19	-1.03	excluded	absent	84.57	0.97	excluded	absent	2.19	-1.03	excluded	absent	6.87	-0.92	excluded	absent	53	[illegible]	[illegible]
224124_at	included	present	216.16	-0.72	included	present	239.22	-0.56	excluded	absent	348.95	0.19	included	present	116.67	-1.41	included	present	371.65	[illegible]	[illegible]
224125_at	excluded	absent	52.67	-0.21	excluded	absent	15.67	-0.72	excluded	absent	11.66	-0.78	excluded	absent	6.1	-0.86	excluded	absent	22.56	[illegible]	[illegible]
224126_at	excluded	absent	89.47	0.88	excluded	absent	5.92	-1.33	included	present	90.96	0.92	excluded	absent	33.09	-0.61	excluded	absent	111.02	[illegible]	[illegible]
224127_at	excluded	absent	109.3	0.73	excluded	absent	1.55	-1.12	excluded	absent	14.77	-0.89	excluded	absent	4.12	-1.08	excluded	absent	69.7	[illegible]	[illegible]
224128_at	excluded	absent	5.58	-0.88	excluded	absent	6.08	-0.87	excluded	absent	6.02	-0.87	excluded	absent	7.5	-0.83	excluded	absent	19.64	[illegible]	[illegible]
224129_s_at	included	present	1310.78	-0.91	included	present	2755.38	2.59	included	present	2974.91	3.13	included	present	2181.23	1.2	included	present	1418.9	[illegible]	[illegible]
224130_s_at	included	present	324.8	-0.39	included	present	373.08	-0.18	included	present	400.8	-0.05	included	present	327.03	-0.38	included	present	338.87	[illegible]	[illegible]
224131_at	included	present	88.55	0.82	included	present	165.35	2.53	excluded	absent	34.86	-0.38	excluded	absent	33.28	-0.42	excluded	absent	16.27	[illegible]	[illegible]
224132_at	excluded	absent	50.21	-0.48	excluded	absent	106.53	0.67	excluded	absent	64.55	-0.19	excluded	absent	11.25	-1.28	excluded	absent	45.63	[illegible]	[illegible]
224133_at	excluded	absent	85.43	0.09	excluded	absent	24.13	-0.93	excluded	absent	14.59	-1.09	excluded	absent	134.16	0.9	excluded	absent	189.65	[illegible]	[illegible]
224134_at	excluded	absent	95.55	-1.45	excluded	absent	129.69	-1.06	excluded	absent	150.66	-0.82	excluded	absent	140.89	-0.93	excluded	absent	151.64	[illegible]	[illegible]
224135_at	included	present	114.98	-0.01	excluded	absent	105.55	-0.07	excluded	absent	85.23	-0.18	excluded	absent	53.9	-0.36	included	present	128.04	[illegible]	[illegible]
224136_at	excluded	absent	44.66	0.48	excluded	absent	52.71	0.72	excluded	absent	11.41	-0.52	excluded	absent	11.59	-0.51	excluded	absent	3.28	[illegible]	[illegible]
224137_at	excluded	absent	38.26	-0.86	excluded	absent	101.44	0.45	excluded	absent	50.01	-0.62	excluded	absent	9.3	-1.47	excluded	absent	61.63	[illegible]	[illegible]
224138_at	excluded	absent	3.33	-0.92	excluded	absent	17.67	-0.48	excluded	absent	4.2	-0.9	excluded	absent	38.19	0.14	excluded	absent	5.05	[illegible]	[illegible]
224139_at	excluded	absent	6.56	-1.27	excluded	absent	5.38	-1.29	excluded	absent	97.73	0.62	included	present	74.18	0.13	excluded	absent	87.73	[illegible]	[illegible]
224140_at	excluded	absent	8.16	-0.57	excluded	absent	10.02	-0.48	excluded	absent	2.04	-0.84	excluded	absent	42.22	0.96	excluded	absent	56.34	[illegible]	[illegible]
224141_at	excluded	absent	2.64	-1.12	excluded	absent	18.01	-0.81	excluded	absent	10.24	-0.97	excluded	absent	3.81	-1.1	excluded	absent	17.76	[illegible]	[illegible]
224142_s_at	excluded	absent	1.97	-0.72	excluded	absent	11.12	-0.48	excluded	absent	12.82	-0.44	excluded	absent	2.26	-0.71	excluded	absent	4.11	[illegible]	[illegible]
224143_at	excluded	absent	80	0.37	excluded	absent	114.93	1.13	excluded	absent	62.65	-0.01	excluded	absent	2.42	-1.33	excluded	absent	14.08	[illegible]	[illegible]
224144_at	excluded	absent	39.29	-0.18	included	present	112.63	1.42	excluded	absent	86.01	0.84	excluded	absent	95.15	1.04	excluded	absent	41.88	[illegible]	[illegible]
224145_s_at	excluded	absent	92.52	-0.93	included	present	250.67	0.78	excluded	absent	152.51	-0.28	excluded	absent	72.5	-1.15	excluded	absent	143.59	[illegible]	[illegible]
224146_s_at	excluded	absent	6.46	-0.63	excluded	absent	12.41	-0.46	excluded	absent	4.61	-0.68	excluded	absent	3.18	-0.72	excluded	absent	7.43	[illegible]	[illegible]
224147_at	excluded	absent	1.34	-0.99	excluded	absent	43.97	0.09	included	present	136.81	2.43	excluded	absent	36.09	-0.11	included	present	136.4	[illegible]	[illegible]
224148_at	included	present	288.11	-0.03	included	present	155.83	-0.28	included	present	121.66	-0.35	included	absent	58.01	-0.47	included	present	335.81	[illegible]	[illegible]
224149_x_at	included	present	2913.08	-0.31	included	present	1114.69	-0.66	included	present	3123.64	-0.26	included	present	1160.24	-0.65	included	present	2432.63	[illegible]	[illegible]
224150_s_at	included	present	195.97	-0.44	included	present	163.65	-0.52	included	present	294.32	-0.2	included	present	287.16	-0.22	included	present	318.23	[illegible]	[illegible]
224151_s_at	included	present	741.73	0.11	included	present	180.65	-1.42	included	present	1190.88	1.33	included	present	620.36	-0.22	included	present	567.82	[illegible]	[illegible]
224152_s_at	included	present	680.77	1.98	included	present	740.57	2.38	included	present	546.6	1.07	included	present	229.4	-1.07	included	present	976.09	[illegible]	[illegible]
224153_s_at	included	present	1470.07	0.7	included	present	1455.03	0.68	included	present	1295.66	0.36	included	present	3116.47	3.92	included	present	932.68	[illegible]	[illegible]
224154_at	excluded	absent	2.77	-1.18	excluded	absent	2.25	-1.2	excluded	absent	7.73	-0.99	excluded	absent	16.88	-0.64	excluded	absent	118.86	[illegible]	[illegible]
224156_x_at	included	present	271.56	-0.54	included	present	370.69	-0.43	included	present	496.44	-0.3	included	present	2164.96	1.45	included	present	511.68	[illegible]	[illegible]
224157_at	excluded	absent	6.16	-0.65	excluded	absent	47.53	0.51	excluded	absent	88.24	1.65	excluded	absent	4.15	-0.71	excluded	absent	22.37	[illegible]	[illegible]
224158_s_at	included	present	282.04	0.31	included	present	129.99	-1.2	included	present	309.24	0.58	included	present	219.25	-0.32	included	absent	239.3	[illegible]	[illegible]
[illegible]	[illegible]	[illegible]	[illegible]	[illegible]	[illegible]	[illegible]	[illegible]	[illegible]	[illegible]	[illegible]	[illegible]	[illegible]	[illegible]	[illegible]	[illegible]	[illegible]	[illegible]	[illegible]	[illegible]	[illegible]	[illegible]

224484_s_at	included	present	227.96	-0.75	included	present	285.81	-0.39	included	present	545.99	1.24	included	present	314.92	-0.21	included	present	316.65	-0.19	included	present	265.55	-0.51	included	present	182.96	-1.03	included	present	922.2	3.58	included	present	265.77	-0.51	included	present	132.25	-1.35	included	present	238.37	-0.68	included	present	208.63	-0.87	included	present	185.16	-1.02	included	present	612	1.65	included	present	220.95	-0.79
224485_s_at	excluded	absent	3.12	-0.47	excluded	absent	34.5	0.75	excluded	absent	69.2	2.09	excluded	absent	61.27	1.78	excluded	absent	17.05	0.07	excluded	absent	19.55	0.17	excluded	absent	5.26	-0.39	excluded	absent	2.8	-0.48	excluded	absent	7.44	-0.3	excluded	absent	3.54	-0.45	excluded	absent	20.94	0.22	excluded	absent	5.22	-0.39	excluded	absent	32.48	0.67	excluded	absent	9.57	-0.22	excluded	absent	1.5	-0.53
224486_s_at	included	present	408.22	-0.26	included	present	1530.55	0.39	included	present	385.14	-0.27	included	present	1182.73	0.19	included	present	763.46	-0.05	included	present	431.46	-0.24	included	present	795.35	-0.03	included	present	808.92	-0.03	included	present	618.63	-0.14	included	present	950.11	0.05	included	present	488.29	-0.21	included	present	1282.04	0.24	included	present	1535.93	0.39	included	present	1316.67	0.26	included	present	1078.7	0.13
224487_at	excluded	absent	51.54	0.02	excluded	absent	15.49	-0.8	excluded	absent	21.76	-0.66	excluded	absent	3.32	-1.08	excluded	absent	117.37	1.51	excluded	absent	120.62	1.58	excluded	absent	10.81	-0.91	excluded	absent	3.33	-1.07	excluded	absent	62.19	0.26	excluded	absent	39.14	-0.26	excluded	absent	85.05	0.77	excluded	absent	62.96	0.27	excluded	absent	37.5	-0.3	excluded	absent	65.73	0.34	excluded	absent	26.73	-0.55
224488_s_at	excluded	absent	2.49	-1.55	excluded	absent	8.83	-1.43	excluded	absent	2.63	-1.55	excluded	absent	2.32	-1.55	excluded	absent	44.48	-0.74	included	present	1825.94	33.53	excluded	absent	17.74	-1.26	excluded	absent	27.08	-1.08	included	present	136.69	1.03	excluded	absent	47.87	-0.68	excluded	absent	11.64	-1.38	excluded	absent	71.75	-0.22	excluded	absent	51.52	-0.61	excluded	absent	49.77	-0.64	excluded	absent	52.1	-0.6
224489_at	included	present	761.72	-0.16	included	present	317.33	-1.87	included	present	1702.58	3.47	included	present	1146.27	1.32	included	present	1497.49	2.68	included	present	610.86	-0.74	included	present	623.88	-0.69	included	present	306.46	-1.91	included	present	1091.51	1.11	included	present	1359.01	2.14	included	present	2989.17	8.42	included	present	2720.79	7.39	included	present	1114.35	1.2	included	present	1551.85	2.89	included	present	354.44	-1.72
224490_s_at	included	present	719.15	0.96	included	present	185.07	-1.41	included	present	1176.68	2.99	included	present	615.42	0.5	included	present	1225.46	3.2	included	present	395.33	-0.48	included	present	776.94	1.21	included	present	303.71	-0.89	included	present	1174.34	2.98	included	present	1003.14	2.22	included	present	2287.69	7.92	included	present	1511.21	4.47	included	present	767.58	1.17	included	present	711.14	0.92	included	present	326.29	-0.79
224491_at	included	present	168.54	-0.05	excluded	absent	96.22	-0.55	included	present	399.31	1.54	included	present	86.34	-0.62	included	present	231.86	0.38	included	present	116.45	-0.41	excluded	absent	92.22	-0.58	excluded	absent	63.23	-0.78	included	present	44.33	-0.91	included	present	93.35	-0.57	included	present	122.36	-0.37	excluded	absent	136.24	-0.28	excluded	absent	70.39	-0.73	excluded	absent	5.88	-1.18	excluded	absent	44	-0.91
224492_s_at	included	present	379.58	0.01	included	present	403.55	0.17	included	present	572.57	1.26	included	present	1282.09	5.82	included	present	327.97	-0.32	included	present	288.79	-0.57	included	present	614.59	1.53	included	present	671.05	1.89	included	present	612.33	1.51	included	present	583.92	1.33	included	present	436.08	0.38	included	present	392	0.09	included	present	709.76	2.14	included	present	754.72	2.43	included	present	498.74	0.78
224493_x_at	included	present	3392.21	-0.51	included	present	5781.83	1.95	included	present	3950.84	0.07	included	present	2603.71	-1.32	included	present	3397.1	-0.5	included	present	3052.18	-0.86	included	present	3462.35	-0.43	included	present	2260.01	-1.67	included	present	3015.33	-0.89	included	present	2482.24	-1.44	included	present	3374.61	-0.52	included	present	3009.99	-0.9	included	present	3822.33	-0.07	included	present	3044.44	-0.86	included	present	2284.39	-1.64
224494_x_at	excluded	absent	121.27	-0.44	included	present	138.9	-0.25	excluded	absent	11.37	-1.66	included	present	107.27	-0.6	excluded	absent	142.79	-0.21	included	present	90.45	-0.78	excluded	absent	97.12	-0.71	excluded	absent	46.83	-1.27	included	present	167.82	0.07	included	present	111.81	-0.55	excluded	absent	78.77	-0.91	excluded	absent	193.92	0.36	excluded	absent	61.06	-1.11	excluded	absent	31.67	-1.43	included	present	110.85	-0.56
224495_at	included	present	474.39	2.08	included	present	313.88	0.94	included	present	158.98	-0.17	included	present	267.34	0.61	included	present	535.85	2.52	included	absent	77.26	-0.75	included	present	378.11	1.4	included	present	239.4	0.41	included	present	137.59	-0.32	included	present	252.48	0.5	included	present	195.9	0.1	included	present	151.6	-0.22	included	present	285.7	0.74	included	present	107.55	-0.53	included	present	600.6	2.98
224496_s_at	included	present	621.78	1.04	included	present	758.35	1.74	included	present	445.38	0.13	included	present	1223.76	4.12	included	present	1404.71	5.05	included	present	240.06	-0.92	included	present	587.78	0.86	included	present	317.12	-0.52	included	present	350.74	-0.35	included	present	542.81	0.63	included	present	720.72	1.55	included	present	600.4	0.93	included	present	774.54	1.82	included	present	428.26	0.05	included	present	765.75	1.78
224497_x_at	excluded	absent	84.32	-0.57	excluded	absent	15.07	-1.57	excluded	absent	45.41	-1.13	included	present	74.25	-0.72	excluded	absent	188.54	0.93	excluded	absent	88.94	-0.51	excluded	absent	15.28	-1.57	excluded	absent	41.33	-1.19	included	present	164.74	0.58	excluded	absent	50.52	-1.06	excluded	absent	27.42	-1.39	excluded	absent	85.27	-0.56	excluded	absent	54.57	-1	excluded	absent	19.21	-1.51	included	present	66.61	-0.83
224498_x_at	included	present	115.51	-0.49	included	present	187.64	0.22	included	present	153.51	-0.12	included	present	290.72	1.23	included	present	222.86	0.56	excluded	absent	19.1	-1.44	included	present	248.79	0.82	included	present	128.91	-0.36	included	present	149.67	-0.16	included	present	136.55	-0.29	included	present	136.83	-0.28	included	present	119.38	-0.46	excluded	absent	54.52	-1.09	excluded	absent	23.58	-1.4	included	present	159.4	-0.06
224499_s_at	excluded	absent	20.78	-0.17	excluded	absent	36.57	0.14	excluded	absent	30.5	0.02	excluded	absent	2.06	-0.55	excluded	absent	15.54	-0.28	excluded	absent	28.03	-0.03	excluded	absent	49.76	0.4	excluded	absent	26.48	-0.06	excluded	absent	16.43	-0.26	excluded	absent	2.38	-0.54	excluded	absent	11.97	-0.35	excluded	absent	3.86	-0.51	excluded	absent	3.07	-0.53	excluded	absent	3.35	-0.52	excluded	absent	16.79	-0.25
224500_s_at	included	present	209.95	0.99	excluded	absent	146.09	0.11	excluded	absent	145.95	0.1	included	present	65.15	-1.02	included	present	190.28	0.72	excluded	absent	271	1.84	included	present	544	5.62	included	present	409.15	3.75	included	present	279.07	1.95	included	present	287.1	2.06	included	present	286.96	2.06	included	present	149.76	0.16	included	present	180.74	0.59	excluded	absent	72.69	-0.91	included	present	652.7	7.12
224501_at	excluded	absent	115.4	0.16	excluded	absent	96	-0.01	excluded	absent	18.02	-0.69	excluded	absent	67.08	-0.26	excluded	absent	9.74	-0.76	excluded	absent	25.66	-0.62	excluded	absent	124.48	0.24	excluded	absent	136.7	0.35	excluded	absent	29.63	-0.58	included	present	265.87	1.47	excluded	absent	107.79	0.1	excluded	absent	20.38	-0.67	excluded	absent	17.45	-0.69	excluded	absent	107.31	0.09	excluded	absent	111.52	0.13
224502_s_at	included	present	2129.77	-0.31	included	present	2006.18	-0.43	included	present	2650.54	0.18	included	present	1919.31	-0.51	included	present	2044.22	-0.39	included	present	1659.99	-0.76	included	present	2273.54	-0.18	included	present	2090.15	-0.35	included	present	1647.95	-0.77	included	present	2075.53	-0.36	included	present	1754.48	-0.67	included	present	2307.16	-0.15	included	present	3352.65	0.84	included	present	1896.14	-0.53	included	present	2228.05	-0.22
224503_s_at	included	present	201.92	-0.43	included	absent	131.18	-1.03	included	present	45.71	-1.75	included	present	305.07	0.44	included	present	275.89	0.2	included	present	227.74	-0.21	included	present	149.04	-0.88	included	present	175.69	-0.65	included	present	160.78	-0.78	included	present	202.8	-0.42	included	present	210.18	-0.36	included	present	244.1	-0.07	included	absent	59.59	-1.63	included	present	101.53	-1.28	included	present	181.42	-0.6
224504_s_at	included	present	908.69	-1.17	included	present	2906.81	1.33	included	present	2252.54	0.51	included	present	2499.49	0.82	included	present	1208.21	-0.79	included	present	1406.92	-0.54	included	present	782.76	-1.32	included	present	1567.74	-0.34	included	present	959.47	-1.1	included	present	1026.36	-1.02	included	present	882.06	-1.2	included	present	1067.78	-0.97	included	present	1148.6	-0.87	included	present	827.29	-1.27	included	present	892.76	-1.19
224505_s_at	included	present	277.36	-0.21	included	present	337.13	-0.15	included	present	576.55	0.06	included	present	231.7	-0.25	included	present	487.06	-0.02	included	present	299.29	-0.19	included	present	372.6	-0.12	included	present	1633.67	1.01	included	present	564.86	0.05	included	present	1199	0.62	included	present	591.75	0.07	included	present	452.76	-0.05	included	present	243.69	-0.24	included	present	673.68	0.15	included	present	186.31	-0.29
224506_s_at	excluded	absent	226.39	-0.06	excluded	absent	31.9	-0.75	excluded	absent	82.19	-0.57	excluded	absent	47.9	-0.69	excluded	absent	190.45	-0.19	included	present	408.58	0.57	included	present	342.15	0.34	included	present	424.59	0.63	included	present	339.15	0.33	included	present	659.17	1.45	excluded	absent	405.14	0.56	excluded	absent	202.85	-0.15	excluded	absent	213.2	-0.11	excluded	absent	76.76	-0.59	included	present	356.94	0.39
224507_s_at	excluded	absent	11.85	-0.54	excluded	absent	9.03	-0.57	excluded	absent	98.04	0.3	included	present	163.72	0.93	excluded	absent	5.45	-0.6	excluded	absent	85.58	0.18	excluded	absent	19.97	-0.46	excluded	absent	2.22	-0.63	excluded	absent	67.9	0	excluded	absent	6.83	-0.59	excluded	absent	16.35	-0.5	excluded	absent	6.64	-0.59	excluded	absent	3.71	-0.62	excluded	absent	2.43	-0.63	excluded	absent	2.36	-0.63
224508_at	excluded	absent	2.34	-0.56	excluded	absent	8.37	-0.49	excluded	absent	56.59	0.08	included	present	155.86	1.27	excluded	absent	48.32	-0.01	excluded	absent	91.05	0.5	excluded	absent	7.23	-0.5	excluded	absent	8.66	-0.49	excluded	absent	22.99	-0.32	excluded	absent	4.32	-0.54	excluded	absent	4.02	-0.54	excluded	absent	4.26	-0.54	excluded	absent	1.95	-0.57	excluded	absent	2.04	-0.57	excluded	absent	29.48	-0.24
224509_s_at	included	present	134.22	-1.11	included	present	403.04	1.2	included	present	184.72	-0.68	included	present	183.65	-0.68	included	present	324.42	0.52	included	absent	109.77	-1.32	excluded	absent	280.53	0.15	included	present	218.64	-0.38	included	present	178.24	-0.73	included	present	367.39	0.89	included	present	234.78	-0.25	included	present	199.99	-0.54	included	present	225.25	-0.33	included	present	184.92	-0.67	included	present	229.2	-0.29
224510_s_at	excluded	absent	13.11	-1.03	included	present	191.54	3.14	included	present	162.56	2.46	excluded	absent	3.81	-1.25	excluded	absent	87.66	0.71	excluded	absent	112.59	1.29	included	present	150.24	2.18	included	present	764.99	16.57	included	present	160.55	2.42	included	present	301.27	5.71	included	present	259.01	4.72	excluded	absent	8.82	-1.13	excluded	absent	48.98	-0.19	excluded	absent	47.06	-0.24	included	present	352.15	6.9
224511_s_at	included	present	528.52	-0.72	included	present	2192.94	1.38	included	present	1163.1	0.08	included	present	699.35	-0.51	included	present	1768.8	0.84	included	present	1467.3	0.46	included	present	720.37	-0.48	included	present	2092.33	1.25	included	present	688.76	-0.52	included	present	815.66	-0.36	included	present	1002.49	-0.12	included	present	2066.28	1.22	included	present	1572.96	0.6	included	present	3495.49	3.03	included	present	974.83	-0.16
224512_s_at	included	present	615.27	0.2	included	present	656.03	0.41	included	present	454.63	-0.6	included	present	470.55	-0.53	included	present	759.64	0.93	included	present	960	1.93	included	present	927.97	1.77	included	present	1268.71	3.48	included	present	518.71	-0.28	included	present	887.33	1.57	included	present	1080.72	2.54	included	present	1057.6	2.42	included	present	114.86	-2.31	included	present	182.86	-1.97	included	present	2253.61	8.43
224513_s_at	included	present	1088.62	-0.43	included	present	2725.28	2.57	included	present	1224.05	-0.18	included	present	2265.12	1.73	included	present	1582.07	0.47	included	present	820.87	-0.92	included	present	1566.4	0.44	included	present	1255.83	-0.13	included	present	664.06	-1.21	included	present	1624.99	0.55	included	present	600.85	-1.33	included	present	783.72	-0.99	included	present	1572.21	0.46	included	present	1855.16	0.97	included	present	1045.98	-0.51
224514_x_at	included	present	231.23	0.21	excluded	absent	136.31	-0.67	excluded	absent	99.67	-1.02	included	present	149.8	-0.55	excluded	absent	11.57	-1.84	included	present	409	1.87	excluded	absent	88.54	-1.12	included	present	245.65	0.35	included	present	316.96	1.01	included	present	154.02	-0.51	included	present	161.06	-0.44	included	present	393.04	1.72	excluded	absent	191.39	-0.16	included	present	142.96	-0.61	included	present	135.44	-0.68
224515_at	excluded	absent	153.17	1.05	excluded	absent	126.13	0.69	excluded	absent	157.72	1.11	excluded	absent	3.49	-0.92	excluded	absent	4.33	-0.91	excluded	absent	73.9	0.01	excluded	absent	88.95	0.21	excluded	absent	80.53	0.09	excluded	absent	138.14	0.85	excluded	absent	175.29	1.34	excluded	absent	87.45	0.19	excluded	absent	155.5	1.08	included	present	206.94	1.76	excluded	absent	2.34	-0.93	excluded	absent	81.71	0.11
224516_s_at	included	present	1437.29	0.93	included	present	354.02	-0.91	included	present	340.26	-0.94	included	present	898.31	0.01	included	present	1626.94	1.26	included	present	1545.47	1.12	included	present	1324.36	0.74	included	present	1167.07	0.47	included	present	1620.82	1.25	included	present	1326.56	0.74	included	present	2457.99	2.67	included	present	663.11	-0.39	included	present	300.11	-1.01	included	present	413.04	-0.81	included	present	2783.96	3.23
224517_at	included	present	1257.29	0.18	included	present	1950.99	1.46	included	present	1869.77	1.31	included	present	2185.19	1.89	included	present	2658.57	2.77	included	present	1797.9	1.18	included	present	2485.8	2.45	included	present	1531.2	0.68	included	present	1895.12	1.36	included	present	3906.44	5.08	included	present	9917.69	16.2	included	present	1713.67	1.02	included	present	1998.4	1.55	included	present	3035.97	3.47	included	present	1188.54	0.05
224518_s_at	included	present	495.94	-0.75	included	present	947.01	0.63	included	present	732.84	-0.02	included	present	652.64	-0.27	included	present	1026.02	0.88	included	present	661.17	-0.24	included	present	716.28	-0.08	included	present	634.96	-0.32	included	present	821.81	0.25	included	present	709.56	-0.1	included	present	473.38	-0.82	included	present	310.33	-1.32	included	present	1387.92	1.99	included	present	1259.96	1.59	included	present	564.04	-0.54
224519_at	included	present	474.9	0.58	included	present	174.74	-0.67	included	present	164.82	-0.71	included	present	516.91	0.76	included	present	505.35	0.71	included	present	190.55	-0.6	included	present	589.71	1.06	included	present	686.62	1.46	included	present	586.18	1.04	included	present	736.29	1.67	included	present	281.63	-0.22	included	present	270.59	-0.27	included	present	637.89	1.26	included	present	129.26	-0.86	included	present	594.88	1.08
224520_s_at	excluded	absent	59.33	-0.33	excluded	absent	66.18	-0.31	included	present	109.01	-0.16	included	present	538.26	1.33	excluded	absent	74.77	-0.28	included	present	266.52	0.39	included	present	68.31	-0.3	excluded	absent	53.35	-0.35	excluded	absent	3.98	-0.52	included	present	272.39	0.41	excluded	absent	81.38	-0.25	excluded	absent	96.9	-0.2	included	present	58.6	-0.33	excluded	absent	41.21	-0.39	excluded	absent	28.78	-0.44
224521_s_at	included	present	222.93	-0.95	included	present	511.37	2.07	included	present	198.89	-1.2	included	present	495.18	1.9	included	present	444.33	1.37	included	present	251.01	-0.65	included	present	278.76	-0.36	included	present	377.72	0.67	included	present	91.63	-2.32	included	present	168.19	-1.52	included	present	872.13	5.84	included	present	162.58	-1.58	included	present	478.74	1.73	included	present	693.83	3.98	included	present	283.68	-0.31
224522_s_at	included	present	885.68	2.49	included	present	566.73	0.74	included	present	675.56	1.34	included	present	334.06	-0.52	included	present	727.96	1.62	included	present	1138.96	3.87	included	present	4118.46	20.12	included	present	2839.21	13.14	included	present	2013.16	8.64	included	present	3357.51	15.97	included	present	3141.16	14.79	included	present	2502.45	11.31	included	present	600.4	0.93	included	present	1199.27	4.2	included	present	6419.88	32.68
224523_s_at	included	present	1516.92	-0.04	included	present	3185.48	1.74	included	present	3330.33	1.9	included	present	844.28	-0.76	included	present	1782.28	0.24	included	present	846.76	-0.76	included	present	1504.8	-0.05	included	present	1819.83	0.28	included	present	1048.5	-0.54	included	present	1343.04	-0.23	included	present	1320.79	-0.25	included	present	1980.2	0.45	included	present	2202.19	0.69	included	present	1460.83	-0.1	included	present	2713.81	1.24
224524_s_at	included	present	593.5	-0.45	included	present	805.75	0.17	included	present	835.06	0.26	included	present	720.6	-0.08	included	present	621.08	-0.37	included	present	305.07	-1.29	included	present	922.85	0.51	included	present	867.85	0.35	included	present	592.88	-0.45	included	present	938.16	0.56	included	present	925.44	0.52	included	present	392.98	-1.03	included	present	561.63	-0.54	included	present	561.02	-0.54	included	present	613.56	-0.39
224525_s_at	included	present	312	0.07	included	absent	96.46	-0.83	included	present	206.12	-0.37	included	present	304.55	0.03	included	present	141.13	-0.64	included	absent	15.6	-1.16	included	present	681.27	1.59	included	present	76.71	-0.91	included	present	397.86	0.42	included	present	449.81	0.64	included	present	376.59	0.33	included	present	351	0.23	included	present	523.38	0.94	included	present	262.4	-0.14	included	present	590.77	1.22
224526_at	excluded	absent	123.06	0.07	excluded	absent	25.68	-0.57	excluded	absent	74.97	-0.24	excluded	absent	8.17	-0.68	excluded	absent	66.92	-0.3	excluded	absent	204.71	0.6	excluded	absent	95.47	-0.11	excluded	absent	33.44	-0.52	included	present	61.52	-0.33	excluded	absent	16.86	-0.62	included	present	94.26	-0.12	excluded	absent	28.53	-0.55	excluded	absent	76.46	-0.23	excluded	absent	88.01	-0.16	excluded	absent	31.36	-0.53
224527_at	excluded	absent	48.68	-0.69	excluded	absent	39.97	-0.83	excluded	absent	133.09	0.68	excluded	absent	62.91	-0.46	excluded	absent	143.97	0.85	excluded	absent	167.83	1.24	excluded	absent	53.78	-0.61	excluded	absent	38.12	-0.86	excluded	absent	82.18	-0.15	excluded	absent	22.11	-1.12	excluded	absent	17.64	-1.19	excluded	absent	76.89	-0.23	excluded	absent	47.98	-0.7	excluded	absent	47.06	-0.72	excluded	absent	28.27	-1.02
224528_s_at	excluded	absent	35.97	2.39	excluded	absent	8.1	-0.15	excluded	absent	3.26	-0.59	excluded	absent	3.42	-0.58	excluded	absent	3.65	-0.56	excluded	absent	5.76	-0.37	excluded	absent	13.18	0.31	excluded	absent	6.07	-0.34	excluded	absent	20.61	0.99	excluded	absent	8.9	-0.08	excluded	absent	2.73	-0.64	excluded	absent	5.02	-0.43	excluded	absent	5.63	-0.38	excluded	absent	48.8	3.57	excluded	absent	1.12	-0.79
224529_s_at	excluded	absent	11.48	-1.21	excluded	absent	77.58	0.64	excluded	absent	6.35	-1.36	excluded	absent	64.73	0.28	excluded	absent	15.78	-1.09	excluded	absent	52.87	-0.05	excluded	absent	80.32	0.72	excluded	absent	70.44	0.44	included	present	69.98	0.43	included	present	340.47	8.04	excluded	absent	75.2	0.58	excluded	absent	50.63	-0.11	excluded	absent	30.92	-0.67	excluded	absent	32.22	-0.63	included	present	156.32	2.86
[illegible]																																																												

224846_at	excluded	absent	95.05	0.54	excluded	absent	206.89	2.69	excluded	absent	33.54
224847_at	included	present	1519.84	2.86	included	present	2638.11	6.1	included	present	10780.37
224848_at	included	present	393.73	2.77	included	present	178.57	0.16	included	present	2032.82
224849_at	included	present	1389.92	0.17	included	present	1623.14	0.59	included	present	2817.68
224850_at	included	present	1439.85	0.47	included	present	1408.77	0.39	included	present	1616.94
224851_at	included	present	2638.74	7.44	included	present	2181.23	5.8	included	present	8579.54
224852_at	included	present	459	0.39	included	present	339.32	-0.32	included	present	507.52
224853_at	included	present	2692.46	-0.03	included	present	2615.37	-0.13	included	present	2428.64
224854_s_at	included	present	2411.35	1.03	included	present	1612.25	-0.29	included	present	2007.32
224855_at	included	present	514.26	-1.54	included	present	772.55	-0.77	included	present	401.34
224856_at	included	present	1142.59	-0.54	included	present	932.65	-0.61	included	present	1951.56
224857_s_at	included	present	902.73	-0.41	included	present	1043.43	-0.21	included	present	764.94
224858_at	included	present	990.41	-0.42	included	present	514.82	-1.33	included	present	743.93
224859_at	excluded	absent	118.91	1	excluded	absent	127.78	1.15	excluded	absent	40.74
224860_at	included	present	321.58	-0.83	included	present	189.04	-1.38	included	present	1250.41
224861_at	included	present	539.67	-0.13	included	present	326.04	-0.74	included	present	536.26
224862_at	included	present	4238.52	0.29	included	present	2255.99	-0.94	included	present	5942.94
224863_at	included	present	3600.7	0.97	included	present	1925.5	-0.44	included	present	4363.9
224864_at	included	present	461	-0.55	included	present	731.57	-0.03	included	present	338.12
224865_at	included	present	939.18	0.48	included	present	1143.82	0.93	included	present	931.37
224866_at	included	present	1359.91	0.3	included	present	928.17	-0.61	included	present	1575.73
224867_at	included	present	8623.22	-1.05	included	present	7381.15	-1.35	included	present	7105.8
224868_at	included	present	123.71	-0.31	included	present	128.3	-0.23	excluded	absent	38.8
224869_s_at	included	present	627.21	-0.58	included	present	1422.71	0.69	included	present	887.21
224870_at	included	absent	254.45	-1.38	included	present	843.79	1.22	included	present	608.81
224871_at	included	present	3230.97	1.89	included	present	1030.98	-0.98	included	present	310.86
224872_at	included	present	519.86	-0.14	included	present	641.27	0.14	included	present	422.89
224873_s_at	included	present	869.81	-0.93	included	present	1057.02	-0.11	included	present	768.9
224874_at	included	present	339.98	0.21	included	present	274.95	-0.27	included	present	274.38
224875_at	included	present	140.37	-0.08	included	present	136.36	-0.15	included	present	299.91
224876_at	included	present	841.15	-0.84	included	present	923.69	-0.58	included	present	2058.92
224877_s_at	excluded	absent	92.05	-1.47	excluded	absent	323.6	-0.01	excluded	absent	139.56
224878_at	included	present	1128.09	1.84	included	present	1250.91	2.38	included	present	1179.85
224879_at	included	present	2000.06	1.29	included	present	1549.7	0.09	included	present	3216.44

225206_s_at	included	present	994.08	0.19	included	present	661.03	-0.84	included	present	1417.6
225207_at	included	present	3153.41	-0.45	included	present	1093.29	-0.62	included	present	480.94
225208_s_at	included	present	1250.43	-0.12	included	present	2405.84	2.23	included	present	1469.75
225209_s_at	included	present	679.3	-0.55	included	present	1255.61	0.57	included	present	274.72
225210_s_at	included	present	1194.2	-0.19	included	present	1830.1	1.91	included	present	1778.98
225211_at	included	absent	184.73	-0.2	included	present	262.61	0.55	included	absent	138.19
225212_at	included	present	233.06	-0.32	excluded	absent	124.66	-0.68	excluded	absent	289.61
225213_at	included	present	2100.26	-0.46	included	present	2671.36	-0.28	included	present	2383.85
225214_at	excluded	absent	357.67	1.06	excluded	absent	118.89	-0.65	excluded	absent	143.2
225215_s_at	included	present	2418.57	-0.38	included	present	1584.53	-1.31	included	present	3924.11
225216_at	included	present	796.12	-0.69	included	present	404.51	-1.42	included	present	882.97
225217_s_at	included	present	287.97	0.26	included	present	218.78	-0.45	included	absent	107.2
225218_at	included	present	514.46	-0.77	included	present	1619.74	3.7	included	present	565.05
225219_at	included	present	2374.81	1.04	included	present	410.17	-1.49	included	present	2393.53
225220_at	included	present	184.99	-1.64	included	present	657.28	0.32	included	present	834.25
225221_at	included	present	3364.34	-0.49	included	present	5396.57	1.01	included	present	4501
225222_at	included	present	487.89	-1.53	included	present	1019.12	0.96	included	present	451.98
225223_at	included	present	1081.47	0.15	included	present	523.47	-0.92	included	present	1159.35
225224_at	included	present	612.39	-0.08	included	present	1564.04	2.87	included	present	776.69
225225_at	included	present	2177.06	-0.47	included	present	1180.99	-1.07	included	present	3503.21
225226_at	included	present	686.01	0.57	included	present	1280.74	3.58	included	present	404.94
225227_at	included	present	840.7	-0.92	included	present	1969.68	1.04	included	present	1208.88
225228_at	included	present	1196.06	0.33	included	present	1032.12	0.01	included	present	682.65
225229_at	included	present	2156.37	-1.4	included	present	3161.36	-0.3	included	present	3241.61
225230_at	included	present	1619.28	1.01	included	present	874.75	-0.14	included	present	1209.83
225231_at	included	present	764.51	1.11	included	present	551.34	0.2	included	present	604.95
225232_at	included	present	824.91	-1.15	included	present	857.55	-1.11	included	present	1086.98
225233_at	included	present	351.21	-0.52	included	present	244.91	-0.79	included	present	1016.84
225234_at	included	present	1070.22	1.17	included	present	1657.66	3.16	included	present	1263.69
225235_at	included	present	396.92	0.1	included	present	1203.07	5.66	included	present	494.3
225236_at	included	present	246.05	-0.18	included	present	315.31	0.5	included	present	432.33
225237_s_at	included	present	293.84	-0.83	included	present	476.34	-0.48	included	present	673.03
225238_at	included	present	1452.84	-0.7	included	present	1468.66	-0.69	included	present	5172.01
225239_at	included	present	10996.37	0.3	included	present	1622.83	-0.98	included	present	6709.74

225565_at	included	present	979.54	1.15	included	present	898.63	0.84	[illegible]
225566_at	included	present	1075.47	7.33	included	present	635.65	3.8	[illegible]
225567_at	included	present	1661.96	0.18	included	present	1360.09	-0.13	[illegible]
225568_at	excluded	absent	260.89	-0.29	excluded	absent	165.02	-0.76	[illegible]
225569_at	included	present	1759.46	-0.17	included	present	1900.08	0	[illegible]
225570_at	included	present	231.64	-0.33	included	absent	119.18	-0.67	[illegible]
225571_at	included	present	3239.19	-0.31	included	present	2837.54	-0.52	[illegible]
225572_at	included	present	1318.47	0.22	included	present	1208.58	-0.05	[illegible]
225573_at	included	present	2016	-0.61	included	present	1097.75	-0.87	[illegible]
225574_at	included	present	985.69	-0.49	included	present	2485.24	0.98	[illegible]
225575_at	included	present	1910.29	-0.37	included	present	1649.32	-0.55	[illegible]
225576_at	included	present	2171.75	0.06	included	present	465.08	-2.04	[illegible]
225577_at	included	present	531.62	0.94	included	present	1164.59	5.07	[illegible]
225578_at	included	present	327.26	-0.68	included	present	1461.45	0.86	[illegible]
225579_at	included	present	2464.83	2.05	included	present	282.9	-1.11	[illegible]
225580_at	included	present	130.72	-0.95	included	absent	81.26	-1.9	[illegible]
225581_s_at	included	present	452.76	-1.34	included	present	355.79	-1.67	[illegible]
225582_at	included	present	270.96	-0.39	included	absent	280.89	-0.36	[illegible]
225583_at	included	present	2459.96	4.52	included	present	1657.07	2.03	[illegible]
225584_at	included	present	376.57	0.17	included	present	614.19	1.88	[illegible]
225585_at	included	present	9473.28	0.23	included	present	14650.15	1.21	[illegible]
225586_at	excluded	absent	134.79	-1.13	included	present	482.23	4.13	[illegible]
225587_at	included	present	379.21	0.63	included	absent	223.42	-0.61	[illegible]
225588_s_at	included	present	1914.99	-1.38	included	present	3019.24	0.19	[illegible]
225589_at	included	present	179.05	-1.21	included	present	562.57	0.98	[illegible]
225590_at	excluded	absent	76.5	0.12	excluded	absent	109.29	0.78	[illegible]
225591_at	included	present	318.64	-0.36	included	present	271.04	-0.43	[illegible]
225592_at	included	present	190.01	1.39	included	present	320.89	3.22	[illegible]
225593_at	included	present	216.33	0.32	included	present	276.09	1	[illegible]
225594_at	included	present	1667.7	-1.12	included	present	2724.56	-0.11	[illegible]
225595_at	included	present	409.86	-0.63	included	present	386.4	-0.71	[illegible]
225596_at	included	present	162.89	-0.36	included	present	344.32	0.5	[illegible]
225597_at	excluded	absent	89.87	0.4	excluded	absent	16.01	-0.88	[illegible]
225598_at	included	present	505.31	-0.29	included	present	142.79	-1.2	[illegible]
[illegible]	[illegible]	[illegible]	[illegible]	[illegible]	[illegible]	[illegible]	[illegible]	[illegible]	[illegible]

225924_at	included	present	1067.51	1.73	included	present	1029.46	1.58	included	present	634.65	0.08	included	present	766.07	0.58	included	present	1436.28	3.13	included	present	971.19	1.36	included	present	1946.57	5.08	included	present	2251.07	6.24	included	present
225925_s_at	included	present	904.35	-0.6	included	present	1078.93	-0.1	included	present	646.54	-1.35	included	present	655.73	-1.32	included	present	853.25	-0.75	included	present	1301.54	0.55	included	present	627.06	-1.41	included	present	873.24	-0.69	included	present
225926_at	included	present	313.6	-0.17	included	present	449.95	1.21	included	present	154.7	-1.78	included	present	192.91	-1.39	included	present	282.19	-0.49	included	present	263.51	-0.68	included	present	454.63	1.25	included	present	556.25	2.28	included	present
225927_at	included	present	1725.16	0.3	included	present	764.17	-0.56	included	present	1721.29	0.3	included	present	1512.75	0.11	included	present	1166.47	-0.2	included	present	1902.3	0.46	included	present	558.71	-0.74	included	present	2269.08	0.79	included	present
225928_at	included	present	403.41	-0.4	included	present	542.18	0.35	included	present	374.31	-0.56	included	present	320.09	-0.86	included	present	459.19	-0.1	included	present	269.19	-1.13	included	present	629.41	0.83	included	present	665.23	1.02	included	present
225929_s_at	included	present	1113.72	1.06	included	present	632.52	-0.04	included	present	421.01	-0.53	included	present	612.33	-0.09	included	present	579.42	-0.16	included	present	2282.27	3.74	included	present	528.07	-0.28	included	present	741.72	0.21	included	present
225930_at	included	present	1362.04	-0.4	included	present	1250.04	-0.51	included	present	1314.48	-0.45	included	present	1019.43	-0.73	included	present	692.85	-1.05	included	present	806.86	-0.94	included	present	1641	-0.13	included	present	1504.79	-0.26	included	present
225931_s_at	included	present	2052.51	0.88	included	present	531.12	-0.81	included	present	1073.76	-0.21	included	present	1498.8	0.27	included	present	976.99	-0.31	included	present	2083.58	0.92	included	present	499.52	-0.84	included	present	951.36	-0.34	included	present
225932_s_at	included	present	7057.59	-0.72	included	present	6701.07	-0.84	included	present	10421.23	0.41	included	present	11587.46	0.81	included	present	17716.69	2.87	included	present	3518.67	-1.91	included	present	5192.92	-1.35	included	present	4255.79	-1.66	included	present
225933_at	included	present	160.81	0.64	included	present	541.19	6.88	included	present	235.59	1.87	included	present	190.09	1.12	included	present	191.39	1.14	excluded	absent	154.34	0.54	included	present	347.02	3.69	included	present	438.61	5.2	included	present
225934_at	included	present	1353.52	1.04	included	present	914.19	-0.18	included	present	417.74	-1.57	included	present	323.44	-1.83	included	present	1382.03	1.12	included	present	1455.08	1.32	included	present	1555.43	1.6	included	present	3401.08	6.75	included	present
225935_at	included	present	888.86	0.25	included	present	481.54	-0.4	included	present	724.75	-0.01	included	present	1272.88	0.85	included	present	901.52	0.27	included	present	1781.95	1.66	included	present	1852.74	1.77	included	present	1126.31	0.62	included	present
225936_at	included	present	675.25	0.47	included	present	1225.01	3.08	included	present	783.72	0.99	included	present	1207.62	2.99	included	present	428.93	-0.69	included	present	598.26	0.11	included	present	869.76	1.39	included	present	906.03	1.57	included	present
225937_at	included	present	880.21	0.39	included	present	332.71	-0.56	included	present	797.83	0.25	included	present	773.93	0.21	included	present	737.49	0.14	included	present	625.61	-0.05	included	present	938.9	0.49	included	present	722.67	0.12	included	present
225939_at	included	present	408.85	0.07	included	present	279.8	-0.56	included	present	169.19	-1.1	included	present	171.7	-1.09	included	present	529.29	0.66	included	present	353.17	-0.2	included	present	827.03	2.12	included	present	282.59	-0.54	included	present
225940_at	included	present	1498.76	-0.38	included	present	1355.24	-0.55	included	present	1553.13	-0.31	included	present	801.88	-1.23	included	present	1736.8	-0.09	included	present	635.8	-1.44	included	present	967.58	-1.03	included	present	1054.46	-0.92	included	present
225941_at	included	present	1580.42	-0.14	included	present	260.61	-2.02	included	present	854.49	-1.18	included	present	592.18	-1.55	included	present	1618.88	-0.09	included	present	356.73	-1.88	included	present	1128.98	-0.78	included	present	851.97	-1.18	included	present
225942_at	included	present	389.6	1.37	included	present	1755.69	10.71	included	present	1196.34	6.88	included	present	851.6	4.52	included	present	1087.46	6.14	included	absent	83.23	-0.73	included	present	473.52	1.94	included	present	117.11	-0.5	included	present
225943_at	included	present	224.2	-0.41	included	present	829.09	3.41	included	present	496.88	1.32	included	present	630.35	2.16	included	present	499.95	1.33	included	present	224.91	-0.4	included	present	250.79	-0.24	included	present	214.75	-0.47	included	present
225944_at	included	present	662.57	1.52	included	present	1348.59	4.68	included	present	1834.6	6.92	included	present	1027.82	3.2	included	present	661.37	1.51	included	present	279.19	-0.25	included	present	533.53	0.92	included	present	295.97	-0.17	included	present
225945_at	included	present	595.2	-1.07	included	present	974.67	0.38	included	present	757.6	-0.45	included	present	1260.47	1.47	included	present	1500.02	2.39	included	present	770.37	-0.4	included	present	1945	4.1	included	present	1402.74	2.02	included	present
225946_at	included	present	968.87	-0.13	included	present	129.76	-1.34	included	absent	131.78	-1.34	included	absent	47.92	-1.46	included	present	187.48	-1.26	included	present	829.66	-0.33	included	present	259.91	-1.15	included	present	154.69	-1.3	included	present
225947_at	included	present	311.32	0.28	included	present	798.65	3.62	included	present	246.64	-0.17	included	present	652.22	2.61	excluded	absent	336.47	0.45	included	present	546.1	1.89	included	present	525.03	1.74	included	present	2235.38	13.47	included	present
225948_at	included	present	450.9	0.31	included	present	556.72	0.74	included	present	272.89	-0.4	included	present	466.08	0.37	included	present	273.07	-0.4	included	present	577.09	0.82	included	present	887.56	2.07	included	present	458.3	0.34	included	present
225949_at	included	present	1610.87	0.38	included	present	626.01	-0.66	included	present	402.39	-0.9	included	present	213.44	-1.1	included	present	1006.28	-0.26	included	present	1461.9	0.22	included	present	2585.35	1.4	included	present	1501.29	0.26	included	present
225950_at	included	present	1616.39	-0.11	included	present	556.05	-1.45	included	present	696.78	-1.27	included	present	441.78	-1.6	included	present	660.92	-1.32	included	present	933.84	-0.97	included	present	1487.16	-0.28	included	present	756.74	-1.2	included	present
225951_s_at	included	present	3971.23	-1.11	included	present	7099.25	0.13	included	present	6720.69	-0.02	included	present	5869	-0.36	included	present	6199.91	-0.23	included	present	2784.67	-1.58	included	present	5071.15	-0.67	included	present	4322.41	-0.97	included	present
225952_at	excluded	absent	4.01	-1.09	excluded	absent	13	-0.85	excluded	absent	7.79	-0.99	excluded	absent	67.12	0.61	excluded	absent	4.12	-1.09	excluded	absent	84.28	1.08	excluded	absent	19.78	-0.67	excluded	absent	126.87	2.23	excluded	absent
225953_at	included	present	938.74	-0.33	included	present	1210.36	0.19	included	present	1101.1	-0.02	included	present	2381.35	2.44	included	present	1073.78	-0.07	included	present	1156.7	0.09	included	present	2221.76	2.14	included	present	1425.45	0.6	included	present
225954_s_at	included	absent	177.06	-0.32	included	present	425.37	0.5	included	absent	269.3	-0.02	included	present	195.29	-0.26	included	absent	216.92	-0.19	included	present	343.44	0.23	included	present	236.14	-0.13	included	present	167.98	-0.35	included	present
225955_at	included	present	91.75	-0.56	excluded	absent	61.93	-0.86	excluded	absent	54.3	-0.94	excluded	absent	88.98	-0.59	excluded	absent	193.41	0.48	included	present	405.86	2.64	included	present	201.49	0.56	included	present	271.4	1.27	included	present
225956_at	included	present	964.27	0.26	included	present	157.29	-2.5	included	present	779.31	-0.38	included	present	968.59	0.27	included	present	448.91	-1.5	included	present	460.63	-1.46	included	present	432.92	-1.56	included	present	355.83	-1.82	included	present
225957_at	included	present	277.4	-0.45	included	absent	76.8	-2.33	included	present	335.57	0.1	included	present	353.33	0.27	included	present	219.89	-0.99	included	present	347.93	0.22	included	present	396.45	0.67	included	present	291.57	-0.31	included	present
225958_at	included	present	1021.32	0.16	included	present	2118.96	3.89	included	present	936.48	-0.13	included	present	1927.78	3.24	included	present	1631.82	2.23	included	present	955.6	-0.06	included	present	1508.37	1.81	included	present	3075.87	7.14	included	present
225959_s_at	included	present	558.36	-0.03	included	present	1685.6	4.19	included	present	1399.08	3.11	included	present	999.54	1.62	included	present	753.76	0.7	included	present	700.04	0.5	included	present	1066.95	1.87	included	present	1571.32	3.76	included	present
225960_at	excluded	absent	69.77	0.36	excluded	absent	73.61	0.45	excluded	absent	84.46	0.71	excluded	absent	27.19	-0.69	excluded	absent	66.98	0.29	excluded	absent	90.9	0.87	excluded	absent	88.14	0.8	excluded	absent	31.17	-0.59	excluded	absent
225961_at	included	present	10908.48	1.3	included	present	10236.76	1.09	included	present	14037.93	2.29	included	present	11756.69	1.57	included	present	12712.59	1.87	included	present	5298.29	-0.47	included	present	7143.82	0.11	included	present	5511.76	-0.41	included	present
225962_at	included	present	148.38	-1.03	included	present	2383.44	10.01	included	present	385.78	0.15	included	present	492.22	0.67	included	present	378.42	0.11	included	present	241.47	-0.57	included	present	742.15	1.9	included	present	670.06	1.55	included	present
225963_at	included	present	449.47	1.2	included	present	408.49	0.82	included	present	408.4	0.82	included	present	471.71	1.4	included	present	420.28	0.93	included	present	262.88	-0.51	included	present	671.15	3.22	included	present	659.72	3.11	included	present
225964_at	included	present	759.4	0.42	included	present	816.53	0.79	included	present	843.44	0.96	included	present	844.12	0.97	included	present	592.17	-0.66	included	present	450.38	-1.57	included	present	718.58	0.16	included	present	718	0.15	included	present
225965_at	included	present	525.19	-0.02	included	present	360.05	-0.46	included	present	346.07	-0.5	included	present	634.81	0.27	included	present	604.79	0.19	included	present	416.2	-0.31	included	present	292.79	-0.64	included	present	494.18	-0.1	included	present
225966_at	excluded	absent	67.65	0.03	excluded	absent	60.47	-0.11	excluded	absent	73.42	0.14	excluded	absent	10.08	-1.11	excluded	absent	16.8	-0.98	excluded	absent	14.91	-1.02	excluded	absent	30.34	-0.71	excluded	absent	71.55	0.11	excluded	absent
225967_s_at	included	present	2129.91	-0.99	included	present	7078.84	1.68	included	present	4913.01	0.51	included	present	3547.19	-0.22	included	present	2766.18	-0.64	included	present	3301.74	-0.36	included	present	2222.49	-0.94	included	present	5577.29	0.87	included	present
225968_at	included	present	1386.78	0.23	included	present	219.97	-1.04	included	present	134.6	-1.13	included	present	340.12	-0.91	included	present	202.12	-1.06	included	present	1205.2	0.04	included	present	443.02	-0.8	included	present	161.62	-1.1	included	present
225969_at	included	present	683.81	1.39	included	present	760.83	1.8	included	absent	336.38	-0.49	included	present	562.08	0.73	included	present	502.96	0.41	included	present	335.06	-0.5	included	present	530.93	0.56	included	present	531.74	0.57	included	present
225970_at	included	present	968.81	0.99	included	present	458.11	-0.4	included	present	652.91	0.13	included	present	1137.05	1.45	included	present	745.01	0.38	included	present	384.53	-0.6	included	present	449.93	-0.42	included	present	822.29	0.59	included	present
225971_at	included	present	290.57	-0.68	included	present	375.67	-0.37	included	present	308.78	-0.61	included	present	791.63	1.12	included	present	253.48	-0.81	included	present	382.35	-0.35	included	present	456.21	-0.08	included	present	556.27	0.27	included	present
225972_at	included	present	559	0.26	included	present	354.97	-0.93	included	present	368.84	-0.85	included	present	314.33	-1.17	included	present	257.19	-1.5	included	present	370.04	-0.84	included	present	594.3	0.47	included	present	240.41	-1.6	included	present
225973_at	included	present	254.78	-0.5	included	present	204.28	-0.62	included	present	233.74	-0.55	included	present	292.15	-0.4	included	absent	124.6	-0.81	included	present	756.34	0.73	included	present	144.63	-0.77	included	present	787.21	0.81	included	present
225974_at	included	present	499.74	-0.17	included	present	291.56	-1.05	included	present	426.37	-0.48	included	present	200.83	-1.43	included	present	320.41	-0.93	included	present	549.93	0.04	included	present	593.27	0.23	included	present	498.58	-0.17	included	present
225975_at	included	present	1013.63	-0.15	included	present	310.95	-0.6	included	present	165.51	-0.69	included	present	1309.23	0.04	included	present	494.53	-0.48	included	present	2851.75	1.02	included	present	815.37	-0.28	included	present	140.1	-0.71	included	present
225976_at	included	present	1146.42	-0.42	included	present	2648.53	3.14	included	present	1997	1.6	included	present	3285.46	4.65	included	present	1609.17	0.68	included	present	1116.46	-0.49	included	present	2985.66	3.94	included	present	2487.82	2.76	included	present
225977_at	included	present	158.72	0.01	excluded	absent	13.61	-0.9	excluded	absent	3.82	-0.96	excluded	absent	139.21	-0.11	excluded	absent	27.94	-0.81	excluded	absent	386.5	1.42	excluded	absent	265.06	0.67	excluded	absent	8.27	-0.93	included	present
225978_at	included	present	2298.74	-1.04	included	present	5516.15	0.92	included	present	3783.53	-0.14	included	present	3243.73	-0.47	included	present	531.56	-2.12	included	present	3209.18	-0.49	included	present	1712.99	-1.4	included	present	2403.94	-0.98	included	present
225979_at	excluded	absent	2.49	-0.72	excluded	absent	3.37	-0.68	excluded	absent	1.95	-0.74	excluded	absent	2.26	-0.73	excluded	absent	13.1	-0.32	excluded	absent	101.22	3	excluded	absent	50.76	1.1	excluded	absent	30.26	0.33	excluded	absent
225980_at	included	present	452.66	-0.01	included	present	261.81	-0.87	included	present	185.52	-1.21	included	present	743.33	1.3	included	present	274.89	-0.81	included	present	684.2	1.04	included	present	1137.96	3.08	included	present	823.31	1.66	included	present
225981_at	included	present	136.48	0.06	included	present	265.8	1.45	excluded	absent	119.13	-0.13	included	present	167.92	0.4	included	present	114.54	-0.17	included	present	185.53	0.59	included	present	788.65	7.06	included	present	374.47	2.62	included	present
225982_at	included	present	149.64	-0.92	included	present	499.75	1.04	included	present	512.51	1.11	included	present	437.69	0.7	included	present	431.13	0.66	included	present	788.87	2.66	included	present	927.92	3.44	included	present	1027.89	4	included	present
225983_s_at	included	present	397.92	5.36	excluded	absent	52.14	-0.13	excluded	absent	70.36	0.16	excluded	absent	53.32	-0.11	excluded	absent	6.19	-0.86	excluded	absent	173.67	1.8	excluded	absent	54.02	-0.1	excluded	absent	42.62	-0.28	excluded	absent
225984_at	included	present	335.58	-0.91	included	present	524.96	-0.38	included	present	298.87	-1.01	included	present	207.29	-1.27	included	present	322.35	-0.95	included	present	1004.96	0.98	included	present	518.33	-0.39	included	present	348.04	-0.87	included	present
225985_at	included	present	374.8	-0.67	included	absent	316.61	-0.9	included	present	690.21	0.6	included	present	362.3	-0.72	included	present	304.91	-0.95	included	present	629.41	0.35	included	present	418.13	-0.5	included	present	244.47	-1.19	included	present
225986_x_at	included	present	1354.23	-1.11	included	present	1679.9	-0.86	included	present	1634.12	-0.89	included	present	1356.93	-1.11	included	present	1613.33	-0.91	included	present	1576.05	-0.94	included	present	3659.65	0.69	included	present	1114.63	-1.3	included	present
225987_at	included	present	1002.7	-0.17	included	present	282.24	-0.47	included	absent	63.89	-0.56	included	absent	13.06	-0.58	included	present	1001.84	-0.17	included	present	7610	2.52	included	present	207.42	-0.5	included	present	81.73	-0.55	included	present
225988_at	included	present	1067.48	-0.77	included	absent	958.53	-1.05	included	present	929.15	-1.13	included	present	1046.88	-0.82	included	present	911.7	-1.17	included	present	1432.63	0.18	included	present	1029.22	-0.87	included	present	1431.63	0.17	included	present
225989_at	included	present	586.69	-0.8	included	present	378.26	-1.67	included	present	579.38	-0.83	included	present	370.87	-1.7	included	present	602.46	-0.73	included	present	342.64	-1.82	included	present	638.24	-0.58	included	present	550.14	-0.95	included	present
225990_at	included	present	426.89	-0.77	included	present	263.98	-0.83	included	absent	124.89	-0.88	included	present	235.79	-0.84	included	present	487.81	-0.75	included	present	3199.66	0.22	included	absent	115.45	-0.88	included	present	234.85	-0.84	included	present
225991_at	included	present	390.14	-0.36	included	present	490.55	0.07	included	present	308.82	-0.71	included	present	359	-0.5	included	present	217.75	-1.11	included	present	237.91	-1.02	included	present	242.35	-1	included	present	408.89	-0.28	included	present
225992_at	included	present	388.72	-0.57	included	present	541.17	-0.38	included	present	965.64	0.15	included	present	717.53	-0.16	included	present	620.99	-0.28	included	present	532.19	-0.39	included	present	690.64	-0.19	included	present	836.19	-0.01	included	present
225993_at	included	present	38.61	-1.52	included	present	147.42	0.55	included	present	199.32	1.53	excluded	absent	93.78	-0.47	excluded	absent	113.58	-0.09	excluded	absent	139.83	0.41	included	present	128.19	0.18	included	present	127.98	0.18	included	present
225994_at	included	present	325.77	0.04	included	present	184.01	-1.38	included	present	739.46	4.19	included	present	651.61	3.31	included	present	378.96	0.58	included	present	555.69	2.35	included	present	217.25	-1.04	included	present	808.73	4.88	included	present
225995_x_at	included	present	958.67	-0.17	included	present	1691.57	2.17	included	present	916.88	-0.31	included	present	1151.48	0.44	included	present	1215.34	0.65	included	present	1630.07	1.98	included	present	1485.82	1.51	included	present	1133.4	0.39	included	present
225996_at	included	present	1097.34	-0.62	included	present	561.38	-0.85	included	present	979.15	-0.67	included	present	638.52	-0.81	included	present	1128.38	-0.61	included	absent	194.35	-1	included	present	3544.37	0.42	included	present	849.1	-0.72	included	present
225997_at	included	present	1456.97	-0.51	included	present	3343.28	1.39	included	present	3398.58	1.45	included	present	1745.81	-0.22	included	present	1490.47	-0.48	included	present	1283.51	-0.69	included	present	1354.68	-0.62	included	present	1096.35	-0.88	included	present
225998_at	included	present	2598.74	1.68	included	present	377.62	-1.02	included	present	435.3	-0.95	included	present	654.08	-0.68	included	present	708.12	-0.62	included	present	554.62	-0.8	included	present	333.8	-1.07	included	present	321.06	-1.09	included	present
225999_at	included	present	437.38	-0.72	included	present	631.49	-0.18	included	present	659.69	-0.1	included	present	529.46	-0.46	included	present	188.17	-1.41	included	present	472.01	-0.62	included	present	229.13	-1.29	included	present	336.87	-1	included	present
226000_at	included	present	917.23	-0.58	included	present	1793.48	0.78	included	present	1253.55	-0.06	included	present	360.06	-1.45	included	present	961.99	-0.51	included	present	1039.47	-0.39	included	present	1256.84	-0.05	included	present	1191.91	-0.16	included	present
226001_at	included	present	529.69	0.32	included	present	258.51	-1.05	included	present	179.46	-1.45	included	present	136	-1.67	included	present	491.9	0.13	included	present	433.38	-0.16	included	present	226.16	-1.21	included	present	645.89	0.91	included	present
226002_at	included	present	479.49	0.65	included	absent	189.49	-0.76	included	absent	109.91	-1.15	included	present	33.63	-1.52	included	absent	212.4	-0.65	included	present	281.89	-0.31	included	present	164.91	-0.88	included	present	195.5	-0.74	included	present
226003_at	included	present	7133.52	2.81	included	present	6684.79	2.54	included	present	12418.51	5.91	included	present	1175.02	-0.69	included	present	11792	5.55	included	present	621.03	-1.02	included	present	7889.31	3.25	included	present	3538.55	0.7	included	present
226004_at	included	present	144.54	-0.43	included	present	192.97	-0.28	included	present	129.22	-0.48	included	present	435.37	0.47	included	present	330.92	0.15	included	present	256.38	-0.08	included	present	325.72	0.13	included	present	446.4	0.5	included	present
226005_at	included	present	838.98	-0.2	included	present	921.13	-0.08	included	present	857.68	-0.17	included	present	886.94	-0.13	included	present	1059.31	0.12	included	present	1224.78	0.36	included	present	1051.11	0.11	included	present	1076.19	0.14	included	present
226006_at	included	present	12054.01	-1.52	included	present	17378.88	-0.41	included	present	23628.63	0.9	included	present	19753	0.09	included	present	19607.31	0.06	included	present	8944.62	-2.16	included	present	8308.61	-2.3	included	present	9656.85	-2.02	included	present

[illegible]

226284_at	included	present	474.27	0.99	included	present	154.52	-1.3	included	present	411.61	0.54	included	present	184.15	-1.09
226285_at	included	present	1550.75	-0.16	included	present	2224.21	0.51	included	present	1534.28	-0.17	included	present	977.49	-0.72
226286_at	excluded	absent	39.5	-0.62	excluded	absent	73.81	0.04	excluded	absent	80.52	0.16	excluded	absent	7.93	-1.22
226287_at	included	present	214.88	-0.38	included	present	1301.03	0.83	included	present	633.6	0.08	included	present	1389.76	0.92
226288_s_at	included	present	1291.2	-0.33	included	present	3696.46	1.56	included	present	621.84	-0.86	included	present	419.8	-1.02
226289_at	included	present	293.49	0.14	included	present	93.77	-1.5	included	present	341.41	0.53	included	present	99.44	-1.46
226290_at	included	present	1300.17	-1.2	included	present	1247	-1.31	included	present	2901.02	2	included	present	4181.58	4.56
226291_at	included	present	904.44	0.67	included	present	1128.16	1.63	included	present	562.66	-0.79	included	present	789.1	0.18
226292_at	included	present	540.07	2.11	included	present	170.25	-0.08	included	present	146.42	-0.22	included	absent	11.96	-1.01
226293_at	included	present	792.39	2.26	included	present	1199.73	4.91	included	present	581.11	0.89	included	present	429.64	-0.1
226294_x_at	included	present	1032.03	-0.78	included	present	885.81	-1.16	included	present	1932.86	1.54	included	present	939.36	-1.02
226295_at	included	present	722.35	1.45	included	present	641.08	0.98	included	present	540.95	0.41	included	present	435.5	-0.2
226296_s_at	included	present	1917.37	-0.71	included	present	5735.14	0.71	included	present	2113.39	-0.63	included	present	2436.86	-0.51
226297_at	included	present	4429.05	0.48	included	present	1517.59	-1.05	included	present	1744.06	-0.93	included	present	3036.02	-0.25
226298_at	included	present	549.29	-1.11	included	present	1555.56	1.15	included	present	625.77	-0.94	included	present	1155.63	0.25
226299_at	excluded	absent	3.32	-0.78	excluded	absent	40.92	-0.19	excluded	absent	136.57	1.33	excluded	absent	3.66	-0.78
226300_at	included	present	607.15	0.33	included	present	1497.38	4.82	included	present	468.58	-0.37	included	present	487.08	-0.28
226301_at	included	present	261.37	-0.87	included	absent	95.66	-1.38	included	absent	98.08	-1.37	included	present	1075.09	1.63
226302_at	included	present	117.45	-0.41	included	absent	116.47	-0.41	included	absent	67.42	-0.47	included	present	2336.28	2.28
226303_at	included	present	156.49	-0.61	included	absent	146.81	-0.61	included	absent	5.06	-0.66	included	absent	23.64	-0.66
226304_at	included	present	1031.37	-0.41	included	present	739.32	-0.45	included	present	330.84	-0.5	included	present	716.88	-0.45
226305_at	included	present	1003.14	0.97	included	absent	112.09	-0.81	included	present	159.04	-0.71	included	absent	115.33	-0.8
226306_at	included	present	491.26	1.83	included	present	385.32	0.99	excluded	absent	155.91	-0.83	excluded	absent	133.64	-1
226307_at	included	present	168.22	-0.44	excluded	absent	145.59	-0.69	excluded	absent	130.59	-0.85	excluded	absent	200.89	-0.08
226308_at	excluded	absent	85.99	-0.4	included	present	158.97	0.21	included	present	323.02	1.6	included	present	805.06	5.66
226309_at	included	present	162.24	-0.35	included	present	172.1	-0.29	included	present	150.4	-0.43	included	present	308.23	0.56
226310_at	included	present	340.21	-0.93	included	present	231.04	-1.45	included	present	357.28	-0.84	included	present	876.24	1.64
226311_at	included	present	346.67	1.17	excluded	absent	41.63	-0.55	included	present	546.95	2.29	included	present	131.77	-0.04
226312_at	included	present	1178.89	-2.14	included	present	1714.78	-1.66	included	present	1398.82	-1.94	included	present	2535.08	-0.94
226313_at	included	present	2704.4	0.23	included	present	6884.29	2.19	included	present	4030.27	0.85	included	present	2849.93	0.3
226314_at	excluded	absent	245.49	0.86	included	present	234.88	0.77	excluded	absent	261.28	1	excluded	absent	117.84	-0.27
226315_at	included	present	719.47	0.48	included	present	1978.68	7.43	included	present	922.6	1.6	included	present	1089.75	2.52
226316_at	included	present	634.08	-0.13	included	present	441.54	-0.89	included	present	921.6	1.01	included	present	714.67	0.19
226317_at	included	present	1556.39	-0.56	included	present	655.48	-2.02	included	present	2397.32	0.81	included	present	1050.37	-1.38

226644_at	included	present	618.85	-0.65	included	present	807.59	-0.41	included	present	565.53	-0.72	included	present	685.81	-0.57	included	present	1523.5	0.5	included	present	471.4	-0.84	included	present	834	[illegible]
226645_at	excluded	absent	38.19	-1.11	excluded	absent	146.51	0.36	excluded	absent	87.63	-0.44	excluded	absent	99.3	-0.28	excluded	absent	42.87	-1.05	included	present	503	5.18	excluded	absent	76.23	[illegible]
226646_at	excluded	absent	115.54	-0.46	excluded	absent	8.22	-0.69	excluded	absent	48.54	-0.61	excluded	absent	35.47	-0.64	excluded	absent	194.74	-0.29	included	present	611.12	0.63	excluded	absent	44.16	[illegible]
226647_at	excluded	absent	61.57	-1.07	excluded	absent	147.12	-0.23	excluded	absent	63.73	-1.05	included	present	232.66	0.6	excluded	absent	138.99	-0.31	excluded	absent	80.88	-0.88	excluded	absent	77.35	[illegible]
226648_at	included	present	634.07	-0.75	included	present	892.51	0.01	included	present	750.77	-0.41	included	present	858.4	-0.09	included	present	485.83	-1.19	included	present	741.53	-0.43	included	present	679.92	[illegible]
226649_at	included	present	396.56	-0.3	included	present	1524.48	0.76	included	present	1897.71	1.1	included	present	1769.26	0.99	included	present	1578.13	0.81	included	present	205.98	-0.47	included	present	470.68	[illegible]
226650_at	included	present	1223.89	1.63	included	present	645.15	-0.49	included	present	581.5	-0.72	included	present	268.24	-1.87	included	present	562.23	-0.79	included	present	615.58	-0.6	included	present	1347.57	[illegible]
226651_at	included	present	393.27	-0.76	included	present	236.03	-0.99	included	absent	165.27	-1.09	included	absent	80.4	-1.21	included	present	275.32	-0.93	included	present	292.25	-0.91	included	present	321.26	[illegible]
226652_at	included	present	325.06	-0.91	included	present	612.43	0.19	included	present	553.05	-0.04	included	present	462.2	-0.38	included	present	397.65	-0.63	included	present	516.12	-0.18	included	present	551.21	[illegible]
226653_at	included	present	661.6	0.91	included	present	961.82	1.99	included	present	594.32	0.66	included	present	523.34	0.41	included	present	619.41	0.75	included	present	175.17	-0.85	included	present	973.71	[illegible]
226654_at	excluded	absent	4.76	-0.46	excluded	absent	92.33	0.99	excluded	absent	29.34	-0.05	excluded	absent	12.86	-0.32	excluded	absent	39.94	0.12	excluded	absent	60.89	0.47	excluded	absent	13.7	[illegible]
226655_at	included	present	970.19	1.16	included	present	260.47	-1.85	included	present	1195.28	2.12	included	present	1408.07	3.03	included	present	941.7	1.04	included	present	474.32	-0.94	included	present	617.38	[illegible]
226656_at	included	present	6338.84	-0.43	included	present	1938.81	-1.57	included	present	5500.14	-0.65	included	present	2796.6	-1.35	included	present	1298.41	-1.73	included	present	4385.41	-0.94	included	present	1766.36	[illegible]
226657_at	included	present	544.33	-0.36	included	present	269.28	-0.89	included	present	770.23	0.07	included	present	533.52	-0.38	included	present	1206.74	0.91	included	present	1349.85	1.19	included	present	926.21	[illegible]
226658_at	included	present	330.87	0.8	excluded	absent	4.99	-1.07	excluded	absent	2.27	-1.09	excluded	absent	53.85	-0.79	included	present	97.48	-0.54	included	present	603.28	2.37	excluded	absent	18.14	[illegible]
226659_at	included	present	123.22	-0.37	excluded	absent	154.48	-0.3	excluded	absent	37.79	-0.56	included	present	188.2	-0.22	excluded	absent	90.93	-0.44	included	present	602.62	0.7	included	present	173.93	[illegible]
226660_at	included	present	855.37	-0.66	included	present	954.26	-0.34	included	present	1460.03	1.31	included	present	1232.98	0.57	included	present	798.64	-0.84	included	present	730.99	-1.06	included	present	2389.66	[illegible]
226661_at	excluded	absent	85.72	-0.26	included	present	312.29	0.1	included	present	1249.51	1.62	included	present	583.92	0.54	included	present	924.58	1.09	excluded	absent	137.08	-0.18	included	present	90.4	[illegible]
226662_at	included	present	720.82	-0.34	included	absent	148.73	-2.28	included	present	941.17	0.41	included	present	1211.7	1.32	included	present	507.19	-1.06	included	present	564.17	-0.87	included	present	503.05	[illegible]
226663_at	included	present	2465.5	3.2	included	present	1227.73	0.76	included	present	3208.08	4.66	included	present	2656.86	3.57	included	present	1733.83	1.76	included	present	1147.62	0.61	included	present	788.82	[illegible]
226664_at	included	present	489.17	-0.32	included	present	381.54	-0.8	included	present	345.2	-0.95	included	present	362.37	-0.88	included	present	636.25	0.32	included	present	684.61	0.53	included	present	460.22	[illegible]
226665_at	included	present	639.23	-0.54	included	present	253.62	-1	included	present	1562.29	0.55	included	present	521.81	-0.68	included	present	597.96	-0.59	included	present	1073.47	-0.03	included	present	646.61	[illegible]
226666_at	included	present	534.58	-0.59	included	present	1224.72	0.67	included	present	364.72	-0.9	included	present	887.65	0.06	included	present	538.85	-0.58	included	present	554.24	-0.55	included	present	1651.71	[illegible]
226667_x_at	included	present	116.66	0.35	included	present	179.41	1.34	excluded	absent	115.17	0.33	excluded	absent	21.36	-1.15	excluded	absent	26.21	-1.07	included	present	144.87	0.8	excluded	absent	71.45	[illegible]
226668_at	included	present	674.78	-0.28	included	present	388.47	-1.55	included	present	648.08	-0.4	included	present	919.38	0.81	included	present	899.15	0.72	included	present	217.64	-2.31	included	present	830.03	[illegible]
226669_at	included	present	388.52	-0.13	included	present	450.67	0.17	included	present	459.82	0.21	included	present	760.76	1.64	included	present	392.35	-0.11	included	present	454.41	0.19	included	present	1070.75	[illegible]
226670_s_at	included	present	477.75	1.31	included	present	928.76	3.81	included	present	251.24	0.05	included	present	848.04	3.37	included	present	534.26	1.62	included	present	308.01	0.36	included	present	551.74	[illegible]
226671_at	included	present	1308.65	1.5	included	present	103.37	-1.1	included	present	174.24	-0.94	included	present	80.33	-1.14	included	absent	254.23	-0.77	included	present	236.16	-0.81	included	present	514.82	[illegible]
226672_s_at	excluded	absent	85.78	0.38	excluded	absent	99.93	0.65	excluded	absent	17.58	-0.92	excluded	absent	13.85	-0.99	excluded	absent	85.11	0.37	excluded	absent	114.64	0.93	excluded	absent	70.97	[illegible]
226673_at	excluded	absent	109.7	0.28	included	present	259.91	2.21	excluded	absent	229.7	1.82	excluded	absent	11.27	-0.99	excluded	absent	301.52	2.74	included	present	629.92	6.97	included	present	839.31	[illegible]
226674_at	included	present	228.59	0.18	included	absent	122.84	-0.65	included	absent	41.13	-1.28	included	absent	55.49	-1.17	included	absent	65.16	-1.09	included	present	232.69	0.21	included	present	507.66	[illegible]
226675_s_at	included	present	28717.87	-2.1	included	present	27041.1	-2.2	included	present	50495.14	-0.86	included	present	22029.53	-2.48	included	present	27222.88	-2.19	included	present	26153.2	-2.25	included	present	20667.32	[illegible]
226676_at	included	present	269.31	0.01	included	present	335.56	0.43	included	absent	39.12	-1.42	included	absent	4.11	-1.64	included	present	428.11	1	included	present	744.84	2.97	included	present	323.71	[illegible]
226677_at	included	present	369.38	0.34	included	present	472.04	0.87	included	absent	72.66	-1.21	included	absent	2.02	-1.58	included	present	230.54	-0.39	included	present	841.79	2.79	included	present	892.98	[illegible]
226678_at	excluded	absent	20.84	-0.3	excluded	absent	9.32	-0.62	excluded	absent	77.25	1.27	excluded	absent	16.17	-0.43	excluded	absent	14.76	-0.47	excluded	absent	80.46	1.36	excluded	absent	25.43	[illegible]
226679_at	included	present	1157.15	1.2	included	present	2540.12	4.47	included	present	443.71	-0.48	included	present	736.25	0.21	included	present	701.03	0.12	included	present	184.49	-1.1	included	present	2820.72	[illegible]
226680_at	included	present	464.3	-0.37	included	present	312.48	-1.1	included	present	413.03	-0.62	included	present	473.34	-0.33	included	present	403.4	-0.66	included	present	454.93	-0.41	included	present	908.9	[illegible]
226681_at	included	present	3086.82	1.87	included	present	1462.15	-0.55	included	present	1835.29	0.01	included	present	2242.06	0.61	included	present	2316.74	0.72	included	present	1857.85	0.04	included	present	3267.04	[illegible]
226682_at	included	present	294.43	-0.79	included	present	136.62	-0.91	included	present	439.47	-0.67	included	present	1107.36	-0.16	included	present	222.82	-0.84	included	present	684.55	-0.49	included	present	1011.59	[illegible]
226683_at	included	present	2747.63	-1.03	included	present	2490.66	-1.19	included	present	7229.07	1.85	included	present	2946.55	-0.9	included	present	4368.55	0.02	included	present	6947.71	1.67	included	present	5880.54	[illegible]
226684_at	included	present	1216.7	0.19	included	present	568.53	-1.19	included	present	719.44	-0.87	included	present	900.96	-0.48	included	present	749.54	-0.81	included	present	484.06	-1.37	included	present	646.04	[illegible]
226685_at	included	present	495.81	-0.33	included	present	270.64	-0.94	included	present	328.79	-0.78	included	present	271.52	-0.93	included	absent	197.98	-1.13	included	present	1078.27	1.26	included	present	289.91	[illegible]
226686_at	included	present	256.73	-0.36	included	present	369.94	0.38	included	present	334.66	0.15	included	present	673.43	2.36	included	present	445.58	0.88	included	present	487.49	1.15	included	present	635.08	[illegible]
226687_at	included	present	145.1	-1	included	present	179.12	-0.56	included	present	169.58	-0.68	included	present	251.14	0.38	included	absent	108.55	-1.47	included	present	131.43	-1.18	included	present	448.81	[illegible]
226688_at	included	present	359	-0.13	included	present	231.75	-1.1	included	present	419.19	0.33	included	present	618.78	1.86	included	present	293.42	-0.63	included	present	211.12	-1.26	included	present	247.72	[illegible]
226689_at	included	present	2430.04	-1.27	included	present	8101.88	1.86	included	present	6923.63	1.21	included	present	8860.29	2.28	included	present	4923.73	0.1	included	present	1847.79	-1.59	included	present	4213.47	[illegible]
226690_at	included	present	453.45	-0.43	included	present	3013.91	1.79	included	absent	89.27	-0.74	included	absent	5.6	-0.81	included	present	932.34	-0.01	included	absent	20.29	-0.8	included	present	1598.55	[illegible]
226691_at	included	present	1172.01	0.47	included	present	1115.08	0.32	included	present	757.26	-0.63	included	present	1204.79	0.55	included	present	997.57	0.01	included	present	1633.3	1.69	included	present	1891.11	[illegible]
226692_at	included	present	2335.73	0.84	included	present	1179.53	-1.15	included	present	1205.96	-1.1	included	present	1489.16	-0.62	included	present	1278.71	-0.98	included	present	1504.57	-0.59	included	present	2906.5	[illegible]
226693_at	included	present	2784.28	-0.57	included	present	2340.65	-0.98	included	present	3208.25	-0.19	included	present	2550.04	-0.79	included	present	3286.2	-0.12	included	present	1763.41	-1.5	included	present	1846.47	[illegible]
226694_at	included	present	3983.22	0.13	included	present	1037.88	-0.88	included	present	574.75	-1.04	included	present	821.05	-0.95	included	present	1379.82	-0.76	included	present	7005.84	1.16	included	present	1388.75	[illegible]
226695_at	included	present	1082.81	-0.52	included	present	1198.53	-0.47	included	present	109.35	-0.91	included	present	113.77	-0.91	included	present	540.74	-0.74	included	present	9977.99	3.13	included	present	273.16	[illegible]
226696_at	included	present	180.73	-0.21	included	present	553.38	4.47	included	present	214.02	0.21	included	present	305.2	1.36	included	present	355.96	1.99	included	present	245.72	0.61	included	present	504.17	[illegible]
226697_at	included	present	2752.3	1.96	included	present	166.76	-1.21	included	present	461.08	-0.85	included	present	1231.47	0.1	included	present	1014.39	-0.17	included	present	2951.34	2.21	included	present	346.88	[illegible]
226698_at	excluded	absent	57.67	-0.35	excluded	absent	19.9	-0.9	excluded	absent	4.76	-1.12	excluded	absent	2.72	-1.15	excluded	absent	234.98	2.23	excluded	absent	121.98	0.58	excluded	absent	103.53	[illegible]
226699_at	excluded	absent	8.95	-0.98	excluded	absent	10.55	-0.95	excluded	absent	8.55	-0.99	excluded	absent	39.42	-0.37	excluded	absent	67.35	0.19	excluded	absent	91.17	0.67	included	present	91.8	[illegible]
226700_at	included	present	261.03	-0.25	included	absent	171.83	-0.8	included	absent	177.48	-0.76	included	present	442.63	0.87	included	absent	127.29	-1.07	included	present	393.9	0.57	included	present	238.34	[illegible]
226701_at	included	present	446.6	0.14	included	present	315.03	-0.21	included	present	353.25	-0.11	included	present	370.01	-0.06	included	present	751.86	0.93	included	present	3913.76	9.17	included	present	161.98	[illegible]
[illegible]	[illegible]	[illegible]	[illegible]	[illegible]	[illegible]	[illegible]	[illegible]	[illegible]	[illegible]	[illegible]	[illegible]	[illegible]	[illegible]	[illegible]	[illegible]	[illegible]	[illegible]	[illegible]	[illegible]	[illegible]	[illegible]	[illegible]	[illegible]	[illegible]	[illegible]	[illegible]	[illegible]	[illegible]

227003_at	included	present	1570.64	-0.93	included	present	1857.21	-0.64	included	present	1037.86	-1.47	included	present	1865.81
227004_at	included	present	143.98	-0.77	included	present	215.89	-0.62	included	present	244.72	-0.56	included	absent	65.44
227005_at	included	present	750.74	-0.89	included	present	586.32	-1.41	included	present	1500.68	1.47	included	present	686.96
227006_at	included	absent	102.39	-0.94	included	absent	44.66	-0.97	included	absent	114.08	-0.93	included	present	346.15
227007_at	excluded	absent	138.16	-0.3	excluded	absent	89.74	-0.65	excluded	absent	51.23	-0.94	excluded	absent	64.53
227008_at	included	present	397.96	-0.44	included	present	399.77	-0.43	included	present	359.3	-0.64	included	present	530.02
227009_at	included	absent	227.78	-0.59	included	present	336.41	0.37	included	absent	120.5	-1.54	included	absent	168.56
227010_at	included	present	144.22	-0.35	excluded	absent	164.87	-0.03	included	present	146.99	-0.31	included	present	154.36
227011_at	excluded	absent	3.92	-0.68	excluded	absent	62.47	0.57	excluded	absent	4.07	-0.67	excluded	absent	20.96
227012_at	included	present	149.67	-1.17	included	present	418.51	1.21	included	present	251.96	-0.26	included	present	853.1
227013_at	included	present	7741.57	0.98	included	present	919.99	-0.82	included	present	269.44	-1	included	present	520.16
227014_at	included	absent	118.61	-0.5	included	present	342.67	0.74	included	present	176.73	-0.18	included	absent	171.59
227015_at	included	present	217.79	-0.36	included	present	405.89	-0.01	included	present	175.98	-0.43	included	present	234.07
227016_at	included	present	847.23	0.9	included	present	1012.27	1.44	included	present	1013.07	1.44	included	present	305.74
227017_at	included	present	590.66	-0.45	included	present	411.26	-1.38	included	present	877.79	1.05	included	present	377.3
227018_at	included	present	1181.36	-0.61	included	present	1039.11	-0.97	included	present	1711.01	0.74	included	present	961.2
227019_at	excluded	absent	79.67	-0.83	included	present	349.27	1.3	included	present	213.4	0.23	included	present	363.53
227020_at	included	present	1943.38	0.73	included	present	440.94	-1.73	included	present	717.34	-1.27	included	present	2454.91
227021_at	included	present	707.81	1.22	included	present	3204.45	15.54	included	present	433.52	-0.36	included	present	2952.05
227022_at	included	present	1021.4	0.75	included	present	427.15	-1.66	included	present	1346.49	2.08	included	present	1381.18
227023_at	excluded	absent	50.25	0.8	excluded	absent	78.04	1.81	excluded	absent	27.88	-0.01	excluded	absent	22.26
227024_s_at	excluded	absent	172.82	-0.04	excluded	absent	152.74	-0.37	excluded	absent	141.1	-0.57	excluded	absent	145.31
227025_at	included	present	562.92	-0.21	included	present	403.68	-1.14	included	present	923.7	1.9	included	present	863.66
227026_at	included	present	2848.58	0.78	included	present	2430.69	0.09	included	present	2254.59	-0.2	included	present	1182.56
227027_at	included	present	641.72	-0.13	included	present	527.17	-0.34	included	present	937.85	0.4	included	present	1307.72
227028_s_at	included	present	346.04	1.7	included	present	271.32	0.65	included	present	136.06	-1.25	included	present	253.77
227029_at	included	present	718.22	-0.34	included	present	1005.94	0.44	included	present	498.96	-0.93	included	present	775.79
227030_at	excluded	absent	7.07	-0.5	excluded	absent	14.96	-0.45	excluded	absent	7.33	-0.5	excluded	absent	8.26
227031_at	included	present	1133.48	1.72	included	present	540.39	-1.07	included	present	514.56	-1.19	included	present	888.6
227032_at	included	present	131.95	-0.75	included	present	1938.89	7.14	included	absent	80.06	-0.98	included	present	332.94
227033_at	included	present	585	0.74	included	present	461	0.24	included	present	446.53	0.19	included	present	702.7
227034_at	included	present	563.44	0.6	included	present	142.97	-0.71	included	present	181.74	-0.59	included	present	75.9
227035_x_at	included	present	714.29	-0.4	included	present	1529.3	2.88	included	present	682.52	-0.52	included	present	1461.76
227036_at	included	present	2047.28	0.23	included	present	1800.13	-0.11	included	present	1133.07	-1	included	present	1684.69
227037_at	included	present	1213.68	0.69	included	present	1591.11	1.4	included	present	2376.36	2.87	included	present	749.32
227038_at	included	present	167.7	-0.37	included	absent	51.16	-0.76	included	absent	40.12	-0.8	included	present	78.59
227039_at	included	present	547.85	0.1	included	present	338.45	-0.53	included	absent	149.47	-1.1	included	absent	222.95
227040_at	included	present	635.25	-0.29	included	present	503.37	-0.91	included	present	654.36	-0.2	included	present	551.81
227041_at	included	present	1331.31	-0.94	included	present	2088.95	-0.33	included	present	2339.15	-0.13	included	present	3895.51
227042_at	excluded	absent	82.31	-0.11	excluded	absent	359.99	4.38	excluded	absent	157.97	1.12	excluded	absent	101.74
227043_at	excluded	absent	150.52	1.08	excluded	absent	38.79	-0.77	excluded	absent	151.67	1.1	excluded	absent	39.54
227044_at	included	present	642.08	-0.54	included	present	1400.41	1.28	included	present	1292.47	1.02	included	present	1603.3
227045_at	included	present	399.22	-0.07	included	present	370.64	-0.21	included	present	534.1	0.62	included	present	1064.5
227046_at	included	present	313.33	-0.54	included	present	1034.66	1.32	included	present	282.83	-0.62	included	present	208.86
227047_x_at	included	present	859.18	0.46	included	present	113.39	-3.3	included	present	293.7	-2.39	included	present	572.26
227048_at	excluded	absent	56.92	-0.38	excluded	absent	91.18	-0.17	excluded	absent	21.15	-0.61	excluded	absent	4.08
227049_at	included	present	519.43	-0.43	included	present	728.7	0.54	included	present	425.5	-0.86	included	present	1614.45
227050_at	included	present	254.85	3.46	included	present	512.53	8.15	excluded	absent	71.98	0.13	excluded	absent	187.75
227051_at	included	present	592.71	2.16	included	absent	87.51	-0.59	included	present	192.09	-0.02	included	present	1470.43
227052_at	included	present	5066.95	-0.8	included	present	3442.45	-1.26	included	present	3743.08	-1.18	included	present	5312.88
227053_at	included	present	250.9	-0.44	excluded	absent	150.1	-0.58	excluded	absent	85.76	-0.66	included	present	366.34
227054_at	included	present	568.53	-0.44	included	present	276.85	-1.55	included	present	574.67	-0.42	included	present	434.24
227055_at	excluded	absent	51.07	-0.26	excluded	absent	5.16	-0.37	excluded	absent	56.26	-0.25	excluded	absent	1.56
227056_at	included	present	747.4	-0.29	included	present	383.03	-0.99	included	present	843.32	-0.11	included	present	654.81
227057_at	excluded	absent	3.92	-0.39	excluded	absent	13.07	0.22	excluded	absent	29.29	1.29	excluded	absent	26.15
227058_at	included	present	131.02	-0.44	included	present	117.36	-0.48	excluded	absent	89.54	-0.57	excluded	absent	11.41
227059_at	included	present	327.8	1.95	included	present	199.34	0.66	included	present	94.7	-0.39	included	present	372.03
227060_at	excluded	absent	98.9	0.32	excluded	absent	110.38	0.48	excluded	absent	69.06	-0.09	excluded	absent	65.65
227061_at	included	absent	72.56	-0.61	included	absent	95.95	-0.59	included	absent	34.6	-0.64	included	absent	4.36
227062_at	included	present	3157.12	-0.39	included	present	274.82	-0.75	included	present	1481.5	-0.6	included	present	732.28
227063_at	included	present	546.42	-0.42	included	present	543.55	-0.42	included	present	312.08	-0.96	included	present	293.3
227064_at	included	present	6910.87	0.88	included	present	4335.16	0.08	included	present	4589.13	0.16	included	present	2743.73
227065_at	included	present	246.58	-0.44	included	present	220.5	-0.66	included	absent	37.92	-2.19	included	absent	203.82
227066_at	included	present	476.57	0.24	included	present	322.69	-0.13	included	absent	105.7	-0.65	included	present	341.43
227067_x_at	included	present	1081.21	-0.05	included	present	406.76	-1	included	present	588.01	-0.75	included	present	381.87
227068_at	included	present	3579.38	1.72	included	present	1411.92	-0.7	included	present	2725.75	0.77	included	present	1950.26

227362_at	included	present	324.28	1.45	included	present	235.23	0.75	included	present	161.61	0.18	included	present	128.63	-0.07	included	present	424.71
227363_s_at	included	present	672.93	-0.45	included	present	1733.81	3.99	included	present	1223.61	1.86	included	present	1143.96	1.53	included	present	840.3
227364_at	included	present	9838.13	-0.54	included	present	4872.48	-1.55	included	present	18774.79	1.27	included	present	6834.37	-1.15	included	present	9440
227365_at	included	present	157.63	-0.47	included	present	1340.95	1.57	included	present	1086.61	1.13	excluded	absent	9.3	-0.73	included	present	592.14
227366_at	included	present	111.88	0.31	excluded	absent	126.38	0.6	excluded	absent	4.34	-1.84	excluded	absent	4.71	-1.83	excluded	absent	14.39
227367_at	included	present	171.1	-1.06	included	present	530.32	0.35	included	present	253.47	-0.73	included	present	915.48	1.85	included	present	374.46
227368_at	included	present	307.61	-0.56	included	present	1078.28	2.84	included	present	453.93	0.08	included	present	372.22	-0.28	included	present	277.96
227369_at	included	present	1141.21	-0.54	included	present	990.34	-0.7	included	present	1950.13	0.33	included	present	3746.72	2.27	included	present	1117.1
227370_at	included	present	2833.74	0.02	included	present	2497.22	-0.09	included	present	834.44	-0.63	included	present	724.88	-0.66	included	present	2192.8
227371_at	included	present	164.87	-0.19	excluded	absent	42.18	-0.66	excluded	absent	72.23	-0.54	included	present	1488.75	4.93	excluded	absent	28.74
227372_s_at	excluded	absent	72.01	-0.5	excluded	absent	1.93	-0.76	included	present	76.45	-0.49	included	present	1792.28	5.84	excluded	absent	5.42
227373_at	included	present	1235.2	-0.54	included	present	1755.93	0.87	included	present	2160.98	1.96	included	present	1027.74	-1.1	included	present	733.33
227374_at	included	present	285.28	0.12	included	present	748.34	4.36	included	present	1035.12	6.99	included	present	361.91	0.82	included	present	458.98
227375_at	included	present	429.69	1.08	included	present	176.52	-1.36	included	present	149.78	-1.62	included	present	722.44	3.9	included	present	227.66
227376_at	included	present	1062.97	0.25	included	present	203.47	-0.8	included	absent	77.94	-0.96	included	absent	124	-0.9	included	present	219.35
227377_at	excluded	absent	4.09	-0.52	excluded	absent	63.83	1.57	excluded	absent	6.86	-0.42	excluded	absent	32.81	0.48	excluded	absent	60.72
227378_x_at	included	present	271.85	-0.35	included	present	460.8	1.84	included	present	255.6	-0.54	included	present	185.6	-1.35	included	present	200.4
227379_at	included	present	1096.91	0.51	included	present	753.38	0.05	included	present	163.82	-0.74	included	present	1628.57	1.22	included	absent	178.01
227380_x_at	included	present	149.07	-0.71	included	present	224.47	0.67	excluded	absent	142.84	-0.83	excluded	absent	86.65	-1.86	excluded	absent	123.32
227381_at	excluded	absent	107.19	-0.23	excluded	absent	6.38	-0.83	excluded	absent	17.04	-0.77	excluded	absent	11.7	-0.8	excluded	absent	14.65
227382_at	included	present	205.17	-0.13	included	absent	110.29	-0.63	included	present	139.28	-0.47	included	present	136.06	-0.49	included	absent	110.97
227383_at	included	present	461.55	-0.38	included	present	152.89	-0.91	included	present	569.16	-0.19	included	present	1131.22	0.77	included	present	1133.71
227384_s_at	included	present	960.14	-0.58	included	present	623.47	-0.87	included	present	1000.3	-0.54	included	present	2100.06	0.41	included	present	3445.4
227385_at	included	present	994.39	-0.59	included	present	2669.17	1.02	included	present	3051.2	1.38	included	present	1124.3	-0.46	included	present	1025.81
227386_s_at	included	present	445.51	-1.27	included	present	6307.8	2.71	included	present	1167.08	-0.78	included	present	5758.68	2.34	included	present	1608.37
227387_at	included	absent	89.28	-1.85	included	present	125.21	-1.57	included	present	229.05	-0.78	included	present	120.98	-1.61	included	present	353.75
227388_at	included	present	1230.29	-0.67	included	present	2189.64	0.36	included	present	1966.42	0.12	included	present	2227.76	0.4	included	present	1893
227389_x_at	included	present	560.04	-0.25	included	present	441.43	-0.55	included	present	778.63	0.3	included	present	753.77	0.24	included	present	570.71
227390_at	included	present	2585.78	0.54	included	absent	39.71	-0.43	included	present	643.04	-0.2	included	present	9759	3.27	included	present	396.71
227391_x_at	included	present	1088.3	-0.45	included	present	795.28	-0.66	included	present	1188.13	-0.38	included	present	2913.8	0.83	included	present	975
227392_at	excluded	absent	119.48	0.57	excluded	absent	84.93	0.07	excluded	absent	37.23	-0.62	excluded	absent	7.28	-1.05	excluded	absent	31.77
227393_at	excluded	absent	8.91	-0.57	excluded	absent	6.31	-0.6	excluded	absent	6.93	-0.59	excluded	absent	109.22	0.27	excluded	absent	58.96
227394_at	included	present	1810.08	-0.2	included	present	7758.12	2.92	included	present	2995.23	0.42	included	present	2979.27	0.42	included	present	2148.62
227395_at	included	present	443.22	0.57	included	present	191.44	-1.11	included	present	405.41	0.32	included	present	205.17	-1.02	included	present	170.93

227722_at	included	present	1076.78	3.22	included	present	362.21	-0.28	included	present	750.5	1.62	included	present	335.87	-0.41	included	present	736.22	1.55	included	present	318.66	-0.5	included	present	1982.71	7.65	included	present	1519.19	5.38	included	present	1253.12	4.08	included	present	1034.41	3.01	included	present	830.23	2.01	included	present	394.75	-0.13	included	present	315	-0.52	included	present	76.56	-1.68	included	present	1810.38	6.81
227723_at	excluded	absent	92.41	0.36	excluded	absent	73.92	0.06	excluded	absent	16.48	-0.86	excluded	absent	20.64	-0.79	excluded	absent	136.43	1.06	excluded	absent	93.86	0.38	excluded	absent	116.47	0.74	excluded	absent	137.84	1.09	excluded	absent	89.16	0.31	excluded	absent	150.14	1.28	excluded	absent	174.36	1.67	excluded	absent	82.53	0.2	excluded	absent	18.66	-0.82	excluded	absent	20.56	-0.79	excluded	absent	75.3	0.09
227724_at	included	present	928.85	-0.86	included	present	1043.88	-0.65	included	present	1060.06	-0.62	included	present	1512.21	0.23	included	present	1765.37	0.7	included	present	1280.08	-0.21	included	present	554.51	-1.57	included	present	696.2	-1.3	included	present	1772.98	0.72	included	present	630.13	-1.42	included	present	1390.36	0	included	present	750.85	-1.2	included	present	932.46	-0.86	included	present	1132.68	-0.48	included	present	669.1	-1.35
227725_at	included	present	162.85	-0.04	excluded	absent	5.04	-0.41	excluded	absent	2.7	-0.41	excluded	absent	1.7	-0.41	excluded	absent	3.25	-0.41	excluded	absent	32.59	-0.34	excluded	absent	3.14	-0.41	excluded	absent	8.39	-0.4	included	present	78.43	-0.23	excluded	absent	1.05	-0.42	excluded	absent	61.02	-0.27	excluded	absent	23.55	-0.36	excluded	absent	1.45	-0.41	excluded	absent	6.97	-0.4	excluded	absent	1.67	-0.41
227726_at	excluded	absent	99.76	-0.69	included	present	185.2	0.51	included	present	137.61	-0.16	excluded	absent	96.59	-0.73	excluded	absent	102.84	-0.65	included	present	461.91	4.41	included	present	228.61	1.13	included	present	335.28	2.63	included	present	396.9	3.5	included	present	289.93	1.99	included	present	257.9	1.54	included	present	235.15	1.22	excluded	absent	65.67	-1.17	excluded	absent	38.91	-1.55	included	present	234.18	1.2
227727_at	included	present	125.58	-0.41	excluded	absent	85.42	-0.52	excluded	absent	28.7	-0.69	excluded	absent	12.93	-0.73	excluded	absent	82.37	-0.53	included	present	1290.14	2.96	excluded	absent	86.08	-0.52	excluded	absent	6.42	-0.75	included	present	269.31	0.01	included	present	124.56	-0.41	excluded	absent	53.16	-0.62	excluded	absent	35.06	-0.67	excluded	absent	22.48	-0.71	excluded	absent	75.16	-0.55	excluded	absent	46.76	-0.64
227728_at	included	present	1151.75	-0.55	included	present	785.83	-1.25	included	present	1141.73	-0.56	included	present	1701.06	0.51	included	present	634.7	-1.54	included	present	831.65	-1.16	included	present	2022.49	1.13	included	present	859.41	-1.11	included	present	1203.27	-0.45	included	present	1980.57	1.05	included	present	1597.11	0.31	included	present	2079.42	1.24	included	present	3995.35	4.92	included	present	1378.52	-0.11	included	present	1887.02	0.87
227729_at	included	present	351.09	-0.32	included	present	294.69	-0.69	included	present	715.51	2.07	included	present	1266.99	5.68	included	present	441.88	0.27	included	present	236.92	-1.07	included	present	175.62	-1.47	included	present	674.84	1.8	included	present	494.43	0.62	included	present	337.46	-0.41	included	present	313.11	-0.57	included	present	1207.71	5.29	included	present	150.5	-1.64	included	present	230.51	-1.11	included	present	201.58	-1.3
227730_at	excluded	absent	291.51	-0.98	included	present	744.25	1.69	excluded	absent	321.3	-0.81	excluded	absent	465.99	0.05	excluded	absent	358.92	-0.58	excluded	absent	318.07	-0.82	excluded	absent	709.34	1.48	excluded	absent	412.65	-0.27	excluded	absent	468.72	0.06	excluded	absent	723.28	1.56	excluded	absent	648.12	1.12	included	present	318.45	-0.82	excluded	absent	411.11	-0.28	excluded	absent	132.15	-1.92	included	present	980.6	3.08
227731_at	included	present	1779.69	-0.28	included	present	498.29	-2.39	included	present	1659.1	-0.47	included	present	1306.14	-1.06	included	present	1498.62	-0.74	included	present	1384.89	-0.93	included	present	1353.88	-0.98	included	present	920.35	-1.69	included	present	1525.29	-0.7	included	present	987.01	-1.58	included	present	1602.25	-0.57	included	present	979.33	-1.6	included	present	988.48	-1.58	included	present	767.03	-1.95	included	present	1097.67	-1.4
227732_at	included	present	286.04	-0.13	included	present	377.81	0.5	included	present	275.36	-0.2	included	present	112.14	-1.31	included	absent	192.03	-0.77	included	present	230.06	-0.51	included	present	399.08	0.64	included	present	362.5	0.39	included	present	380.19	0.51	included	present	128.47	-1.2	included	present	401.68	0.66	included	present	410.54	0.72	included	present	219.87	-0.58	included	present	355.71	0.35	included	present	400.94	0.66
227733_at	excluded	absent	74.31	-0.19	excluded	absent	136.07	0.31	excluded	absent	8.56	-0.72	excluded	absent	138.81	0.33	excluded	absent	70.8	-0.21	excluded	absent	7.89	-0.72	included	present	298.37	1.62	included	present	251.09	1.24	included	present	320.44	1.8	included	present	451.78	2.86	excluded	absent	142.97	0.37	excluded	absent	163.96	0.54	excluded	absent	231.86	1.09	included	present	103.65	0.05	included	present	333.26	1.91
227734_s_at	excluded	absent	4.19	-0.69	excluded	absent	17.35	-0.19	excluded	absent	36.26	0.52	excluded	absent	4.81	-0.66	excluded	absent	17.56	-0.19	excluded	absent	17.44	-0.19	excluded	absent	96.91	2.8	excluded	absent	15.46	-0.26	excluded	absent	73.57	1.92	excluded	absent	18.31	-0.16	excluded	absent	29.36	0.26	excluded	absent	99.14	2.88	excluded	absent	30.15	0.29	excluded	absent	7.86	-0.55	excluded	absent	44.65	0.83
227735_s_at	excluded	absent	60.41	-0.06	excluded	absent	3.09	-0.24	excluded	absent	36.97	-0.13	excluded	absent	30.17	-0.15	excluded	absent	7.86	-0.22	excluded	absent	1.7	-0.24	excluded	absent	2.65	-0.24	excluded	absent	37.61	-0.13	excluded	absent	64.07	-0.05	excluded	absent	1.42	-0.24	excluded	absent	56.07	-0.07	excluded	absent	37.54	-0.13	excluded	absent	4.19	-0.23	excluded	absent	1.53	-0.24	excluded	absent	9.05	-0.22
227736_at	excluded	absent	1.61	-0.19	excluded	absent	40.32	-0.13	excluded	absent	12.3	-0.17	excluded	absent	1.71	-0.19	excluded	absent	2.98	-0.19	excluded	absent	2.37	-0.19	excluded	absent	2.36	-0.19	excluded	absent	2.69	-0.19	excluded	absent	2.56	-0.19	excluded	absent	27.14	-0.15	excluded	absent	32.95	-0.14	excluded	absent	1.82	-0.19	excluded	absent	1.96	-0.19	excluded	absent	1.22	-0.19	excluded	absent	1.01	-0.19
227737_at	included	present	615.11	2.04	included	present	520.49	1.39	included	present	527.42	1.43	included	present	620.8	2.08	included	present	299.97	-0.14	included	present	413.32	0.64	included	present	566.56	1.7	included	present	472.35	1.05	included	present	402.97	0.57	included	present	272.18	-0.33	included	present	161.53	-1.1	included	present	664.89	2.38	included	present	420.65	0.7	included	present	374.87	0.38	included	present	382.71	0.43
227738_s_at	included	present	142.86	-0.78	excluded	absent	284.95	0.67	included	absent	109	-1.12	included	absent	118.49	-1.02	included	absent	142.05	-0.78	included	present	372.86	1.57	included	present	175.58	-0.44	included	present	243.73	0.25	included	absent	141.12	-0.79	included	absent	134.48	-0.86	included	absent	166.55	-0.53	excluded	absent	341.15	1.25	excluded	absent	296.71	0.79	included	present	206.65	-0.12	included	present	162.9	-0.57
227739_at	excluded	absent	88.13	-0.59	included	present	183.74	0.48	excluded	absent	61.77	-0.89	excluded	absent	140.62	0	excluded	absent	32.04	-1.22	excluded	absent	288.23	1.66	excluded	absent	140.44	-0.01	included	present	262.04	1.36	included	present	180.99	0.45	included	present	431.2	3.26	included	present	317.07	1.98	included	present	222.73	0.92	excluded	absent	58.16	-0.93	excluded	absent	71.41	-0.78	included	present	256.64	1.3
227740_at	included	present	7315.57	2.39	included	present	1843.14	-1.18	included	present	5636.19	1.29	included	present	7195.62	2.31	included	present	5114.38	0.95	included	present	1925.25	-1.13	included	present	4039.37	0.25	included	present	2697.24	-0.62	included	present	2662.95	-0.65	included	present	2550.85	-0.72	included	present	2043.93	-1.05	included	present	1636.43	-1.32	included	present	7152.58	2.28	included	present	4632.63	0.64	included	present	1638.93	-1.31
227741_at	included	present	699.66	0	included	present	198.34	-1.54	included	present	928.3	0.7	included	present	937.92	0.73	included	present	461	-0.73	included	present	415.66	-0.87	included	present	519.19	-0.55	included	present	584.08	-0.35	included	present	1257.96	1.72	included	present	453.88	-0.75	included	present	574.64	-0.38	included	present	300.23	-1.23	included	present	260.22	-1.35	included	present	142.73	-1.71	included	present	788.41	0.27
227742_at	excluded	absent	3.08	-0.73	excluded	absent	115.54	-0.28	included	present	470.63	1.16	included	present	586.83	1.62	excluded	absent	32.9	-0.61	excluded	absent	156.87	-0.11	excluded	absent	3.09	-0.73	excluded	absent	31.34	-0.61	excluded	absent	30.34	-0.62	excluded	absent	40.9	-0.58	excluded	absent	40.92	-0.58	excluded	absent	11.78	-0.69	included	present	149.1	-0.14	excluded	absent	5.31	-0.72	excluded	absent	17.29	-0.67
227743_at	included	present	148.67	-0.36	included	absent	73.59	-0.9	included	absent	12.62	-1.33	included	present	195.95	-0.02	included	present	217.72	0.13	included	absent	145.84	-0.38	included	absent	188.07	-0.08	included	present	217.34	0.13	included	present	166.96	-0.23	included	present	105.62	-0.67	included	present	87.41	-0.8	included	absent	137.1	-0.44	included	present	151.5	-0.34	included	present	206.36	0.05	included	present	77.87	-0.87
227744_s_at	included	present	182.88	0.85	included	present	258.31	2.09	included	present	202.46	1.17	included	present	261.99	2.15	excluded	absent	283.03	2.49	included	present	370.47	3.93	included	present	302.85	2.82	included	present	749.84	10.16	included	present	492.58	5.93	included	present	397.88	4.38	included	present	908.9	12.77	included	present	206.05	1.23	excluded	absent	7.14	-2.04	excluded	absent	2.37	-2.12	included	present	554.95	6.96
227745_at	included	present	88.58	-0.97	included	present	149.34	0.13	included	present	225.94	1.53	included	present	209.53	1.23	excluded	absent	166.9	0.45	excluded	absent	148.28	0.11	included	present	310.72	3.07	included	present	88.94	-0.97	included	present	239.87	1.78	included	present	121.74	-0.37	included	present	195.68	0.98	included	absent	94.53	-0.87	included	present	160.27	0.33	included	absent	67.36	-1.36	included	present	86.45	-1.01
227746_at	excluded	absent	187.65	0	included	present	262.86	0.55	included	present	476.4	2.13	included	present	456.51	1.98	excluded	absent	360.08	1.27	excluded	absent	77.93	-0.82	included	present	698.34	3.77	included	present	301.17	0.83	included	present	371.27	1.35	included	present	685.45	3.67	included	present	482.26	2.17	included	present	194.26	0.04	included	present	148.5	-0.29	included	present	190.52	0.02	included	present	909.27	5.33
227747_at	excluded	absent	47.81	-0.48	excluded	absent	6.07	-0.55	included	present	91.3	-0.4	included	present	283.92	-0.07	excluded	absent	8.57	-0.55	excluded	absent	219.17	-0.18	excluded	absent	84.53	-0.42	excluded	absent	68.05	-0.44	excluded	absent	105.89	-0.38	excluded	absent	49.98	-0.48	excluded	absent	66.06	-0.45	included	present	150.26	-0.3	excluded	absent	79.55	-0.42	included	present	64.66	-0.45	included	present	85.8	-0.41
227748_at	included	present	832.18	3.32	included	present	381.25	0.25	included	present	325.67	-0.12	included	present	592.6	1.69	included	present	652.41	2.1	included	present	142.83	-1.37	included	present	271.23	-0.49	included	present	470.16	0.86	included	present	380.64	0.25	included	present	126.03	-1.48	included	present	334.51	-0.06	included	absent	26.99	-2.15	included	present	175.62	-1.14	included	present	127.42	-1.47	included	present	202.58	-0.96
227749_at	included	present	307.58	0.33	included	present	667.56	1.8	included	present	586.74	1.47	excluded	absent	77.55	-0.62	included	present	316.85	0.37	excluded	absent	171.08	-0.23	included	present	1181.5	3.91	excluded	absent	107.67	-0.49	included	present	1985.85	7.21	included	present	697.29	1.93	included	present	635.39	1.67	included	present	1633.96	5.77	included	present	782.18	2.27	included	present	434.5	0.85	included	present	4449.91	17.31
227750_at	included	present	185.3	-1.07	included	present	364.67	-0.95	included	absent	145.44	-1.1	included	present	292.16	-1	included	present	469.54	-0.88	included	present	989.95	-0.53	included	present	537.82	-0.83	included	present	605.68	-0.79	included	present	758.23	-0.69	included	present	1622.86	-0.11	included	present	671.9	-0.74	included	present	951.59	-0.56	included	present	993.54	-0.53	included	present	1258.31	-0.35	included	present	397.06	-0.93
227751_at	included	present	170.99	-1.14	included	present	220.98	-0.73	included	present	224.05	-0.71	included	present	436.97	1.02	included	present	185.06	-1.02	included	present	170.44	-1.14	included	present	357.44	0.37	included	present	266.31	-0.37	included	present	444.12	1.08	included	present	149.86	-1.31	included	present	362.03	0.41	included	present	204.78	-0.86	included	present	331.58	0.16	included	present	483.13	1.39	included	present	111.43	-1.62
227752_at	included	present	109.03	-0.42	included	absent	101.97	-0.46	included	absent	2.64	-0.94	included	absent	7.21	-0.91	included	absent	67.72	-0.62	included	absent	109.44	-0.42	included	absent	3.44	-0.93	included	absent	5.42	-0.92	included	present	80.35	-0.56	included	present	73.63	-0.59	included	absent	29.82	-0.81	included	absent	63.31	-0.64	included	absent	24	-0.83	included	absent	59.78	-0.66	included	absent	25.34	-0.83
227753_at	included	present	275.04	0.03	included	present	184.39	-0.13	excluded	absent	93.25	-0.3	excluded	absent	1.35	-0.46	excluded	absent	23.43	-0.42	excluded	absent	45.31	-0.38	excluded	absent	111.23	-0.27	excluded	absent	89.83	-0.3	included	present	94.39	-0.3	excluded	absent	16.24	-0.44	excluded	absent	255.23	-0.01	excluded	absent	21.48	-0.43	excluded	absent	124.1	-0.24	excluded	absent	24.66	-0.42	excluded	absent	84.32	-0.31
227754_at	included	present	1294.18	1.39	included	present	1665.45	2.58	included	present	1079.65	0.7	included	present	1563.36	2.26	included	present	1005.37	0.46	included	present	420.06	-1.41	included	present	983.17	0.39	included	present	682.09	-0.57	included	present	842.09	-0.06	included	present	616.37	-0.78	included	present	1057.07	0.63	included	present	700.1	-0.52	included	present	1246.12	1.24	included	present	1140.65	0.9	included	present	549.96	-1
227755_at	included	present	543.76	-0.48	included	present	186.75	-0.85	included	present	376.76	-0.65	included	present	5855.93	4.91	included	present	365.13	-0.67	included	present	713.55	-0.31	included	present	695.62	-0.33	included	present	700.86	-0.32	included	present	809.1	-0.21	included	present	374.09	-0.66	included	present	210.82	-0.82	included	present	841.74	-0.18	included	present	370.84	-0.66	included	present	378.93	-0.65	included	present	476.9	-0.55

[illegible]

228081_at	included	present	409.38	-0.45	included	present	579.21	0.22	included	present	445.46	-0.3	included	present	258.37	-1.04	included	present	349.14	-0.68	included	present	367.78	-0.61	included	present	625.69	0.4	included	present	314.34	-0.82	included	present	453.75	-0.27	included	present	328.56	-0.76	included	present	362.48	-0.63	included	present	701.28	0.7	included	present	1151.44	2.47	included	present	1085.11	2.21	included	present	427.86	-0.37
228082_at	included	present	697.78	0.28	included	present	433.15	-0.16	included	present	704.75	0.29	included	absent	107.41	-0.7	included	present	300.13	-0.38	included	present	655.43	0.21	included	absent	49.13	-0.79	included	present	596.05	0.11	included	present	356.04	-0.29	included	present	93.28	-0.72	included	present	634.73	0.17	included	present	249.24	-0.46	included	present	184.34	-0.57	included	present	572.45	0.07	included	absent	71.61	-0.76
228083_at	included	present	232.54	2.13	excluded	absent	196.36	1.53	excluded	absent	17.27	-1.44	excluded	absent	98.39	-0.09	excluded	absent	143.3	0.65	included	present	213.99	1.82	excluded	absent	80.81	-0.39	included	present	50.28	-0.89	included	present	133.14	0.48	included	present	101.12	-0.05	included	present	118.76	0.24	included	present	211.55	1.78	included	present	199.61	1.59	included	present	85.55	-0.31	excluded	absent	57.6	-0.77
228084_at	included	present	521.86	-0.75	included	present	476.8	-0.93	included	present	396.39	-1.26	included	present	834.85	0.52	included	absent	164.56	-2.2	included	present	559.01	-0.6	included	present	281.18	-1.73	included	present	625.89	-0.33	included	present	482.66	-0.91	included	present	667.69	-0.16	included	present	445.65	-1.06	included	present	779.79	0.3	included	present	311.3	-1.61	included	present	631.73	-0.3	included	present	94.5	-2.49
228085_at	included	present	121.62	0.83	included	present	195.13	2.18	excluded	absent	116.8	0.75	excluded	absent	64.19	-0.22	excluded	absent	124.82	0.89	excluded	absent	105.13	0.53	excluded	absent	41.53	-0.64	excluded	absent	51.6	-0.45	included	present	94.43	0.33	excluded	absent	5.45	-1.3	excluded	absent	58.11	-0.33	excluded	absent	47.07	-0.54	excluded	absent	2.6	-1.35	excluded	absent	3.46	-1.34	excluded	absent	53.75	-0.41
228086_at	excluded	absent	30.85	-0.59	excluded	absent	3.92	-1.15	excluded	absent	4.38	-1.14	excluded	absent	90.29	0.65	excluded	absent	6.38	-1.1	excluded	absent	91.05	0.67	excluded	absent	52.67	-0.14	included	present	51.1	-0.17	excluded	absent	47.41	-0.25	included	present	67.64	0.18	excluded	absent	20.14	-0.82	included	present	64.88	0.12	included	present	71.1	0.25	included	present	103.05	0.92	excluded	absent	27.56	-0.66
228087_at	included	present	191.05	0.94	included	present	102.96	-0.51	included	absent	86.24	-0.78	included	present	174.51	0.67	excluded	absent	192.54	0.97	included	present	254.5	1.99	included	present	228.43	1.56	included	present	214.98	1.34	included	present	236.06	1.68	included	present	169.22	0.58	excluded	absent	154.09	0.33	excluded	absent	184.9	0.84	included	present	241.88	1.78	included	present	442.8	5.09	included	present	209.11	1.24
228088_at	included	present	471.38	-0.22	included	present	774.16	0.63	included	present	523.99	-0.07	included	present	745.08	0.55	included	present	622.38	0.2	included	present	310.67	-0.67	included	present	1038.68	1.37	included	present	274.78	-0.77	included	present	782.73	0.65	included	present	571.76	0.06	included	present	926.93	1.06	included	present	303.94	-0.69	included	present	932.2	1.07	included	present	464.28	-0.24	included	present	1827.03	3.58
228089_x_at	included	present	291.54	-0.73	included	present	432.98	0.04	included	present	298.7	-0.69	included	present	196.86	-1.25	included	present	272.46	-0.83	included	present	1455.45	5.62	included	present	723.04	1.62	included	present	3265.29	15.5	included	present	1322.53	4.9	included	present	1519.85	5.97	included	present	998.27	3.13	included	present	1715.19	7.04	included	present	379.71	-0.25	included	present	871.99	2.44	included	present	1447.72	5.58
228090_at	included	absent	177.24	-1.03	included	absent	105.87	-1.31	included	present	126.04	-1.23	included	present	685.74	0.98	included	absent	18	-1.66	included	present	128.98	-1.22	included	absent	109.49	-1.3	included	absent	54.95	-1.52	included	present	308.25	-0.51	included	present	160.69	-1.1	included	present	684.24	0.98	included	present	810.12	1.48	included	present	241.43	-0.78	included	absent	160.04	-1.1	included	present	89.86	-1.38
228091_at	included	present	226.03	-1.03	included	present	214.61	-1.12	included	present	184.07	-1.35	included	present	472.11	0.85	included	present	335.5	-0.19	included	present	319.84	-0.31	included	present	311.71	-0.37	included	present	263.91	-0.74	included	present	425.4	0.5	included	present	341.71	-0.15	included	present	354.26	-0.05	included	present	443.8	0.64	included	present	332.38	-0.22	included	present	381.72	0.16	included	present	284.39	-0.58
228092_at	included	present	146.62	-0.55	included	present	186.83	-0.37	included	present	117.51	-0.68	included	present	152.92	-0.52	included	present	278.3	0.05	included	present	218.14	-0.23	included	present	211.18	-0.26	included	present	160.34	-0.49	included	present	176.62	-0.41	included	absent	44.86	-1.01	included	present	285.16	0.08	included	present	223.12	-0.2	included	present	165.82	-0.46	included	present	150.21	-0.53	included	present	157.43	-0.5
228093_at	included	present	362.91	0	included	present	334.03	-0.22	included	present	225.12	-1.04	included	present	641.47	2.11	included	absent	194.7	-1.27	included	present	392.61	0.23	included	present	294.18	-0.52	included	present	632.73	2.05	included	present	348.16	-0.11	included	present	398.81	0.27	included	present	361.29	-0.01	included	present	252.57	-0.84	included	present	291.04	-0.54	included	present	295.57	-0.51	included	present	271.03	-0.7
228094_at	included	present	224.37	-0.14	included	present	169.15	-0.26	excluded	absent	70.96	-0.47	excluded	absent	5.52	-0.61	included	present	286.11	-0.01	included	present	604.23	0.67	excluded	absent	88.74	-0.43	excluded	absent	5.56	-0.61	included	present	329.93	0.08	excluded	absent	14.36	-0.59	included	present	125.49	-0.36	included	present	321.1	0.06	excluded	absent	86.07	-0.44	excluded	absent	5.85	-0.61	included	present	207.99	-0.18
228095_at	included	present	1132.55	0.36	included	present	3475.46	5.58	included	present	3297.74	5.18	included	present	2885.81	4.26	included	present	1278.28	0.68	included	present	1073.58	0.23	included	present	1064.99	0.21	included	present	1736.16	1.7	included	present	916.04	-0.13	included	present	1058.88	0.19	included	present	1388.67	0.93	included	present	1771.51	1.78	included	present	2215.96	2.77	included	present	4335.42	7.49	included	present	1925.86	2.12
228096_at	included	present	196.99	-0.5	included	present	73.77	-1.4	included	absent	103.37	-1.18	included	absent	71.83	-1.41	included	present	342.42	0.56	included	present	132.41	-0.97	included	present	433.32	1.22	included	present	277.01	0.08	included	present	320.85	0.4	included	present	255.94	-0.07	included	present	253.99	-0.09	included	present	397.95	0.96	included	present	305.51	0.29	included	present	333.25	0.49	included	present	403.69	1
228097_at	excluded	absent	74.7	-0.01	excluded	absent	4.32	-1.18	excluded	absent	2.56	-1.21	excluded	absent	72.71	-0.04	excluded	absent	12.87	-1.04	excluded	absent	41.2	-0.57	excluded	absent	35.88	-0.66	excluded	absent	55.65	-0.33	excluded	absent	60.72	-0.24	excluded	absent	38.98	-0.61	excluded	absent	36.23	-0.65	excluded	absent	32.26	-0.72	excluded	absent	33.75	-0.69	excluded	absent	5.38	-1.17	excluded	absent	29.26	-0.77
228098_s_at	included	present	249.34	-0.73	included	present	166.96	-0.92	included	absent	152.16	-0.96	included	present	1065.4	1.14	included	present	355.11	-0.49	included	present	748.01	0.41	included	present	206.32	-0.83	included	present	538.73	-0.07	included	present	648.32	0.18	included	present	416.58	-0.35	included	present	108.08	-1.06	included	present	274.37	-0.68	included	present	304.07	-0.61	included	present	80.74	-1.12	included	absent	162.54	-0.93
228099_at	included	present	372.2	-0.55	included	present	389.34	-0.46	included	present	577.32	0.61	included	present	1343.61	4.94	included	present	313.93	-0.88	included	present	280.45	-1.07	included	present	241.99	-1.29	included	present	1218.73	4.24	included	present	405.93	-0.36	included	present	242.22	-1.29	included	present	421.29	-0.28	included	present	439.81	-0.17	included	present	427.18	-0.24	included	present	679.1	1.18	included	present	243.55	-1.28
228100_at	excluded	absent	32.33	-0.64	excluded	absent	2.07	-0.74	excluded	absent	2.95	-0.73	excluded	absent	33.44	-0.64	excluded	absent	21.43	-0.68	excluded	absent	60.69	-0.56	excluded	absent	58.1	-0.57	excluded	absent	59.43	-0.56	excluded	absent	5.1	-0.73	excluded	absent	24.75	-0.67	excluded	absent	45.55	-0.6	excluded	absent	26.22	-0.66	excluded	absent	218.42	-0.08	excluded	absent	75.21	-0.51	excluded	absent	4.55	-0.73
228101_at	included	present	502.87	-0.2	included	present	259.19	-0.61	included	present	167.92	-0.76	included	present	209.54	-0.69	included	present	521.23	-0.17	included	absent	210.3	-0.69	included	present	1105.59	0.8	included	present	526.73	-0.16	included	present	485.74	-0.23	included	present	1298.32	1.12	included	present	276.9	-0.58	included	present	459.58	-0.27	included	present	278.43	-0.57	included	present	449	-0.29	included	present	935.96	0.52
228102_at	included	present	3009.51	5.85	included	present	1435.56	2.18	included	present	1364.87	2.02	included	present	119.85	-0.88	included	present	1111.99	1.43	included	present	1061.92	1.31	included	present	1934.85	3.35	included	present	649.02	0.35	included	present	1858.78	3.17	included	present	2460.02	4.57	included	present	1670.01	2.73	included	present	874.77	0.88	included	present	305.77	-0.45	included	present	1600	2.57	included	present	1659.39	2.71
228103_s_at	included	present	12832.58	2.74	included	present	7709.45	1.31	included	present	5473.85	0.69	included	present	587.62	-0.68	included	present	5629.91	0.73	included	present	2035.4	-0.27	included	present	4173.44	0.32	included	present	1398.47	-0.45	included	present	6680.7	1.02	included	present	8646.61	1.57	included	present	5719.02	0.75	included	present	4852.06	0.51	included	present	1068.62	-0.54	included	present	4132.14	0.31	included	present	5204.25	0.61
228104_at	included	present	372.88	-0.47	included	present	4457.87	3.32	included	present	281.71	-0.55	included	present	1055.35	0.17	included	present	1253.35	0.35	included	present	268.9	-0.56	included	present	3399.72	2.34	included	present	245.9	-0.58	included	present	459.14	-0.39	included	present	1199	0.3	included	present	2190.01	1.22	included	present	2014.48	1.06	included	present	2580.91	1.58	included	present	2909.82	1.89	included	present	453.48	-0.39
228105_at	included	present	508.89	0.11	included	present	240.04	-1.19	included	present	299.32	-0.9	included	present	245.65	-1.16	included	present	516.69	0.15	included	present	318.85	-0.81	included	present	375.54	-0.53	included	present	257.55	-1.11	included	present	671.69	0.9	included	present	428.1	-0.28	included	present	687.36	0.97	included	present	394.03	-0.44	included	present	192.16	-1.42	included	present	454.71	-0.15	included	present	232.39	-1.23
228106_at	included	present	924.79	-1.03	included	present	5112.56	5.41	included	present	1750.12	0.24	included	present	2095.87	0.77	included	present	718.15	-1.34	included	present	908.86	-1.05	included	present	1992.94	0.62	included	present	2648.99	1.62	included	present	1442.44	-0.23	included	present	1402.32	-0.29	included	present	1611.48	0.03	included	present	1143.18	-0.69	included	present	1783.97	0.29	included	present	1764.46	0.26	included	present	1886.64	0.45
228107_at	included	present	155.3	-0.49	included	present	149.7	-0.5	included	present	154.68	-0.49	included	absent	13.32	-0.68	included	present	191.22	-0.45	included	present	263.08	-0.35	included	present	253.04	-0.37	included	present	1002	0.6	included	present	333.06	-0.26	included	present	297.68	-0.31	included	present	178.77	-0.46	included	present	425.74	-0.14	included	present	385.1	-0.2	included	present	618.64	0.1	included	present	317.36	-0.28
228108_at	included	present	145.22	-1.1	included	present	852.06	-0.02	included	present	434.07	-0.66	included	present	370.58	-0.75	included	present	533.99	-0.51	included	present	272.86	-0.9	included	present	395.24	-0.72	included	present	886.21	0.03	included	present	395.55	-0.72	included	present	2368.74	2.3	included	present	399.17	-0.71	included	present	1061.88	0.3	included	present	660.63	-0.31	included	present	845.42	-0.03	included	present	750.01	-0.18
228109_at	included	present	471.03	-0.7	included	present	468.01	-0.71	included	absent	298.63	-0.99	included	present	235.13	-1.1	included	present	1001.01	0.2	included	present	1613.12	1.23	included	present	544.61	-0.58	included	present	316.86	-0.96	included	present	762.74	-0.21	included	present	464.06	-0.71	included	present	641.76	-0.41	included	present	810.01	-0.13	included	present	1075.92	0.32	included	present	891.3	0.01	included	present	401.8	-0.82
228110_x_at	excluded	absent	119.63	0.61	excluded	absent	61.38	-0.51	excluded	absent	49.79	-0.73	included	present	129.45	0.8	excluded	absent	41.47	-0.9	excluded	absent	64.46	-0.45	included	present	203.94	2.24	excluded	absent	86.47	-0.03	included	present	130.15	0.82	excluded	absent	82.56	-0.1	included	present	238.41	2.91	excluded	absent	11.49	-1.47	excluded	absent	9.05	-1.52	excluded	absent	26.11	-1.19	included	present	117.99	0.58
228111_s_at	included	present	410.2	-0.39	included	present	375.4	-0.49	included	absent	73.29	-1.43	included	absent	13.86	-1.62	included	absent	174.78	-1.12	included	present	355.15	-0.56	included	absent	162.69	-1.15	included	present	118.08	-1.29	included	absent	224.46	-0.96	included	present	347.05	-0.58	included	present	245.42	-0.9	included	absent	80.5	-1.41	included	absent	185.64	-1.08	included	absent	121.28	-1.28	included	absent	113.01	-1.31
228112_at	included	present	144.22	-0.79	excluded	absent	248.03	0.22	included	absent	61.16	-1.6	included	absent	94.38	-1.28	included	absent	219.18	-0.06	included	present	252.19	0.26	included	absent	101.97	-1.2	included	absent	92.31	-1.3	included	absent	189.37	-0.35	included	absent	90	-1.32	included	absent	96.97	-1.25	included	present	111.09	-1.11	included	present	135.09	-0.88	included	absent	31.49	-1.89	included	absent	38.49	-1.82
228113_at	included	absent	83.89	-0.59	included	absent	11.24	-0.9	included	absent	2.69	-0.93	included	absent	5.25	-0.92	included	absent	117.57	-0.45	included	present	163.85	-0.25	included	absent	39.8	-0.78	included	present	217.48	-0.02	included	present	210.88	-0.05	included	present	244.95	0.09	included	absent	48.83	-0.74	included	present	145.45	-0.33	included	absent	55.92	-0.71	included	absent	39.91	-0.78	included	present	71.78	-0.64
228114_x_at	included	absent	149.64	-0.89	excluded	absent	235.23	0.31	included	present	156.76	-0.79	included	present	157.02	-0.78	included	absent	132.31	-1.13	included	present	255.36	0.59	included	present	258.79	0.64	included	present	254.26	0.57	included	present	294.85	1.14	included	present	442.7	3.2	included	present	337.01	1.73	included	present	214.26	0.02	included	absent	175.02	-0.53	included	absent	150.64	-0.87	included	present	329.12	1.62

[illegible]

228440_at	included	absent	13.78	-0.6	included	present	201.18	-0.47	included	absent	57.29	-0.57	included	present	485.85	-0.27	included	absent	18.19
228441_s_at	excluded	absent	27.32	-0.45	excluded	absent	86.84	-0.31	excluded	absent	17.71	-0.48	included	present	193.94	-0.04	excluded	absent	57.44
228442_at	excluded	absent	240.85	0.44	excluded	absent	108.05	-0.61	excluded	absent	12.56	-1.36	included	present	462.1	2.18	excluded	absent	136.13
228443_s_at	included	present	911.29	2.3	included	present	666.72	1.12	included	present	626.22	0.92	included	present	498.4	0.31	included	present	736.27
228444_at	included	present	234.96	-0.3	included	present	408.34	0.78	included	absent	120.14	-1.01	included	absent	36.02	-1.53	excluded	absent	315.07
228445_at	included	present	488.15	0.36	included	absent	235.91	-1.12	included	absent	258.88	-0.99	included	present	296.82	-0.76	included	present	277.88
228446_at	included	present	2595.83	-1.31	included	present	3247.73	-0.73	included	present	4144.47	0.06	included	present	2125.45	-1.72	included	present	2424.72
228447_at	included	present	81.4	1.16	excluded	absent	65.37	0.69	excluded	absent	2.85	-1.13	included	present	60.87	0.56	excluded	absent	24.34
228448_at	included	present	257.18	0.26	included	present	1399.94	7.84	included	present	129.11	-0.59	included	absent	146.11	-0.48	included	present	443.81
228449_at	included	present	190.51	-0.22	included	present	381.15	0.08	included	absent	92.2	-0.38	included	present	230.12	-0.16	included	present	204.89
228450_at	included	present	406.98	1.57	included	present	153.96	-0.36	included	absent	5.3	-1.49	included	present	248.35	0.36	included	absent	22.63
228451_at	excluded	absent	15.55	-0.56	excluded	absent	11.1	-0.62	excluded	absent	118.71	0.8	excluded	absent	16.12	-0.55	excluded	absent	256.83
228452_at	included	present	591.9	-0.07	included	present	310.16	-0.95	included	present	725.69	0.35	included	present	922.2	0.96	included	present	272.36
228453_at	included	present	220.52	-0.92	included	present	248.64	-0.63	included	present	158.63	-1.55	included	present	399.25	0.9	included	absent	187.26
228454_at	included	present	1575.68	-0.12	included	present	2574.52	1.29	included	present	2044.55	0.54	included	present	3795.17	3.01	included	present	2276.74
228455_at	included	present	120.98	-0.97	included	present	256.77	-0.14	included	present	612.07	2.04	included	present	511.98	1.42	included	present	512.16
228456_s_at	included	present	2803.72	1.22	included	present	1998.43	0.44	included	present	1510.25	-0.03	included	present	1252.17	-0.28	included	present	1401.57
228457_at	included	present	193.34	-1.06	included	present	435.96	-0.04	included	present	196.45	-1.05	included	present	211.35	-0.99	included	present	347.92
228458_at	excluded	absent	76.85	-0.97	included	present	194.69	0.57	included	present	214.22	0.83	excluded	absent	68.35	-1.08	excluded	absent	30.84
228459_at	excluded	absent	45.86	0.47	excluded	absent	8.13	-0.55	excluded	absent	70.9	1.15	excluded	absent	8.15	-0.55	excluded	absent	5.32
228460_at	excluded	absent	58.48	-0.93	included	present	155.98	0.96	excluded	absent	113.92	0.14	excluded	absent	89.55	-0.33	excluded	absent	198.67
228461_at	included	present	219.27	0.35	excluded	absent	4.23	-1.54	excluded	absent	4.61	-1.53	included	present	115.23	-0.57	excluded	absent	71.49
228462_at	excluded	absent	5.53	-0.56	excluded	absent	4.45	-0.57	excluded	absent	68.63	-0.17	included	present	3706.05	22.42	excluded	absent	21.85
228463_at	excluded	absent	17.78	-0.36	excluded	absent	30.4	-0.19	excluded	absent	35.15	-0.13	excluded	absent	4.77	-0.52	excluded	absent	5.43
228464_at	included	present	190.06	-1.04	included	present	307.22	-0.29	included	present	1049.22	4.5	included	present	746.22	2.55	included	present	545.61
228465_at	included	present	1745.99	-0.03	included	present	939.22	-0.94	included	present	513.69	-1.43	included	present	1908.69	0.16	included	present	1065.67
228466_at	included	present	892.04	-1.15	included	present	164.25	-3.44	included	present	765.32	-1.55	included	present	732.82	-1.65	included	present	589.92
228467_at	included	present	348.69	0.37	included	present	250.6	-0.52	included	present	221.46	-0.78	included	present	672.81	3.31	included	absent	154.25
228468_at	included	present	219.02	0.87	included	present	376.77	2.85	included	present	472.17	4.06	included	present	155.06	0.06	included	present	221.47
228469_at	included	present	448.94	-0.56	included	present	401.49	-0.62	included	present	576.26	-0.41	included	present	240.02	-0.82	included	present	314.67
228470_at	excluded	absent	7.25	-0.74	excluded	absent	5.49	-0.8	excluded	absent	7.7	-0.73	excluded	absent	36.16	0.17	excluded	absent	68.33
228471_at	included	present	679.89	0.16	included	present	346.76	-0.44	included	present	480.21	-0.2	included	present	519.8	-0.13	included	present	545.5
228472_at	excluded	absent	3.44	-1.03	excluded	absent	44.96	-0.22	excluded	absent	26.54	-0.58	excluded	absent	24.36	-0.62	excluded	absent	89.67
228473_at	excluded	absent	18.94	-0.81	excluded	absent	83.29	-0.51	excluded	absent	14.59	-0.83	excluded	absent	66.72	-0.59	excluded	absent	168.09

[illegible]

228799_at	included	present	120.23	-0.19	included	present	157.23	0.43	included	present	183.31
228800_x_at	excluded	absent	73.48	0.77	excluded	absent	7.04	-0.99	excluded	absent	27.65
228801_at	included	present	436.76	-0.52	included	present	131.55	-1.54	included	present	538.26
228802_at	included	present	216.65	-0.43	included	present	148.22	-0.47	included	absent	55.09
228803_at	included	present	350.08	-0.89	included	present	153.2	-1.62	included	present	468.34
228804_at	excluded	absent	113.57	0.29	excluded	absent	84.36	0	excluded	absent	61.59
228805_at	included	present	268.1	-0.21	included	present	431.71	0.29	included	present	367.71
228806_at	excluded	absent	7.77	-0.63	excluded	absent	11.7	-0.61	excluded	absent	157.52
228807_at	included	present	1196.98	0.9	included	present	157.08	-0.62	included	absent	21.35
228808_s_at	excluded	absent	121.81	-0.04	excluded	absent	171.58	0.57	excluded	absent	105
228809_at	included	present	167.04	-0.08	included	present	239.38	0.79	included	present	94.35
228810_at	included	present	238.18	0.07	included	present	258.79	0.22	included	present	144.7
228811_at	included	present	657.26	-1.1	included	present	611.38	-1.16	included	present	765.34
228812_at	included	present	320.84	-0.48	included	present	353.91	-0.38	included	present	321.71
228813_at	included	present	668.86	0.09	included	present	293.09	-0.9	included	present	183.78
228814_at	excluded	absent	151.13	1.92	excluded	absent	111.28	1.07	excluded	absent	71.76
228815_s_at	excluded	absent	115.2	0.05	excluded	absent	147.75	0.54	excluded	absent	64.25
228816_at	excluded	absent	66.72	-0.76	excluded	absent	234.07	1.34	included	present	245.5
228817_at	included	present	416.12	-0.22	included	present	1156.2	3.99	included	present	365.83
228818_at	included	present	441.37	-0.58	included	present	218.71	-0.85	included	present	288.72
228819_at	included	present	174.76	0.03	included	present	425.13	1.55	excluded	absent	40.97
228820_at	included	absent	118.69	-0.85	included	present	643.23	6.32	included	present	227.56
228821_at	excluded	absent	9.3	-1.24	excluded	absent	127.68	0.28	included	present	157
228822_s_at	included	present	1390.68	-0.1	included	present	1353.31	-0.18	included	present	1490.9
228823_at	excluded	absent	219.47	-0.64	included	present	464.93	1.27	excluded	absent	211.2
228824_s_at	included	present	456.24	-0.02	included	present	184.49	-0.43	included	present	1213.69
228825_at	included	present	163.74	-0.05	excluded	absent	106.25	-0.18	included	present	303.93
228826_at	included	present	100.98	-0.56	included	present	106.18	-0.54	included	absent	80.81
228827_at	included	present	2071.91	1.05	included	present	2891.49	2.14	included	present	2442.27
228828_at	included	present	190.3	-0.06	included	present	243.29	0.25	included	present	87.08
228829_at	included	present	134.55	-1.57	included	present	187.53	-1.07	included	present	118.98
228830_s_at	included	present	2170.22	0.67	included	present	828.62	-1.31	included	present	765.2
228831_s_at	included	present	186.07	-0.46	included	absent	113.81	-0.59	included	absent	33.92
228832_at	included	present	211.22	-0.24	excluded	absent	287.35	0.26	excluded	absent	7.57

229158_at	excluded	absent	82.47	0.17	excluded	absent	4.38	-0.39	excluded	absent	7.56	-0.36	excluded	absent	61.24	0.02	excluded	absent	20.36	-0.27	excluded	absent	34.06	-0.18	excluded	absent	52.79	-0.04	included	present	86.93	0.2	excluded	absent
229159_at	included	present	500.64	3.4	included	present	215.43	0.66	included	present	584.7	4.21	included	present	715.66	5.47	included	present	284.13	1.32	included	present	298.02	1.45	included	present	1055.37	8.73	included	present	286.05	1.34	included	present
229160_at	included	absent	21.8	-0.75	included	absent	1.32	-0.83	included	absent	24.29	-0.74	included	absent	3.05	-0.82	included	absent	1.45	-0.83	included	present	111.98	-0.43	included	absent	2.29	-0.82	included	present	129.01	-0.37	included	absent
229161_at	excluded	absent	77.74	-0.07	excluded	absent	138.86	1.22	excluded	absent	9.26	-1.51	excluded	absent	72.09	-0.19	excluded	absent	23.56	-1.21	excluded	absent	127.95	0.99	excluded	absent	7.02	-1.56	excluded	absent	79.87	-0.02	excluded	absent
229162_s_at	excluded	absent	104.78	1.29	excluded	absent	17.85	-0.69	excluded	absent	7.01	-0.94	excluded	absent	29.27	-0.43	excluded	absent	79.69	0.72	excluded	absent	150.1	2.33	excluded	absent	121.58	1.68	excluded	absent	80.02	0.73	excluded	absent
229163_at	included	present	1804.41	-0.83	included	present	1666.42	-0.84	included	present	601.17	-0.98	included	present	773.55	-0.96	included	present	3158.36	-0.65	included	present	1915.66	-0.81	included	present	3381.69	-0.63	included	present	3969.56	-0.55	included	present
229164_s_at	excluded	absent	88.28	-0.73	excluded	absent	16.18	-1.86	excluded	absent	39.74	-1.49	excluded	absent	121.22	-0.21	excluded	absent	153.21	0.29	excluded	absent	169.61	0.54	excluded	absent	215.84	1.27	included	present	123.37	-0.18	excluded	absent
229165_at	excluded	absent	15.89	-0.85	excluded	absent	259.23	2.03	excluded	absent	147.85	0.71	excluded	absent	20.86	-0.79	excluded	absent	129.17	0.49	excluded	present	256.08	1.99	excluded	present	413.16	3.86	included	present	688.91	7.13	included	present
229166_s_at	excluded	absent	6.41	-0.64	excluded	absent	4.54	-0.69	excluded	absent	48.32	0.61	excluded	absent	2.32	-0.76	excluded	absent	2.75	-0.75	excluded	absent	10.64	-0.51	excluded	absent	29.68	0.05	included	present	103.82	2.25	excluded	absent
229167_at	included	present	1177.52	-0.18	included	present	246.62	-2.47	included	present	900.16	-0.86	included	present	1078.27	-0.42	included	present	923.81	-0.8	included	present	885.26	-0.9	included	present	734.26	-1.27	included	present	846.29	-1	included	present
229168_at	excluded	absent	6.13	-0.83	excluded	absent	35.63	-0.04	excluded	absent	42.78	0.16	excluded	absent	24.4	-0.34	excluded	absent	4.71	-0.87	excluded	absent	44.33	0.2	excluded	absent	40.74	0.1	excluded	absent	43.86	0.19	excluded	absent
229169_at	included	absent	8.83	-0.78	included	absent	12.94	-0.76	included	absent	2.82	-0.8	included	absent	73.73	-0.53	included	absent	8.7	-0.78	included	absent	34.96	-0.68	included	absent	58.39	-0.59	included	absent	41.61	-0.65	included	absent
229170_s_at	included	present	95.88	-0.79	included	present	156.75	-0.66	included	present	159.25	-0.65	included	present	460.16	0.03	included	absent	133.35	-0.71	included	present	116.76	-0.75	included	present	325.36	-0.27	included	present	180.03	-0.6	included	present
229171_at	excluded	absent	135.28	1.31	excluded	absent	148.22	1.6	excluded	absent	75.57	-0.03	excluded	absent	113.38	0.82	excluded	absent	3.41	-1.64	excluded	present	95.01	0.41	excluded	present	130.75	1.21	included	present	144.53	1.52	included	present
229172_at	included	present	473.1	-0.6	included	present	2380.63	1.98	included	absent	171.48	-1.01	included	absent	52.72	-1.17	included	present	384.25	-0.72	included	present	3037.77	2.86	included	absent	481.22	-0.59	included	present	527.92	-0.53	included	present
229173_at	included	present	595.34	0.21	included	present	282.92	-1.43	included	present	810.81	1.34	included	present	3018.02	12.95	included	absent	455.98	-0.52	included	present	225.33	-1.73	included	present	897.94	1.8	included	present	646.67	0.48	included	present
229174_at	included	present	230.03	-0.17	included	present	148.34	-0.84	included	present	216.38	-0.29	included	present	197.04	-0.44	included	absent	112.55	-1.14	included	absent	473.47	1.83	included	present	385.37	1.1	included	present	424.98	1.43	included	present
229175_at	included	present	424.36	-0.48	included	present	461.42	-0.34	included	present	929.08	1.35	included	present	571.36	0.06	included	present	797.52	0.87	included	present	570.57	0.05	included	present	431.88	-0.45	included	present	408.28	-0.53	included	present
229176_at	included	present	316.08	1.26	included	present	160.16	-0.48	included	present	181.87	-0.24	included	present	202.23	-0.01	included	present	288.97	0.96	excluded	absent	251.06	0.53	included	present	283.57	0.9	included	present	259.85	0.63	included	present
229177_at	included	present	577.96	1	included	absent	50.05	-0.79	included	absent	82.22	-0.68	included	absent	95.98	-0.64	included	absent	4.53	-0.94	included	present	866.15	1.97	included	absent	55.89	-0.77	included	absent	11.36	-0.92	included	present
229178_at	included	present	115.68	0.61	included	present	127.04	0.76	included	present	281.24	2.88	excluded	absent	44.2	-0.38	excluded	absent	171.65	1.38	excluded	absent	83.88	0.17	included	present	174.09	1.41	included	present	181.94	1.52	included	present
229179_at	included	present	400.95	1.24	included	present	145.04	-1.54	included	present	417.74	1.42	included	present	270.1	-0.19	included	present	489.45	2.2	included	present	356.12	0.75	included	present	250.92	-0.39	included	present	446.78	1.73	included	present
229180_at	excluded	absent	44.9	-0.36	excluded	absent	4.71	-0.9	excluded	absent	68.9	-0.04	included	present	140.01	0.9	excluded	absent	72.74	0.01	excluded	absent	11.77	-0.8	excluded	absent	11.02	-0.81	excluded	absent	104.2	0.43	excluded	absent
229181_s_at	included	present	161.42	0.66	included	present	174.54	0.91	included	present	258.55	2.57	included	present	172.65	0.88	excluded	absent	164.5	0.72	included	present	224.33	1.89	included	present	198.3	1.38	included	present	529.43	7.9	included	present
229182_at	included	present	154.75	0.34	included	present	531.69	6.23	included	present	138.33	0.09	excluded	absent	127.6	-0.08	included	present	316.53	2.87	included	present	127.43	-0.08	included	present	276.17	2.24	included	present	404.08	4.24	included	present
229183_at	excluded	absent	11.5	-0.57	excluded	absent	7.67	-0.65	excluded	absent	9.59	-0.61	excluded	absent	9.88	-0.6	excluded	absent	92.64	1.29	excluded	absent	145.87	2.51	excluded	present	89.22	1.21	included	present	196.4	3.66	excluded	absent
229184_at	included	present	130.79	-0.91	included	present	111.16	-1.2	included	present	226.76	0.5	included	present	126.15	-0.98	included	absent	147.29	-0.67	included	absent	149.99	-0.63	included	present	172.4	-0.3	included	present	160.8	-0.47	included	present
229185_at	included	absent	98.31	-0.86	included	present	349.53	2.21	included	present	122.06	-0.57	included	present	182.38	0.17	included	present	322.35	1.88	included	present	17.46	-1.85	included	present	300.91	1.62	included	absent	62.37	-1.3	included	present
229186_s_at	excluded	absent	10.56	-1.28	excluded	absent	57.22	0.07	excluded	absent	44.48	-0.3	excluded	absent	3.07	-1.49	excluded	absent	61.99	0.21	excluded	absent	41.06	-0.4	included	present	76.56	0.63	excluded	absent	60.76	0.17	included	present
229187_at	included	present	4385.6	1.72	included	absent	164.05	-1.27	included	present	4919.25	2.09	included	present	4508.76	1.8	included	present	1436.27	-0.37	included	present	232.56	-1.22	included	present	4458.23	1.77	included	present	1934.68	-0.02	included	present
229188_s_at	included	present	327.27	-0.43	included	present	319.76	-0.48	included	present	372.35	-0.18	included	present	479.36	0.42	included	present	391.3	-0.07	included	present	271.97	-0.74	included	present	267.62	-0.77	included	present	426.8	0.12	included	present
229189_s_at	excluded	absent	75.41	-0.77	included	present	131.32	-0.06	excluded	absent	175.08	0.49	included	present	94.61	-0.53	excluded	absent	142.67	0.08	included	present	150.12	0.17	included	present	537.23	5.06	included	present	94.69	-0.53	included	present
229190_at	excluded	absent	23.63	-1.51	included	present	183.69	0.46	excluded	absent	69.97	-0.94	excluded	absent	85.12	-0.75	excluded	absent	178.53	0.39	excluded	absent	114.23	-0.39	included	present	485.47	4.16	excluded	absent	66.03	-0.99	excluded	absent
229191_at	excluded	absent	11.26	-1.22	excluded	absent	61.25	-0.31	excluded	absent	9.7	-1.25	excluded	absent	30.71	-0.87	excluded	absent	41.58	-0.67	excluded	absent	269.63	3.47	excluded	absent	162.25	1.52	excluded	absent	84.38	0.11	excluded	present
229192_s_at	excluded	absent	121.3	5.46	excluded	absent	117.69	5.27	excluded	absent	9.56	-0.4	excluded	absent	54.01	1.93	excluded	absent	4.05	-0.69	excluded	absent	132.56	6.05	excluded	absent	148.9	6.91	excluded	absent	135.98	6.23	excluded	absent
229193_at	included	present	553.65	0.01	included	present	587.19	0.15	included	present	2159.84	6.56	included	present	878.39	1.34	included	present	1482.9	3.8	included	present	235.81	-1.28	included	present	2660.23	8.6	included	present	510.94	-0.16	included	present
229194_at	included	present	798.49	-1.18	included	present	109.24	-3.12	included	present	288.46	-2.61	included	present	1702.79	1.37	included	present	360.48	-2.41	included	present	810.99	-1.14	included	present	340.95	-2.47	included	present	332.87	-2.49	included	present
229195_at	excluded	absent	45.69	-0.65	excluded	absent	110.64	0.5	excluded	absent	23.25	-1.05	excluded	absent	17.8	-1.15	excluded	absent	7.97	-1.32	excluded	absent	5.47	-1.37	excluded	absent	117.58	0.62	excluded	absent	73.02	-0.17	excluded	absent
229196_at	excluded	absent	37.99	-0.26	excluded	absent	66.72	0.46	excluded	absent	3.41	-1.14	excluded	absent	74.53	0.66	excluded	absent	9.02	-1	excluded	absent	84.69	0.92	excluded	absent	3.56	-1.14	excluded	absent	6.35	-1.07	excluded	absent
229197_at	excluded	absent	3.79	-1.38	excluded	absent	59.27	-0.39	excluded	absent	5.97	-1.35	excluded	absent	45.67	-0.63	excluded	absent	121.88	0.74	excluded	absent	89.53	0.16	excluded	absent	105.79	0.45	excluded	absent	93.53	0.23	included	present
229198_at	excluded	absent	313.93	1.55	excluded	absent	161.19	-0.13	excluded	absent	219	0.5	excluded	absent	94.5	-0.87	excluded	absent	492.62	3.51	excluded	absent	165.96	-0.08	excluded	absent	160.02	-0.15	included	present	311.42	1.52	excluded	absent
229199_at	included	present	1146.79	6.53	included	present	569.71	2.7	included	present	455.86	1.95	included	present	683.78	3.46	included	present	682.69	3.45	included	present	171.46	0.06	included	present	1289.45	7.48	included	present	422.08	1.72	included	present
229200_at	included	present	2018.78	1.44	included	present	7398.16	9.28	included	present	3552.66	3.68	included	present	4069.42	4.43	included	present	936.52	-0.14	included	present	1010.16	-0.03	included	present	476.66	-0.81	included	present	941.88	-0.13	included	present
229201_at	included	present	807.63	0.06	included	present	617.83	-0.13	included	present	240.66	-0.51	included	absent	20.23	-0.73	included	present	638.02	-0.11	included	present	137.64	-0.61	included	present	2143.48	1.41	included	present	284.96	-0.46	included	present
229202_at	included	present	762.37	-0.01	included	present	1539.36	1.55	included	present	675.23	-0.19	included	present	411.17	-0.72	included	present	634.41	-0.27	included	present	306.66	-0.93	included	present	543.7	-0.45	included	present	530.04	-0.48	included	present
229203_at	excluded	absent	28.61	-0.34	excluded	absent	31.77	-0.27	excluded	absent	42.58	-0.02	excluded	absent	24.31	-0.44	excluded	absent	5	-0.9	excluded	absent	23.71	-0.46	excluded	absent	58.53	0.36	excluded	absent	6.19	-0.87	excluded	absent
229204_at	included	present	3575.24	-1.47	included	present	4012.13	-1.04	included	present	3688.05	-1.36	included	present	2581.75	-2.44	included	present	10714.13	5.52	included	present	3343.99	-1.69	included	present	4410.8	-0.65	included	present	3965.48	-1.09	included	present
229205_at	included	absent	96.41	-0.35	included	present	633.4	0.73	included	present	201.44	-0.14	included	present	496.11	0.45	included	present	636.76	0.73	included	present	131.16	-0.28	included	present	558.74	0.58	included	present	565.35	0.59	included	present
229206_at	included	present	308.41	0.69	included	absent	206.02	-0.35	included	present	587.47	3.52	included	present	347.73	1.09	included	present	349.13	1.1	included	present	281.96	0.42	included	present	218.1	-0.23	included	present	120.3	-1.22	included	present
229207_x_at	excluded	absent	128.95	1.1	excluded	absent	4.38	-0.83	excluded	absent	81.31	0.36	excluded	absent	81.2	0.36	excluded	absent	66.68	0.14	excluded	absent	6.82	-0.79	excluded	absent	166.81	1.68	excluded	absent	39.4	-0.29	excluded	absent
229208_at	excluded	absent	152.06	1.32	excluded	absent	84.58	0.06	excluded	absent	58.83	-0.42	excluded	present	103.2	0.41	included	present	190.9	2.05	excluded	absent	69.87	-0.22	included	present	119.4	0.71	included	present	136.81	1.04	excluded	absent
229209_at	excluded	absent	6.53	-1.73	excluded	absent	149.94	1.26	excluded	absent	77.74	-0.24	excluded	absent	83.52	-0.12	excluded	absent	3.8	-1.78	excluded	absent	13.42	-1.58	included	present	82.75	-0.14	included	present	135.75	0.97	included	present
229210_at	included	present	213.55	-0.57	included	absent	80.71	-1.31	included	present	260.33	-0.31	included	absent	107.67	-1.16	included	present	293.61	-0.13	included	present	252.83	-0.35	included	present	270.44	-0.25	included	present	414.03	0.55	excluded	absent
229211_at	included	present	144.67	-1.33	included	present	462	2.76	included	present	111.19	-1.77	included	present	313.77	0.85	included	absent	97.36	-1.94	included	present	348.24	1.29	included	present	328.99	1.04	included	present	247.99	0	included	present
229212_at	included	present	2589.91	4.45	included	present	3191.75	6.73	included	present	2710.7	4.91	included	present	2596.94	4.47	included	present	2422.76	3.81	included	present	1407.25	-0.04	included	present	3520.55	7.98	included	present	3605.88	8.3	included	present
229213_at	included	present	360.68	0.31	included	present	232.05	-0.66	included	present	183.47	-1.02	included	present	219.31	-0.75	included	present	203.76	-0.87	included	present	245.18	-0.56	included	present	565.83	1.86	included	present	746.17	3.21	included	present
229214_at	included	present	626.27	-0.21	included	absent	104.39	-1.13	included	present	479.31	-0.47	included	present	294.25	-0.8	included	present	455.96	-0.51	included	present	373.02	-0.66	included	present	130.16	-1.09	included	present	271.63	-0.84	included	present
229215_at	excluded	absent	120.35	0.48	excluded	absent	35.14	-0.52	excluded	absent	12.54	-0.78	excluded	absent	2.5	-0.9	excluded	absent	6.4	-0.86	excluded	absent	181.85	1.21	excluded	absent	8.01	-0.84	excluded	absent	3.65	-0.89	excluded	absent
229216_s_at	included	present	2089.59	0.75	included	present	3173.14	2.96	included	present	2806.33	2.21	included	present	3110.7	2.83	included	present	3052.21	2.72	included	present	1886.72	0.34	included	present	2628.3	1.85	included	present	3708.04	4.05	included	present
229217_at	excluded	absent	94.13	0.4	excluded	absent	27.09	-0.71	excluded	absent	93.84	0.4	excluded	absent	123.95	0.9	excluded	absent	153.75	1.39	excluded	absent	154.35	1.4	excluded	absent	156.35	1.43	excluded	absent	42.41	-0.45	excluded	absent
229218_at	included	present	899.44	0.08	included	present	195.34	-0.57	included	absent	70.63	-0.68	included	absent	93.62	-0.66	included	absent	198.29	-0.57	included	present	3496.26	2.45	included	present	65.78	-0.69	included	absent	2.3	-0.74	included	present
229219_s_at	included	present	432.73	0.76	included	present	226.53	-0.72	included	present	231.64	-0.68	included	absent	121.01	-1.47	included	present	499.04	1.24	included	present	444.33	0.85	included	present	205.74	-0.86	included	present	327.68	0.01	included	present
229220_x_at	included	present	247.94	-0.94	included	present	461.77	0.39	included	present	509.74	0.69	included	present	892.09	3.06	included	present	367.19	-0.2	included	present	614.9	1.34	included	present	555.55	0.97	included	present	520.55	0.75	included	present
229221_at	included	present	129.58	-0.38	excluded	absent	2.88	-0.74	excluded	absent	125.14	-0.39	excluded	absent	2.68	-0.74	excluded	absent	110.06	-0.44	included	present	184.23	-0.22	included	present	155.62	-0.31	excluded	absent	45.08	-0.62	included	present
229222_at	included	present	865.45	-0.23	included	absent	66.58	-1.14	included	present	154.92	-1.04	included	absent	11.68	-1.21	included	absent	42.34	-1.17	included	present	352.65	-0.81	included	absent	55.22	-1.16	included	absent	13.41	-1.2	included	present
229223_at	included	absent	74.03	-0.63	included	absent	111.55	-0.37	included	present	126.03	-0.27	included	present	183.12	0.13	included	absent	121.64	-0.3	included	absent	97.57	-0.47	included	present	124.16	-0.28	included	present	66.55	-0.68	included	present
229224_x_at	excluded	absent	39.04	-0.2	excluded	absent	106.68	1.59	excluded	absent	2.1	-1.18	excluded	absent	1.92	-1.18	excluded	absent	41.04	-0.14	excluded	absent	48.8	0.06	excluded	absent	79.3	0.87	included	present	69.38	0.61	excluded	absent
229225_at	included	present	4946.17	5.41	included	present	1962.87	1.45	included	present	3105.16	2.97	included	present	259.19	-0.81	included	present	2600.13	2.3	included	present	1539.54	0.89	included	present	3034.46	2.88	included	present	1136.67	0.36	included	present
229226_at	included	present	273.92	-0.44	included	absent	142.78	-1.16	included	present	370.24	0.09	included	present	210.08	-0.79	included	present	321.68	-0.17	included	present	291	-0.34	included	present	326.43	-0.15	included	present	153.63	-1.1	included	present
229227_at	included	present	326.37	-0.74	included	present	740.74	0.47	included	present	239.37	-1	included	present	491.48	-0.26	included	present	479.85	-0.3	included	present	216.84	-1.06	included	present	344.75	-0.69	included	absent	113.63	-1.37	included	present
229228_at	included	present	1908.55	0.04	included	present	495.78	-0.67	included	present	2439.71	0.31	included	present	668.86	-0.59	included	present	9141.98	3.72	included	present	979.29	-0.43	included	present	2566.46	0.38	included	present	302.31	-0.77	included	present
229229_at	excluded	absent	3.8	-0.2	excluded	absent	1.42	-0.2	excluded	absent	55.04	-0.18	excluded	absent	33.03	-0.19	excluded	absent	1.66	-0.2	excluded	absent	9.73	-0.2	excluded	absent	2.03	-0.2	excluded	absent	1.25	-0.2	excluded	absent
229230_at	included	present	113.08	-0.15	excluded	absent	126.72	-0.1	excluded	absent	5.18	-0.52	excluded	absent	3.13	-0.53	excluded	absent	5.1	-0.52	excluded	absent	44.8	-0.38	excluded	absent	2.54	-0.53	excluded	absent	33.69	-0.42	excluded	absent
229231_at	included	present	1121.37	-0.74	included	present	1965.01	0.42	included	present	1195.28	-0.64	included	present	1612.97	-0.07	included	present	3360.8	2.33	included	present	936.23	-0.99	included	present	1797.81	0.19	included	present	2042.43	0.52	included	present
229232_at	included	present	1294.84	5.64	included	present	1216.71	5.12	included	present	1066.28	4.13	included	present	1090.94	4.3	included	present	641.58	1.34	included	present	719.64	1.85	included	present	971.18	3.51	included	present	1005.33	3.73	included	present
229233_at	included	present	302.26	-0.53	included	present	178.54	-0.63	included	present	204.58	-0.61	included	absent	2.05	-0.78	included	present	413.71	-0.44	included	absent	63.76	-0.73	included	present	664.97	-0.22	included	absent	75.67	-0.72	included	present
229234_at	included	present	127.32	-0.41	included	absent	67.94	-0.91	included	present	96.55	-0.67	included	absent	201.05	0.21	included	absent	67.55	-0.92	included	absent	92.54	-0.7	included	present	135.65	-0.34	included	absent	43.13	-1.12	included	present
229235_at	included	present	278.74	-0.32	included	present	539.95	1.46	included	present	318.14	-0.05	included	present	359.12	0.23	included	present	378.83	0.36	included	present	687.51	2.47	included	present	522.16	1.34	included	present	489.11	1.11	included	present
229236_s_at	included	present	229.55	-0.01	included	present	168.14	-0.66	included	present	286.45	0.59	included	present	246.57	0.17	included	present	168.42	-0.66	included	present	277.03	0.49	included	present	397.1	1.77	included	present	879.21	6.9	included	present
229237_s_at	excluded	absent	84.94	-0.68	excluded	absent	116.75	-0.37	excluded	absent	225.39	0.66	excluded	absent	77.66	-0.75	excluded	absent	131.21	-0.24	excluded	absent	384.32	2.17	excluded	absent	133.04	-0.22	included	present	448.57	2.78	included	present
229238_at	included	present	805.78	0.44	included	present	1332.31	1.27	included	present	378.4	-0.23	included	present	387.34	-0.22	included	present	307.56	-0.34	included	present	55.9	-0.74	included	present	727.62	0.32	included	present	362.88	-0.25	included	present
229239_x_at	excluded	absent	36.88	-0.57	excluded	absent	27.8	-0.67	excluded	absent	16.78	-0.8	excluded	absent	14.59	-0.83	excluded	absent	24.79	-0.71	excluded	absent	224.53	1.56	excluded	absent	67.91	-0.22	excluded	absent	77.44	-0.11	excluded	absent

229517_at	included	present	706.65	1.09	included	present	188.95	-0.5	included	present	265.36	-0.26	included	present	442.23	0.28	included	present	396.26	0.14	included	present	254.29	-0.3	included	present	159.07	-0.59	included	present	312.91	-0.12	included	present	224.41	-0.39	included	present	217.32	-0.41	included	present	459.48	0.33	included	present	375.97	0.08	included	present	386.26	0.11	included	present	560.91	0.65	included	present	259.4	-0.28
229518_at	included	present	103.19	-0.39	excluded	absent	3.02	-0.56	excluded	absent	16.92	-0.53	excluded	absent	82.64	-0.43	excluded	absent	67.51	-0.45	included	present	632.9	0.47	excluded	absent	3.71	-0.56	excluded	absent	22.59	-0.52	excluded	absent	83.26	-0.43	excluded	absent	61.56	-0.46	excluded	absent	45.8	-0.49	excluded	absent	3.63	-0.56	excluded	absent	47.71	-0.48	excluded	absent	3.01	-0.56	included	present	76.15	-0.44
229519_at	included	present	1820.11	-0.91	included	present	2675.01	0.38	included	present	4127.19	2.58	included	present	3083.13	1	included	present	1828.76	-0.89	included	present	1698.74	-1.09	included	present	2683.96	0.4	included	present	2046.87	-0.56	included	present	2001.45	-0.63	included	present	1590.51	-1.25	included	present	1963.73	-0.69	included	present	3157.19	1.11	included	present	2573.07	0.23	included	present	2544.77	0.19	included	present	1179.26	-1.87
229520_s_at	included	present	254.18	-0.57	included	present	246.45	-0.62	included	present	162.5	-1.18	included	present	274.82	-0.43	included	present	202.44	-0.91	included	present	95.51	-1.62	included	present	358.46	0.12	included	present	207.6	-0.88	included	present	482.68	0.95	included	present	139.53	-1.33	included	present	227.89	-0.74	included	present	486.32	0.97	included	present	167.46	-1.15	included	present	248.38	-0.61	included	present	107.79	-1.54
229521_at	included	present	282.73	0.23	included	present	579.11	1.32	included	present	158.23	-0.22	included	present	233.9	0.06	included	present	322.76	0.38	included	present	629.28	1.5	included	present	126.44	-0.34	included	present	381.11	0.59	included	present	327.51	0.4	included	present	225.4	0.02	included	present	322.02	0.38	included	present	836.48	2.26	included	present	328.62	0.4	included	present	354.97	0.5	included	present	448.41	0.84
229522_at	excluded	absent	6.88	-0.71	excluded	absent	28.19	-0.57	excluded	absent	3.67	-0.74	included	present	260.15	1.03	excluded	absent	4.31	-0.73	excluded	absent	6.42	-0.72	excluded	absent	37.52	-0.5	excluded	absent	1.55	-0.75	excluded	absent	6.54	-0.72	excluded	absent	7.02	-0.71	excluded	absent	3.18	-0.74	excluded	absent	47.78	-0.43	excluded	absent	3.42	-0.74	excluded	absent	18.54	-0.63	excluded	absent	3.75	-0.74
229523_at	included	present	242.2	-0.27	included	present	115.99	-0.66	included	absent	60.65	-0.83	included	absent	49.57	-0.86	included	absent	19.5	-0.96	included	absent	12.78	-0.98	included	absent	50.21	-0.86	included	present	122.97	-0.64	included	absent	108.39	-0.68	included	present	144.42	-0.57	included	present	135.2	-0.6	included	absent	71.05	-0.8	included	present	128.41	-0.62	included	present	167.27	-0.5	included	absent	49.31	-0.86
229524_at	excluded	absent	138.53	0.12	excluded	absent	171.53	0.58	excluded	absent	115.26	-0.21	excluded	absent	151.04	0.29	excluded	absent	155.45	0.35	included	present	439.75	4.37	excluded	absent	169.96	0.56	included	present	262.83	1.87	included	present	326.2	2.77	included	present	219.69	1.26	included	present	245.78	1.63	excluded	absent	112.67	-0.25	excluded	absent	93.88	-0.51	excluded	absent	59.14	-1.01	included	present	245.41	1.62
229525_at	included	present	637.83	-0.38	included	present	690.25	-0.16	included	present	1326.63	2.52	included	present	475.31	-1.06	included	present	1145.76	1.76	included	present	1051.67	1.36	included	present	730.7	0.01	included	present	993.4	1.12	included	present	500.58	-0.96	included	present	1085.04	1.5	included	present	787.68	0.25	included	present	667.97	-0.25	included	present	992.84	1.12	included	present	656.58	-0.3	included	present	661.36	-0.28
229526_at	included	present	266.44	-0.08	included	present	142.3	-0.47	included	present	172.29	-0.38	included	present	185.66	-0.33	included	absent	165.96	-0.4	included	absent	124.99	-0.53	included	present	232.07	-0.19	included	present	213.5	-0.24	included	present	230.88	-0.19	included	present	161.77	-0.41	included	present	178.99	-0.35	included	absent	91.43	-0.63	included	present	250.14	-0.13	included	present	154.7	-0.43	included	present	168.45	-0.39
229527_s_at	included	present	180.93	-1.35	included	present	298.87	-0.76	included	present	348.96	-0.51	included	absent	175.17	-1.38	included	present	246.66	-1.02	included	present	344.41	-0.53	included	present	425.76	-0.12	included	present	498.47	0.24	included	present	534.58	0.42	included	present	204.25	-1.24	included	present	387.32	-0.32	included	present	679.75	1.15	included	absent	266.25	-0.92	included	present	138.39	-1.57	included	present	492.1	0.21
229528_at	included	present	391.22	1.67	included	present	259.69	0.23	included	present	253.94	0.16	included	present	391.88	1.68	included	present	468.58	2.52	included	present	343.92	1.15	included	present	1049.19	8.88	included	present	798.03	6.13	included	present	779.68	5.93	included	present	570.02	3.63	included	present	1728.05	16.33	included	present	304.05	0.71	included	present	1279.5	11.41	included	present	1324.12	11.9	included	present	1455.58	13.34
229529_at	excluded	absent	5.12	-0.41	excluded	absent	15.5	-0.37	excluded	absent	13.87	-0.37	excluded	absent	9.49	-0.39	excluded	absent	7.74	-0.4	excluded	absent	28.13	-0.31	excluded	absent	2.52	-0.43	excluded	absent	4.21	-0.42	excluded	absent	11.18	-0.39	excluded	absent	1.22	-0.43	excluded	absent	6.64	-0.41	excluded	absent	12.28	-0.38	excluded	absent	11.1	-0.39	excluded	absent	7.98	-0.4	excluded	absent	1.08	-0.43
229530_at	included	present	414.24	-0.95	included	present	615.34	-0.75	included	present	619.46	-0.74	included	present	749.89	-0.61	included	present	1048.48	-0.31	included	present	2918.15	1.56	included	present	289.26	-1.07	included	present	1457.45	0.1	included	present	2205.34	0.85	included	present	728.69	-0.63	included	present	724.79	-0.64	included	present	629.48	-0.73	included	present	2045.95	0.69	included	present	2522.83	1.16	included	present	200.81	-1.16
229531_at	included	present	3530.48	0.36	included	present	1463.27	-0.99	included	present	1387.38	-1.03	included	present	2092.12	-0.58	included	present	2341.35	-0.41	included	present	1741.14	-0.8	included	present	2252.74	-0.47	included	present	1546.05	-0.93	included	present	2019.01	-0.62	included	present	2465.66	-0.33	included	present	1923.04	-0.69	included	present	5633.42	1.73	included	present	8347.35	3.5	included	present	8476.43	3.58	included	present	1929.93	-0.68
229532_at	included	present	306.05	-0.17	included	present	724.05	2.54	included	present	460.43	0.83	included	present	523.32	1.24	included	present	429.62	0.63	included	present	276.18	-0.37	included	present	421.73	0.58	included	present	357.36	0.16	included	present	269.25	-0.41	included	present	233.75	-0.64	included	present	527.73	1.27	included	present	270.57	-0.4	included	present	651.02	2.07	included	present	464.99	0.86	included	present	326.51	-0.04
229533_x_at	included	present	394.75	-1.87	included	present	767.04	-0.5	included	present	925.21	0.08	included	present	2132.46	4.52	included	present	815.55	-0.32	included	present	871.15	-0.12	included	present	778.43	-0.46	included	present	487.11	-1.53	included	present	763.04	-0.52	included	present	656.04	-0.91	included	present	2838	7.12	included	present	802.49	-0.37	included	present	1183.3	1.03	included	present	980.52	0.28	included	present	1131.35	0.84
229534_at	excluded	absent	76.68	-0.25	excluded	absent	18.95	-0.74	excluded	absent	3.17	-0.87	included	present	90.03	-0.13	excluded	absent	3.32	-0.87	excluded	absent	76.65	-0.25	excluded	absent	36.11	-0.59	excluded	absent	112.5	0.06	excluded	absent	26.99	-0.67	excluded	absent	14.92	-0.77	excluded	absent	40.35	-0.56	excluded	absent	90.41	-0.13	excluded	absent	46.27	-0.51	excluded	absent	7.82	-0.83	included	present	77.86	-0.24
229535_at	included	present	179.14	-1.28	included	present	346.23	1.31	included	present	177.55	-1.31	included	present	201.27	-0.94	included	absent	150.46	-1.73	included	present	231.87	-0.46	included	present	297.16	0.55	included	present	144.58	-1.82	included	present	436.15	2.71	included	present	278.44	0.26	included	present	512.45	3.9	included	present	251.6	-0.16	included	present	159.05	-1.6	included	present	143.18	-1.84	included	present	482.8	3.44
229536_at	excluded	absent	25.19	-0.42	excluded	absent	109.79	1.33	excluded	absent	127.46	1.69	excluded	absent	62.94	0.36	excluded	absent	158.59	2.34	excluded	absent	41.78	-0.08	excluded	absent	98.09	1.09	included	present	196.44	3.12	included	present	116.97	1.48	included	present	105.04	1.23	included	present	443.61	8.23	excluded	absent	97.96	1.08	excluded	absent	59.09	0.28	excluded	absent	61.37	0.33	included	present	498.37	9.36
229537_at	included	present	97.27	-0.54	included	present	204.04	-0.12	included	present	108.05	-0.5	included	present	133	-0.4	included	absent	68.47	-0.65	included	present	132.07	-0.4	included	absent	62.96	-0.67	included	present	48.77	-0.73	included	present	166.18	-0.27	included	absent	43.11	-0.75	included	absent	2.23	-0.91	included	absent	27.83	-0.81	included	present	194	-0.16	included	present	80.06	-0.61	included	present	241.24	0.03
229538_s_at	included	present	136.84	0.14	included	present	1458.67	8.51	included	present	1787.63	10.59	included	present	999.6	5.6	included	present	463.98	2.21	included	present	228.75	0.73	included	present	135.64	0.14	included	present	1230.68	7.07	included	present	156.31	0.27	included	present	100.43	-0.09	included	present	779.16	4.21	included	present	578.11	2.94	included	present	1147.18	6.54	included	present	3214.99	19.62	included	present	987.69	5.53
229539_at	included	present	642.4	1.92	included	present	484.04	0.93	included	present	175.48	-0.99	included	present	140.6	-1.21	included	present	662.94	2.05	included	present	319.23	-0.1	included	present	478.17	0.9	included	present	690.01	2.22	included	present	266.16	-0.43	included	present	508.16	1.08	included	present	399.05	0.4	included	present	387.64	0.33	included	present	270.05	-0.4	included	present	659.84	2.03	included	present	362.43	0.17
229540_at	excluded	absent	67.92	-0.41	excluded	absent	71.64	-0.34	excluded	absent	81.54	-0.15	included	present	103.87	0.28	excluded	absent	66.38	-0.44	excluded	absent	68.58	-0.4	included	present	62.26	-0.52	excluded	absent	32.1	-1.09	included	present	95.03	0.11	included	present	96.47	0.14	included	present	82.95	-0.12	excluded	absent	60.66	-0.55	excluded	absent	35.31	-1.03	excluded	absent	58.48	-0.59	excluded	absent	25.66	-1.22
229541_at	included	present	3145.43	1.6	included	present	914.13	-2.24	included	present	6766.15	7.84	included	present	1638.11	-0.99	included	present	3611.33	2.4	included	present	1292.28	-1.59	included	present	5551.85	5.75	included	present	2207.76	-0.01	included	present	2530.65	0.54	included	present	2105.92	-0.19	included	present	2269.76	0.09	included	present	481.91	-2.99	included	present	3306.26	1.88	included	present	1951.56	-0.45	included	present	2780.12	0.97
229542_at	excluded	absent	17.87	-0.25	excluded	absent	2.52	-0.3	excluded	absent	2.24	-0.3	excluded	absent	60.33	-0.12	excluded	absent	3.76	-0.3	excluded	absent	106.98	0.02	excluded	absent	4.22	-0.3	excluded	absent	1.5	-0.31	excluded	absent	8.39	-0.28	excluded	absent	1.91	-0.3	excluded	absent	15.63	-0.26	excluded	absent	5.25	-0.29	excluded	absent	41.23	-0.18	excluded	absent	4.75	-0.3	excluded	absent	13.8	-0.27
229543_at	included	present	416.5	-0.15	included	present	327.59	-0.23	included	present	290.11	-0.26	included	absent	31.43	-0.48	included	present	525.61	-0.06	included	present	1141.05	0.46	included	present	409.85	-0.16	included	present	333.86	-0.22	included	present	590.09	-0.01	included	present	109.38	-0.42	included	present	304.21	-0.25	included	present	1665.18	0.91	included	present	1564.59	0.82	included	present	923.2	0.28	included	present	1868.13	1.08
229544_at	included	present	398.82	0.42	included	present	241.75	-0.38	included	present	910.22	3.03	included	present	337.98	0.11	included	present	277.94	-0.19	included	present	209.44	-0.54	included	present	1860.61	7.88	included	present	212.48	-0.53	included	present	565.26	1.27	included	present	219.86	-0.49	included	present	904.63	3	included	present	604.69	1.47	included	present	1356.33	5.31	included	present	551.26	1.2	included	present	1508.85	6.08
229545_at	excluded	absent	52.82	-0.76	excluded	absent	115.9	-0.45	excluded	absent	65.67	-0.69	excluded	absent	97.63	-0.54	excluded	absent	91.2	-0.57	excluded	absent	16.59	-0.94	excluded	absent	46.16	-0.79	included	present	237.39	0.15	excluded	absent	55.84	-0.74	excluded	absent	42.9	-0.81	excluded	absent	58.8	-0.73	excluded	absent	14.7	-0.95	excluded	absent	32.14	-0.86	included	present	171.58	-0.17	excluded	absent	33.54	-0.85
229546_at	excluded	absent	6.28	-1.08	excluded	absent	49.7	-0.45	excluded	absent	42.75	-0.55	excluded	absent	35.05	-0.67	excluded	absent	117.92	0.53	excluded	absent	4.1	-1.11	included	present	109.9	0.42	excluded	absent	32.32	-0.7	included	present	45.86	-0.51	included	present	301.66	3.19	included	present	122.05	0.59	included	present	227	2.11	included	present	169.38	1.27	included	present	1195.21	16.09	included	present	84.04	0.04
229547_s_at	included	present	325.97	0.57	included	absent	180.49	-1.34	included	present	296.28	0.18	included	present	251.08	-0.42	excluded	absent	348.77	0.87	included	absent	166.15	-1.53	included	present	342.58	0.79	included	present	338.51	0.73	included	present	362.26	1.05	included	present	324.88	0.55	included	present	410.91	1.68	included	absent	269.38	-0.18	included	present	272.44	-0.13	included	absent	105.67	-2.33	included	present	380.12	1.28
229548_at	excluded	absent	10.92	-1.33	excluded	absent	152.97	0.01	excluded	absent	122.91	-0.27	included	present	177.08	0.24	excluded	absent	132.82	-0.18	included	present	231.18	0.75	included	present	173.14	0.2	included	present	88.52	-0.6	excluded	absent	30.85	-1.14	included	present	140.37	-0.11	excluded	absent	127.61	-0.23	included	present	309.5	1.49	excluded	absent	26.73	-1.18	included	present	275.22	1.17	included	present	108.51	-0.41
229549_at	included	present	899.54	0.76	included	present	282.97	-0.61	included	present	380.96	-0.39	included	present	360.85	-0.44	included	present	669.18	0.25	included	present	992.09	0.97	included	present	1249.54	1.54	included	present	601.19	0.1	included	present	373.63	-0.41	included	present	432.84	-0.28	included	present	1218.11	1.47	included	present	463.65	-0.21	included	present	826.59	0.6	included	present	1361.9	1.79	included	present	1159.12	1.34
229550_at	excluded	absent	90.23	-0.14	included	present	253.15	1.18	included	present	197.64	0.73	included	present	220.32	0.91	excluded	absent	215.21	0.87	excluded	absent	24.89	-0.66	included	present	209.31	0.82	included	present	637.97	4.28	excluded	absent	153.66	0.37	included	present	317.79	1.7	included	present	467.61	2.9	included	present	382.31	2.22	included	present	215.87	0.88	included	present	277.34	1.37	included	present	179.35	0.58
[illegible]																																																													

229876_at	included	present	217.14	-0.44	included	present	109.35	-0.56	included	present	299.25	-0.35	included	present	529.95	-0.09	included	absent	80.94
229877_at	included	absent	80.4	-1.02	included	present	205.93	-0.26	included	absent	121.21	-0.77	included	present	144.41	-0.63	included	absent	152.18
229878_at	included	present	533.44	-0.63	included	present	1160.4	1.55	included	present	1052.19	1.17	included	present	1065.46	1.22	included	present	406.71
229879_at	included	present	560.08	0.17	included	absent	115.27	-1.06	included	present	344.54	-0.43	included	present	358.3	-0.39	included	present	344.41
229880_at	excluded	absent	62.58	-0.35	included	present	234.55	1.21	included	present	255.68	1.41	excluded	absent	4	-0.89	excluded	absent	126.2
229881_at	included	present	501.49	2.99	included	present	388.63	1.96	included	present	496.34	2.94	included	present	194.79	0.18	included	present	705.31
229882_at	excluded	absent	106.88	-0.64	excluded	absent	80.19	-0.93	excluded	absent	128.55	-0.4	excluded	absent	105.87	-0.65	included	present	188.51
229883_at	excluded	absent	57.55	-0.35	included	present	812.54	12.52	included	present	99.45	0.37	included	present	162.72	1.45	excluded	absent	8.38
229884_s_at	included	present	233.3	-0.49	included	present	257.83	-0.25	included	present	113.52	-1.66	included	present	215.51	-0.66	included	present	306.35
229885_at	included	present	275.81	0.01	included	present	401.46	1.05	included	present	358.94	0.7	included	present	688.84	3.43	included	absent	202.08
229886_at	excluded	absent	71.97	-0.44	included	present	106.95	0.11	included	present	151.14	0.81	included	present	251.98	2.39	excluded	absent	93.16
229887_at	included	present	123.46	-0.4	included	present	114.33	-0.44	included	absent	2.63	-0.87	included	absent	3.86	-0.87	included	absent	4.31
229888_at	excluded	absent	125.11	0.13	excluded	absent	77.23	-0.09	excluded	absent	65.44	-0.15	included	present	84.98	-0.06	excluded	absent	119.62
229889_at	included	present	171.57	-0.21	included	present	247.28	0.05	included	present	139.62	-0.32	included	present	482.9	0.87	included	present	369.28
229890_at	included	present	119.84	-0.56	included	present	243.29	-0.23	included	absent	52.14	-0.75	included	absent	63.27	-0.72	included	absent	23.37
229891_x_at	included	present	468.22	-0.86	included	present	554.53	-0.57	included	present	529.61	-0.65	included	present	538.56	-0.62	included	present	941
229892_at	included	present	328.36	-0.13	included	present	350.51	-0.1	included	absent	33.23	-0.49	included	present	517.23	0.11	included	present	263.62
229893_at	included	present	555.15	0.57	included	present	492.8	0.35	included	present	161.9	-0.82	included	present	668.78	0.97	included	present	254.02
229894_s_at	excluded	absent	60.27	0.35	excluded	absent	13.11	-0.57	excluded	absent	4.38	-0.74	excluded	absent	5.45	-0.72	excluded	absent	60.81
229895_s_at	included	present	267.7	0.13	included	absent	203.92	-0.4	included	present	322.54	0.58	included	present	278.54	0.22	included	present	230.27
229896_at	included	present	2548.77	1.17	included	present	1787.28	-0.1	included	present	3335.82	2.47	included	present	3447.51	2.66	included	present	5763.6
229897_at	included	present	205.76	-0.45	included	present	149.36	-0.97	included	present	73.03	-1.68	included	present	236.96	-0.16	included	present	241.08
229898_at	included	present	748.51	-0.55	included	present	266.81	-1.2	included	present	411.09	-1.01	included	present	368.57	-1.06	included	present	301.35
229899_s_at	included	absent	61.77	-1.57	included	present	113.14	-1.03	included	present	219.43	0.09	included	present	183.16	-0.29	included	present	298.03
229900_at	included	present	374.37	-0.31	included	absent	82.68	-1.03	included	present	135.15	-0.9	included	present	161.51	-0.84	included	present	313.26
229901_at	excluded	absent	4.6	-0.6	excluded	absent	4.01	-0.61	excluded	absent	2.69	-0.63	excluded	absent	183.53	1.71	excluded	absent	70.62
229902_at	excluded	absent	68.83	0.34	excluded	absent	65.46	0.27	excluded	absent	2.08	-1.06	excluded	absent	30.55	-0.46	excluded	absent	58.8
229903_x_at	included	present	1127.93	-0.98	included	present	1498.09	-0.28	included	present	1570.73	-0.14	included	present	2075.57	0.81	included	present	1903.8
229904_at	excluded	absent	15.14	-0.88	excluded	absent	7.62	-0.96	excluded	absent	11.69	-0.92	excluded	absent	28.54	-0.73	excluded	absent	18.72
229905_at	included	present	3772.94	1.44	included	present	339.2	-1.08	included	present	1153.58	-0.48	included	present	992.08	-0.6	included	present	1085.31
229906_at	included	present	308.78	1.47	included	present	426.49	2.96	included	present	166.45	-0.33	included	present	205.48	0.17	included	present	404.35
229907_at	included	absent	94.89	-0.51	included	present	128	-0.14	included	absent	118.98	-0.24	included	absent	6.17	-1.48	included	present	334.13
229908_s_at	included	present	287.79	-0.4	included	present	497.92	1.17	included	present	159.01	-1.36	included	present	249.7	-0.68	included	absent	223.36
229909_at	excluded	absent	78.95	-0.37	excluded	absent	6.93	-0.82	excluded	absent	3.85	-0.84	excluded	absent	85.84	-0.33	excluded	absent	17.26
229910_at	included	present	281.48	-0.46	included	present	207.56	-0.65	included	absent	85.72	-0.96	included	present	305.54	-0.4	included	present	175.62
229911_at	excluded	absent	16.79	-0.42	excluded	absent	19.33	-0.35	excluded	absent	13.84	-0.51	excluded	absent	8.61	-0.67	excluded	absent	18.15
229912_at	included	present	258.9	1.3	included	present	669.82	4.96	excluded	absent	6.44	-0.94	included	present	336.57	1.99	excluded	absent	94.46
229913_at	excluded	absent	75.9	-0.26	excluded	absent	9.88	-0.5	excluded	absent	108.55	-0.15	excluded	absent	68.33	-0.29	excluded	absent	22.98
229914_at	included	present	1039.7	-0.68	included	present	2209.72	0.61	included	present	1525.9	-0.14	included	present	1807.01	0.17	included	present	1392.32
229915_at	excluded	absent	40.54	-0.17	excluded	absent	58.86	0.24	excluded	absent	12.18	-0.81	excluded	absent	30.55	-0.4	excluded	absent	8.08
229916_at	excluded	absent	84.75	-0.13	excluded	absent	67.02	-0.22	excluded	absent	75.22	-0.18	excluded	absent	6.32	-0.53	excluded	absent	55.42
229917_at	excluded	absent	130.97	-0.5	included	present	1571.84	2.05	included	present	194.22	-0.39	excluded	absent	59.69	-0.63	included	present	370.07
229918_at	excluded	absent	8.44	-1.34	included	present	223.99	4.1	excluded	absent	53.17	-0.21	excluded	absent	46.53	-0.38	excluded	absent	6.51
229919_at	excluded	absent	125.51	1.1	excluded	absent	166.23	2.07	included	present	164.4	2.03	excluded	absent	115.34	0.86	included	present	150.73
229920_at	excluded	absent	52.68	-0.74	excluded	absent	94.48	0	excluded	absent	10.55	-1.49	excluded	absent	24.93	-1.23	excluded	absent	64.16
229921_at	included	present	8222.82	0.52	included	present	6253.8	0.24	included	present	2910.49	-0.24	included	present	131.87	-0.63	included	present	4338.67
229922_at	excluded	absent	18.81	0.13	excluded	absent	9.24	-0.27	excluded	absent	11.71	-0.17	excluded	absent	5.91	-0.41	excluded	absent	7.14
229923_at	included	present	217.39	-0.99	included	present	206.87	-1.07	included	present	344.96	-0.11	included	present	174.2	-1.29	included	present	325.39
[illegible]																			

230235_at	included	present	469.01	-0.3	included	present	256.37	-1.26	included	present	357.48	-0.8	included	present	480.24	-0.25	included	present	252.6	-1.28	included	present	216.6	-1.45	included	present	752.87	0.99	included	present	426.06	-0.49	included	present	728.79	0.88	included	present	624.28	0.41	included	present	601.25	0.3	included	present	852.41	1.45	included	present	898.69	1.66	included	present	1510.86	4.44	included	present	847.27	1.42
230236_at	excluded	absent	4.97	-0.32	excluded	absent	102.71	-0.07	excluded	absent	40.8	-0.23	excluded	absent	1.94	-0.33	excluded	absent	5.74	-0.32	excluded	absent	2.89	-0.33	excluded	absent	57.91	-0.19	excluded	absent	7.04	-0.32	excluded	absent	5.78	-0.32	excluded	absent	23.5	-0.27	excluded	absent	9.6	-0.31	excluded	absent	6.29	-0.32	included	present	80.52	-0.13	excluded	absent	45.57	-0.22	included	present	27.41	-0.26
230237_at	included	present	1513.77	6.34	excluded	absent	130.9	0	included	present	148.86	0.09	included	present	5515.75	24.67	included	present	135.46	0.03	excluded	absent	26.43	-0.47	excluded	absent	9.36	-0.55	excluded	absent	2.14	-0.59	excluded	absent	4.71	-0.57	included	present	1640.34	6.92	included	present	449.76	1.47	excluded	absent	1.73	-0.59	excluded	absent	60.52	-0.32	excluded	absent	1.88	-0.59	excluded	absent	2.63	-0.58
230238_at	included	absent	40.23	-0.58	included	present	332.21	-0.5	included	absent	31.86	-0.59	included	present	440.93	-0.46	included	present	293.1	-0.51	included	absent	98.82	-0.57	included	present	126.01	-0.56	included	absent	14.77	-0.59	included	present	222.41	-0.53	included	present	78.65	-0.57	included	absent	63.21	-0.58	included	present	521.42	-0.44	included	present	235.36	-0.52	included	present	262.82	-0.52	included	present	658.14	-0.4
230239_at	excluded	absent	152.1	0.36	excluded	absent	37.11	-1.27	excluded	absent	10.91	-1.64	excluded	absent	29.05	-1.38	excluded	absent	160.38	0.47	excluded	absent	80.42	-0.65	excluded	absent	102.89	-0.34	excluded	absent	137.74	0.15	excluded	absent	315.17	2.66	excluded	absent	116.8	-0.14	excluded	absent	132.66	0.08	excluded	absent	231.1	1.47	excluded	absent	240.46	1.6	excluded	absent	50.72	-1.07	included	present	158.89	0.45
230240_at	excluded	absent	81.41	0.15	included	present	141.02	1.09	excluded	absent	9.23	-1	included	present	139.48	1.07	included	present	182.33	1.75	excluded	absent	201.85	2.06	excluded	absent	91.06	0.3	included	present	137.62	1.04	excluded	absent	110.41	0.61	excluded	absent	79.76	0.12	excluded	absent	113.24	0.65	excluded	absent	68.69	-0.06	excluded	absent	132.5	0.96	included	present	163.4	1.45	excluded	absent	49.28	-0.36
230241_at	included	present	204.66	-0.39	included	present	355.55	0.69	included	present	243.55	-0.12	included	present	354.19	0.68	excluded	absent	604.92	2.48	included	present	366.67	0.77	included	present	571.29	2.23	included	present	475.74	1.55	included	present	352.02	0.66	included	present	432.15	1.24	included	present	272.21	0.09	included	present	353.79	0.68	included	absent	249.98	-0.07	included	present	486.41	1.63	included	present	291.74	0.23
230242_at	included	present	802.22	-0.1	included	present	459.45	-0.51	included	present	157.96	-0.87	included	present	326.17	-0.67	included	present	326.28	-0.67	included	present	281.43	-0.72	included	present	718.66	-0.2	included	present	181.98	-0.84	included	present	539.4	-0.41	included	present	179.38	-0.84	included	present	496.01	-0.46	included	present	154.63	-0.87	included	present	147.04	-0.88	included	absent	133.03	-0.9	included	present	527.74	-0.43
230243_at	included	present	79.21	-0.84	included	present	84.1	-0.76	included	absent	110.92	-0.32	included	present	147.92	0.3	included	absent	94.42	-0.59	included	present	89.45	-0.67	included	absent	56.94	-1.21	included	present	155.23	0.42	included	present	68.62	-1.02	included	present	89.53	-0.67	included	present	157.7	0.46	included	present	149.05	0.32	included	present	164.97	0.58	included	present	171.57	0.69	included	present	127.77	-0.04
230244_at	excluded	absent	25.19	0.12	excluded	absent	3.1	-0.74	excluded	absent	5.39	-0.65	excluded	absent	112.05	3.48	excluded	absent	119.13	3.75	excluded	absent	2.56	-0.76	excluded	absent	5.64	-0.64	excluded	absent	2.55	-0.76	excluded	absent	8.08	-0.54	excluded	absent	46.7	0.95	excluded	absent	3.5	-0.72	excluded	absent	32.76	0.41	excluded	absent	5.25	-0.65	excluded	absent	3.07	-0.74	excluded	absent	1.33	-0.8
230245_s_at	included	present	130.51	-0.12	included	present	118.98	-0.15	excluded	absent	23.68	-0.38	included	present	898.56	1.75	excluded	absent	20.74	-0.39	excluded	absent	108.75	-0.17	excluded	absent	37.92	-0.35	excluded	absent	6.46	-0.42	included	present	302.2	0.3	excluded	absent	68.5	-0.27	excluded	absent	133.27	-0.11	included	present	234.91	0.13	excluded	absent	96.93	-0.2	included	present	420.76	0.59	included	present	114.02	-0.16
230246_at	excluded	absent	100.81	0.75	excluded	absent	51.72	-0.08	excluded	absent	7.5	-0.84	excluded	absent	5.69	-0.87	excluded	absent	10.44	-0.79	included	present	138.63	1.4	excluded	absent	6.49	-0.86	excluded	absent	77.62	0.36	excluded	absent	58.42	0.03	excluded	absent	68.73	0.21	excluded	absent	10.13	-0.79	excluded	absent	30.57	-0.45	excluded	absent	53.35	-0.06	excluded	absent	61.46	0.08	excluded	absent	1.64	-0.94
230247_at	excluded	absent	46.31	-0.24	excluded	absent	40.17	-0.36	excluded	absent	7.15	-1.01	excluded	absent	5.56	-1.05	excluded	absent	82.5	0.49	excluded	absent	92.46	0.68	excluded	absent	91.64	0.67	excluded	absent	46.97	-0.22	excluded	absent	61.11	0.06	excluded	absent	18.82	-0.78	excluded	absent	56.47	-0.03	excluded	absent	83.5	0.5	excluded	absent	61.92	0.08	excluded	absent	145.6	1.74	excluded	absent	34.22	-0.48
230248_x_at	included	absent	80.32	-0.9	included	present	432.35	2.71	included	present	245.53	0.79	included	present	425.4	2.64	included	present	501.59	3.42	included	absent	45.96	-1.26	included	present	655.8	5	included	present	200.64	0.33	included	present	311.14	1.46	included	present	322.32	1.58	included	present	257.09	0.91	included	present	181.32	0.13	included	present	430.57	2.69	included	present	359.96	1.97	included	present	299.81	1.35
230249_at	included	present	564.95	0.32	included	present	150.07	-0.43	included	present	444.36	0.1	included	present	109.45	-0.5	included	present	397.25	0.02	included	absent	143.83	-0.44	included	present	277.26	-0.2	included	present	234.14	-0.27	included	present	215.83	-0.31	included	present	551.35	0.3	included	present	347.17	-0.07	included	present	389.86	0.01	included	present	441.8	0.1	included	present	555.24	0.3	included	present	790.46	0.73
230250_at	included	present	243.3	-0.82	included	present	163.94	-1.08	included	present	160.81	-1.09	included	absent	39.76	-1.5	included	present	384.44	-0.34	included	present	1305.08	2.73	included	present	150.65	-1.13	included	present	117.3	-1.24	included	present	909.44	1.41	included	present	511.53	0.08	included	present	363.28	-0.42	included	present	396.23	-0.31	included	present	606.71	0.4	included	present	682.93	0.65	included	present	114.48	-1.25
230251_at	excluded	absent	40.12	-0.38	excluded	absent	16.17	-0.43	excluded	absent	58.09	-0.34	included	present	174.67	-0.11	excluded	absent	3.12	-0.46	excluded	absent	183.38	-0.09	excluded	absent	2.04	-0.46	excluded	absent	29.14	-0.4	excluded	absent	3.99	-0.45	excluded	absent	1.87	-0.46	excluded	absent	4.46	-0.45	excluded	absent	9.19	-0.44	excluded	absent	2.71	-0.46	excluded	absent	2.85	-0.46	excluded	absent	5.14	-0.45
230252_at	included	present	374.62	0.35	included	absent	129.56	-0.65	included	absent	88.52	-0.81	included	absent	45.88	-0.99	included	absent	67.12	-0.9	included	present	189.86	-0.4	included	absent	132.07	-0.64	included	absent	56.33	-0.94	included	present	217.9	-0.29	included	absent	96.48	-0.78	included	absent	88.56	-0.81	included	present	162.97	-0.51	included	absent	63.17	-0.92	included	present	214.27	-0.3	included	absent	78.77	-0.85
230253_at	included	present	425.99	5.63	excluded	absent	100.61	-0.3	excluded	absent	130.41	0.24	included	present	153.38	0.66	excluded	absent	4.9	-2.05	included	present	234.91	2.15	included	present	92.49	-0.45	excluded	absent	66.22	-0.93	included	present	173.74	1.03	excluded	absent	11.47	-1.93	excluded	absent	179.13	1.13	included	present	157.51	0.73	excluded	absent	71.78	-0.83	excluded	absent	57.12	-1.1	excluded	absent	65.7	-0.94
230254_at	included	present	239.16	1.48	included	present	107.46	-0.05	excluded	absent	51.92	-0.69	excluded	absent	109.69	-0.02	included	present	133.17	0.25	included	present	356.09	2.83	included	present	123.74	0.14	excluded	absent	26.7	-0.98	included	present	224.59	1.31	excluded	absent	68.7	-0.5	included	present	141.13	0.34	excluded	absent	142.32	0.36	excluded	absent	79.1	-0.38	included	present	144.54	0.38	excluded	absent	20.88	-1.05
230255_at	excluded	absent	4.64	-0.74	excluded	absent	100.75	-0.55	excluded	absent	21.08	-0.71	excluded	absent	170.86	-0.42	excluded	absent	199.58	-0.36	excluded	absent	14.07	-0.72	excluded	absent	98.43	-0.56	excluded	absent	103.02	-0.55	excluded	absent	118.54	-0.52	included	present	445.54	0.12	excluded	absent	219.08	-0.32	excluded	absent	87.45	-0.58	excluded	absent	102.64	-0.55	excluded	absent	128.25	-0.5	excluded	absent	79.97	-0.59
230256_at	included	present	1071.39	1.36	included	present	507.4	-0.52	included	present	1536.82	2.91	included	present	1257.73	1.98	included	present	2114.13	4.84	included	present	885.86	0.74	included	present	837.84	0.58	included	present	185.77	-1.6	included	present	789.55	0.42	included	present	869.2	0.68	included	present	507.68	-0.52	included	present	260.18	-1.35	included	present	598.61	-0.22	included	present	313.29	-1.17	included	present	184.54	-1.6
230257_s_at	included	present	233.59	-0.55	included	present	205.67	-0.85	included	present	385	1.13	included	present	314.71	0.35	included	present	399	1.29	included	present	733.56	4.99	included	present	518.13	2.6	included	present	684.19	4.44	included	present	489.82	2.29	included	present	1040.53	8.39	included	present	294.31	0.13	included	present	422.43	1.54	included	present	271.72	-0.12	included	present	139.98	-1.58	included	present	578.2	3.27
230258_at	included	present	345.91	0.84	included	absent	73.93	-1.02	included	present	194.22	-0.2	included	absent	73.07	-1.03	included	absent	108.72	-0.79	included	present	339.23	0.79	included	absent	51.86	-1.18	included	absent	19.79	-1.4	included	present	216.51	-0.05	included	absent	80.63	-0.98	included	absent	71.36	-1.04	included	present	207.78	-0.11	included	present	272.18	0.33	included	present	184.41	-0.27	included	absent	38.33	-1.27
230259_at	excluded	absent	121.41	-0.07	excluded	absent	172.56	0.1	excluded	absent	20.85	-0.41	excluded	absent	9	-0.45	excluded	absent	33.24	-0.37	excluded	absent	7.03	-0.45	excluded	absent	22.81	-0.4	excluded	absent	271.29	0.43	excluded	absent	16.78	-0.42	excluded	absent	16.02	-0.42	excluded	absent	25.6	-0.39	excluded	absent	334.67	0.64	excluded	absent	8.97	-0.45	excluded	absent	13.72	-0.43	excluded	absent	157.15	0.05
230260_s_at	included	present	254.86	-0.37	included	present	245.77	-0.4	included	present	230.63	-0.45	included	present	191.33	-0.56	included	present	466.42	0.26	included	present	1897.59	4.57	included	present	1602.82	3.69	included	present	1242.46	2.6	included	present	2469.74	6.3	included	present	595.49	0.65	included	present	2059.66	5.06	included	present	241.71	-0.41	included	present	405.42	0.08	included	present	254.15	-0.38	included	present	869.01	1.48
230261_at	included	present	269.1	0.47	included	present	226.47	0.21	included	present	685.64	2.99	included	present	2883.16	16.33	included	absent	134.73	-0.35	included	present	896.46	4.27	included	present	702.89	3.1	included	present	129.54	-0.38	included	present	497.93	1.86	included	present	249.07	0.35	included	present	212.63	0.12	included	present	2073.24	11.41	included	present	631.16	2.66	included	present	631.1	2.66	included	present	892.84	4.25
230262_at	included	present	157.8	-0.56	included	present	1690.71	0.25	included	present	229.28	-0.52	included	present	1026.8	-0.1	included	present	538.49	-0.36	excluded	absent	12.65	-0.64	included	present	797.17	-0.22	included	present	245.55	-0.51	included	present	519.86	-0.37	included	present	1412.38	0.1	included	present	1670.64	0.24	included	present	989.96	-0.12	included	present	1025.6	-0.1	included	present	2065.3	0.44	included	present	1116.05	-0.06
230263_s_at	included	present	789.1	0.09	included	absent	72.95	-1.11	included	present	229.24	-0.85	included	present	242.19	-0.83	included	absent	143.94	-0.99	included	present	303.57	-0.73	included	present	123.45	-1.03	included	present	298.03	-0.73	included	present	216.6	-0.87	included	absent	61.37	-1.13	included	absent	58.93	-1.14	included	present	283.98	-0.76	included	present	182.06	-0.93	included	present	247.38	-0.82	included	present	85.95	-1.09
230264_s_at	included	present	8744.1	2.27	included	present	1964.01	-0.28	included	present	3612.66	0.34	included	present	821.37	-0.71	included	present	3487.63	0.29	included	present	1962.45	-0.28	included	present	1771.1	-0.35	included	present	1552.42	-0.44	included	present	2641.8	-0.03	included	present	2249.37	-0.17	included	present	1621.02	-0.41	included	present	3004.15	0.11	included	present	2967.25	0.1	included	present	2313.47	-0.15	included	present	2255.38	-0.17
230265_at	included	present	4599.42	0.26	included	present	1288.97	-1.95	included	present	3663.07	-0.36	included	present	5029.9	0.55	included	present	3833.05	-0.25	included	present	3009.18	-0.8	included	present	2004.52	-1.47	included	present	1871.54	-1.56	included	present	2300.49	-1.27	included	present	2000.29	-1.47	included	present	2017.66	-1.46	included	present	1095.1	-2.08	included	present	2246.5	-1.31	included	present	1519.43	-1.79	included	present	1391.73	-1.88
230266_at	excluded	absent	140.38	0.42	excluded	absent	27.15	-0.88	excluded	absent	49.89	-0.62	excluded	absent	36.76	-0.77	excluded	absent	179.26	0.87	included	present	311.1	2.38	excluded	absent	83.85	-0.23	excluded	absent	98.92	-0.06	included	present	136.85	0.38	excluded	absent	100.46	-0.04	included	present	102.26	-0.02	included	present	178.32	0.86	excluded	absent	98	-0.07	excluded	absent	153.48	0.57	included	present	58.09	-0.53
230267_at	excluded	absent	115.05	-0.25	excluded	absent	123.13	-0.15	excluded	absent	50.73	-1.07	excluded	absent	190.73	0.71	excluded	absent	329.89	2.49	excluded	absent	160.87	0.33	excluded	absent	138.49	0.05	excluded	absent	116.58	-0.23	included	present	185.04	0.64	excluded	absent	116.28	-0.23	excluded	absent	199.69	0.83	included	present	519.59	4.9	excluded	absent	318.61	2.34	excluded	absent	105.75	-0.37	included	present	109.91	-0.31
230268_at	excluded	absent	64.21	-0.17	excluded	absent	2.51	-1.46	excluded	absent	36.01	-0.76	excluded	absent	1.46	-1.48	excluded	absent	70.68	-0.04	excluded	absent	128.81	1.18	excluded	absent	80.37	0.17	included	present	56.05	-0.34	included	present	65.15	-0.15	included	present	80.45	0.17	excluded	absent	29.79	-0.89	excluded	absent	21.68	-1.06	excluded	absent	109.07	0.77	excluded	absent	20.5	-1.09	excluded	absent	14.61	-1.21

230594_at	excluded	absent	44.2	-0.18	excluded	absent	42.7	-0.22	included	present	83.82	[illegible]
230595_at	included	present	329.07	-0.38	excluded	absent	35.97	-0.44	excluded	absent	76.75	[illegible]
230596_at	included	present	127.05	-0.49	included	present	569.88	1.62	included	absent	39.49	[illegible]
230597_at	excluded	absent	47.73	-0.31	included	present	582.66	3.03	excluded	absent	26.62	[illegible]
230598_at	included	present	85.3	-0.88	included	present	179.7	0.26	included	present	242.72	[illegible]
230599_at	included	present	242.43	-0.48	included	absent	82.7	-1.44	included	present	701.32	[illegible]
230600_at	excluded	absent	1.92	-0.43	excluded	absent	71.15	0.18	excluded	absent	39.17	[illegible]
230601_s_at	excluded	absent	70.64	-0.03	excluded	absent	25.78	-0.3	excluded	absent	33.83	[illegible]
230602_at	excluded	absent	37.79	-0.21	excluded	absent	2.18	-0.25	excluded	absent	39.61	[illegible]
230603_at	excluded	absent	36.95	-0.22	excluded	absent	13.87	-0.47	excluded	absent	82.46	[illegible]
230604_at	included	absent	75.96	-1.06	included	present	194.61	0.59	included	absent	43.8	[illegible]
230605_at	included	present	122.73	-1.13	included	present	293.36	0.65	included	absent	54.32	[illegible]
230606_at	excluded	absent	187.68	1.5	included	present	319.74	3.35	excluded	absent	52.66	[illegible]
230607_at	included	present	150.37	-0.91	included	absent	80.81	-1.37	included	present	180.99	[illegible]
230608_at	excluded	absent	6.82	-0.23	included	present	212.17	-0.18	excluded	absent	87.95	[illegible]
230609_at	included	absent	12.11	-1.29	included	absent	47.75	-0.94	included	absent	68.96	[illegible]
230610_at	excluded	absent	123	-0.14	excluded	absent	116.39	-0.22	included	present	144.87	[illegible]
230611_at	excluded	absent	125.98	-0.08	excluded	absent	5.36	-0.39	excluded	absent	52.41	[illegible]
230612_at	included	present	315.02	1.62	included	present	199.54	0.37	included	present	118.07	[illegible]
230613_at	excluded	absent	92.49	-0.57	excluded	absent	166.02	0.48	excluded	absent	103.2	[illegible]
230614_at	excluded	absent	48.51	0.16	included	present	115.77	1.19	excluded	absent	1.62	[illegible]
230615_at	excluded	absent	7.09	-0.93	excluded	absent	18.76	-0.72	excluded	absent	50.27	[illegible]
230616_at	excluded	absent	36.95	-0.75	excluded	absent	92.61	0.42	excluded	absent	73.16	[illegible]
230617_at	excluded	absent	1.87	-0.78	excluded	absent	44.24	0.85	excluded	absent	27.44	[illegible]
230618_s_at	included	present	5792.75	0.13	included	present	5904.5	0.21	included	present	8944.43	[illegible]
230619_at	included	present	862.66	-0.14	included	present	294.9	-1.22	included	present	915.22	[illegible]
230620_at	included	present	629.01	-0.34	included	present	837.73	-0.03	included	present	373.25	[illegible]
230621_at	included	present	3708.96	3.5	included	present	4491.5	4.97	included	present	4728.59	[illegible]
230622_at	included	present	205.72	0.64	included	present	176.44	0.27	excluded	absent	160.52	[illegible]
230623_x_at	included	present	940.81	-0.44	included	present	723.71	-0.86	included	present	874.29	[illegible]
230624_at	included	absent	89.59	-1.36	included	absent	142.52	-1.14	included	present	133.09	[illegible]
230625_s_at	excluded	absent	4.24	-0.59	excluded	absent	69.16	-0.45	excluded	absent	10.51	[illegible]
230626_at	excluded	absent	72.98	-0.32	excluded	absent	52.76	-0.34	excluded	absent	65.32	[illegible]
230627_at	excluded	absent	131.92	0.4	excluded	absent	177.79	1.02	excluded	absent	28	[illegible]
[illegible]	[illegible]	[illegible]	[illegible]	[illegible]	[illegible]	[illegible]	[illegible]	[illegible]	[illegible]	[illegible]	[illegible]	[illegible]

230953_at	excluded	absent	2.9	-0.31	included	present	410.88	0.37	excluded	absent	8.05	-0.3	excluded	absent	2.66	-0.31	excluded	absent	3.24
230954_at	excluded	absent	130.46	-0.17	included	present	468.57	1.12	included	present	220.28	0.17	included	present	295.91	0.46	excluded	absent	439.34
230955_s_at	excluded	absent	245.44	0.16	excluded	absent	333.21	0.7	excluded	absent	126.76	-0.58	excluded	absent	95.75	-0.77	included	present	323.81
230956_at	excluded	absent	131.81	0.06	excluded	absent	4.38	-0.51	excluded	absent	3.03	-0.51	excluded	absent	2.75	-0.52	excluded	absent	29.18
230957_at	included	present	115.47	-0.25	included	absent	103.61	-0.25	included	present	231.86	-0.2	included	present	276.34	-0.18	included	absent	165.96
230958_s_at	included	present	4346.29	-0.71	included	present	282.81	-2.67	included	present	572.95	-2.53	included	present	2999.39	-1.36	included	present	1023.47
230959_at	included	present	3183.14	-0.36	included	present	11466.32	3.6	included	present	8217.63	2.05	included	present	2119.86	-0.86	included	present	17335.28
230960_at	excluded	absent	1.94	-0.9	included	present	245.36	2.96	excluded	absent	53.21	-0.08	excluded	absent	3.19	-0.88	excluded	absent	130.72
230961_at	included	present	275.53	1.22	included	present	173.72	0.01	included	present	270.29	1.16	included	present	193.91	0.25	included	present	300.97
230962_at	included	present	337.23	-0.06	included	present	639.4	0.59	included	present	253.95	-0.24	included	absent	76.41	-0.62	included	present	304.16
230963_at	excluded	absent	10.21	-0.89	excluded	absent	5.02	-0.9	excluded	absent	27.44	-0.85	excluded	absent	51.55	-0.8	excluded	absent	10.46
230964_at	included	present	598.78	3.42	excluded	absent	49.26	-0.28	excluded	absent	44.87	-0.31	excluded	absent	8.81	-0.56	excluded	absent	43.35
230965_at	excluded	absent	10.62	-0.57	excluded	absent	36.68	-0.29	excluded	absent	63.29	0	excluded	absent	6.57	-0.61	excluded	absent	8.39
230966_at	excluded	absent	9.34	-0.78	excluded	absent	96.27	0.3	excluded	absent	8.68	-0.79	excluded	absent	13.28	-0.73	excluded	absent	10.92
230967_s_at	included	present	510.27	-1.07	included	present	579.21	-0.83	included	present	582.61	-0.82	included	present	340.58	-1.67	included	present	265.98
230968_at	included	present	359.97	-0.21	included	present	1434.93	3.1	included	present	6179.43	17.69	included	present	1122.85	2.14	included	present	1700.04
230969_at	excluded	absent	42.4	-0.22	excluded	absent	57.31	-0.21	excluded	absent	10.08	-0.25	excluded	absent	57	-0.21	excluded	absent	69.9
230970_at	excluded	absent	70.56	-0.26	excluded	absent	8.68	-0.83	included	present	172.46	0.67	included	present	95.99	-0.03	excluded	absent	213.76
230971_x_at	excluded	absent	36.84	0.13	excluded	absent	17.07	-0.42	excluded	absent	4.32	-0.78	excluded	absent	24.24	-0.22	excluded	absent	58.04
230972_at	excluded	absent	173.65	-0.32	excluded	absent	120.56	-0.42	excluded	absent	69.26	-0.51	excluded	absent	146.68	-0.37	excluded	absent	109.97
230973_at	excluded	absent	100.46	-0.54	excluded	absent	194.27	-0.47	excluded	absent	3.73	-0.62	excluded	absent	7.89	-0.61	excluded	absent	87.15
230974_at	included	present	201.06	-0.45	included	present	660.08	3.72	included	present	193.67	-0.51	included	present	345.4	0.86	included	present	435.52
230975_at	excluded	absent	26.9	-0.2	excluded	absent	41.16	-0.17	excluded	absent	74.08	-0.1	excluded	absent	2.9	-0.25	excluded	absent	2.5
230976_at	excluded	absent	2.67	-0.69	excluded	absent	27.77	-0.43	excluded	absent	78.84	0.11	excluded	absent	34.72	-0.36	excluded	absent	14.59
230977_at	excluded	absent	256	0	included	present	473.53	0.77	excluded	absent	45.64	-0.74	excluded	absent	124.23	-0.47	excluded	absent	112.31
230978_at	excluded	absent	79.45	0	excluded	absent	68.76	-0.21	excluded	absent	60	-0.37	excluded	absent	35.32	-0.84	excluded	absent	19.21
230979_at	excluded	absent	58.62	-0.25	excluded	absent	58.4	-0.25	excluded	absent	57	-0.27	excluded	absent	79.36	0.11	excluded	absent	13.49
230980_x_at	included	absent	212.73	-0.38	included	present	246.43	-0.31	included	absent	156.76	-0.51	included	absent	4.36	-0.84	included	absent	130.43
230981_at	excluded	absent	96.9	-0.25	excluded	absent	13.63	-0.38	excluded	absent	72.88	-0.29	included	present	205.77	-0.08	excluded	absent	148.39
230982_at	excluded	absent	5.96	-0.67	excluded	absent	14.52	-0.45	excluded	absent	2.67	-0.75	excluded	absent	120.72	2.24	excluded	absent	5.85
230983_at	excluded	absent	66.47	-0.24	excluded	absent	6.25	-0.29	excluded	absent	54.21	-0.25	included	present	437.92	0.03	included	present	331.98
230984_s_at	excluded	absent	11.06	-0.84	excluded	absent	47.82	-0.35	excluded	absent	54.27	-0.27	included	present	194.71	1.59	excluded	absent	108.79
230985_at	excluded	absent	2.73	-0.34	excluded	absent	3.32	-0.34	excluded	absent	2.97	-0.34	excluded	absent	2.49	-0.34	excluded	absent	16.02
230986_at	included	present	255.04	-0.33	included	absent	83.77	-0.77	included	absent	50.53	-0.86	included	absent	39.61	-0.89	included	absent	3.21

231312_at	included	present	88.74	-0.44	included	absent	93.09	-0.43	included	absent	101.12	-0.41	included	present
231313_at	excluded	absent	110.37	0.76	excluded	absent	17.14	-0.65	excluded	absent	36.75	-0.35	excluded	absent
231314_at	excluded	absent	22.38	-0.66	included	present	634.37	4.49	excluded	absent	19.48	-0.69	excluded	absent
231315_at	excluded	absent	5.04	-0.38	included	present	85.57	0.05	excluded	absent	9.45	-0.36	included	present
231316_at	included	present	262.95	-1.13	included	present	194.81	-1.47	included	present	348.95	-0.71	included	present
231317_at	excluded	absent	18.4	-1.45	excluded	absent	122.63	0.44	excluded	absent	131.19	0.6	excluded	absent
231318_at	excluded	absent	58.45	-0.51	excluded	absent	105.3	-0.08	excluded	absent	46.78	-0.62	excluded	absent
231319_x_at	included	present	512.83	-0.58	included	present	6988.6	1.72	included	present	1021.12	-0.4	included	present
231320_at	excluded	absent	37.58	-0.3	excluded	absent	13.01	-0.92	excluded	absent	16.35	-0.84	excluded	absent
231321_s_at	included	present	230.22	0.34	included	absent	109.71	-0.92	included	present	79.39	-1.24	included	absent
231322_at	excluded	absent	49.72	-0.67	excluded	absent	106.89	0.44	excluded	absent	48.72	-0.69	excluded	absent
231323_at	excluded	absent	34.12	-0.77	excluded	absent	6.56	-1.19	excluded	absent	36.31	-0.73	excluded	absent
231324_at	excluded	absent	82.6	-0.33	included	present	112.18	0.04	excluded	absent	94.94	-0.18	excluded	absent
231325_at	included	absent	64.18	-0.7	included	present	212.87	0.1	included	absent	73.41	-0.65	included	present
231326_s_at	excluded	absent	130.6	0.09	excluded	absent	118.84	-0.14	excluded	absent	73.02	-1.02	excluded	absent
231327_at	excluded	absent	7.98	-0.86	excluded	absent	148.19	1.02	excluded	absent	109.2	0.5	excluded	absent
231328_s_at	included	present	187.37	-0.28	included	present	200.77	-0.13	included	present	143.5	-0.78	included	present
231329_at	included	present	272.61	-0.84	included	absent	123.98	-1.25	included	present	198.11	-1.04	included	absent
231330_at	excluded	absent	39.24	-0.21	excluded	absent	11.78	-0.36	excluded	absent	67.36	-0.06	excluded	absent
231331_at	excluded	absent	88.14	0.14	excluded	absent	46.42	-0.39	excluded	absent	9.59	-0.85	included	present
231332_at	included	present	98.08	-0.22	excluded	absent	35.05	-1.22	included	present	136.24	0.38	included	present
231333_at	excluded	absent	27.28	-0.26	excluded	absent	9.08	-0.4	excluded	absent	10.1	-0.39	excluded	absent
231334_at	excluded	absent	5.3	-1.02	excluded	absent	79.78	-0.12	excluded	absent	55.76	-0.41	excluded	absent
231335_at	excluded	absent	4.84	-0.24	excluded	absent	4.64	-0.24	excluded	absent	13.71	-0.23	excluded	absent
231336_at	excluded	absent	81.99	-0.43	excluded	absent	109.79	-0.34	excluded	absent	3.84	-0.7	excluded	absent
231337_at	included	present	127.5	0.94	included	present	108	0.63	excluded	absent	76.08	0.14	excluded	absent
231338_at	excluded	absent	2.85	-0.66	excluded	absent	64.86	0.52	excluded	absent	41.86	0.08	excluded	absent
231339_at	included	present	106.04	-0.17	excluded	absent	58.45	-0.23	excluded	absent	2.72	-0.3	excluded	absent
231340_at	excluded	absent	77.11	0.15	excluded	absent	5.34	-0.95	excluded	absent	3.46	-0.98	excluded	absent
231341_at	excluded	absent	24.24	-0.5	excluded	absent	2.75	-0.67	included	present	273.32	1.49	included	present
231342_at	excluded	absent	17.03	-0.48	excluded	absent	45.52	0.34	excluded	absent	85.03	1.48	excluded	absent
231343_at	included	present	316.41	-0.34	included	present	1446.43	3.2	included	present	92.45	-1.04	included	present
231344_at	excluded	absent	2.67	-0.57	excluded	absent	3.26	-0.56	excluded	absent	1.86	-0.57	excluded	absent
231345_s_at	excluded	absent	19.39	-1.59	included	present	266.32	0.44	excluded	absent	66.56	-1.2	excluded	absent
231346_s_at	excluded	absent	5.25	-0.25	excluded	absent	8.82	-0.24	excluded	absent	6.33	-0.24	excluded	absent
231347_at	excluded	absent	47.34	0.06	excluded	absent	8.6	-0.4	excluded	absent	50.04	0.09	excluded	absent
231348_s_at	included	absent	13.54	-0.84	included	present	244.31	-0.74	included	present	132.03	-0.79	included	present
231349_at	excluded	absent	48.38	0.25	excluded	absent	4.09	-0.7	excluded	absent	78.3	0.89	excluded	absent
231350_at	excluded	absent	6.62	-0.25	excluded	absent	8.95	-0.25	excluded	absent	7.36	-0.25	excluded	absent
231351_at	excluded	absent	117.54	-0.26	included	present	159.55	0.1	excluded	absent	33.21	-1	excluded	absent
231352_at	excluded	absent	2.27	-0.16	excluded	absent	2.59	-0.16	excluded	absent	9.11	-0.15	excluded	absent
231353_at	excluded	absent	76.08	0.77	excluded	absent	74.44	0.72	excluded	absent	62.56	0.41	excluded	absent
[illegible]	[illegible]	[illegible]	[illegible]	[illegible]	[illegible]	[illegible]	[illegible]	[illegible]	[illegible]	[illegible]	[illegible]	[illegible]	[illegible]	[illegible]

231671_at	excluded	absent	38.66	-0.15	excluded	absent	2.39	-0.15	included	present	249.15	[illegible]
231672_at	included	absent	63.11	-0.25	included	absent	62.73	-0.25	included	absent	93.08	[illegible]
231673_at	excluded	absent	17.87	-0.4	excluded	absent	9.99	-0.55	excluded	absent	2.62	[illegible]
231674_at	excluded	absent	2.79	-0.61	excluded	absent	3	-0.61	included	present	47.49	[illegible]
231675_s_at	excluded	absent	6.55	-0.2	excluded	absent	11.23	-0.2	included	present	285.54	[illegible]
231676_s_at	included	present	174.84	-0.1	excluded	absent	12.29	-0.56	excluded	absent	74.27	[illegible]
231677_at	excluded	absent	47.44	-0.03	excluded	absent	36.65	-0.09	excluded	absent	5.51	[illegible]
231678_s_at	excluded	absent	48.26	-0.18	excluded	absent	2.62	-0.2	included	present	275.71	[illegible]
231679_at	excluded	absent	3.96	-0.78	excluded	absent	4.8	-0.75	excluded	absent	2.04	[illegible]
231680_at	included	present	289.9	0.82	excluded	absent	51.37	-0.58	excluded	absent	82.63	[illegible]
231681_x_at	included	present	573.91	1.35	included	present	680.3	1.9	included	present	1339.54	[illegible]
231682_at	excluded	absent	14.85	-0.45	excluded	absent	13.88	-0.47	excluded	absent	84.56	[illegible]
231683_at	excluded	absent	12.05	-0.24	excluded	absent	79.33	-0.19	included	present	79.45	[illegible]
231684_at	excluded	absent	27.88	-0.18	excluded	absent	2.09	-0.2	included	present	196.98	[illegible]
231685_at	excluded	absent	10.37	-0.9	excluded	absent	141.13	0.56	excluded	absent	8.46	[illegible]
231686_at	included	present	8104.21	0.79	included	present	141.71	-0.5	included	present	1747.03	[illegible]
231687_at	excluded	absent	52.33	-0.4	excluded	absent	14.88	-0.56	excluded	absent	3.18	[illegible]
231688_at	excluded	absent	38.08	-0.01	excluded	absent	0.91	-0.58	excluded	absent	55.21	[illegible]
231689_at	included	present	875.45	1.2	included	present	330.74	-1.75	included	present	823.76	[illegible]
231690_at	excluded	absent	33.57	0.06	excluded	absent	3.16	-0.65	excluded	absent	89.81	[illegible]
231691_at	excluded	absent	25.68	-0.15	excluded	absent	5.02	-0.16	excluded	absent	78.86	[illegible]
231692_at	included	present	166.51	0.16	included	present	256.06	1.75	excluded	absent	23.27	[illegible]
231693_at	excluded	absent	41.08	-0.29	excluded	absent	59.95	-0.29	included	present	349.99	[illegible]
231694_at	excluded	absent	21.49	-0.21	excluded	absent	45.31	-0.2	excluded	absent	170.15	[illegible]
231695_at	included	absent	122.68	-0.59	included	absent	125.18	-0.58	included	absent	66.94	[illegible]
231696_x_at	included	present	1531.99	0.77	included	present	911.36	-0.49	included	present	786.38	[illegible]
231697_s_at	included	present	858.09	-0.54	included	present	620.71	-0.62	included	present	753.97	[illegible]
231698_at	included	present	508.26	-0.33	included	present	368.4	-0.41	included	present	360.04	[illegible]
231699_at	included	present	164.77	-0.91	included	absent	79.58	-1.3	included	absent	91	[illegible]
231700_at	excluded	absent	74.9	-0.16	excluded	absent	104.65	0.16	excluded	absent	59.74	[illegible]
231701_s_at	included	present	359.41	-0.19	included	present	136.86	-0.33	included	present	296.36	[illegible]
231702_at	excluded	absent	49.54	-0.11	excluded	absent	2.41	-0.18	excluded	absent	55.32	[illegible]
231703_s_at	excluded	absent	39.2	-0.18	excluded	absent	4.35	-0.2	included	present	211.48	[illegible]
231704_at	included	present	174.59	-0.22	included	absent	84.49	-0.24	included	present	2071.87	[illegible]
[illegible]	[illegible]	[illegible]	[illegible]	[illegible]	[illegible]	[illegible]	[illegible]	[illegible]	[illegible]	[illegible]	[illegible]	[illegible]

232030_at	excluded	absent	54.09	-0.99	excluded	absent	40.59	-1.27	excluded	absent	108.98	0.14	included	present	128.59	0.54	excluded	absent	70.78	-0.65	excluded	absent	83.67	-0.38	included	present	117.22	0.31	included	absent	50.17	-1.07	included	present	71.58	-0.63	included	present	67.74	-0.71	included	present	132.52	0.62	included	present	188.47	1.77	included	present	112.65	0.21	included	present	62.39	-0.82	included	present	61.27	-0.84
232031_s_at	excluded	absent	82.16	-0.38	excluded	absent	47.16	-1.17	included	present	90.48	-0.2	excluded	absent	118.11	0.42	excluded	absent	110	0.24	excluded	absent	60.13	-0.88	included	present	157.3	1.3	included	absent	76.94	-0.5	included	present	92.84	-0.14	included	present	222.88	2.77	included	present	240.72	3.17	excluded	absent	161.92	1.4	included	present	112.73	0.3	included	present	82.4	-0.38	included	present	173.78	1.67
232032_x_at	excluded	absent	266.89	1.83	excluded	absent	105.7	-0.4	excluded	absent	44.94	-1.23	excluded	absent	64.35	-0.97	included	present	235.04	1.39	included	present	679.44	7.52	included	present	344.34	2.9	included	present	395.98	3.61	included	present	382.79	3.43	included	present	534.52	5.52	excluded	absent	157.94	0.33	included	present	191.4	0.79	excluded	absent	110.11	-0.33	included	present	139.36	0.07	included	present	437.87	4.19
232033_at	included	present	806.23	0.47	included	present	1212.05	1.57	included	present	1567.81	2.55	included	present	3841.35	8.75	included	present	1157.75	1.43	included	present	264.59	-1.01	included	present	510.27	-0.34	included	present	330.59	-0.83	included	present	388.84	-0.67	included	present	385.43	-0.68	included	present	485.66	-0.41	included	present	468.8	-0.45	included	present	1348.76	1.95	included	present	1498.06	2.36	included	present	379.15	-0.7
232034_at	included	present	1247.68	0.05	included	present	7181.18	5.64	included	present	1254.15	0.05	included	present	1044.46	-0.15	included	present	1507.46	0.29	included	present	309.49	-0.84	included	present	1344.66	0.14	included	present	1164.92	-0.03	included	present	845.28	-0.33	included	present	1398.62	0.19	included	present	593.63	-0.57	included	present	214.15	-0.93	included	present	1240.43	0.04	included	present	2505.72	1.23	included	present	787.2	-0.39
232035_at	included	present	332.39	0.24	included	present	357.3	0.32	included	absent	50.37	-0.68	included	present	310.71	0.17	included	present	247.69	-0.04	included	present	185.77	-0.24	included	present	239.81	-0.06	included	present	434.1	0.56	included	present	125.55	-0.43	included	present	95.35	-0.53	included	present	95.4	-0.53	included	absent	76.22	-0.59	included	absent	53.18	-0.67	included	present	154.99	-0.34	included	present	332.93	0.24
232036_at	excluded	absent	184.05	0.56	excluded	absent	8.94	-1.55	included	present	142.04	0.05	included	present	162.64	0.3	excluded	absent	14.09	-1.49	included	present	403.54	3.19	excluded	absent	50.51	-1.05	included	present	90.74	-0.56	included	present	293.1	1.87	included	present	154.71	0.2	included	present	283.87	1.76	excluded	absent	116.96	-0.25	excluded	absent	9.01	-1.55	excluded	absent	1.84	-1.63	excluded	absent	60.04	-0.93
232037_at	excluded	absent	111.23	1.2	included	present	126.37	1.58	excluded	absent	71.43	0.2	excluded	absent	5.96	-1.45	included	present	150.48	2.18	excluded	absent	107.17	1.09	excluded	absent	103.08	0.99	excluded	absent	75.51	0.3	excluded	absent	62.24	-0.04	excluded	absent	54.38	-0.23	excluded	absent	73.81	0.26	excluded	absent	81.25	0.44	excluded	absent	75.82	0.31	excluded	absent	25.42	-0.96	excluded	absent	100.48	0.93
232038_at	included	present	111.42	0.3	excluded	absent	74.4	-0.3	excluded	absent	2.68	-1.46	included	present	77.44	-0.25	excluded	absent	41.03	-0.84	included	present	87.85	-0.09	included	present	151.68	0.95	included	present	89.94	-0.05	included	present	101.23	0.13	included	present	102.28	0.15	included	present	86.85	-0.1	included	present	110.78	0.29	included	present	270.96	2.88	included	present	152.91	0.97	included	present	104.98	0.19
232039_at	excluded	absent	21.35	-0.58	excluded	absent	2.77	-1.06	excluded	absent	4.18	-1.03	included	present	86.04	1.11	excluded	absent	10.8	-0.85	excluded	absent	55.55	0.31	excluded	absent	27.47	-0.42	excluded	absent	30.33	-0.34	excluded	absent	12.99	-0.8	excluded	absent	17.79	-0.67	included	present	54.92	0.3	excluded	absent	48.2	0.12	excluded	absent	47.38	0.1	excluded	absent	42.03	-0.04	excluded	absent	18.76	-0.64
232040_at	excluded	absent	79.81	0.08	excluded	absent	21.98	-0.71	excluded	absent	11.14	-0.86	excluded	absent	14.23	-0.81	excluded	absent	66.4	-0.1	excluded	absent	76.26	0.03	excluded	absent	60.87	-0.18	excluded	absent	15.58	-0.8	excluded	absent	33.21	-0.56	excluded	absent	5.05	-0.94	excluded	absent	10.98	-0.86	excluded	absent	5.03	-0.94	included	present	139.69	0.9	excluded	absent	43.33	-0.42	excluded	absent	24.5	-0.67
232041_at	excluded	absent	41.49	0.47	excluded	absent	2.87	-0.93	excluded	absent	17.59	-0.39	excluded	absent	8.62	-0.72	excluded	absent	3.52	-0.9	excluded	absent	72.03	1.58	excluded	absent	10.06	-0.67	excluded	absent	3.01	-0.92	excluded	absent	7.13	-0.77	excluded	absent	45.74	0.63	excluded	absent	38.52	0.37	excluded	absent	35.69	0.26	excluded	absent	4.62	-0.86	excluded	absent	21.19	-0.26	excluded	absent	3.61	-0.9
232042_at	included	present	114.91	-0.18	excluded	absent	26.66	-0.76	excluded	absent	24.34	-0.78	included	present	140.49	-0.01	excluded	absent	121.64	-0.13	included	present	133.42	-0.05	excluded	absent	44.65	-0.64	included	present	318.26	1.17	included	present	159.91	0.12	included	absent	78.34	-0.42	included	present	250.17	0.72	included	absent	124.89	-0.11	included	absent	29.6	-0.74	included	absent	82.97	-0.39	included	present	94.7	-0.31
232043_at	excluded	absent	7.94	-0.64	excluded	absent	37.56	0.56	excluded	absent	29.08	0.21	excluded	absent	67.42	1.76	excluded	absent	10.03	-0.56	excluded	absent	3.86	-0.81	excluded	absent	5.7	-0.73	excluded	absent	4.23	-0.79	excluded	absent	6.21	-0.71	excluded	absent	12.65	-0.45	excluded	absent	6.04	-0.72	excluded	absent	2.97	-0.84	excluded	absent	3.74	-0.81	excluded	absent	27.9	0.16	excluded	absent	3.08	-0.84
232044_at	included	present	866.36	-0.75	included	present	1257.32	0.14	included	present	1204.86	0.02	included	present	1354.2	0.36	included	present	865.52	-0.75	included	present	495.73	-1.59	included	present	781.26	-0.94	included	present	317.76	-1.99	included	present	880.86	-0.72	included	present	718.19	-1.08	included	present	1296.1	0.22	included	present	544.75	-1.48	included	present	335.78	-1.95	included	present	476.98	-1.63	included	present	509.16	-1.56
232045_at	excluded	absent	9.34	-0.72	excluded	absent	15.65	-0.65	excluded	absent	56.89	-0.15	excluded	absent	19.29	-0.6	excluded	absent	21.99	-0.57	excluded	absent	18.41	-0.61	excluded	absent	80.49	0.14	excluded	absent	14.58	-0.66	excluded	absent	9.87	-0.72	excluded	absent	34.56	-0.42	excluded	absent	76.68	0.09	excluded	absent	11.79	-0.69	excluded	absent	10.19	-0.71	excluded	absent	2.3	-0.81	excluded	absent	35.99	-0.4
232046_at	excluded	absent	89.24	-0.49	excluded	absent	30.92	-0.98	excluded	absent	57.93	-0.75	excluded	absent	31.73	-0.97	excluded	absent	210.93	0.53	excluded	absent	176.81	0.24	excluded	absent	6.25	-1.19	excluded	absent	34.95	-0.95	excluded	absent	96.68	-0.43	excluded	absent	109.83	-0.32	excluded	absent	16.04	-1.11	excluded	absent	69.92	-0.65	excluded	absent	109.36	-0.32	excluded	absent	64.59	-0.7	excluded	absent	24.52	-1.03
232047_at	included	present	462.06	1.77	included	absent	51.21	-1.69	included	absent	83.07	-1.42	included	present	136.84	-0.97	included	absent	186.78	-0.55	included	absent	103.34	-1.25	included	present	195.13	-0.48	included	absent	9.91	-2.04	included	present	169.83	-0.69	included	present	144.78	-0.9	included	present	559.01	2.58	included	absent	119.35	-1.12	included	present	141.85	-0.93	included	present	136.58	-0.97	included	present	71.68	-1.52
232048_at	included	present	144.44	-0.12	excluded	absent	55.19	-0.7	included	present	170.35	0.05	included	present	108.6	-0.35	included	present	175.11	0.08	included	present	169.58	0.05	excluded	absent	77.35	-0.56	included	present	155.1	-0.05	excluded	absent	173.46	0.07	included	present	76.61	-0.56	excluded	absent	86.57	-0.5	included	present	78.48	-0.55	excluded	absent	28.39	-0.88	included	present	31.73	-0.85	included	present	169.86	0.05
232049_at	excluded	absent	82.05	-0.5	included	present	88.48	-0.39	included	present	142.8	0.57	included	present	169.29	1.04	included	present	166.68	0.99	excluded	absent	43.26	-1.19	included	present	135.3	0.44	included	present	116.09	0.1	included	present	140.28	0.53	included	present	80.72	-0.53	included	present	172.33	1.09	included	present	231.83	2.14	included	present	229.7	2.11	included	present	87.74	-0.4	included	present	173.07	1.11
232050_at	included	present	702.41	0.12	included	present	1639.07	3.23	included	present	877.97	0.7	included	present	496.7	-0.57	included	present	522.85	-0.48	included	present	301.18	-1.22	included	present	488.35	-0.59	included	present	777.36	0.37	included	present	687.79	0.07	included	present	1019.83	1.17	included	present	776	0.36	included	present	1332.71	2.21	included	present	737.08	0.23	included	present	3316.24	8.8	included	present	447.35	-0.73
232051_at	excluded	absent	5.15	-0.72	excluded	absent	5.18	-0.72	excluded	absent	3.01	-0.78	excluded	absent	8.91	-0.62	excluded	absent	189.84	4.37	excluded	absent	128.18	2.67	excluded	absent	32.7	0.04	excluded	absent	44.55	0.36	excluded	absent	14.84	-0.45	excluded	absent	9.7	-0.6	excluded	absent	10.28	-0.58	excluded	absent	15.67	-0.43	excluded	absent	7.84	-0.65	excluded	absent	4.62	-0.74	excluded	absent	8.43	-0.63
232052_at	included	present	497.14	-0.03	included	present	487.01	-0.07	included	present	788.48	1.19	included	present	1131.29	2.62	included	present	617.75	0.48	included	present	344.36	-0.66	included	present	614.2	0.46	included	present	310.88	-0.8	included	present	614.69	0.47	included	present	438.91	-0.27	included	present	780.16	1.16	included	present	406.98	-0.4	included	present	446.17	-0.24	included	present	360.64	-0.6	included	present	549.89	0.19
232053_x_at	included	present	709.65	1.91	included	present	168.36	-0.53	included	absent	45.44	-1.09	included	present	167.91	-0.53	included	absent	194.47	-0.41	included	present	387.24	0.45	included	present	1375.72	4.91	included	present	1277.01	4.47	included	present	1301.42	4.58	included	present	734.98	2.02	included	present	1510.67	5.52	included	present	299.53	0.06	included	present	104.64	-0.82	included	present	96.68	-0.86	included	present	2172.13	8.5
232054_at	included	absent	89.41	-0.47	included	absent	92.18	-0.47	included	present	135.47	-0.37	included	absent	5.04	-0.66	included	present	508.67	0.46	included	absent	52.77	-0.56	included	present	185.23	-0.26	included	absent	50.88	-0.56	included	present	909.51	1.36	included	present	185.94	-0.26	included	absent	5.27	-0.66	included	present	1138.45	1.87	included	present	688.61	0.87	included	present	129.91	-0.38	included	present	234.76	-0.15
232055_at	included	present	101.06	0.83	excluded	absent	80.51	0.39	included	present	154.91	1.98	included	present	187.97	2.69	excluded	absent	6.32	-1.2	excluded	absent	84.07	0.46	included	present	97.27	0.74	included	present	41.7	-0.45	included	present	118.73	1.2	excluded	absent	26.12	-0.78	excluded	absent	66.01	0.07	excluded	absent	64.35	0.04	included	present	106.39	0.94	included	present	84.68	0.47	excluded	absent	42.33	-0.43
232056_at	excluded	absent	3.24	-0.28	excluded	absent	54.98	-0.2	excluded	absent	15.14	-0.26	excluded	absent	46.26	-0.22	excluded	absent	1.62	-0.28	excluded	absent	3.85	-0.28	excluded	absent	32.35	-0.24	excluded	absent	21.84	-0.25	excluded	absent	3.33	-0.28	excluded	absent	2.12	-0.28	excluded	absent	21.24	-0.25	excluded	absent	44.27	-0.22	excluded	absent	37.43	-0.23	excluded	absent	21.86	-0.25	excluded	absent	4.55	-0.28
232057_at	included	present	2769.98	-0.11	included	present	4052.01	0.77	included	present	6297.05	2.3	included	present	2207.37	-0.49	included	present	1889.63	-0.71	included	present	2757.43	-0.12	included	present	1714.36	-0.83	included	present	2270.44	-0.45	included	present	1847.77	-0.74	included	present	1696.57	-0.84	included	present	2202.58	-0.49	included	present	1492.83	-0.98	included	present	2006.2	-0.63	included	present	1454.85	-1	included	present	1460.98	-1
232058_at	included	present	573.86	-0.39	included	present	200.92	-1.23	included	present	275.63	-1.06	included	present	564.9	-0.41	included	present	232.66	-1.16	included	present	519.83	-0.52	included	present	177.52	-1.28	included	present	71.51	-1.52	included	present	380.3	-0.83	included	present	213.78	-1.2	included	present	249.12	-1.12	included	present	100.09	-1.46	included	present	232.15	-1.16	included	present	171.33	-1.3	included	present	60.76	-1.54
232059_at	excluded	absent	50.67	-0.92	excluded	absent	50.17	-0.92	included	present	88	-0.64	included	present	364.22	1.39	excluded	absent	42.58	-0.98	excluded	absent	79.33	-0.71	excluded	absent	79.75	-0.7	included	present	743.39	4.18	included	absent	51.38	-0.91	excluded	absent	43.69	-0.97	excluded	absent	69.02	-0.78	excluded	absent	32.08	-1.05	included	present	355.06	1.32	included	present	365.87	1.4	excluded	absent	29.15	-1.08
232060_at	included	present	172.52	1.94	excluded	absent	49.24	-0.04	excluded	absent	1.37	-0.8	excluded	absent	3.29	-0.77	excluded	absent	63.74	0.2	included	present	211.99	2.57	excluded	absent	2.39	-0.79	excluded	absent	36.57	-0.24	included	present	131.59	1.28	excluded	absent	1.12	-0.81	excluded	absent	28.42	-0.37	excluded	absent	5.49	-0.74	included	present	90.73	0.63	included	present	125.94	1.19	excluded	absent	41.81	-0.16
232061_at	included	present	945.3	5.16	included	present	319.49	0.97	excluded	absent	72.1	-0.68	included	present	1603.27	9.56	excluded	absent	132.8	-0.27	excluded	absent	126.48	-0.32	included	present	114.35	-0.4	included	present	403.31	1.54	included	present	148.04	-0.17	excluded	absent	40.01	-0.89	excluded	absent	93.42	-0.54	excluded	absent	9.56	-1.1	included	present	220.46	0.31	included	present	1155.39	6.56	included	present	1094.28	6.15
232062_at	excluded	absent	44.35	-0.38	excluded	absent	153.2	0.32	excluded	absent	42.86	-0.39	excluded	absent	137.93	0.22	included	present	260.07	1	excluded	absent	79.54	-0.15	excluded	absent	197.65	0.6	excluded	absent	214.01	0.7	excluded	absent	63.24	-0.26	included	present	329.07	1.44	included	present	270.19	1.06	excluded	absent	171.35	0.43	excluded	absent	40	-0.41	excluded	absent	31.66	-0.46	included	present	311.35	1.33
232063_x_at	included	present	237.22	-0.36	included	present	371.55	0.8	included	present	290.64	0.1	included	present	439.37	1.39	included	present	315.71	0.32	included	present	170.64	-0.94	included	present	312.84	0.29	included	present	81.26	-1.72	included	present	576.97	2.58	included	present	138.8	-1.22	included	present	482.42	1.76	included	present	160.35	-1.03	included	present	182.3	-0.84	included	present	212.87	-0.58	included	present	143.3	-1.18

[illegible]

232389_at	excluded	absent	66.5	-0.68	excluded	absent	17.1	-0.85
232390_at	included	present	215.52	-0.24	excluded	absent	119.09	-0.44
232391_at	excluded	absent	19.72	-0.71	excluded	absent	2.46	-1.24
232392_at	included	present	1734.24	0.8	included	present	656.1	-0.72
232393_at	included	present	149.6	1.36	excluded	absent	6.35	-1.29
232394_at	excluded	absent	63.46	0.01	excluded	absent	92.05	0.72
232395_x_at	included	present	102.41	-1.09	included	present	351.66	0.78
232396_at	included	present	176.34	0.55	excluded	absent	150.17	0.2
232397_at	excluded	absent	49.12	-0.03	excluded	absent	55.38	0.04
232398_at	excluded	absent	48.11	-0.33	included	present	243.3	0.1
232399_at	included	present	189.26	0.52	included	present	213.45	0.71
232400_at	included	present	178.26	2.21	excluded	absent	75.73	0.12
232401_at	excluded	absent	104.08	-0.58	excluded	absent	2.64	-1.02
232402_at	excluded	absent	67.31	-0.54	excluded	absent	27.99	-0.93
232403_at	included	present	844.43	-0.33	included	present	459.27	-1.72
232404_at	included	present	559.08	-0.23	included	present	125.71	-1.35
232405_at	excluded	absent	128.67	0.14	excluded	absent	13.47	-1.36
232406_at	included	present	938.81	-0.06	included	absent	201.79	-0.63
232407_at	excluded	absent	5.61	-0.19	excluded	absent	2.4	-0.19
232408_at	included	present	121.12	0.52	excluded	absent	98.93	0.17
232409_x_at	excluded	absent	3.09	-0.79	excluded	absent	2.27	-0.82
232410_at	included	present	112.2	0.08	excluded	absent	67.74	-0.89
232411_at	excluded	absent	33.89	-0.41	excluded	absent	48.93	-0.34
232412_at	included	present	137.13	0.44	included	absent	84.46	-0.74
232413_at	included	absent	110.81	-0.24	included	present	139.49	0.23
232414_at	excluded	absent	44.51	-0.28	excluded	absent	42.3	-0.34
232415_at	excluded	absent	4.86	-1.68	excluded	absent	31.56	-1.11
232416_at	included	present	187.18	-0.49	included	present	1032.26	0.51
232417_x_at	included	absent	79.22	-1.27	included	present	685.12	0.04
232418_at	excluded	absent	4.17	-0.64	excluded	absent	2.56	-0.7
232419_at	excluded	absent	40.07	0.09	excluded	absent	77.15	1.12
232420_x_at	included	present	455.09	0.7	included	absent	237.58	-0.29
232421_at	excluded	absent	119.29	0.14	excluded	absent	97.3	0
232422_at	excluded	absent	3.42	-0.37	excluded	absent	42.73	-0.21
232423_at	included	present	170.73	1.54	excluded	absent	78.18	0.26
232424_at	included	present	776.7	2.11	included	absent	2.57	-1.26
232425_at	included	present	408.13	-1.31	included	present	248.08	-1.62
232426_at	excluded	absent	76.96	-0.45	excluded	absent	40.84	-0.52
232427_at	included	present	168.86	-0.74	included	present	233.96	-0.32
232428_at	excluded	absent	59.36	-0.13	excluded	absent	7.18	-0.31
232429_at	included	present	244.18	0.62	included	present	74.65	-0.86
232430_at	included	present	83.52	-0.73	included	absent	12.59	-1.43
232431_at	included	present	150.12	0.03	included	absent	63.73	-1.24
232432_s_at	included	present	1078.38	1.62	included	present	588.67	-0.18
232433_at	excluded	absent	70.39	-0.06	excluded	absent	23.59	-0.53
232434_at	excluded	absent	8.51	-0.72	excluded	absent	121.81	1.12
232435_at	included	present	1017.74	-0.12	included	present	1037.92	-0.08
232436_at	included	present	309.06	1.46	included	present	155.67	-0.24
232437_at	excluded	absent	176.22	-0.52	excluded	absent	115.56	-1.29
232438_at	excluded	absent	23.33	-1.06	excluded	absent	10.05	-1.23
232439_at	included	present	126.2	0.24	included	present	155.03	0.73
232440_at	excluded	absent	9.64	-1.15	excluded	absent	37.45	-0.76
232441_at	included	present	176.19	0.22	excluded	absent	94.46	-0.69
232442_at	excluded	absent	84.84	-0.18	excluded	absent	48.66	-0.8
232443_at	excluded	absent	10.06	0.18	excluded	absent	3.44	-0.36
232444_at	included	present	322.59	1.29	included	absent	37.67	-1.91
232445_at	excluded	absent	55.61	-0.57	excluded	absent	129.02	0.55
232446_at	included	present	125.3	0.59	included	present	1855.08	29.93
232447_at	included	present	112.73	-0.66	included	absent	110.87	-0.69
232448_at	excluded	absent	23.34	0.01	excluded	absent	11.4	-0.29
232449_at	included	present	201.25	-0.41	included	present	249.75	-0.39
232450_at	excluded	absent	34.77	-0.28	excluded	absent	55.76	0.36
232451_at	excluded	absent	2.24	-1.4	excluded	absent	4.61	-1.34
232452_at	excluded	absent	76.08	1.19	included	present	340.1	8.12
232453_at	included	present	125.09	0.91	included	present	155.45	1.35
232454_at	included	absent	146.12	-0.83	included	present	395.25	2.27
232455_x_at	included	present	149.21	0.05	excluded	absent	118.12	-0.39
232456_at	excluded	absent	96.21	0.84	excluded	absent	47.32	0.12
232457_at	included	present	181.17	-0.72	included	absent	190.73	-0.69
232458_at	included	present	627.47	1.16	excluded	absent	74.63	-0.49
232459_at	included	present	162.89	-0.45	included	absent	60.01	-0.98
232460_at	included	present	212.81	1.01	included	present	510.83	5.59
232461_at	included	present	159.41	0.74	excluded	absent	77.91	-0.5
232462_s_at	included	present	481.27	1.97	included	present	510.7	2.22
232463_at	included	present	142.19	0.59	excluded	absent	42.23	-0.88
232464_at	included	present	732.1	0.68	included	present	267.77	-0.69
232465_at	excluded	absent	102.99	-0.12	excluded	absent	43.81	-0.84
232466_at	included	present	856.28	0.53	included	present	164.13	-1.16
232467_at	included	present	166.85	1.51	excluded	absent	36.37	-0.47
232468_at	included	present	149.65	0.68	excluded	absent	87.55	-0.4
232469_x_at	included	present	253.28	-1.42	included	present	624.67	0.66
232470_at	excluded	absent	35.2	-0.77	excluded	absent	68.44	-0.1
232471_at	included	present	124.32	-0.31	excluded	absent	17.84	-1.1
232472_at	included	present	236.76	-0.12	included	absent	78.67	-0.84
232473_at	included	present	340.1	-0.58	included	present	380.55	-0.3
232474_at	excluded	absent	74.99	0.54	excluded	absent	83.9	0.73
232475_at	excluded	absent	69.86	-0.34	excluded	absent	169.99	1.5
232476_at	included	present	212.29	-0.44	included	present	366.03	0.09
232477_at	included	present	421.68	1.49	included	present	203.09	-0.79
232478_at	included	present	181.33	-0.48	included	present	252.16	-0.29
232479_at	excluded	absent	2.02	-0.79	excluded	absent	48.64	0.22
232480_at	excluded	absent	82.9	0.23	excluded	absent	64.11	-0.08
232481_s_at	included	present	610.42	0.36	included	present	736.09	0.56
232482_at	excluded	absent	1.42	-0.6	excluded	absent	30.77	-0.19
232483_at	included	present	97.01	0.76	excluded	absent	4.3	-1.2
232484_at	excluded	absent	135.59	0.24	excluded	absent	33.6	-0.92
232485_at	included	present	210.72	-0.07	excluded	absent	117.59	-0.37
232486_at	included	present	829.29	-0.22	included	present	3861.97	2.28
232487_at	excluded	absent	46.44	-0.13	excluded	absent	3.7	-1.18
232488_at	included	present	391.94	-0.41	included	present	238.86	-0.87
232489_at	included	present	479.56	0.25	included	present	361.31	-0.27
232490_s_at	included	present	322.32	-0.31	included	present	450.14	0.78
232491_at	excluded	absent	40.66	-0.75	excluded	absent	2.21	-1.03
232492_at	excluded	absent	139.23	0.12	excluded	absent	119.6	-0.01
232493_at	included	present	440.87	1.53	included	present	248.02	-0.54
232494_at	excluded	absent	4.12	-0.18	excluded	absent	34.59	-0.15
232495_x_at	included	present	1456.66	-0.33	included	present	1613.7	0.03
232496_at	included	present	109.04	-0.39	included	present	88.99	-0.72
232497_at	included	present	191.8	-0.12	included	present	688.29	5.79
232498_at	included	present	905.22	-0.36	included	present	501.77	-0.86
232499_at	excluded	absent	27.21	-0.15	excluded	absent	31.92	-0.03
232500_at	included	present	328.8	-0.23	included	present	213.83	-0.47
232501_at	included	present	526.19	-0.67	included	present	6083.15	5.84
232502_at	excluded	absent	69.01	-0.47	included	present	104.89	0.14
232503_at	included	present	614.68	1.91	included	present	506.94	1.11
232504_at	included	present	266.61	0.63	included	present	180.67	0.23
232505_at	included	present	478.53	-0.5	included	present	714.95	0.2
232506_s_at	included	present	192.93	-0.19	included	present	353.44	0.09
232507_at	excluded	absent	37.97	-0.47	included	present	85.77	0.03
232508_at	excluded	absent	92.52	0.11	excluded	absent	15.77	-1.11
232509_at	excluded	absent	200.48	-0.18	excluded	absent	173.75	-0.29
232510_s_at	included	present	293.53	-0.32	included	present	857.67	3.37
232511_at	included	present	570.99	-0.17	included	present	178.22	-0.93
232512_at	included	present	1567.81	0.63	included	present	2168.74	1.25
232513_x_at	excluded	absent	7.49	-0.26	excluded	absent	68.07	-0.21
232514_at	excluded	absent	3.45	-0.59	excluded	absent	81.6	-0.27
232515_at	included	present	379.85	-0.94	included	present	593.13	-0.15
232516_x_at	included	present	979.08	0.87	included	present	922	0.66
232517_s_at	excluded	absent	56.59	0.15	excluded	absent	72.19	0.49
232518_at	excluded	absent	3.09	-1.03	excluded	absent	43.29	0.27
232519_at	excluded	absent	51.37	-0.25	excluded	absent	84.22	0.42
232520_s_at	included	present	1068.81	2.44	included	present	1870.41	6.87
232521_at	included	present	883.13	-0.55	included	present	780.05	-0.64
232522_at	included	present	100.41	-0.23	excluded	absent	8.97	-1.28
232523_at	included	absent	23.73	-0.75	included	absent	105.74	-0.7
232524_x_at	included	present	332.56	-0.7	included	present	613.44	-0.2
232525_at	included	present	190.46	-0.08	included	present	167.34	-0.35
232526_at	excluded	absent	67.39	-0.01	excluded	absent	4.35	-1.43
232527_at	included	present	1515.33	0.34	included	present	734.25	-0.85
232528_at	included	present	455.04	0.23	included	absent	110.14	-1.87
232529_at	included	present	1591.67	-0.28	included	present	1342.67	-0.55
232530_at	included	absent	65.61	-0.6	included	absent	8.09	-0.88
232531_at	included	absent	37.89	-0.76	included	absent	31.21	-0.77
232532_at	excluded	absent	69.97	-0.39	excluded	absent	89.19	-0.07
232533_at	included	absent	109.09	-0.53	included	absent	19.09	-1.78
232534_at	excluded	absent	9.92	-0.3	excluded	absent	6.74	-0.61
232535_at	included	present	138.91	-0.64	included	present	235.29	0.96
232536_at	excluded	absent	65.45	-0.03	excluded	absent	43.99	-0.38
232537_x_at	included	present	160.58	-0.05	included	present	161.87	-0.03
232538_at	included	present	404.36	1.18	included	present	353.54	0.87
232539_at	excluded	absent	18.9	-0.89	excluded	absent	177.41	0.74
232540_x_at	included	present	342.57	-0.76	included	present	398.69	-0.47
232541_at	included	present	906.58	0.89	included	absent	68.41	-0.52
232542_at	excluded	absent	5.63	-0.8	excluded	absent	114.41	1.09
232543_x_at	included	present	169.65	-0.44	included	absent	99.52	-0.54
232544_at	included	present	1206.15	2.75	included	absent	110.22	-0.59
232545_at	excluded	absent	3.49	-0.63	excluded	absent	6.75	-0.48
232546_at	excluded	absent	36.66	0.43	excluded	absent	81.35	2.03
232547_at	included	present	283.41	-0.6	included	present	1431.02	0.02
232548_at	excluded	absent	40.97	0.06	excluded	absent	77.2	0.93
232549_at	included	present	128.75	0.4	excluded	absent	77.19	-0.29
232550_at	excluded	absent	58.05	-0.12	excluded	absent	160.85	2.29
232551_at	excluded	absent	35.09	-0.62	excluded	absent	5.7	-1.26
232552_at	included	absent	79.83	-0.75	included	present	241.04	-0.01
232553_at	included	present	216.55	0.11	included	present	108.94	-0.42
232554_at	excluded	absent	104.24	0.03	excluded	absent	24.4	-1.1
232555_at	included	present	268.19	-0.33	included	absent	62.25	-0.65
232556_at	included	present	1329	-0.14	included	absent	238.28	-0.76
232557_at	excluded	absent	148.07	0.5	excluded	absent	142.3	0.41
232558_at	excluded	absent	99.25	1.17	excluded	absent	40.62	-0.59
232559_at	included	present	140.89	0.27	excluded	absent	55.37	-1.28
232560_at	included	present	113.24	0.7	excluded	absent	75.79	-0.23
232561_at	excluded	absent	55.69	1.53	excluded	absent	4.78	-0.43
232562_at	included	present	251.63	-0.53	included	present	268.05	-0.51
232563_at	included	present	122.1	-0.09	excluded	absent	72.07	-0.59
232564_at	excluded	absent	103.49	0.17	excluded	absent	7.32	-0.95
232565_at	included	present	228.41	-0.99	included	present	224.25	-1.01
232566_at	excluded	absent	22.07	-0.34	excluded	absent	22.94	-0.32
232567_at	included	present	109.5	0.69	excluded	absent	21.32	-0.31
232568_at	included	present	540.71	0.05	included	present	98.09	-0.47
232569_at	included	present	327.7	-0.05	included	present	606.46	0.78
232570_s_at	included	present	187.75	-0.25	excluded	absent	3.33	-0.84
232571_at	excluded	absent	98.63	-0.17	included	present	191.28	1.92
232572_at	excluded	absent	28.51	-0.99	excluded	absent	15.78	-1.16
232573_at	included	absent	8.2	-0.52	included	absent	25.13	-0.5
232574_at	excluded	absent	174.76	-0.29	included	present	382.14	1.57
232575_at	excluded	absent	34.64	-0.75	excluded	absent	30.89	-0.78
232576_at	included	present	214.92	0.59	included	absent	44.44	-2.56
232577_at	excluded	absent	2.16	-0.46	excluded	absent	6.88	-0.4
232578_at	excluded	absent	2.76	-1.05	excluded	absent	0.92	-1.1
232579_at	included	present	170.16	-0.57	included	present	205.74	-0.24
232580_x_at	excluded	absent	52.97	-0.9	excluded	absent	11.39	-1.18
232581_x_at	excluded	absent	68.62	1.19	excluded	absent	67.4	1.15
232582_at	excluded	absent	92.05	-0.39	excluded	absent	43.01	-0.89
232583_at	excluded	absent	234.88	0.19	excluded	absent	177.4	-0.18
232584_at	included	present	260.2	-0.65	included	present	460.91	-0.22
232585_at	included	absent	107.17	-1.4	included	present	158.11	-0.78
232586_x_at	included	present	265.68	2.82	included	present	141.7	0.5
232587_at	included	present	328.7	-0.24	included	present	236.17	-0.6
232588_at	excluded	absent	129.1	0.4	excluded	absent	3.67	-2.17
232589_at	included	absent	110.96	-0.91	included	present	160.73	-0.53
232590_at	included	present	107.44	-0.8	included	absent	84.62	-1.11
232591_s_at	included	present	1145.37	2.4	included	present	580.41	-0.3
232592_at	included	present	338.49	-0.99	included	present	280.1	-1.26
232593_at	included	present	110.17	-0.08	excluded	absent	76.52	-0.19
232594_at	included	absent	110.4	-1.18	included	present	267.5	-0.12
232595_at	included	absent	128.78	-0.43	included	absent	4.11	-2.37
232596_at	excluded	absent	3.68	-0.24	included	present	291.73	0.13
232597_x_at	included	present	2523.16	-0.22	included	present	2621.15	-0.14
232598_at	excluded	absent	62.91	-0.19	excluded	absent	79.36	-0.12
232599_at	excluded	absent	64.13	-0.7	excluded	absent	76.97	-0.4
232600_at	included	present	377.52	-0.63	included	present	1346.27	1.85
232601_at	included	present	1317.45	-0.57	included	present	1101.25	-0.88
232602_at	included	present	90.24	-0.24	excluded	absent	106.69	-0.21
232603_at	excluded	absent	3.97	-0.67	excluded	absent	3.86	-0.67
232604_at	included	present	123.85	-0.09	excluded	absent	75.05	-0.33
232605_s_at	included	present	109.67	-0.1	excluded	absent	48.44	-0.58
232606_at	excluded	absent	59.12	-0.39	excluded	absent	68.7	-0.27
232607_at	excluded	absent	149.18	1.05	excluded	absent	75.91	-0.02
232608_x_at	excluded	absent	99.14	0.35	excluded	absent	107.71	0.47
232609_at	excluded	absent	5.08	-0.89	excluded	absent	46.63	-0.39
232610_at	included	present	912.03	0.22	included	absent	40.36	-0.59
232611_at	included	absent	66.08	-0.78	included	present	415.22	0.79
232612_s_at	included	present	1702.7	3.05	included	present	1934.59	4.05
232613_at	included	present	163.07	-0.13	excluded	absent	177	0.06
232614_at	included	present	657.42	-0.02	included	present	528.63	-0.22
232615_at	included	present	2036.18	-0.11	included	present	975.37	-0.82
232616_at	excluded	absent	10.98	-0.56	excluded	absent	5.57	-0.73
232617_at	included	present	696.43	0.11	included	present	555.07	-0.07
232618_at	excluded	absent	3.78	-0.54	excluded	absent	72.3	-0.4
232619_at	excluded	absent	125.69	0.29	excluded	absent	253.81	1.95
232620_at	excluded	absent	5.89	-0.75	excluded	absent	64.98	0.07
232621_at	excluded	absent	226.98	0.86	excluded	absent	81.48	-0.9
232622_at	included	present	332.25	0.79	included	present	109.56	-1.5
232623_at	excluded	absent	5.34	-0.51	excluded	absent	9.21	-0.44
232624_at	excluded	absent	5.05	-0.6	excluded	absent	6.01	-0.5
232625_at	excluded	absent	86.34	0.91	excluded	absent	4.27	-0.76
232626_at	included	present	156.37	-0.29	included	present	187.9	0.16
232627_at	excluded	absent	53.85	-0.66	excluded	absent	54.68	-0.65
232628_at	included	present	278.87	-0.34	included	present	125.02	-1.18
232629_at	excluded	absent	71.34	-0.22	included	present	99.06	-0.16
232630_at	excluded	absent	65.03	0.44	included	present	95.91	1.25
232631_at	excluded	absent	30.4	0.25	excluded	absent	41.43	0.64
232632_at	included	present	182.66	-0.18	included	present	124.57	-0.91
232633_at	included	present	125.08	-0.14	included	absent	70.89	-1.01
232634_at	excluded	absent	3.64	-0.5	excluded	absent	9.39	-0.2
232635_at	included	present	76.7	-0.53	included	present	179.53	0.86
232636_at	included	absent	4.32	-0.8	included	absent	154.32	-0.55
232637_at	included	present	557.11	0.1	included	present	316.83	-0.87
232638_at	excluded	absent	63.42	1.55	excluded	absent	5.6	-0.63
232639_at	excluded	absent	5.95	-0.58	excluded	absent	4.14	-0.6
232640_at	excluded	absent	82.03	-0.53	excluded	absent	195.56	0.72
232641_at	included	present	83.18	-0.28	excluded	absent	9.18	-1.18
232642_at	excluded	absent	58.63	0.51	excluded	absent	39.86	0.11
232643_at	included	present	403.57	0.08	excluded	absent	22.12	-0.7
232644_x_at	included	present	9044.71	0.09	included	present	5593.68	-1.07
232645_at	included	present	249.1	0.49	included	absent	162.22	-0.33
232646_at	excluded	absent	3.64	-0.82	excluded	absent	15.88	-0.53
232647_at	included	present	93.31	-0.03	excluded	absent	73.16	-0.16
232648_at	included	present	2401.18	0.42	included	present	1253.62	-1.02
232649_at	excluded	absent	121.19	0.25	excluded	absent	68.31	-0.16
232650_at	excluded	absent	83.37	1.19	excluded	absent	15.03	-0.67
232651_at	excluded	absent	101.85	0.56	excluded	absent	174.79	1.77
232652_x_at	excluded	absent	17.44	-2.12	excluded	absent	176.99	0.07
232653_at	included	present	714.78	-1.18	included	present	243.57	-1.88
232654_s_at	excluded	absent	2.85	-0.26	excluded	absent	28.98	-0.24
232655_at	excluded	absent	34.36	0.58	excluded	absent	10.01	-0.45
232656_at	excluded	absent	78.42	1.63	excluded	absent	38.38	0.41
232657_at	excluded	absent	2.89	-0.35	excluded	absent	2.62	-0.36
232658_at	excluded	absent	16.62	-1.78	excluded	absent	36.36	-1.45
232659_at	included	present	218.95	0.82	included	present	209.76	0.69
232660_at	excluded	absent	11.59	-1.35	included	present	127.85	0.5
232661_s_at	included	present	1365.61	0.17	included	present	1546.71	0.43
232662_x_at	included	present	976.53	0.09	included	present	509.12	-0.71
232663_s_at	excluded	absent	140.83	0.23	excluded	absent	80.4	-0.59
232664_at	excluded	absent	50.36	-0.09	included	present	281.72	2.66
232665_x_at	included	present	1266.66	0.51	included	present	763.13	-0.38
232666_at	excluded	absent	48.71	0.8	excluded	absent	14.16	-0.26
232667_at	included	present	495.48	1.58	included	present	167.16	0.11
232668_at	included	present	153.04	-0.07	included	absent	6.89	-1.31
232669_at	excluded	absent	11.06	-0.85	excluded	absent	34.77	-0.69
232670_at	included	present	490.56	0.51	included	present	208.28	-1.05
232671_at	excluded	absent	47.2	-0.2	excluded	absent	41.75	-0.28
232672_x_at	included	present	303.54	-0.32	included	present	571.5	0.92
232673_at	excluded	absent	50.48	-0.62	excluded	absent	4.25	-1.71
232674_at	excluded	absent	73.89	0	excluded	absent	14	-0.69
232675_s_at	excluded	absent	108.95	0.45	excluded	absent	106.44	0.41
232676_x_at	included	present	1221.66	-0.05	included	present	2865.13	1.94
232677_at	excluded	absent	75.14	0.38	excluded	absent	41.21	-0.34
232678_at	excluded	absent	88.29	0.59	excluded	absent	45.72	-0.2
232679_at	excluded	absent	51.68	-0.25	included	present	546.88	0.26
232680_at	excluded	absent	1.57	-0.26	excluded	absent	49.91	-0.17
232681_at	included	present	722.21	-0.09	included	present	625.74	-0.38
232682_at	excluded	absent	29.36	-0.67	excluded	absent	159.65	1.17
232683_s_at	included	present	916.45	-0.2	included	present	2238.84	4.09
232684_at	included	present	118.33	0.54	excluded	absent	42.68	-0.83
232685_at	included	present	166.33	0.09	excluded	absent	116.96	-0.39
232686_at	excluded	absent	42.46	-0.28	excluded	absent	68.64	0.12
232687_at	included	present	116.73	-0.56	included	present	539.46	2
232688_at	included	present	211.55	0.42	included	absent	44.06	-1.53
232689_at	excluded	absent	80.84	0.39	excluded	absent	61.16	-0.04
232690_at	included	present	151.32	1.42	included	present	188.12	2.22
232691_at	excluded	absent	32.72	-0.11	excluded	absent	39.09	0.13
232692_at	included	present	208.5	-0.53	included	present	662.8	-0.24
232693_s_at	excluded	absent	71.97	-0.8	excluded	absent	17.28	-1.53
232694_at	excluded	absent	12.98	-0.92	excluded	absent	83.6	-0.08
232695_at	excluded	absent	30.47	-0.73	included	present	117.51	-0.38
232696_at	included	present	130.72	-0.84	included	present	420.77	0.44
232697_at	excluded	absent	28.59	0.25	excluded	absent	22.02	0.04
232698_at	excluded	absent	8.69	-0.22	excluded	absent	65.16	-0.19
232699_at	excluded	absent	28.6	0.04	excluded	absent	111.22	2.44
232700_at	included	present	134.03	0.32	excluded	absent	10.86	-1.16
232701_at	excluded	absent	126.21	0.7	excluded	absent	85.8	0.17
232702_at	included	present	293.27	0.39	included	absent	184.03	-0.68
232703_at	included	present	360.85	-0.05	included	present	191.85	-0.94
232704_s_at	included	present	204.78	-0.29	included	absent	82.47	-0.49
232705_at	included	present	206.37	0.34	excluded	absent	122.16	-0.41
232706_s_at	excluded	absent	226	-0.16	excluded	absent	259.8	0.13
232707_at	excluded	absent	3.45	-0.3	excluded	absent	12.12	-0.26
232708_at	excluded	absent	93.9	0.28	excluded	absent	13.48	-1.25
232709_at	excluded	absent	85.74	-0.44	excluded	absent	157.78	0.32
232710_at	included	present	337.47	0.22	included	present	443.03	1.04
232711_at	included	present	95.27	0.34	excluded	absent	1.94	-1.22
232712_at	included	present	838.45	8.73	excluded	absent	9.82	-0.75
232713_at	excluded	absent	58.04	0.58	excluded	absent	78.18	1.19
232714_at	excluded	absent	6.68	-1.26	excluded	absent	71.72	0.31
232715_at	included	present	201.7	2.26	excluded	absent	11.57	-1.2
232716_at	included	present	1336.74	3.46	included	present	208.9	-0.67
232717_at	excluded	absent	40.19	-0.97	excluded	absent	87.98	-0.15
232718_at	excluded	absent	61.01	0.28	excluded	absent	99.25	1.31
232719_at	excluded	absent	3.91	-0.74	excluded	absent	20.78	0.12
232720_at	included	present	847.86	0.62	included	present	1636.19	1.96
232721_at	excluded	absent	68.58	-0.52	included	present	114.76	-0.17
232722_at	included	present	175.56	0.36	excluded	absent	101.11	-0.28
232723_at	excluded	absent	36.47	-0.53	excluded	absent	6.09	-1.35
232724_at	included	present	484.74	0.88	excluded	absent	266.22	-0.13
232725_s_at	excluded	absent	102.59	1.01	excluded	absent	120.49	1.36
232726_at	included	present	218.73	0.66	included	present	174.13	0.29
232727_at	excluded	absent	65	-0.17	excluded	absent	86.66	0.15
232729_at	included	present	622.19	-0.39	included	present	220.09	-0.52
232730_at	included	present	323.02	-0.22	included	present	475.18	1.51
232731_x_at	excluded	absent	5.63	-1.36	excluded	absent	257.61	1.78
232732_at	excluded	absent	59.49	-0.21	excluded	absent	156.62	2.12
232733_s_at	excluded	absent	73.51	-0.45	included	present	151.99	0.5
232734_at	included	present	164.95	-0.04	included	present	81.63	-0.55
232735_at	excluded	absent	61.12	-0.57	included	present	115.74	-0.13
232736_s_at	excluded	absent	95.45	-0.15	excluded	absent	34.23	-0.24
232737_s_at	excluded	absent	42.65	-0.09	excluded	absent	1.19	-0.43
232738_at	excluded	absent	102.15	0.19	included	present	115.1	0.42
232739_at	excluded	absent	5.91	-0.21	excluded	absent	128.55	0.35
232740_at	included	present	700.76	-0.44	included	present	1626.63	2.37
232741_at	included	present	133.13	-0.46	included	absent	100.12	-0.72
232742_at	included	present	287.27	-0.29	included	absent	42.76	-1.62
232743_at	excluded	absent	5.66	-0.48	excluded	absent	13.25	-0.04
232744_x_at	excluded	absent	70.68	-0.78	excluded	absent	66.47	-0.86
232745_x_at	excluded	absent	78.26	-0.4	excluded	absent	16.4	-0.76
232746_at	included	present	210.69	0.1	included	absent	115.5	-0.51
232747_at	included	absent	128.2	-0.42	included	absent	7.04	-1.65
232748_at	excluded	absent	12.77	-0.56	excluded	absent	3.43	-0.86

[illegible]

233108_at	included	present	324.12	0	included	present	584.21	2.34	included	present	777.89	4.08	included	present	490.98	1.5	included	present	755.77	3.88	included	absent	266.25	-0.52	included	absent	320.86	-0.03	excluded	absent	353.19	0.26	included	present	407.63	0.75	included	present	482.91	1.43	included	present	787.46	4.17	included	present	277.54	-0.42	included	present	349.18	0.23	included	present	487.2	1.47	included	present	379.92	0.5
233109_at	excluded	absent	79.89	-0.15	excluded	absent	12.43	-1.01	excluded	absent	39.4	-0.67	excluded	absent	1.25	-1.16	excluded	absent	9.21	-1.05	included	present	153.83	0.8	excluded	absent	37.07	-0.7	excluded	absent	3.79	-1.12	included	present	96.57	0.07	excluded	absent	52.52	-0.5	excluded	absent	8.88	-1.06	included	present	87.13	-0.05	included	present	81.97	-0.12	excluded	absent	36.64	-0.7	excluded	absent	9.35	-1.05
233110_s_at	included	present	102.76	-0.15	excluded	absent	121.96	0.14	excluded	absent	61.75	-0.77	excluded	absent	52.39	-0.91	included	present	66.44	-0.7	included	present	244.49	1.99	excluded	absent	146.06	0.5	included	present	186.05	1.11	included	present	169.1	0.85	included	present	118.95	0.1	excluded	absent	105.36	-0.11	included	present	217.22	1.58	included	present	95.21	-0.26	included	present	90.54	-0.33	included	present	71.87	-0.62
233111_at	included	present	197.46	-0.13	excluded	absent	76.07	-0.4	excluded	absent	84.31	-0.38	excluded	absent	49.42	-0.46	included	present	265.34	0.02	included	present	86.01	-0.38	excluded	absent	40.87	-0.48	excluded	absent	5.87	-0.56	included	present	190.68	-0.14	excluded	absent	30.86	-0.5	included	present	98.39	-0.35	included	present	241.24	-0.03	excluded	absent	57.13	-0.44	included	present	138.44	-0.26	excluded	absent	58.33	-0.44
233112_at	excluded	absent	17.88	-0.81	excluded	absent	62.09	-0.12	included	present	153.44	1.3	excluded	absent	6.96	-0.98	excluded	absent	174.1	1.63	excluded	absent	17.59	-0.81	included	present	104.33	0.54	excluded	absent	7.04	-0.98	included	present	75.52	0.09	included	present	234.47	2.57	excluded	absent	107.35	0.59	excluded	absent	5.87	-1	excluded	absent	176.61	1.67	excluded	absent	92.39	0.35	excluded	absent	43	-0.42
233113_at	included	present	132.81	0.36	excluded	absent	7.83	-1.12	excluded	absent	77.82	-0.29	excluded	absent	41.03	-0.73	excluded	absent	5.79	-1.15	excluded	absent	137.63	0.42	excluded	absent	2.19	-1.19	excluded	absent	52.01	-0.6	included	present	110.71	0.1	included	present	204.29	1.21	included	present	194.26	1.09	excluded	absent	2.52	-1.19	excluded	absent	9.18	-1.11	excluded	absent	24.2	-0.93	excluded	absent	14.93	-1.04
233114_at	included	present	101.05	-1.09	included	present	242.3	0.61	included	present	116.01	-0.91	included	present	214.87	0.28	included	present	237.36	0.55	included	present	159.86	-0.39	included	present	150.01	-0.5	included	absent	28.36	-1.97	included	present	185.82	-0.07	included	absent	110.24	-0.98	included	present	105.68	-1.04	included	absent	91.03	-1.22	included	present	226.93	0.42	included	absent	141.47	-0.61	included	present	41.02	-1.82
233115_at	excluded	absent	44.89	0.5	excluded	absent	42.52	0.43	included	present	114.91	2.56	included	present	117.41	2.63	excluded	absent	175.43	4.34	excluded	absent	2.72	-0.74	excluded	absent	1.72	-0.77	excluded	absent	40.48	0.37	excluded	absent	27.37	-0.01	excluded	absent	9.81	-0.53	excluded	absent	32.55	0.14	excluded	absent	104.65	2.26	excluded	absent	64.08	1.06	excluded	absent	12.95	-0.44	excluded	absent	58.93	0.91
233116_at	excluded	absent	157.87	0.6	excluded	absent	19.83	-1.34	excluded	absent	147.06	0.45	excluded	absent	6.9	-1.52	excluded	absent	36.1	-1.11	excluded	absent	14	-1.42	excluded	absent	67.37	-0.67	included	present	119.6	0.06	excluded	absent	84.94	-0.43	excluded	absent	52.07	-0.89	excluded	absent	59.56	-0.78	excluded	absent	54.17	-0.86	excluded	absent	70.38	-0.63	excluded	absent	58.18	-0.8	excluded	absent	43.4	-1.01
233117_at	included	present	218.79	0.73	included	present	102.37	-0.28	included	present	552.25	3.64	excluded	absent	74.73	-0.52	included	present	209.14	0.65	excluded	absent	76.14	-0.51	included	present	208.08	0.64	excluded	absent	75.77	-0.51	included	present	140.32	0.05	included	present	181.78	0.41	included	present	170.07	0.31	excluded	absent	132.9	-0.02	included	present	141.34	0.06	excluded	absent	59.73	-0.65	included	present	53.38	-0.71
233118_at	included	present	256.94	-0.49	included	absent	26.7	-0.99	included	present	76.56	-0.88	included	present	182.86	-0.65	included	present	354.86	-0.28	included	present	553.19	0.16	included	present	89.16	-0.85	included	absent	5.74	-1.04	included	present	257.45	-0.49	included	absent	54.36	-0.93	included	absent	65.65	-0.9	included	present	332.62	-0.32	included	present	202.4	-0.61	included	present	200.79	-0.61	included	absent	33.35	-0.97
233119_at	excluded	absent	5.4	-0.47	excluded	absent	6.99	-0.37	excluded	absent	4.95	-0.5	excluded	absent	15.68	0.17	excluded	absent	11.57	-0.09	excluded	absent	7.32	-0.35	excluded	absent	5.23	-0.48	excluded	absent	11.77	-0.07	excluded	absent	7.4	-0.35	excluded	absent	6.39	-0.41	excluded	absent	12.84	-0.01	excluded	absent	21.55	0.53	excluded	absent	16.78	0.24	excluded	absent	4.13	-0.55	excluded	absent	14.8	0.11
233120_at	excluded	absent	40.69	-0.3	excluded	absent	15.54	-0.89	excluded	absent	7.12	-1.09	excluded	absent	41.03	-0.3	excluded	absent	68.4	0.34	excluded	absent	19.87	-0.79	excluded	absent	22.38	-0.73	excluded	absent	2.1	-1.21	excluded	absent	59.57	0.14	excluded	absent	51.38	-0.05	excluded	absent	56.7	0.07	excluded	absent	2.98	-1.19	excluded	absent	40.07	-0.32	excluded	absent	10.78	-1	excluded	absent	20.33	-0.78
233121_at	excluded	absent	117.8	0.24	excluded	absent	40.69	-1.17	excluded	absent	32.36	-1.32	included	present	95.59	-0.16	excluded	absent	87.09	-0.32	included	present	96.55	-0.15	excluded	absent	34.92	-1.27	included	present	66.54	-0.69	included	present	82.11	-0.41	included	present	65.28	-0.72	excluded	absent	25.78	-1.44	excluded	absent	77.41	-0.49	excluded	absent	54.46	-0.91	excluded	absent	36.74	-1.24	excluded	absent	16.28	-1.61
233122_at	included	present	357.14	1.35	included	present	224.45	0.41	included	present	539.43	2.65	included	present	735.33	4.05	included	present	643.94	3.4	included	present	281.99	0.82	included	present	387.02	1.57	included	present	105.8	-0.44	included	present	390.36	1.59	included	present	174.19	0.05	included	present	223.54	0.4	included	present	72.6	-0.67	included	present	131.44	-0.25	included	present	80.1	-0.62	included	present	140.74	-0.19
233123_at	included	present	323.4	-0.08	included	absent	58.19	-0.65	included	present	254.01	-0.23	included	present	128.31	-0.5	included	present	296.65	-0.14	included	present	205.16	-0.33	included	present	135.05	-0.48	included	present	82.08	-0.6	included	present	375.25	0.03	included	present	124.43	-0.51	included	present	101.44	-0.55	included	present	135.65	-0.48	included	present	65.4	-0.63	included	absent	46.44	-0.67	included	absent	30.59	-0.71
233124_s_at	included	present	2758.97	-0.19	included	present	2838.53	-0.12	included	present	3712.11	0.65	included	present	1532.79	-1.27	included	present	2360.45	-0.54	included	present	2134.03	-0.74	included	present	1413.17	-1.37	included	present	2763.84	-0.18	included	present	1565.53	-1.24	included	present	1692.46	-1.13	included	present	1185.19	-1.58	included	present	1835.02	-1	included	present	1441.02	-1.35	included	present	2722.23	-0.22	included	present	1422.17	-1.37
233125_at	included	present	151.82	0.22	included	present	159.18	0.33	excluded	absent	116.88	-0.31	included	present	278.39	2.13	excluded	absent	137.2	-0.01	included	present	238.02	1.52	included	present	252.16	1.73	excluded	absent	63.33	-1.12	included	present	235.23	1.48	included	present	77.86	-0.9	included	present	1235.92	16.63	included	present	102.63	-0.53	included	present	299.27	2.45	included	present	226.36	1.34	included	present	201.74	0.97
233126_s_at	excluded	absent	2.72	-0.88	excluded	absent	10.71	-0.77	excluded	absent	74.15	0.16	excluded	absent	4.55	-0.86	excluded	absent	51.21	-0.17	excluded	absent	4.79	-0.85	excluded	absent	33.7	-0.43	excluded	absent	18.1	-0.66	excluded	absent	40.24	-0.33	excluded	absent	1.82	-0.9	excluded	absent	48.05	-0.22	excluded	absent	3.21	-0.88	excluded	absent	26.84	-0.53	excluded	absent	26.84	-0.53	excluded	absent	3.64	-0.87
233127_at	included	present	84.03	-0.45	excluded	absent	7.75	-1.14	excluded	absent	89.19	-0.41	excluded	absent	92.9	-0.37	excluded	absent	146.85	0.12	included	present	130.72	-0.03	included	present	113.02	-0.19	excluded	absent	30.63	-0.94	included	present	109.73	-0.22	included	present	77.92	-0.51	excluded	absent	45.91	-0.8	included	present	107.89	-0.24	included	present	200.66	0.61	included	present	311.74	1.62	included	present	47.61	-0.78
233128_at	excluded	absent	166.48	2.86	excluded	absent	162.43	2.77	excluded	absent	40.86	-0.08	excluded	absent	6.55	-0.88	excluded	absent	132.17	2.06	excluded	absent	106.3	1.45	excluded	absent	27.95	-0.38	excluded	absent	45.12	0.02	excluded	absent	94.51	1.18	excluded	absent	54.87	0.25	excluded	absent	11.54	-0.77	excluded	absent	94.52	1.18	excluded	absent	126.19	1.92	excluded	absent	66.28	0.52	excluded	absent	3.94	-0.94
233129_at	excluded	absent	6.59	-0.85	excluded	absent	8.2	-0.84	excluded	absent	8.14	-0.84	excluded	absent	11.71	-0.79	excluded	absent	11.39	-0.8	excluded	absent	5.88	-0.86	excluded	absent	6.45	-0.86	excluded	absent	5.18	-0.87	excluded	absent	30.25	-0.58	excluded	absent	12.16	-0.79	excluded	absent	14.16	-0.77	excluded	absent	4.9	-0.87	excluded	absent	6.02	-0.86	excluded	absent	7.22	-0.85	excluded	absent	8.23	-0.83
233130_at	included	absent	73.35	-1.05	included	absent	5.1	-1.29	included	absent	2.53	-1.29	included	absent	76.93	-1.04	included	absent	74.21	-1.05	included	absent	131.91	-0.85	included	absent	8	-1.28	included	absent	47.26	-1.14	included	present	68.81	-1.07	included	absent	25.14	-1.22	included	absent	3.62	-1.29	included	absent	42.78	-1.16	included	absent	57.88	-1.1	included	absent	30.65	-1.2	included	absent	37.9	-1.17
233131_at	excluded	absent	73.31	-0.47	included	present	148.85	0.09	excluded	absent	56.87	-0.6	excluded	absent	97.52	-0.29	excluded	absent	75.52	-0.46	excluded	absent	84.55	-0.39	excluded	absent	67.24	-0.52	excluded	absent	78.9	-0.43	excluded	absent	71.08	-0.49	excluded	absent	50.47	-0.64	excluded	absent	86.67	-0.37	excluded	absent	37.41	-0.74	excluded	absent	92.9	-0.33	excluded	absent	37.55	-0.74	excluded	absent	28.9	-0.8
233132_at	included	present	841.25	-0.22	included	present	1317.02	0.81	included	present	1209.27	0.57	included	present	1635.18	1.5	included	present	1739.13	1.72	included	present	836.69	-0.23	included	present	778.69	-0.36	included	present	470.81	-1.02	included	present	810.61	-0.29	included	present	1252.19	0.67	included	present	1800.85	1.86	included	present	2207.59	2.74	included	present	634.84	-0.67	included	present	2139.48	2.59	included	present	1619.59	1.46
233133_at	excluded	absent	47.16	-0.05	included	present	97.3	1.11	excluded	absent	1.9	-1.09	included	present	173.08	2.87	included	present	145.71	2.23	excluded	absent	56.9	0.18	included	present	153.86	2.42	excluded	absent	33.32	-0.37	included	present	78.49	0.68	included	present	91.22	0.97	included	present	104.76	1.29	included	present	102.41	1.23	included	present	108.93	1.38	included	present	64.89	0.36	excluded	absent	45.68	-0.08
233134_at	included	present	303.36	0.04	included	present	225.58	-0.09	included	present	312.7	0.05	included	present	1427.13	1.89	included	present	733.53	0.75	excluded	absent	97.9	-0.3	excluded	absent	128.61	-0.25	included	present	417.01	0.22	included	present	386.29	0.17	included	present	315.85	0.06	included	present	614.08	0.55	included	present	376.1	0.16	included	present	1249.55	1.6	included	present	264.04	-0.03	included	present	128.46	-0.25
233135_at	excluded	absent	5.73	-0.98	excluded	absent	87.35	-0.13	excluded	absent	56.68	-0.45	excluded	absent	2.59	-1.01	included	present	86.29	-0.14	excluded	absent	18.34	-0.85	included	present	76.91	-0.24	excluded	absent	32.96	-0.7	excluded	absent	22.29	-0.81	excluded	absent	77.64	-0.23	excluded	absent	44.02	-0.58	excluded	absent	26.03	-0.77	excluded	absent	63.3	-0.38	excluded	absent	3.86	-1	excluded	absent	1	-1.03
233136_at	excluded	absent	84.32	1.61	included	present	87.73	1.72	excluded	absent	3	-0.99	excluded	absent	5.67	-0.9	excluded	absent	13.34	-0.66	excluded	absent	47.89	0.44	excluded	absent	4.33	-0.95	excluded	absent	31.35	-0.08	excluded	absent	32.87	-0.04	excluded	absent	87.21	1.7	excluded	absent	1.46	-1.04	excluded	absent	70.05	1.15	excluded	absent	3.22	-0.98	excluded	absent	8.62	-0.81	included	present	62.15	0.9
233137_at	excluded	absent	138.49	-0.03	excluded	absent	27.9	-0.33	excluded	absent	8.76	-0.38	excluded	absent	6.05	-0.39	excluded	absent	340.44	0.52	excluded	absent	190.3	0.11	excluded	absent	86.92	-0.17	excluded	absent	5	-0.39	included	present	207.2	0.16	excluded	absent	3.71	-0.39	excluded	absent	35.52	-0.31	excluded	absent	173.31	0.07	excluded	absent	111.99	-0.1	excluded	absent	4.82	-0.39	excluded	absent	176.76	0.08
233138_at	included	absent	153.65	-0.74	included	absent	5.56	-1.02	included	absent	155.06	-0.74	included	present	282.25	-0.5	included	present	487.23	-0.11	included	absent	114.13	-0.81	included	absent	120.69	-0.8	included	present	105.17	-0.83	included	present	151.59	-0.74	included	absent	181.47	-0.69	included	absent	100.16	-0.84	included	absent	147.67	-0.75	included	absent	108.21	-0.82	included	absent	2.76	-1.02	included	absent	19.41	-0.99
233139_at	included	present	131.05	-0.55	included	present	295.52	-0.3	included	present	128.68	-0.55	excluded	absent	11.63	-0.73	excluded	absent	68.36	-0.64	excluded	absent	44.97	-0.68	included	present	177.69	-0.48	included	present	472.6	-0.03	included	present	147.56	-0.52	excluded	absent	43.37	-0.68	included	present	782.6	0.45	included	present	284.79	-0.31	included	present	461.9	-0.04	included	present	888.77	0.61	included	present	58.88	-0.66
233140_s_at	included	present	278.58	-1.05	included	present	384.02	-0.15	included	present	350.32	-0.44	included	present	737.89	2.88	included	present	785.18	3.28	included	present	433.03	0.27	included	present	601.05	1.71	included	present	1193.92	6.78	included	present	683.17	2.41	included	present	959.39	4.77	included	present	817.89	3.56	included	present	254.23	-1.26	included	present	439.15	0.32	included	present	1036.64	5.43	included	present	646.87	2.1
233141_s_at	included	present	285.12	0.02	included	present	205.21	-0.39	included	present	309.91	0.15	included	present	144.02	-0.71	included	present	334.67	0.28	included	present	203.98	-0.4	included	present	185.12	-0.5	included	absent	122.48	-0.82	included	present	266.27	-0.08	included	present	163.31	-0.61	included	present	252.03	-0.15	included	absent	29.79	-1.3	included	absent	124.75	-0.81	included	present	114.17	-0.86	included	present	145.48	-0.7
233142_at	excluded	absent	75.71	0.26	included	present	422.31	6.09	included	present	793.55	12.35	excluded	absent	6.35	-0.91	included	present	250.44	3.2	excluded	absent	48.47	-0.2	excluded	absent	73.54	0.22	included	present	196.68	2.29	included	present	91.83	0.53	excluded	absent	86.28	0.43	included	present	862.71	13.51	included	present	292.13	3.9	included	present	149.1	1.49	excluded	absent	56.49	-0.07	included	present	510.03	7.57
233143_at	excluded	absent	24.27	-0.2	excluded	absent	7.99	-0.25	excluded	absent	2.65	-0.27	excluded	absent	32.15	-0.18	excluded	absent	67.73	-0.07	excluded	absent	5.38	-0.26	excluded	absent	7.18	-0.26	excluded	absent	8.38	-0.25	excluded	absent	29.46	-0.19	excluded	absent	23.57	-0.21	excluded	absent	19.78	-0.22	excluded	absent	7.15	-0.26	excluded	absent	9.55	-0.25	excluded	absent	2.84	-0.27	excluded	absent	5.98	-0.26
233144_s_at	excluded	absent	5.6	-0.57	excluded	absent	13.25	-0.25	excluded	absent	39.27	0.84	excluded	absent	2.82	-0.68	excluded	absent	13.16	-0.25	excluded	absent	49.06	1.25	excluded	absent	2.86	-0.68	excluded	absent	37.18	0.75	excluded	absent	15.98	-0.13	excluded	absent	3.66	-0.65	excluded	absent	37.42	0.76	excluded	absent	11.08	-0.34	excluded	absent	10.42	-0.36	excluded	absent	4.08	-0.63	excluded	absent	8.14	-0.46
233145_at	excluded	absent	44.26	-0.66	included	present	162.93	1.43	excluded	absent	8.74	-1.29	excluded	absent	31.58	-0.89	excluded	absent	101.44	0.35	excluded	absent	9.17	-1.28	included	present	154.18	1.28	excluded	absent	70.62	-0.2	excluded	absent	65.71	-0.29	excluded	absent	18.02	-1.13	included	present	160.43	1.39	excluded	absent	139.66	1.02	included	present	121.3	0.7	included	present	101.56	0.35	included	present	72.72	-0.16
233146_at	excluded	absent	6.45	-0.97	excluded	absent	3.78	-1.05	excluded	absent	4.66	-1.02	excluded	absent	6.71	-0.97	excluded	absent	61.95	0.51	excluded	absent	9.98	-0.88	excluded	absent	64.49	0.58	excluded	absent	46.24	0.09	excluded	absent	45.15	0.06	excluded	absent	2.58	-1.08	excluded	absent	120.5	2.08	excluded	absent	9.06	-0.9	excluded	absent	9.63	-0.89	excluded	absent	6.87	-0.96	included	present	78.55	0.96
233147_at	excluded	absent	56.28	1.55	excluded	absent	3.45	-0.59	excluded	absent	3.57	-0.59	excluded	absent	7.9	-0.41	excluded	absent	4.05	-0.57	excluded	absent	4.33	-0.56	excluded	absent	44.13	1.05	excluded	absent	7.94	-0.41	excluded	absent	14.85	-0.13	excluded	absent	15.09	-0.12	excluded	absent	7.25	-0.44	excluded	absent	3.5	-0.59	excluded	absent	12.48	-0.23	excluded	absent	1.44	-0.67	excluded	absent	5.49	-0.51
233148_at	excluded	absent	72.78	0.3	excluded	absent	120.3	1.29	excluded	absent	63.05	0.1	excluded	absent	9.5	-1.02	excluded	absent	16.52	-0.87	excluded	absent	51.94	-0.14	excluded	absent	25	-0.7	excluded	absent	23.66	-0.72	excluded	absent	70.56	0.25	excluded	absent	10.73	-0.99	excluded	absent	88.74	0.63	excluded	absent	8.5	-1.04	excluded	absent	85.9	0.57	excluded	absent	43.23	-0.32	excluded	absent	49.63	-0.18
233149_at	excluded	absent	67.06	-0.26	excluded	absent	2.91	-0.41	excluded	absent	36.71	-0.33	included	present	436.67	0.64	excluded	absent	46.51	-0.31	excluded	absent	7.79	-0.4	excluded	absent	3.52	-0.41	excluded	absent	65.32	-0.26	excluded	absent	9.14	-0.4	excluded	absent	54.8	-0.29	excluded	absent	8.7	-0.4	excluded	absent	59.19	-0.28	excluded	absent	11	-0.39	excluded	absent	5.77	-0.41	excluded	absent	1.59	-0.42

[illegible]

233469_at	excluded	absent	21.41	-0.74	excluded	absent	106.87	1.37	excluded	absent	2.33	-1.21	excluded	absent
233470_at	excluded	absent	10.29	-0.55	excluded	absent	67.53	-0.04	excluded	absent	11.82	-0.53	excluded	absent
233471_at	excluded	absent	5.41	-0.6	excluded	absent	2.67	-0.61	excluded	absent	4.27	-0.61	excluded	absent
233472_at	included	present	248.21	0.23	included	present	169.09	-0.65	included	present	835.13	6.77	included	present
233473_x_at	included	present	1030.25	-0.29	included	present	1050.31	-0.26	included	present	1097.06	-0.18	included	present
233474_at	excluded	absent	59.22	0.33	excluded	absent	22.3	-0.58	excluded	absent	11.08	-0.85	excluded	absent
233475_at	excluded	absent	66.82	0.27	excluded	absent	10.52	-0.69	excluded	absent	13.44	-0.64	included	present
233476_at	excluded	absent	81.02	-0.37	excluded	absent	127.62	0.21	excluded	absent	3.95	-1.34	excluded	absent
233477_at	excluded	absent	5.23	-0.86	excluded	absent	179.5	3.97	excluded	absent	8.91	-0.76	excluded	absent
233478_at	excluded	absent	36.77	-0.05	excluded	absent	5.59	-0.71	included	present	65.72	0.57	excluded	absent
233479_at	excluded	absent	7.01	-1.23	excluded	absent	69.97	-0.3	excluded	absent	52.56	-0.56	excluded	absent
233480_at	excluded	absent	92.44	-0.7	excluded	absent	79.51	-0.8	included	present	119.9	-0.49	included	present
233481_at	excluded	absent	87.06	0.71	excluded	absent	46.58	-0.2	included	present	91.44	0.81	excluded	absent
233482_at	excluded	absent	88.17	-0.45	excluded	absent	21.58	-0.67	excluded	absent	3.05	-0.73	excluded	absent
233483_at	excluded	absent	102.68	0.93	excluded	absent	10.11	-0.5	excluded	absent	14.27	-0.44	excluded	absent
233484_at	included	present	425.37	0.14	excluded	absent	160.26	-0.62	excluded	absent	140.99	-0.68	included	present
233485_at	excluded	absent	60.98	-0.35	excluded	absent	81.06	-0.07	excluded	absent	69.49	-0.23	excluded	absent
233486_at	excluded	absent	30.2	-0.21	excluded	absent	99.86	0.57	excluded	absent	7.97	-0.46	excluded	absent
233487_s_at	excluded	absent	95.29	1.59	excluded	absent	42.1	-0.06	excluded	absent	80.47	1.13	excluded	absent
233488_at	excluded	absent	8.92	-0.56	excluded	absent	6.34	-0.58	excluded	absent	7.57	-0.57	excluded	absent
233489_at	excluded	absent	106.94	-0.12	excluded	absent	52.57	-0.53	included	present	103.1	-0.15	excluded	absent
233490_at	included	present	349.82	1.26	included	present	175.6	-0.21	included	present	865.87	5.63	included	present
233491_at	excluded	absent	62.79	1.21	excluded	absent	43.04	0.51	excluded	absent	57.89	1.03	excluded	absent
233492_s_at	included	present	83.61	0.24	excluded	absent	56.19	-0.19	excluded	absent	1.59	-1.06	excluded	absent
233493_at	included	present	232.24	1.31	excluded	absent	3.84	-0.85	excluded	absent	50.87	-0.4	excluded	absent
233494_at	excluded	absent	80.05	-0.67	excluded	absent	4.74	-1.09	excluded	absent	7.73	-1.07	included	present
233495_at	included	present	351.4	-0.88	included	present	357.23	-0.85	included	present	1065.91	2.18	included	present
233496_s_at	included	present	2953.28	-0.15	included	present	1499.37	-0.64	included	present	2211.27	-0.4	included	present
233497_at	excluded	absent	33.49	-0.61	excluded	absent	47.37	-0.29	excluded	absent	16.84	-1.01	excluded	absent
233498_at	included	absent	8.25	-1.06	included	absent	4.52	-1.07	included	absent	3.01	-1.08	included	present
233499_at	excluded	absent	57.73	-0.52	excluded	absent	86.77	-0.38	excluded	absent	37.98	-0.61	excluded	absent
233500_x_at	included	present	475.39	-0.04	included	present	136.32	-0.37	included	absent	83.83	-0.42	included	present
233501_at	excluded	absent	3.51	-0.37	excluded	absent	3.51	-0.37	excluded	absent	2.41	-0.38	excluded	absent
233502_at	excluded	absent	77.21	-0.16	excluded	absent	6.11	-1.11	excluded	absent	31.68	-0.77	excluded	absent
[illegible]														

233829_at	excluded	absent	60.55	0.17	excluded	absent	18.98	-0.89
233830_at	excluded	absent	19.58	-0.65	included	present	93.28	-0.22
233831_at	included	present	199.76	2.06	excluded	absent	42.4	-0.62
233832_at	excluded	absent	82.3	-0.05	excluded	absent	86.86	0.05
233833_at	included	present	87.69	-0.19	excluded	absent	5.74	-1.87
233834_at	included	present	126.07	-0.92	included	absent	57.35	-1.54
233835_at	excluded	absent	92.71	0.02	excluded	absent	27.08	-0.59
233836_at	included	present	256.49	0.17	included	absent	19.29	-2.22
233837_at	excluded	absent	45.55	-0.25	excluded	absent	7.89	-0.92
233838_at	excluded	absent	3.42	-1.57	excluded	absent	1.47	-1.63
233839_at	included	absent	85.51	-0.6	included	absent	107.17	-0.58
233840_at	included	present	160.26	0.36	excluded	absent	157.94	0.31
233841_s_at	included	present	1671.91	1.75	included	present	3165.67	7.43
233842_x_at	included	present	2193.07	1.01	included	present	2345.15	1.31
233843_at	excluded	absent	35.15	0.69	excluded	absent	66.2	2.22
233844_at	included	present	452.16	0.4	included	absent	94.76	-1.14
233845_at	included	absent	78.03	-0.86	included	absent	4.59	-1.83
233846_at	excluded	absent	162.93	0.44	excluded	absent	45.02	-0.83
233847_x_at	excluded	absent	2.23	-0.42	excluded	absent	1.43	-0.44
233848_x_at	included	present	328.58	-0.39	included	present	267.89	-0.78
233849_s_at	included	present	741.25	-0.01	included	present	220.19	-1.77
233850_s_at	excluded	absent	170.22	0.02	excluded	absent	150.62	-0.2
233851_s_at	included	present	119.45	0.11	included	present	228.62	2.29
233852_at	included	present	353.43	-0.15	included	present	631.87	1.45
233853_at	included	present	136.78	-0.07	included	absent	73.79	-0.98
233854_x_at	excluded	absent	37.38	-0.01	excluded	absent	79.37	1.03
233855_at	excluded	absent	160.18	0.26	included	present	120.46	-0.2
233856_at	excluded	absent	78.32	0.11	excluded	absent	108.78	0.52
233857_s_at	excluded	absent	46.35	0.41	excluded	absent	7.7	-0.6
233858_at	excluded	absent	63.95	0.7	excluded	absent	3.77	-1.03
233859_at	included	present	253.7	0.41	included	present	588.51	3.28
233860_s_at	excluded	absent	3.32	-0.49	excluded	absent	29.02	0.3
233861_at	included	present	98.51	-0.43	included	present	121.87	-0.18
233862_at	included	present	184.12	1.68	excluded	absent	116.93	0.15

234188_at	excluded	absent	43.26	0.06	excluded	absent	45.15	0.1	excluded	absent	7	-0.64	excluded	absent	120.65	1.55
234189_at	excluded	absent	96.28	-0.43	excluded	absent	6.48	-1.2	excluded	absent	5.98	-1.2	excluded	absent	86.99	-0.51
234190_at	excluded	absent	79.05	-0.1	excluded	absent	16.53	-1.53	excluded	absent	47.88	-0.81	excluded	absent	62.83	-0.47
234191_at	excluded	absent	44.68	-0.37	excluded	absent	34.02	-0.63	excluded	absent	69.39	0.23	excluded	absent	11.62	-1.18
234192_s_at	included	present	134.62	-0.38	included	present	540.97	-0.2	included	present	795.7	-0.09	included	present	892.12	-0.05
234193_at	excluded	absent	138.53	0.08	included	absent	48.67	-1.68	included	absent	58.34	-1.49	included	present	101.15	-0.65
234194_at	included	present	157.49	2.52	excluded	absent	63.44	0.36	excluded	absent	31.33	-0.38	excluded	absent	30.15	-0.41
234195_at	excluded	absent	73.98	0.03	excluded	absent	122.13	0.95	excluded	absent	6.22	-1.27	excluded	absent	16.58	-1.07
234196_at	included	present	245.46	0.77	excluded	absent	9.36	-0.82	excluded	absent	82.94	-0.33	excluded	absent	51.56	-0.54
234197_at	excluded	absent	5.03	-0.47	excluded	absent	2.13	-0.48	excluded	absent	51.14	-0.36	excluded	absent	2.06	-0.48
234198_at	excluded	absent	3.94	-0.51	excluded	absent	11.6	-0.24	excluded	absent	3.01	-0.55	excluded	absent	3.89	-0.51
234199_at	excluded	absent	94.18	-0.19	excluded	absent	53.16	-0.51	excluded	absent	70.61	-0.37	excluded	absent	12.38	-0.84
234200_at	excluded	absent	77.63	1.4	excluded	absent	4.39	-0.98	excluded	absent	4.27	-0.99	excluded	absent	4.46	-0.98
234201_x_at	excluded	absent	250.67	1.02	excluded	absent	210.97	0.62	excluded	absent	106.01	-0.45	excluded	absent	10.93	-1.41
234202_at	included	present	108.72	1.09	excluded	absent	42.15	-0.16	excluded	absent	82.88	0.61	excluded	absent	5.05	-0.85
234203_at	included	absent	114.5	-0.87	included	absent	78.2	-1.47	included	absent	62.77	-1.72	included	present	297.36	2.14
234204_at	excluded	absent	47.79	0.16	excluded	absent	21.14	-0.53	excluded	absent	4.5	-0.96	excluded	absent	64.32	0.59
234205_at	excluded	absent	89.89	-0.11	excluded	absent	35.73	-0.72	excluded	absent	3.36	-1.08	excluded	absent	1.86	-1.1
234206_at	included	present	152.78	0.79	excluded	absent	110.92	0.16	excluded	absent	30.43	-1.07	excluded	absent	29.41	-1.08
234207_at	excluded	absent	78.03	-0.58	excluded	absent	66.19	-0.72	excluded	absent	66.66	-0.72	excluded	absent	98.31	-0.34
234208_at	excluded	absent	2.77	-0.65	excluded	absent	41.57	0.75	excluded	absent	13.32	-0.27	excluded	absent	1.09	-0.71
234209_at	excluded	absent	64.6	0.18	excluded	absent	93.46	0.95	excluded	absent	38.4	-0.51	excluded	absent	44.38	-0.36
234210_x_at	included	present	82.43	-0.41	included	present	206.15	0.98	excluded	absent	105.88	-0.15	excluded	absent	59.7	-0.67
234211_at	excluded	absent	20.67	-0.86	excluded	absent	41.56	-0.3	excluded	absent	42.64	-0.27	excluded	absent	112.74	1.61
234212_at	included	present	501.42	-0.21	included	present	439.72	-0.4	included	present	465.59	-0.32	included	present	222.69	-1.06
234213_at	excluded	absent	2.55	-0.64	excluded	absent	3.09	-0.64	excluded	absent	3.46	-0.63	excluded	absent	2.46	-0.64
234214_at	excluded	absent	130.5	0.58	excluded	absent	119.17	0.33	excluded	absent	94.53	-0.21	excluded	absent	100.25	-0.09
234215_at	included	present	313.71	-0.77	included	absent	49.69	-2.57	included	present	551.38	0.85	included	present	306.06	-0.82
234216_at	excluded	absent	32.64	-0.1	excluded	absent	37.44	0.04	excluded	absent	42.4	0.19	excluded	absent	37.44	0.04
234217_at	excluded	absent	37	0.33	excluded	absent	7.61	-0.69	excluded	absent	2.13	-0.88	excluded	absent	16.45	-0.39
234218_at	excluded	absent	24.02	-0.47	excluded	absent	48.62	-0.23	excluded	absent	3.03	-0.67	excluded	absent	1.75	-0.68
234219_at	excluded	absent	5.25	-0.49	excluded	absent	77.48	0	excluded	absent	5.8	-0.49	excluded	absent	32.38	-0.31
234220_at	included	present	918.77	2.48	included	present	284.58	0.24	excluded	absent	131.66	-0.3	excluded	absent	39.92	-0.62
234221_at	excluded	absent	14.29	-0.63	excluded	absent	4.99	-0.72	excluded	absent	1.73	-0.75	excluded	absent	3.33	-0.74
[illegible]	[illegible]	[illegible]	[illegible]	[illegible]	[illegible]	[illegible]	[illegible]	[illegible]	[illegible]	[illegible]	[illegible]	[illegible]	[illegible]	[illegible]	[illegible]	[illegible]

234548_at	excluded	absent	36.56	-0.06	excluded	absent	3.21	-1	excluded	absent	7.97
234549_at	included	present	90.39	-0.45	included	present	136.42	-0.4	included	absent	32.31
234550_at	excluded	absent	2.39	-0.67	excluded	absent	4	-0.62	excluded	absent	2.12
234551_at	excluded	absent	8.53	-0.22	excluded	absent	38.93	2.13	excluded	absent	13.09
234552_at	excluded	absent	3.19	-0.6	excluded	absent	6.32	-0.5	excluded	absent	55.36
234553_at	excluded	absent	5.23	-0.42	excluded	absent	28.31	0.33	excluded	absent	8.41
234554_at	excluded	absent	11.05	-0.56	excluded	absent	45.59	0.44	excluded	absent	81.11
234555_at	excluded	absent	12.85	-0.3	excluded	absent	15.19	-0.2	excluded	absent	4.48
234556_at	excluded	absent	58.16	0.31	excluded	absent	6.45	-0.95	excluded	absent	6.63
234557_at	excluded	absent	5.76	-0.72	excluded	absent	4.75	-0.75	excluded	absent	51.79
234558_at	excluded	absent	73.05	0.08	excluded	absent	4.01	-1.41	excluded	absent	69.76
234559_at	excluded	absent	43.45	-0.67	excluded	absent	92.11	0.18	excluded	absent	47.91
234560_at	excluded	absent	150.06	0.57	excluded	absent	110.84	0.18	excluded	absent	113.48
234561_at	included	present	196.28	-0.54	included	absent	171.21	-0.67	included	absent	9.75
234562_x_at	included	present	1792.03	-0.95	included	present	1456.43	-1.53	included	present	1982.38
234563_at	excluded	absent	144.4	-0.06	excluded	absent	2.61	-0.74	included	present	458.34
234564_at	excluded	absent	2.65	-0.46	excluded	absent	2.79	-0.45	excluded	absent	3.97
234565_x_at	included	present	349.2	0.82	excluded	absent	65.63	-1.37	excluded	absent	378.81
234566_at	excluded	absent	36.64	-1.06	included	present	75.11	-0.46	excluded	absent	68.44
234567_at	included	absent	160.81	-0.56	included	absent	125.85	-0.88	included	absent	99.04
234568_at	excluded	absent	5.13	-1	excluded	absent	98.1	1.42	excluded	absent	124.21
234569_at	excluded	absent	78.3	0.33	excluded	absent	36.29	-0.77	included	present	162.48
234570_at	excluded	absent	95.96	1.23	excluded	absent	9.72	-0.79	excluded	absent	132.32
234571_at	excluded	absent	5.61	-1.68	excluded	absent	2.07	-1.78	excluded	absent	18.84
234572_at	excluded	absent	39.23	0.75	excluded	absent	71.78	2.1	excluded	absent	37.06
234573_at	included	present	309.59	-0.78	included	present	206.42	-1.84	included	present	246.56
234574_at	included	present	154.25	1.15	excluded	absent	60.7	-0.68	excluded	absent	73.11
234575_at	excluded	absent	124.7	0.27	excluded	absent	88.38	-0.44	excluded	absent	182.33
234576_at	excluded	absent	47.63	0.62	excluded	absent	37.6	0.26	excluded	absent	3.07
234577_at	excluded	absent	77.59	-0.08	excluded	absent	50.98	-0.7	excluded	absent	96.59
234578_at	included	present	640.48	0.2	included	present	202.67	-1.97	included	present	652.57
234579_at	excluded	absent	36.53	0.46	excluded	absent	2.22	-0.6	excluded	absent	2.5
234580_at	included	present	261.48	0.36	excluded	absent	123.29	-0.34	included	present	207.46
234581_at	excluded	absent	38.6	0.88	excluded	absent	27.25	0.34	excluded	absent	36.35

[illegible]

234907_x_at	excluded	absent	77.98	-0.32	excluded	absent	41.44	-1	excluded	absent	72.95	-0.41	excluded	absent	64.68	-0.57	excluded	absent	8.01
234908_s_at	excluded	absent	7.12	-0.89	excluded	absent	111.7	0.89	excluded	absent	87.73	0.48	excluded	absent	85.36	0.44	excluded	absent	69.55
234909_at	excluded	absent	6.78	-0.43	excluded	absent	6.65	-0.44	excluded	absent	11.57	-0.22	excluded	absent	2.78	-0.61	excluded	absent	13.97
234910_at	included	present	1069.67	1.51	included	present	792.07	0.5	included	present	409.22	-0.9	included	present	795.12	0.51	included	present	964.2
234911_at	excluded	absent	3.94	-0.89	excluded	absent	6.33	-0.82	excluded	absent	80.77	1.39	excluded	absent	3.37	-0.91	excluded	absent	17.92
234912_at	excluded	absent	63.7	2.18	excluded	absent	4.19	-0.64	excluded	absent	10.11	-0.36	excluded	absent	7.75	-0.47	excluded	absent	71.84
234913_at	excluded	absent	31.15	0.22	excluded	absent	8.16	-0.48	excluded	absent	8.27	-0.48	excluded	absent	2.76	-0.64	excluded	absent	8.78
234914_at	included	present	182.75	0.5	included	absent	58.39	-1.85	included	present	208.65	0.99	included	present	137.17	-0.36	excluded	absent	184.17
234915_s_at	included	present	387.65	0.34	included	present	464.08	1.05	included	present	525.52	1.63	included	present	663.59	2.92	included	present	546.48
234916_at	excluded	absent	8.2	-0.34	excluded	absent	9.48	-0.31	excluded	absent	79.93	1.58	excluded	absent	78.15	1.53	excluded	absent	144.72
234917_at	excluded	absent	101.25	1.42	excluded	absent	3.49	-0.75	excluded	absent	25.23	-0.27	excluded	absent	20.61	-0.37	excluded	absent	111.22
234918_at	excluded	absent	58.47	-0.18	excluded	absent	63.19	-0.07	excluded	absent	40.52	-0.57	excluded	absent	37.81	-0.63	excluded	absent	67.77
234919_s_at	excluded	absent	3.47	-0.87	excluded	absent	12.35	-0.82	excluded	absent	84.81	-0.41	excluded	absent	38.36	-0.67	excluded	absent	10.82
234920_at	included	present	337.57	1.05	included	present	253.78	0.08	included	present	407.09	1.86	included	present	462.71	2.51	included	present	487.51
234921_at	included	present	347.88	0.41	included	present	273.74	-0.15	included	present	265.27	-0.21	included	present	255.71	-0.28	included	present	269.19
234922_s_at	excluded	absent	64.89	-0.53	excluded	absent	9.14	-1.36	excluded	absent	16.4	-1.25	excluded	absent	9.1	-1.36	excluded	absent	12.45
234923_at	included	present	109.11	-0.75	included	present	136.47	-0.29	included	absent	101.93	-0.87	included	present	131.47	-0.37	included	present	225.39
234924_s_at	excluded	absent	67.83	-1.39	excluded	absent	37.51	-1.98	included	present	123.33	-0.31	included	present	103.84	-0.69	excluded	absent	163.25
234925_at	excluded	absent	40.23	-0.21	excluded	absent	4.11	-0.24	excluded	absent	14.31	-0.23	excluded	absent	13.36	-0.23	excluded	absent	15.15
234926_s_at	included	present	4051.47	1.4	included	present	4292.37	1.68	included	present	2610.16	-0.26	included	present	2105.85	-0.85	included	present	2809.78
234927_s_at	excluded	absent	3.44	-0.65	excluded	absent	4.28	-0.6	excluded	absent	44.7	1.85	excluded	absent	19.78	0.34	excluded	absent	4.25
234928_x_at	excluded	absent	15.5	-0.53	excluded	absent	31.13	-0.23	excluded	absent	16.35	-0.52	excluded	absent	18.43	-0.48	excluded	absent	15.66
234929_s_at	included	present	579.1	-0.58	included	present	823.4	-0.28	included	present	526.43	-0.64	included	present	697.61	-0.43	included	present	1223.99
234930_at	excluded	absent	26.08	-0.38	excluded	absent	25.38	-0.4	excluded	absent	4.91	-0.99	excluded	absent	76.89	1.06	excluded	absent	21.53
234931_at	excluded	absent	80.02	0.44	excluded	absent	15.93	-0.59	excluded	absent	7.56	-0.72	excluded	absent	55.78	0.05	excluded	absent	90.17
234932_s_at	included	present	196.55	0.34	excluded	absent	107.07	-0.19	excluded	absent	95.42	-0.26	included	present	627.14	2.89	excluded	absent	16.47
234933_at	included	present	406.37	0.44	included	present	134.58	-0.89	included	present	261.5	-0.27	included	absent	144.37	-0.84	included	present	345.13
234934_at	included	present	265.09	-0.36	included	present	194.94	-0.54	included	present	246.06	-0.41	included	present	805.48	1	included	present	555.65
234935_at	excluded	absent	107.16	0.04	excluded	absent	128.53	0.4	excluded	absent	168.44	1.06	excluded	absent	81.5	-0.39	excluded	absent	9.92
234936_s_at	included	present	586.85	0.14	included	present	394.57	-0.67	included	present	407.97	-0.61	included	present	314.27	-1.01	included	present	452.43
234937_x_at	included	present	811.44	-0.55	included	present	1010.21	-0.11	included	present	1022.78	-0.08	included	present	2124.79	2.38	included	present	674.56
234938_at	excluded	absent	147.56	0.56	excluded	absent	41.89	-0.52	excluded	absent	86.57	-0.06	excluded	absent	10.07	-0.85	excluded	absent	79.42
234939_s_at	included	present	1193.52	1.3	included	present	1361.99	1.93	included	present	883.73	0.13	included	present	1072.43	0.84	included	present	1922.3
234940_s_at	excluded	absent	2.9	-0.51	excluded	absent	41.66	-0.31	excluded	absent	106.13	0.01	excluded	absent	67.08	-0.19	excluded	absent	7.62

[illegible]

[illegible]

present	246.3	-0.23	included	present	895.74	1.59	included	present	176.01	-0.42	included	present	582.98	0.71	included
present	644.55	-0.69	included	present	3793.61	4.27	included	present	1995.7	1.44	included	present	2038.63	1.5	included
present	10344.57	38.27	included	absent	89.17	-1.06	included	present	217.2	-0.56	included	present	14437.09	53.96	included
absent	112.31	-0.35	included	absent	6.2	-0.82	included	absent	3.93	-0.83	included	absent	36.9	-0.69	included
present	1712.52	0.3	included	present	773.19	-0.22	included	present	881.33	-0.16	included	present	673.98	-0.28	included
present	211.94	-0.42	included	present	153.52	-0.53	included	absent	35.03	-0.74	included	present	189.35	-0.46	included
present	447.22	0.17	included	present	698.21	0.8	included	present	368.31	-0.04	included	present	178.79	-0.52	included
present	275.02	2.19	included	present	168.26	0.14	included	present	282.29	2.33	included	present	139.49	-0.42	included
present	12573.56	0.81	included	present	640.81	-1.12	included	present	3841.44	-0.6	included	present	1155.41	-1.04	included
present	525.3	0.64	included	present	537.22	0.68	included	present	223.99	-0.47	included	absent	79.24	-1.01	included
present	171.47	-0.27	included	absent	79.96	-1.72	included	present	247.09	0.92	included	present	112.9	-1.2	included
absent	3.05	-0.82	excluded	absent	30.82	0.29	excluded	absent	12.3	-0.45	excluded	absent	33.55	0.39	excluded
present	73.48	-0.04	included	present	135.02	0.72	included	present	140.78	0.79	included	present	126.82	0.62	excluded
present	198.42	-0.31	included	present	652.84	5.17	included	present	708.89	5.85	included	present	284.44	0.73	included
present	2371.21	4.71	included	present	1108.89	1.33	included	present	17418.12	45.03	included	present	306.61	-0.82	included
present	214.51	-0.94	included	present	332.41	-0.16	included	present	192.56	-1.08	included	present	412.36	0.36	included
present	106.43	-0.37	excluded	absent	95.04	-0.51	included	present	100.91	-0.44	excluded	absent	29.49	-1.29	excluded
present	458.84	-0.02	included	present	450.29	-0.06	included	present	932.58	2.22	included	present	1378.35	4.32	included
present	323.56	0.9	included	absent	118.71	-0.41	included	absent	39.07	-0.93	included	absent	105.49	-0.5	excluded
present	315.98	0.11	included	present	98.73	-0.67	included	present	108.2	-0.63	included	present	124.66	-0.57	included
present	1156.99	1.13	included	absent	16.34	-0.89	included	absent	111.76	-0.73	included	absent	10.41	-0.91	included
present	375.15	0.59	included	present	518.51	2.11	included	present	1284.53	10.22	included	present	525.87	2.18	included
present	425.09	-0.07	included	present	1520.21	3.91	included	present	1204.59	2.76	included	present	553.05	0.39	included
present	369.78	0.53	included	present	62.66	-1.22	included	present	385.65	0.62	included	absent	93.71	-1.04	included
absent	366.92	1.81	excluded	absent	119.96	-0.85	included	present	348.12	1.61	included	present	458.27	2.79	excluded
present	198.38	0.06	included	absent	5.97	-2.2	included	absent	105.55	-1.03	included	present	226.64	0.4	included
present	799.01	0.54	included	present	1089.81	1.1	included	present	412.21	-0.21	included	present	696.41	0.34	included
present	106.95	-0.68	included	present	80.71	-1.11	included	absent	81.19	-1.1	included	present	127.82	-0.35	included
present	494.75	0.13	included	present	425.93	-0.07	included	present	271.73	-0.53	included	present	427.28	-0.07	included
present	252.69	0.86	included	present	255.7	0.9	excluded	absent	188.78	0.06	included	present	293.01	1.37	included
present	267.37	-0.35	included	present	212.64	-0.93	included	present	919.06	6.55	included	present	297.5	-0.03	included
absent	69.41	-0.75	included	present	92.39	-0.38	included	present	132.48	0.26	included	present	284.03	2.7	excluded
present	236.28	-0.58	included	absent	113.02	-0.99	included	absent	39.15	-1.24	included	present	290.95	-0.4	included
present	1217.52	-0.12	included	present	649.37	-0.62	included	present	332.76	-0.9	included	present	123.97	-1.08	included
present	329.11	-0.49	included	present	335.36	-0.45	included	present	255.44	-0.95	included	present	624.41	1.37	included
present	872.83	2.61	included	absent	82.59	-1.7	included	present	172.98	-1.21	included	present	403.25	0.05	included
present	1231.64	-1	included	present	6032.93	3.34	included	present	5937.25	3.25	included	present	1274.18	-0.97	included
absent	25.27	-0.48	excluded	absent	6.26	-0.58	excluded	absent	34.75	-0.44	excluded	absent	81.59	-0.21	excluded
present	319.53	1.67	included	absent	98.4	-1.17	included	present	187.8	-0.02	included	present	84.46	-1.35	excluded
absent	2.35	-0.21	excluded	absent	2.56	-0.21	excluded	absent	82.86	-0.18	excluded	absent	4.12	-0.21	excluded
absent	8.89	-0.2	excluded	absent	13.8	-0.2	excluded	absent	45.32	-0.19	excluded	absent	10.37	-0.2	excluded
present	375.23	0.06	included	present	449	0.63	included	present	457.93	0.7	included	present	252.07	-0.88	included
absent	154.13	-0.15	included	present	209.8	0.26	excluded	absent	16.27	-1.16	included	present	144.79	-0.21	excluded
absent	234.82	-0.32	included	present	269.86	-0.25	excluded	absent	181.55	-0.44	excluded	absent	83.04	-0.66	excluded
present	368.01	0.35	included	present	274.52	-0.15	included	present	389.69	0.46	included	absent	191.93	-0.59	included
present	442.01	1.39	excluded	absent	31.71	-0.71	excluded	absent	40.71	-0.67	excluded	absent	69.79	-0.52	excluded
absent	82.3	0.37	excluded	absent	83.78	0.4	included	present	71.39	0.11	excluded	absent	4.12	-1.47	excluded
present	347.57	-0.22	included	present	608.82	0.77	included	present	211.5	-0.73	included	present	243.66	-0.61	included
present	1169.03	0.26	included	present	591.8	-0.9	included	present	1282.36	0.49	included	present	1659.68	1.26	included
present	502.43	1.04	included	present	370.66	0.21	included	present	522.74	1.16	included	present	775.27	2.75	included
absent	96.79	0.34	excluded	absent	162.88	1.55	excluded	absent	62.94	-0.27	excluded	absent	73.21	-0.09	excluded
present	265.88	0.66	included	present	152.52	-0.45	included	present	391.29	1.88	included	present	508.45	3.03	included
absent	84.68	-0.23	excluded	absent	234.04	2.18	excluded	absent	10.02	-1.44	excluded	absent	140.87	0.68	excluded
present	173.87	-1.12	included	absent	103.38	-1.97	included	present	340.42	0.9	included	present	416.48	1.82	included
present	3513.02	0.16	included	present	3846.35	0.43	included	present	4421.94	0.88	included	present	2828.75	-0.38	included
absent	167.36	0.67	excluded	absent	103.45	-0.25	excluded	absent	160.86	0.57	excluded	absent	219.99	1.42	excluded
absent	5.12	-1.12	excluded	absent	19.92	-0.88	excluded	absent	13.81	-0.98	excluded	absent	63.63	-0.18	excluded
present	1274.16	-0.43	included	present	1668.24	0.07	included	present	3567.78	2.47	included	present	3899.5	2.89	included
present	1852.71	-0.68	included	present	180.33	-2.5	included	present	503.48	-2.15	included	present	2792.79	0.35	included

[illegible]

235625_at	included	present	1363.48	2.77	included	present	1034.3	1.35	included	present	883.02	0.69	included	present	567.47	-0.67	[illegible]
235626_at	excluded	absent	306.76	-0.38	included	present	679.91	0.49	included	present	397.07	-0.17	included	present	1362.83	2.08	[illegible]
235627_at	included	present	205.02	-0.17	included	absent	172.04	-0.21	included	present	179.2	-0.2	included	present	128.16	-0.27	[illegible]
235628_x_at	included	present	328.92	1.08	included	absent	77.64	-1.8	included	present	162.46	-0.83	included	present	134.08	-1.15	[illegible]
235629_at	included	present	480.02	0.03	included	present	215.64	-0.46	included	present	414	-0.09	included	present	366.66	-0.18	[illegible]
235630_at	included	present	662.7	2.84	included	absent	115.48	-1.07	included	present	585.78	2.29	included	present	279.7	0.1	[illegible]
235631_at	included	present	3611.84	1.12	included	present	724.84	-0.58	included	present	3098.18	0.82	included	present	481.6	-0.72	[illegible]
235632_at	excluded	absent	160.35	1.62	excluded	absent	4.48	-1.49	excluded	absent	9.12	-1.39	excluded	absent	53.67	-0.5	[illegible]
235633_at	excluded	absent	79.57	-0.41	excluded	absent	81.95	-0.4	excluded	absent	71.48	-0.47	included	present	139.44	0.02	[illegible]
235634_at	included	present	494.28	-0.71	included	present	1390.93	0	included	present	1756.23	0.29	included	absent	48.23	-1.07	[illegible]
235635_at	included	present	436.79	-0.74	included	present	183.93	-1.24	included	present	219.67	-1.17	included	present	729.39	-0.17	[illegible]
235636_at	excluded	absent	74.52	-0.49	excluded	absent	45.34	-1.07	excluded	absent	81.54	-0.35	excluded	absent	96.22	-0.06	[illegible]
235637_s_at	excluded	absent	119.55	-0.06	excluded	absent	44.49	-1.32	excluded	absent	55.7	-1.13	included	present	137.45	0.24	[illegible]
235638_at	excluded	absent	1.23	-0.73	excluded	absent	5.03	-0.69	excluded	absent	5.2	-0.69	included	present	1560.92	15.5	[illegible]
235639_at	included	present	2275.52	15.39	excluded	absent	2.37	-0.72	excluded	absent	7.19	-0.68	excluded	absent	41.53	-0.44	[illegible]
235640_at	included	absent	389.79	-0.84	included	present	510.83	-0.48	included	present	531.36	-0.42	included	present	536.1	-0.41	[illegible]
235641_at	excluded	absent	29.63	-0.69	excluded	absent	4.97	-0.89	included	present	136.6	0.14	included	present	71.61	-0.37	[illegible]
235642_at	excluded	absent	21.89	-0.44	excluded	absent	120.52	-0.23	excluded	absent	5.54	-0.48	excluded	absent	5.29	-0.48	[illegible]
235643_at	included	present	1104.27	0.65	included	present	167.34	-0.72	included	absent	114.8	-0.79	included	absent	90.48	-0.83	[illegible]
235644_at	excluded	absent	13.73	-0.43	excluded	absent	100.51	0.2	included	present	186.09	0.82	excluded	absent	73.66	0	[illegible]
235645_at	included	present	1257.72	0.28	included	present	1544.68	0.8	included	present	1518.63	0.75	included	present	3379.93	4.14	[illegible]
235646_at	excluded	absent	147.97	-0.18	excluded	absent	94.42	-1.12	excluded	absent	73.76	-1.48	included	present	129.97	-0.5	[illegible]
235647_at	included	present	376.31	-0.06	included	present	261.45	-0.63	included	present	210.1	-0.89	included	present	312.48	-0.38	[illegible]
235648_at	excluded	absent	41.9	-1.21	excluded	absent	47.47	-1.11	included	present	136.39	0.47	included	present	288.49	3.18	[illegible]
235649_at	included	present	1285.32	3.4	included	absent	24.36	-1.17	included	absent	34.09	-1.13	included	absent	11.99	-1.21	[illegible]
235650_at	excluded	absent	62.9	-0.67	excluded	absent	92.8	-0.5	excluded	absent	103.69	-0.44	excluded	absent	118.64	-0.35	[illegible]
235651_at	included	present	182.53	-0.36	included	absent	112.43	-0.44	included	present	107.94	-0.45	included	present	226.72	-0.31	[illegible]
235652_at	included	present	848.99	0.1	included	absent	187.28	-1.12	included	present	610.07	-0.34	included	present	894.32	0.19	[illegible]
235653_s_at	included	present	128.86	0.59	excluded	absent	50.55	-0.73	included	present	130.86	0.62	included	present	161.29	1.13	[illegible]
235654_at	included	present	209.95	3.07	included	present	75.38	0.05	included	present	131.37	1.31	included	present	142.32	1.55	[illegible]
235655_at	included	present	169.19	0.73	included	present	96.1	-0.41	included	present	178.17	0.87	included	present	99.7	-0.36	[illegible]
235656_s_at	included	present	157.28	0.05	included	present	126.18	-0.33	included	present	163.49	0.12	included	present	147.54	-0.07	[illegible]
235657_at	included	present	120.45	0.35	included	present	690.11	6.97	excluded	absent	50.97	-0.46	included	present	237.09	1.7	[illegible]
235658_at	included	present	279.39	0.69	included	present	261.45	0.55	included	present	158.18	-0.25	included	present	616.12	3.29	[illegible]
[illegible]	[illegible]	[illegible]	[illegible]	[illegible]	[illegible]	[illegible]	[illegible]	[illegible]	[illegible]	[illegible]	[illegible]	[illegible]	[illegible]	[illegible]	[illegible]	[illegible]	[illegible]

235984_at	included	present	85.66	-0.55	included	present	109.23	-0.25	included	present	102.07	-0.34	included	present
235985_at	included	present	361.56	0.54	included	absent	63.92	-1.55	included	present	478.74	1.36	included	present
235986_at	excluded	absent	2.25	-0.78	excluded	absent	3.24	-0.76	excluded	absent	8.96	-0.66	excluded	absent
235987_at	included	present	386.51	0.25	included	present	539.63	0.89	included	present	288.11	-0.16	included	absent
235988_at	excluded	absent	30.21	0.04	excluded	absent	2.15	-0.6	excluded	absent	6.2	-0.5	excluded	absent
235989_at	excluded	absent	98.63	0.35	included	present	116.4	0.7	excluded	absent	69.99	-0.22	excluded	absent
235990_at	included	present	175.79	-0.08	excluded	absent	219.82	0.49	included	absent	72.71	-1.43	included	present
235991_at	excluded	absent	78.83	-0.49	included	present	171.44	1.23	excluded	absent	105.42	0	included	present
235992_s_at	excluded	absent	23.03	0.03	excluded	absent	123.05	3.48	excluded	absent	3.94	-0.63	excluded	absent
235993_at	included	present	291.42	1.73	included	present	204.94	0.03	included	present	301.83	1.94	included	present
235994_s_at	excluded	absent	64.35	1.04	excluded	absent	26.78	-0.02	excluded	absent	4.23	-0.66	excluded	absent
235995_at	included	absent	121.31	-1.12	included	absent	84.48	-1.45	included	present	175.95	-0.63	included	present
235996_at	included	present	462.34	-0.06	included	present	90.17	-0.47	included	present	151.37	-0.41	included	absent
235997_at	excluded	absent	95.76	0.75	excluded	absent	135.94	1.54	excluded	absent	130.8	1.44	excluded	absent
235998_at	excluded	absent	125.79	1.31	excluded	absent	30.58	-0.46	excluded	absent	8.31	-0.87	excluded	absent
235999_at	included	present	476.79	-0.57	included	present	300.9	-1.1	included	present	744.84	0.25	included	present
236000_s_at	included	present	1451.61	-0.58	included	present	1294.4	-0.73	included	present	3276.44	1.17	included	present
236001_at	excluded	absent	2.18	-0.45	excluded	absent	15.11	-0.38	excluded	absent	5.87	-0.43	excluded	absent
236002_at	included	present	290.87	0.06	included	absent	52.41	-2.08	included	absent	78.27	-1.85	included	absent
236003_x_at	excluded	absent	35.2	-0.02	included	present	127.62	1.63	included	present	144.11	1.93	excluded	absent
236004_at	included	absent	93.18	-1.1	included	present	247.03	0.17	included	absent	80.62	-1.21	included	present
236005_at	excluded	absent	87.02	1.74	excluded	absent	35.06	0.17	excluded	absent	73.18	1.32	excluded	absent
236006_s_at	included	present	202.91	-0.19	included	absent	89.83	-1.25	included	present	204.65	-0.18	included	present
236007_at	included	present	735.81	-0.44	included	present	882.64	-0.19	included	present	739.26	-0.43	included	present
236008_at	excluded	absent	13.03	-0.75	excluded	absent	6.28	-0.91	excluded	absent	1.65	-1.02	excluded	absent
236009_at	excluded	absent	5.1	-0.34	excluded	absent	2.2	-0.35	excluded	absent	43.23	-0.25	excluded	absent
236010_at	included	present	305.14	0.1	included	absent	89.02	-1.1	included	absent	77.48	-1.16	included	absent
236011_at	excluded	absent	86.43	-0.78	excluded	absent	253.69	0.69	excluded	absent	139.59	-0.31	excluded	absent
236012_at	included	present	208.81	-1.67	included	present	262.26	-1.13	included	present	369.41	-0.04	included	present
236013_at	included	present	246.22	-0.5	included	present	3371.53	1.44	excluded	absent	2.24	-0.65	excluded	absent
236014_at	excluded	absent	12.66	-0.37	excluded	absent	4.02	-0.6	excluded	absent	13.05	-0.36	excluded	absent
236015_at	excluded	absent	67.51	-0.83	excluded	absent	161.4	0.95	excluded	absent	125.53	0.27	excluded	absent
236016_at	included	present	177.78	-0.59	included	present	238.51	-0.04	included	present	150.94	-0.83	included	present
236017_at	included	present	514.47	0.09	included	present	1395.72	4.88	included	present	920.51	2.3	included	present

236343_at	included	present	310.08	-0.52	included	present	244.4	-0.78	included	present	177.34
236344_at	included	present	153.11	-0.35	excluded	absent	9.86	-0.85	excluded	absent	5.3
236345_at	excluded	absent	7.3	-0.89	excluded	absent	45.62	-0.55	excluded	absent	17.72
236346_at	included	present	588.16	1.62	included	present	426.94	0.62	included	present	1078.22
236347_at	included	present	244.17	-0.51	included	present	246.96	-0.51	included	present	236.94
236348_at	excluded	absent	135.43	1.33	excluded	absent	5.28	-1.05	included	present	122.15
236349_at	excluded	absent	44.4	0.04	excluded	absent	24.48	-0.23	excluded	absent	4.17
236350_at	included	present	1263.74	3.06	included	present	372.86	-0.28	included	present	1079.48
236351_at	excluded	absent	12.4	-0.73	excluded	absent	3.61	-0.75	excluded	absent	2.53
236352_at	excluded	absent	79.11	0.06	excluded	absent	73.58	0.03	excluded	absent	45.22
236353_at	included	present	121.46	-0.34	included	present	252.61	0.98	included	present	252.47
236354_at	included	present	235.59	1.97	excluded	absent	91.59	-0.41	included	present	182.98
236355_s_at	included	present	146.86	-0.27	included	absent	104.43	-0.76	included	present	87.22
236356_at	included	present	277.16	-0.07	included	present	110.66	-1.24	included	present	577.82
236357_at	excluded	absent	53.37	-0.85	excluded	absent	6.53	-1.82	excluded	absent	48.59
236358_at	included	present	1108.79	0.27	included	present	1162.17	0.38	included	present	1370.54
236359_at	included	present	804.55	-0.37	included	present	492.35	-0.43	included	absent	69.79
236360_at	included	present	383.41	1.16	excluded	absent	6.44	-0.82	excluded	absent	4.21
236361_at	excluded	absent	215.86	-0.25	excluded	absent	80.46	-0.62	excluded	absent	37.16
236362_at	excluded	absent	6.75	-1.51	excluded	absent	69.88	-0.35	excluded	absent	41.34
236363_at	included	present	216.36	0.79	included	absent	48.07	-2.35	included	absent	141.48
236364_at	excluded	absent	1.65	-1.09	excluded	absent	31.28	-0.43	excluded	absent	18.22
236365_at	excluded	absent	68.91	0.3	excluded	absent	4.47	-1.27	excluded	absent	44.61
236366_at	excluded	absent	107.42	1.33	excluded	absent	78.55	0.66	excluded	absent	15.2
236367_at	included	present	253.72	-0.41	included	present	211.08	-0.75	included	present	321.23
236368_at	included	present	603.76	-0.04	included	present	240.45	-1.34	included	present	727.62
236369_at	included	present	353.06	0.77	included	present	693.13	3.46	included	present	289.49
236370_at	excluded	absent	4.59	-2.24	excluded	absent	7.74	-2.18	excluded	absent	41.32
236371_s_at	included	present	1387.86	-0.28	included	present	1653.21	0.28	included	present	2308.49
236372_at	excluded	absent	105.39	0.81	excluded	absent	11.58	-1.31	included	present	130.78
236373_at	included	present	116.95	-0.18	included	present	110.08	-0.22	excluded	absent	37.53
236374_at	excluded	absent	13.22	-0.27	excluded	absent	2.46	-0.29	excluded	absent	39.67
236375_at	included	present	171	-1.87	included	absent	147.31	-1.94	included	present	303.75
236376_at	excluded	absent	44.81	-0.02	excluded	absent	1.93	-0.85	excluded	absent	8.21
[illegible]	[illegible]	[illegible]	[illegible]	[illegible]	[illegible]	[illegible]	[illegible]	[illegible]	[illegible]	[illegible]	[illegible]

236702_at	excluded	absent	30.23	-1.32	excluded	absent	128.64	-0.46	excluded	absent	14.13	-1.46	excluded	absent	108.32	-0.64
236703_at	included	absent	123.19	-0.58	included	present	247.65	-0.03	included	present	219.54	-0.16	included	present	188.42	-0.29
236704_at	included	present	317.83	-0.79	included	present	257.34	-0.87	included	present	257.61	-0.87	included	present	879.23	-0.07
236705_at	included	present	264.79	0.3	included	present	507.54	1.22	included	present	381.33	0.74	excluded	absent	31.29	-0.59
236706_at	excluded	absent	95.71	-0.34	excluded	absent	74.64	-0.57	excluded	absent	65.17	-0.67	excluded	absent	66.97	-0.65
236707_at	excluded	absent	82.29	-0.23	excluded	absent	6.13	-0.43	excluded	absent	1.57	-0.44	excluded	absent	46.67	-0.32
236708_at	included	present	147.79	0.84	included	present	157.54	1.01	excluded	absent	36.87	-1.09	excluded	absent	55.47	-0.76
236709_at	included	present	208.52	0.36	excluded	absent	14.5	-0.55	excluded	absent	14.91	-0.55	excluded	absent	4.38	-0.6
236710_at	excluded	absent	20.11	-0.48	excluded	absent	2.11	-0.6	excluded	absent	2.6	-0.6	excluded	absent	4.5	-0.58
236711_at	excluded	absent	97.41	-0.18	excluded	absent	70.4	-0.5	excluded	absent	73.19	-0.47	excluded	absent	59.34	-0.63
236712_at	included	present	158.96	3.2	excluded	absent	60.21	0.36	excluded	absent	2.41	-1.31	excluded	absent	66.24	0.53
236713_at	included	present	328.43	0.49	included	absent	194.58	-0.63	included	present	419.02	1.24	included	present	187.29	-0.69
236714_at	excluded	absent	5.91	-0.52	included	present	103.89	-0.22	excluded	absent	1.88	-0.53	excluded	absent	22.39	-0.47
236715_x_at	included	present	1306.05	1.28	included	present	737.24	-0.51	included	present	906.14	0.02	included	present	517.23	-1.2
236716_at	included	present	147.9	-0.41	excluded	absent	3.39	-0.71	excluded	absent	23.84	-0.67	excluded	absent	2.16	-0.71
236717_at	excluded	absent	100.44	-0.66	excluded	absent	43.78	-1.04	excluded	absent	10.5	-1.26	excluded	absent	2.97	-1.31
236718_at	excluded	absent	98.57	0.11	excluded	absent	74.61	-0.26	excluded	absent	69.87	-0.34	excluded	absent	65.81	-0.4
236719_at	included	present	719.26	2.08	included	present	203.2	-0.66	included	present	233.98	-0.5	included	present	284.23	-0.23
236720_at	excluded	absent	9.21	-1.05	excluded	absent	83.55	0.45	excluded	absent	55.13	-0.12	excluded	absent	137.23	1.54
236721_at	excluded	absent	85.79	0.23	included	present	116.92	1.05	included	present	97.93	0.55	included	present	138.27	1.61
236722_at	included	present	115.58	0.01	excluded	absent	92.83	-0.46	excluded	absent	119.84	0.1	excluded	absent	118.21	0.07
236723_at	included	present	936.96	-0.78	included	present	318	-2.03	included	present	3029.12	3.45	included	present	3112.66	3.62
236724_at	excluded	absent	29.09	-0.08	excluded	absent	6.15	-0.68	excluded	absent	94.72	1.64	excluded	absent	12.12	-0.53
236725_at	excluded	absent	29.73	-0.46	excluded	absent	16.06	-0.64	excluded	absent	7.64	-0.75	included	present	132.51	0.87
236726_at	excluded	absent	85.04	1.8	excluded	absent	6.96	-0.5	excluded	absent	2.31	-0.64	excluded	absent	5.3	-0.55
236727_at	excluded	absent	56.57	-0.11	excluded	absent	9.45	-0.61	excluded	absent	70.46	0.03	excluded	absent	70.87	0.04
236728_at	included	absent	42.33	-1.48	included	absent	5.43	-1.92	included	absent	150.69	-0.18	included	present	132.21	-0.4
236729_at	excluded	absent	42.16	-0.42	excluded	absent	2.56	-1.22	excluded	absent	76.48	0.27	included	present	62.8	-0.01
236730_at	included	present	208.38	1.43	excluded	absent	31.34	-0.58	excluded	absent	92.25	0.11	excluded	absent	59.86	-0.25
236731_at	excluded	absent	1.71	-1.07	excluded	absent	1.85	-1.06	excluded	absent	33.43	-0.4	excluded	absent	35.3	-0.37
236732_at	excluded	absent	188.06	1.14	excluded	absent	36.24	-1.14	excluded	absent	89.89	-0.33	excluded	absent	74.4	-0.56
236733_at	included	present	300.88	-1.02	included	present	420.8	-0.25	included	present	511.03	0.33	included	present	1002.9	3.48
236734_at	excluded	absent	41.6	-0.57	included	present	640.9	1.05	excluded	absent	3.1	-0.68	included	present	175.9	-0.21
236735_at	included	absent	179.53	-0.74	included	absent	173.6	-0.8	included	absent	161.59	-0.92	included	absent	103.5	-1.49

[illegible]

237061_at	included	present	1260.34	1.84	included	present	538.39	-0.68	included	present	1456.47	2.52	included	present	3746.31	10.48	included	present	681.5
237062_at	included	present	572.3	-0.63	included	present	236.98	-1.71	included	present	417.11	-1.13	included	present	626.95	-0.45	included	present	655.29
237063_at	excluded	absent	29.47	-0.38	excluded	absent	26.53	-0.45	excluded	absent	4.54	-0.93	included	present	64.84	0.4	excluded	absent	8.17
237064_x_at	excluded	absent	40.47	-0.44	included	present	103.7	0.39	excluded	absent	79.74	0.07	included	present	92.3	0.24	excluded	absent	126.23
237065_s_at	excluded	absent	59.32	-0.14	excluded	absent	37.56	-0.3	excluded	absent	4.66	-0.54	included	present	106.36	0.2	included	present	168.14
237066_at	excluded	absent	2.7	-0.53	excluded	absent	5.03	-0.4	excluded	absent	6.57	-0.31	excluded	absent	2.78	-0.53	excluded	absent	34.92
237067_at	excluded	absent	86.15	-0.53	excluded	absent	58.16	-1.09	excluded	absent	131.08	0.38	excluded	absent	52.26	-1.21	excluded	absent	158.22
237068_at	excluded	absent	8.2	-2.19	excluded	absent	49.07	-1.14	excluded	absent	63.67	-0.76	included	present	131.07	0.97	excluded	absent	5.21
237069_s_at	excluded	absent	60.95	0.59	excluded	absent	65.38	0.67	excluded	absent	4.68	-0.44	excluded	absent	7.8	-0.39	excluded	absent	95.53
237070_at	excluded	absent	54.29	-0.15	included	present	93.16	0.66	excluded	absent	60.77	-0.02	excluded	absent	2.46	-1.23	excluded	absent	105.8
237071_at	excluded	absent	42.3	-0.85	included	present	104.73	-0.19	excluded	absent	36.96	-0.91	excluded	absent	87.02	-0.38	included	present	207.26
237072_at	excluded	absent	107.49	0.92	excluded	absent	26.64	-0.57	included	present	101.65	0.81	included	present	134.55	1.41	included	present	186.53
237073_at	excluded	absent	56.48	0.31	excluded	absent	25.74	-0.58	excluded	absent	2.66	-1.25	excluded	absent	1.61	-1.28	excluded	absent	102.03
237074_at	included	present	231.55	0.1	included	present	122.91	-0.87	included	present	191.57	-0.26	included	present	127.93	-0.83	included	present	275.1
237075_at	excluded	absent	1.51	-0.72	included	present	91.97	-0.16	excluded	absent	7.52	-0.68	excluded	absent	33.09	-0.52	excluded	absent	58.76
237076_at	included	present	119.65	-0.67	included	absent	73.95	-0.98	included	present	223.32	0.02	included	present	198.56	-0.15	included	present	224.74
237077_at	included	present	88.59	-0.11	excluded	absent	4.11	-0.16	excluded	absent	3.91	-0.16	excluded	absent	7.08	-0.16	excluded	absent	45.52
237078_at	excluded	absent	81.59	0.89	excluded	absent	75.1	0.75	excluded	absent	117.64	1.65	excluded	absent	33.52	-0.13	excluded	absent	27.28
237079_at	excluded	absent	2.22	-0.51	excluded	absent	7.64	-0.41	excluded	absent	48.94	0.4	excluded	absent	6.42	-0.43	excluded	absent	2.1
237080_at	excluded	absent	112.27	0.24	excluded	absent	168.71	0.99	excluded	absent	38.99	-0.74	excluded	absent	7.17	-1.16	excluded	absent	11.49
237081_at	included	present	108.55	-0.69	excluded	absent	168.7	0.24	included	present	214	0.95	included	present	220.29	1.04	included	present	234.4
237082_at	included	present	126.48	0.44	excluded	absent	10.92	-2.04	included	present	266.38	3.45	excluded	absent	54.98	-1.09	excluded	absent	158.51
237083_at	excluded	absent	29.66	-1.08	included	present	184.07	1.42	included	present	107.56	0.18	included	present	112.96	0.27	included	present	269.08
237084_at	included	present	674.51	-0.35	included	present	430.96	-0.92	included	present	825.6	0	included	present	686.31	-0.32	included	absent	511.12
237085_x_at	included	present	254.05	0.52	included	present	670.34	3.25	included	present	227.85	0.35	included	present	300.68	0.83	included	present	365.27
237086_at	excluded	absent	45.77	0.08	excluded	absent	45.76	0.08	excluded	absent	1.19	-0.53	included	present	245.07	2.83	excluded	absent	7.68
237087_at	excluded	absent	13.19	-0.26	excluded	absent	76.7	-0.03	included	present	162.21	0.27	excluded	absent	4.56	-0.29	excluded	absent	36.71
237088_at	excluded	absent	51.33	-0.07	excluded	absent	47.49	-0.15	excluded	absent	136.42	1.67	excluded	absent	67.13	0.25	excluded	absent	191.45
237089_at	excluded	absent	1.73	-0.79	excluded	absent	63.18	0.82	excluded	absent	18.42	-0.35	excluded	absent	2.57	-0.77	excluded	absent	11.9
237090_at	excluded	absent	69.81	-0.07	excluded	absent	84.18	0	excluded	absent	48.98	-0.18	excluded	absent	78.61	-0.03	excluded	absent	87.69
237091_at	excluded	absent	150.27	0.7	excluded	absent	22.68	-0.86	excluded	absent	128.86	0.44	excluded	absent	4.65	-1.08	excluded	absent	12.64
237092_at	excluded	absent	9.54	-0.66	excluded	absent	8.71	-0.68	excluded	absent	10.34	-0.64	excluded	absent	35.99	-0.06	excluded	absent	11.1
237093_at	excluded	absent	69.61	5.59	excluded	absent	4.74	-0.54	excluded	absent	9.7	-0.07	excluded	absent	3.13	-0.69	excluded	absent	7.87
237094_at	included	present	545.7	2.96	excluded	absent	23.35	-0.69	excluded	absent	113.52	-0.06	excluded	absent	178.9	0.4	excluded	absent	4.36
237095_at	included	present	349.51	-0.96	included	present	328.79	-1.14	included	present	509.4	0.44	included	present	425.38	-0.3	included	present	642.23
237096_at	excluded	absent	68.16	0.29	excluded	absent	97.65	1.05	excluded	absent	51.34	-0.15	included	present	149.14	2.39	excluded	absent	134.21
237097_at	included	present	155.33	1.08	excluded	absent	159.97	1.15	excluded	absent	3.52	-1.15	excluded	absent	10.19	-1.05	excluded	absent	93.55
237098_at	included	present	113.53	-0.34	excluded	absent	50.82	-1.08	excluded	absent	93.08	-0.58	excluded	absent	67.48	-0.88	included	present	202.83
237099_at	excluded	absent	64.21	-0.15	excluded	absent	51.48	-0.15	excluded	absent	39.54	-0.15	excluded	absent	72.2	-0.15	excluded	absent	5.5
237100_at	excluded	absent	81.35	0.8	excluded	absent	46.76	0.04	excluded	absent	48.52	0.08	excluded	absent	2.61	-0.93	excluded	absent	145.66
237101_at	excluded	absent	2.24	-0.68	excluded	absent	3.51	-0.64	excluded	absent	5.4	-0.58	excluded	absent	23.76	-0.03	excluded	absent	61.45
237102_at	excluded	absent	9.26	-0.57	excluded	absent	76.6	0.51	excluded	absent	5.13	-0.63	excluded	absent	2.6	-0.67	excluded	absent	90.46
237103_at	excluded	absent	10.35	-0.79	excluded	absent	68.13	0.92	excluded	absent	35.8	-0.04	excluded	absent	17.57	-0.58	excluded	absent	14.35
237104_at	included	present	243.91	-0.3	included	present	259.38	-0.27	included	present	213.31	-0.36	included	absent	110.42	-0.57	included	present	136.85
237105_at	included	present	552.57	2.39	included	present	356.38	0.91	included	present	779.43	4.09	included	present	1145.19	6.84	included	present	495.37
237106_at	included	present	339.35	-0.79	included	present	241.87	-1.25	included	present	404.73	-0.49	included	present	461.25	-0.22	included	present	429.05
237107_at	included	present	162.55	-0.14	included	present	128.23	-0.74	included	present	384.53	3.7	included	present	376.39	3.56	included	present	165.35
237108_x_at	excluded	absent	370.94	1.96	excluded	absent	183.66	0.26	excluded	absent	3.36	-1.37	excluded	absent	19.19	-1.22	excluded	absent	6.62

237420_at	excluded	absent	68.16	-0.39	excluded	absent	18.65	-1.37	excluded	absent	43.51	-0.88	excluded	absent	47.12	-0.81	included	present	151.65	1.28	excluded	absent	89.69	0.04	excluded	absent	83.35	-0.08	excluded	absent	11.43	-1.52	excluded	absent	45.77	-0.83	excluded	absent	82.68	-0.1	included	present	182.25	1.88	excluded	absent	94.76	0.14	excluded	absent	78.95	-0.17	excluded	absent	57.3	-0.6	included	present	61.39	-0.52
237421_at	included	present	109.67	0.95	excluded	absent	14.24	-0.52	excluded	absent	16.81	-0.48	excluded	absent	5.5	-0.66	excluded	absent	155.19	1.66	excluded	absent	92.87	0.69	excluded	absent	10.26	-0.58	excluded	absent	5.66	-0.65	excluded	absent	9.96	-0.59	excluded	absent	43.14	-0.07	excluded	absent	172.71	1.93	excluded	absent	60.25	0.19	excluded	absent	66.4	0.29	included	present	106.75	0.91	excluded	absent	47.09	-0.01
237422_at	included	present	88.12	-1.05	included	present	116	-0.98	included	present	493.3	-0.08	included	present	261.23	-0.63	included	present	391.16	-0.32	included	absent	70.48	-1.09	included	present	367.79	-0.38	included	present	102.05	-1.01	included	present	218.9	-0.73	included	present	159.85	-0.87	included	present	331.19	-0.46	included	present	230.36	-0.71	included	present	602.75	0.18	included	present	366.89	-0.38	included	present	60.58	-1.11
237423_at	excluded	absent	38.51	-0.09	included	present	398.51	8.82	excluded	absent	31.79	-0.25	excluded	absent	8.86	-0.82	excluded	absent	45.91	0.1	excluded	absent	75.92	0.84	excluded	absent	75.71	0.83	included	present	237.79	4.85	excluded	absent	43.85	0.05	excluded	absent	5.51	-0.9	included	present	160.33	2.93	excluded	absent	147.31	2.61	included	present	131.95	2.23	excluded	absent	58.14	0.4	excluded	absent	74.26	0.8
237424_at	excluded	absent	70.25	0.28	excluded	absent	67.6	0.23	excluded	absent	8	-0.89	excluded	absent	7.79	-0.89	excluded	absent	69.33	0.26	excluded	absent	70.12	0.28	excluded	absent	34.17	-0.4	excluded	absent	49.32	-0.11	excluded	absent	54.21	-0.02	excluded	absent	16.81	-0.72	excluded	absent	3.58	-0.97	excluded	absent	7.22	-0.9	excluded	absent	46.85	-0.16	excluded	absent	3.01	-0.98	excluded	absent	5.33	-0.94
237425_at	excluded	absent	0.84	-0.42	excluded	absent	80.7	-0.07	excluded	absent	2.53	-0.41	excluded	absent	21.88	-0.32	excluded	absent	20.91	-0.33	excluded	absent	3.34	-0.4	excluded	absent	30.96	-0.28	excluded	absent	12.66	-0.36	excluded	absent	5.18	-0.4	excluded	absent	9.19	-0.38	excluded	absent	3.43	-0.4	excluded	absent	7.88	-0.38	excluded	absent	25.76	-0.31	excluded	absent	34.61	-0.27	excluded	absent	11.34	-0.37
237426_at	excluded	absent	70.2	-0.39	excluded	absent	77.4	-0.32	excluded	absent	2.72	-1.07	excluded	absent	24.79	-0.85	excluded	absent	4.76	-1.05	included	present	233.98	1.26	excluded	absent	28.37	-0.81	excluded	absent	22.07	-0.88	included	present	123.73	0.15	excluded	absent	16.16	-0.93	excluded	absent	33.67	-0.76	included	present	104.76	-0.04	excluded	absent	54.48	-0.55	excluded	absent	58.81	-0.5	excluded	absent	47.68	-0.62
237427_at	excluded	absent	44.77	0.24	excluded	absent	9.15	-0.71	excluded	absent	54.63	0.51	excluded	absent	2.36	-0.89	excluded	absent	15.57	-0.54	excluded	absent	3.59	-0.86	excluded	absent	5.12	-0.82	excluded	absent	25.56	-0.27	excluded	absent	7.83	-0.74	excluded	absent	4.23	-0.84	excluded	absent	10.38	-0.68	excluded	absent	8.52	-0.73	excluded	absent	79.98	1.18	excluded	absent	20.07	-0.42	excluded	absent	45.77	0.27
237428_at	excluded	absent	48.91	0.17	excluded	absent	1.56	-0.42	excluded	absent	43.56	0.1	excluded	absent	4.77	-0.38	excluded	absent	13.72	-0.27	excluded	absent	1.33	-0.42	excluded	absent	25.45	-0.12	excluded	absent	1.34	-0.42	excluded	absent	2.65	-0.4	excluded	absent	27.35	-0.1	excluded	absent	1.76	-0.41	excluded	absent	4.47	-0.38	excluded	absent	71.99	0.45	excluded	absent	33.46	-0.02	excluded	absent	5.21	-0.37
237429_at	excluded	absent	110.17	0.06	excluded	absent	126.91	0.28	excluded	absent	22.33	-1.1	excluded	absent	41.4	-0.85	excluded	absent	5.08	-1.33	excluded	absent	112.39	0.09	excluded	absent	69.44	-0.48	excluded	absent	90.49	-0.2	excluded	absent	125.25	0.26	excluded	absent	54.06	-0.68	excluded	absent	68.26	-0.49	excluded	absent	4.92	-1.33	excluded	absent	160.85	0.72	included	present	302.04	2.58	excluded	absent	42.66	-0.83
237430_at	excluded	absent	3.8	-0.76	excluded	absent	5.47	-0.69	excluded	absent	7.68	-0.59	excluded	absent	75.6	2.33	excluded	absent	6.05	-0.66	excluded	absent	13.99	-0.32	excluded	absent	5.13	-0.7	excluded	absent	4.5	-0.73	excluded	absent	7.52	-0.6	excluded	absent	3.58	-0.77	excluded	absent	5.25	-0.7	excluded	absent	7.93	-0.58	excluded	absent	5.14	-0.7	excluded	absent	2.38	-0.82	excluded	absent	3.39	-0.78
237431_at	excluded	absent	75.87	0.83	excluded	absent	53.14	0.36	excluded	absent	80.03	0.92	excluded	absent	1.61	-0.71	excluded	absent	2.62	-0.69	excluded	absent	102.03	1.37	excluded	absent	9.5	-0.55	excluded	absent	2.69	-0.69	excluded	absent	27.7	-0.17	excluded	absent	28.53	-0.15	excluded	absent	18.05	-0.37	excluded	absent	47.52	0.24	excluded	absent	32.6	-0.07	excluded	absent	70.15	0.71	excluded	absent	17.73	-0.38
237432_at	excluded	absent	113.55	-0.17	excluded	absent	59.1	-0.96	included	present	157.72	0.47	excluded	absent	133.68	0.12	excluded	absent	223.55	1.42	excluded	absent	111.47	-0.2	excluded	absent	66.08	-0.86	excluded	absent	44.31	-1.17	included	present	145.42	0.29	excluded	absent	88.62	-0.53	excluded	absent	31.02	-1.36	excluded	absent	139.16	0.2	excluded	absent	62.49	-0.91	excluded	absent	46.34	-1.14	excluded	absent	50.29	-1.08
237433_at	excluded	absent	23.92	-0.25	included	present	116.5	2.45	excluded	absent	47.59	0.44	excluded	absent	19.91	-0.37	excluded	absent	68.51	1.05	excluded	absent	39.09	0.19	included	present	101.7	2.02	excluded	absent	34.55	0.06	included	present	61.75	0.85	excluded	absent	33.51	0.03	excluded	absent	48.43	0.46	excluded	absent	2.42	-0.87	excluded	absent	57.16	0.72	excluded	absent	21.79	-0.31	excluded	absent	36.02	0.1
237434_x_at	excluded	absent	79.98	0.31	excluded	absent	2.97	-1.3	excluded	absent	5.96	-1.24	excluded	absent	48.65	-0.34	excluded	absent	4.22	-1.28	excluded	absent	94.2	0.61	excluded	absent	97.67	0.69	excluded	absent	64.19	-0.02	excluded	absent	71.11	0.13	excluded	absent	30.49	-0.73	excluded	absent	155.24	1.9	excluded	absent	121.96	1.2	excluded	absent	89.64	0.52	excluded	absent	45.06	-0.42	excluded	absent	158.97	1.97
237435_at	excluded	absent	1.65	-0.8	excluded	absent	3.14	-0.73	excluded	absent	27.48	0.42	excluded	absent	0.99	-0.83	excluded	absent	11.37	-0.34	excluded	absent	66.98	2.29	excluded	absent	35.99	0.82	excluded	absent	32.85	0.68	excluded	absent	5.29	-0.63	excluded	absent	3.84	-0.7	excluded	absent	4.4	-0.67	excluded	absent	1.3	-0.82	excluded	absent	79.48	2.88	excluded	absent	3.67	-0.7	excluded	absent	25.63	0.33
237436_at	excluded	absent	41.3	-0.05	excluded	absent	9.47	-0.33	excluded	absent	41.56	-0.05	excluded	absent	66.28	0.16	excluded	absent	8.47	-0.34	excluded	absent	9.59	-0.33	excluded	absent	18.5	-0.25	excluded	absent	6.44	-0.36	excluded	absent	16.82	-0.27	excluded	absent	9.13	-0.33	excluded	absent	1.79	-0.4	excluded	absent	30.42	-0.15	excluded	absent	3.55	-0.38	excluded	absent	10.48	-0.32	excluded	absent	17.37	-0.26
237437_s_at	excluded	absent	5.4	-0.86	excluded	absent	11.87	-0.78	excluded	absent	77.4	-0.01	excluded	absent	104.88	0.32	excluded	absent	116.63	0.46	excluded	absent	48.16	-0.35	excluded	absent	34.73	-0.51	excluded	absent	12.62	-0.77	excluded	absent	38.31	-0.47	excluded	absent	12.2	-0.78	excluded	absent	66.03	-0.14	excluded	absent	5	-0.86	excluded	absent	12.15	-0.78	excluded	absent	66.89	-0.13	excluded	absent	43.44	-0.41
237438_at	excluded	absent	111.73	1.25	excluded	absent	68.58	0.26	excluded	absent	59.37	0.05	excluded	absent	75.02	0.41	excluded	absent	3.27	-1.24	excluded	absent	5.08	-1.2	included	present	90.53	0.77	excluded	absent	37.18	-0.46	excluded	absent	83.05	0.59	excluded	absent	94.53	0.86	excluded	absent	56.43	-0.02	excluded	absent	125.57	1.57	excluded	absent	56.6	-0.01	excluded	absent	26.15	-0.71	excluded	absent	37.97	-0.44
237439_at	included	absent	29.22	-1.04	included	absent	3.91	-1.11	included	absent	95.14	-0.84	included	present	194.45	-0.53	included	absent	82.63	-0.87	included	absent	43.85	-0.99	included	absent	25.79	-1.05	included	absent	29.44	-1.04	included	absent	34.44	-1.02	included	absent	6.41	-1.11	included	absent	20.83	-1.06	included	absent	24.5	-1.05	included	present	77.23	-0.89	included	absent	23.35	-1.05	included	absent	2.53	-1.12
237440_at	included	present	261.18	0.57	included	absent	91.2	-1.12	included	present	121.41	-0.82	included	absent	72.32	-1.31	included	absent	18.91	-1.84	included	absent	79.99	-1.24	included	present	114.01	-0.9	included	absent	29.26	-1.74	included	present	170.8	-0.33	included	absent	58.03	-1.45	included	present	141.24	-0.63	included	absent	38.64	-1.65	included	absent	165.77	-0.38	included	present	110.05	-0.94	included	present	101.13	-1.03
237441_at	included	present	368.22	1.19	included	absent	58.09	-1.34	included	present	313.84	0.75	included	present	542.45	2.61	included	absent	8.82	-1.74	included	present	180.64	-0.34	included	present	193.33	-0.24	included	present	328.61	0.87	included	present	464.09	1.97	included	present	48.53	-1.42	included	present	314.56	0.75	included	present	96.91	-1.03	included	absent	233.14	0.09	included	absent	40.33	-1.49	included	present	112.32	-0.9
237442_at	included	present	161.25	0.43	excluded	absent	26.27	-0.65	excluded	absent	2.79	-0.84	excluded	absent	3.98	-0.83	included	present	173.33	0.53	included	present	111.25	0.03	excluded	absent	13.2	-0.76	excluded	absent	1.18	-0.86	included	present	100.15	-0.06	excluded	absent	35.32	-0.58	included	present	89.11	-0.15	excluded	absent	71.63	-0.29	excluded	absent	40.47	-0.54	excluded	absent	1.75	-0.85	excluded	absent	1.52	-0.85
237443_at	included	present	77.32	1.13	excluded	absent	4.36	-1.09	excluded	absent	7.2	-1	excluded	absent	7.2	-1	excluded	absent	68.49	0.86	excluded	absent	4.88	-1.07	excluded	absent	36.56	-0.11	excluded	absent	7.31	-1	excluded	absent	50.21	0.31	excluded	absent	52.53	0.38	excluded	absent	2.35	-1.15	excluded	absent	4.27	-1.09	excluded	absent	36.62	-0.11	excluded	absent	30.57	-0.29	excluded	absent	24.94	-0.46
237444_at	included	present	7312.82	-0.13	included	present	8624.67	0.21	included	present	3453.6	-1.11	included	present	2252.33	-1.42	included	present	5813.81	-0.51	included	present	4813.92	-0.77	included	present	2911.34	-1.25	included	present	3330.67	-1.15	included	present	3728.71	-1.04	included	present	3599.45	-1.08	included	present	1190.12	-1.69	included	present	2756.53	-1.29	included	present	1652.76	-1.58	included	present	3276.73	-1.16	included	present	1515.57	-1.61
237445_at	excluded	absent	3.31	-0.76	excluded	absent	6.39	-0.65	excluded	absent	10.29	-0.51	excluded	absent	6.44	-0.65	excluded	absent	41.83	0.64	excluded	absent	14.74	-0.35	excluded	absent	24.84	0.02	excluded	absent	42.28	0.65	excluded	absent	11.23	-0.48	excluded	absent	10.19	-0.51	excluded	absent	6.65	-0.64	excluded	absent	5.27	-0.69	excluded	absent	49.53	0.92	excluded	absent	5.56	-0.68	excluded	absent	9.62	-0.54
237446_at	excluded	absent	2.22	-0.63	excluded	absent	2.18	-0.63	excluded	absent	61.85	0.29	excluded	absent	3.73	-0.6	excluded	absent	46.63	0.05	excluded	absent	2.23	-0.63	included	present	69.64	0.41	excluded	absent	75.64	0.5	excluded	absent	34.2	-0.14	excluded	absent	14.26	-0.44	included	present	136.23	1.43	excluded	absent	79.89	0.56	excluded	absent	44.75	0.02	excluded	absent	28.36	-0.23	excluded	absent	0.92	-0.65
237447_at	excluded	absent	81.69	0.15	excluded	absent	80.62	0.13	excluded	absent	18.07	-1.03	excluded	absent	37.66	-0.67	excluded	absent	148.47	1.4	excluded	absent	92.56	0.36	excluded	absent	5.51	-1.26	excluded	absent	80.01	0.12	excluded	absent	152.39	1.47	included	present	140.43	1.25	excluded	absent	86.18	0.24	excluded	absent	16.97	-1.05	excluded	absent	27.51	-0.86	excluded	absent	46.43	-0.5	excluded	absent	25.1	-0.9
237448_at	included	present	126.04	0.21	included	present	200.09	1.33	included	present	207.8	1.45	included	present	631.18	7.83	included	present	211.12	1.5	excluded	absent	83.4	-0.43	included	present	316.55	3.09	excluded	absent	63.11	-0.73	included	present	138.31	0.4	included	present	84.13	-0.42	excluded	absent	101.81	-0.15	included	present	104.38	-0.11	included	present	399.34	4.34	included	present	316.07	3.08	included	present	141.15	0.44
237449_at	excluded	absent	2.4	-0.39	excluded	absent	30.52	-0.25	excluded	absent	18.07	-0.31	excluded	absent	9.67	-0.35	excluded	absent	46.35	-0.18	excluded	absent	5.47	-0.38	excluded	absent	9.54	-0.36	excluded	absent	9.25	-0.36	excluded	absent	18.35	-0.31	excluded	absent	1.02	-0.4	excluded	absent	20.18	-0.3	excluded	absent	54.5	-0.14	excluded	absent	9.54	-0.36	excluded	absent	2.81	-0.39	excluded	absent	1.06	-0.4
237450_at	excluded	absent	2.56	-0.46	excluded	absent	7.15	-0.43	excluded	absent	2.84	-0.46	excluded	absent	41.9	-0.17	excluded	absent	11.63	-0.4	excluded	absent	14.1	-0.38	excluded	absent	72.83	0.06	excluded	absent	25.35	-0.29	excluded	absent	2.48	-0.46	excluded	absent	7.73	-0.42	excluded	absent	3.74	-0.45	excluded	absent	86.55	0.16	excluded	absent	4	-0.45	excluded	absent	12.45	-0.39	excluded	absent	50.03	-0.11
237451_x_at	included	present	384.54	-0.55	included	present	456.48	-0.18	included	present	371.11	-0.62	included	present	128.54	-1.86	included	present	1243.29	3.87	included	present	1082.07	3.04	included	present	856.91	1.88	included	present	717.16	1.16	included	present	711.52	1.14	included	present	446.11	-0.23	included	present	403.37	-0.45	included	present	612.32	0.63	included	present	879.09	2	included	present	1280.62	4.06	included	present	299.4	-0.98
237452_at	excluded	absent	73.65	-0.39	excluded	absent	6.05	-0.99	excluded	absent	101.66	-0.15	excluded	absent	81.07	-0.33	excluded	absent	123.22	0.04	included	present	229.3	0.98	excluded	absent	100.43	-0.16	included	present	68.84	-0.43	included	present	77.86	-0.35	included	present	69.04	-0.43	excluded	absent	66.85	-0.45	included	present	184.84	0.59	excluded	absent	34.64	-0.73	excluded	absent	88.67	-0.26	included	present	34.27	-0.74
237453_at	excluded	absent	19.07	-0.79	excluded	absent	90.42	-0.27	excluded	absent	123.59	-0.03	excluded	absent	98.93	-0.21	excluded	absent	33.14	-0.68	excluded	absent	17.44	-0.8	excluded	absent	60.01	-0.49	excluded	absent	99.87	-0.2	excluded	absent	123.77	-0.03	excluded	absent	65.25	-0.45	included	present	170.02	0.3	excluded	absent	148.16	0.15	excluded	absent	33.81	-0.68	excluded	absent	78.92	-0.35	excluded	absent	55.69	-0.52
[illegible]	[illegible]	[illegible]	[illegible]	[illegible]	[illegible]	[illegible]	[illegible]	[illegible]	[illegible]	[illegible]	[illegible]	[illegible]	[illegible]	[illegible]	[illegible]	[illegible]	[illegible]	[illegible]	[illegible]	[illegible]	[illegible]	[illegible]	[illegible]	[illegible]	[illegible]	[illegible]	[illegible]	[illegible]	[illegible]	[illegible]	[illegible]	[illegible]	[illegible]	[illegible]	[illegible]	[illegible]	[illegible]	[illegible]	[illegible]	[illegible]	[illegible]	[illegible]	[illegible]	[illegible]	[illegible]	[illegible]	[illegible]	[illegible]	[illegible]	[illegible]	[illegible]	[illegible]	[illegible]	[illegible]	[illegible]	[illegible]	[illegible]	[illegible]	[illegible]	[illegible]

237779_at	excluded	absent	75.64	-0.27	excluded	absent	127.22	0.1	excluded	absent	74.27	-0.28	excluded	absent	73.1	-0.29	excluded	absent	148.41
237780_at	excluded	absent	2.2	-0.61	excluded	absent	53.9	1.37	excluded	absent	6.02	-0.46	excluded	absent	13.04	-0.2	excluded	absent	93.69
237781_at	excluded	absent	7.51	-0.27	excluded	absent	33.55	-0.2	excluded	absent	2.62	-0.29	excluded	absent	1.92	-0.29	excluded	absent	2.52
237782_at	excluded	absent	89.79	0.01	excluded	absent	12.07	-1.23	excluded	absent	7.73	-1.3	excluded	absent	105.75	0.26	excluded	absent	99.98
237783_at	included	present	248.98	-0.31	included	present	247.3	-0.31	included	present	451.14	-0.09	included	present	251.97	-0.3	included	absent	434.01
237784_at	included	present	165.35	-0.14	included	absent	55.17	-1.29	included	present	223.04	0.47	included	present	268.06	0.94	included	present	188.08
237785_at	included	present	119.67	-1.07	included	present	230.75	0.1	included	present	255.42	0.36	included	present	170.65	-0.53	included	present	198.38
237786_at	excluded	absent	27.47	-0.86	excluded	absent	184.42	0.28	excluded	absent	39.02	-0.78	excluded	absent	1.96	-1.05	included	present	375.04
237787_at	excluded	absent	80.29	0.87	excluded	absent	11.31	-0.43	excluded	absent	36.86	0.06	excluded	absent	52.65	0.35	excluded	absent	49.6
237788_at	excluded	absent	43.15	-0.34	excluded	absent	30.14	-0.62	excluded	absent	41.41	-0.38	excluded	absent	52.59	-0.14	excluded	absent	39.84
237789_at	excluded	absent	79.58	-0.38	excluded	absent	16.1	-1	excluded	absent	12.23	-1.04	excluded	absent	88.92	-0.29	excluded	absent	30.32
237790_at	included	present	109.83	-0.4	included	present	128.43	-0.33	included	present	224.28	0.03	included	present	233.48	0.07	included	present	293.57
237791_at	excluded	absent	3.96	-0.84	excluded	absent	5.79	-0.81	excluded	absent	32.29	-0.34	excluded	absent	14.19	-0.66	excluded	absent	4.4
237792_at	excluded	absent	9.36	-0.93	excluded	absent	7.71	-0.97	excluded	absent	6.3	-1.01	excluded	absent	7.62	-0.97	excluded	absent	55.32
237793_at	excluded	absent	6.77	-0.62	excluded	absent	7.38	-0.61	excluded	absent	37.85	-0.21	excluded	absent	27.44	-0.35	excluded	absent	3.03
237794_at	excluded	absent	82.99	-0.51	included	present	370.84	0.09	excluded	absent	161.53	-0.35	excluded	absent	21.12	-0.64	included	present	509.69
237795_s_at	included	present	260.18	1.2	included	present	145.73	-0.14	included	present	546.3	4.54	included	present	156.56	-0.01	included	present	318.76
237796_at	excluded	absent	48.2	0.89	excluded	absent	10.31	-0.47	excluded	absent	14.54	-0.32	excluded	absent	85.11	2.22	excluded	absent	140.79
237797_at	excluded	absent	92.25	0.49	excluded	absent	32.21	-1.06	included	present	170.21	2.5	included	present	114.58	1.07	included	present	153.69
237798_at	included	present	156.15	-1.03	included	present	163.97	-1	included	present	239.59	-0.8	included	present	395.38	-0.38	included	present	230.78
237799_at	excluded	absent	15.41	-0.18	excluded	absent	11.17	-0.19	excluded	absent	37.62	-0.1	excluded	absent	9.64	-0.2	excluded	absent	3.02
237800_at	excluded	absent	157.05	0.32	excluded	absent	12.85	-0.4	excluded	absent	7.8	-0.42	excluded	absent	15.66	-0.38	excluded	absent	5.63
237801_at	excluded	absent	32.22	0.09	excluded	absent	45	0.47	excluded	absent	23.7	-0.17	excluded	absent	81.94	1.57	excluded	absent	55.47
237802_at	included	present	5452.27	19.52	included	present	126.31	-0.38	excluded	absent	75.3	-0.57	included	present	564.2	1.26	excluded	absent	6.22
237803_x_at	included	present	474.31	0.22	included	absent	54.05	-1.48	included	present	181.22	-0.96	included	present	515.95	0.39	included	present	215.25
237804_at	excluded	absent	10.58	-0.34	excluded	absent	67.16	-0.05	excluded	absent	3.87	-0.38	excluded	absent	28.04	-0.25	excluded	absent	11.42
237805_at	excluded	absent	40.49	0.02	excluded	absent	31.87	-0.13	excluded	absent	34.62	-0.08	included	present	214.55	3	excluded	absent	32.4
237806_s_at	excluded	absent	50.52	-0.16	excluded	absent	2.55	-0.31	excluded	absent	37.92	-0.2	included	present	71.48	-0.1	excluded	absent	14.45
237807_at	excluded	absent	48.96	-0.23	excluded	absent	9.54	-0.36	excluded	absent	13.16	-0.35	excluded	absent	32.72	-0.29	excluded	absent	130.83
237808_at	included	absent	33.97	-1.66	included	absent	68.75	-1.24	included	absent	72.24	-1.2	included	present	120	-0.63	included	present	602.49
237809_at	excluded	absent	1.71	-0.69	excluded	absent	2.3	-0.66	excluded	absent	53.04	1.7	excluded	absent	4.64	-0.55	excluded	absent	6.68
237810_at	included	present	70.37	-0.5	excluded	absent	6.94	-1.54	included	present	105.79	0.08	included	present	121.2	0.34	included	present	199.51
237811_at	excluded	absent	11.05	-0.29	excluded	absent	22.71	0.18	excluded	absent	9.54	-0.35	excluded	absent	1.81	-0.67	excluded	absent	21.35
237812_at	excluded	absent	27.91	-0.16	excluded	absent	11.99	-0.33	excluded	absent	11.47	-0.34	excluded	absent	12.95	-0.32	excluded	absent	70.35
237813_at	excluded	absent	50.19	-0.28	excluded	absent	43.68	-0.43	excluded	absent	75.67	0.3	included	present	98.34	0.82	excluded	absent	114.4
237814_at	included	absent	69.03	-0.64	included	absent	1.34	-0.84	included	absent	94.71	-0.57	included	absent	5.9	-0.83	included	absent	141.44
237815_at	excluded	absent	2.73	-0.55	excluded	absent	10.91	-0.22	excluded	absent	2.01	-0.58	excluded	absent	3.55	-0.52	excluded	absent	35.76
237816_at	excluded	absent	71.68	0.54	excluded	absent	142.99	2.27	excluded	absent	44.58	-0.12	excluded	absent	50.95	0.04	excluded	absent	125.2
237817_at	included	present	87.28	-0.73	included	absent	73.1	-0.91	included	absent	27.1	-1.48	included	present	68.05	-0.97	included	absent	20.7
237818_at	excluded	absent	80.88	0.94	excluded	absent	33.95	-0.19	excluded	absent	31.25	-0.25	excluded	absent	56.4	0.35	excluded	absent	5.11
237819_at	included	present	77.55	-0.17	excluded	absent	48.91	-0.68	excluded	absent	6.39	-1.43	excluded	absent	85.59	-0.03	excluded	absent	16.59
237820_at	excluded	absent	14.26	-0.92	excluded	absent	3.75	-1.1	excluded	absent	98.87	0.48	excluded	absent	98.5	0.47	excluded	absent	6.24
237821_at	excluded	absent	7.5	-1.08	excluded	absent	9.3	-1.04	excluded	absent	2.77	-1.18	excluded	absent	56.5	0.04	excluded	absent	10.38
237822_at	excluded	absent	4.71	-0.46	included	present	192.03	2.68	excluded	absent	38.93	0.11	excluded	absent	52.47	0.34	included	present	514
237823_at	excluded	absent	84.02	-0.29	excluded	absent	92.49	-0.11	excluded	absent	44.1	-1.16	included	present	110.56	0.28	included	present	203.98
237824_at	excluded	absent	10.6	-0.31	excluded	absent	49.92	-0.15	excluded	absent	5.94	-0.33	excluded	absent	8.32	-0.32	excluded	absent	13.78
237825_x_at	excluded	absent	57.37	0.07	included	present	114.9	1.65	excluded	absent	37.41	-0.47	excluded	absent	33.58	-0.58	excluded	absent	67.66
237826_at	excluded	absent	56.86	-0.79	excluded	absent	40.4	-1.14	excluded	absent	53.76	-0.86	included	present	119.43	0.52	excluded	absent	6.22
237827_at	excluded	absent	50.15	-0.21	excluded	absent	88.77	-0.09	excluded	absent	35.38	-0.26	excluded	absent	6.12	-0.35	excluded	absent	3.89
237828_at	included	present	387.91	-0.02	included	present	1922.53	2.12	included	present	2505.45	2.93	included	present	1634.6	1.71	included	present	3093.83
237829_at	excluded	absent	9.33	-0.74	excluded	absent	54.92	0.38	excluded	absent	2.24	-0.92	excluded	absent	48.7	0.23	excluded	absent	11.32
237830_at	excluded	absent	1.91	-0.61	excluded	absent	42.45	-0.07	excluded	absent	33.3	-0.19	excluded	absent	71.94	0.32	excluded	absent	3.33
237831_x_at	excluded	absent	5.43	-0.63	excluded	absent	3.49	-0.69	excluded	absent	4.64	-0.65	excluded	absent	4.64	-0.65	excluded	absent	6.36
237832_at	excluded	absent	3.33	-0.65	excluded	absent	7.76	-0.62	excluded	absent	1.08	-0.66	excluded	absent	1.67	-0.66	excluded	absent	13.56
237833_s_at	included	absent	81	-0.7	included	present	116.31	-0.56	included	absent	82.04	-0.7	included	present	1102.11	3.58	included	absent	97.85
237834_at	excluded	absent	3.48	-0.6	excluded	absent	61.44	1.98	excluded	absent	3.45	-0.6	excluded	absent	97.82	3.6	excluded	absent	13.23
237835_at	excluded	absent	13.08	-0.6	excluded	absent	436.79	8.55	excluded	absent	38.83	-0.05	excluded	absent	74.5	0.72	included	present	458.2
237836_at	included	present	136.01	-0.46	included	absent	5.93	-1.3	included	absent	86.79	-0.78	included	absent	138.66	-0.44	included	present	174.22
237837_at	included	present	511.77	0.41	included	present	171.93	-0.55	included	present	267.43	-0.28	included	present	283.87	-0.23	included	present	456.15
237838_at	excluded	absent	6.37	-1.03	excluded	absent	53.05	-0.42	included	present	87.9	0.04	excluded	absent	31.87	-0.7	excluded	absent	101.66
237839_at	included	present	120.75	-0.3	included	absent	63.77	-0.8	included	absent	4.31	-1.32	included	absent	86	-0.61	included	absent	135.8
237840_at	excluded	absent	79.25	1.29	excluded	absent	64.49	0.84	excluded	absent	2.39	-1.03	excluded	absent	90.45	1.62	excluded	absent	95.76
237841_at	included	present	429.67	-0.08	included	present	434.56	-0.05	included	present	667.36	1.37	included	present	517.07	0.45	included	present	1036.76
237842_at	excluded	absent	13.31	-1.07	excluded	absent	3.77	-1.27	excluded	absent	3.59	-1.28	excluded	absent	24.25	-0.84	excluded	absent	17.58
237843_at	excluded	absent	160.59	-0.11	excluded	absent	21.38	-1.25	excluded	absent	15.32	-1.29	included	present	213.16	0.32	excluded	absent	37.99
237844_at	excluded	absent	100.91	1.23	excluded	absent	9.28	-1.09	excluded	absent	72.87	0.52	excluded	absent	2.74	-1.26	excluded	absent	42.96
237845_at	included	absent	133.75	-0.37	included	absent	73.85	-0.62	included	absent	154.42	-0.28	included	present	223.52	0	included	absent	187.33
237846_at	excluded	absent	112.21	0.77	excluded	absent	39.71	-0.54	excluded	absent	68.27	-0.03	excluded	absent	36.52	-0.6	excluded	absent	117.7
237847_at	excluded	absent	38.64	-0.1	excluded	absent	4.58	-1.15	excluded	absent	28.55	-0.41	excluded	absent	63.2	0.66	excluded	absent	78.79
237848_at	excluded	absent	53.65	-0.01	excluded	absent	5.07	-0.3	excluded	absent	1.65	-0.32	excluded	absent	2.43	-0.31	excluded	absent	17.32
237849_at	included	present	102.33	-0.99	included	absent	105.03	-0.98	included	absent	42.6	-1.26	included	absent	164.22	-0.72	included	absent	9.42
237850_at	excluded	absent	40.34	0.1	excluded	absent	41.42	0.14	excluded	absent	38.96	0.06	excluded	absent	1.52	-1.02	excluded	absent	56.46
237851_at	included	present	372.21	-0.45	included	present	326.42	-0.58	included	present	284.4	-0.71	included	present	323	-0.59	included	present	368.51
237852_at	excluded	absent	10.5	-0.81	excluded	absent	55.21	0.18	excluded	absent	72.68	0.56	excluded	absent	9.49	-0.83	excluded	absent	11.18
237853_x_at	excluded	absent	2.75	-0.72	excluded	absent	38.53	0.62	excluded	absent	2.49	-0.73	excluded	absent	3.64	-0.69	excluded	absent	6.51
237854_at	excluded	absent	78.82	0.36	excluded	absent	47.67	-0.29	excluded	absent	87.44	0.54	excluded	absent	69.2	0.16	excluded	absent	66.1
237855_at	excluded	absent	16.94	-0.58	excluded	absent	16.51	-0.59	excluded	absent	39.57	-0.03	excluded	absent	63.13	0.55	excluded	absent	20.41
237856_at	included	present	121.44	-0.28	included	present	165.94	0.26	included	absent	10.92	-1.64	included	present	132.35	-0.15	included	absent	101.39
237857_at	included	present	980.94	0.65	included	present	275.98	-0.7	included	present	1845.06	2.31	included	present	1050.08	0.79	included	present	1377.72
237858_s_at	excluded	absent	12.11	-1.07	excluded	absent	120.66	-0.23	excluded	absent	8.99	-1.1	excluded	absent	176.31	0.2	excluded	absent	119.57
237859_at	excluded	absent	74.87	-0.08	included	present	106.91	0.5	included	present	114.64	0.64	excluded	absent	42.81	-0.67	included	present	295.22
237860_at	included	present	808.3	5.19	included	present	248.88	0.38	included	present	2647.94	21.01	included	present	55.62	-1.29	included	present	916.42
237861_at	excluded	absent	57.13	-0.84	excluded	absent	59.46	-0.8	included	present	138.12	0.67	included	present	114.32	0.22	excluded	absent	157.68
237862_at	excluded	absent	23.38	0.2	excluded	absent	20.72	0.07	excluded	absent	2.31	-0.8	excluded	absent	4.47	-0.7	excluded	absent	3.87
237863_at	excluded	absent	101.41	-0.17	excluded	absent	93.27	-0.26	included	present	297.08	1.89	included	present	382.44	2.79	included	present	212
237864_at	excluded	absent	38.5	-0.33	excluded	absent	66.06	0.39	excluded	absent	37.89	-0.35	excluded	absent	15.52	-0.93	excluded	absent	33.38
237865_x_at	included	present	325.96	0.7	included	absent	89.72	-0.38	included	present	113	-0.27	included	absent	21.38	-0.69	included	absent	40.44
237866_at	excluded	absent	78.7	0.49	excluded	absent	2.23	-1.09	included	present	130.62	1.56	excluded	absent	2.52	-1.08	excluded	absent	4.64
237867_s_at	included	present	179.14	0.33	excluded	absent	4.33	-0.9	excluded	absent	64.48	-0.48	excluded	absent	41.94	-0.63	excluded	absent	12.1
237868_x_at	included	present	734.64	1.71	included	present	371.43	-0.28	included	present	802.93	2.08	included	present	336.01	-0.47	included	present	967.78
237869_at	excluded	absent	70.48	-0.74	excluded	absent	98.43	-0.2	excluded	absent	39.17	-1.35	excluded	absent	47.57	-1.19	included	present	210.82
237870_at	excluded	absent	87.06	-0.79	excluded	absent	12.47	-1.17	excluded	absent	76.54	-0.84	excluded	absent	37.94	-1.04	included	present	201.06
237871_x_at	excluded	absent	72.02	0.5	excluded	absent	41.13	-0.03	excluded	absent	2.84	-0.69	excluded	absent	2.94	-0.69	excluded	absent	3.98
237872_at	excluded	absent	38.59	-0.24	excluded	absent	88.27	0.02	excluded	absent	4.58	-0.41	excluded	absent	5.06	-0.41	excluded	absent	8.87
237873_s_at	excluded	absent	39.15	-0.72	excluded	absent	42.81	-0.64	excluded	absent	3.55	-1.49	included	present	74.06	0.04	included	present	205.63
237874_at	excluded	absent	27.73	0.1	excluded	absent	21.52	-0.13	excluded	absent	3.56	-0.78	excluded	absent	2.34	-0.82	excluded	absent	57.9
237875_at	excluded	absent	118.93	0.84	excluded	absent	43.5	-0.16	excluded	absent	6.68	-0.64	excluded	absent	18.22	-0.49	excluded	absent	55.28
237876_at	excluded	absent	40.71	-0.05	excluded	absent	4.33	-0.96	excluded	absent	6.73	-0.9	excluded	absent	1.95	-1.02	excluded	absent	2.89
237877_at	excluded	absent	169.74	0.09	excluded	absent	210.02	0.59	included	present	364.18	2.5	included	present	365.58	2.52	excluded	absent	281.63
237878_at	excluded	absent	12.55	-1.28	excluded	absent	83.31	0.59	excluded	absent	48.58	-0.33	excluded	absent	24.04	-0.98	excluded	absent	103.71
237879_at	excluded	absent	57.27	-0.24	excluded	absent	4.97	-0.91	excluded	absent	23.35	-0.68	excluded	absent	35.3	-0.53	excluded	absent	32.19
237880_at	excluded	absent	36.99	-0.48	excluded	absent	10.17	-1.07	excluded	absent	45.56	-0.29	excluded	absent	37.7	-0.46	excluded	absent	16.07
237881_at	excluded	absent	127.89	0.49	excluded	absent	16.53	-1.15	excluded	absent	4.88	-1.32	excluded	absent	7.2	-1.29	excluded	absent	160.57
237882_at	excluded	absent	52.97	-0.18	excluded	absent	79.62	0.41	excluded	absent	44.21	-0.38	excluded	absent	8.95	-1.16	excluded	absent	149.59
237883_at	excluded	absent	56.9	0.04	excluded	absent	24.72	-0.74	excluded	absent	2.4	-1.28	excluded	absent	45.61	-0.23	excluded	absent	163.04
237884_x_at	included	present	153.46	-0.33	included	present	111.15	-0.79	included	present	146.49	-0.41	included	present	177.14	-0.08	included	present	249.25
237885_at	included	absent	55.52	-0.78	included	absent	4.99	-0.93	included	absent	65.71	-0.75	included	absent	36.54	-0.84	included	absent	95.55
237886_at	excluded	absent	6.8	-0.59	excluded	absent	60.69	1.51	excluded	absent	35.17	0.52	excluded	absent	33.9	0.47	excluded	absent	52.47
237887_at	excluded	absent	56.34	0.14	excluded	absent	32.33	-0.33	excluded	absent	66.29	0.33	excluded	absent	15.12	-0.67	excluded	absent	139.79
237888_at	excluded	absent	38.88	-0.16	excluded	absent	3.13	-0.27	included	present	79.78	-0.04	excluded	absent	8.86	-0.25	excluded	absent	29.51
237889_s_at	excluded	absent	72.27	-0.18	excluded	absent	53.66	-0.2	included	present	193.15	-0.02	excluded	absent	44.25	-0.21	excluded	absent	63.07
237890_at	excluded	absent	35.18	-0.48	excluded	absent	15.08	-0.79	excluded	absent	3.39	-0.98	excluded	absent	4.43	-0.96	excluded	absent	145.33
237891_at	included	present	383.29	1.97	included	present	84.47	-1.69	included	present	184.08	-0.47	included	present	188.49	-0.42	included	present	446
237892_at	included	present	279.14	0	excluded	absent	95.18	-0.59	included	present	218.5	-0.19	included	present	194.74	-0.27	included	absent	169.18
237893_at	included	present	446.18	-0.08	included	absent	49.8	-1.65	included	present	1041.13	2.27	included	present	284.44	-0.72	included	present	415.87
237894_at	excluded	absent	73.63	0.17	excluded	absent	6.95	-0.79	excluded	absent	12.43	-0.72	excluded	absent	6.07	-0.81	excluded	absent	6.65
237895_at	included	present	354.22	-0.11	included	present	152.65	-0.96	included	present	355.53	-0.11	included	present	334.42	-0.2	included	present	424.44
237896_at	excluded	absent	23.98	-0.78	excluded	absent	2.69	-1.3	excluded	absent	108.17	1.28	excluded	absent	34.13	-0.53	excluded	absent	55.58
237897_at	included	present	206.86	-0.3	included	present	115.47	-1.34	included	present	573.32	3.86	included	present	288.22	0.63	included	present	289.82
237898_at	excluded	absent	43.48	0.32	excluded	absent	37.36	0.14	included	present	85.82	1.55	excluded	absent	13.07	-0.56	included	present	152.8
237899_at	excluded	absent	2.3	-1.14	excluded	absent	53.75	-0.31	excluded	absent	3.12	-1.13	excluded	absent	11.78	-0.99	excluded	absent	19.61
237900_at	excluded	absent	91.77	1.79	excluded	absent	30.73	0.08	excluded	absent	36.3	0.23	excluded	absent	9.08	-0.53	excluded	absent	4.95
237901_at	excluded	absent	137.1	-0.26	excluded	absent	81.4	-0.94	excluded	absent	100.15	-0.71	excluded	absent	87.86	-0.86	included	present	181.98
237902_at	excluded	absent	66.31	0.2	excluded	absent	2.1	-1.04	excluded	absent	5.27	-0.97	excluded	absent	35.69	-0.39	excluded	absent	6.45
237903_at	included	present	412.96	-0.63	included	present	120.47	-1.02	included	present	548.09	-0.45	included	present	263.56	-0.83	included	present	246.25
237904_at	excluded	absent	2.21	-0.81	excluded	absent	1.81	-0.83	excluded	absent	2.28	-0.8	excluded	absent	5.88	-0.62	excluded	absent	78.64
237905_at	excluded	absent	2.35	-0.22	excluded	absent	43.53	-0.15	excluded	absent	39.36	-0.16	excluded	absent	13.73	-0.2	excluded	absent	4.15
237906_at	excluded	absent	1.51	-0.81	excluded	absent	16.35	-0.33	excluded	absent	8.67	-0.58	excluded	absent	3.64	-0.74	excluded	absent	7.83
237907_at	included	present	162.66	1.21	excluded	absent	15.92	-1.56	included	present	166.26	1.28	included	present	97.52	-0.02	included	present	148.67
237908_at	included	present	97.79	0.56	excluded	absent	1.41	-1.3	included	present	77.65	0.17	excluded	absent	71.1	0.04	excluded	absent	38.66
237909_at	excluded	absent	91.91	0.16	excluded	absent	159.68	0.64	excluded	absent	7.17	-0.45	excluded	absent	3.22	-0.48	excluded	absent	5.71
237910_x_at	included	present	1784.24	1.78	included	present	707.35	-0.75	included	present	2138.62	2.61	included	present	1874.01	1.99	included	present	1464.18
237911_at	excluded	absent	4.14	-0.96	excluded	absent	2.58	-1.01	excluded	absent	23.98	-0.32	excluded	absent	61.19	0.88	excluded	absent	35.68
237912_at	included	present	402.35	-0.82	included	present	643.36	-0.36	included	present	178.31	-1.25	included	present	504.71	-0.62	included	present	412.63
237913_at	excluded	absent	69.03	0.08	excluded	absent	68.58	0.07	excluded	absent	1.99	-0.87	excluded	absent	42.66	-0.3	excluded	absent	3.92
237914_s_at	included	present	1380.8	0.65	included	present	875.38	-0.72	included	present	1480.16	0.92	included	present	2417.1	3.47	included	present	899.1
237915_at	excluded	absent	6.23	-0.72	excluded	absent	4.94	-0.76	excluded	absent	27.83	0.04	excluded	absent	57.74	1.08	excluded	absent	10.1
237916_at	included	present	177.32	-0.1	included	present	238.18	0.55	included	present	185.7	-0.01	included	present	108.58	-0.82	included	present	139.16
237917_at	included	present	315.54	-0.57	included	present	158.06	-1.29	included	present	664.44	1.03	included	present	285.02	-0.71	included	present	682.53
237918_at	excluded	absent	4.61	-1.18	excluded	absent	2.99	-1.23	excluded	absent	16.26	-0.87	included	present	72.63	0.65	excluded	absent	7.15
237919_at	included	present	2777.32	0.11	included	present	2086.05	-0.28	included	present	2118.16	-0.26	included	present	3309.91	0.41	included	present	2321.2
237920_at	excluded	absent	1.9	-0.6	excluded	absent	40.95	0.58	excluded	absent	5.72	-0.48	excluded	absent	3.06	-0.56	excluded	absent	66.13
237921_at	excluded	absent	2.53	-0.45	excluded	absent	2.58	-0.45	excluded	absent	2.76	-0.44	excluded	absent	2.98	-0.43	excluded	absent	4.86
237922_at	excluded	absent	26.1	-0.01	excluded	absent	21.51	-0.16	excluded	absent	11.55	-0.48	excluded	absent	8.18	-0.59	excluded	absent	15.16
237923_at	excluded	absent	42.61	0.35	excluded	absent	3.8	-0.79	excluded	absent	4.02	-0.78	excluded	absent	73.33	1.25	excluded	absent	5.45
237924_at	excluded	absent	1.89	-0.85	excluded	absent	31.32	0.09	excluded	absent	97.64	2.22	excluded	absent	32.1	0.12	excluded	absent	4.57
237925_at	excluded	absent	129.06	0.76	excluded	absent	48.8	-0.42	excluded	absent	70.58	-0.1	excluded	absent	54.3	-0.34	excluded	absent	63.05
237926_s_at	included	present	118.07	0.25	excluded	absent	19.7	-0.98	excluded	absent	10.16	-1.1	excluded	absent	58.8	-0.49	excluded	absent	148.63
237927_at	excluded	absent	42.99	0.33	excluded	absent	0.84	-0.89	excluded	absent	2.29	-0.85	included	present	618.42	16.93	excluded	absent	18.3
237928_at	excluded	absent	72.07	-0.09	excluded	absent	8.35	-1.31	included	present	93.74	0.33	excluded	absent	3.66	-1.4	excluded	absent	2.24
237929_at	excluded	absent	9.42	-0.23	excluded	absent	16.82	-0.15	excluded	absent	8.85	-0.24	excluded	absent	3.11	-0.3	excluded	absent	15.55
237930_at	excluded	absent	42.36	2	excluded	absent	15.33	0.33	excluded	absent	3.11	-0.43	excluded	absent	79.74	4.3	excluded	absent	57.65
237931_at	excluded	absent	43.76	-1.06	excluded	absent	11.17	-1.77	included	present	86.17	-0.13	excluded	absent	27.71	-1.41	excluded	absent	138.56
237932_at	excluded	absent	2.09	-1.01	excluded	absent	69.21	0.47	excluded	absent	6.49	-0.91	excluded	absent	42.53	-0.12	excluded	absent	9.05
237933_at	excluded	absent	13.78	-0.81	excluded	absent	26.45	-0.69	excluded	absent	39.45	-0.56	excluded	absent	29.24	-0.66	excluded	absent	69.74
237934_at	excluded	absent	109.07	0.86	excluded	absent	81.23	0.37	excluded	absent	4.47	-0.98	excluded	absent	3.18	-1	excluded	absent	20.56
237935_at	excluded	absent	8.01	-0.75	excluded	absent	8.75	-0.74	excluded	absent	41.16	0.06	excluded	absent	5.11	-0.83	excluded	absent	23.61
237936_at	included	absent	7.32	-1.5	included	present	83.51	-0.72	included	absent	57.15	-0.99	included	absent	26.92	-1.3	included	absent	22.25
237937_x_at	excluded	absent	13.41	-0.77	included	present	47.52	-0.57	included	absent	2.66	-0.83	included	present	66.66	-0.46	included	absent	119.86
237938_at	included	present	456.32	0.58	included	absent	58.94	-0.59	included	absent	92.06	-0.5	included	absent	19.38	-0.71	included	absent	91.73
237939_at	included	present	861.91	0.9	excluded	absent	134.19	-0.36	included	present	17227.77	29.1	excluded	absent	2.22	-0.59	included	present	911.3
237940_s_at	excluded	absent	92.17	-0.68	included	present	1148.06	5.67	included	present	778.19	3.45	included	present	674.65	2.82	included	present	697.33
237941_at	excluded	absent	36.98	-0.75	excluded	absent	289.34	2.75	included	present	202.46	1.55	included	present	174.56	1.16	included	absent	136.64
237942_at	included	present	210.48	-0.77	included	present	180.12	-0.99	included	present	224.33	-0.66	included	present	157.01	-1.17	included	present	229.05
237943_at	excluded	absent	57.67	-1.17	excluded	absent	33.35	-1.52	included	present	81.82	-0.83	included	present	180.99	0.57	excluded	absent	93.84
237944_at	excluded	absent	3.7	-1.13	excluded	absent	3.38	-1.14	excluded	absent	4.11	-1.12	excluded	absent	40.51	-0.45	excluded	absent	33.31
237945_at	included	present	1402.17	0.5	included	absent	104.69	-0.78	included	present	331.55	-0.55	included	present	2348.53	1.43	included	present	2226.62
237946_at	excluded	absent	7.21	-0.52	excluded	absent	48.94	0.03	excluded	absent	20.83	-0.34	excluded	absent	1.65	-0.59	excluded	absent	76.04
237947_at	excluded	absent	7.23	-0.25	excluded	absent	13.41	0.03	excluded	absent	10.75	-0.09	excluded	absent	34.21	0.99	excluded	absent	8.22
237948_at	excluded	absent	16.56	-0.24	excluded	absent	1.65	-0.78	excluded	absent	7.32	-0.58	excluded	absent	2.07	-0.77	excluded	absent	13.36
237949_at	included	present	299.14	-0.99	included	present	293.63	-1.01	included	present	150.56	-1.46	included	present	277.56	-1.06	included	present	472.77
237950_s_at	included	present	172.39	-0.2	included	absent	81.62	-0.84	included	present	298.56	0.69	included	present	225.27	0.18	included	present	297.88
237951_at	excluded	absent	19.42	-1.43	excluded	absent	0.75	-1.85	included	present	118.99	0.82	excluded	absent	67.24	-0.35	excluded	absent	80.04
237952_at	included	present	197.7	0.34	excluded	absent	54.59	-0.73	excluded	absent	10.98	-1.06	included	absent	116.73	-0.27	included	absent	132.5
237953_at	excluded	absent	6.18	-0.46	excluded	absent	2.31	-0.51	excluded	absent	45.87	0.02	excluded	absent	1.95	-0.51	excluded	absent	137.06
237954_x_at	included	present	1097.33	2.13	included	present	502.57	-0.16	included	present	765.35	0.85	included	present	495.16	-0.19	included	present	888.06
237955_at	excluded	absent	15.47	-0.92	excluded	absent	2.76	-1.11	excluded	absent	30.92	-0.69	excluded	absent	1.68	-1.12	excluded	absent	67.67
237956_s_at	excluded	absent	33.45	-0.21	excluded	absent	36.08	-0.21	excluded	absent	5.91	-0.23	excluded	absent	1.78	-0.23	excluded	absent	3.44
237957_at	excluded	absent	54.95	0.98	excluded	absent	66.6	1.33	excluded	absent	1.41	-0.66	included	present	64.39	1.27	excluded	absent	54.72
237958_at	excluded	absent	23.08	-0.46	excluded	absent	31.08	-0.22	excluded	absent	91.64	1.59	excluded	absent	62.73	0.72	excluded	absent	3.7
237959_at	excluded	absent	77.43	-0.02	excluded	absent	83.8	0.09	included	present	98.24	0.33	excluded	absent	89.26	0.18	excluded	absent	78.91
237960_at	excluded	absent	3.14	-0.72	excluded	absent	100.5	2.08	excluded	absent	33.7	0.16	excluded	absent	12.25	-0.46	excluded	absent	1.46
237961_at	excluded	absent	30.1	-0.08	excluded	absent	20.66	-0.36	excluded	absent	3.21	-0.86	excluded	absent	6.1	-0.77	excluded	absent	4.05
237962_x_at	included	present	339.64	-1.23	included	present	276.52	-1.45	included	present	970.19	1.03	included	present	632.74	-0.18	included	present	658.05
237963_x_at	excluded	absent	41.22	-1.3	included	present	115.13	-0.04	included	present	145.26	0.47	excluded	absent	79.53	-0.65	included	present	132.85
237964_at	excluded	absent	53.89	0.91	excluded	absent	40.59	0.47	excluded	absent	2.89	-0.78	excluded	absent	5.61	-0.69	excluded	absent	4.26
237965_at	excluded	absent	6.5	-0.24	excluded	absent	3.43	-0.42	excluded	absent	2.68	-0.46	excluded	absent	9.17	-0.09	excluded	absent	5.27
237966_at	excluded	absent	7.72	-0.66	excluded	absent	3.06	-0.85	excluded	absent	1.66	-0.9	excluded	absent	27.95	0.13	excluded	absent	7.14
237967_at	excluded	absent	38.39	-0.02	excluded	absent	78.62	0.3	excluded	absent	53	0.1	excluded	absent	28.28	-0.1	excluded	absent	7.85
237968_at	included	present	157.61	-0.77	included	present	130.44	-0.88	included	present	729.45	1.7	included	present	215.85	-0.52	included	present	350.38
237969_at	included	present	1381.82	7.91	included	absent	17.28	-0.91	included	absent	80.69	-0.5	included	absent	76.84	-0.53	included	absent	2.91
237970_at	excluded	absent	7.84	-0.67	excluded	absent	10.9	-0.59	excluded	absent	44.24	0.19	excluded	absent	57.55	0.51	excluded	absent	4.61
237971_at	excluded	absent	18.64	-0.61	excluded	absent	47.89	0.18	excluded	absent	5.79	-0.96	excluded	absent	3.11	-1.03	excluded	absent	10.26
237972_at	excluded	absent	89.47	0.19	excluded	absent	70.42	-0.2	excluded	absent	48.82	-0.63	excluded	absent	27.84	-1.05	excluded	absent	91.47
237973_at	excluded	absent	3.17	-1.3	excluded	absent	47.82	-0.19	excluded	absent	4.97	-1.25	excluded	absent	87.12	0.78	excluded	absent	9.41
237974_at	excluded	absent	49.12	-0.53	excluded	absent	1.43	-0.75	excluded	absent	3.63	-0.74	included	present	79.05	-0.39	excluded	absent	7.94
237975_at	excluded	absent	53.33	-0.28	excluded	absent	46.64	-0.43	excluded	absent	47.29	-0.42	excluded	absent	26.53	-0.88	excluded	absent	21.33
237976_at	excluded	absent	49.35	0.56	excluded	absent	109.99	2.33	excluded	absent	27.01	-0.09	excluded	absent	43.19	0.38	excluded	absent	65.84
237977_at	excluded	absent	48.6	0.83	excluded	absent	7.38	-0.59	excluded	absent	15.24	-0.32	excluded	absent	15.41	-0.31	excluded	absent	32.81
237978_at	included	present	174.69	2.85	excluded	absent	36.14	-0.09	excluded	absent	2.46	-0.8	excluded	absent	2.78	-0.8	excluded	absent	60.39
237979_at	excluded	absent	2.51	-0.8	excluded	absent	5.47	-0.71	excluded	absent	5.68	-0.71	excluded	absent	38.84	0.21	excluded	absent	4.55
237980_at	excluded	absent	4.1	-0.35	excluded	absent	85.49	0.12	excluded	absent	40.05	-0.15	excluded	absent	143.34	0.45	excluded	absent	46.32
237981_at	included	present	873.95	-0.11	included	absent	5.2	-0.24	included	absent	43.84	-0.24	included	absent	2.23	-0.24	included	absent	66.67
237982_at	excluded	absent	64.37	-0.23	included	present	116.04	-0.21	excluded	absent	1.97	-0.25	excluded	absent	144.4	-0.21	excluded	absent	66.76
237983_at	excluded	absent	2.93	-0.98	excluded	absent	36.32	-0.15	excluded	absent	51.21	0.22	included	present	83.22	1.02	excluded	absent	8.78
237984_x_at	excluded	absent	2.1	-0.91	excluded	absent	2.09	-0.91	excluded	absent	4.03	-0.84	excluded	absent	1.64	-0.92	excluded	absent	2.91
237985_at	excluded	absent	3.06	-0.54	excluded	absent	4.37	-0.49	excluded	absent	3.12	-0.54	excluded	absent	2.5	-0.56	excluded	absent	4.96
237986_at	excluded	absent	12.27	-0.62	excluded	absent	55.82	-0.12	excluded	absent	6.55	-0.69	excluded	absent	28.45	-0.43	excluded	absent	104.75
237987_x_at	excluded	absent	2.22	-0.22	excluded	absent	17.42	-0.15	excluded	absent	10.02	-0.18	excluded	absent	11	-0.18	excluded	absent	61.23
237988_at	included	present	159.72	-0.53	included	present	121.92	-1.02	included	present	199.75	-0.01	included	present	115.55	-1.1	included	present	248.34
237989_at	excluded	absent	52.64	-0.13	excluded	absent	1.29	-0.17	excluded	absent	2.11	-0.17	excluded	absent	2.15	-0.17	excluded	absent	10.07
237990_x_at	excluded	absent	43.42	1.42	excluded	absent	12.22	-0.1	excluded	absent	5.54	-0.43	excluded	absent	15.2	0.04	excluded	absent	2.73
237991_at	excluded	absent	43.87	1.64	excluded	absent	2.74	-0.46	excluded	absent	1.58	-0.52	excluded	absent	2.8	-0.46	excluded	absent	60.59
237992_at	excluded	absent	1.32	-0.65	excluded	absent	11.68	-0.18	excluded	absent	1.62	-0.64	excluded	absent	4.49	-0.51	excluded	absent	56.49
237993_at	excluded	absent	72.82	0.25	excluded	absent	22.47	-0.69	excluded	absent	17.94	-0.77	excluded	absent	29.94	-0.55	excluded	absent	9.43
237994_at	included	present	209.6	-0.37	included	absent	88.83	-1.18	included	present	157.86	-0.72	included	absent	70.52	-1.31	included	absent	85.8
237995_at	excluded	absent	73.46	-0.2	included	present	589.73	3.11	included	present	1303.06	7.7	included	present	229.17	0.8	included	present	973.01
237996_at	excluded	absent	61.93	0.69	excluded	absent	1.54	-0.5	excluded	absent	1.29	-0.5	excluded	absent	1.56	-0.5	excluded	absent	3.09
237997_s_at	excluded	absent	1.91	-0.47	excluded	absent	4.78	-0.39	excluded	absent	21.94	0.06	excluded	absent	34.05	0.38	excluded	absent	2.69
237998_at	excluded	absent	94.13	-0.57	excluded	absent	25.99	-1.49	excluded	absent	117.47	-0.25	excluded	absent	46.59	-1.21	excluded	absent	134.66
237999_at	excluded	absent	1.72	-1.18	excluded	absent	26.94	-0.71	included	present	147.66	1.51	excluded	absent	19.04	-0.86	excluded	absent	24.16
238000_at	included	present	189.82	-0.52	included	present	284.76	0.42	included	present	184.36	-0.57	included	present	358.97	1.16	included	present	174.73
238001_at	included	present	277.36	-0.09	included	present	215.82	-0.58	included	present	507.47	1.76	included	present	169.48	-0.95	included	present	627.66
238002_at	included	present	3187	0.2	included	present	1918.86	-0.55	included	present	1774.93	-0.64	included	present	1142.34	-1.01	included	present	1279.11
238003_at	included	present	318.99	-0.49	excluded	absent	9.61	-0.73	excluded	absent	4.26	-0.74	excluded	absent	65.79	-0.69	excluded	absent	9.41
238004_at	included	present	205.34	-0.2	included	present	382.08	1.13	included	present	84.28	-1.11	included	present	262.64	0.23	included	present	361.36
238005_s_at	included	present	514.9	-0.63	included	present	938.82	0.64	included	present	796.68	0.21	included	present	847.02	0.36	included	present	382.44
238006_at	included	present	496.31	0.24	included	present	497.9	0.25	included	present	648.69	1.02	included	present	597.98	0.76	included	absent	221.62
238007_at	included	present	276.64	1.16	included	present	302.32	1.5	included	present	231.08	0.56	included	present	433.25	3.25	included	present	201.97
238008_at	excluded	absent	2.47	-0.43	excluded	absent	27.09	-0.41	excluded	absent	7.26	-0.42	excluded	absent	304.87	-0.2	excluded	absent	8.85
238009_at	included	present	1133.9	1.07	included	present	408.27	-0.56	included	present	131.61	-1.18	included	present	358.57	-0.67	included	present	253.99
238010_at	included	present	386.73	0.34	included	present	289.45	-0.46	included	present	315.95	-0.24	included	present	260.96	-0.69	included	present	241.17
238011_at	included	present	2778.66	-0.57	included	present	4028.34	0.74	included	present	3598.77	0.29	included	present	2053.9	-1.33	included	present	2009.1
238012_at	included	present	91.19	-0.16	included	present	139.47	0.52	included	present	208.73	1.5	included	present	194.73	1.3	included	present	208.04
238013_at	included	present	1073.55	0.6	included	present	171.34	-1.54	included	present	315.55	-1.2	included	present	626.8	-0.46	included	present	546.12
238014_at	included	present	668.25	-0.03	included	present	401.5	-0.68	included	present	1261.48	1.41	included	present	1407.97	1.77	included	present	815.98
238015_at	excluded	absent	145.37	-0.15	included	present	471.23	2.07	included	present	1108.26	6.42	included	present	296.26	0.88	included	present	336.13
238016_s_at	excluded	absent	125.81	0.53	excluded	absent	109.42	0.26	excluded	absent	149.94	0.93	excluded	absent	13	-1.35	excluded	absent	72.11
238017_at	included	present	387.37	-0.03	excluded	absent	32.78	-0.4	excluded	absent	1.16	-0.44	excluded	absent	3.75	-0.43	excluded	absent	5.12
238018_at	included	absent	24.84	-0.62	included	present	130.42	-0.46	included	present	151.34	-0.43	included	present	105.17	-0.5	included	absent	47.91
238019_at	excluded	absent	48.11	-0.5	excluded	absent	9.6	-0.94	excluded	absent	73.7	-0.21	excluded	absent	4.46	-1	excluded	absent	75.36
238020_at	included	present	262.74	0.3	included	absent	117.04	-1.33	included	present	224.64	-0.13	included	present	179.41	-0.63	included	absent	128.98
238021_s_at	included	present	157.77	-0.55	included	present	5296.18	0.81	included	present	7679.47	1.44	included	present	1856.88	-0.1	included	present	4787.09
238022_at	included	absent	72.91	-0.41	included	present	917.17	0.13	included	present	1808.86	0.71	included	present	412.31	-0.19	included	present	1127.23
238023_at	excluded	absent	30.23	-0.4	excluded	absent	90.36	1.09	excluded	absent	31.16	-0.37	excluded	absent	67.15	0.52	excluded	absent	84.67
238024_at	included	present	152.6	-0.43	included	present	215.32	0.36	included	present	349.52	2.06	included	present	201.23	0.19	included	absent	85.39
238025_at	included	present	283.51	0.03	included	present	87.39	-0.93	included	absent	80.95	-0.96	included	absent	22.57	-1.25	included	present	225.11
238026_at	included	present	837.24	-1.15	included	present	1188.33	-0.47	included	present	1485.52	0.1	included	present	656.68	-1.49	included	present	764.12
238027_at	excluded	absent	38.62	-0.31	excluded	absent	90.07	-0.16	excluded	absent	54.24	-0.27	excluded	absent	51.21	-0.28	excluded	absent	106.48
238028_at	excluded	absent	122.64	-0.2	excluded	absent	77.29	-0.31	excluded	absent	71.98	-0.33	included	present	292.6	0.24	excluded	absent	19.86
238029_s_at	included	present	567.01	0.29	included	present	933.24	1.34	included	present	360.47	-0.3	included	present	606.23	0.4	included	present	214.27
238030_at	excluded	absent	32.63	-0.57	excluded	absent	83.89	0.24	excluded	absent	80.34	0.18	included	present	174.29	1.66	excluded	absent	84.71
238031_at	excluded	absent	17.39	-0.63	excluded	absent	92.61	0.94	excluded	absent	19.72	-0.58	excluded	absent	84.09	0.77	excluded	absent	103.49
238032_at	included	present	386.94	0.24	included	absent	120.36	-1.01	included	absent	79.88	-1.2	included	absent	24.18	-1.46	included	absent	171.96
238033_at	excluded	absent	43.63	-0.07	excluded	absent	79.43	0.86	excluded	absent	37.46	-0.23	excluded	absent	67	0.54	excluded	absent	2.18
238034_at	included	present	1368.29	1.1	included	present	425.7	-0.74	included	present	394.3	-0.8	included	present	1235.81	0.84	included	present	529.45
238035_at	included	present	356.91	-0.61	included	present	277.39	-0.83	included	present	791.26	0.55	included	present	1204.13	1.65	included	present	567.61
238036_at	excluded	absent	91.46	-0.16	excluded	absent	3.37	-1.36	excluded	absent	3.61	-1.36	excluded	absent	45.8	-0.78	excluded	absent	20.15
238037_at	included	present	455.49	0.47	included	absent	113.67	-0.85	included	present	218.99	-0.44	included	absent	250.37	-0.32	included	present	160.36
238038_at	included	present	479.5	-1.58	included	present	2516.36	2.56	included	present	2085.39	1.68	included	present	1735.78	0.97	included	present	783.11
238039_at	excluded	absent	364.26	0.76	excluded	absent	279.12	0.18	excluded	absent	256.04	0.02	excluded	absent	632.27	2.57	excluded	absent	282.82
238040_at	included	present	420.64	0.12	included	present	180.91	-0.86	included	present	491.88	0.41	included	present	491.46	0.41	included	present	225.36
238041_at	included	present	322.5	-0.97	included	present	549.55	-0.54	included	present	262.38	-1.08	included	present	1123.11	0.53	included	present	569.39
238042_at	excluded	absent	97.29	-0.97	included	present	184.69	-0.34	included	present	138.63	-0.67	included	present	261.68	0.21	excluded	absent	212.55
238043_at	included	present	393.04	-0.67	included	present	408.45	-0.59	included	present	524.59	0.02	included	present	629.93	0.57	included	present	831.82
238044_at	excluded	absent	114.16	0.37	excluded	absent	16.22	-0.98	excluded	absent	160.81	1.02	excluded	absent	12.57	-1.04	excluded	absent	107.21
238045_at	included	present	917.96	2.07	included	present	591.38	0.55	included	present	1273.59	3.73	included	present	444.49	-0.14	included	present	1279.61
238046_x_at	excluded	absent	62.4	0.69	excluded	absent	23.83	-0.49	excluded	absent	49.52	0.3	excluded	absent	40.36	0.02	excluded	absent	12.34
238047_at	included	present	1664.72	0.67	included	present	308.35	-0.08	included	present	50.33	-0.22	excluded	absent	6.15	-0.24	included	present	4311.35
238048_at	included	present	265.65	-0.43	included	absent	129.8	-1.11	included	present	354.68	0.01	included	present	418.96	0.33	included	present	276.3
238049_at	included	present	239.08	-0.99	included	absent	56.96	-1.71	included	absent	50.09	-1.74	included	present	262.11	-0.9	included	absent	45.17
238050_at	included	present	253.03	0.34	excluded	absent	107.49	-0.19	included	present	273.4	0.42	excluded	absent	33.28	-0.46	excluded	absent	6.82
238051_x_at	excluded	absent	12.37	-0.61	excluded	absent	25.77	0.34	excluded	absent	10.67	-0.73	excluded	absent	27.68	0.47	excluded	absent	15.59
238052_at	excluded	absent	61.11	-0.25	excluded	absent	2.94	-1.83	excluded	absent	4.97	-1.78	excluded	absent	80.96	0.28	excluded	absent	14.37
238053_at	included	present	160.88	0.8	excluded	absent	76.09	-0.65	included	present	165.53	0.88	included	present	286	2.93	excluded	absent	66.35
238054_at	excluded	absent	68.92	-0.03	excluded	absent	38.48	-0.21	excluded	absent	5.53	-0.41	excluded	absent	3.04	-0.42	excluded	absent	108.36
238055_at	included	present	426.87	-0.5	included	present	743.45	-0.09	included	absent	110.85	-0.9	included	present	809.69	-0.01	included	present	410.32
238056_at	included	present	371.76	-1.01	included	present	256.52	-1.36	included	present	348.67	-1.08	included	present	396.84	-0.94	included	present	266.7
238057_at	included	present	310.41	-0.24	included	present	288.55	-0.48	included	present	343.38	0.12	included	present	317.17	-0.17	included	present	166.17
238058_at	included	present	90.28	-1.6	included	present	148.68	-1.46	included	present	733.13	-0.06	included	present	781.66	0.06	included	present	687.19
238059_at	excluded	absent	43.85	-0.44	excluded	absent	7.71	-1.13	excluded	absent	98.98	0.63	excluded	absent	4.49	-1.19	excluded	absent	149.48
238060_s_at	excluded	absent	111.23	0.71	excluded	absent	124.92	1.09	excluded	absent	98.23	0.35	excluded	absent	103.69	0.5	excluded	absent	77.14
238061_at	excluded	absent	33.18	-0.41	excluded	absent	99.01	0.25	excluded	absent	47.24	-0.27	excluded	absent	3.49	-0.7	excluded	absent	7.36
238062_at	excluded	absent	103.37	-0.25	excluded	absent	127.93	-0.2	excluded	absent	7.27	-0.46	excluded	absent	33.64	-0.4	excluded	absent	7.07
238063_at	included	present	186.84	-0.13	excluded	absent	71.18	-0.31	excluded	absent	55.51	-0.34	included	present	1248.19	1.59	excluded	absent	139.89
238064_at	included	present	109.09	-0.99	included	absent	39.14	-1.44	included	present	125.12	-0.88	included	present	154.26	-0.7	included	present	221.62
238065_at	excluded	absent	91.06	-0.9	included	present	136.72	-0.23	included	present	207.1	0.82	included	present	118.25	-0.5	included	present	353.92
238066_at	included	present	847.15	-0.6	included	present	320.06	-1.04	included	present	208.69	-1.13	included	present	313.18	-1.04	included	present	1707.29
238067_at	included	present	120.26	0.18	excluded	absent	47.11	-0.57	excluded	absent	40.14	-0.64	excluded	absent	64.21	-0.39	excluded	absent	45.74
238068_at	included	present	299.1	-0.31	included	absent	170.7	-1.51	included	present	314.3	-0.16	included	present	160.93	-1.6	included	present	567.75
238069_at	included	present	344.95	0.77	included	present	138.04	-1.01	included	present	209.35	-0.4	included	present	445.57	1.63	included	present	184.17
238070_at	excluded	absent	7.59	-1.08	included	present	163.14	1.67	excluded	absent	32.93	-0.63	excluded	absent	102.27	0.59	excluded	absent	18.13
238071_at	included	present	83.21	-0.54	included	absent	78.97	-0.56	included	present	119.58	-0.41	included	present	185.54	-0.16	included	present	135.32
238072_at	included	present	134.15	1.2	excluded	absent	89.19	0.43	excluded	absent	7.06	-0.98	excluded	absent	42.19	-0.37	excluded	absent	13.89
238073_at	included	present	4233.79	2	included	present	10061.66	5.63	included	present	7816.74	4.23	included	present	3576.33	1.59	included	present	10086.93
238074_at	excluded	absent	149.06	1.36	excluded	absent	67.4	-0.07	excluded	absent	17.73	-0.95	excluded	absent	104.39	0.58	excluded	absent	4.89
238075_at	excluded	absent	27.34	-1.27	included	present	478.68	7.43	included	present	863.4	14.85	included	present	722.55	12.13	excluded	absent	107.16
238076_at	included	present	114.65	-1.35	included	present	196	-0.96	included	present	254.13	-0.69	included	present	265.45	-0.63	included	present	285.39
238077_at	included	present	723.12	-0.29	included	present	1059.68	0.6	included	present	1437.05	1.59	included	present	488.84	-0.91	included	present	911.17
238078_at	included	present	259.73	0.45	included	present	218.54	-0.12	included	present	117.99	-1.51	included	present	316.74	1.23	included	present	270.79
238079_at	excluded	absent	69.56	-0.11	excluded	absent	8.47	-0.22	excluded	absent	52.53	-0.14	excluded	absent	61.87	-0.13	excluded	absent	195.81
238080_at	excluded	absent	4.38	-0.62	excluded	absent	30.33	0.17	excluded	absent	2.85	-0.66	excluded	absent	52.46	0.85	excluded	absent	13.32
238081_at	included	absent	81.99	-0.96	included	absent	54.1	-1.18	included	present	77.86	-0.99	included	present	58.75	-1.14	included	absent	51.08
238082_at	excluded	absent	95.73	-0.18	excluded	absent	90.78	-0.21	excluded	absent	11.47	-0.74	excluded	absent	68.84	-0.36	excluded	absent	138.02
238083_at	excluded	absent	20.34	-0.42	excluded	absent	19.61	-0.44	excluded	absent	67.54	1.16	excluded	absent	38.42	0.18	excluded	absent	23.27
238084_at	included	present	136.8	-0.19	excluded	absent	108.39	-0.46	excluded	absent	125.59	-0.29	included	present	162.25	0.05	included	present	169.38
238085_at	excluded	absent	56.3	1.92	excluded	absent	76.13	2.92	excluded	absent	49.67	1.59	excluded	absent	61.46	2.18	excluded	absent	3.59
238086_at	excluded	absent	46.53	-0.41	excluded	absent	65.2	-0.2	excluded	absent	57.27	-0.29	excluded	absent	117.44	0.38	excluded	absent	37.25
238087_at	included	present	380.26	1.4	excluded	absent	307.1	0.74	excluded	absent	265.71	0.36	included	present	265.89	0.36	included	present	208.79
238088_at	excluded	absent	3.57	-0.81	excluded	absent	12.46	-0.67	excluded	absent	23.7	-0.5	excluded	absent	107.75	0.83	excluded	absent	8.6
238089_at	included	present	712.5	0.39	included	present	387.95	-0.35	included	present	738.62	0.45	included	present	503.43	-0.09	included	present	325.4
238090_at	included	present	437.45	-0.06	included	absent	121.94	-1.59	included	present	486.85	0.18	included	present	332.59	-0.57	included	present	439.96
238091_at	excluded	absent	58.96	-0.05	excluded	absent	2.87	-0.84	excluded	absent	2.79	-0.84	excluded	absent	11.89	-0.71	excluded	absent	94.43
238092_at	excluded	absent	82.97	-0.19	excluded	absent	114.02	0.3	excluded	absent	3.02	-1.46	excluded	absent	117.9	0.36	excluded	absent	10.29
238093_at	excluded	absent	79.57	-0.51	excluded	absent	94.84	-0.23	excluded	absent	99.09	-0.16	excluded	absent	169.31	1.11	excluded	absent	82.73
238094_at	excluded	absent	41.6	0.19	excluded	absent	44.6	0.29	excluded	absent	48.86	0.43	excluded	absent	40.65	0.16	excluded	absent	148.07
238095_at	excluded	absent	6.82	-0.58	excluded	absent	1.72	-0.66	excluded	absent	8.89	-0.55	excluded	absent	10.69	-0.52	excluded	absent	63.78
238096_at	included	present	761.12	0.06	included	present	1173.95	0.39	included	present	390.39	-0.25	included	present	998.11	0.25	included	present	749.67
238097_at	excluded	absent	120.51	0.61	excluded	absent	73.65	0.03	excluded	absent	2.76	-0.85	excluded	absent	3.37	-0.84	excluded	absent	6.55
238098_at	included	present	168.15	-0.85	included	present	325.82	0.09	included	present	516.1	1.24	included	present	479.34	1.02	included	present	207.1
238099_at	excluded	absent	5.26	-0.58	excluded	absent	3.77	-0.61	excluded	absent	2.17	-0.64	excluded	absent	2.97	-0.63	excluded	absent	76.45
238100_at	included	present	1008.65	-0.27	included	present	1232.84	-0.11	included	present	573.11	-0.58	included	present	176.35	-0.87	included	present	1018.64
238101_at	excluded	absent	6.54	-0.84	included	present	317.18	4.05	excluded	absent	40.04	-0.32	excluded	absent	53.75	-0.1	excluded	absent	80.65
238102_s_at	excluded	absent	81.33	-0.08	excluded	absent	16.98	-1.36	excluded	absent	20.72	-1.29	excluded	absent	53.28	-0.64	excluded	absent	118.63
238103_at	excluded	absent	28.69	-0.25	excluded	absent	21.3	-0.26	excluded	absent	59.3	-0.2	included	present	73.84	-0.18	excluded	absent	3.03
238104_at	excluded	absent	122.51	1.3	excluded	absent	87.56	0.57	excluded	absent	15.69	-0.93	excluded	absent	45.68	-0.31	excluded	absent	81.59
238105_x_at	excluded	absent	29.93	-0.26	excluded	absent	31.45	-0.22	excluded	absent	6.49	-0.85	excluded	absent	14.53	-0.65	excluded	absent	28.86
238106_at	included	present	122.46	0.05	excluded	absent	2.57	-0.93	excluded	absent	5.28	-0.91	included	present	526.38	3.34	excluded	absent	161.68
238107_at	included	present	109.68	-0.52	included	present	379.69	1.62	included	present	105.36	-0.55	included	present	214.63	0.31	included	absent	153.9
238108_at	excluded	absent	126.76	-0.61	excluded	absent	91.06	-1.06	excluded	absent	113.88	-0.77	excluded	absent	156.42	-0.23	excluded	absent	27.54
238109_at	included	present	887.39	0.48	included	present	884.9	0.47	included	present	1398.2	2.19	included	present	1076.6	1.11	included	present	2465.22
238110_at	excluded	absent	62.29	0.48	excluded	absent	52.37	0.24	excluded	absent	92.35	1.21	excluded	absent	44.99	0.06	excluded	absent	20.34
238111_at	excluded	absent	5.03	-0.71	excluded	absent	53.9	1.08	excluded	absent	5.82	-0.68	excluded	absent	10.26	-0.52	excluded	absent	5.59
238112_at	excluded	absent	8.88	-0.97	excluded	absent	52.22	-0.12	excluded	absent	13.61	-0.88	excluded	absent	3.78	-1.07	excluded	absent	50.57
238113_at	excluded	absent	4.65	-0.64	excluded	absent	23.4	-0.57	excluded	absent	19.69	-0.58	excluded	absent	6.49	-0.63	excluded	absent	153.16
238114_at	included	present	442.61	-1.01	included	present	296.75	-1.46	included	present	1054.55	0.86	included	present	743.65	-0.09	included	present	792.67
238115_at	included	present	776.97	0.01	included	present	525.59	-0.38	included	present	979.61	0.32	included	present	431.61	-0.52	included	present	649.8
238116_at	included	present	143.8	-0.27	included	absent	46.61	-0.3	included	absent	56.09	-0.3	included	present	67.73	-0.29	included	absent	97.36
238117_at	excluded	absent	22.98	-0.41	excluded	absent	108.51	1.93	excluded	absent	6.15	-0.87	excluded	absent	52.74	0.4	excluded	absent	33.33
238118_s_at	excluded	absent	2.54	-0.7	excluded	absent	44.52	0.9	excluded	absent	26.44	0.21	excluded	absent	3.83	-0.65	excluded	absent	130.75
238119_at	included	present	182.93	0.05	included	absent	93.43	-1.47	included	present	161.1	-0.32	included	absent	104.51	-1.28	included	absent	119.6
238120_at	excluded	absent	16.02	-0.62	excluded	absent	9.31	-0.79	excluded	absent	63.58	0.57	excluded	absent	4.96	-0.9	excluded	absent	39.91
238121_at	included	absent	94.18	-0.9	included	absent	107.53	-0.72	included	absent	76.29	-1.15	included	absent	78.5	-1.12	included	absent	145.81
238122_at	included	present	381.72	0.16	included	present	204.88	-1.06	included	present	705.36	2.38	included	present	754.17	2.72	included	present	300.86
238123_at	included	present	2608.22	2.85	included	present	471.6	0.17	excluded	absent	41.76	-0.37	excluded	absent	6.41	-0.41	excluded	absent	56.67
238124_at	excluded	absent	72.73	-0.23	excluded	absent	37.4	-0.25	excluded	absent	40.93	-0.25	excluded	absent	57.11	-0.24	excluded	absent	15.35
238125_at	included	present	171.92	1.75	excluded	absent	78.11	0.21	excluded	absent	5.2	-0.98	excluded	absent	13.17	-0.85	excluded	absent	71.98
238126_at	included	present	121.06	0.06	included	absent	76.88	-0.93	included	present	89.64	-0.64	included	present	134.07	0.35	included	absent	88.96
238127_at	included	present	316.42	-0.19	included	present	202.63	-0.53	included	present	180.05	-0.6	included	absent	103.39	-0.83	included	present	293.11
238128_at	excluded	absent	14.44	-0.23	excluded	absent	127.26	3.74	excluded	absent	8.88	-0.43	excluded	absent	4.9	-0.57	excluded	absent	8.1
238129_s_at	included	present	653.02	16.05	excluded	absent	15.76	-1.12	included	present	157.79	2.7	included	present	156.12	2.66	excluded	absent	15.37
238130_at	included	present	128.08	-0.35	included	present	388.82	2.3	included	present	318.29	1.59	included	present	163.48	0.01	included	present	288.27
238131_at	included	present	176.17	-0.56	included	present	696.56	2.45	included	present	71.03	-1.17	included	present	274.32	0.01	included	present	279.2
238132_at	excluded	absent	75.75	-0.38	excluded	absent	59.67	-0.57	excluded	absent	89.69	-0.21	excluded	absent	113.88	0.08	excluded	absent	331.68
238133_at	included	present	179.28	0.21	included	present	833.27	2.95	included	present	907.09	3.26	included	present	160.84	0.14	included	present	1657.53
238134_at	included	present	192.53	-1.01	included	present	1640.5	1.79	included	present	1205.67	0.95	included	present	784.08	0.13	included	present	3216.49
238135_at	included	present	117.68	0.72	excluded	absent	3.23	-1.59	excluded	absent	79.97	-0.04	excluded	absent	41.61	-0.82	excluded	absent	13.29
238136_at	excluded	absent	82.96	-0.62	included	present	341.42	-0.07	excluded	absent	44.24	-0.71	excluded	absent	84.95	-0.62	excluded	absent	34.45
238137_at	included	absent	70.55	-1.55	included	absent	60.65	-1.65	included	present	173.83	-0.47	included	present	164.29	-0.57	included	present	245.11

238138_at	excluded	absent	2.37	-0.85	excluded	absent	2	-0.86	excluded	absent	66.2	0.71	excluded	absent	66.37	0.71	excluded	absent	70.33
238139_at	excluded	absent	77.98	0.32	excluded	absent	70.33	0.19	excluded	absent	88.01	0.49	excluded	absent	4.62	-0.93	excluded	absent	142.13
238140_at	included	present	76.97	1.26	excluded	absent	2.07	-0.97	excluded	absent	4.57	-0.89	excluded	absent	1.62	-0.98	excluded	absent	65.73
238141_s_at	excluded	absent	71.68	0.12	excluded	absent	4.97	-0.73	excluded	absent	6.83	-0.71	included	present	131.96	0.89	excluded	absent	143.64
238142_at	excluded	absent	103.37	0.2	excluded	absent	71.7	-0.3	included	present	190.43	1.59	included	present	149.32	0.94	excluded	absent	77.61
238143_at	excluded	absent	2.71	-0.46	excluded	absent	11.51	-0.39	excluded	absent	2.22	-0.46	excluded	absent	0.86	-0.47	excluded	absent	3.64
238144_s_at	excluded	absent	47.1	-0.94	excluded	absent	167.04	0.64	excluded	absent	121.95	0.05	excluded	absent	96.78	-0.29	included	present	246.1
238145_at	included	present	263.28	1.23	included	present	281.2	1.5	included	present	243.71	0.95	included	present	320.7	2.07	included	present	231.28
238146_at	included	present	679.57	1.19	included	present	195.84	-1.94	included	present	365.16	-0.84	included	present	239.18	-1.66	included	present	317.74
238147_at	excluded	absent	44.32	-0.27	excluded	absent	143.24	1.45	excluded	absent	11.94	-0.83	excluded	absent	4.92	-0.96	excluded	absent	105.93
238148_s_at	excluded	absent	139.94	1.19	excluded	absent	47.42	-0.63	included	present	82.2	0.06	excluded	absent	59.44	-0.39	included	present	168.33
238149_at	included	absent	98.73	-0.27	included	present	256.32	2.11	included	present	84.93	-0.47	included	absent	35.62	-1.22	included	present	126.96
238150_at	excluded	absent	93.64	2.28	excluded	absent	15.76	-0.37	excluded	absent	10.67	-0.54	excluded	absent	86.92	2.05	excluded	absent	81.16
238151_at	included	present	1116.29	0.07	included	present	450.82	-1.01	included	present	771.51	-0.49	included	present	776	-0.48	included	present	758.53
238152_at	excluded	absent	12.87	-0.94	excluded	absent	3.34	-1.08	excluded	absent	45.26	-0.48	excluded	absent	35.84	-0.61	excluded	absent	173.11
238153_at	included	present	224.82	-0.46	included	present	1350.19	3.66	included	present	327.39	-0.08	included	present	555.24	0.75	included	present	247.4
238154_at	included	absent	205.51	-0.8	included	present	147.12	-0.91	included	present	363.26	-0.49	included	present	415.53	-0.39	included	present	337.75
238155_at	included	present	150.89	-0.63	included	absent	6.8	-1.62	included	present	228.31	-0.09	included	present	102.41	-0.96	included	absent	68.53
238156_at	included	present	734.19	1.05	included	present	339.92	-0.47	included	present	1886.57	5.51	included	present	881.48	1.62	included	present	473.72
238157_at	excluded	absent	64.17	-0.03	excluded	absent	6.3	-0.72	excluded	absent	58	-0.1	excluded	absent	61.95	-0.05	included	present	153.94
238158_at	excluded	absent	71.36	0.17	excluded	absent	47.03	-0.35	excluded	absent	38.63	-0.53	excluded	absent	16.66	-1	excluded	absent	4.47
238159_at	included	absent	140.47	-0.66	included	present	261.62	0.18	included	present	768.4	3.72	included	present	274.88	0.28	included	present	1040.27
238160_at	excluded	absent	33.66	-0.11	excluded	absent	5.89	-0.21	excluded	absent	62.06	-0.01	excluded	absent	34.05	-0.11	excluded	absent	85.58
238161_at	included	present	145.34	0.48	excluded	absent	130.13	0.2	excluded	absent	96.21	-0.42	excluded	absent	81.79	-0.68	excluded	absent	134.97
238162_at	excluded	absent	14.4	-0.78	excluded	absent	47.69	0.16	excluded	absent	5	-1.05	excluded	absent	40.65	-0.04	excluded	absent	27.29
238163_at	excluded	absent	10.07	-0.62	excluded	absent	46.78	0.3	excluded	absent	7.65	-0.68	excluded	absent	10.88	-0.6	excluded	absent	58.88
238164_at	included	present	215.98	-0.27	included	present	139.32	-0.7	included	present	159.17	-0.59	included	present	242.43	-0.12	included	absent	245.73
238165_at	excluded	absent	166.48	-0.14	excluded	absent	120.92	-0.39	excluded	absent	7.11	-1.01	included	present	152.97	-0.21	included	absent	116.62
238166_s_at	included	absent	139.73	-0.59	included	present	164.54	-0.42	included	absent	39.32	-1.29	included	absent	17.41	-1.44	included	absent	106.84
238167_at	excluded	absent	263.33	-0.14	excluded	absent	294.37	0.03	excluded	absent	101.95	-0.98	excluded	absent	30.96	-1.35	excluded	absent	73.25
238168_at	excluded	absent	71.39	0.25	excluded	absent	2.31	-0.75	excluded	absent	32.65	-0.31	excluded	absent	40.26	-0.2	excluded	absent	75.57
238169_at	excluded	absent	36.43	-0.63	excluded	absent	71.95	-0.26	excluded	absent	32.62	-0.67	excluded	absent	1.24	-0.99	excluded	absent	122.4
238170_at	included	present	388.56	-0.11	included	present	214.53	-0.94	included	present	571.01	0.76	included	present	448.94	0.18	included	present	676.74
238171_at	included	present	179.54	-0.64	included	absent	98.84	-1.18	included	present	143.48	-0.89	included	absent	38.71	-1.59	included	present	192.09
238172_at	included	present	307.83	-0.13	included	present	227.97	-0.61	included	present	202.44	-0.76	included	absent	131.96	-1.18	included	absent	248.58
238173_at	excluded	absent	55.02	-0.89	excluded	absent	61.03	-0.75	excluded	absent	70.58	-0.53	excluded	absent	72.87	-0.48	excluded	absent	62.96
238174_at	included	present	85.46	-0.1	excluded	absent	112.48	0.47	included	present	153.41	1.34	included	present	182.03	1.95	excluded	absent	176.88
238175_at	excluded	absent	3.28	-1.22	excluded	absent	92.3	0.25	excluded	absent	9.81	-1.11	excluded	absent	15.78	-1.01	excluded	absent	6.21
238176_at	included	present	187.76	1.09	excluded	absent	106.12	-0.1	excluded	absent	96.22	-0.24	excluded	absent	116.26	0.05	excluded	absent	26.77
238177_at	excluded	absent	58.24	-0.2	excluded	absent	23.65	-0.25	excluded	absent	124.41	-0.09	excluded	absent	6.07	-0.28	excluded	absent	79
238178_at	included	present	288.73	0.6	included	absent	104.31	-1.16	included	absent	38.91	-1.79	included	absent	46.36	-1.72	included	absent	67.49
238179_at	excluded	absent	94.63	-0.35	excluded	absent	60.63	-1.02	included	present	141.04	0.56	included	present	153.43	0.8	included	present	127.17
238180_at	excluded	absent	54.83	-0.82	excluded	absent	76.11	-0.56	excluded	absent	34.56	-1.06	excluded	absent	4.43	-1.42	excluded	absent	17.98
238181_at	excluded	absent	3.84	-1	excluded	absent	7.35	-0.91	excluded	absent	13.81	-0.76	excluded	absent	22.7	-0.55	excluded	absent	2.94
238182_at	excluded	absent	125.49	1.3	excluded	absent	74.93	0.07	excluded	absent	80.2	0.2	included	present	137.54	1.6	excluded	absent	30.25
238183_at	included	absent	337.55	-1.2	included	present	2386.99	3.77	included	present	917.93	0.21	included	absent	274.38	-1.35	included	absent	585.34
238184_at	excluded	absent	13.75	-0.53	excluded	absent	2.97	-0.78	excluded	absent	1.64	-0.81	excluded	absent	2.98	-0.78	excluded	absent	16.95
238185_at	included	present	489.69	0.25	included	present	237.91	-0.58	included	present	585.58	0.57	included	present	96.01	-1.05	included	present	442.27
238186_at	included	present	351.14	0.34	included	present	151.96	-0.87	included	present	1347.39	6.37	included	present	205.99	-0.54	included	present	356.87
238187_at	excluded	absent	12.95	-0.66	excluded	absent	36.88	-0.45	excluded	absent	40.17	-0.42	excluded	absent	60.95	-0.24	excluded	absent	8.29
238188_at	excluded	absent	48.92	-0.38	excluded	absent	97.03	0.43	excluded	absent	49.12	-0.37	included	present	65.74	-0.1	included	present	129.69
238189_at	included	present	384.22	-0.1	included	present	334.38	-0.39	included	present	263.74	-0.8	included	present	739.89	1.96	included	present	309.18
238190_at	included	present	374.92	1.34	included	present	526.6	2.99	included	present	223.89	-0.31	included	present	171.12	-0.89	included	present	417.18
238191_at	included	present	151.48	-0.68	included	present	180.45	-0.47	included	present	165.25	-0.58	included	absent	107.31	-0.99	included	present	137.05
238192_at	excluded	absent	46.01	0.06	excluded	absent	24.85	-0.44	excluded	absent	36.45	-0.17	excluded	absent	89.05	1.07	excluded	absent	17.94
238193_at	included	present	347.06	1.73	included	absent	109.91	-1.04	included	present	702.19	5.89	included	absent	9.02	-2.22	included	present	371.05
238194_at	excluded	absent	2.8	-0.35	included	present	492.04	1.28	excluded	absent	83.46	-0.08	excluded	absent	20.78	-0.29	included	present	1852.08
238195_at	excluded	absent	2.3	-0.86	excluded	absent	73.81	0.71	excluded	absent	2.36	-0.86	excluded	absent	16.09	-0.56	excluded	absent	7.28
238196_at	excluded	absent	48	-0.32	excluded	absent	133.68	1.39	excluded	absent	4.2	-1.19	excluded	absent	6.3	-1.15	excluded	absent	126.22
238197_at	excluded	absent	5.38	-0.69	excluded	absent	49.79	-0.14	excluded	absent	19.75	-0.52	excluded	absent	7.83	-0.66	excluded	absent	3.65
238198_at	excluded	absent	3.91	-1.17	excluded	absent	76.98	0.39	excluded	absent	13.39	-0.97	excluded	absent	10.78	-1.03	excluded	absent	7
238199_x_at	included	present	1890.79	-0.49	included	present	1168.85	-1.15	included	present	1509.26	-0.84	included	present	1668.29	-0.69	included	present	1964.25
238200_at	included	present	111.02	1.41	excluded	absent	10.61	-1.01	excluded	absent	45.58	-0.17	excluded	absent	36.09	-0.4	excluded	absent	100.6
238201_at	excluded	absent	72.14	0.31	excluded	absent	84.77	0.47	excluded	absent	14.7	-0.4	excluded	absent	7.92	-0.49	excluded	absent	7.34
238202_at	excluded	absent	21.46	-0.35	excluded	absent	25.56	-0.3	excluded	absent	41.31	-0.08	excluded	absent	5.32	-0.58	excluded	absent	61.73
238203_at	excluded	absent	33.21	0.01	excluded	absent	60.53	0.94	excluded	absent	25.99	-0.24	excluded	absent	34.63	0.06	excluded	absent	59.1
238204_at	excluded	absent	134.49	-0.14	excluded	absent	4.03	-0.78	excluded	absent	48.99	-0.56	excluded	absent	3.52	-0.79	excluded	absent	57.76
238205_at	excluded	absent	5.25	-0.38	excluded	absent	78.16	0.09	excluded	absent	2.68	-0.39	excluded	absent	2.02	-0.4	excluded	absent	173.63
238206_at	excluded	absent	2.75	-0.7	excluded	absent	8.55	-0.65	excluded	absent	2.33	-0.7	excluded	absent	1.93	-0.7	excluded	absent	2.71
238207_at	included	present	209.13	0.53	included	present	190.48	0.28	included	present	251.31	1.08	included	present	145.15	-0.31	included	present	188.59
238208_at	excluded	absent	39.68	-0.45	excluded	absent	14.38	-1.09	excluded	absent	41.45	-0.41	excluded	absent	4.21	-1.35	excluded	absent	129.66
238209_at	excluded	absent	5.74	-0.88	excluded	absent	3.55	-0.95	excluded	absent	6.68	-0.86	excluded	absent	4.43	-0.92	excluded	absent	5.08
238210_at	excluded	absent	59.34	-1.24	excluded	absent	83.49	-0.84	included	present	56.87	-1.28	excluded	absent	37.06	-1.61	excluded	absent	170.91
238211_at	excluded	absent	1.76	-0.42	excluded	absent	100.53	0.19	excluded	absent	32.02	-0.23	excluded	absent	69.02	0	excluded	absent	51.53
238212_at	included	present	170.74	0.42	included	present	426.95	2.42	excluded	absent	46.05	-0.55	included	present	250.88	1.05	excluded	absent	113.17
238213_at	excluded	absent	5.02	-0.78	excluded	absent	2.24	-0.87	excluded	absent	5.77	-0.76	excluded	absent	9.16	-0.66	excluded	absent	126.64
238214_at	included	present	267.25	-0.08	included	absent	157.16	-0.87	included	present	129.1	-1.07	included	present	189.72	-0.64	included	absent	83.81
238215_at	excluded	absent	92.55	1.26	excluded	absent	64.31	0.65	excluded	absent	13.28	-0.46	excluded	absent	32.86	-0.03	excluded	absent	94.63
238216_at	included	present	281.84	0.77	excluded	absent	129.14	-0.42	excluded	absent	216.44	0.26	excluded	absent	2.09	-1.41	excluded	absent	141.2
238217_at	excluded	absent	56.29	0	excluded	absent	85.63	0.35	excluded	absent	75.43	0.23	included	present	110.8	0.65	excluded	absent	8.41
238218_at	excluded	absent	59.64	0.05	excluded	absent	19.28	-0.25	excluded	absent	40.29	-0.09	excluded	absent	31.99	-0.15	excluded	absent	4.65
238219_at	excluded	absent	16.68	-0.2	excluded	absent	7.67	-0.21	excluded	absent	10.58	-0.21	excluded	absent	2.51	-0.22	excluded	absent	66.04

[illegible]

238497_at	included	present	501.63	-0.38	included	present	286.49	-0.87	included	present	1426.46	1.73	included	present	587.62	-0.19	included	absent	222.02	-1.02	[illegible]
238498_at	excluded	absent	121.16	-0.53	excluded	absent	92.83	-0.62	excluded	absent	252.33	-0.12	excluded	absent	159.76	-0.41	included	present	464.8	0.56	[illegible]
238499_at	excluded	absent	7.42	-0.63	excluded	absent	5.42	-0.67	excluded	absent	4.62	-0.68	excluded	absent	3.51	-0.7	excluded	absent	20.04	-0.42	[illegible]
238500_at	excluded	absent	74.45	-0.19	excluded	absent	38.13	-0.68	excluded	absent	19.68	-0.94	excluded	absent	47.61	-0.56	excluded	absent	139.38	0.69	[illegible]
238501_at	included	present	144.24	-0.14	included	present	278.99	1.76	included	present	346.57	2.72	included	present	234.74	1.14	excluded	absent	268.24	1.61	[illegible]
238502_at	included	present	133.71	-1.07	included	present	113.66	-1.19	included	present	356.17	0.2	included	present	253.04	-0.39	included	present	189.45	-0.75	[illegible]
238503_at	excluded	absent	92.11	-0.44	excluded	absent	53.83	-0.95	included	present	103.67	-0.28	included	present	111.81	-0.17	excluded	absent	2.73	-1.63	[illegible]
238504_at	included	present	133.26	-0.05	included	present	234.2	1.49	included	present	154.38	0.28	included	present	173.47	0.57	included	present	224.33	1.34	[illegible]
238505_at	included	present	369.25	2.49	included	present	163.72	0.24	included	present	176.88	0.39	included	absent	80.18	-0.67	included	present	204.99	0.7	[illegible]
238506_at	included	present	262.86	1.51	included	absent	86.37	-1.47	included	present	128.68	-0.75	included	present	468.49	4.99	included	present	141.04	-0.54	[illegible]
238507_at	excluded	absent	62.35	-0.1	included	present	130.97	1.57	excluded	absent	48.36	-0.44	included	present	73.65	0.17	excluded	absent	51.88	-0.36	[illegible]
238508_at	excluded	absent	138.95	0.83	included	present	94.18	0.18	excluded	absent	14.31	-0.97	excluded	absent	85.8	0.06	excluded	absent	42.75	-0.56	[illegible]
238509_at	included	present	618.43	-0.35	included	present	793.26	0.03	included	present	650.09	-0.28	included	present	603.25	-0.38	included	present	1010.48	0.5	[illegible]
238510_at	included	present	624.75	-1.1	included	present	1606.84	3.3	included	present	710.67	-0.72	included	present	687.1	-0.82	included	present	631.9	-1.07	[illegible]
238511_at	excluded	absent	96.21	0.21	included	present	207.96	2.12	included	present	135.18	0.88	included	present	132.29	0.83	excluded	absent	57.95	-0.44	[illegible]
238512_at	included	present	1097.55	0.32	included	present	334.15	-1.15	included	present	1607.72	1.29	included	present	742.2	-0.36	included	present	920.55	-0.02	[illegible]
238513_at	included	present	197.48	0.39	excluded	absent	7.76	-0.77	excluded	absent	50.9	-0.5	included	present	289.53	0.96	excluded	absent	1.35	-0.81	[illegible]
238514_at	excluded	absent	27.28	-1.29	included	present	205.68	1.17	included	present	168.28	0.66	excluded	absent	108	-0.17	excluded	absent	81.31	-0.54	[illegible]
238515_at	excluded	absent	148.84	0.2	excluded	absent	108.89	-0.39	excluded	absent	89.14	-0.68	excluded	absent	2.09	-1.97	excluded	absent	138.9	0.05	[illegible]
238516_at	included	present	2216.99	0.74	included	present	678	-1.07	included	present	1602.18	0.02	included	present	2675.76	1.28	included	present	1226.01	-0.42	[illegible]
238517_at	included	present	1348.52	3.04	included	present	1527.6	3.66	included	present	3083.93	9.09	included	present	630.54	0.53	included	present	1549.85	3.74	[illegible]
238518_x_at	excluded	absent	43.16	-0.36	excluded	absent	113.31	-0.07	excluded	absent	78.42	-0.21	excluded	absent	64.17	-0.27	excluded	absent	123.87	-0.03	[illegible]
238519_at	included	present	522.33	0.67	included	present	414.96	0.12	included	present	268.33	-0.63	included	present	348.78	-0.22	included	present	1068.28	3.46	[illegible]
238520_at	included	present	127.21	-0.04	included	present	219.32	1.4	included	present	113.28	-0.25	included	present	246.58	1.83	included	absent	10.33	-1.86	[illegible]
238521_at	included	present	348.53	-0.36	excluded	absent	15.47	-0.8	included	present	113.74	-0.67	included	present	631.79	0.02	excluded	absent	11.85	-0.81	[illegible]
238522_at	excluded	absent	335.74	0.02	excluded	absent	140.15	-0.68	excluded	absent	43.93	-1.02	excluded	absent	112.4	-0.78	excluded	absent	87.7	-0.87	[illegible]
238523_at	excluded	absent	193.68	-0.3	excluded	absent	156.9	-0.65	excluded	absent	94.48	-1.25	excluded	absent	172.66	-0.5	excluded	absent	180.52	-0.42	[illegible]
238524_at	included	present	158.85	-0.26	included	absent	98.82	-0.41	included	present	238.64	-0.05	included	present	367	0.28	included	present	294.97	0.1	[illegible]
238525_at	included	present	298.48	-0.14	excluded	absent	210.01	-0.87	included	present	331.47	0.13	included	present	436.83	1	included	present	269.68	-0.38	[illegible]
238526_at	included	present	159.79	-0.68	included	present	420.42	0.21	included	present	372.79	0.05	included	present	2107.99	5.96	included	present	255.31	-0.36	[illegible]
238527_at	excluded	absent	7.14	-0.86	excluded	absent	43.44	-0.35	excluded	absent	95.17	0.36	excluded	absent	15.98	-0.73	excluded	absent	179.83	1.54	[illegible]
238528_at	included	present	1087.64	1.62	included	present	1062.83	1.49	included	present	1002.74	1.16	included	present	656.47	-0.72	included	present	1093.6	1.66	[illegible]
238529_at	excluded	absent	131.55	1.66	excluded	absent	34.49	-0.36	excluded	absent	125.15	1.53	excluded	absent	166.95	2.39	excluded	absent	47.67	-0.08	[illegible]
238530_at	included	present	168.37	0.6	included	present	77.22	-1.21	included	present	290.86	3.04	included	present	238.25	1.99	included	present	132.37	-0.12	[illegible]
238531_x_at	excluded	absent	2.46	-0.77	excluded	absent	9.37	-0.56	excluded	absent	20.65	-0.22	excluded	absent	2.7	-0.76	excluded	absent	3.03	-0.75	[illegible]
238532_at	included	absent	86.36	-0.41	included	absent	96.02	-0.39	included	present	327.85	0.05	included	present	74.36	-0.44	included	present	364.66	0.12	[illegible]
238533_at	excluded	absent	8.71	-0.52	included	present	142.97	0.01	included	present	84.09	-0.22	included	present	268.79	0.52	excluded	absent	179.85	0.16	[illegible]
238534_at	excluded	absent	72.76	-0.47	excluded	absent	4.26	-1.28	excluded	absent	39.31	-0.87	excluded	absent	196.7	0.97	excluded	absent	125.45	0.14	[illegible]
238535_at	included	present	86.78	-0.4	included	present	85.55	-0.41	included	present	256.2	1.9	included	present	180.05	0.87	included	present	183.15	0.91	[illegible]
238536_at	included	present	992.96	0.18	included	present	298.13	-1.3	included	present	488.11	-0.9	included	present	733.44	-0.37	included	present	242.25	-1.42	[illegible]
238537_at	excluded	absent	0.69	-1.08	excluded	absent	1.94	-1.07	excluded	absent	1.91	-1.07	included	present	670.63	6.52	excluded	absent	36.03	-0.68	[illegible]
238538_at	included	present	317.77	-0.83	included	present	891.37	3.95	excluded	absent	4.18	-3.45	included	present	654.93	1.98	included	present	344.06	-0.61	[illegible]
238539_at	included	present	437.7	0.53	included	absent	69.4	-1.55	included	present	418.98	0.42	included	present	1173.67	4.7	included	present	325.23	-0.11	[illegible]
238540_at	included	present	601.26	0.83	included	present	203.99	-1.44	included	present	267.61	-1.07	included	present	414.52	-0.23	included	present	661.76	1.18	[illegible]
238541_at	excluded	absent	3.28	-0.62	excluded	absent	19.85	0.06	excluded	absent	2.55	-0.65	excluded	absent	8.16	-0.42	excluded	absent	73.17	2.24	[illegible]
238542_at	excluded	absent	22.72	-0.9	excluded	absent	41.35	-0.46	excluded	absent	74.26	0.32	included	present	133.15	1.72	excluded	absent	4.39	-1.33	[illegible]
238543_x_at	excluded	absent	63.05	0.08	excluded	absent	43.09	-0.28	excluded	absent	14.69	-0.79	excluded	absent	90.04	0.56	excluded	absent	26.93	-0.57	[illegible]
238544_at	excluded	absent	145.66	0.38	excluded	absent	17.63	-1.05	excluded	absent	56.9	-0.61	excluded	absent	51.67	-0.67	excluded	absent	158.97	0.53	[illegible]
238545_at	excluded	absent	24.83	-0.64	excluded	absent	40.15	-0.41	excluded	absent	38.76	-0.43	excluded	absent	61.01	-0.1	excluded	absent	114.93	0.71	[illegible]
238546_at	included	present	421.63	-0.55	included	present	700.49	-0.22	included	present	1282.47	0.49	included	present	213	-0.81	included	present	285.66	-0.72	[illegible]
238547_at	included	present	267.42	1.43	excluded	absent	388.65	2.93	included	present	151.75	-0.01	included	present	231.14	0.98	excluded	absent	125.32	-0.34	[illegible]
238548_at	included	present	667.29	3.34	included	present	243.12	-0.37	included	present	643.99	3.14	included	present	262.97	-0.2	included	present	522.6	2.07	[illegible]
238549_at	included	present	585.26	0.38	included	present	552.56	0.22	included	present	295.89	-1.01	included	present	2248.87	8.33	included	present	880.62	1.79	[illegible]
238550_at	included	present	231.92	0.42	included	present	127.68	-0.9	included	present	288.95	1.14	included	present	338.66	1.77	included	absent	168.8	-0.38	[illegible]
238551_at	included	present	580.86	1.26	included	present	300.5	-0.44	included	present	293.19	-0.49	included	present	811.49	2.67	included	present	270.62	-0.63	[illegible]
238552_at	included	present	293.99	-0.28	included	present	78.43	-1.76	included	present	220.41	-0.79	included	present	385.09	0.34	included	present	538.84	1.4	[illegible]
238553_at	included	present	479.78	-0.35	included	present	808.28	0.91	included	present	729.95	0.61	included	present	411.98	-0.61	included	present	226.76	-1.32	[illegible]
238554_at	included	present	163.65	-0.64	included	present	344.49	-0.25	included	present	116.25	-0.75	included	present	155.14	-0.66	included	absent	150.62	-0.67	[illegible]
238555_at	included	present	153.73	-1.19	included	present	462.35	0.95	included	present	307.22	-0.13	included	present	137.7	-1.3	included	present	451.07	0.87	[illegible]
238556_at	excluded	absent	39.88	-1.27	excluded	absent	21.17	-1.55	excluded	absent	2.46	-1.82	excluded	absent	131.92	0.1	included	present	165.5	0.6	[illegible]
238557_at	excluded	absent	73.26	-0.19	included	present	255.1	3.92	excluded	absent	48.67	-0.75	included	present	77.81	-0.09	excluded	absent	128.09	1.05	[illegible]
238558_at	included	present	1544.39	-0.57	included	present	412.73	-0.9	included	present	554.45	-0.85	included	present	533.64	-0.86	included	present	924.2	-0.75	[illegible]
238559_at	included	present	79.67	0.17	excluded	absent	44.39	-0.37	excluded	absent	1.71	-1.03	excluded	absent	2.76	-1.01	excluded	absent	7.82	-0.94	[illegible]
238560_at	included	present	274.64	-0.77	included	present	161.09	-1.41	included	present	190.53	-1.25	included	present	134.89	-1.56	included	present	325.21	-0.48	[illegible]
238561_s_at	included	absent	80.22	-1.12	included	present	132.15	-0.33	included	present	627.04	7.19	included	present	165.5	0.18	included	present	228.1	1.13	[illegible]
238562_at	included	present	173.62	-0.26	included	present	176.81	-0.21	included	present	745.24	7.89	included	present	281.3	1.27	included	present	188.6	-0.05	[illegible]
[illegible]																					

238856_s_at	included	present	736.94	1.34	included	present	358.53	-1.05	included	present	639.52	0.72	included	present	660.34	0.86	[illegible]
238857_at	excluded	absent	2.36	-0.85	excluded	absent	44.05	0.81	excluded	absent	4.55	-0.77	excluded	absent	75.85	2.07	[illegible]
238858_at	excluded	absent	73	-0.72	excluded	absent	82.86	-0.64	included	present	166.91	0	included	present	410.03	1.86	[illegible]
238859_at	included	present	1384.52	0.81	included	present	1261.97	0.48	included	present	2276.98	3.22	included	present	2063.88	2.64	[illegible]
238860_at	included	present	910.79	-0.25	included	present	3112.46	5.3	included	present	1182.89	0.44	included	present	1390.65	0.96	[illegible]
238861_at	included	present	233.36	-0.57	included	present	48.34	-1.52	included	present	331.9	-0.06	included	present	755.95	2.11	[illegible]
238862_at	included	present	135.72	-0.38	included	absent	13.42	-0.74	included	absent	3.91	-0.77	included	present	125.25	-0.41	[illegible]
238863_x_at	included	present	387.74	-0.91	included	present	881.24	1.58	included	present	1517.16	4.8	included	present	713.15	0.73	[illegible]
238864_at	excluded	absent	4.75	-0.97	excluded	absent	10.46	-0.9	excluded	absent	16.11	-0.84	excluded	absent	77.84	-0.19	[illegible]
238865_at	included	present	121.29	0.03	excluded	absent	62.13	-0.39	included	present	153.13	0.26	included	present	557.79	3.14	[illegible]
238866_at	included	present	1252.81	-0.03	included	present	3849.35	3.15	included	present	1243.61	-0.04	included	present	3184.13	2.33	[illegible]
238867_at	excluded	absent	86	-0.17	included	present	143.63	-0.03	included	present	105.94	-0.12	included	present	180.6	0.06	[illegible]
238868_at	included	present	423.19	1.16	included	present	87.59	-0.83	included	absent	112.25	-0.68	included	present	207.66	-0.11	[illegible]
238869_at	included	present	184.5	-0.8	included	absent	140.71	-0.97	included	present	213.74	-0.68	included	present	276.81	-0.44	[illegible]
238870_at	excluded	absent	50.66	0.28	excluded	absent	118.13	1.73	excluded	absent	2.73	-0.75	included	present	183.8	3.14	[illegible]
238871_at	included	present	1127.72	-0.25	included	present	1176.29	-0.19	included	present	1094.27	-0.29	included	present	1300.48	-0.04	[illegible]
238872_at	included	present	173.42	0.66	included	present	1308.13	12.76	included	present	152.52	0.44	included	present	213.73	1.09	[illegible]
238873_at	included	present	262.81	-0.44	included	present	378.21	0.09	included	present	283.85	-0.34	included	present	149.26	-0.95	[illegible]
238874_at	included	present	299.82	0.17	included	absent	76.92	-1.24	included	absent	51.67	-1.4	included	present	83.04	-1.2	[illegible]
238875_at	included	absent	158.43	-0.47	included	absent	23.63	-1.09	included	absent	137.23	-0.57	included	absent	13.37	-1.14	[illegible]
238876_at	excluded	absent	5.3	-0.21	excluded	absent	11.38	0.55	excluded	absent	5.92	-0.14	excluded	absent	33.03	3.26	[illegible]
238877_at	excluded	absent	37.32	-0.32	excluded	absent	43.46	-0.29	included	present	305.07	1.09	excluded	absent	1.47	-0.51	[illegible]
238878_at	included	present	93.69	-0.54	excluded	absent	39.26	-0.72	included	present	422.46	0.58	included	present	815.86	1.91	[illegible]
238879_at	included	present	215.14	-1.05	included	present	186.33	-1.4	included	present	223.85	-0.95	included	present	336.87	0.41	[illegible]
238880_at	included	present	301.34	-0.13	included	present	179.52	-0.98	included	present	209.36	-0.77	included	present	156.62	-1.14	[illegible]
238881_at	included	present	229.97	-0.68	included	present	432.48	0.83	included	present	320.36	-0.01	included	present	499.74	1.32	[illegible]
238882_at	included	present	458.01	-0.26	included	present	251.38	-0.55	included	present	244.16	-0.56	included	present	268.29	-0.53	[illegible]
238883_at	included	present	490.31	-0.08	included	absent	150.31	-1.32	included	present	193.27	-1.16	included	present	250.27	-0.95	[illegible]
238884_at	included	present	104.72	-0.57	included	absent	10.9	-1.49	included	present	117.99	-0.44	included	present	151.75	-0.11	[illegible]
238885_at	included	present	131.04	-0.42	included	present	2127.23	11.58	included	present	845.43	3.88	included	present	274.22	0.44	[illegible]
238886_at	included	present	198.15	-0.41	included	absent	148.3	-0.92	included	absent	128.84	-1.12	included	present	307.49	0.72	[illegible]
238887_at	included	present	197.84	0.32	included	present	287.06	1.39	included	present	129.87	-0.5	included	absent	146.06	-0.31	[illegible]
238888_at	included	present	210.67	0.5	included	absent	111.27	-1.01	included	absent	124.59	-0.81	included	present	229.44	0.78	[illegible]
238889_at	included	present	343.9	-0.28	included	present	729.22	1.02	included	present	263.23	-0.55	included	present	95.67	-1.12	[illegible]
238890_at	included	present	204.05	-0.54	included	present	275.13	-0.12	included	present	242.17	-0.31	included	present	373.87	0.45	[illegible]
238891_at	excluded	absent	65.7	-0.21	excluded	absent	7.72	-0.73	excluded	absent	13.12	-0.68	excluded	absent	65.42	-0.21	[illegible]
238892_at	included	present	436.95	0.62	included	present	164.21	-1.42	included	present	233.67	-0.9	included	present	383.18	0.22	[illegible]
238893_at	excluded	absent	167.63	-0.07	excluded	absent	66.81	-0.79	excluded	absent	12.18	-1.17	excluded	absent	3.34	-1.24	[illegible]
238894_at	included	present	302.64	1.24	included	absent	97.89	-0.76	included	present	93.37	-0.8	included	present	191.38	0.16	[illegible]
238895_at	included	present	241.25	-0.77	included	present	119.31	-1.06	included	present	542.44	-0.05	included	present	383.33	-0.43	[illegible]
238896_at	included	present	251.27	1.94	excluded	absent	214.85	1.38	included	present	254.59	2	excluded	absent	49.48	-1.2	[illegible]
238897_at	excluded	absent	58.78	0.12	excluded	absent	11.49	-1.24	excluded	absent	15.92	-1.11	excluded	absent	43.27	-0.32	[illegible]
[illegible]	[illegible]	[illegible]	[illegible]	[illegible]	[illegible]	[illegible]	[illegible]	[illegible]	[illegible]	[illegible]	[illegible]	[illegible]	[illegible]	[illegible]	[illegible]	[illegible]	[illegible]

239215_at	excluded	absent	74.5	0.01	excluded	absent	89.04	0.26	excluded	absent	78.12	0.07	excluded	absent	95.59	0.38
239216_at	excluded	absent	6.73	-0.56	excluded	absent	7.04	-0.56	excluded	absent	12.76	-0.51	excluded	absent	31.29	-0.36
239217_x_at	excluded	absent	38.79	-0.73	excluded	absent	114.03	-0.12	excluded	absent	92.03	-0.3	excluded	absent	66.09	-0.51
239218_at	included	present	445.28	-0.27	included	absent	1.99	-0.89	included	absent	3.38	-0.89	included	absent	54.2	-0.82
239219_at	excluded	absent	10.1	-0.53	excluded	absent	80.98	1.62	excluded	absent	163.15	4.11	excluded	absent	63.76	1.1
239220_at	excluded	absent	85.34	-0.6	excluded	absent	57.81	-0.71	excluded	absent	14.82	-0.89	excluded	absent	6.7	-0.92
239221_at	excluded	absent	48.65	-0.54	excluded	absent	93.34	-0.08	excluded	absent	58.68	-0.44	excluded	absent	89.26	-0.12
239222_at	excluded	absent	78.67	0.59	excluded	absent	85.51	0.77	excluded	absent	78.18	0.58	excluded	absent	13.86	-1.03
239223_s_at	included	present	838.62	0.42	included	present	793.18	0.21	included	present	1205.63	2.16	included	present	853.95	0.49
239224_at	included	absent	175.14	-0.25	included	absent	43.94	-2.42	included	present	302.65	1.86	included	absent	117.61	-1.2
239225_at	included	present	177.48	2.47	included	present	138	1.42	excluded	absent	106.42	0.58	excluded	absent	178.45	2.5
239226_at	excluded	absent	125.94	0.53	excluded	absent	78.85	-0.02	excluded	absent	2.4	-0.91	excluded	absent	90.62	0.12
239227_at	included	present	115.65	-0.45	included	absent	51.03	-1.02	included	absent	96.85	-0.61	included	absent	100.28	-0.58
239228_at	included	present	165.49	0.63	included	present	102.62	-0.69	included	present	116.08	-0.41	included	present	123.31	-0.25
239229_at	excluded	absent	7.16	-0.78	included	present	76.88	-0.35	excluded	absent	11.42	-0.75	excluded	absent	21.32	-0.69
239230_at	included	present	107.34	0.47	excluded	absent	10.62	-0.97	excluded	absent	55.11	-0.31	excluded	absent	7.56	-1.02
239231_at	included	present	294.21	0.21	included	absent	156	-0.48	included	present	397.14	0.71	included	present	502.75	1.23
239232_at	included	absent	52.9	-0.94	included	absent	128.8	-0.46	included	present	160.63	-0.26	included	present	300.85	0.62
239233_at	included	present	76.84	0.43	excluded	absent	75.53	0.41	excluded	absent	42.86	-0.21	included	present	208.29	2.92
239234_at	included	present	420.15	-0.72	included	present	280.41	-1.18	included	present	312.29	-1.07	included	present	455.44	-0.61
239235_at	excluded	absent	39.39	0.22	excluded	absent	3.86	-0.62	excluded	absent	74.18	1.05	excluded	absent	4.1	-0.62
239236_at	included	present	359.33	0.87	included	present	139.97	-1.32	included	present	259.13	-0.13	included	present	300.47	0.28
239237_at	included	present	150.42	-0.09	excluded	absent	2.2	-0.41	excluded	absent	2.37	-0.41	excluded	absent	26.27	-0.36
239238_at	included	present	672.03	-0.56	included	present	830.07	-0.32	included	present	2414.84	2.08	included	present	1203.35	0.24
239239_at	excluded	absent	33.5	-0.11	excluded	absent	106.72	1.72	excluded	absent	38.74	0.02	excluded	absent	61.9	0.6
239240_at	included	present	242.54	0.66	included	absent	40.61	-1.45	included	present	222.64	0.46	included	present	290.15	1.16
239241_at	included	present	101.33	-0.82	included	absent	4.16	-1.43	included	absent	34.15	-1.24	included	present	248.61	0.11
239242_at	excluded	absent	88.88	-0.3	included	present	152.16	0.8	included	present	81.18	-0.43	excluded	absent	65.71	-0.7
239243_at	included	present	141.11	-0.89	included	absent	1.82	-2.22	included	present	276.11	0.4	included	present	169.02	-0.62
239244_at	excluded	absent	40.67	-0.17	excluded	absent	80.48	0.14	excluded	absent	72.75	0.08	excluded	absent	9.49	-0.42
239245_at	included	present	278.42	-0.79	included	absent	90.73	-1.74	included	present	283.48	-0.76	included	present	211.4	-1.13
239246_at	included	present	197.12	-0.89	included	present	899.55	0.63	included	present	604.31	-0.01	included	present	711.99	0.23
239247_at	included	present	174.24	0.53	excluded	absent	86.82	-0.34	excluded	absent	105.56	-0.16	included	present	295.7	1.74
239248_at	included	present	406.69	0.81	included	present	176.31	-1.08	included	present	166.07	-1.17	included	present	206.14	-0.84
239249_at	included	present	262.67	-0.21	excluded	absent	80.12	-0.35	excluded	absent	91.27	-0.34	excluded	absent	174.2	-0.28
239250_at	included	present	306.09	-0.36	included	present	242.94	-0.7	included	present	316.7	-0.3	included	present	923.97	3.05
239251_at	included	present	201.82	-0.11	included	absent	21.2	-1.12	included	absent	71.5	-0.84	included	absent	78.85	-0.8
239252_at	included	present	1314.59	-0.06	included	present	254.82	-1	included	present	1984.8	0.53	included	present	776.01	-0.54
239253_at	excluded	absent	68.83	2.38	excluded	absent	35.78	0.85	excluded	absent	91.61	3.44	included	present	101.41	3.89
239254_at	excluded	absent	60.67	0.17	excluded	absent	156.32	2.74	excluded	absent	9.26	-1.21	excluded	absent	15.3	-1.04
239255_at	excluded	absent	39.57	-0.57	included	present	111.83	1.22	excluded	absent	66.79	0.1	excluded	absent	41.17	-0.53
239256_at	excluded	absent	42.49	-0.09	excluded	absent	64.68	0.48	excluded	absent	29.77	-0.42	excluded	absent	29.9	-0.41

[illegible]

239933_x_at	excluded	absent	58.47	5.69	excluded	absent	74.95	7.87	excluded	absent	6.89	-1.13	[illegible]
239934_x_at	excluded	absent	49.33	-0.39	excluded	absent	51.02	-0.38	excluded	absent	52.74	-0.37	[illegible]
239935_at	excluded	absent	14.18	-0.65	excluded	absent	88.24	0.83	excluded	absent	35.47	-0.22	[illegible]
239936_at	excluded	absent	120.49	0.46	excluded	absent	48.77	-0.62	included	present	120.85	0.47	[illegible]
239937_at	included	absent	140.08	-0.63	included	present	143.12	-0.61	included	present	197.13	-0.22	[illegible]
239938_x_at	excluded	absent	90.62	-0.1	excluded	absent	6.21	-1.32	excluded	absent	5.56	-1.33	[illegible]
239939_at	excluded	absent	46.85	-0.62	excluded	absent	21.52	-0.8	excluded	absent	110.62	-0.18	[illegible]
239940_at	excluded	absent	74.37	0.14	excluded	absent	13.39	-1.1	excluded	absent	15.16	-1.06	[illegible]
239941_at	excluded	absent	40.26	-0.26	excluded	absent	13	-0.81	excluded	absent	56.1	0.06	[illegible]
239942_at	excluded	absent	42.88	-0.17	excluded	absent	3.77	-1.03	excluded	absent	3.26	-1.05	[illegible]
239943_x_at	included	present	283.7	-0.55	included	present	277.24	-0.59	included	present	535.22	0.89	[illegible]
239944_at	included	present	1372.76	-0.09	included	present	899.3	-0.78	included	present	3630.96	3.2	[illegible]
239945_at	excluded	absent	16.44	-0.07	excluded	absent	57.24	1.37	excluded	absent	5.77	-0.44	[illegible]
239946_at	included	absent	81.77	-0.38	included	present	121.29	-0.27	included	present	243.79	0.06	[illegible]
239947_at	excluded	absent	82.76	0.2	excluded	absent	88.32	0.31	included	present	230.19	3	[illegible]
239948_at	excluded	absent	76.44	-0.15	excluded	absent	48.63	-0.69	excluded	absent	6.63	-1.49	[illegible]
239949_at	excluded	absent	1.49	-1.13	excluded	absent	91.13	0.89	excluded	absent	63.75	0.27	[illegible]
239950_at	excluded	absent	55.28	0.27	excluded	absent	2.43	-0.63	excluded	absent	21.15	-0.31	[illegible]
239951_at	included	absent	94.16	-0.55	included	absent	51.57	-1.11	included	present	152.13	0.23	[illegible]
239952_at	excluded	absent	70.49	-0.36	excluded	absent	123.22	0.46	excluded	absent	105.03	0.17	[illegible]
239953_at	excluded	absent	81.76	0.21	excluded	absent	3.88	-1.13	excluded	absent	47.39	-0.39	[illegible]
239954_at	included	present	181.51	0.4	included	present	81.5	-0.7	included	present	133.64	-0.12	[illegible]
239955_at	included	present	164.72	-0.23	excluded	absent	5.25	-1.31	excluded	absent	14.32	-1.25	[illegible]
239956_at	excluded	absent	61.08	0.3	excluded	absent	7.95	-1.12	excluded	absent	2.85	-1.26	[illegible]
239957_at	included	present	602.62	-0.27	included	present	239.74	-1.51	included	present	621.53	-0.21	[illegible]
239958_at	excluded	absent	52.19	-0.38	excluded	absent	7.02	-1.45	included	present	212.31	3.39	[illegible]
239959_x_at	excluded	absent	615.22	0.13	excluded	absent	544.3	-0.22	excluded	absent	556.9	-0.16	[illegible]
239960_x_at	included	present	1703.86	0.72	included	present	523.99	-1.58	included	present	1694.02	0.7	[illegible]
239961_at	excluded	absent	30.76	-0.15	excluded	absent	80.94	1.42	excluded	absent	3.69	-0.99	[illegible]
239962_at	included	present	101.15	-0.65	excluded	absent	7.69	-2.06	included	present	128.58	-0.24	[illegible]
239963_at	included	present	460.91	-0.19	included	present	124.53	-1.72	included	present	141.19	-1.64	[illegible]
239964_at	excluded	absent	50.59	1.7	excluded	absent	3.5	-0.4	excluded	absent	1.93	-0.47	[illegible]
239965_at	included	absent	118.03	-0.89	included	absent	113.63	-0.91	included	present	200	-0.52	[illegible]
239966_at	included	present	210.36	0	included	absent	51.11	-0.93	included	absent	3.45	-1.2	[illegible]
239967_at	included	present	120.29	0.05	excluded	absent	69.31	-0.64	excluded	absent	44.87	-0.96	[illegible]
239968_at	excluded	absent	4.08	-0.81	excluded	absent	5.22	-0.77	excluded	absent	3.1	-0.84	[illegible]
239969_at	included	present	185.7	-0.25	excluded	absent	92.26	-1.29	excluded	absent	290.35	0.92	[illegible]
239970_at	excluded	absent	72.62	0.36	excluded	absent	6.65	-1.07	excluded	absent	38.18	-0.39	[illegible]
239971_at	excluded	absent	89.22	0.17	excluded	absent	74.41	-0.06	excluded	absent	40.23	-0.61	[illegible]
239972_at	excluded	absent	35.06	-0.2	excluded	absent	5.15	-0.28	excluded	absent	18.45	-0.24	[illegible]
239973_at	included	present	437.22	1.78	included	absent	48.49	-0.81	included	present	193.73	0.16	[illegible]
239974_at	excluded	absent	7.42	-0.97	excluded	absent	12.83	-0.88	included	present	134.48	1.19	[illegible]
[illegible]	[illegible]	[illegible]	[illegible]	[illegible]	[illegible]	[illegible]	[illegible]	[illegible]	[illegible]	[illegible]	[illegible]	[illegible]	[illegible]

240292_x_at	excluded	absent	69.58	-0.7	excluded	absent	78.86	-0.69	excluded	absent	3.47	-0.76	excluded	absent	27.57	-0.73	excluded	absent	19.78	-0.74
240293_at	excluded	absent	116.68	-0.04	excluded	absent	111.85	-0.12	excluded	absent	37.43	-1.37	excluded	absent	89.88	-0.49	excluded	absent	137.97	0.32
240294_at	excluded	absent	76.94	-0.6	included	present	129.42	-0.17	excluded	absent	50.75	-0.82	included	present	89.65	-0.5	excluded	absent	56.55	-0.77
240295_at	included	present	150.53	1.31	excluded	absent	76.65	0.19	included	present	165.04	1.53	excluded	absent	24.15	-0.61	excluded	absent	221.26	2.39
240296_at	included	present	195.72	-0.14	included	absent	42.87	-1.63	included	present	242.94	0.32	included	present	248.56	0.37	excluded	absent	211.86	0.02
240297_at	included	present	261.83	1.03	included	absent	42.91	-1.12	included	present	155.77	-0.01	included	present	187.78	0.3	included	present	378.45	2.17
240298_at	excluded	absent	4.01	-1.01	excluded	absent	17.11	-0.74	excluded	absent	6.75	-0.95	excluded	absent	6.05	-0.96	excluded	absent	84.76	0.61
240299_at	excluded	absent	2.4	-0.23	included	present	223.47	0.01	excluded	absent	3.11	-0.23	excluded	absent	2.13	-0.23	excluded	absent	12.73	-0.22
240300_at	excluded	absent	104.69	-0.46	excluded	absent	109.25	-0.4	excluded	absent	8.42	-1.8	included	present	90.13	-0.67	excluded	absent	113.95	-0.34
240301_at	excluded	absent	35.94	0.06	excluded	absent	2.82	-0.38	excluded	absent	2.26	-0.39	excluded	absent	24.42	-0.1	excluded	absent	4.03	-0.37
240302_at	excluded	absent	112.94	-0.13	excluded	absent	76.79	-0.74	excluded	absent	63.34	-0.97	included	present	128.67	0.13	excluded	absent	142.63	0.37
240303_at	excluded	absent	20.57	-0.29	excluded	absent	6.25	-0.32	excluded	absent	39.2	-0.24	included	present	789.36	1.7	excluded	absent	34.82	-0.25
240304_s_at	excluded	absent	8.37	-0.27	excluded	absent	2.86	-0.27	excluded	absent	12.91	-0.27	included	present	3017.87	1.44	excluded	absent	7.32	-0.27
240305_at	excluded	absent	218.25	0.43	excluded	absent	18.3	-1.14	excluded	absent	94.64	-0.54	excluded	absent	138.47	-0.2	excluded	absent	144.25	-0.15
240306_at	excluded	absent	82.74	-0.37	excluded	absent	19.7	-0.86	excluded	absent	61.55	-0.54	excluded	absent	222.89	0.71	excluded	absent	66.86	-0.49
240307_at	included	present	445.95	-0.36	included	present	247.32	-0.84	included	present	431.57	-0.39	included	present	457.61	-0.33	included	present	760.99	0.4
240308_at	excluded	absent	104.37	1.2	excluded	absent	40.64	-0.3	excluded	absent	64.64	0.27	excluded	absent	37.02	-0.38	excluded	absent	8.66	-1.05
240309_at	included	absent	137.36	-0.65	included	absent	122.56	-0.72	included	present	155.17	-0.57	included	present	235.27	-0.19	included	present	191.23	-0.4
240310_at	included	present	137.18	-0.45	excluded	absent	83	-0.89	included	present	307.75	0.92	included	present	305.08	0.9	excluded	absent	138.7	-0.44
240311_at	excluded	absent	132.59	1.94	excluded	absent	5.91	-1.02	excluded	absent	84.83	0.83	excluded	absent	54.53	0.12	excluded	absent	16.95	-0.76
240312_at	included	present	147.27	1.11	excluded	absent	15.88	-0.56	excluded	absent	21.2	-0.5	excluded	absent	2.08	-0.74	excluded	absent	12.83	-0.6
240313_at	excluded	absent	1.97	-0.27	excluded	absent	51.95	-0.16	excluded	absent	4.68	-0.27	excluded	absent	2.15	-0.27	excluded	absent	87.95	-0.08
240314_at	included	present	136.98	-0.54	included	present	121.51	-0.73	included	present	193.73	0.16	included	present	142.84	-0.47	excluded	absent	261.34	0.99
240315_at	excluded	absent	6.05	-1.34	excluded	absent	50.63	-0.25	excluded	absent	19.99	-1	excluded	absent	34.92	-0.63	excluded	absent	34.9	-0.63
240316_at	excluded	absent	14.01	-0.29	excluded	absent	8.88	-0.31	excluded	absent	30.31	-0.21	excluded	absent	3.92	-0.34	excluded	absent	94.45	0.1
240317_at	included	present	413.13	0.22	included	present	137.27	-0.93	included	present	705.17	1.43	included	present	981.11	2.58	included	absent	90.66	-1.13
240318_at	excluded	absent	20.24	-0.93	excluded	absent	87.76	0.13	excluded	absent	35.52	-0.69	excluded	absent	37.61	-0.66	excluded	absent	40.65	-0.61
240319_at	excluded	absent	10.23	-0.92	included	present	192.27	2.9	excluded	absent	9.37	-0.94	excluded	absent	30.63	-0.49	excluded	absent	127.66	1.54
240320_at	included	present	173.93	0.25	excluded	absent	45.04	-0.67	excluded	absent	84.33	-0.39	excluded	absent	92.76	-0.33	excluded	absent	97.32	-0.3
240321_at	included	present	426.57	2.22	included	present	144.7	-0.56	included	present	328.2	1.25	included	absent	81.45	-1.18	included	present	397.24	1.93
240322_at	excluded	absent	32.12	0.2	excluded	absent	68.96	1.33	excluded	absent	37.4	0.36	excluded	absent	66.28	1.25	excluded	absent	7.66	-0.56
240323_at	excluded	absent	94.2	-0.41	excluded	absent	144.13	-0.01	included	present	266.47	0.98	included	present	279.16	1.08	excluded	absent	209	0.52
240324_at	included	present	125.37	0.15	excluded	absent	22.84	-1.47	included	present	302.55	2.96	included	present	182.19	1.05	included	present	373.96	4.09
240325_x_at	excluded	absent	11.31	-1.09	excluded	absent	38.3	-0.34	excluded	absent	6.65	-1.22	excluded	absent	6.83	-1.22	excluded	absent	141.48	2.53

240651_at	excluded	absent	4.91	-1.06	excluded	absent	127.39	0.63	included	present	76.07	[illegible]	[illegible]
240652_at	included	present	395.61	0.99	included	present	81.94	-0.91	included	present	87.97	[illegible]	[illegible]
240653_at	excluded	absent	2.19	-0.69	excluded	absent	7.48	-0.43	excluded	absent	40.36	[illegible]	[illegible]
240654_at	excluded	absent	73.43	1.44	excluded	absent	5.63	-0.8	excluded	absent	4.17	[illegible]	[illegible]
240655_at	included	absent	114.89	-0.43	included	absent	107.84	-0.48	included	absent	38.58	[illegible]	[illegible]
240656_at	excluded	absent	70.67	-0.21	excluded	absent	20.74	-1.03	excluded	absent	6.45	[illegible]	[illegible]
240657_at	excluded	absent	43.57	-0.24	excluded	absent	46.28	-0.18	excluded	absent	76.92	[illegible]	[illegible]
240658_at	excluded	absent	2.11	-0.37	excluded	absent	39.23	-0.07	excluded	absent	7.67	[illegible]	[illegible]
240659_x_at	excluded	absent	38.32	0.18	excluded	absent	18.09	-0.55	excluded	absent	66.43	[illegible]	[illegible]
240660_at	excluded	absent	40.01	-0.18	excluded	absent	32.69	-0.34	excluded	absent	33.26	[illegible]	[illegible]
240661_at	excluded	absent	12.28	-0.72	excluded	absent	10	-0.77	excluded	absent	16.15	[illegible]	[illegible]
240662_at	excluded	absent	77.52	0.41	excluded	absent	22.3	-0.45	excluded	absent	2.68	[illegible]	[illegible]
240663_at	excluded	absent	102.85	0.36	excluded	absent	38.87	-0.8	excluded	absent	120.54	[illegible]	[illegible]
240664_at	excluded	absent	82.98	0.86	excluded	absent	15.07	-0.68	excluded	absent	4.11	[illegible]	[illegible]
240665_at	included	present	274.52	0.05	included	absent	25.41	-1.11	included	absent	35.93	[illegible]	[illegible]
240666_at	excluded	absent	9.97	-0.64	excluded	absent	40.4	0.46	excluded	absent	5.16	[illegible]	[illegible]
240667_at	excluded	absent	40.28	-0.36	excluded	absent	62	0.14	excluded	absent	33.39	[illegible]	[illegible]
240668_s_at	excluded	absent	133.89	0.08	included	present	108.09	-0.26	excluded	absent	9.2	[illegible]	[illegible]
240669_at	excluded	absent	46.53	-0.25	excluded	absent	116.17	1.27	excluded	absent	119.39	[illegible]	[illegible]
240670_at	excluded	absent	3.14	-0.81	excluded	absent	1.67	-0.85	excluded	absent	32.33	[illegible]	[illegible]
240671_at	included	present	173.42	0.56	excluded	absent	79.18	-0.37	excluded	absent	4.2	[illegible]	[illegible]
240672_at	excluded	absent	30.16	-0.2	excluded	absent	47.6	-0.14	excluded	absent	8.63	[illegible]	[illegible]
240673_at	included	present	149.2	0.15	excluded	absent	3.26	-1.96	excluded	absent	77.69	[illegible]	[illegible]
240674_at	excluded	absent	84.08	0.64	included	present	92.72	0.87	excluded	absent	56.32	[illegible]	[illegible]
240675_at	excluded	absent	42.42	0.39	excluded	absent	3.44	-0.86	excluded	absent	18.36	[illegible]	[illegible]
240676_at	included	present	166.25	0.5	excluded	absent	60.28	-0.66	included	present	251.46	[illegible]	[illegible]
240677_at	excluded	absent	121.84	0.68	included	present	213.36	2.22	included	present	168.58	[illegible]	[illegible]
240678_at	excluded	absent	35.66	0.45	excluded	absent	2.28	-0.56	excluded	absent	3.85	[illegible]	[illegible]
240679_at	excluded	absent	22.66	0.29	excluded	absent	4.58	-0.41	excluded	absent	2.15	[illegible]	[illegible]
240680_at	excluded	absent	9.98	-0.89	excluded	absent	61.76	0.43	excluded	absent	5.65	[illegible]	[illegible]
240681_at	excluded	absent	1.37	-0.96	excluded	absent	3.25	-0.91	excluded	absent	63.32	[illegible]	[illegible]
240682_at	excluded	absent	1.87	-0.65	excluded	absent	13.93	-0.44	excluded	absent	39.73	[illegible]	[illegible]
240683_at	included	present	237.97	-0.35	included	absent	86.49	-1.23	included	present	199.23	[illegible]	[illegible]
240684_at	excluded	absent	78.43	1.58	excluded	absent	15.54	-0.68	included	present	76.4	[illegible]	[illegible]

241010_x_at	excluded	absent	142.11	-0.44	excluded	absent	108.4	-0.6	excluded	absent	140.62	-0.44	excluded	absent	239.34	0.04	excluded	absent	192.9	-0.19	excluded	absent	208.79	-0.11	excluded	absent	115.87	-0.56	excluded	absent	83.27	-0.72	excluded	absent	240.1	0.04	excluded	absent	57.83	-0.85	excluded	absent	195.22	-0.18	excluded	absent	12.13	-1.07	excluded	absent	81.88	-0.73	excluded	absent	9.72	-1.08	excluded	absent	93.67	-0.67
241011_at	included	present	119.73	1.85	excluded	absent	3.73	-1.33	excluded	absent	97.29	1.23	included	present	204.49	4.17	excluded	absent	7.28	-1.23	included	present	152.02	2.73	excluded	absent	82.89	0.84	excluded	absent	48.76	-0.09	included	present	84.35	0.88	included	present	51.88	-0.01	included	present	160.49	2.96	included	present	80	0.76	included	present	122.13	1.91	included	present	77.39	0.69	excluded	absent	36.06	-0.44
241012_at	excluded	absent	5.1	-0.69	excluded	absent	4.2	-0.71	excluded	absent	1.39	-0.8	excluded	absent	3.68	-0.73	excluded	absent	83.84	1.65	excluded	absent	65.55	1.11	excluded	absent	4.09	-0.72	excluded	absent	21.49	-0.2	excluded	absent	14.7	-0.4	excluded	absent	2.79	-0.76	excluded	absent	1.87	-0.78	excluded	absent	5.73	-0.67	excluded	absent	48.14	0.59	excluded	absent	6.53	-0.64	excluded	absent	6.08	-0.66
241013_at	excluded	absent	62.9	-0.06	excluded	absent	72.51	0.08	excluded	absent	4.8	-0.91	excluded	absent	2.22	-0.94	excluded	absent	14.36	-0.77	excluded	absent	24.16	-0.63	excluded	absent	16.1	-0.74	excluded	absent	49.31	-0.26	excluded	absent	17.41	-0.72	excluded	absent	11.47	-0.81	excluded	absent	29.73	-0.54	included	present	98.78	0.46	excluded	absent	28.2	-0.57	excluded	absent	2.26	-0.94	excluded	absent	27.94	-0.57
241014_at	included	present	250.55	1.28	included	present	255.29	1.33	excluded	absent	1.63	-1.45	excluded	absent	3.87	-1.43	excluded	absent	52.84	-0.89	included	present	256.79	1.35	excluded	absent	45.06	-0.98	included	present	951.28	8.96	included	present	132.1	-0.02	excluded	absent	58.85	-0.83	excluded	absent	33.69	-1.1	excluded	absent	65.77	-0.75	included	present	161.77	0.3	included	present	71.22	-0.69	excluded	absent	10.07	-1.36
241015_at	included	present	11781.26	2.06	included	present	2953.86	-0.77	included	present	9658.48	1.38	included	present	769.51	-1.47	included	present	9477.82	1.33	included	present	2936.69	-0.77	included	present	3779.95	-0.5	included	present	4182.42	-0.37	included	present	4930.88	-0.13	included	present	5914.02	0.18	included	present	3040.75	-0.74	included	present	2731.06	-0.84	included	present	9610.23	1.37	included	present	5090.96	-0.08	included	present	1977.81	-1.08
241016_at	included	present	262.24	-0.01	included	present	119.54	-1.29	included	present	119.61	-1.29	included	present	155.57	-0.97	included	present	281.7	0.17	included	present	204.48	-0.53	included	present	109.02	-1.38	included	absent	58.72	-1.83	included	present	208.54	-0.49	included	present	79.1	-1.65	included	present	175.36	-0.79	included	present	65.89	-1.77	included	present	116.32	-1.32	included	present	63.63	-1.79	included	present	57.28	-1.85
241017_at	included	present	207.69	0.86	excluded	absent	62.32	-0.76	included	present	166.54	0.4	included	present	114.02	-0.18	excluded	absent	107.47	-0.26	excluded	absent	83.18	-0.53	included	present	179.65	0.55	excluded	absent	112.69	-0.2	included	present	124.25	-0.07	included	present	163.07	0.36	included	present	185.2	0.61	included	present	260.19	1.45	included	present	206.44	0.85	included	present	578.46	5.01	included	present	183.95	0.6
241018_at	excluded	absent	51.32	-0.01	excluded	absent	38.73	-0.31	excluded	absent	47.7	-0.1	excluded	absent	5.17	-1.13	excluded	absent	177.62	3.07	excluded	absent	72.64	0.51	excluded	absent	31.1	-0.5	included	present	43.74	-0.19	excluded	absent	30.67	-0.51	excluded	absent	30.23	-0.52	excluded	absent	43.85	-0.19	excluded	absent	43.75	-0.19	excluded	absent	33.09	-0.45	excluded	absent	19.69	-0.78	included	present	44.13	-0.18
241019_at	excluded	absent	20.74	-1.43	excluded	absent	86.07	0.39	excluded	absent	46.55	-0.71	included	present	100.77	0.79	excluded	absent	73.97	0.05	excluded	absent	111.6	1.1	excluded	absent	21.01	-1.42	excluded	absent	21.77	-1.4	included	present	51.09	-0.58	excluded	absent	54.61	-0.49	excluded	absent	60.96	-0.31	excluded	absent	71.77	-0.01	excluded	absent	43.47	-0.8	excluded	absent	41.96	-0.84	excluded	absent	29.39	-1.19
241020_at	excluded	absent	168.91	-0.82	excluded	absent	177.14	-0.78	excluded	absent	84.56	-1.23	excluded	absent	252.81	-0.42	excluded	absent	193.74	-0.7	excluded	absent	225.05	-0.55	excluded	absent	175.45	-0.79	excluded	absent	66.21	-1.32	excluded	absent	136.18	-0.98	included	present	187.33	-0.73	excluded	absent	303.46	-0.17	excluded	absent	153.64	-0.9	included	present	239.22	-0.48	excluded	absent	93.93	-1.18	excluded	absent	81.5	-1.24
241021_at	excluded	absent	72.18	-0.36	excluded	absent	34.88	-0.68	excluded	absent	11.27	-0.88	excluded	absent	55.95	-0.5	excluded	absent	132.59	0.15	excluded	absent	56.61	-0.5	excluded	absent	11.42	-0.88	excluded	absent	73.08	-0.36	excluded	absent	44.62	-0.6	excluded	absent	2.26	-0.96	excluded	absent	39.8	-0.64	excluded	absent	49.52	-0.56	excluded	absent	6.5	-0.92	excluded	absent	43.8	-0.6	excluded	absent	5.68	-0.93
241022_at	excluded	absent	2.13	-0.77	excluded	absent	1.39	-0.8	excluded	absent	5.51	-0.64	excluded	absent	1.39	-0.8	excluded	absent	2.93	-0.74	excluded	absent	91.12	2.52	excluded	absent	2.45	-0.76	excluded	absent	38.11	0.56	excluded	absent	3.58	-0.72	excluded	absent	1.92	-0.78	excluded	absent	3.9	-0.7	excluded	absent	2.01	-0.77	excluded	absent	4.56	-0.68	excluded	absent	23.82	0.03	excluded	absent	0.4	-0.83
241023_at	excluded	absent	50.92	-0.39	excluded	absent	4.03	-1.15	excluded	absent	8.3	-1.08	excluded	absent	41.57	-0.54	excluded	absent	69.01	-0.1	excluded	absent	48.79	-0.43	excluded	absent	14.85	-0.97	excluded	absent	43.43	-0.51	included	present	88.51	0.22	excluded	absent	79.01	0.06	excluded	absent	86.36	0.18	excluded	absent	8.01	-1.08	excluded	absent	92.84	0.29	excluded	absent	22.39	-0.85	excluded	absent	4.75	-1.14
241024_at	included	present	174.21	0.27	included	present	567.82	4.39	included	present	259.74	1.17	included	present	458.85	3.25	included	present	410.33	2.74	included	present	144.67	-0.04	included	present	280.2	1.38	included	present	256.28	1.13	included	present	329.93	1.9	included	present	307.36	1.66	included	present	275.46	1.33	included	present	350.06	2.11	included	present	188.61	0.42	included	present	1150.05	10.47	included	present	251.96	1.09
241025_at	included	present	115.49	0.21	excluded	absent	95.31	-0.04	included	present	244.88	1.81	excluded	absent	173.17	0.92	included	present	349.4	3.1	excluded	absent	4.29	-1.17	excluded	absent	66.66	-0.4	included	present	94.2	-0.06	included	present	138.12	0.49	excluded	absent	49	-0.62	included	present	156.5	0.71	excluded	absent	2.28	-1.19	excluded	absent	128.09	0.36	excluded	absent	61.33	-0.46	excluded	absent	26.74	-0.89
241026_at	excluded	absent	12.63	-0.52	excluded	absent	32.82	-0.02	excluded	absent	6.25	-0.68	excluded	absent	2.51	-0.77	excluded	absent	94.45	1.51	excluded	absent	72.4	0.96	excluded	absent	4.62	-0.72	excluded	absent	18.16	-0.38	excluded	absent	29.69	-0.1	excluded	absent	53.01	0.48	excluded	absent	7.62	-0.65	excluded	absent	3.14	-0.76	excluded	absent	12.24	-0.53	included	present	187.3	3.81	excluded	absent	5.92	-0.69
241027_at	included	present	61.22	-0.19	excluded	absent	44.95	-0.58	excluded	absent	39.89	-0.7	included	present	109.7	0.98	excluded	absent	114.9	1.1	excluded	absent	4.3	-1.56	excluded	absent	54.09	-0.36	included	present	69	0	included	present	45.53	-0.57	excluded	absent	48.52	-0.5	included	present	91.62	0.54	excluded	absent	76.48	0.18	included	present	76.04	0.17	included	present	109	0.96	excluded	absent	23.92	-1.09
241028_at	excluded	absent	46.6	0.2	excluded	absent	4.77	-1.1	included	present	122	2.54	excluded	absent	30.32	-0.3	excluded	absent	15.9	-0.75	excluded	absent	75.83	1.11	excluded	absent	39.46	-0.02	excluded	absent	13.67	-0.82	excluded	absent	55.63	0.48	included	present	62.27	0.69	excluded	absent	81.86	1.29	excluded	absent	2.02	-1.18	excluded	absent	18.09	-0.68	excluded	absent	69.62	0.91	excluded	absent	31.88	-0.26
241029_at	excluded	absent	6.07	-0.41	excluded	absent	4.04	-0.44	excluded	absent	13.96	-0.3	excluded	absent	3.61	-0.45	excluded	absent	7.83	-0.39	excluded	absent	11.29	-0.34	excluded	absent	9.8	-0.36	excluded	absent	1.68	-0.48	excluded	absent	19.57	-0.21	excluded	absent	27.37	-0.1	excluded	absent	49.51	0.23	excluded	absent	52.7	0.28	excluded	absent	59.38	0.38	excluded	absent	20	-0.21	excluded	absent	1.53	-0.48
241030_at	excluded	absent	96.52	-0.01	excluded	absent	72.12	-0.29	excluded	absent	4.35	-1.07	excluded	absent	26.02	-0.82	excluded	absent	102.6	0.06	excluded	absent	3.51	-1.08	excluded	absent	59.67	-0.43	excluded	absent	20.77	-0.88	excluded	absent	49.87	-0.55	excluded	absent	24.73	-0.83	excluded	absent	46.68	-0.58	excluded	absent	35.63	-0.71	excluded	absent	66.17	-0.36	excluded	absent	45.69	-0.59	excluded	absent	19.29	-0.9
241031_at	excluded	absent	4.76	-0.3	excluded	absent	91.97	-0.14	excluded	absent	34.54	-0.25	included	present	607.63	0.8	excluded	absent	156.12	-0.03	excluded	absent	66.42	-0.19	included	present	514.42	0.63	excluded	absent	46.56	-0.23	included	present	235.64	0.12	included	present	132.78	-0.07	included	present	76.3	-0.17	excluded	absent	52.52	-0.21	excluded	absent	49.03	-0.22	included	present	1125.93	1.75	included	present	527.97	0.65
241032_at	excluded	absent	33.33	-0.11	excluded	absent	44.44	0.18	excluded	absent	116.65	2.08	excluded	absent	44.9	0.19	excluded	absent	53.13	0.41	excluded	absent	1.95	-0.93	excluded	absent	82.02	1.17	excluded	absent	3.35	-0.9	included	present	70.57	0.87	excluded	absent	21.4	-0.42	excluded	absent	10.91	-0.7	excluded	absent	20.65	-0.44	excluded	absent	31.22	-0.17	excluded	absent	15.31	-0.58	excluded	absent	4.38	-0.87
241033_at	included	present	366.07	-0.63	included	present	170.27	-1.8	included	present	583.56	0.67	included	present	321.81	-0.89	included	present	1044.41	3.42	included	present	330.23	-0.84	included	present	341.95	-0.77	included	present	266.23	-1.23	included	present	559.19	0.52	included	present	195.22	-1.65	included	present	460.56	-0.07	included	present	367.67	-0.62	included	present	496.86	0.15	included	present	491.52	0.12	included	present	128.83	-2.05
241034_at	included	absent	119.14	-0.59	included	absent	2.59	-1.82	included	present	142.69	-0.34	included	present	309.73	1.43	included	absent	5.17	-1.79	included	present	134.51	-0.43	included	present	99.94	-0.79	included	absent	25.89	-1.57	included	present	171.72	-0.03	included	present	95.56	-0.84	included	absent	40.78	-1.42	included	present	125	-0.53	included	absent	7.92	-1.76	included	absent	36.2	-1.46	included	present	45.73	-1.36
241035_s_at	included	present	115.61	-0.46	included	absent	94.33	-0.73	included	absent	98.82	-0.68	included	present	175.38	0.29	excluded	absent	159.6	0.09	included	absent	72.82	-1.01	included	present	208.97	0.72	included	present	92.07	-0.76	included	present	116.33	-0.46	included	present	89.2	-0.8	included	present	108.36	-0.56	included	absent	61.55	-1.15	included	present	185.54	0.42	included	present	89.74	-0.79	included	present	132.35	-0.25
241036_at	included	present	145.48	0.04	excluded	absent	63.21	-0.91	included	present	404.32	3.03	included	present	528.71	4.46	included	present	218.83	0.89	excluded	absent	90.95	-0.59	included	present	344.01	2.33	included	present	76.3	-0.76	included	present	284.39	1.64	included	present	70.75	-0.82	excluded	absent	106.15	-0.41	included	present	847.24	8.13	included	present	98.11	-0.51	included	present	113.24	-0.33	included	present	269.98	1.48
241037_at	excluded	absent	44.79	0	excluded	absent	57.09	0.37	excluded	absent	57.89	0.39	excluded	absent	2.13	-1.27	excluded	absent	8.97	-1.07	excluded	absent	5.9	-1.16	excluded	absent	47.16	0.07	excluded	absent	31.83	-0.39	excluded	absent	64.71	0.59	excluded	absent	71.78	0.81	excluded	absent	47.48	0.08	excluded	absent	39.55	-0.16	excluded	absent	88.22	1.3	excluded	absent	27	-0.53	excluded	absent	5.91	-1.16
241038_at	excluded	absent	71.55	-0.53	included	present	164.7	0.32	excluded	absent	70.8	-0.53	excluded	absent	4.7	-1.14	included	present	354.16	2.05	excluded	absent	6.54	-1.12	excluded	absent	48.72	-0.74	excluded	absent	9.72	-1.09	excluded	absent	47.69	-0.74	included	present	163.25	0.31	included	present	124.96	-0.04	excluded	absent	7.11	-1.11	included	present	111.76	-0.16	included	present	93.8	-0.32	excluded	absent	54.6	-0.68
241039_at	excluded	absent	8.14	-0.32	excluded	absent	3.44	-0.63	excluded	absent	2.86	-0.67	excluded	absent	5.62	-0.49	excluded	absent	4.35	-0.57	excluded	absent	9.92	-0.2	excluded	absent	36.29	1.58	excluded	absent	4.36	-0.57	excluded	absent	30.18	1.17	excluded	absent	1.59	-0.76	excluded	absent	12.16	-0.05	excluded	absent	4.45	-0.57	excluded	absent	20.37	0.51	excluded	absent	8.41	-0.3	excluded	absent	5.98	-0.46
241040_at	excluded	absent	65.59	0.42	excluded	absent	3.12	-1.27	excluded	absent	3.08	-1.27	included	present	83.28	0.9	excluded	absent	27.84	-0.6	excluded	absent	6.47	-1.18	excluded	absent	53.62	0.1	excluded	absent	24.72	-0.68	excluded	absent	5.42	-1.21	excluded	absent	59.5	0.26	excluded	absent	42.36	-0.21	excluded	absent	78.6	0.77	included	present	76.37	0.71	excluded	absent	32.23	-0.48	excluded	absent	3.11	-1.27
241041_at	included	present	89.32	-0.75	included	absent	91.32	-0.73	included	present	391.81	2.19	included	absent	87.08	-0.77	included	present	238.58	0.7	included	present	129.97	-0.35	included	present	398.84	2.26	included	absent	20.66	-1.42	included	present	216.19	0.49	included	present	240.62	0.72	included	present	353.85	1.82	included	absent	129.62	-0.36	included	present	135.25	-0.3	included	present	78.84	-0.85	included	present	61.97	-1.01
241042_at	excluded	absent	6.73	-0.74	excluded	absent	5.63	-0.77	excluded	absent	2.35	-0.87	excluded	absent	1.39	-0.9	excluded	absent	76.55	1.31	excluded	absent	17.84	-0.41	excluded	absent	12.94	-0.56	excluded	absent	1.3	-0.9	excluded	absent	4.82	-0.8	excluded	absent	3.98	-0.82	excluded	absent	22.09	-0.29	excluded	absent	25.93	-0.18	excluded	absent	9.76	-0.65	excluded	absent	3	-0.85	excluded	absent	2.67	-0.86
241043_at	excluded	absent	174.94	-0.21	excluded	absent	220.56	0.24	excluded	absent	261.7	0.65	included	present	290.85	0.93	excluded	absent	140.19	-0.55	excluded	absent	89.94	-1.05	excluded	absent	124.73	-0.7	excluded	absent	43.9	-1.5	included	present	90.22	-1.04	excluded	absent	17.45	-1.76	included	present	203.75	0.07	excluded	absent	131.33	-0.64	included	present	254.53	0.57	included	present	137.66	-0.58	excluded	absent	14.14	-1.79

241369_at	included	present	249.28	0.39	excluded	absent	31.35	-0.56	included	present	107.62	-0.23	excluded	absent	69.13	-0.4	[illegible]
241370_at	included	present	1226.53	0.77	included	present	393.27	-0.73	included	present	2340	2.78	included	present	817.96	0.04	[illegible]
241371_at	excluded	absent	108.79	-0.66	excluded	absent	49.46	-0.98	excluded	absent	4.82	-1.23	excluded	absent	6.07	-1.22	[illegible]
241372_at	included	present	441.16	0.13	included	present	183.45	-2.05	included	present	259.07	-1.41	included	present	243.16	-1.55	[illegible]
241373_at	included	present	293.87	-1.43	included	present	361.73	-0.97	included	present	569.48	0.41	included	present	308.6	-1.33	[illegible]
241374_at	included	present	348.93	1.58	included	present	104.05	-0.88	included	present	158.58	-0.33	included	present	124.07	-0.68	[illegible]
241375_at	excluded	absent	79	0.07	excluded	absent	17.57	-1.09	excluded	absent	115.81	0.77	excluded	absent	160.12	1.62	[illegible]
241376_at	included	present	1224.06	3.62	included	present	182.1	-1.3	included	present	196.15	-1.23	included	present	1082.87	2.96	[illegible]
241377_s_at	excluded	absent	40.1	-0.56	excluded	absent	119.72	1.01	excluded	absent	4.47	-1.26	excluded	absent	19.11	-0.97	[illegible]
241378_at	excluded	absent	27.66	-0.99	excluded	absent	123.44	0.73	excluded	absent	8.79	-1.33	excluded	absent	31.09	-0.93	[illegible]
241379_at	excluded	absent	49.01	-0.56	excluded	absent	43.16	-0.7	excluded	absent	75.76	0.07	excluded	absent	1.51	-1.68	[illegible]
241380_at	excluded	absent	130.34	-0.68	excluded	absent	62.78	-1.36	excluded	absent	52.4	-1.47	included	present	183.08	-0.14	[illegible]
241381_at	excluded	absent	20.19	-0.76	excluded	absent	42.81	-0.35	excluded	absent	46.95	-0.27	excluded	absent	9.33	-0.96	[illegible]
241382_at	included	absent	132.98	-0.44	included	absent	41.81	-0.45	included	absent	7.88	-0.46	included	absent	70.66	-0.45	[illegible]
241383_at	excluded	absent	108.71	0.05	excluded	absent	13.48	-0.97	included	present	163.09	0.63	included	present	243.88	1.5	[illegible]
241384_x_at	included	present	249.13	-0.15	included	present	119.25	-1.36	included	present	94.57	-1.59	included	present	260.28	-0.05	[illegible]
241385_at	included	absent	114.34	-1.05	included	present	156.3	-0.51	included	present	169.8	-0.33	included	present	161.09	-0.44	[illegible]
241386_at	included	present	233.53	-0.38	included	present	1175.07	2.2	included	present	1441.59	2.93	included	present	2082.29	4.68	[illegible]
241387_at	excluded	absent	146.31	-0.27	excluded	absent	104.3	-0.69	excluded	absent	119.06	-0.55	excluded	absent	96.01	-0.77	[illegible]
241388_at	included	present	553.33	-0.23	included	present	725.63	0.55	included	present	772	0.76	included	present	935.92	1.5	[illegible]
241389_at	included	present	234.72	-0.04	included	present	617.1	1.87	included	present	434.43	0.96	included	present	497.01	1.27	[illegible]
241390_at	excluded	absent	4.88	-0.44	excluded	absent	1.03	-0.52	excluded	absent	8.22	-0.38	excluded	absent	2.42	-0.49	[illegible]
241391_at	included	present	647.37	1.21	included	present	87.81	-2.27	included	present	814.82	2.25	included	present	466.81	0.09	[illegible]
241392_at	included	present	774.31	0.79	included	present	167.48	-1.18	included	present	766.86	0.77	included	present	1262.43	2.38	[illegible]
241393_at	included	present	163.75	-0.56	included	absent	111.06	-1.38	included	present	166.43	-0.52	included	present	446.93	3.87	[illegible]
241394_at	excluded	absent	2.27	-0.44	excluded	absent	2.47	-0.43	excluded	absent	3.43	-0.38	excluded	absent	1.7	-0.46	[illegible]
241395_at	included	present	261.15	0.52	included	present	288.33	0.83	included	present	340.33	1.43	included	present	305.06	1.03	[illegible]
241396_at	excluded	absent	45.29	-0.4	excluded	absent	73.4	-0.06	excluded	absent	12.97	-0.79	excluded	absent	135.89	0.69	[illegible]
241397_at	excluded	absent	6.9	-0.36	excluded	absent	10.36	-0.35	included	present	119.84	-0.12	included	present	852.39	1.42	[illegible]
241398_at	excluded	absent	73.41	-0.4	excluded	absent	9.34	-0.58	excluded	absent	9.34	-0.58	excluded	absent	70.21	-0.41	[illegible]
241399_at	excluded	absent	62.6	-0.5	included	present	236.78	-0.03	excluded	absent	42.52	-0.56	included	present	446.93	0.54	[illegible]
241400_at	included	present	92.23	-0.65	included	present	101.89	-0.59	included	present	96.71	-0.62	included	absent	79.47	-0.73	[illegible]
241401_at	included	present	185.54	-0.61	included	absent	50.06	-1.34	included	absent	80.21	-1.18	included	absent	22.54	-1.49	[illegible]
241402_at	included	absent	162.43	-1.21	included	present	464.64	-0.02	included	present	670.13	0.8	included	present	1753.16	5.09	[illegible]

[illegible]

241728_at	excluded	absent	79.97	-0.13	excluded	absent	7.21	-1.57	excluded	absent	84.6	-0.04	included	present	75.42	-0.22	excluded	absent
241729_at	included	present	294.09	0.83	included	present	236.84	0.49	excluded	absent	67.59	-0.52	included	present	132.37	-0.13	included	present
241730_at	included	present	117.33	-0.59	included	absent	85.96	-0.95	included	present	234.77	0.78	included	present	267	1.15	included	absent
241731_x_at	included	present	123.4	0.33	included	present	79.85	-0.44	excluded	absent	63.54	-0.73	excluded	absent	79.27	-0.45	excluded	absent
241732_at	excluded	absent	1.66	-0.89	excluded	absent	82.27	1.23	excluded	absent	72.05	0.96	excluded	absent	56.9	0.56	excluded	absent
241733_at	included	present	262.36	0.45	included	present	474.73	1.41	included	present	486.33	1.47	included	present	236	0.33	included	present
241734_at	included	present	175.42	0.77	excluded	absent	109.73	-0.52	included	present	283.78	2.88	included	present	235.39	1.94	included	present
241735_at	included	present	147.36	-0.81	included	absent	107.36	-1.07	included	absent	112.19	-1.04	included	absent	35.67	-1.53	included	absent
241736_at	included	present	324.11	0.2	included	present	156.61	-1.68	included	present	701.9	4.43	included	present	582.92	3.1	included	present
241737_x_at	included	present	410.29	-0.08	included	present	614.95	0.57	included	present	1198.79	2.41	included	present	1078.55	2.03	included	present
241738_at	included	present	422.1	-0.88	included	present	230.5	-1.68	included	present	1567.04	3.88	included	present	696.6	0.26	included	present
241739_at	excluded	absent	64.2	-0.32	excluded	absent	3.59	-1.28	excluded	absent	1.9	-1.31	excluded	absent	57.62	-0.42	excluded	absent
241740_at	excluded	absent	38.6	-0.8	excluded	absent	6.36	-1.29	excluded	absent	40.96	-0.76	excluded	absent	4.83	-1.31	excluded	absent
241741_at	included	present	227.76	-0.8	included	present	854.11	0.65	included	present	922.19	0.8	included	present	952.51	0.87	included	present
241742_at	included	present	103.71	0.69	excluded	absent	6.01	-0.95	excluded	absent	24.88	-0.63	excluded	absent	2.2	-1.01	excluded	absent
241743_at	excluded	absent	128.87	0.37	excluded	absent	315.16	2.93	excluded	absent	151.35	0.68	excluded	absent	49.63	-0.72	excluded	absent
241744_x_at	excluded	absent	5.24	-0.83	excluded	absent	40.06	-0.26	excluded	absent	1.64	-0.89	excluded	absent	31.58	-0.4	excluded	absent
241745_at	included	present	757.74	0.17	included	present	102.43	-1.48	included	present	380.37	-0.78	included	present	776.23	0.21	included	present
241746_at	excluded	absent	14.5	-1.52	included	present	168.25	2.77	excluded	absent	66.79	-0.06	excluded	absent	58.47	-0.29	excluded	absent
241747_s_at	excluded	absent	279.88	0.32	included	present	739.11	3.01	excluded	absent	276.8	0.3	excluded	absent	283.75	0.34	excluded	absent
241748_x_at	excluded	absent	31.13	-0.75	excluded	absent	19.82	-0.94	excluded	absent	17.26	-0.99	excluded	absent	53.07	-0.37	excluded	absent
241749_at	excluded	absent	15.52	-0.37	included	present	220.6	0.24	included	present	160.77	0.07	included	present	65.19	-0.22	included	present
241750_x_at	included	present	116.58	-0.6	included	absent	54.08	-0.95	included	absent	34.27	-1.07	included	present	127.56	-0.54	included	absent
241751_at	excluded	absent	117.2	0.19	excluded	absent	67.63	-0.57	excluded	absent	86.53	-0.28	excluded	absent	89.75	-0.23	excluded	absent
241752_at	included	present	576.75	-0.33	included	present	1301.9	0.79	included	present	2568.86	2.76	included	present	463.72	-0.51	included	present
241753_at	included	present	101.34	0.01	excluded	absent	49.84	-0.1	excluded	absent	1.7	-0.21	excluded	absent	12.59	-0.19	excluded	absent
241754_at	included	present	190.85	-0.57	included	present	226.39	-0.33	included	present	606.14	2.21	included	present	687.86	2.76	included	present
241755_at	included	present	360.83	0.46	included	present	140.58	-1.71	included	present	667.46	3.48	included	present	466.89	1.5	included	present
241756_at	included	present	82.27	-1.07	included	absent	57.35	-1.35	included	present	136.83	-0.46	included	absent	106.01	-0.81	included	absent
241757_x_at	included	present	259.04	-0.75	included	present	147.36	-1.43	included	present	189.09	-1.18	included	present	234.77	-0.9	included	present
241758_at	included	present	889.7	0	included	present	3597.73	2.16	included	present	241.58	-0.52	excluded	absent	47.13	-0.67	included	present
241759_at	excluded	absent	3.79	-1.3	excluded	absent	104.71	1.07	excluded	absent	63.13	0.09	excluded	absent	44.79	-0.34	excluded	absent
241760_x_at	excluded	absent	17.35	-1.03	excluded	absent	44.21	-0.64	excluded	absent	10.64	-1.12	excluded	absent	25.9	-0.9	excluded	absent
241761_at	excluded	absent	3.11	-1.22	excluded	absent	94.36	0.14	excluded	absent	31.78	-0.79	included	present	96.43	0.17	excluded	absent

[illegible]

242087_x_at	excluded	absent	46.11	-1.03	excluded	absent	227.78	1.93	excluded	absent	[illegible]
242088_at	included	present	362.51	-0.9	included	present	359	-0.92	included	present	[illegible]
242089_at	included	present	253.38	-0.07	included	present	91.58	-0.99	included	present	[illegible]
242090_x_at	excluded	absent	35.63	-0.14	excluded	absent	66.3	0.73	excluded	absent	[illegible]
242091_at	excluded	absent	41.94	0.02	excluded	absent	73.93	0.92	included	present	[illegible]
242092_at	included	present	1163.91	1.69	included	present	200.24	-1.22	included	present	[illegible]
242093_at	included	absent	83.09	-0.63	included	absent	2.04	-0.77	included	absent	[illegible]
242094_at	included	present	207.7	0.17	included	absent	50.21	-0.96	included	present	[illegible]
242095_at	excluded	absent	40.32	0.44	excluded	absent	8.61	-0.63	excluded	absent	[illegible]
242096_at	included	present	66.33	0.1	included	present	68.5	0.16	included	present	[illegible]
242097_at	excluded	absent	26.08	-0.6	included	present	296.91	1.44	included	present	[illegible]
242098_at	excluded	absent	103.29	0.55	excluded	absent	2.81	-0.95	excluded	absent	[illegible]
242099_at	included	present	191.81	-0.73	included	present	148.37	-0.99	included	present	[illegible]
242100_at	excluded	absent	2.2	-0.97	excluded	absent	56.59	-0.31	excluded	absent	[illegible]
242101_at	included	present	124.12	0.62	excluded	absent	16.6	-0.92	included	present	[illegible]
242102_at	excluded	absent	2.21	-0.33	excluded	absent	9.73	-0.26	included	present	[illegible]
242103_at	excluded	absent	188.64	0.51	included	present	231.8	0.97	excluded	absent	[illegible]
242104_at	excluded	absent	92.25	-0.24	excluded	absent	64.46	-0.3	included	present	[illegible]
242105_at	excluded	absent	37.24	0.3	excluded	absent	12.32	-0.53	excluded	absent	[illegible]
242106_at	included	present	195.61	0.4	included	present	183.33	0.24	included	present	[illegible]
242107_x_at	included	present	288.91	7.31	excluded	absent	32.7	-0.52	excluded	absent	[illegible]
242108_at	excluded	absent	81.32	-0.65	excluded	absent	7.41	-1.52	included	present	[illegible]
242109_at	excluded	absent	92.15	-0.49	excluded	absent	76.07	-0.79	excluded	absent	[illegible]
242110_at	included	present	469.54	1.37	included	absent	22.72	-2.22	included	present	[illegible]
242111_at	included	present	618.07	0.4	included	present	371.55	-0.44	included	present	[illegible]
242112_at	included	present	95.63	0.88	excluded	absent	60.7	0.02	included	present	[illegible]
242113_at	excluded	absent	76.31	0.04	included	present	110.88	0.85	excluded	absent	[illegible]
242114_at	included	present	435.63	-0.59	included	absent	184.1	-1.42	included	absent	[illegible]
242115_at	included	present	142.69	-0.12	included	present	230.19	1.08	included	present	[illegible]
242116_x_at	included	present	86.82	-1.24	included	present	184.23	0.54	included	absent	[illegible]
242117_at	included	present	564.93	-0.46	included	present	212.29	-1.28	included	present	[illegible]
242118_x_at	excluded	absent	3.38	-0.23	excluded	absent	1.31	-0.24	excluded	absent	[illegible]
242119_at	excluded	absent	71.07	-0.59	excluded	absent	103.49	-0.4	excluded	absent	[illegible]
242120_at	included	present	195.84	1.84	included	present	370.36	6.01	included	present	[illegible]
[illegible]											

242446_at	excluded	absent	94.71	0.43	excluded	absent	73.43	-0.07	excluded	absent	93.02	0.39	excluded	absent	47.98	-0.67	excluded	absent	64.53	-0.28	excluded	absent	72.28	-0.1	excluded	absent	61.37	-0.36	excluded	absent	73.18	-0.08	included	present	137.23	1.42	excluded	absent	67.76	-0.21	included	present	96.52	0.47	excluded	absent	80.35	0.09	included	present	94.2	0.41	excluded	absent	44.81	-0.74	excluded	absent	61.68	-0.35
242447_at	included	present	859.95	-0.5	included	present	323.49	-1.09	included	present	193.78	-1.23	included	present	376.79	-1.03	included	absent	73.65	-1.36	included	present	705.97	-0.67	included	present	275.38	-1.14	included	present	535.14	-0.85	included	present	1098.98	-0.24	included	present	342.31	-1.07	included	present	464.03	-0.93	included	present	441.13	-0.96	included	present	673.05	-0.7	included	present	1200.74	-0.12	included	present	913.56	-0.44
242448_at	included	present	455.83	0.23	included	absent	374.1	-0.09	excluded	absent	553.28	0.61	included	present	328.53	-0.26	included	present	533.79	0.54	included	absent	119.64	-1.08	included	present	385.52	-0.04	included	present	117.47	-1.09	included	present	304.43	-0.36	included	present	484.56	0.34	included	present	288.43	-0.42	included	absent	388.49	-0.03	included	absent	278.36	-0.46	included	present	580.15	0.72	included	absent	234.48	-0.63
242449_at	excluded	absent	110.48	0.06	excluded	absent	138.99	0.41	excluded	absent	98.77	-0.08	excluded	absent	111.61	0.08	excluded	absent	15.03	-1.11	excluded	absent	74.65	-0.38	excluded	absent	11.12	-1.16	excluded	absent	72.64	-0.4	excluded	absent	14.24	-1.12	excluded	absent	78.01	-0.34	excluded	absent	57.88	-0.59	excluded	absent	87.3	-0.22	excluded	absent	59.87	-0.56	included	present	146.36	0.5	excluded	absent	55.68	-0.61
242450_at	included	present	162.68	1.12	excluded	absent	94.81	0.18	included	present	270.87	2.62	included	present	97.54	0.22	included	present	154.15	1	excluded	absent	83.53	0.02	included	present	123.61	0.58	excluded	absent	46.13	-0.5	included	present	175.07	1.29	included	present	151.44	0.96	included	present	189.75	1.5	included	present	64.11	-0.25	included	present	61.21	-0.29	excluded	absent	35.56	-0.64	included	present	81.93	0
242451_x_at	included	present	288.09	-0.5	included	absent	301.42	-0.41	included	present	240.76	-0.81	included	present	488.3	0.79	included	present	256.86	-0.7	included	present	396.91	0.2	included	present	210.34	-1	included	present	383.85	0.12	included	present	193.65	-1.11	included	present	275.36	-0.58	included	present	321.65	-0.28	included	present	323.31	-0.27	included	present	281.19	-0.54	included	present	574.65	1.35	included	present	273.46	-0.59
242452_at	included	present	157.25	1.01	included	present	538.71	7.31	included	present	184.55	1.46	included	present	125.17	0.48	included	present	218.06	2.01	excluded	absent	47.98	-0.8	included	present	297.73	3.33	excluded	absent	16.23	-1.32	excluded	absent	91.73	-0.07	excluded	absent	55.26	-0.68	included	present	353.9	4.26	included	present	169.82	1.22	included	present	248.68	2.52	excluded	absent	150.52	0.9	included	present	218.99	2.03
242453_at	excluded	absent	106.1	1.18	excluded	absent	85.85	0.73	excluded	absent	124.02	1.58	excluded	absent	104.04	1.13	excluded	absent	72.81	0.44	excluded	absent	96.6	0.97	excluded	absent	34.31	-0.42	excluded	absent	42.94	-0.23	excluded	absent	43.26	-0.22	excluded	absent	28.92	-0.54	excluded	absent	89.62	0.81	excluded	absent	18.75	-0.77	excluded	absent	75.96	0.51	excluded	absent	72.4	0.43	excluded	absent	5.07	-1.07
242454_at	excluded	absent	3.19	-0.51	excluded	absent	3.33	-0.51	excluded	absent	71.8	0.1	excluded	absent	53.13	-0.07	excluded	absent	16.48	-0.4	excluded	absent	1.97	-0.53	excluded	absent	23	-0.34	excluded	absent	42.19	-0.17	excluded	absent	42.55	-0.16	excluded	absent	1.31	-0.53	excluded	absent	28.72	-0.29	excluded	absent	30.9	-0.27	excluded	absent	83.1	0.2	excluded	absent	41.76	-0.17	excluded	absent	3.33	-0.51
242455_at	included	present	319.31	0.11	included	present	718.35	1.23	excluded	absent	49.77	-0.64	included	present	586.67	0.86	included	present	298.03	0.05	excluded	absent	64.18	-0.6	excluded	absent	102.12	-0.5	excluded	absent	60.03	-0.61	excluded	absent	48.72	-0.65	excluded	absent	98.59	-0.51	included	present	450.62	0.48	excluded	absent	122.74	-0.44	included	present	163.95	-0.32	included	present	115.43	-0.46	included	present	229.67	-0.14
242456_at	excluded	absent	14.31	-1.04	excluded	absent	95.18	1.01	included	present	147.3	2.33	excluded	absent	111.82	1.43	excluded	absent	29.8	-0.65	excluded	absent	2.2	-1.35	excluded	absent	45.21	-0.26	included	present	118.4	1.6	excluded	absent	58.69	0.09	excluded	absent	1.6	-1.36	excluded	absent	57.38	0.05	excluded	absent	102.68	1.2	excluded	absent	63.28	0.2	excluded	absent	1.32	-1.37	excluded	absent	15.22	-1.02
242457_at	included	present	115.84	-0.98	included	absent	39.98	-1.56	included	present	198.4	-0.35	included	present	242.22	-0.02	included	absent	217.87	-0.21	included	absent	135.13	-0.84	included	present	133.99	-0.85	included	absent	35.06	-1.6	included	present	330.35	0.65	included	present	353.56	0.83	included	present	146.21	-0.75	included	absent	29.61	-1.64	included	present	157	-0.67	included	present	266.81	0.17	included	absent	48.71	-1.5
242458_at	included	present	398.93	-0.46	included	present	603.7	-0.28	included	present	1090.57	0.17	included	present	1163.74	0.23	included	present	693.95	-0.19	included	present	296.63	-0.56	included	present	965.81	0.05	included	present	672.33	-0.21	included	present	823.44	-0.08	included	present	249.09	-0.6	included	present	1115.41	0.19	included	present	239.3	-0.61	included	present	458.68	-0.41	included	present	315.01	-0.54	included	present	607.17	-0.27
242459_at	excluded	absent	2.7	-0.63	excluded	absent	3.53	-0.62	excluded	absent	3.49	-0.62	excluded	absent	27.08	-0.37	excluded	absent	14.94	-0.5	excluded	absent	3.3	-0.63	excluded	absent	24.77	-0.39	excluded	absent	44.66	-0.18	excluded	absent	50.56	-0.11	excluded	absent	5.29	-0.61	excluded	absent	43.19	-0.19	excluded	absent	13.35	-0.52	excluded	absent	2.51	-0.64	included	present	52.79	-0.09	excluded	absent	25.87	-0.38
242460_at	excluded	absent	2.57	-0.69	excluded	absent	2.61	-0.68	excluded	absent	3.62	-0.63	excluded	absent	30.7	0.66	excluded	absent	43.37	1.27	excluded	absent	4.86	-0.58	excluded	absent	33.26	0.79	excluded	absent	1.85	-0.72	excluded	absent	40.89	1.15	excluded	absent	6.3	-0.51	excluded	absent	5.82	-0.53	excluded	absent	73.56	2.72	excluded	absent	3.77	-0.63	excluded	absent	42.26	1.22	excluded	absent	12.73	-0.2
242461_at	excluded	absent	114.94	1.51	excluded	absent	3.38	-1.21	excluded	absent	4.09	-1.19	excluded	absent	80.43	0.67	excluded	absent	84.69	0.77	excluded	absent	21.85	-0.76	excluded	absent	8.45	-1.08	excluded	absent	64.68	0.29	excluded	absent	48.26	-0.11	excluded	absent	43.86	-0.22	excluded	absent	7.48	-1.11	excluded	absent	2.45	-1.23	excluded	absent	3.08	-1.21	excluded	absent	4.25	-1.19	excluded	absent	5.19	-1.16
242462_at	included	present	130.91	-0.31	included	present	309.5	2.37	included	present	356.66	3.08	included	present	672.6	7.82	included	present	257.28	1.59	included	present	132.36	-0.28	included	present	246.72	1.43	included	present	217.62	1	included	present	279.2	1.92	included	present	170.89	0.29	included	present	386.35	3.53	included	present	190.24	0.58	included	present	202.4	0.77	included	present	269.44	1.77	included	present	152	0.01
242463_x_at	included	present	940.32	-0.08	included	present	346.66	-0.95	included	present	1086.41	0.13	included	present	1148.38	0.22	included	present	485.88	-0.74	included	present	1068.41	0.1	included	present	380.35	-0.9	included	present	847.57	-0.22	included	present	704.38	-0.43	included	present	271.62	-1.06	included	present	277	-1.05	included	present	448.93	-0.8	included	present	744.98	-0.37	included	present	440.21	-0.81	included	present	229.81	-1.12
242464_at	excluded	absent	5.65	-0.63	excluded	absent	51.2	1.34	excluded	absent	73.86	2.33	excluded	absent	1.49	-0.81	excluded	absent	8.83	-0.49	excluded	absent	15.74	-0.19	excluded	absent	34.2	0.61	excluded	absent	29.03	0.38	excluded	absent	0.99	-0.83	excluded	absent	23.11	0.13	excluded	absent	1.86	-0.8	excluded	absent	4.39	-0.69	excluded	absent	1.1	-0.83	excluded	absent	21.98	0.08	excluded	absent	1.02	-0.83
242465_at	excluded	absent	52.83	-0.25	excluded	absent	30.04	-0.63	excluded	absent	34.45	-0.55	included	present	100.57	0.54	excluded	absent	56.75	-0.18	excluded	absent	110	0.7	excluded	absent	82.23	0.24	excluded	absent	45.05	-0.38	excluded	absent	62.28	-0.09	excluded	absent	44.56	-0.39	included	present	57.24	-0.18	excluded	absent	243.73	2.92	included	present	116.52	0.81	included	present	55.58	-0.2	included	present	75.76	0.13
242466_at	excluded	absent	99.55	0.3	excluded	absent	41.35	-0.43	excluded	absent	35.82	-0.5	excluded	absent	69.08	-0.08	excluded	absent	76.42	0.01	excluded	absent	62.93	-0.16	excluded	absent	48.57	-0.34	excluded	absent	10.89	-0.81	excluded	absent	57.37	-0.23	excluded	absent	21.37	-0.68	excluded	absent	17.87	-0.72	included	present	31.41	-0.55	excluded	absent	144.14	0.86	excluded	absent	84.01	0.11	excluded	absent	3.93	-0.9
242467_at	included	present	213.3	-1.2	included	present	168.55	-1.34	included	present	220.1	-1.18	included	present	434.4	-0.52	included	present	416.79	-0.57	included	present	511.79	-0.28	included	present	376.67	-0.69	included	present	148.98	-1.4	included	present	486.22	-0.36	included	present	169.22	-1.34	included	present	376.62	-0.69	included	present	376.39	-0.7	included	present	212.31	-1.2	included	present	233.81	-1.14	included	present	93.73	-1.57
242468_at	excluded	absent	62.84	0.11	excluded	absent	36.59	-0.34	excluded	absent	36.39	-0.35	excluded	absent	36.61	-0.34	excluded	absent	81.94	0.44	excluded	absent	6.53	-0.86	excluded	absent	41.61	-0.26	excluded	absent	20.82	-0.62	excluded	absent	1.37	-0.95	excluded	absent	23.84	-0.56	included	present	46.55	-0.17	excluded	absent	51.42	-0.09	included	present	80.74	0.41	excluded	absent	27.8	-0.5	excluded	absent	2.18	-0.94
242469_at	excluded	absent	111.45	0.15	excluded	absent	38.78	-0.63	excluded	absent	3.41	-1.01	excluded	absent	60.85	-0.4	excluded	absent	14.32	-0.9	excluded	absent	77.25	-0.22	excluded	absent	30.93	-0.72	excluded	absent	4.39	-1	excluded	absent	33.01	-0.69	excluded	absent	44.1	-0.58	excluded	absent	19.77	-0.84	excluded	absent	47.18	-0.54	excluded	absent	28.44	-0.74	excluded	absent	20.59	-0.83	excluded	absent	22.7	-0.81
242470_at	included	present	194.74	-0.18	included	present	241.91	0.11	included	present	251.87	0.17	included	present	797.77	3.6	included	present	366.87	0.89	included	absent	77.8	-0.92	included	present	578.89	2.22	included	present	76.89	-0.92	included	present	178.6	-0.29	included	present	405.36	1.14	included	present	306.72	0.52	included	present	196.22	-0.18	included	present	383.9	1	included	present	194.91	-0.18	included	present	302.31	0.49
242471_at	included	present	503.65	0.79	included	absent	74.18	-1.77	included	present	241.14	-0.78	included	present	95.46	-1.64	included	present	657.91	1.71	included	present	507.03	0.81	included	present	221.24	-0.89	included	present	92.91	-1.66	included	present	512.32	0.84	included	present	236.7	-0.8	included	present	540.6	1.01	included	present	459.25	0.53	included	present	207.33	-0.98	included	present	145.64	-1.35	included	present	112.15	-1.55
242472_x_at	included	present	542.92	-0.05	included	present	441.83	-0.55	included	present	690.51	0.68	included	present	553.83	0.01	included	present	522.36	-0.15	included	present	283.66	-1.33	included	present	408.19	-0.71	included	absent	145.96	-2.01	included	present	485.65	-0.33	included	present	218.94	-1.65	included	present	330.06	-1.1	included	present	235.38	-1.57	included	present	283.83	-1.33	included	present	216.86	-1.66	included	present	134.33	-2.06
242473_at	excluded	absent	141.4	2.65	excluded	absent	168.53	3.43	excluded	absent	91.66	1.23	excluded	absent	88.9	1.15	excluded	absent	220.11	4.91	excluded	absent	2.56	-1.33	excluded	absent	104.31	1.59	included	present	133.55	2.43	included	present	91.63	1.23	excluded	absent	96.43	1.36	included	present	188.52	4.01	excluded	absent	69.65	0.6	excluded	absent	77.12	0.81	excluded	absent	61.48	0.36	excluded	absent	81.33	0.93
242474_s_at	included	present	683.61	2	included	present	458.77	0.68	included	present	807.67	2.72	included	present	596.4	1.49	included	present	198.86	-0.83	included	present	258.67	-0.48	included	present	841.52	2.92	included	present	1308.38	5.64	included	present	722.52	2.22	included	present	221.71	-0.7	included	present	348.09	0.04	included	present	492.75	0.88	included	present	588.9	1.44	included	present	437.92	0.56	included	present	632.67	1.7
242475_at	included	present	685.4	0.42	included	present	829.45	0.87	included	present	825.91	0.86	included	present	604.01	0.17	included	present	550.5	0.01	included	present	527.15	-0.07	included	present	620.58	0.22	included	present	796.48	0.77	included	present	662.14	0.35	included	present	395.84	-0.47	included	present	1193.78	2	included	present	541.62	-0.02	included	absent	199.17	-1.08	included	present	222.08	-1.01	included	present	609.01	0.19
242476_at	included	present	408.68	-0.47	included	present	593.4	0.03	included	present	619.86	0.1	included	present	727.61	0.4	included	present	1004.81	1.15	included	present	342.59	-0.64	included	present	1395.76	2.2	included	present	544.55	-0.1	included	present	1141.91	1.52	included	present	1624.85	2.82	included	present	654.25	0.2	included	present	650.3	0.19	included	present	1759.36	3.18	included	present	393.27	-0.51	included	present	753.84	0.47
242477_at	included	present	272.16	-0.26	included	present	148.66	-0.92	included	absent	47.02	-1.46	included	present	454.61	0.72	included	absent	3.81	-1.69	included	present	481.6	0.86	included	present	159.06	-0.86	included	absent	87.99	-1.24	included	present	230.76	-0.48	included	absent	72.13	-1.33	included	absent	61.61	-1.38	included	present	209.05	-0.6	included	absent	86.68	-1.25	included	absent	69.68	-1.34	included	absent	63.38	-1.37
242478_at	excluded	absent	1.42	-0.61	excluded	absent	67.38	2.24	excluded	absent	1.92	-0.59	excluded	absent	14.86	-0.03	excluded	absent	3.71	-0.51	excluded	absent	44.46	1.25	excluded	absent	51.99	1.58	excluded	absent	9.64	-0.25	excluded	absent	4.09	-0.49	excluded	absent	0.67	-0.64	excluded	absent	47.22	1.37	excluded	absent	31.19	0.68	excluded	absent	30.12	0.63	excluded	absent	20.69	0.23	excluded	absent	0.7	-0.64
242479_s_at	excluded	absent	10.07	-0.65	excluded	absent	39.02	0.47	excluded	absent	1.67	-0.97	excluded	absent	22.17	-0.18	excluded	absent	6.04	-0.8	excluded	absent	194.36	6.5	excluded	absent	68.16	1.6	excluded	absent	13.24	-0.53	excluded	absent	51.89	0.97	excluded	absent	8.21	-0.72	excluded	absent	17.55	-0.36	excluded	absent	53.33	1.03	excluded	absent	7.03	-0.77	excluded	absent	33.41	0.26	excluded	absent	41.06	0.55

[illegible]

present	891.88	1.85	included	present	1206.82	3.24	included	present	648.17	0.77	included	present	992.57	2.29	included	present
absent	5.22	-0.78	excluded	absent	33.42	0.11	excluded	absent	43.94	0.44	excluded	absent	65.74	1.12	excluded	absent
present	146.37	0.05	included	present	173.03	0.32	included	present	149.21	0.08	included	present	264.34	1.27	included	present
absent	240.75	-0.58	included	absent	92.4	-1.45	included	present	219.06	-0.71	included	present	258.37	-0.48	included	absent
absent	57.43	-0.71	included	absent	16.03	-0.76	included	absent	2.37	-0.78	included	absent	49.98	-0.72	included	absent
absent	85.1	-1.18	excluded	absent	129.75	-0.49	excluded	absent	133.53	-0.43	excluded	absent	60.68	-1.56	excluded	absent
present	119.3	1.14	excluded	absent	5.15	-1.69	included	present	93.35	0.49	included	present	101.61	0.7	excluded	absent
present	605.44	-0.67	included	present	1132.57	0.68	included	present	1074.64	0.53	included	present	1596.37	1.86	included	present
absent	52.34	0.16	excluded	absent	107.01	1.49	excluded	absent	4.08	-1.02	excluded	absent	44.08	-0.04	excluded	absent
present	156.21	-0.14	excluded	absent	40.16	-0.51	excluded	absent	54.64	-0.47	excluded	absent	4.04	-0.63	excluded	absent
present	122.48	0.02	included	absent	12.6	-1.93	included	present	151.51	0.53	included	absent	117.53	-0.07	included	absent
absent	4.11	-0.57	excluded	absent	8.33	-0.47	excluded	absent	10.42	-0.42	excluded	absent	2.28	-0.61	excluded	absent
absent	7.62	-0.59	excluded	absent	9.64	-0.53	excluded	absent	3.12	-0.72	excluded	absent	8.77	-0.56	excluded	absent
absent	81	-0.9	excluded	absent	33.04	-1.94	included	present	78.03	-0.97	excluded	absent	71.69	-1.1	excluded	absent
absent	79.47	-0.43	included	present	150.41	-0.24	included	present	221.87	-0.06	included	present	198.09	-0.12	included	absent
present	122.13	-0.09	excluded	absent	89.95	-0.49	included	present	340.21	2.58	included	present	142.66	0.16	excluded	absent
absent	75.73	-0.5	excluded	absent	12.86	-0.73	included	present	123.11	-0.32	excluded	absent	94.31	-0.43	excluded	absent
present	674.41	4.03	included	present	368.7	2	excluded	absent	9.03	-0.4	excluded	absent	44.33	-0.16	excluded	absent
absent	1.9	-0.89	excluded	absent	5.62	-0.83	excluded	absent	2.56	-0.88	excluded	absent	72.71	0.2	excluded	absent
present	413.12	-0.58	included	absent	86.36	-1.94	included	absent	13.48	-2.24	included	present	241.34	-1.29	included	absent
absent	116.6	-0.29	included	present	1543.77	9.17	excluded	absent	22.13	-0.91	excluded	absent	5.35	-1.02	included	present
present	940.27	3.18	included	present	126.84	-1.17	included	present	1094.48	4.01	included	present	853.66	2.72	included	present
present	435.48	-0.22	included	present	170.7	-0.37	included	present	257.57	-0.32	included	present	570.5	-0.15	included	present
present	1088.39	3.27	included	present	575.21	0.93	included	present	174.12	-0.89	included	present	917.4	2.49	included	present
present	3855.3	0.57	included	present	1893.96	-1.02	included	present	3908.81	0.62	included	present	3599.56	0.37	included	present
absent	6.45	-0.44	excluded	absent	61.56	1.64	excluded	absent	5.22	-0.48	excluded	absent	36.6	0.7	excluded	absent
absent	76.97	-0.53	excluded	absent	128.2	-0.01	included	present	206.91	0.8	included	present	137.88	0.09	included	present
absent	6.45	-1.09	excluded	absent	23.57	-0.87	excluded	absent	27.35	-0.82	excluded	absent	60.87	-0.4	excluded	absent
absent	24.23	-0.24	excluded	absent	6.74	-0.39	excluded	absent	4.52	-0.41	excluded	absent	3.29	-0.42	excluded	absent
absent	127.18	1.21	excluded	absent	24.06	-0.99	excluded	absent	37.02	-0.72	excluded	absent	68.5	-0.04	excluded	absent
present	433.64	0.59	included	present	111.98	-0.85	included	present	240.18	-0.28	included	present	182.6	-0.54	included	present
present	143.82	-0.51	included	absent	70.97	-0.65	included	present	228.27	-0.34	included	present	95.04	-0.6	included	present
present	435.64	-0.25	included	present	461.46	-0.13	included	present	391.24	-0.46	included	present	698.47	1	included	present
absent	44.55	-0.66	excluded	absent	85	0.09	excluded	absent	53.04	-0.5	excluded	absent	72.7	-0.14	excluded	absent
[illegible]																

[illegible]	60.66	-0.49	included	present	164.41	-0.02	excluded	absent	53.11	-0.53	excluded	absent	37.3
[illegible]	131.5	0.61	excluded	absent	48.74	-0.92	included	present	569.81	8.69	included	present	177.54
[illegible]	74.08	-0.18	included	present	113.59	0.52	included	present	155.76	1.26	included	present	126.27
[illegible]	87.4	-0.39	excluded	absent	76	-0.61	included	present	115.15	0.14	included	present	294.63
[illegible]	185.08	5.75	excluded	absent	5.57	-0.49	excluded	absent	81.96	2.17	excluded	absent	7.49
[illegible]	315.12	-0.21	included	present	191.16	-0.31	included	present	820.73	0.21	included	present	1095.86
[illegible]	95.89	0.06	included	present	123.94	0.61	excluded	absent	145.17	1.03	included	present	62.89
[illegible]	127	0.44	included	present	208.86	1.68	excluded	absent	111.15	0.2	excluded	absent	8.09
[illegible]	128.8	0.78	excluded	absent	6.76	-1.27	excluded	absent	42.56	-0.67	excluded	absent	26.85
[illegible]	83.9	-0.35	excluded	absent	6.57	-1.09	excluded	absent	53.33	-0.64	excluded	absent	1.85
[illegible]	53.72	-0.33	included	present	113.42	-0.29	excluded	absent	68.99	-0.32	included	present	6824.85
[illegible]	59.47	0.41	excluded	absent	44.57	-0.03	excluded	absent	79.93	1	included	present	610.07
[illegible]	1.68	-0.64	excluded	absent	34.19	0.47	excluded	absent	53.98	1.14	excluded	absent	3.14
[illegible]	942.05	0.39	included	present	404.77	-1.15	included	present	540.9	-0.76	included	present	818.73
[illegible]	49.19	-0.71	excluded	absent	2.57	-1.47	excluded	absent	2.2	-1.47	included	present	34.29
[illegible]	153.83	-0.14	included	absent	9.31	-1.61	included	absent	84.26	-0.85	included	absent	39.88
[illegible]	89.36	-0.56	included	present	269.6	2.78	excluded	absent	71.05	-0.9	excluded	absent	5.6
[illegible]	8.46	-1.06	excluded	absent	10.75	-0.99	excluded	absent	11.27	-0.98	excluded	absent	33.37
[illegible]	49.92	-0.03	excluded	absent	65.65	0.44	excluded	absent	39.56	-0.34	included	present	101.11
[illegible]	114.65	-0.49	included	absent	14.8	-1.1	included	absent	136.87	-0.36	included	absent	7.34
[illegible]	106.16	-0.21	excluded	absent	67.57	-0.5	excluded	absent	50.06	-0.63	included	present	88.7
[illegible]	125.24	-0.68	included	absent	6.34	-2.05	included	absent	66.67	-1.36	included	absent	2.08
[illegible]	9.37	-0.86	excluded	absent	72.56	0.29	excluded	absent	19.39	-0.68	excluded	absent	33.65
[illegible]	223.14	1.03	included	present	617.36	5.29	included	present	368.28	2.6	included	present	225.55
[illegible]	161.02	0.41	excluded	absent	14.84	-1.01	excluded	absent	105.37	-0.13	excluded	absent	137.23
[illegible]	144.32	-0.61	included	present	128.57	-0.85	included	present	187.62	0.02	included	present	459.3
[illegible]	207.43	0.94	excluded	absent	64.06	-1.03	included	present	161.65	0.31	included	present	199.57
[illegible]	8.07	-1.3	excluded	absent	12.14	-1.22	excluded	absent	75.6	0.01	excluded	absent	41.28
[illegible]	84.37	1.37	excluded	absent	62.57	0.77	excluded	absent	5	-0.82	excluded	absent	2.24
[illegible]	2.55	-0.54	excluded	absent	1.58	-0.58	excluded	absent	38.44	0.93	excluded	absent	23.36
[illegible]	80.89	2.48	excluded	absent	5.21	-0.62	excluded	absent	37.77	0.71	excluded	absent	2.01
[illegible]	325.4	-0.14	included	present	183.08	-0.78	included	present	981.79	2.83	included	present	1143.1
[illegible]	118.63	1.44	excluded	absent	9.31	-1.27	included	present	184.56	3.07	included	present	205.1
[illegible]	165.22	0	included	present	213.41	0.49	included	present	113.03	-0.53	included	present	181.4
[illegible]	[illegible]	[illegible]	[illegible]	[illegible]	[illegible]	[illegible]	[illegible]	[illegible]	[illegible]	[illegible]	[illegible]	[illegible]	[illegible]

243522_at	included	present	230.12	-0.47	included	present	223.4	-0.51	included	present	268.91	-0.24	[illegible]
243523_at	excluded	absent	24.98	-1.26	excluded	absent	190.13	0.94	excluded	absent	80.29	-0.53	[illegible]
243524_at	excluded	absent	114.07	0.14	excluded	absent	47.74	-1.19	excluded	absent	98.63	-0.17	[illegible]
243525_at	included	present	246.62	0.35	included	present	150.03	-0.6	included	present	267.28	0.55	[illegible]
243526_at	excluded	absent	63.94	0.05	excluded	absent	178.93	1.35	excluded	absent	65.37	0.06	[illegible]
243527_at	included	present	233.78	-0.42	included	absent	70.79	-1.35	included	present	170.6	-0.78	[illegible]
243528_at	included	present	122.3	-0.01	excluded	absent	3.15	-1.71	excluded	absent	60.61	-0.89	[illegible]
243529_at	included	present	312.62	-1.02	included	present	543.72	0.21	included	present	808.83	1.63	[illegible]
243530_at	included	present	199.34	-0.61	included	present	293.62	0.08	included	present	746.11	3.41	[illegible]
243531_at	included	present	246.28	0.55	included	present	284.62	0.92	included	present	185.83	-0.04	[illegible]
243532_at	excluded	absent	159.84	1.37	excluded	absent	8.58	-1.45	excluded	absent	122.66	0.67	[illegible]
243533_x_at	excluded	absent	2.48	-0.89	excluded	absent	51.5	-0.3	excluded	absent	63.86	-0.15	[illegible]
243534_at	included	present	98.51	-0.18	excluded	absent	85.65	-0.37	excluded	absent	33.14	-1.15	[illegible]
243535_at	excluded	absent	59.36	0.2	included	present	139.69	2.15	excluded	absent	7.78	-1.04	[illegible]
243536_x_at	excluded	absent	79.48	0.17	excluded	absent	110.26	0.69	excluded	absent	81.02	0.2	[illegible]
243537_at	included	present	374.37	1.21	included	absent	184.87	-0.66	included	present	267.06	0.16	[illegible]
243538_at	excluded	absent	85.79	-1.33	excluded	absent	141.74	-0.39	excluded	absent	125.39	-0.67	[illegible]
243539_at	included	present	429.73	0.66	included	present	411.62	0.56	included	present	367.68	0.32	[illegible]
243540_at	excluded	absent	77.48	0.07	excluded	absent	41.61	-0.32	excluded	absent	3.4	-0.73	[illegible]
243541_at	excluded	absent	28.78	-0.35	excluded	absent	47.72	0.06	excluded	absent	4.13	-0.88	[illegible]
243542_at	included	present	77.77	-0.42	excluded	absent	86.89	-0.31	included	present	119.91	0.05	[illegible]
243543_at	excluded	absent	2.28	-0.85	excluded	absent	14.01	-0.68	excluded	absent	2.73	-0.84	[illegible]
243544_at	included	present	254.61	-0.11	excluded	absent	5.3	-0.54	excluded	absent	60.35	-0.44	[illegible]
243545_at	excluded	absent	74.28	1.24	excluded	absent	13.09	-0.47	excluded	absent	12.85	-0.47	[illegible]
243546_at	included	present	512.84	0.68	excluded	absent	113.08	-0.79	included	present	344.32	0.06	[illegible]
243547_at	included	present	141.63	-0.22	included	present	140.52	-0.23	included	present	93.64	-0.69	[illegible]
243548_x_at	excluded	absent	52.05	0.58	excluded	absent	44.23	0.31	excluded	absent	19.02	-0.56	[illegible]
243549_at	excluded	absent	6.77	-0.72	excluded	absent	54.32	0.22	excluded	absent	26.14	-0.34	[illegible]
243550_at	included	present	810.21	1.45	included	present	446.83	-0.11	included	present	585.44	0.49	[illegible]
243551_at	excluded	absent	45.19	-0.19	excluded	absent	2.92	-1.06	excluded	absent	44.41	-0.21	[illegible]
243552_at	included	present	273.23	0.61	included	present	428.92	1.73	included	present	752.6	4.04	[illegible]
243553_x_at	excluded	absent	514.48	1.02	included	present	360.74	0.07	included	present	683.37	2.07	[illegible]
243554_at	included	present	87.57	-1.01	included	absent	71.39	-1.23	included	present	268.7	1.43	[illegible]
243555_at	excluded	absent	73.48	0	excluded	absent	42.25	-0.25	included	present	110.6	0.31	[illegible]
[illegible]	[illegible]	[illegible]	[illegible]	[illegible]	[illegible]	[illegible]	[illegible]	[illegible]	[illegible]	[illegible]	[illegible]	[illegible]	[illegible]

243881_at	excluded	absent	158.96	-0.45	included	present	171.42	-0.43	excluded	absent	224.1	-0.33	excluded	absent	95.28	-0.57	excluded	absent	139	-0.49	excluded	absent	48.55	-0.66	included	present	146.44	-0.48	excluded	absent	38.9	-0.67	included	absent
243882_at	included	present	270.35	0.55	excluded	absent	20.61	-0.43	excluded	absent	5	-0.49	excluded	absent	36.16	-0.37	excluded	absent	59.6	-0.27	excluded	absent	34.81	-0.37	excluded	absent	91.52	-0.15	excluded	absent	41.05	-0.35	included	present
243883_at	excluded	absent	2.33	-0.61	excluded	absent	2.19	-0.62	excluded	absent	2.06	-0.62	excluded	absent	1.66	-0.64	excluded	absent	4.03	-0.54	excluded	absent	1.98	-0.62	excluded	absent	7.06	-0.42	excluded	absent	4.09	-0.54	excluded	absent
243884_at	excluded	absent	74.22	-0.1	excluded	absent	8.69	-0.33	excluded	absent	2.14	-0.35	excluded	absent	34.83	-0.24	excluded	absent	18.36	-0.29	excluded	absent	1.49	-0.35	excluded	absent	33.97	-0.24	excluded	absent	0.96	-0.35	included	absent
243885_x_at	excluded	absent	54.44	-0.17	excluded	absent	32.92	-0.66	excluded	absent	28.38	-0.76	excluded	absent	33.89	-0.64	excluded	absent	74.67	0.29	included	present	240.16	4.05	excluded	absent	43.34	-0.42	excluded	absent	31.02	-0.7	included	present
243886_at	excluded	absent	4.45	-0.97	excluded	absent	9.68	-0.86	excluded	absent	4.25	-0.97	excluded	absent	2.56	-1.01	excluded	absent	10.63	-0.84	excluded	absent	8.46	-0.88	excluded	absent	48.33	-0.03	excluded	absent	7.64	-0.9	excluded	absent
243887_at	included	present	292.3	0.36	included	present	470.81	2.09	included	present	551.73	2.87	included	present	349.77	0.92	included	present	246.44	-0.08	included	present	247	-0.07	included	present	660.82	3.92	included	present	580.32	3.14	included	present
243888_at	included	present	132.01	-0.55	included	present	426.25	1.28	included	present	183.05	-0.23	included	present	364.78	0.9	included	present	435.03	1.33	included	present	164.71	-0.35	included	present	745.54	3.26	included	present	109.84	-0.69	included	present
243889_at	excluded	absent	3.65	-0.7	excluded	absent	5.21	-0.66	excluded	absent	4.31	-0.69	excluded	absent	2.99	-0.72	excluded	absent	3.82	-0.7	excluded	absent	4.46	-0.68	excluded	absent	6.16	-0.64	excluded	absent	50.52	0.49	excluded	absent
243890_at	excluded	absent	48.44	-0.77	included	present	186.04	1.31	excluded	absent	2.62	-1.46	excluded	absent	122.21	0.34	excluded	absent	155.8	0.85	included	present	175.36	1.15	included	present	117.72	0.27	excluded	absent	9.41	-1.36	included	present
243891_at	excluded	absent	111.51	0.07	excluded	absent	96.59	-0.11	excluded	absent	98.65	-0.08	excluded	absent	44.88	-0.74	excluded	absent	165.61	0.74	excluded	absent	21.37	-1.03	excluded	absent	126.74	0.26	excluded	absent	18.99	-1.06	excluded	absent
243892_at	excluded	absent	88.32	0.15	excluded	absent	4.73	-0.31	excluded	absent	7.35	-0.29	excluded	absent	23.9	-0.2	excluded	absent	88.89	0.15	excluded	absent	6.25	-0.3	excluded	absent	2.3	-0.32	excluded	absent	3.29	-0.31	excluded	absent
243893_at	excluded	absent	142.83	-0.21	included	present	235.01	0.41	excluded	absent	82.07	-0.62	included	present	319.86	0.98	included	present	279.55	0.71	excluded	absent	126.44	-0.32	included	present	236.74	0.42	included	present	654.92	3.24	included	present
243894_at	included	present	416.65	0.33	included	present	391.76	0.26	included	present	192.13	-0.32	included	present	236.39	-0.19	included	present	433.55	0.38	included	present	336.33	0.1	included	present	529.8	0.66	included	present	435.31	0.38	included	present
243895_x_at	included	present	1137.63	-1.15	included	present	1570.95	-0.35	included	present	972.58	-1.46	included	present	1578.69	-0.34	included	present	1845.27	0.15	included	present	1117.03	-1.19	included	present	739.81	-1.89	included	present	238.26	-2.82	included	present
243896_at	included	present	81.35	-0.26	excluded	absent	44.97	-0.3	excluded	absent	5.36	-0.35	included	present	184.93	-0.14	excluded	absent	6.66	-0.35	excluded	absent	3.53	-0.35	excluded	absent	48.36	-0.3	excluded	absent	50.58	-0.29	excluded	absent
243897_at	excluded	absent	13.3	-1.88	excluded	absent	122.17	0.21	excluded	absent	82.89	-0.55	excluded	absent	33.1	-1.5	excluded	absent	3.62	-2.07	excluded	absent	49.33	-1.19	excluded	absent	143.97	0.63	excluded	absent	40.61	-1.36	included	present
243898_at	excluded	absent	92.43	-1.08	excluded	absent	14.99	-1.98	excluded	absent	12.58	-2.01	excluded	absent	97.61	-1.02	excluded	absent	247.25	0.73	excluded	absent	44.71	-1.63	excluded	absent	169.67	-0.18	excluded	absent	81.73	-1.2	included	present
243899_at	included	present	610.08	2.57	included	present	309.31	0.38	included	present	1435.72	8.58	included	present	777.54	3.79	included	present	1068.43	5.91	included	absent	198.93	-0.42	included	present	699.82	3.22	included	present	324.44	0.49	included	present
243900_at	excluded	absent	13.36	-1.2	excluded	absent	100.33	0.75	excluded	absent	71.77	0.11	excluded	absent	32.58	-0.77	excluded	absent	84.53	0.4	excluded	absent	36.56	-0.68	excluded	absent	32.59	-0.77	excluded	absent	24.37	-0.95	excluded	absent
243901_at	excluded	absent	37.78	-0.26	excluded	absent	1.98	-0.32	excluded	absent	69.74	-0.21	excluded	absent	5.4	-0.31	excluded	absent	151.07	-0.08	excluded	absent	67.77	-0.21	excluded	absent	47.59	-0.24	excluded	absent	1.32	-0.32	excluded	absent
243902_at	included	absent	165.63	-0.56	included	present	145.8	-0.66	included	present	501.26	1.14	included	present	122.7	-0.78	included	present	746.64	2.38	included	absent	207.39	-0.35	included	present	261.23	-0.07	included	absent	44.63	-1.17	included	present
243903_at	excluded	absent	75.91	0.35	excluded	absent	19.34	-1.07	excluded	absent	3.87	-1.46	excluded	absent	60.75	-0.03	excluded	absent	87.82	0.65	included	present	189.83	3.21	included	present	218.59	3.93	excluded	absent	24.56	-0.94	included	present
243904_at	included	absent	123.84	-0.74	included	absent	3.82	-2.08	included	present	134.93	-0.62	included	present	179.1	-0.13	included	absent	68.75	-1.36	included	present	155.44	-0.39	included	present	179.17	-0.13	included	present	109.76	-0.9	included	present
243905_at	excluded	absent	1.98	-0.63	excluded	absent	56.2	1.16	excluded	absent	5.99	-0.5	excluded	absent	2.52	-0.61	excluded	absent	3.21	-0.59	excluded	absent	6.69	-0.48	excluded	absent	81.85	2.01	excluded	absent	2.14	-0.63	excluded	absent
243906_at	excluded	absent	85.02	0	excluded	absent	103.06	0.34	excluded	absent	2.72	-1.56	excluded	absent	10.12	-1.42	excluded	absent	62.07	-0.43	excluded	absent	115.73	0.58	excluded	absent	37.73	-0.89	excluded	absent	57.89	-0.51	excluded	absent
243907_at	included	absent	2.61	-1.82	included	absent	78.36	-0.95	included	absent	78.7	-0.94	included	present	487.97	3.77	included	absent	121.75	-0.45	included	absent	72.76	-1.01	included	present	63.16	-1.12	included	present	82.25	-0.9	included	present
243908_at	excluded	absent	38.45	-1.22	excluded	absent	3.12	-1.97	included	present	87.48	-0.19	excluded	absent	1.73	-2	excluded	absent	81.17	-0.32	excluded	absent	78.1	-0.39	included	present	45.77	-1.07	included	present	54.57	-0.88	included	present
243909_x_at	excluded	absent	46.42	-0.15	excluded	absent	65.13	0.36	excluded	absent	136.57	2.3	excluded	absent	67.21	0.42	excluded	absent	1.45	-1.37	excluded	absent	1.98	-1.35	included	present	109.71	1.57	included	present	67.15	0.42	excluded	absent
243910_x_at	included	present	333.18	1.34	included	present	172.5	-0.64	included	present	935.51	8.79	included	present	861.19	7.87	included	present	390.5	2.05	included	present	226.12	0.14	included	present	534.77	3.84	included	present	204.57	-0.25	included	present
243911_at	included	present	167.78	-0.28	included	absent	65.5	-1.64	included	present	92.92	-1.28	included	absent	104.41	-1.13	included	absent	146.23	-0.57	included	present	192.88	0.05	included	present	164.42	-0.33	included	absent	60.21	-1.71	included	present
243912_x_at	included	present	219.17	0.61	excluded	absent	8.88	-1.93	excluded	absent	163.14	-0.07	excluded	absent	128.67	-0.48	excluded	absent	248.64	0.96	excluded	absent	340.59	2.07	included	present	161.07	-0.09	included	present	244.99	0.92	included	present
243913_at	excluded	absent	16.72	-0.46	excluded	absent	58.22	-0.15	excluded	absent	105.59	0.2	included	present	112.95	0.26	excluded	absent	93.65	0.11	excluded	absent	33.52	-0.33	included	present	155.54	0.57	excluded	absent	27.34	-0.38	excluded	absent
243914_at	excluded	absent	99.91	0.27	excluded	absent	231.24	2.75	excluded	absent	121.11	0.67	excluded	absent	74.08	-0.22	excluded	absent	22.93	-1.19	excluded	absent	174.66	1.68	excluded	absent	129.19	0.82	excluded	absent	69.51	-0.31	excluded	absent
243915_at	included	present	136.8	-0.51	included	absent	87.57	-0.77	included	present	173.02	-0.31	included	absent	82.25	-0.8	included	present	148.95	-0.44	included	present	146.35	-0.45	included	present	125.38	-0.57	included	present	112.68	-0.64	included	present
243916_x_at	included	present	859.62	0.42	included	present	936.52	0.68	included	present	1392.75	2.2	included	present	1118.09	1.28	included	present	978.65	0.82	included	present	1337.45	2.02	included	present	1066.46	1.11	included	present	1859.35	3.77	included	present
243917_at	excluded	absent	30.69	-0.24	excluded	absent	10.29	-0.37	excluded	absent	1.62	-0.43	excluded	absent	13.67	-0.35	excluded	absent	3.02	-0.42	excluded	absent	122.22	0.35	excluded	absent	28.72	-0.25	excluded	absent	5.23	-0.41	excluded	absent
243918_at	excluded	absent	63.7	-0.81	included	present	334.52	2.51	excluded	absent	38.28	-1.13	excluded	absent	127.68	-0.03	excluded	absent	143.99	0.17	excluded	absent	132.54	0.03	included	present	391.93	3.22	included	present	241.69	1.37	included	present
243919_at	included	present	98.91	-0.76	included	absent	7.12	-2.09	included	present	136.69	-0.21	included	absent	105.25	-0.67	excluded	absent	149.59	-0.02	excluded	absent	117.29	-0.49	excluded	absent	59.2	-1.33	included	absent	34.51	-1.69	included	present
243920_x_at	excluded	absent	80.5	-0.93	excluded	absent	7.62	-1.76	included	present	324.96	1.85	excluded	absent	190.54	0.32	excluded	absent	160.78	-0.02	excluded	absent	119.92	-0.49	included	present	479.49	3.6	included	present	127.05	-0.4	included	present

[illegible]

244240_at	excluded	absent	1.54	-0.35	excluded	absent	29.51	-0.14	excluded	absent	36.98	-0.08	excluded	absent	3.98	-0.33	[illegible]
244241_x_at	excluded	absent	49.13	-0.7	included	present	143.19	0.57	excluded	absent	40.33	-0.82	excluded	absent	119.7	0.26	[illegible]
244242_at	included	present	144.7	-0.13	included	absent	68.64	-1.39	included	absent	136.68	-0.26	included	absent	78.57	-1.23	[illegible]
244243_at	excluded	absent	3.9	-0.95	excluded	absent	68.52	0.8	excluded	absent	2.26	-1	excluded	absent	2.37	-0.99	[illegible]
244244_at	excluded	absent	5.79	-0.9	excluded	absent	35.93	-0.15	excluded	absent	5.83	-0.9	excluded	absent	33.63	-0.21	[illegible]
244245_at	excluded	absent	3.05	-0.53	excluded	absent	5.34	-0.41	excluded	absent	4.17	-0.47	excluded	absent	1.93	-0.58	[illegible]
244246_at	included	present	108.83	-0.54	excluded	absent	16.12	-1.75	excluded	absent	59.43	-1.18	included	present	571.19	5.47	[illegible]
244247_at	included	present	327.97	-0.71	included	present	167.92	-1.3	included	present	504.92	-0.06	included	present	1799.43	4.69	[illegible]
244248_at	excluded	absent	43.1	-1.04	excluded	absent	50.59	-0.95	included	present	114.14	-0.15	included	present	81.02	-0.56	[illegible]
244249_at	included	present	144.41	-0.57	included	absent	111.23	-0.75	included	absent	40.99	-1.15	included	present	115.94	-0.73	[illegible]
244250_at	excluded	absent	71.43	-0.45	excluded	absent	19.69	-1.06	included	present	175.76	0.78	excluded	absent	15.76	-1.11	[illegible]
244251_at	included	present	87.1	0.26	excluded	absent	37.05	-0.42	excluded	absent	47.07	-0.28	excluded	absent	20.96	-0.64	[illegible]
244252_at	excluded	absent	19.19	-0.76	excluded	absent	26.14	-0.57	excluded	absent	5.14	-1.14	excluded	absent	4.32	-1.16	[illegible]
244253_at	excluded	absent	3.18	-0.63	excluded	absent	6.6	-0.5	excluded	absent	1.84	-0.67	excluded	absent	9.13	-0.41	[illegible]
244254_at	included	present	118.03	-0.6	included	absent	91.86	-0.75	included	present	318.22	0.52	included	present	342.28	0.66	[illegible]
244255_at	excluded	absent	2.83	-0.36	excluded	absent	11.36	-0.33	excluded	absent	27.44	-0.28	included	present	256.63	0.48	[illegible]
244256_at	included	present	343.08	-0.51	included	present	1728.8	-0.08	excluded	absent	11.98	-0.62	excluded	absent	23.75	-0.61	[illegible]
244257_at	excluded	absent	5.02	-0.37	excluded	absent	7.32	-0.21	excluded	absent	58.65	3.33	excluded	absent	15.58	0.36	[illegible]
244258_at	excluded	absent	44.61	-0.42	excluded	absent	83.34	0.4	excluded	absent	44.91	-0.42	excluded	absent	89.87	0.54	[illegible]
244259_s_at	included	present	348.39	0.41	included	present	253.89	-0.02	included	present	137.97	-0.55	included	present	447.84	0.86	[illegible]
244260_at	excluded	absent	66.52	-0.58	excluded	absent	10.21	-0.95	excluded	absent	3.06	-1	excluded	absent	11.37	-0.94	[illegible]
244261_at	excluded	absent	5.54	-0.81	excluded	absent	1.96	-0.83	excluded	absent	33.09	-0.69	included	present	288.69	0.47	[illegible]
244262_x_at	excluded	absent	35.31	-0.32	excluded	absent	2.63	-1.1	excluded	absent	41.74	-0.17	excluded	absent	19.53	-0.69	[illegible]
244263_at	excluded	absent	2.77	-1.02	excluded	absent	6.35	-0.93	excluded	absent	82.53	0.98	excluded	absent	2.95	-1.02	[illegible]
244264_at	excluded	absent	3.04	-0.9	excluded	absent	58.13	1.44	excluded	absent	42.81	0.79	excluded	absent	35.4	0.48	[illegible]
244265_at	excluded	absent	15.56	-0.82	excluded	absent	3.24	-1.1	excluded	absent	40.72	-0.24	included	present	98.99	1.09	[illegible]
244266_at	excluded	absent	38.56	0.41	excluded	absent	80.52	2.05	excluded	absent	45.21	0.67	excluded	absent	54.8	1.05	[illegible]
244267_at	excluded	absent	127.16	0.65	excluded	absent	67.01	-0.09	excluded	absent	54.02	-0.25	excluded	absent	31.12	-0.53	[illegible]
244268_x_at	included	present	238.84	-0.18	included	present	224.03	-0.3	included	present	279.24	0.16	included	present	266.84	0.05	[illegible]
244269_at	excluded	absent	3.75	-0.98	excluded	absent	89.32	1.11	excluded	absent	19.72	-0.59	excluded	absent	42.58	-0.03	[illegible]
244270_at	included	absent	150.99	-0.65	included	absent	112.84	-0.83	included	present	333.37	0.2	included	present	420.47	0.61	[illegible]
244271_at	included	present	152.53	-1	included	present	408.48	0.68	included	present	199.39	-0.69	included	present	593.59	1.9	[illegible]
244272_s_at	included	present	238.54	-0.67	included	absent	33.57	-0.86	included	present	212.61	-0.69	included	present	172.72	-0.73	[illegible]
244273_at	excluded	absent	42.02	-0.44	excluded	absent	66.71	-0.25	excluded	absent	117.22	0.13	excluded	absent	86.94	-0.1	[illegible]
[illegible]																	

[illegible]	14.87	-0.53	excluded	absent	5.55	-0.58	excluded	absent	66.98	-0.25	excluded	absent	45.77	-0.36
[illegible]	46.31	-0.73	included	present	92.55	-0.1	excluded	absent	74.78	-0.34	excluded	absent	73.66	-0.36
[illegible]	12.83	-0.64	excluded	absent	3.24	-0.86	excluded	absent	3.73	-0.85	excluded	absent	53.23	0.3
[illegible]	75.37	-0.26	excluded	absent	61.37	-0.51	included	present	138.26	0.88	included	present	99.69	0.18
[illegible]	12.66	-0.67	excluded	absent	5.76	-0.79	excluded	absent	72.52	0.36	excluded	absent	103.18	0.89
[illegible]	65.83	-0.97	excluded	absent	68.17	-0.93	excluded	absent	96.55	-0.43	included	present	119.36	-0.02
[illegible]	40.67	-1.72	included	absent	82.41	-1.31	included	absent	80.29	-1.34	included	absent	60.45	-1.53
[illegible]	98.84	-0.1	excluded	absent	5.87	-1.15	excluded	absent	57.08	-0.57	excluded	absent	11.86	-1.09
[illegible]	191.72	-0.6	excluded	absent	171.53	-0.8	excluded	absent	185.74	-0.66	excluded	absent	204.22	-0.48
[illegible]	70.09	0.43	included	present	129	1.34	excluded	absent	2.1	-0.62	excluded	absent	2.78	-0.61
[illegible]	321.26	0.73	included	absent	171.07	-0.73	included	present	589.17	3.34	included	present	265.71	0.19
[illegible]	1264.02	0.71	included	present	280.72	-1.43	included	present	376.29	-1.22	included	present	164.88	-1.68
[illegible]	105.62	-0.87	included	absent	7.31	-2.11	included	present	162.99	-0.14	included	absent	122.08	-0.66
[illegible]	387.18	4.09	excluded	absent	78.23	-0.61	included	present	219.09	1.53	excluded	absent	44.25	-1.12
[illegible]	17.51	-0.47	excluded	absent	4.04	-0.86	excluded	absent	3.38	-0.88	excluded	absent	16.03	-0.51
[illegible]	160.57	-0.44	included	absent	8.34	-2.12	included	present	158.51	-0.46	included	present	148.35	-0.57
[illegible]	21.57	-0.77	excluded	absent	35.24	-0.51	excluded	absent	12.94	-0.94	excluded	absent	5.83	-1.08
[illegible]	386.16	-0.04	included	present	338.69	-0.24	included	present	718.37	1.35	included	present	408.69	0.05
[illegible]	108.36	-0.39	excluded	absent	18.79	-0.47	excluded	absent	87.03	-0.41	excluded	absent	30.18	-0.46
[illegible]	80.37	-0.3	excluded	absent	72.23	-0.39	excluded	absent	1.38	-1.23	excluded	absent	75.71	-0.35
[illegible]	19.31	-0.3	excluded	absent	38.12	0.31	excluded	absent	1.75	-0.86	excluded	absent	2.08	-0.85
[illegible]	139.36	-0.79	included	present	337.68	0.44	included	present	348.04	0.51	included	absent	129.5	-0.85
[illegible]	84.43	-0.54	excluded	absent	59.4	-0.88	excluded	absent	228.21	1.39	excluded	absent	104.35	-0.28
[illegible]	156.84	-0.96	included	present	191.54	-0.66	included	present	203.41	-0.56	included	present	309.46	0.36
[illegible]	112.86	-0.48	included	present	1867.18	3.02	included	present	864.91	1.02	excluded	absent	35.92	-0.64
[illegible]	18.14	-0.88	excluded	absent	15.24	-0.94	excluded	absent	120.85	1.33	excluded	absent	79.15	0.43
[illegible]	87.6	-1.08	included	absent	46.48	-1.43	included	present	119.34	-0.82	included	present	87.48	-1.09
[illegible]	5.24	-0.74	excluded	absent	5.89	-0.73	excluded	absent	50.76	0.02	excluded	absent	5.17	-0.74
[illegible]	96.82	0.78	excluded	absent	85.1	0.52	excluded	absent	28.08	-0.79	included	present	68.22	0.13
[illegible]	157.56	-0.46	included	absent	32	-1.79	included	present	210.37	0.1	included	present	193.43	-0.08
[illegible]	57.91	0.74	excluded	absent	29.01	-0.24	excluded	absent	76.19	1.36	excluded	absent	40.49	0.15
[illegible]	104.62	0.93	excluded	absent	6.33	-1.13	excluded	absent	73.59	0.28	excluded	absent	48.96	-0.24
[illegible]	330.89	-0.78	included	present	1628.4	2.89	included	present	608.8	0.01	included	present	551.94	-0.15
[illegible]	2.27	-1.03	excluded	absent	83.41	0.26	excluded	absent	58.37	-0.14	excluded	absent	12.1	-0.88
[illegible]	246.98	-1.34	included	present	141.98	-2.01	included	present	365.13	-0.58	included	present	947.33	3.15
[illegible]	52.12	-1.09	excluded	absent	15.08	-1.35	included	present	122.12	-0.61	excluded	absent	30.69	-1.24
[illegible]	166.77	-1.4	included	present	732.18	-0.25	included	present	758.6	-0.2	included	present	994.38	0.28
[illegible]	128.35	0.29	excluded	absent	1.43	-1.33	excluded	absent	95.7	-0.13	excluded	absent	39.22	-0.85
[illegible]	46.36	-1.15	excluded	absent	38.08	-1.31	excluded	absent	116.75	0.24	excluded	absent	34.69	-1.38
[illegible]	78.82	-0.58	excluded	absent	66.93	-0.71	excluded	absent	42.82	-0.96	included	present	85.19	-0.52
[illegible]	95.66	-1.13	included	absent	10.67	-2.39	included	present	74	-1.45	included	present	141.66	-0.45
[illegible]	62.29	0.08	excluded	absent	105.88	1.22	included	present	167.67	2.83	included	present	268.17	5.46

[illegible]

34689_at	excluded	absent	106.07	-1.13	excluded	absent	31.01	-1.88	excluded	absent	104.78	-1.14	excluded	absent	170.69	-0.48
34697_at	included	present	236.46	-0.49	included	present	185.06	-0.93	included	present	282.81	-0.1	included	present	296.72	0.02
34726_at	included	present	809.9	-0.05	included	present	2459.05	2.64	included	present	496.98	-0.56	included	present	989.94	0.24
34764_at	included	present	392.63	-0.9	included	present	1336.22	2.41	included	present	369.88	-0.98	included	present	327.12	-1.13
34846_at	excluded	absent	39.38	-0.25	excluded	absent	21.57	-0.63	excluded	absent	85.87	0.74	excluded	absent	67.58	0.35
34858_at	included	present	1945.66	4	included	present	1691.8	3.22	included	present	574.53	-0.23	included	present	536.1	-0.35
34868_at	included	present	373.44	-0.78	included	present	603.94	1.04	included	present	360.63	-0.88	included	present	356.99	-0.91
35147_at	included	absent	193.54	-0.84	included	present	570.87	0.63	included	present	303.81	-0.41	included	present	462.65	0.21
35148_at	excluded	absent	9.1	-0.49	excluded	absent	14.02	-0.47	excluded	absent	7.24	-0.5	excluded	absent	100.28	-0.13
35150_at	included	present	170.45	0.7	excluded	absent	91.03	-0.36	excluded	absent	50.05	-0.92	excluded	absent	114.23	-0.05
35156_at	included	present	411.94	-0.65	included	present	554.02	0.41	included	present	497.18	-0.01	included	present	397.57	-0.76
35160_at	included	present	198.67	-0.33	included	present	311.52	0.48	included	present	209.85	-0.25	included	present	215.32	-0.21
35179_at	included	present	813.54	1.15	included	present	864.8	1.5	included	present	781.11	0.93	included	present	375.77	-1.83
35201_at	included	present	3811.04	-0.46	included	present	4178.73	-0.05	included	present	7813.55	3.93	included	present	8161.86	4.32
35254_at	included	present	1014.8	0.34	included	present	1530.33	1.74	included	present	878.75	-0.03	included	present	1012.37	0.33
35265_at	included	present	627.66	1.32	included	present	497.71	0.71	included	present	230.55	-0.55	included	present	243.55	-0.48
35436_at	included	present	425.61	1.72	included	present	187.32	-0.35	included	present	331.57	0.9	included	present	323.92	0.83
35617_at	included	absent	142.3	-0.42	included	present	443.55	3.03	included	present	189.74	0.12	included	present	203.36	0.28
35626_at	included	present	1230.14	3	included	present	1240.08	3.04	included	present	665.13	0.81	included	present	827.51	1.44
35666_at	included	present	288.95	-0.65	included	present	1771.59	0.21	included	present	677.29	-0.42	included	absent	208.13	-0.7
35671_at	included	present	248.59	-0.63	included	present	417.27	0.92	included	absent	69.14	-2.28	included	present	304.9	-0.12
35685_at	included	present	1197.71	-0.89	included	present	4435.24	7.76	included	present	1330.23	-0.53	included	present	1751.9	0.59
35776_at	included	present	1213.9	1.53	included	present	871.74	0.26	included	present	415.57	-1.43	included	present	473.33	-1.21
35820_at	included	present	1361.96	0.05	included	present	1876.48	0.71	included	present	925.9	-0.51	included	present	243.26	-1.39
35846_at	included	present	233.49	-0.7	included	present	271.64	-0.59	included	present	154.57	-0.95	included	present	191.59	-0.83
35974_at	included	present	114.05	-0.3	included	absent	48.93	-0.44	included	absent	77.49	-0.38	included	present	74.95	-0.39
36004_at	excluded	absent	200.02	0.81	excluded	absent	171.58	0.42	excluded	absent	81.07	-0.81	excluded	absent	60.8	-1.09
36019_at	included	present	465.74	-0.49	included	present	1213.54	5.65	included	present	375.71	-1.23	included	present	597.86	0.6
36030_at	included	present	1308.03	-0.05	included	present	1339.35	0.02	included	present	630.1	-1.73	included	present	542.02	-1.95
36084_at	included	present	221.05	0.43	included	present	430.26	3.11	included	present	186.98	0	included	absent	115.37	-0.92
36129_at	included	present	395.92	-0.74	included	present	1239.3	1.58	included	absent	232.97	-1.19	included	present	411.57	-0.7
36475_at	excluded	absent	154.53	0.21	excluded	absent	128.87	-0.11	excluded	absent	112.65	-0.31	excluded	absent	27.95	-1.37
36499_at	included	present	532.78	-0.41	included	present	854.15	0.02	included	present	210.6	-0.84	included	present	460.52	-0.51
36545_s_at	excluded	absent	8.37	-0.85	excluded	absent	36.01	-0.17	excluded	absent	34.41	-0.21	excluded	absent	110.86	1.67

64899_at	included	present	177.43	-0.39	included	present	137.7	-0.68	included	absent	68.66	-1.17	included	absent	75.09	-1.12	included	absent	174.85	-0.41	included	present	310.28	0.55	included	present	533.57	2.15	included	present	332.86	0.72	included	present	407.08	1.24	included	present	508.1	1.96	included	present	161.07	-0.51	included	present	215.26	-0.12	included	absent	59.43	-1.23	included	absent	17.58	-1.53	included	present	641.86	2.92
64900_at	included	present	164.73	-0.25	included	present	161.77	-0.27	included	present	124.54	-0.59	included	present	177.17	-0.15	included	absent	112.93	-0.68	included	present	195.07	0	included	present	203.64	0.08	included	present	381.38	1.56	included	present	243.32	0.41	included	present	141.03	-0.45	included	present	265.23	0.59	included	present	86.11	-0.91	included	present	404.25	1.76	included	present	261.85	0.56	included	present	143.67	-0.43
64942_at	included	present	480.41	3.1	included	present	1053.29	9.48	included	absent	103.03	-1.1	included	present	173.61	-0.31	included	present	594.49	4.38	included	present	562.02	4.01	included	present	986.19	8.74	included	present	1165.89	10.74	included	present	524.24	3.59	included	present	1625.6	15.86	included	present	462.44	2.9	included	present	253.44	0.58	included	present	274.44	0.81	included	present	205.58	0.04	included	present	988.27	8.76
65086_at	included	present	160.32	0.14	included	present	118.59	-0.41	excluded	absent	93.63	-0.75	excluded	absent	108.83	-0.54	excluded	absent	78.56	-0.95	included	present	436.06	3.82	included	present	224.01	0.99	included	present	270.86	1.62	included	present	248.61	1.32	included	present	290.07	1.88	included	present	168.66	0.25	included	present	334.1	2.46	excluded	absent	56.69	-1.24	excluded	absent	61.15	-1.18	included	present	242.06	1.23
65133_i_at	excluded	absent	5.47	-1.22	excluded	absent	40.99	-0.5	excluded	absent	96.23	0.62	excluded	absent	34.66	-0.63	excluded	absent	5.44	-1.22	excluded	absent	24.83	-0.83	excluded	absent	18.96	-0.95	excluded	absent	53.68	-0.24	excluded	absent	111.6	0.93	excluded	absent	70.42	0.1	excluded	absent	50.98	-0.3	excluded	absent	33.07	-0.66	excluded	absent	7.1	-1.19	excluded	absent	5.04	-1.23	excluded	absent	88.32	0.46
65438_at	included	present	520.89	2.11	included	present	530.51	2.21	included	present	301.11	-0.14	included	present	460.11	1.49	included	present	222.91	-0.94	included	present	409.63	0.97	included	present	496.55	1.86	included	present	503.94	1.94	included	present	403.79	0.91	included	present	576.7	2.68	included	present	154.34	-1.64	included	present	759.84	4.56	included	present	319.62	0.05	included	present	463.2	1.52	included	present	408.14	0.95
65472_at	included	present	292	-0.06	included	present	539.66	1.33	included	present	503.5	1.13	included	present	716.87	2.33	included	present	307.35	0.03	included	present	249.19	-0.3	included	present	698.8	2.23	included	present	504.93	1.14	included	present	383.34	0.45	included	present	352.11	0.28	included	present	434.44	0.74	included	present	499.67	1.11	included	present	892.12	3.32	included	present	1340.34	5.84	included	present	359.63	0.32
65493_at	included	present	371.67	1.5	included	present	668.44	5.49	included	present	549.89	3.9	included	present	474.44	2.88	included	present	563.9	4.08	included	present	268.01	0.1	included	present	619.14	4.83	included	present	615.22	4.77	included	present	496.5	3.18	included	present	571.11	4.18	included	present	255.31	-0.07	included	present	206.89	-0.72	included	present	533.78	3.68	included	present	562.87	4.07	included	present	848.84	7.92
65517_at	excluded	absent	56.27	-0.49	excluded	absent	5.04	-0.64	excluded	absent	72.21	-0.45	included	present	299.45	0.22	excluded	absent	11.85	-0.62	excluded	absent	31.31	-0.57	excluded	absent	10.9	-0.63	excluded	absent	50.17	-0.51	excluded	absent	57.45	-0.49	excluded	absent	33.47	-0.56	excluded	absent	5.82	-0.64	excluded	absent	56.17	-0.49	excluded	absent	58.82	-0.49	excluded	absent	21.86	-0.59	excluded	absent	4.94	-0.64
65521_at	included	present	234.9	1.21	included	present	158.57	0.11	included	present	247.19	1.39	included	present	187.79	0.53	excluded	absent	148.14	-0.04	included	present	381.09	3.32	included	present	349.48	2.87	included	present	373.18	3.21	included	present	292.62	2.05	included	present	619.99	6.77	included	present	555.51	5.84	included	present	150.88	0	included	present	231.6	1.17	included	present	108.81	-0.6	included	present	421.07	3.9
65585_at	included	present	332.89	0	included	present	364.27	0.17	included	present	244.21	-0.48	included	absent	62.34	-1.47	included	present	311.1	-0.12	included	present	297.11	-0.2	included	present	724.68	2.12	included	present	978.61	3.5	included	present	506.54	0.94	included	present	642.82	1.68	included	present	741.17	2.21	included	present	828.3	2.69	included	present	427.96	0.51	included	present	227.91	-0.57	included	present	509.56	0.96
65588_at	included	present	404.65	-0.05	included	present	631.6	1.26	included	present	553.59	0.81	included	present	389.27	-0.14	included	present	388.57	-0.14	included	present	677.14	1.52	included	present	336.54	-0.44	included	present	891.98	2.77	included	present	341.46	-0.42	included	present	312.14	-0.59	included	present	626.99	1.23	included	present	310.13	-0.6	included	present	229.66	-1.06	included	present	210.51	-1.17	included	present	443.18	0.17
65591_at	included	present	305.12	1.03	included	present	216.39	-0.06	included	present	264.07	0.53	included	present	260.06	0.48	included	present	261.34	0.49	included	absent	163.14	-0.72	included	present	342.97	1.5	included	present	413.52	2.37	included	present	361.17	1.72	included	present	266.61	0.56	included	present	416.98	2.41	included	present	134.95	-1.07	included	absent	96.95	-1.54	included	absent	56.19	-2.04	included	present	341.49	1.48
65630_at	included	present	307.75	0.27	included	present	192.26	-0.87	included	present	78.61	-1.98	included	present	277.35	-0.03	included	present	267.78	-0.13	included	present	393.48	1.11	included	present	359.28	0.77	included	present	156.45	-1.22	included	present	293.98	0.13	included	present	294.87	0.14	included	present	250.11	-0.3	included	present	288.15	0.07	included	present	261.64	-0.19	included	present	369.69	0.87	included	present	290.86	0.1
65635_at	included	present	473.6	-0.11	included	present	253.78	-0.86	included	present	282.55	-0.77	included	present	493.82	-0.05	included	present	375.25	-0.45	included	present	807.09	1.02	included	present	448.7	-0.2	included	present	622.45	0.39	included	present	697.02	0.65	included	present	462.53	-0.15	included	present	465.39	-0.14	included	present	755.52	0.85	included	present	433.32	-0.25	included	present	205.77	-1.03	included	present	601.56	0.32
65718_at	included	present	784.74	0.97	included	present	451.74	-0.24	included	present	376.68	-0.51	included	present	453.08	-0.23	included	present	371.38	-0.53	included	present	971.56	1.64	included	present	435.39	-0.3	included	present	381.8	-0.49	included	present	966.62	1.63	included	present	311.64	-0.74	included	present	464.54	-0.19	included	present	590.69	0.27	included	present	675.44	0.57	included	present	1062.36	1.97	included	present	372.88	-0.52
65770_at	included	present	522.34	0.46	included	present	916.32	2.89	included	absent	302.62	-0.9	included	present	332.44	-0.72	included	present	373.97	-0.46	included	present	472.86	0.15	included	present	468.22	0.12	included	present	961.22	3.17	included	present	512.56	0.4	included	present	626.86	1.1	included	present	464.75	0.1	included	present	420.2	-0.17	included	absent	280.85	-1.04	included	present	284.03	-1.02	included	present	601.94	0.95
65884_at	included	present	209.32	-0.27	included	present	161.81	-0.78	included	present	236.69	0.02	included	absent	106.64	-1.37	excluded	absent	265.16	0.33	included	present	514.19	2.99	included	present	277.96	0.46	included	present	303.51	0.74	included	present	252.84	0.19	included	present	359.65	1.34	included	present	435.49	2.15	included	absent	120.45	-1.22	included	absent	31.44	-2.17	included	absent	64.91	-1.81	included	present	347.66	1.21
66053_at	included	present	2161.15	2.55	included	present	2018.2	2.15	included	present	2944.53	4.71	included	present	1795.28	1.54	included	present	2416.08	3.25	included	present	1169.49	-0.19	included	present	1007.48	-0.64	included	present	1409.22	0.47	included	present	2001.52	2.11	included	present	1214.78	-0.07	included	present	1767.15	1.46	included	present	1420.77	0.5	included	present	492.35	-2.06	included	present	808.86	-1.19	included	present	1605.27	1.01
71933_at	excluded	absent	27.56	-0.41	excluded	absent	46.81	0.06	excluded	absent	52.28	0.19	excluded	absent	4.45	-0.97	excluded	absent	3.67	-0.99	excluded	absent	3.66	-0.99	excluded	absent	14.18	-0.73	excluded	absent	32.26	-0.29	excluded	absent	2.75	-1.01	excluded	absent	28.7	-0.38	excluded	absent	45.03	0.02	excluded	absent	2.33	-1.02	excluded	absent	10.09	-0.83	excluded	absent	22.09	-0.54	excluded	absent	2.47	-1.02
74694_s_at	excluded	absent	90.78	0.15	excluded	absent	41.29	-1.13	excluded	absent	9.91	-1.94	excluded	absent	45.58	-1.02	excluded	absent	3.7	-2.1	included	present	190.55	2.72	included	present	107.93	0.59	included	present	138.65	1.38	included	present	103.02	0.46	included	present	58.16	-0.7	excluded	absent	36.25	-1.26	included	present	367.19	7.28	included	present	156.4	1.84	included	present	156.62	1.85	included	present	82.35	-0.07
76897_s_at	included	present	206.06	1.01	included	present	84.67	-0.84	included	present	194.3	0.83	included	present	68.5	-1.09	included	present	251.13	1.7	included	present	344.36	3.12	included	present	222.81	1.27	included	present	273.67	2.04	included	present	406.03	4.06	included	present	364.82	3.43	included	present	477.89	5.16	included	absent	84.68	-0.84	included	present	119.75	-0.31	included	present	66.29	-1.12	included	present	355.21	3.29
77508_r_at	excluded	absent	25.74	-0.47	excluded	absent	63.54	0.48	excluded	absent	52.57	0.2	excluded	absent	11.96	-0.82	excluded	absent	82.43	0.96	excluded	absent	25.07	-0.49	excluded	absent	63.83	0.49	excluded	absent	39.45	-0.13	excluded	absent	59.69	0.38	excluded	absent	16.65	-0.7	excluded	absent	65.62	0.53	excluded	absent	14.28	-0.76	excluded	absent	89.75	1.14	excluded	absent	18.36	-0.66	excluded	absent	16.07	-0.72
78047_s_at	included	present	306.92	0.42	included	present	270.21	0.08	included	absent	180.84	-0.75	included	absent	161.42	-0.93	included	present	236.63	-0.23	included	present	477.9	2.02	included	present	432.97	1.6	included	absent	211.02	-0.47	included	present	225.53	-0.34	included	present	376.59	1.07	included	absent	196.91	-0.6	included	present	278.34	0.16	included	absent	142.19	-1.11	included	absent	100.27	-1.5	included	present	331.89	0.66
78330_at	excluded	absent	10.59	-0.67	excluded	absent	98.05	1.65	excluded	absent	31.53	-0.11	excluded	absent	60.09	0.65	excluded	absent	44.47	0.23	excluded	absent	84.21	1.29	excluded	absent	63.49	0.74	excluded	absent	14.17	-0.57	excluded	absent	7.73	-0.74	excluded	absent	52.22	0.44	excluded	absent	80.99	1.2	excluded	absent	16.08	-0.52	excluded	absent	8.09	-0.73	excluded	absent	27.98	-0.2	excluded	absent	11.12	-0.65
78383_at	included	present	208.1	-1.12	included	present	165.76	-1.61	included	present	181.53	-1.43	included	present	165.53	-1.62	included	absent	97.3	-2.42	included	present	522.36	2.57	included	present	864.47	6.59	included	present	371.75	0.8	included	present	433.76	1.53	included	present	671.16	4.32	included	present	178.52	-1.46	included	present	587.52	3.34	included	present	326.2	0.27	included	present	403.9	1.18	included	present	795.06	5.77
78495_at	included	present	329.43	-0.18	included	present	670.46	2.82	included	present	499.41	1.32	included	present	432.19	0.73	included	present	636.18	2.52	included	present	456.94	0.94	included	present	587.28	2.09	included	present	533.46	1.61	included	present	314.4	-0.31	included	present	562.03	1.87	included	present	523.68	1.53	included	present	638.86	2.54	included	present	587.73	2.09	included	present	621.78	2.39	included	present	592.65	2.13
79005_at	included	present	767.49	0.4	included	present	1000.18	1.38	included	present	1020.39	1.47	included	present	1453.55	3.3	included	present	601.01	-0.31	included	present	1157.15	2.04	included	present	1289.77	2.6	included	present	1116.83	1.87	included	present	961.86	1.22	included	present	682.16	0.03	included	present	769.5	0.4	included	present	1297.21	2.64	included	present	729.73	0.24	included	present	744.5	0.3	included	present	819.66	0.62
81737_at	included	present	1536.23	-0.41	included	present	458.62	-1.41	included	present	1857.37	-0.12	included	present	751.38	-1.14	included	present	1413.27	-0.53	included	present	1212.45	-0.71	included	present	1148.14	-0.77	included	present	501.65	-1.37	included	present	2550.48	0.52	included	present	2022.85	0.03	included	present	2810.47	0.76	included	present	619.85	-1.26	included	present	373.25	-1.48	included	present	362.08	-1.49	included	present	684.99	-1.2
81811_at	included	present	439.65	0.71	included	present	781.83	3.82	included	present	464.16	0.94	included	present	465.2	0.95	included	present	427.7	0.61	included	absent	281.57	-0.72	included	present	234.36	-1.15	included	present	228.81	-1.2	included	present	392.8	0.29	included	present	384.77	0.22	included	present	445.11	0.76	included	present	319.31	-0.38	included	present	777.44	3.78	included	present	987.97	5.69	included	present	112.37	-2.26
823_at	included	present	150	-0.72	included	absent	75.46	-1.13	included	absent	100.31	-0.99	included	absent	4.02	-1.51	included	absent	53.62	-1.24	included	present	387.15	0.55	included	present	222.92	-0.33	included	present	112.51	-0.93	included	present	310.61	0.14	included	present	116.52	-0.9	included	present	258.74	-0.14	included	present	151.95	-0.71	included	absent	85.01	-1.07	included	absent	18.73	-1.43	included	present	172.82	-0.6
87100_at	included	present	2672.27	0.98	included	present	1017.06	-0.52	included	present	1261.85	-0.3	included	present	3623.33	1.85	included	present	716.14	-0.8	included	present	1777.52	0.17	included	present	1179.74	-0.38	included	present	2469.56	0.8	included	present	1767.16	0.16	included	present	1222.72	-0.34	included	present	2379.33	0.72	included	present	1526.34	-0.06	included	present	2145.39	0.5	included	present	1383.44	-0.19	included	present	1841.89	0.23
89476_r_at	included	present	248.78	1.02	excluded	absent	101.71	-0.65	excluded	absent	18.46	-1.59	excluded	absent	27.16	-1.49	excluded	absent	27.5	-1.49	excluded	absent	34.72	-1.41	excluded	absent	115.65	-0.49	included	present	204.91	0.52	excluded	absent	217.4	0.66	included	present	148.27	-0.12	excluded	absent	29.73	-1.46	included	present	302.9	1.63	excluded	absent	12.28	-1.66	included	present	130.2	-0.32	excluded	absent	144.3	-0.16
89948_at	excluded	absent	31.2	-1.56	excluded	absent	117.12	0.38	excluded	absent	19.73	-1.82	excluded	absent	84.32	-0.36	excluded	absent	31.28	-1.56	excluded	absent	131.38	0.7	included	present	181.02	1.83	included	present	163.87	1.44	included	present	202.37	2.31	excluded	absent	88.44	-0.27	included	present	117.51	0.39	excluded	absent	97.54	-0.06	excluded	absent	116.85	0.38	included	present	111.99	0.27	included	present	190.35	2.04
89977_at	excluded	absent	91.23	-0.18	excluded	absent	34.63	-0.23	excluded	absent	32.11	-0.23	excluded	absent	2.84	-0.25	excluded	absent	34.9	-0.23	excluded	absent	65.43	-0.2	excluded	absent	7.51	-0.25	excluded	absent	3.89	-0.25	included	present	147.48	-0.13	excluded	absent	17.59	-0.24	excluded	absent	29.13	-0.23	excluded	absent	69.25	-0.2	excluded	absent	132.73	-0.14	excluded	absent	3.19	-0.25	excluded	absent	7.98	-0.25
90265_at	excluded	absent	192.17	0.16	excluded	absent	96.36	-0.66	excluded	absent	57.4	-1	excluded	absent	41.07	-1.14	excluded	absent	103.45	-0.6	excluded	absent	74.71	-0.85	excluded	absent	100.78	-0.63	excluded	absent	21.49	-1.31	excluded	absent	156.27	-0.15	excluded	absent	134.15	-0.34	excluded	absent	16.24	-1.36	excluded	absent	501.23	2.84	excluded	absent	111.01	-0.54	excluded	absent	22.47	-1.3	excluded	absent	120.19	-0.46
90610_at	included	absent	237.6	-0.34	included	present	372.38	0.59	included	absent	126.76	-1.11	included	present	165.9	-0.84	included	absent	199.4	-0.61	included	present	360.58	0.51	included	absent	227.69	-0.41	included	present	201.28	-0.59	included	present	250.03	-0.26	included	absent	135.06	-1.05	included	present	399.5	0.78	included	present	704.2	2.88	included	present	157.27	-0.9	included	present	150.31	-0.95	included	present	325.82	0.27
91580_at	excluded	absent	9.98	-0.35	excluded	absent	5.2	-0.54	excluded	absent	26.69	0.32	excluded	absent	1.91	-0.67	excluded	absent	84.67	2.65	excluded	absent	76.1	2.31	excluded	absent	17.45	-0.05	excluded	absent	4.8	-0.56	excluded	absent	7.03	-0.47	excluded	absent	56.02	1.5	excluded	absent	84.3	2.64	excluded	absent	4.36	-0.58	excluded	absent	16.48	-0.09	excluded	absent	26.5	0.31	excluded	absent	3.49	-0.61
91617_at	included	absent	104.73	-0.82	included	present	224.84	0.78	included	absent	51.87	-1.52	included	present	105.14	-0.81	included	present	200.12	0.45	included	present	344.54	2.36	included	present	214.78	0.64	included	present	189.11	0.3	included	present	206.8	0.54	included	present	494.49	4.35	included	present	190.18	0.32	included	present	319.73	2.03	included	present	373.52	2.75	included	present	488.63	4.27	included	present	286.03	1.59
91682_at	excluded	absent	130.12	0.66	excluded	absent	134.44	0.74	excluded	absent	66.52	-0.63	excluded	absent	63.83	-0.69	excluded	absent	98.08	0.01	excluded	absent	151.94	1.1	included	present	182.7	1.72	included	present	89.9	-0.16	included	present	258.33	3.26	included	present	164.76	1.36	included	present	222.15	2.52	included	absent	128.05	0.62	included	present	124.22	0.54	included	present	121.53	0.48	included	present	353.07	5.18
91684_g_at	excluded	absent	105.45	0.75	excluded	absent	15.52	-1.18	excluded	absent	105.86	0.76	excluded	absent	108.47	0.82	excluded	absent	287.03	4.65	excluded	absent	133.68	1.36	excluded	absent	200.79	2.8	included	present	322.98	5.43	included	present	198.02	2.74	included	present	286.94	4.65	included	present	327.7	5.53	excluded	absent	69.5	-0.02	excluded	absent	26.82	-0.93	excluded	absent	6.21	-1.38	included	present	415.99	7.42
91703_at	included	present	178.6	-0.02	excluded	absent	120.75	-0.42	excluded	absent	61.49	-0.84	excluded	absent	6.96	-1.23	included	present	178.11	-0.02	included	present	205.36	0.17	excluded	absent	48.33	-0.93	included	present	157.82	-0.16	excluded	absent	83.08	-0.69	included	present	220.35	0.28	excluded	absent	21.48	-1.12	included	present	412.08	1.63	included	present	130.79	-0.35	included	present	112.71	-0.48	excluded	absent	27.18	-1.08
91816_f_at	included	present	171.15	-0.46	included	present	91.56	-1.1	included	present	448.18	1.77	included	present	489.85	2.11	included	present	203.7	-0.2	included	present	200.43	-0.22	included	present	416.22	1.51	included	present	430.08	1.62	included	present	408.74	1.45	included	present	320.75	0.74	included	present	444.82	1.74	included	present	171.26	-0.46	included	present	105.21	-0.99	included	present	84.13	-1.16	included	present	709.14	3.87
91826_at	included	present	112.02	-0.37	included	present	267.19	-0.28	included	present	189.61	-0.33	included	present	136.39	-0.36	excluded	absent	131.58	-0.36	included	present	173.67	-0.34	included	absent	43.51	-0.41	included	present	338.54	-0.24	excluded	absent	81.55	-0.39	excluded	absent	104.08	-0.37	excluded	absent	75.43	-0.39	excluded	absent	35.39	-0.41	excluded	absent	59.62	-0.4	included	present	124.8	-0.36	included	present	62.66	-0.4
91920_at	excluded	absent	16.71	-0.66	excluded	absent	13.74	-0.68	excluded	absent	4.19	-0.75	excluded	absent	6.26	-0.74	excluded	absent	4.74	-0.75	excluded	absent	3.71	-0.75	excluded	absent	46.76	-0.44	excluded	absent	5.42	-0.74	excluded	absent	7.21	-0.73	excluded	absent	50.89	-0.41	excluded	absent	14.68	-0.67	excluded	absent	6.07	-0.74	excluded	absent	61.23	-0.33	excluded	absent	1.87	-0.77	excluded	absent	19.29	-0.64
91952_at	excluded	absent	67.62	0.59	excluded	absent	91.15	1.24	excluded	absent	49.99	0.1	excluded	absent	6.8	-1.09	excluded	absent	14.17	-0.89	excluded	absent	121.94	2.09	excluded	absent	93.69	1.31	excluded	absent	112.88	1.84	included	present	127.08	2.23	included	present	147.62	2.8	excluded	absent	110.34	1.77	excluded	absent	31.78	-0.4	excluded	absent	4.55	-1.15	excluded	absent	27.85	-0.51	included	present	248.36	5.58
AFFX-BioB-3_	included	present	4362.19	-1.96	included	present	5637.87	-1.36	included	present	7884.44	-0.3	included	present	6749.42	-0.84	included	present	11156.22	1.23	included	present	8633.14	0.05	included	present	4899.74	-1.7	included	present	3502.18	-2.36	included	present	2718.18	-2.73	included	present	2753.61	-2.71	included	present	3964.84	-2.14	included	present	4717.92	-1.79	included	present	2290.18	-2.93	included	present	310.38	-3.86	included	present	1346.87	-3.37
AFFX-BioB-5_	included	present	2987.72	-1.79	included	present	3419.46	-1.49	included	present	5197.45	-0.22	included	present	4173.32	-0.95	included	present	7553.97	1.46	included	present	5494.7	-0.01	included	present	3315.22	-1.56	included	present	2317.58	-2.27	included	present	2070.99	-2.45	included	present	1798.31	-2.64	included	present	2525.04	-2.12	included	present	3193.43	-1.65	included	present	1641.45	-2.75	included	present	283.89	-3.72	included	present	891.28	-3.29
AFFX-BioB-M	included	present	5200.12	-1.81	included	present	5607.55	-1.64	included	present	8172.67	-0.58	included	present	7377.2	-0.91	included	present	12810.42	1.33	included	present	9843.08	0.1	included	present	5823.87	-1.55	included	present	3854.54	-2.37	included	present	3149.33	-2.66	included	present	3335.16	-2.58	included	present	4297.42	-2.18	included	present	5258.11	-1.79	included	present	2621.47	-2.88	included	present	360.57	-3.81	included	present	1426.13	-3.37
AFFX-BioC-3_	included	present	7799.28	-1.83	included	present	7183.85	-1.99	included	present	12408.87	-0.58	included	present	11400.56	-0.86	included	present	19360.79	1.29	included	present	16512.18	0.52	included	present	8900.63	-1.53	included	present	5677.3	-2.4	included	present	5134.21	-2.55	included	present	5230.2	-2.52	included	present	7360.14	-1.95	included	present	8745.91	-1.57	included	present	8553.57	-1.62	included	present	948.04	-3.67	included	present	4467.29	-2.73
AFFX-BioC-5_	included	present	9726.73	-1.82	included	present	9159.18	-1.94	included	present	16047.35	-0.47	included	present	12839.35	-1.15	included	present	24870.51	1.41	included	present	17391.99	-0.18	included	present	10369.64	-1.68	included	present	6898.7	-2.42	included	present	5927.65	-2.63	included	present	6412.65	-2.52	included	present	8401.83	-2.1	included	present	10446.95	-1.66	included	present	9711.13	-1.82	included	present	1134.89	-3.65	included	present	5489.91	-2.72
AFFX-BioDn-3	included	present	36049.07	-2.01	included	present	35243.61	-2.04	included	present	52196.05	-1.3	included	present	46449.34	-1.55	included	present	86200.48	0.17	included	present	67978.17	-0.62	included	present	39521.32	-1.85	included	present	26239.61	-2.43	included	present	21168.68	-2.65	included	present	21863.16	-2.62	included	present	30015.74	-2.27	included	present	37407.35	-1.95	included	present	37089.14	-1.96	included	present	6177.31	-3.3	included	present	20275.77	-2.69
AFFX-BioDn-5	included	present	23195.43	-1.92	included	present	20519.78	-2.1	included	present	36479.05	-0.98	included	present	31454.05	-1.34	included	present	57440.88	0.49	included	present	44608.62	-0.41	included	present	25425.12	-1.76	included	present	16958.98	-2.35	included	present	14236.98	-2.54	included	present	14133.61	-2.55	included	present	19676.26	-2.16	included	present	24435.36	-1.83	included	present	24564.17	-1.82	included	present	4254.56	-3.24	included	present	13667.27	-2.58
AFFX-CreX-3_	included	present	140008.2	-1.22	included	present	86409.04	-2.22	included	present	171520.2	-0.62	included	present	150153.2	-1.03	included	present	259882.5	1.04	included	present	201803.4	-0.06	included	present	116243.5	-1.66	included	present	75807.02	-2.42	included	present	59445.82	-2.73	included	present	77436.14	-2.39	included	present	97346.35	-2.02	included	present	116660.3	-1.66	included	present	107593.2	-1.83	included	present	33778.82	-3.21	included	present	64232.45	-2.64
AFFX-CreX-5_	included	present	130031.7	-1.26	included	present	83655.74	-2.1	included	present	171257.5	-0.5	included	present	139563	-1.08	included	present	265531	1.22	included	present	183779.2	-0.28	included	present	108153.3	-1.66	included	present	68941.11	-2.37	included	present	55932.02	-2.61	included	present	78644.97	-2.19	included	present	97325.18	-1.85	included	present	115338	-1.52	included	present	103919	-1.73	included	present	33886.8	-3.01	included	present	67024.98	-2.41
AFFX-DapX-3	excluded	absent	64.33	1.86	excluded	absent	17.36	-0.09	excluded	absent	2.1	-0.73	excluded	absent	3.04	-0.69	excluded	absent	7.49	-0.5	excluded	absent	38.24	0.78	excluded	absent	3.4	-0.67	excluded	absent	23.32	0.16	excluded	absent	8.94	-0.44	excluded	absent	5.21	-0.6	excluded	absent	7.5	-0.5	excluded	absent	8.92	-0.44	excluded	absent	10.76	-0.37	excluded	absent	27.19	0.32	excluded	absent	1.71	-0.74
AFFX-DapX-5	excluded	absent	3.75	-0.91	excluded	absent	19.69	-0.26	excluded	absent	47.22	0.85	excluded	absent	3.1	-0.94	excluded	absent	3.34	-0.93	excluded	absent	2.72	-0.95	excluded	absent	43.67	0.71	excluded	absent	4.3	-0.89	excluded	absent	20.39	-0.24	excluded	absent	21.54	-0.19	excluded	absent	53.99	1.13	excluded	absent	29.25	0.12	excluded	absent	23.89	-0.09	excluded	absent	4.77	-0.87	excluded	absent	16.95	-0.38
AFFX-DapX-M	excluded	absent	10.49	-0.01	excluded	absent	4.5	-0.36	excluded	absent	7.4	-0.19	excluded	absent	3.71	-0.4	excluded	absent	58.98	2.82	excluded	absent	32.2	1.26	excluded	absent	9.93	-0.04	excluded	absent	4.83	-0.34	excluded	absent	2.92	-0.45	excluded	absent	2.86	-0.45	excluded	absent	4.9	-0.33	excluded	absent	8.77	-0.11	excluded	absent	3.79	-0.4	excluded	absent	5.52	-0.3	excluded	absent	1.72	-0.52
AFFX-HSAC07	included	present	53145.07	-1.03	included	present	52381.52	-1.07	included	present	64923.46	-0.35	included	present	41738.32	-1.69	included	present	70763.39	-0.01	included	present	51754.62	-1.11	included	present	25467.42	-2.63	included	present	23951.42	-2.72	included	present	26515.9	-2.57	included	present	36221.23	-2.01	included	present	38425.79	-1.88	included	present	52683.69	-1.06	included	present	35824.96	-2.03	included	present	32815.33	-2.2	included	present	31086.91	-2.3
AFFX-HSAC07	included	present	5031.29	-0.09	included	present	2146.13	-1.07	included	present	3475.21	-0.62	included	present	1811.21	-1.18	included	present	3648.19	-0.56	included	present	25267.73	6.78	included	present	8618.82	1.13	included	present	9415.05	1.4	included	present	8661.57	1.14	included	present	10834.25	1.88	included	present	14140.07	3	included	present	6040.06	0.25	included	present	280.86	-1.7	included	present	268.74	-1.71	included	present	18054.97	4.33
AFFX-HSAC07	included	present	12222.01	0.73	included	present	8514.69	-0.08	included	present	7995.41	-0.19	included	present	4462.98	-0.96	included	present	9840.04	0.21	included	present	33441.24	5.36	included	present	14936.79	1.32	included	present	18811.69	2.17	included	present	17338.58	1.85	included	present	23734.33	3.24	included	present	24407.73	3.39	included	present	15737.44	1.5	included	present	10710.25	0.4	included	present	10925.9	0.45	included	present	28685.41	4.33
AFFX-hum_al	included	present	112336.1	-0.88	included	present	91449.36	-1.4	included	present	152609.6	0.13	included	present	88588.38	-1.47	included	present	170229.9	0.57	included	present	79897.72	-1.68	included	present	52085.87	-2.38	included	present	34101.97	-2.83	included	present	44426.37	-2.57	included	present	71634.74	-1.89	included	present	75129.88	-1.8	included	present	83644.76	-1.59	included	present	83939.57	-1.58	included	present	61770.96	-2.14	included	present	47089.77	-2.5
AFFX-HUMGA	included	present	10044.84	-0.15	included	present	12713.03	0.22	included	present	8392.28	-0.37	included	present	12048.84	0.13	included	present	8096.29	-0.41	included	present	22706.03	1.59	included	present	14810.63	0.51	included	present	18267.68	0.98	included	present	11365.67	0.03	included	present	22813.99	1.61	included	present	20937.47	1.35	included	present	16580.76	0.75	included	present	6169.68	-0.68	included	present	8023.12	-0.42	included	present	25822.74	2.02
AFFX-HUMGA	included	present	9464.6	0.27	included	present	4653.75	-0.59	included	present	10961.73	0.53	included	present	11015.59	0.54	included	present	7328.05	-0.11	included	present	25729.9	3.15	included	present	14170.97	1.1	included	present	19811.38	2.1	included	present	13218.81	0.93	included	present	19724.14	2.09	included	present	28245.28	3.6	included	present	8301.37	0.06	included	present	2619.6	-0.95	included	present	3187.75	-0.85	included	present	21884.23	2.47
AFFX-HUMGA	included	present	5926.71	0.22	included	present	5428.37	0.1	included	present	4564.77	-0.11	included	present	6011.09	0.24	included	present	4551.81	-0.11	included	present	13214.42	1.99	included	present	7507.07	0.61	included	present	13203	1.99	included	present	7549.11	0.62	included	present	14973.87	2.42	included	present	19142.29	3.42	included	present	8159.05	0.76	included	present	638.79	-1.06	included	present	891	-1	included	present	17544.55	3.04
AFFX-HUMISG	included	present	2243.65	0.18	included	present	1048.45	-0.35	included	present	941.42	-0.4	included	present	1706.95	-0.06	included	present	744.14	-0.49	included	present	6066.7	1.9	included	present	1285.12	-0.25	included	present	2052.04	0.1	included	present	1973	0.06	included	present	940.42	-0.4	included	present	948.08	-0.4	included	present	1332.28	-0.22	included	present	1882.16	0.02	included	present	1383.75	-0.2	included	present	4205.61	1.06
AFFX-HUMISG	included	present	1766.38	0.92	included	present	712.75	-0.13	included	present	1010.75	0.17	included	present	1177.9	0.33	included	present	840.35	0	included	present	3673.39	2.81	included	present	526.39	-0.32	included	present	938.7	0.09	included	present	1071.02	0.23	included	present	521.15	-0.32	included	present	598.19	-0.24	included	present	301.21	-0.54	included	present	996.34	0.15	included	present	590.19	-0.25	included	present	2735.75	1.88
AFFX-HUMISG	included	present	687.33	0.43	included	present	156.5	-0.43	included	present	345.35	-0.12	included	present	342	-0.13	included	present	316.02	-0.17	included	present	1771.45	2.17	included	present	226.11	-0.31	included	present	435.8	0.02	included	present	516.21	0.15	included	present	224.4	-0.32	included	present	261.13	-0.26	included	present	150.76	-0.44	included	absent	86.36	-0.54	included	present	88.36	-0.54	included	present	1643.23	1.97
AFFX-HUMISG	included	present	1181.36	0.32	included	present	309.7	-0.5	included	present	447.96	-0.37	included	present	827.86	-0.01	included	present	404.79	-0.41	included	present	2901.59	1.94	included	present	380.73	-0.43	included	present	875.3	0.03	included	present	904.08	0.06	included	present	417.91	-0.4	included	present	522.62	-0.3	included	present	584.62	-0.24	included	present	689.35	-0.14	included	present	490.25	-0.33	included	present	2307.63	1.38
AFFX-HUMRG	included	present	87853.3	-0.98	included	present	76217.55	-1.25	included	present	88073.1	-0.98	included	present	59396.9	-1.64	included	present	128984.4	-0.03	included	present	82846.21	-1.1	included	present	40932.21	-2.07	included	present	32989.11	-2.26	included	present	45301.51	-1.97	included	present	64586.26	-1.52	included	present	70208.63	-1.39	included	present	75514.23	-1.27	included	present	20330.44	-2.55	included	present	14230.32	-2.69	included	present	48468.79	-1.9
AFFX-HUMRG	included	present	43229.46	-0.78	included	present	31207.62	-1.2	included	present	43073.71	-0.79	included	present	27664.97	-1.33	included	present	54826.35	-0.37	included	present	64378.6	-0.03	included	present	31181.7	-1.2	included	present	28756.1	-1.29	included	present	30546.92	-1.23	included	present	43739.51	-0.76	included	present	45229.36	-0.71	included	present	59858.88	-0.19	included	present	13127	-1.84	included	present	8157.73	-2.02	included	present	35335.13	-1.06
AFFX-HUMRG	included	present	102467.4	-0.76	included	present	82927.09	-1.23	included	present	109844	-0.58	included	present	69610.35	-1.56	included	present	165675	0.78	included	present	98106.8	-0.86	included	present	51710.29	-1.99	included	present	36750.53	-2.36	included	present	47679.07	-2.09	included	present	80325.22	-1.3	included	present	80162.89	-1.3	included	present	82058.3	-1.25	included	present	31854.16	-2.48	included	present	22941.15	-2.69	included	present	51437.38	-2
AFFX-LysX-3_	excluded	absent	4.4	-0.77	excluded	absent	6.89	-0.65	excluded	absent	1.99	-0.9	excluded	absent	3.16	-0.84	excluded	absent	74.67	2.8	excluded	absent	2.41	-0.88	excluded	absent	32.53	0.66	excluded	absent	7.08	-0.64	excluded	absent	1.45	-0.92	excluded	absent	25.98	0.32	excluded	absent	4.15	-0.79	excluded	absent	3.94	-0.8	excluded	absent	33.03	0.68	excluded	absent	2.5	-0.87	excluded	absent	14.85	-0.24
AFFX-LysX-5_	excluded	absent	3.5	-0.68	excluded	absent	7.77	-0.48	excluded	absent	57.72	1.9	excluded	absent	9.46	-0.4	excluded	absent	7.91	-0.47	excluded	absent	12.94	-0.23	excluded	absent	3.73	-0.67	excluded	absent	7.06	-0.51	excluded	absent	30.99	0.63	excluded	absent	4.29	-0.64	excluded	absent	23.24	0.26	excluded	absent	9.23	-0.41	excluded	absent	52.94	1.67	excluded	absent	15.03	-0.13	excluded	absent	1.64	-0.77
AFFX-LysX-M	excluded	absent	34.63	1.1	excluded	absent	5.14	-0.62	excluded	absent	3.62	-0.71	excluded	absent	30.77	0.87	excluded	absent	27.33	0.67	excluded	absent	52.96	2.16	excluded	absent	4.81	-0.64	excluded	absent	10.16	-0.33	excluded	absent	5.61	-0.59	excluded	absent	3.25	-0.73	excluded	absent	42.19	1.54	excluded	absent	80.35	3.76	excluded	absent	5.5	-0.6	excluded	absent	5.7	-0.59	excluded	absent	25.2	0.55
AFFX-M2783	excluded	absent	35.15	0.04	excluded	absent	43.01	0.29	excluded	absent	16.21	-0.56	excluded	absent	7.76	-0.83	excluded	absent	42.77	0.29	included	present	254.95	7.07	excluded	absent	151.92	3.78	excluded	absent	68.63	1.11	excluded	absent	95.15	1.96	excluded	absent	51.8	0.58	excluded	absent	65.05	1	included	present	91.95	1.86	excluded	absent	14.07	-0.63	excluded	absent	1.53	-1.03	excluded	absent	52.25	0.59
AFFX-M2783	included	present	91750.96	-0.77	included	present	80420.09	-1.03	included	present	103210.9	-0.5	included	present	79236.17	-1.06	included	present	159367.4	0.79	included	present	89703.88	-0.81	included	present	39625.25	-1.97	included	present	36945.25	-2.03	included	present	35678.09	-2.06	included	present	55837.7	-1.6	included	present	60055.29	-1.5	included	present	84524.91	-0.93	included	present	17052.55	-2.49	included	present	15187.75	-2.54	included	present	44419.88	-1.86
AFFX-M2783	included	present	2672.75	-0.53	included	present	3577.7	0.2	included	present	2480.84	-0.69	included	present	2022.26	-1.06	included	present	3426.64	0.08	included	present	7147.45	3.1	included	present	3760.85	0.35	included	present	3109.18	-0.18	included	present	3092.49	-0.19	included	present	2890.51	-0.35	included	present	3775.4	0.36	included	present	4957.94	1.33	included	present	2172.02	-0.94	included	present	1400.54	-1.57	included	present	3521.26	0.16
AFFX-PheX-3_	excluded	absent	37.19	0.24	excluded	absent	30.29	-0.02	excluded	absent	39.42	0.32	excluded	absent	4.35	-1	excluded	absent	19.12	-0.44	excluded	absent	4.01	-1.02	excluded	absent	3.89	-1.02	excluded	absent	4.43	-1	excluded	absent	18.93	-0.45	excluded	absent	9.6	-0.81	excluded	absent	17.91	-0.49	excluded	absent	7.89	-0.87	excluded	absent	9.38	-0.81	excluded	absent	18.45	-0.47	excluded	absent	1.88	-1.1
AFFX-PheX-5_	excluded	absent	39.96	0.9	excluded	absent	14.03	-0.15	excluded	absent	4.76	-0.52	excluded	absent	38.52	0.84	excluded	absent	19.24	0.06	excluded	absent	14.82	-0.11	excluded	absent	5.3	-0.5	excluded	absent	21.39	0.15	excluded	absent	3.98	-0.55	excluded	absent	6.51	-0.45	excluded	absent	11.71	-0.24	excluded	absent	4.63	-0.52	excluded	absent	1.89	-0.63	excluded	absent	3.01	-0.59	excluded	absent	3.8	-0.56
AFFX-PheX-M	excluded	absent	22.01	-0.14	excluded	absent	12.86	-0.52	excluded	absent	16.53	-0.37	excluded	absent	3.26	-0.91	excluded	absent	4.35	-0.87	excluded	absent	88.37	2.59	excluded	absent	25.01	-0.02	excluded	absent	44.17	0.77	excluded	absent	6.23	-0.79	excluded	absent	47.19	0.89	excluded	absent	17.55	-0.33	excluded	absent	43.32	0.73	excluded	absent	42.72	0.71	excluded	absent	9.02	-0.68	excluded	absent	4.54	-0.86
AFFX-r2-Bs-d	excluded	absent	12.81	0.21	excluded	absent	40.17	2.16	excluded	absent	3.96	-0.42	excluded	absent	1.8	-0.57	excluded	absent	9.83	0	excluded	absent	0.86	-0.64	excluded	absent	1.32	-0.6	excluded	absent	4.32	-0.39	excluded	absent	1.91	-0.56	excluded	absent	22.77	0.92	excluded	absent	37.69	1.99	excluded	absent	1.71	-0.58	excluded	absent	0.95	-0.63	excluded	absent	1.92	-0.56	excluded	absent	0.64	-0.65
AFFX-r2-Bs-d	excluded	absent	91.47	4.23	excluded	absent	29.93	0.84	excluded	absent	3.38	-0.62	excluded	absent	5.56	-0.5	excluded	absent	23.87	0.51	excluded	absent	44.15	1.62	excluded	absent	2.86	-0.65	excluded	absent	11.97	-0.15	excluded	absent	8.46	-0.34	excluded	absent	13.65	-0.06	excluded	absent	19.35	0.26	excluded	absent	1.85	-0.7	excluded	absent	33.86	1.06	excluded	absent	23.52	0.49	excluded	absent	17.72	0.17
AFFX-r2-Bs-d	excluded	absent	38.59	0.72	excluded	absent	17.45	-0.18	excluded	absent	39.64	0.77	excluded	absent	2.72	-0.81	excluded	absent	58.72	1.59	excluded	absent	83.09	2.63	excluded	absent	34.5	0.55	excluded	absent	10.58	-0.47	excluded	absent	13.77	-0.34	excluded	absent	2.73	-0.81	excluded	absent	7.58	-0.6	excluded	absent	34.42	0.55	excluded	absent	52.46	1.32	excluded	absent	2.28	-0.83	excluded	absent	21.71	0
AFFX-r2-Bs-ly	excluded	absent	38.83	-0.06	excluded	absent	97.53	1.53	excluded	absent	5.98	-0.94	excluded	absent	34.71	-0.17	excluded	absent	77.3	0.98	excluded	absent	67.72	0.72	excluded	absent	7.82	-0.89	excluded	absent	3.13	-1.02	excluded	absent	14	-0.73	excluded	absent	42.41	0.04	excluded	absent	1.81	-1.06	excluded	absent	38.77	-0.06	excluded	absent	35.28	-0.15	excluded	absent	20.28	-0.56	excluded	absent	0.89	-1.08
AFFX-r2-Bs-ly	excluded	absent	28.46	0	excluded	absent	7.89	-0.91	excluded	absent	5.13	-1.03	excluded	absent	1.95	-1.17	excluded	absent	69.2	1.78	excluded	absent	35.81	0.32	excluded	absent	1.65	-1.18	excluded	absent	0.7	-1.22	excluded	absent	1.83	-1.17	excluded	absent	7.3	-0.93	excluded	absent	24.93	-0.16	excluded	absent	4.21	-1.07	excluded	absent	20.53	-0.35	excluded	absent	25.7	-0.13	excluded	absent	22.83	-0.25
AFFX-r2-Bs-ly	excluded	absent	4.68	-0.51	excluded	absent	3.26	-0.58	excluded	absent	2.43	-0.62	excluded	absent	14.25	-0.05	excluded	absent	3.36	-0.58	excluded	absent	1.28	-0.68	excluded	absent	30.8	0.75	excluded	absent	0.97	-0.69	excluded	absent	4.28	-0.53	excluded	absent	1.33	-0.67	excluded	absent	2.18	-0.63	excluded	absent	10.44	-0.23	excluded	absent	2.22	-0.63	excluded	absent	5.51	-0.47	excluded	absent	2.1	-0.64
AFFX-r2-Bs-pl	excluded	absent	13.53	-0.44	excluded	absent	3.75	-0.68	excluded	absent	6.28	-0.62	excluded	absent	4.58	-0.66	excluded	absent	11.5	-0.49	excluded	absent	6.19	-0.62	excluded	absent	3.24	-0.69	excluded	absent	11.66	-0.49	excluded	absent	31.34	-0.01	excluded	absent	91.1	1.42	excluded	absent	19.21	-0.31	excluded	absent	8.19	-0.57	excluded	absent	64.87	0.79	excluded	absent	3.88	-0.67	excluded	absent	3.43	-0.68
AFFX-r2-Bs-pl	excluded	absent	3.61	-0.78	excluded	absent	2.6	-0.81	excluded	absent	60.3	1.25	excluded	absent	39.69	0.52	excluded	absent	85.03	2.14	excluded	absent	11.66	-0.49	excluded	absent	33.22	0.28	excluded	absent	2.86	-0.8	excluded	absent	3.28	-0.79	excluded	absent	4.69	-0.74	excluded	absent	28.7	0.12	excluded	absent	2.69	-0.81	excluded	absent	50.23	0.89	excluded	absent	22.91	-0.09	excluded	absent	3.23	-0.79
AFFX-r2-Bs-pl	excluded	absent	2.71	-0.54	excluded	absent	65.27	4.4	excluded	absent	20.19	0.84	excluded	absent	2.59	-0.55	excluded	absent	5.02	-0.36	excluded	absent	82.25	5.74	excluded	absent	32.04	1.77	excluded	absent	2.59	-0.55	excluded	absent	4.38	-0.41	excluded	absent	8.04	-0.12	excluded	absent	2.33	-0.57	excluded	absent	4.74	-0.38	excluded	absent	29.41	1.57	excluded	absent	38.66	2.3	excluded	absent	0.59	-0.71
AFFX-r2-Bs-th	excluded	absent	9.62	-0.49	excluded	absent	76.65	3.03	excluded	absent	3.02	-0.83	excluded	absent	22.54	0.19	excluded	absent	4.66	-0.75	excluded	absent	3.51	-0.81	excluded	absent	4.4	-0.76	excluded	absent	6.48	-0.65	excluded	absent	49	1.58	excluded	absent	8.02	-0.57	excluded	absent	48.35	1.55	excluded	absent	54.86	1.89	excluded	absent	62.01	2.26	excluded	absent	2.53	-0.86	excluded	absent	12.76	-0.32
AFFX-r2-Bs-th	excluded	absent	24.55	-0.3	excluded	absent	1.8	-1.07	excluded	absent	11.27	-0.75	excluded	absent	29.6	-0.13	excluded	absent	33.03	-0.01	excluded	absent	7.03	-0.89	excluded	absent	1.14	-1.09	excluded	absent	51.99	0.62	excluded	absent	28.73	-0.16	excluded	absent	1.96	-1.06	excluded	absent	47.19	0.46	excluded	absent	30.26	-0.11	excluded	absent	36.05	0.09	excluded	absent	12.51	-0.71	excluded	absent	25	-0.28
AFFX-r2-Bs-th	excluded	absent	40.09	1.43	excluded	absent	13.47	-0.01	excluded	absent	4.49	-0.49	excluded	absent	11.76	-0.1	excluded	absent	2.44	-0.6	excluded	absent	8.16	-0.3	excluded	absent	5.37	-0.45	excluded	absent	6.35	-0.39	excluded	absent	4.76	-0.48	excluded	absent	38.77	1.36	excluded	absent	3.62	-0.54	excluded	absent	3.65	-0.54	excluded	absent	39.27	1.38	excluded	absent	22.53	0.48	excluded	absent	3.58	-0.54
AFFX-r2-Ec-bi	included	present	5881.28	-2.26	included	present	8194.25	-1.27	included	present	9683.89	-0.63	included	present	8488.67	-1.14	included	present	15141.17	1.7	included	present	11961.53	0.34	included	present	6911.43	-1.82	included	present	4888.05	-2.68	included	present	3975.61	-3.07	included	present	3834.21	-3.14	included	present	5212.75	-2.55	included	present	6159.86	-2.14	included	present	3402.44	-3.32	included	present	2744.95	-3.6	included	present	1813.7	-4
AFFX-r2-Ec-bi	included	present	4770.96	-2.08	included	present	5447.43	-1.69	included	present	7894.02	-0.26	included	present	6668.12	-0.97	included	present	12418.35	2.39	included	present	7577.19	-0.44	included	present	4562.97	-2.2	included	present	2897.42	-3.18	included	present	2447.83	-3.44	included	present	3344.98	-2.92	included	present	4318.42	-2.35	included	present	4696.54	-2.13	included	present	2161.27	-3.61	included	present	1987.18	-3.71	included	present	1233.34	-4.15
AFFX-r2-Ec-bi	included	present	4279.83	-2.54	included	present	6121.64	-1.44	included	present	8359.56	-0.1	included	present	6846.89	-1	included	present	11793.72	1.95	included	present	8939.36	0.25	included	present	5089.92	-2.05	included	present	3560.31	-2.97	included	present	3083.08	-3.25	included	present	2865.07	-3.38	included	present	4181.9	-2.59	included	present	4758.96	-2.25	included	present	2446.39	-3.63	included	present	2137.82	-3.82	included	present	1286.39	-4.33
AFFX-r2-Ec-bi	included	present	11858.29	-2.43	included	present	15254.68	-1.78	included	present	20454.08	-0.79	included	present	17365.79	-1.38	included	present	32910.31	1.58	included	present	22665.71	-0.37	included	present	13164.91	-2.18	included	present	8825.64	-3	included	present	6912.36	-3.37	included	present	8240.7	-3.11	included	present	10619.16	-2.66	included	present	12898.44	-2.23	included	present	12446.75	-2.31	included	present	10850.54	-2.62	included	present	6838.62	-3.38
AFFX-r2-Ec-bi	included	present	12423.53	-2.32	included	present	15375.46	-1.76	included	present	20893.07	-0.72	included	present	18313.9	-1.2	included	present	33319.74	1.63	included	present	25291.7	0.12	included	present	13457.12	-2.12	included	present	8671.62	-3.02	included	present	7492.52	-3.25	included	present	8435.41	-3.07	included	present	11455.6	-2.5	included	present	13287.48	-2.15	included	present	13197.27	-2.17	included	present	10731.59	-2.64	included	present	7550.37	-3.24
AFFX-r2-Ec-bi	included	present	61745.3	-2.36	included	present	60455.07	-2.41	included	present	88721.59	-1.38	included	present	78860.62	-1.74	included	present	131541.7	0.18	included	present	106982.2	-0.71	included	present	63447.57	-2.3	included	present	38918.38	-3.19	included	present	32457.83	-3.42	included	present	37617.52	-3.24	included	present	53049.61	-2.68	included	present	60030.26	-2.42	included	present	56086.39	-2.56	included	present	46921.58	-2.9	included	present	34845.18	-3.34
AFFX-r2-Ec-bi	included	present	45752.86	-2.35	included	present	49733.98	-2.17	included	present	66507.75	-1.44	included	present	59214.18	-1.76	included	present	104106.8	0.22	included	present	84135.94	-0.66	included	present	48228.13	-2.24	included	present	32283.55	-2.94	included	present	26647.6	-3.19	included	present	28930.79	-3.09	included	present	36836.18	-2.74	included	present	46370.02	-2.32	included	present	46776.7	-2.3	included	present	41284.7	-2.55	included	present	24670.44	-3.28
AFFX-r2-P1-c	included	present	179267.3	-1.39	included	present	173458.9	-1.48	included	present	222518.9	-0.74	included	present	200092.3	-1.08	included	present	357146.7	1.3	included	present	246869.7	-0.37	included	present	144005.9	-1.93	included	present	95049.73	-2.67	included	present	76638.87	-2.95	included	present	105850	-2.51	included	present	132153.6	-2.11	included	present	148670.5	-1.86	included	present	143327.4	-1.94	included	present	112897.7	-2.4	included	present	86304.02	-2.8
AFFX-r2-P1-c	included	present	141160.3	-1.52	included	present	126420.5	-1.81	included	present	189541.9	-0.58	included	present	167949.4	-1	included	present	316289.1	1.87	included	present	199143.5	-0.4	included	present	114032.4	-2.05	included	present	73699.66	-2.83	included	present	57379.32	-3.14	included	present	89374.35	-2.52	included	present	100135.3	-2.32	included	present	118913.4	-1.95	included	present	113036.5	-2.07	included	present	86255.09	-2.58	included	present	68216.33	-2.93
AFFX-ThrX-3_	excluded	absent	13.48	-0.38	excluded	absent	4.61	-0.74	excluded	absent	5.54	-0.7	excluded	absent	3.19	-0.8	excluded	absent	16.58	-0.26	excluded	absent	5.25	-0.72	excluded	absent	8	-0.6	excluded	absent	6.97	-0.65	excluded	absent	8.44	-0.59	excluded	absent	19.64	-0.13	excluded	absent	48.97	1.06	excluded	absent	54.67	1.29	excluded	absent	34.77	0.48	excluded	absent	33.14	0.42	excluded	absent	8.99	-0.56
AFFX-ThrX-5_	excluded	absent	46.36	0.41	excluded	absent	7.54	-1.18	excluded	absent	1.92	-1.41	excluded	absent	3.76	-1.34	excluded	absent	6.6	-1.22	excluded	absent	10.42	-1.06	excluded	absent	62.93	1.09	excluded	absent	2.29	-1.4	excluded	absent	15.1	-0.87	excluded	absent	8.12	-1.16	excluded	absent	9.76	-1.09	excluded	absent	9.73	-1.09	excluded	absent	26.22	-0.42	excluded	absent	2.83	-1.38	excluded	absent	1.89	-1.41
AFFX-ThrX-M	excluded	absent	1.81	-0.8	excluded	absent	32.74	0.66	excluded	absent	2.51	-0.77	excluded	absent	1.43	-0.82	excluded	absent	46.21	1.3	excluded	absent	12.47	-0.3	excluded	absent	4.61	-0.67	excluded	absent	15.88	-0.14	excluded	absent	3.97	-0.7	excluded	absent	48.04	1.38	excluded	absent	14.59	-0.2	excluded	absent	17.04	-0.08	excluded	absent	3.35	-0.73	excluded	absent	4.05	-0.7	excluded	absent	4.87	-0.66
AFFX-TrpnX-3	excluded	absent	7.23	-0.79	excluded	absent	5.35	-0.86	excluded	absent	23.18	-0.21	excluded	absent	44.55	0.56	excluded	absent	51.67	0.82	excluded	absent	7.87	-0.77	excluded	absent	38.19	0.33	excluded	absent	4.99	-0.87	excluded	absent	21.07	-0.29	excluded	absent	8.67	-0.74	excluded	absent	36.72	0.28	excluded	absent	27.06	-0.07	excluded	absent	2.74	-0.95	excluded	absent	1.72	-0.99	excluded	absent	3.12	-0.94
AFFX-TrpnX-5	excluded	absent	39.23	0.28	excluded	absent	4.13	-0.8	excluded	absent	73.56	1.33	excluded	absent	6.92	-0.71	excluded	absent	37.79	0.24	excluded	absent	10.37	-0.61	excluded	absent	26.93	-0.1	excluded	absent	4.2	-0.8	excluded	absent	2.32	-0.85	excluded	absent	21.05	-0.28	excluded	absent	12.52	-0.54	excluded	absent	62.74	1	excluded	absent	36.11	0.18	excluded	absent	6.36	-0.73	excluded	absent	11.75	-0.56
AFFX-TrpnX-M	excluded	absent	8.25	-0.64	excluded	absent	40.88	0.65	excluded	absent	2.12	-0.88	excluded	absent	33.76	0.37	excluded	absent	29.22	0.19	excluded	absent	6.66	-0.7	excluded	absent	9.71	-0.58	excluded	absent	42.54	0.72	excluded	absent	5.82	-0.73	excluded	absent	26.53	0.09	excluded	absent	22.88	-0.06	excluded	absent	21.29	-0.12	excluded	absent	20.8	-0.14	excluded	absent	5.46	-0.75	excluded	absent	1.16	-0.92
