# Supplementary material for: Feasibility of implementing molecular-guided therapy for the treatment of patients with relapsed or refractory neuroblastoma
Source: Cancer Med. 2015 Feb 26;4(6):871–86. doi: 10.1002/cam4.436 (PMC4472210; doi:10.1002/cam4.436)
Supplement: Supplementary file 3 [file cam40004-0871-sd3.pdf]

Acarbose	ethacrynic acid	propylthiouracil	albendazole
acetylsalicylic acid	etodolac	pyridostigmine bromide	aldesleukin
adalimumab	etoposide (Vepesid)	rabeprazole sodium	amantadine
alprazolam	felbamate	raloxifene hydrochloride	amikacin
ambenonium chloride	felodipine	ramipril	aminocaproic acid
amiloride hydrochloride	fexofenadine	reserpine	amoxapine
amitriptyline hydrochloride	fludrocortisone	Rifamycin	amoxicillin
anakinra	fluvastatin	Rituxumab	ascorbic acid
anastrozole	gefitinib	rosiglitazone maleate	atovaquone
Cytarabine (Ara-C)	gemcitabine	rosuvastatin calcium	azacitidine
atorvastatin	ibuprofen	Saha	aztreonam
azathioprine	imatinib	simvastatin	baclofen
balsalazide	indomethacin	sirolimus (rapamycin)	betamethasone
BCNU	ketoprofen	sodium phenylbutyrate	bromocriptine
bevacizumab	ketorolac	sorafenib	brompheniramine
bortezomib (velcade)	lansoprazole	sulfasalazine	budesonide
bumetanide	leflunomide	sulindac	bupropion
carbamazepine	lenalidomide	sulindac sulfide	buspirone
carbidopa/Levodopa	lovastatin	sunitinib malate	calcitriol
carboplatin	mercaptopurine	tacrolimus	captopril
CCNU	metformin	tamoxifen	carbinoxamine
celecoxib	methimazole	Tarceva	cefaclor
cetuximab	methotrexate	temsirolimus	cefadroxil
chlorothiazide	Methyl CCNU	temozolomide	cefazolin
chlorproMAZINE	minocycline	thalidomide	cefepime
chlorproPAMIDE	mitoxantrone	theophylline	cefotaxime
cyclosporine	nabumetone	thioguanine	cefotetan
cisplatin	naproxen	thioridazine hydrochloride	cefoxitin
clofarabine	nitisinone	Thio-tepa	ceftazidime
clofibrate	omeprazole	tioguanine	cefuroxime
clopidogrel	orlistat	TOLBUTamide	cetirizine
clozapine	oxaliplatin		chlorambucil
cyclophosphamide	oxaprozin	tolmetin	chlorzoxazone
dantrolene	paclitaxel	topiramate	ciprofloxacin
dasatinib	pemetrexed	topotecan	citalopram
DAUNOrubicin	pentamidine	torsemide	clemastine
decitabine	phentolamine	tranylcypromine	clindamycin
deferoxamine	piperazine	trastuzumab	clomipramine
dexamethasone	piroxicam	trazodone	clonidine
diclofenac	pravastatin sodium	tretinoin	colchicine
donepezil hydrochloride	prazosin hydrochloride	valproic acid	cortisone
DOXOrubicin	prednisoLONE	verapamil	cyanocobalamin
doxycycline	prochlorperazine	vinBLAStine	cytarabine
epirubicin	pazopanib	vorinostat	erlotinib
everolimus	ixabepilone	romidepsin	vinorelbine
hydroxyurea	peg-asparaginase	vandetanib	zolendronic acid
crizotinib			

Supplemental Table #3- Clinical Trial Drug List
